# Supplementary figures and images for: Ephrin A1 functions as a ligand of EGFR to promote EMT and metastasis in gastric cancer (part 1 of 5)
Source: EMBO J. 2025 Jan 21;44(5):1464–87. doi: 10.1038/s44318-025-00363-x (PMC11876641; doi:10.1038/s44318-025-00363-x)

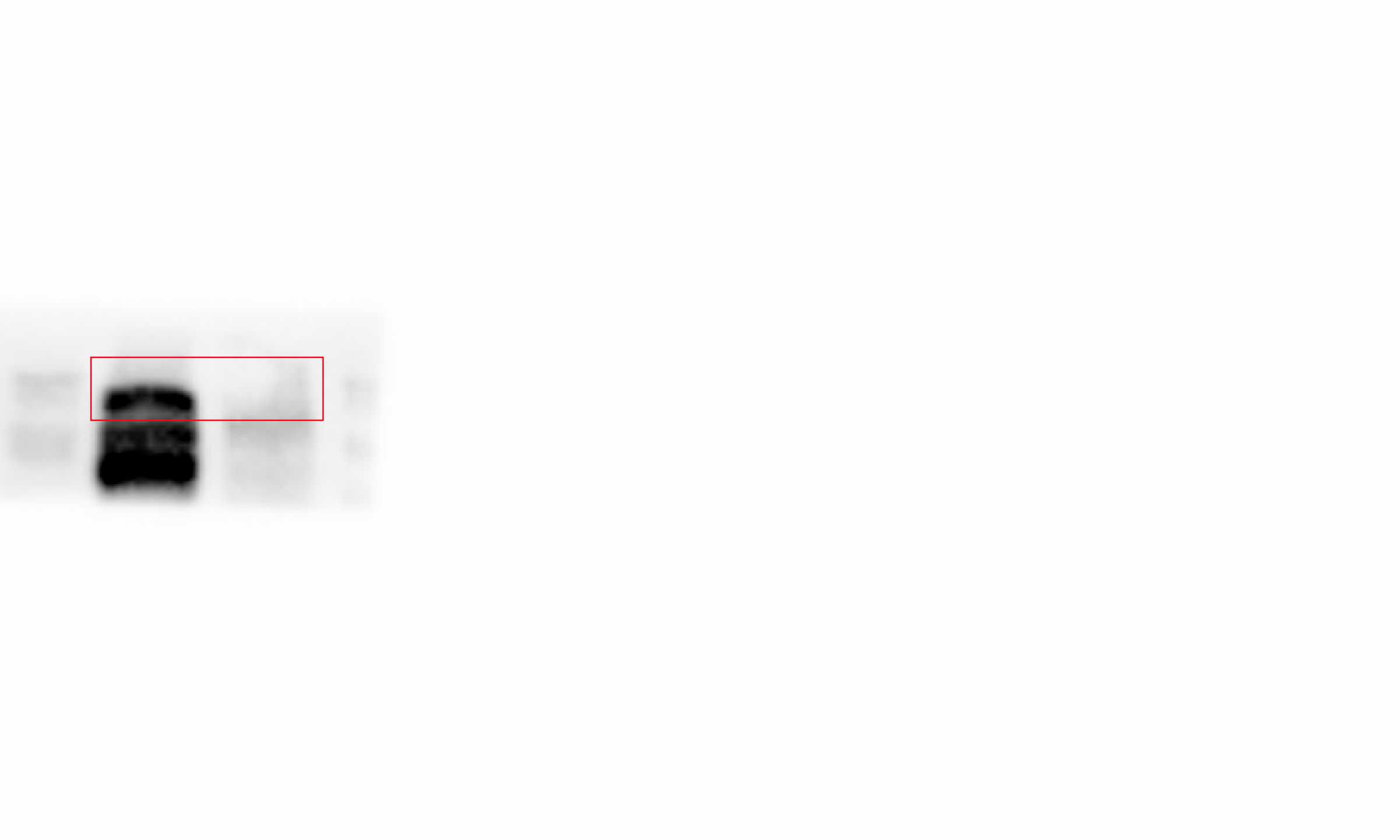

Supplement: Supplementary file 2 — Source data Fig. 1 [file 44318_2025_363_MOESM2_ESM.zip › Figure 1/1A/1 E-cad.tif]

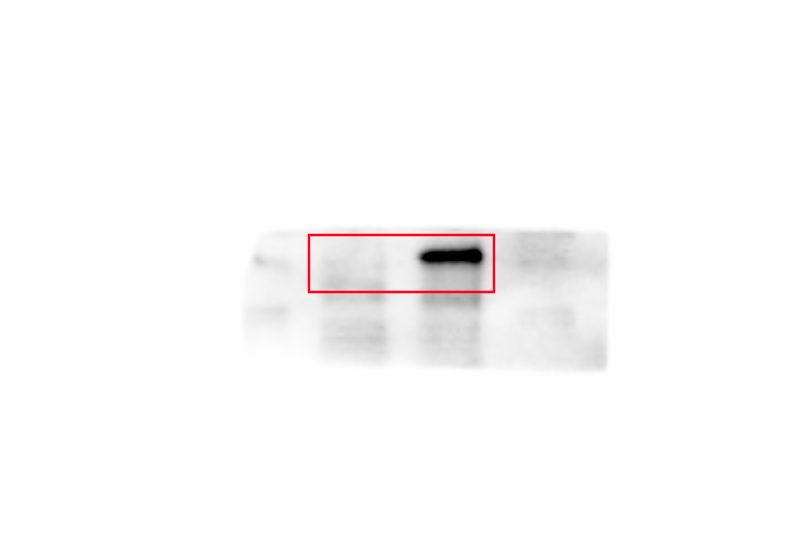

Supplement: Supplementary file 2 — Source data Fig. 1 [file 44318_2025_363_MOESM2_ESM.zip › Figure 1/1A/2 N-cad.tif]

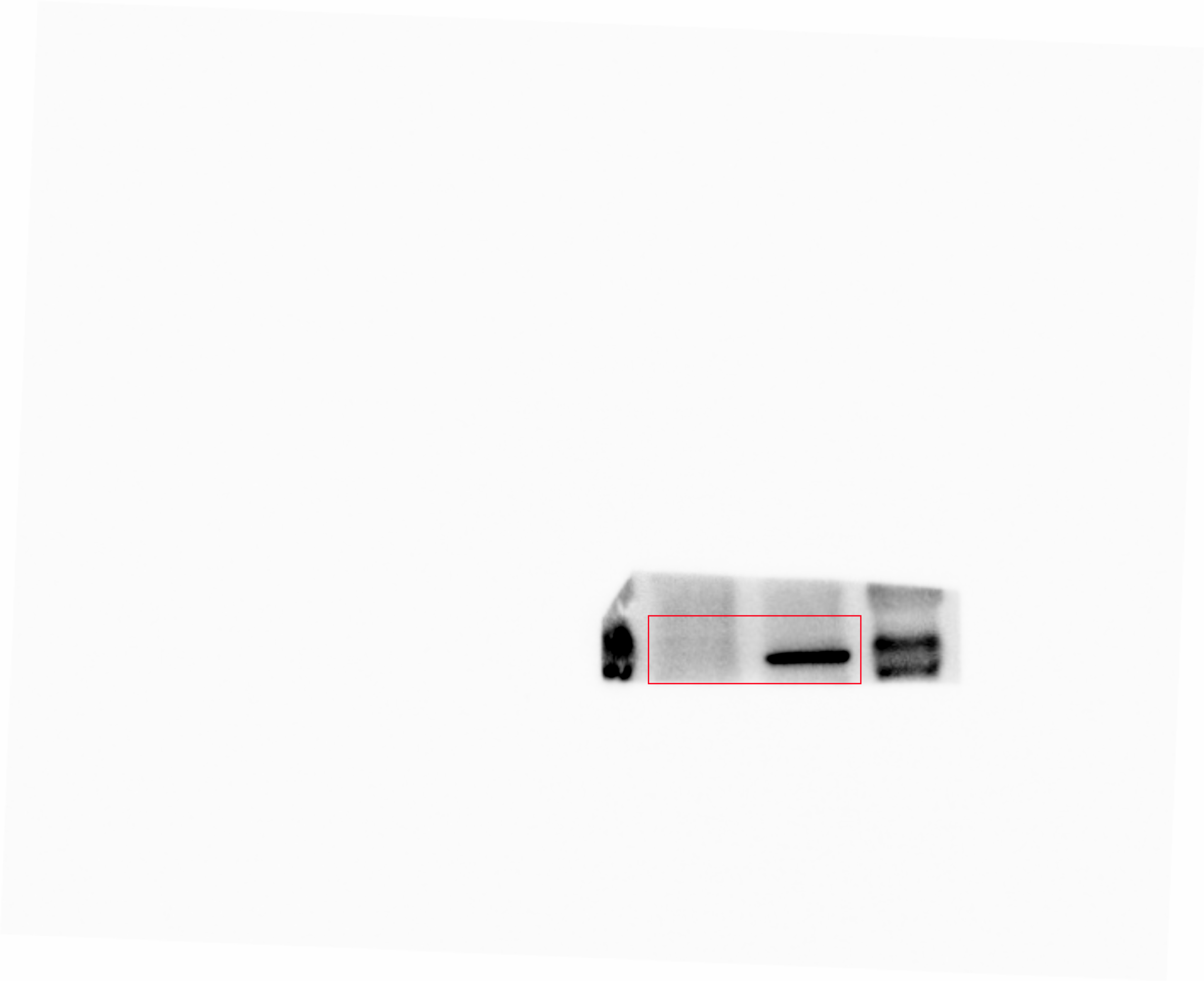

Supplement: Supplementary file 2 — Source data Fig. 1 [file 44318_2025_363_MOESM2_ESM.zip › Figure 1/1A/3 vimentin.tif]

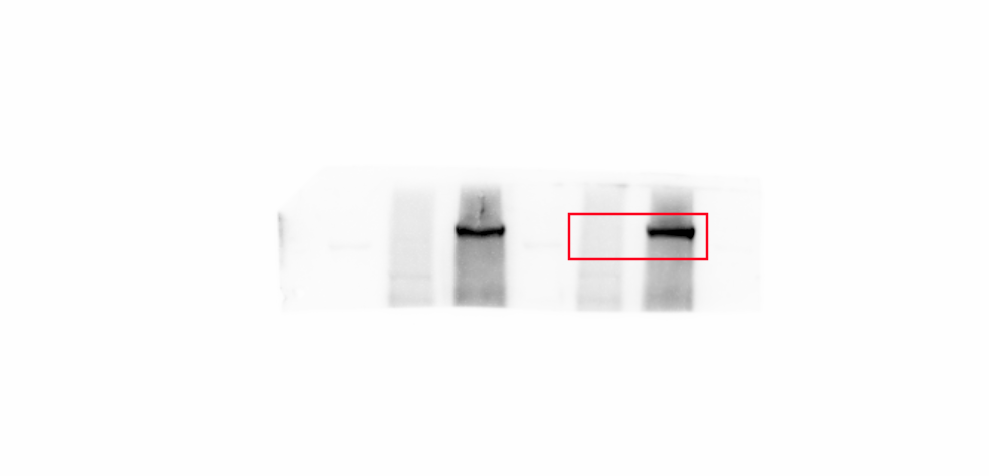

Supplement: Supplementary file 2 — Source data Fig. 1 [file 44318_2025_363_MOESM2_ESM.zip › Figure 1/1A/4 ZEB1.tif]

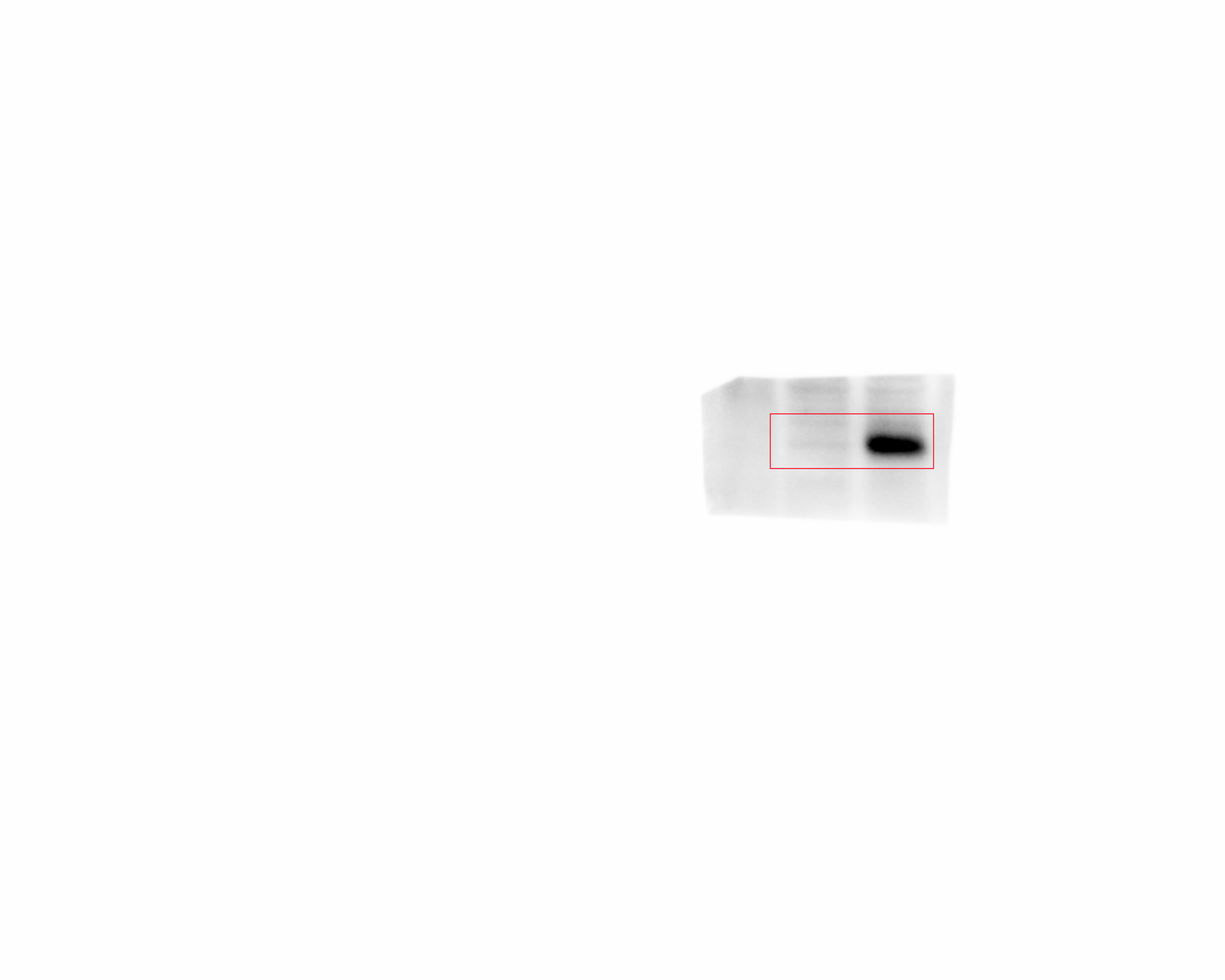

Supplement: Supplementary file 2 — Source data Fig. 1 [file 44318_2025_363_MOESM2_ESM.zip › Figure 1/1A/5 EphrinA1.tif]

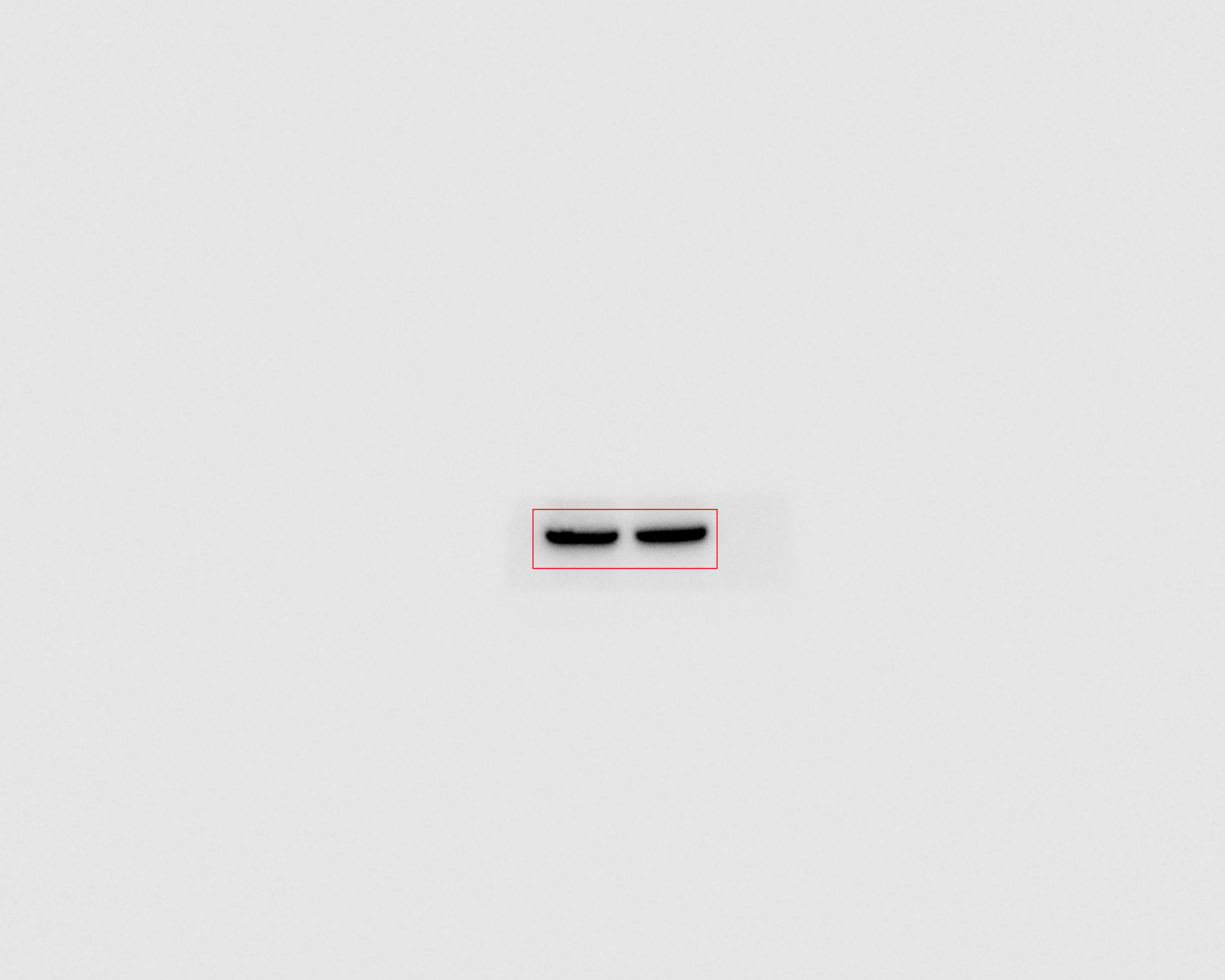

Supplement: Supplementary file 2 — Source data Fig. 1 [file 44318_2025_363_MOESM2_ESM.zip › Figure 1/1A/6 actin.tif]

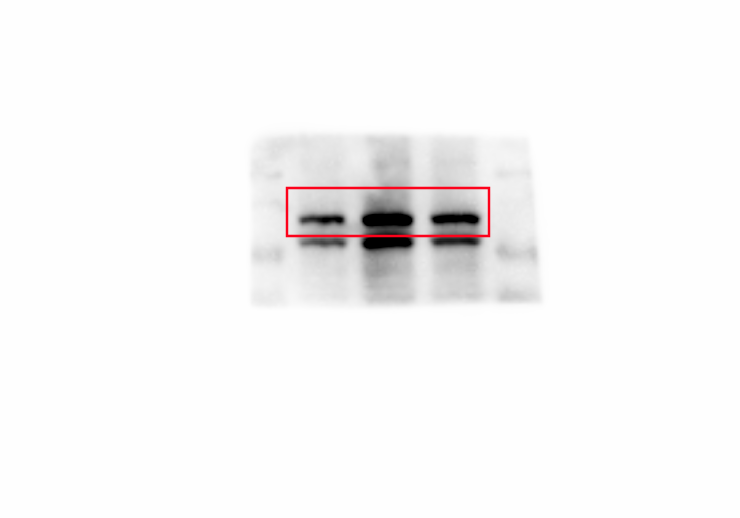

Supplement: Supplementary file 2 — Source data Fig. 1 [file 44318_2025_363_MOESM2_ESM.zip › Figure 1/1B/1 E-cad.tif]

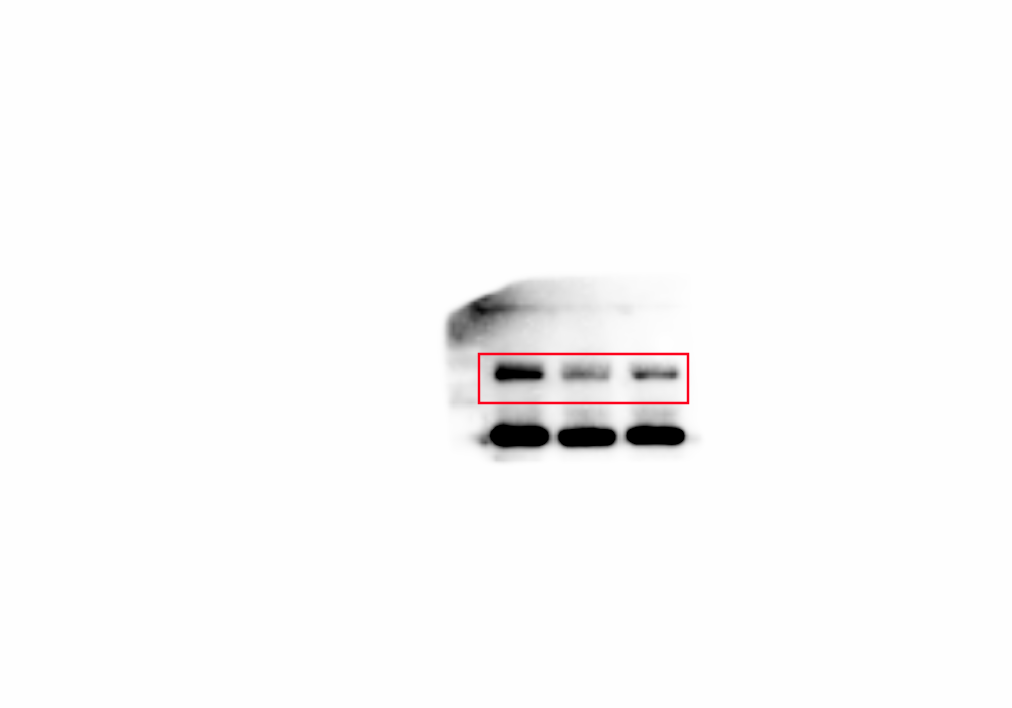

Supplement: Supplementary file 2 — Source data Fig. 1 [file 44318_2025_363_MOESM2_ESM.zip › Figure 1/1B/2 N-cad.tif]

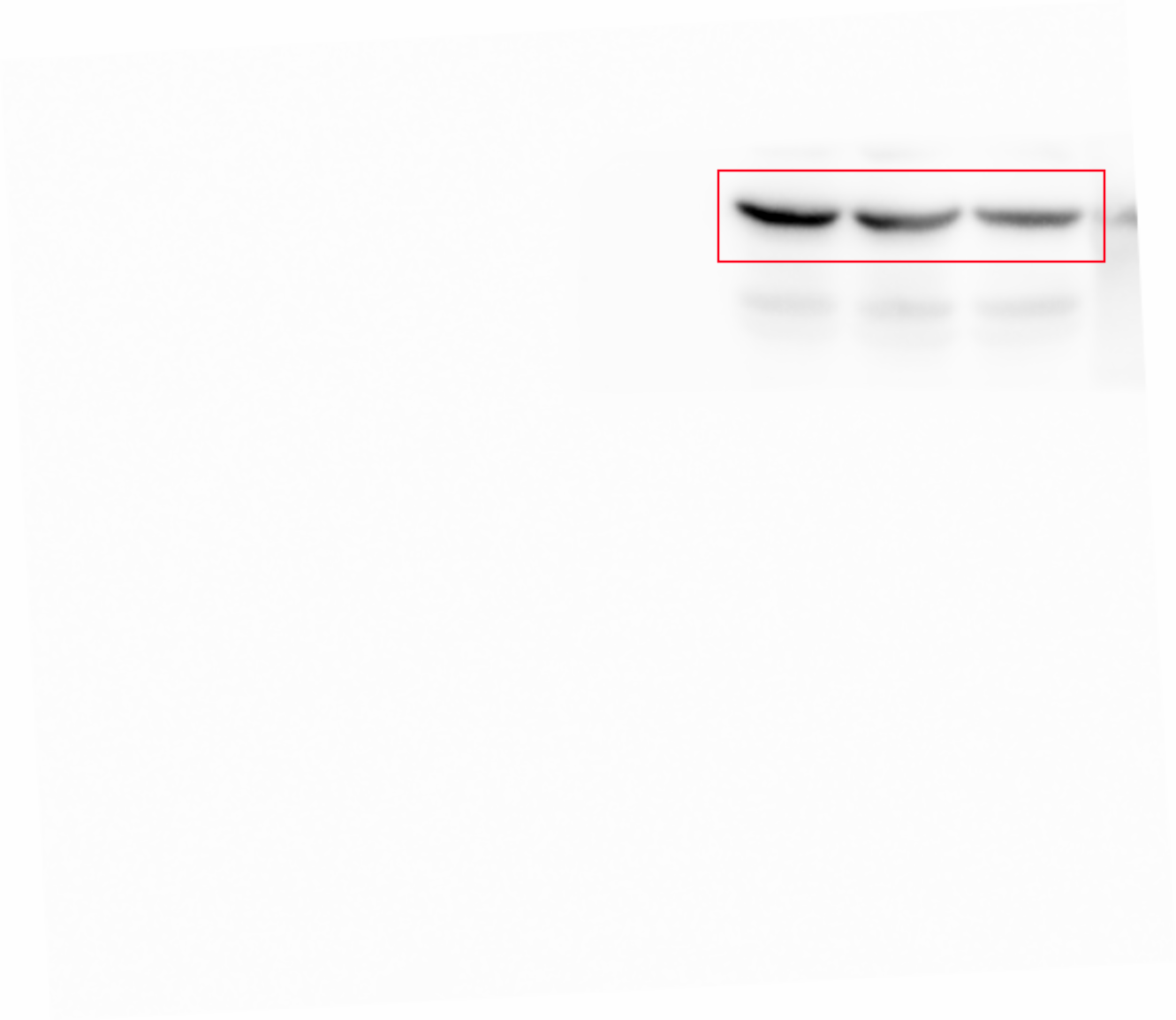

Supplement: Supplementary file 2 — Source data Fig. 1 [file 44318_2025_363_MOESM2_ESM.zip › Figure 1/1B/3 vimentin.tif]

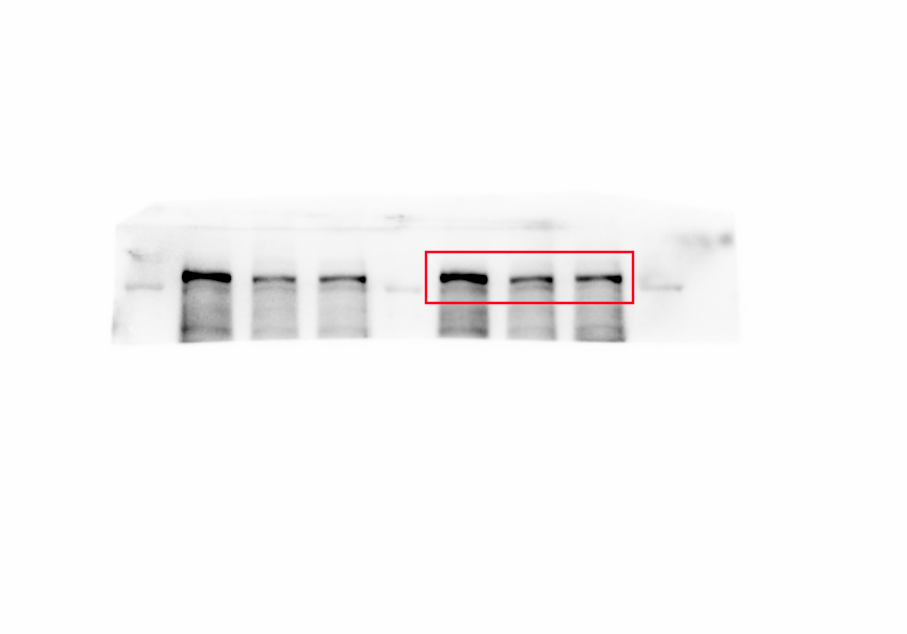

Supplement: Supplementary file 2 — Source data Fig. 1 [file 44318_2025_363_MOESM2_ESM.zip › Figure 1/1B/4 ZEB1.tif]

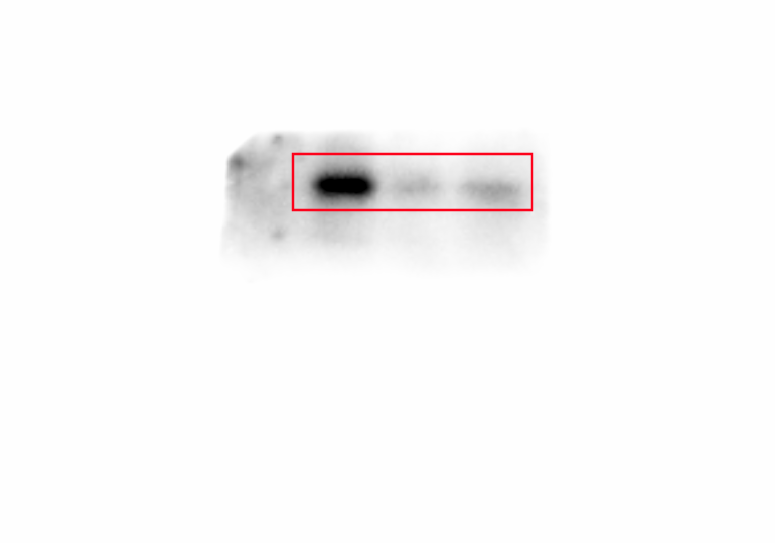

Supplement: Supplementary file 2 — Source data Fig. 1 [file 44318_2025_363_MOESM2_ESM.zip › Figure 1/1B/5 Ephrin A1.tif]

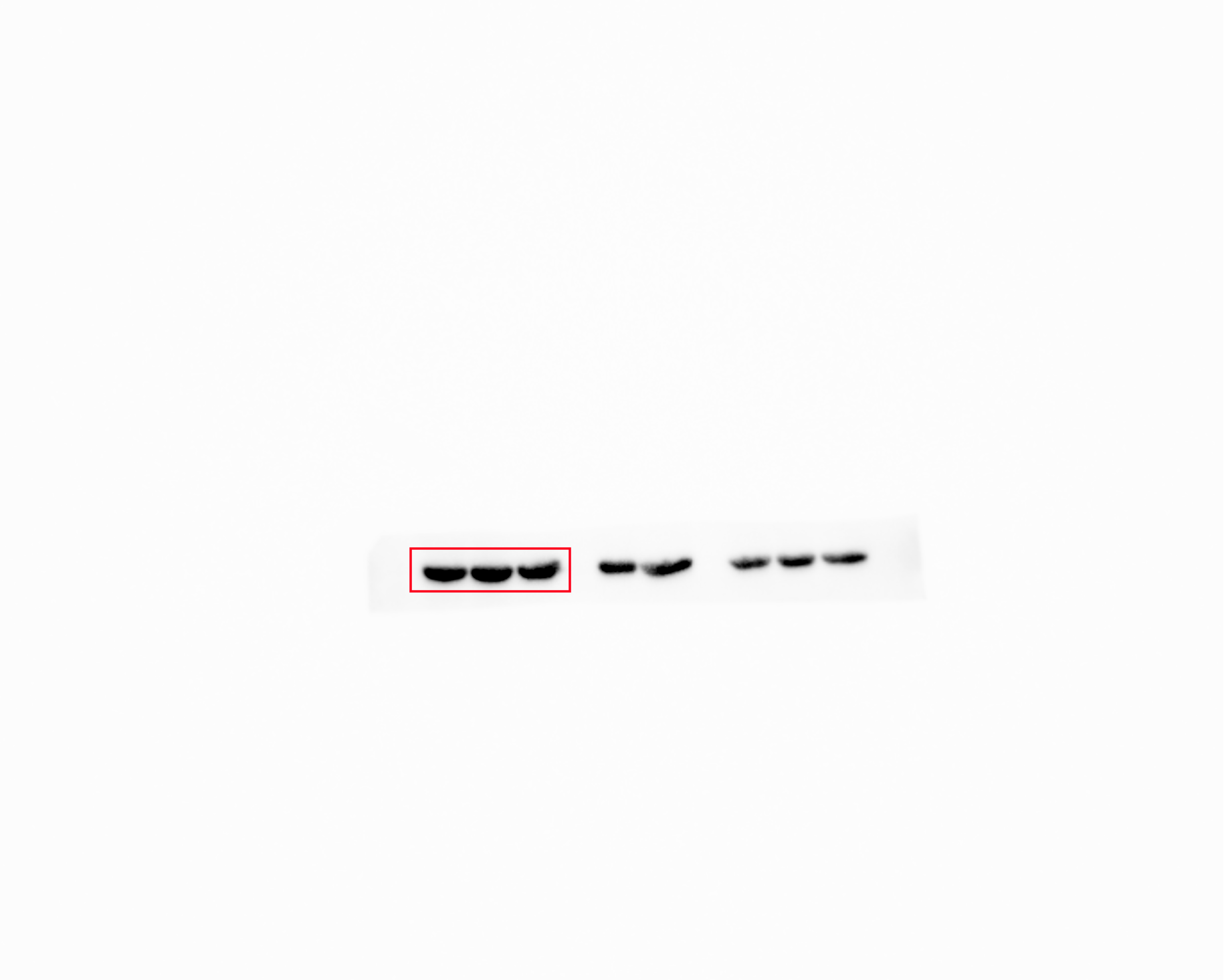

Supplement: Supplementary file 2 — Source data Fig. 1 [file 44318_2025_363_MOESM2_ESM.zip › Figure 1/1B/6 actin.tif]

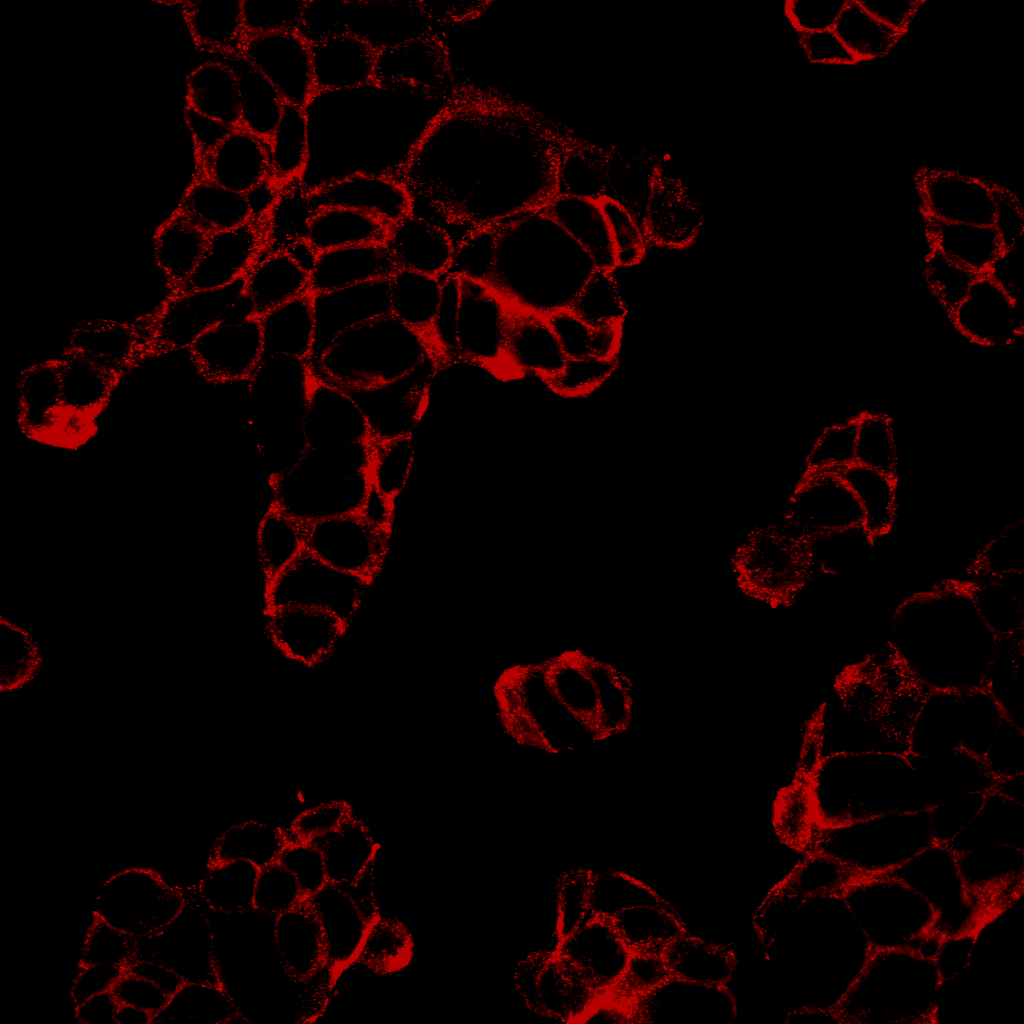

Supplement: Supplementary file 2 — Source data Fig. 1 [file 44318_2025_363_MOESM2_ESM.zip › Figure 1/1C/Control-E-cad/E-cad.tif]

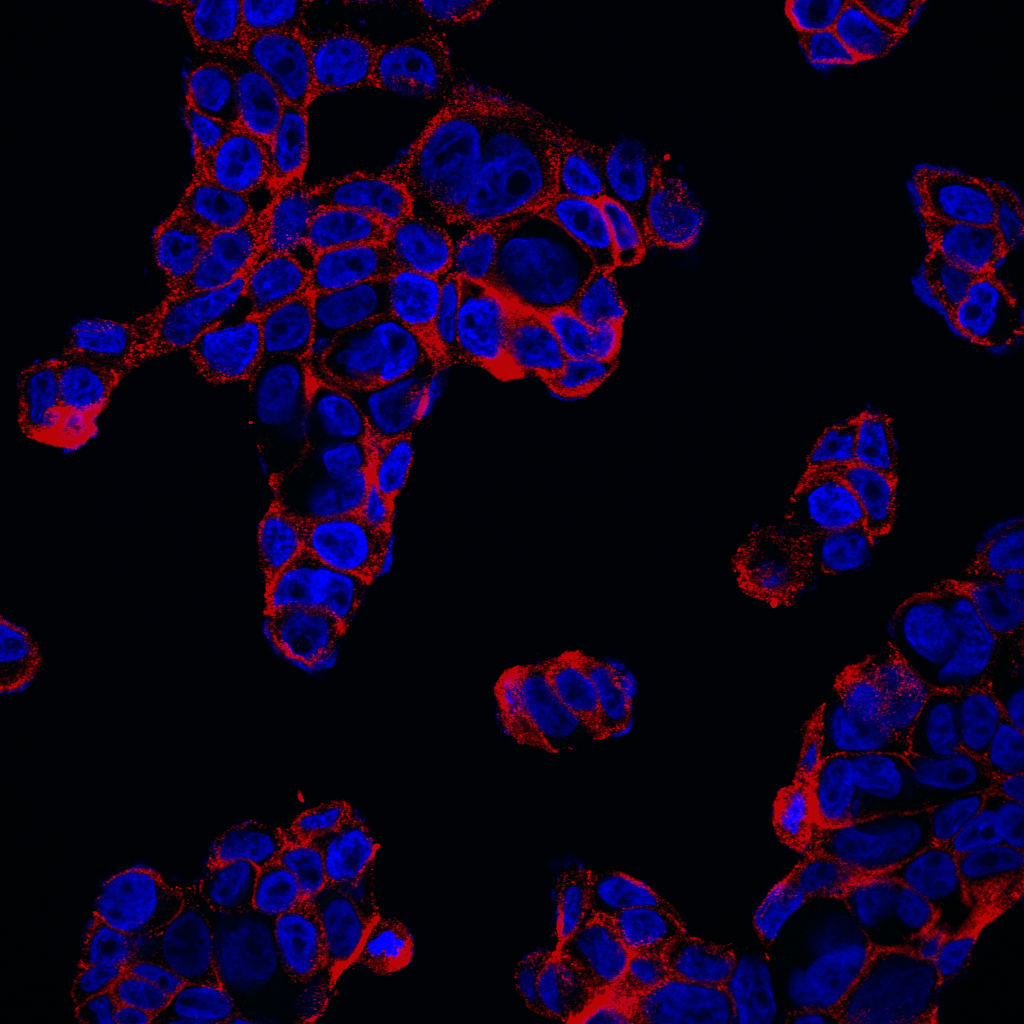

Supplement: Supplementary file 2 — Source data Fig. 1 [file 44318_2025_363_MOESM2_ESM.zip › Figure 1/1C/Control-E-cad/merge.tif]

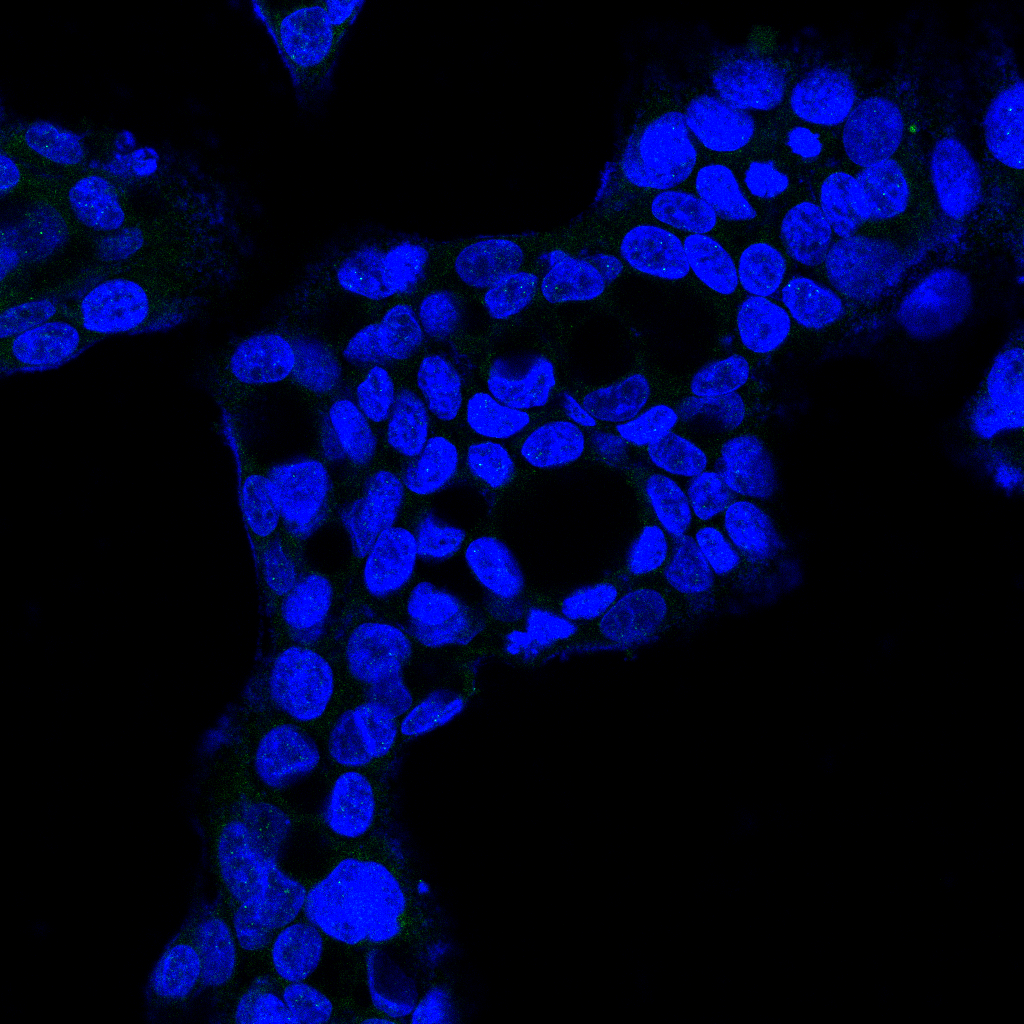

Supplement: Supplementary file 2 — Source data Fig. 1 [file 44318_2025_363_MOESM2_ESM.zip › Figure 1/1C/Control-N-cad/merge.tif]

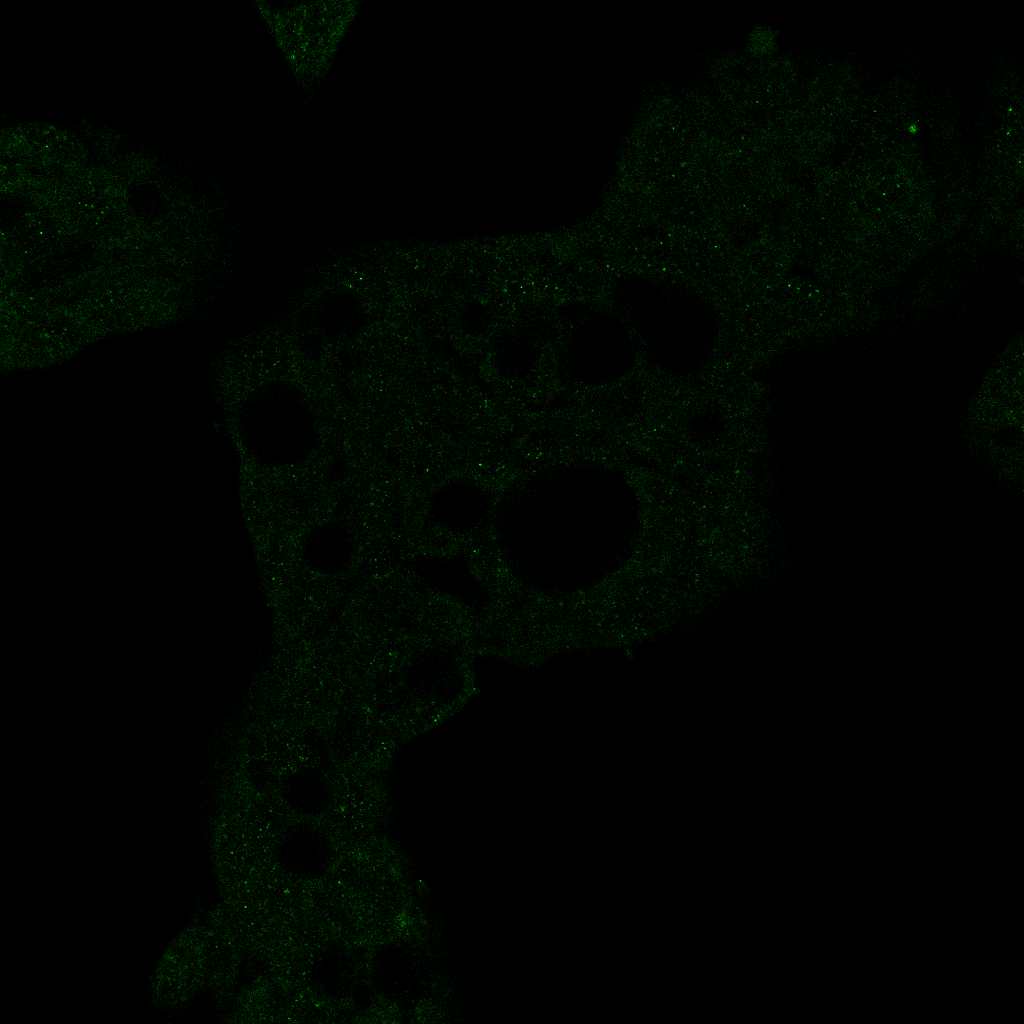

Supplement: Supplementary file 2 — Source data Fig. 1 [file 44318_2025_363_MOESM2_ESM.zip › Figure 1/1C/Control-N-cad/N-cad.tif]

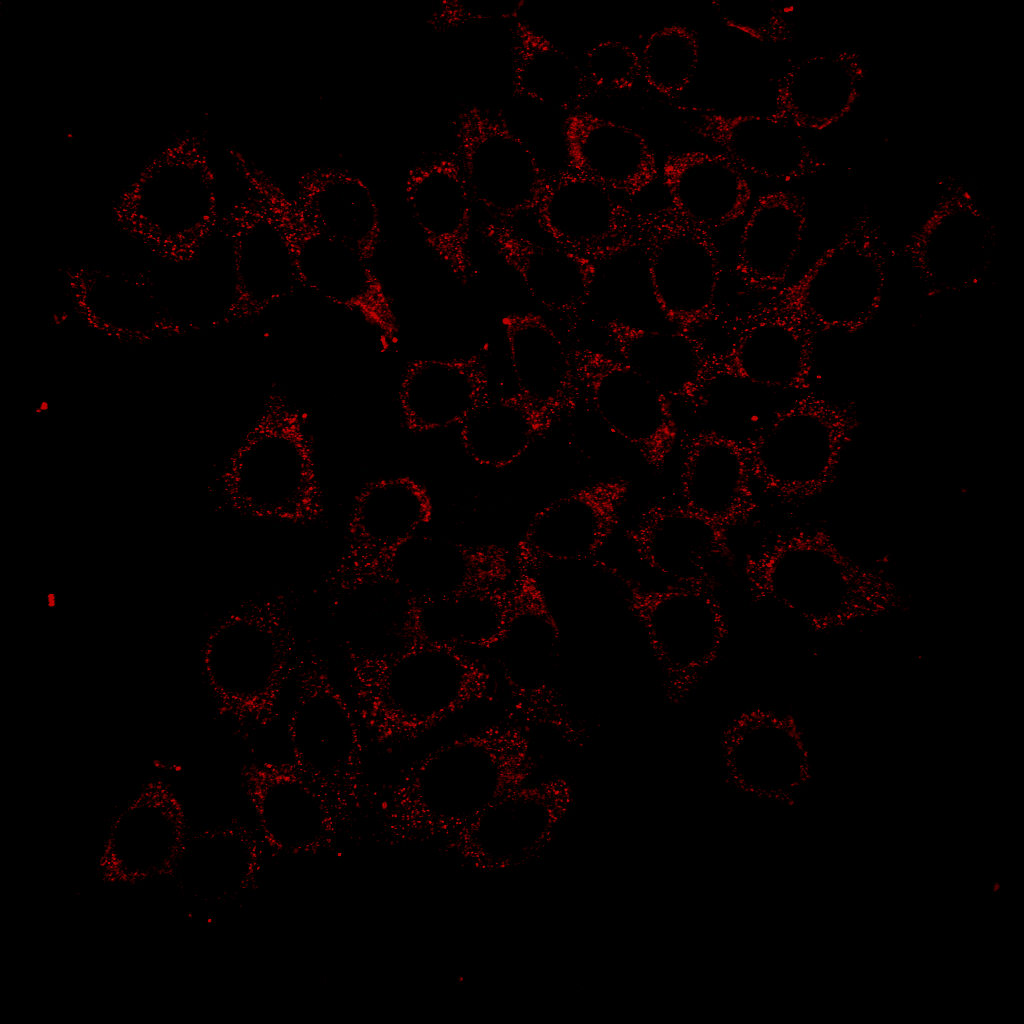

Supplement: Supplementary file 2 — Source data Fig. 1 [file 44318_2025_363_MOESM2_ESM.zip › Figure 1/1C/Ephrin A1-E-cad/E-cad.tif]

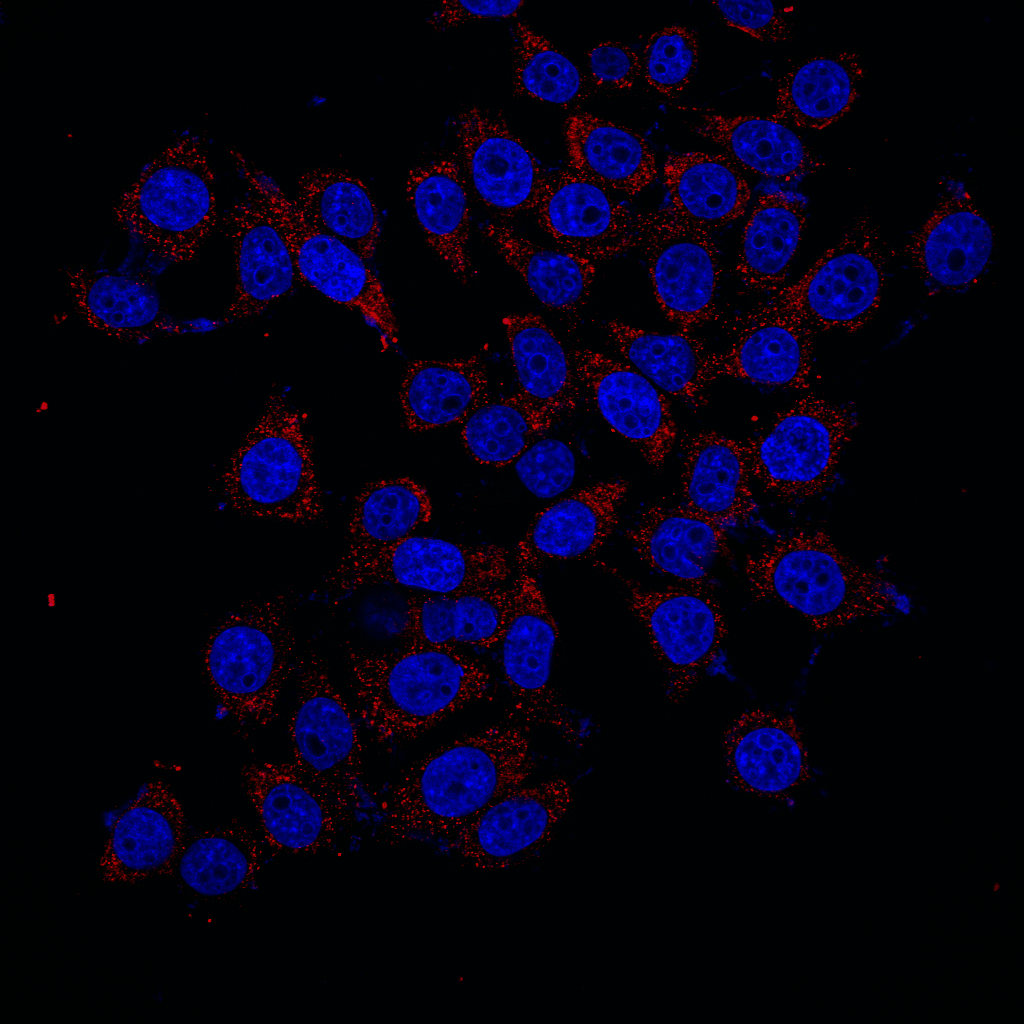

Supplement: Supplementary file 2 — Source data Fig. 1 [file 44318_2025_363_MOESM2_ESM.zip › Figure 1/1C/Ephrin A1-E-cad/merge.tif]

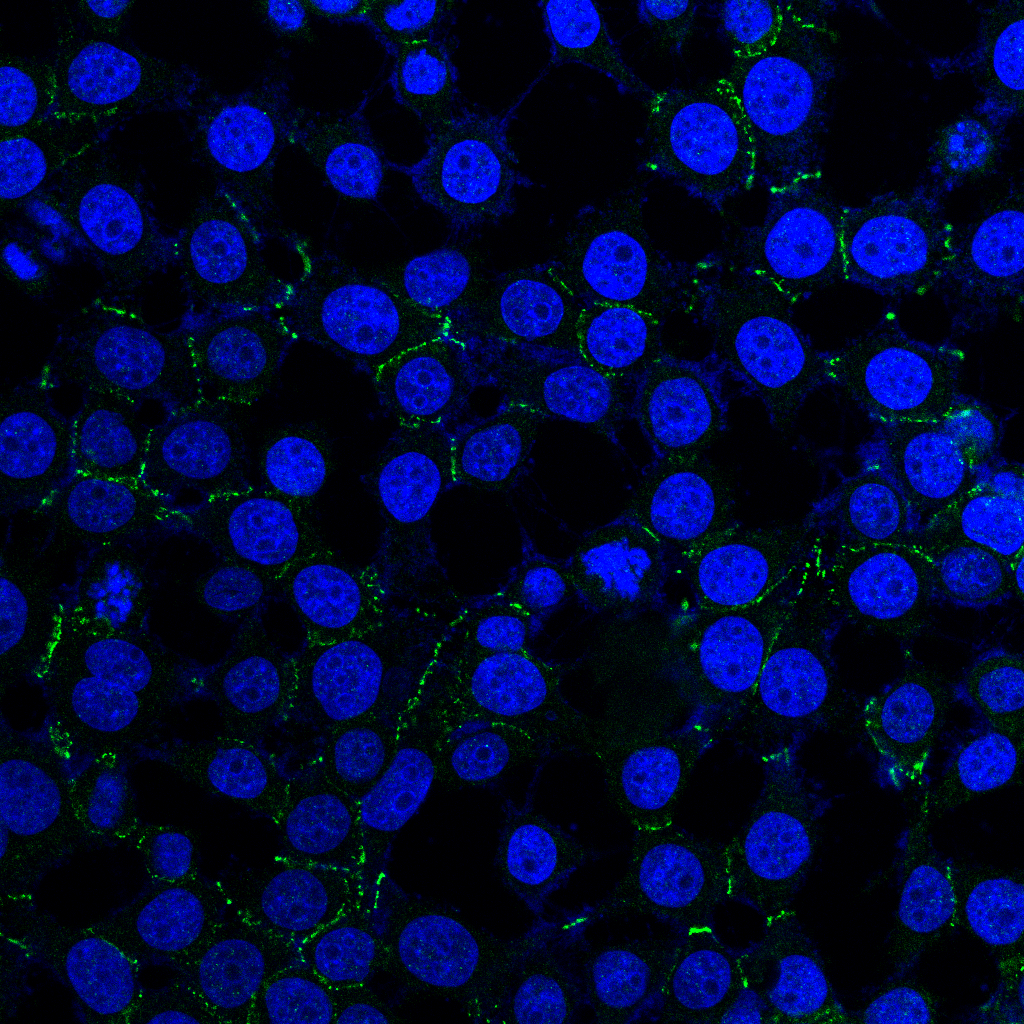

Supplement: Supplementary file 2 — Source data Fig. 1 [file 44318_2025_363_MOESM2_ESM.zip › Figure 1/1C/Ephrin A1-N-cad/merge.tif]

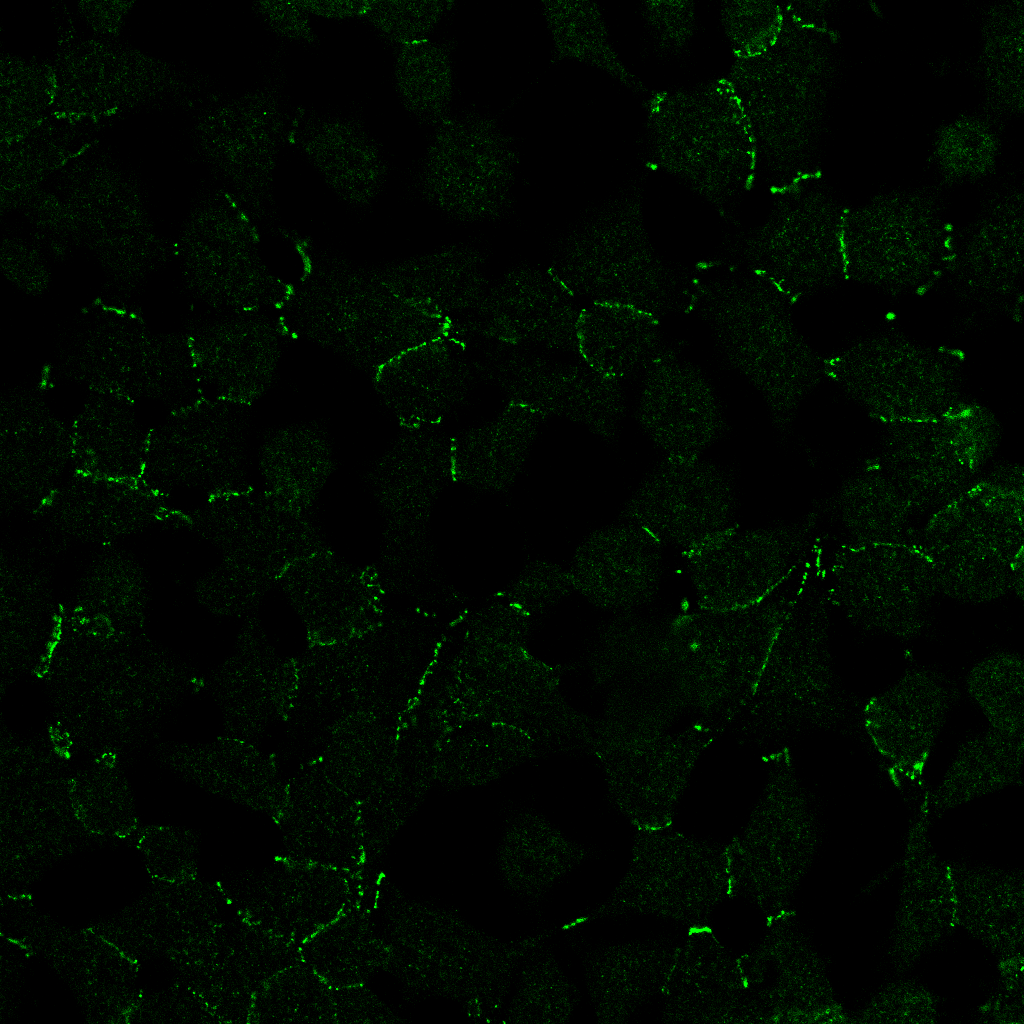

Supplement: Supplementary file 2 — Source data Fig. 1 [file 44318_2025_363_MOESM2_ESM.zip › Figure 1/1C/Ephrin A1-N-cad/N-cad.tif]

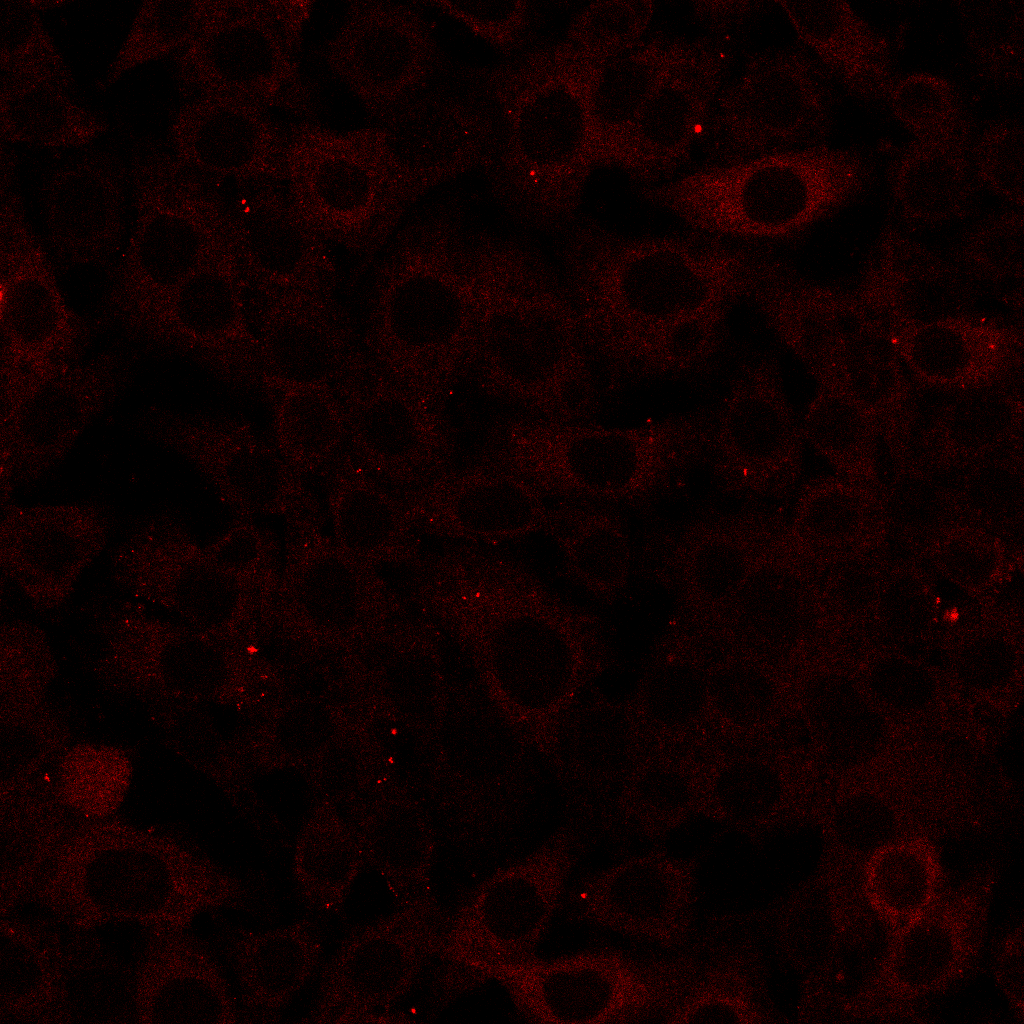

Supplement: Supplementary file 2 — Source data Fig. 1 [file 44318_2025_363_MOESM2_ESM.zip › Figure 1/1D/siControl-E-cad/E-cad.tif]

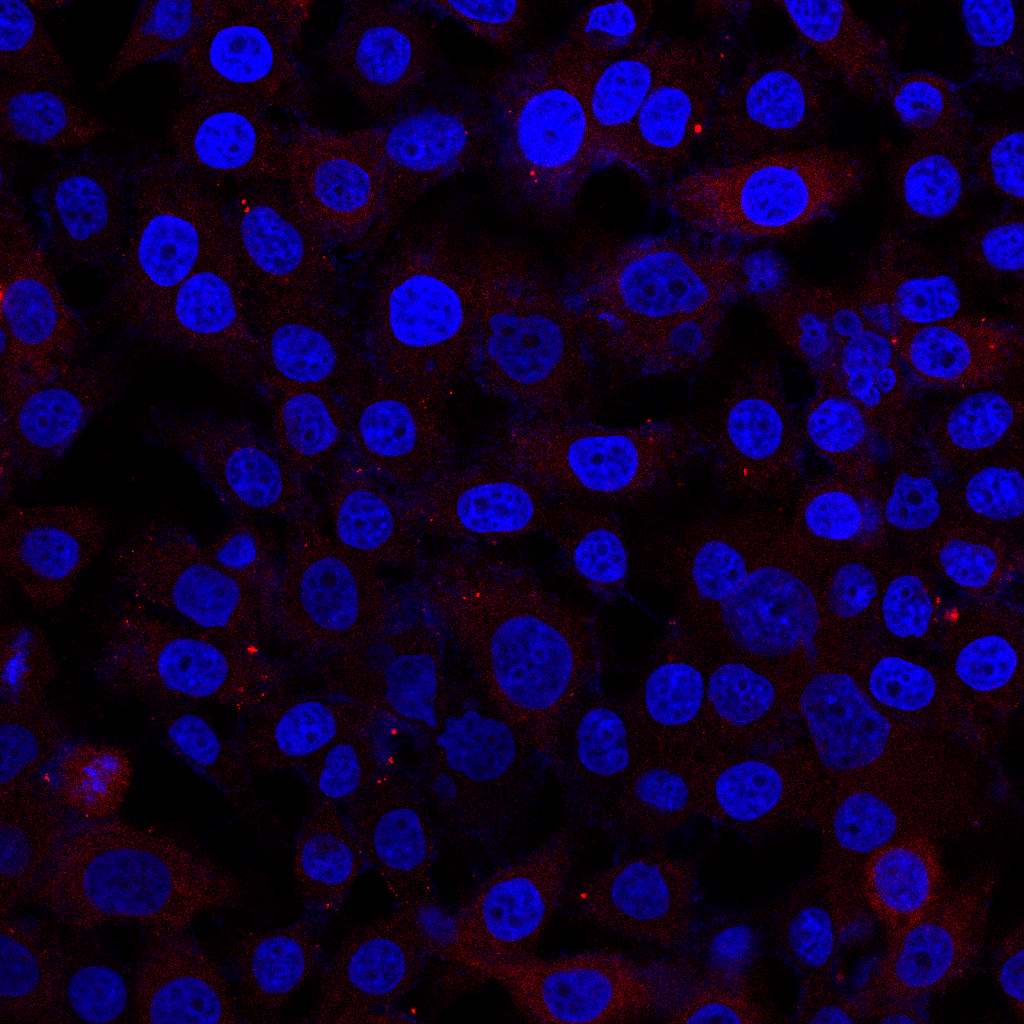

Supplement: Supplementary file 2 — Source data Fig. 1 [file 44318_2025_363_MOESM2_ESM.zip › Figure 1/1D/siControl-E-cad/merge.tif]

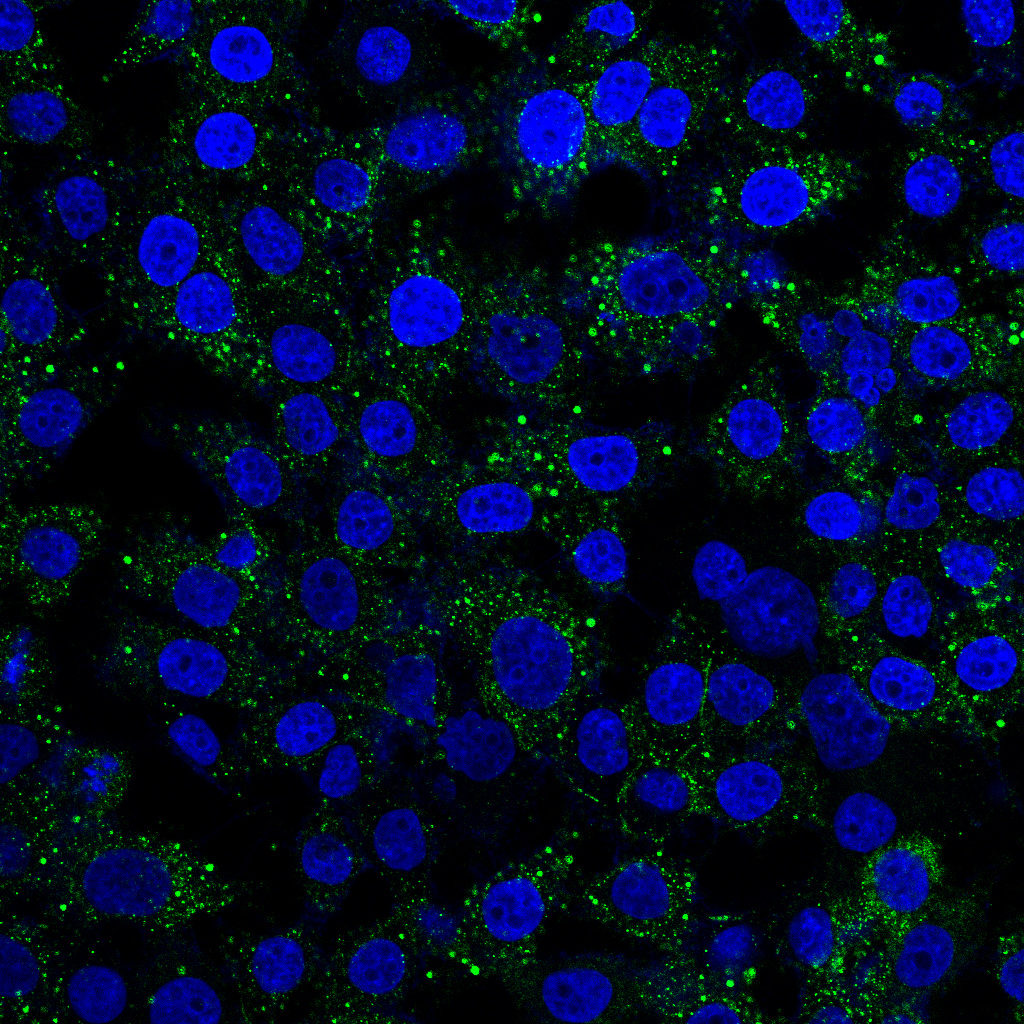

Supplement: Supplementary file 2 — Source data Fig. 1 [file 44318_2025_363_MOESM2_ESM.zip › Figure 1/1D/siControl-N-cad/merge.tif]

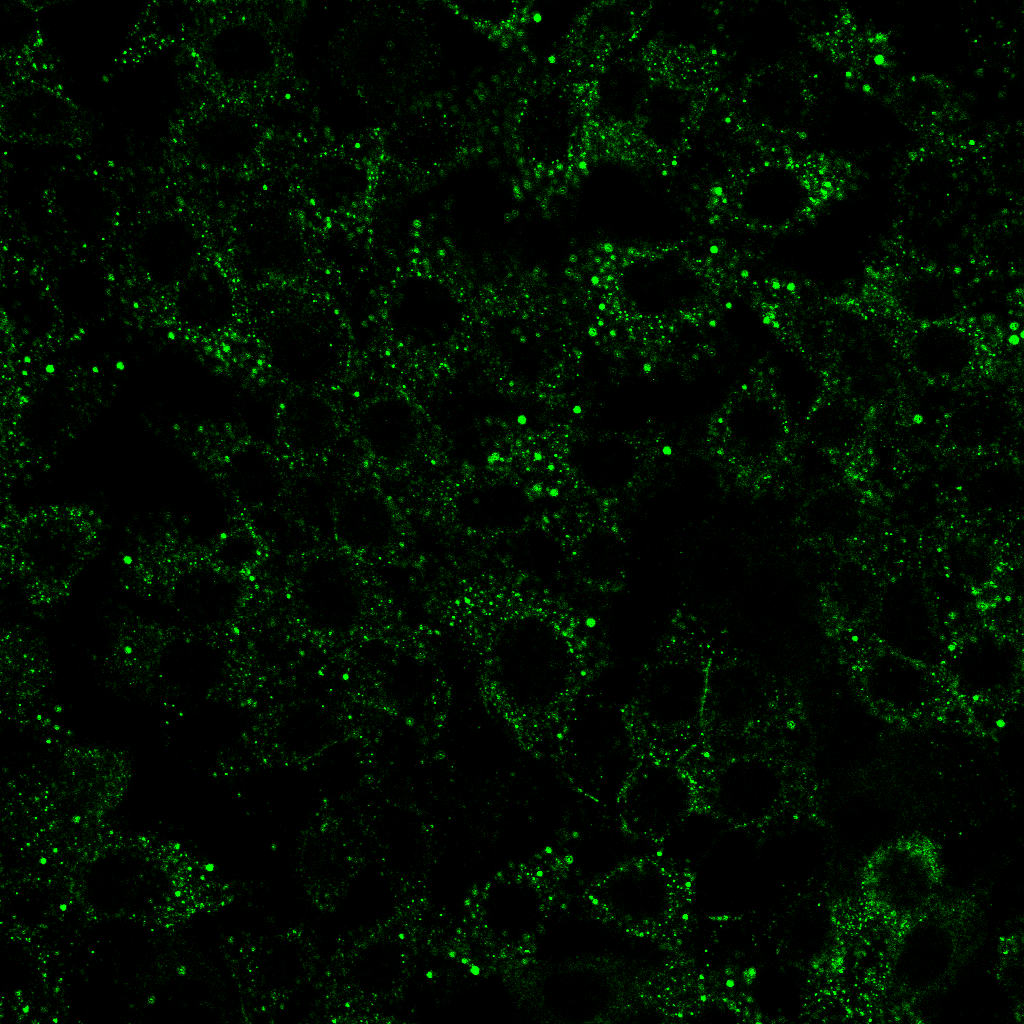

Supplement: Supplementary file 2 — Source data Fig. 1 [file 44318_2025_363_MOESM2_ESM.zip › Figure 1/1D/siControl-N-cad/N-cad.tif]

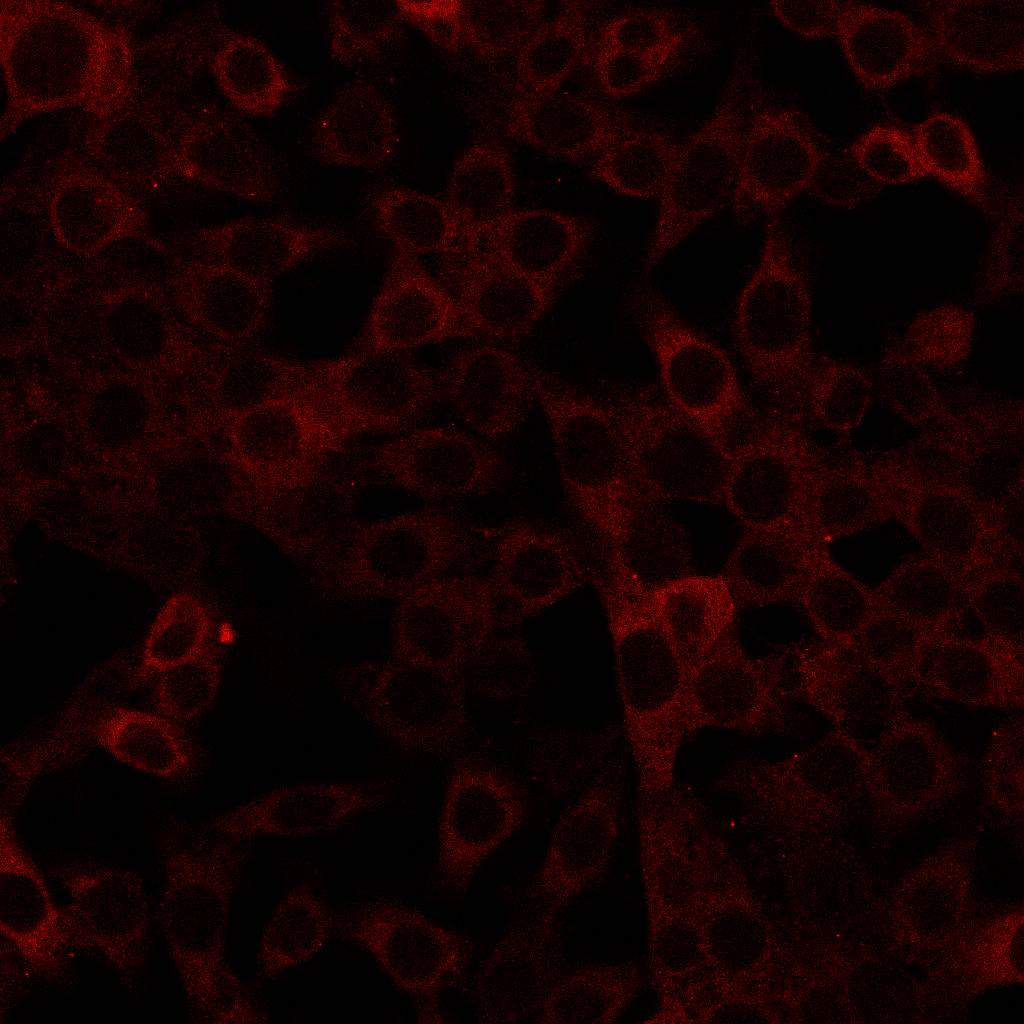

Supplement: Supplementary file 2 — Source data Fig. 1 [file 44318_2025_363_MOESM2_ESM.zip › Figure 1/1D/siEphrin A1-1-E-cad/E-cad.tif]

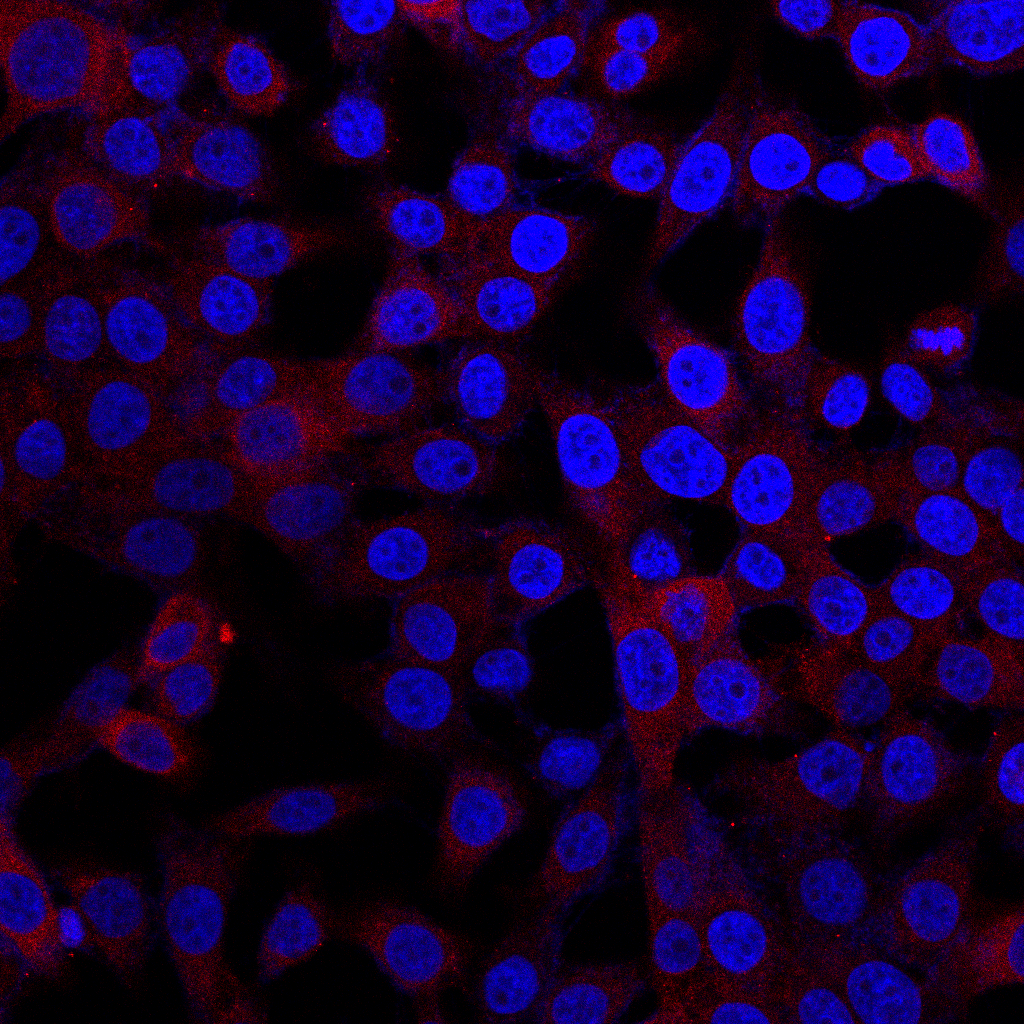

Supplement: Supplementary file 2 — Source data Fig. 1 [file 44318_2025_363_MOESM2_ESM.zip › Figure 1/1D/siEphrin A1-1-E-cad/merge.tif]

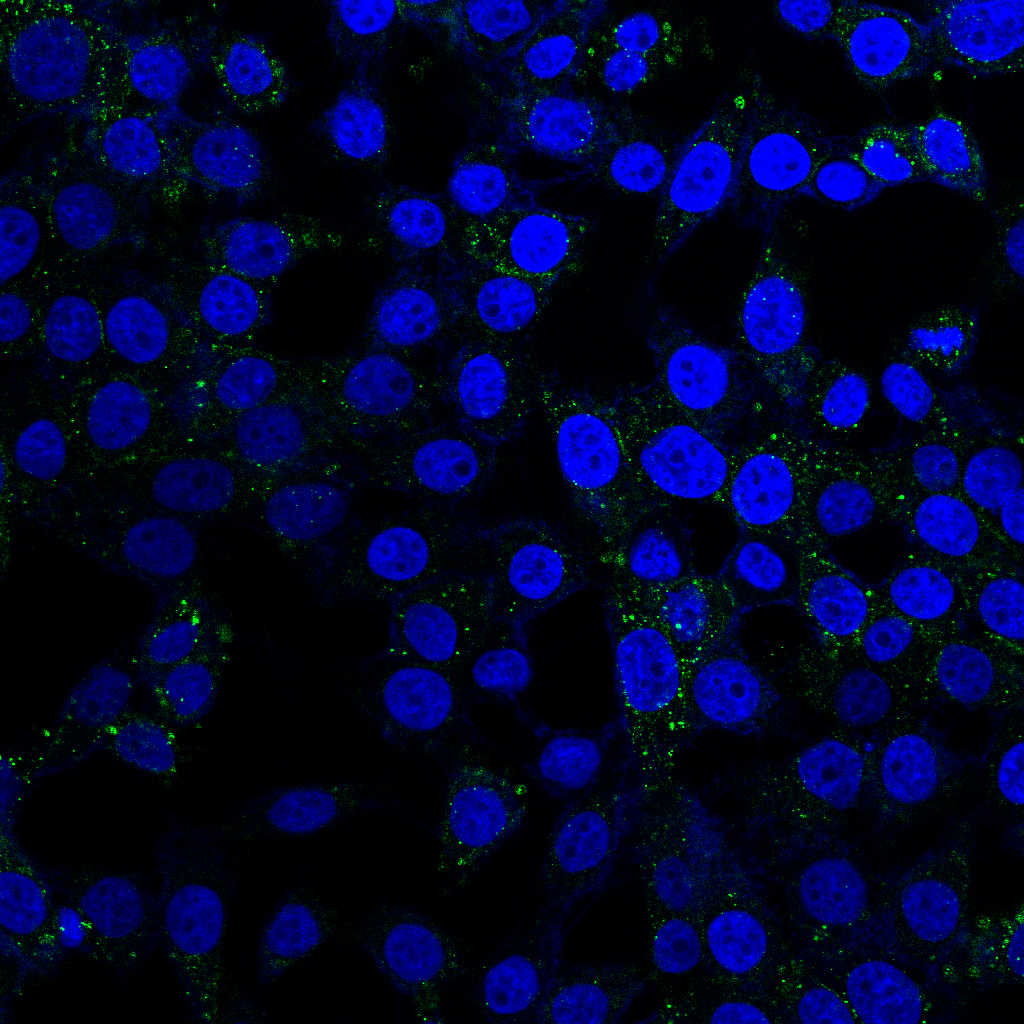

Supplement: Supplementary file 2 — Source data Fig. 1 [file 44318_2025_363_MOESM2_ESM.zip › Figure 1/1D/siEphrin A1-1-N-cad/merge.tif]

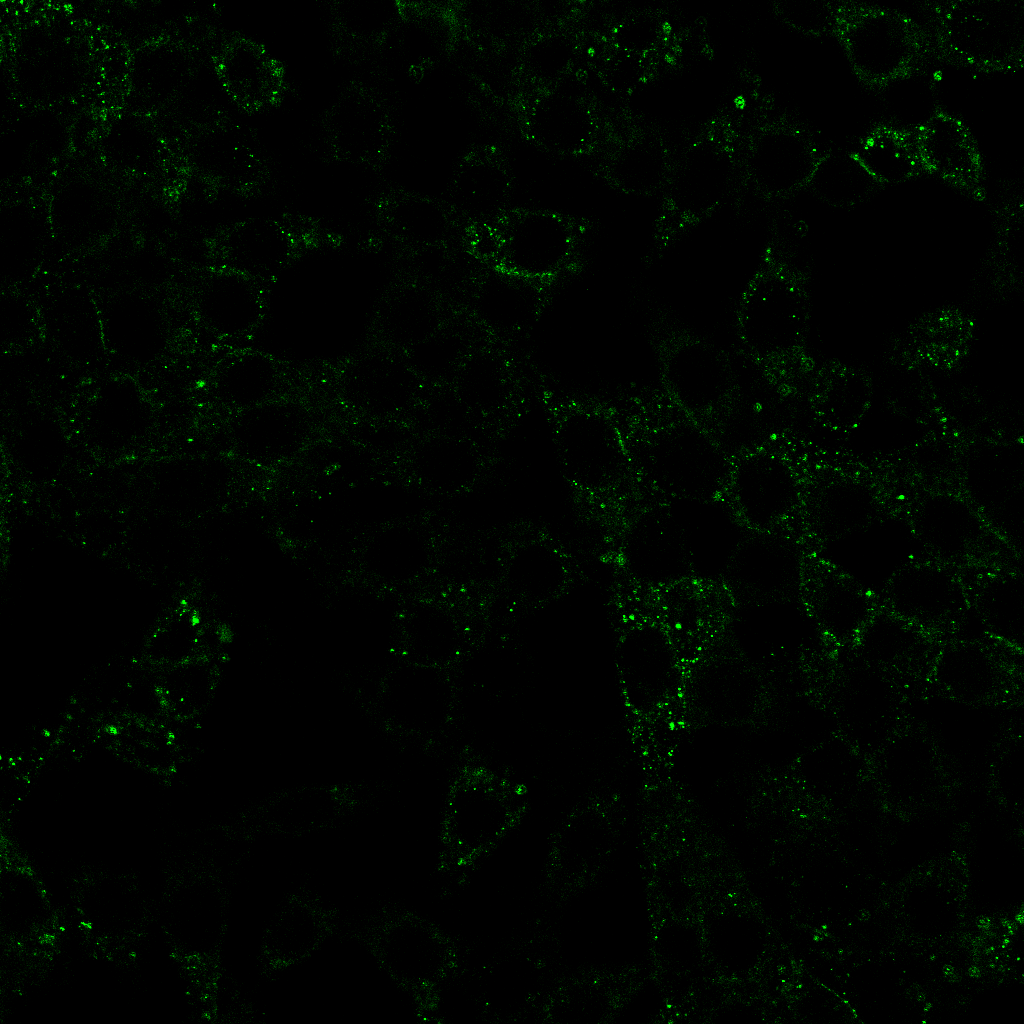

Supplement: Supplementary file 2 — Source data Fig. 1 [file 44318_2025_363_MOESM2_ESM.zip › Figure 1/1D/siEphrin A1-1-N-cad/N-cad.tif]

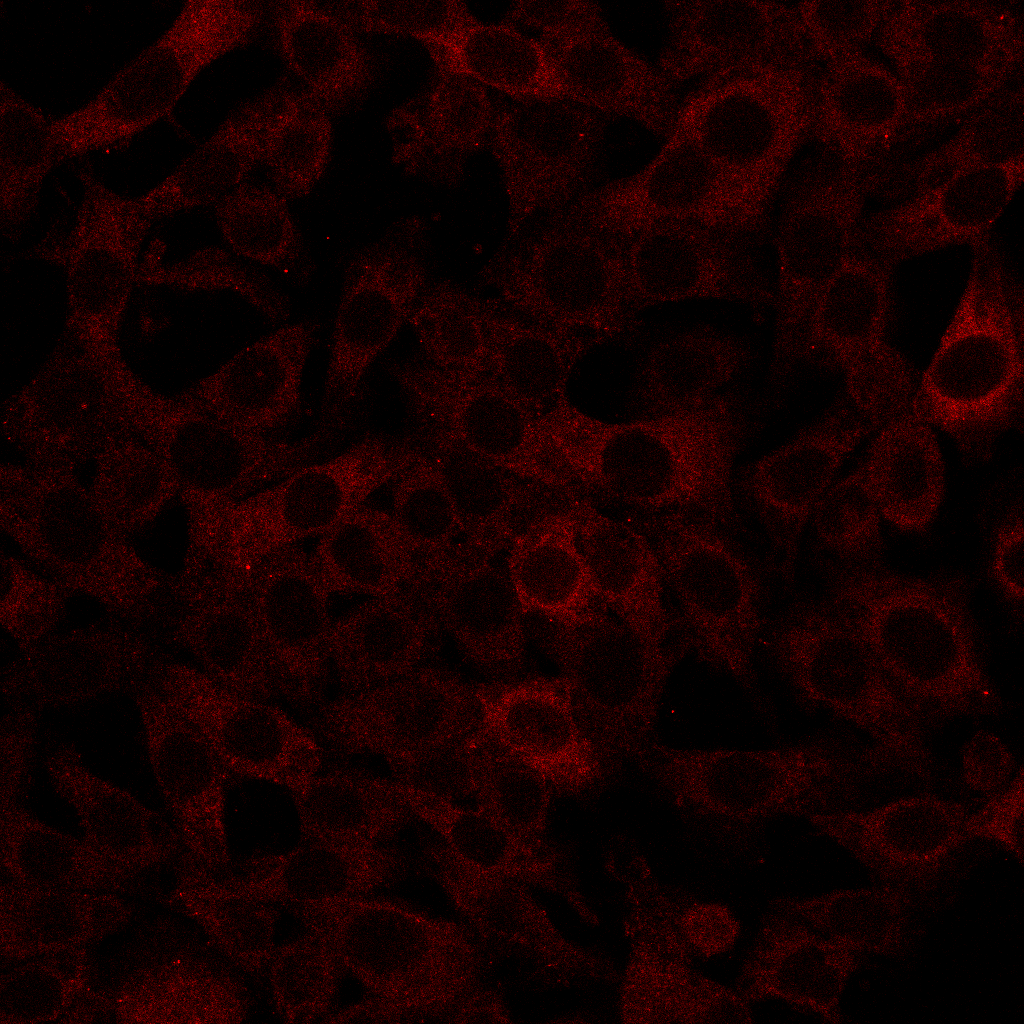

Supplement: Supplementary file 2 — Source data Fig. 1 [file 44318_2025_363_MOESM2_ESM.zip › Figure 1/1D/siEphrin A1-2-E-cad/E-cad.tif]

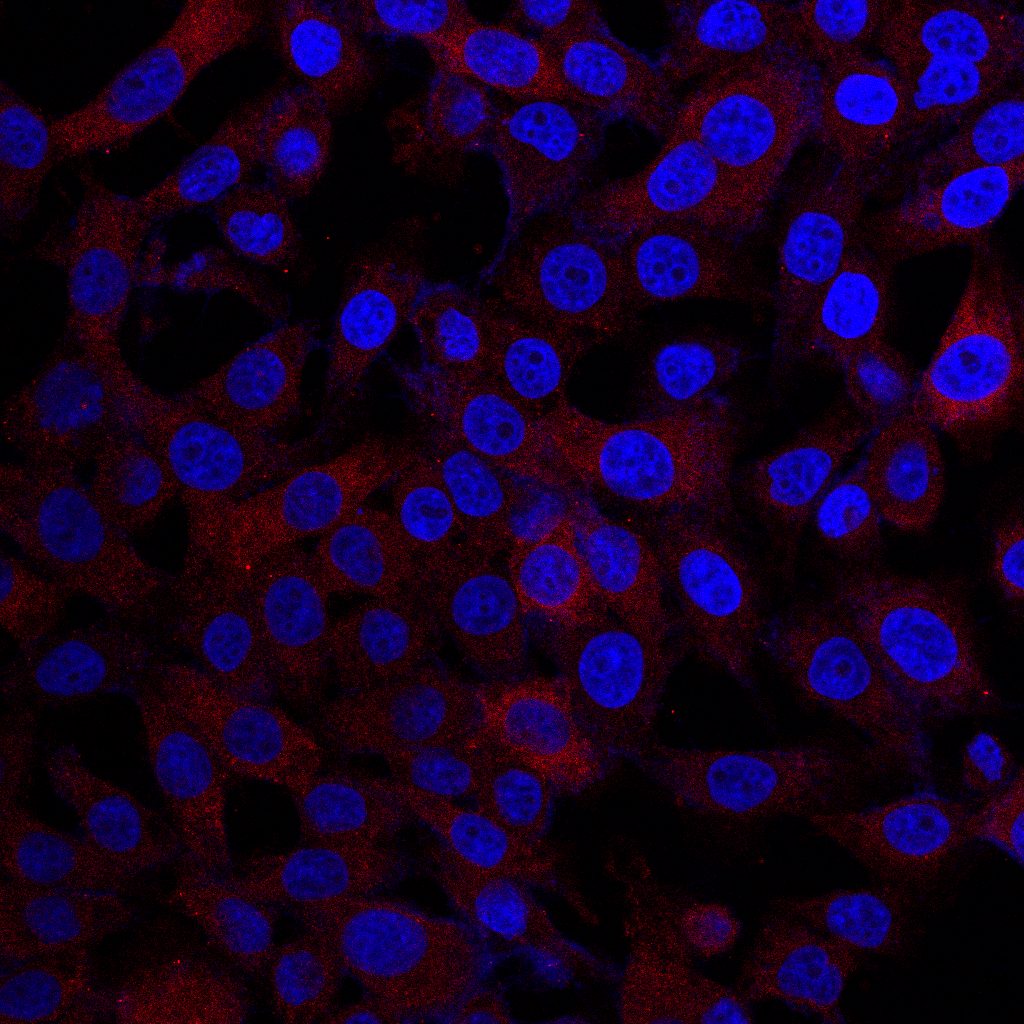

Supplement: Supplementary file 2 — Source data Fig. 1 [file 44318_2025_363_MOESM2_ESM.zip › Figure 1/1D/siEphrin A1-2-E-cad/merge.tif]

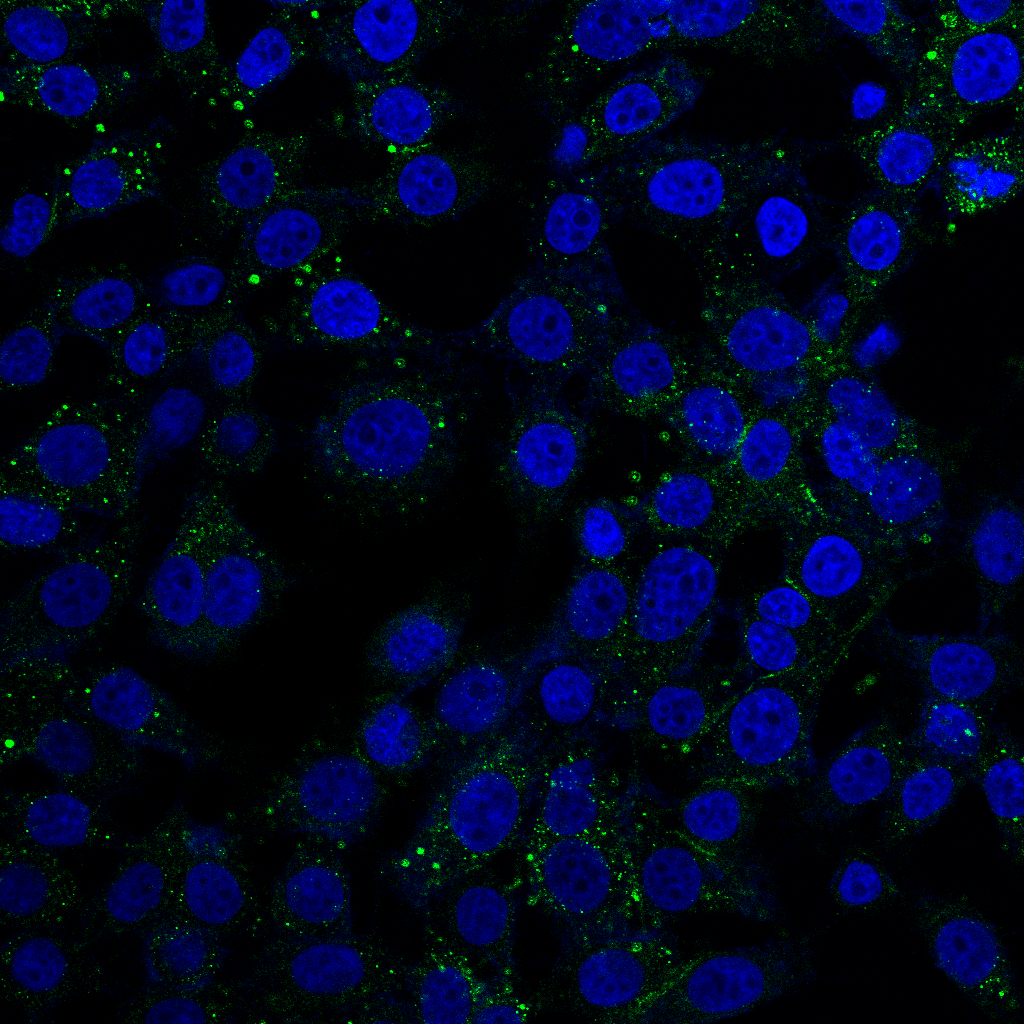

Supplement: Supplementary file 2 — Source data Fig. 1 [file 44318_2025_363_MOESM2_ESM.zip › Figure 1/1D/siEphrin A1-2-N-cad/merge.tif]

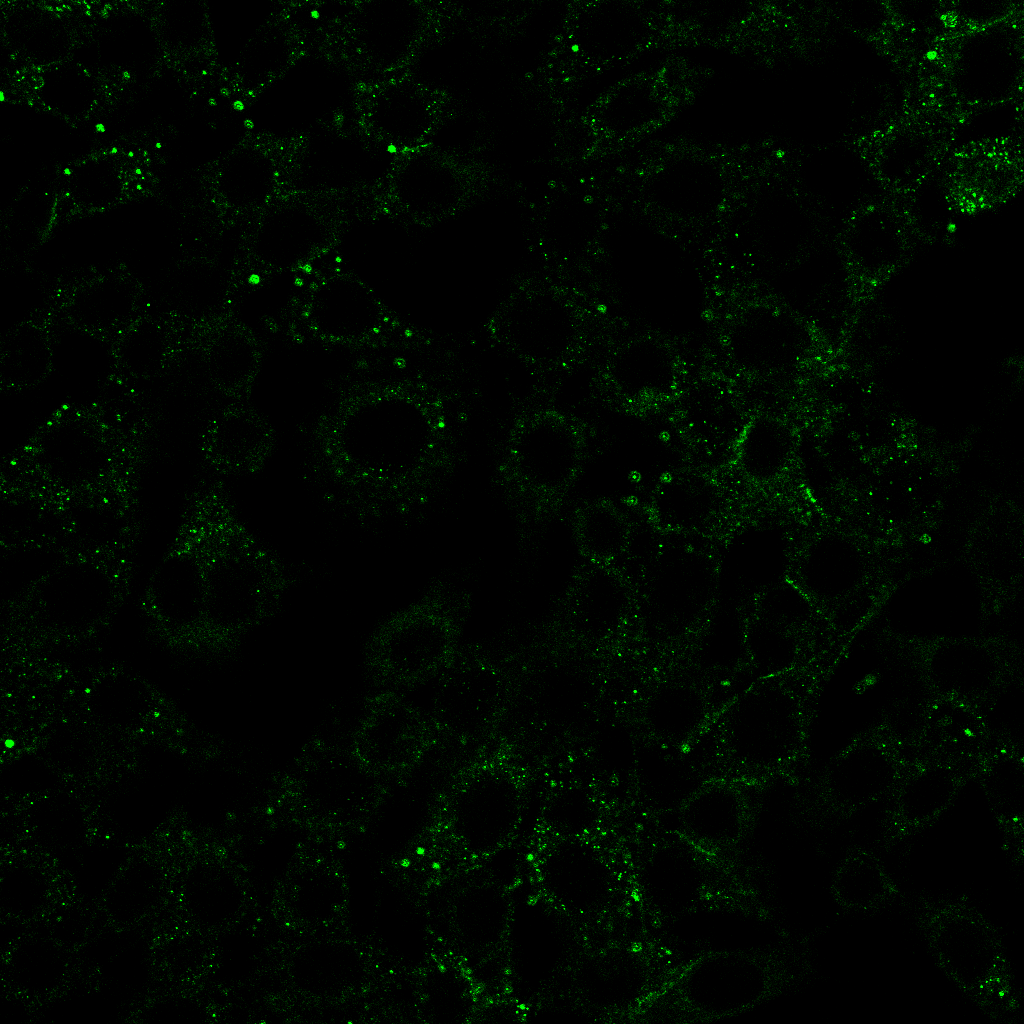

Supplement: Supplementary file 2 — Source data Fig. 1 [file 44318_2025_363_MOESM2_ESM.zip › Figure 1/1D/siEphrin A1-2-N-cad/N-cad.tif]

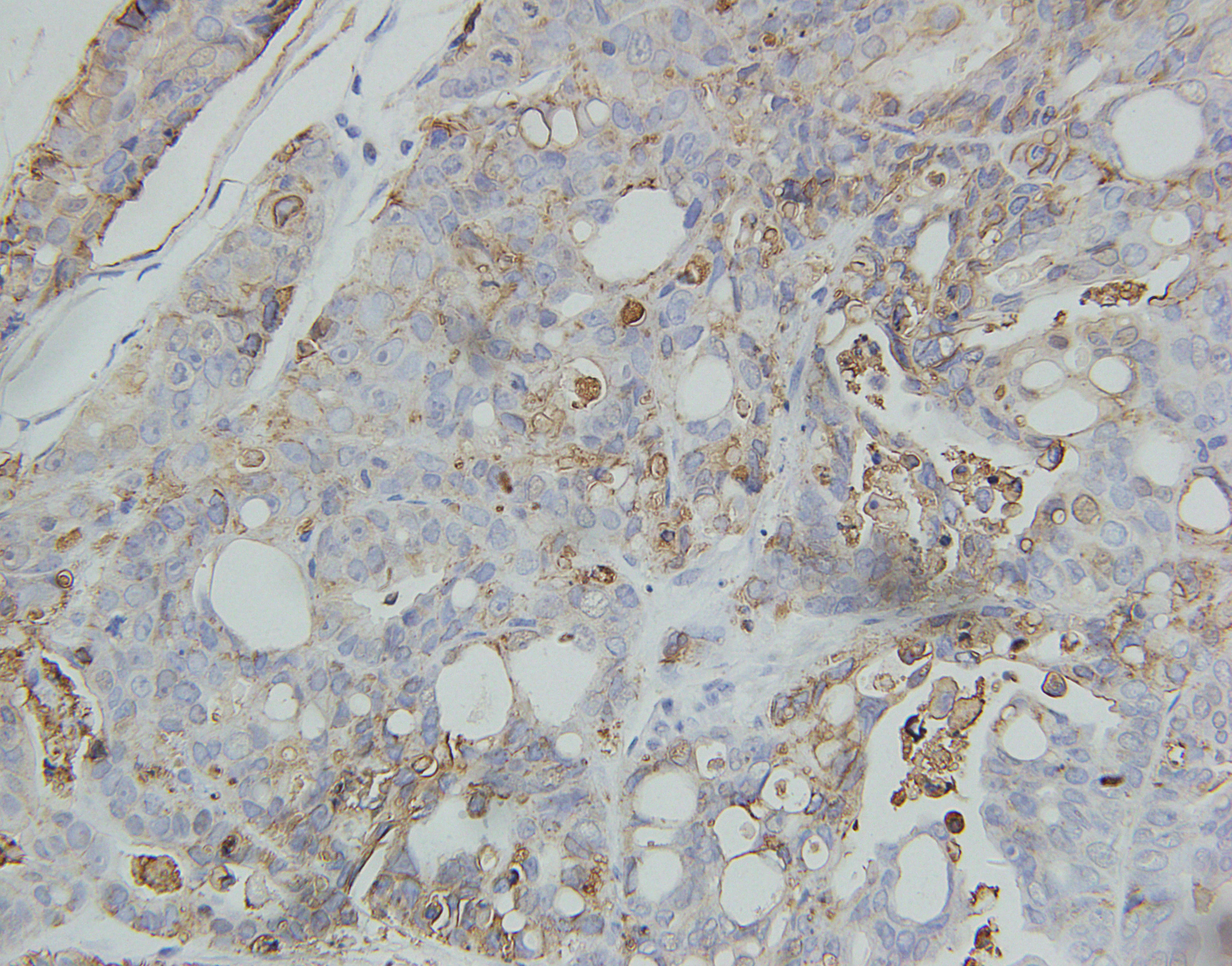

Supplement: Supplementary file 2 — Source data Fig. 1 [file 44318_2025_363_MOESM2_ESM.zip › Figure 1/1E/Control-E-cad.tif]

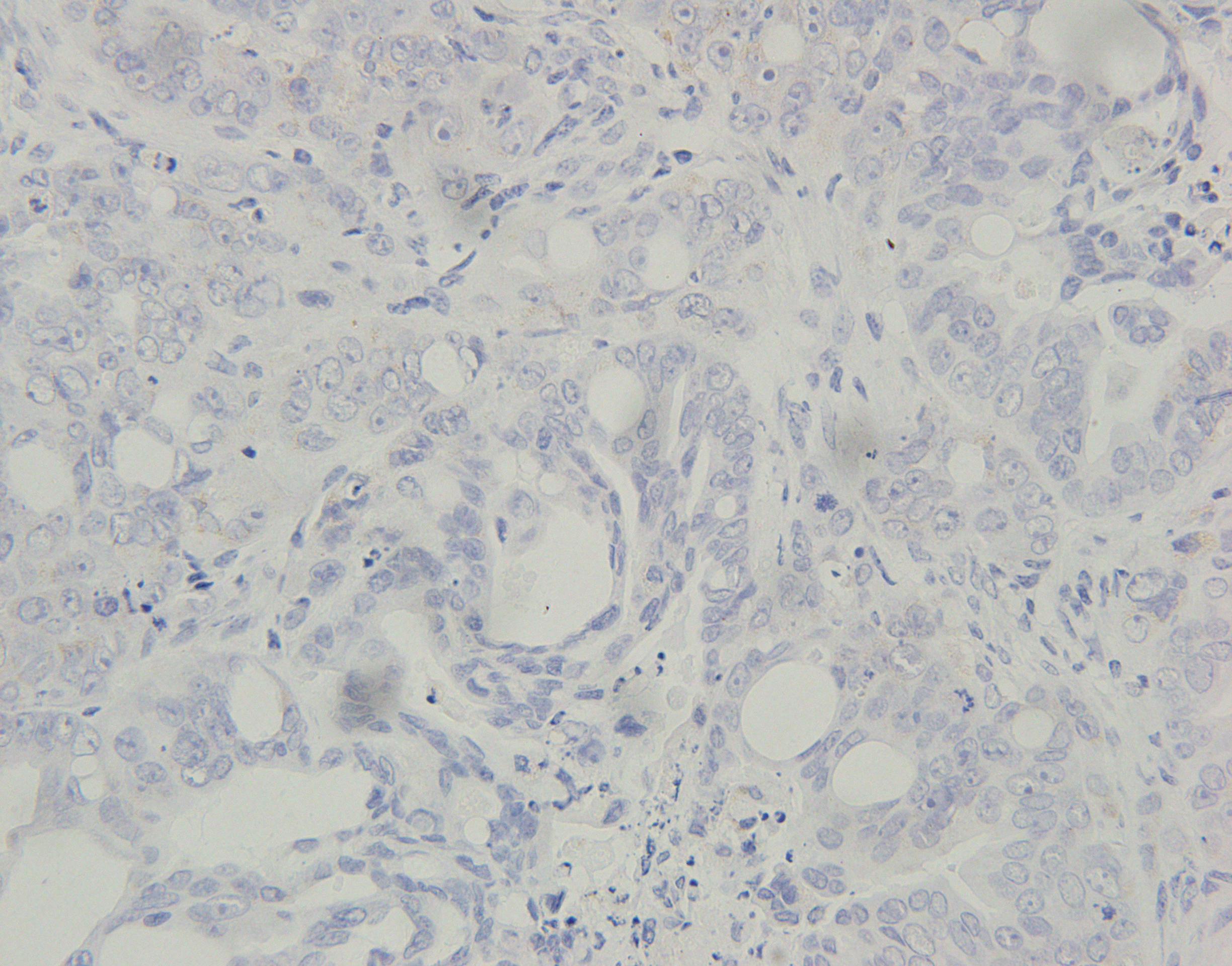

Supplement: Supplementary file 2 — Source data Fig. 1 [file 44318_2025_363_MOESM2_ESM.zip › Figure 1/1E/Control-N-cad.tif]

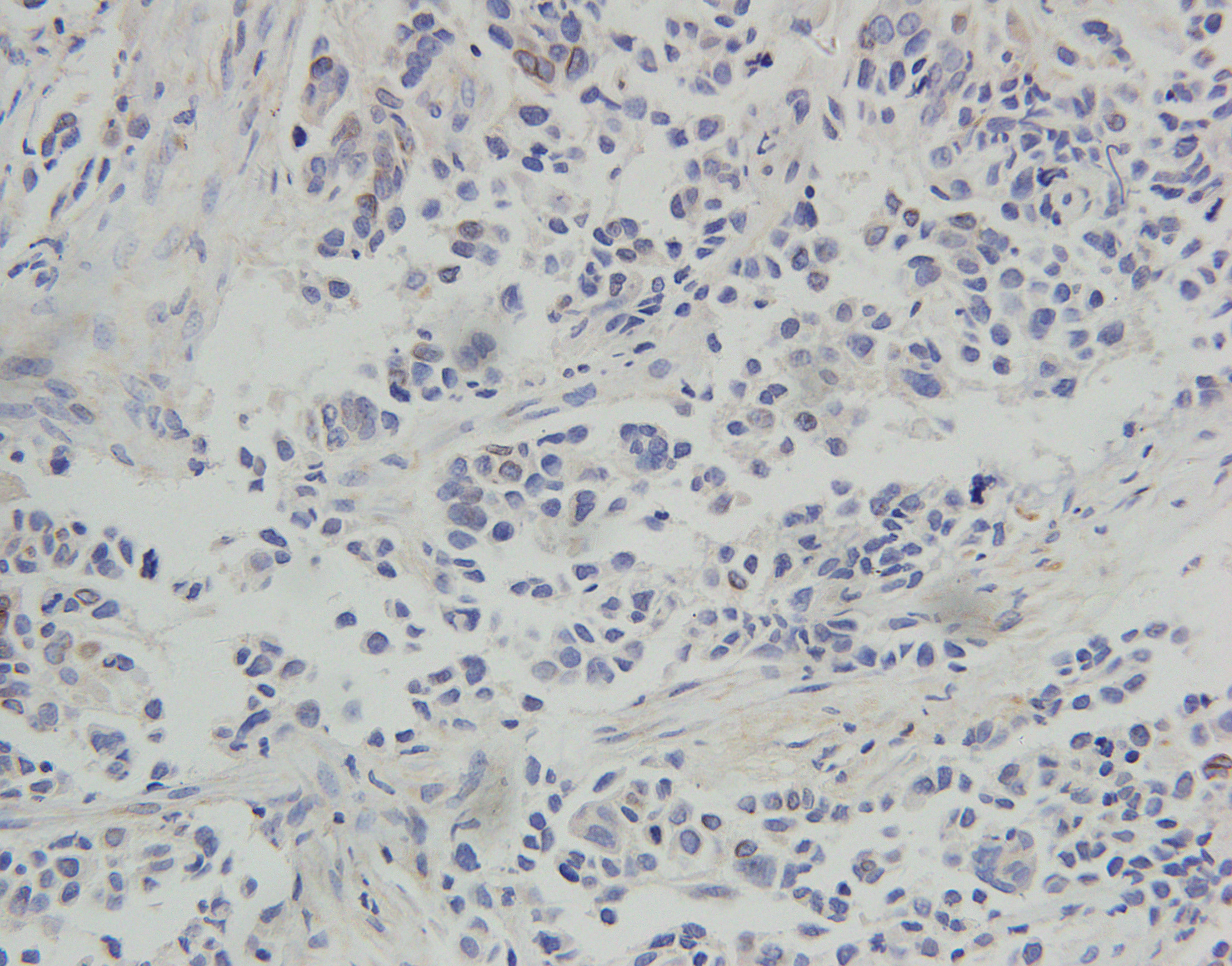

Supplement: Supplementary file 2 — Source data Fig. 1 [file 44318_2025_363_MOESM2_ESM.zip › Figure 1/1E/Ephrin A1-E-cad.tif]

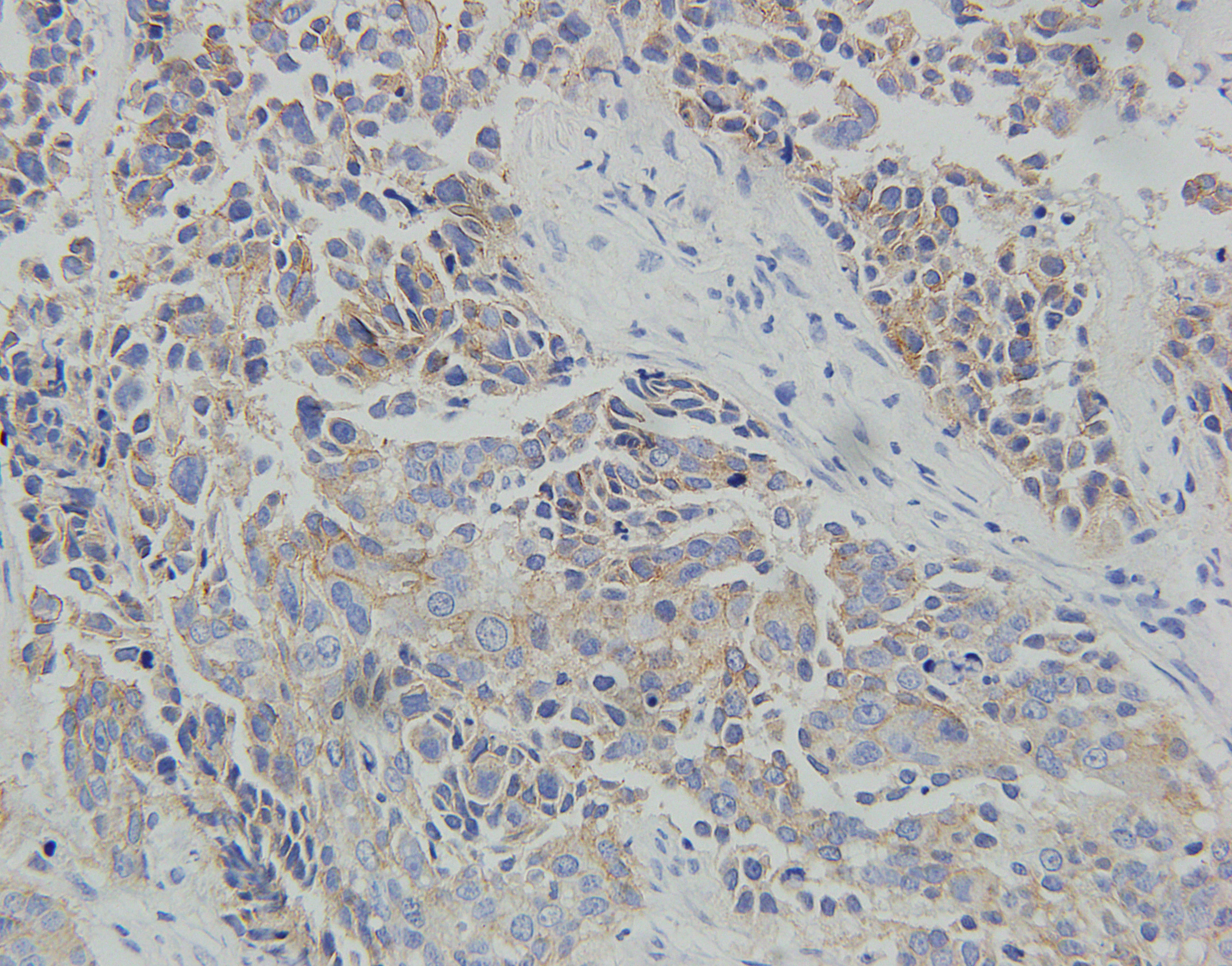

Supplement: Supplementary file 2 — Source data Fig. 1 [file 44318_2025_363_MOESM2_ESM.zip › Figure 1/1E/Ephrin A1-N-cad.tif]

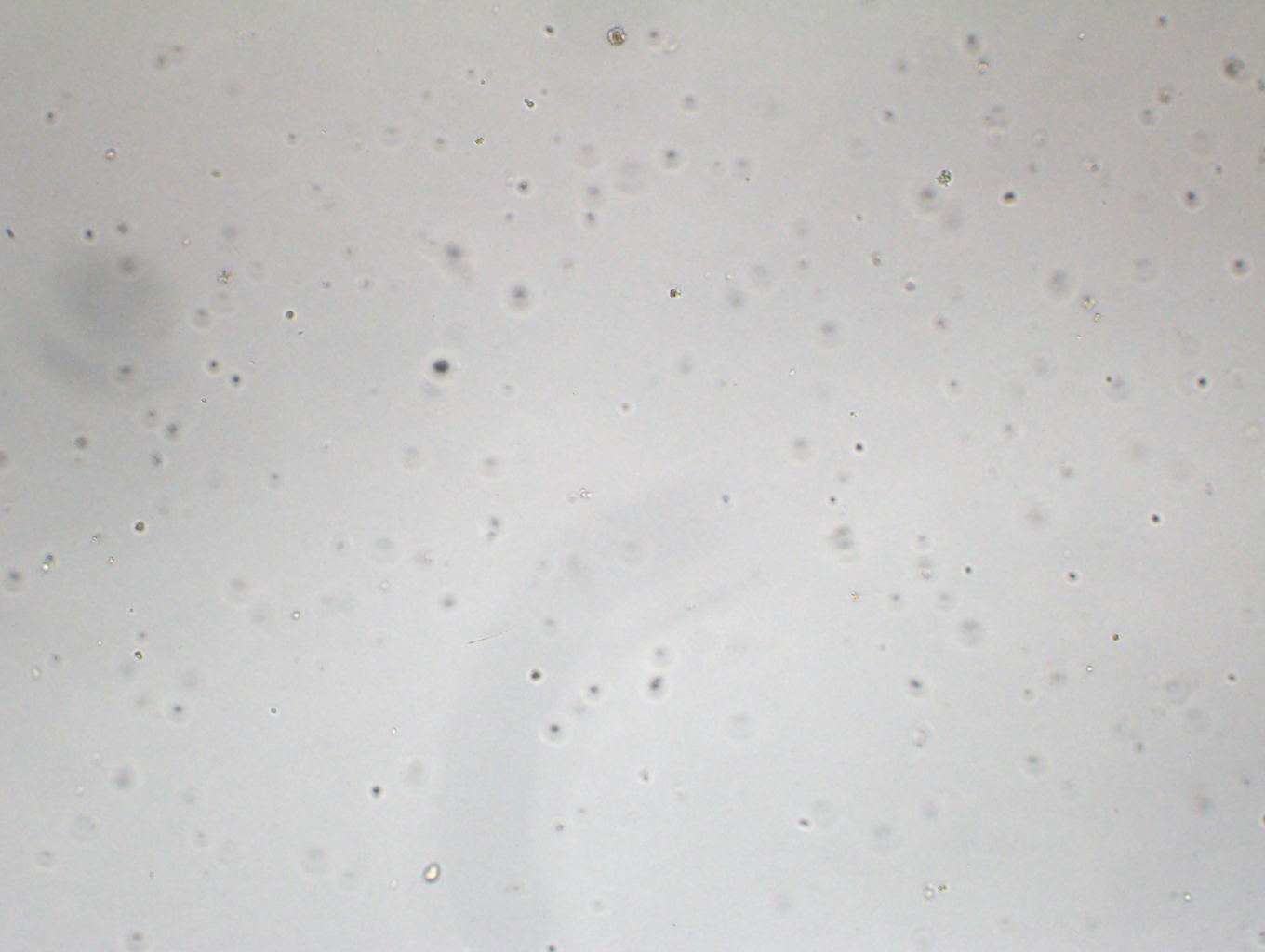

Supplement: Supplementary file 2 — Source data Fig. 1 [file 44318_2025_363_MOESM2_ESM.zip › Figure 1/1F/Control (1).tif]

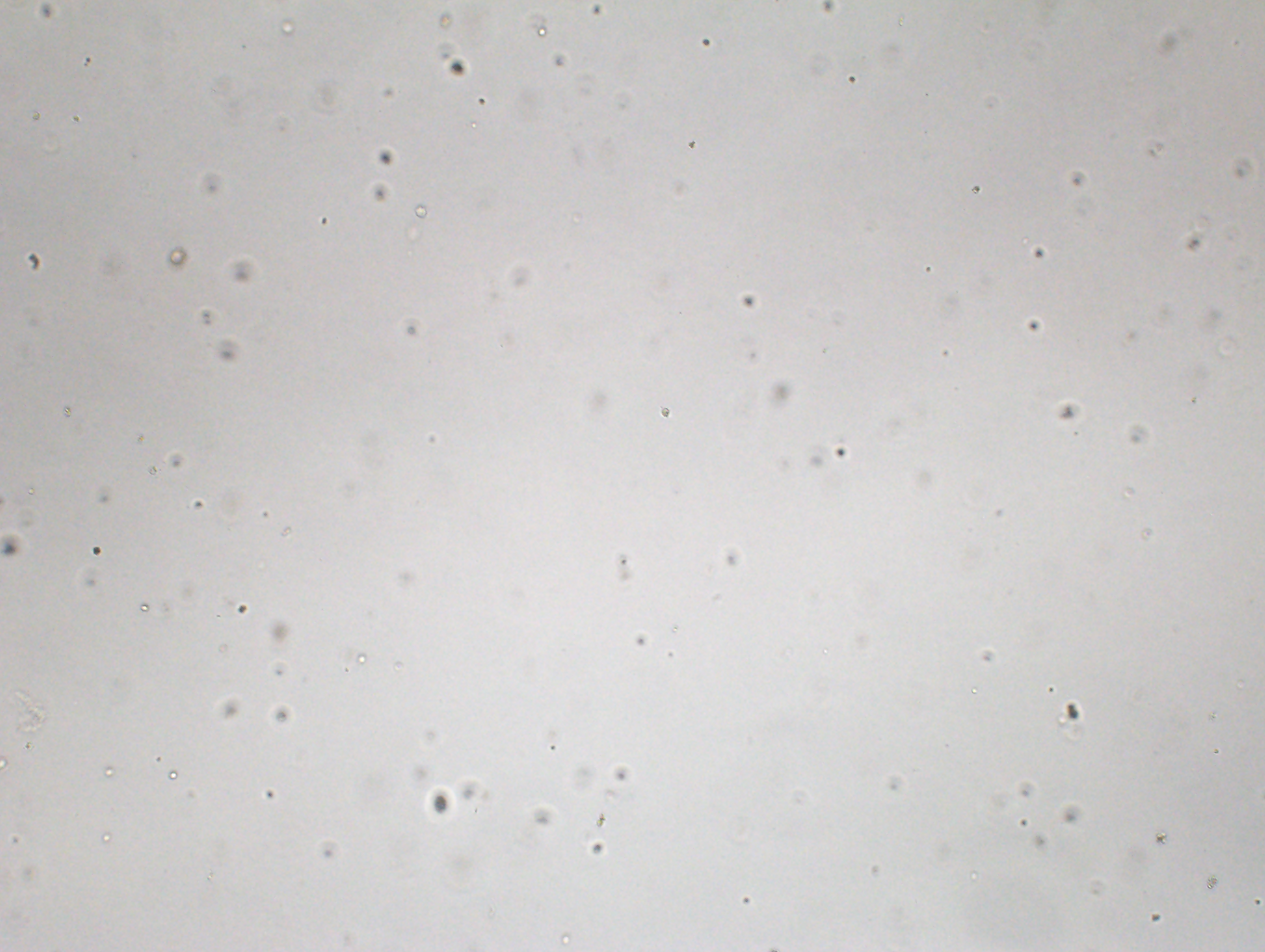

Supplement: Supplementary file 2 — Source data Fig. 1 [file 44318_2025_363_MOESM2_ESM.zip › Figure 1/1F/Control (2).tif]

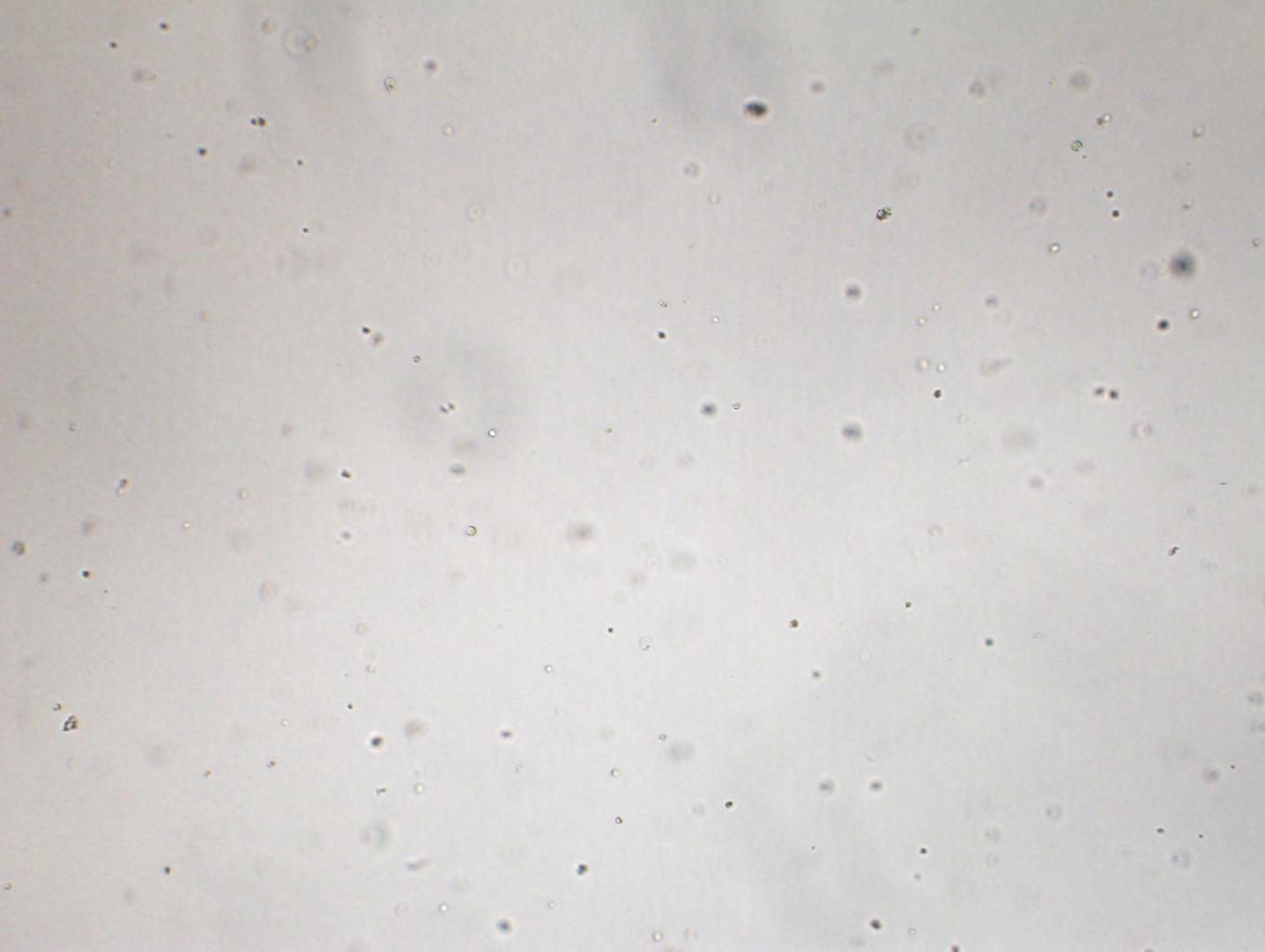

Supplement: Supplementary file 2 — Source data Fig. 1 [file 44318_2025_363_MOESM2_ESM.zip › Figure 1/1F/Control (3).tif]

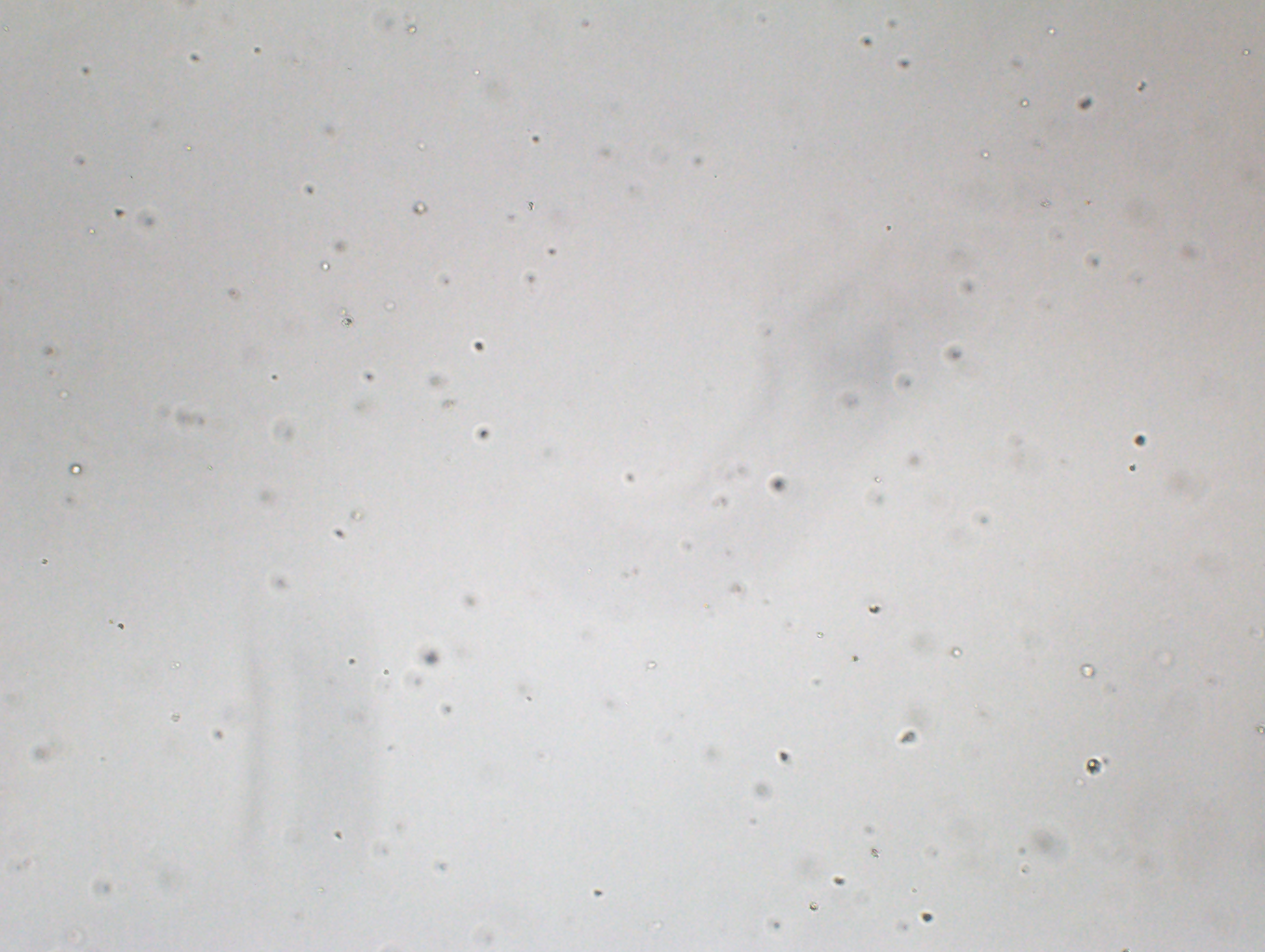

Supplement: Supplementary file 2 — Source data Fig. 1 [file 44318_2025_363_MOESM2_ESM.zip › Figure 1/1F/Control (4).tif]

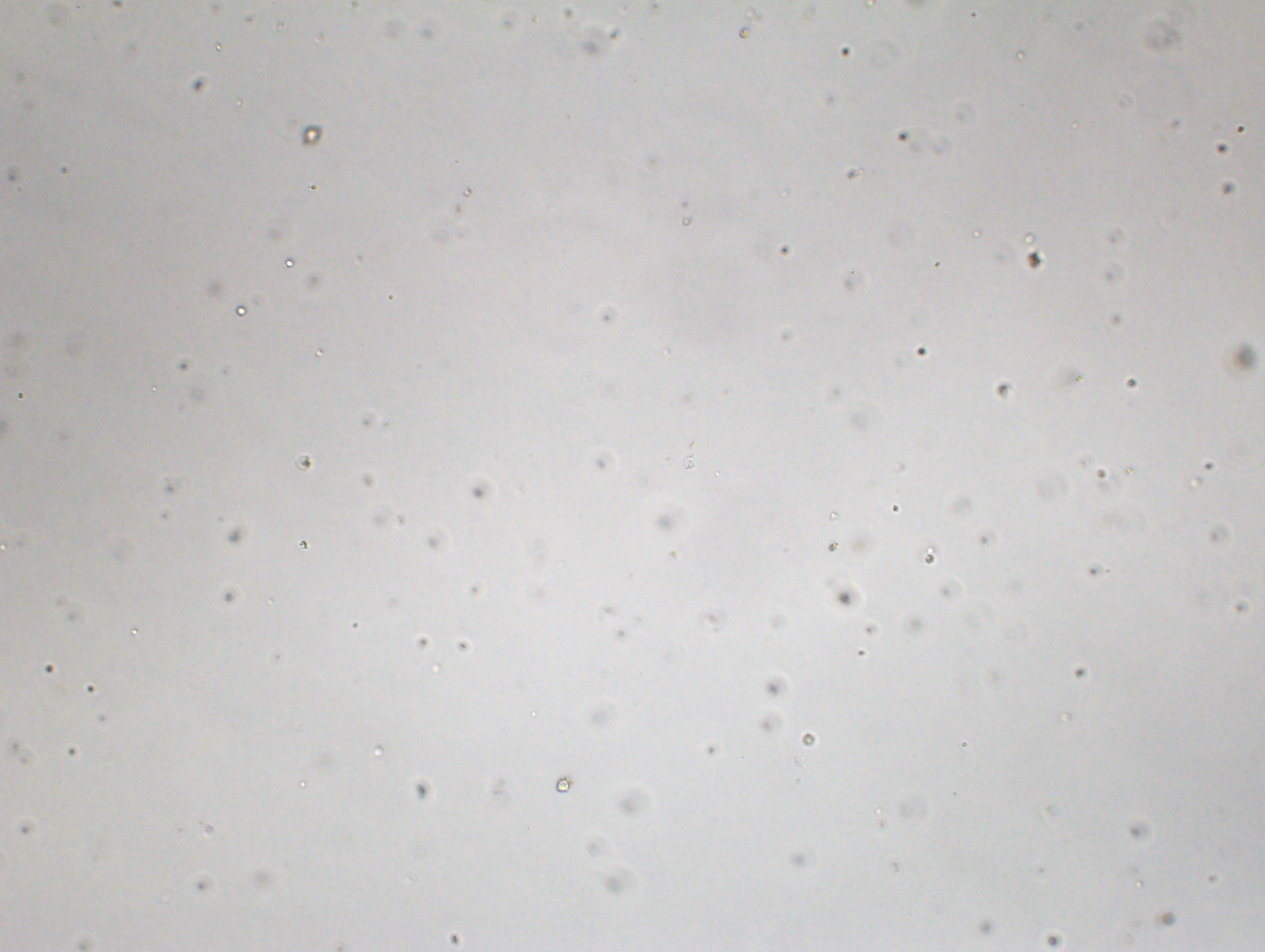

Supplement: Supplementary file 2 — Source data Fig. 1 [file 44318_2025_363_MOESM2_ESM.zip › Figure 1/1F/Control (5).tif]

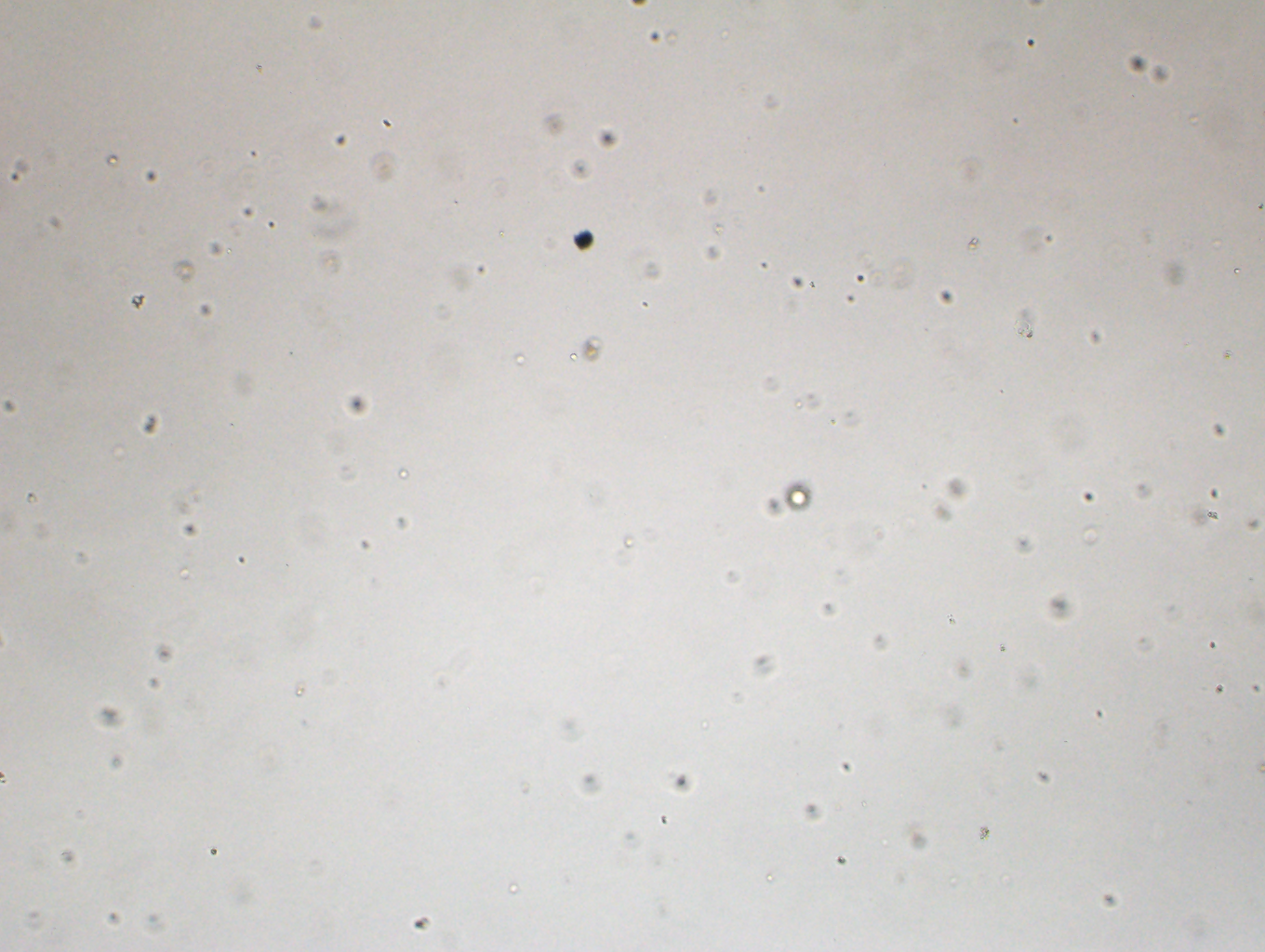

Supplement: Supplementary file 2 — Source data Fig. 1 [file 44318_2025_363_MOESM2_ESM.zip › Figure 1/1F/Control (6)-displayed in 1F.tif]

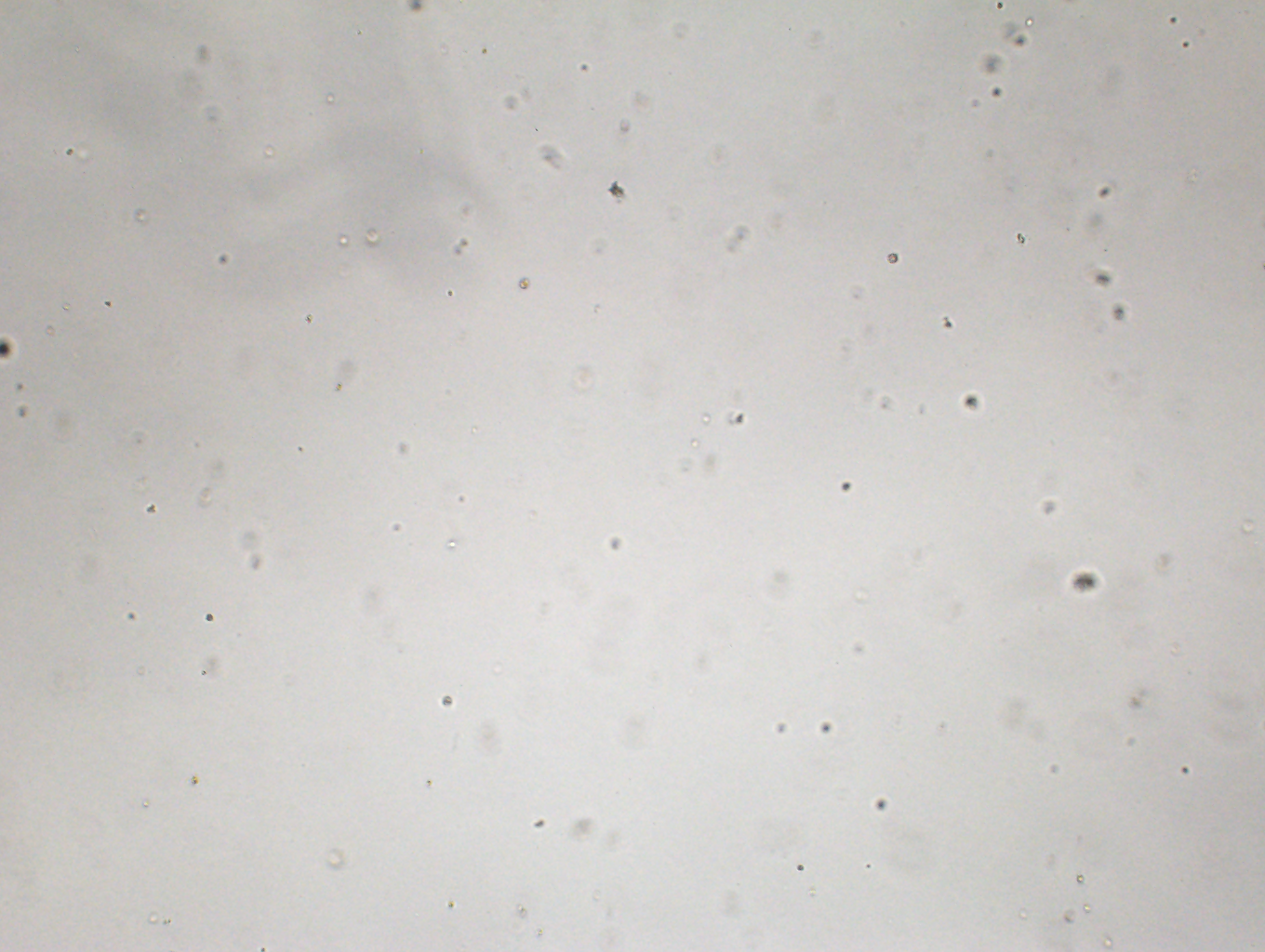

Supplement: Supplementary file 2 — Source data Fig. 1 [file 44318_2025_363_MOESM2_ESM.zip › Figure 1/1F/Control (7).tif]

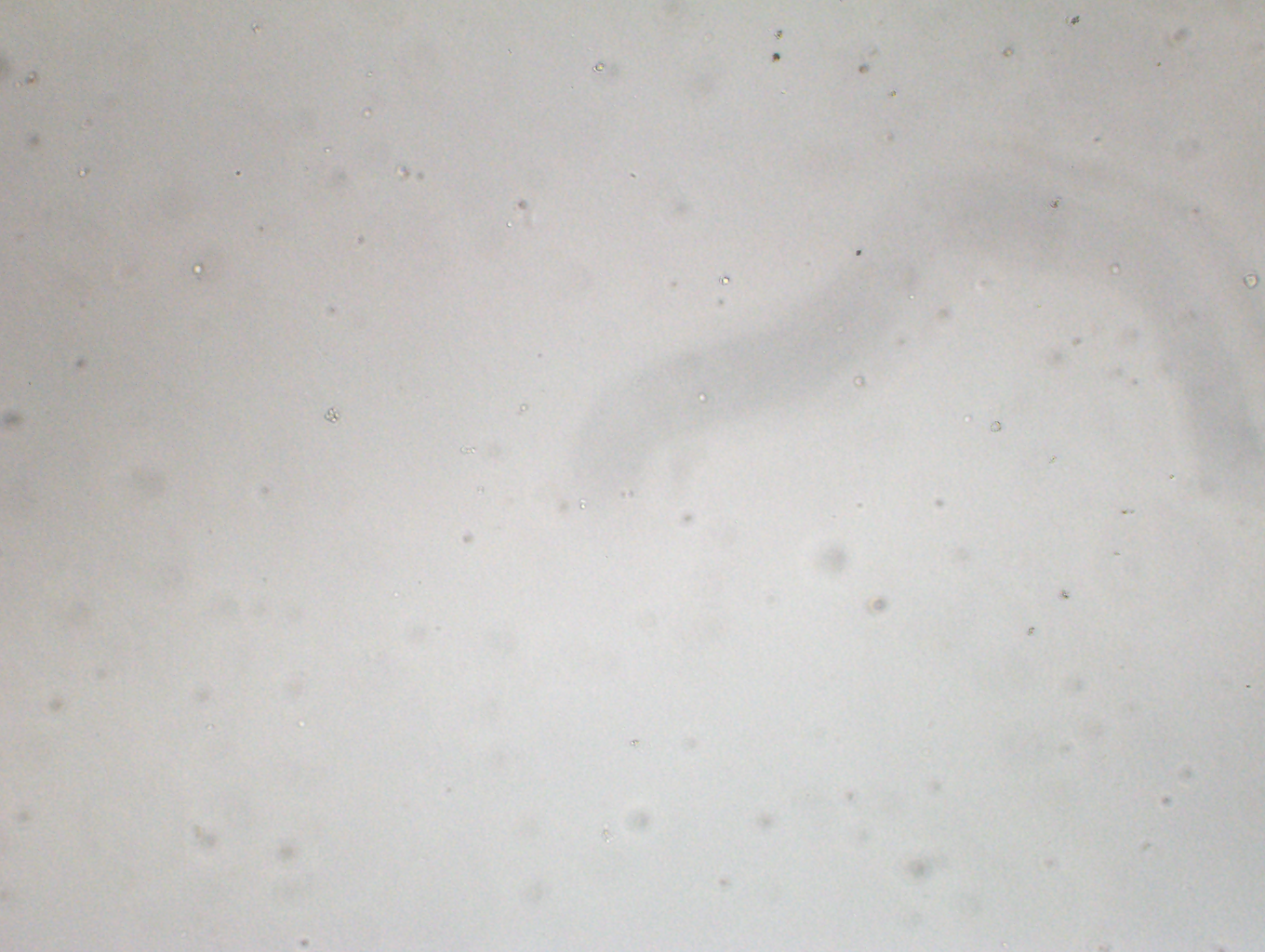

Supplement: Supplementary file 2 — Source data Fig. 1 [file 44318_2025_363_MOESM2_ESM.zip › Figure 1/1F/Control (8).tif]

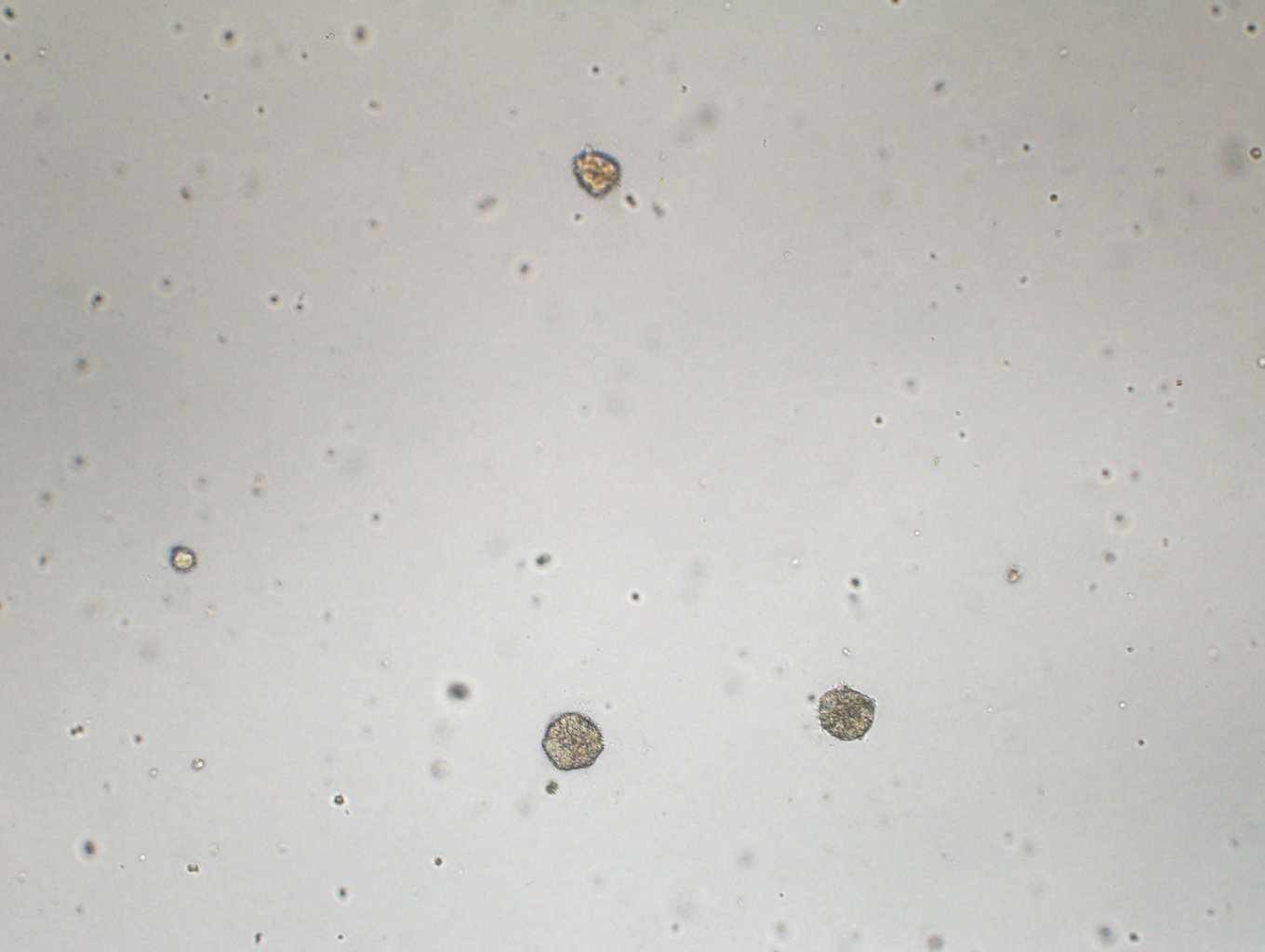

Supplement: Supplementary file 2 — Source data Fig. 1 [file 44318_2025_363_MOESM2_ESM.zip › Figure 1/1F/Ephrin A1 (1).tif]

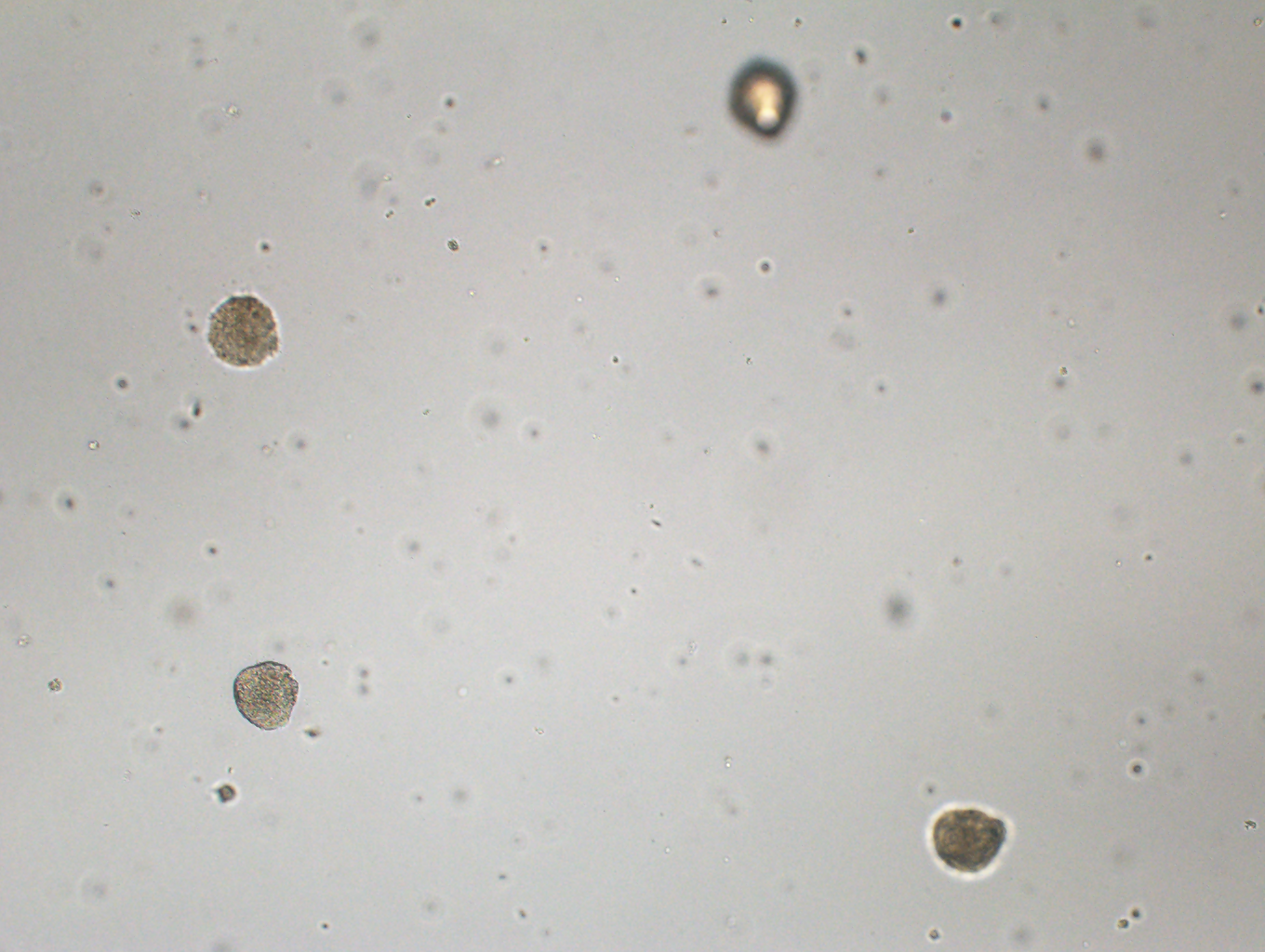

Supplement: Supplementary file 2 — Source data Fig. 1 [file 44318_2025_363_MOESM2_ESM.zip › Figure 1/1F/Ephrin A1 (2).tif]

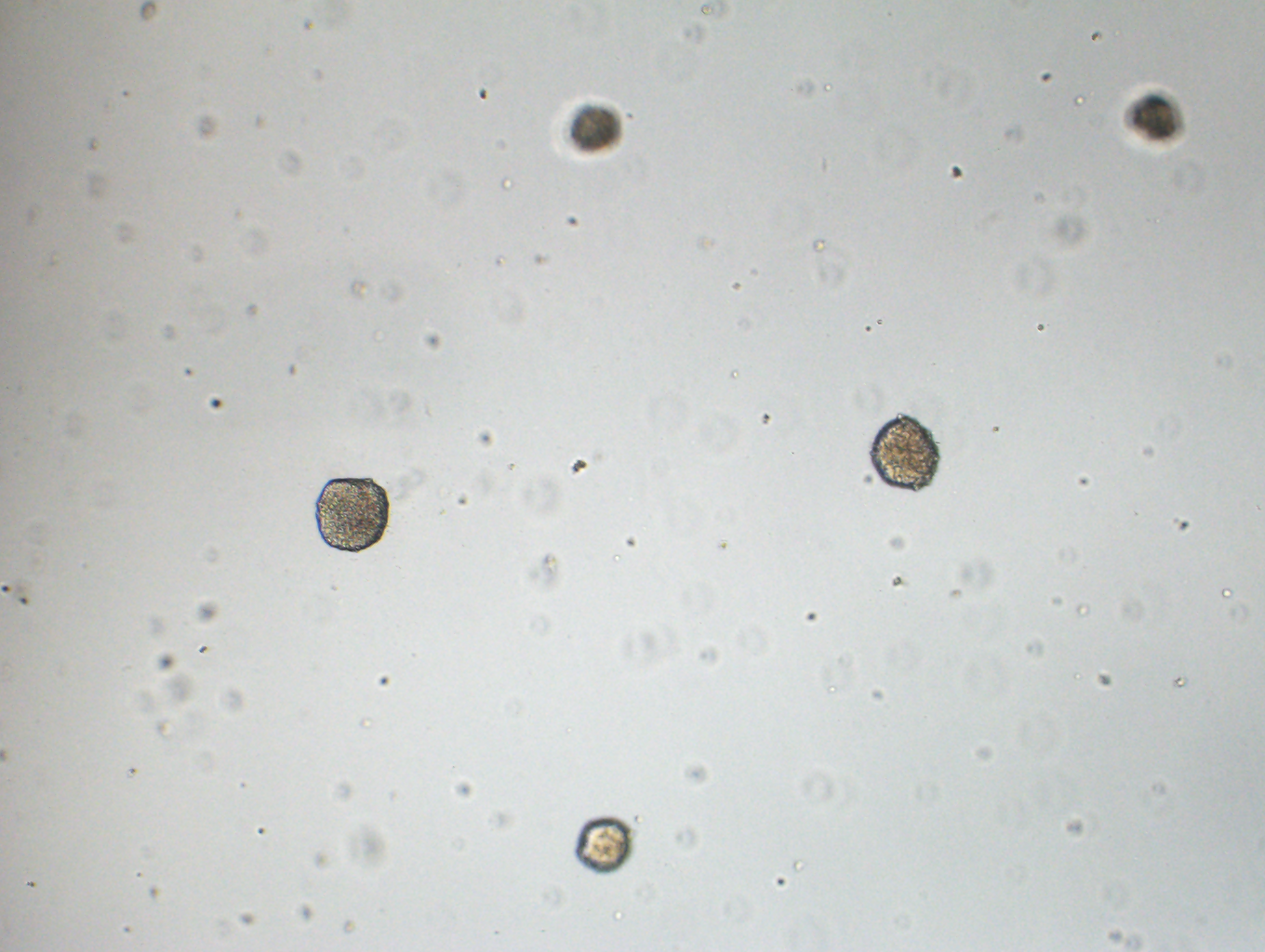

Supplement: Supplementary file 2 — Source data Fig. 1 [file 44318_2025_363_MOESM2_ESM.zip › Figure 1/1F/Ephrin A1 (3).tif]

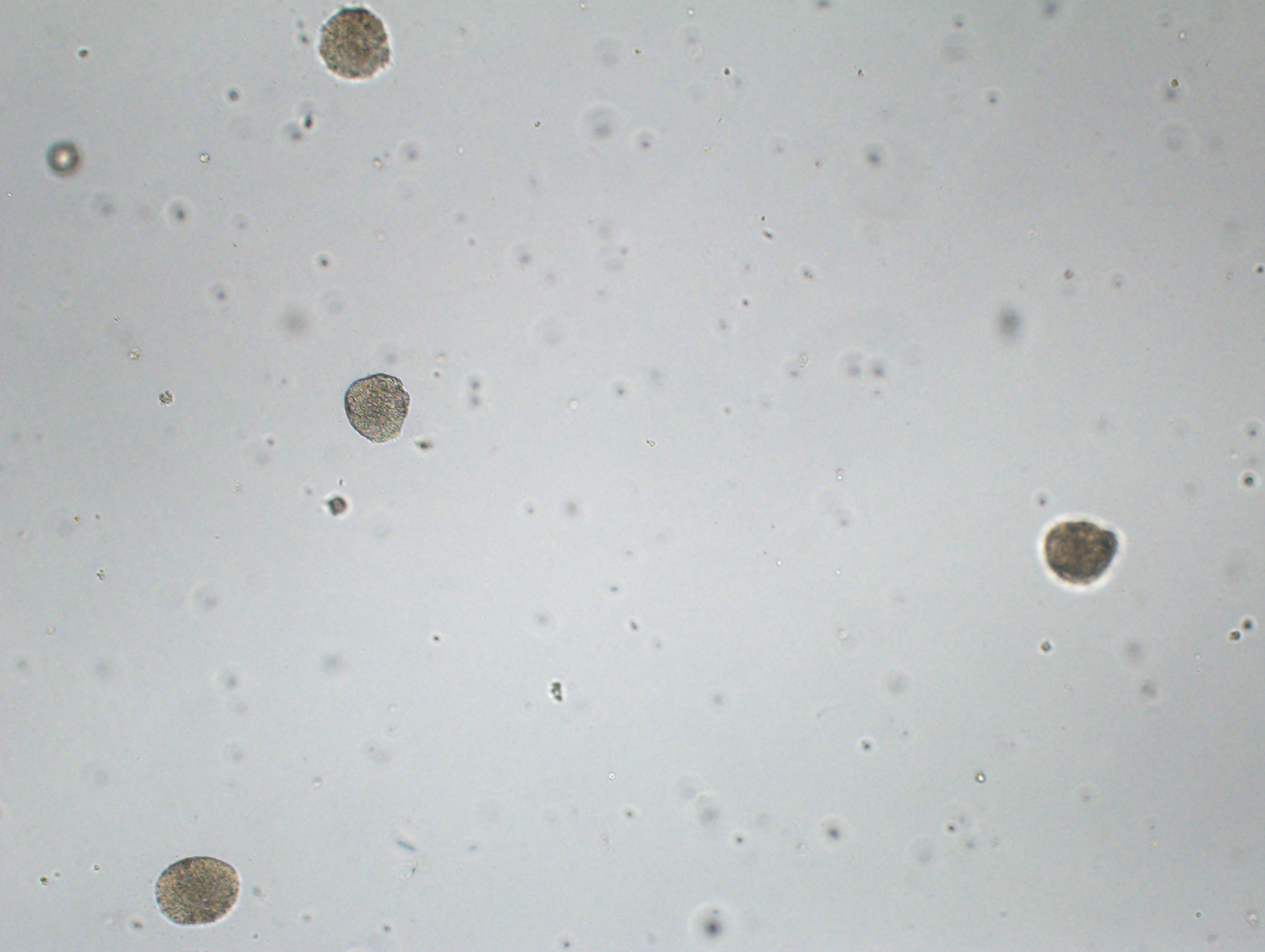

Supplement: Supplementary file 2 — Source data Fig. 1 [file 44318_2025_363_MOESM2_ESM.zip › Figure 1/1F/Ephrin A1 (4).tif]

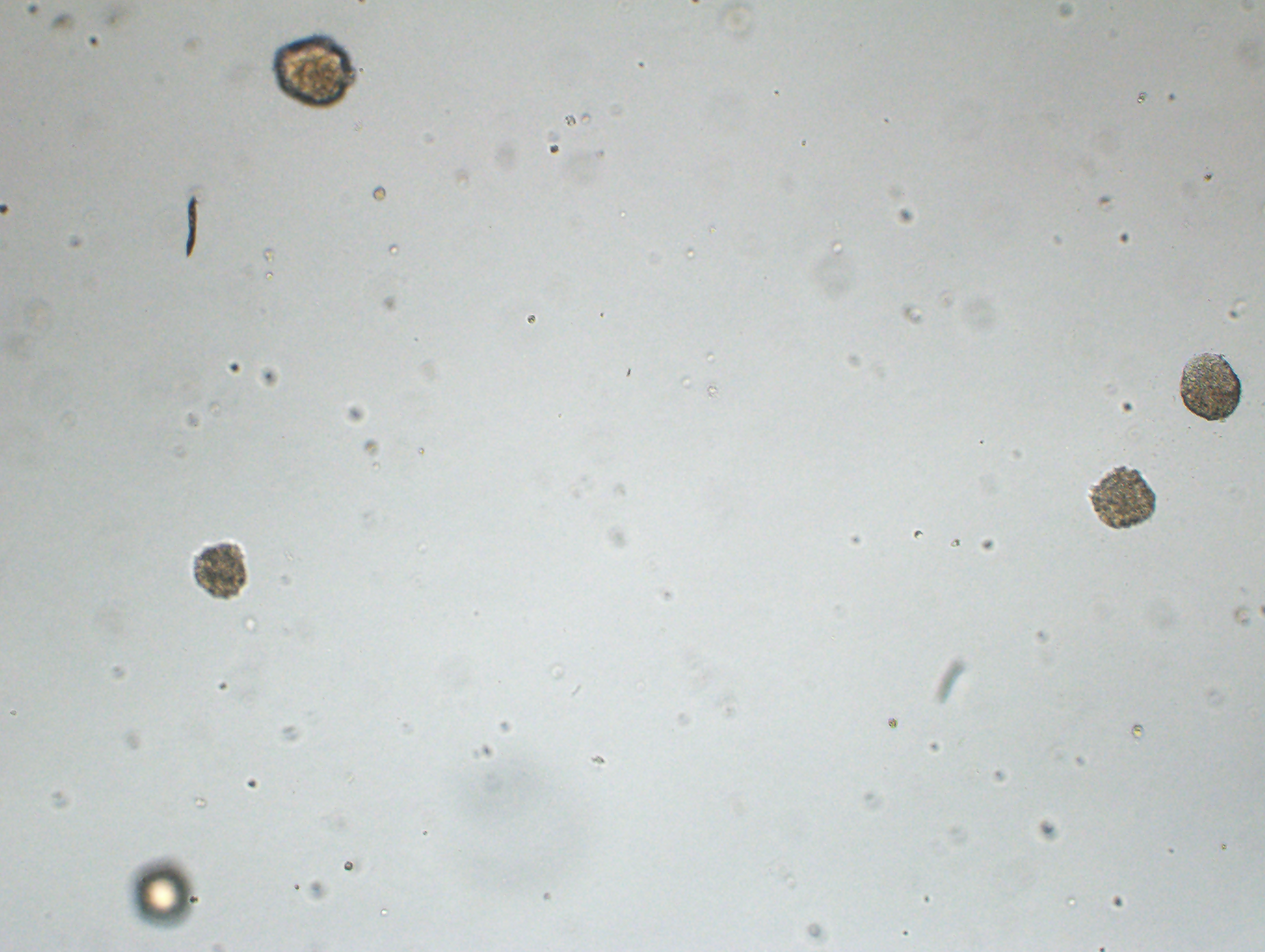

Supplement: Supplementary file 2 — Source data Fig. 1 [file 44318_2025_363_MOESM2_ESM.zip › Figure 1/1F/Ephrin A1 (5).tif]

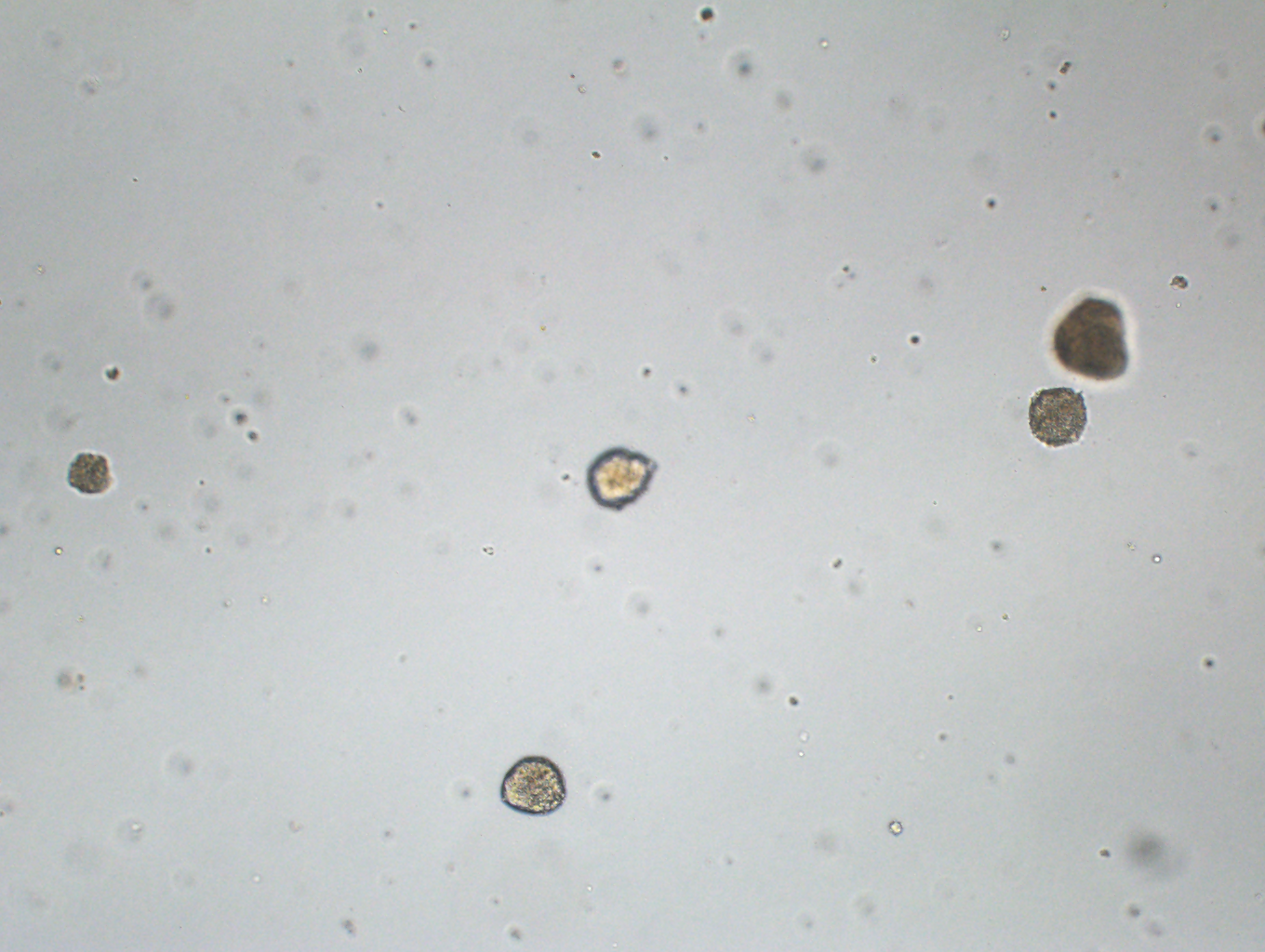

Supplement: Supplementary file 2 — Source data Fig. 1 [file 44318_2025_363_MOESM2_ESM.zip › Figure 1/1F/Ephrin A1 (6).tif]

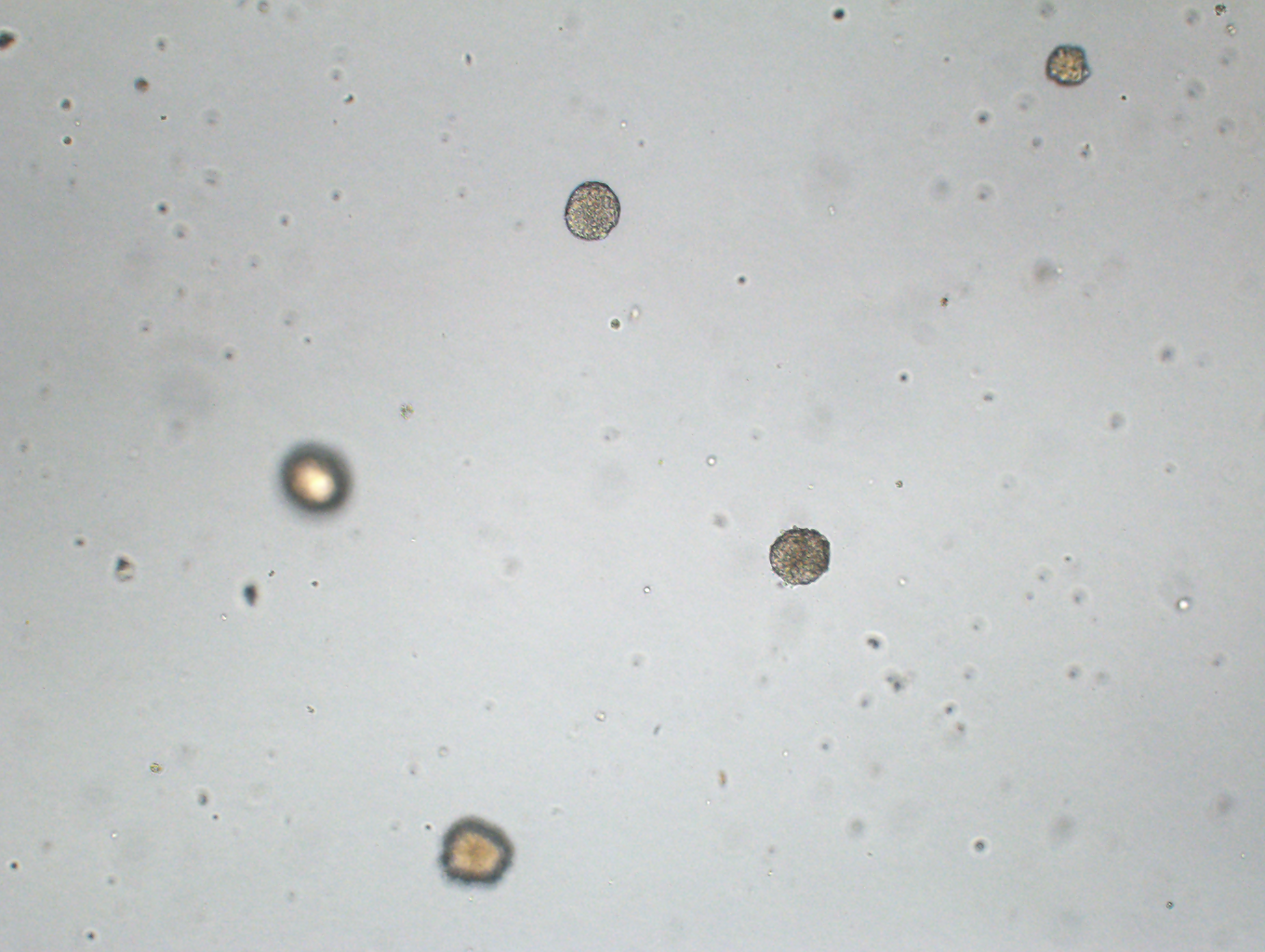

Supplement: Supplementary file 2 — Source data Fig. 1 [file 44318_2025_363_MOESM2_ESM.zip › Figure 1/1F/Ephrin A1 (7)-displayed in 1F.tif]

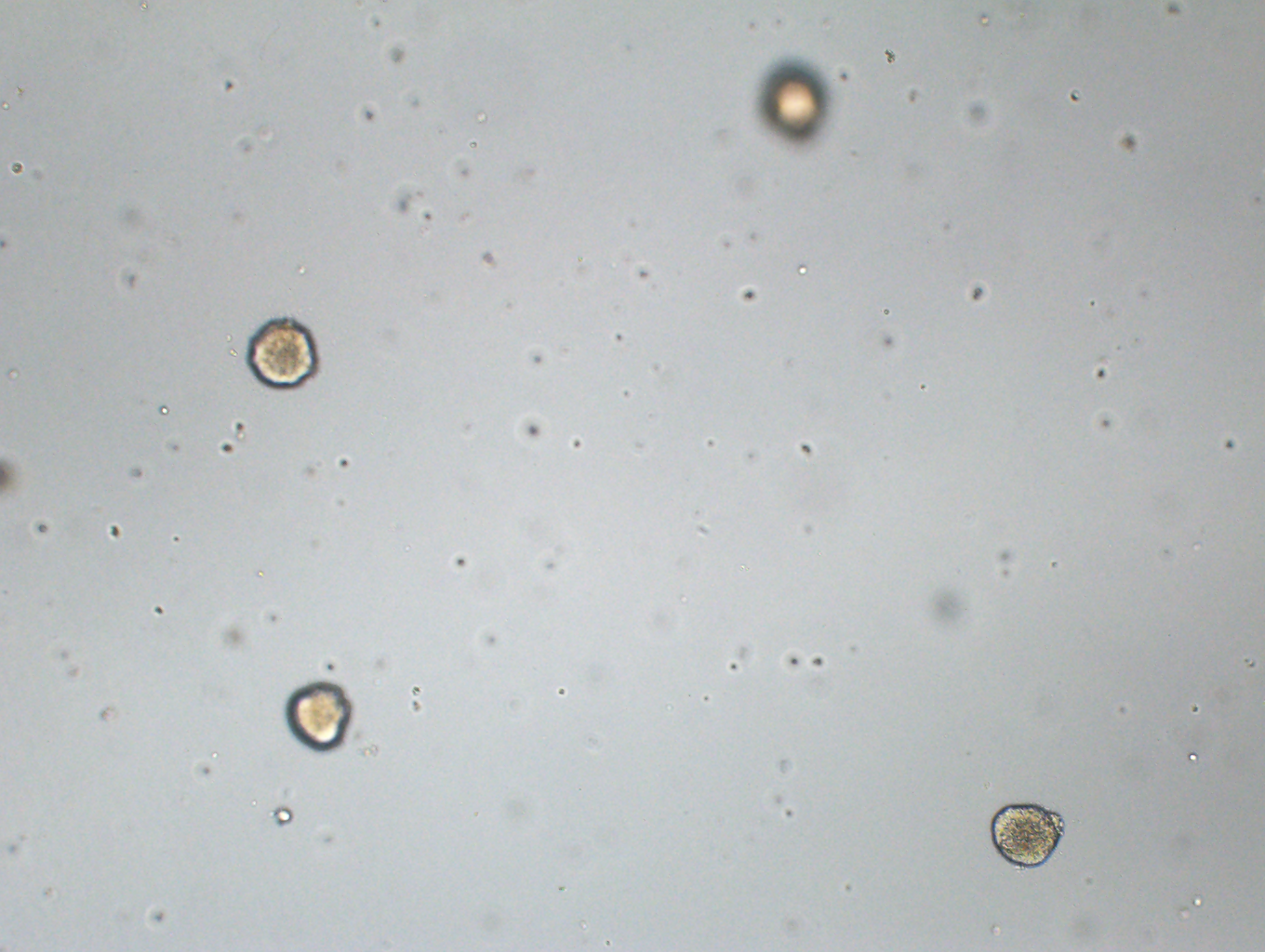

Supplement: Supplementary file 2 — Source data Fig. 1 [file 44318_2025_363_MOESM2_ESM.zip › Figure 1/1F/Ephrin A1 (8).tif]

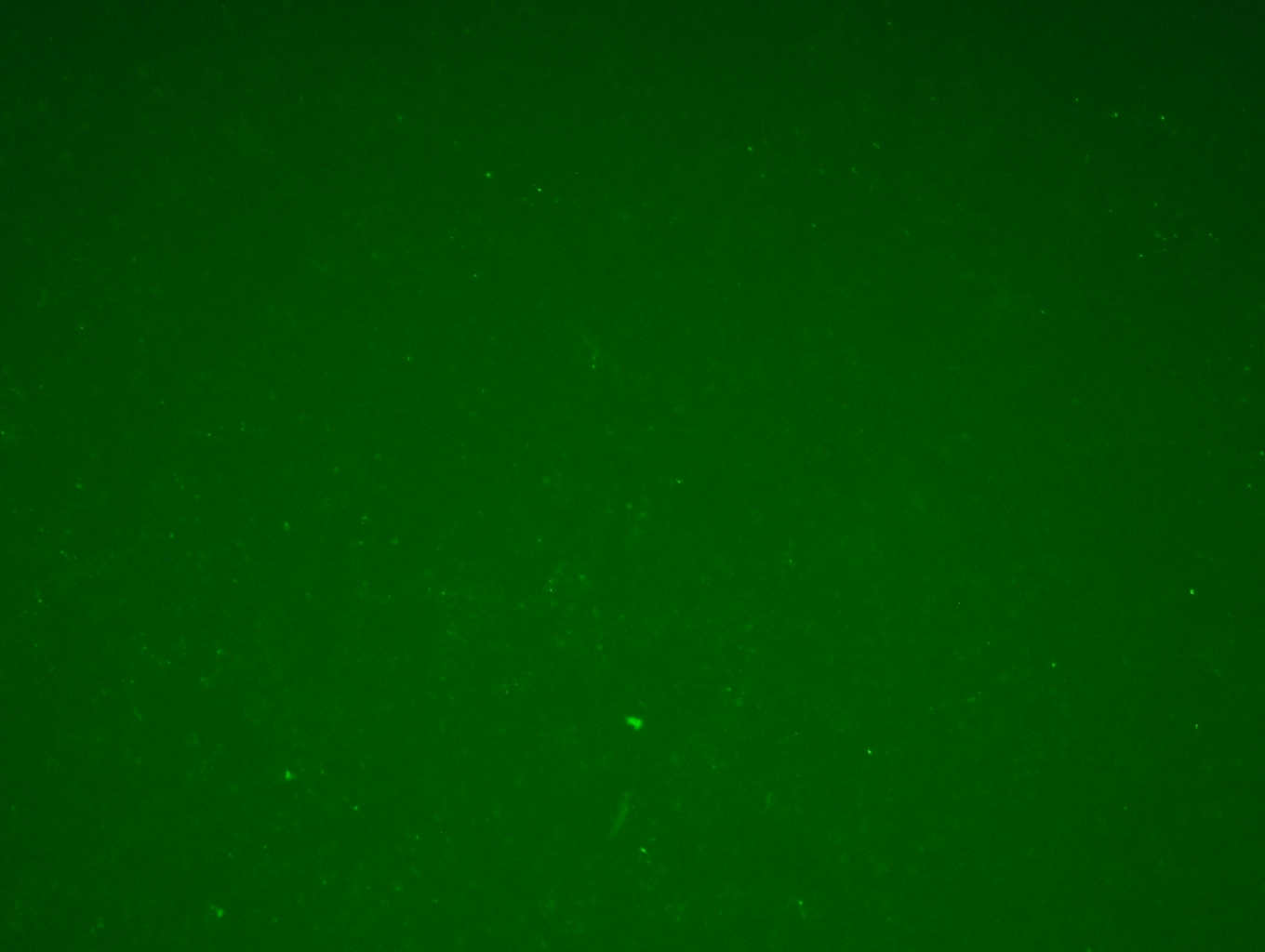

Supplement: Supplementary file 2 — Source data Fig. 1 [file 44318_2025_363_MOESM2_ESM.zip › Figure 1/1I/Control (1).tif]

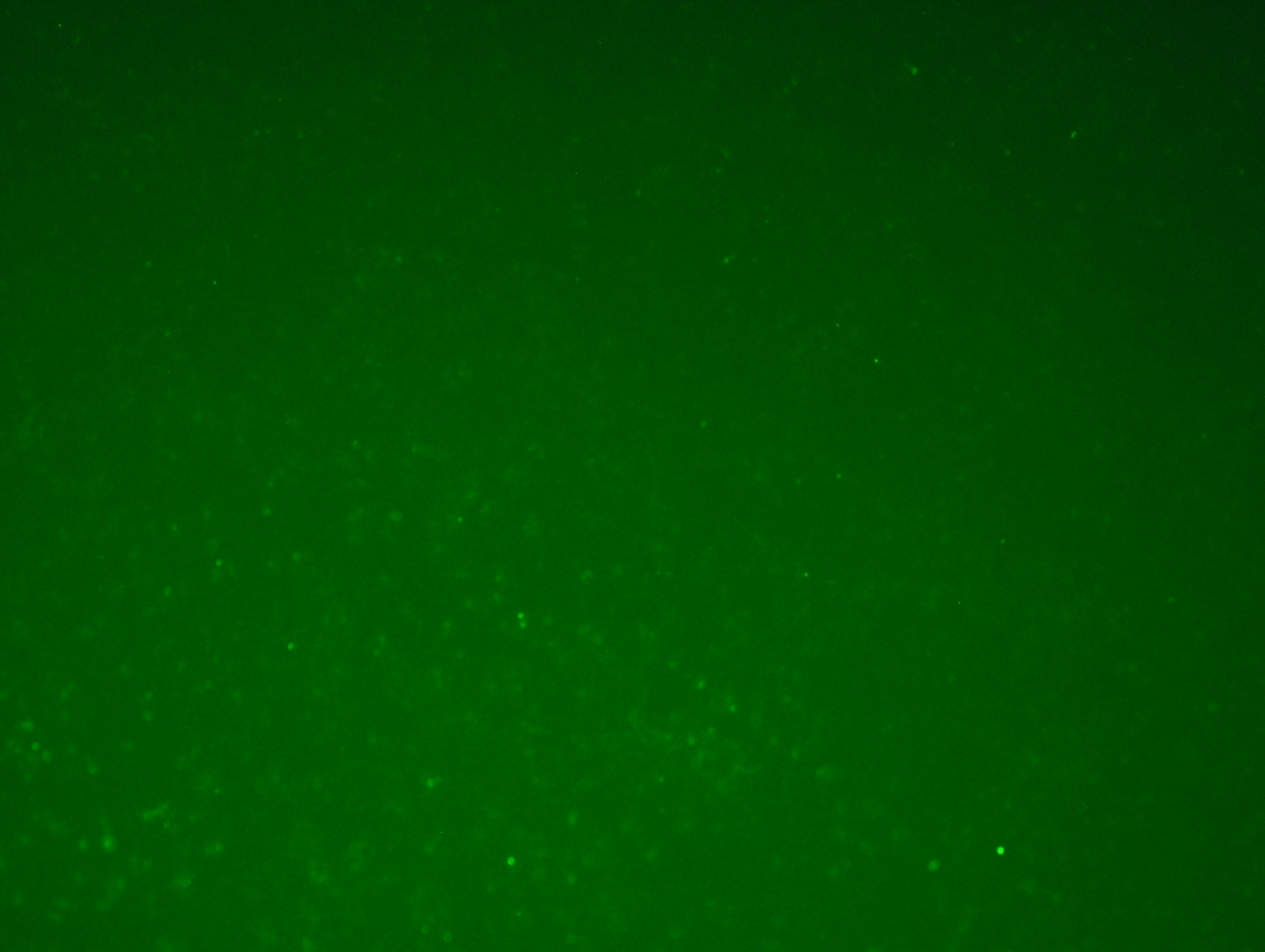

Supplement: Supplementary file 2 — Source data Fig. 1 [file 44318_2025_363_MOESM2_ESM.zip › Figure 1/1I/Control (2).tif]

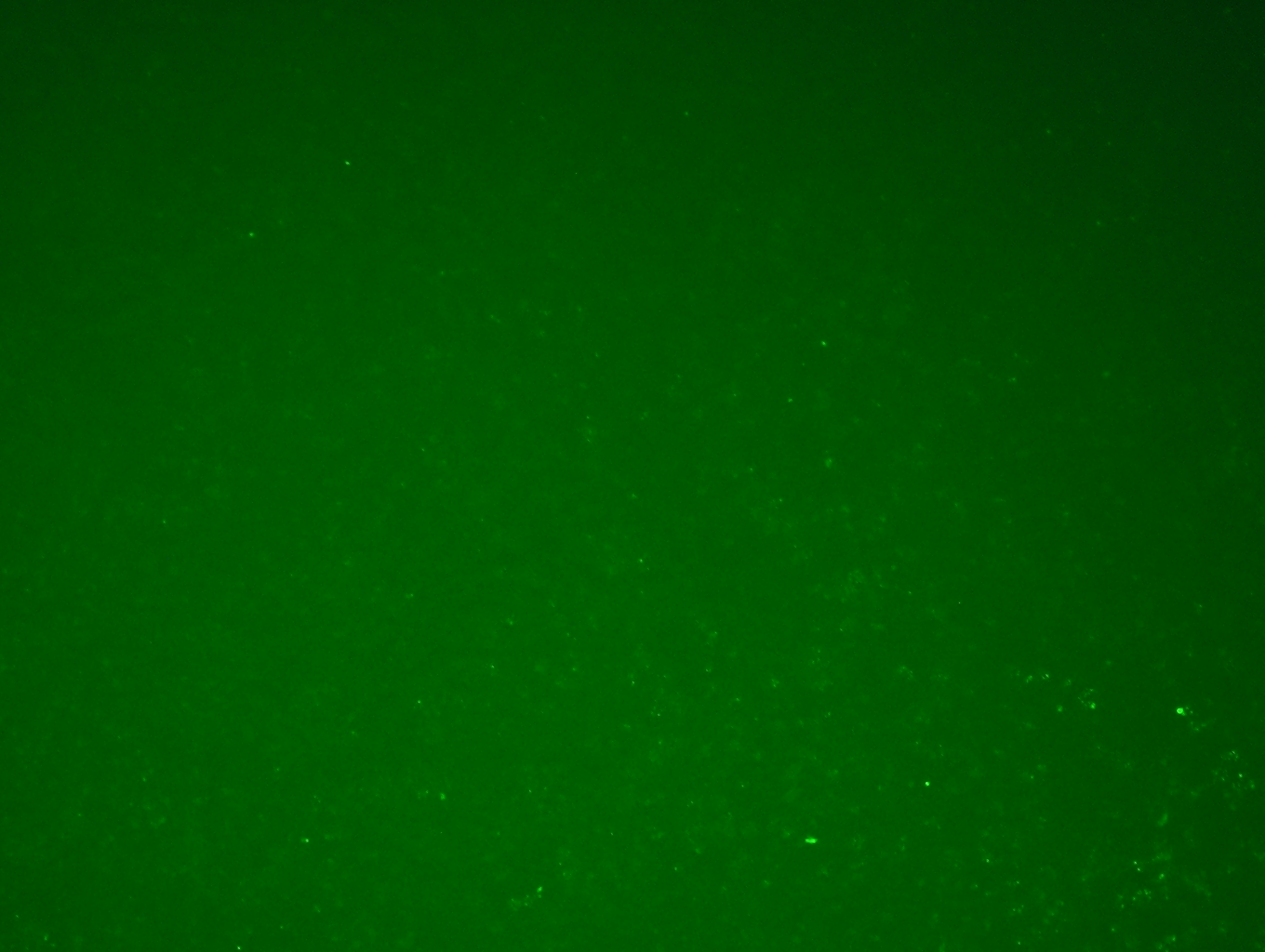

Supplement: Supplementary file 2 — Source data Fig. 1 [file 44318_2025_363_MOESM2_ESM.zip › Figure 1/1I/Control (3)-displayed in 1I.tif]

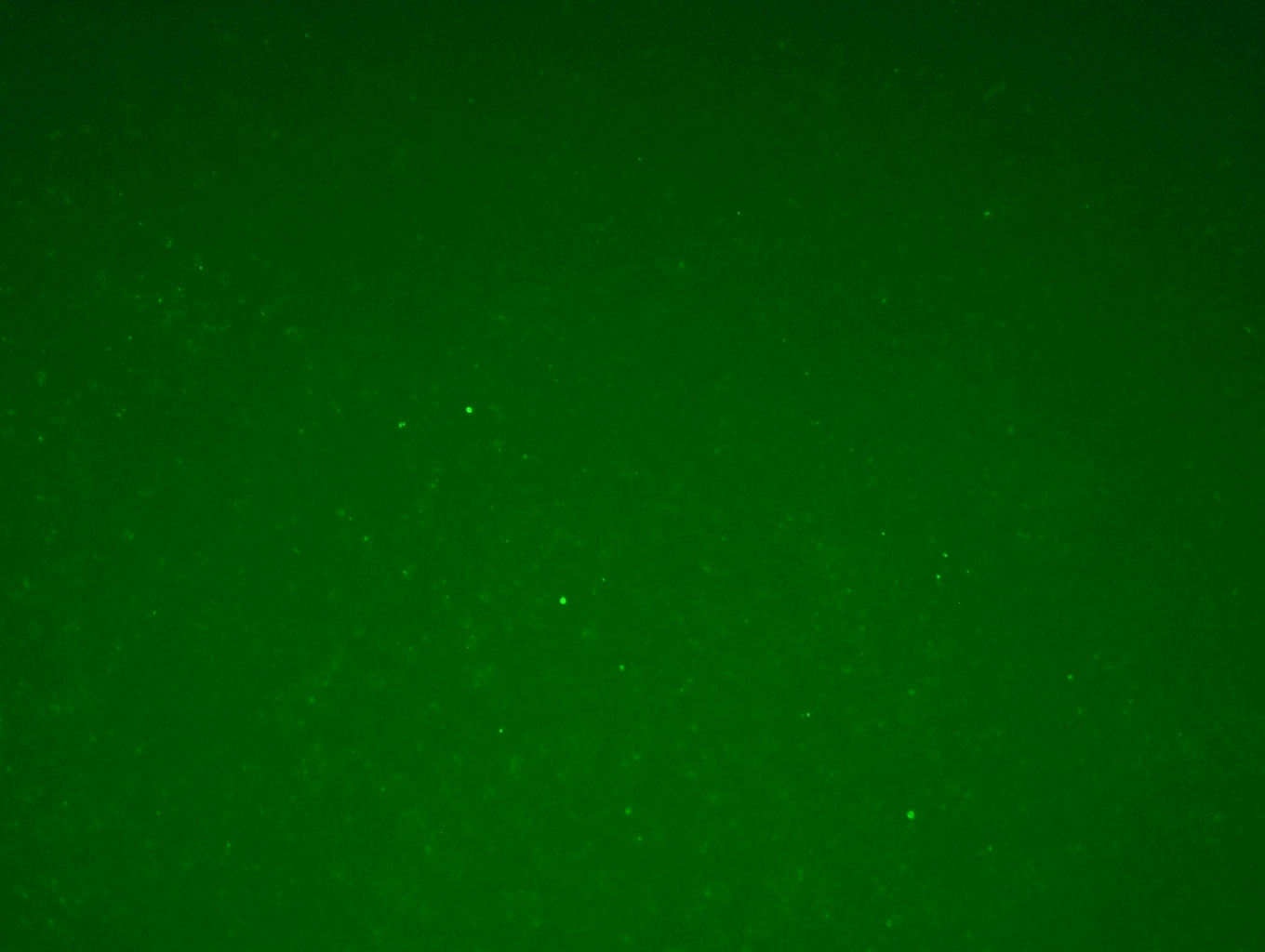

Supplement: Supplementary file 2 — Source data Fig. 1 [file 44318_2025_363_MOESM2_ESM.zip › Figure 1/1I/Control (4).tif]

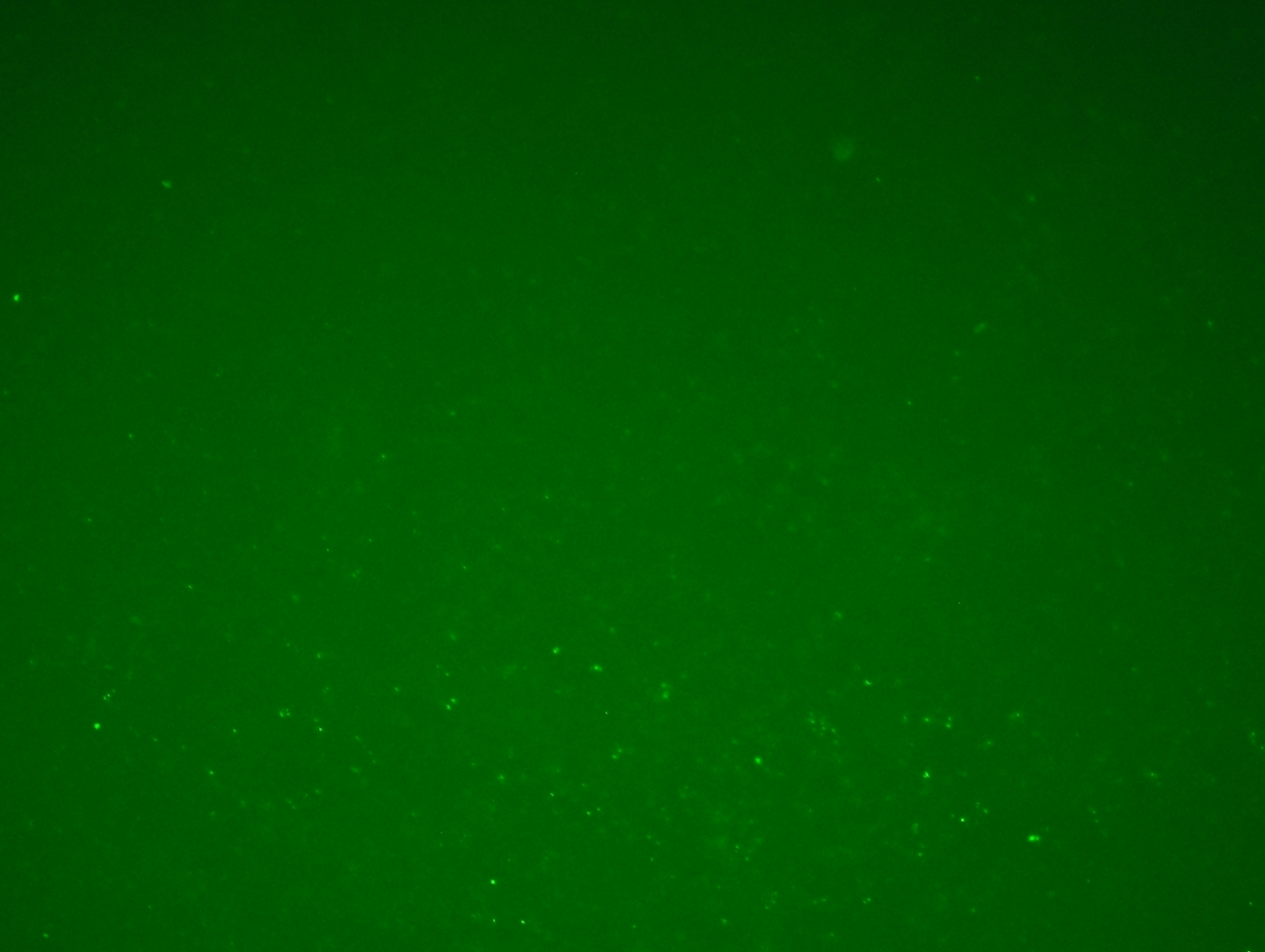

Supplement: Supplementary file 2 — Source data Fig. 1 [file 44318_2025_363_MOESM2_ESM.zip › Figure 1/1I/Control (5).tif]

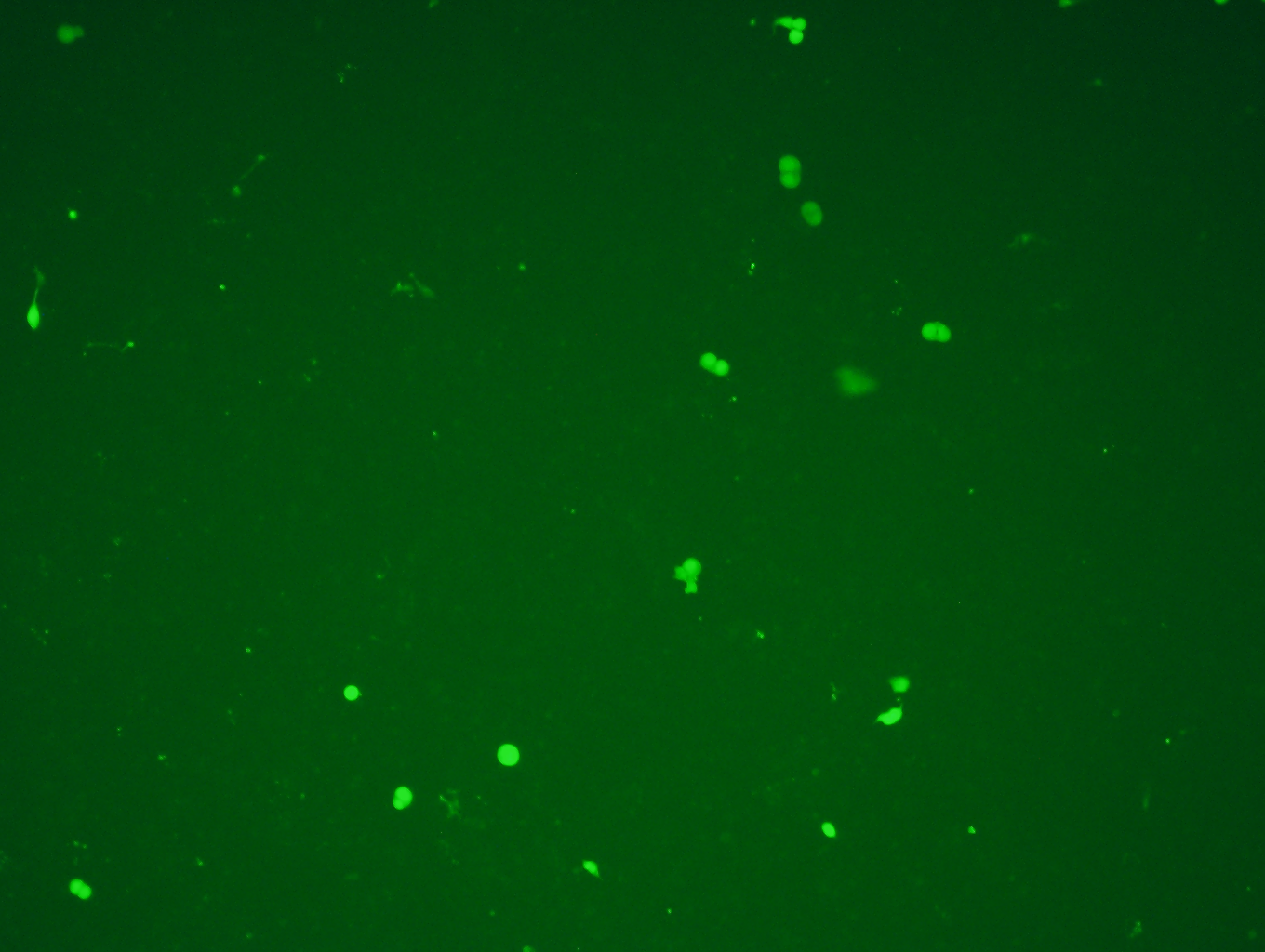

Supplement: Supplementary file 2 — Source data Fig. 1 [file 44318_2025_363_MOESM2_ESM.zip › Figure 1/1I/Ephrin A1 (1).tif]

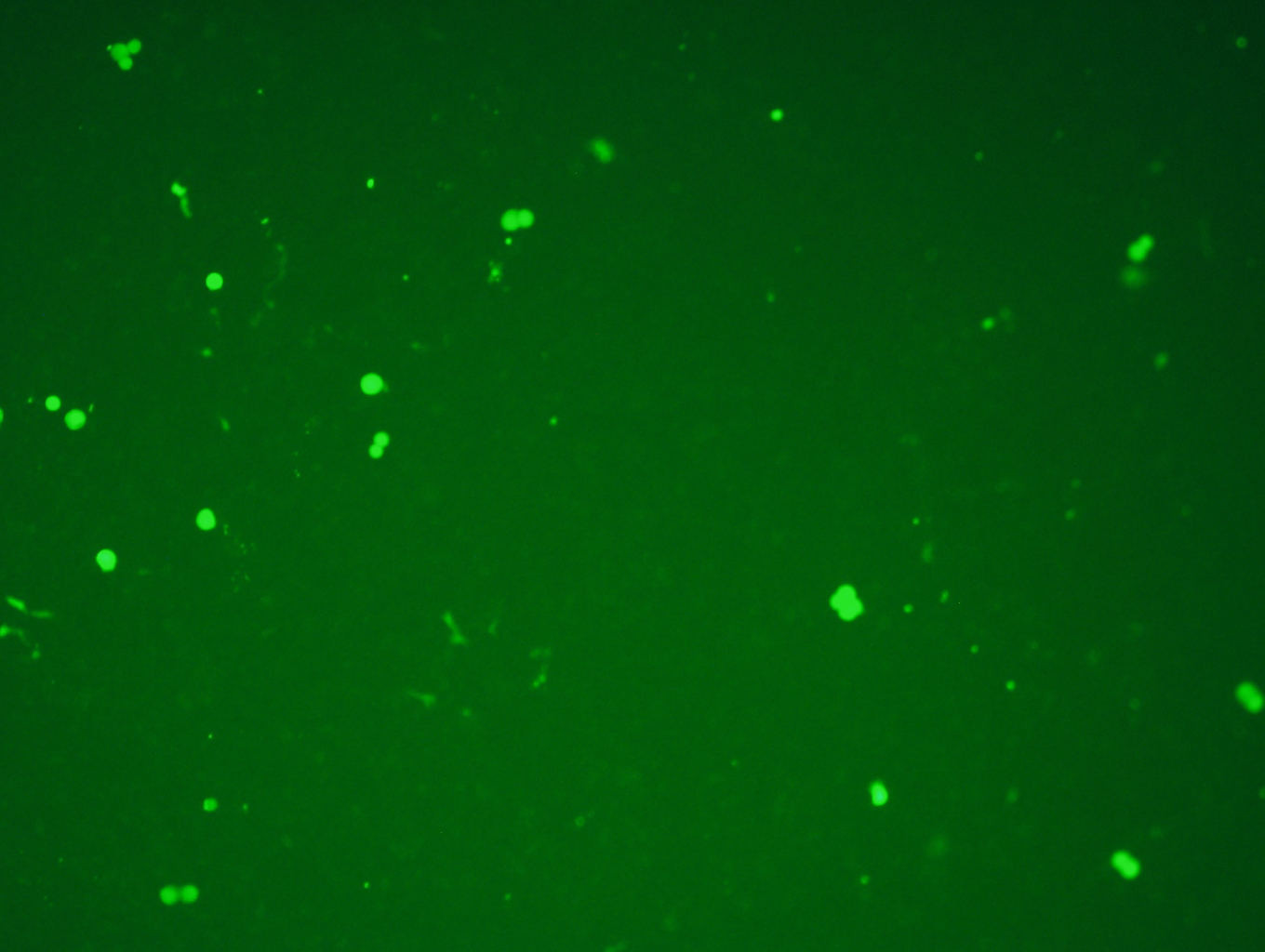

Supplement: Supplementary file 2 — Source data Fig. 1 [file 44318_2025_363_MOESM2_ESM.zip › Figure 1/1I/Ephrin A1 (2)-displayed in 1I.tif]

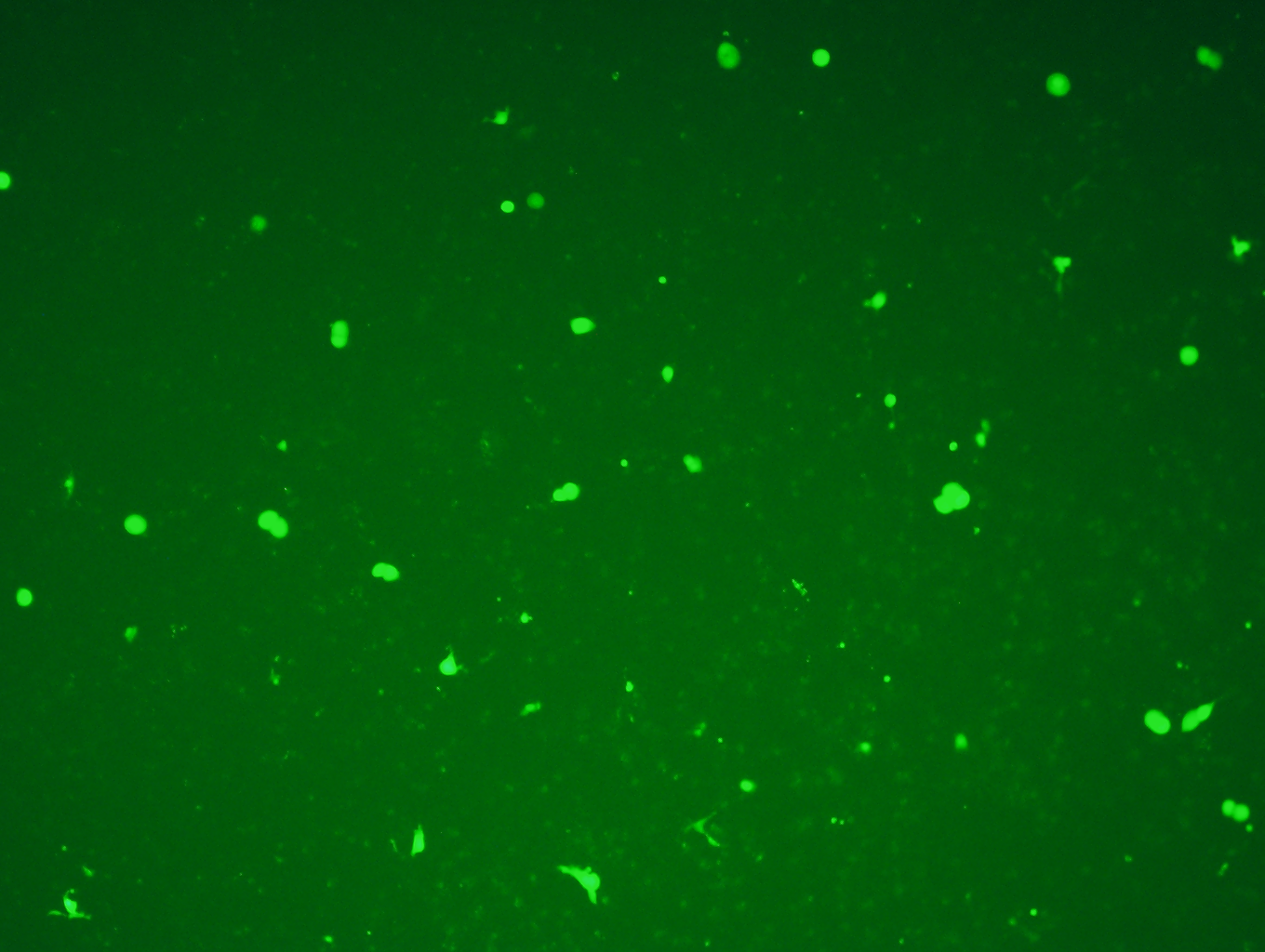

Supplement: Supplementary file 2 — Source data Fig. 1 [file 44318_2025_363_MOESM2_ESM.zip › Figure 1/1I/Ephrin A1 (3).tif]

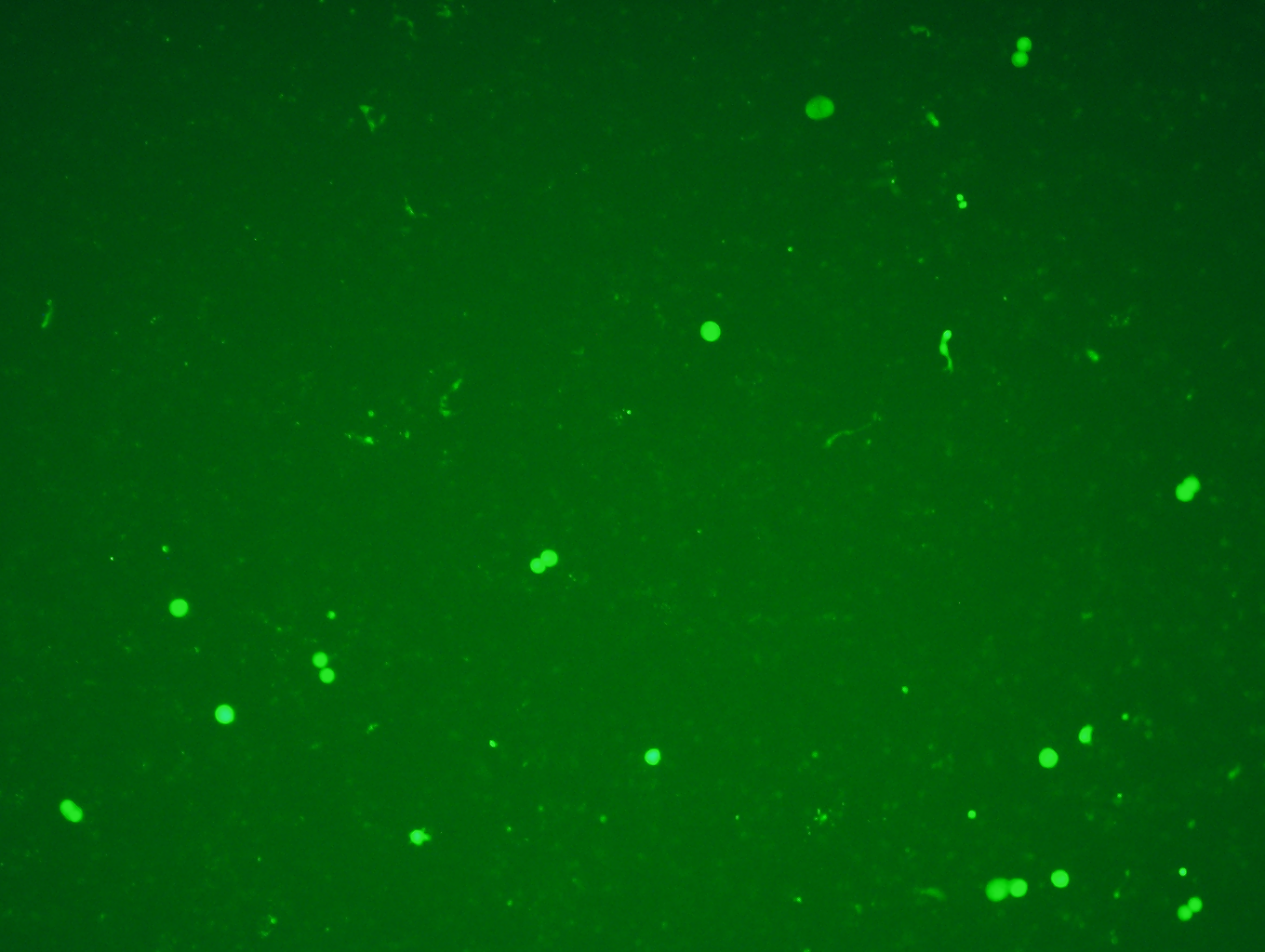

Supplement: Supplementary file 2 — Source data Fig. 1 [file 44318_2025_363_MOESM2_ESM.zip › Figure 1/1I/Ephrin A1 (4).tif]

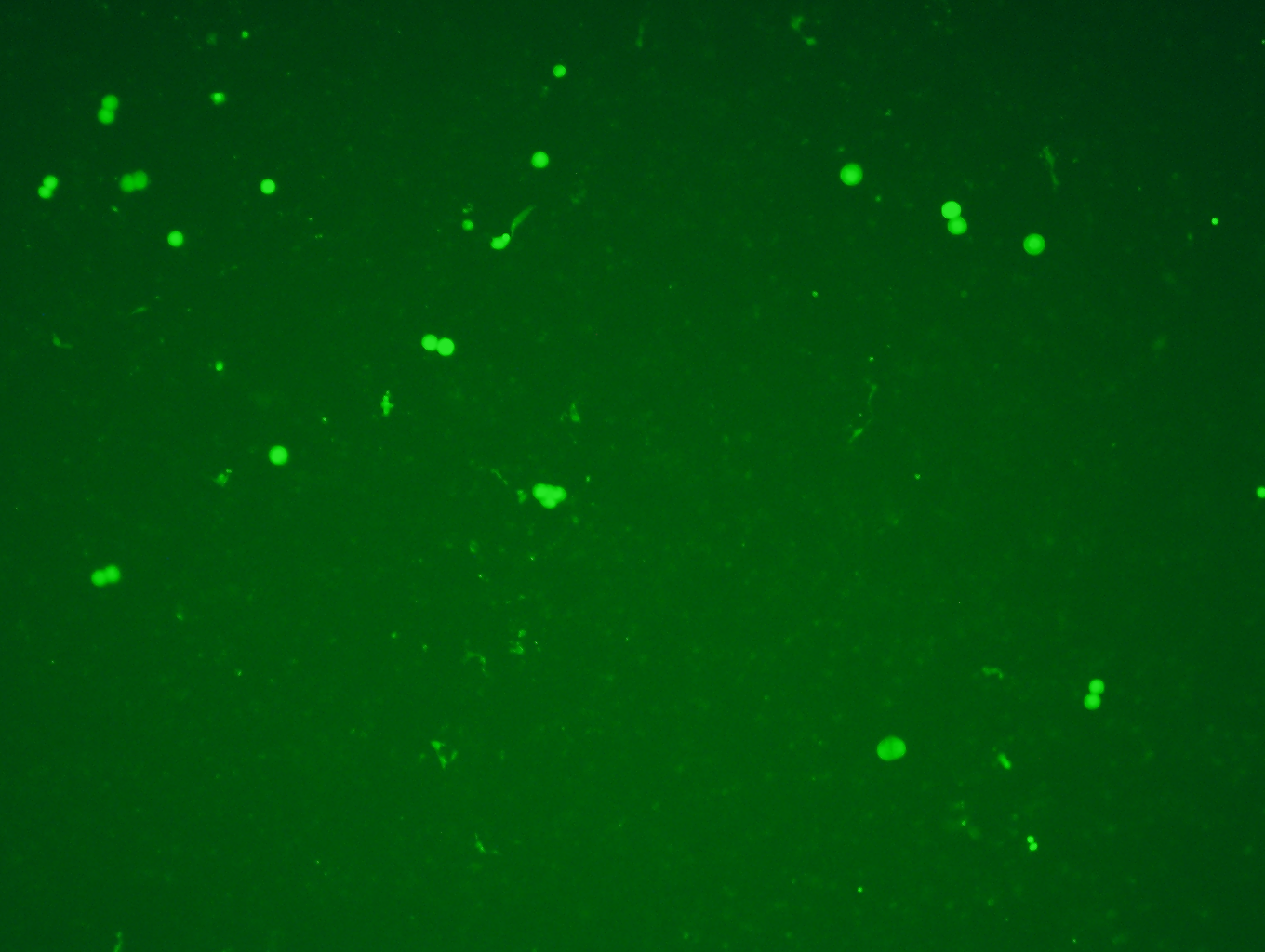

Supplement: Supplementary file 2 — Source data Fig. 1 [file 44318_2025_363_MOESM2_ESM.zip › Figure 1/1I/Ephrin A1 (5).tif]

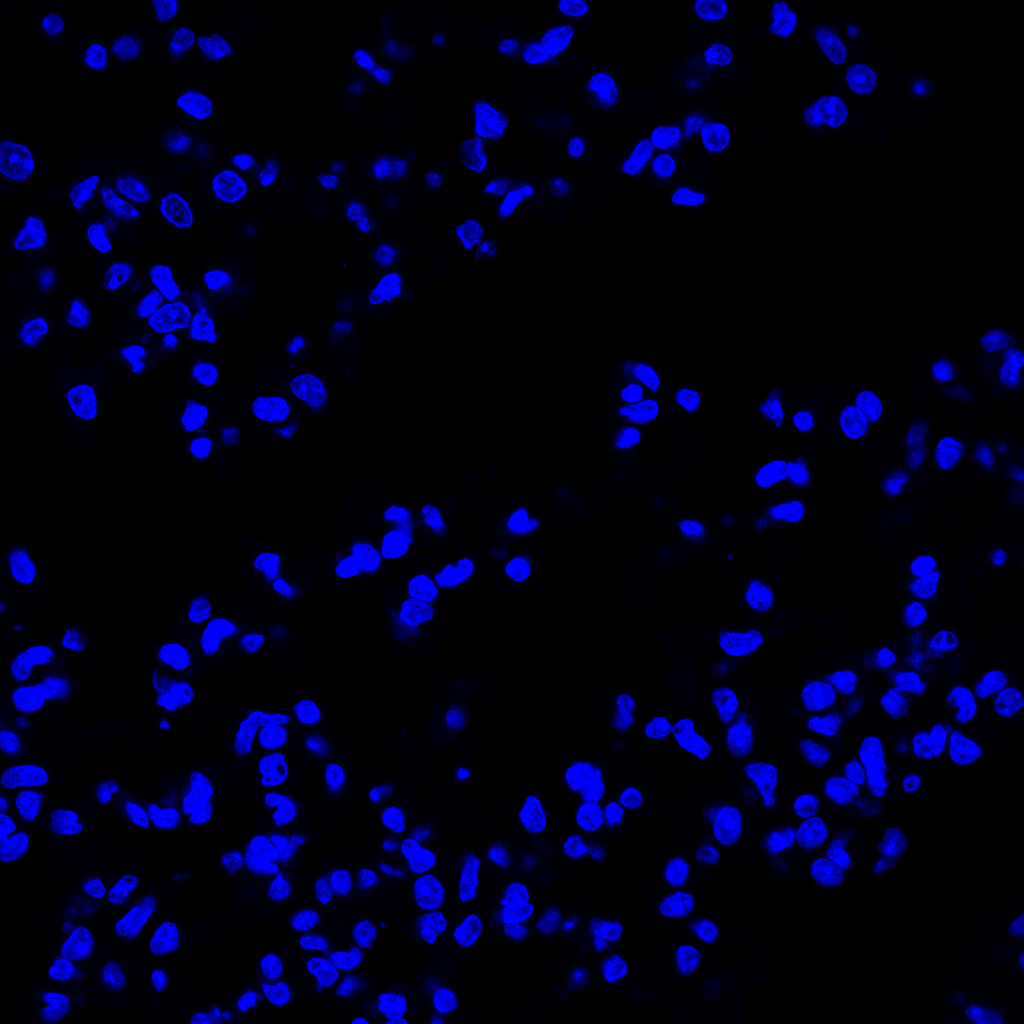

Supplement: Supplementary file 2 — Source data Fig. 1 [file 44318_2025_363_MOESM2_ESM.zip › Figure 1/1L/Control/CTRL_00022_C001T001.tif]

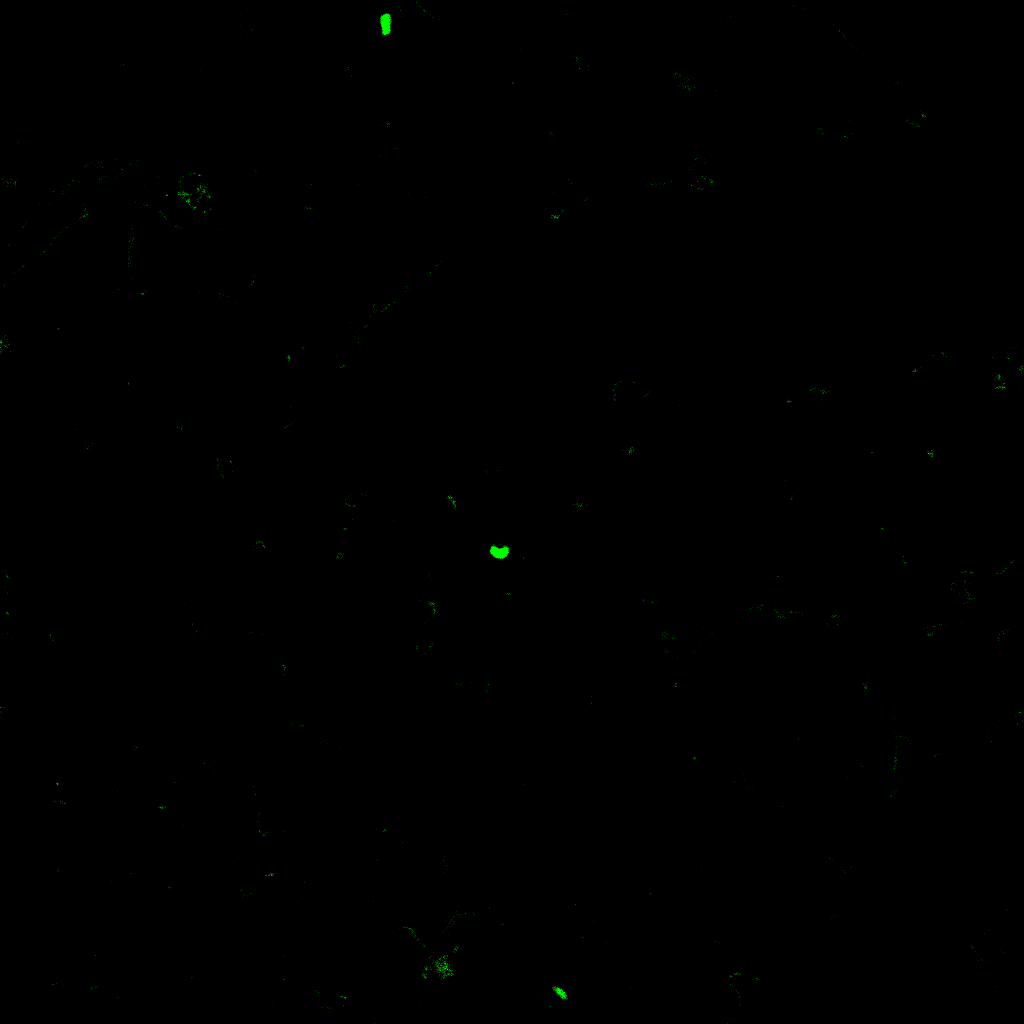

Supplement: Supplementary file 2 — Source data Fig. 1 [file 44318_2025_363_MOESM2_ESM.zip › Figure 1/1L/Control/CTRL_00022_C002T001.tif]

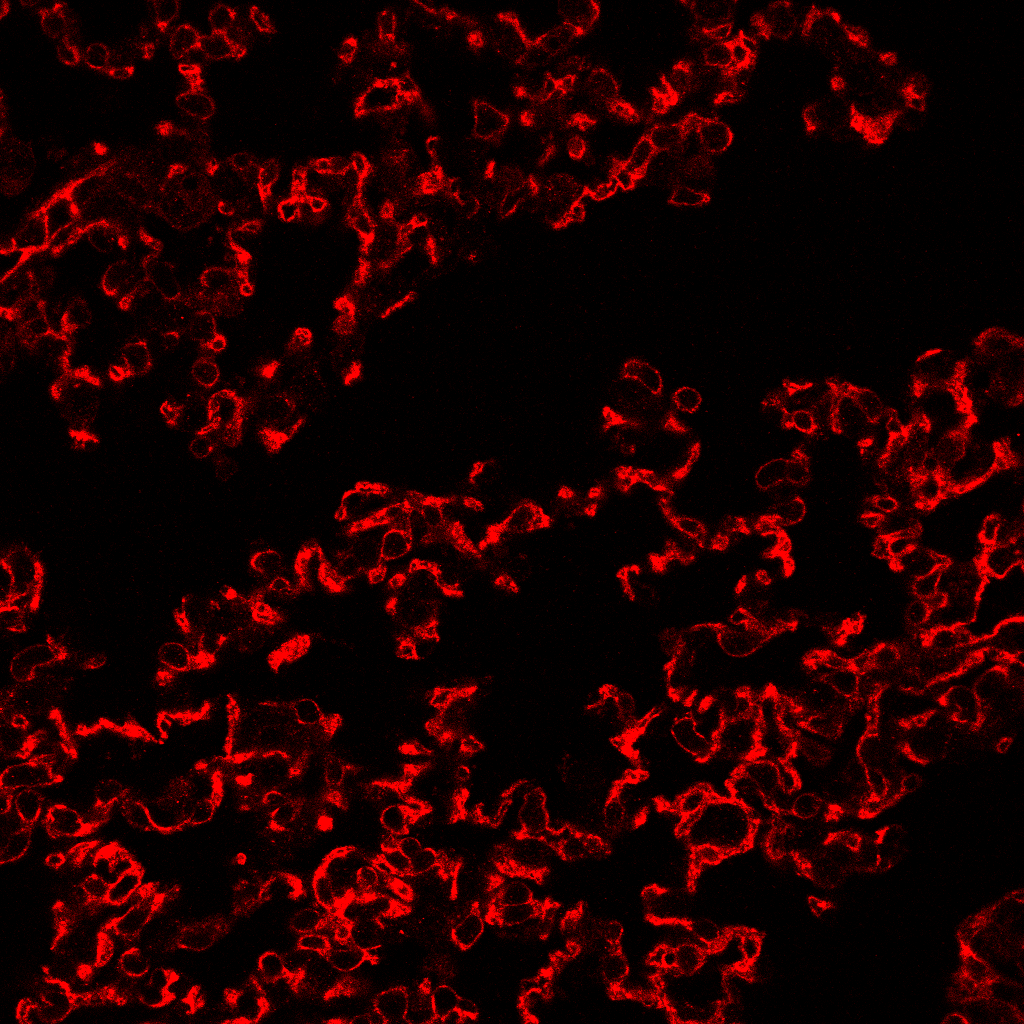

Supplement: Supplementary file 2 — Source data Fig. 1 [file 44318_2025_363_MOESM2_ESM.zip › Figure 1/1L/Control/CTRL_00022_C003T001.tif]

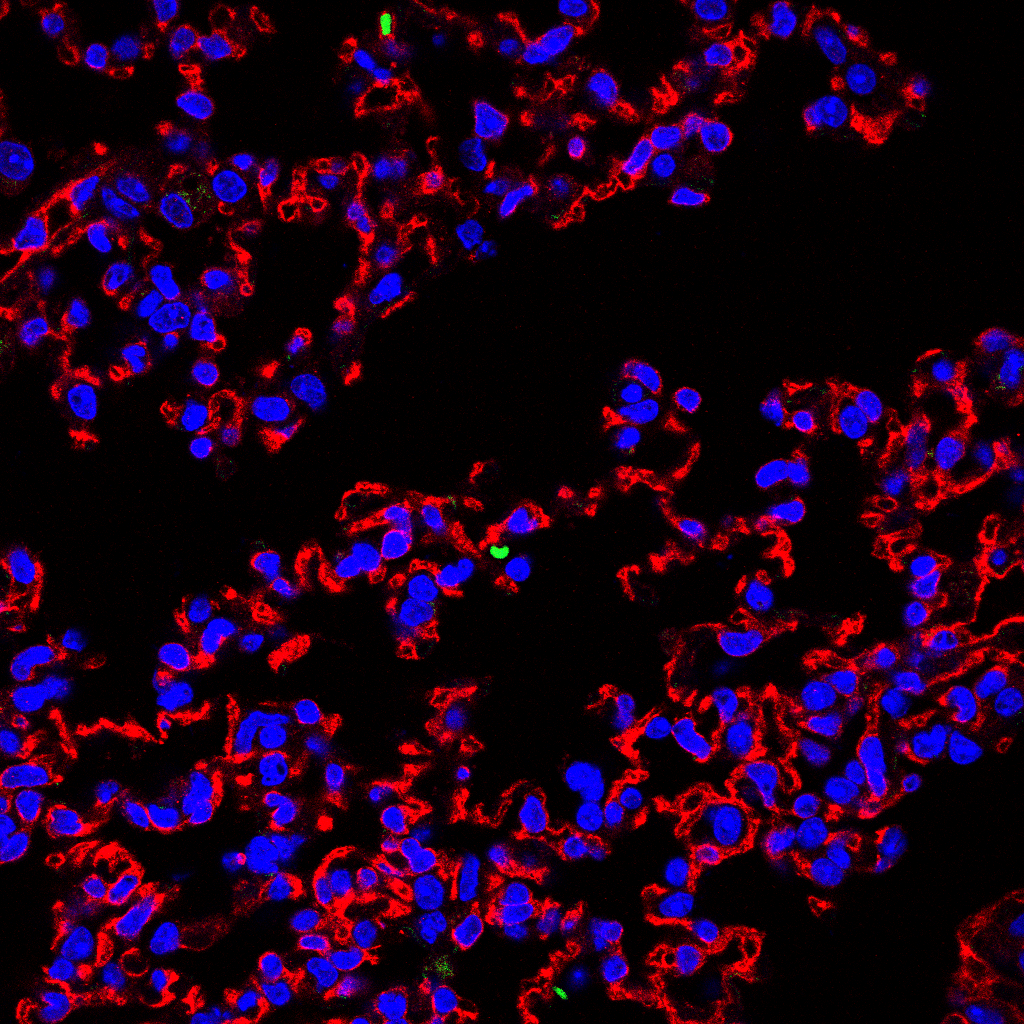

Supplement: Supplementary file 2 — Source data Fig. 1 [file 44318_2025_363_MOESM2_ESM.zip › Figure 1/1L/Control/CTRL_00022_T001.tif]

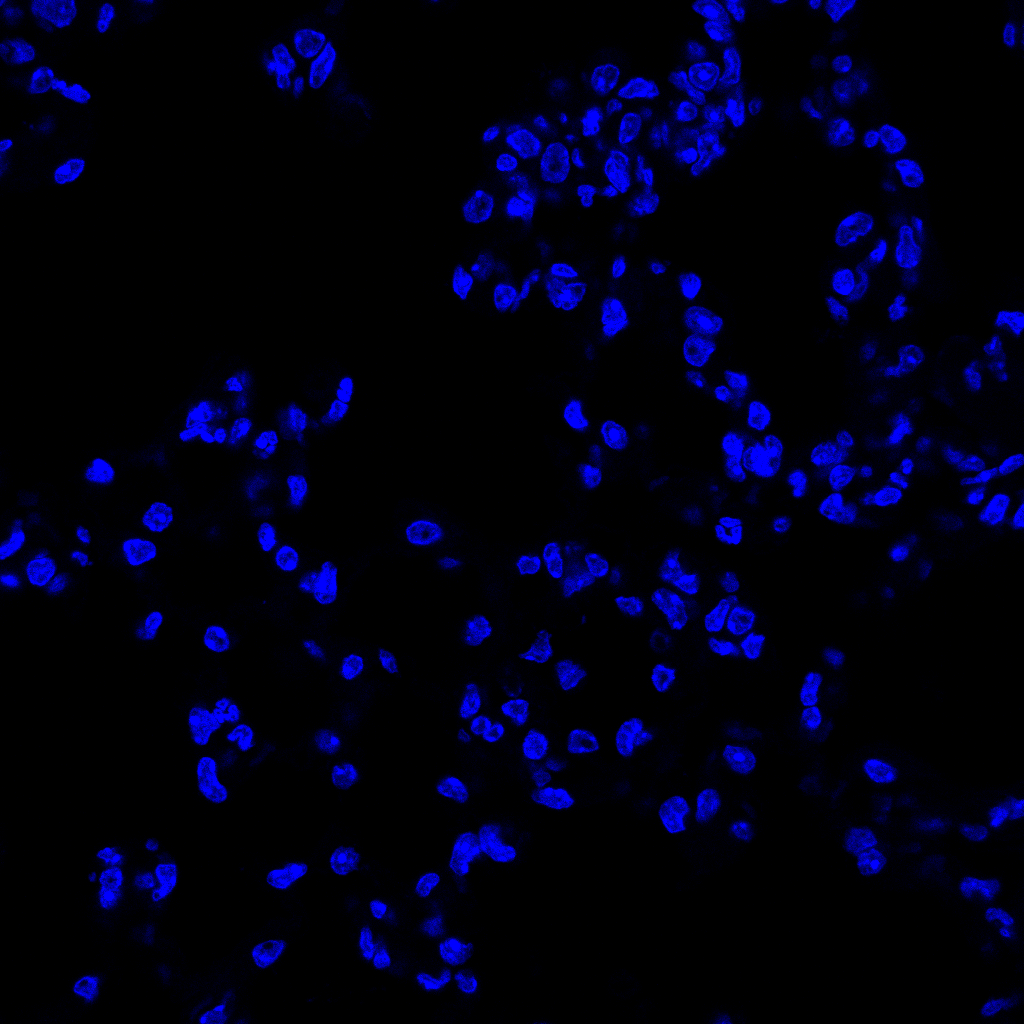

Supplement: Supplementary file 2 — Source data Fig. 1 [file 44318_2025_363_MOESM2_ESM.zip › Figure 1/1L/Ephrin A1/EA1-3_0004_C001T001.tif]

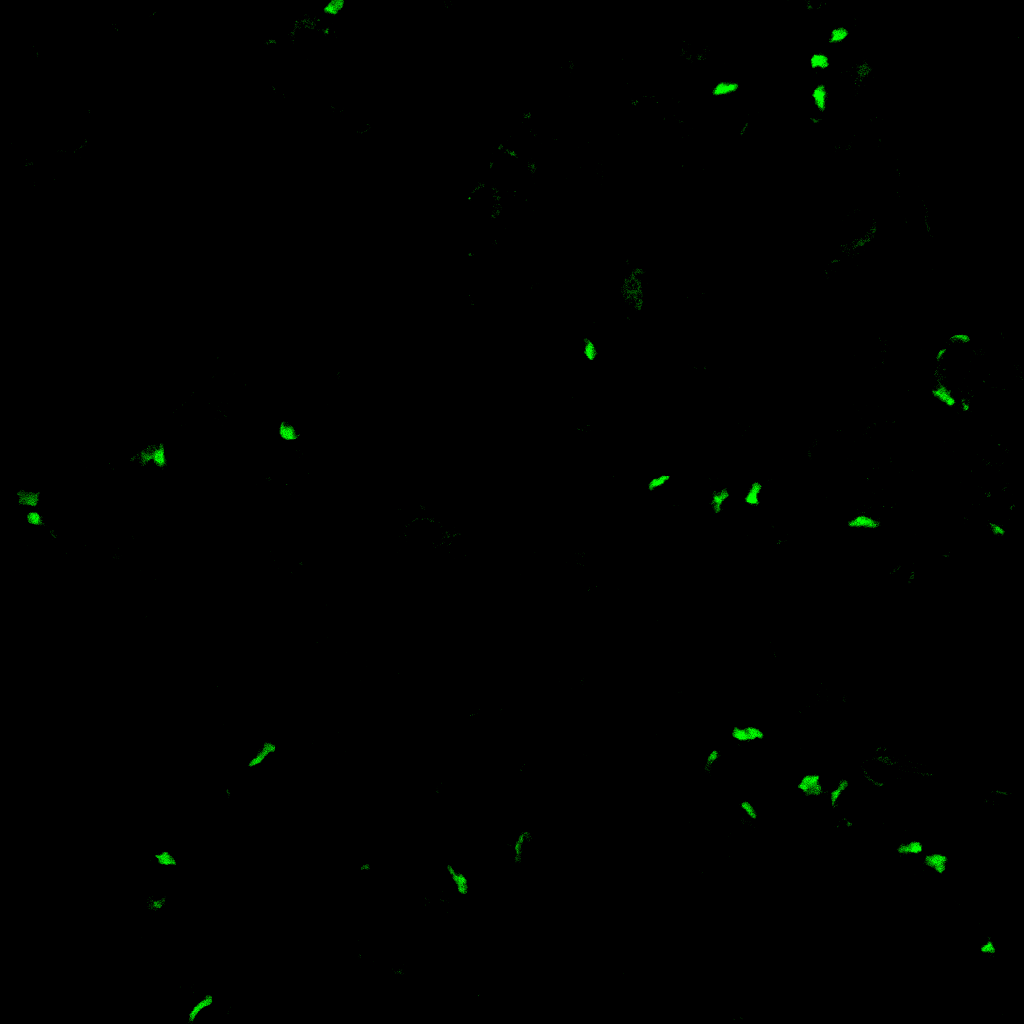

Supplement: Supplementary file 2 — Source data Fig. 1 [file 44318_2025_363_MOESM2_ESM.zip › Figure 1/1L/Ephrin A1/EA1-3_0004_C002T001.tif]

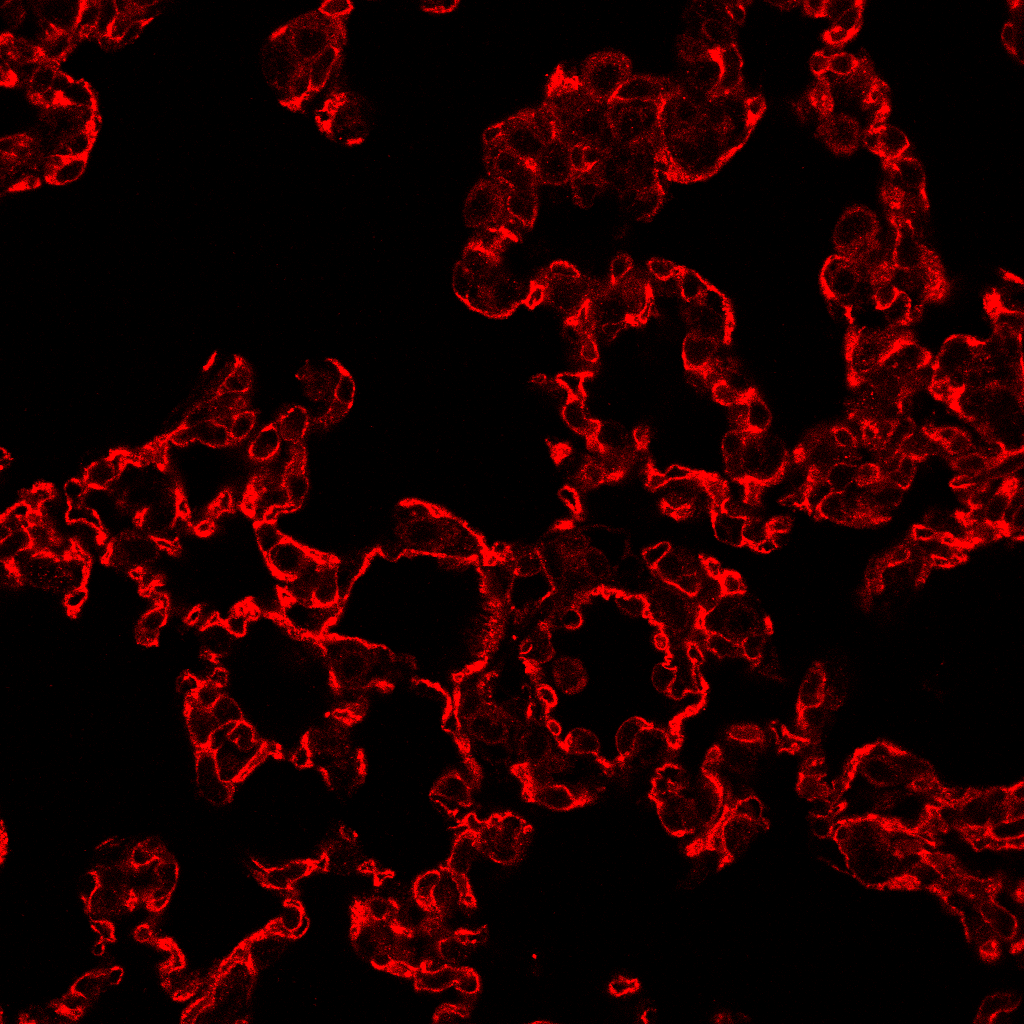

Supplement: Supplementary file 2 — Source data Fig. 1 [file 44318_2025_363_MOESM2_ESM.zip › Figure 1/1L/Ephrin A1/EA1-3_0004_C003T001.tif]

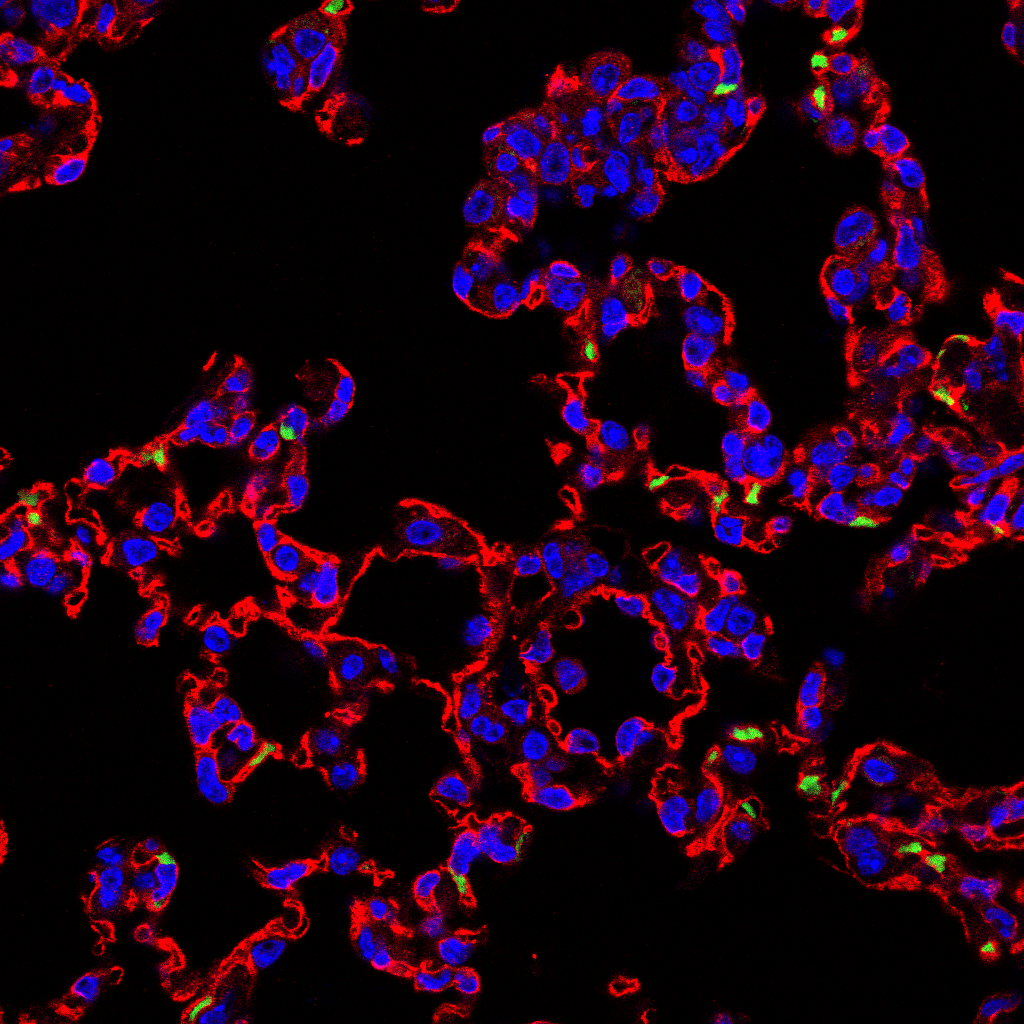

Supplement: Supplementary file 2 — Source data Fig. 1 [file 44318_2025_363_MOESM2_ESM.zip › Figure 1/1L/Ephrin A1/EA1-3_0004_T001.tif]

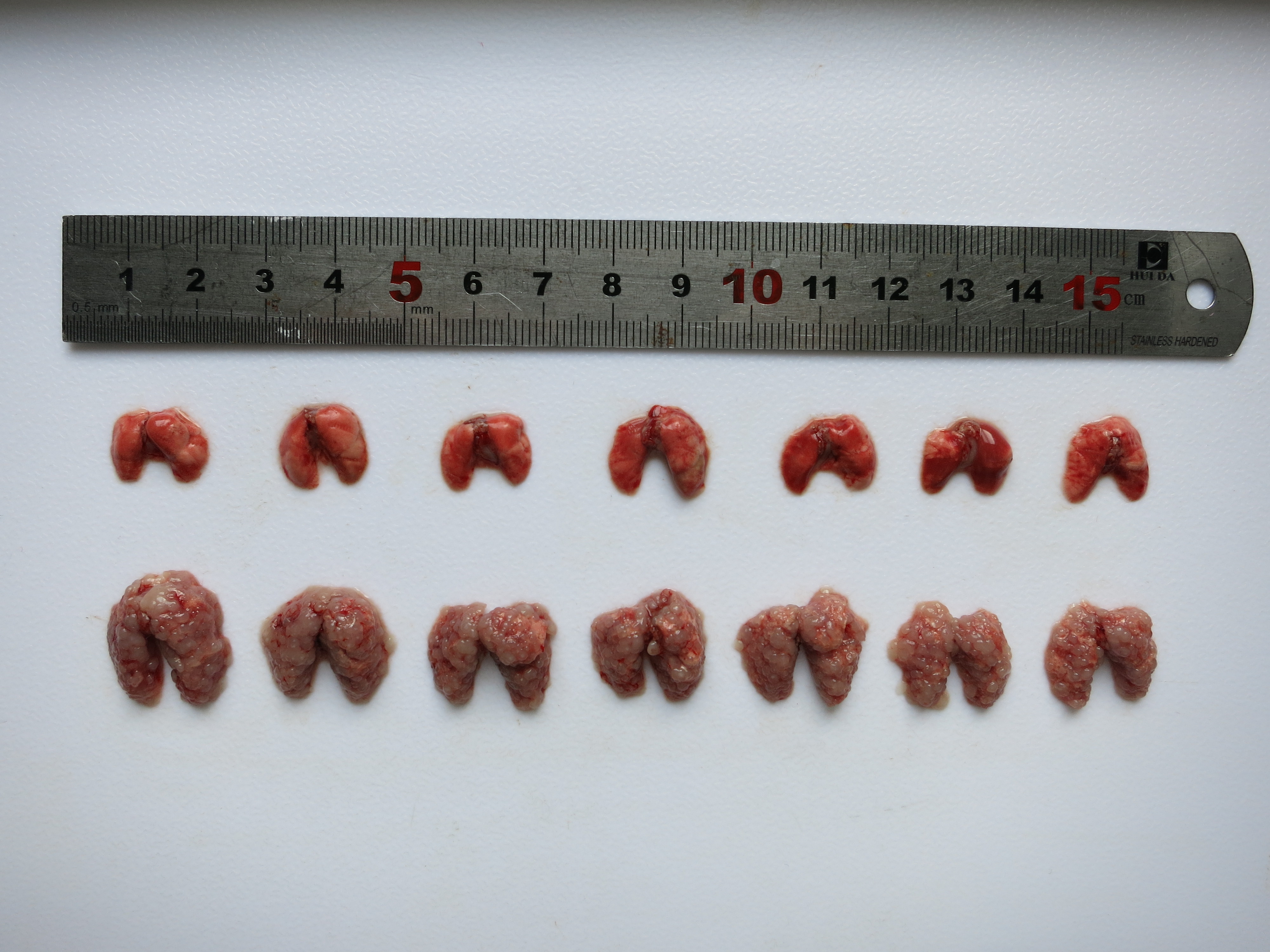

Supplement: Supplementary file 2 — Source data Fig. 1 [file 44318_2025_363_MOESM2_ESM.zip › Figure 1/1N/1N.JPG]

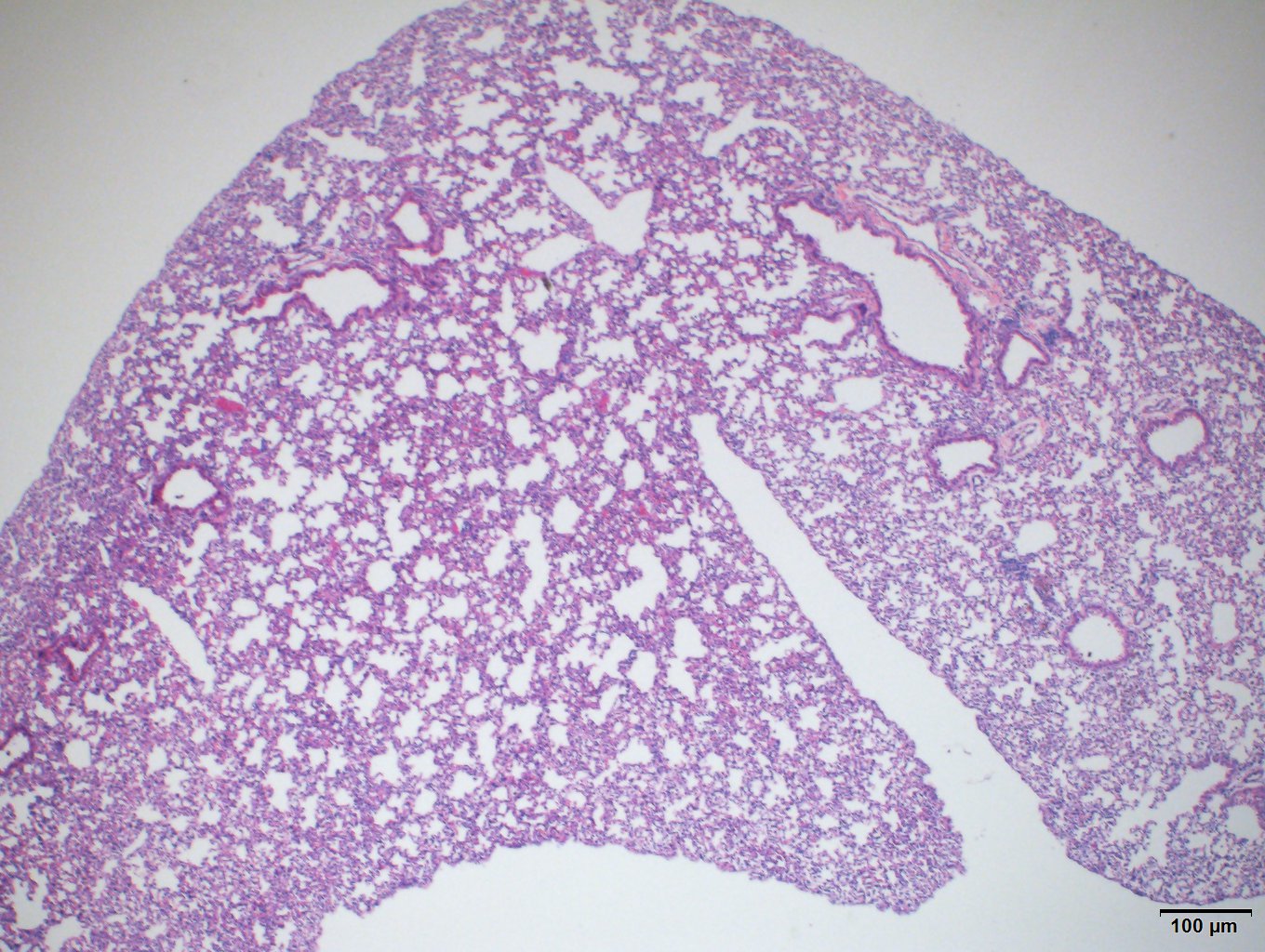

Supplement: Supplementary file 2 — Source data Fig. 1 [file 44318_2025_363_MOESM2_ESM.zip › Figure 1/1P/Control (1).jpg]

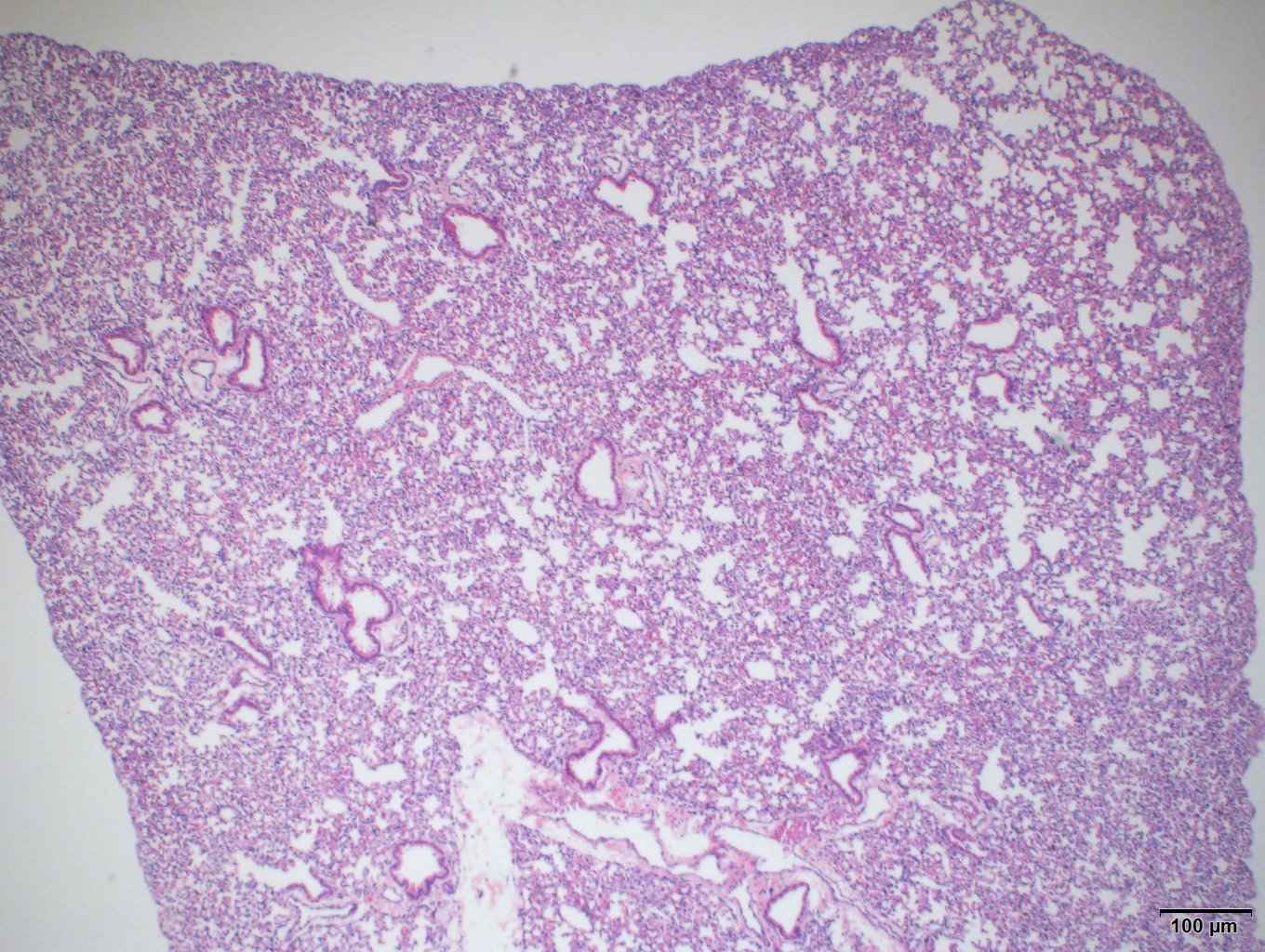

Supplement: Supplementary file 2 — Source data Fig. 1 [file 44318_2025_363_MOESM2_ESM.zip › Figure 1/1P/Control (2).jpg]

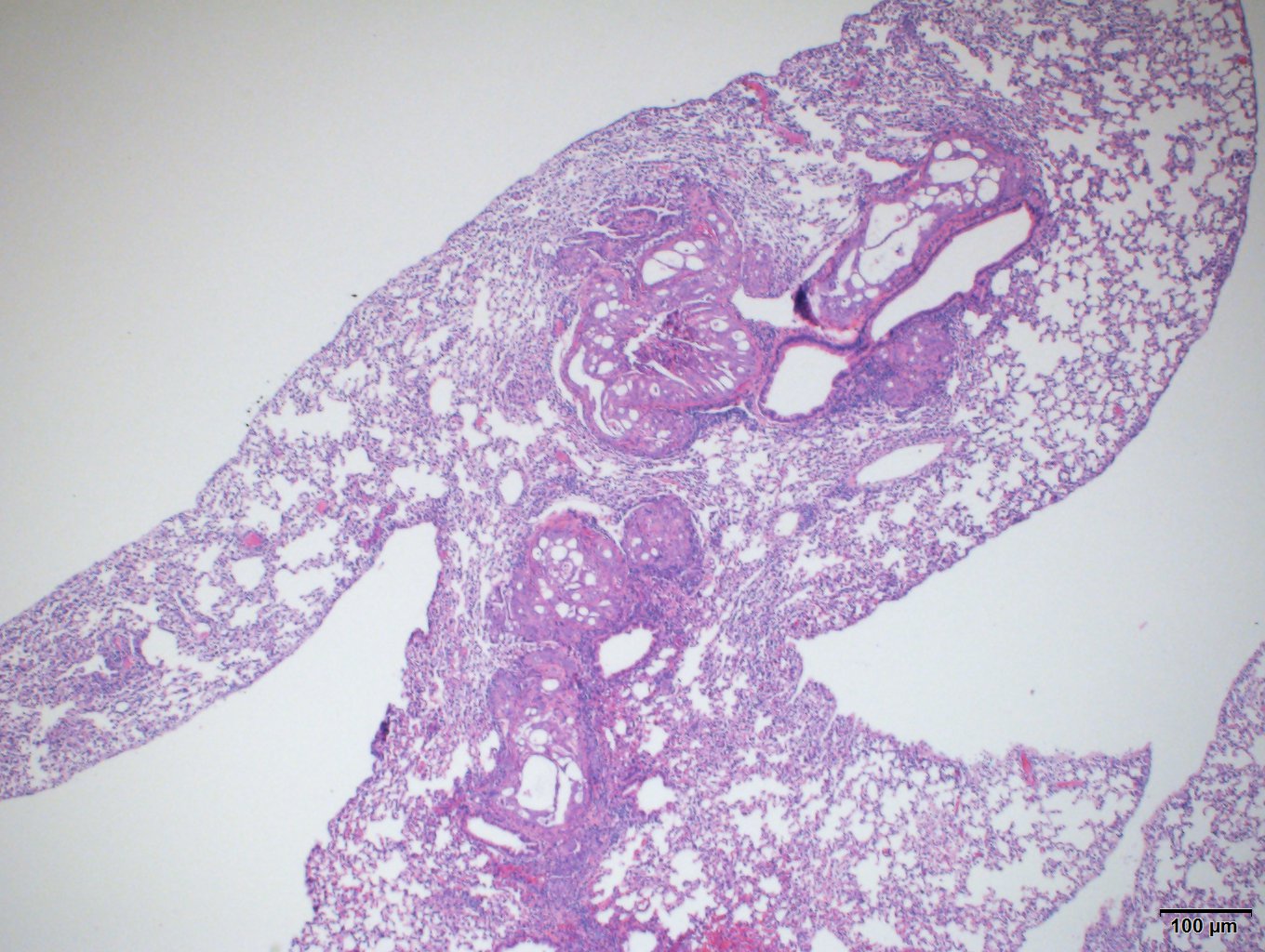

Supplement: Supplementary file 2 — Source data Fig. 1 [file 44318_2025_363_MOESM2_ESM.zip › Figure 1/1P/Control (3).jpg]

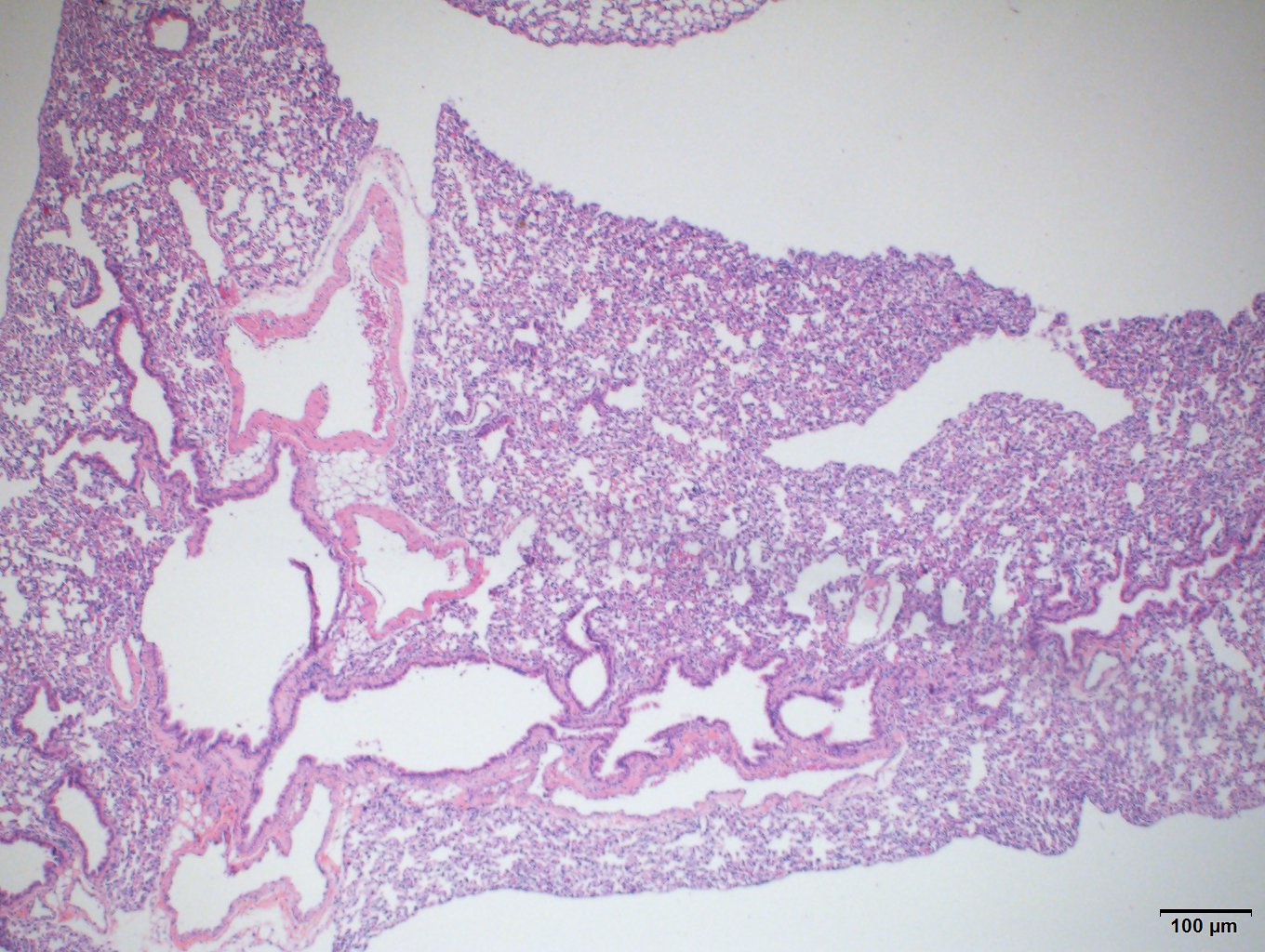

Supplement: Supplementary file 2 — Source data Fig. 1 [file 44318_2025_363_MOESM2_ESM.zip › Figure 1/1P/Control (4).jpg]

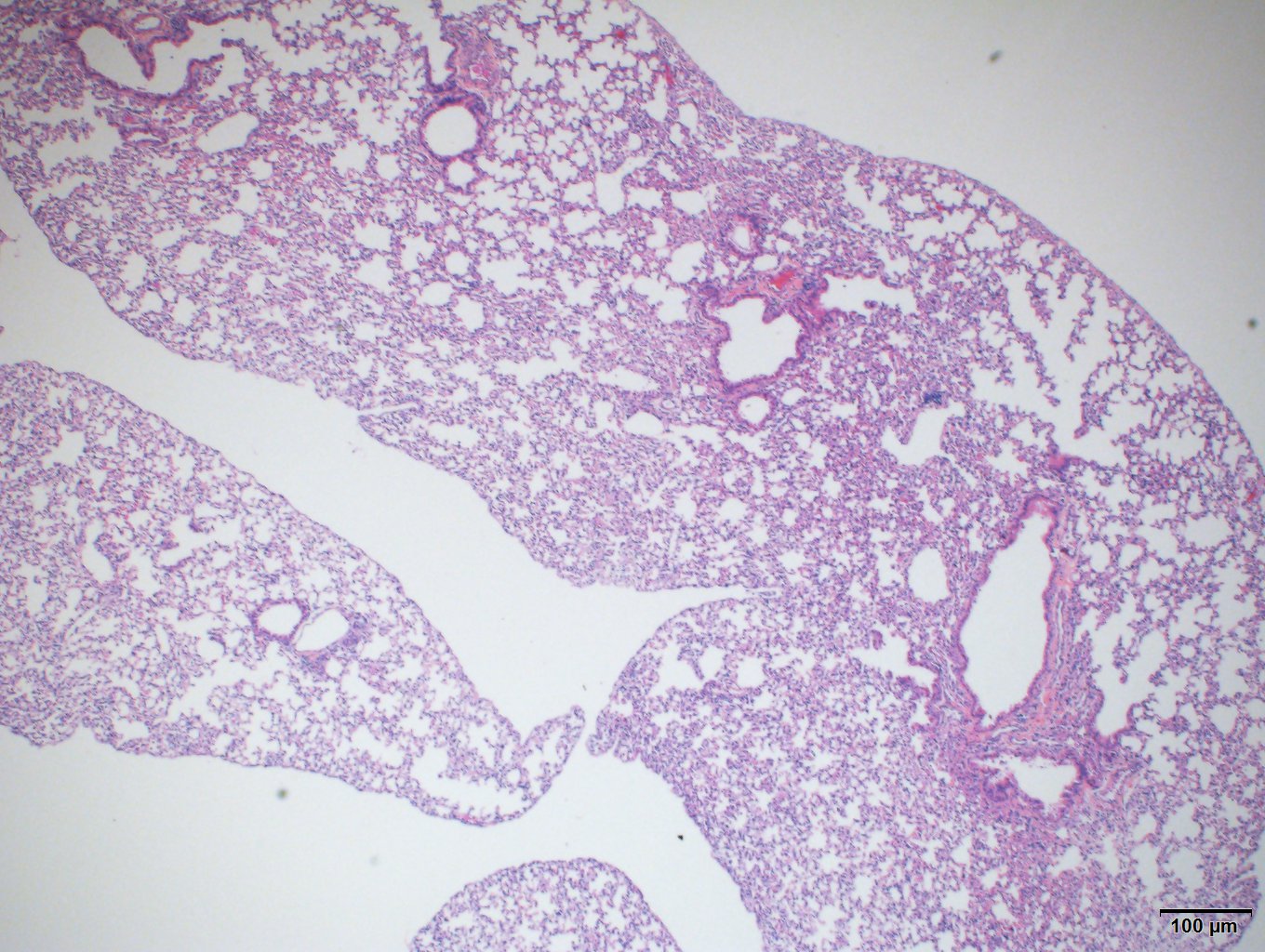

Supplement: Supplementary file 2 — Source data Fig. 1 [file 44318_2025_363_MOESM2_ESM.zip › Figure 1/1P/Control (5).jpg]

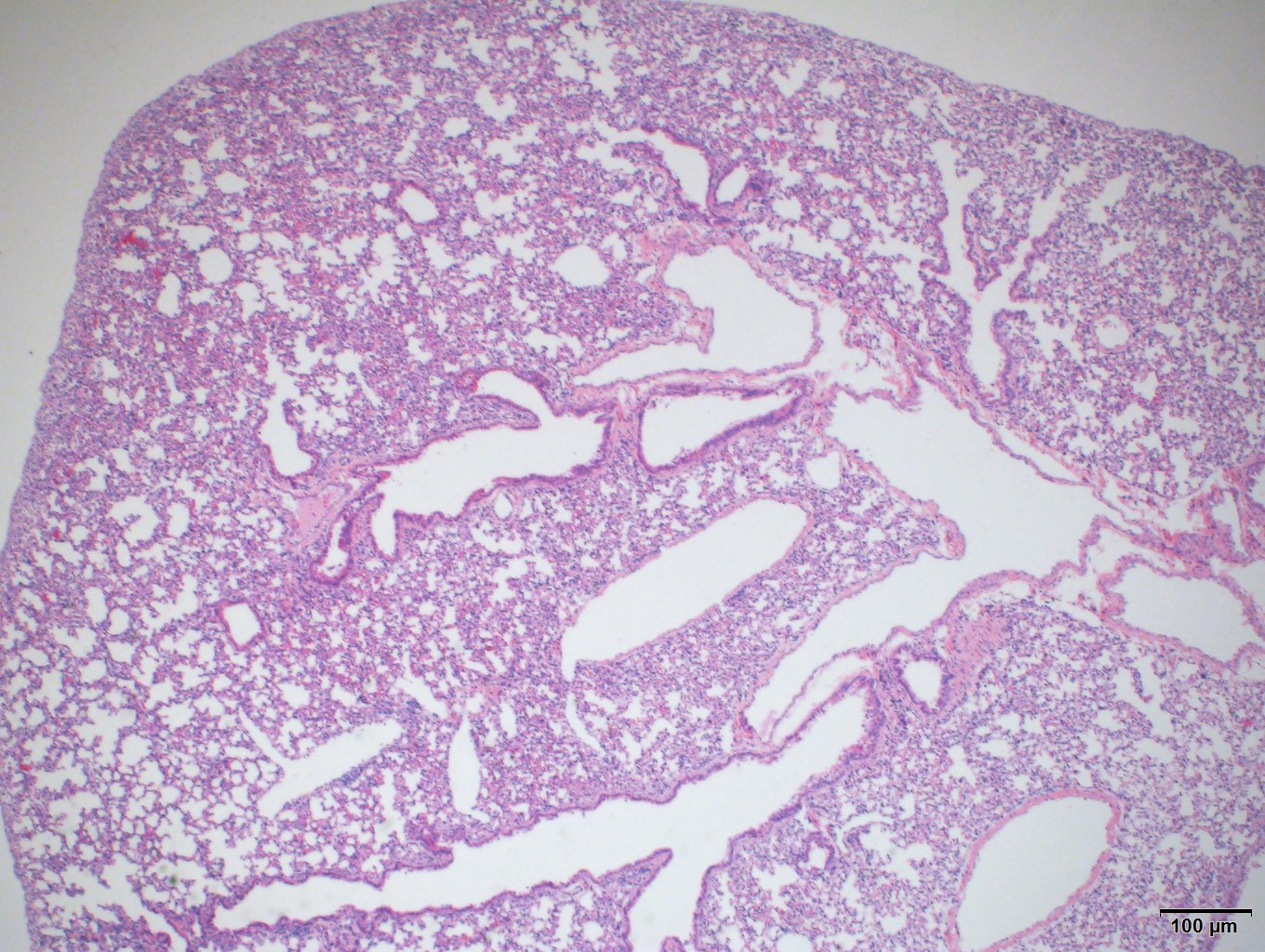

Supplement: Supplementary file 2 — Source data Fig. 1 [file 44318_2025_363_MOESM2_ESM.zip › Figure 1/1P/Control (6)-displayed in 1P.jpg]

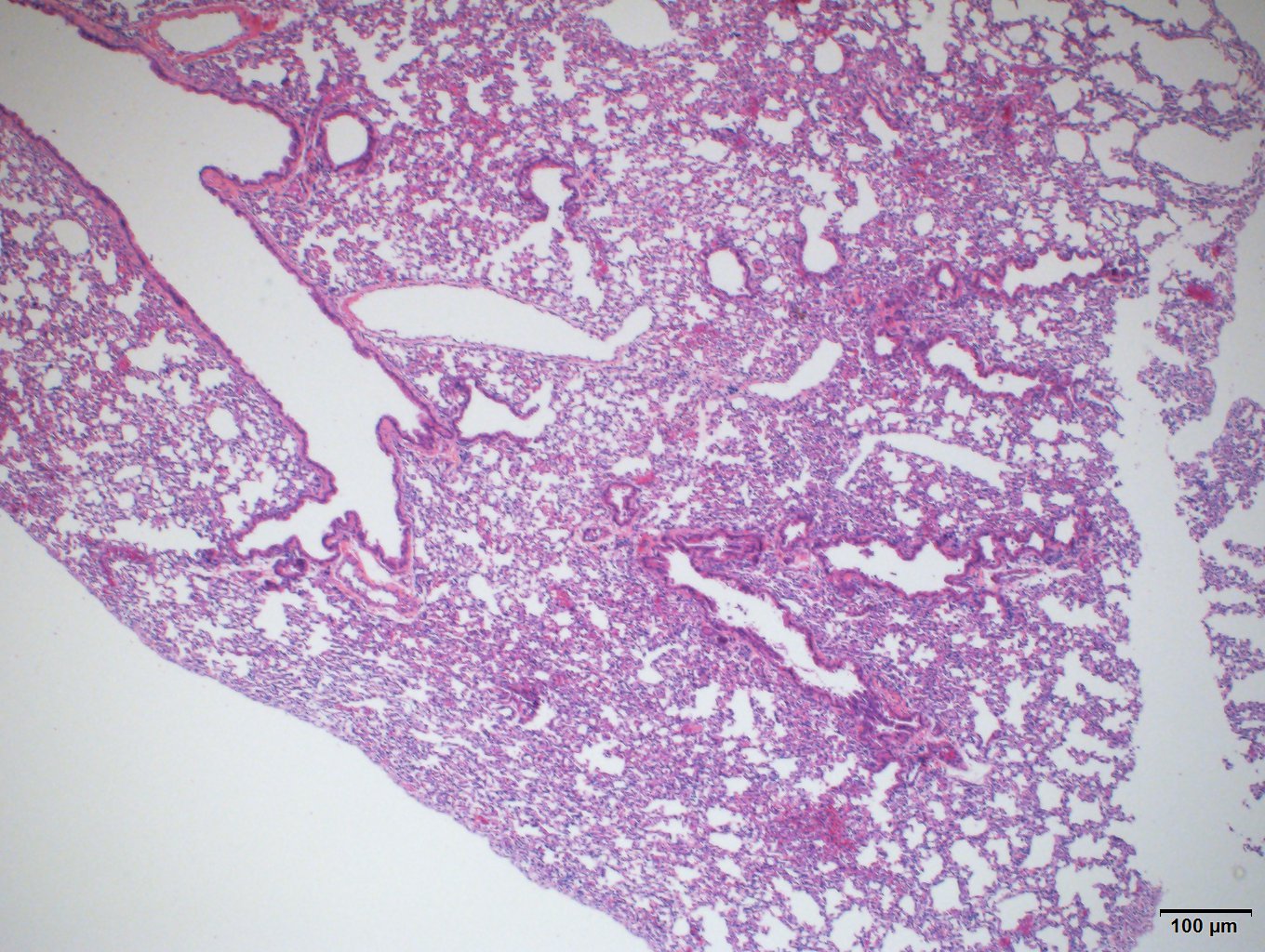

Supplement: Supplementary file 2 — Source data Fig. 1 [file 44318_2025_363_MOESM2_ESM.zip › Figure 1/1P/Control (7).jpg]

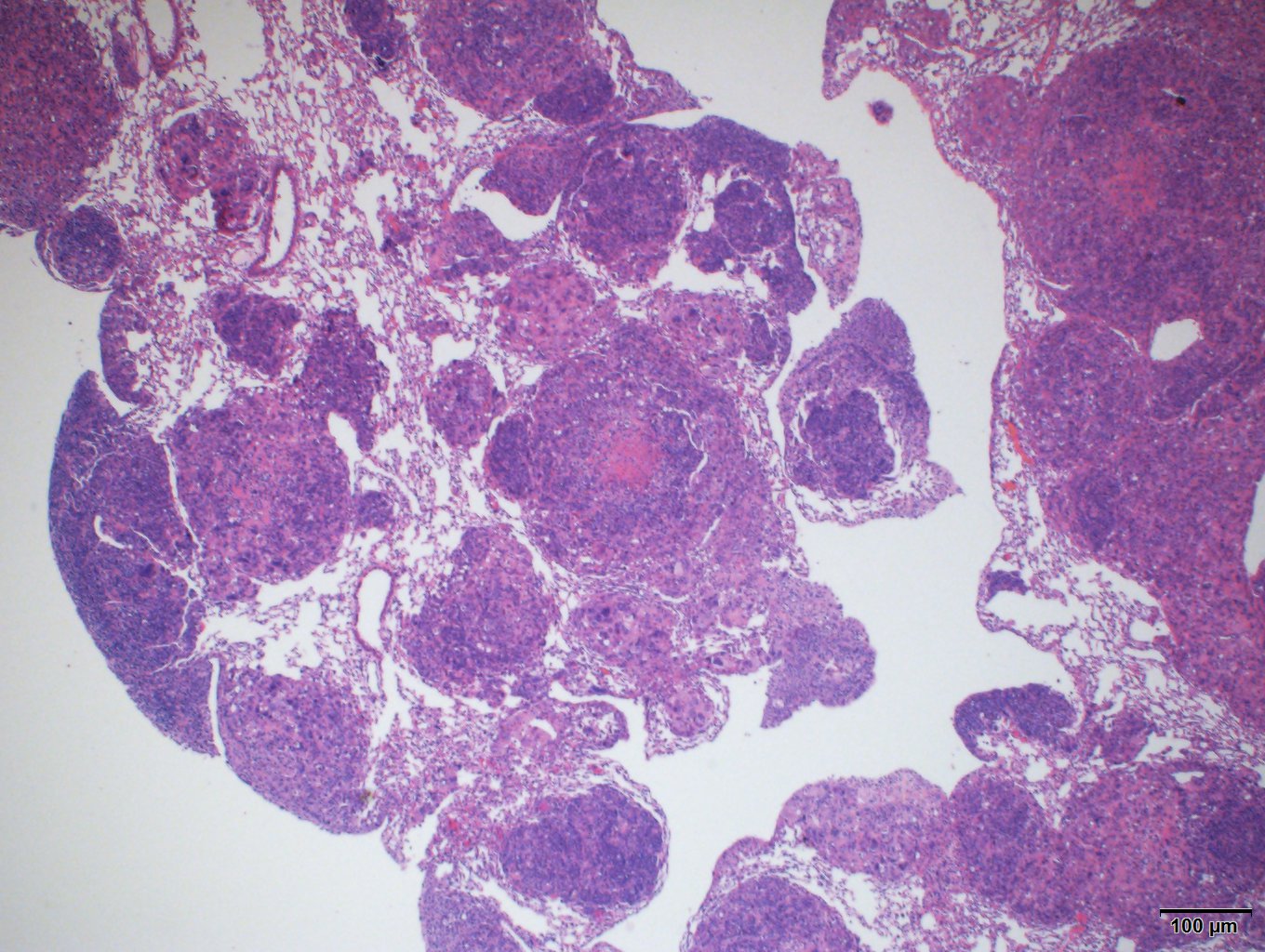

Supplement: Supplementary file 2 — Source data Fig. 1 [file 44318_2025_363_MOESM2_ESM.zip › Figure 1/1P/Ephrin A1 (1).jpg]

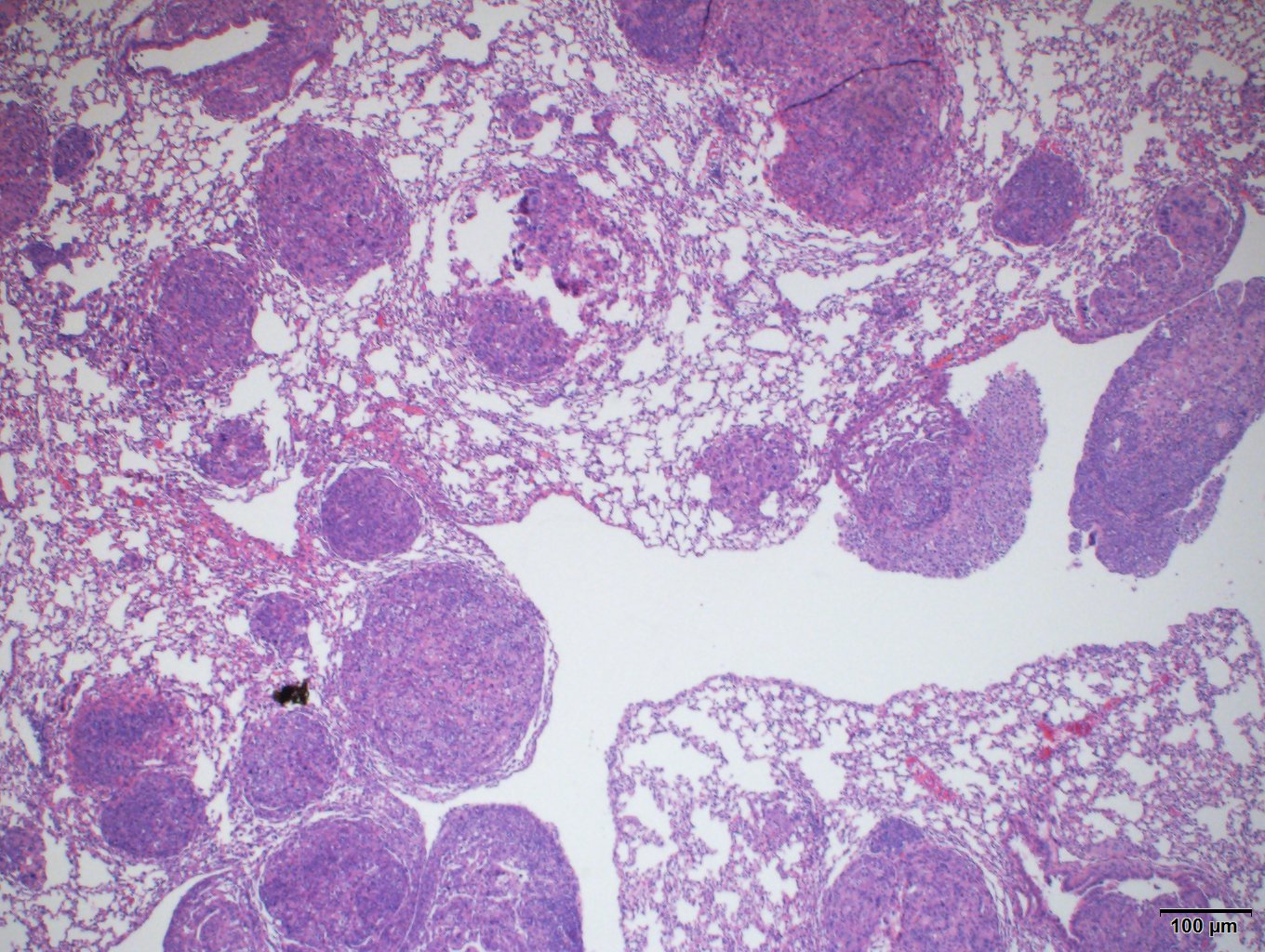

Supplement: Supplementary file 2 — Source data Fig. 1 [file 44318_2025_363_MOESM2_ESM.zip › Figure 1/1P/Ephrin A1 (2).jpg]

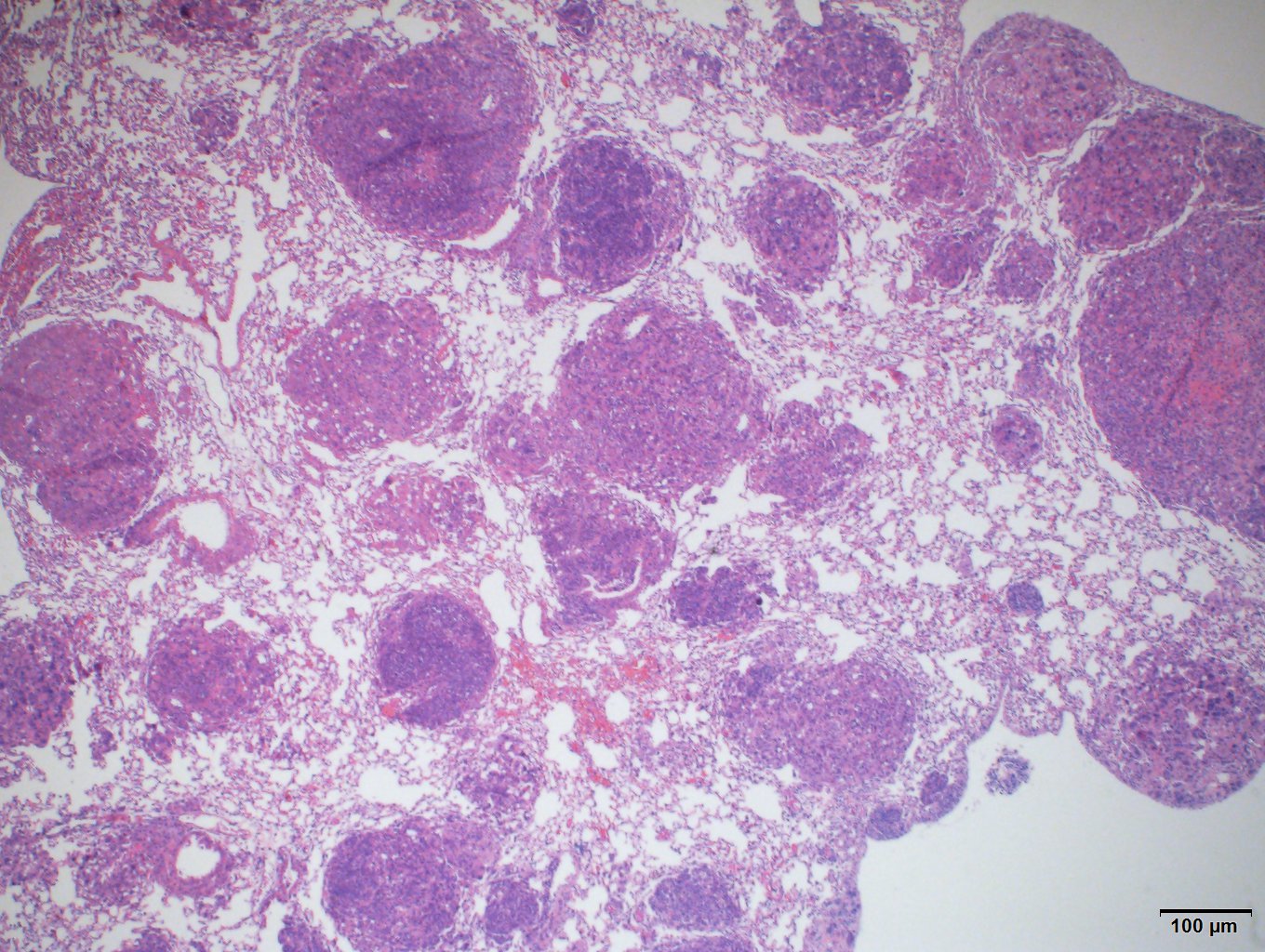

Supplement: Supplementary file 2 — Source data Fig. 1 [file 44318_2025_363_MOESM2_ESM.zip › Figure 1/1P/Ephrin A1 (3).jpg]

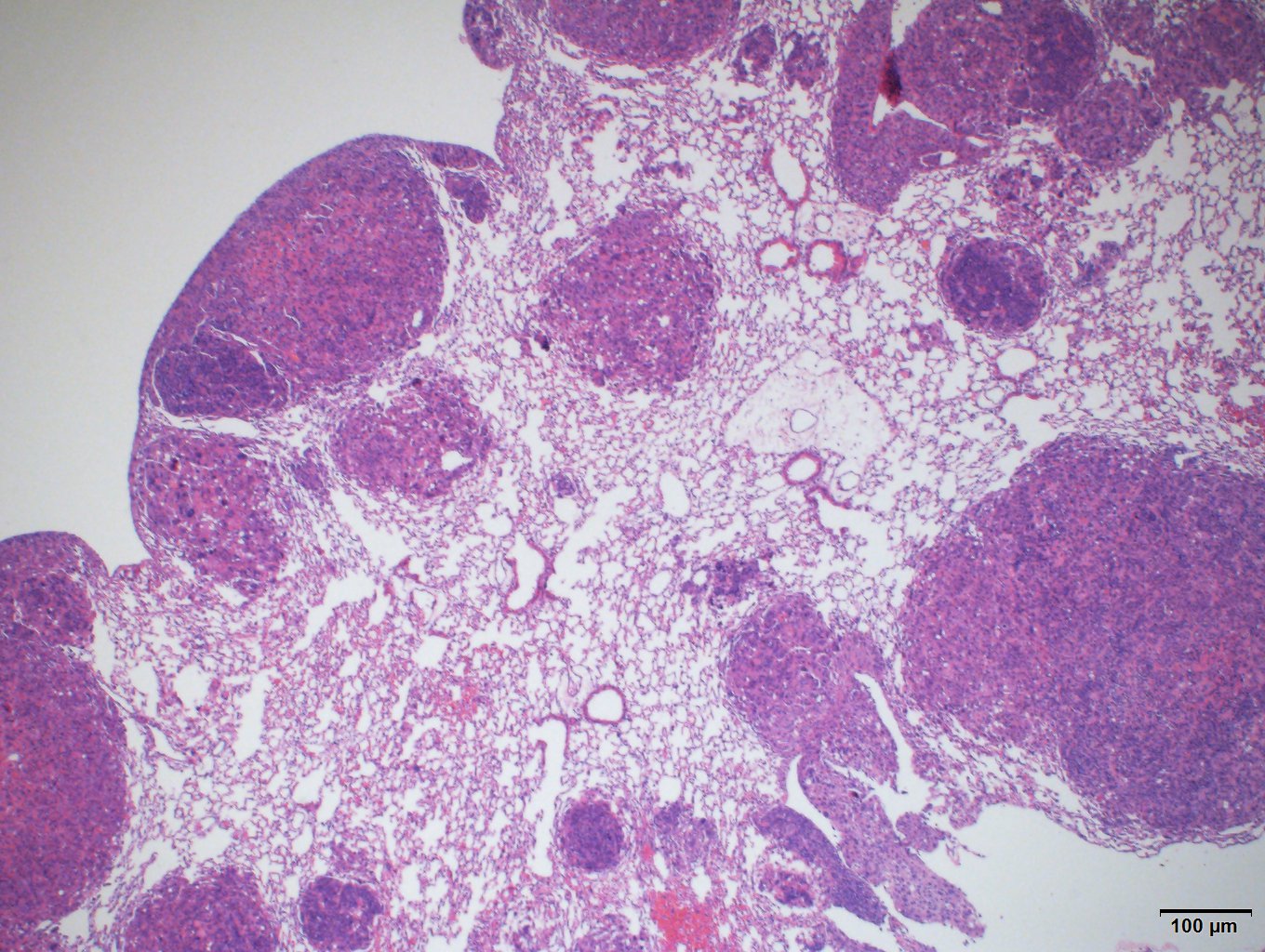

Supplement: Supplementary file 2 — Source data Fig. 1 [file 44318_2025_363_MOESM2_ESM.zip › Figure 1/1P/Ephrin A1 (4).jpg]

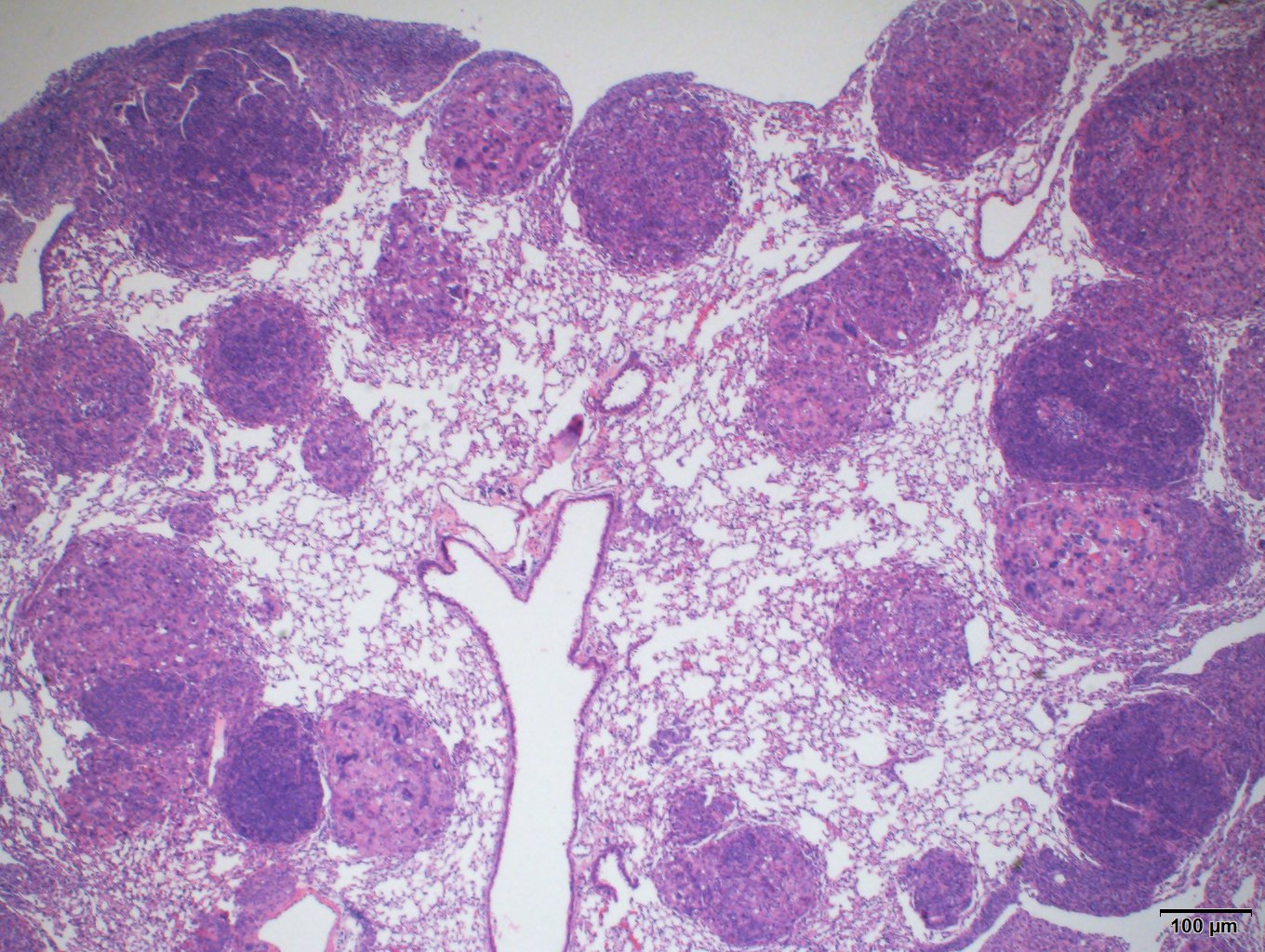

Supplement: Supplementary file 2 — Source data Fig. 1 [file 44318_2025_363_MOESM2_ESM.zip › Figure 1/1P/Ephrin A1 (5)-displayed in 1P.jpg]

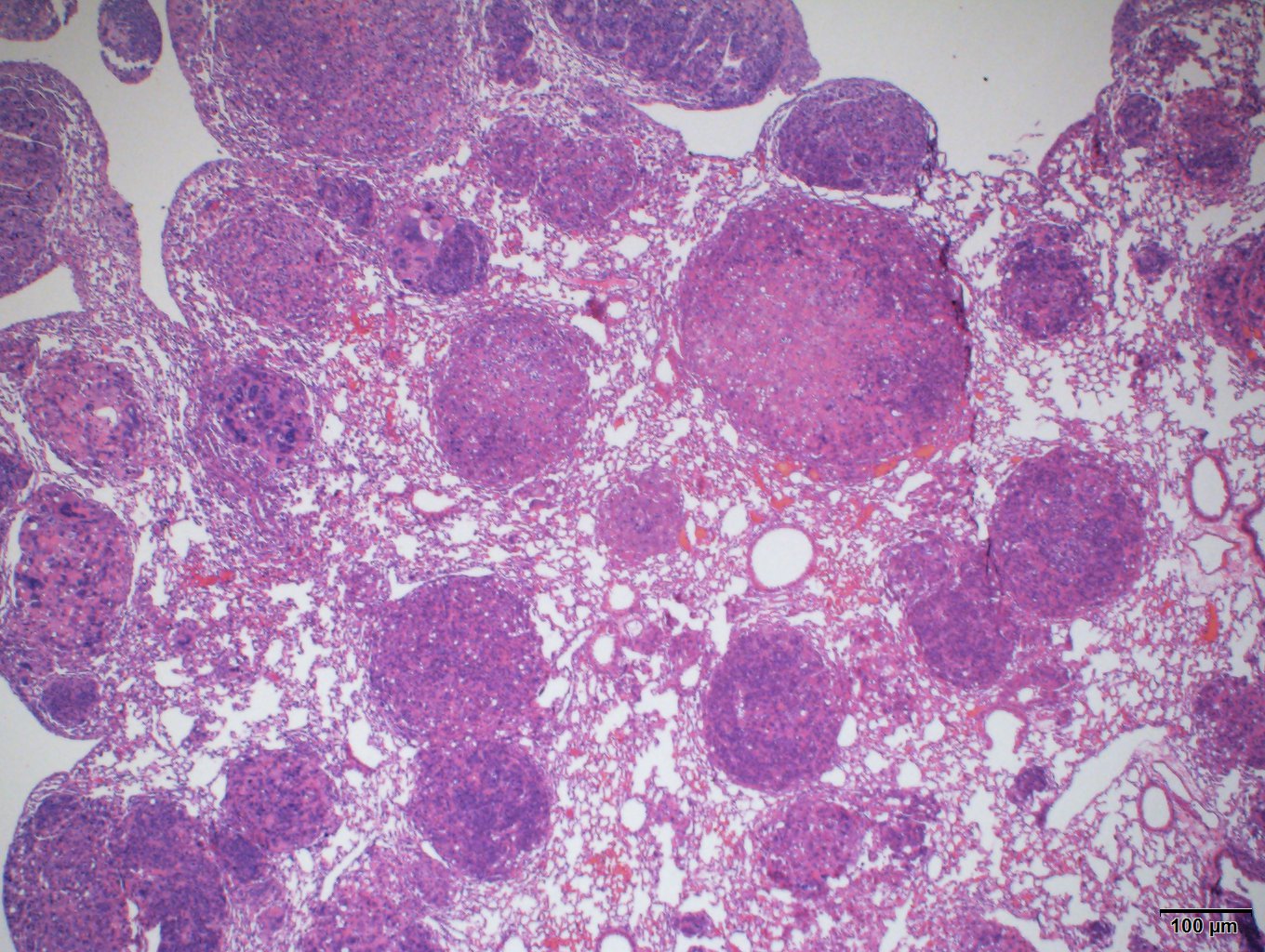

Supplement: Supplementary file 2 — Source data Fig. 1 [file 44318_2025_363_MOESM2_ESM.zip › Figure 1/1P/Ephrin A1 (6).jpg]

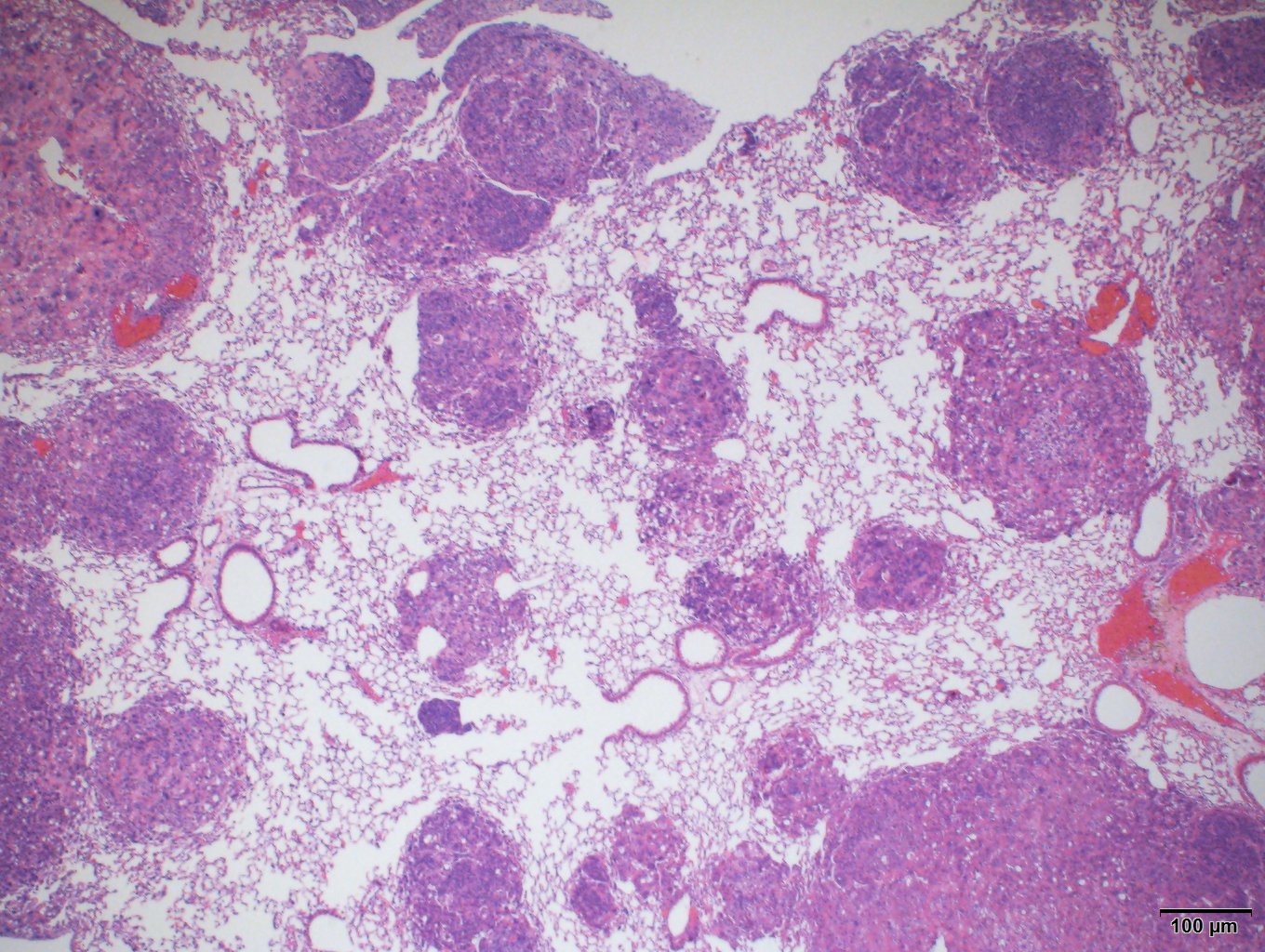

Supplement: Supplementary file 2 — Source data Fig. 1 [file 44318_2025_363_MOESM2_ESM.zip › Figure 1/1P/Ephrin A1 (7).jpg]

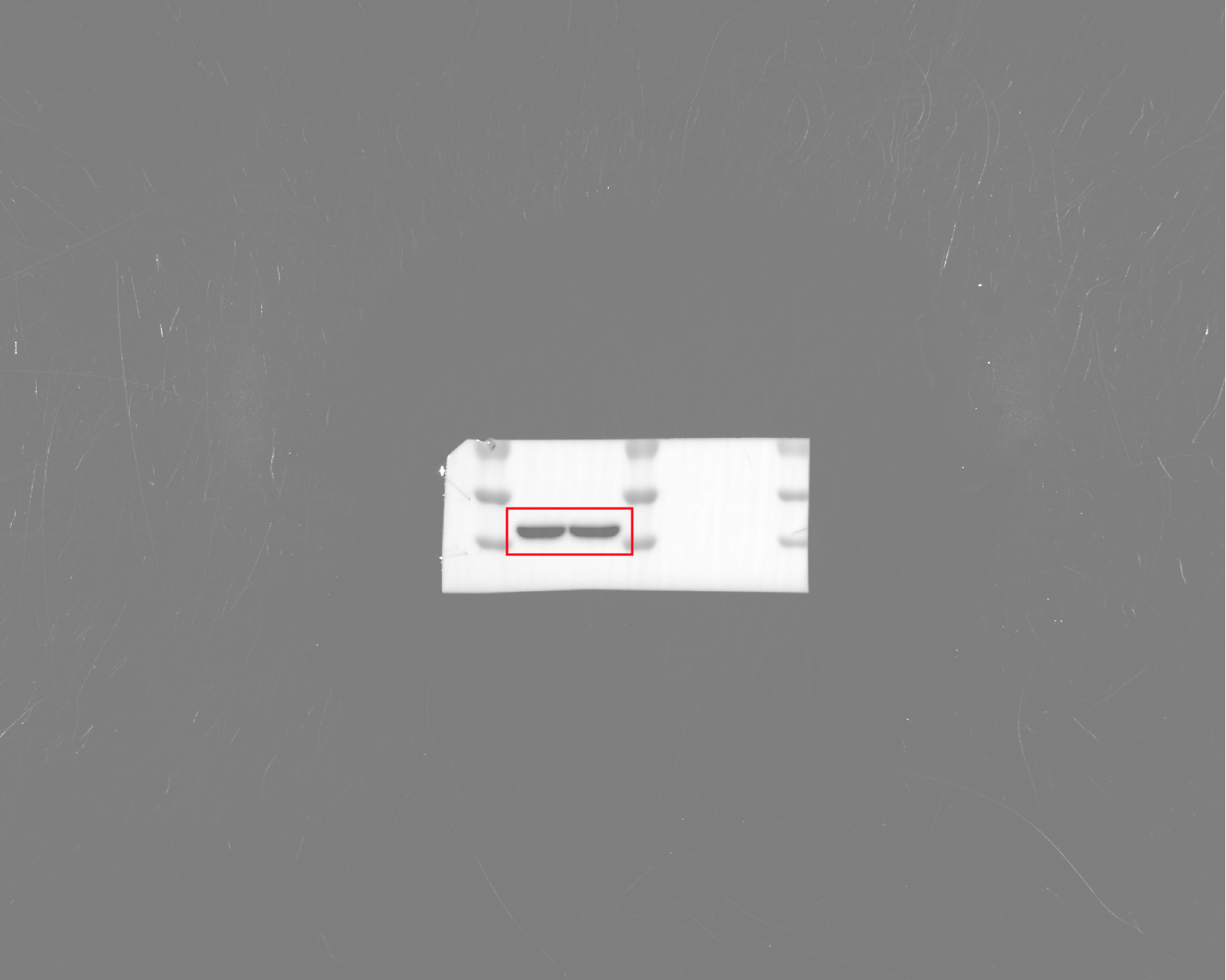

Supplement: Supplementary file 3 — Source data Fig. 2 [file 44318_2025_363_MOESM3_ESM.zip › Figure 2/2A/actin .tif]

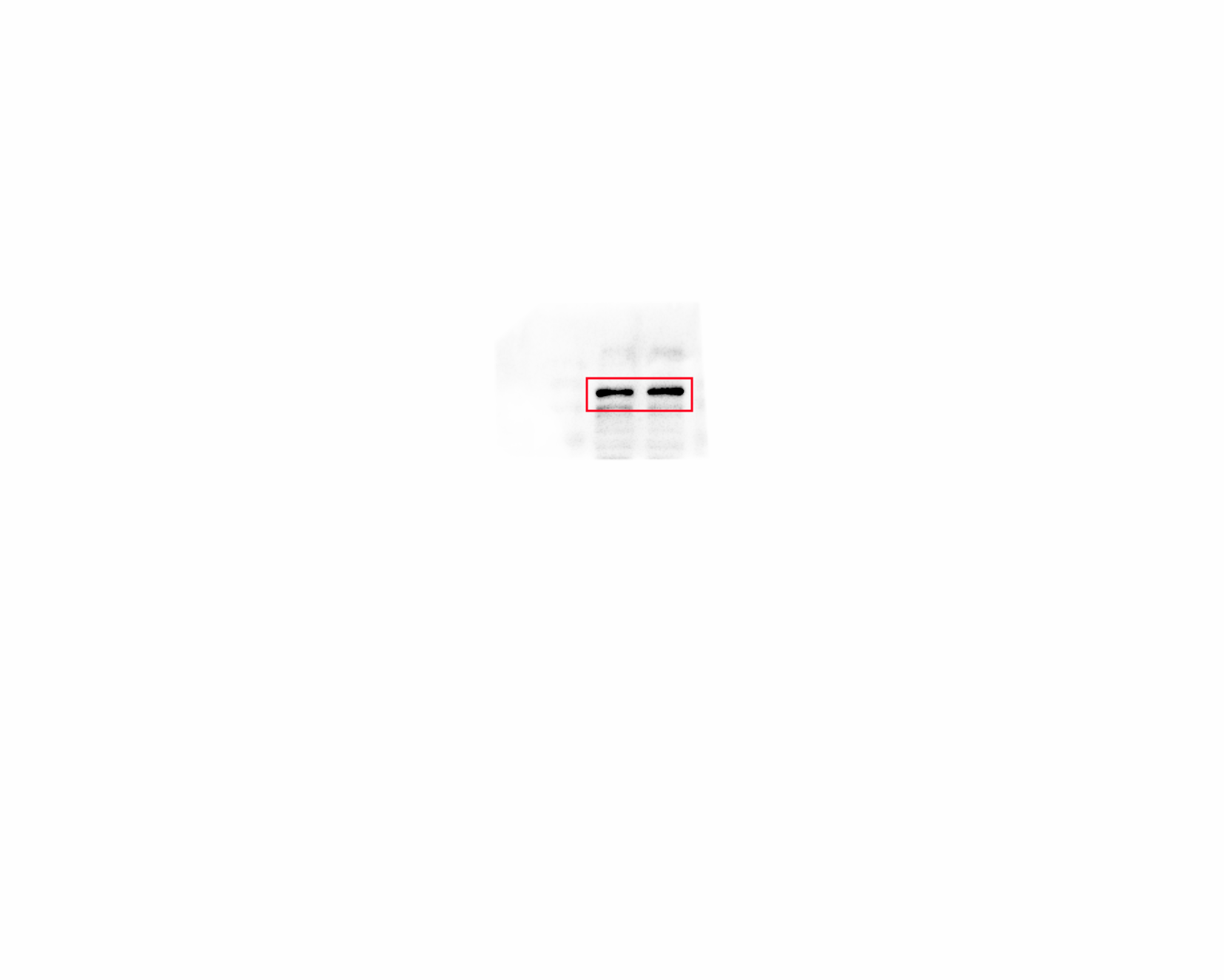

Supplement: Supplementary file 3 — Source data Fig. 2 [file 44318_2025_363_MOESM3_ESM.zip › Figure 2/2A/EphA1 input.tif]

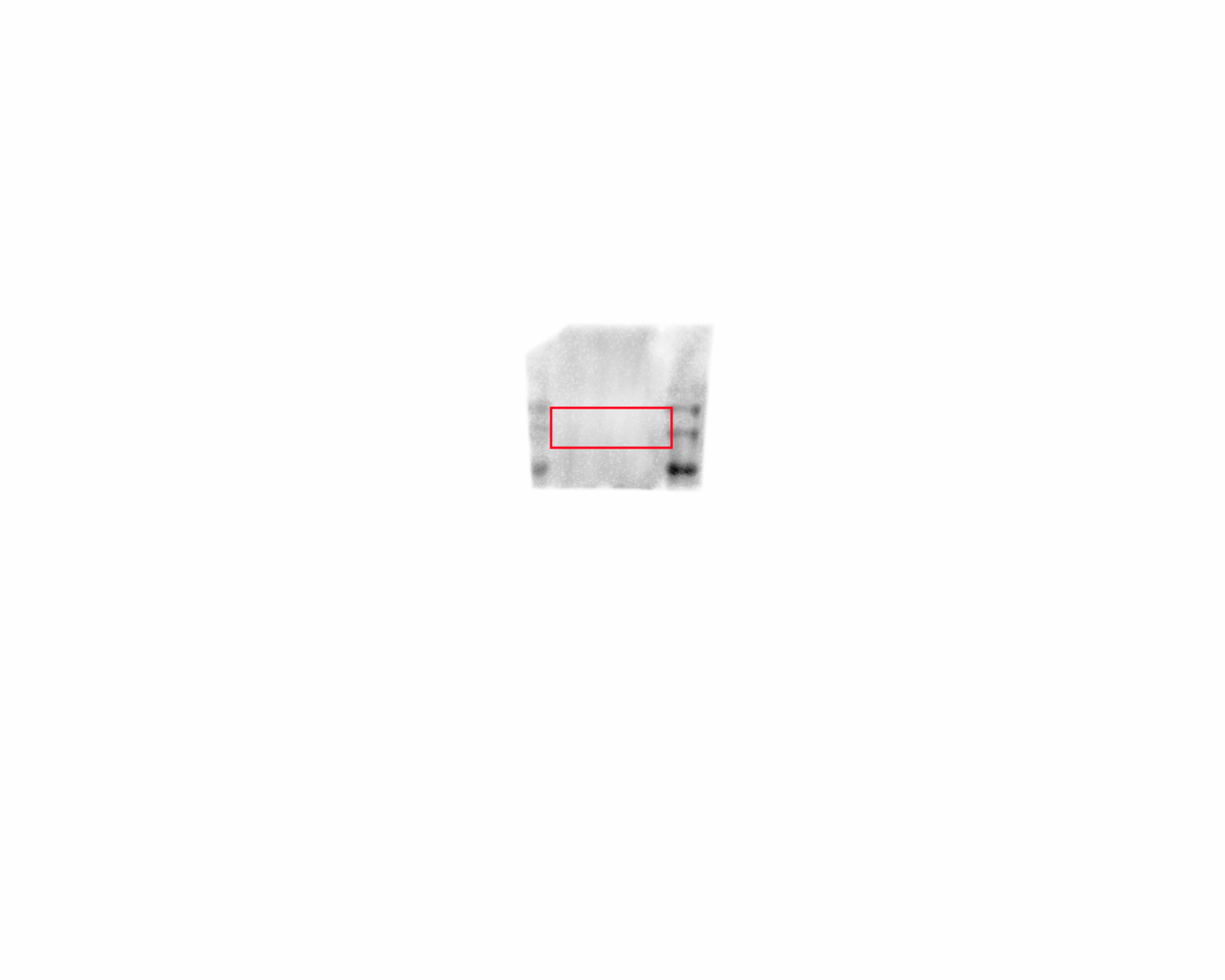

Supplement: Supplementary file 3 — Source data Fig. 2 [file 44318_2025_363_MOESM3_ESM.zip › Figure 2/2A/EphA1 ip.tif]

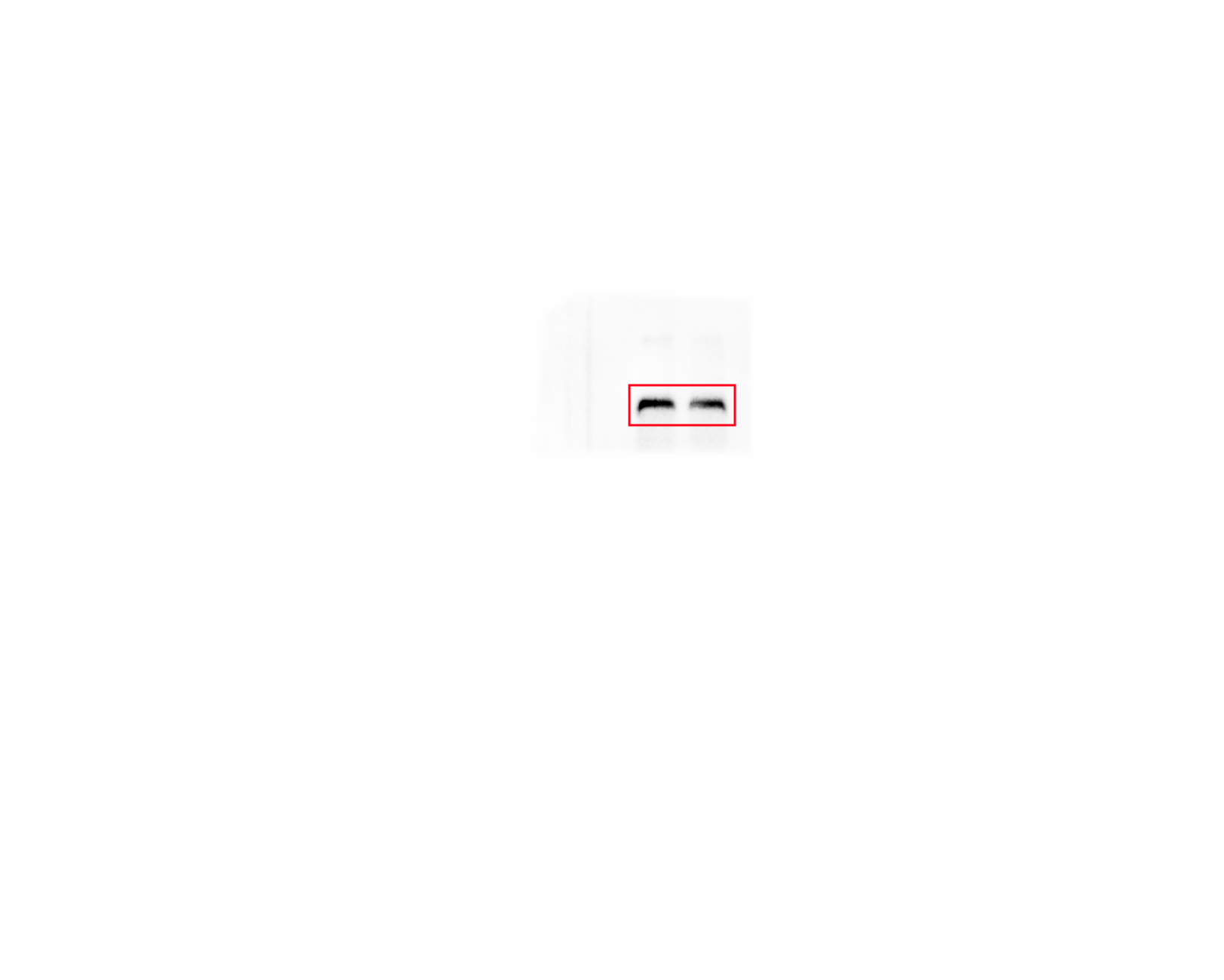

Supplement: Supplementary file 3 — Source data Fig. 2 [file 44318_2025_363_MOESM3_ESM.zip › Figure 2/2A/EphA2 input.tif]

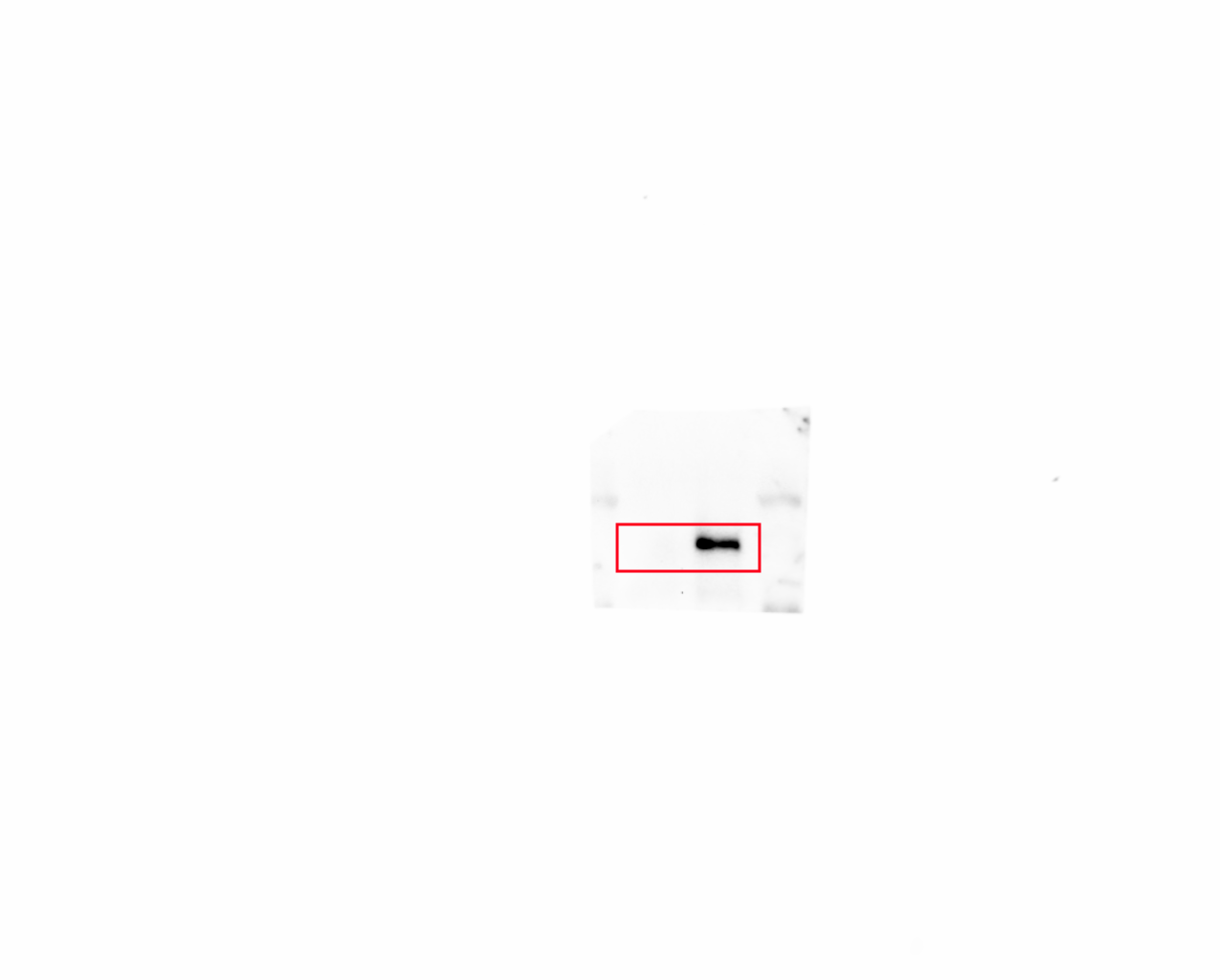

Supplement: Supplementary file 3 — Source data Fig. 2 [file 44318_2025_363_MOESM3_ESM.zip › Figure 2/2A/EphA2-IP.tif]

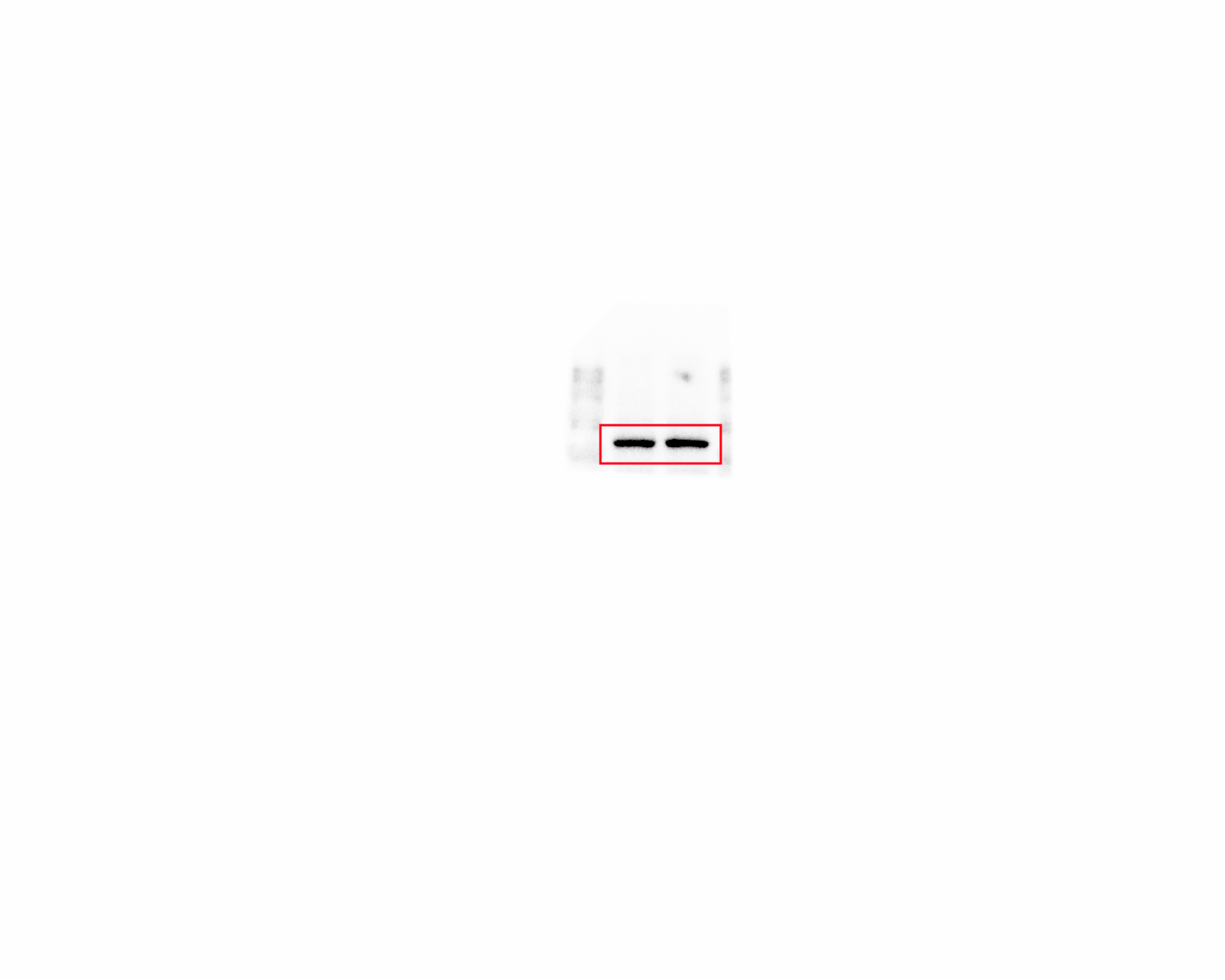

Supplement: Supplementary file 3 — Source data Fig. 2 [file 44318_2025_363_MOESM3_ESM.zip › Figure 2/2A/EphA4 input.tif]

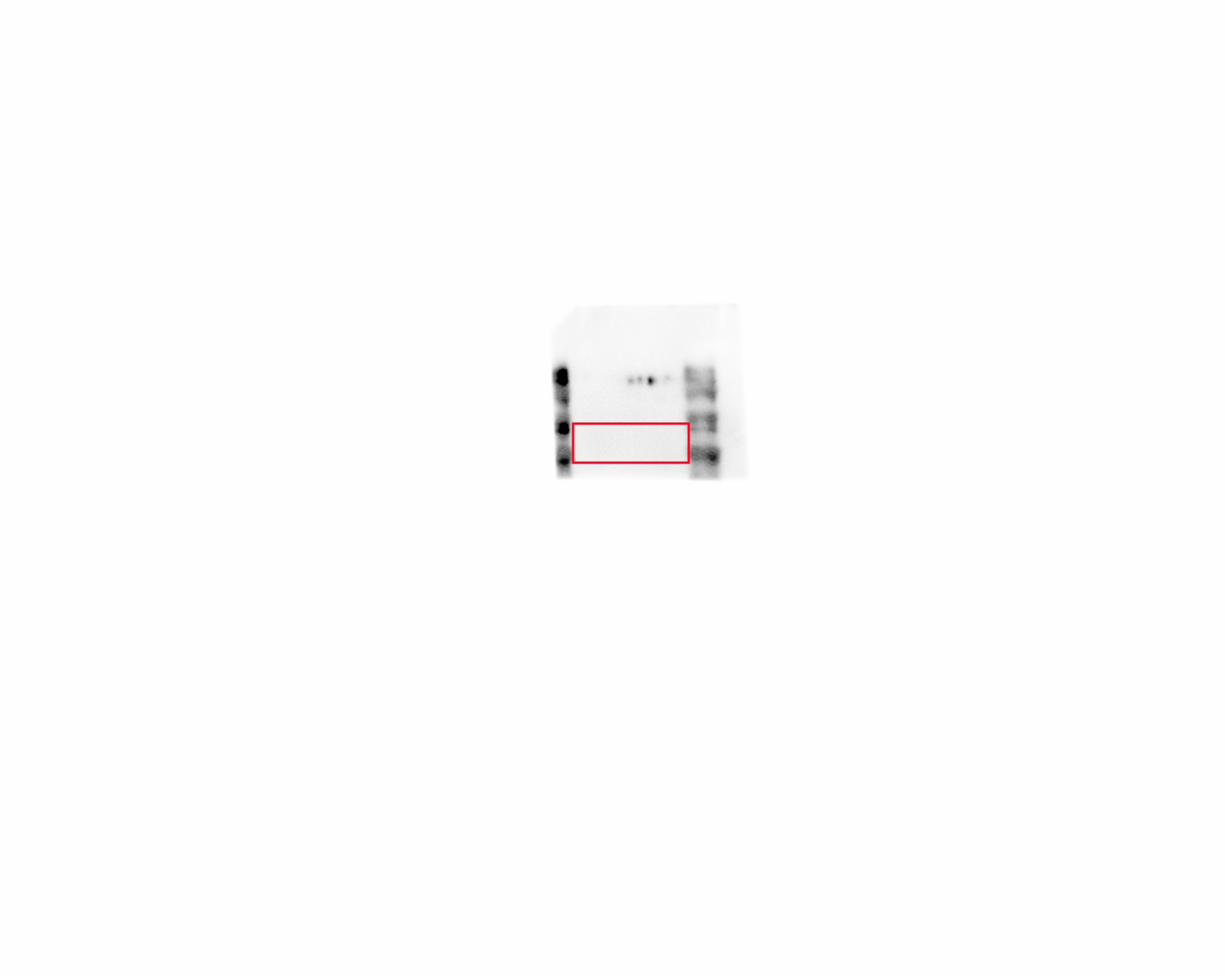

Supplement: Supplementary file 3 — Source data Fig. 2 [file 44318_2025_363_MOESM3_ESM.zip › Figure 2/2A/EphA4 ip.tif]

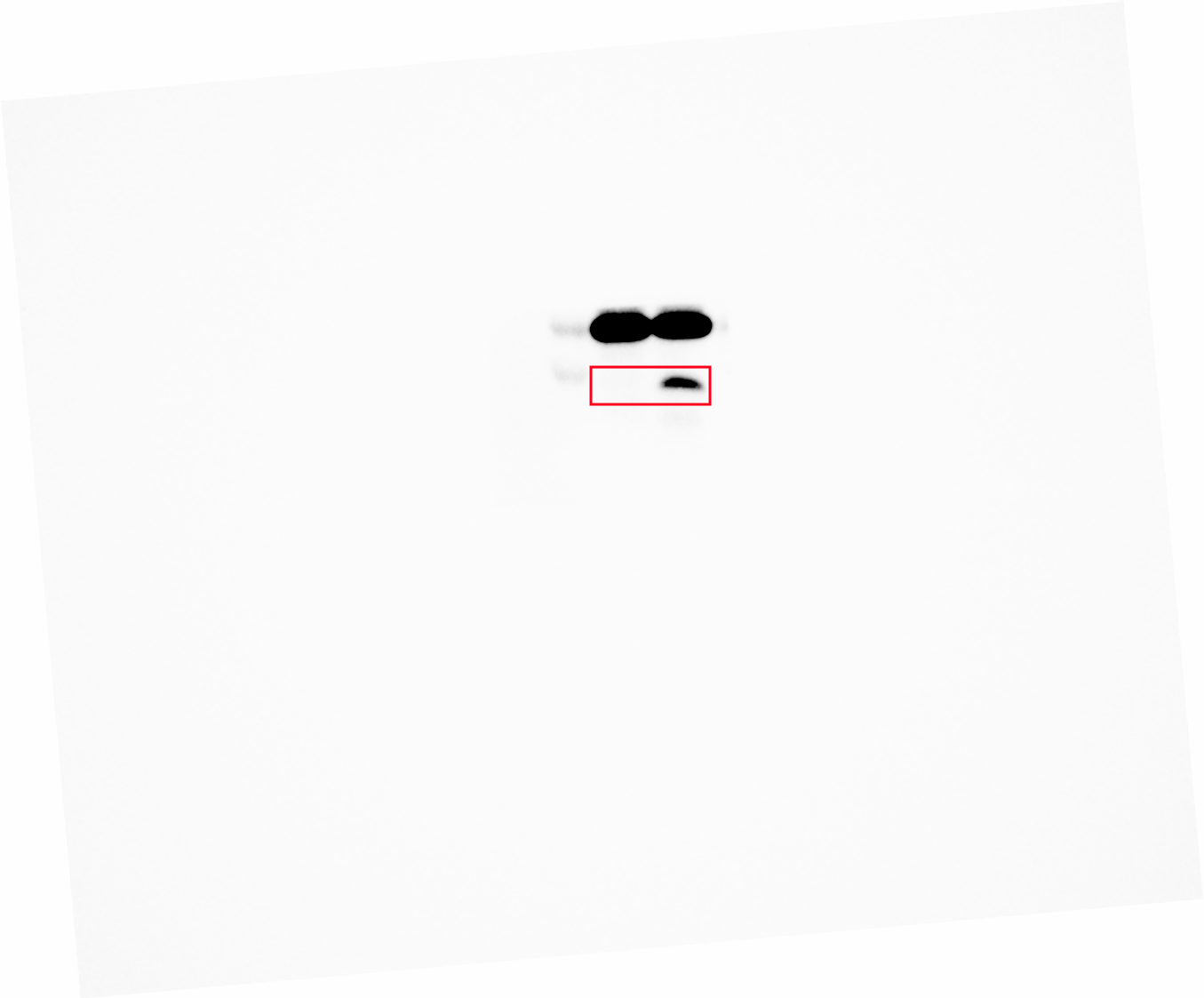

Supplement: Supplementary file 3 — Source data Fig. 2 [file 44318_2025_363_MOESM3_ESM.zip › Figure 2/2A/flag input.tif]

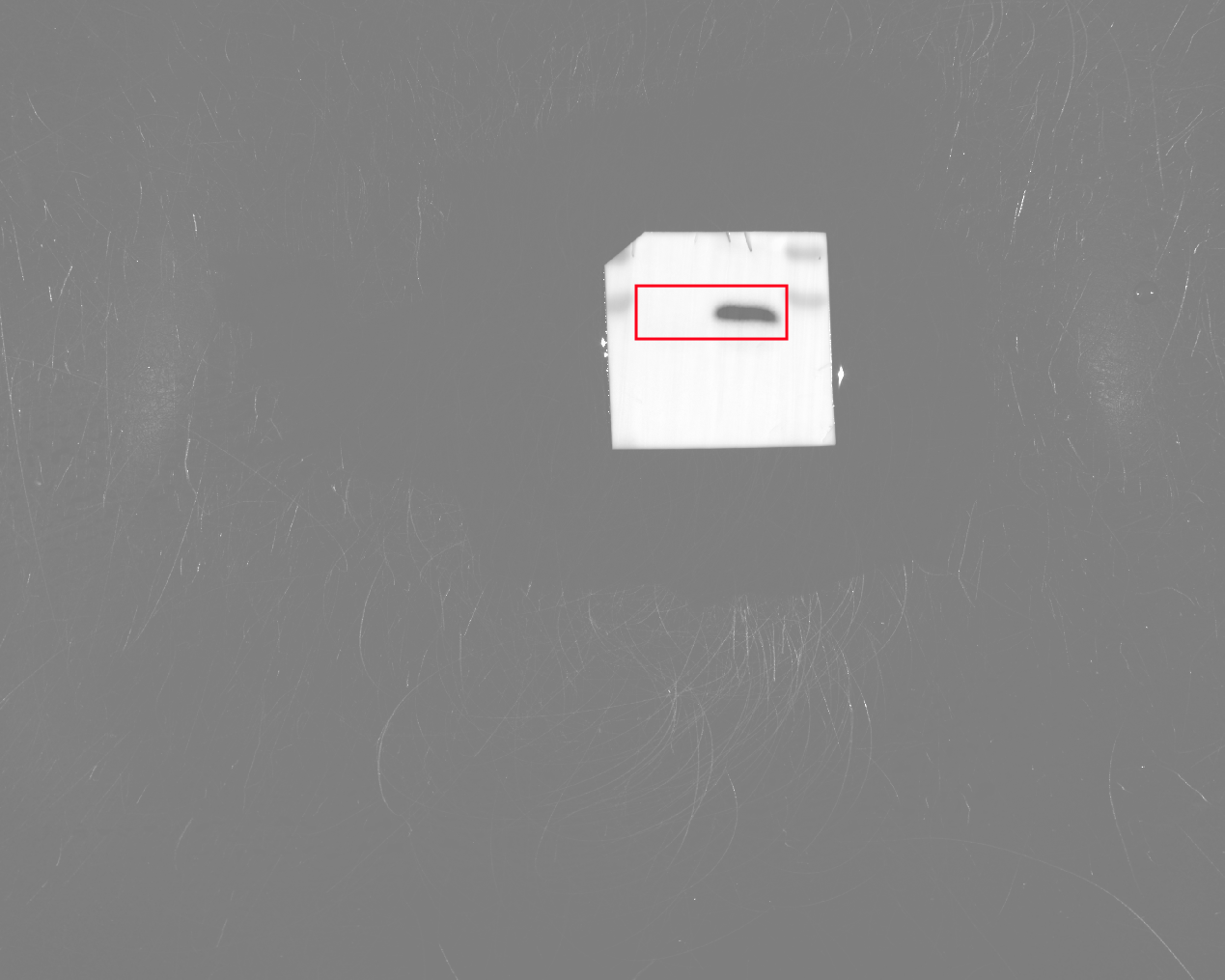

Supplement: Supplementary file 3 — Source data Fig. 2 [file 44318_2025_363_MOESM3_ESM.zip › Figure 2/2A/flag ip.tif]

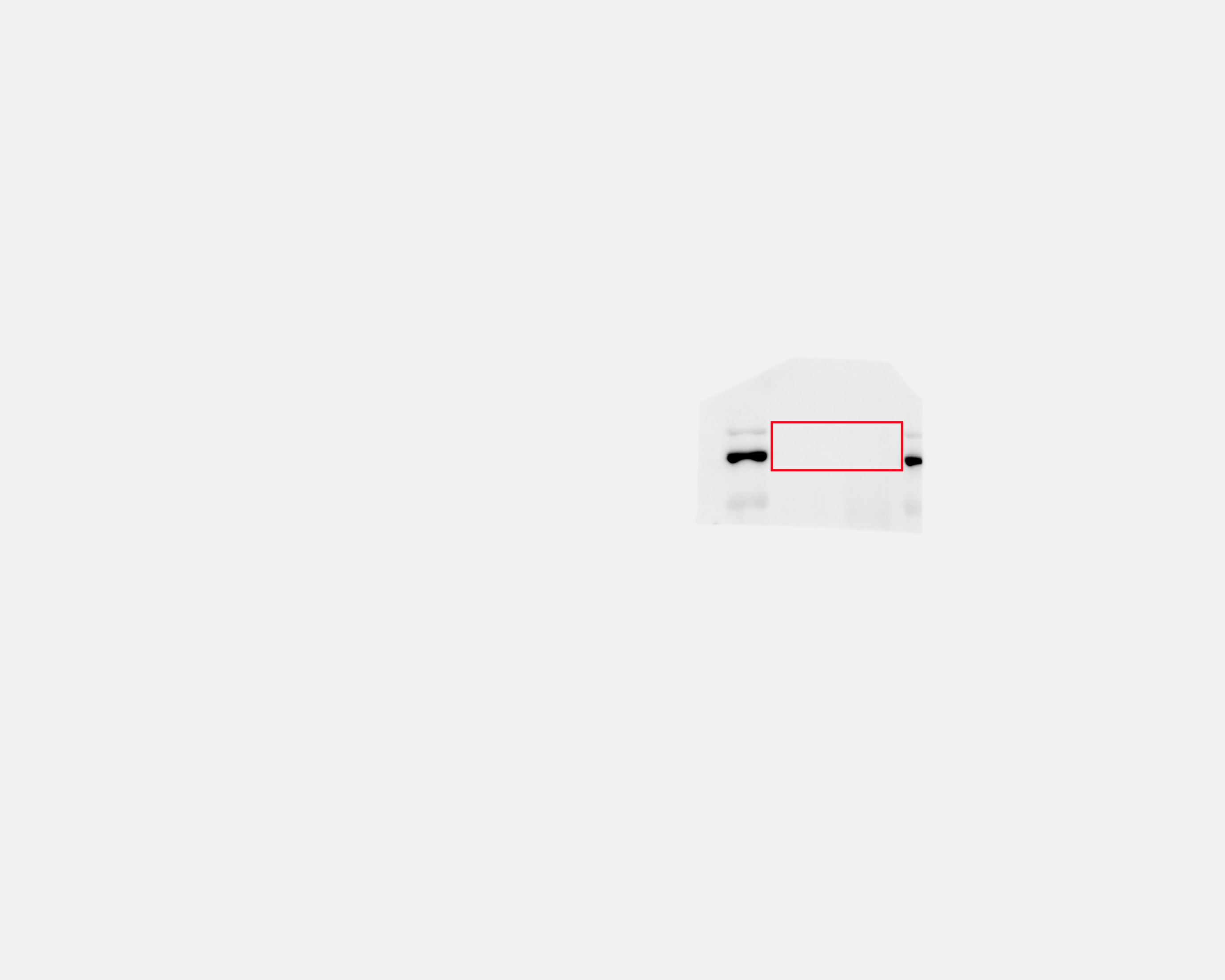

Supplement: Supplementary file 3 — Source data Fig. 2 [file 44318_2025_363_MOESM3_ESM.zip › Figure 2/2B/1 EphA1 IP.tif]

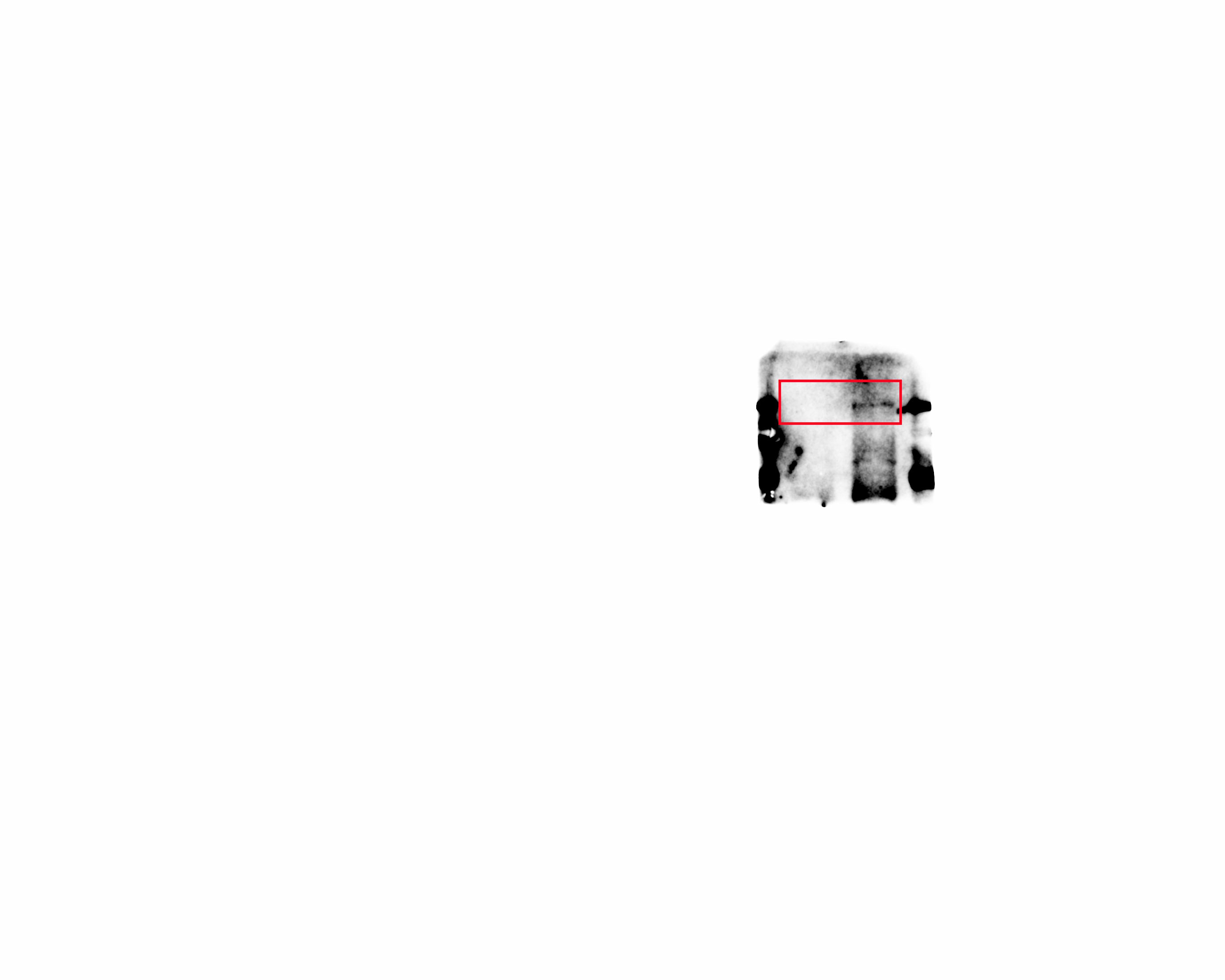

Supplement: Supplementary file 3 — Source data Fig. 2 [file 44318_2025_363_MOESM3_ESM.zip › Figure 2/2B/2 EphA2 IP.tif]

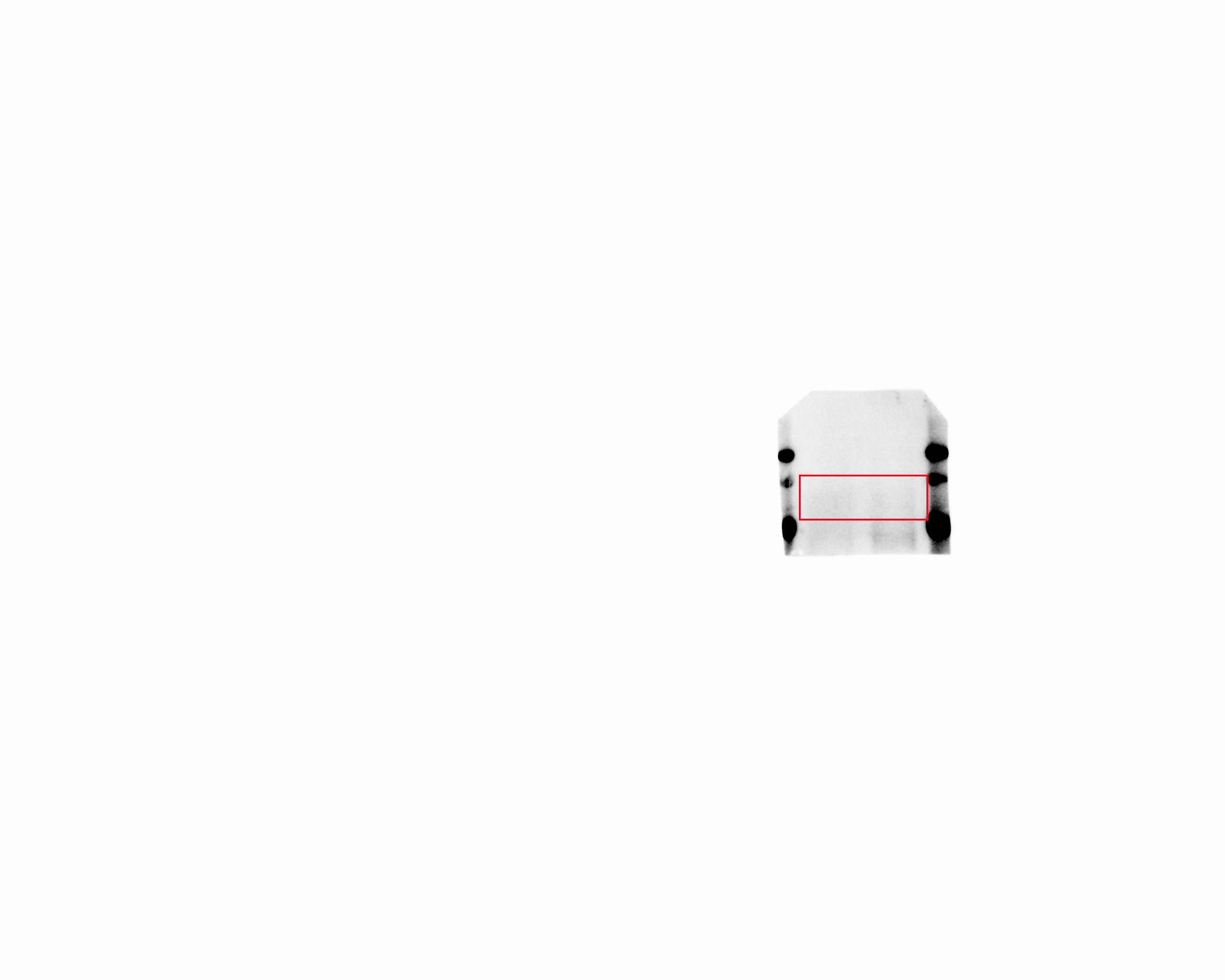

Supplement: Supplementary file 3 — Source data Fig. 2 [file 44318_2025_363_MOESM3_ESM.zip › Figure 2/2B/3 EphA4 IP.tif]

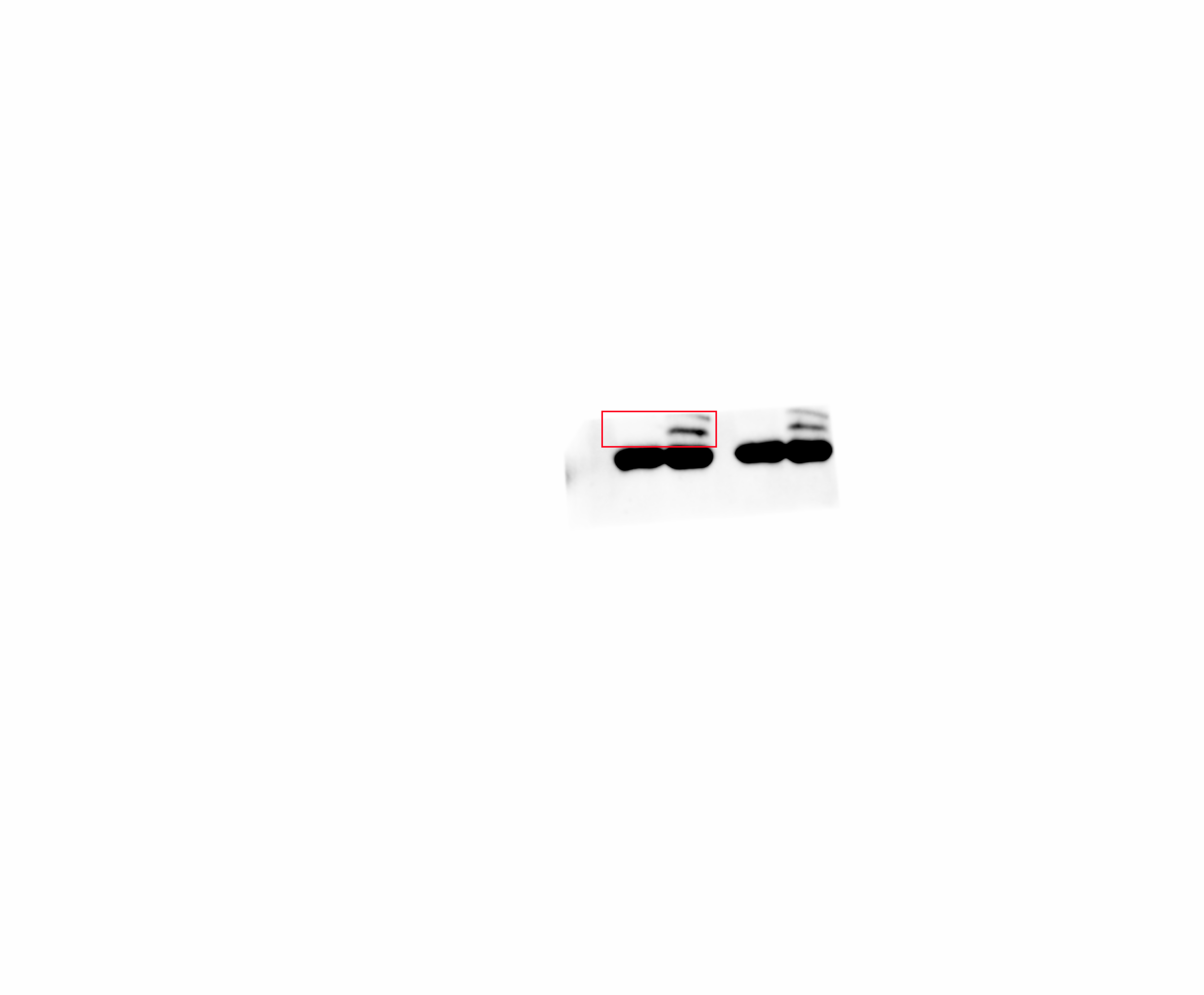

Supplement: Supplementary file 3 — Source data Fig. 2 [file 44318_2025_363_MOESM3_ESM.zip › Figure 2/2B/4 flag IP.tif]

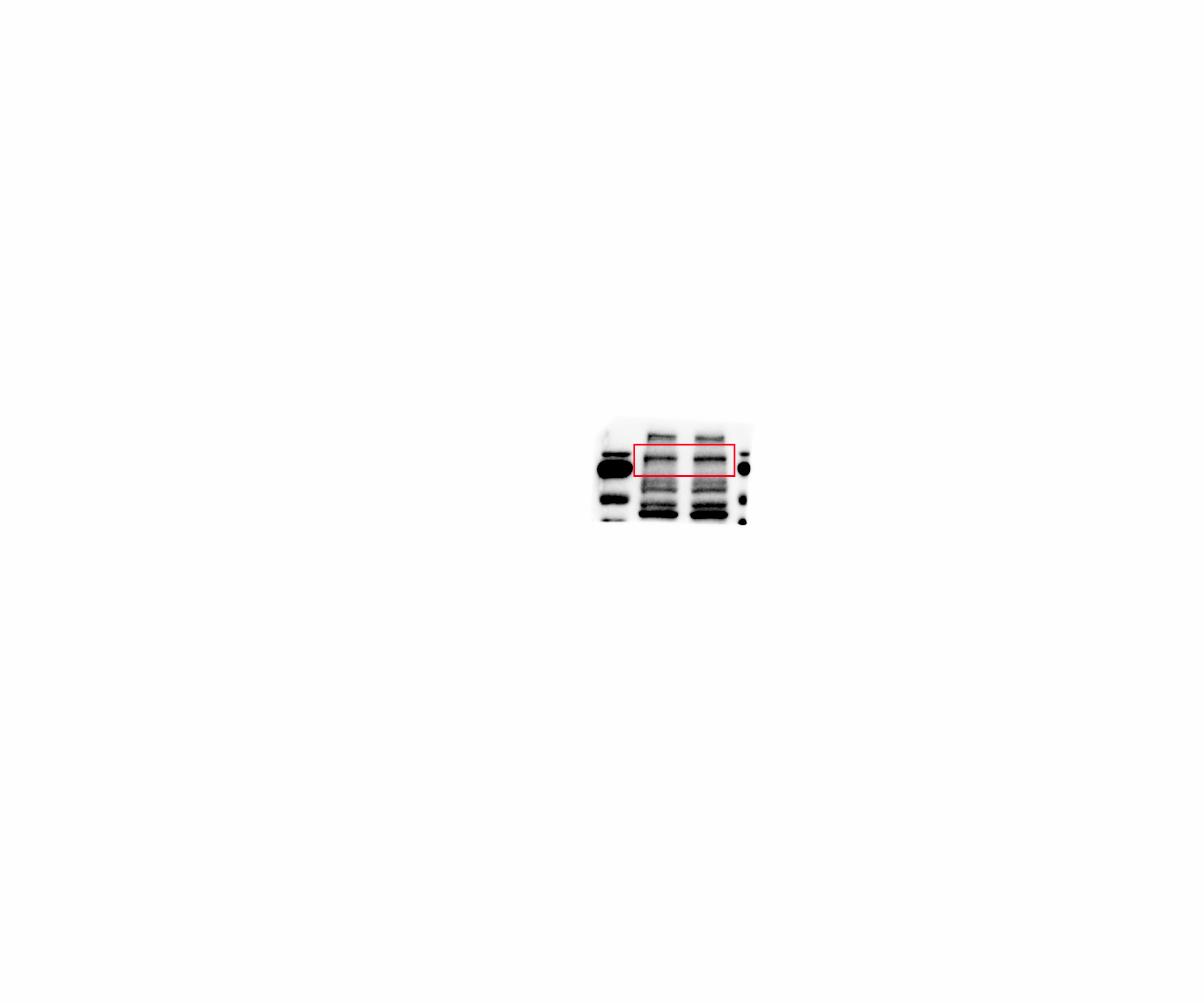

Supplement: Supplementary file 3 — Source data Fig. 2 [file 44318_2025_363_MOESM3_ESM.zip › Figure 2/2B/5 EphA1 input.tif]

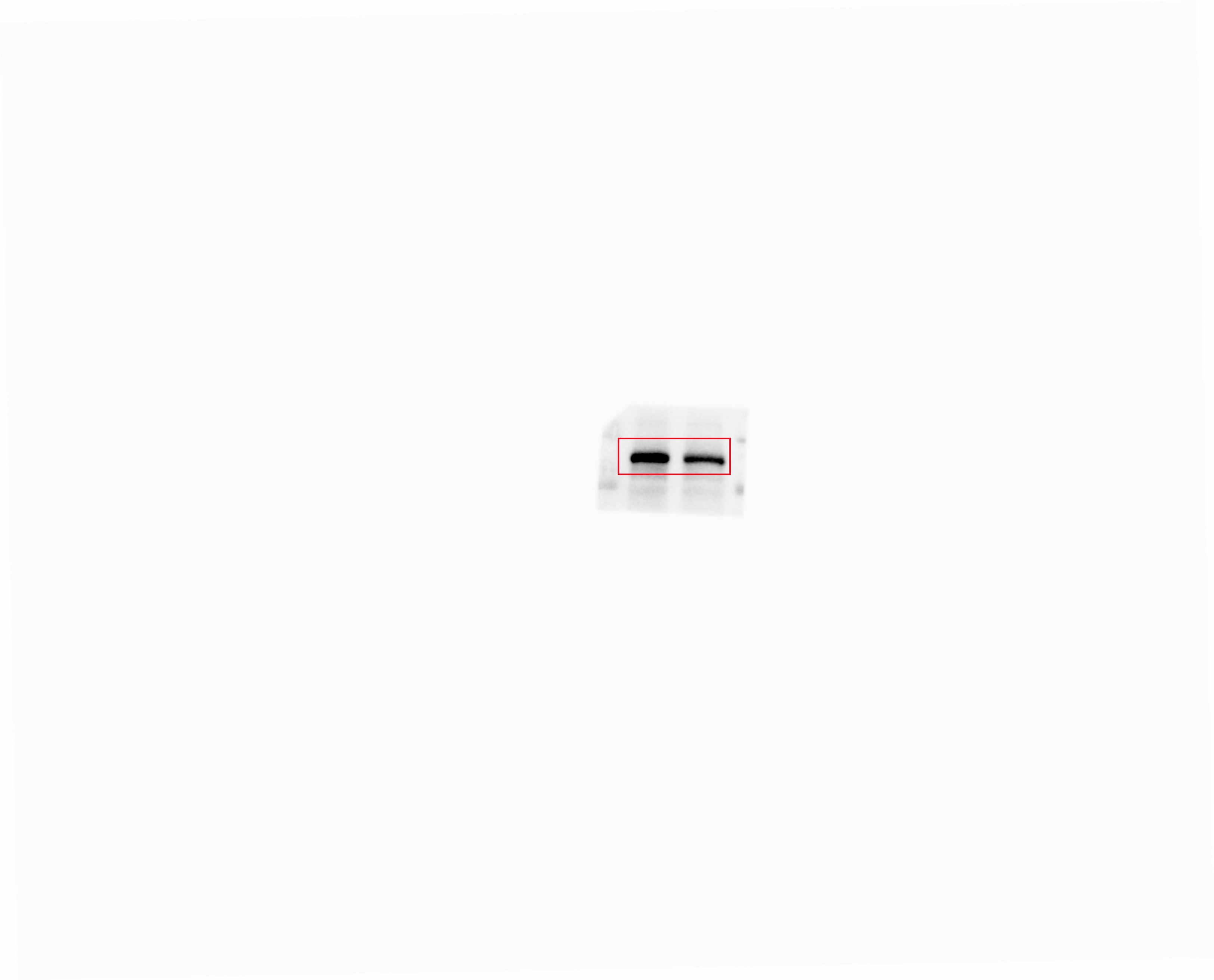

Supplement: Supplementary file 3 — Source data Fig. 2 [file 44318_2025_363_MOESM3_ESM.zip › Figure 2/2B/6 EphA2 input.tif]
