# Supplementary figures and images for: Ephrin A1 functions as a ligand of EGFR to promote EMT and metastasis in gastric cancer (part 2 of 5)
Source: EMBO J. 2025 Jan 21;44(5):1464–87. doi: 10.1038/s44318-025-00363-x (PMC11876641; doi:10.1038/s44318-025-00363-x)

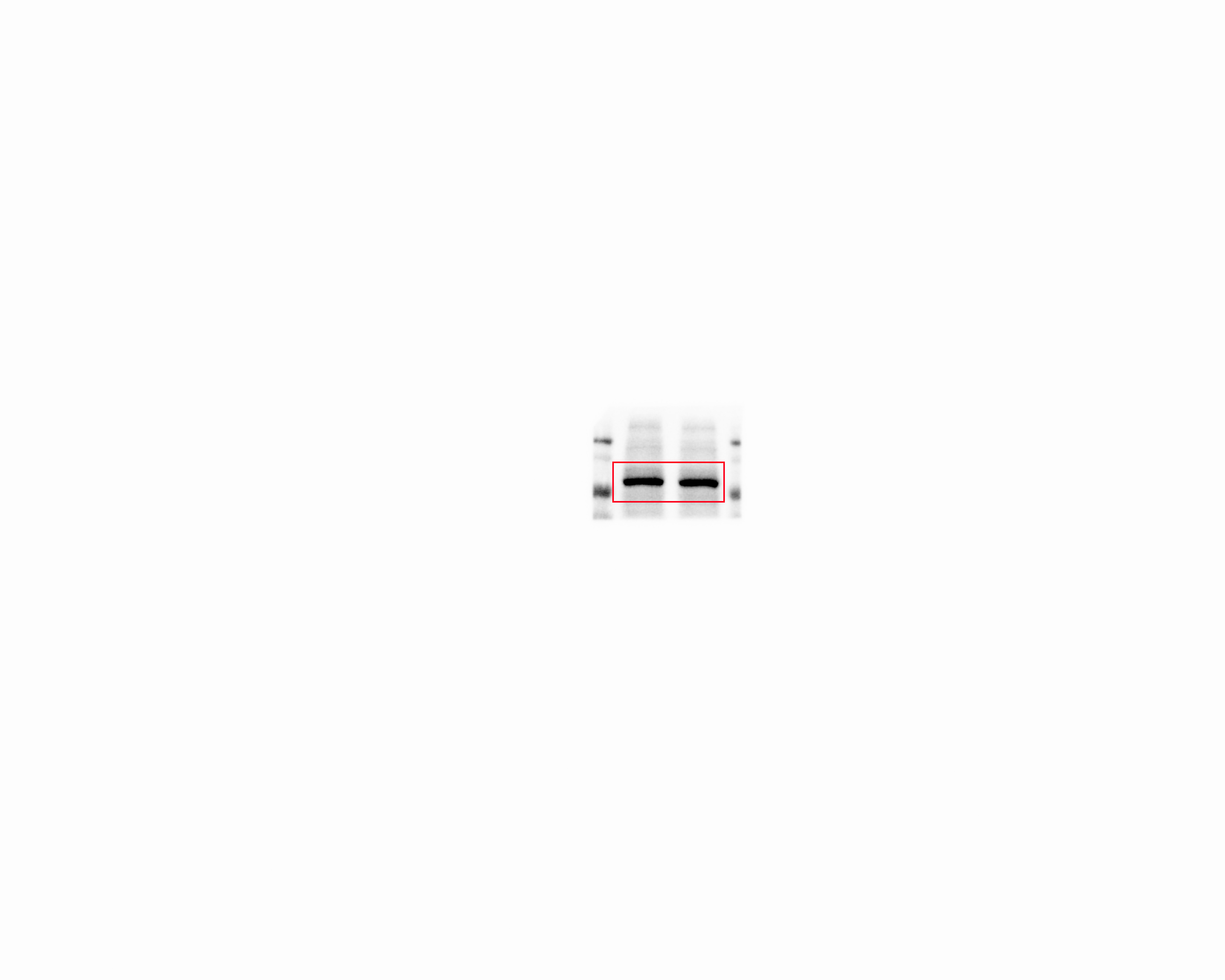

Supplement: Supplementary file 3 — Source data Fig. 2 [file 44318_2025_363_MOESM3_ESM.zip › Figure 2/2B/7 EphA4 input.tif]

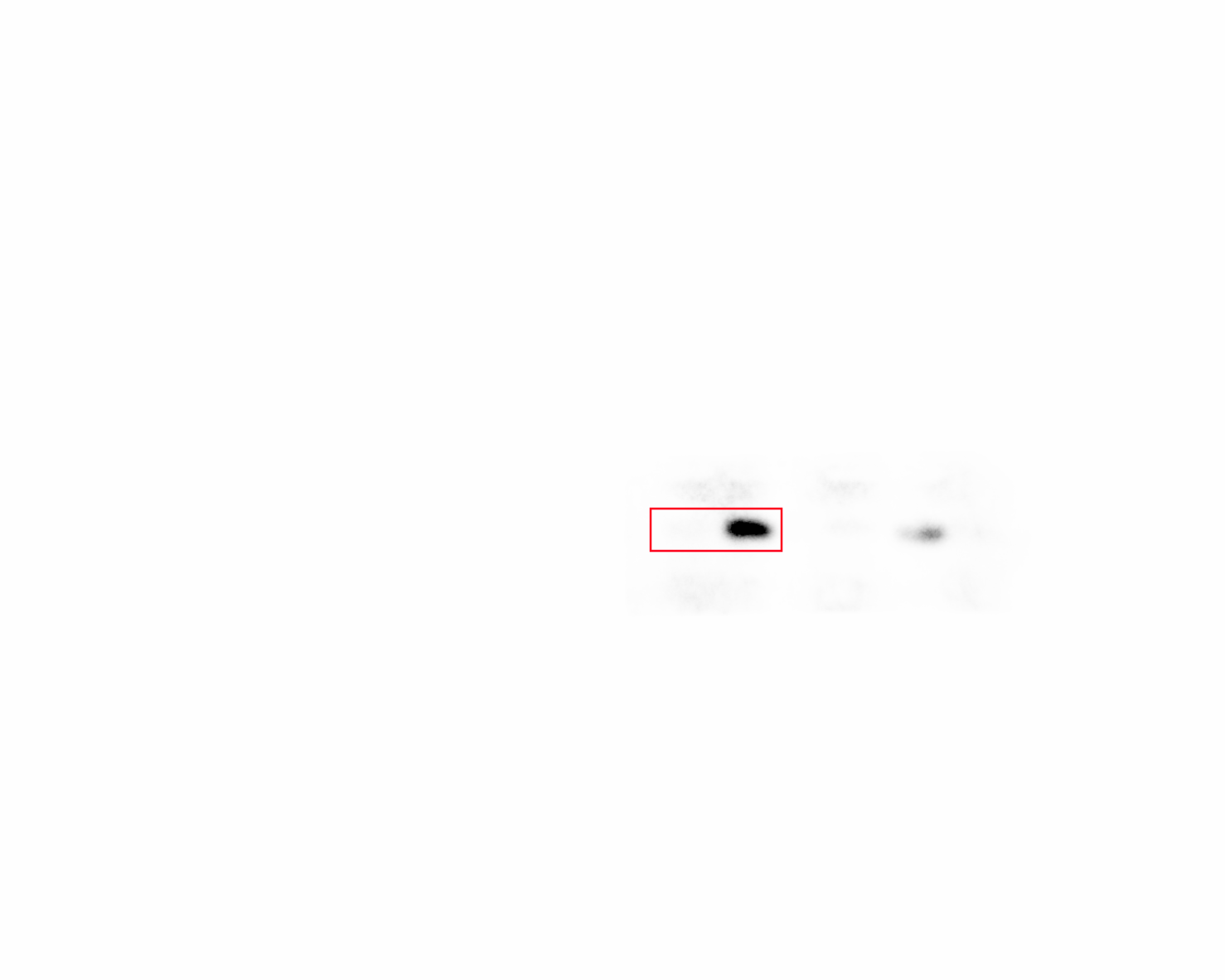

Supplement: Supplementary file 3 — Source data Fig. 2 [file 44318_2025_363_MOESM3_ESM.zip › Figure 2/2B/8 flag input.tif]

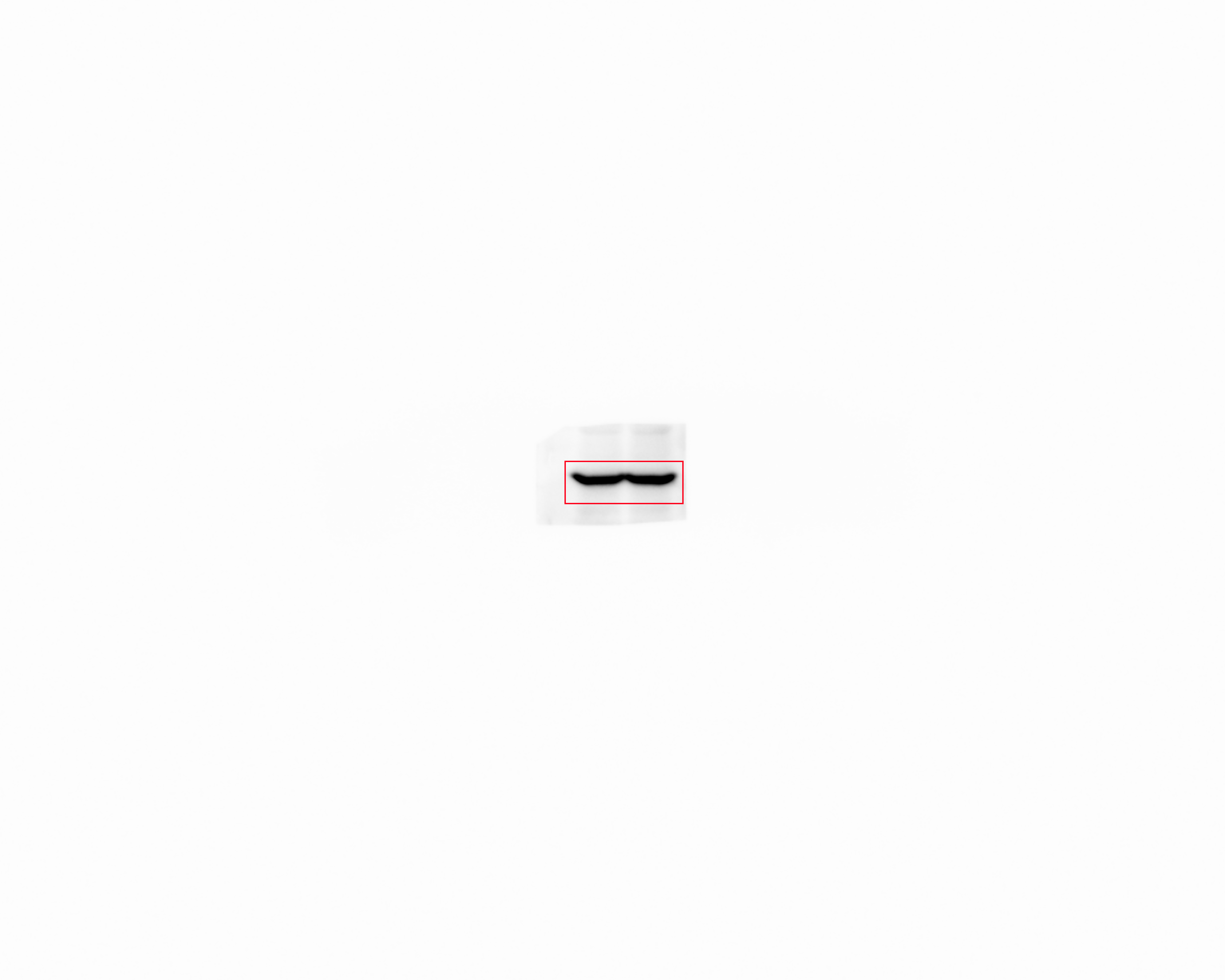

Supplement: Supplementary file 3 — Source data Fig. 2 [file 44318_2025_363_MOESM3_ESM.zip › Figure 2/2B/9 actin.tif]

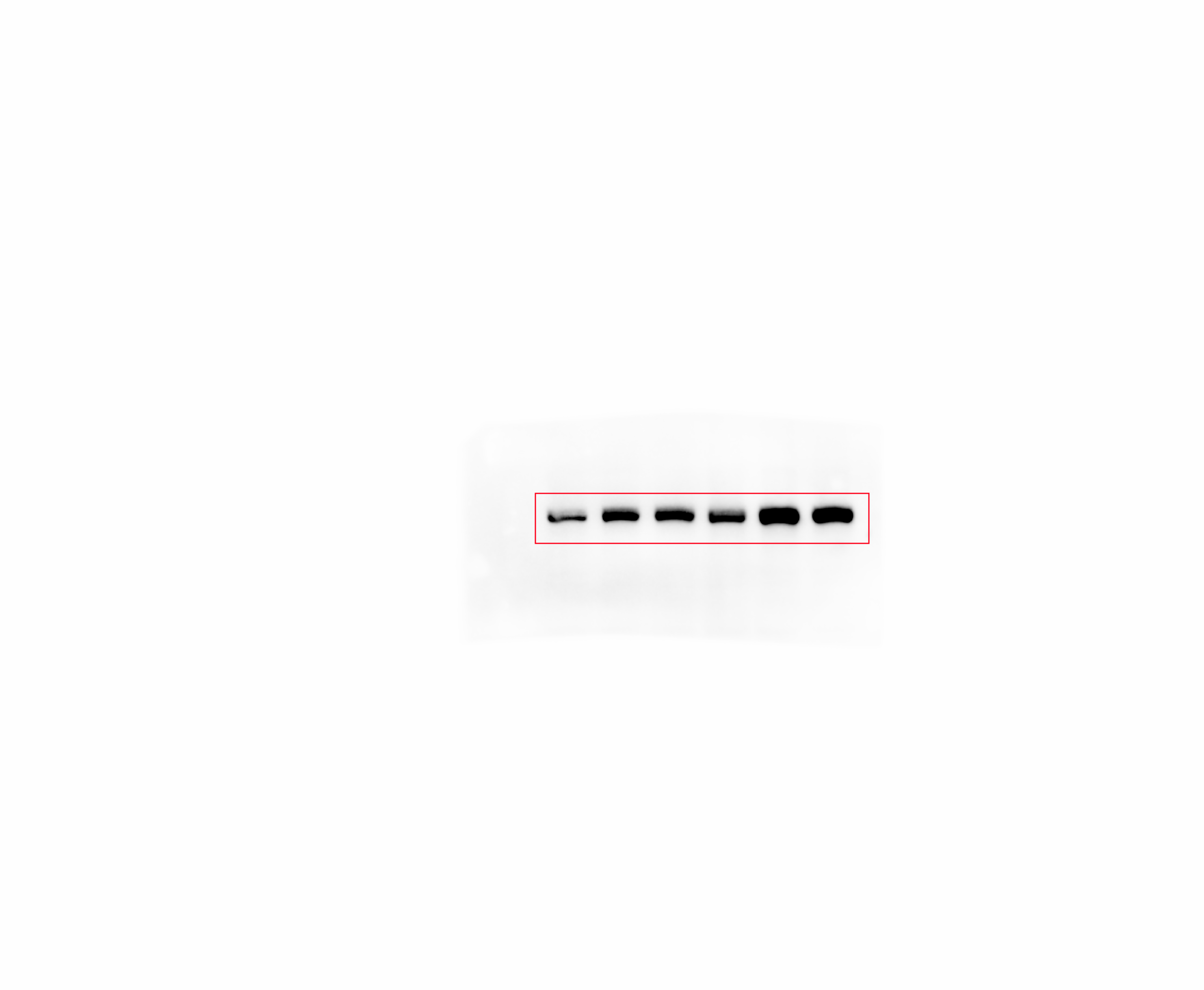

Supplement: Supplementary file 3 — Source data Fig. 2 [file 44318_2025_363_MOESM3_ESM.zip › Figure 2/2D/1 E-cad.tif]

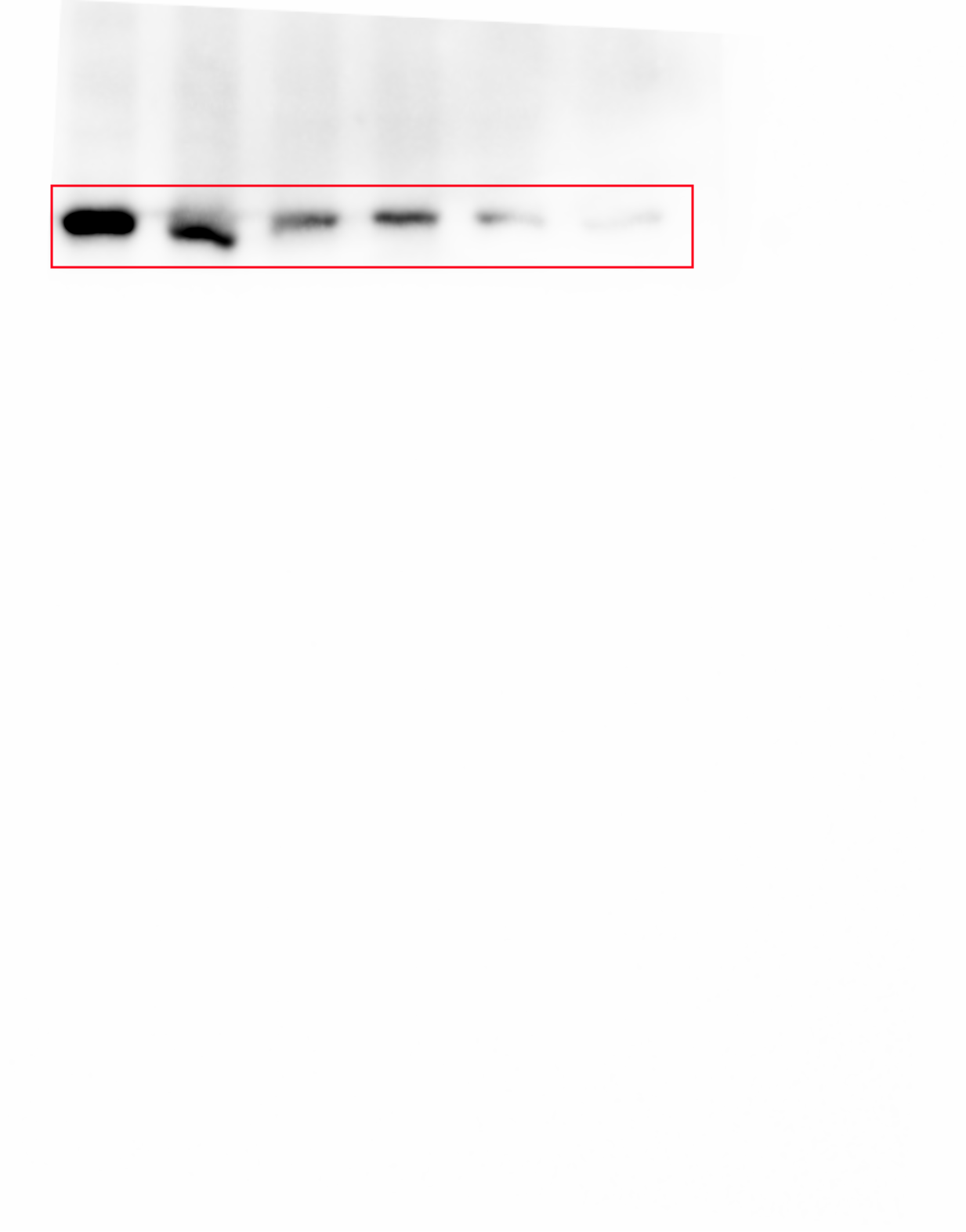

Supplement: Supplementary file 3 — Source data Fig. 2 [file 44318_2025_363_MOESM3_ESM.zip › Figure 2/2D/2 N-cad.tif]

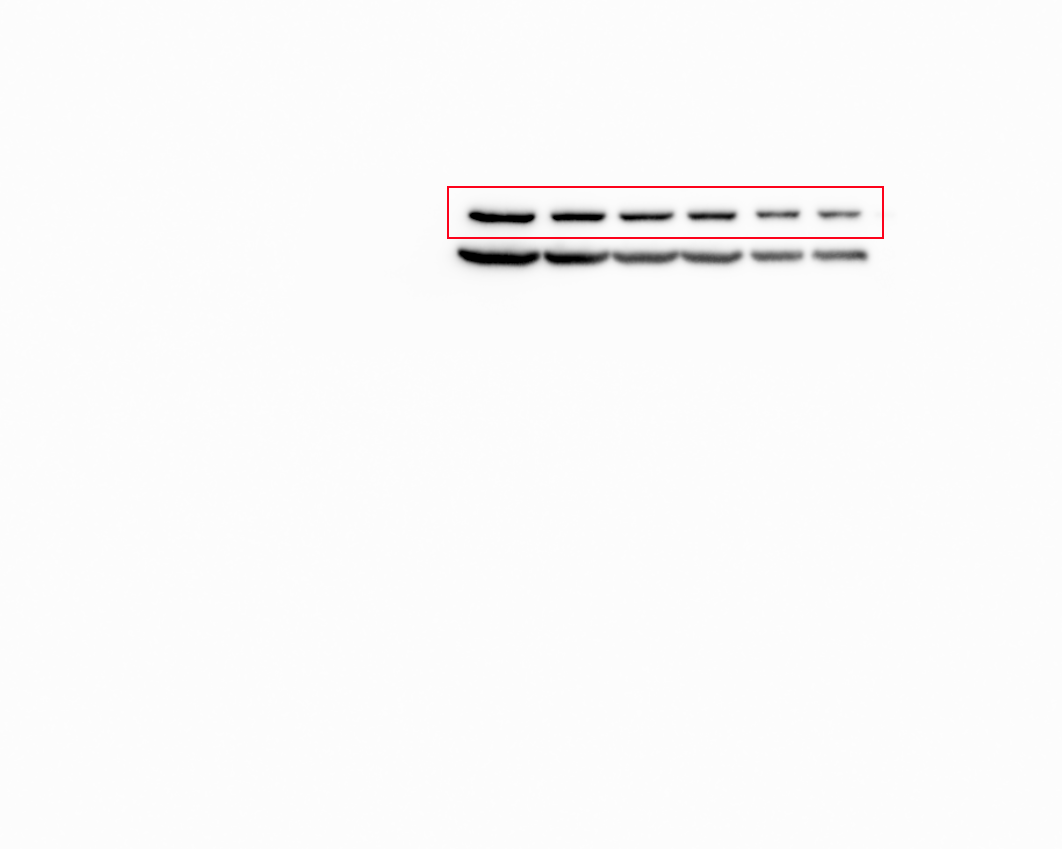

Supplement: Supplementary file 3 — Source data Fig. 2 [file 44318_2025_363_MOESM3_ESM.zip › Figure 2/2D/3 vimentin.tif]

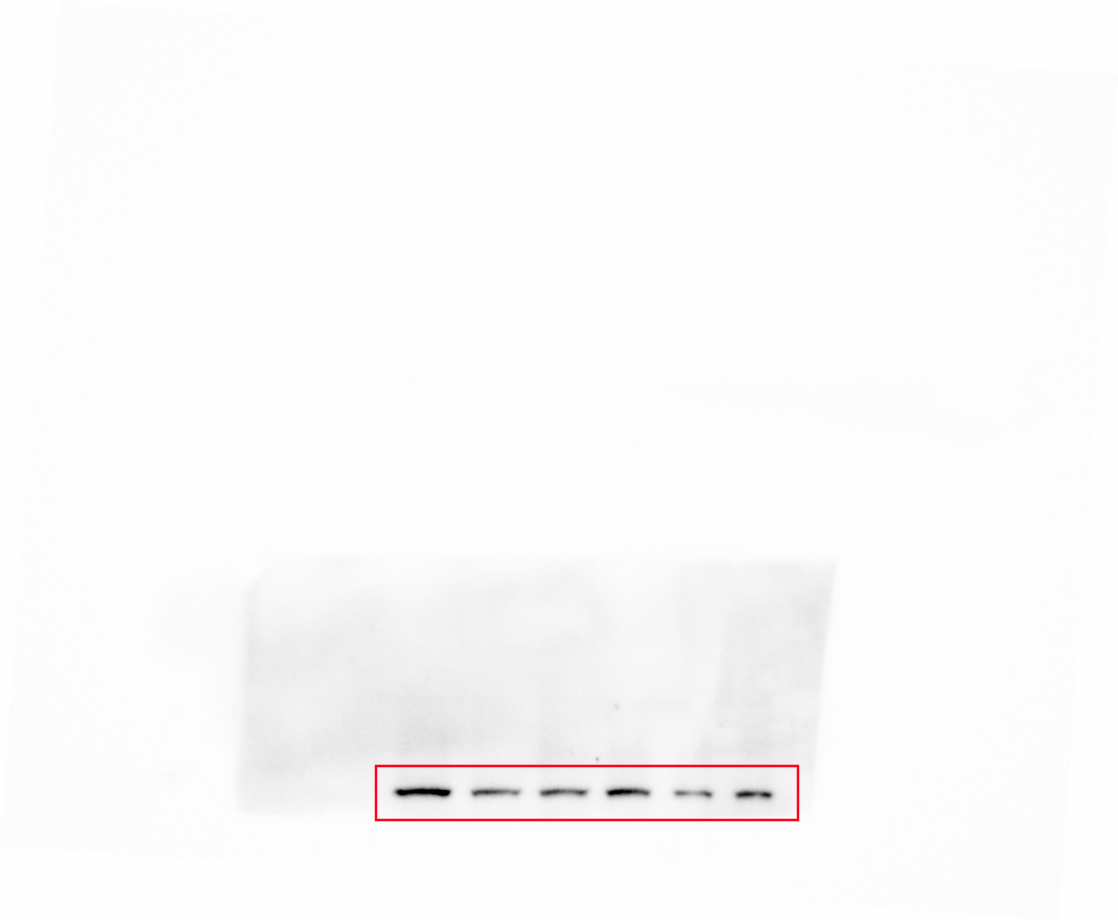

Supplement: Supplementary file 3 — Source data Fig. 2 [file 44318_2025_363_MOESM3_ESM.zip › Figure 2/2D/4 ZEB1.tif]

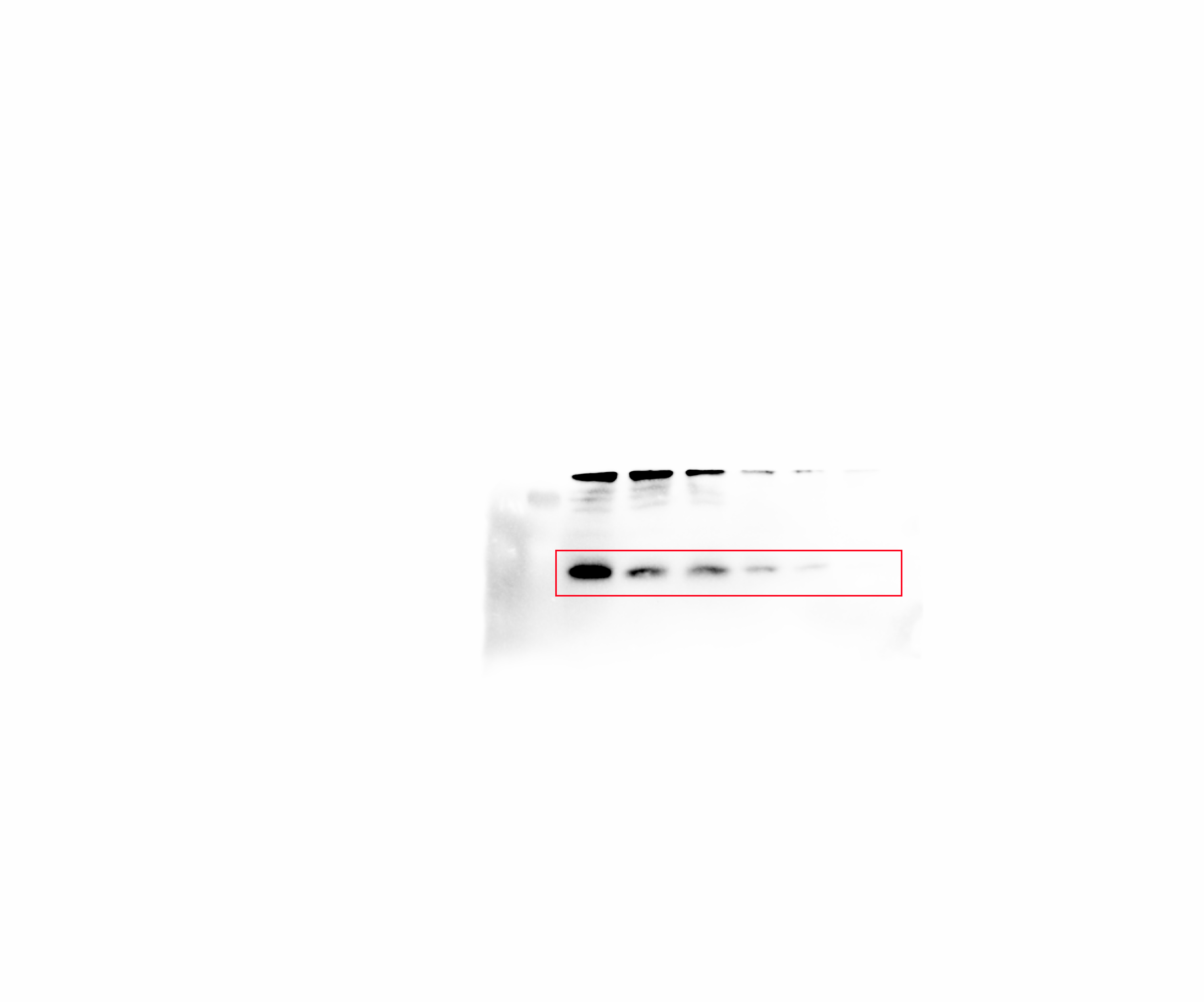

Supplement: Supplementary file 3 — Source data Fig. 2 [file 44318_2025_363_MOESM3_ESM.zip › Figure 2/2D/5 Ephrin A1.tif]

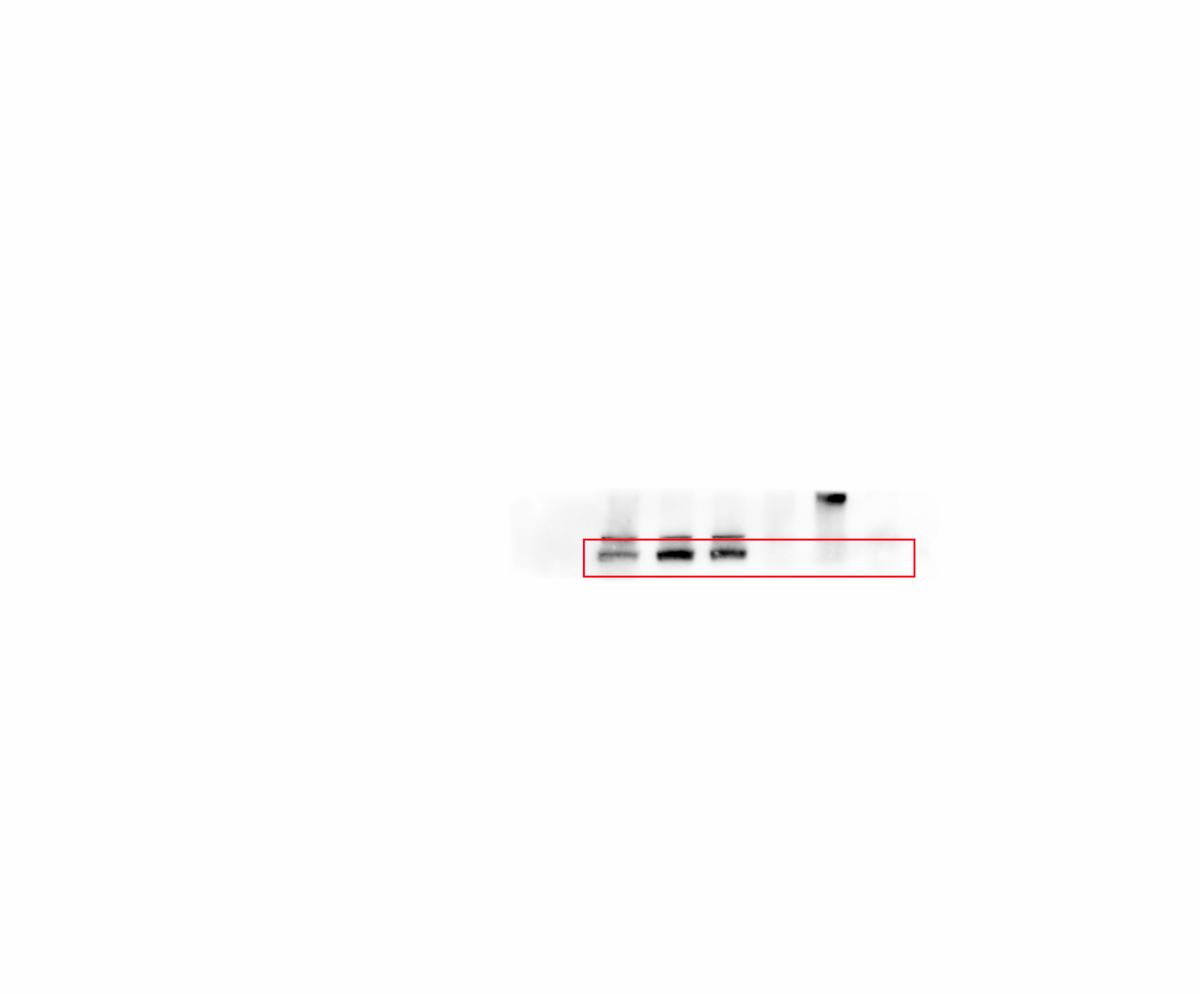

Supplement: Supplementary file 3 — Source data Fig. 2 [file 44318_2025_363_MOESM3_ESM.zip › Figure 2/2D/6 EphA2.tif]

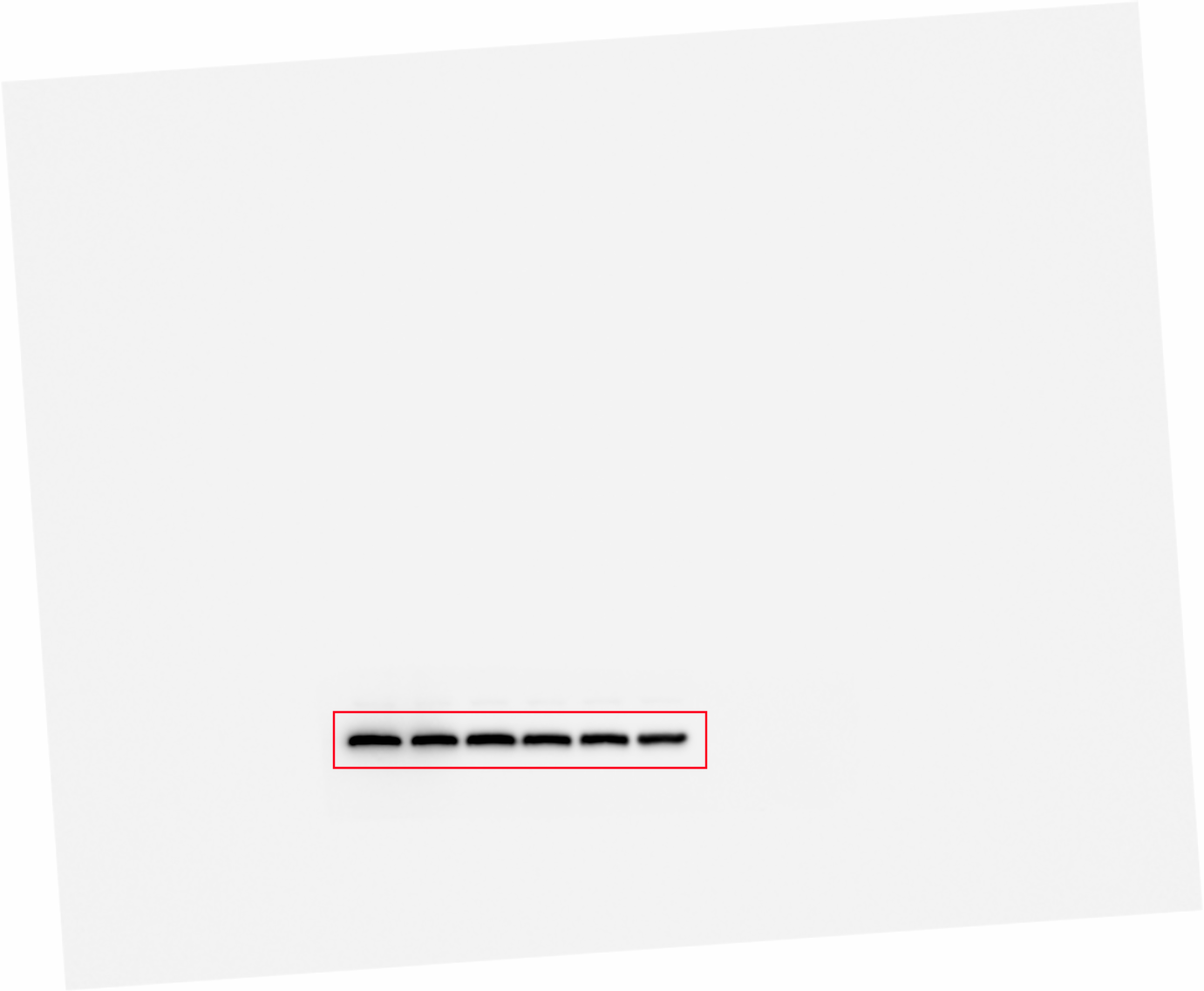

Supplement: Supplementary file 3 — Source data Fig. 2 [file 44318_2025_363_MOESM3_ESM.zip › Figure 2/2D/7 actin.tif]

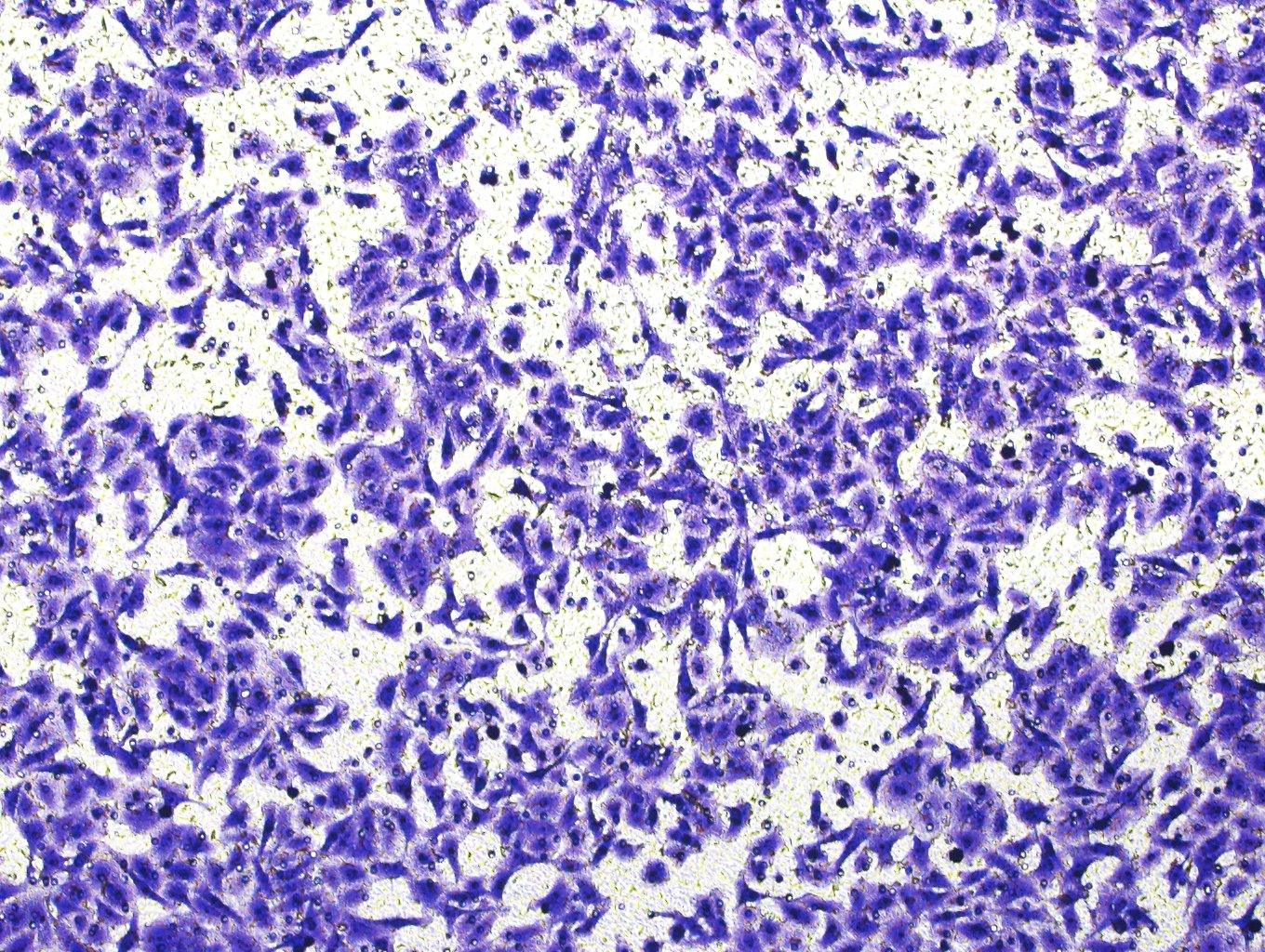

Supplement: Supplementary file 3 — Source data Fig. 2 [file 44318_2025_363_MOESM3_ESM.zip › Figure 2/2E/Control (1).jpg]

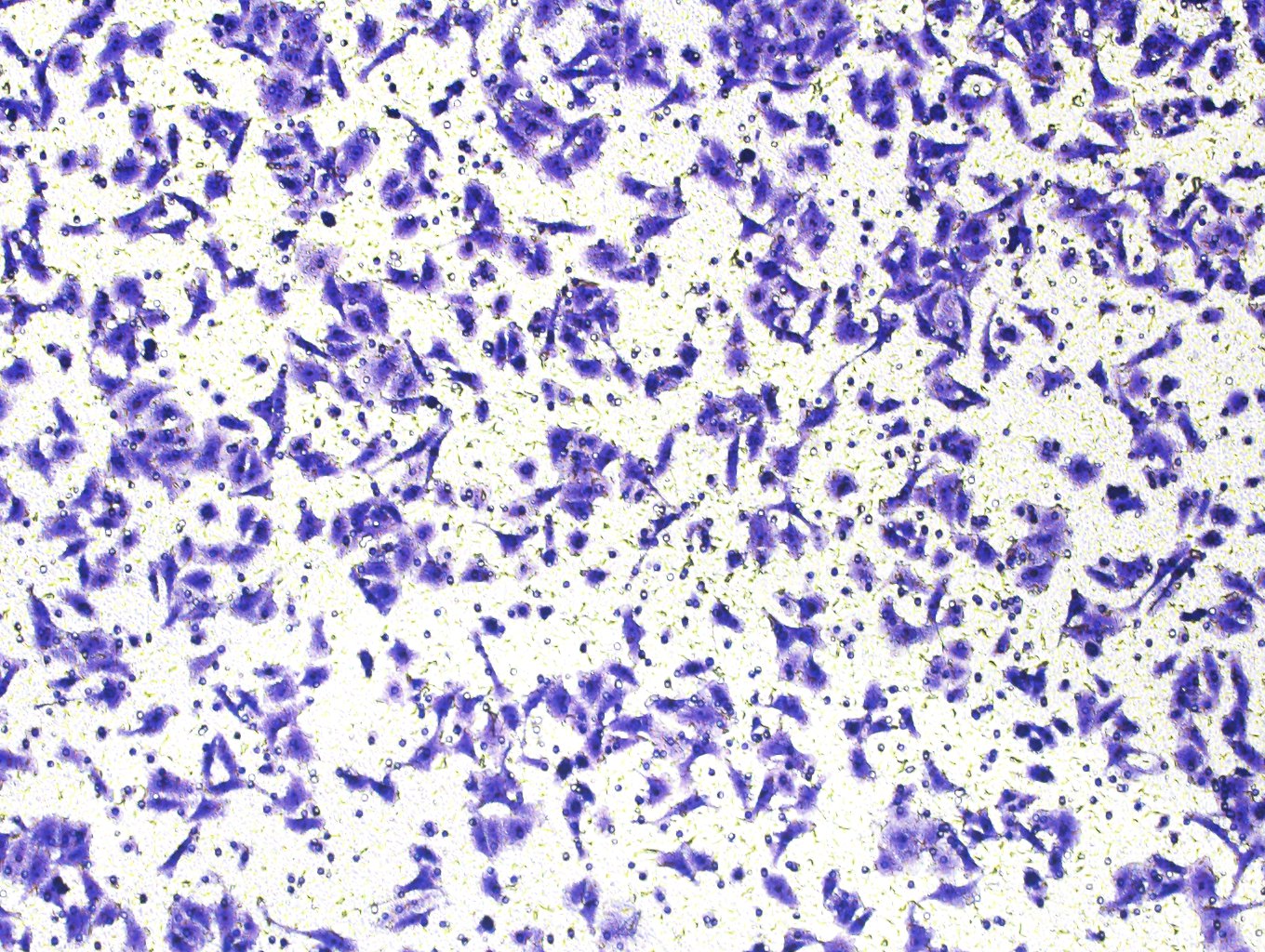

Supplement: Supplementary file 3 — Source data Fig. 2 [file 44318_2025_363_MOESM3_ESM.zip › Figure 2/2E/Control (2).jpg]

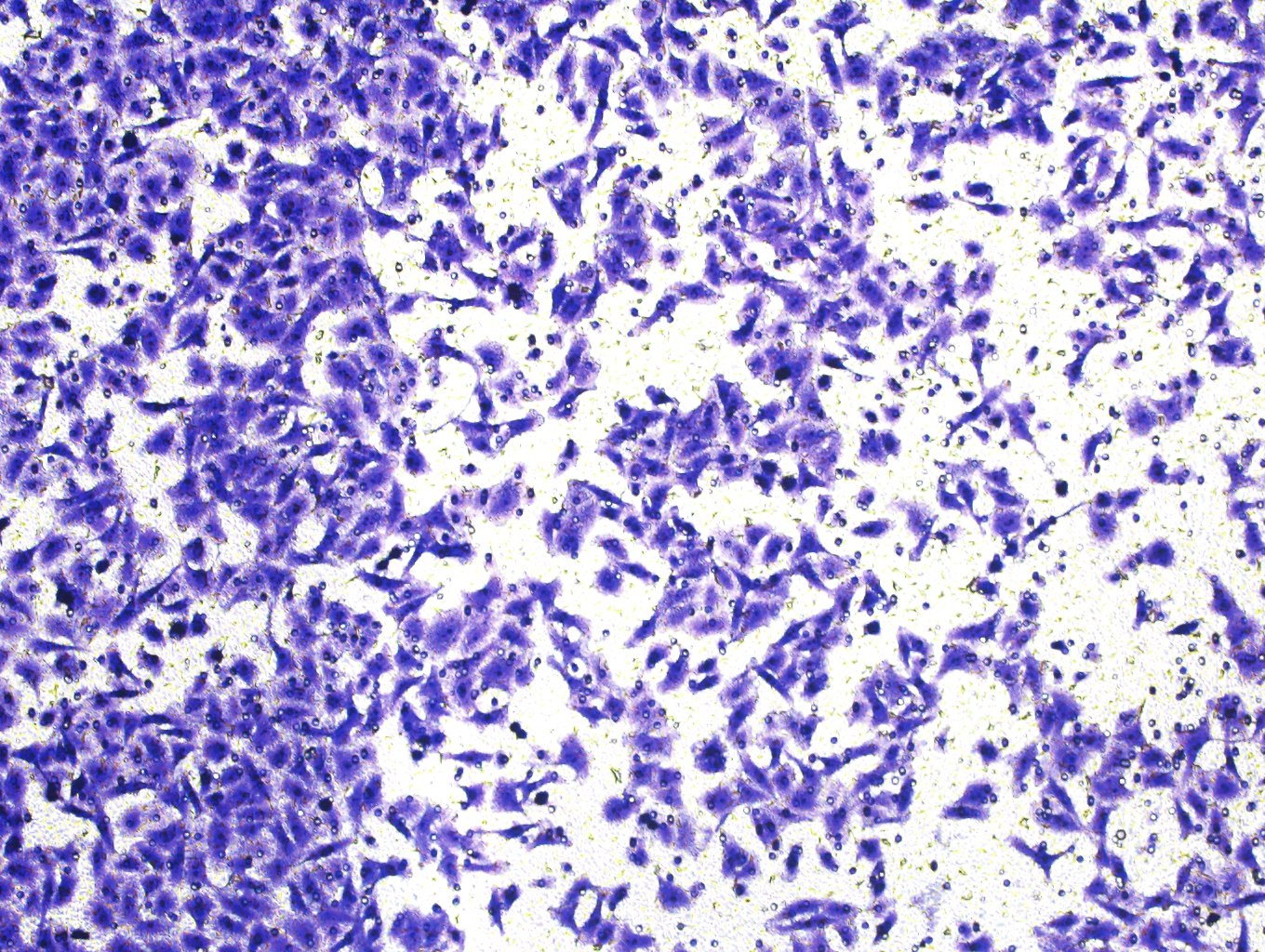

Supplement: Supplementary file 3 — Source data Fig. 2 [file 44318_2025_363_MOESM3_ESM.zip › Figure 2/2E/Control (3).jpg]

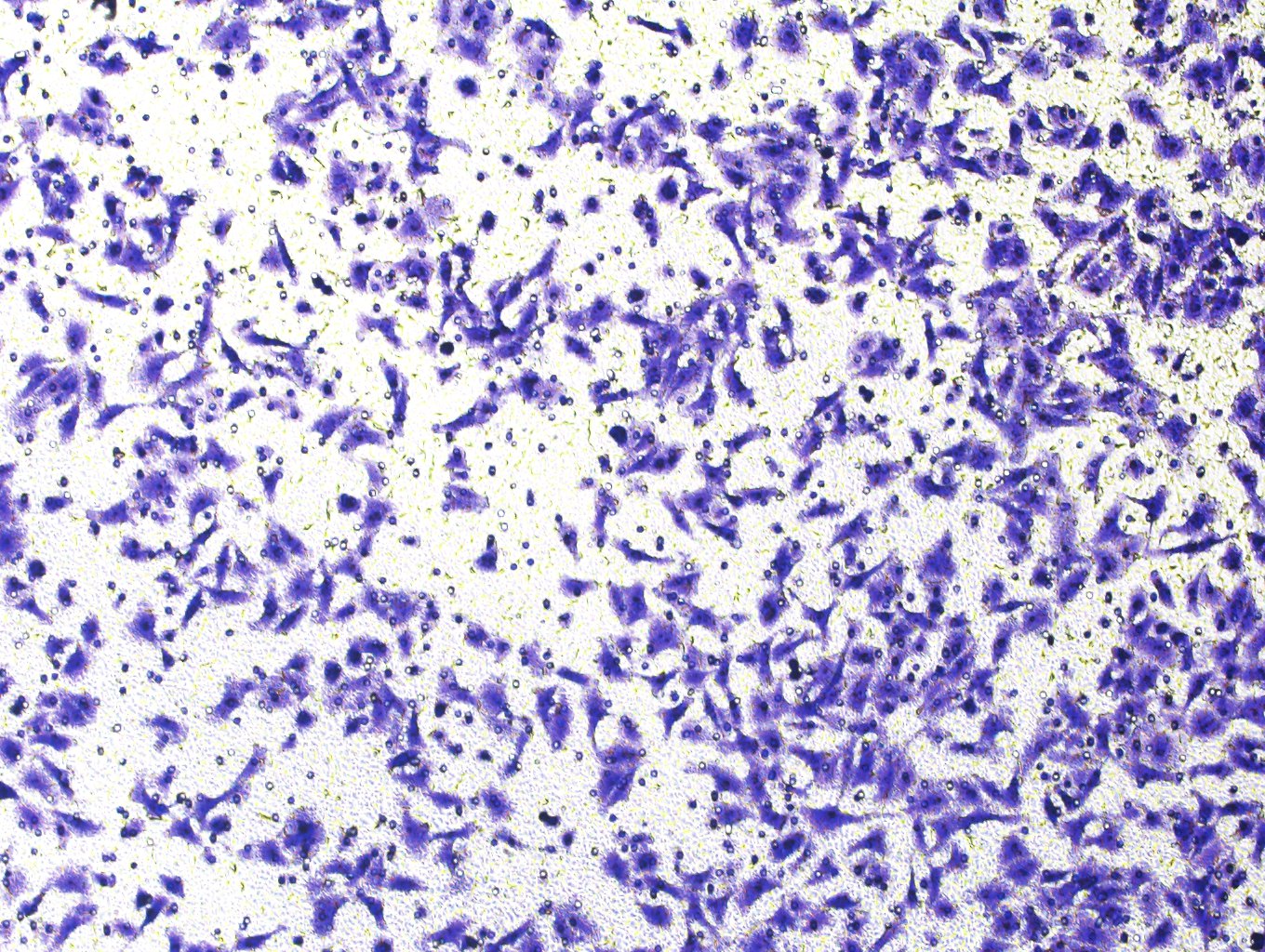

Supplement: Supplementary file 3 — Source data Fig. 2 [file 44318_2025_363_MOESM3_ESM.zip › Figure 2/2E/Control (4).jpg]

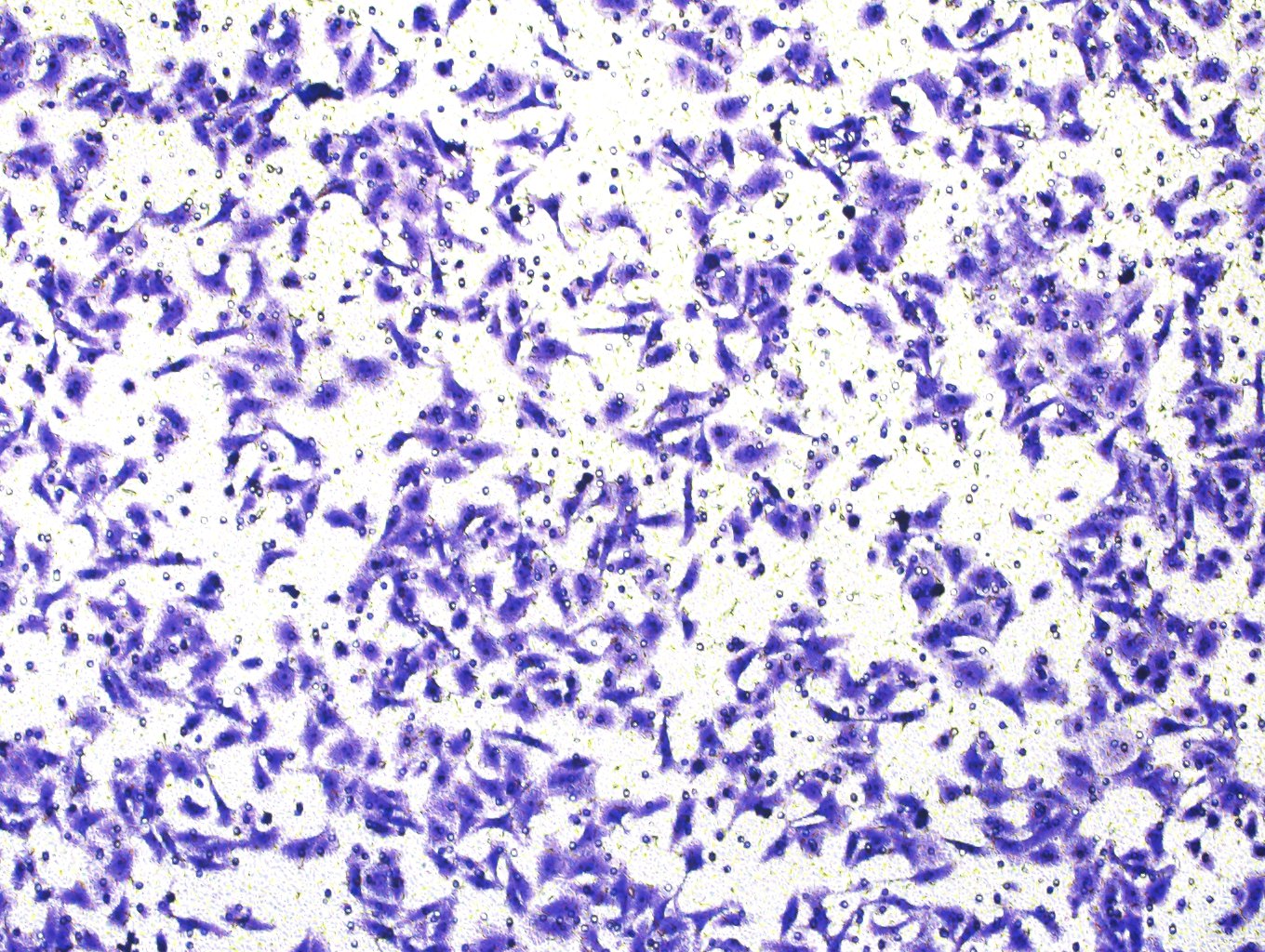

Supplement: Supplementary file 3 — Source data Fig. 2 [file 44318_2025_363_MOESM3_ESM.zip › Figure 2/2E/Control (5)-displayed in 2E.jpg]

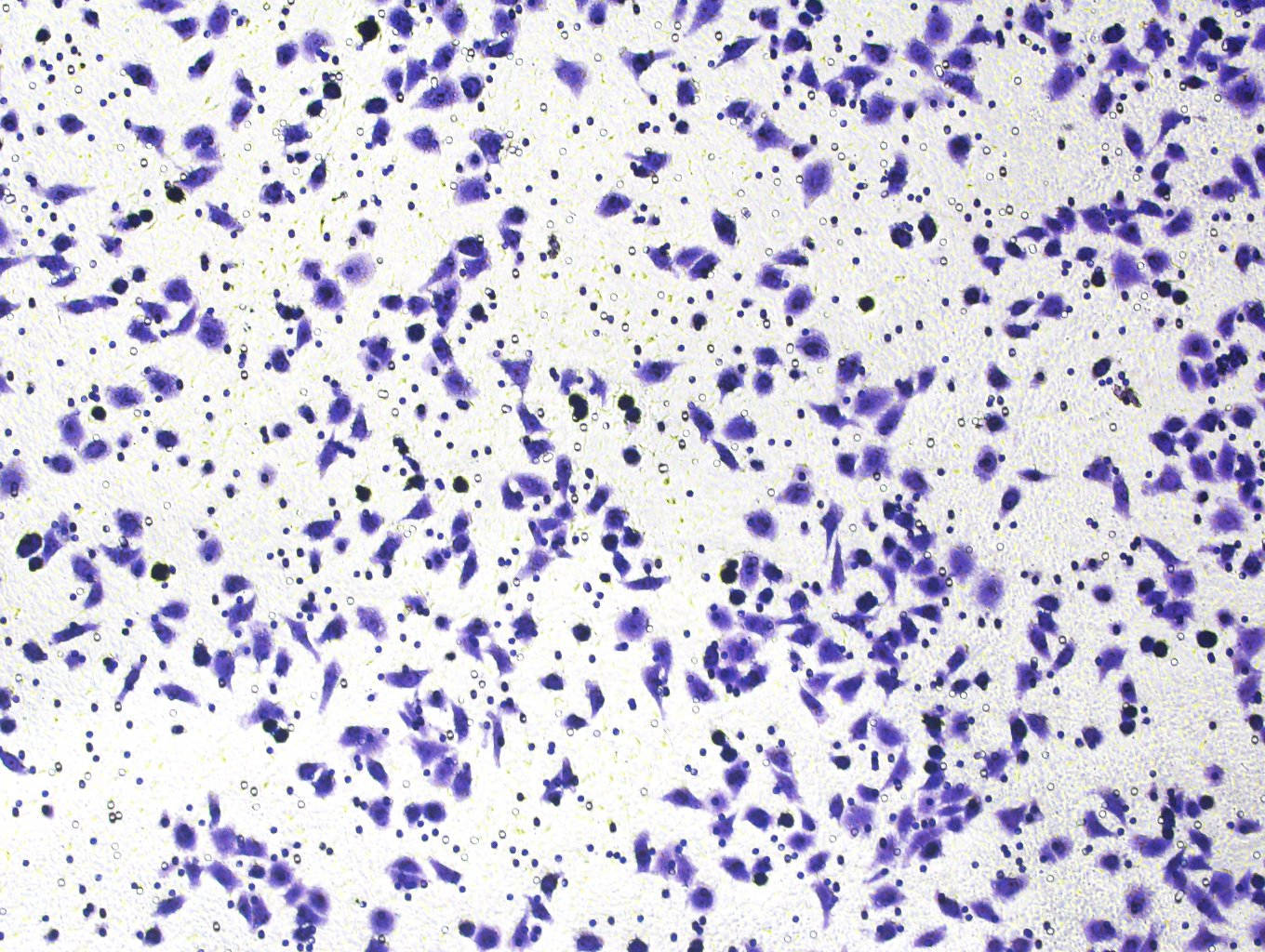

Supplement: Supplementary file 3 — Source data Fig. 2 [file 44318_2025_363_MOESM3_ESM.zip › Figure 2/2E/siEphrin A1-1 (1).jpg]

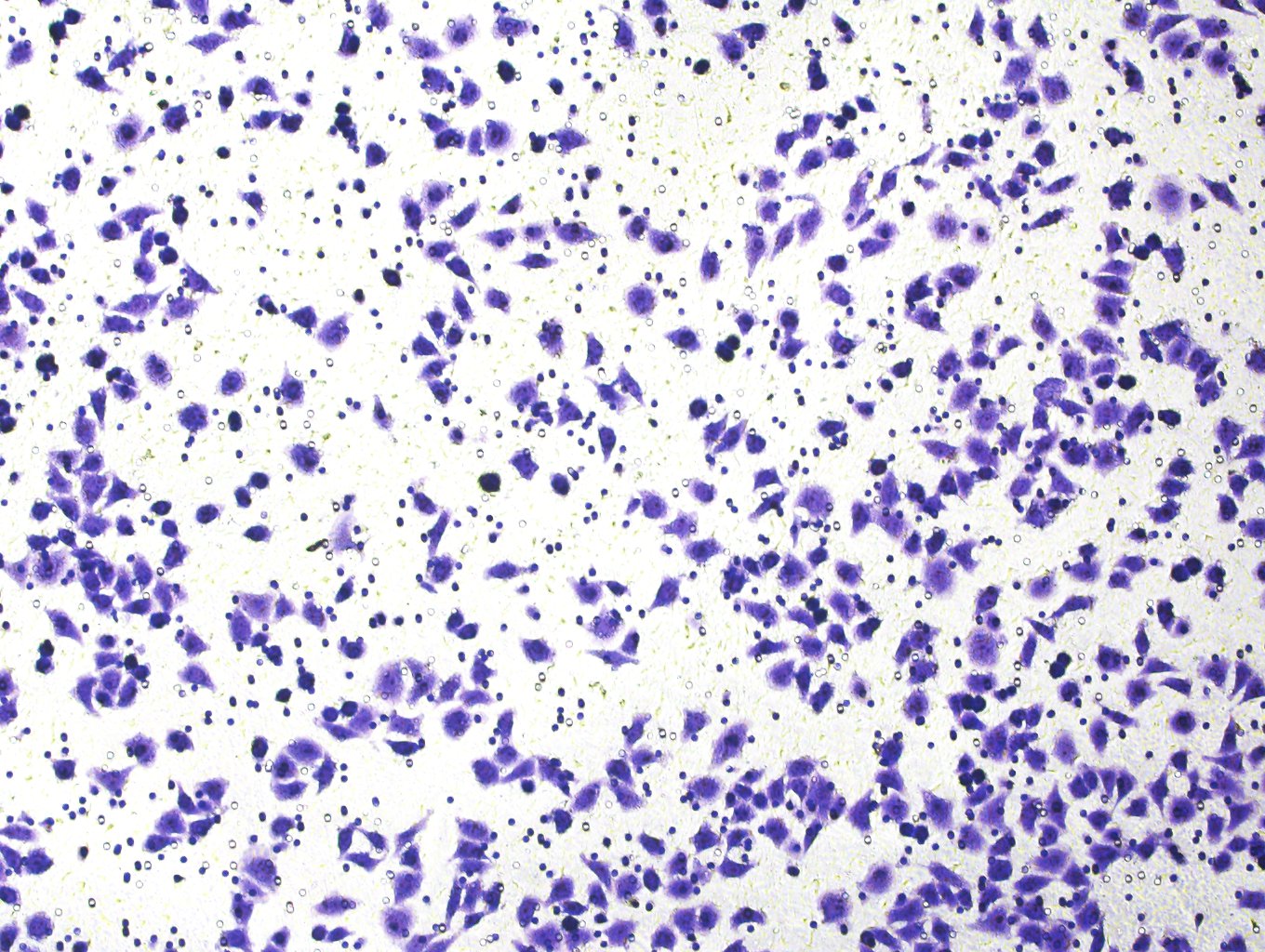

Supplement: Supplementary file 3 — Source data Fig. 2 [file 44318_2025_363_MOESM3_ESM.zip › Figure 2/2E/siEphrin A1-1 (2).jpg]

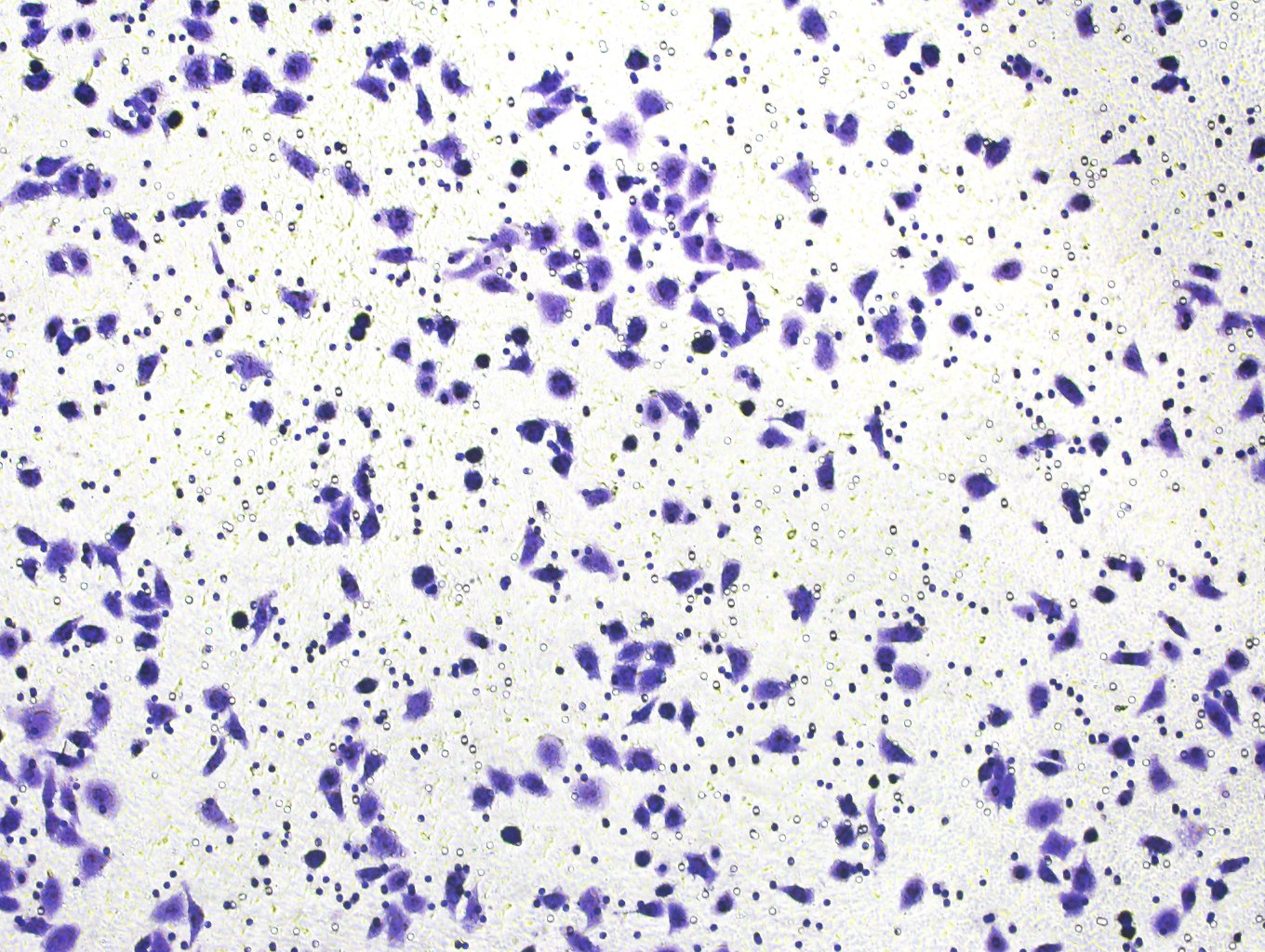

Supplement: Supplementary file 3 — Source data Fig. 2 [file 44318_2025_363_MOESM3_ESM.zip › Figure 2/2E/siEphrin A1-1 (3)-displayed in 2E.jpg]

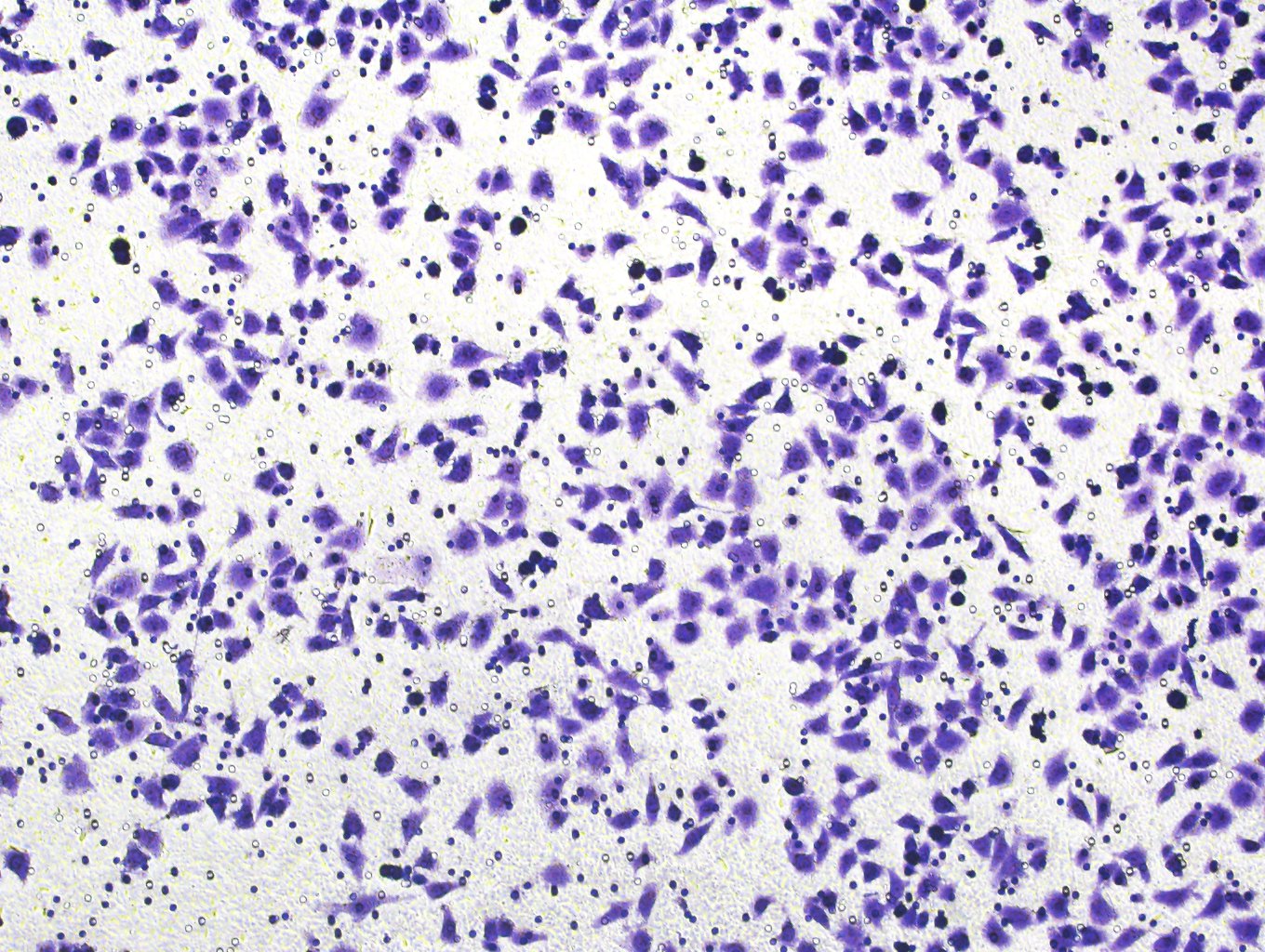

Supplement: Supplementary file 3 — Source data Fig. 2 [file 44318_2025_363_MOESM3_ESM.zip › Figure 2/2E/siEphrin A1-1 (4).jpg]

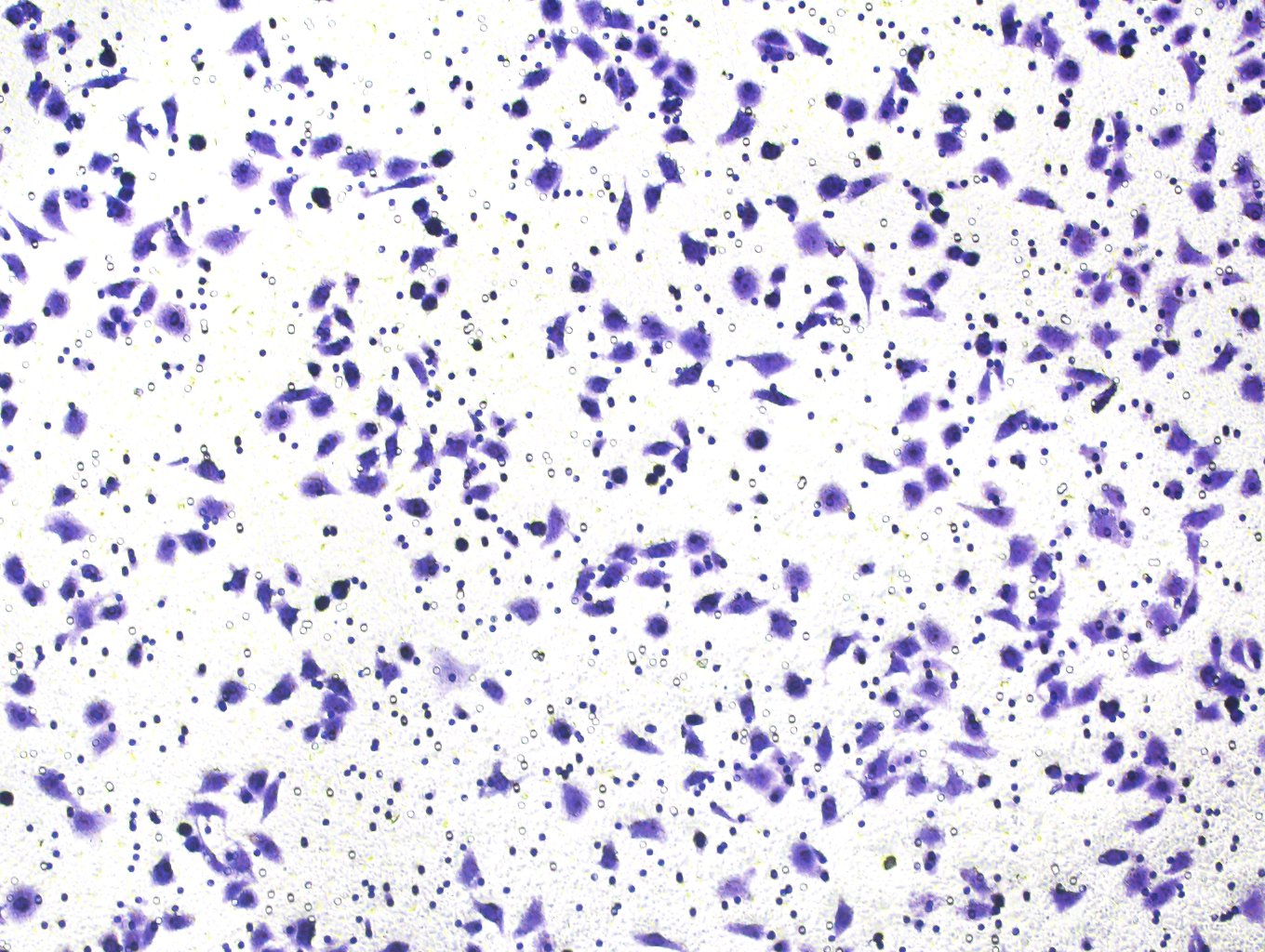

Supplement: Supplementary file 3 — Source data Fig. 2 [file 44318_2025_363_MOESM3_ESM.zip › Figure 2/2E/siEphrin A1-1 (5).jpg]

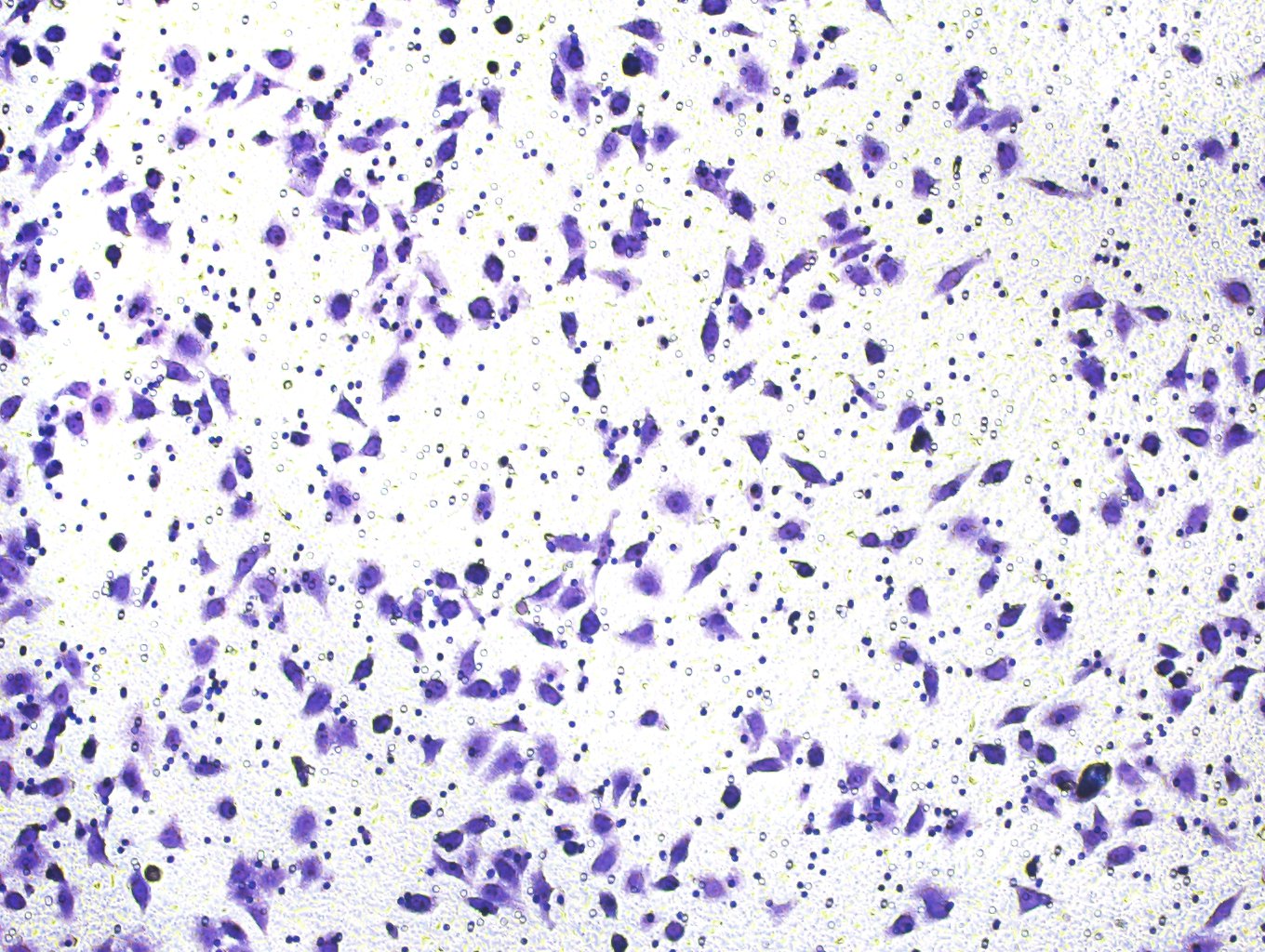

Supplement: Supplementary file 3 — Source data Fig. 2 [file 44318_2025_363_MOESM3_ESM.zip › Figure 2/2E/siEphrin A1-2 (1).jpg]

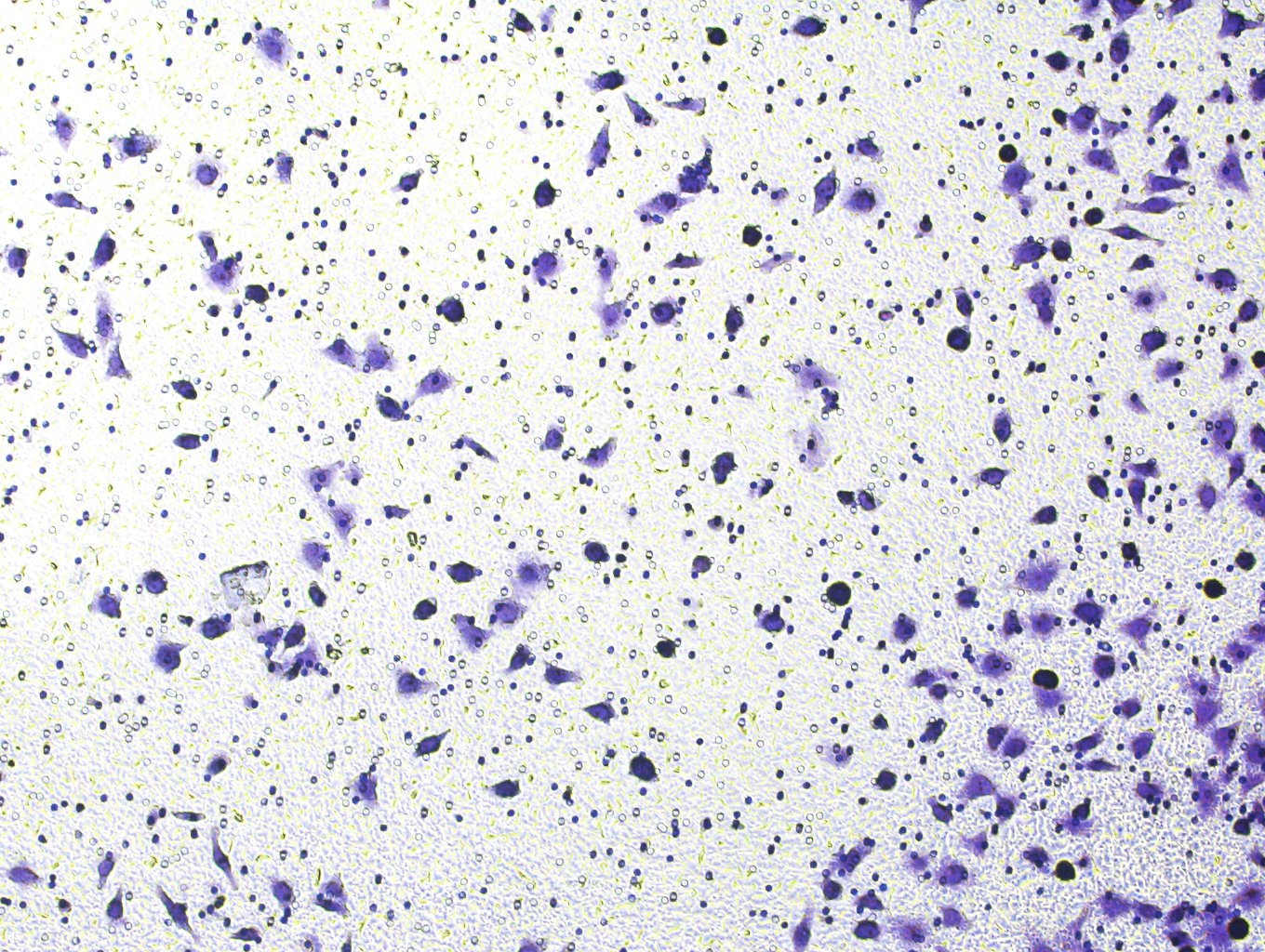

Supplement: Supplementary file 3 — Source data Fig. 2 [file 44318_2025_363_MOESM3_ESM.zip › Figure 2/2E/siEphrin A1-2 (2).jpg]

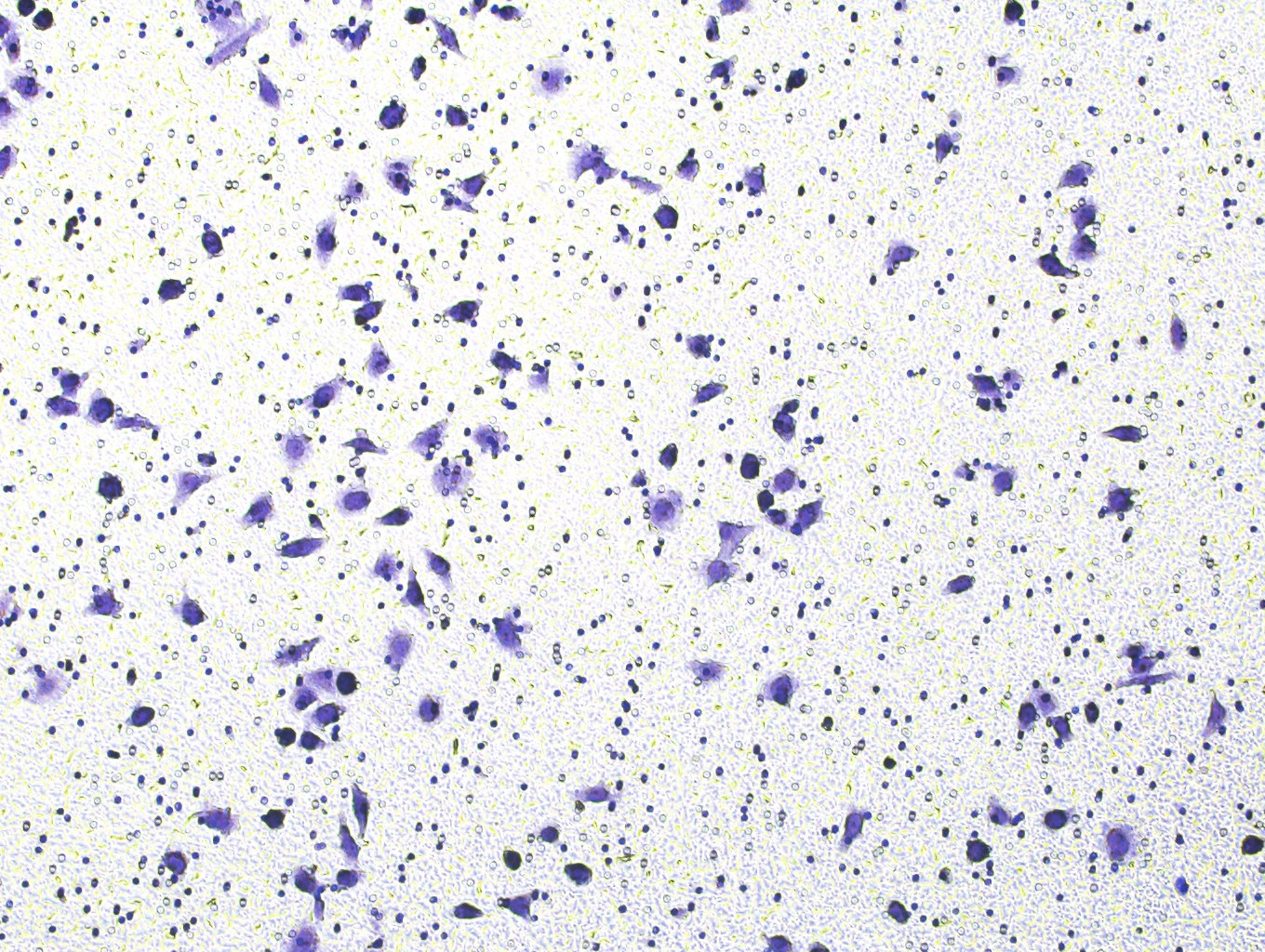

Supplement: Supplementary file 3 — Source data Fig. 2 [file 44318_2025_363_MOESM3_ESM.zip › Figure 2/2E/siEphrin A1-2 (3).jpg]

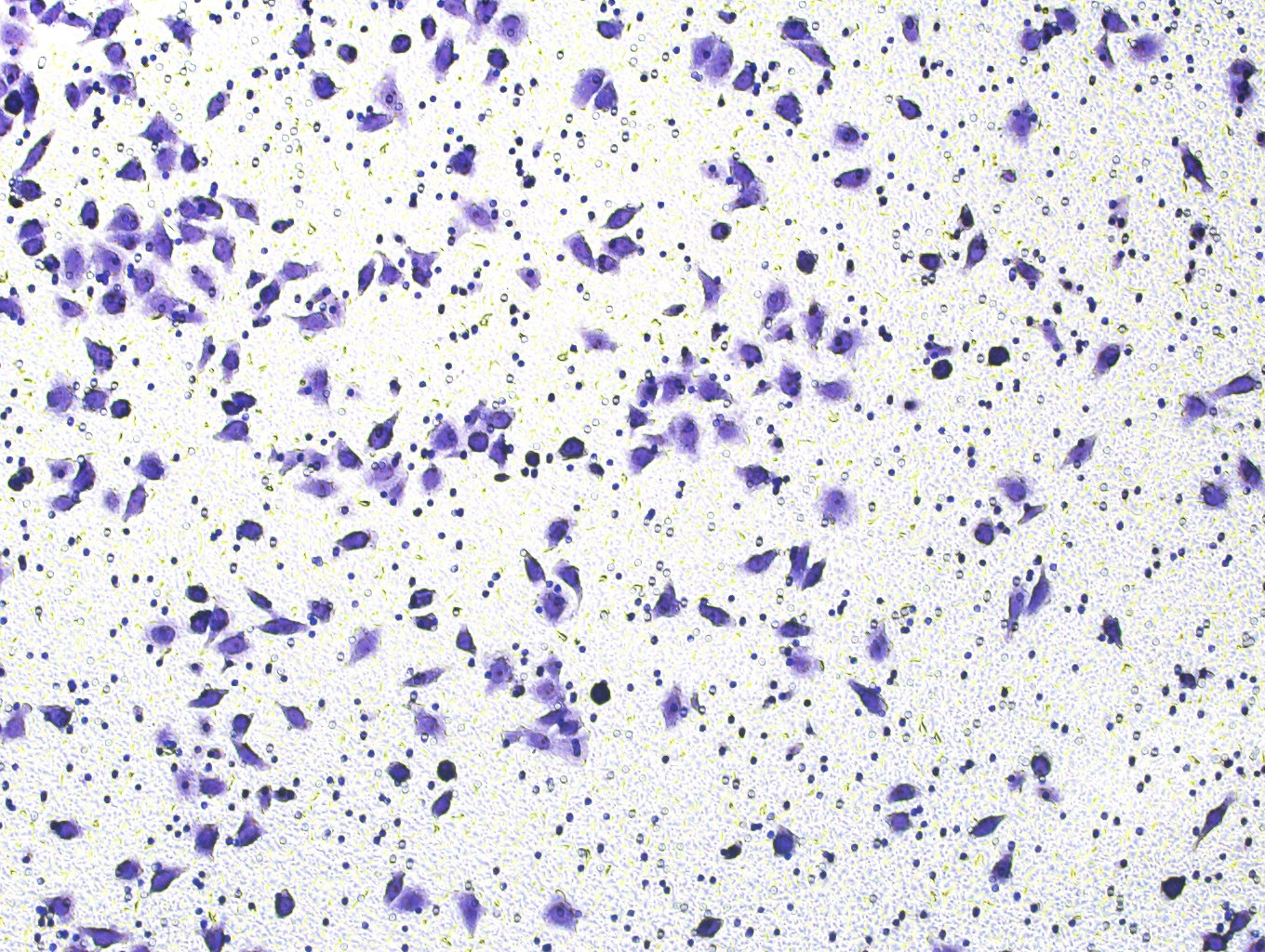

Supplement: Supplementary file 3 — Source data Fig. 2 [file 44318_2025_363_MOESM3_ESM.zip › Figure 2/2E/siEphrin A1-2 (4)-displayed in 2E.jpg]

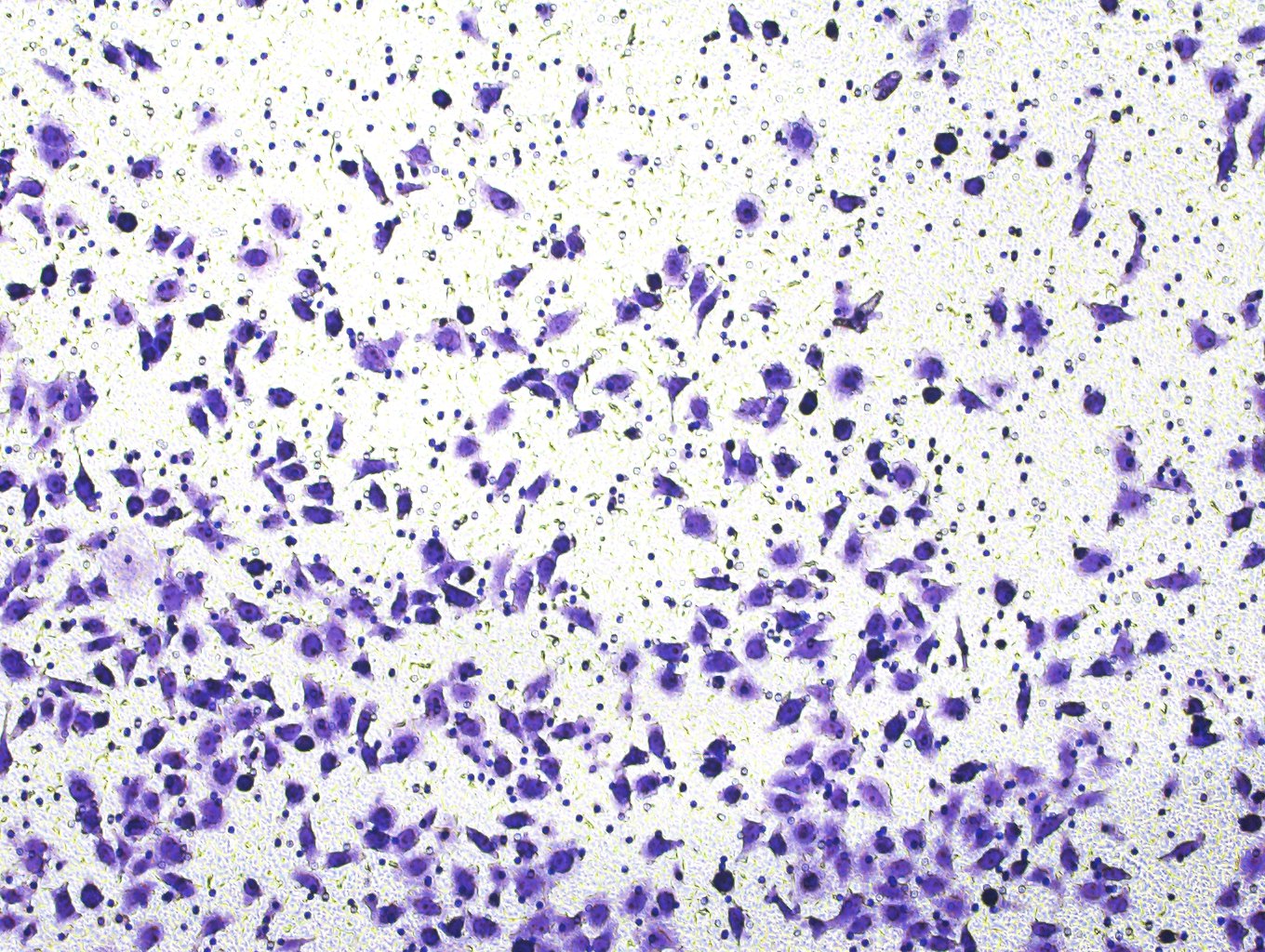

Supplement: Supplementary file 3 — Source data Fig. 2 [file 44318_2025_363_MOESM3_ESM.zip › Figure 2/2E/siEphrin A1-2 (5).jpg]

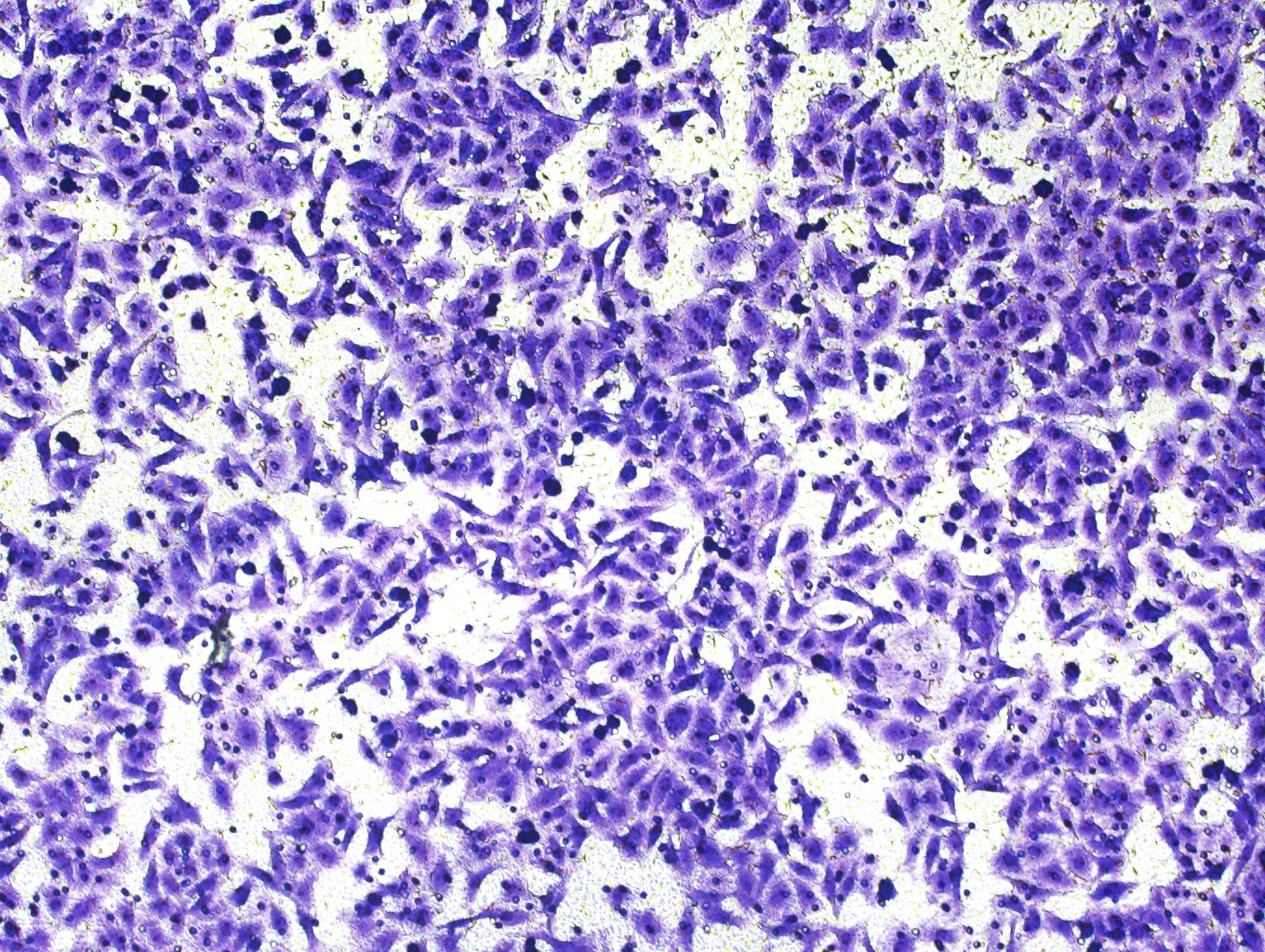

Supplement: Supplementary file 3 — Source data Fig. 2 [file 44318_2025_363_MOESM3_ESM.zip › Figure 2/2G/Control (1).jpg]

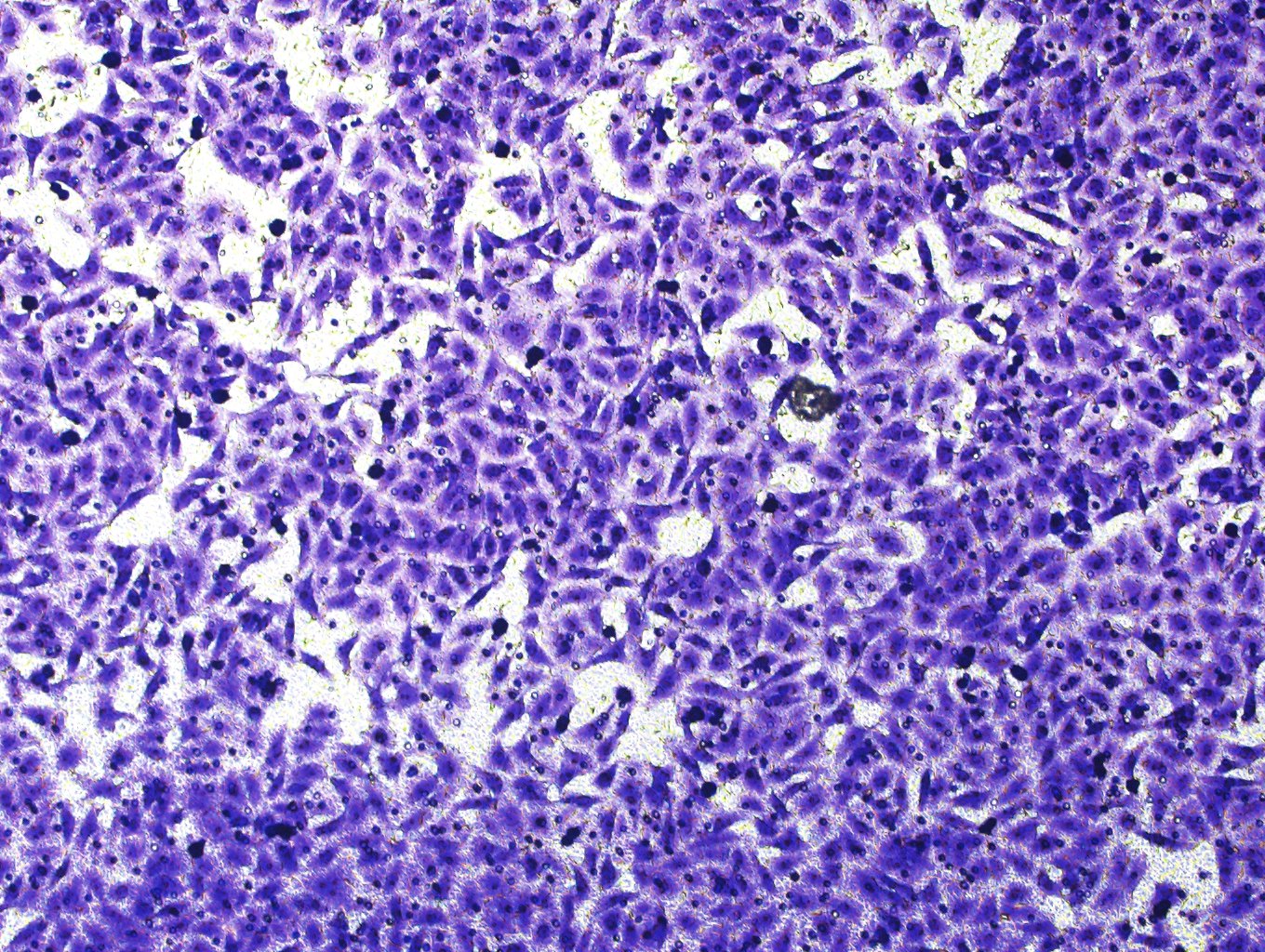

Supplement: Supplementary file 3 — Source data Fig. 2 [file 44318_2025_363_MOESM3_ESM.zip › Figure 2/2G/Control (2).jpg]

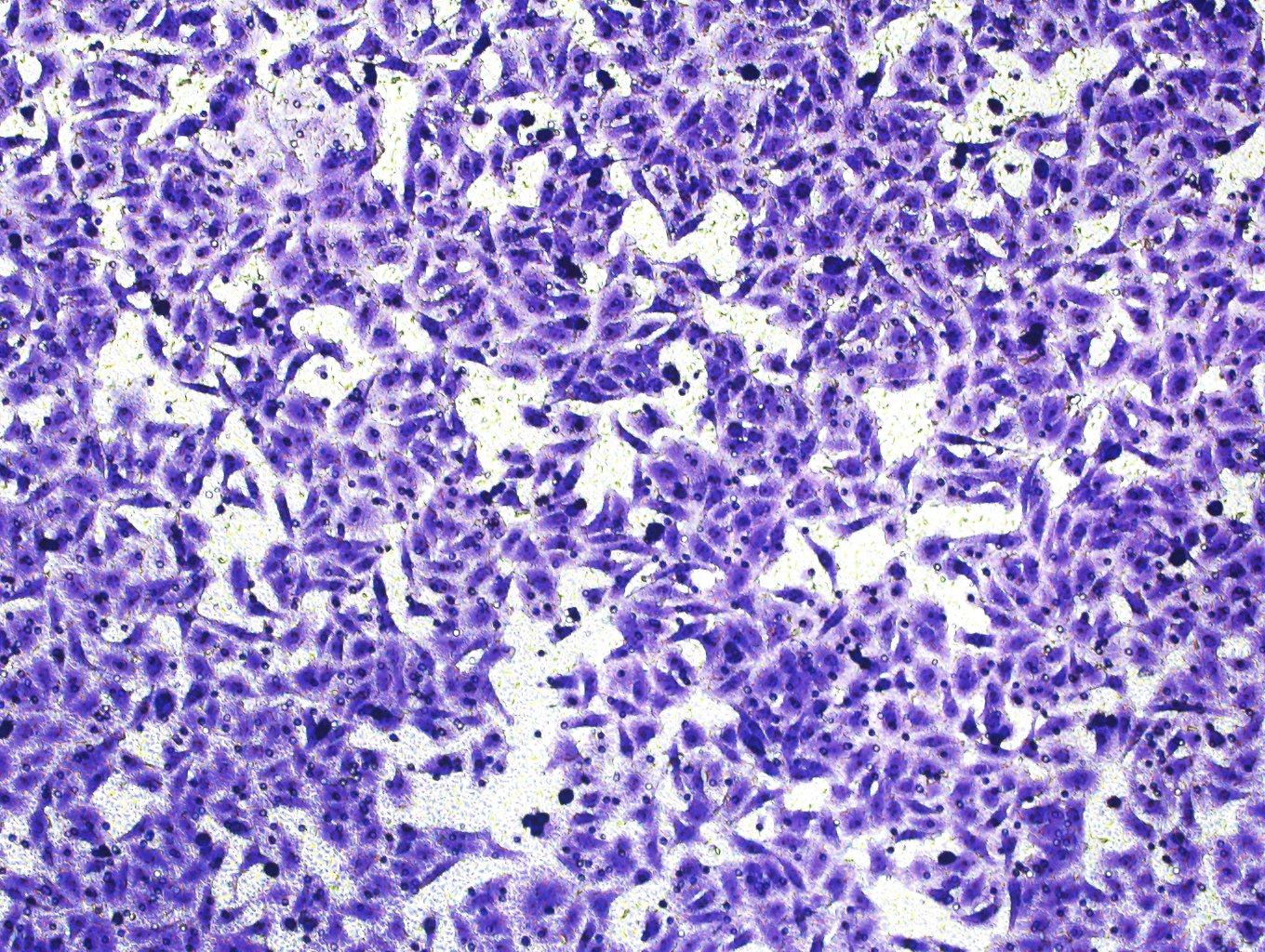

Supplement: Supplementary file 3 — Source data Fig. 2 [file 44318_2025_363_MOESM3_ESM.zip › Figure 2/2G/Control (3).jpg]

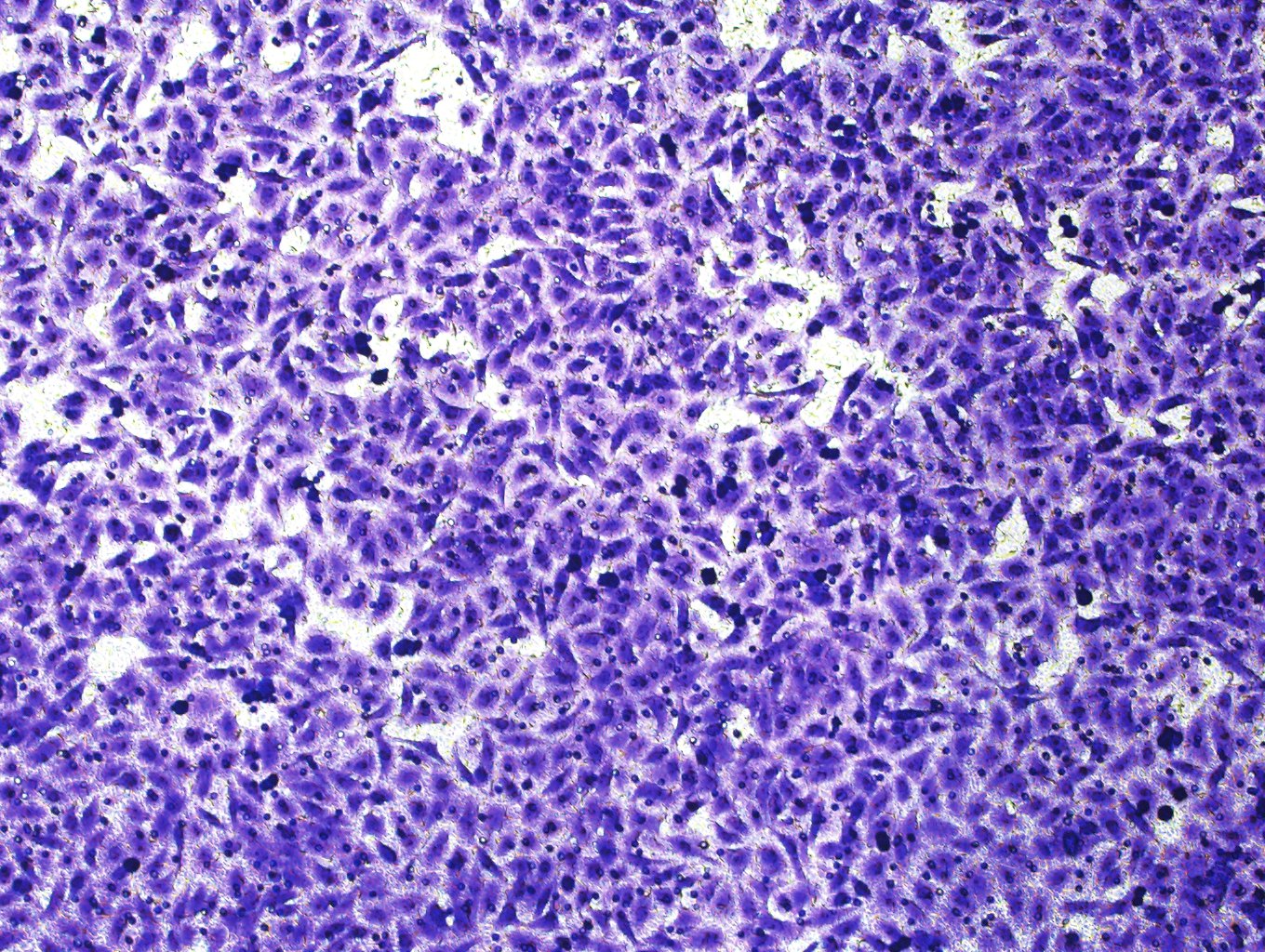

Supplement: Supplementary file 3 — Source data Fig. 2 [file 44318_2025_363_MOESM3_ESM.zip › Figure 2/2G/Control (4).jpg]

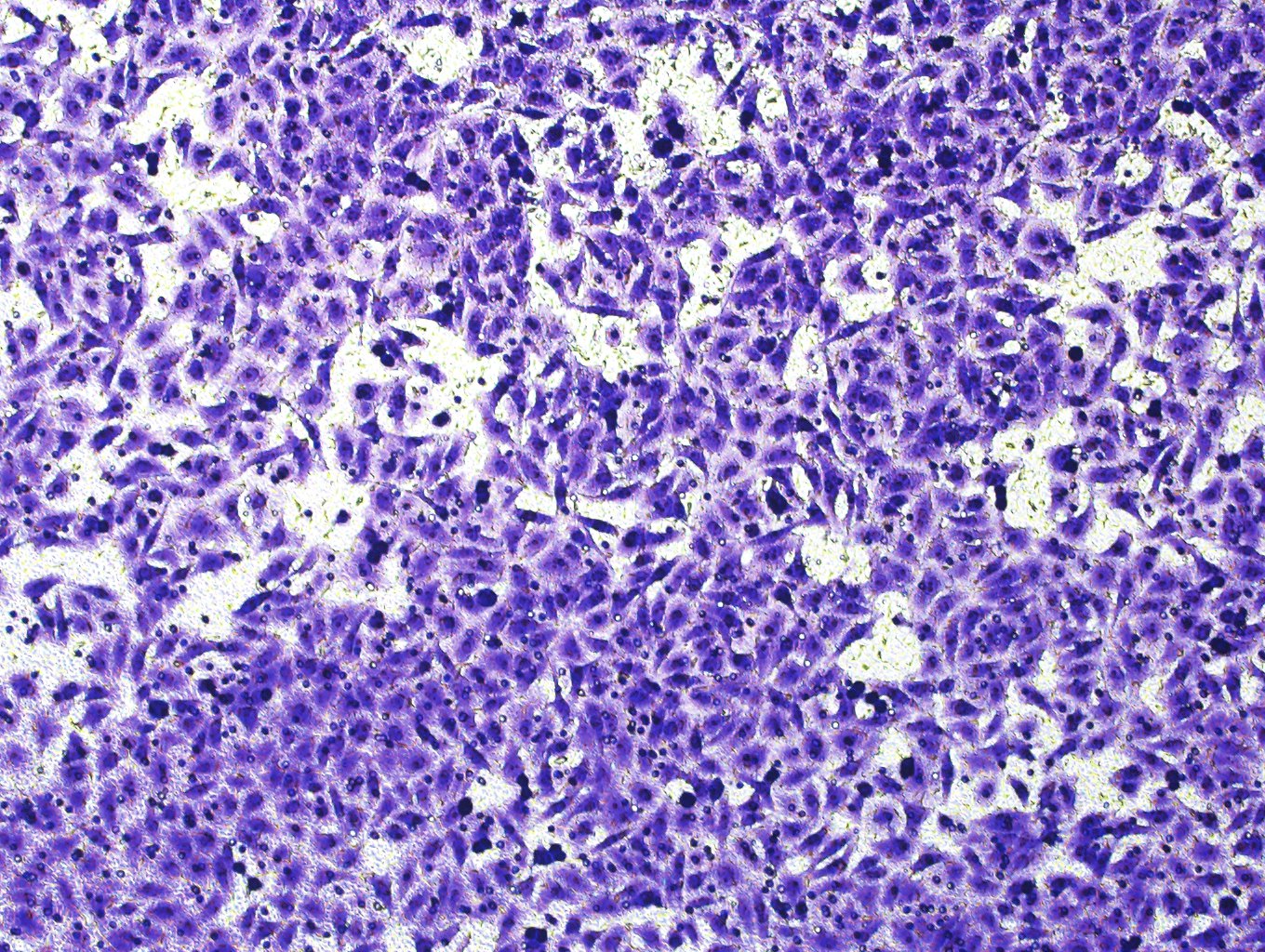

Supplement: Supplementary file 3 — Source data Fig. 2 [file 44318_2025_363_MOESM3_ESM.zip › Figure 2/2G/Control (5)-displayed in 2G.jpg]

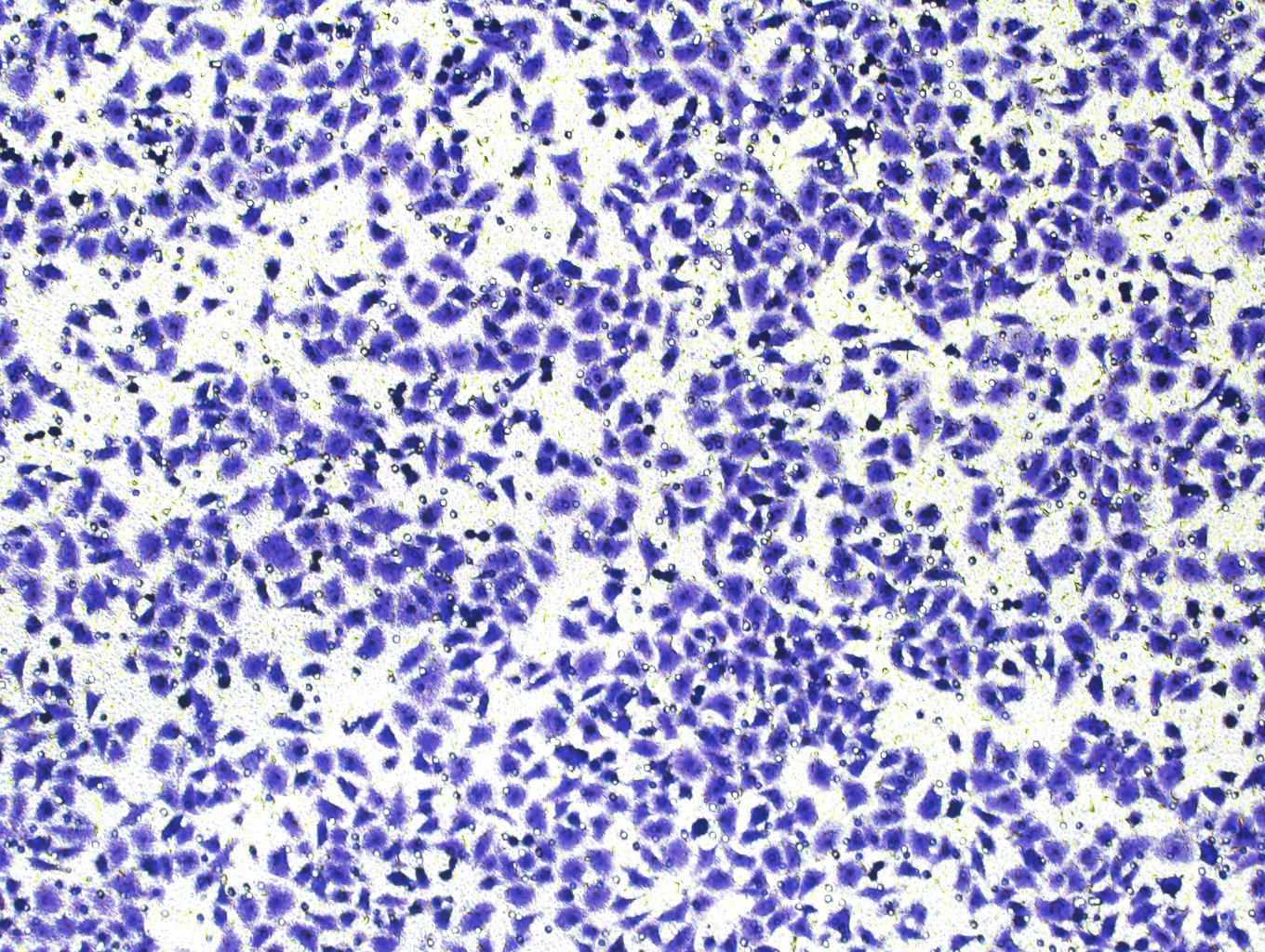

Supplement: Supplementary file 3 — Source data Fig. 2 [file 44318_2025_363_MOESM3_ESM.zip › Figure 2/2G/siEphrin A1-1 (1).jpg]

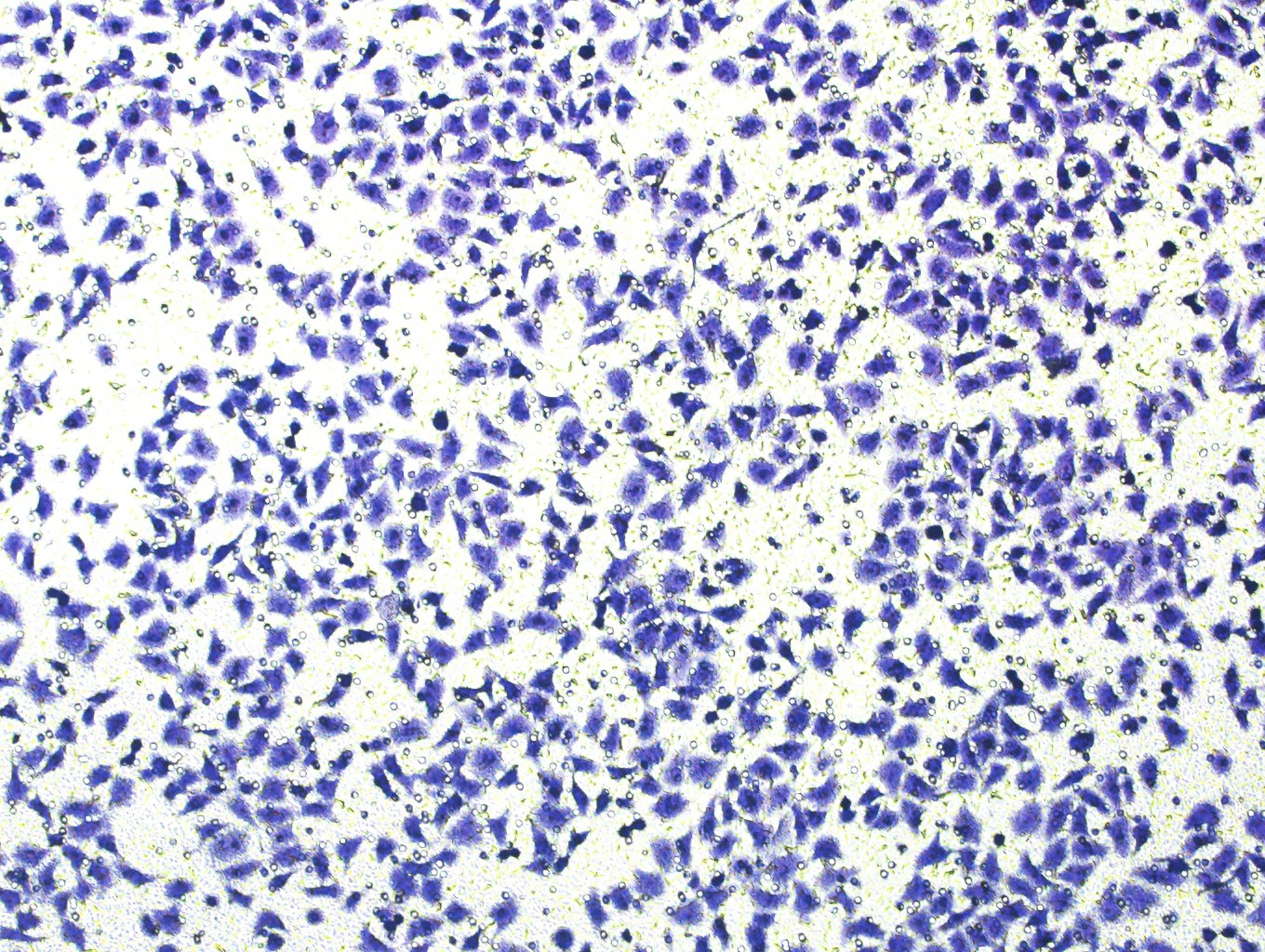

Supplement: Supplementary file 3 — Source data Fig. 2 [file 44318_2025_363_MOESM3_ESM.zip › Figure 2/2G/siEphrin A1-1 (2).jpg]

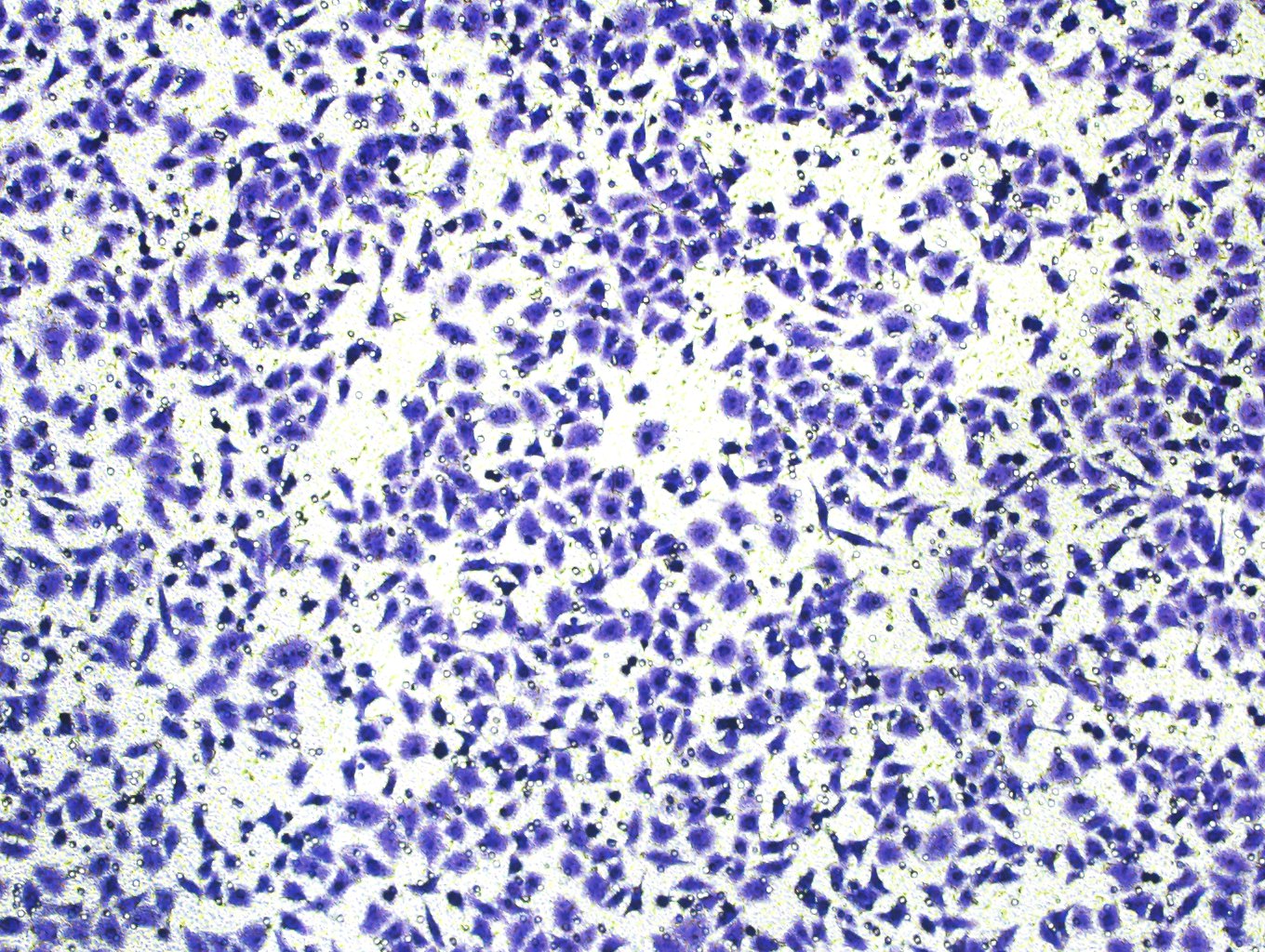

Supplement: Supplementary file 3 — Source data Fig. 2 [file 44318_2025_363_MOESM3_ESM.zip › Figure 2/2G/siEphrin A1-1 (3).jpg]

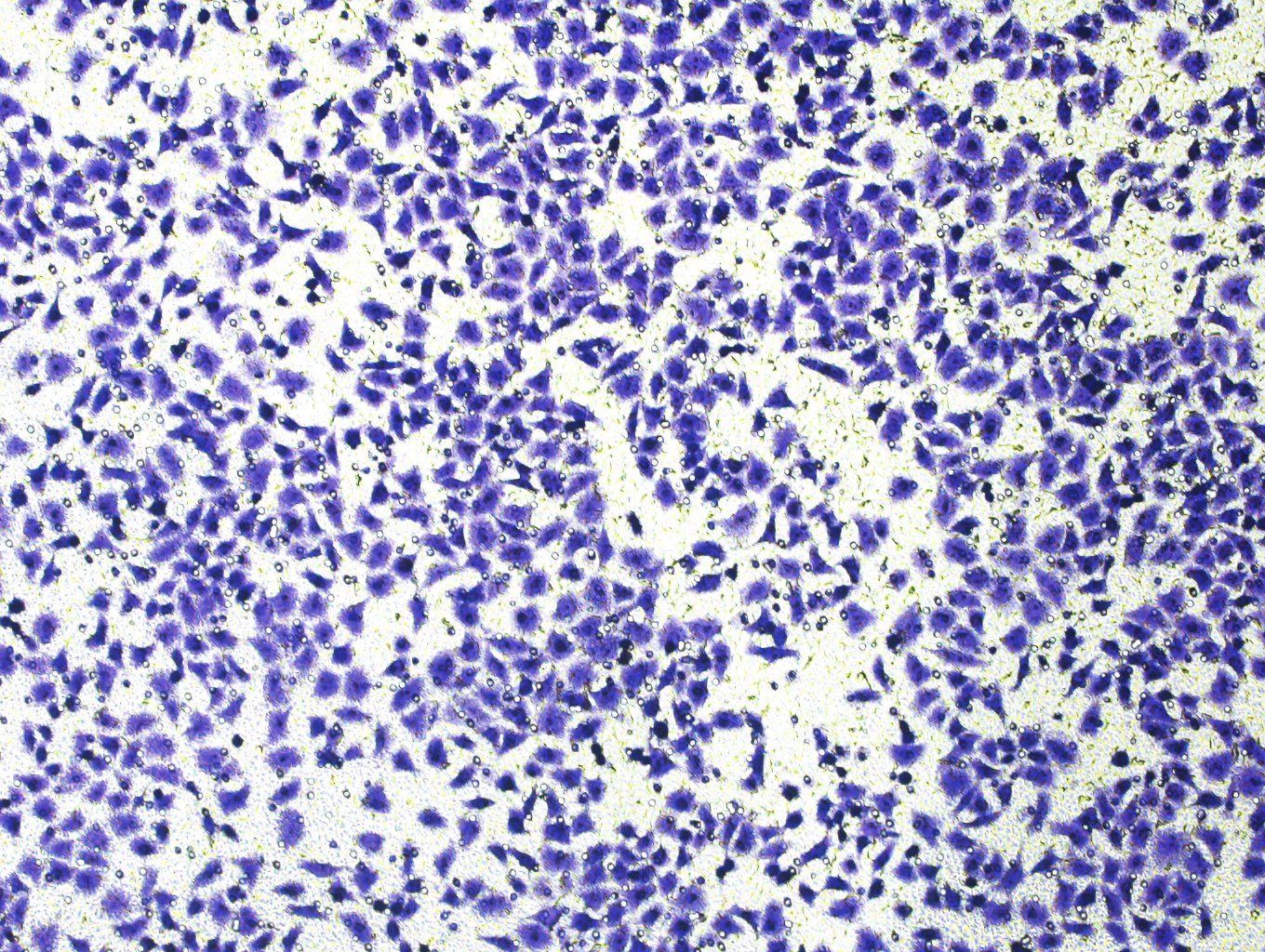

Supplement: Supplementary file 3 — Source data Fig. 2 [file 44318_2025_363_MOESM3_ESM.zip › Figure 2/2G/siEphrin A1-1 (4).jpg]

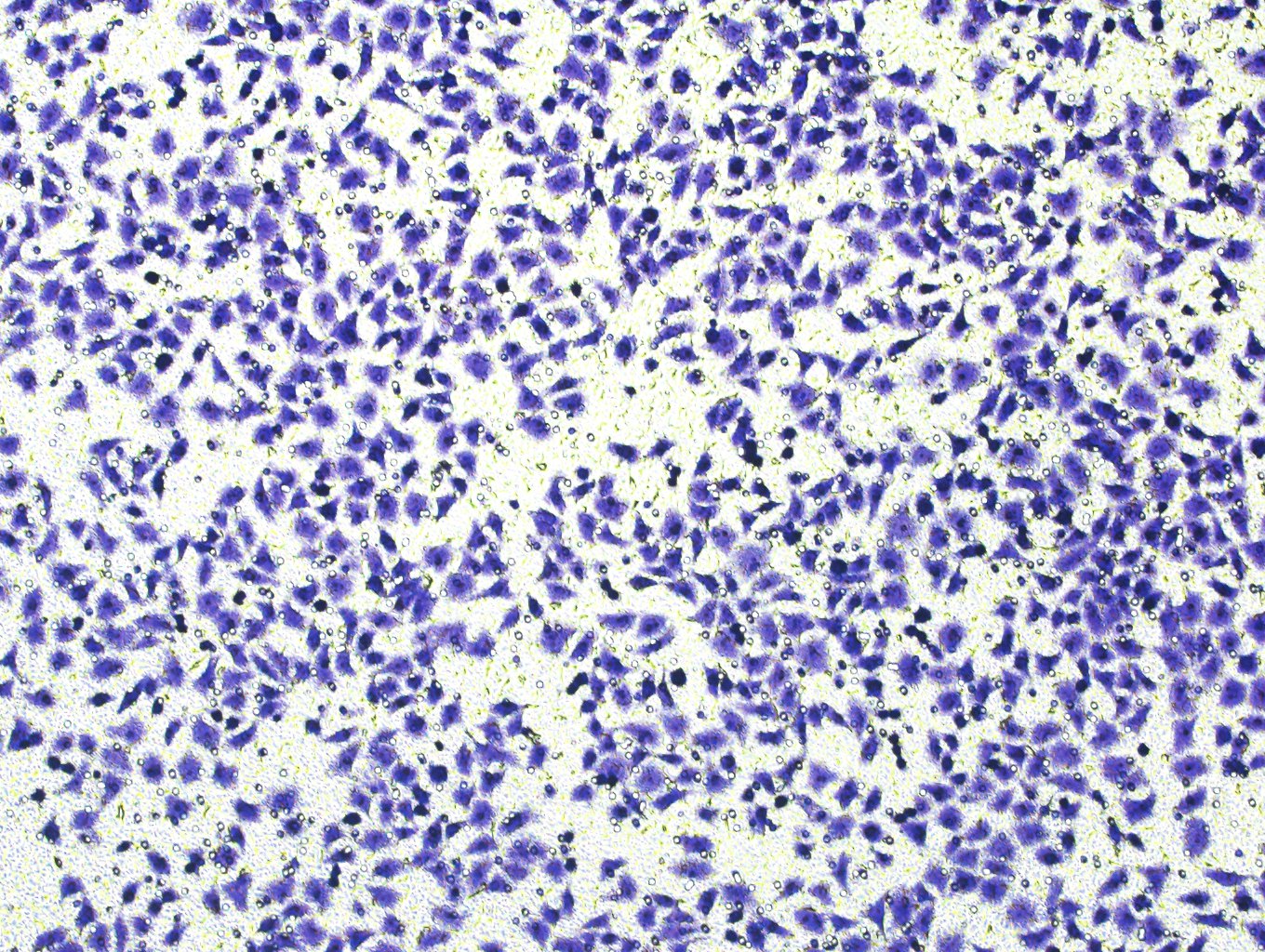

Supplement: Supplementary file 3 — Source data Fig. 2 [file 44318_2025_363_MOESM3_ESM.zip › Figure 2/2G/siEphrin A1-1 (5)-displayed in 2G.jpg]

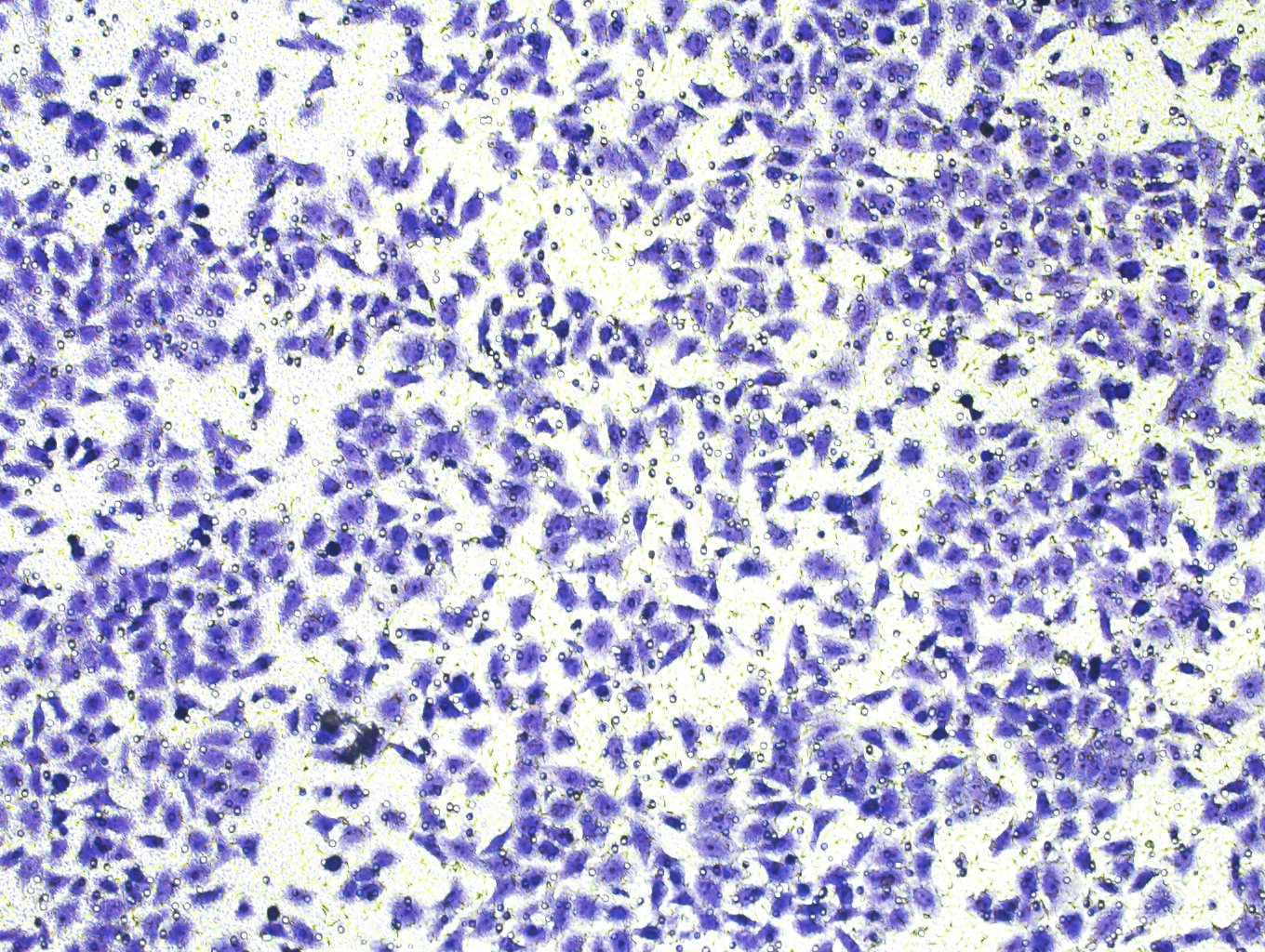

Supplement: Supplementary file 3 — Source data Fig. 2 [file 44318_2025_363_MOESM3_ESM.zip › Figure 2/2G/siEphrin A1-2 (1).jpg]

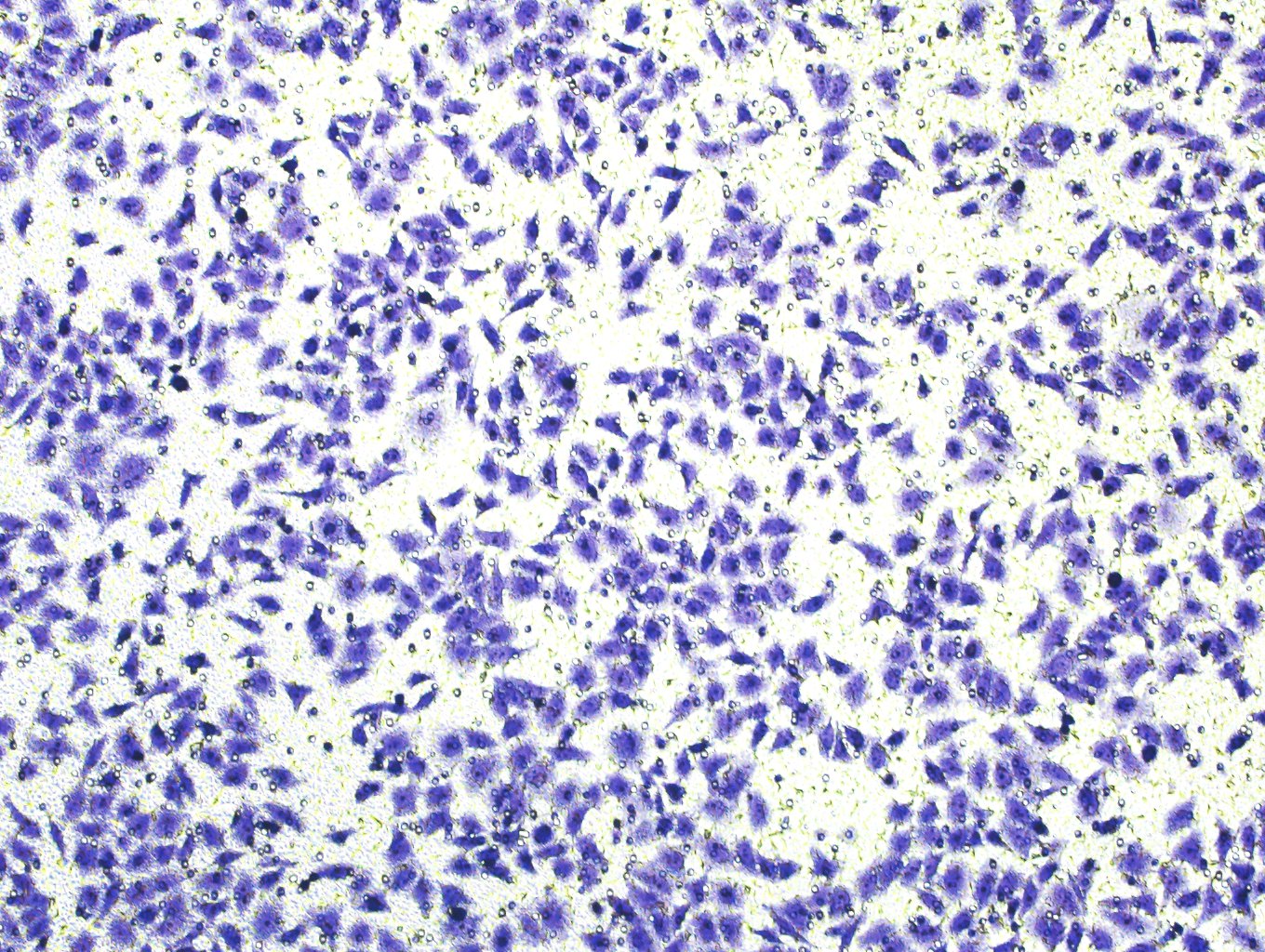

Supplement: Supplementary file 3 — Source data Fig. 2 [file 44318_2025_363_MOESM3_ESM.zip › Figure 2/2G/siEphrin A1-2 (2).jpg]

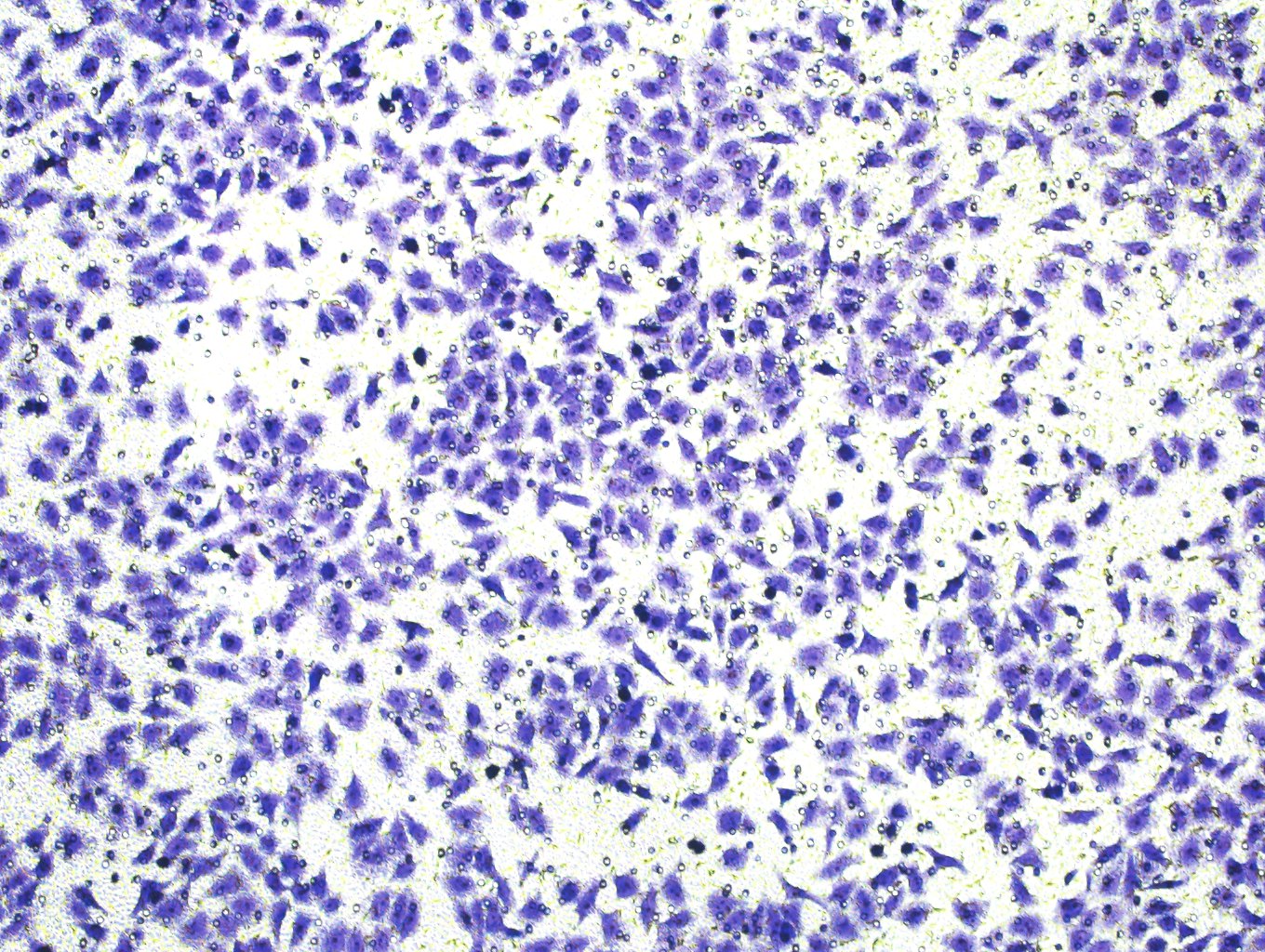

Supplement: Supplementary file 3 — Source data Fig. 2 [file 44318_2025_363_MOESM3_ESM.zip › Figure 2/2G/siEphrin A1-2 (3).jpg]

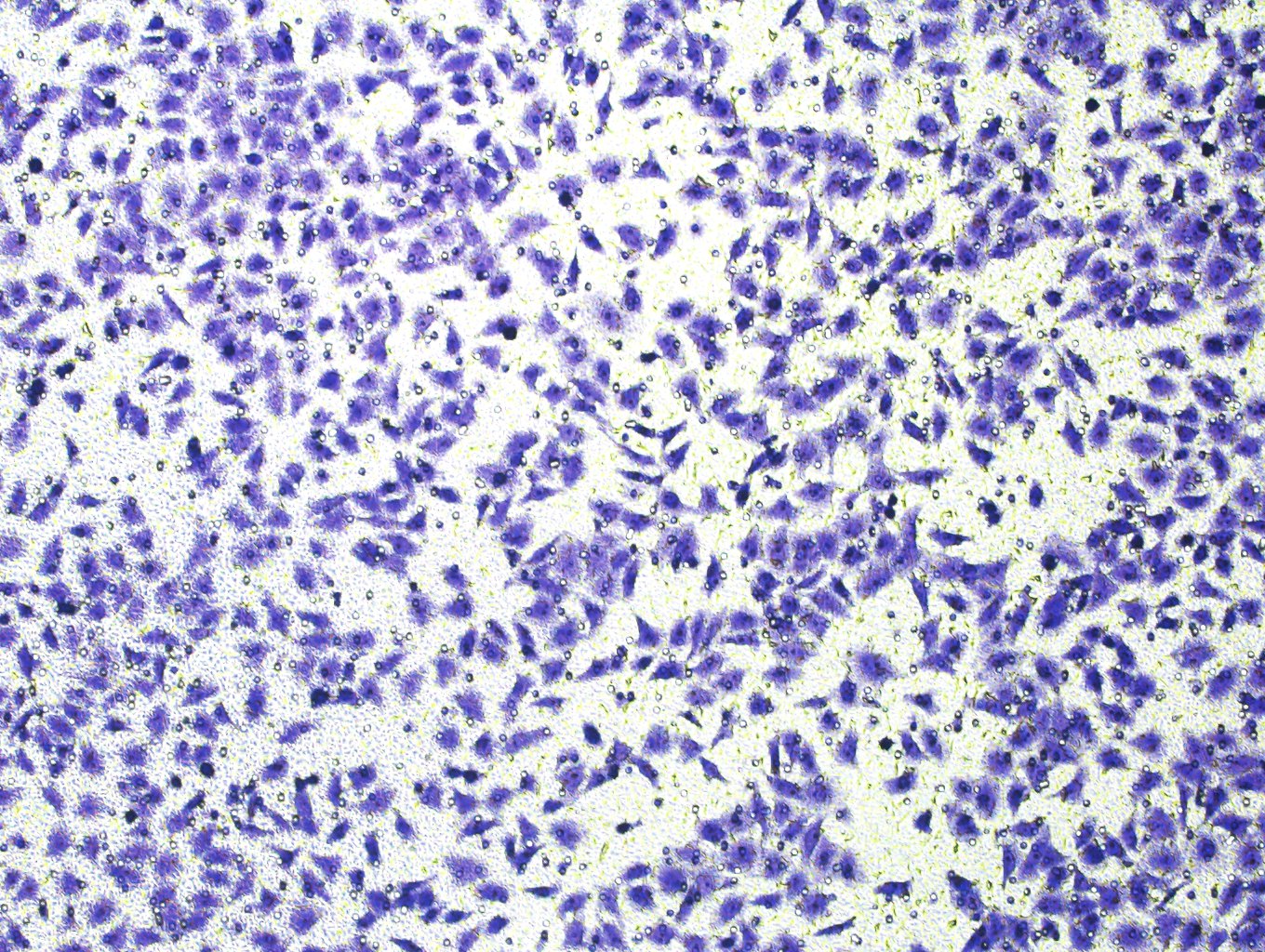

Supplement: Supplementary file 3 — Source data Fig. 2 [file 44318_2025_363_MOESM3_ESM.zip › Figure 2/2G/siEphrin A1-2 (4)-displayed in 2G.jpg]

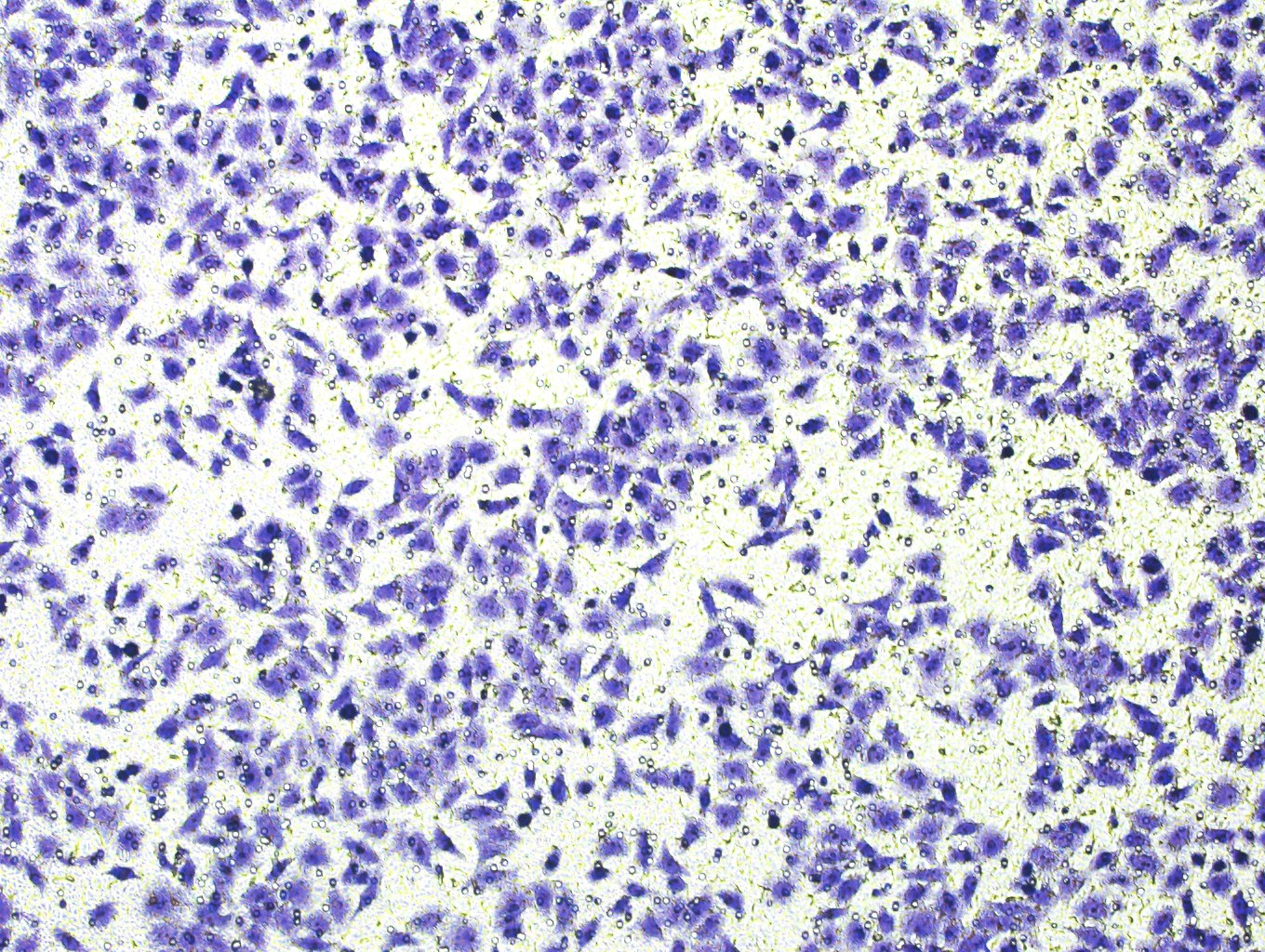

Supplement: Supplementary file 3 — Source data Fig. 2 [file 44318_2025_363_MOESM3_ESM.zip › Figure 2/2G/siEphrin A1-2 (5).jpg]

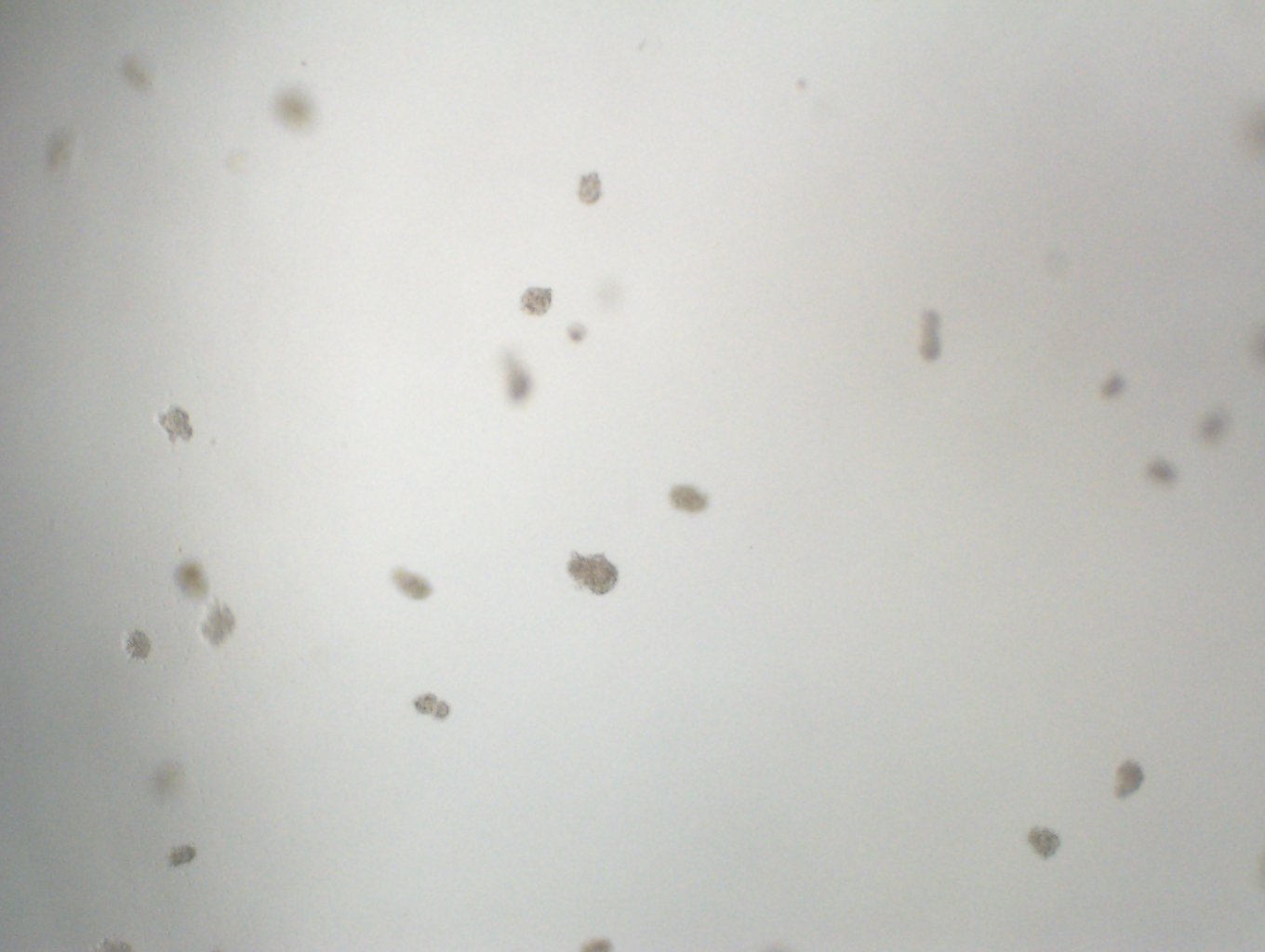

Supplement: Supplementary file 3 — Source data Fig. 2 [file 44318_2025_363_MOESM3_ESM.zip › Figure 2/2I/Control (1).jpg]

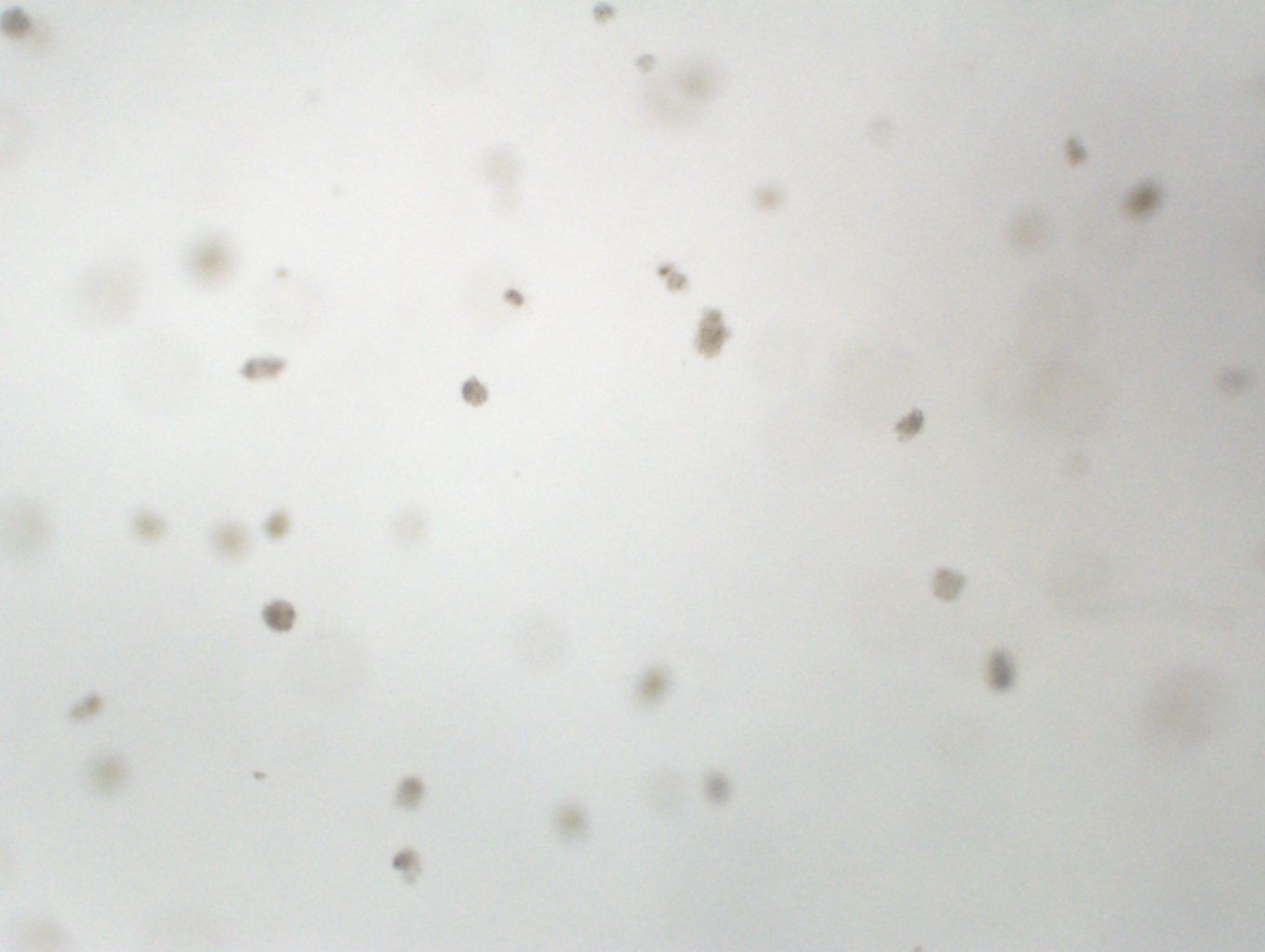

Supplement: Supplementary file 3 — Source data Fig. 2 [file 44318_2025_363_MOESM3_ESM.zip › Figure 2/2I/Control (10).jpg]

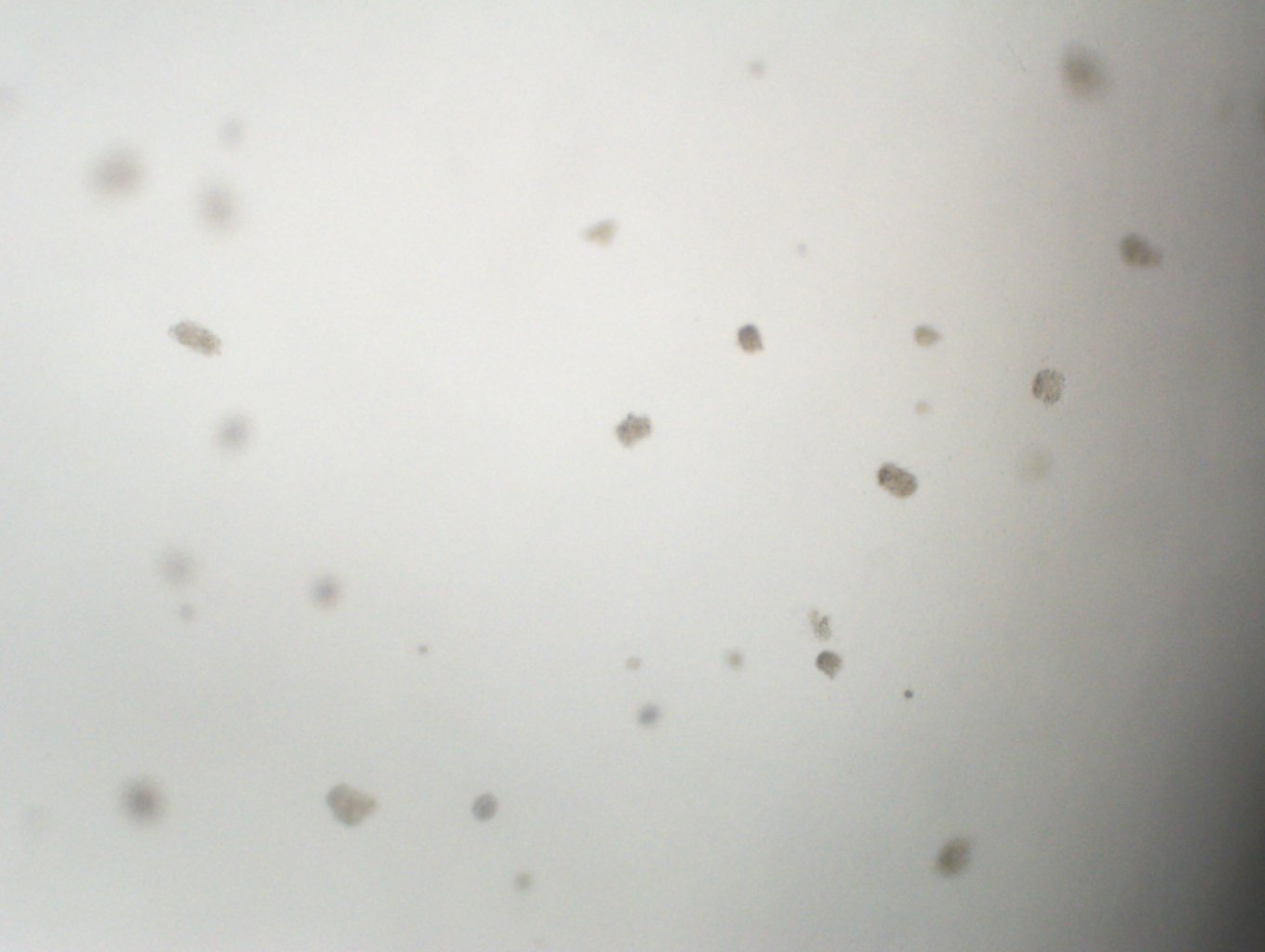

Supplement: Supplementary file 3 — Source data Fig. 2 [file 44318_2025_363_MOESM3_ESM.zip › Figure 2/2I/Control (2).jpg]

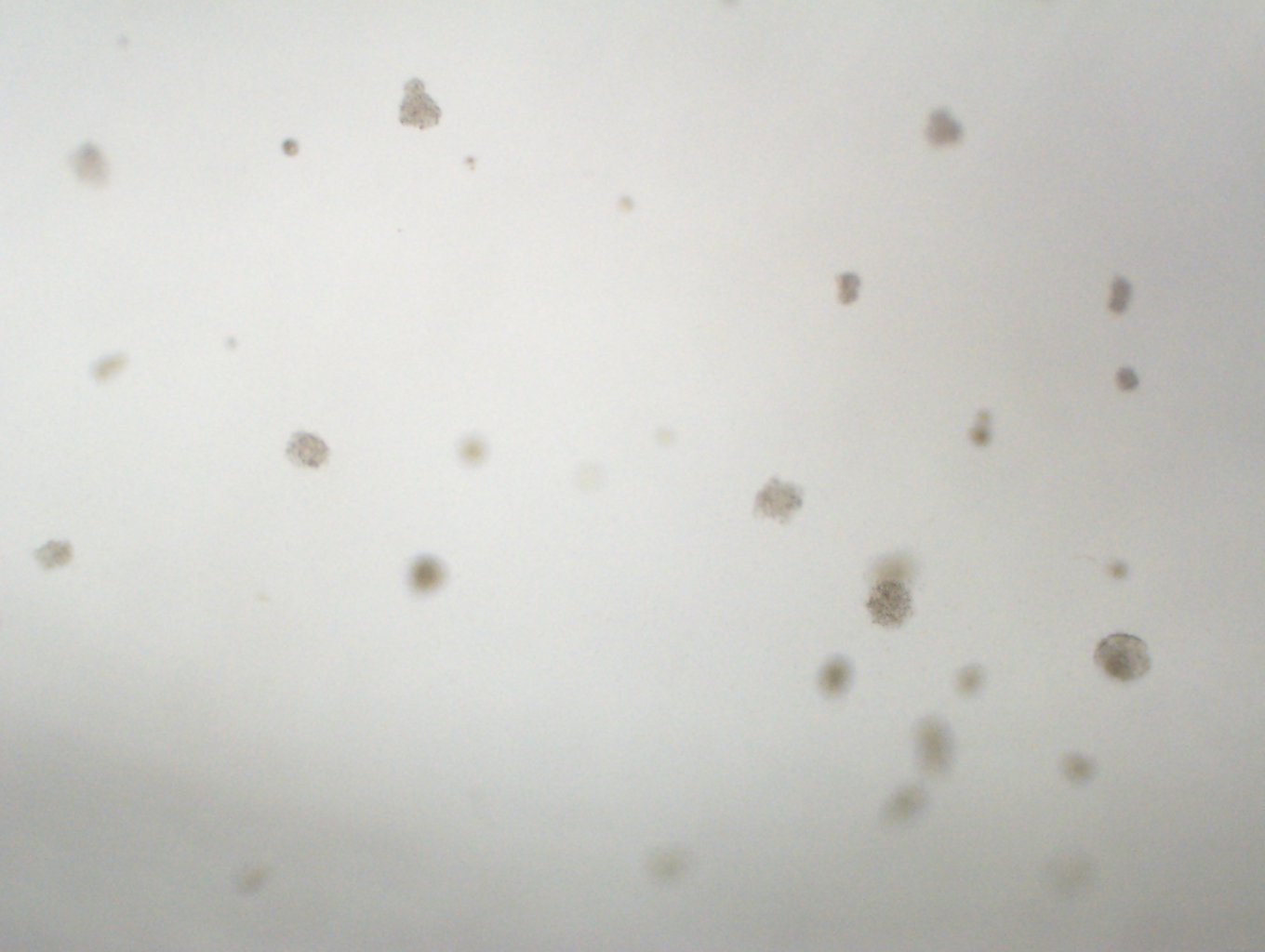

Supplement: Supplementary file 3 — Source data Fig. 2 [file 44318_2025_363_MOESM3_ESM.zip › Figure 2/2I/Control (3).jpg]

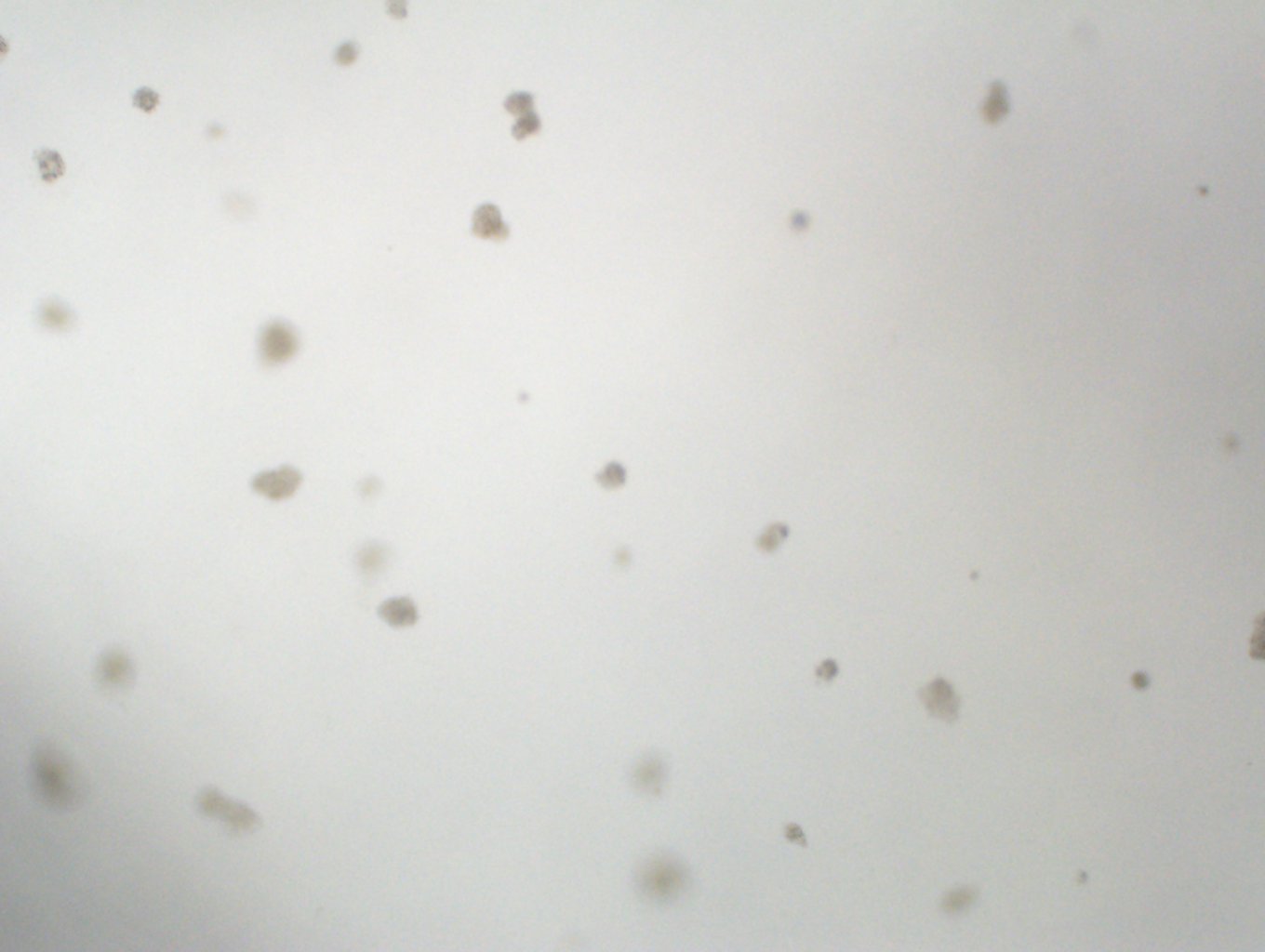

Supplement: Supplementary file 3 — Source data Fig. 2 [file 44318_2025_363_MOESM3_ESM.zip › Figure 2/2I/Control (4).jpg]

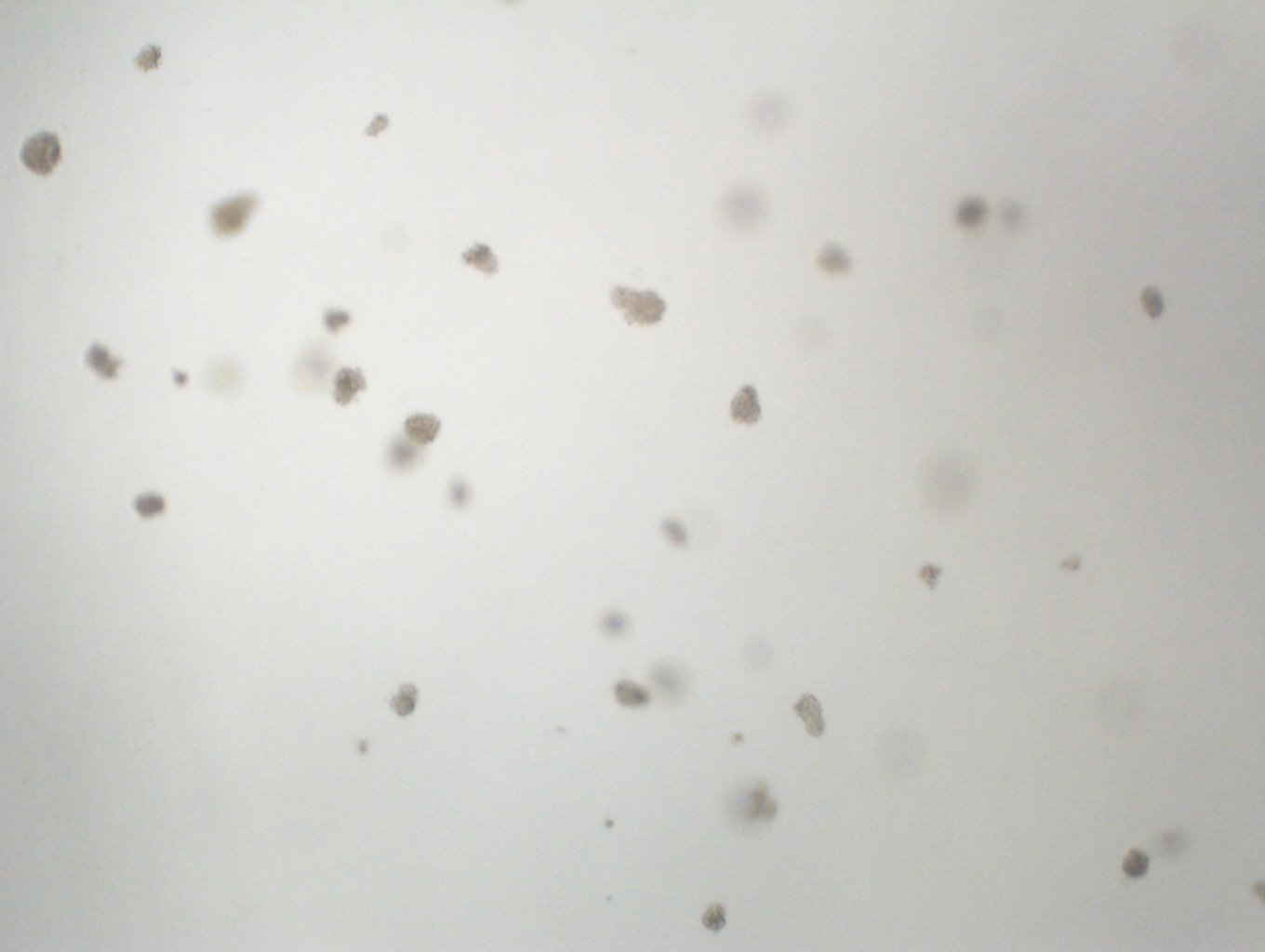

Supplement: Supplementary file 3 — Source data Fig. 2 [file 44318_2025_363_MOESM3_ESM.zip › Figure 2/2I/Control (5).jpg]

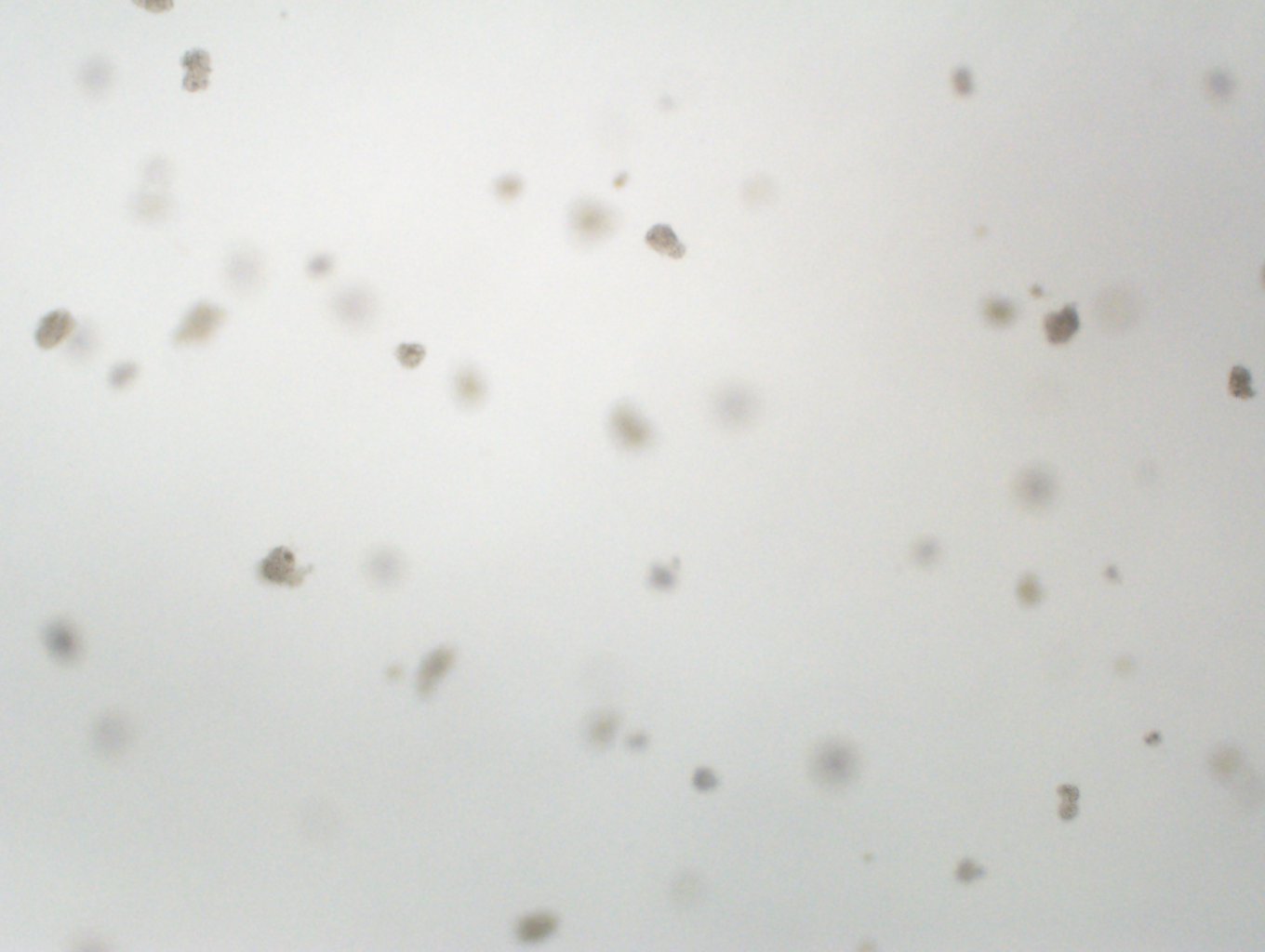

Supplement: Supplementary file 3 — Source data Fig. 2 [file 44318_2025_363_MOESM3_ESM.zip › Figure 2/2I/Control (6).jpg]

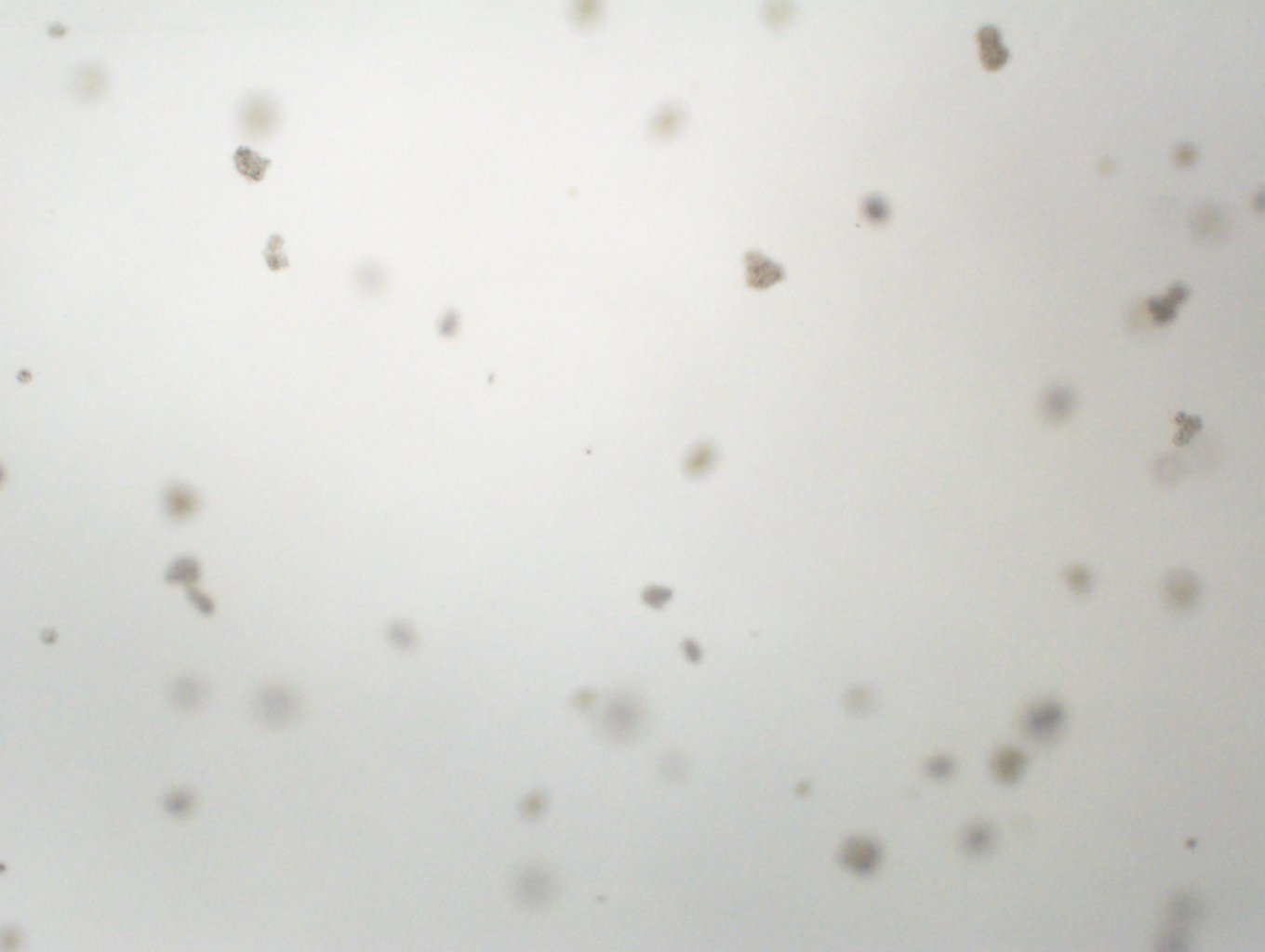

Supplement: Supplementary file 3 — Source data Fig. 2 [file 44318_2025_363_MOESM3_ESM.zip › Figure 2/2I/Control (7).jpg]

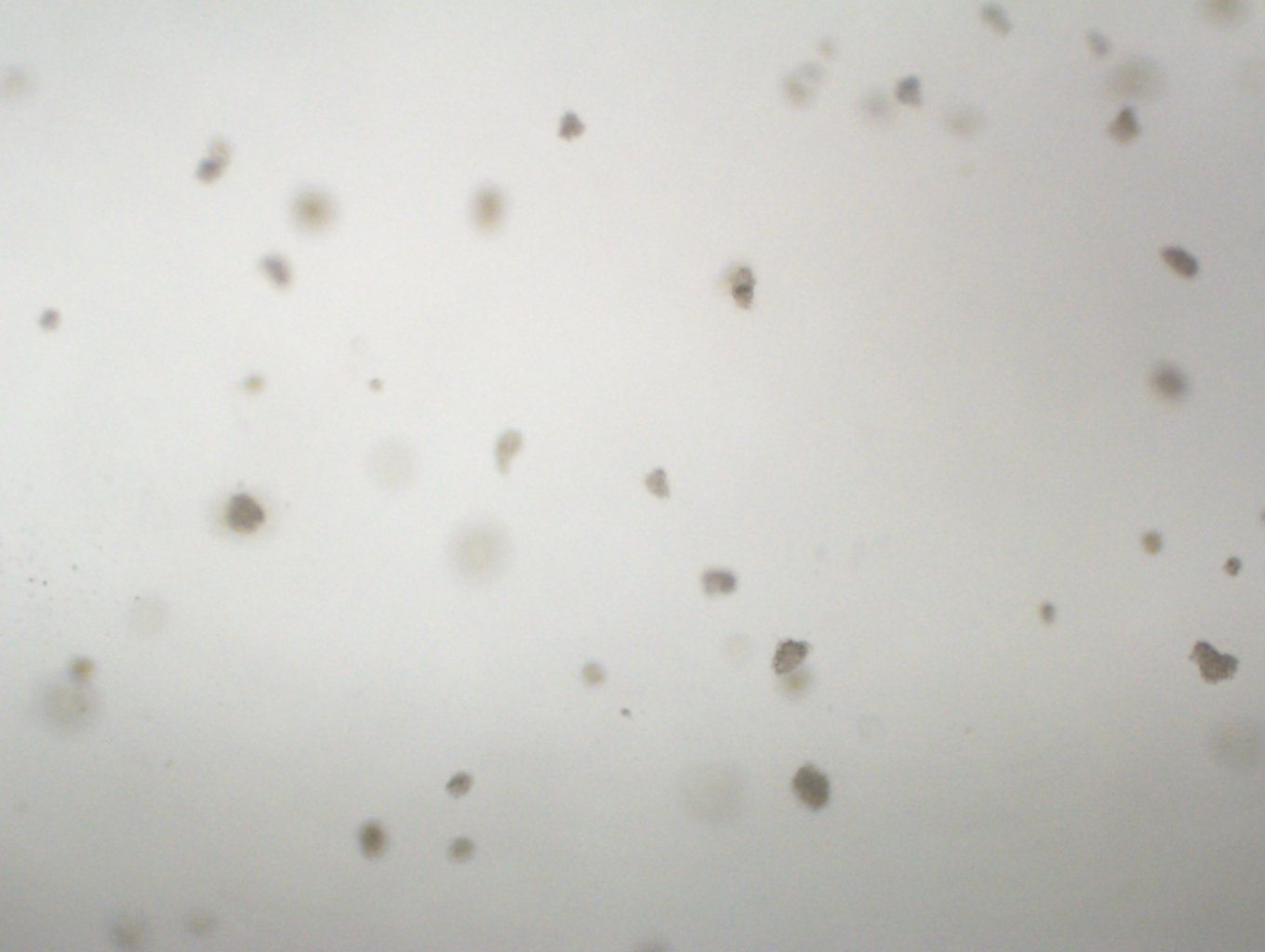

Supplement: Supplementary file 3 — Source data Fig. 2 [file 44318_2025_363_MOESM3_ESM.zip › Figure 2/2I/Control (8)-displayed in 2I.jpg]

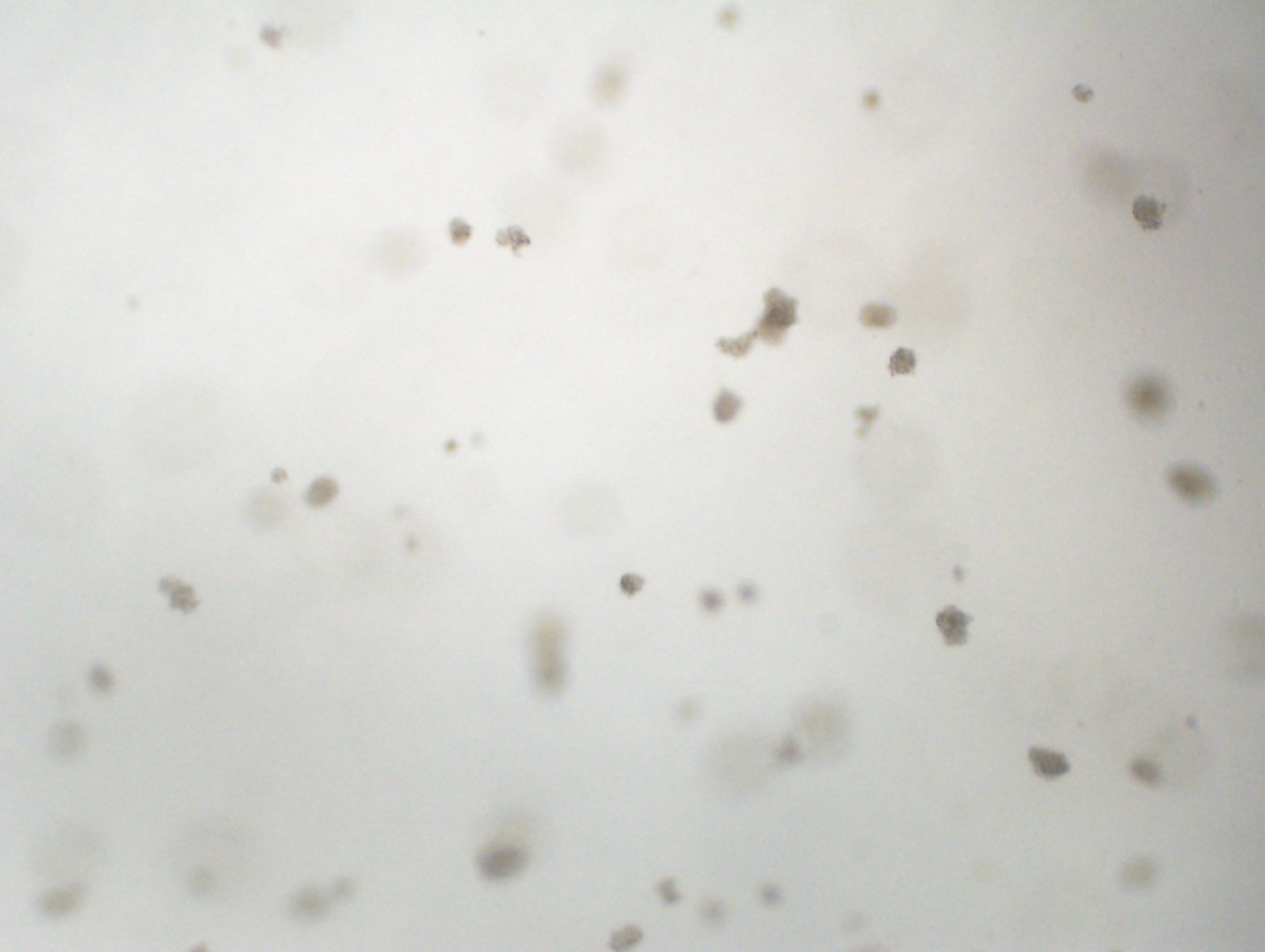

Supplement: Supplementary file 3 — Source data Fig. 2 [file 44318_2025_363_MOESM3_ESM.zip › Figure 2/2I/Control (9).jpg]

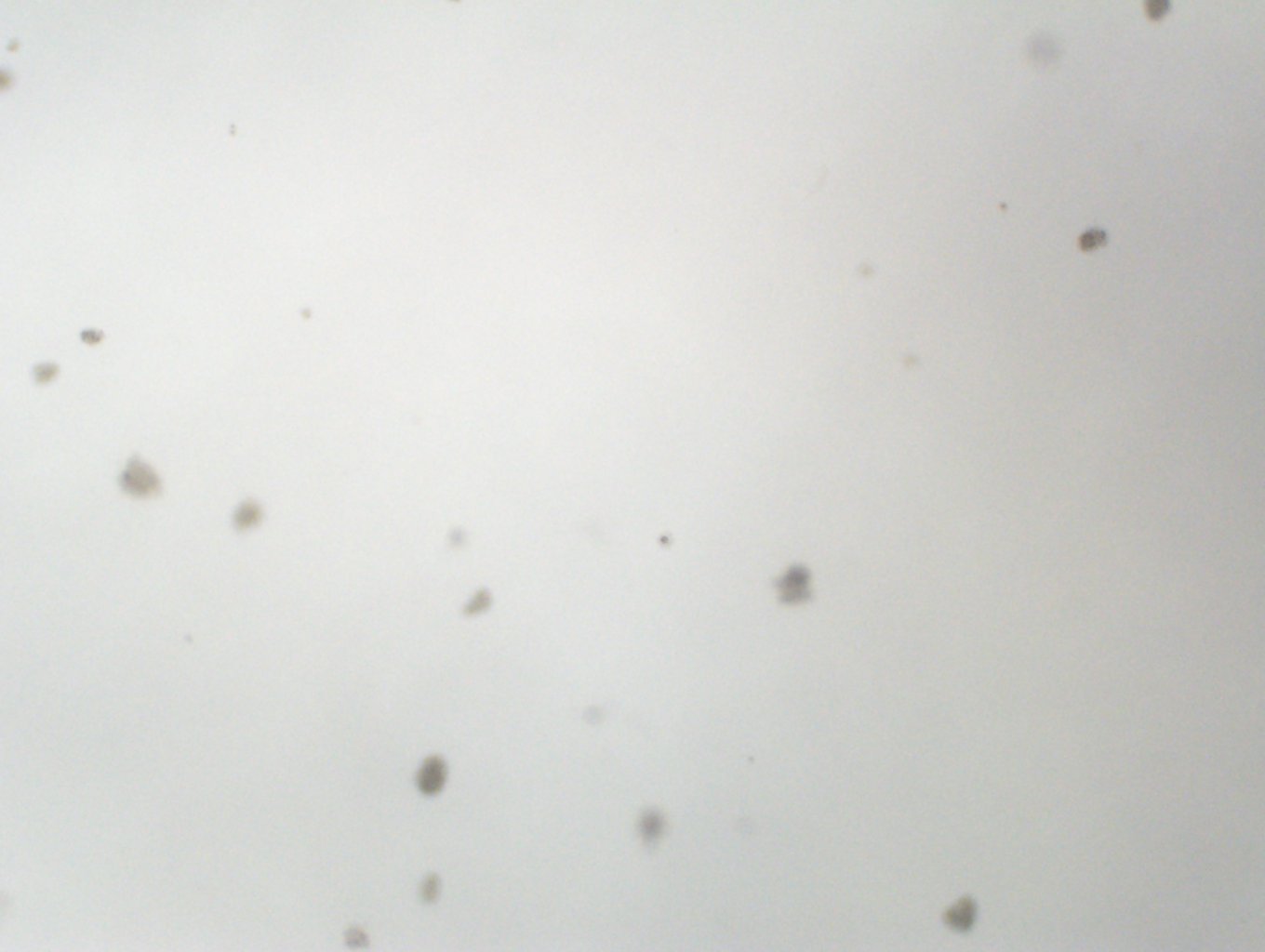

Supplement: Supplementary file 3 — Source data Fig. 2 [file 44318_2025_363_MOESM3_ESM.zip › Figure 2/2I/siEphrin A1-1 (1).jpg]

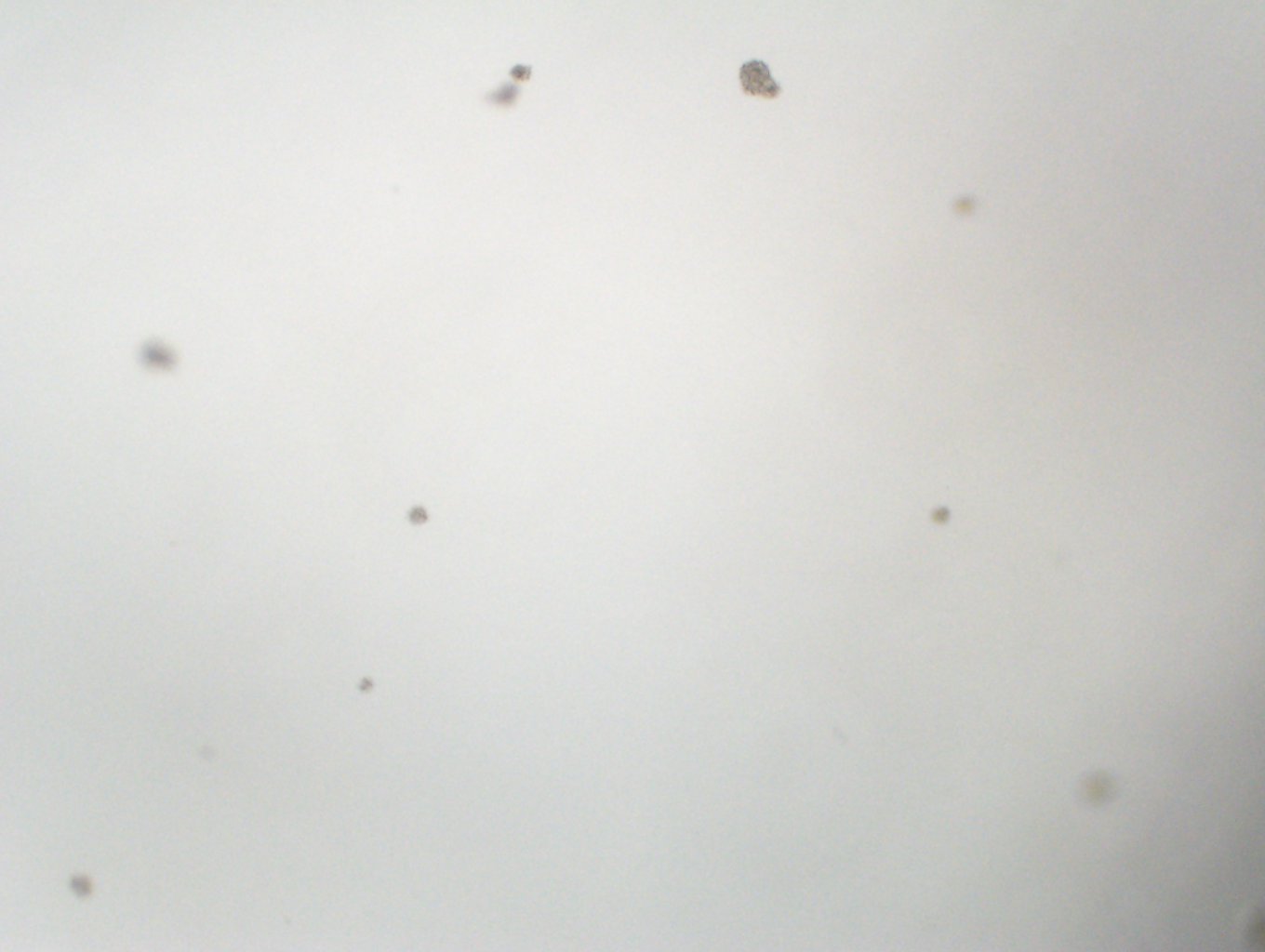

Supplement: Supplementary file 3 — Source data Fig. 2 [file 44318_2025_363_MOESM3_ESM.zip › Figure 2/2I/siEphrin A1-1 (10).jpg]

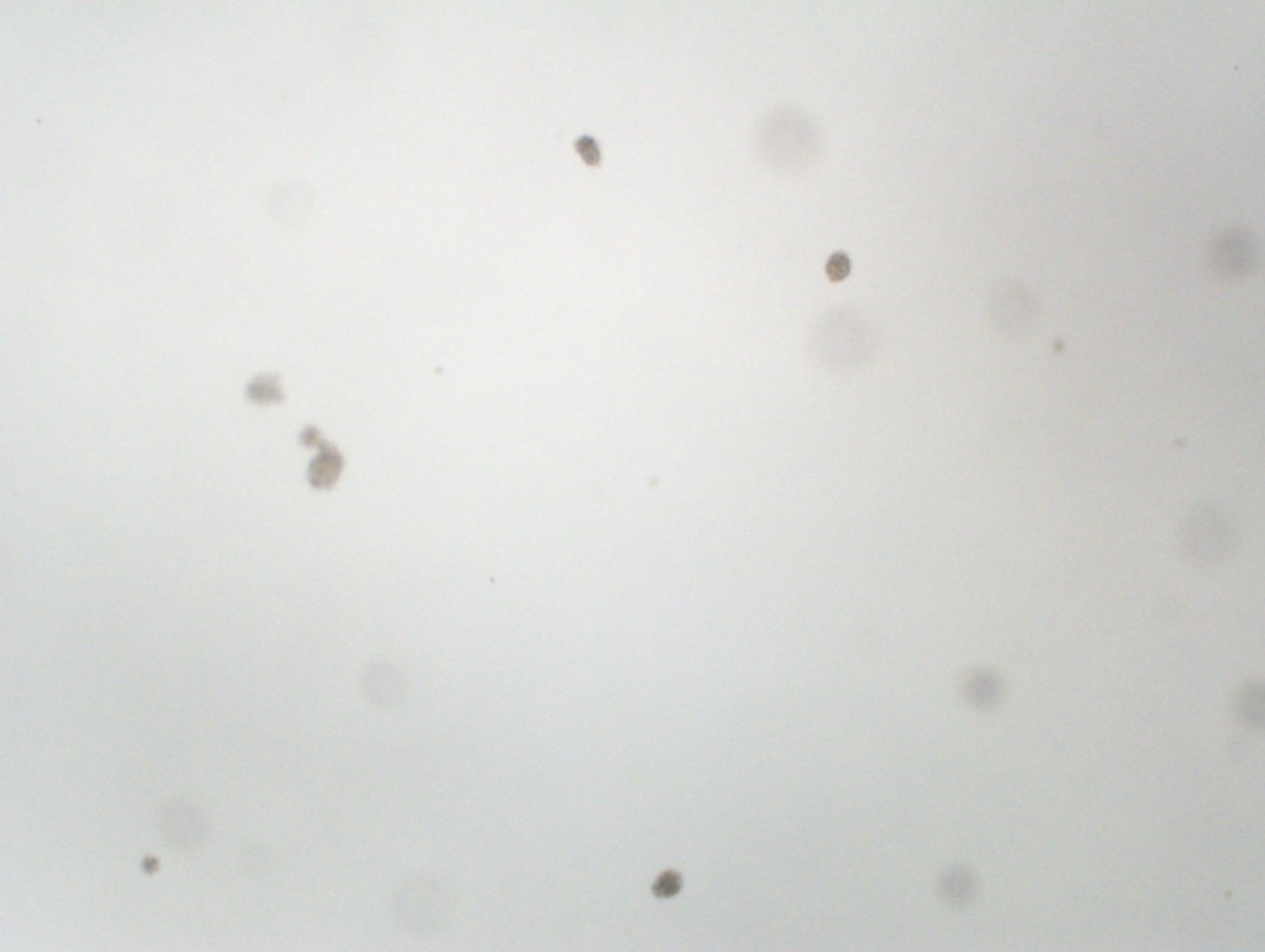

Supplement: Supplementary file 3 — Source data Fig. 2 [file 44318_2025_363_MOESM3_ESM.zip › Figure 2/2I/siEphrin A1-1 (2).jpg]

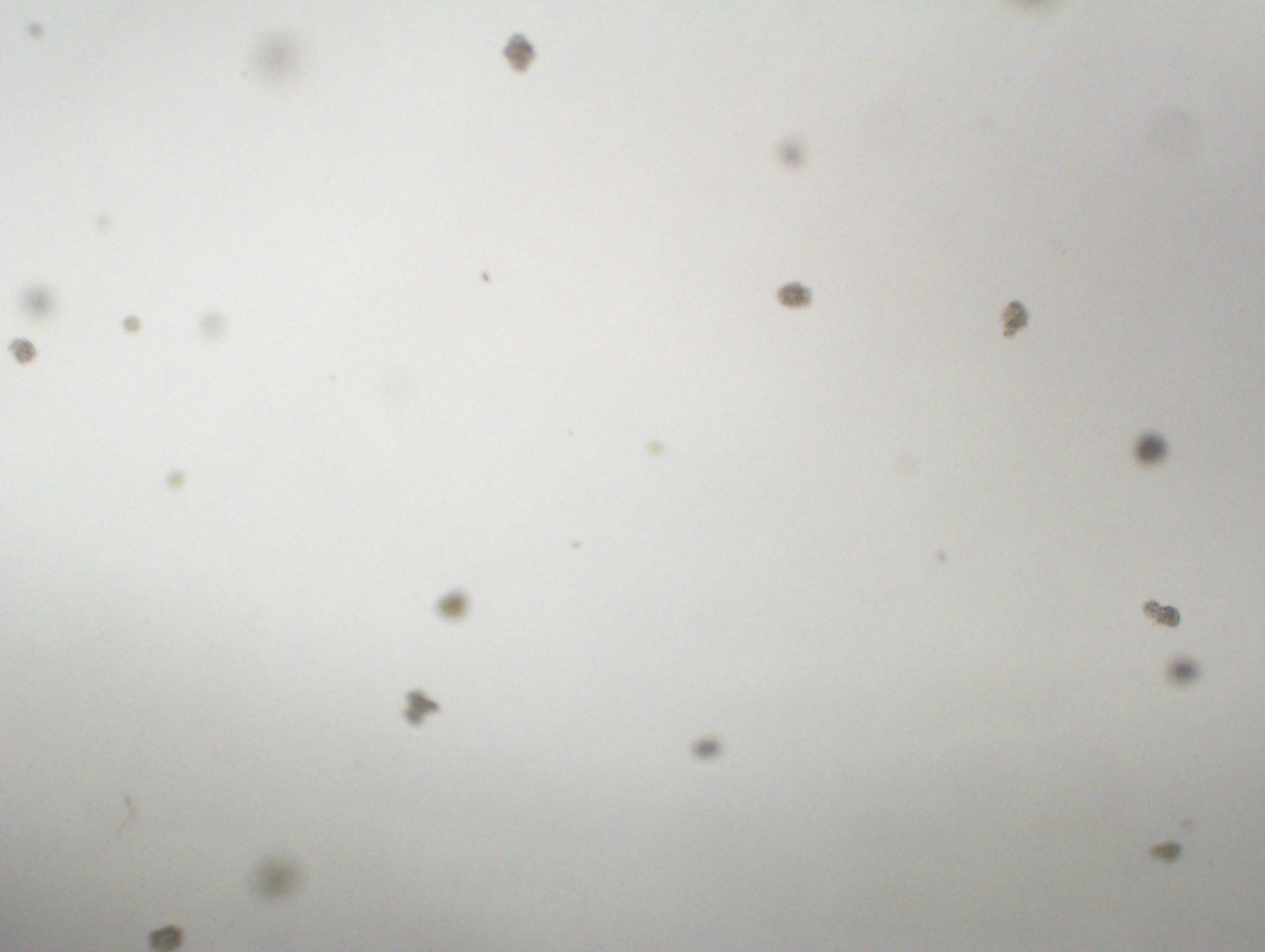

Supplement: Supplementary file 3 — Source data Fig. 2 [file 44318_2025_363_MOESM3_ESM.zip › Figure 2/2I/siEphrin A1-1 (3)-displayed in 2I.jpg]

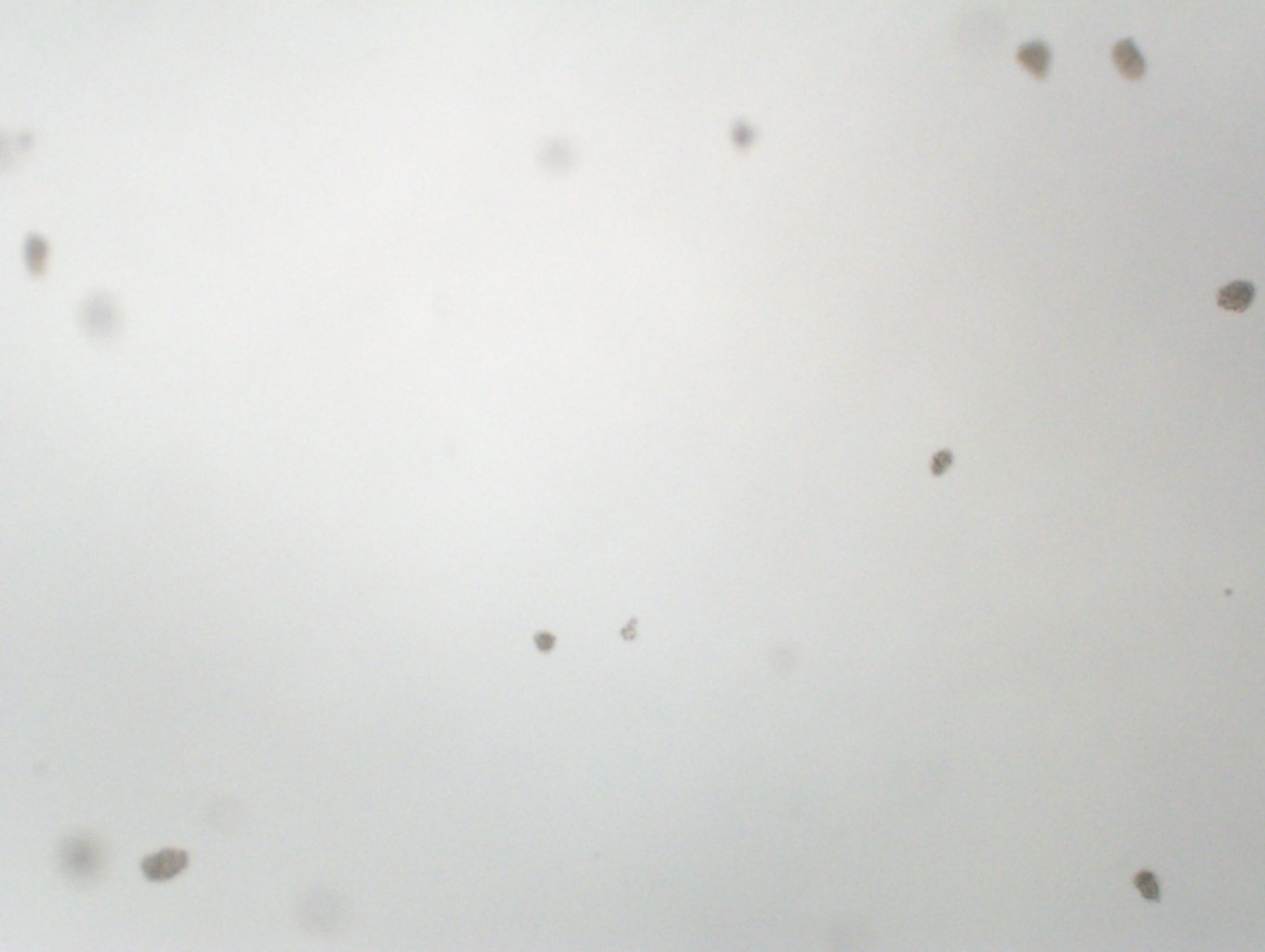

Supplement: Supplementary file 3 — Source data Fig. 2 [file 44318_2025_363_MOESM3_ESM.zip › Figure 2/2I/siEphrin A1-1 (4).jpg]

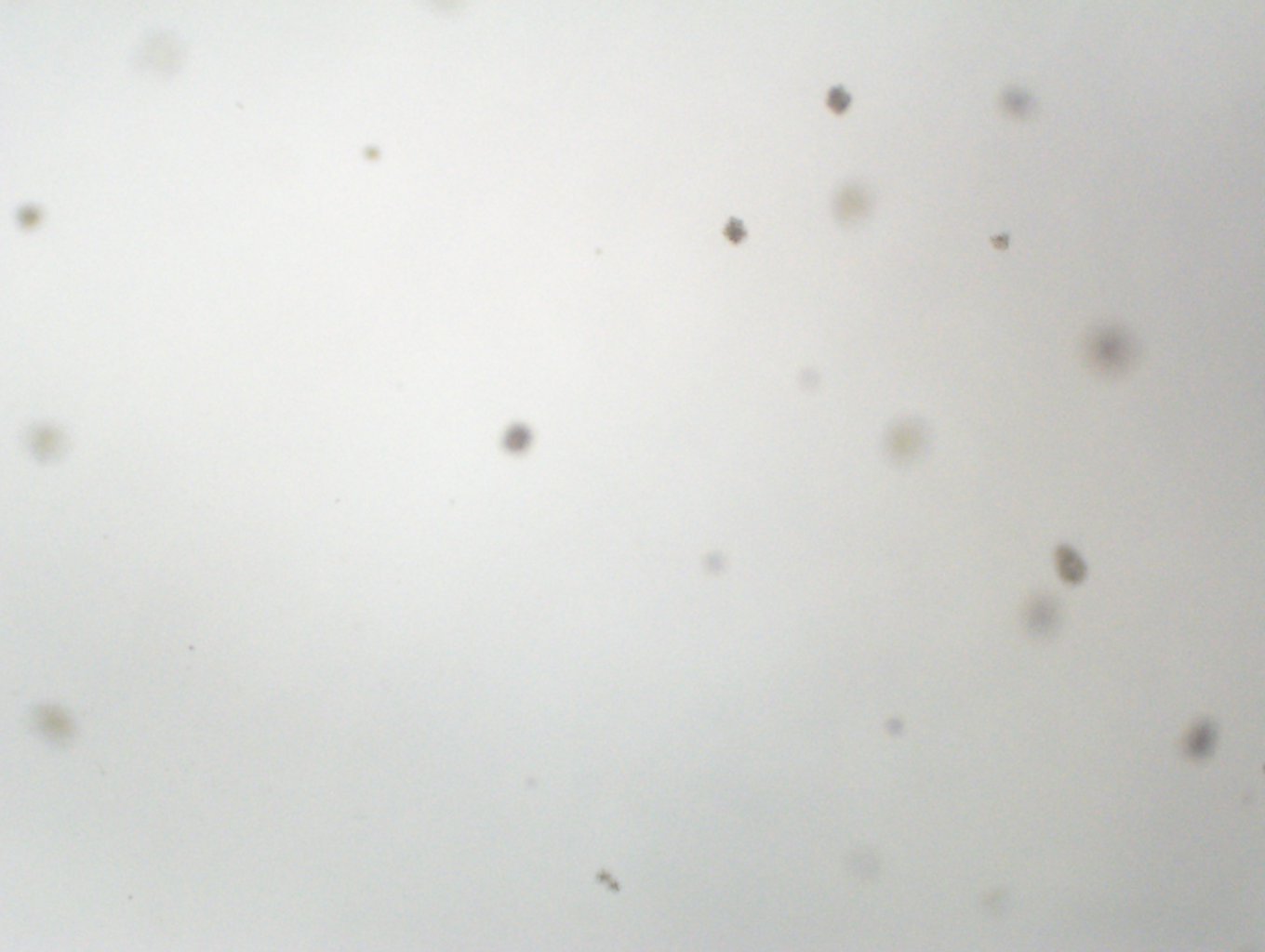

Supplement: Supplementary file 3 — Source data Fig. 2 [file 44318_2025_363_MOESM3_ESM.zip › Figure 2/2I/siEphrin A1-1 (5).jpg]

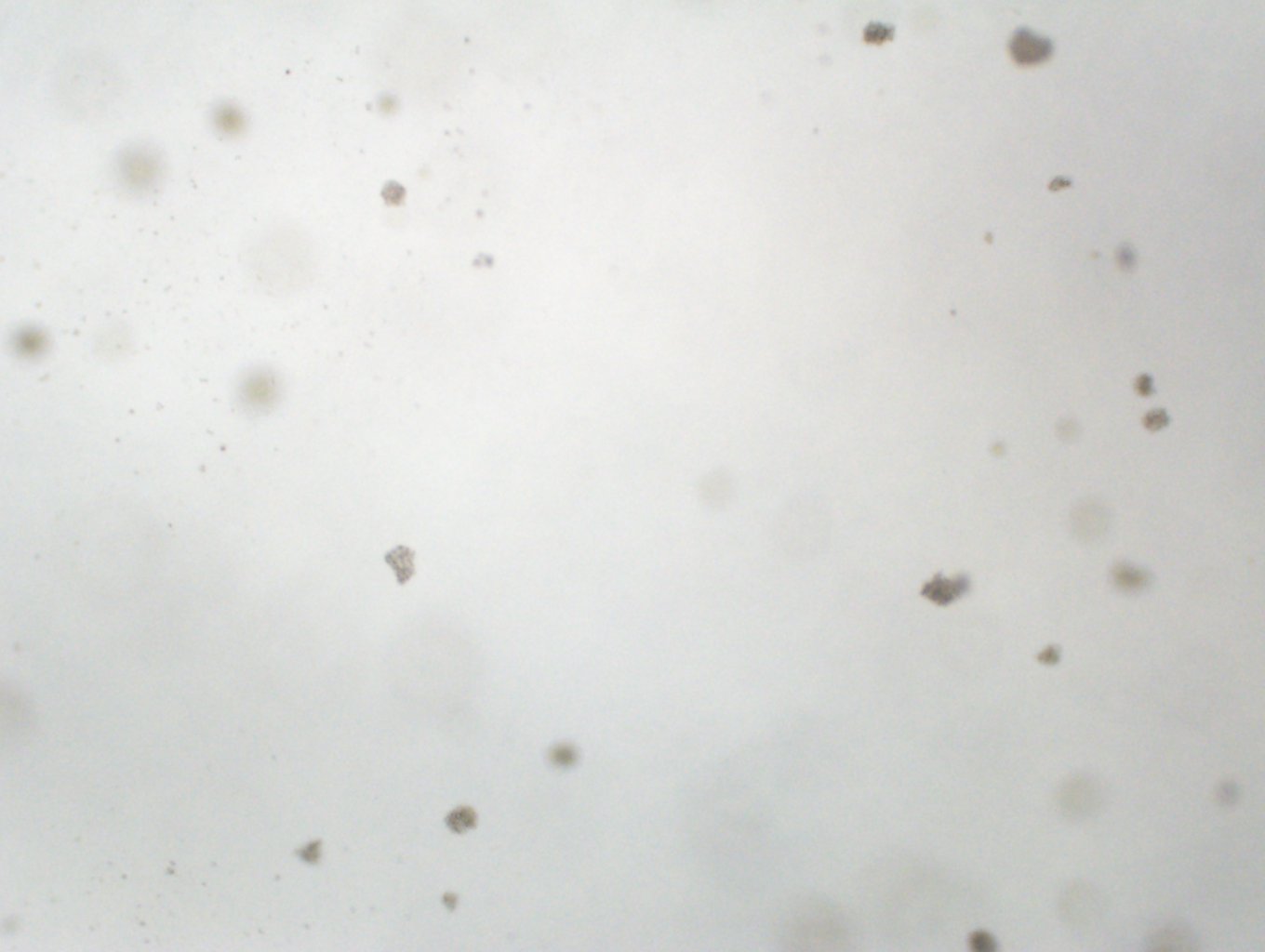

Supplement: Supplementary file 3 — Source data Fig. 2 [file 44318_2025_363_MOESM3_ESM.zip › Figure 2/2I/siEphrin A1-1 (6).jpg]

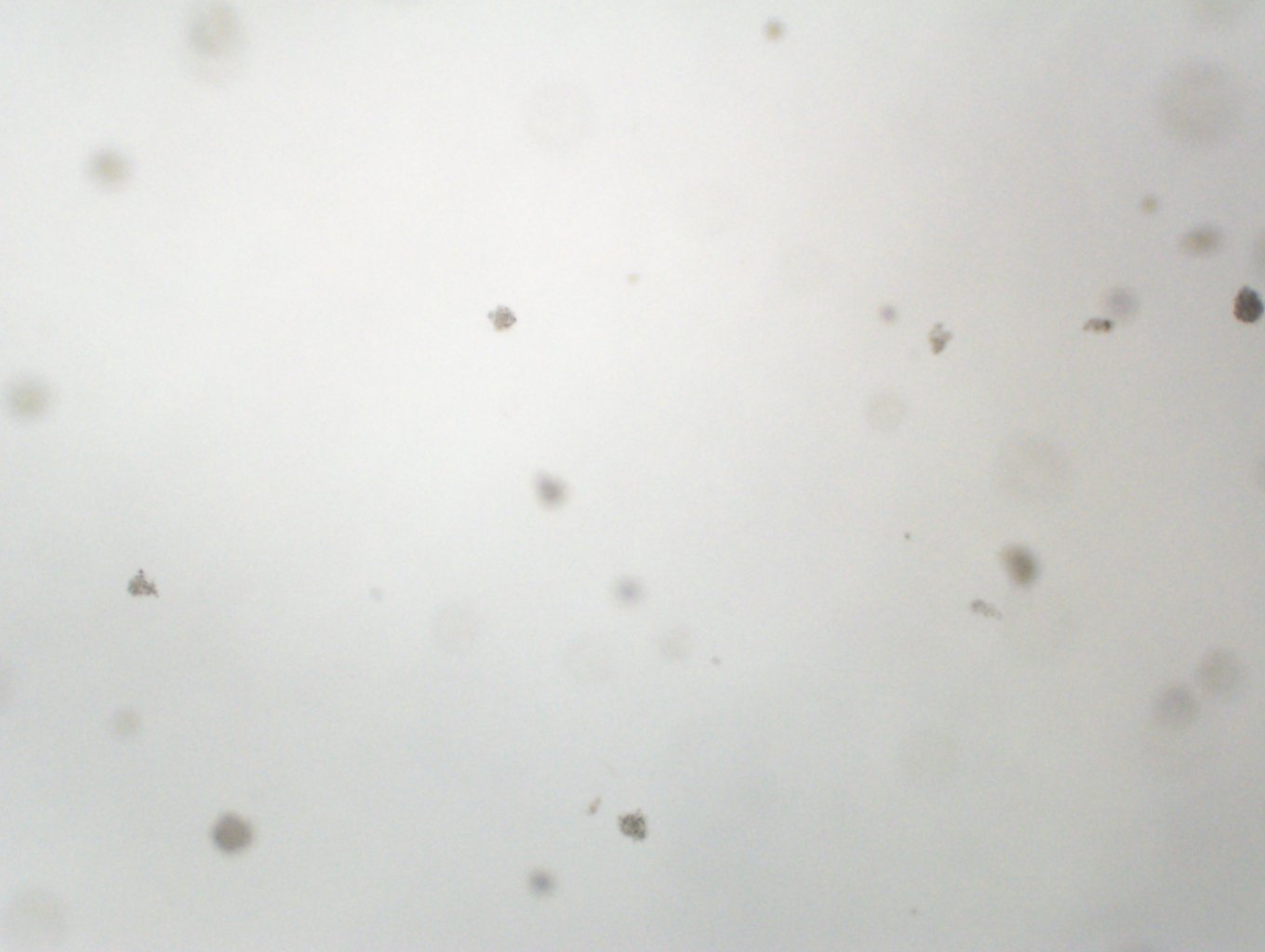

Supplement: Supplementary file 3 — Source data Fig. 2 [file 44318_2025_363_MOESM3_ESM.zip › Figure 2/2I/siEphrin A1-1 (7).jpg]

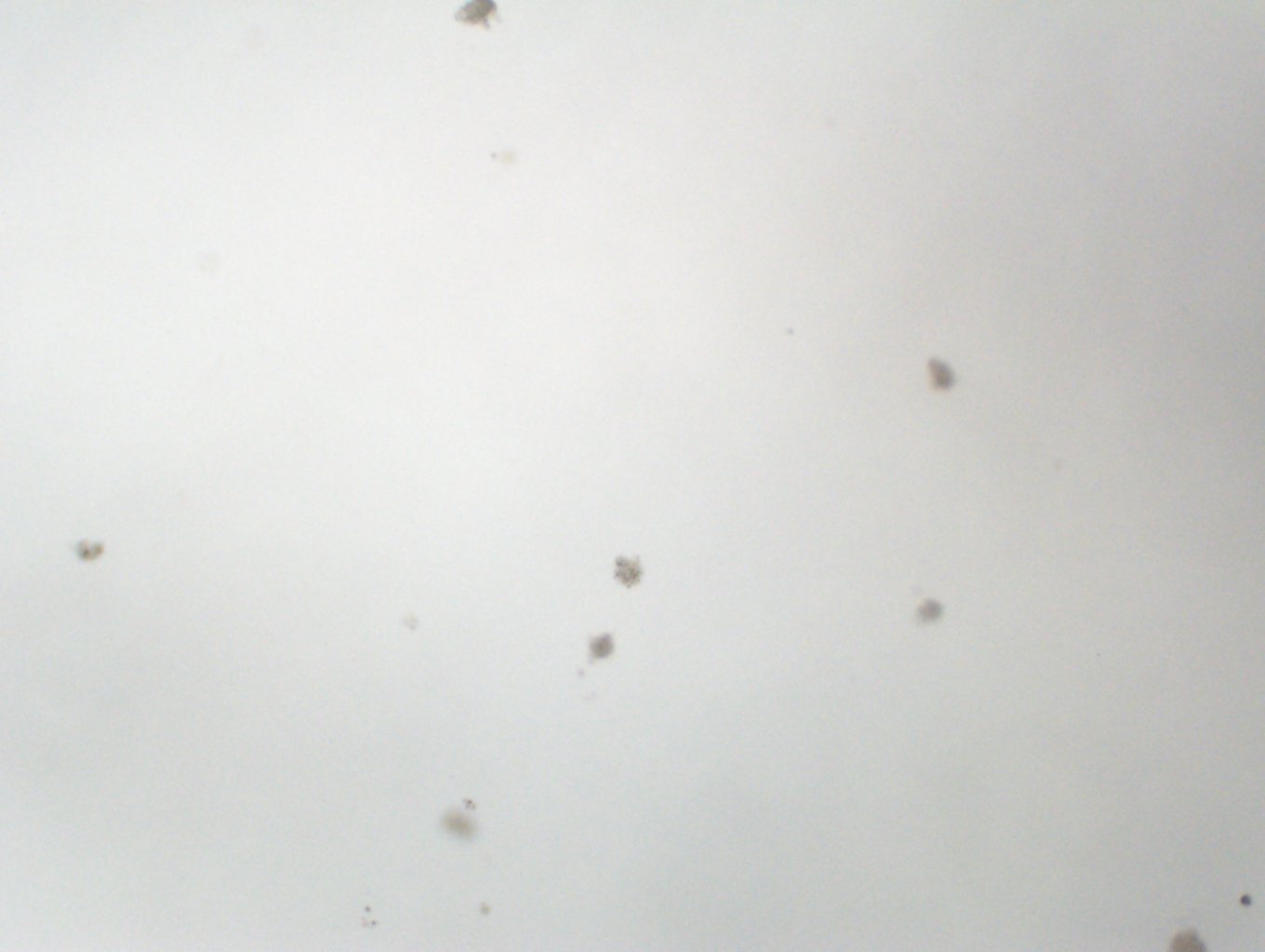

Supplement: Supplementary file 3 — Source data Fig. 2 [file 44318_2025_363_MOESM3_ESM.zip › Figure 2/2I/siEphrin A1-1 (8).jpg]

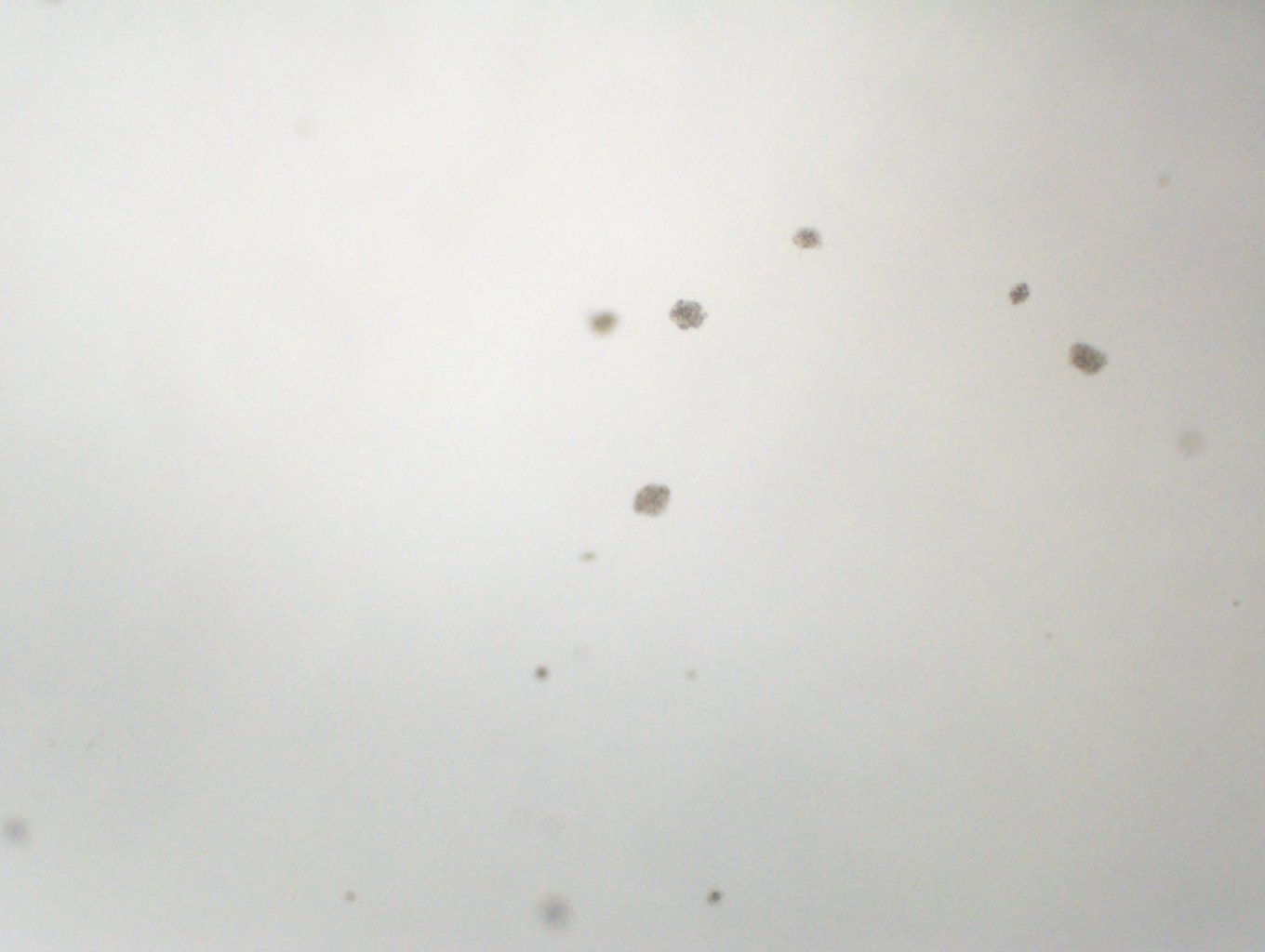

Supplement: Supplementary file 3 — Source data Fig. 2 [file 44318_2025_363_MOESM3_ESM.zip › Figure 2/2I/siEphrin A1-1 (9).jpg]

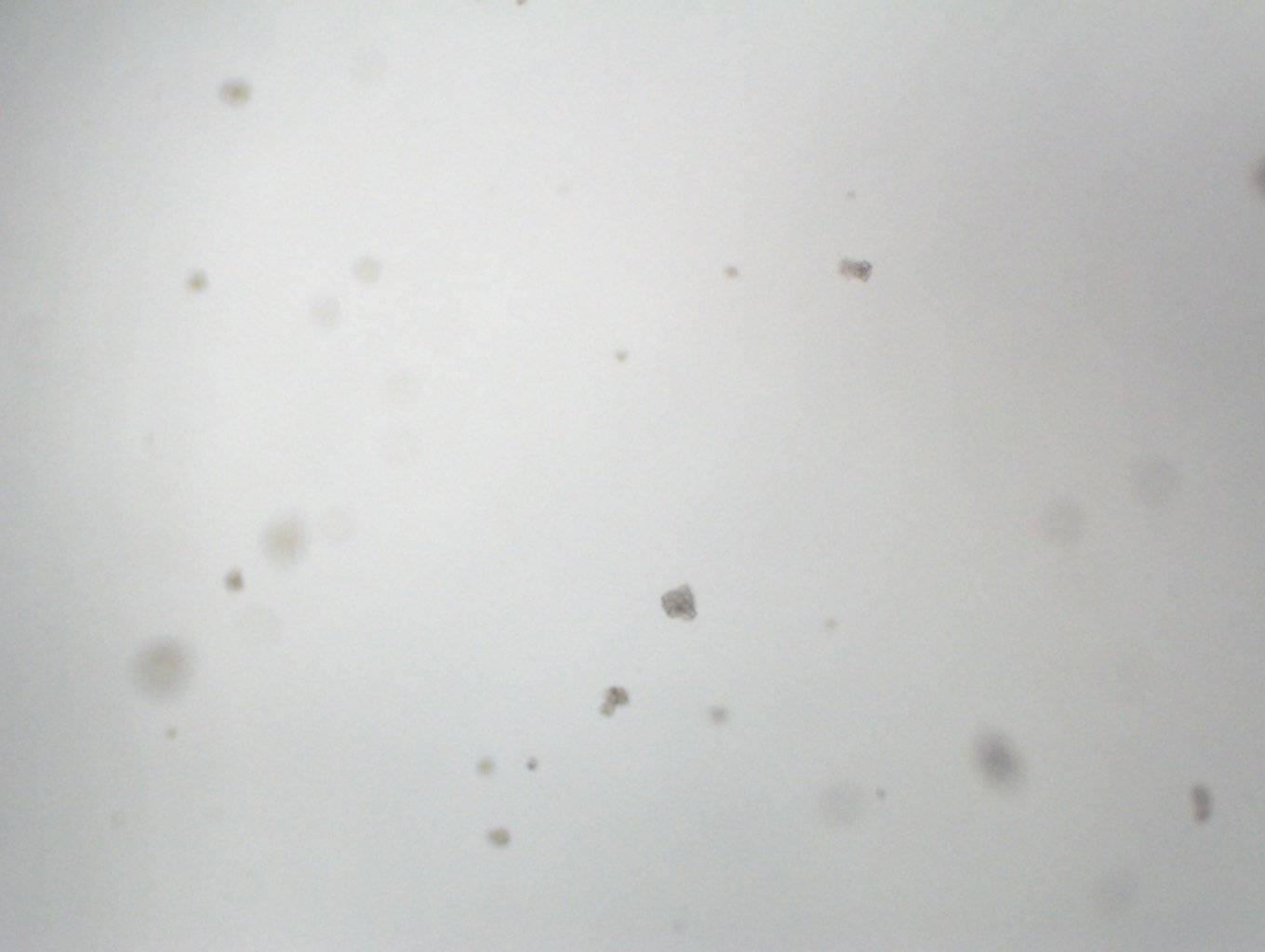

Supplement: Supplementary file 3 — Source data Fig. 2 [file 44318_2025_363_MOESM3_ESM.zip › Figure 2/2I/siEphrin A1-2 (1)-displayed in 2I.jpg]

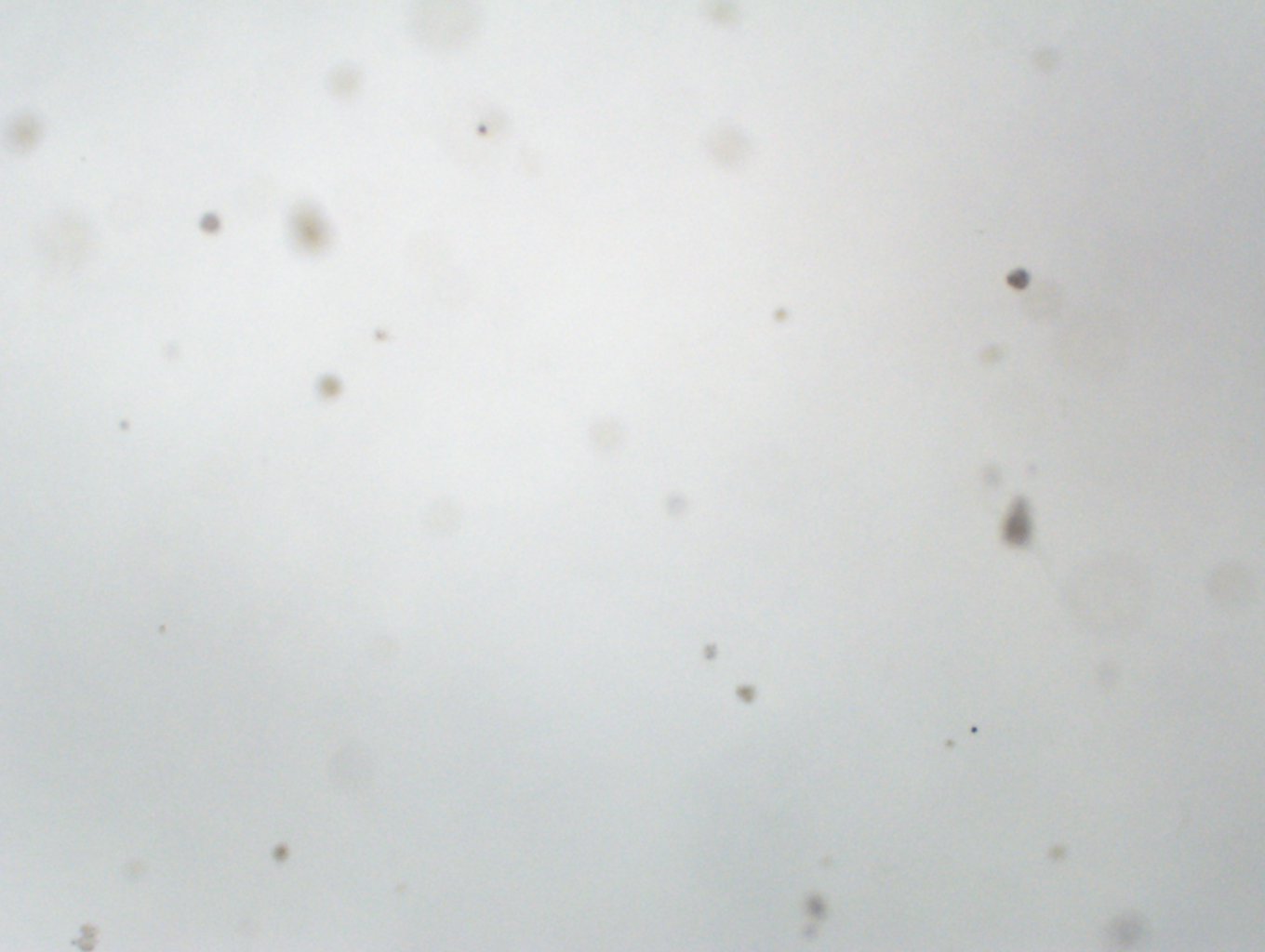

Supplement: Supplementary file 3 — Source data Fig. 2 [file 44318_2025_363_MOESM3_ESM.zip › Figure 2/2I/siEphrin A1-2 (10).jpg]

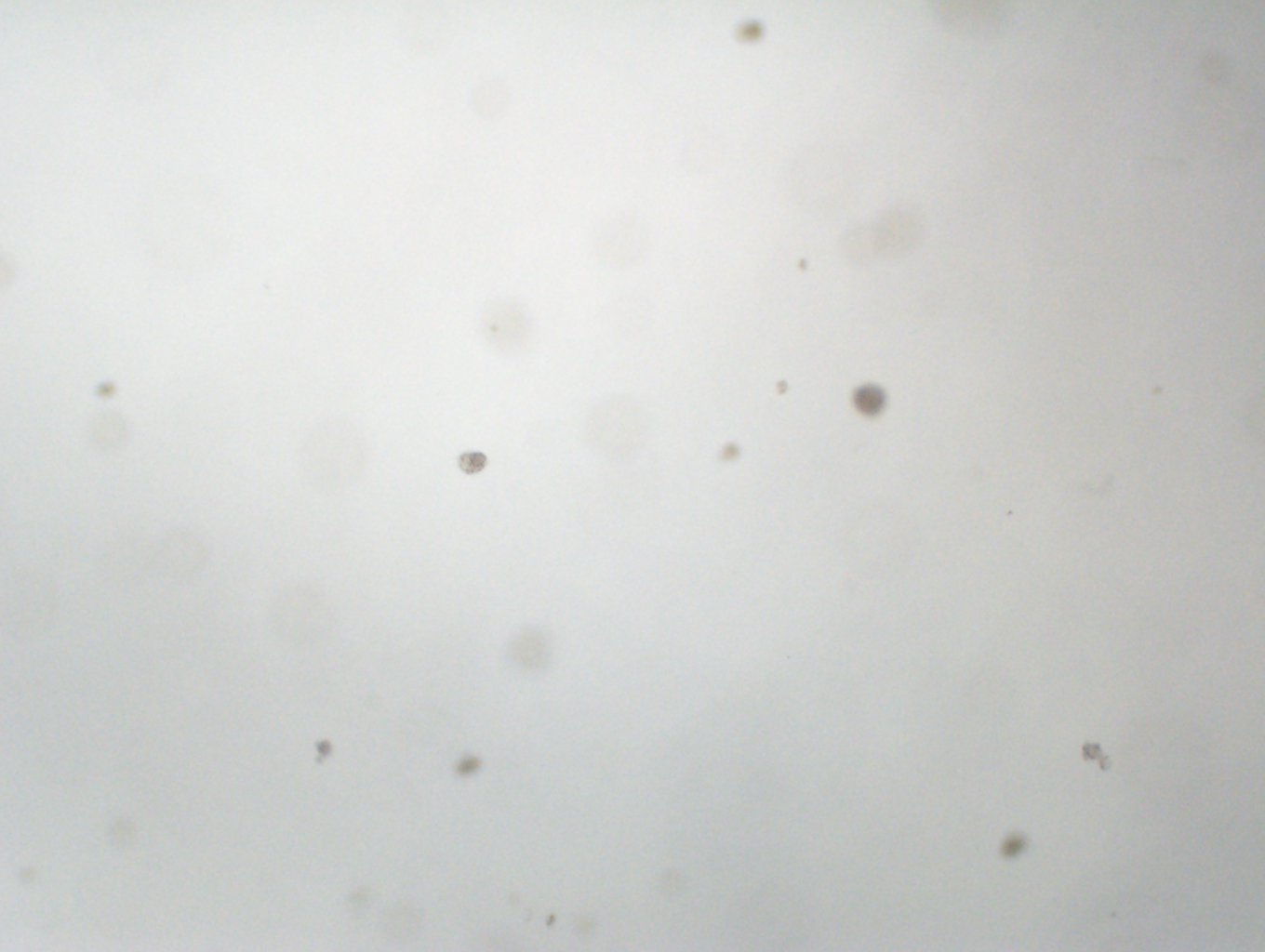

Supplement: Supplementary file 3 — Source data Fig. 2 [file 44318_2025_363_MOESM3_ESM.zip › Figure 2/2I/siEphrin A1-2 (2).jpg]

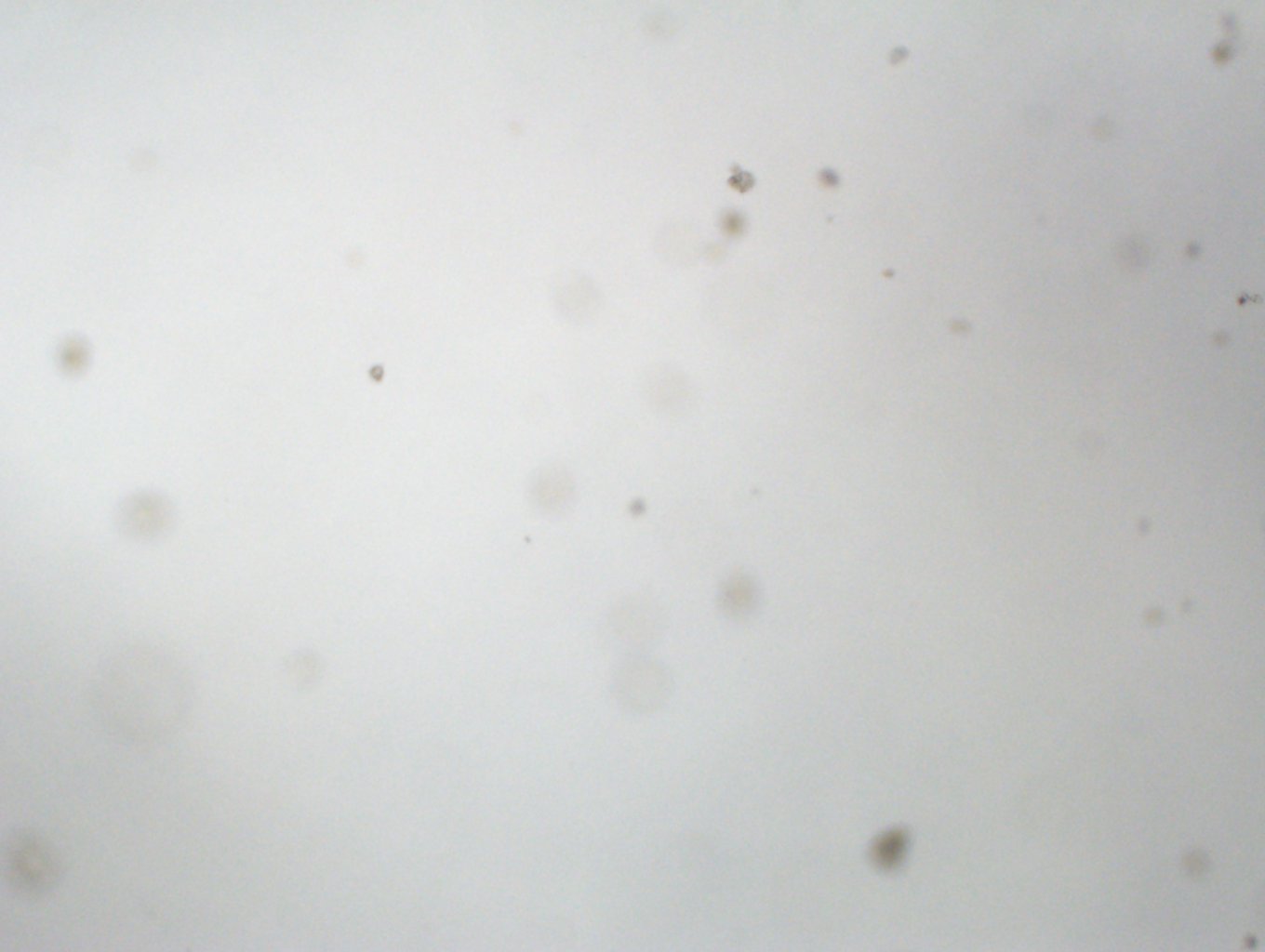

Supplement: Supplementary file 3 — Source data Fig. 2 [file 44318_2025_363_MOESM3_ESM.zip › Figure 2/2I/siEphrin A1-2 (3).jpg]

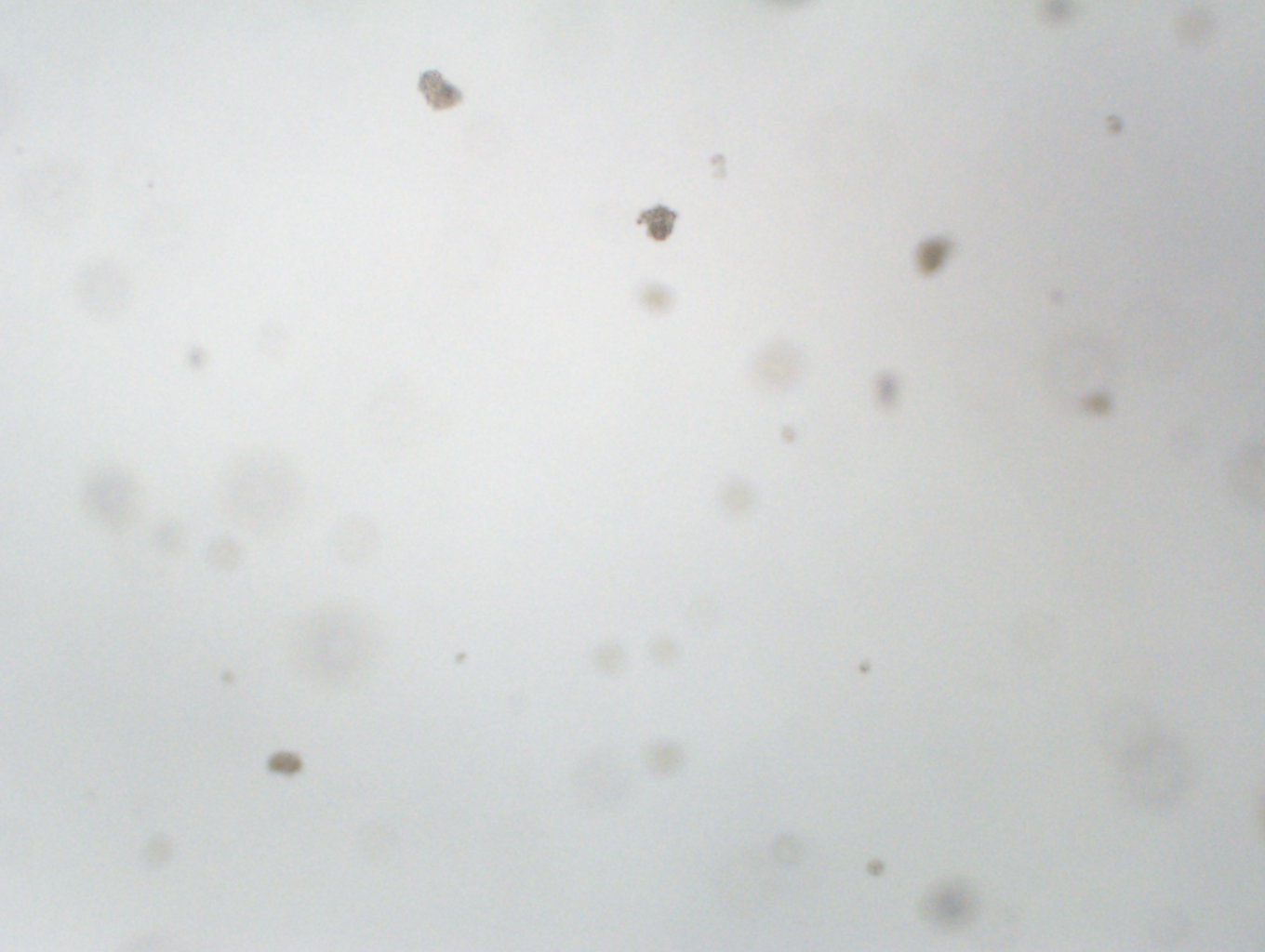

Supplement: Supplementary file 3 — Source data Fig. 2 [file 44318_2025_363_MOESM3_ESM.zip › Figure 2/2I/siEphrin A1-2 (4).jpg]

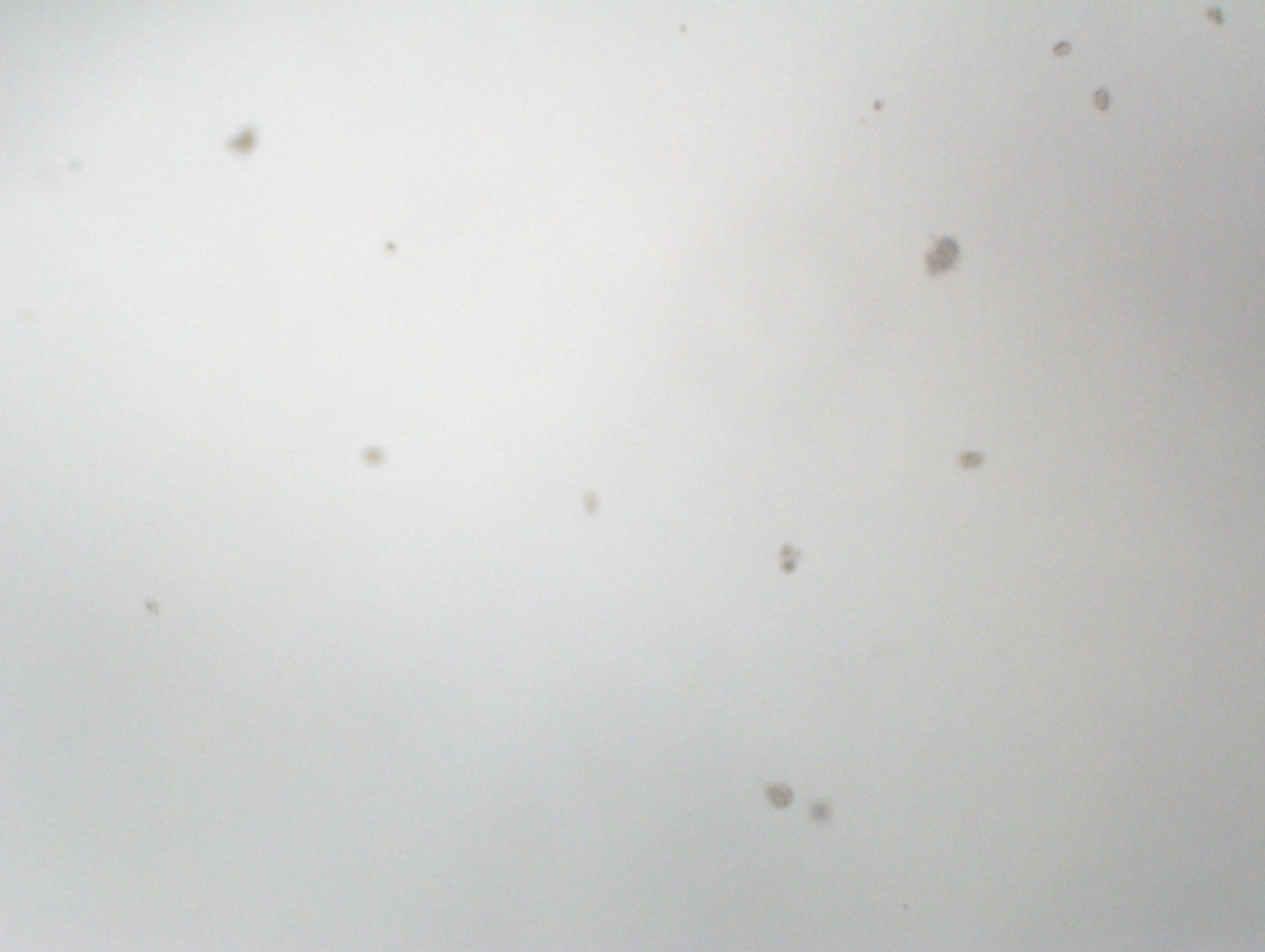

Supplement: Supplementary file 3 — Source data Fig. 2 [file 44318_2025_363_MOESM3_ESM.zip › Figure 2/2I/siEphrin A1-2 (5).jpg]

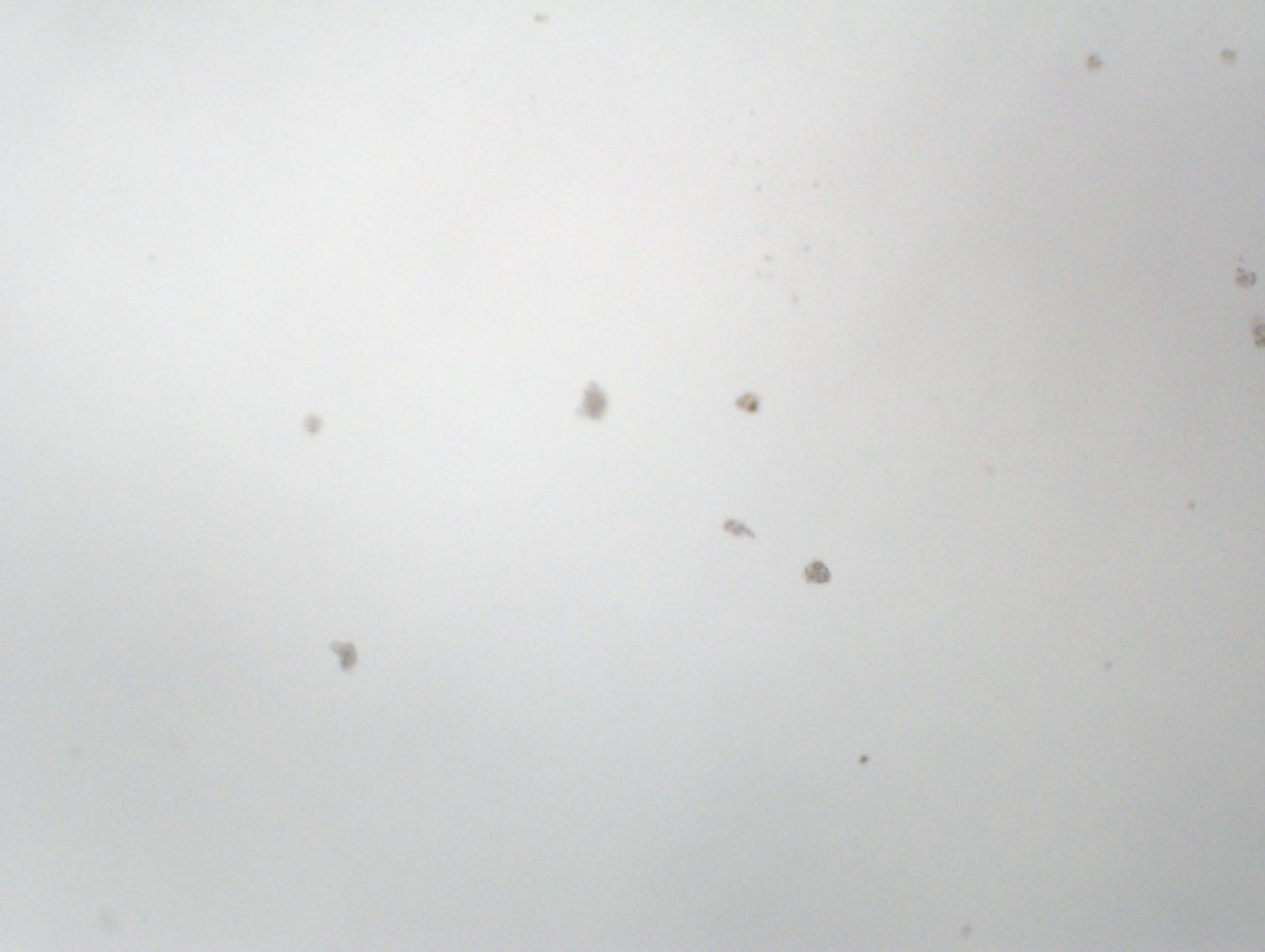

Supplement: Supplementary file 3 — Source data Fig. 2 [file 44318_2025_363_MOESM3_ESM.zip › Figure 2/2I/siEphrin A1-2 (6).jpg]

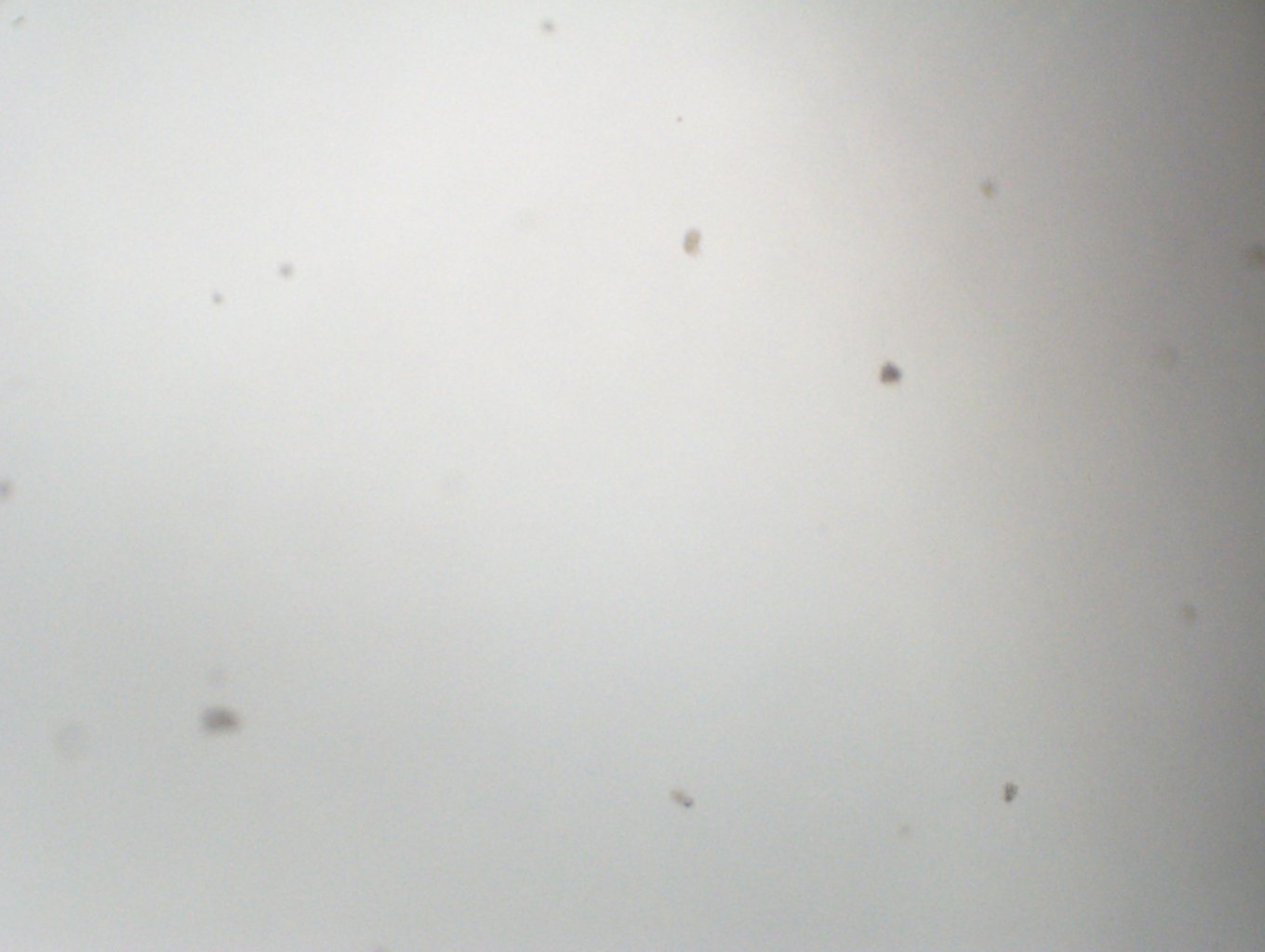

Supplement: Supplementary file 3 — Source data Fig. 2 [file 44318_2025_363_MOESM3_ESM.zip › Figure 2/2I/siEphrin A1-2 (7).jpg]

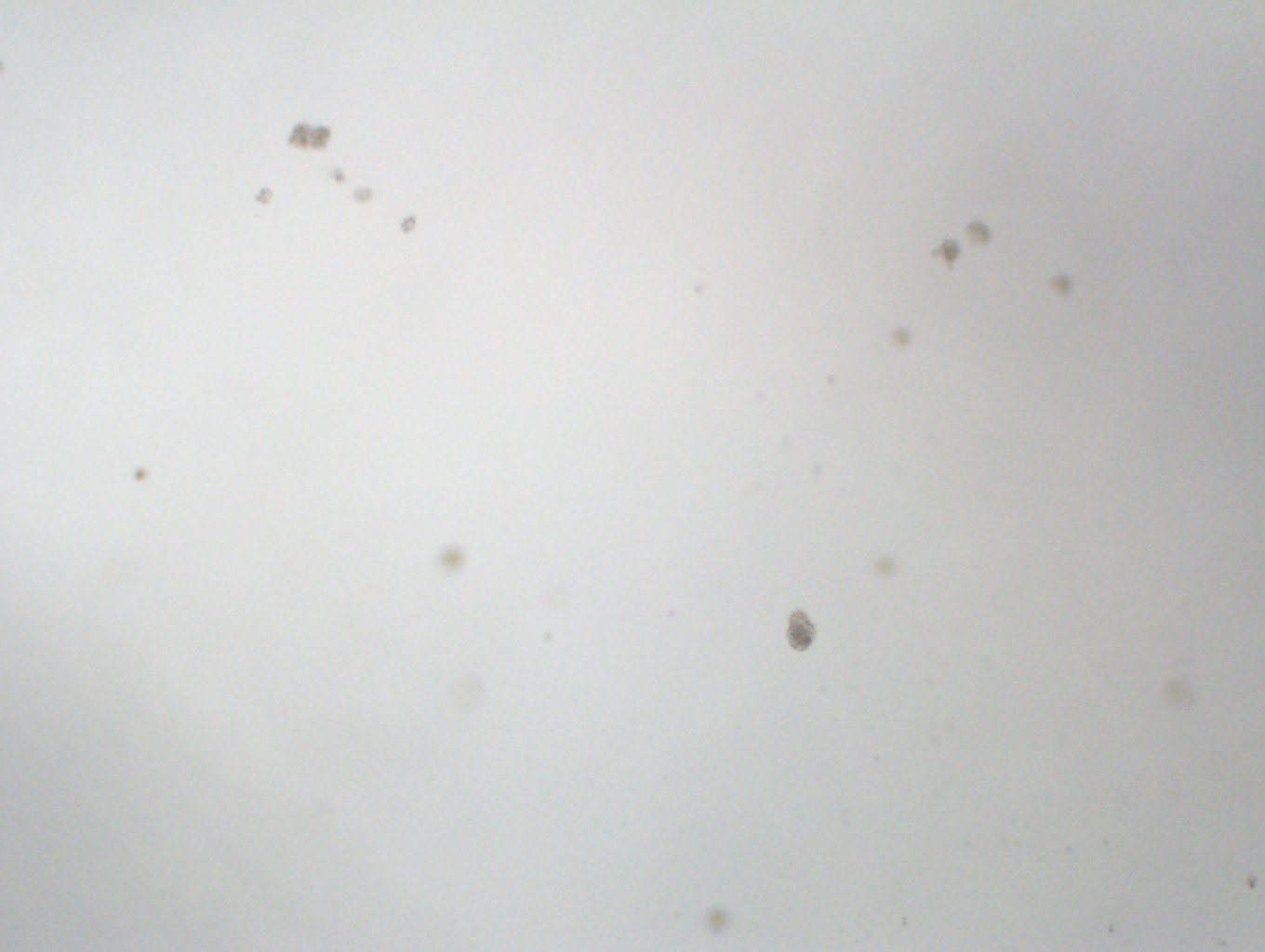

Supplement: Supplementary file 3 — Source data Fig. 2 [file 44318_2025_363_MOESM3_ESM.zip › Figure 2/2I/siEphrin A1-2 (8).jpg]

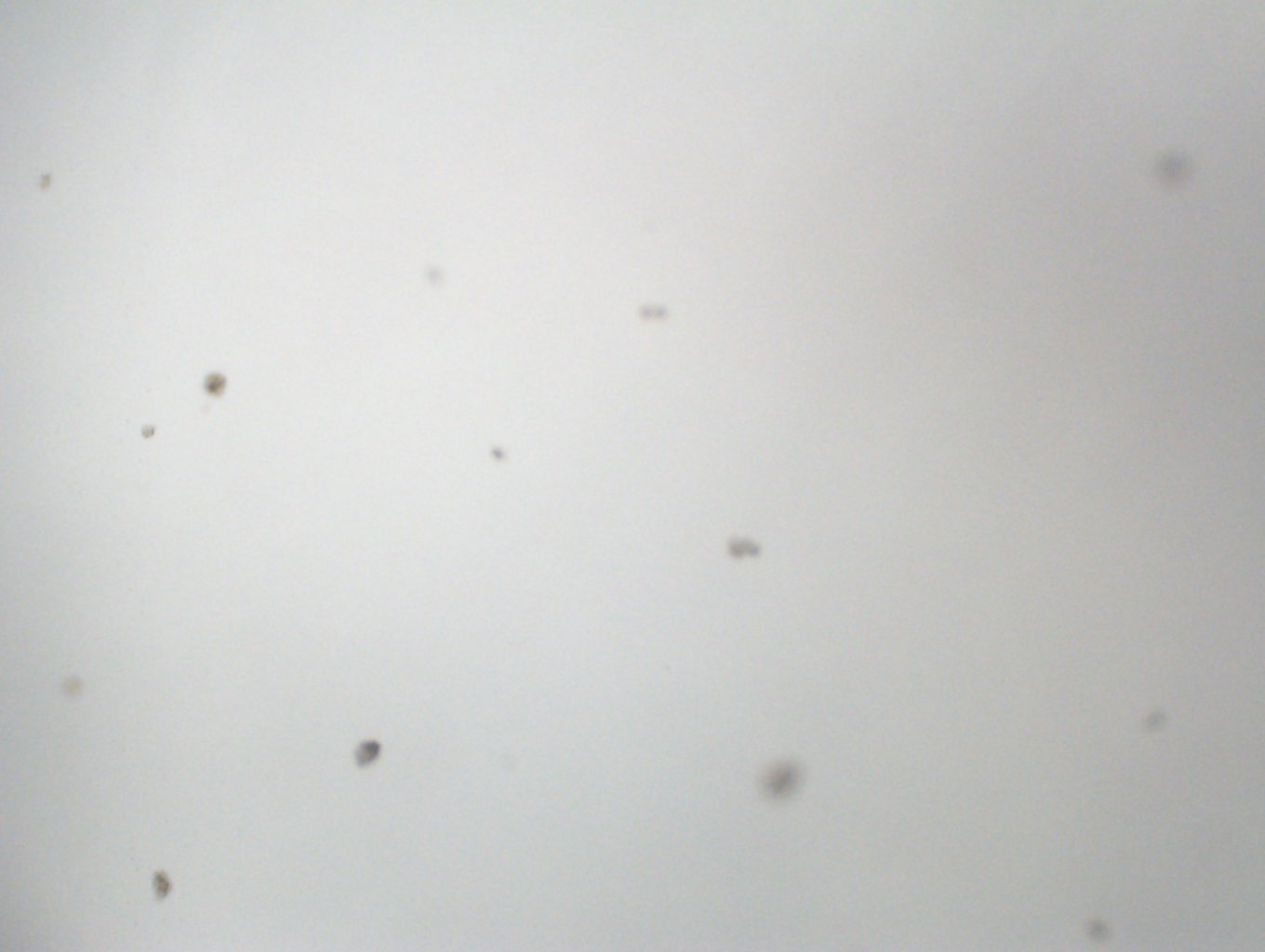

Supplement: Supplementary file 3 — Source data Fig. 2 [file 44318_2025_363_MOESM3_ESM.zip › Figure 2/2I/siEphrin A1-2 (9).jpg]

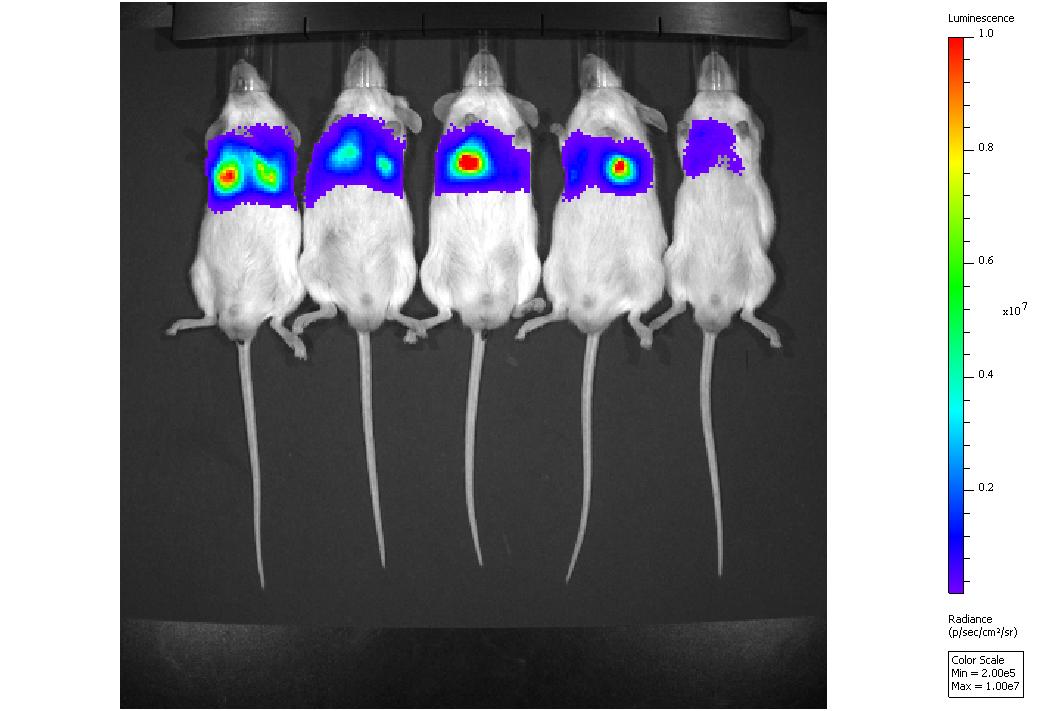

Supplement: Supplementary file 3 — Source data Fig. 2 [file 44318_2025_363_MOESM3_ESM.zip › Figure 2/2L/shControl.jpg]

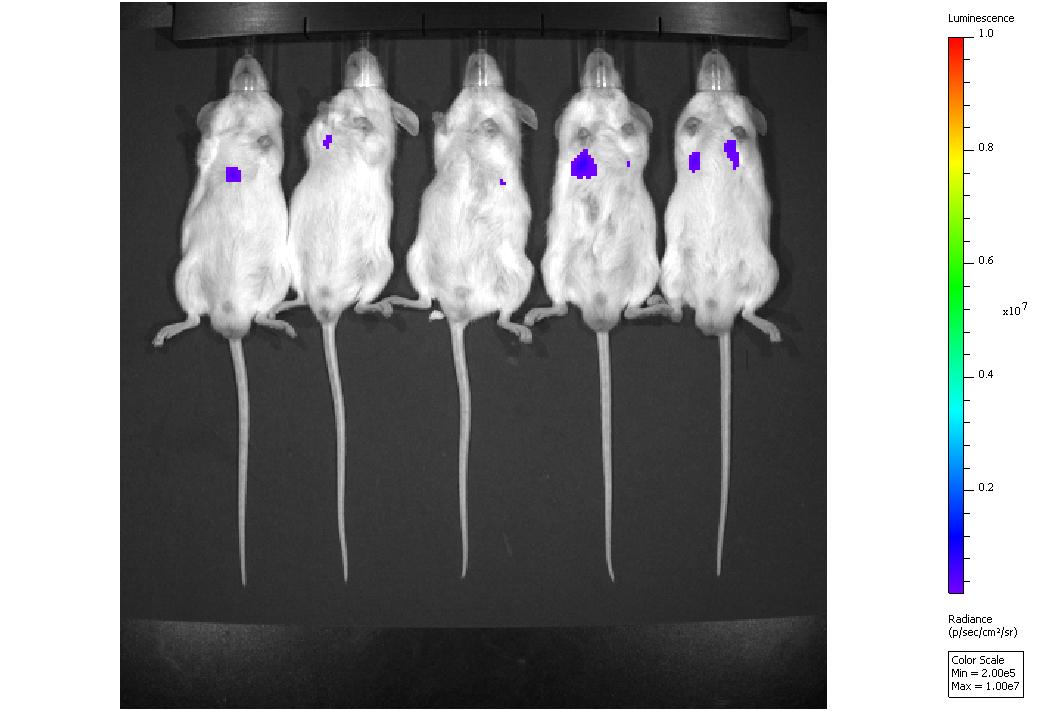

Supplement: Supplementary file 3 — Source data Fig. 2 [file 44318_2025_363_MOESM3_ESM.zip › Figure 2/2L/shEphrin A1-1.jpg]

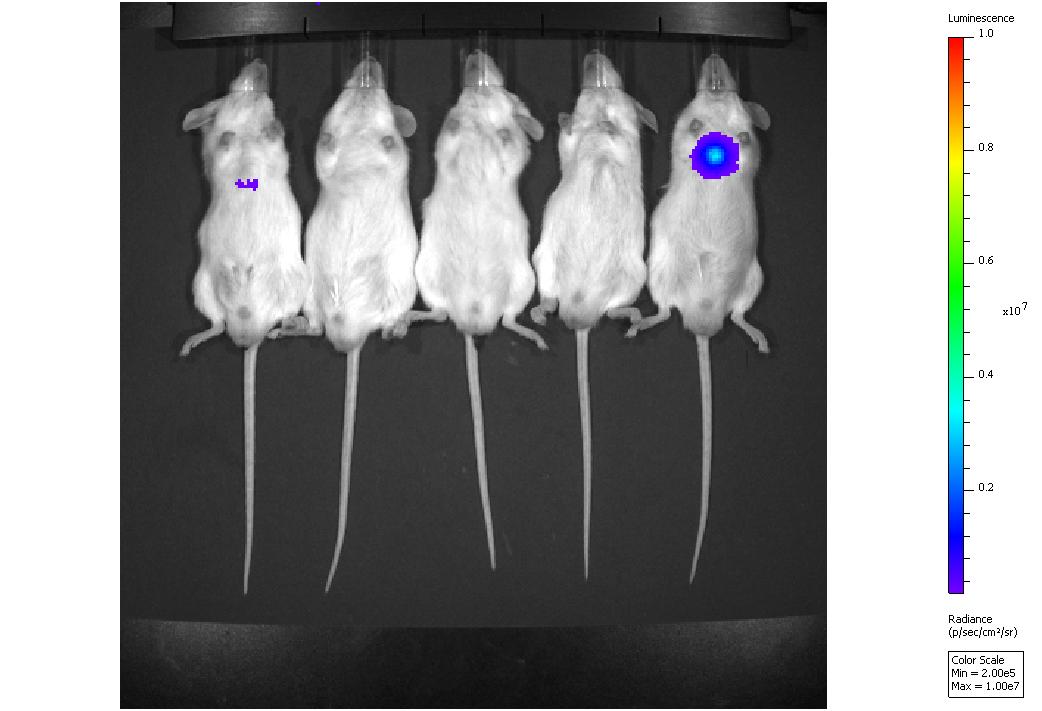

Supplement: Supplementary file 3 — Source data Fig. 2 [file 44318_2025_363_MOESM3_ESM.zip › Figure 2/2L/shEphrin A1-2.jpg]

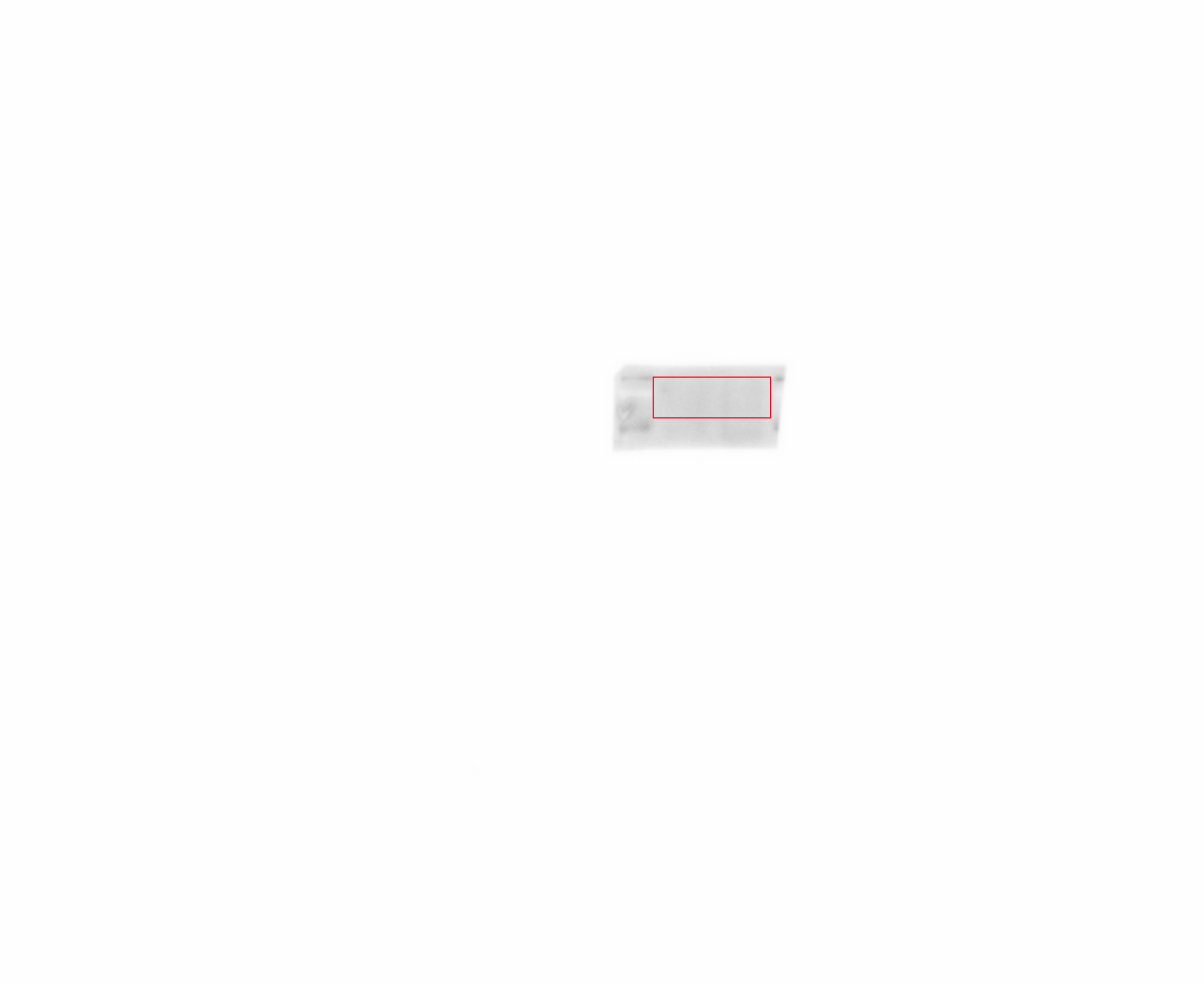

Supplement: Supplementary file 4 — Source data Fig. 3 [file 44318_2025_363_MOESM4_ESM.zip › Figure 3/3A/1 integrin av IP.tif]

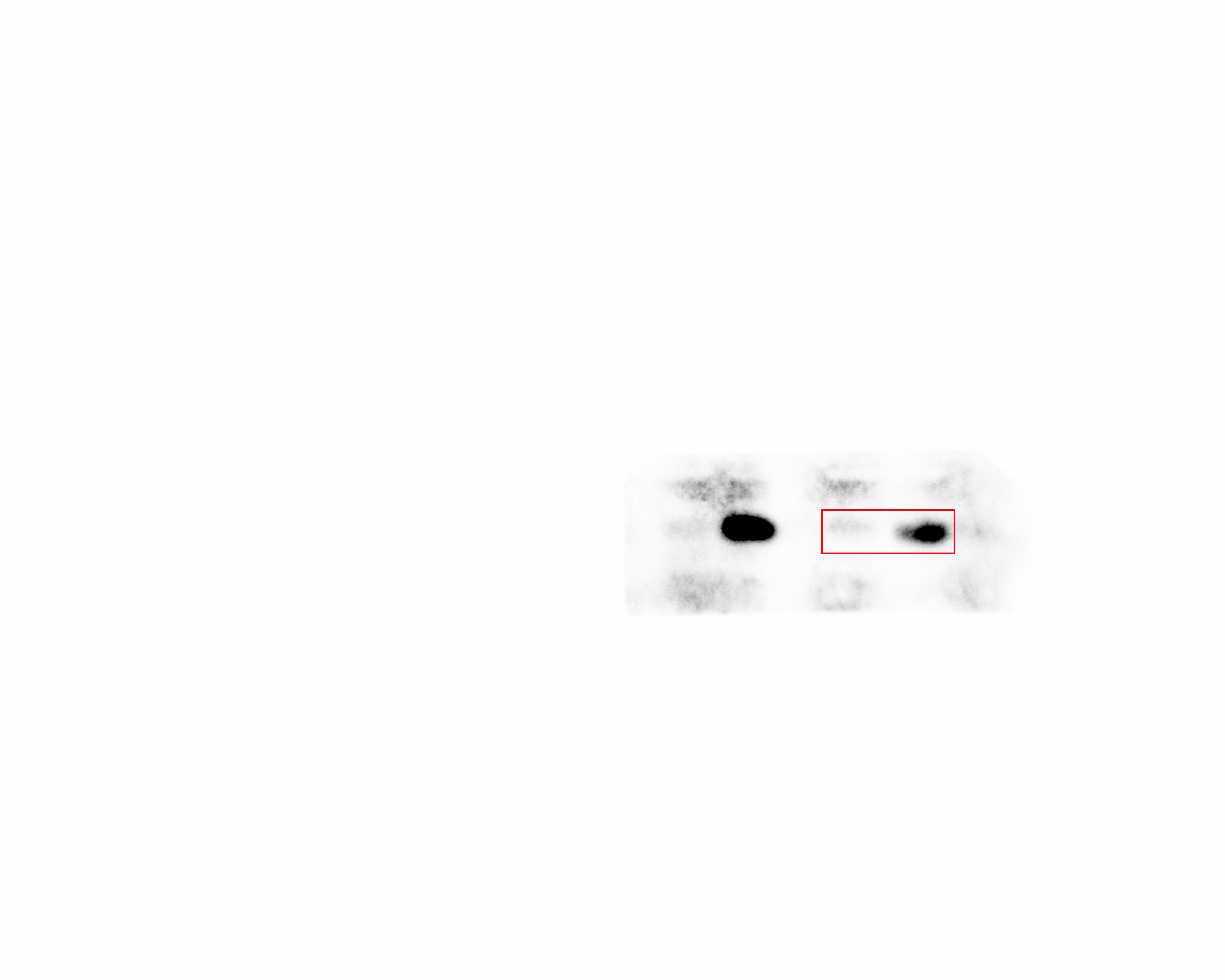

Supplement: Supplementary file 4 — Source data Fig. 3 [file 44318_2025_363_MOESM4_ESM.zip › Figure 3/3A/10 flag input.tif]

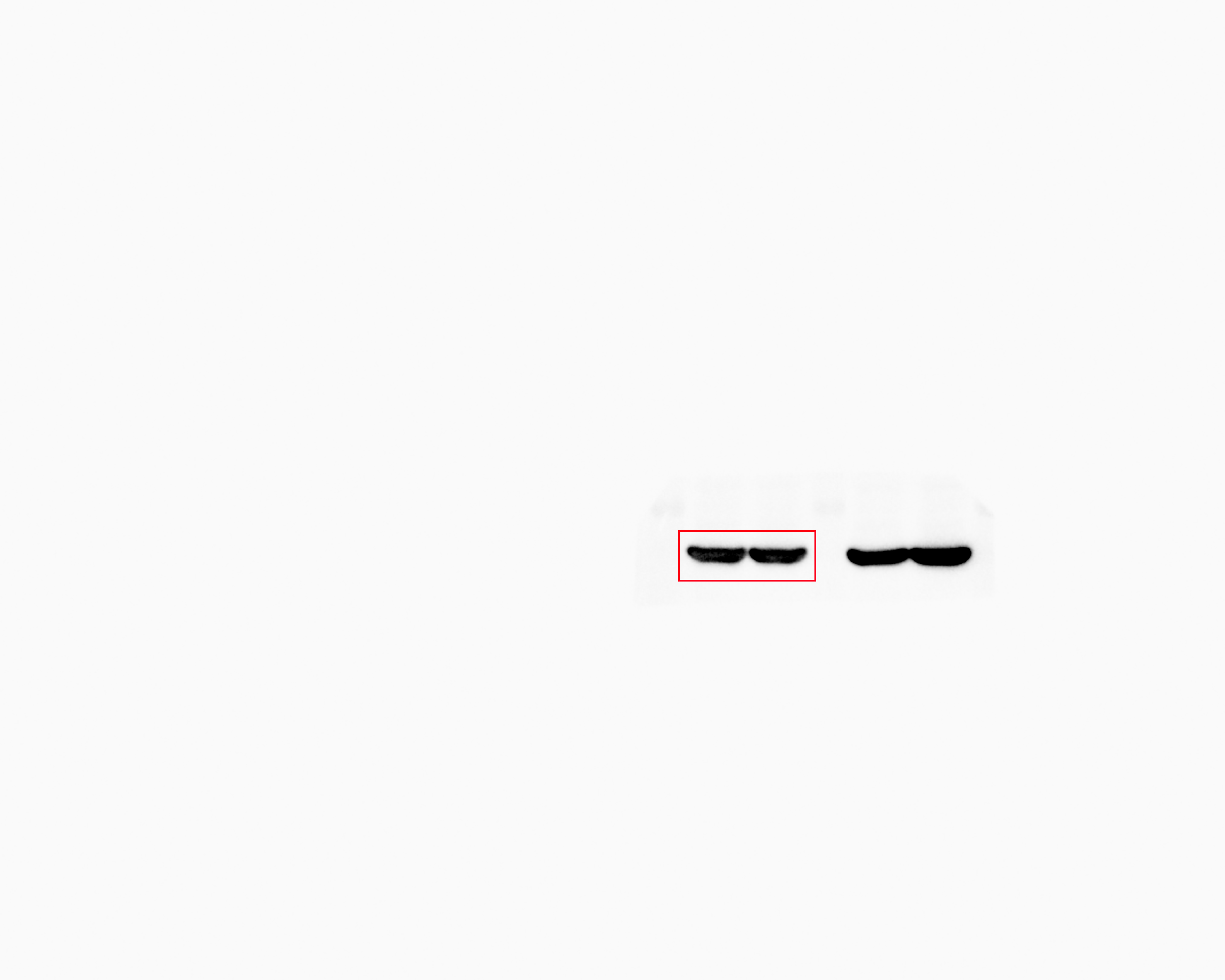

Supplement: Supplementary file 4 — Source data Fig. 3 [file 44318_2025_363_MOESM4_ESM.zip › Figure 3/3A/11 actin.tif]

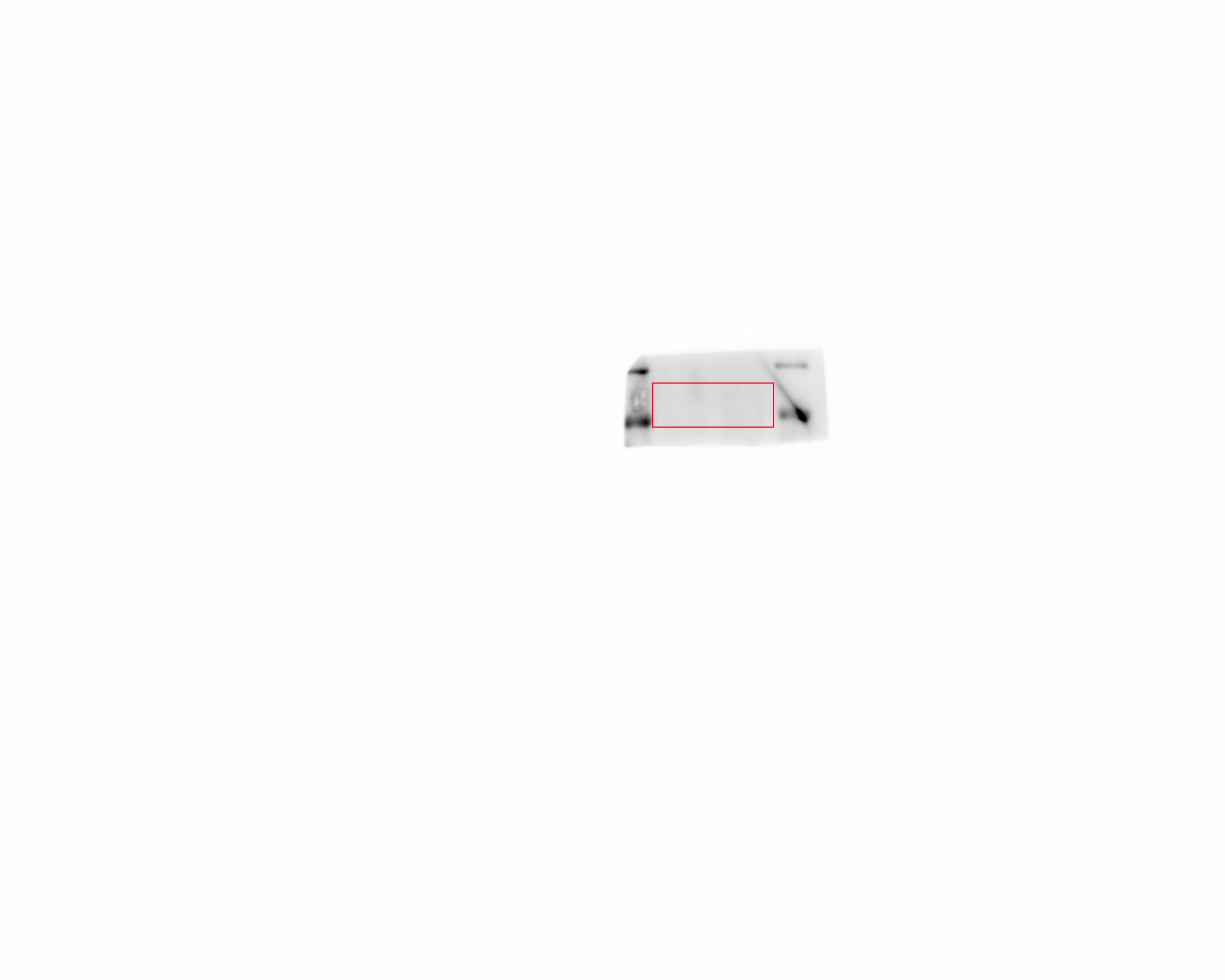

Supplement: Supplementary file 4 — Source data Fig. 3 [file 44318_2025_363_MOESM4_ESM.zip › Figure 3/3A/2 integrin b1 IP.tif]

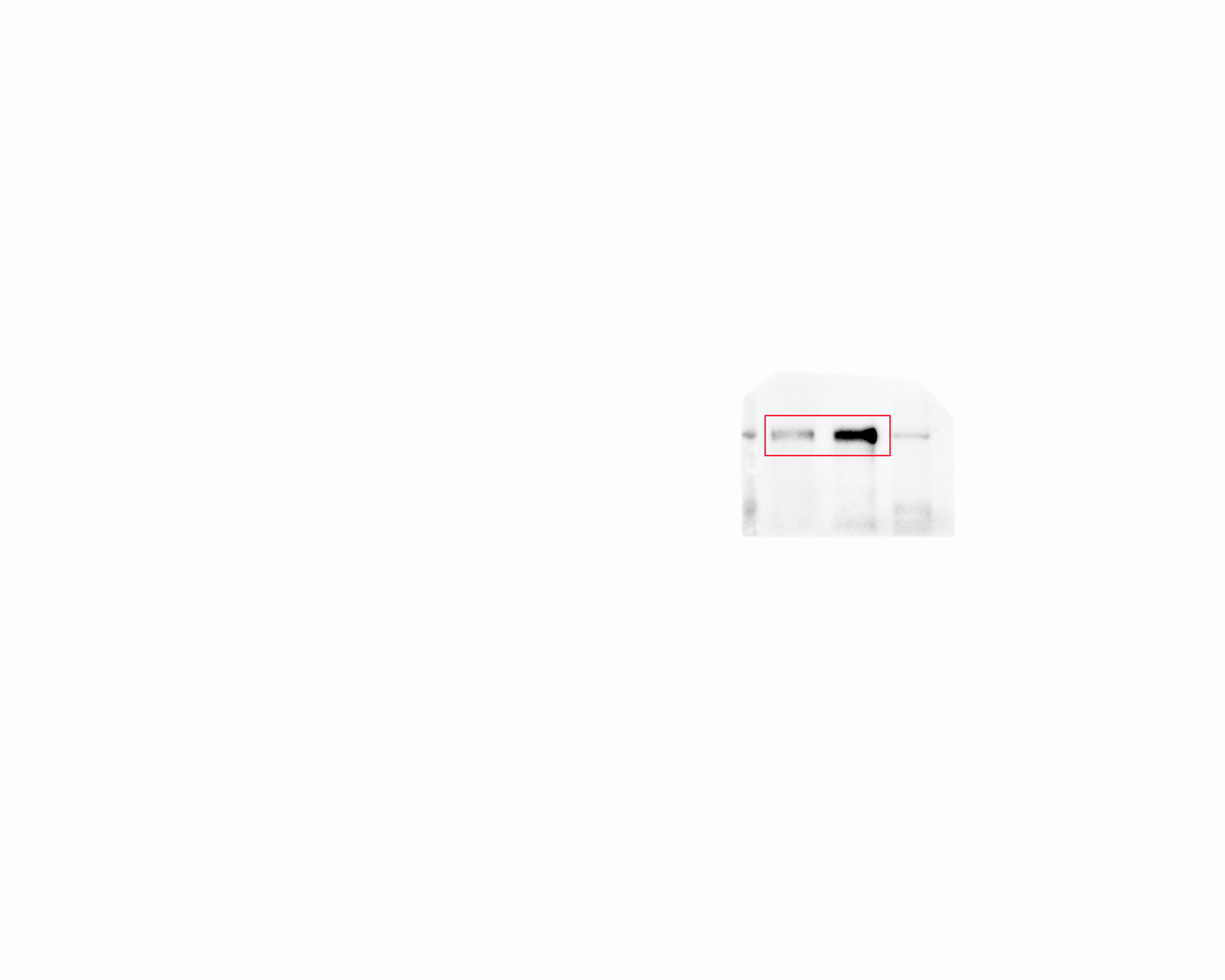

Supplement: Supplementary file 4 — Source data Fig. 3 [file 44318_2025_363_MOESM4_ESM.zip › Figure 3/3A/3 EGFR IP.tif]

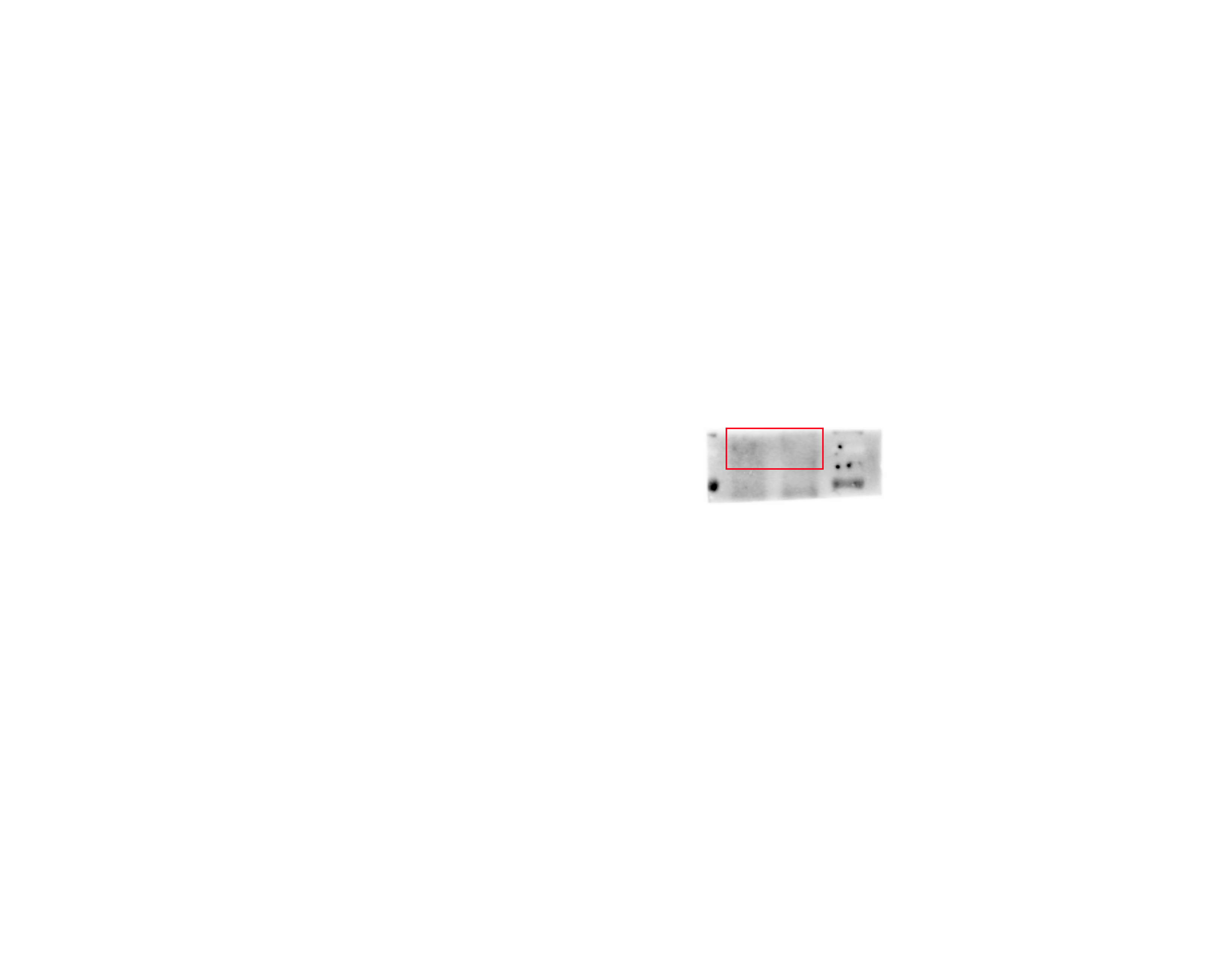

Supplement: Supplementary file 4 — Source data Fig. 3 [file 44318_2025_363_MOESM4_ESM.zip › Figure 3/3A/4 FGFR IP.tif]

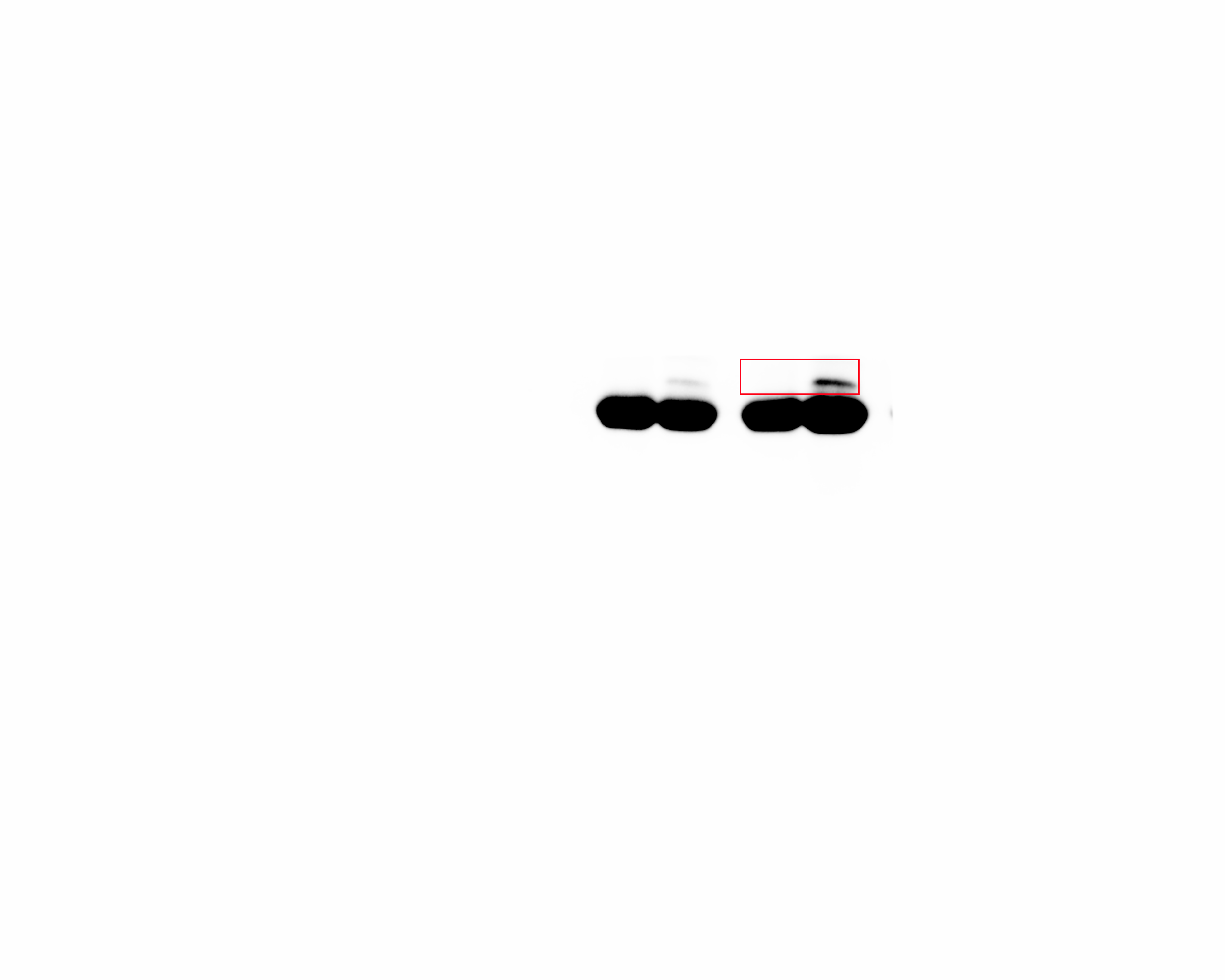

Supplement: Supplementary file 4 — Source data Fig. 3 [file 44318_2025_363_MOESM4_ESM.zip › Figure 3/3A/5 flag IP.tif]

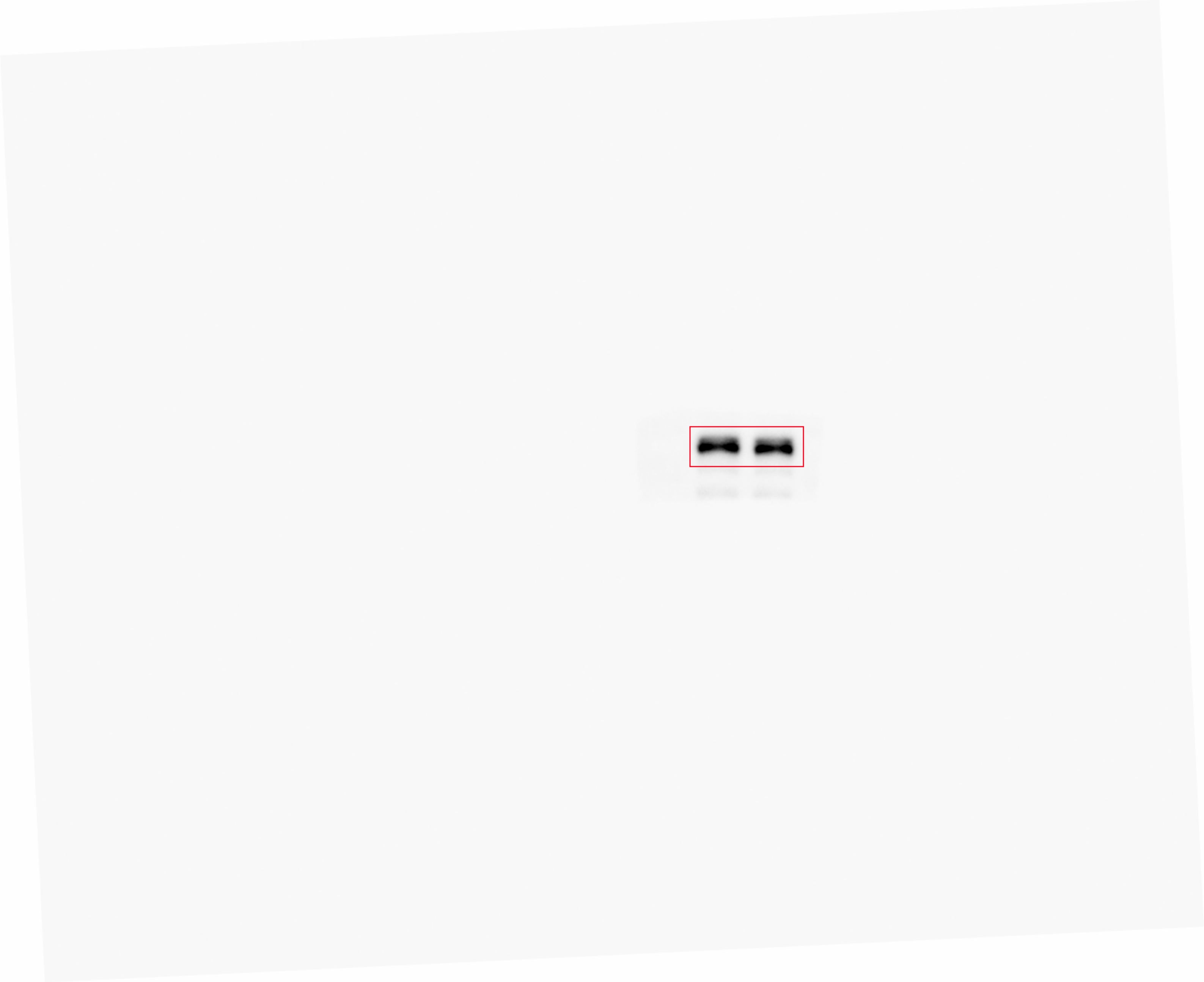

Supplement: Supplementary file 4 — Source data Fig. 3 [file 44318_2025_363_MOESM4_ESM.zip › Figure 3/3A/6 integrin av input.tif]

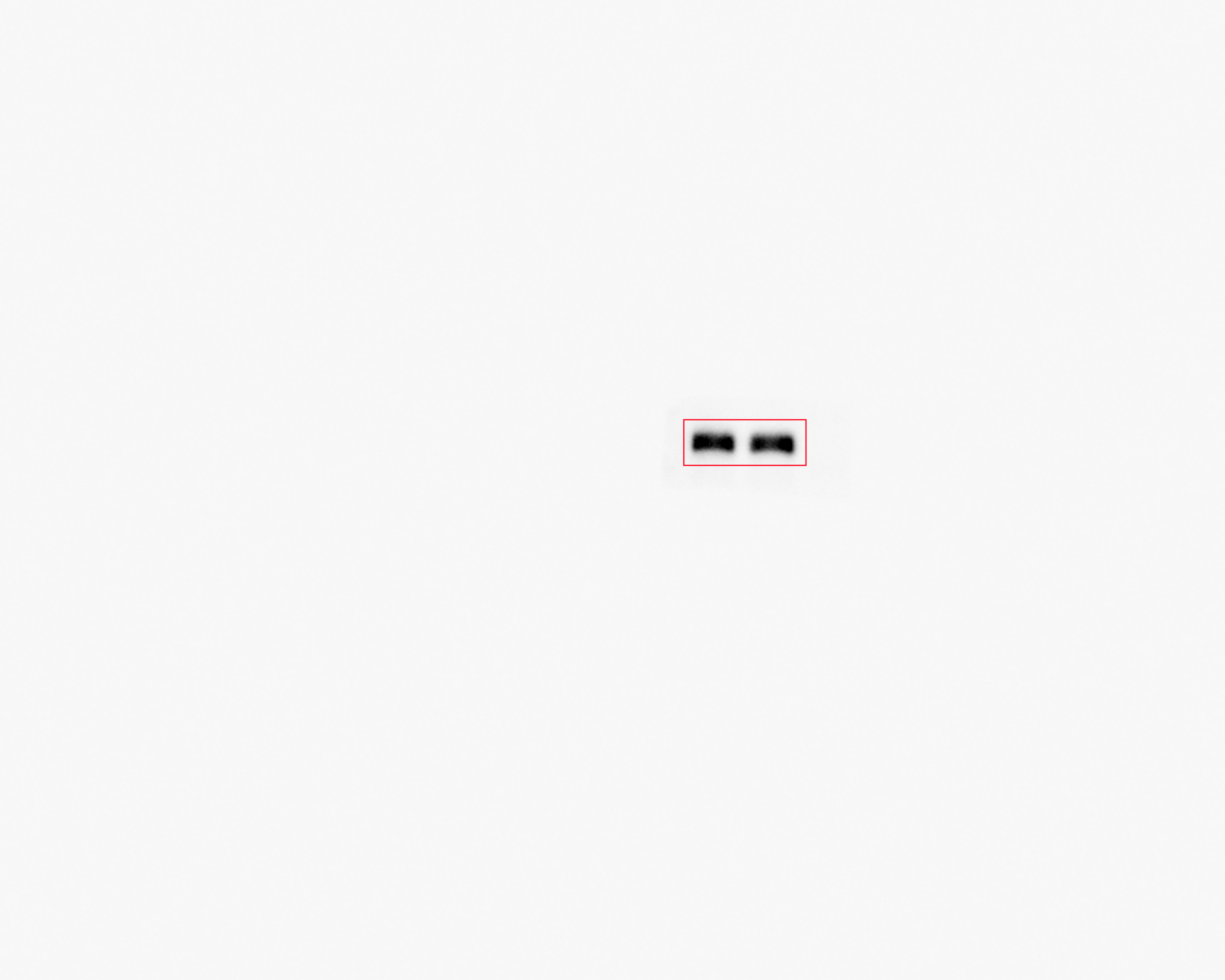

Supplement: Supplementary file 4 — Source data Fig. 3 [file 44318_2025_363_MOESM4_ESM.zip › Figure 3/3A/7 integrin b1 input.tif]

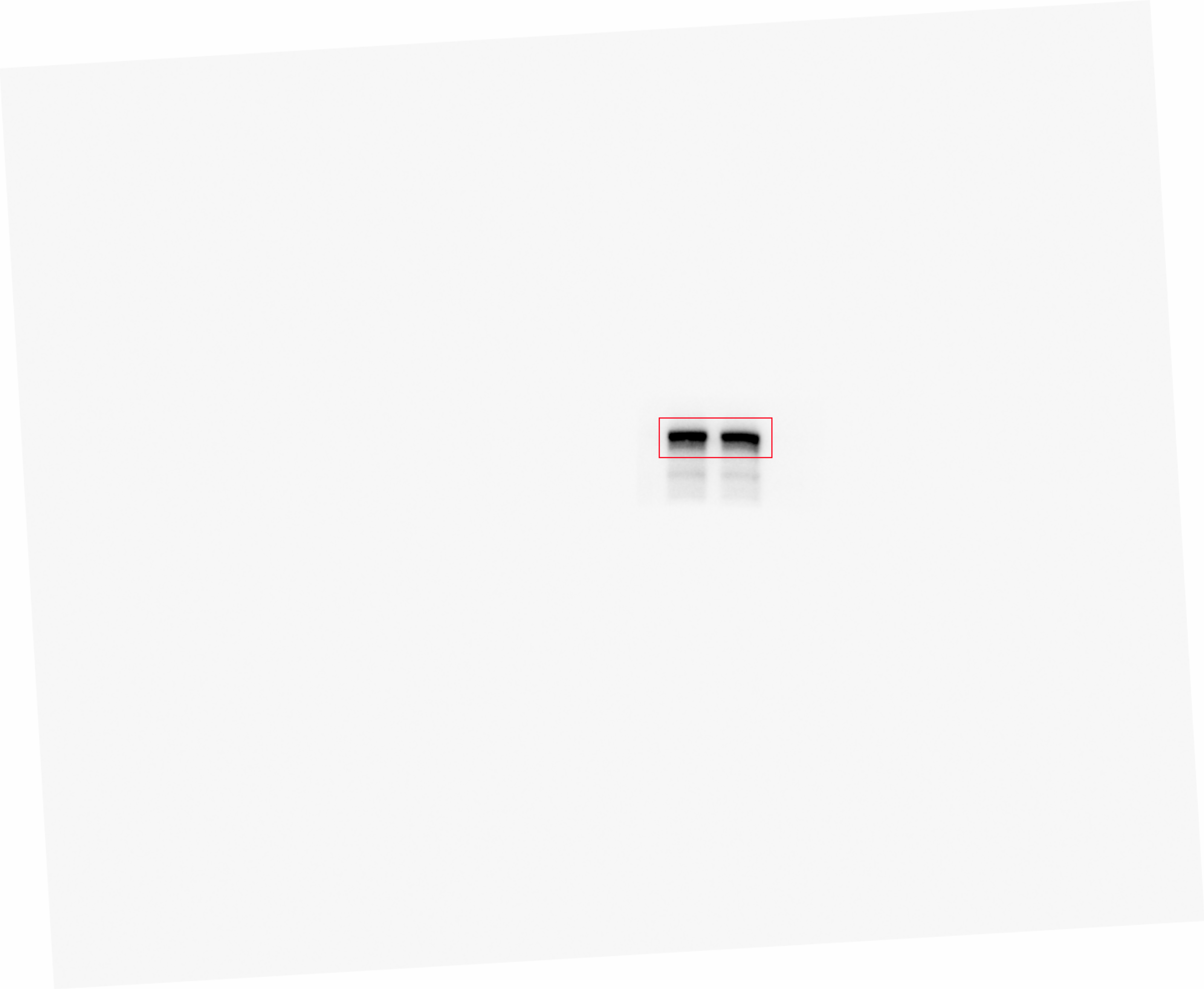

Supplement: Supplementary file 4 — Source data Fig. 3 [file 44318_2025_363_MOESM4_ESM.zip › Figure 3/3A/8 EGFR input.tif]

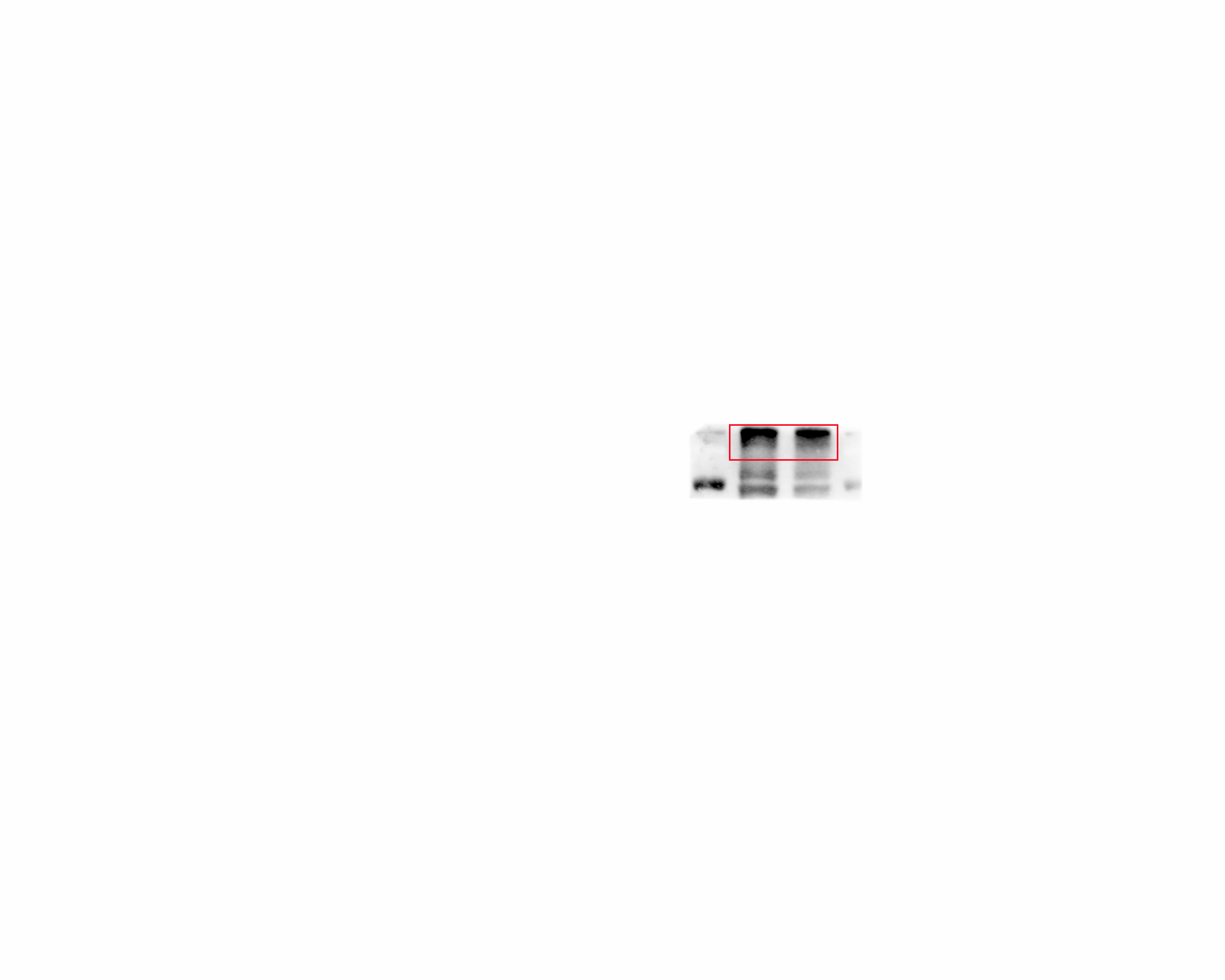

Supplement: Supplementary file 4 — Source data Fig. 3 [file 44318_2025_363_MOESM4_ESM.zip › Figure 3/3A/9 FGFR input.tif]

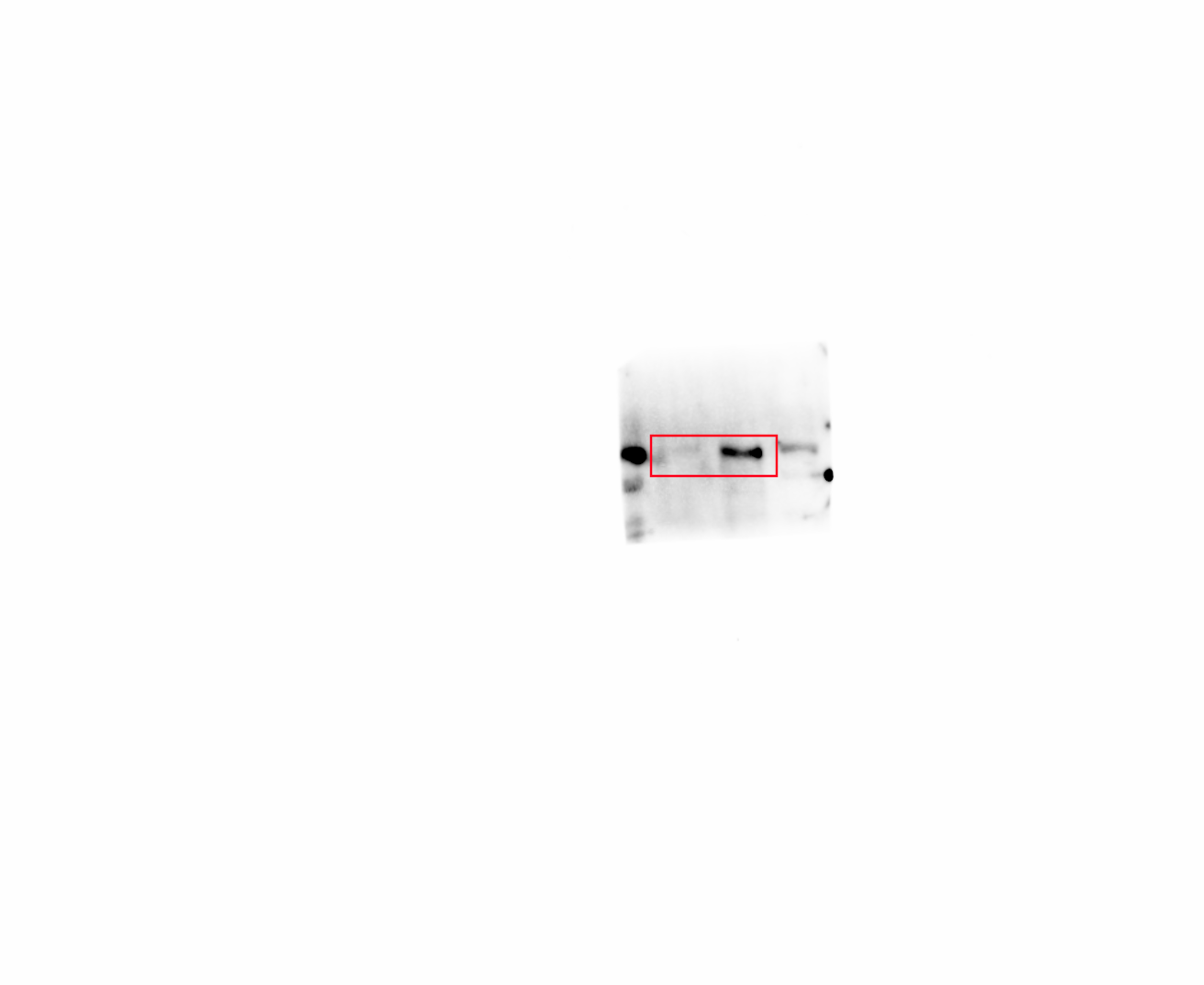

Supplement: Supplementary file 4 — Source data Fig. 3 [file 44318_2025_363_MOESM4_ESM.zip › Figure 3/3B/1 EGFR IP.tif]

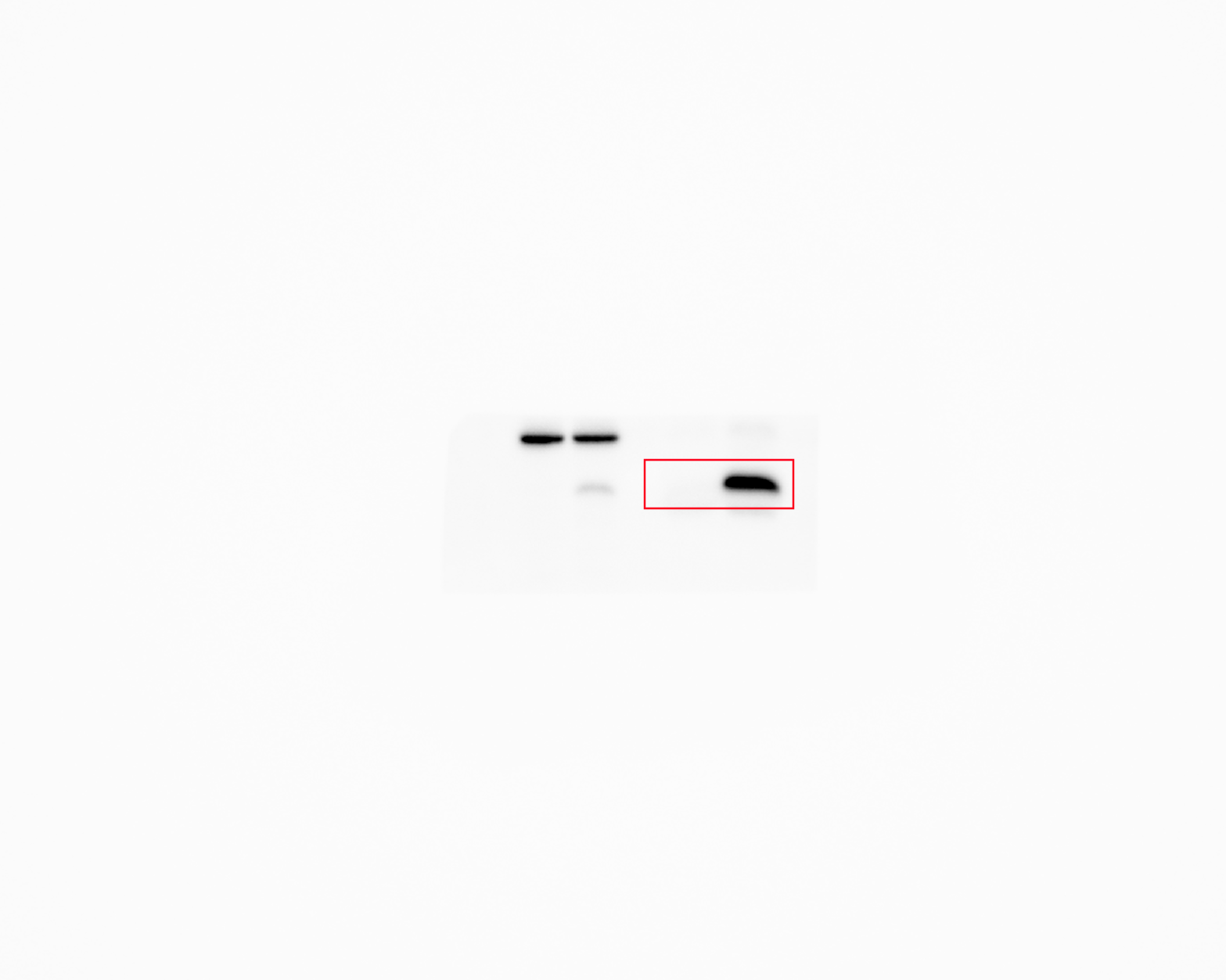

Supplement: Supplementary file 4 — Source data Fig. 3 [file 44318_2025_363_MOESM4_ESM.zip › Figure 3/3B/2 flag IP.tif]

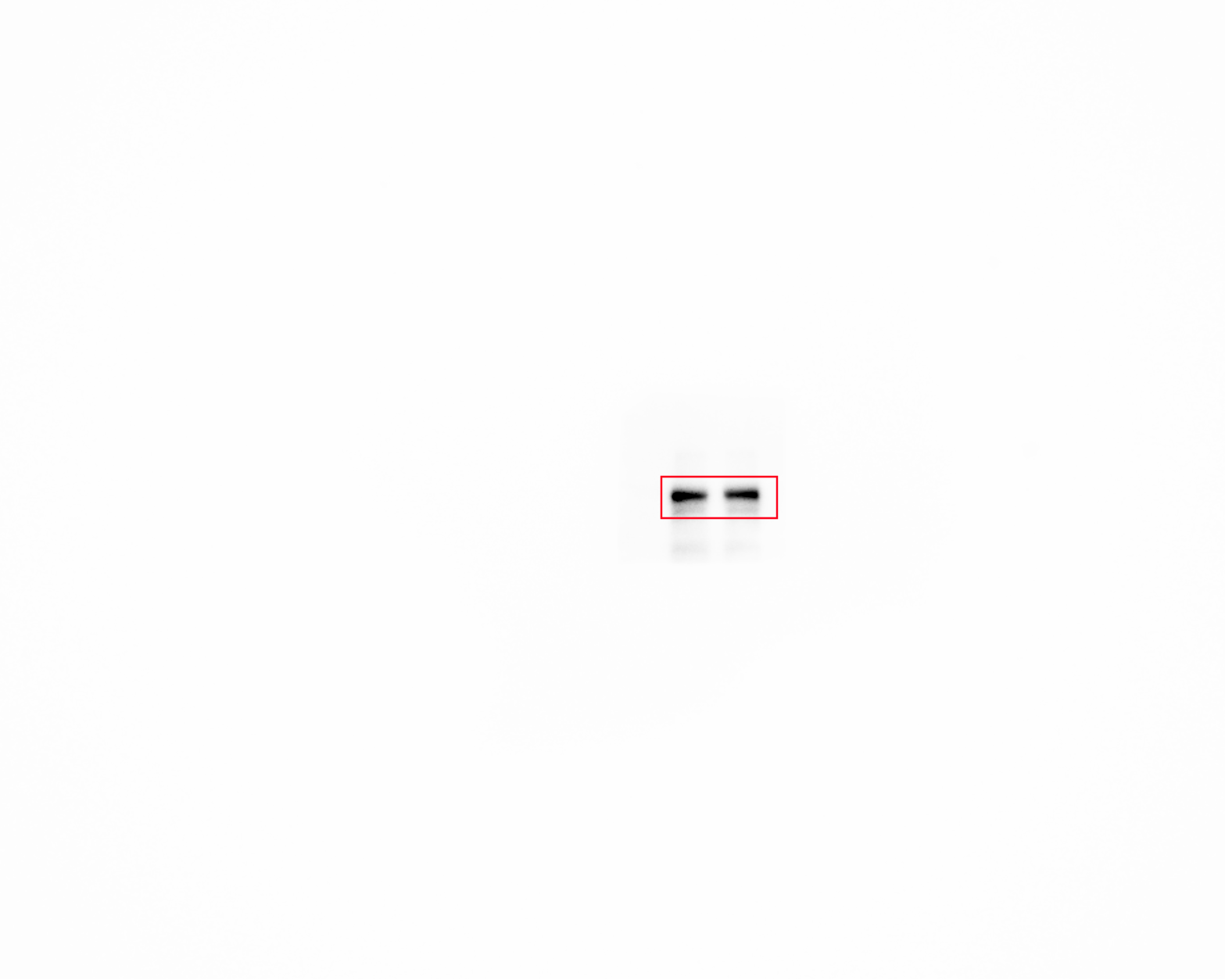

Supplement: Supplementary file 4 — Source data Fig. 3 [file 44318_2025_363_MOESM4_ESM.zip › Figure 3/3B/3 EGFR input.tif]

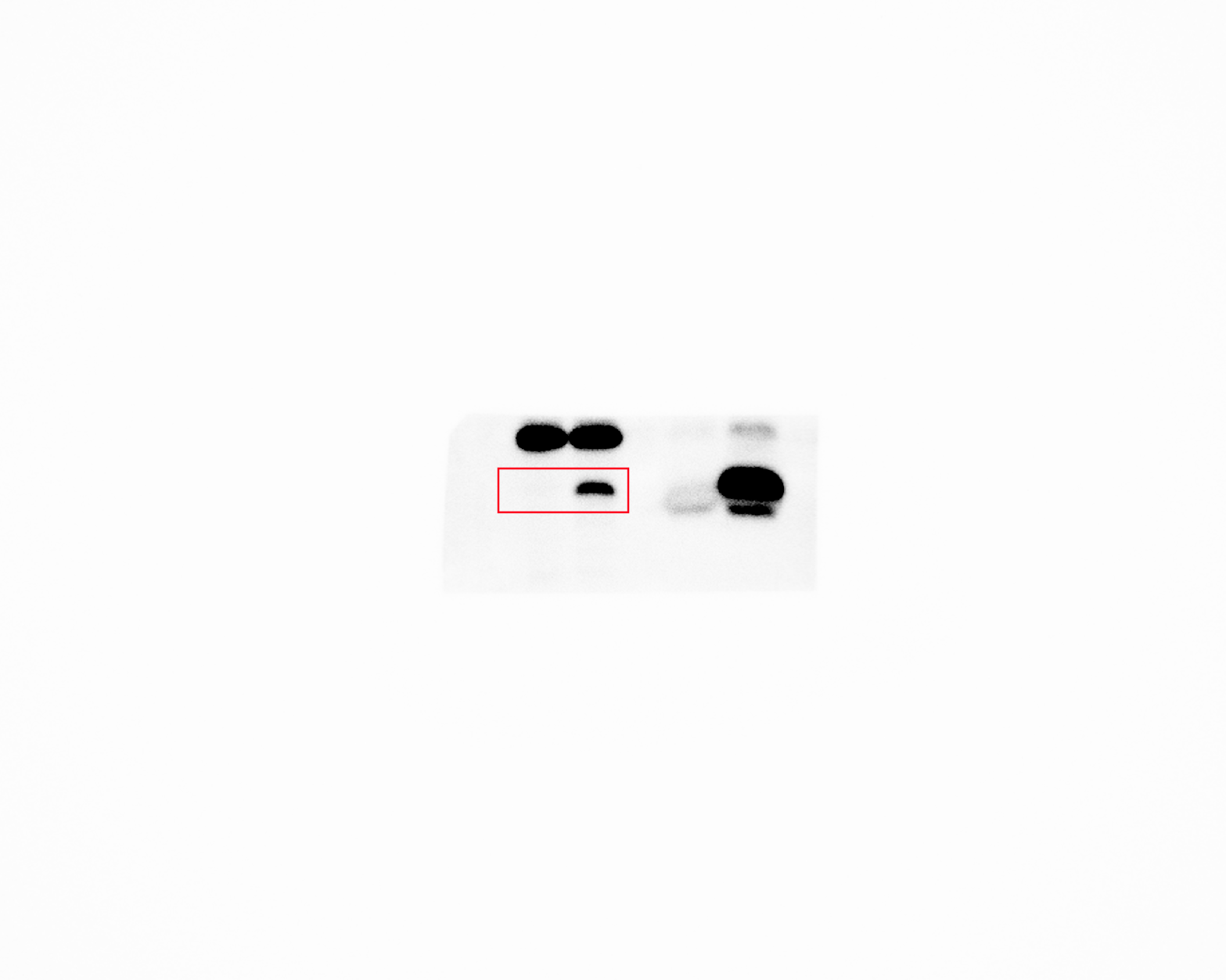

Supplement: Supplementary file 4 — Source data Fig. 3 [file 44318_2025_363_MOESM4_ESM.zip › Figure 3/3B/4 flag input.tif]

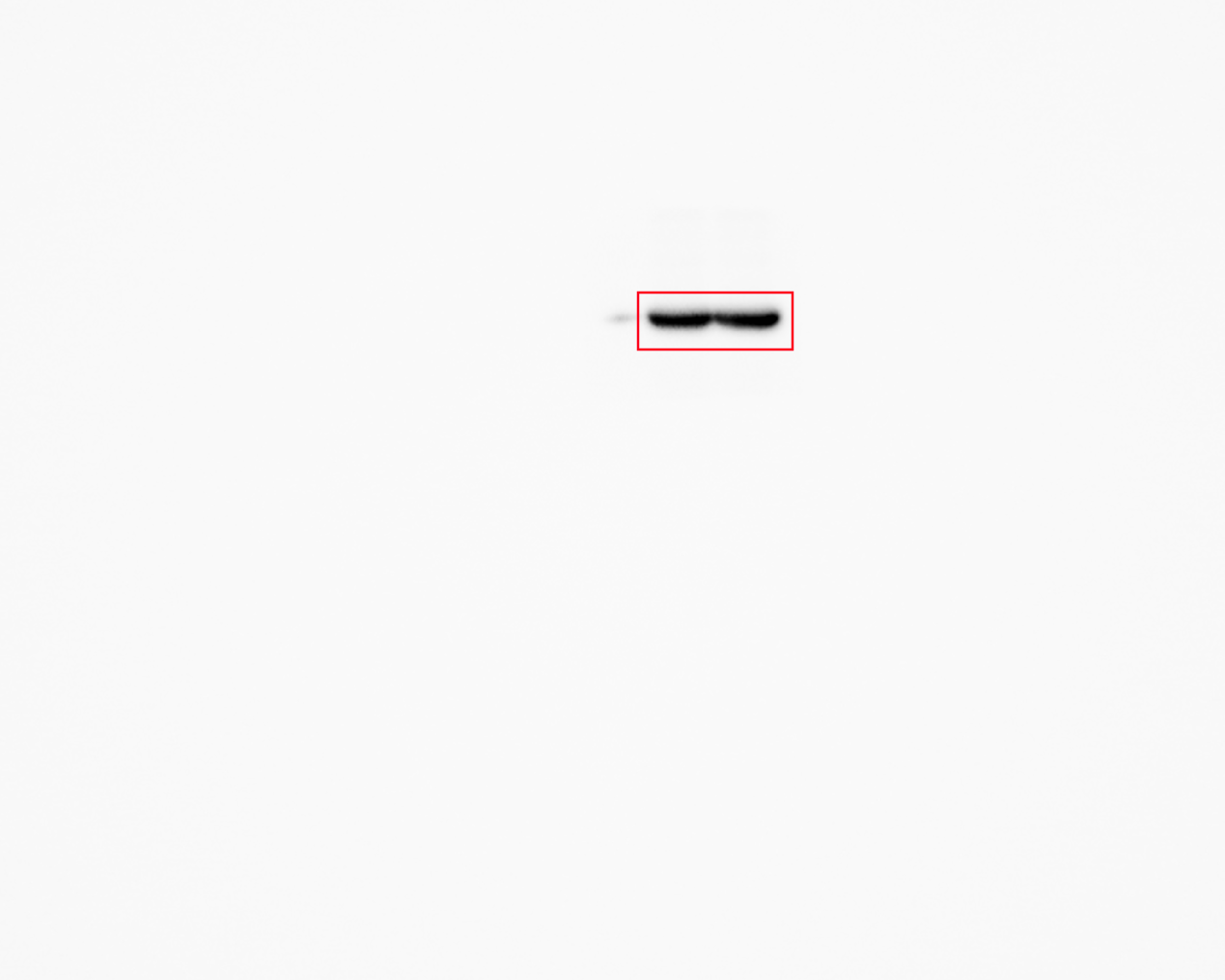

Supplement: Supplementary file 4 — Source data Fig. 3 [file 44318_2025_363_MOESM4_ESM.zip › Figure 3/3B/5 actin.tif]

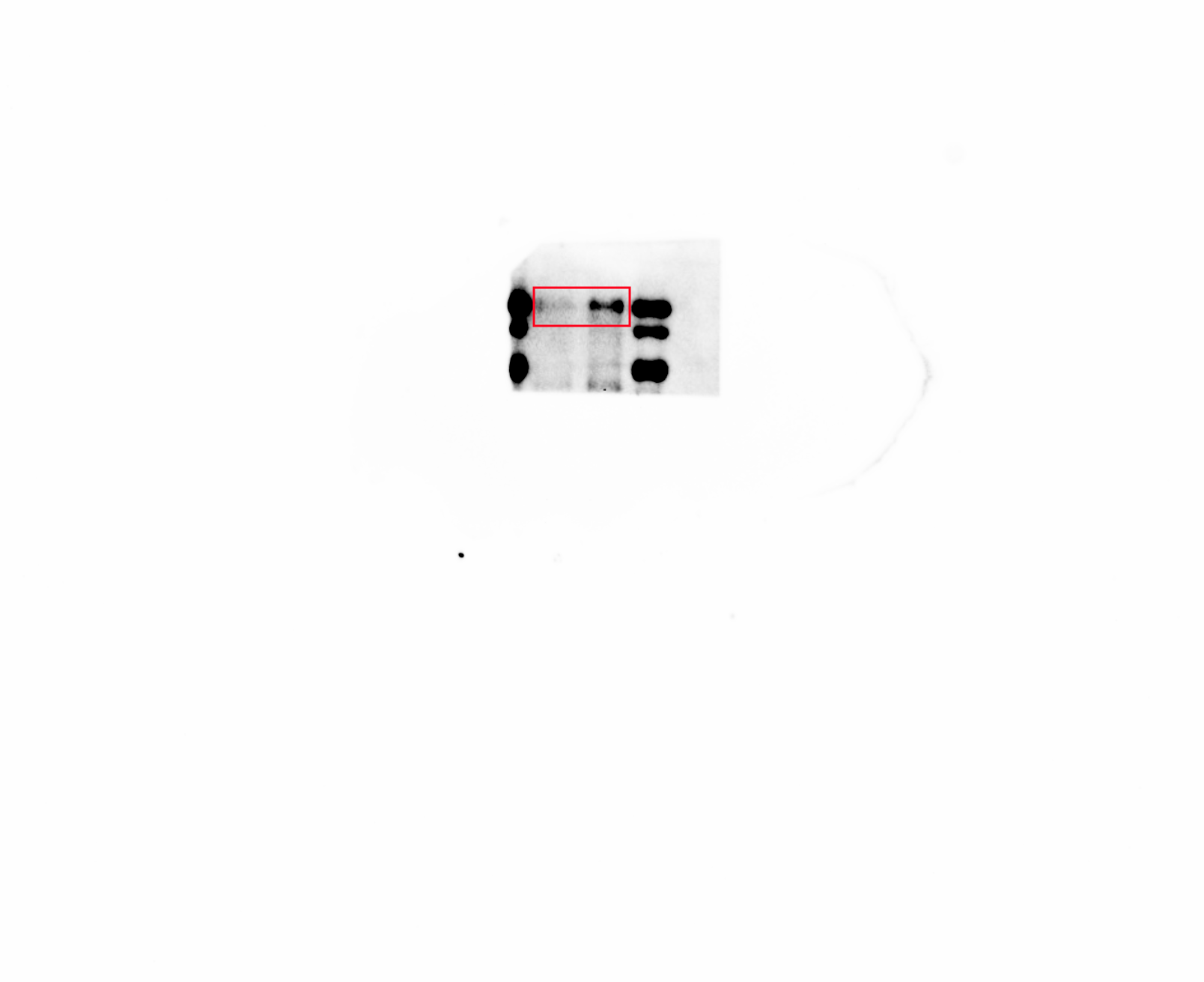

Supplement: Supplementary file 4 — Source data Fig. 3 [file 44318_2025_363_MOESM4_ESM.zip › Figure 3/3C/1 EGFR IP.tif]

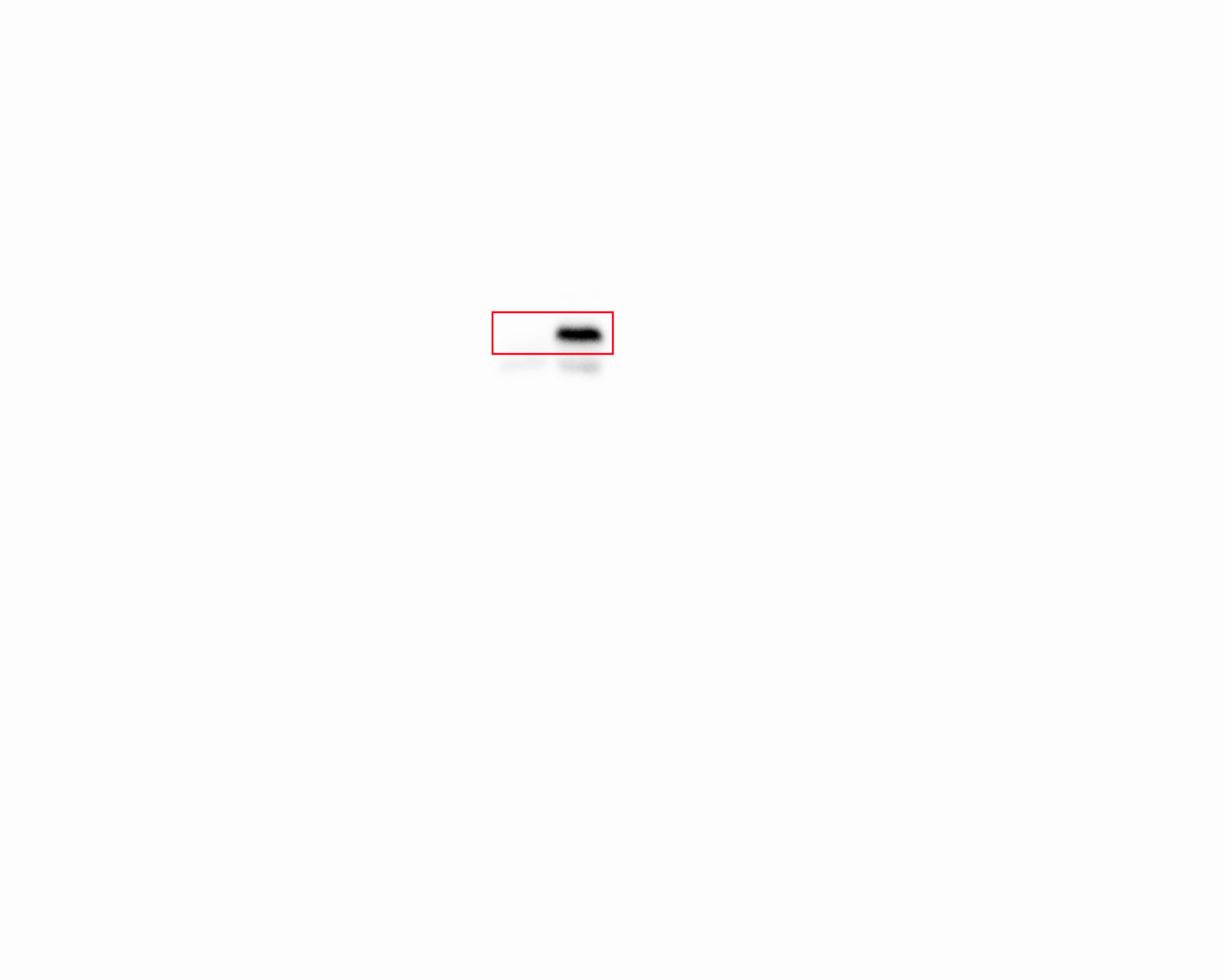

Supplement: Supplementary file 4 — Source data Fig. 3 [file 44318_2025_363_MOESM4_ESM.zip › Figure 3/3C/2 flag IP.tif]

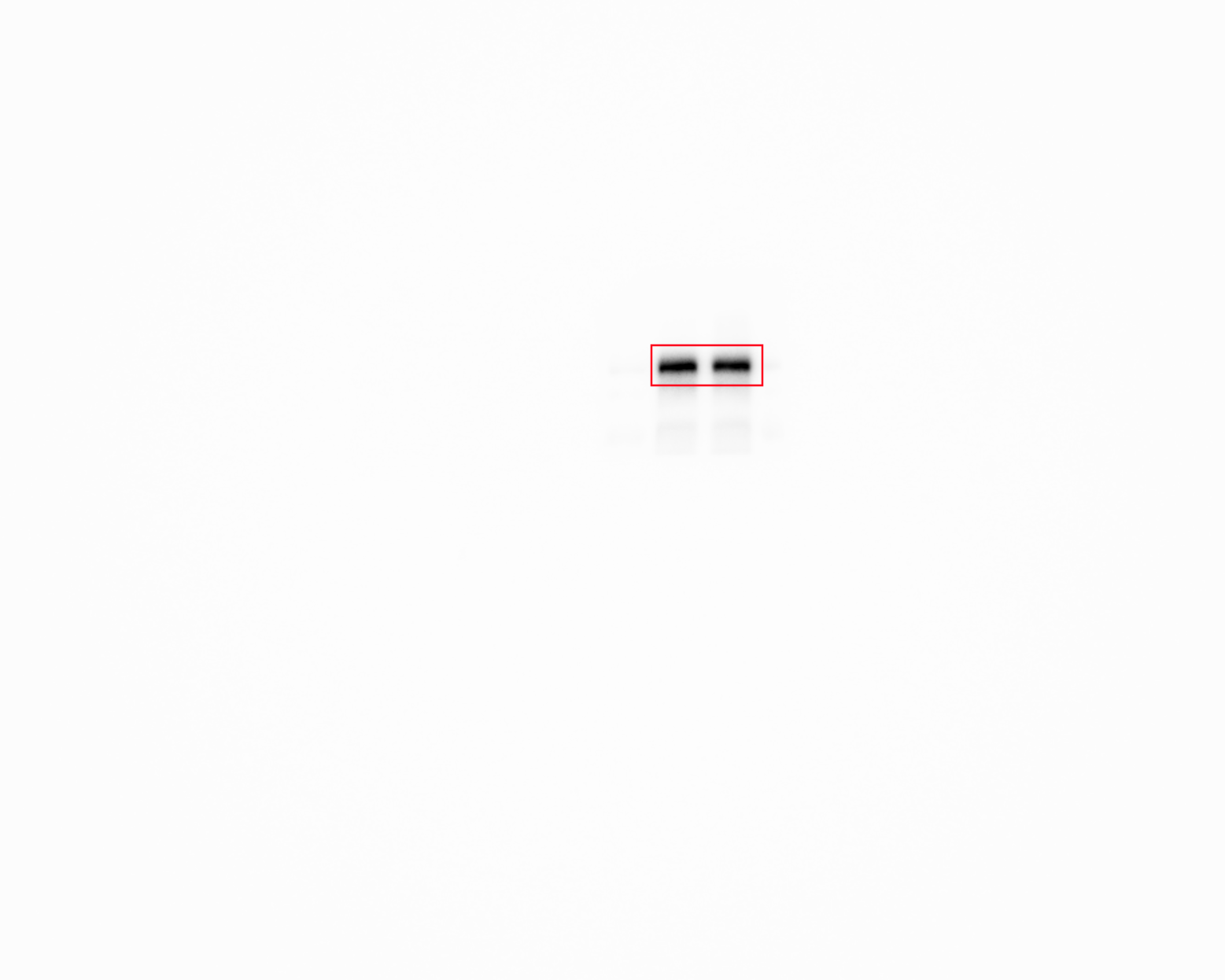

Supplement: Supplementary file 4 — Source data Fig. 3 [file 44318_2025_363_MOESM4_ESM.zip › Figure 3/3C/3 EGFR input.tif]

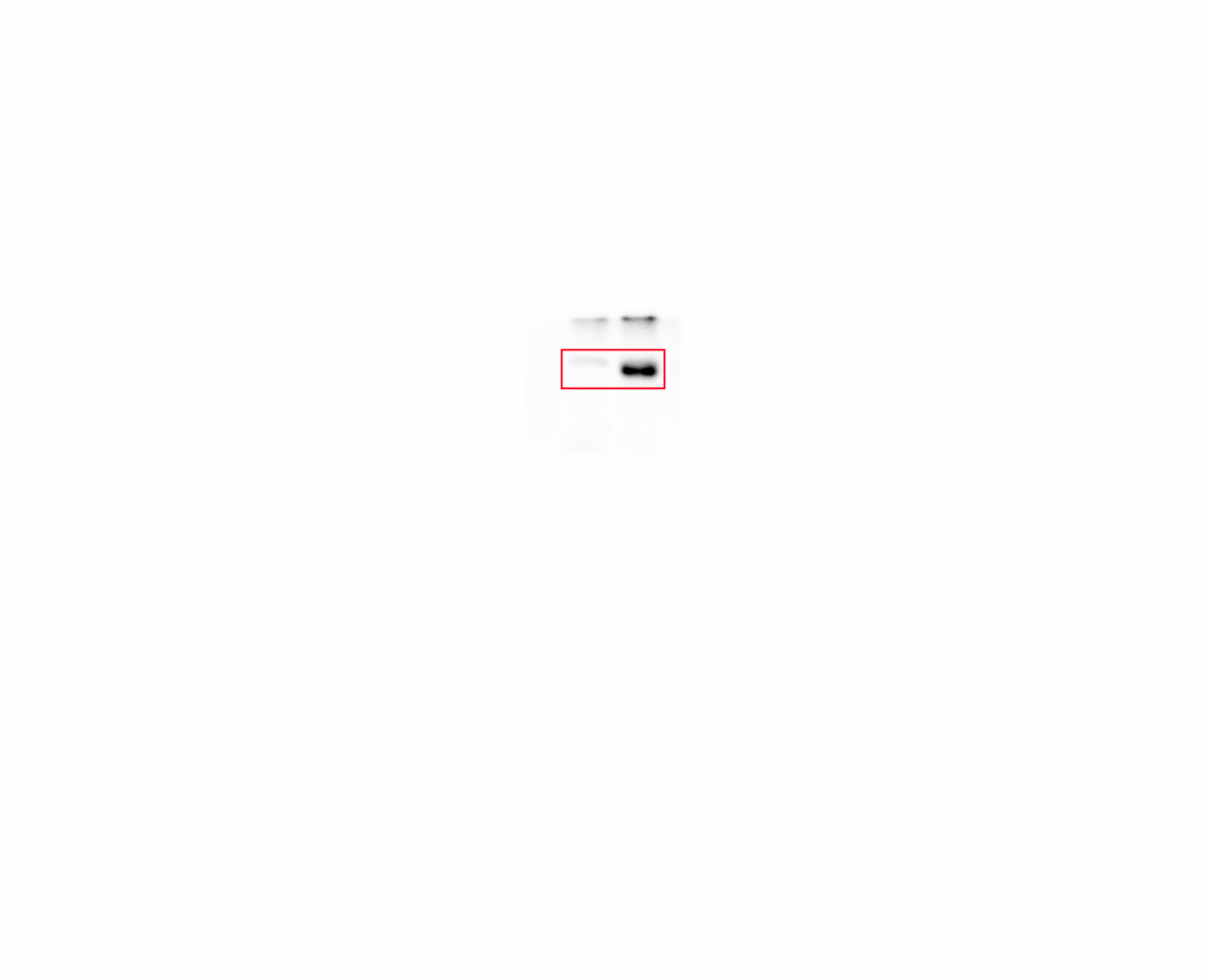

Supplement: Supplementary file 4 — Source data Fig. 3 [file 44318_2025_363_MOESM4_ESM.zip › Figure 3/3C/4 flag input.tif]

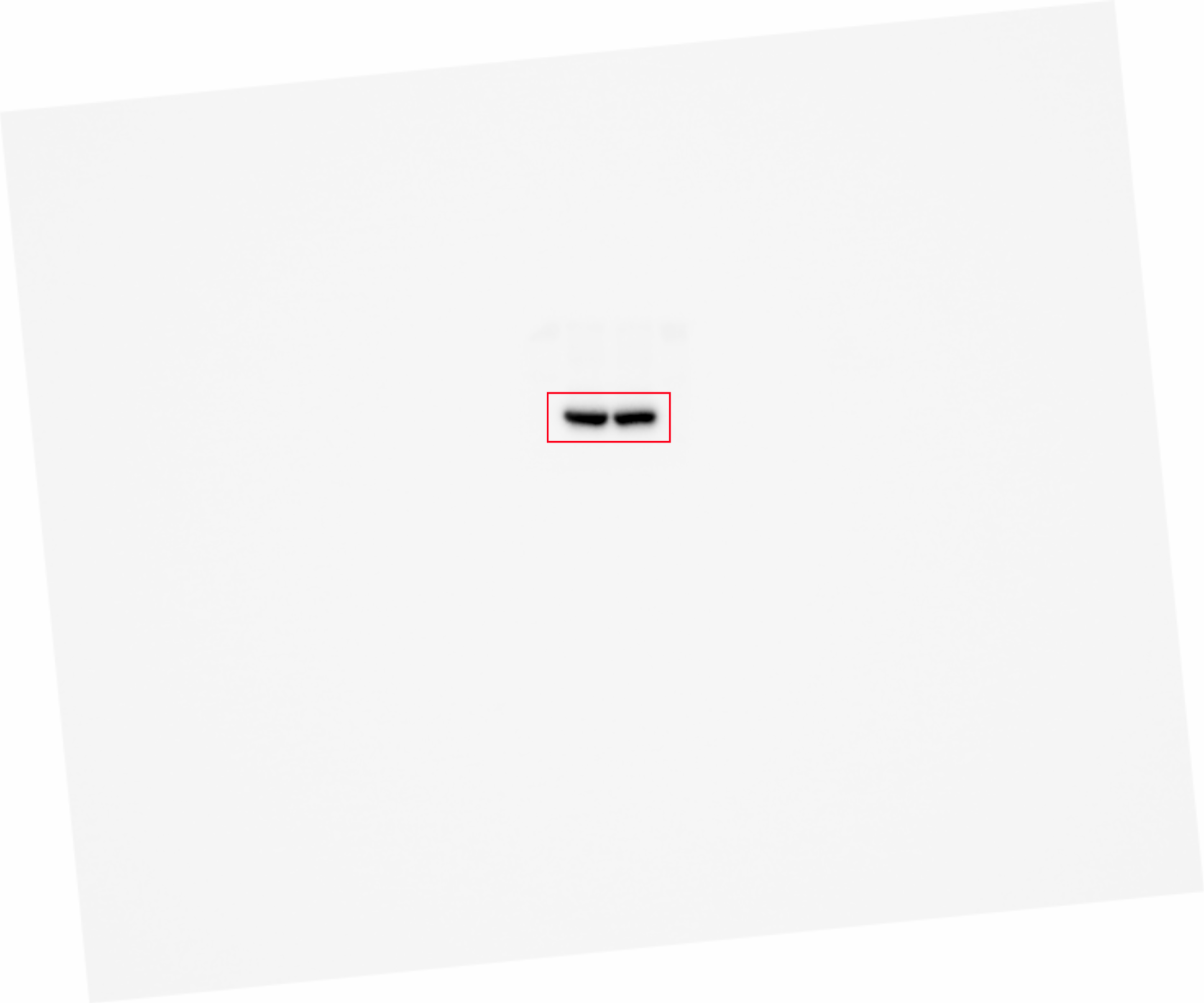

Supplement: Supplementary file 4 — Source data Fig. 3 [file 44318_2025_363_MOESM4_ESM.zip › Figure 3/3C/5 actin.tif]

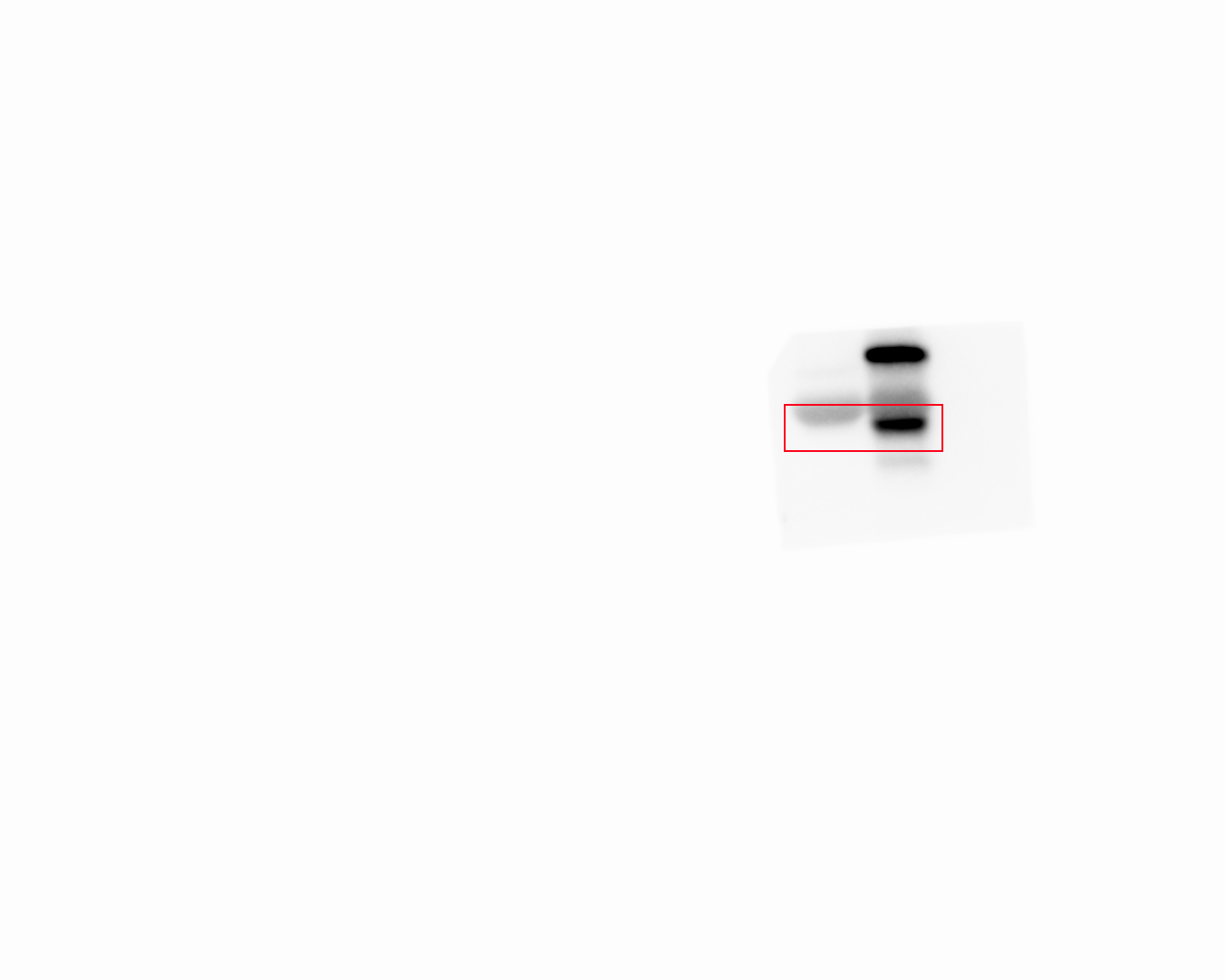

Supplement: Supplementary file 4 — Source data Fig. 3 [file 44318_2025_363_MOESM4_ESM.zip › Figure 3/3D/1 Ephrin A1 IP.tif]

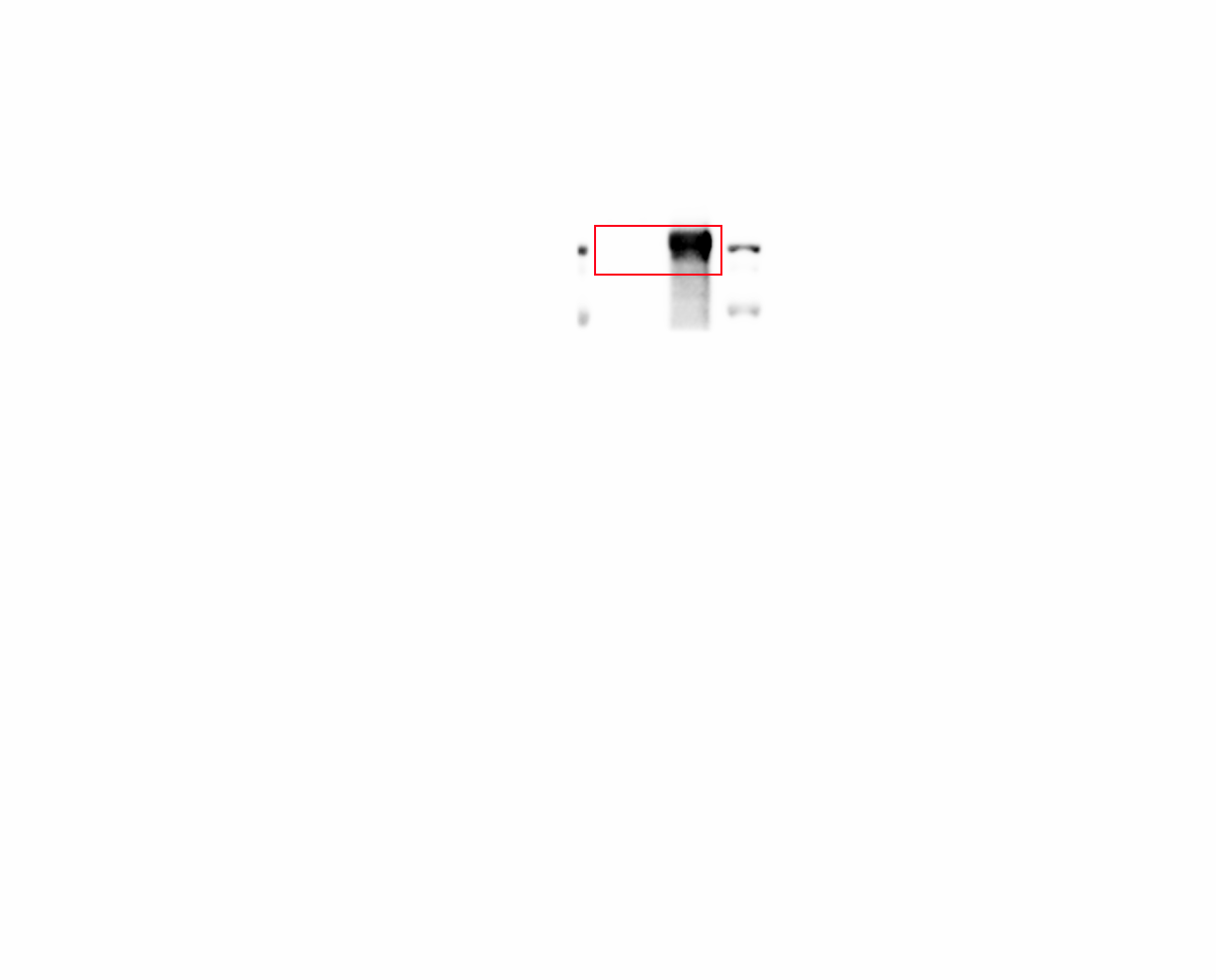

Supplement: Supplementary file 4 — Source data Fig. 3 [file 44318_2025_363_MOESM4_ESM.zip › Figure 3/3D/2 EGFR IP.tif]

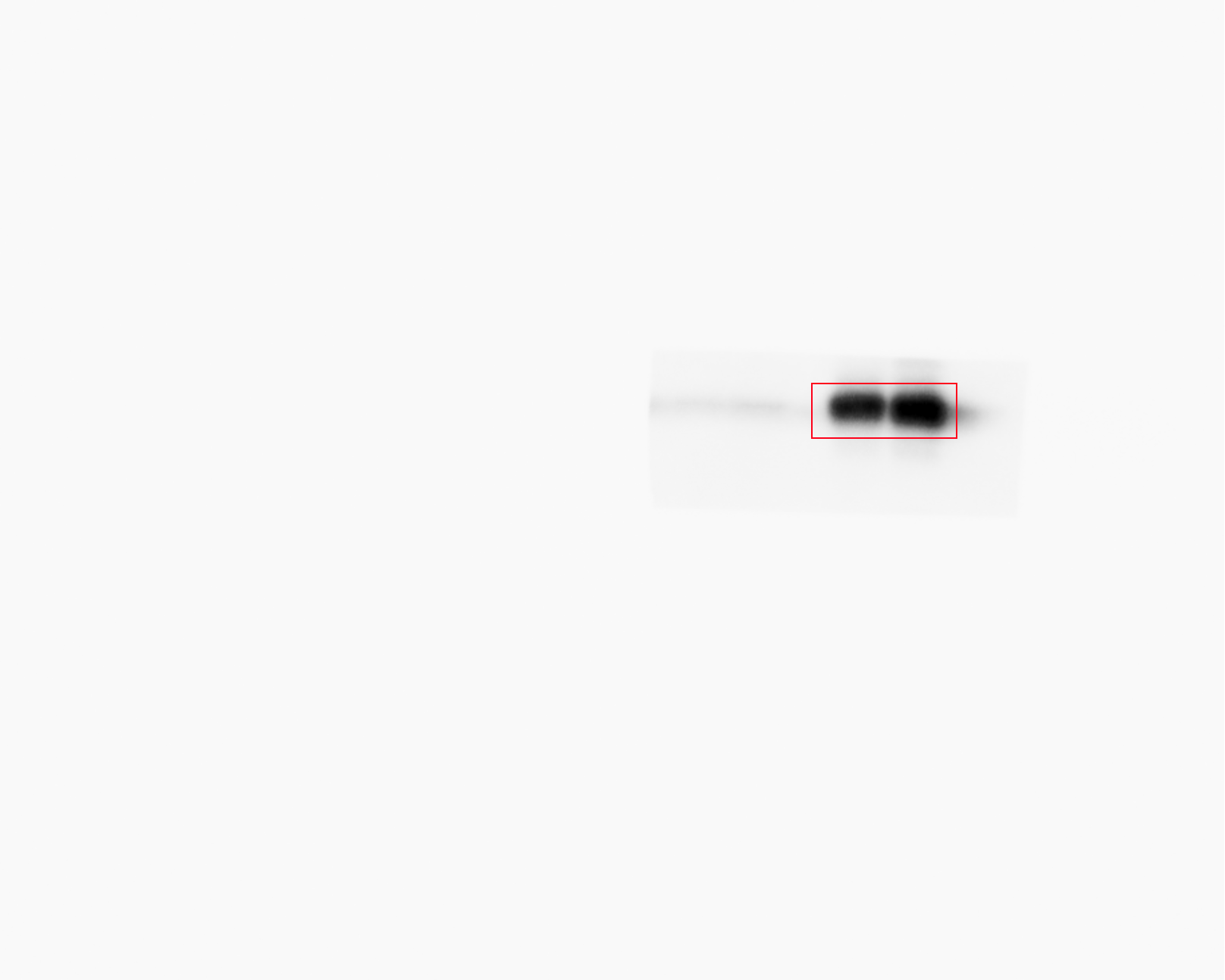

Supplement: Supplementary file 4 — Source data Fig. 3 [file 44318_2025_363_MOESM4_ESM.zip › Figure 3/3D/3 Ephrin A1 input.tif]

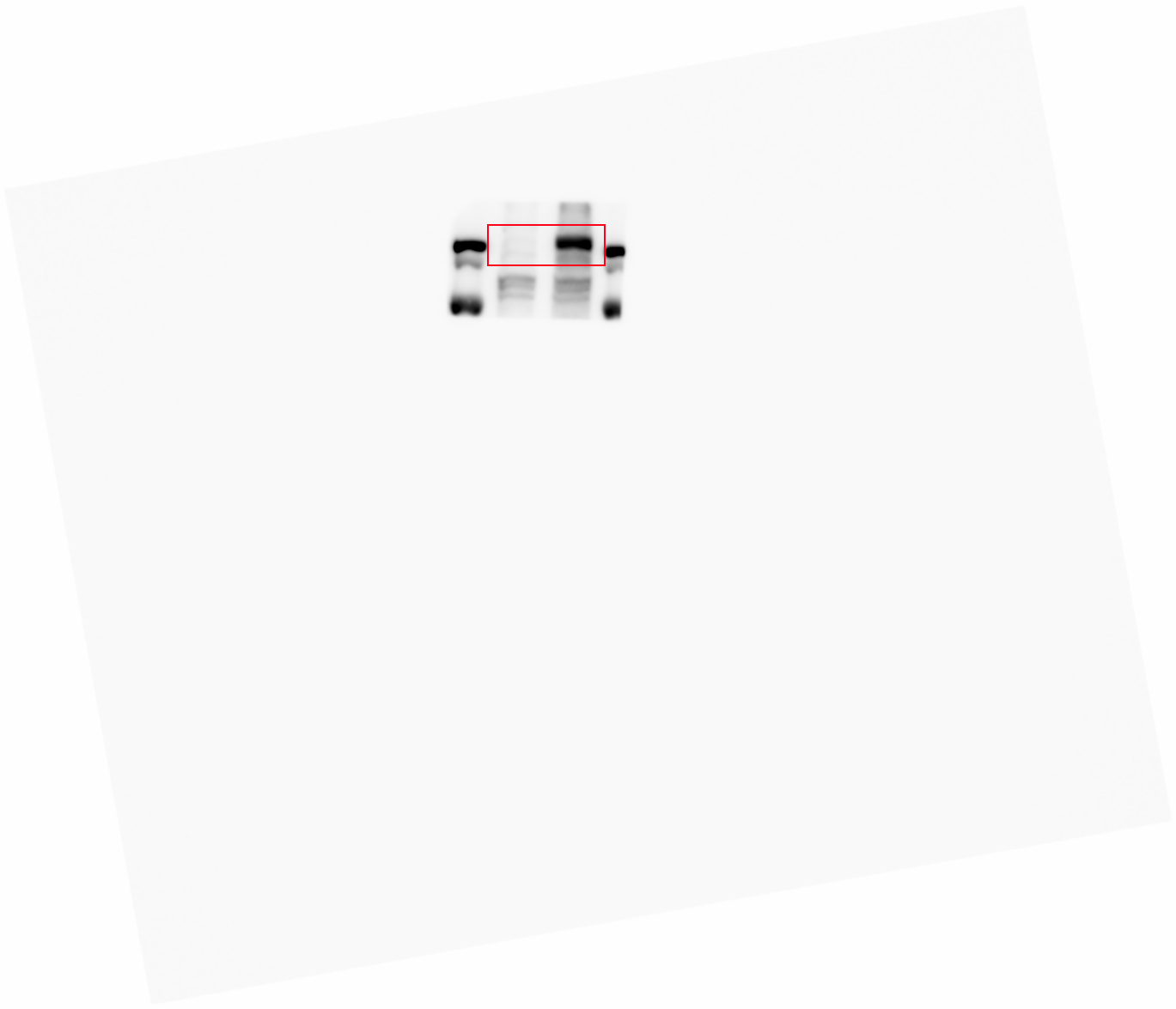

Supplement: Supplementary file 4 — Source data Fig. 3 [file 44318_2025_363_MOESM4_ESM.zip › Figure 3/3D/4 EGFR input.tif]

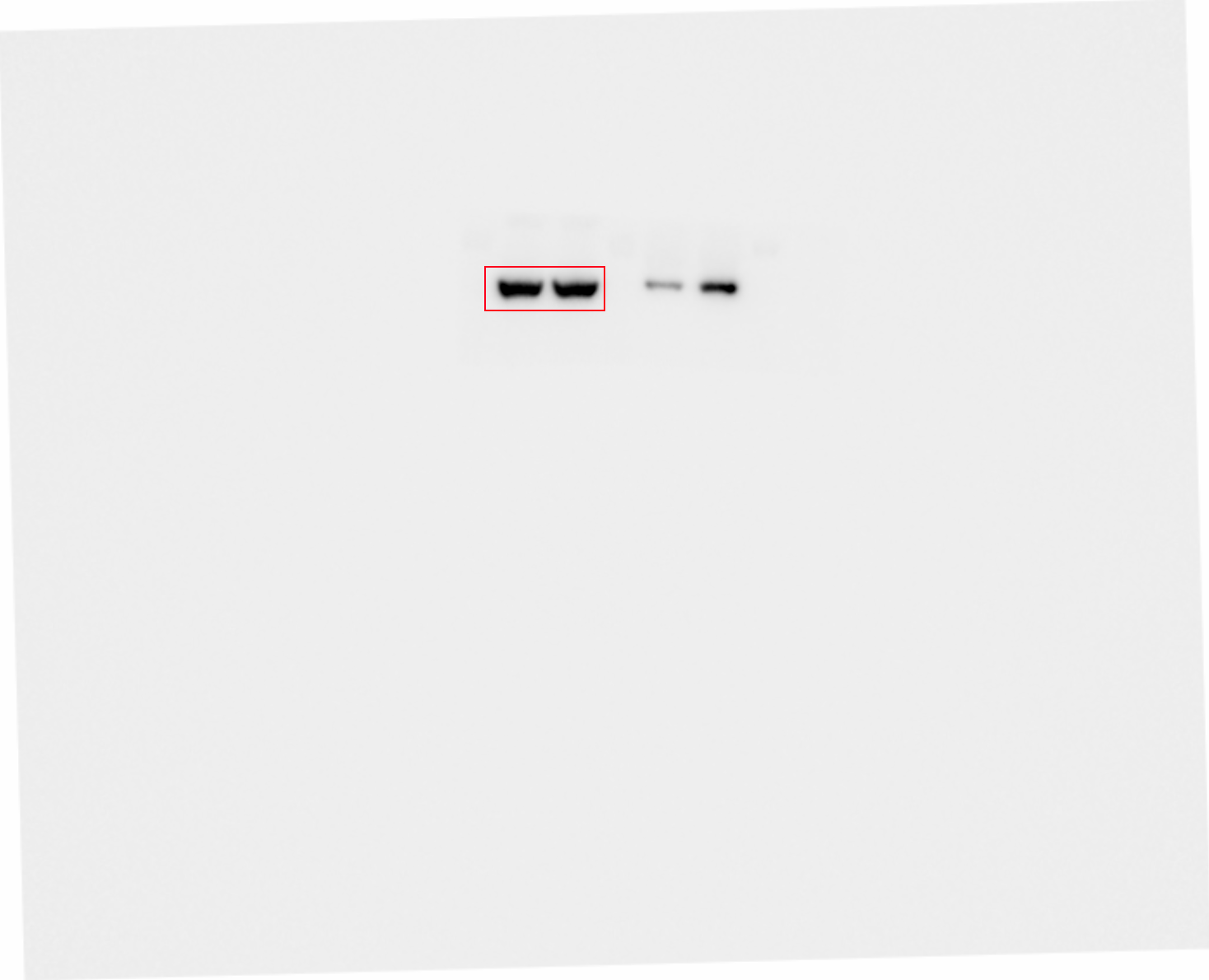

Supplement: Supplementary file 4 — Source data Fig. 3 [file 44318_2025_363_MOESM4_ESM.zip › Figure 3/3D/5 actin.tif]

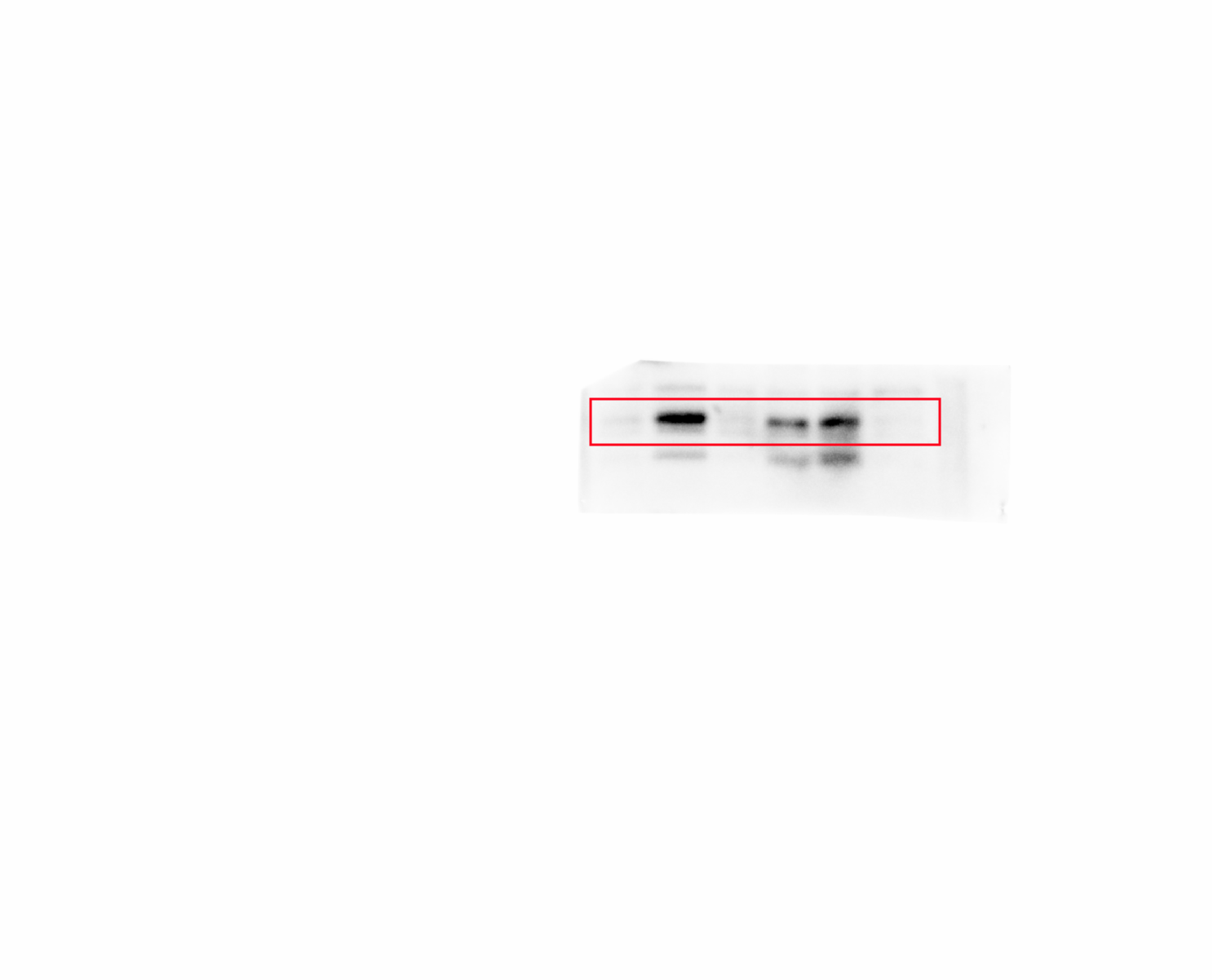

Supplement: Supplementary file 4 — Source data Fig. 3 [file 44318_2025_363_MOESM4_ESM.zip › Figure 3/3F/1 Flag-Ephrin A1 IP.tif]
