# Supplementary figures and images for: Ephrin A1 functions as a ligand of EGFR to promote EMT and metastasis in gastric cancer (part 3 of 5)
Source: EMBO J. 2025 Jan 21;44(5):1464–87. doi: 10.1038/s44318-025-00363-x (PMC11876641; doi:10.1038/s44318-025-00363-x)

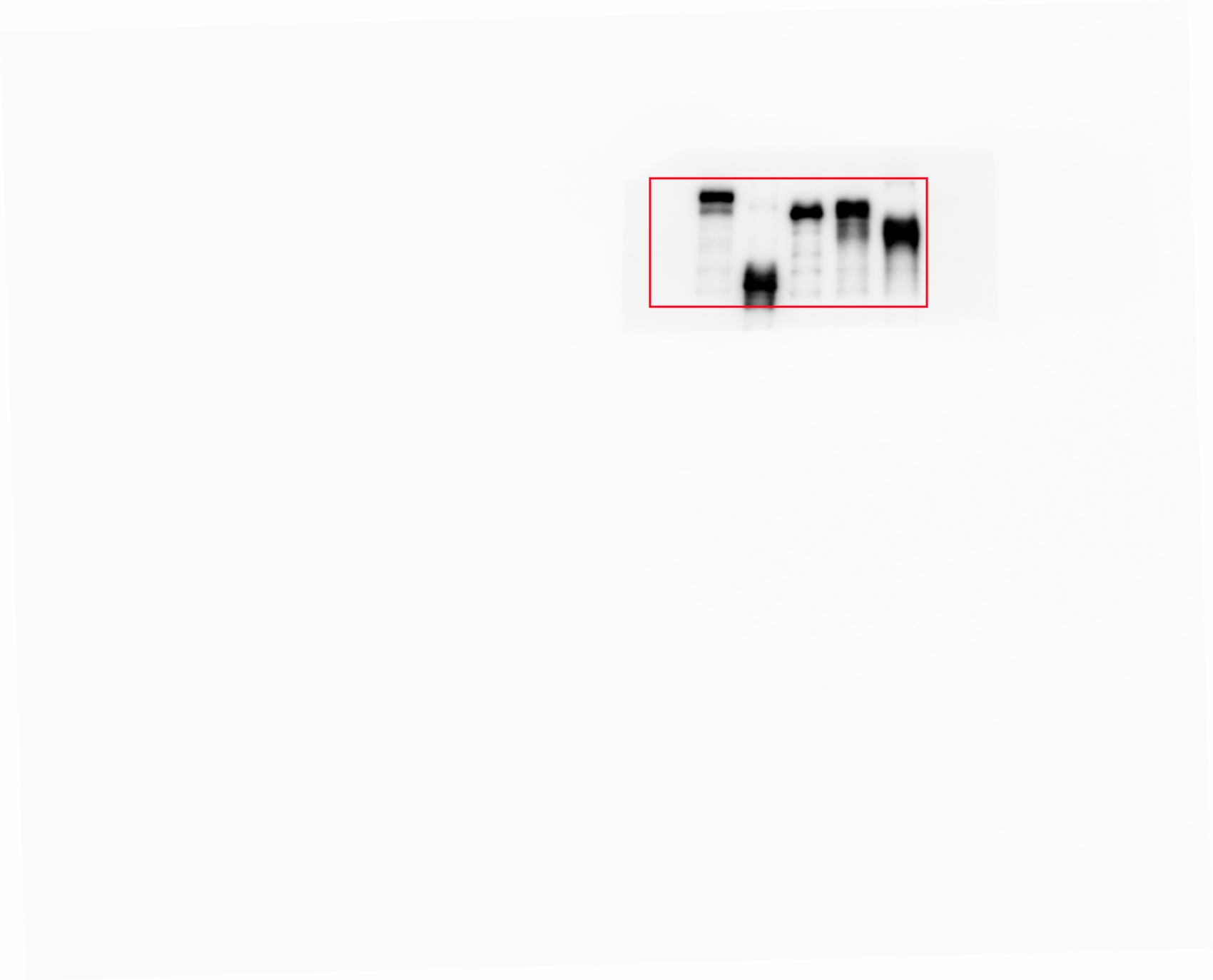

Supplement: Supplementary file 4 — Source data Fig. 3 [file 44318_2025_363_MOESM4_ESM.zip › Figure 3/3F/2 myc-EGFR IP.tif]

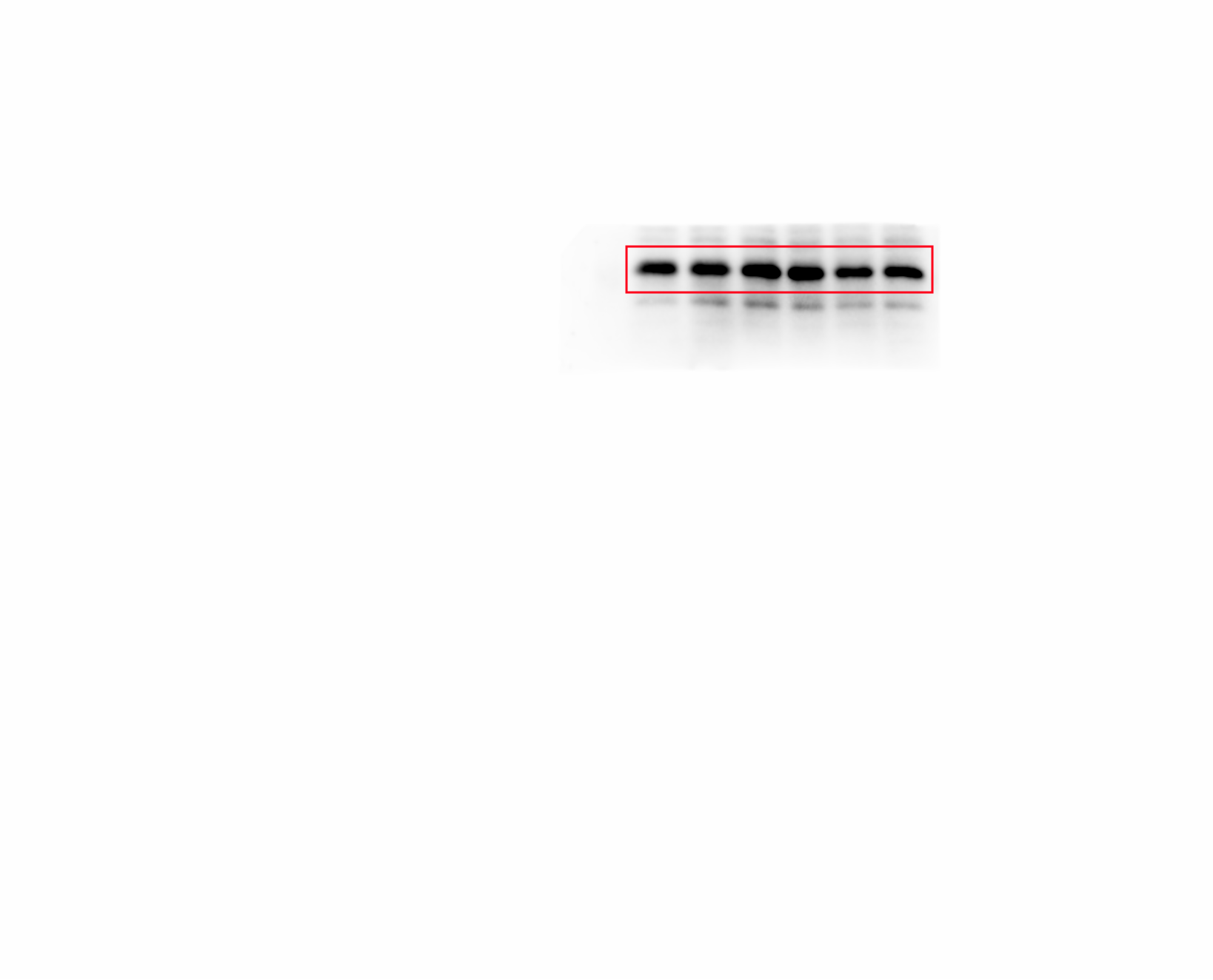

Supplement: Supplementary file 4 — Source data Fig. 3 [file 44318_2025_363_MOESM4_ESM.zip › Figure 3/3F/3 Flag-Ephrin A1 input.tif]

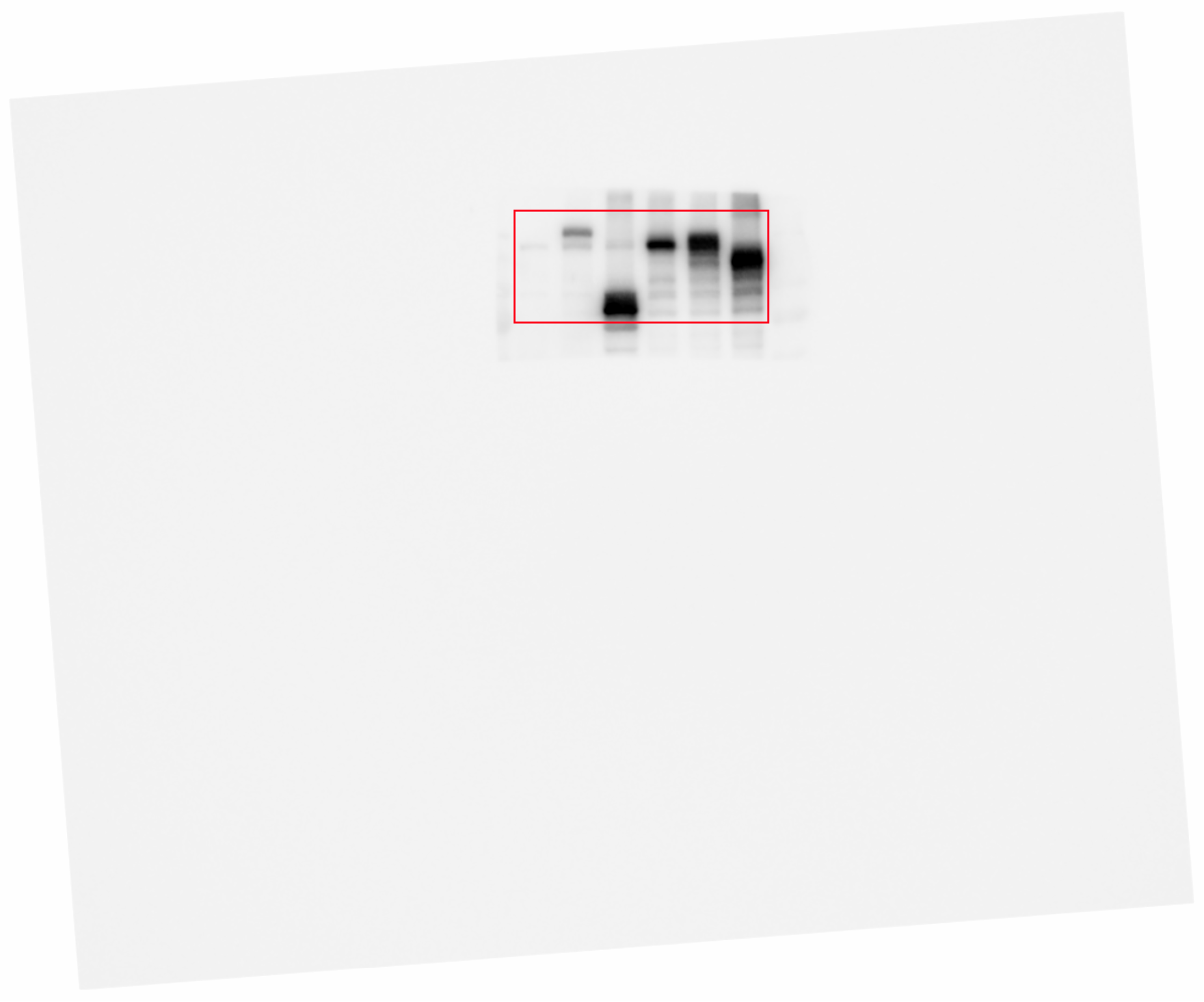

Supplement: Supplementary file 4 — Source data Fig. 3 [file 44318_2025_363_MOESM4_ESM.zip › Figure 3/3F/4 myc-EGFR input .tif]

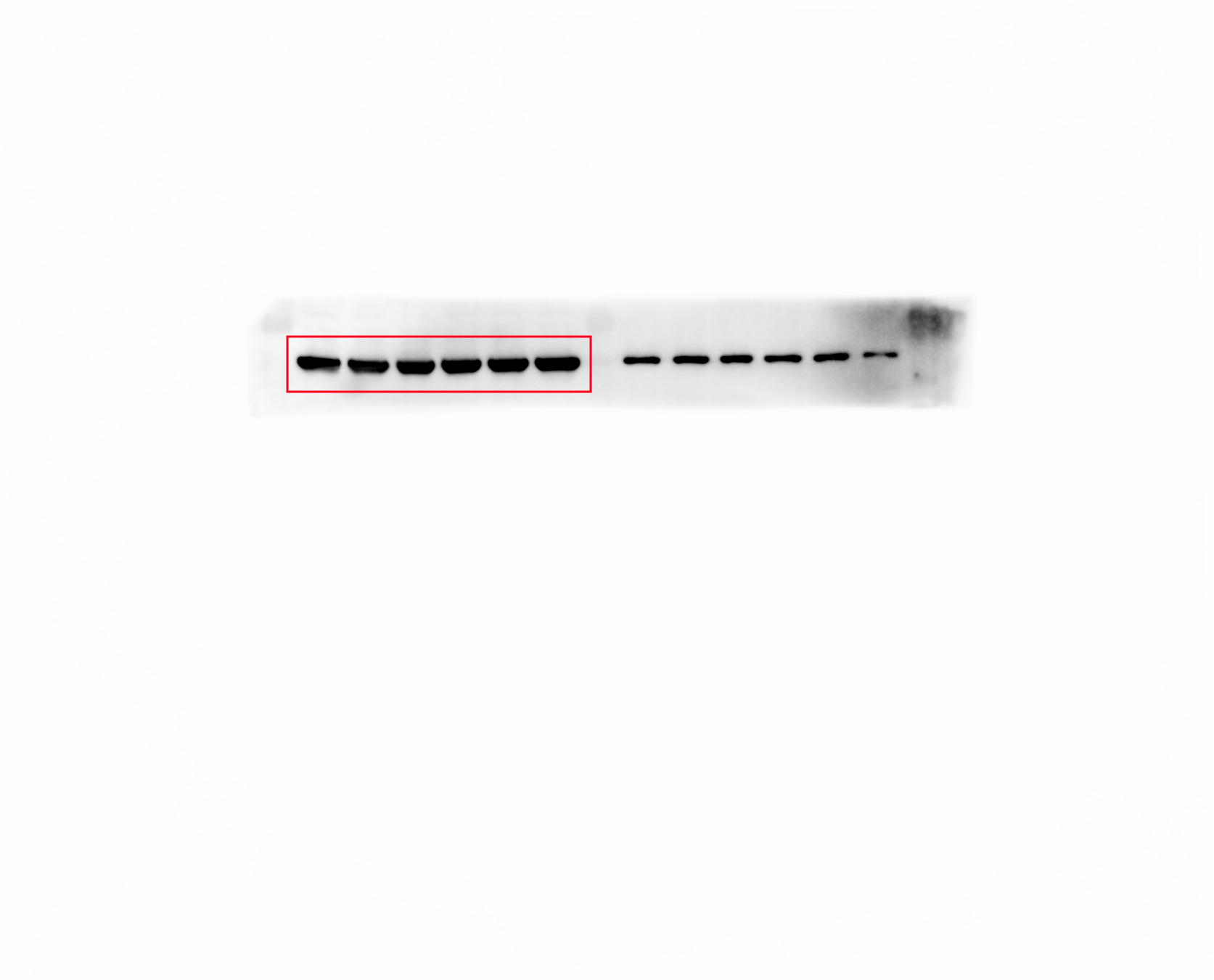

Supplement: Supplementary file 4 — Source data Fig. 3 [file 44318_2025_363_MOESM4_ESM.zip › Figure 3/3F/5 actin.tif]

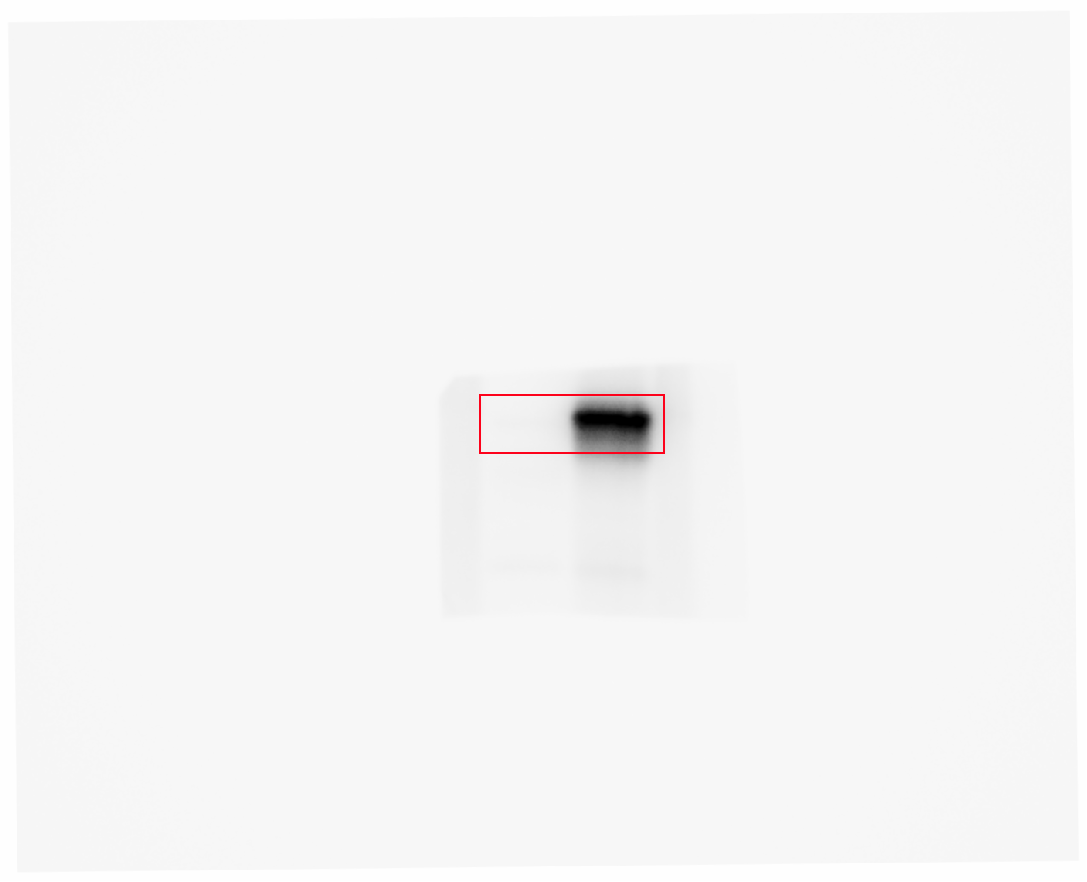

Supplement: Supplementary file 5 — Source data Fig. 4 [file 44318_2025_363_MOESM5_ESM.zip › Figure 4/4A/1 pulldown Ephrin A1.tif]

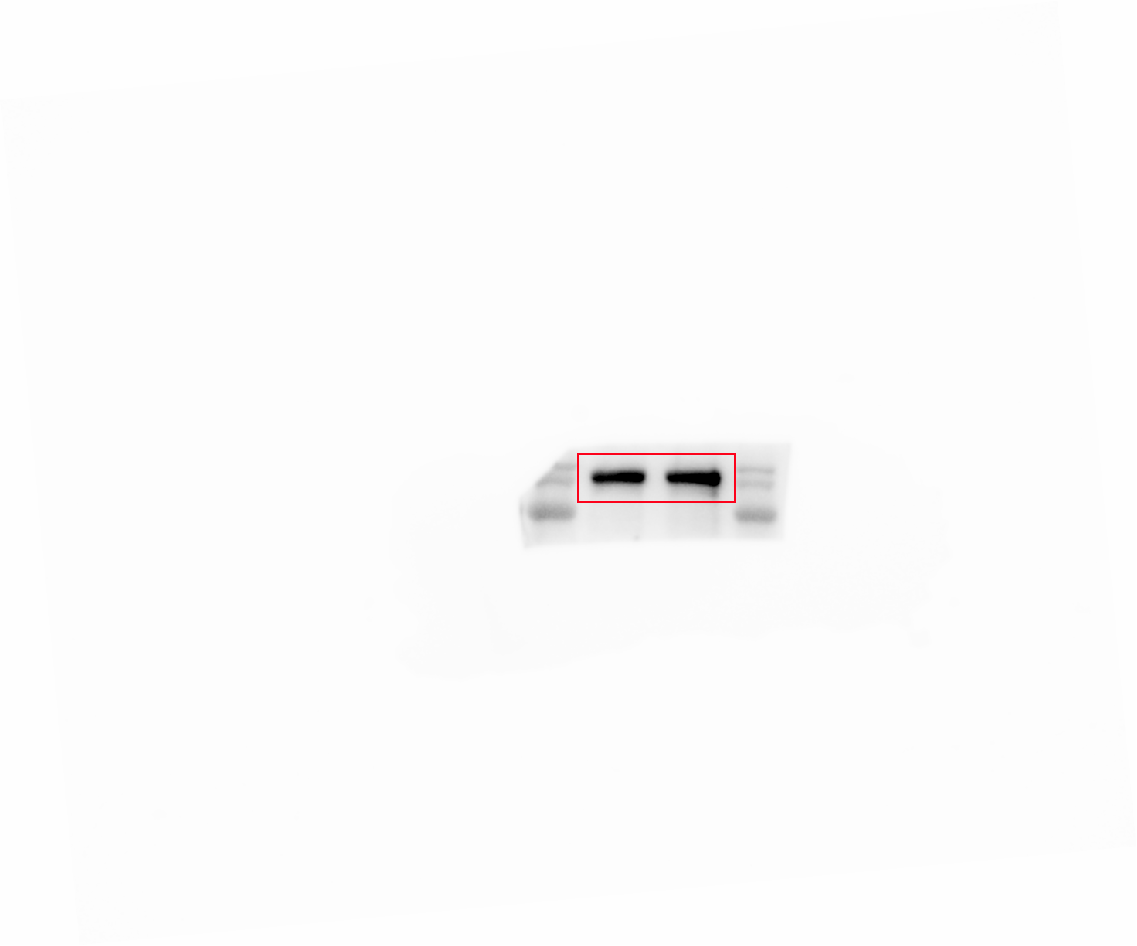

Supplement: Supplementary file 5 — Source data Fig. 4 [file 44318_2025_363_MOESM5_ESM.zip › Figure 4/4A/2 pull down EGFR.tif]

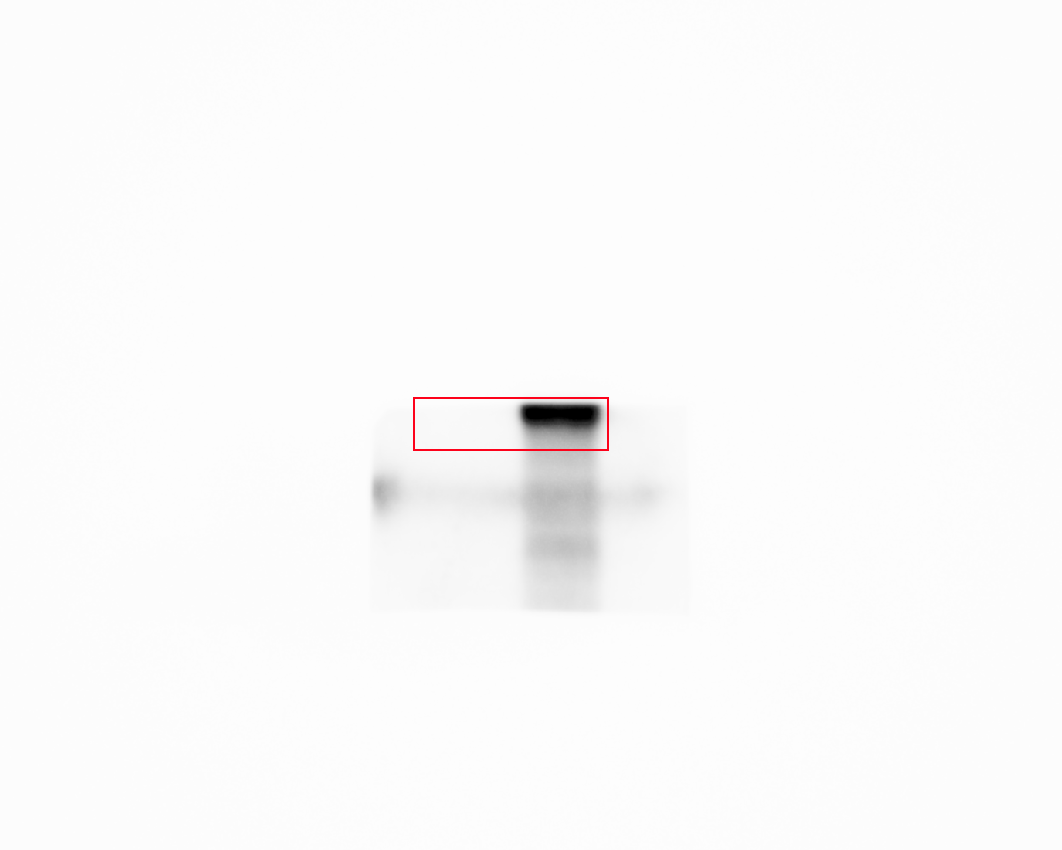

Supplement: Supplementary file 5 — Source data Fig. 4 [file 44318_2025_363_MOESM5_ESM.zip › Figure 4/4A/3 input Ephrin A1.tif]

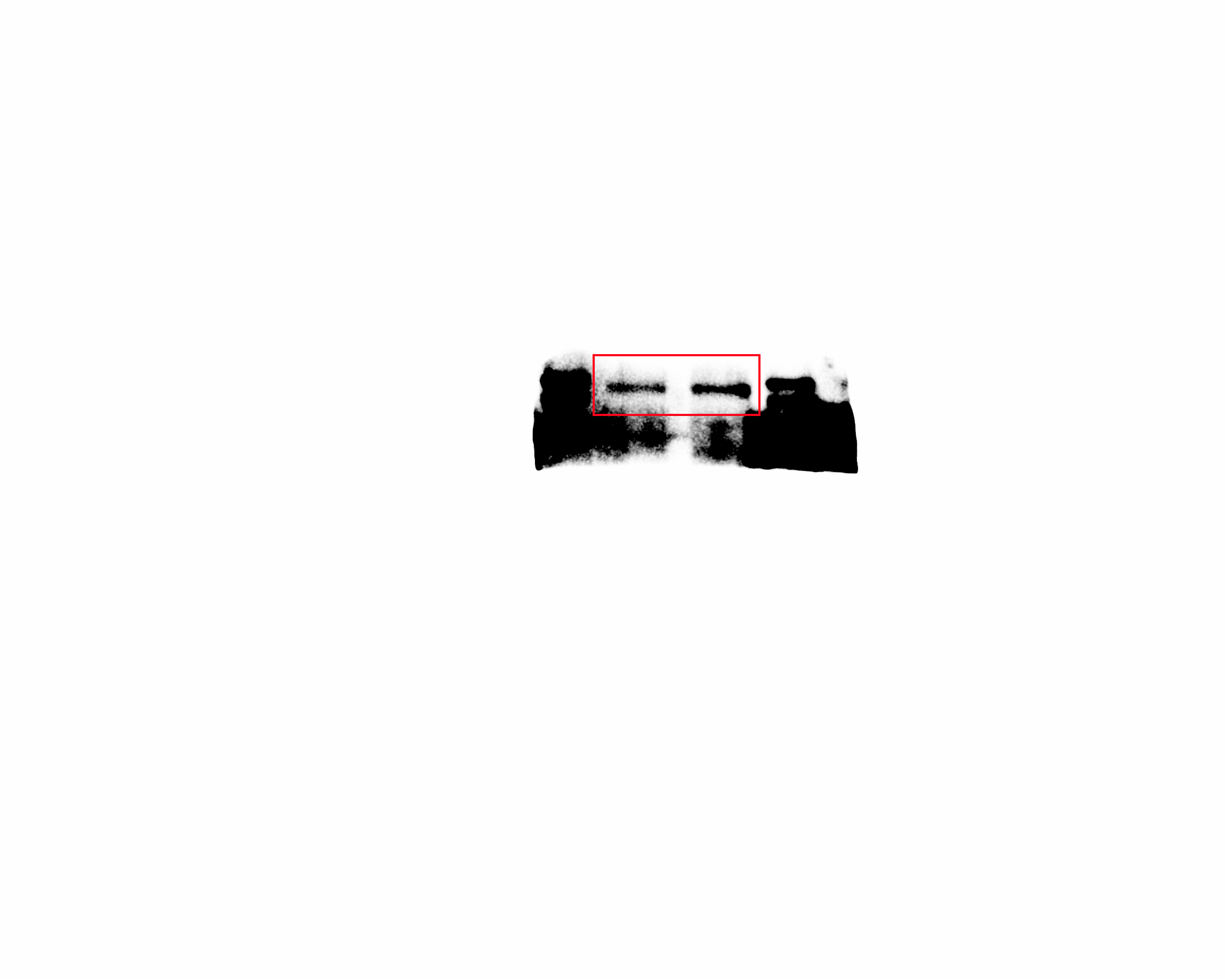

Supplement: Supplementary file 5 — Source data Fig. 4 [file 44318_2025_363_MOESM5_ESM.zip › Figure 4/4A/4 input EGFR.tif]

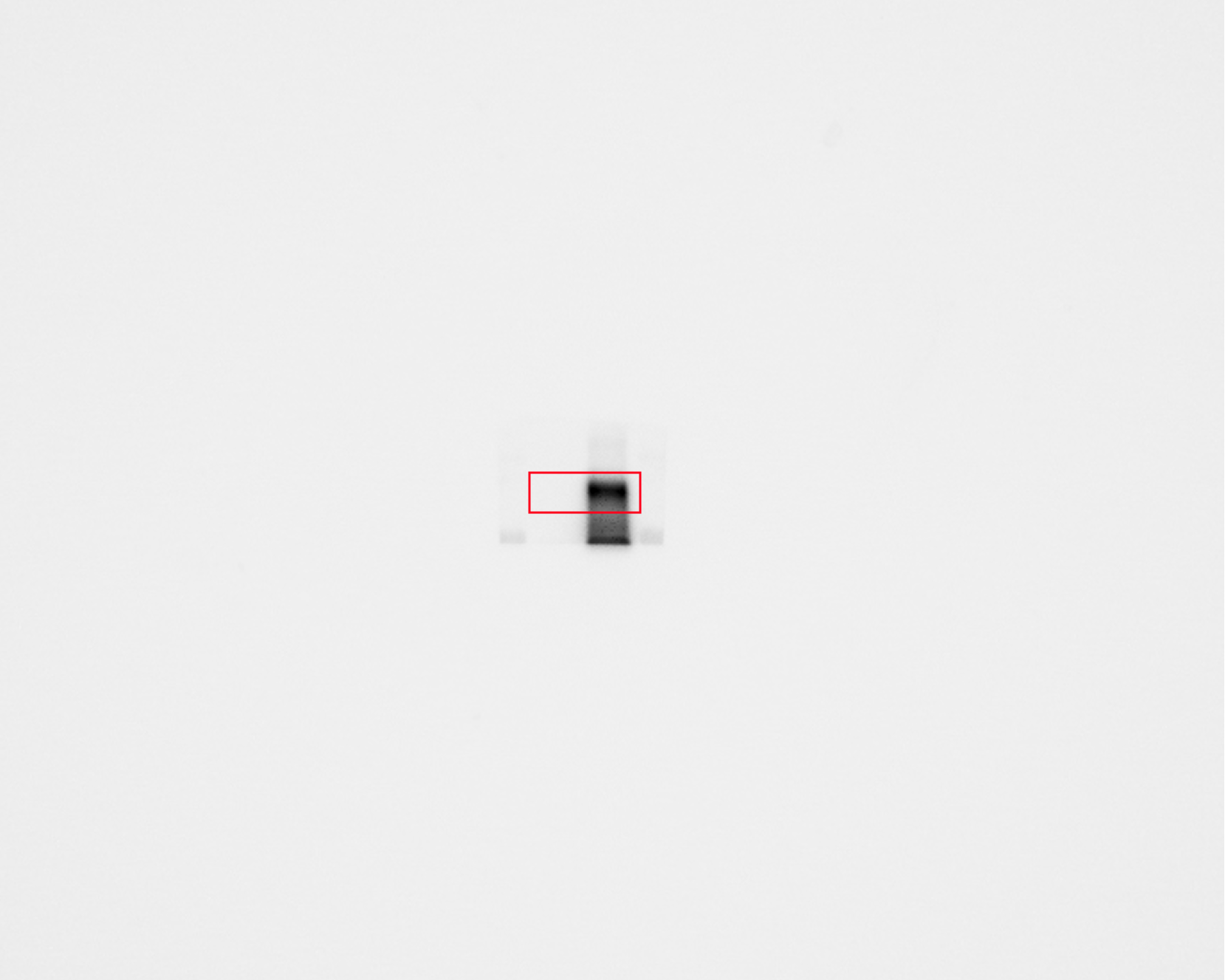

Supplement: Supplementary file 5 — Source data Fig. 4 [file 44318_2025_363_MOESM5_ESM.zip › Figure 4/4B/1 pulldown EGFR.tif]

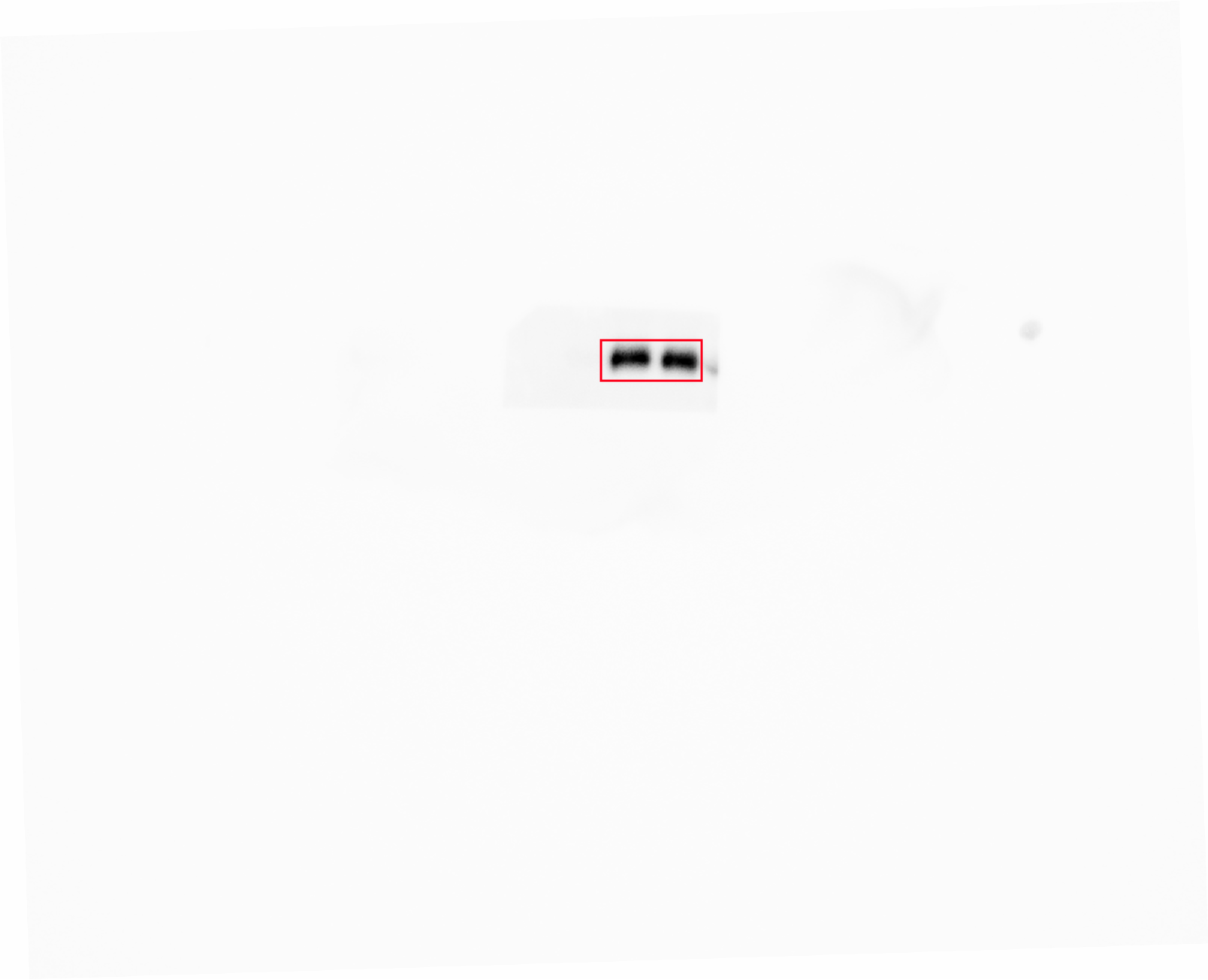

Supplement: Supplementary file 5 — Source data Fig. 4 [file 44318_2025_363_MOESM5_ESM.zip › Figure 4/4B/2 pulldown Ephrin A1.tif]

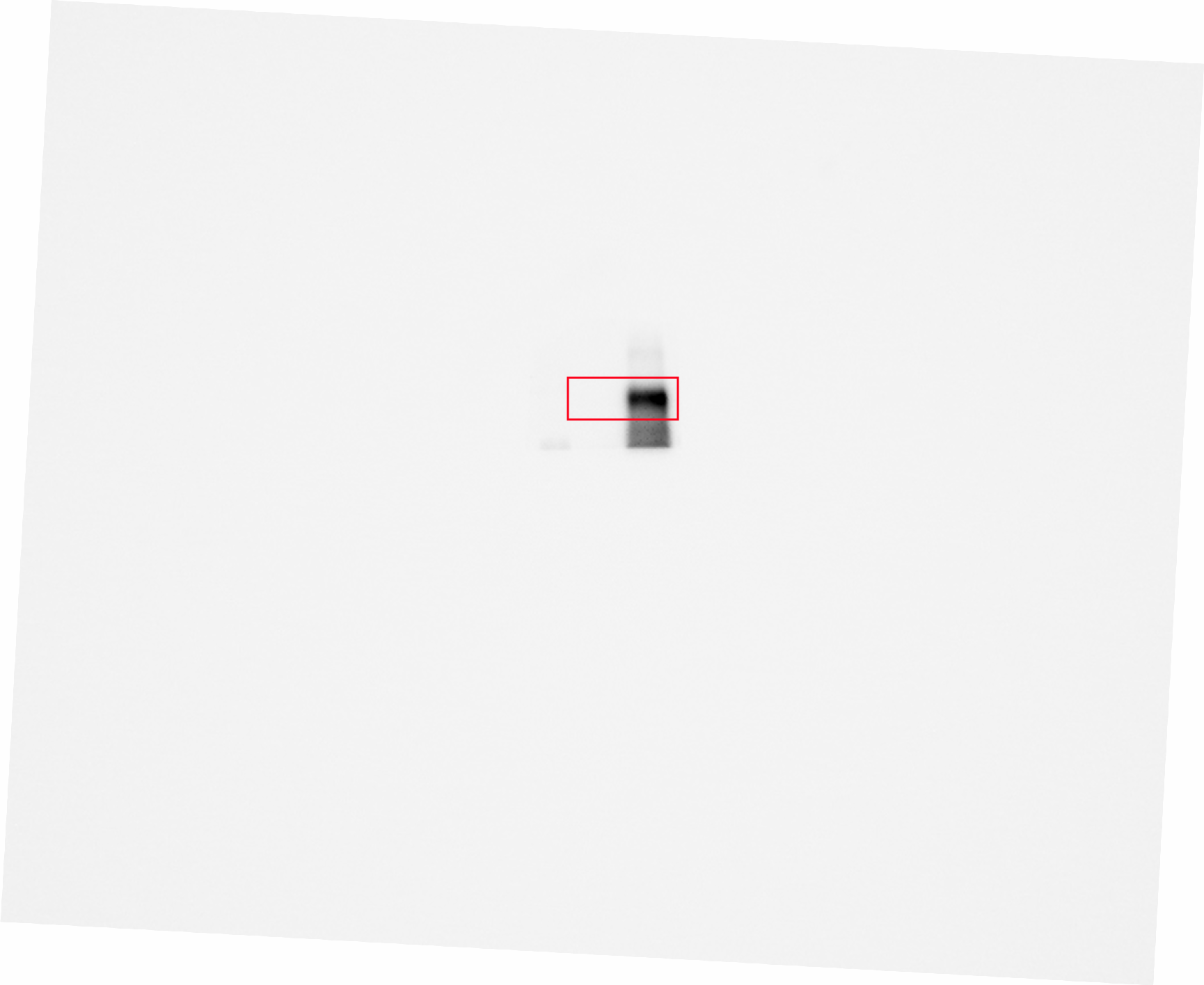

Supplement: Supplementary file 5 — Source data Fig. 4 [file 44318_2025_363_MOESM5_ESM.zip › Figure 4/4B/3 input EGFR.tif]

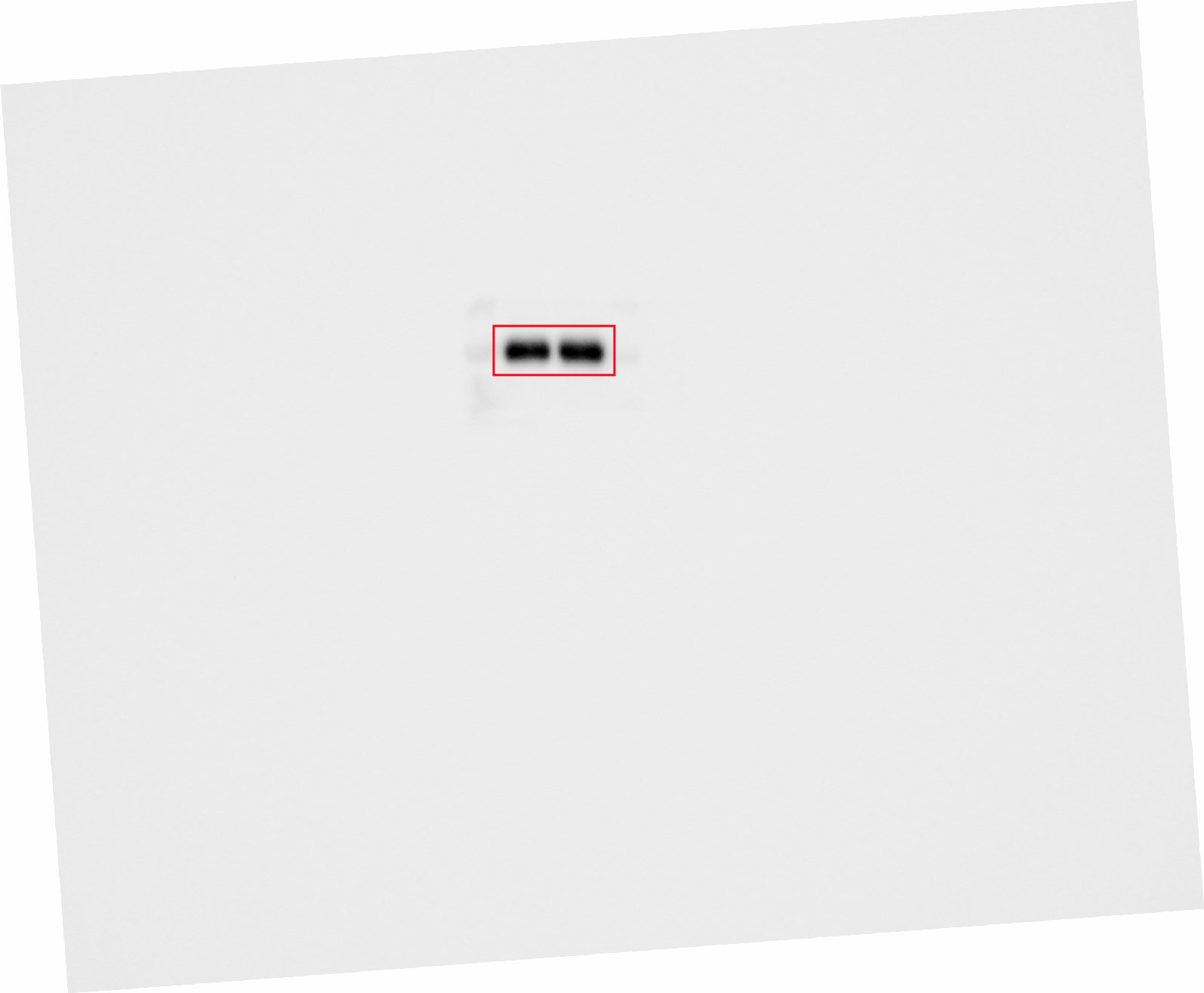

Supplement: Supplementary file 5 — Source data Fig. 4 [file 44318_2025_363_MOESM5_ESM.zip › Figure 4/4B/4 input Ephrin A1.tif]

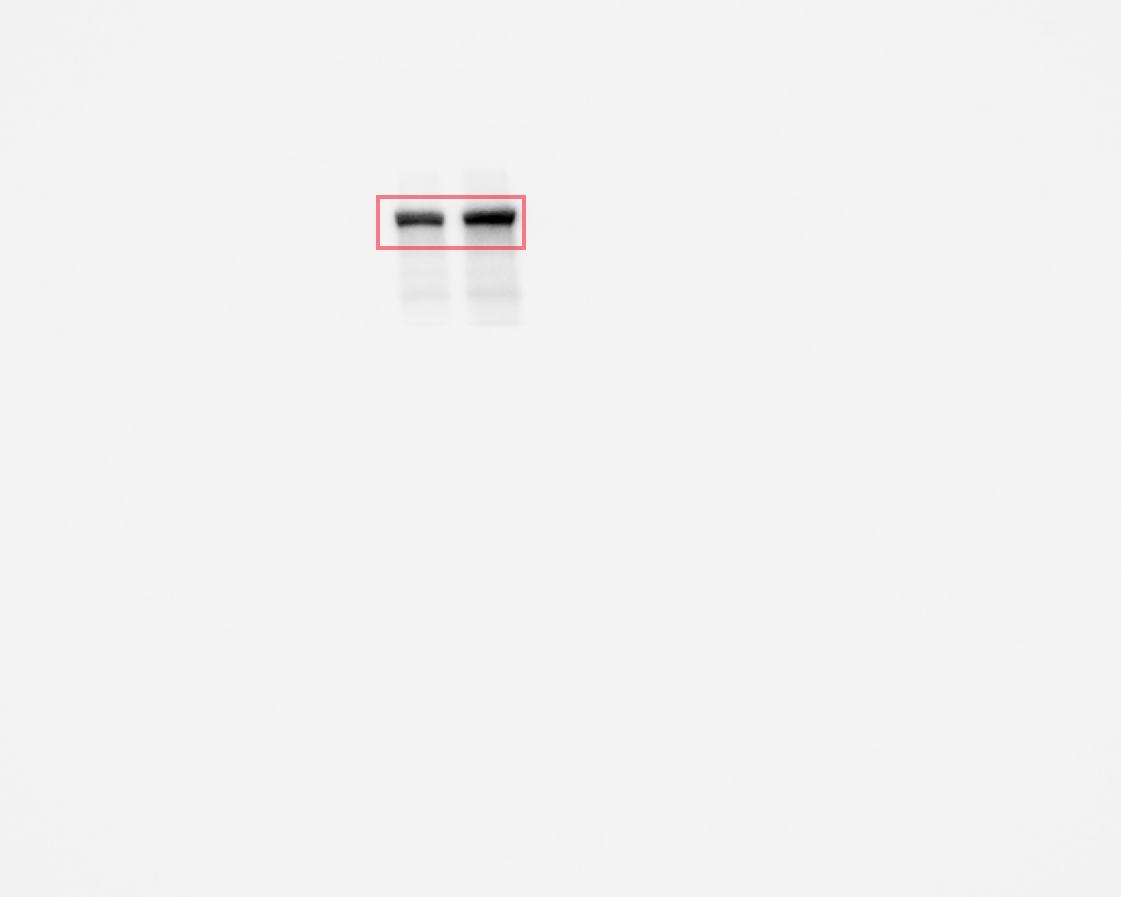

Supplement: Supplementary file 5 — Source data Fig. 4 [file 44318_2025_363_MOESM5_ESM.zip › Figure 4/4C/1 P-EGFR.tif]

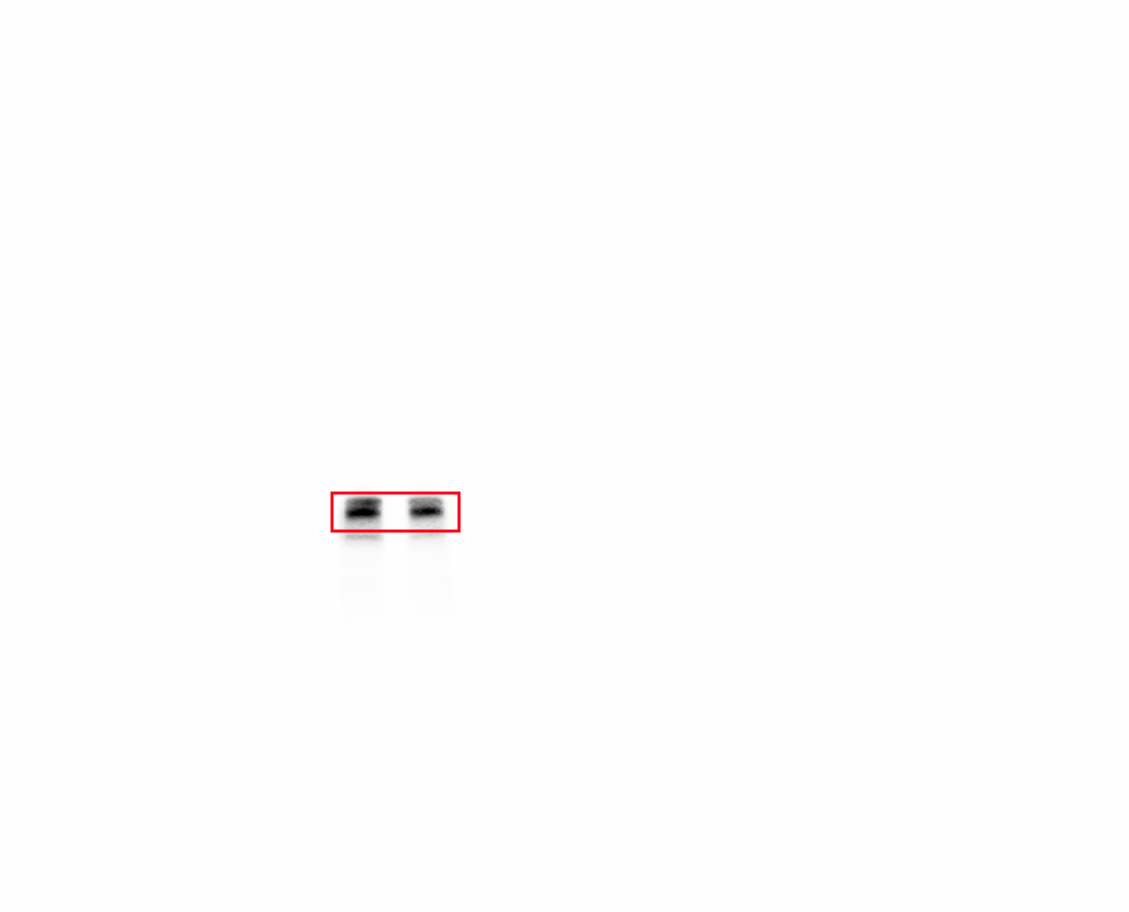

Supplement: Supplementary file 5 — Source data Fig. 4 [file 44318_2025_363_MOESM5_ESM.zip › Figure 4/4C/2 EGFR.tif]

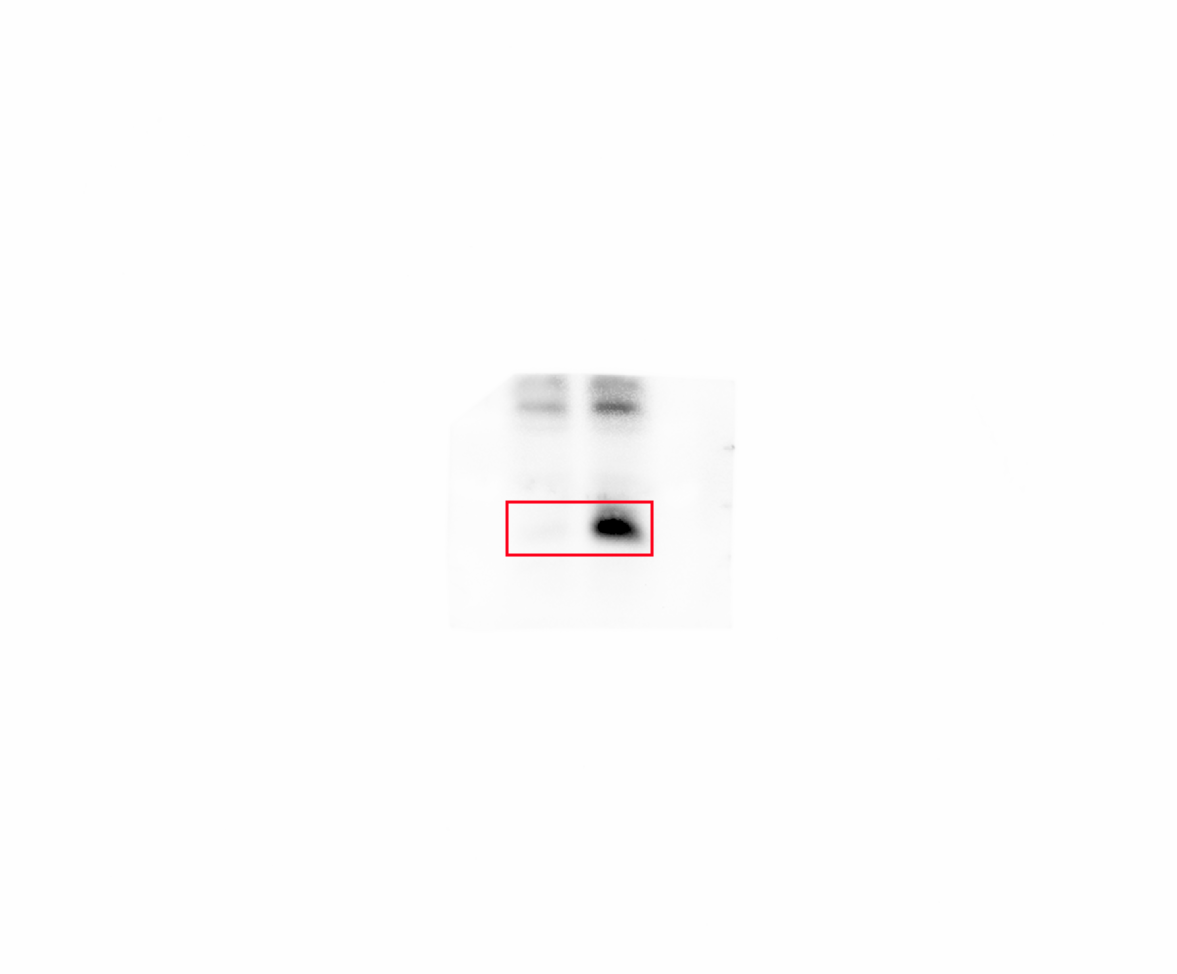

Supplement: Supplementary file 5 — Source data Fig. 4 [file 44318_2025_363_MOESM5_ESM.zip › Figure 4/4C/3 Ephrin A1.tif]

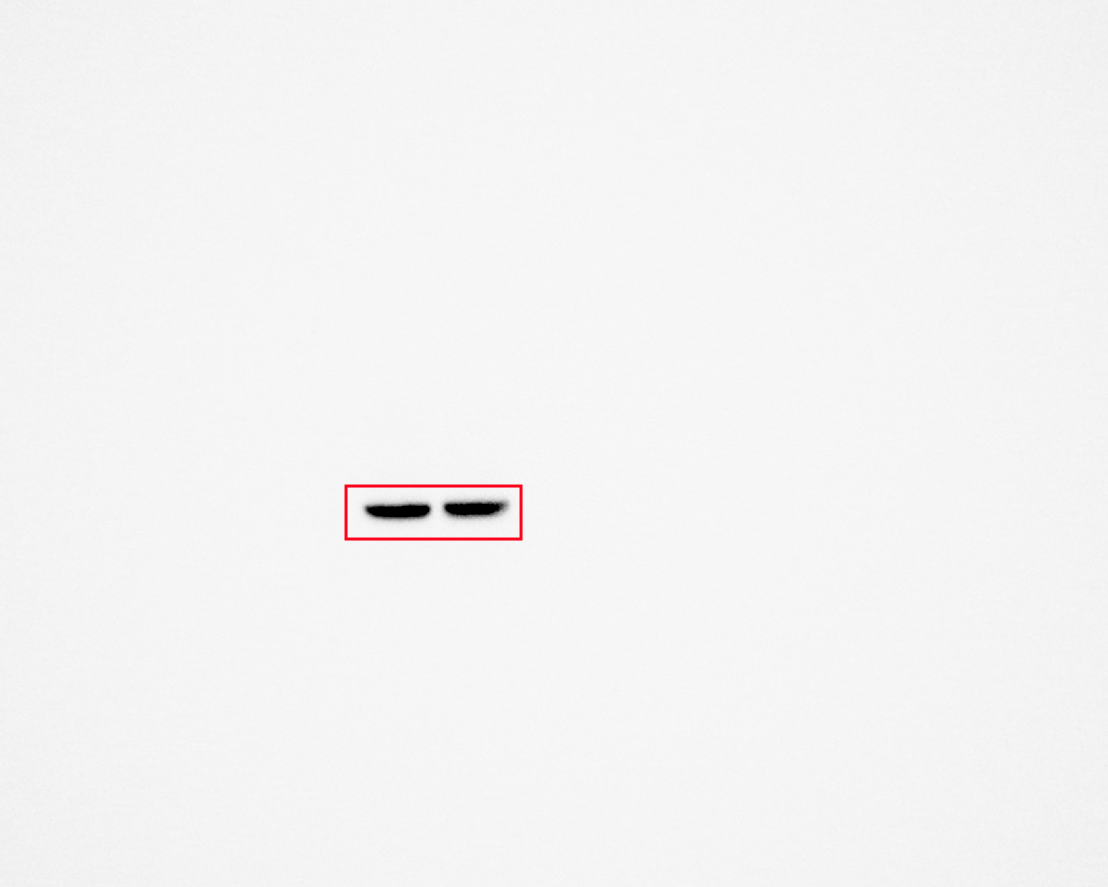

Supplement: Supplementary file 5 — Source data Fig. 4 [file 44318_2025_363_MOESM5_ESM.zip › Figure 4/4C/4 actin.tif]

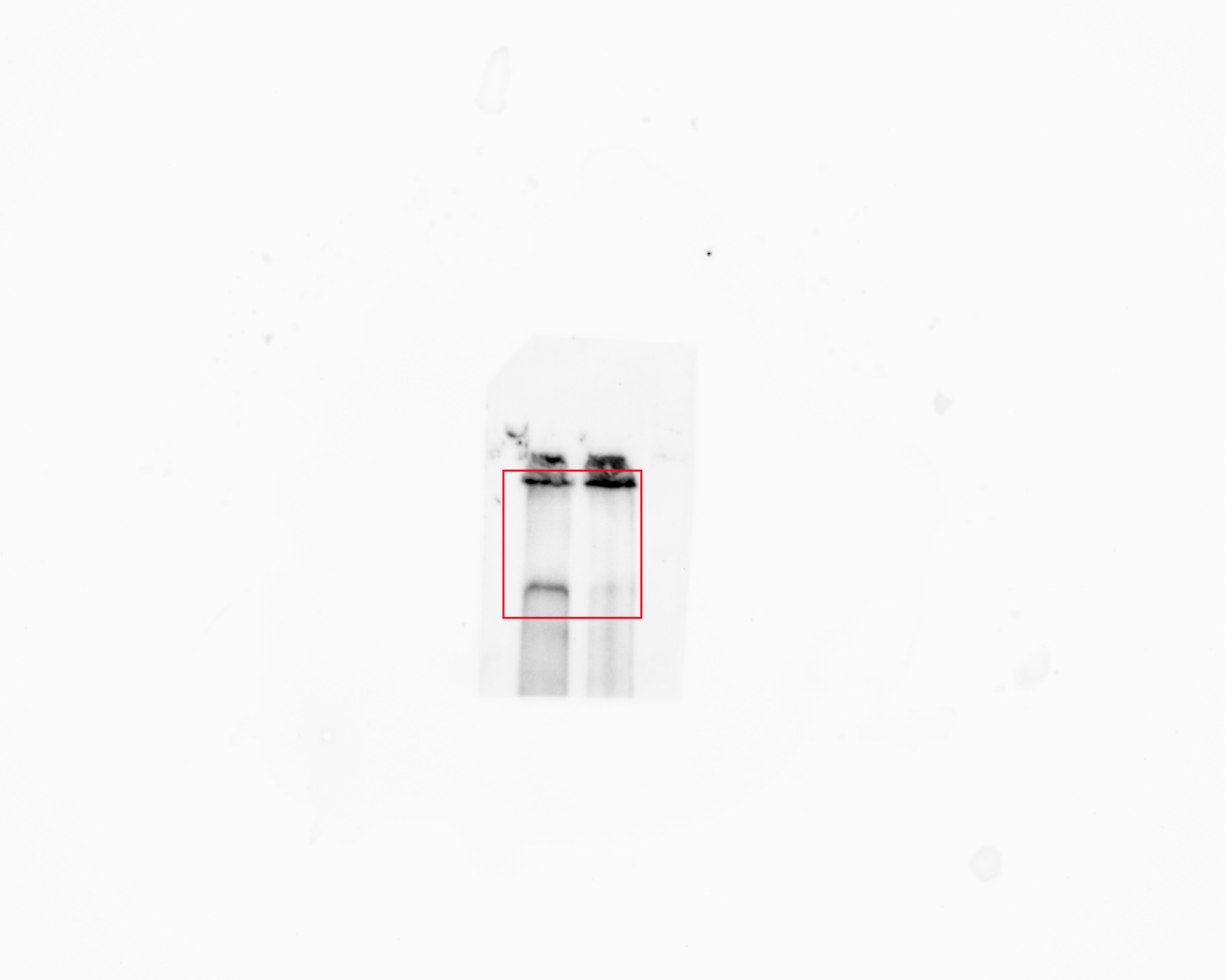

Supplement: Supplementary file 5 — Source data Fig. 4 [file 44318_2025_363_MOESM5_ESM.zip › Figure 4/4D/dimer.tif]

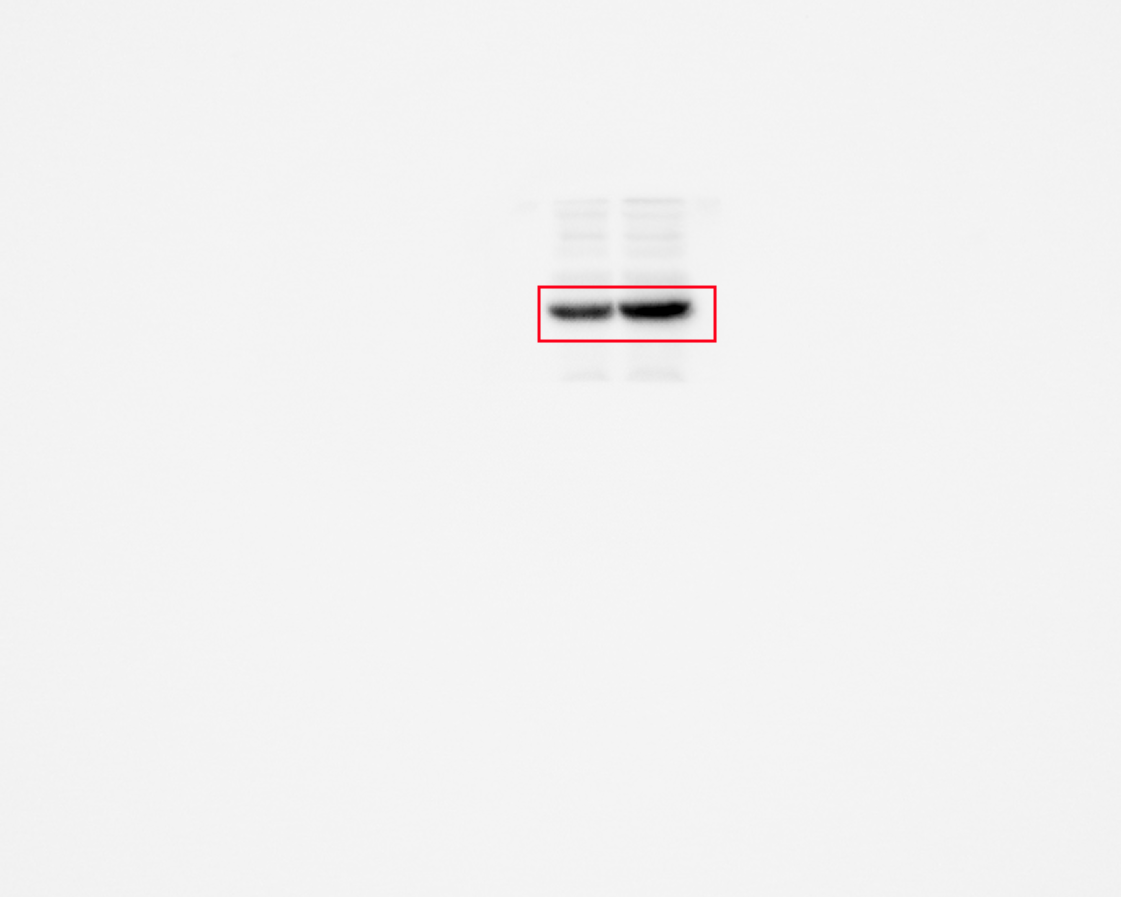

Supplement: Supplementary file 5 — Source data Fig. 4 [file 44318_2025_363_MOESM5_ESM.zip › Figure 4/4E/1 P-ERK.tif]

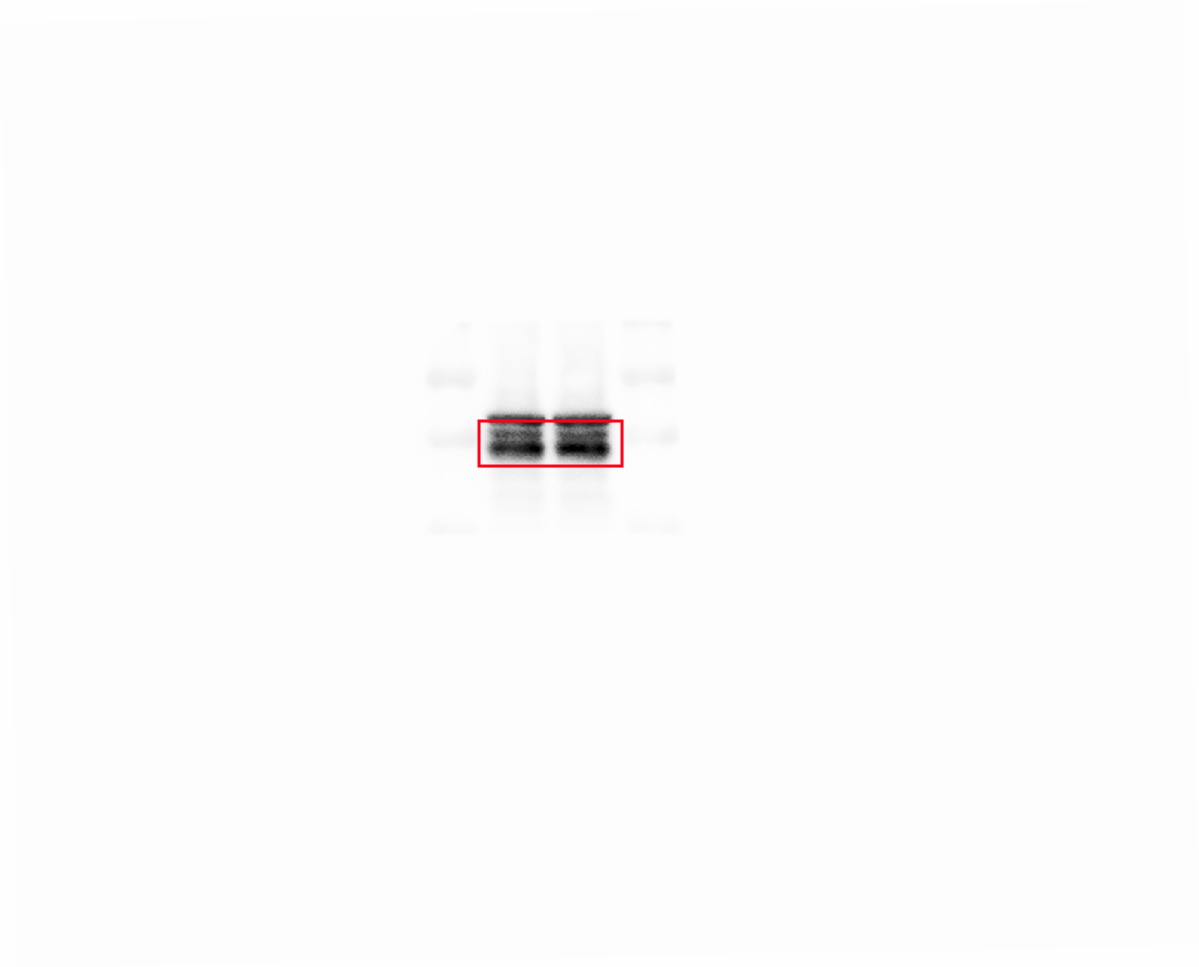

Supplement: Supplementary file 5 — Source data Fig. 4 [file 44318_2025_363_MOESM5_ESM.zip › Figure 4/4E/2 ERK.tif]

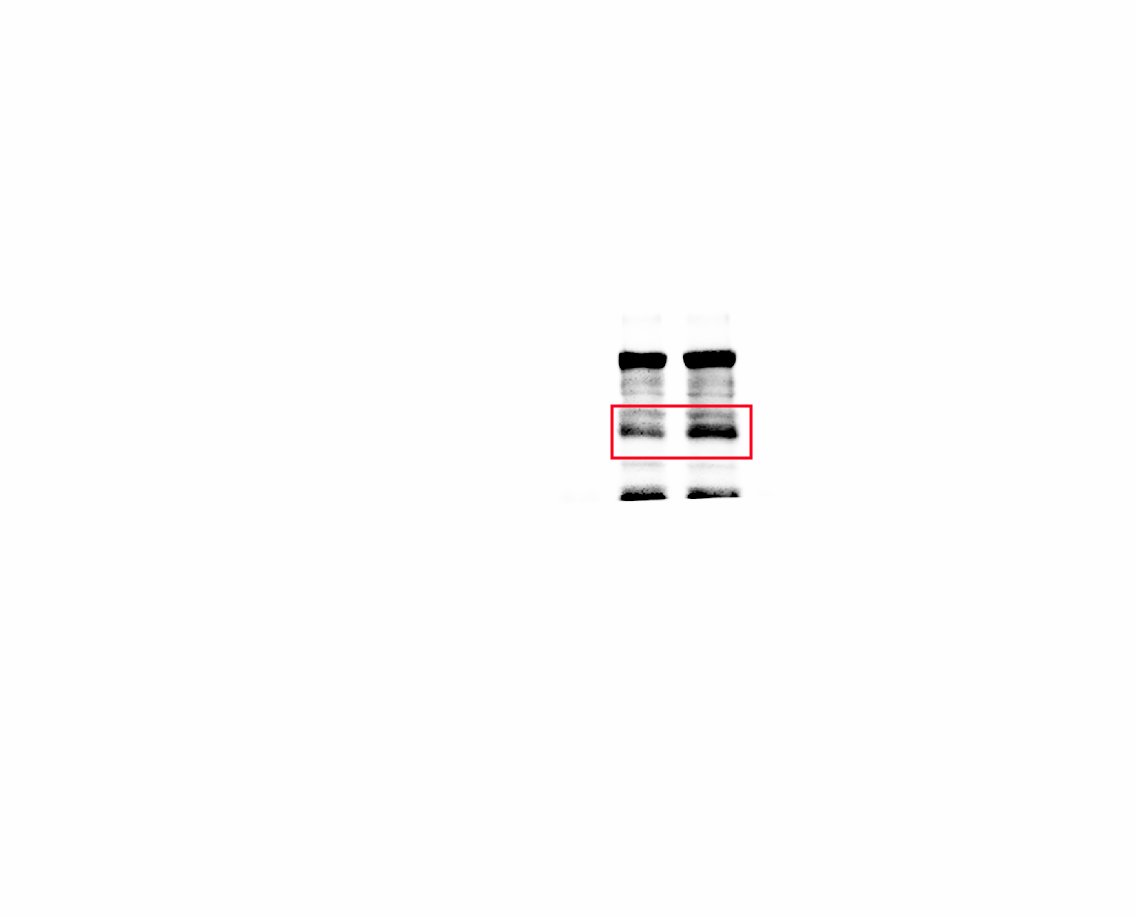

Supplement: Supplementary file 5 — Source data Fig. 4 [file 44318_2025_363_MOESM5_ESM.zip › Figure 4/4E/3 P-FAK.tif]

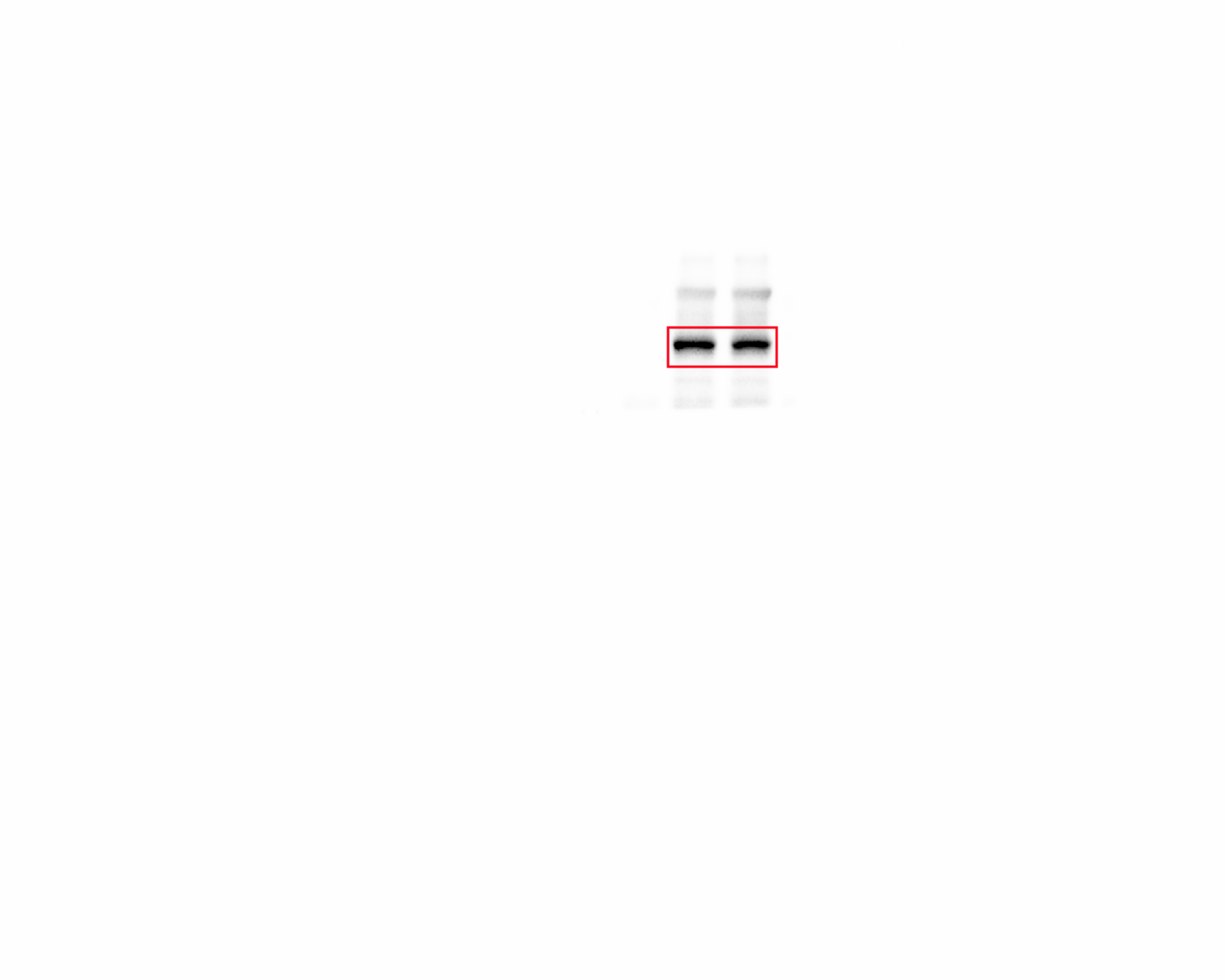

Supplement: Supplementary file 5 — Source data Fig. 4 [file 44318_2025_363_MOESM5_ESM.zip › Figure 4/4E/4 FAK.tif]

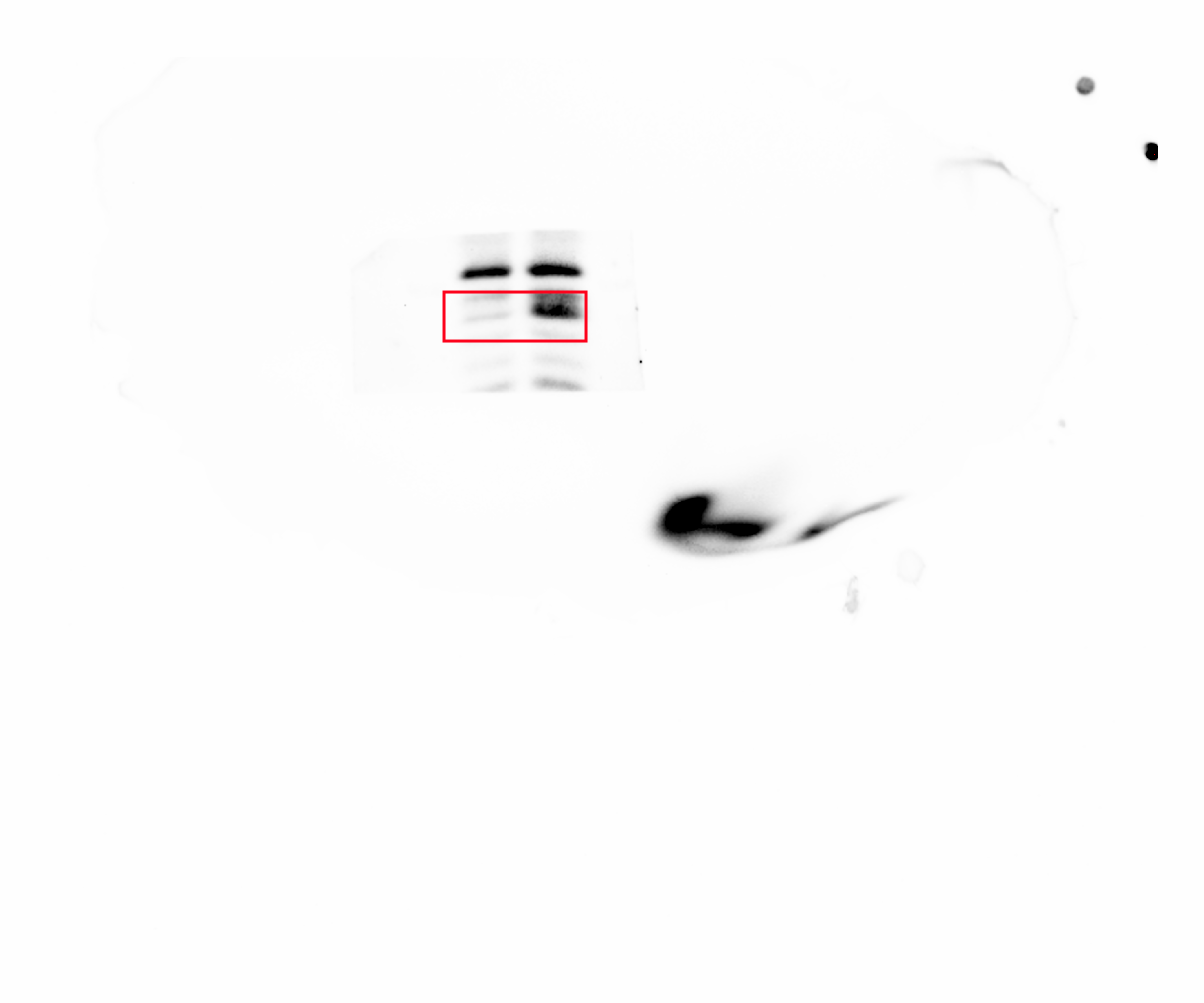

Supplement: Supplementary file 5 — Source data Fig. 4 [file 44318_2025_363_MOESM5_ESM.zip › Figure 4/4E/5 Ephrin A1.tif]

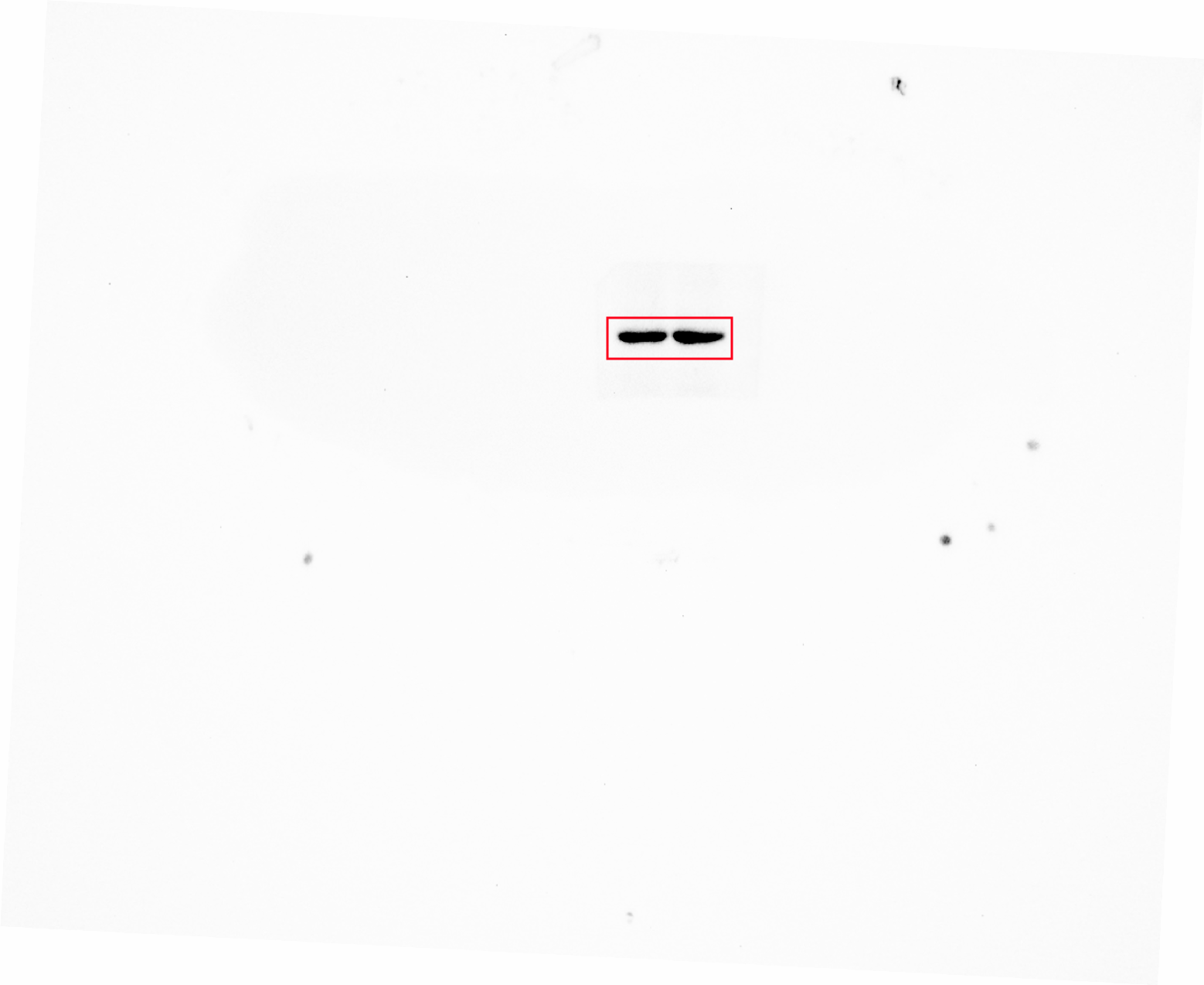

Supplement: Supplementary file 5 — Source data Fig. 4 [file 44318_2025_363_MOESM5_ESM.zip › Figure 4/4E/6 actin.tif]

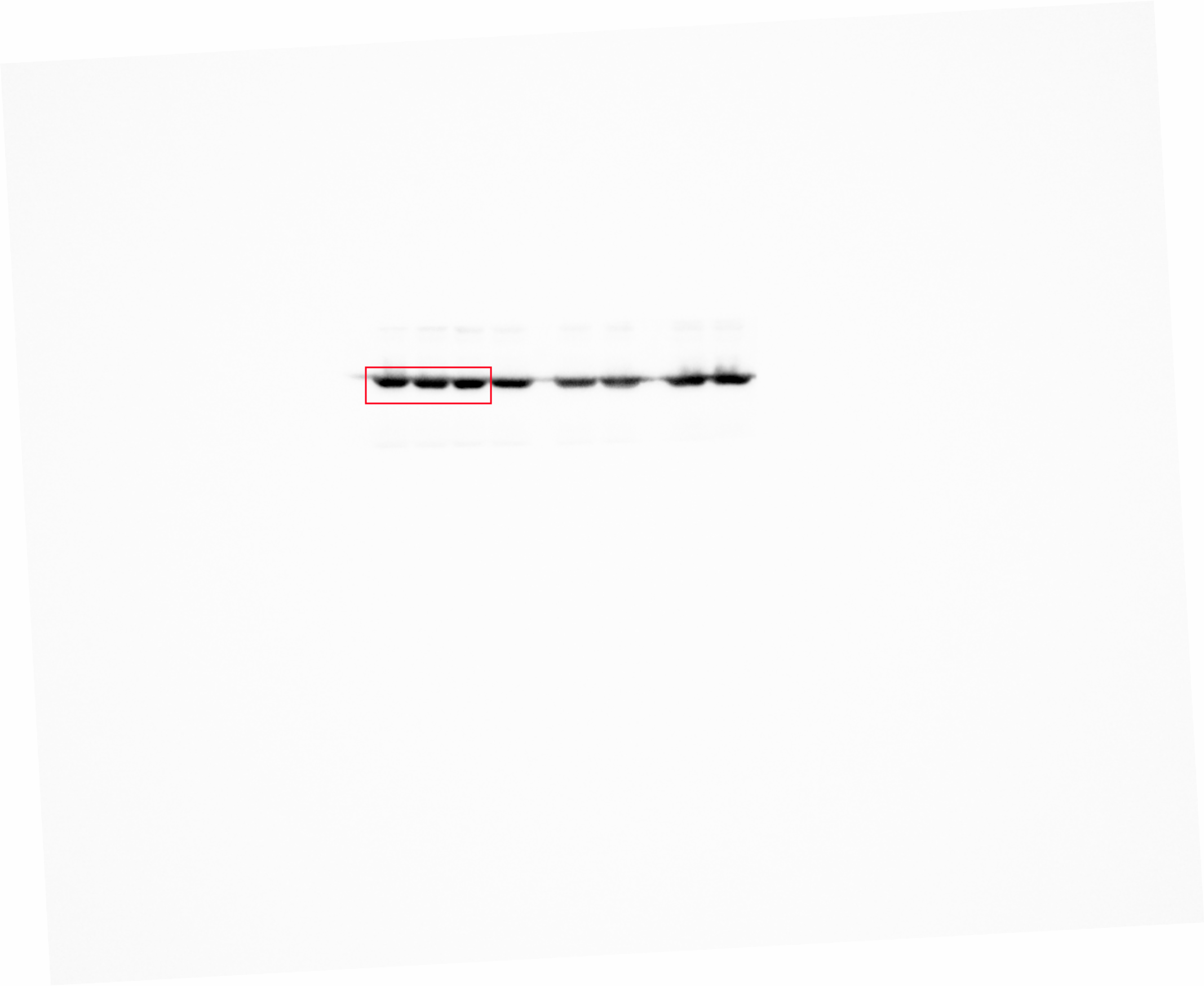

Supplement: Supplementary file 5 — Source data Fig. 4 [file 44318_2025_363_MOESM5_ESM.zip › Figure 4/4F/actin (4).tif]

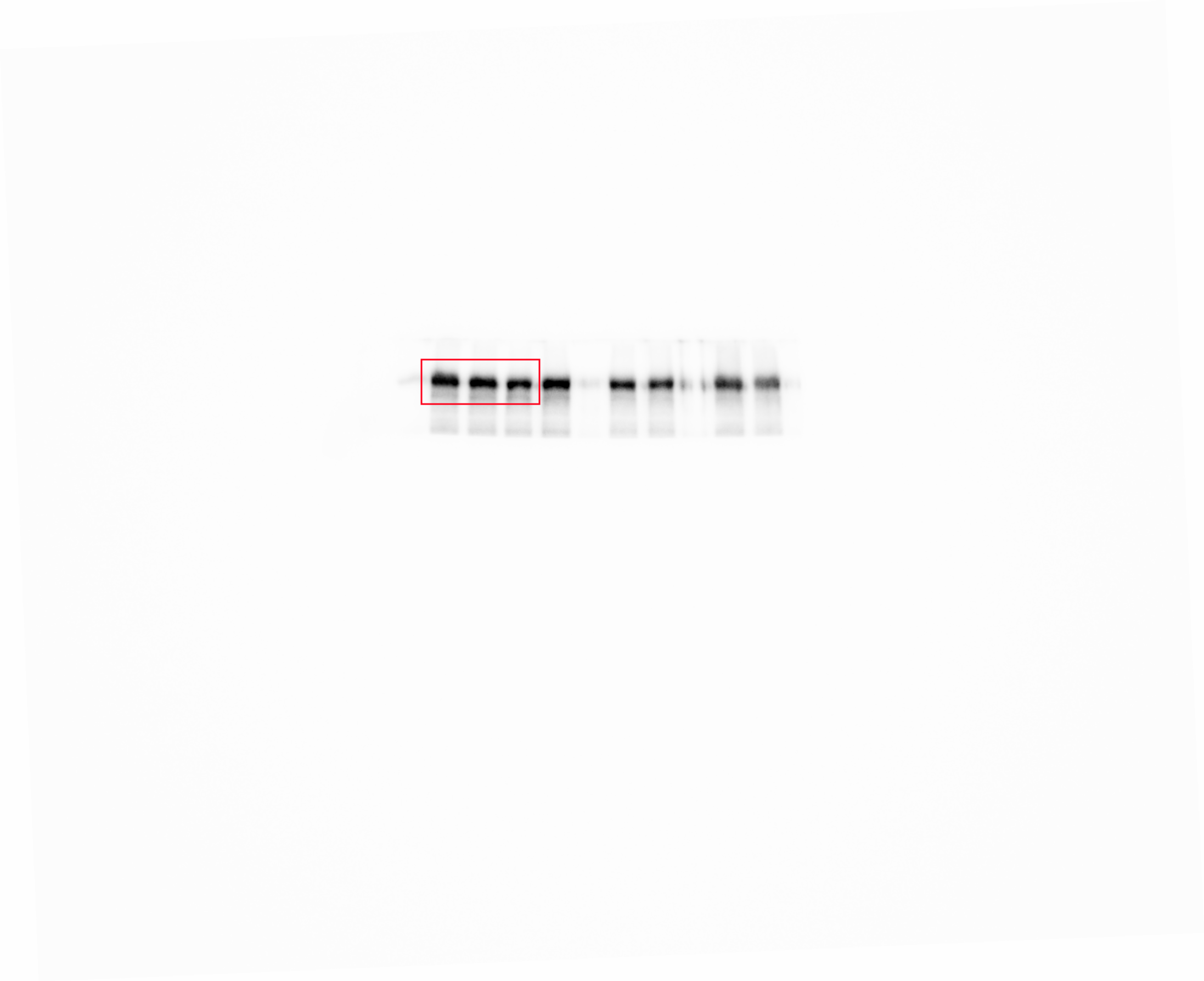

Supplement: Supplementary file 5 — Source data Fig. 4 [file 44318_2025_363_MOESM5_ESM.zip › Figure 4/4F/EGFR (2).tif]

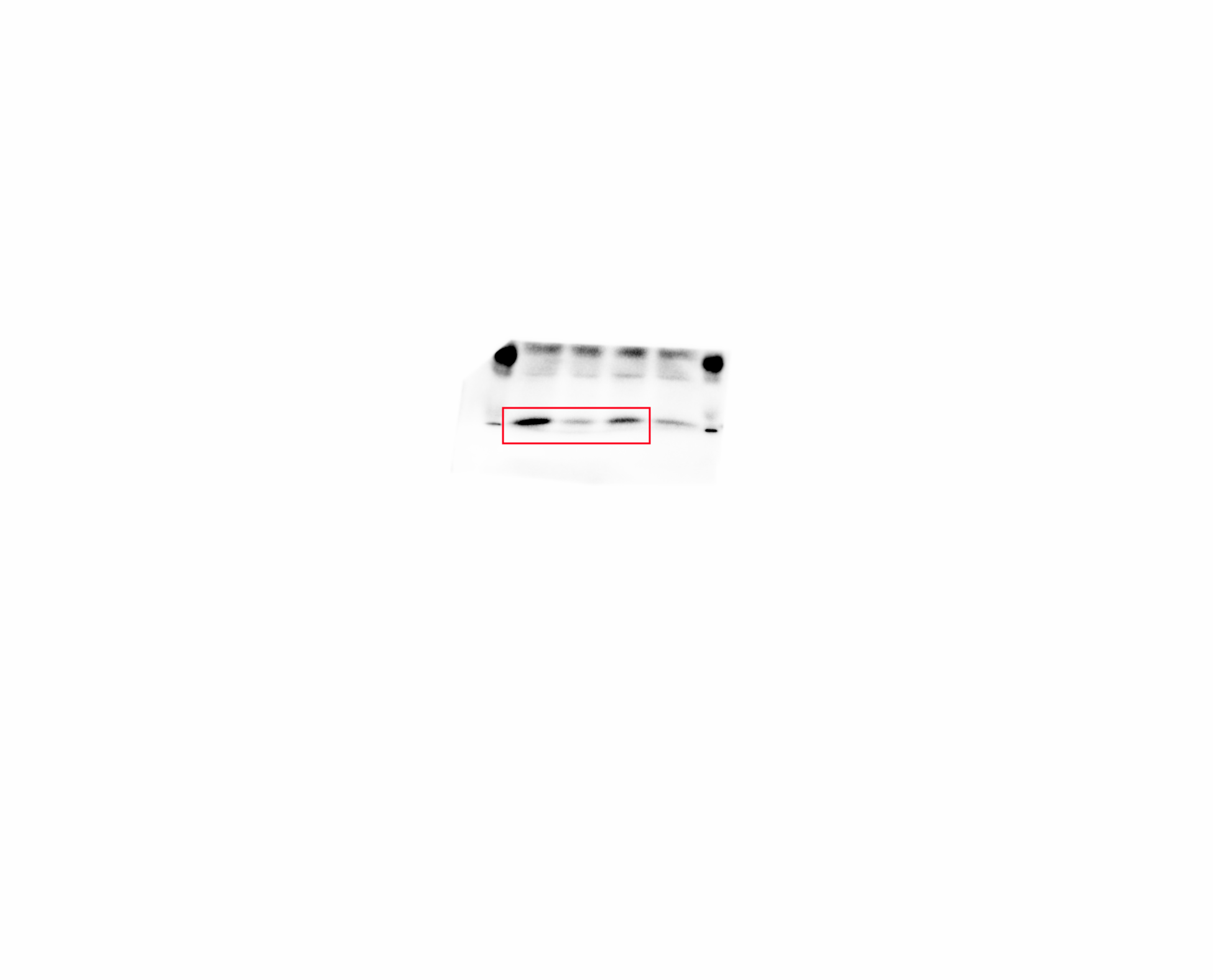

Supplement: Supplementary file 5 — Source data Fig. 4 [file 44318_2025_363_MOESM5_ESM.zip › Figure 4/4F/EphrinA1-si.tif]

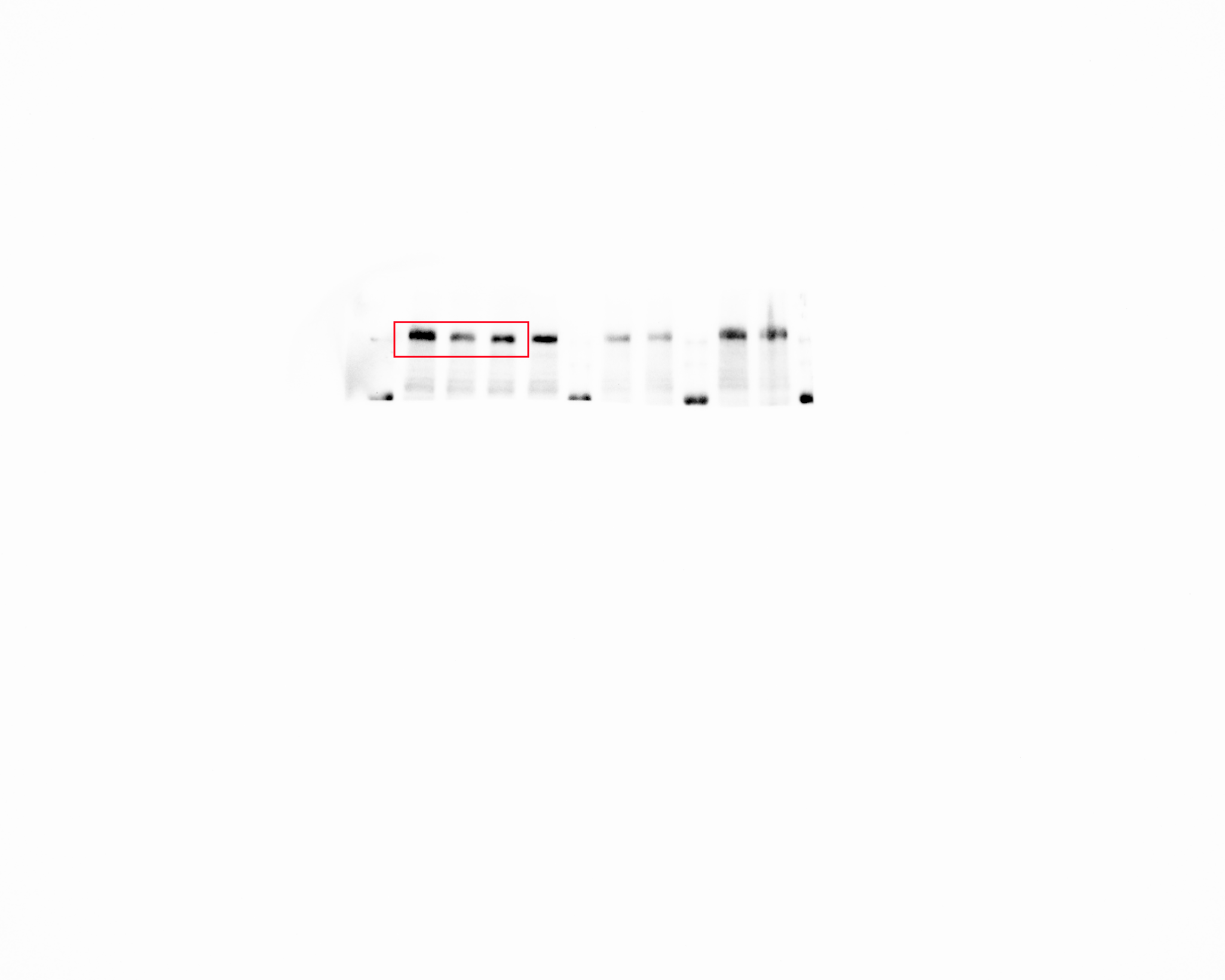

Supplement: Supplementary file 5 — Source data Fig. 4 [file 44318_2025_363_MOESM5_ESM.zip › Figure 4/4F/p-EGFR.tif]

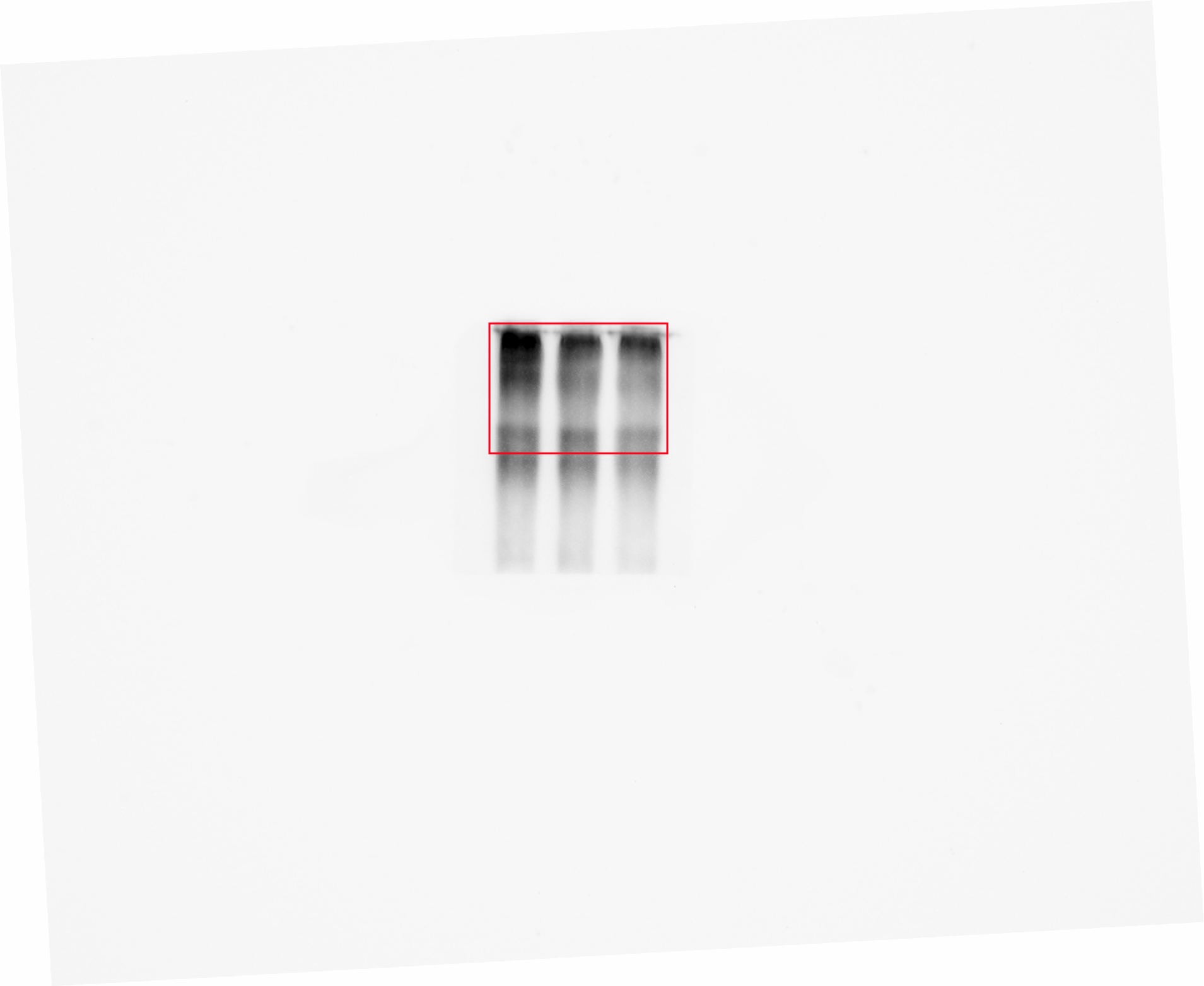

Supplement: Supplementary file 5 — Source data Fig. 4 [file 44318_2025_363_MOESM5_ESM.zip › Figure 4/4G/dimer.tif]

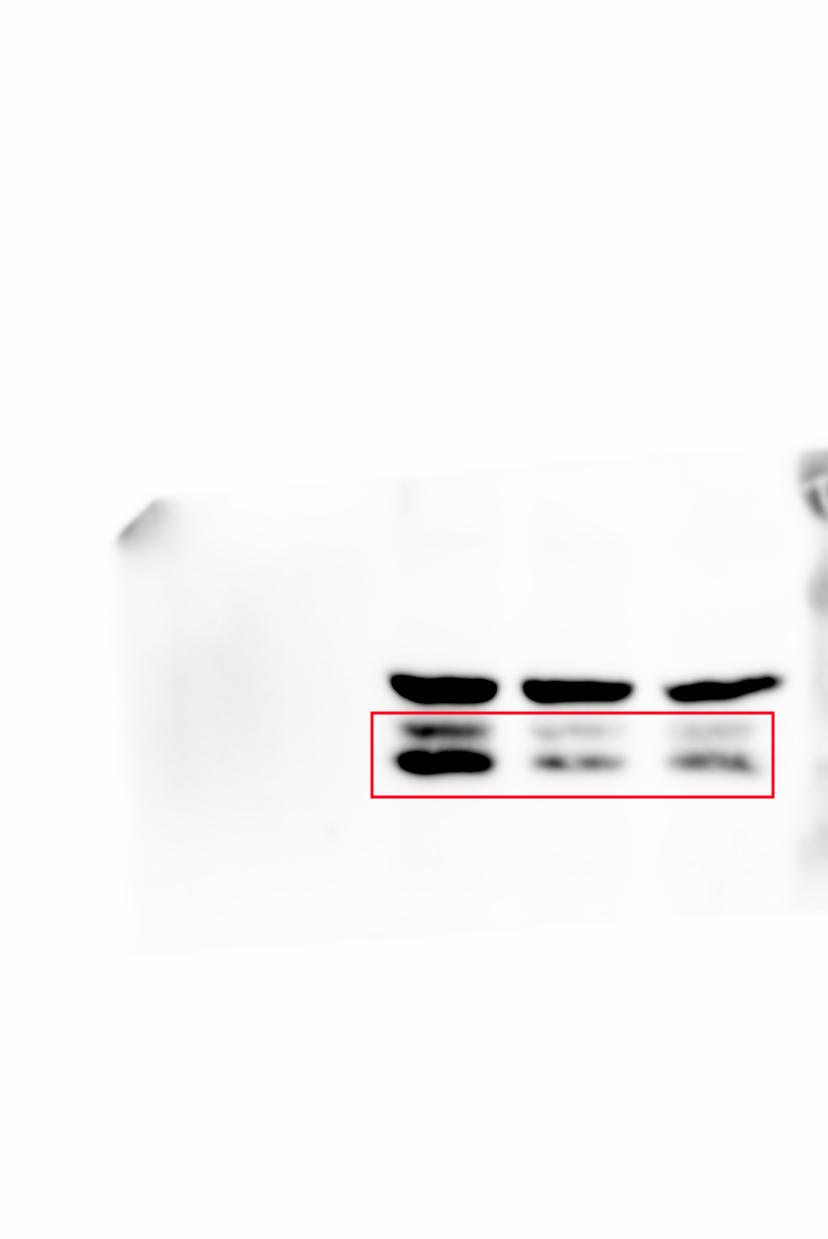

Supplement: Supplementary file 5 — Source data Fig. 4 [file 44318_2025_363_MOESM5_ESM.zip › Figure 4/4H/1 p-erk.tif]

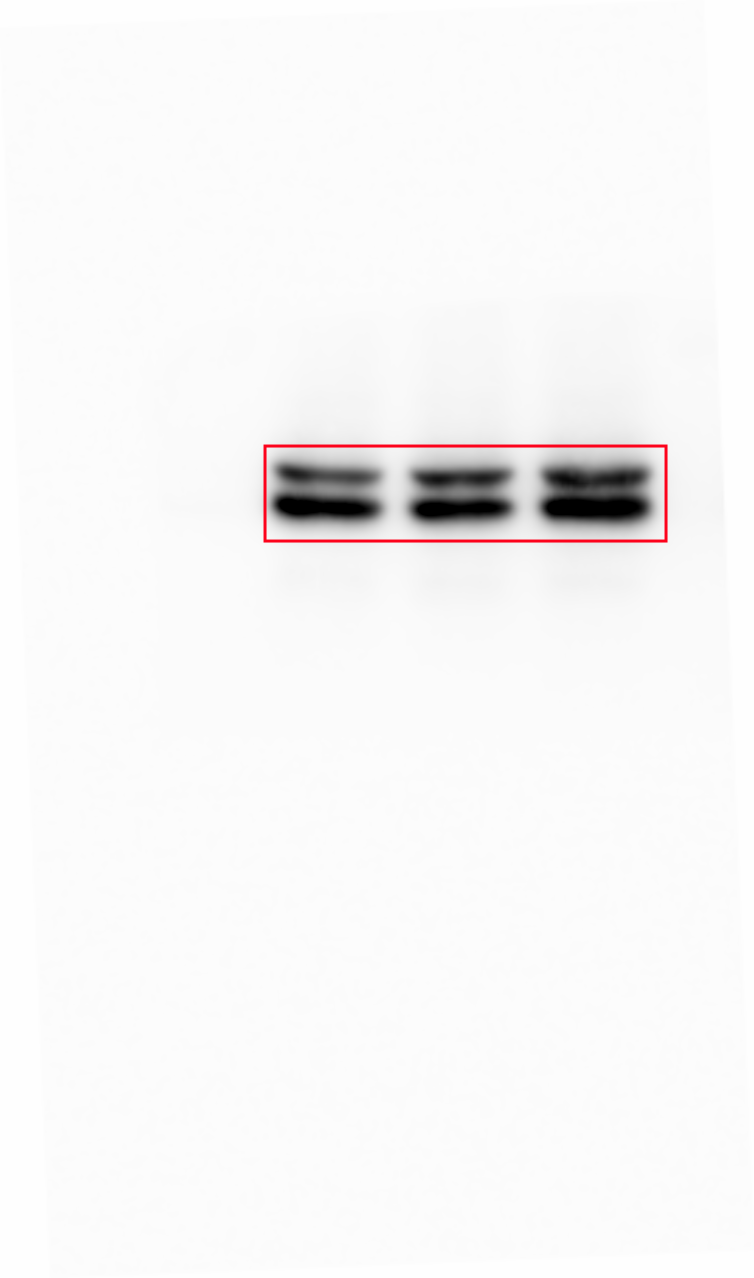

Supplement: Supplementary file 5 — Source data Fig. 4 [file 44318_2025_363_MOESM5_ESM.zip › Figure 4/4H/2 erk.tif]

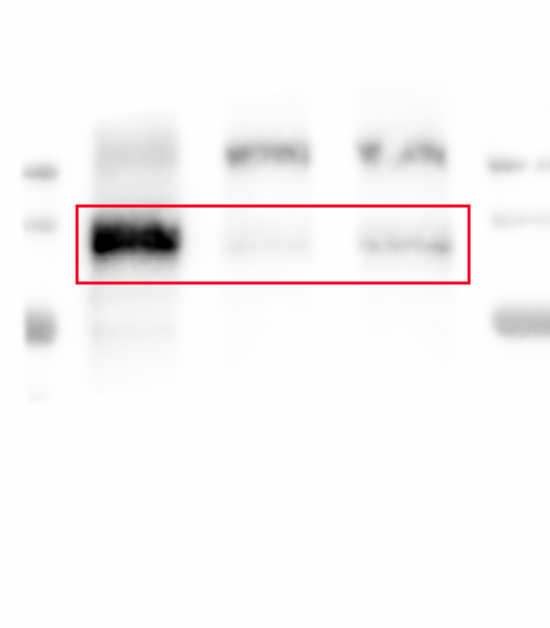

Supplement: Supplementary file 5 — Source data Fig. 4 [file 44318_2025_363_MOESM5_ESM.zip › Figure 4/4H/3 p-fak.tif]

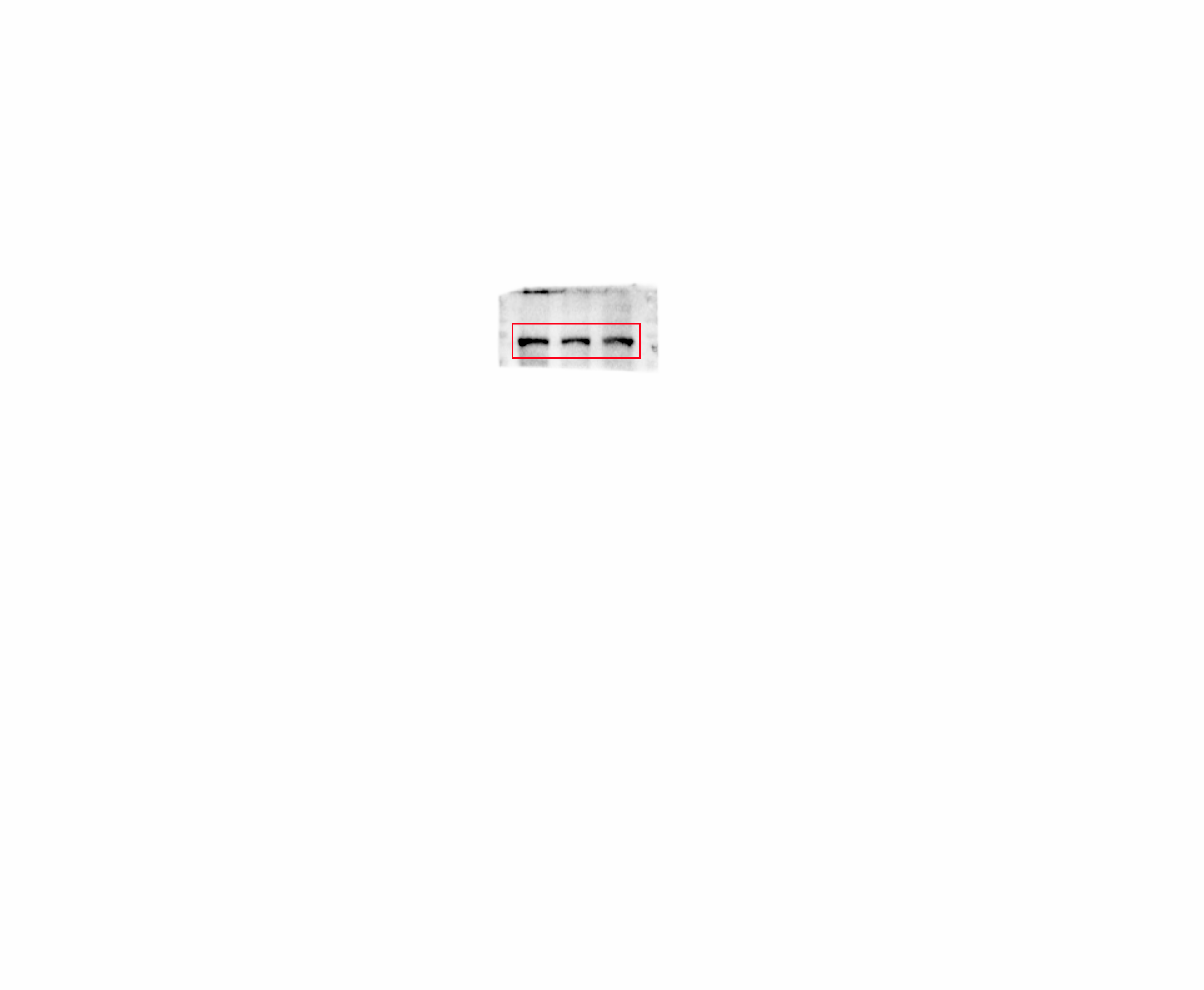

Supplement: Supplementary file 5 — Source data Fig. 4 [file 44318_2025_363_MOESM5_ESM.zip › Figure 4/4H/4 fak.tif]

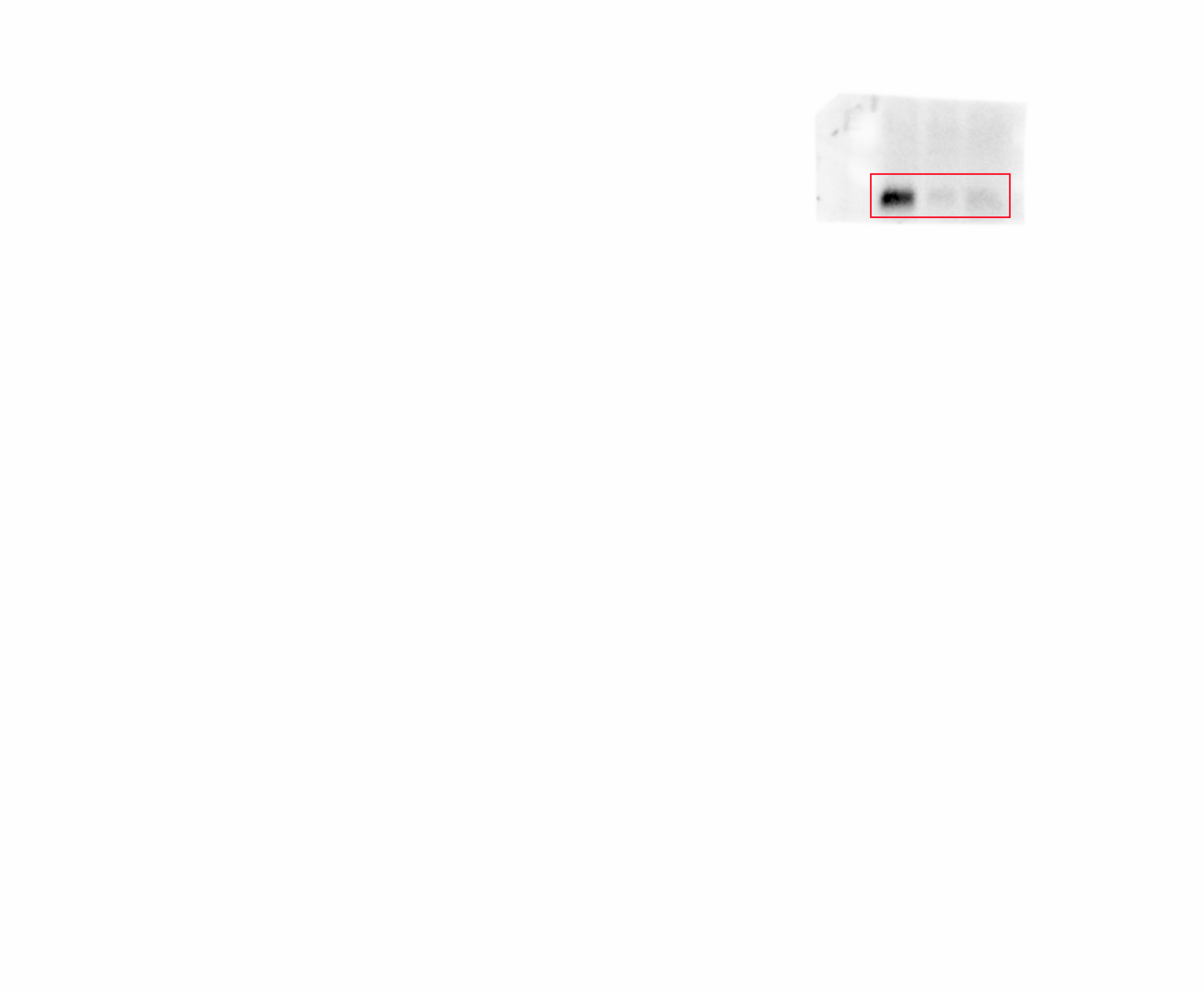

Supplement: Supplementary file 5 — Source data Fig. 4 [file 44318_2025_363_MOESM5_ESM.zip › Figure 4/4H/7 Ephrin A1.tif]

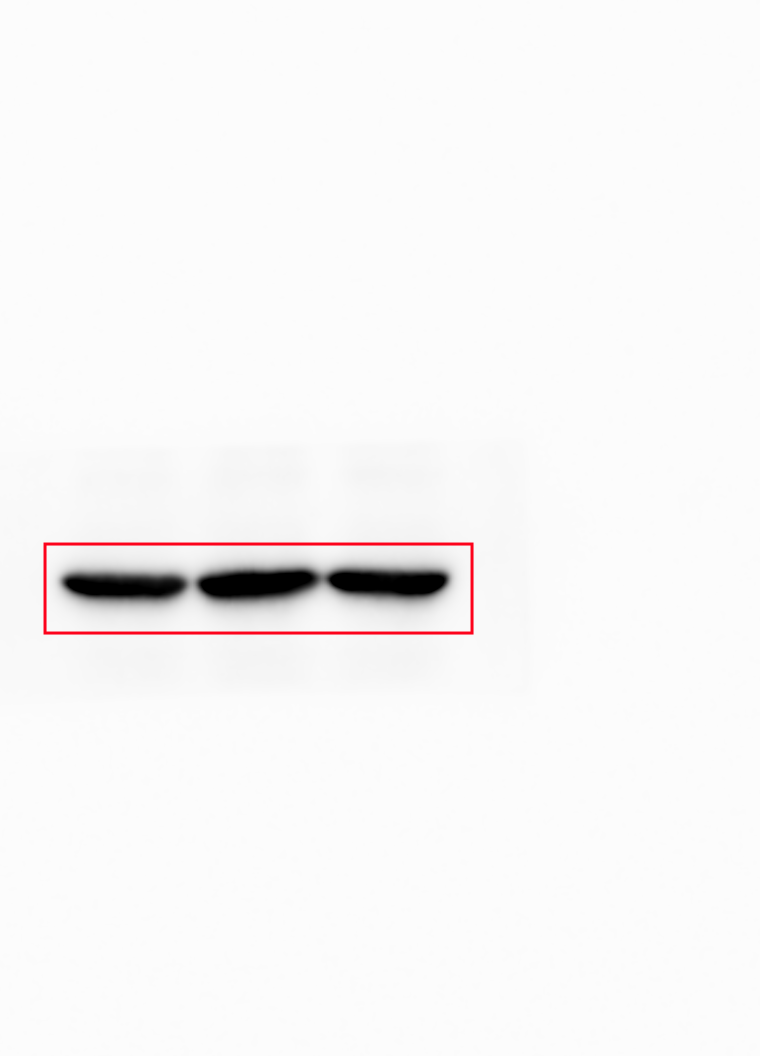

Supplement: Supplementary file 5 — Source data Fig. 4 [file 44318_2025_363_MOESM5_ESM.zip › Figure 4/4H/8 actin.tif]

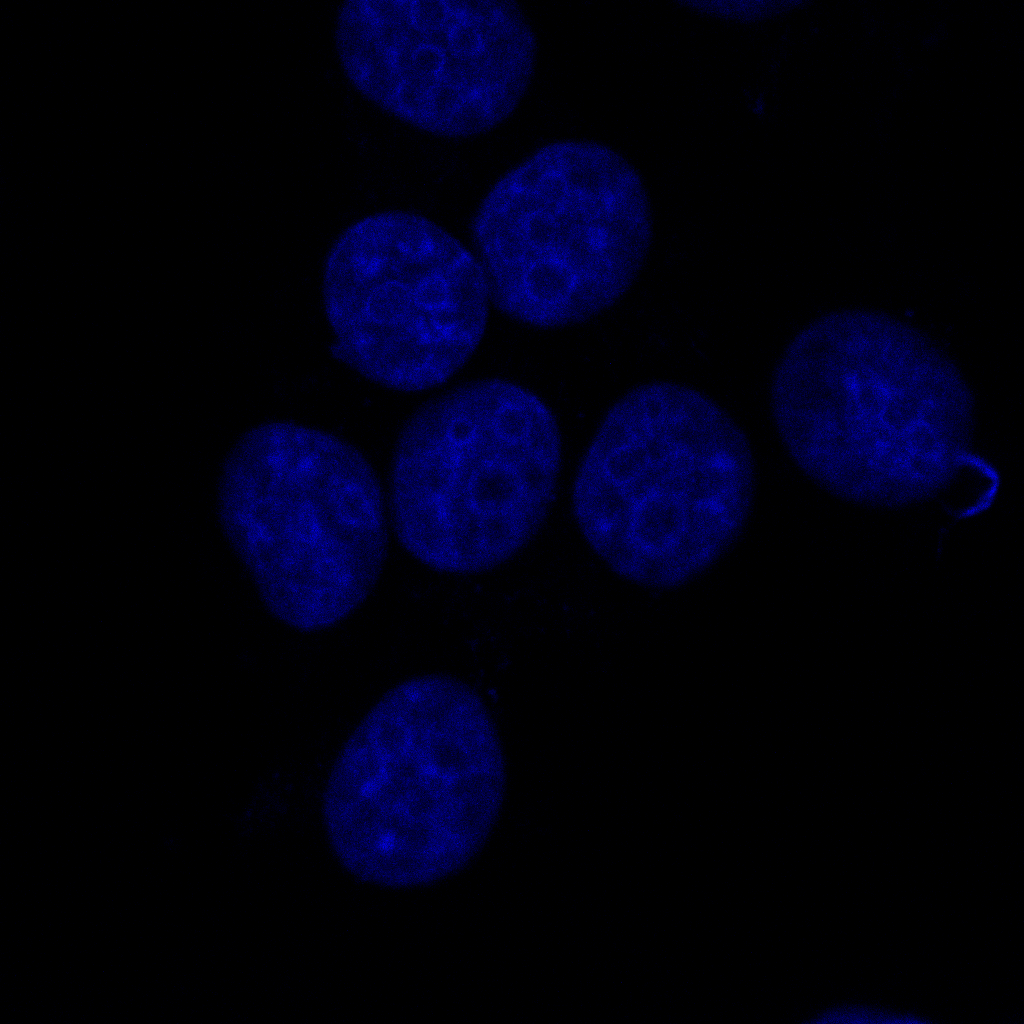

Supplement: Supplementary file 5 — Source data Fig. 4 [file 44318_2025_363_MOESM5_ESM.zip › Figure 4/4I/EGF/DAPI.tif]

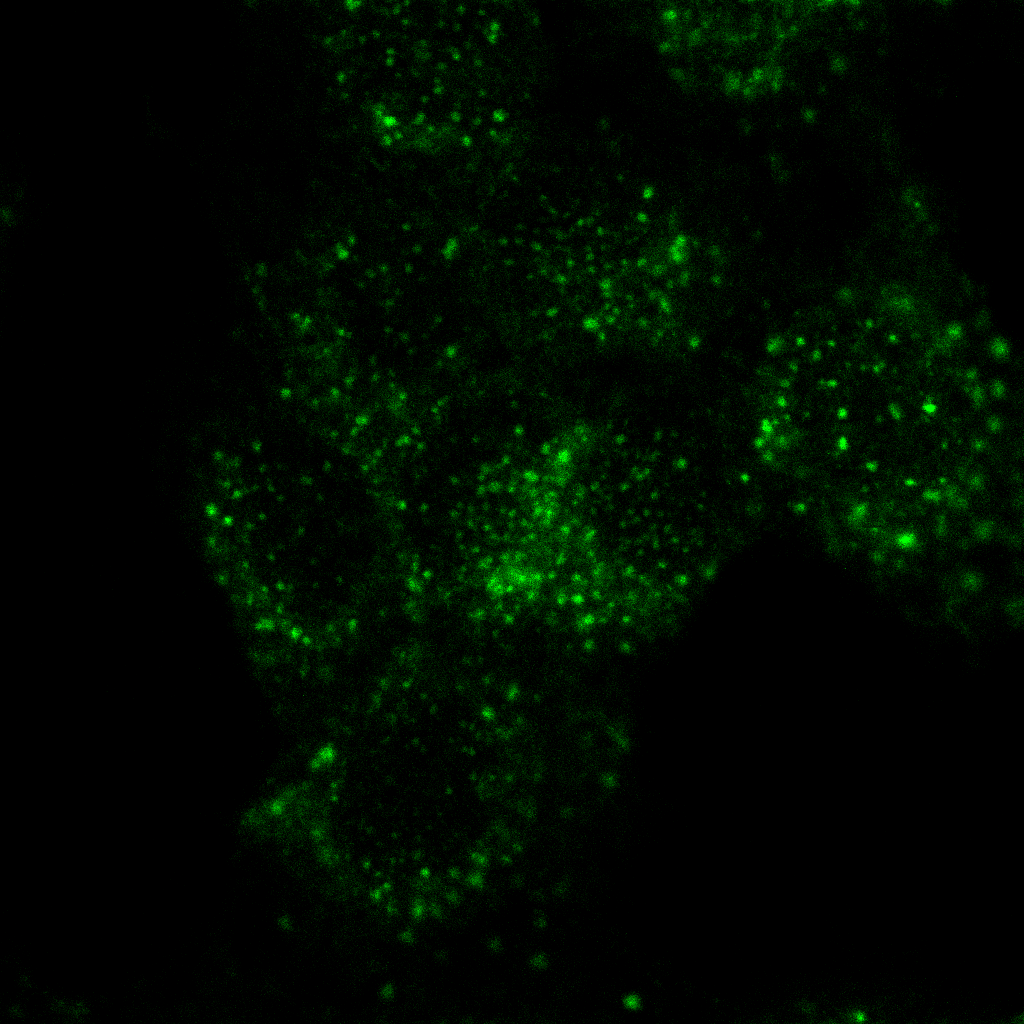

Supplement: Supplementary file 5 — Source data Fig. 4 [file 44318_2025_363_MOESM5_ESM.zip › Figure 4/4I/EGF/EGFR.tif]

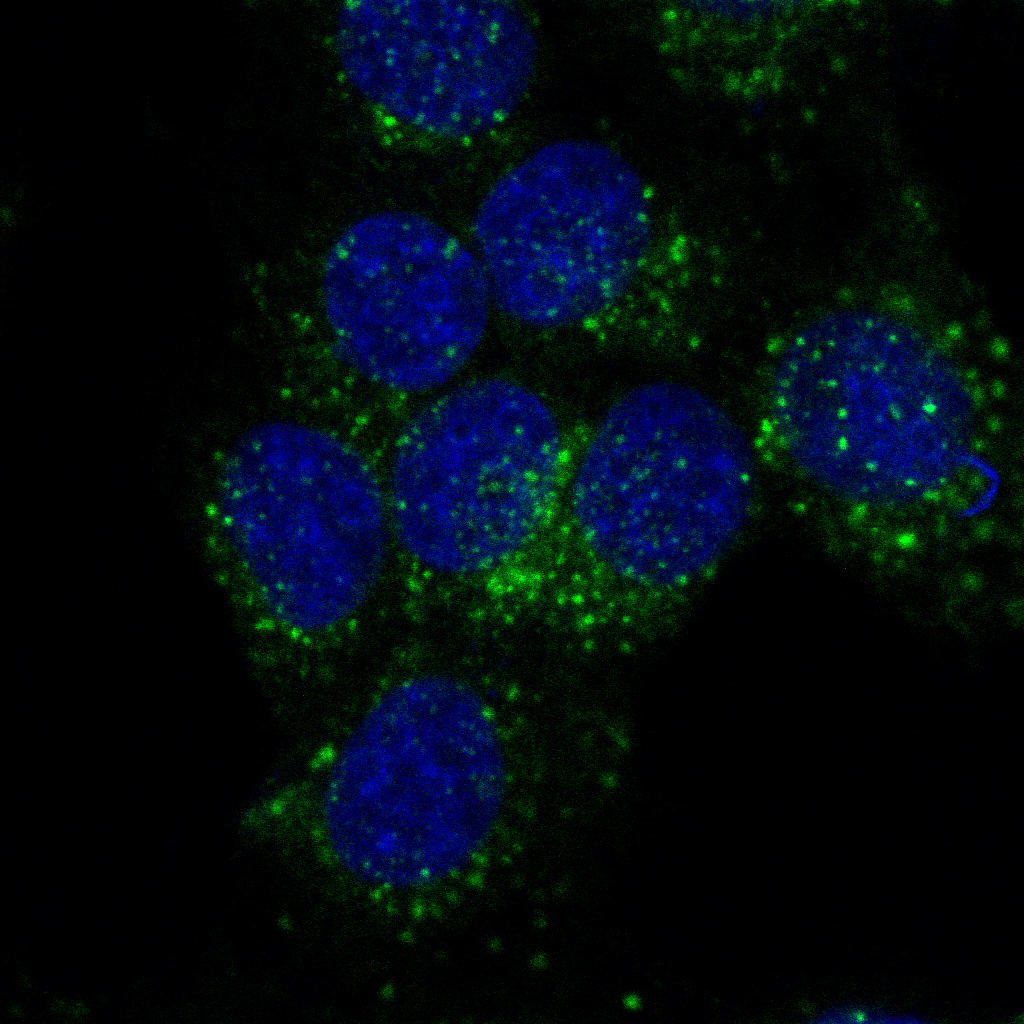

Supplement: Supplementary file 5 — Source data Fig. 4 [file 44318_2025_363_MOESM5_ESM.zip › Figure 4/4I/EGF/MERGE.tif]

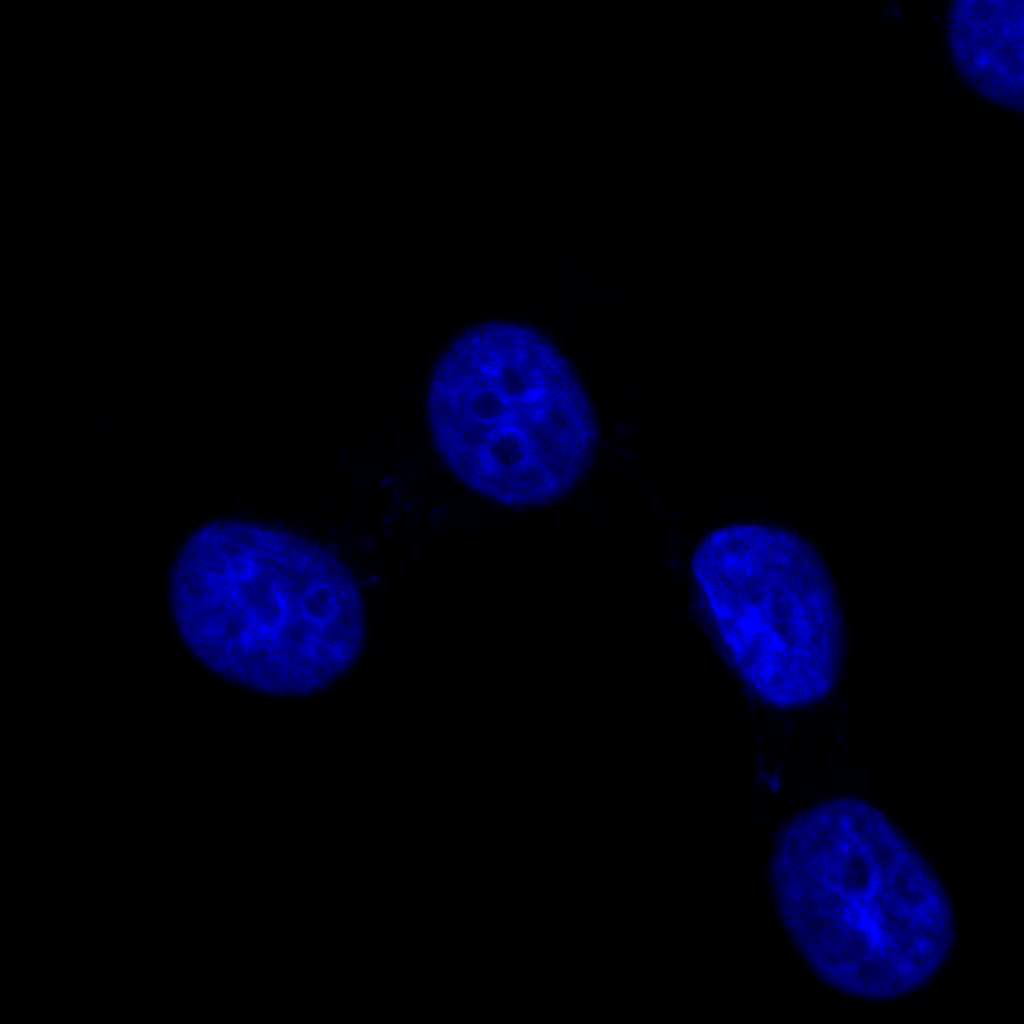

Supplement: Supplementary file 5 — Source data Fig. 4 [file 44318_2025_363_MOESM5_ESM.zip › Figure 4/4I/Ephrin A1-Fc/DAPI.tif]

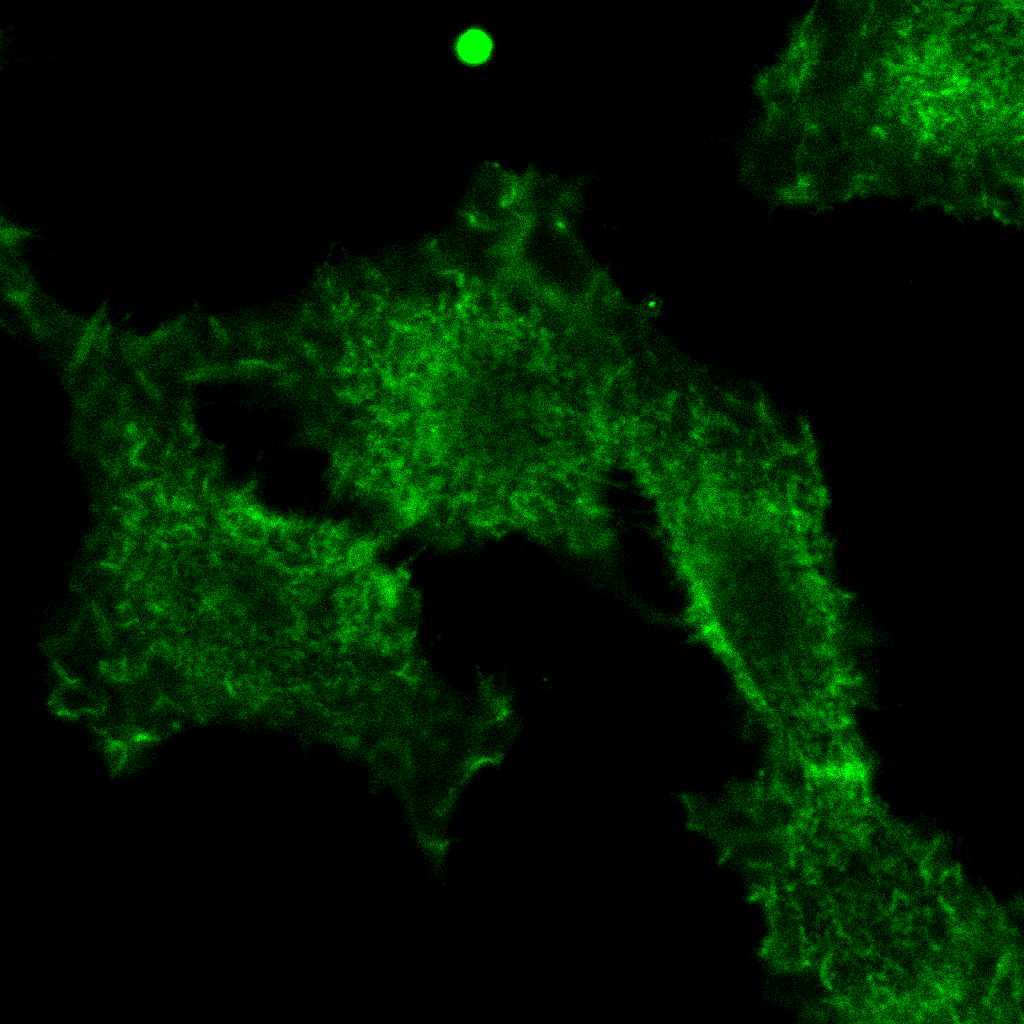

Supplement: Supplementary file 5 — Source data Fig. 4 [file 44318_2025_363_MOESM5_ESM.zip › Figure 4/4I/Ephrin A1-Fc/EGFR.tif]

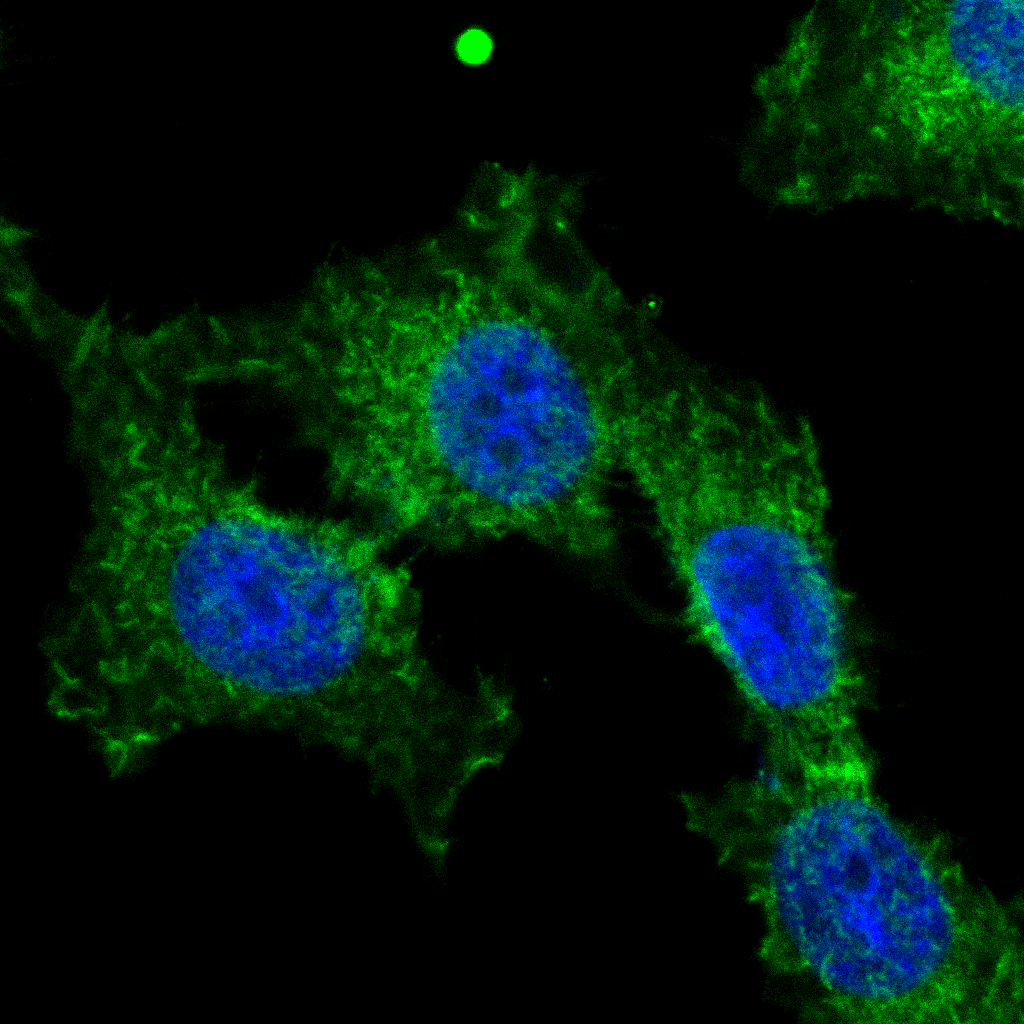

Supplement: Supplementary file 5 — Source data Fig. 4 [file 44318_2025_363_MOESM5_ESM.zip › Figure 4/4I/Ephrin A1-Fc/MERGE.tif]

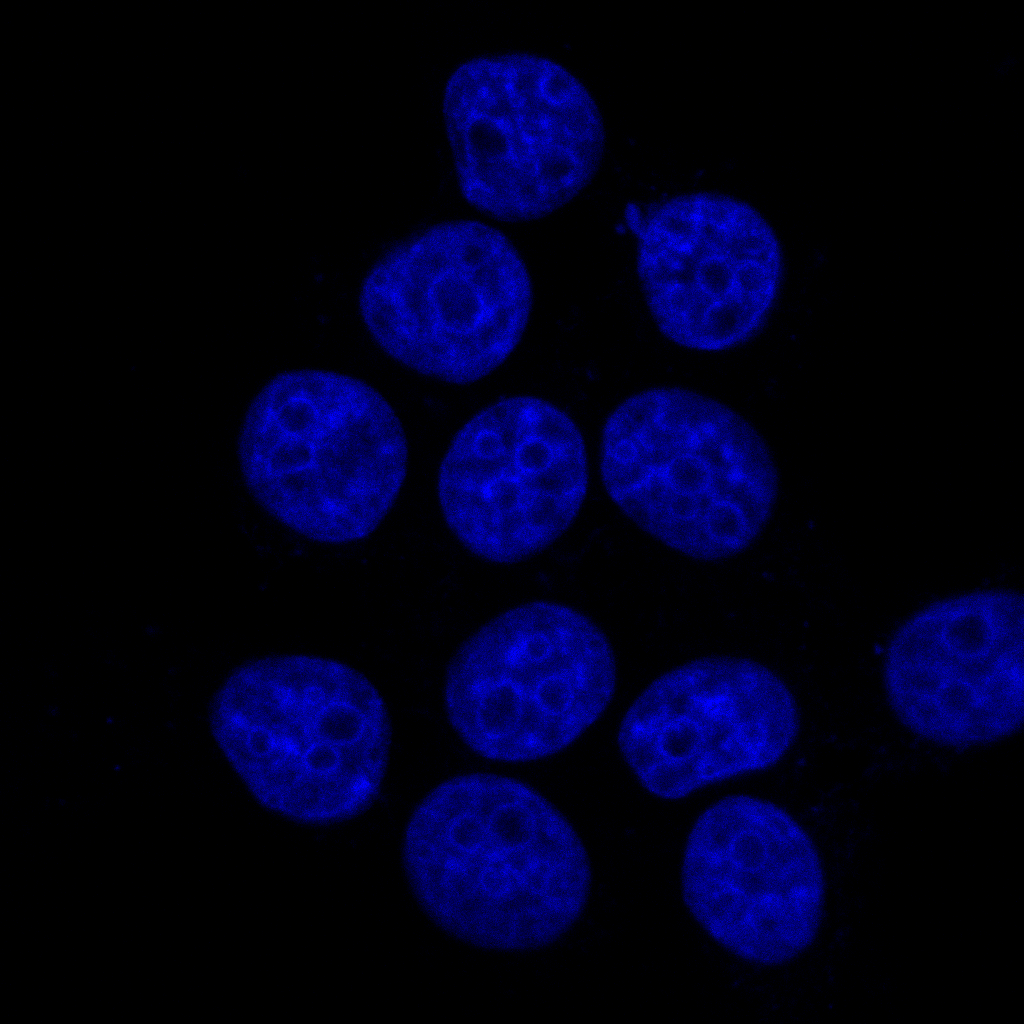

Supplement: Supplementary file 5 — Source data Fig. 4 [file 44318_2025_363_MOESM5_ESM.zip › Figure 4/4I/PBS/DAPI.tif]

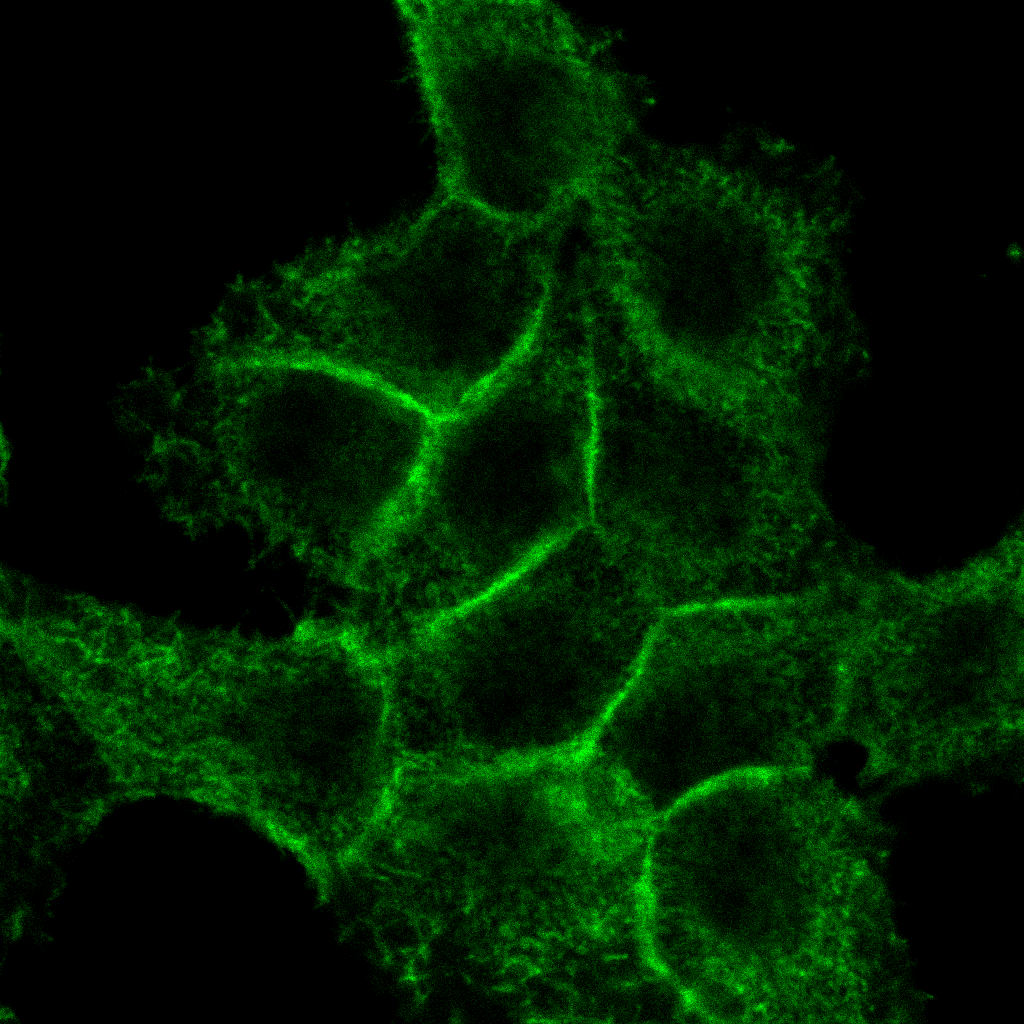

Supplement: Supplementary file 5 — Source data Fig. 4 [file 44318_2025_363_MOESM5_ESM.zip › Figure 4/4I/PBS/EGFR.tif]

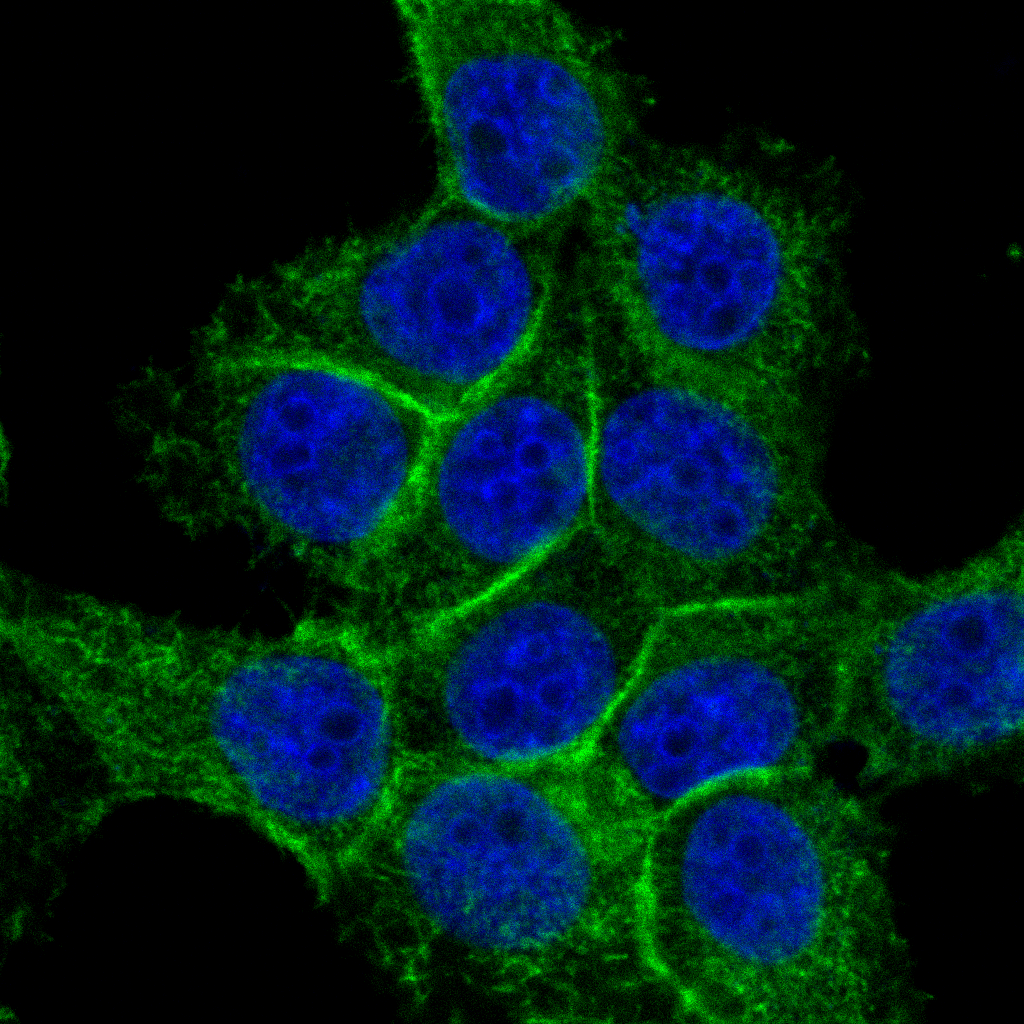

Supplement: Supplementary file 5 — Source data Fig. 4 [file 44318_2025_363_MOESM5_ESM.zip › Figure 4/4I/PBS/MERGE.tif]

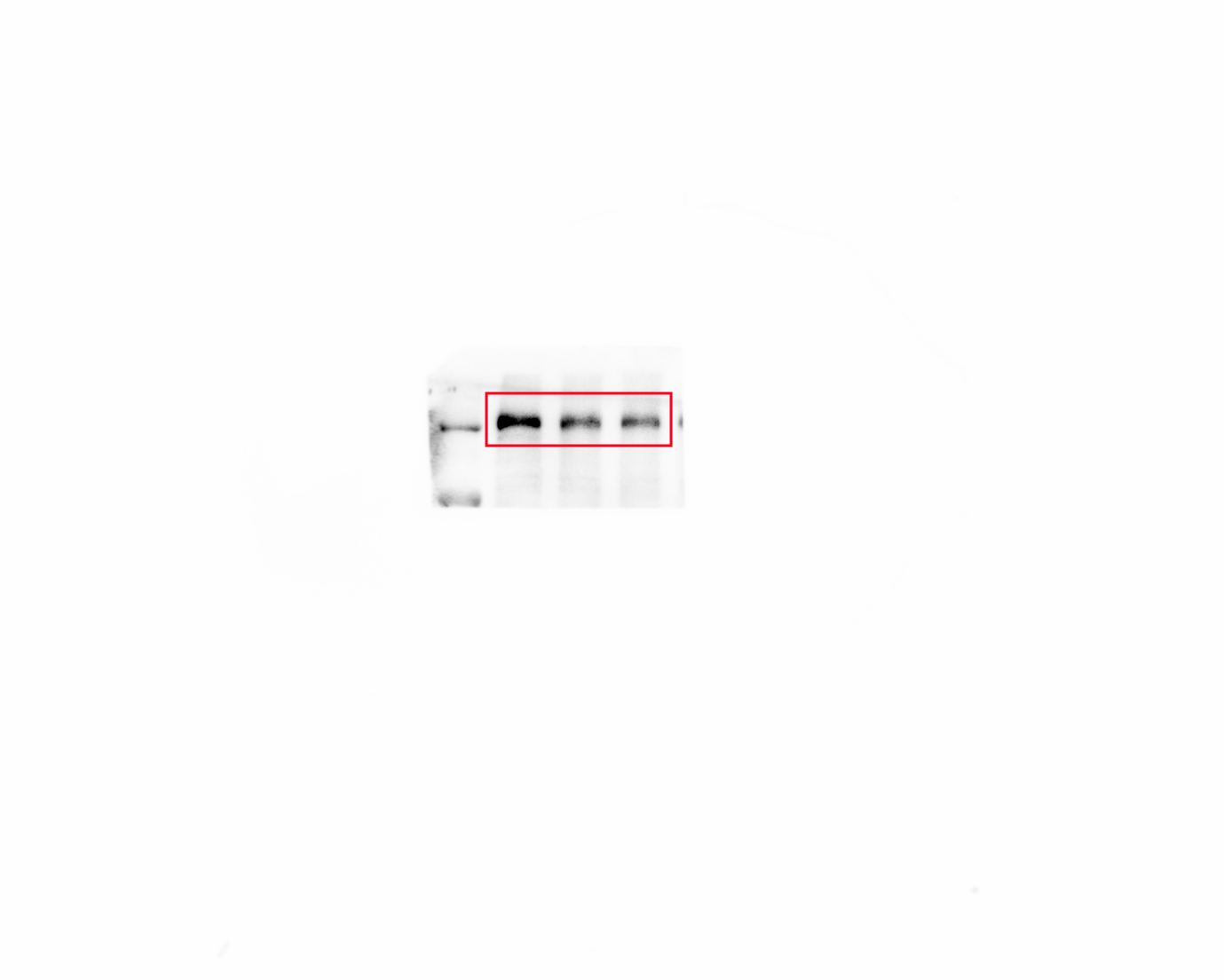

Supplement: Supplementary file 5 — Source data Fig. 4 [file 44318_2025_363_MOESM5_ESM.zip › Figure 4/4J/1 P-EGFR.tif]

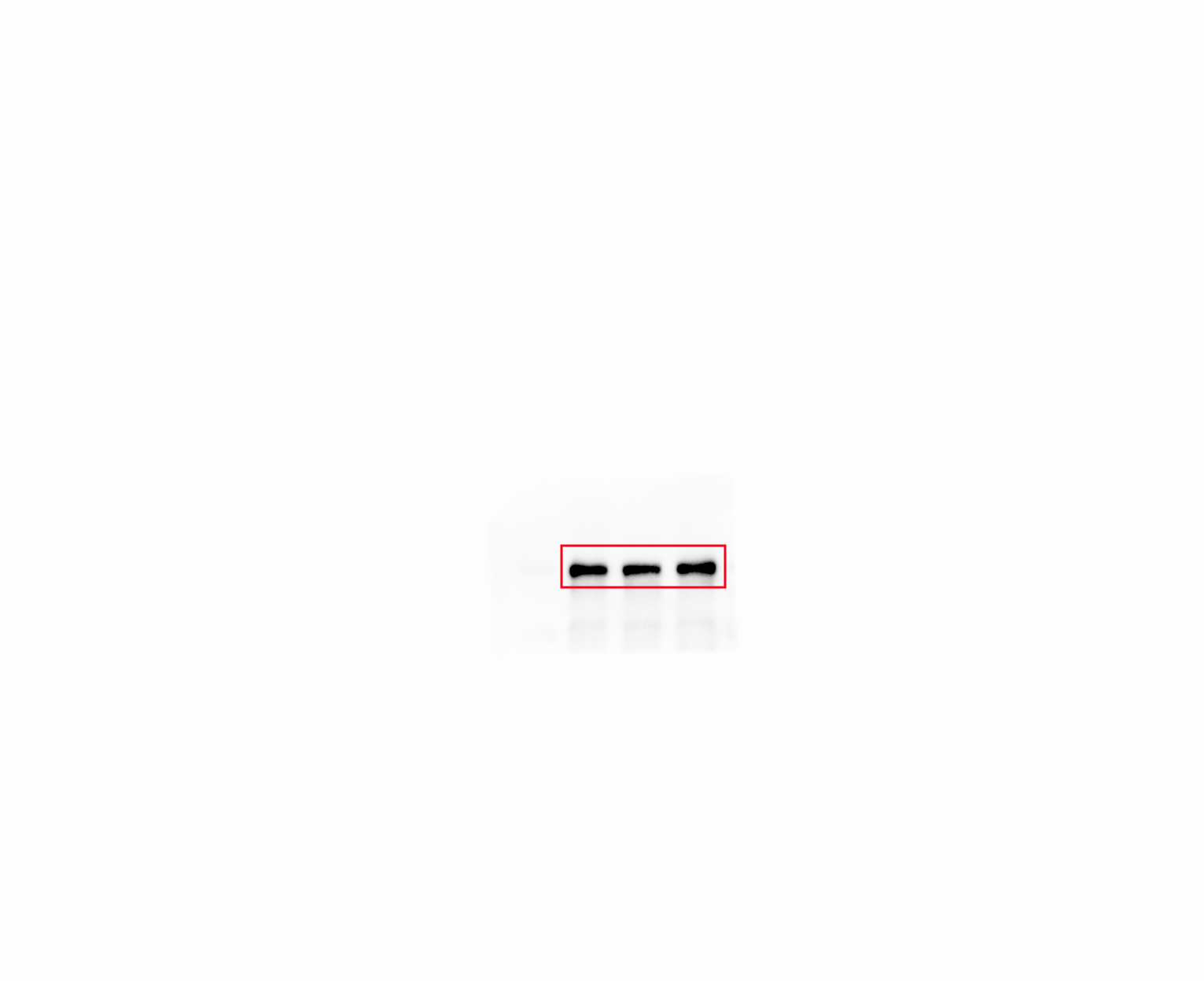

Supplement: Supplementary file 5 — Source data Fig. 4 [file 44318_2025_363_MOESM5_ESM.zip › Figure 4/4J/2 EGFR.tif]

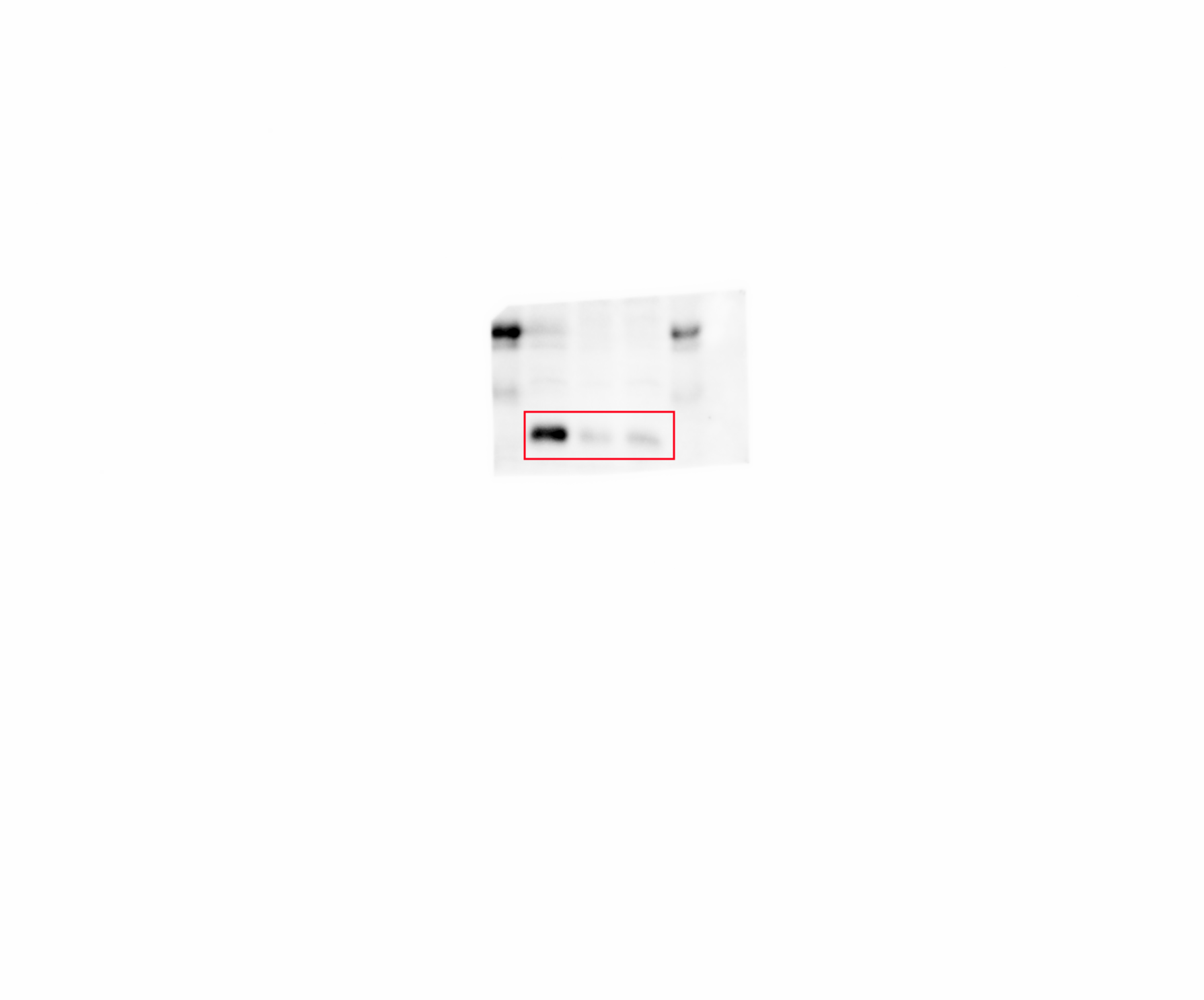

Supplement: Supplementary file 5 — Source data Fig. 4 [file 44318_2025_363_MOESM5_ESM.zip › Figure 4/4J/3 Ephrin A1.tif]

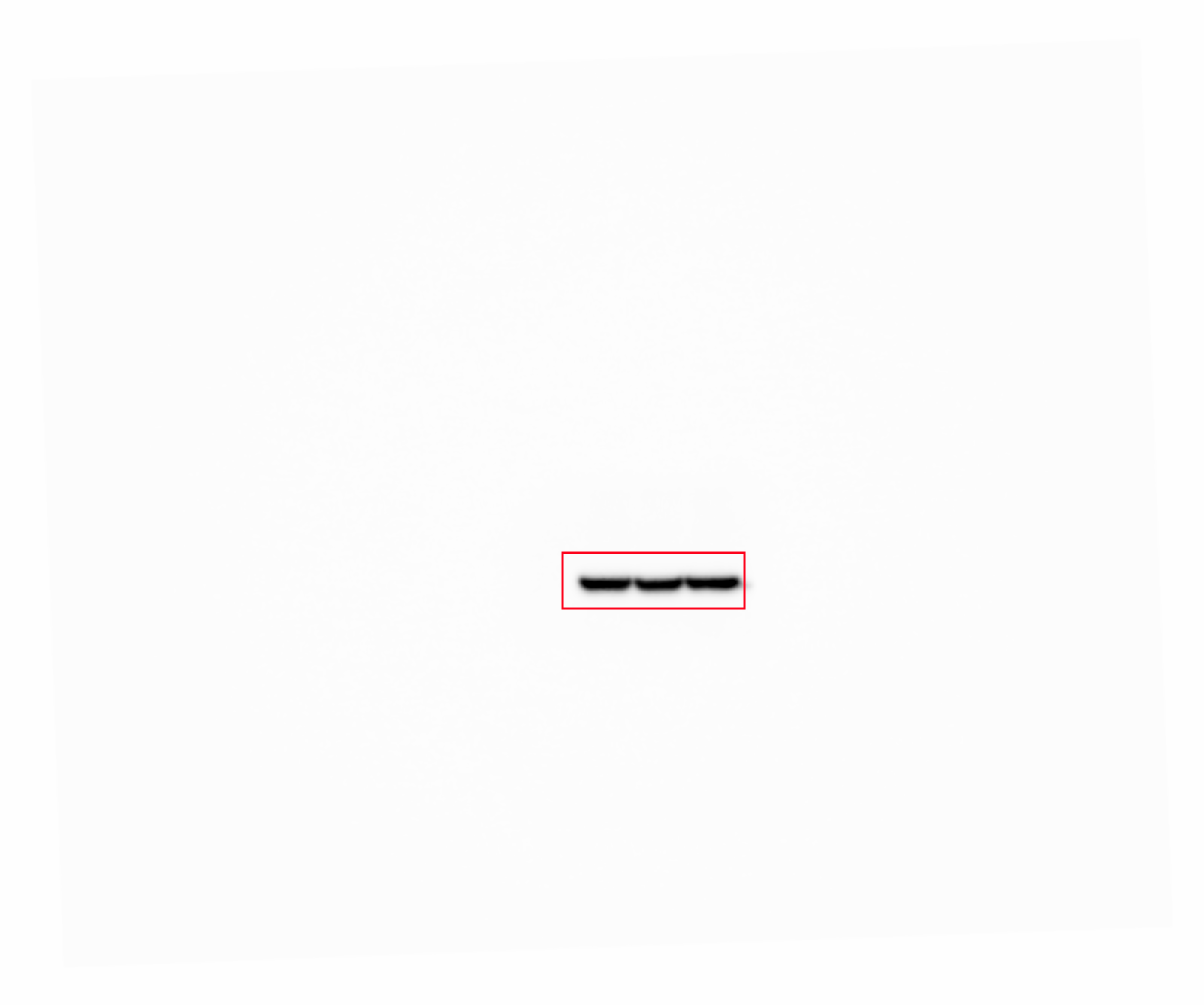

Supplement: Supplementary file 5 — Source data Fig. 4 [file 44318_2025_363_MOESM5_ESM.zip › Figure 4/4J/4 actin.tif]

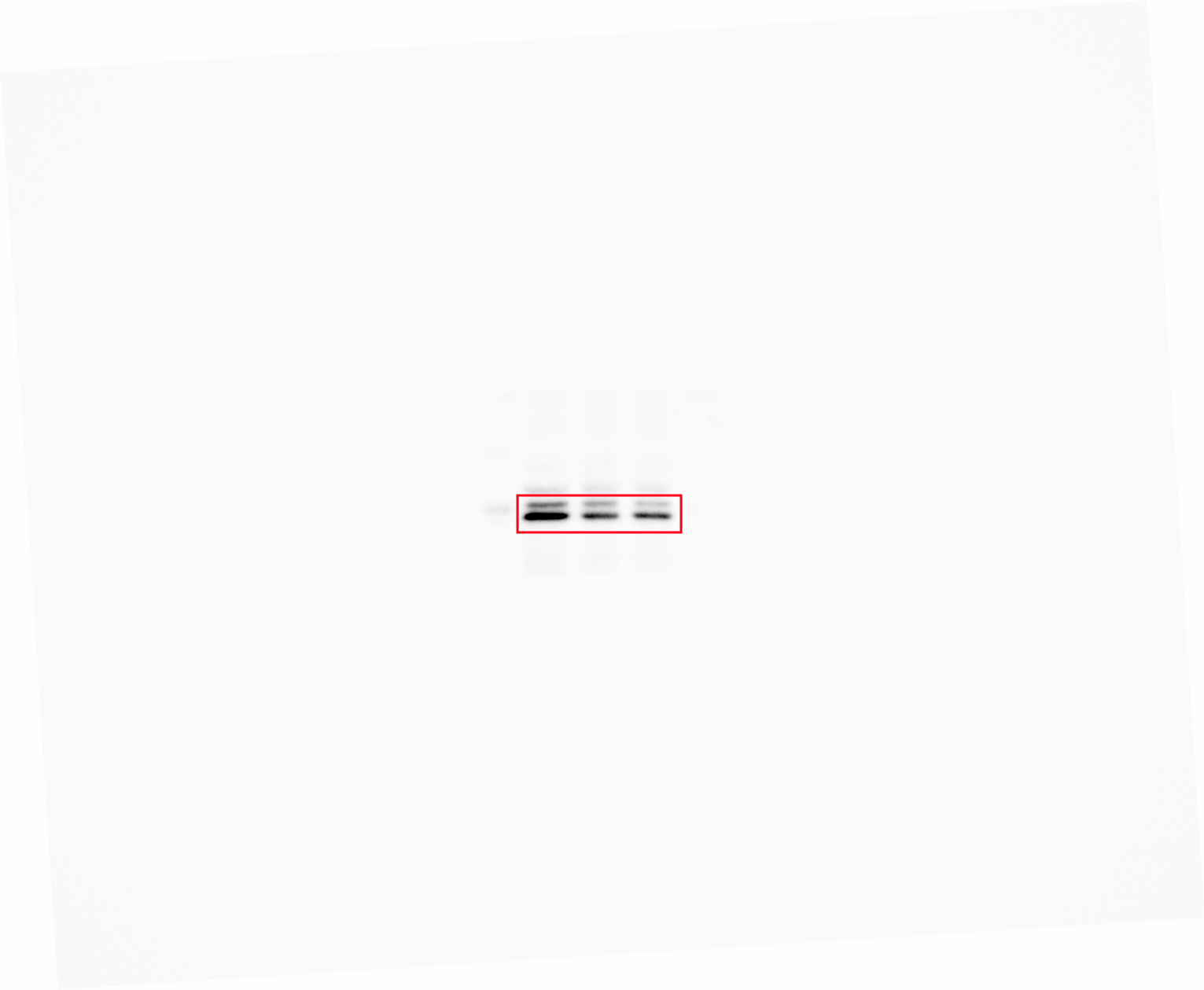

Supplement: Supplementary file 5 — Source data Fig. 4 [file 44318_2025_363_MOESM5_ESM.zip › Figure 4/4K/1 P-ERK.tif]

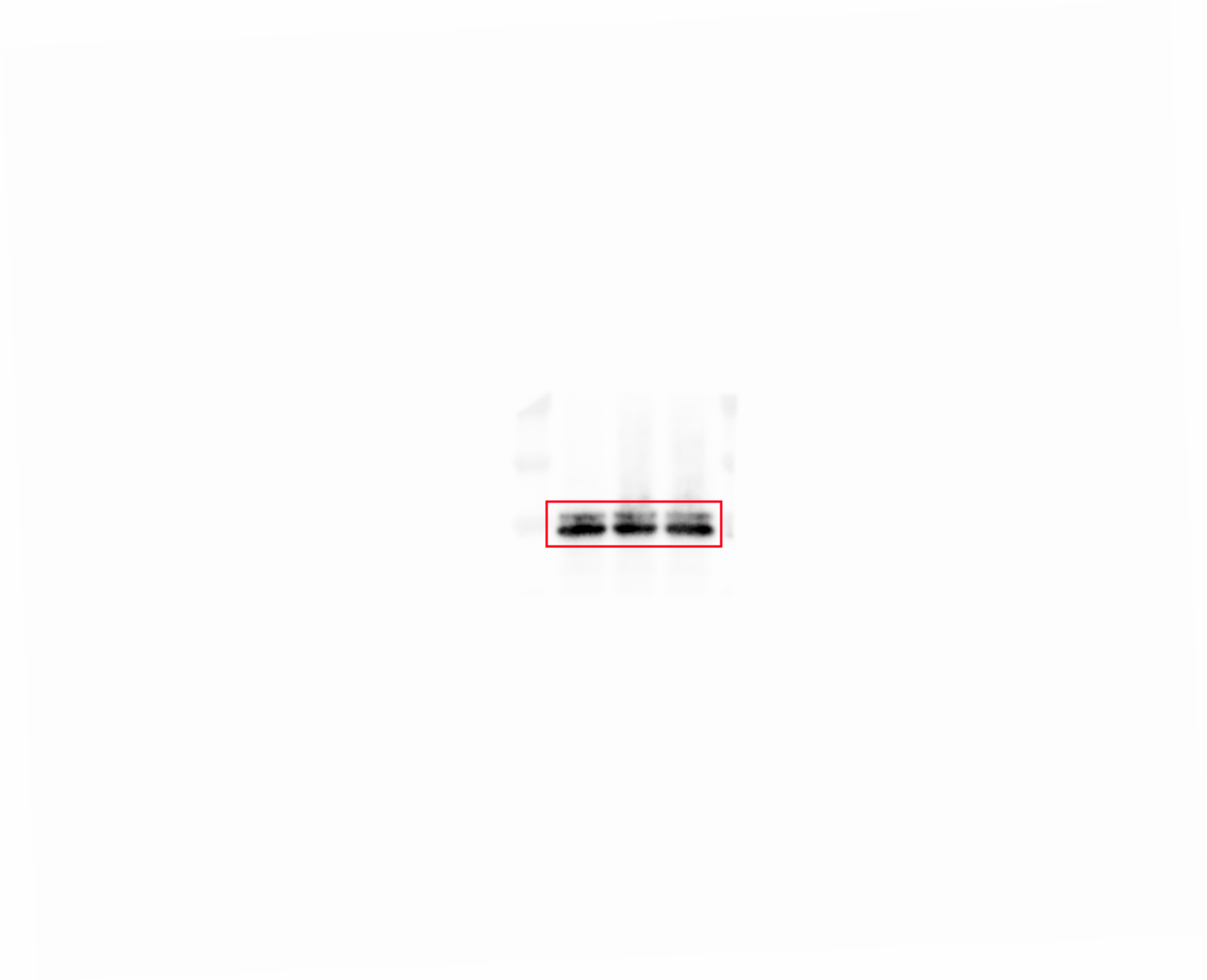

Supplement: Supplementary file 5 — Source data Fig. 4 [file 44318_2025_363_MOESM5_ESM.zip › Figure 4/4K/2 ERK.tif]

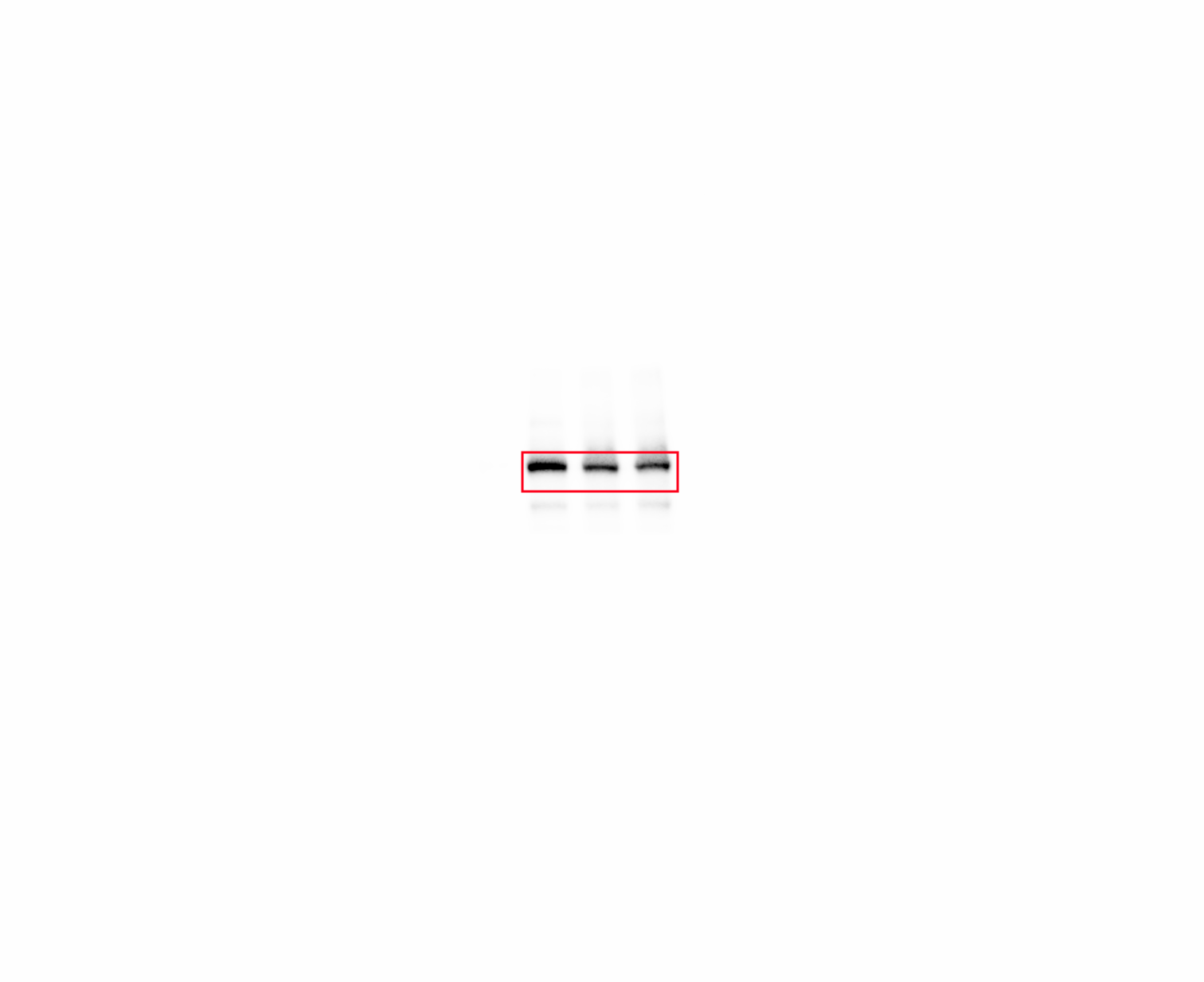

Supplement: Supplementary file 5 — Source data Fig. 4 [file 44318_2025_363_MOESM5_ESM.zip › Figure 4/4K/3 P-FAK.tif]

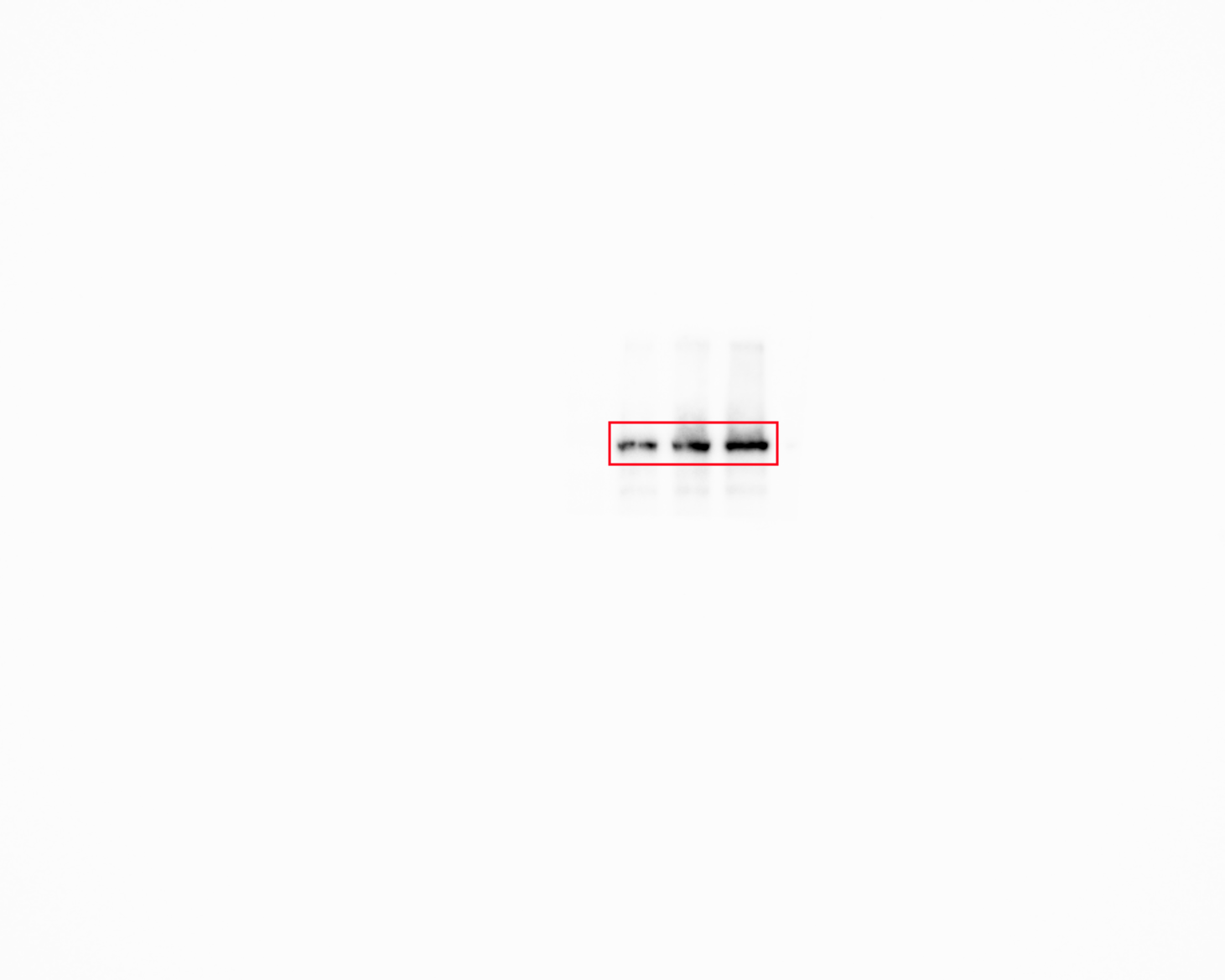

Supplement: Supplementary file 5 — Source data Fig. 4 [file 44318_2025_363_MOESM5_ESM.zip › Figure 4/4K/4 FAK.tif]

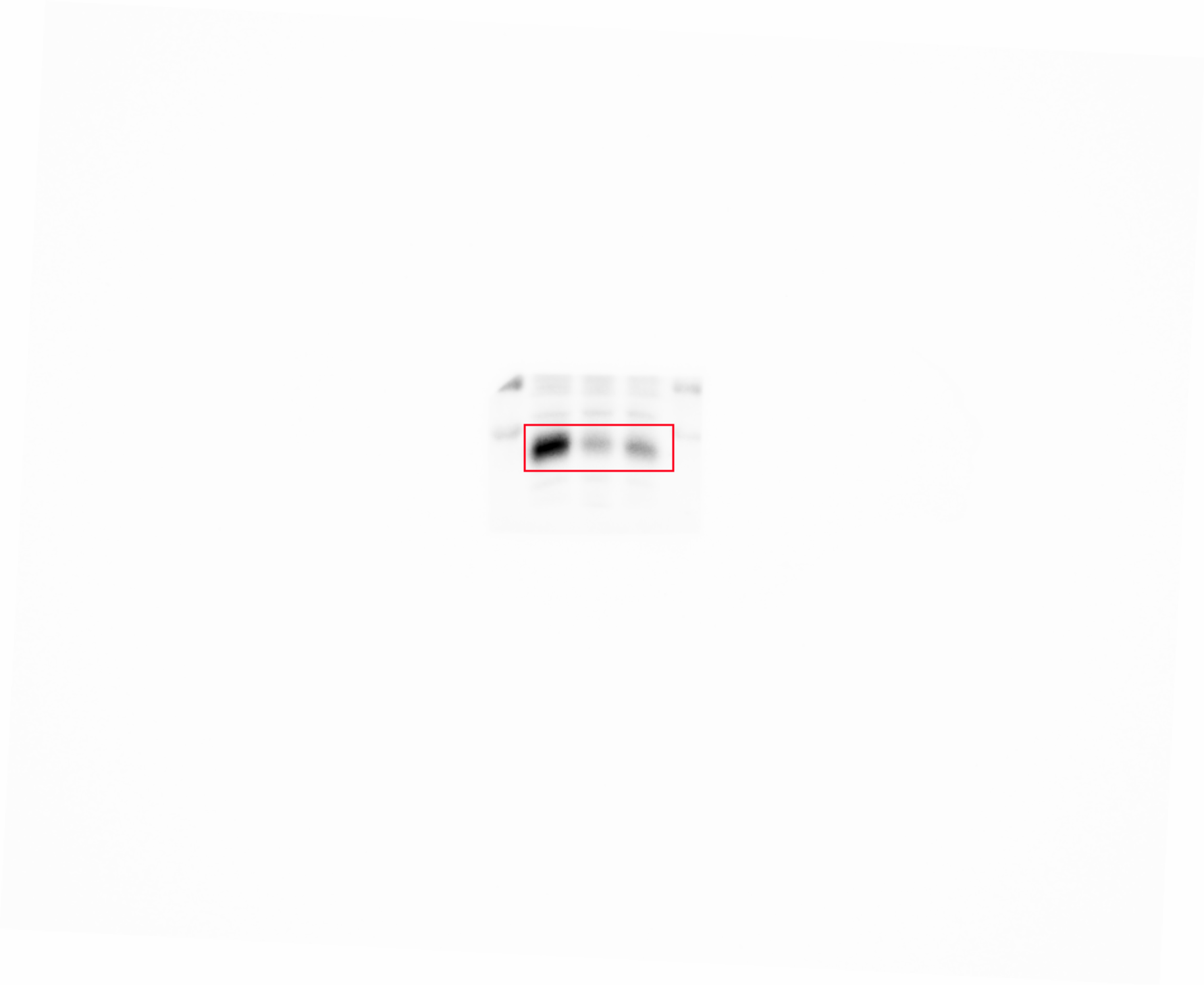

Supplement: Supplementary file 5 — Source data Fig. 4 [file 44318_2025_363_MOESM5_ESM.zip › Figure 4/4K/5 Ephrin A1.tif]

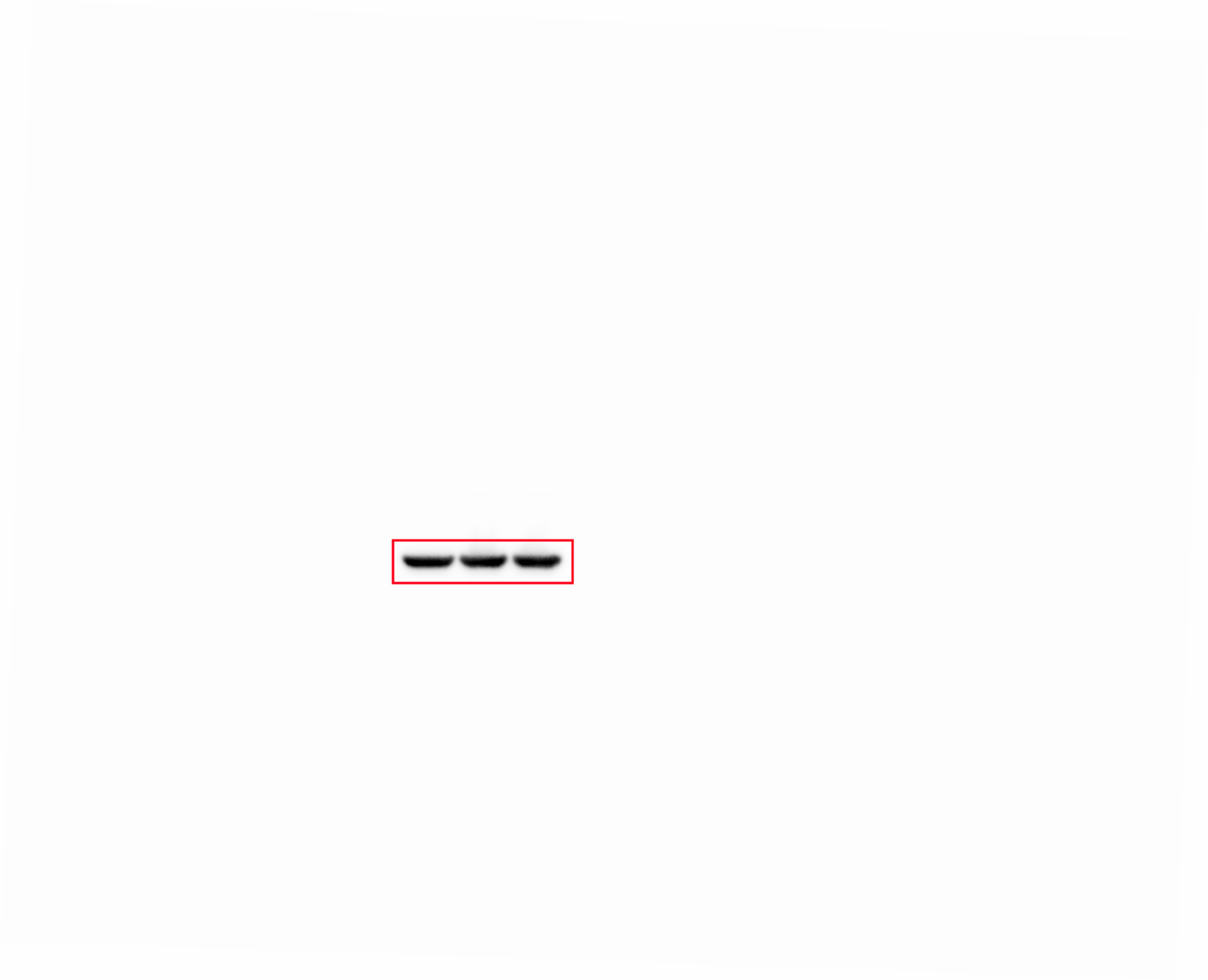

Supplement: Supplementary file 5 — Source data Fig. 4 [file 44318_2025_363_MOESM5_ESM.zip › Figure 4/4K/6 actin.tif]

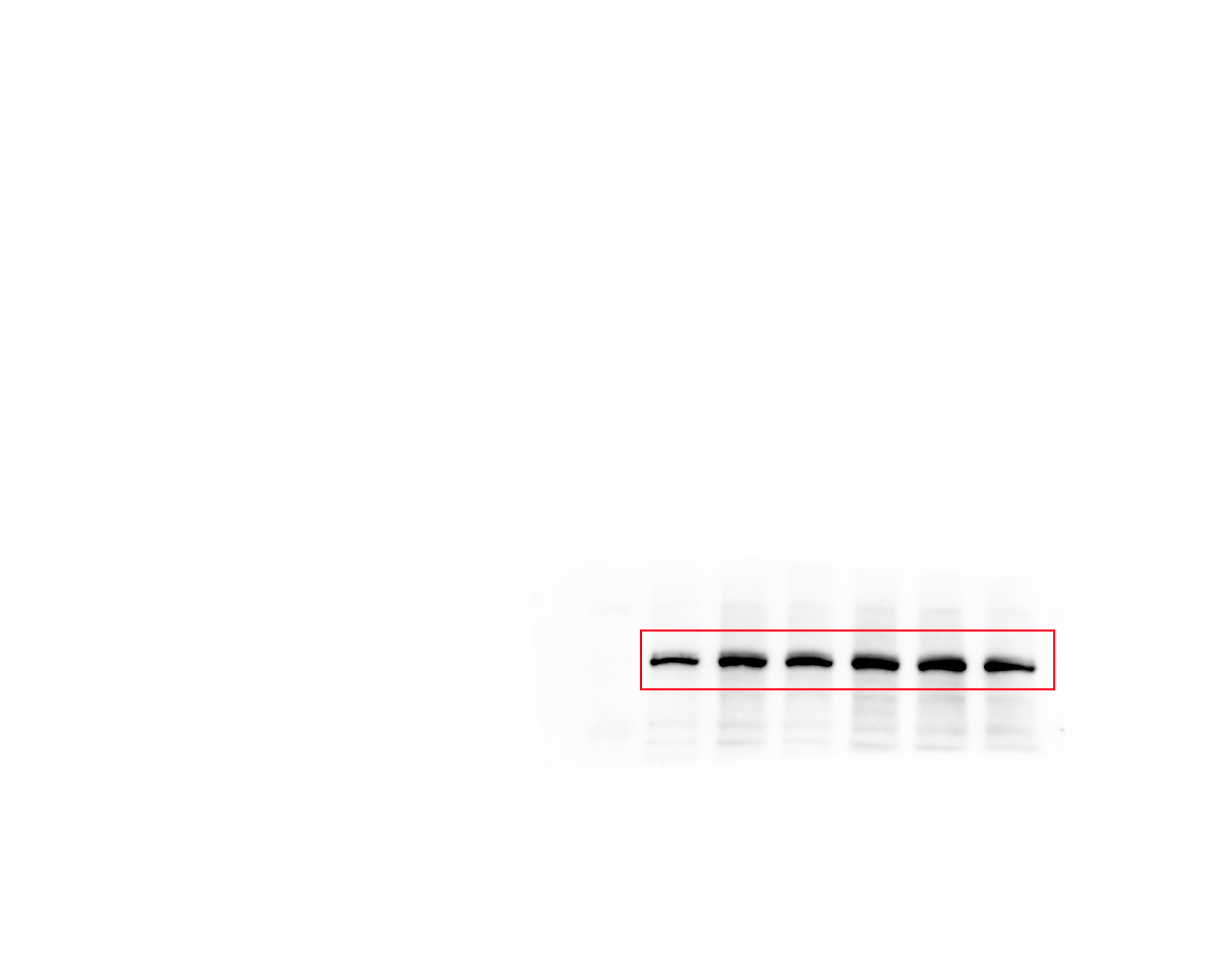

Supplement: Supplementary file 6 — Source data Fig. 5 [file 44318_2025_363_MOESM6_ESM.zip › Figure 5/5B/1 E-cad.tif]

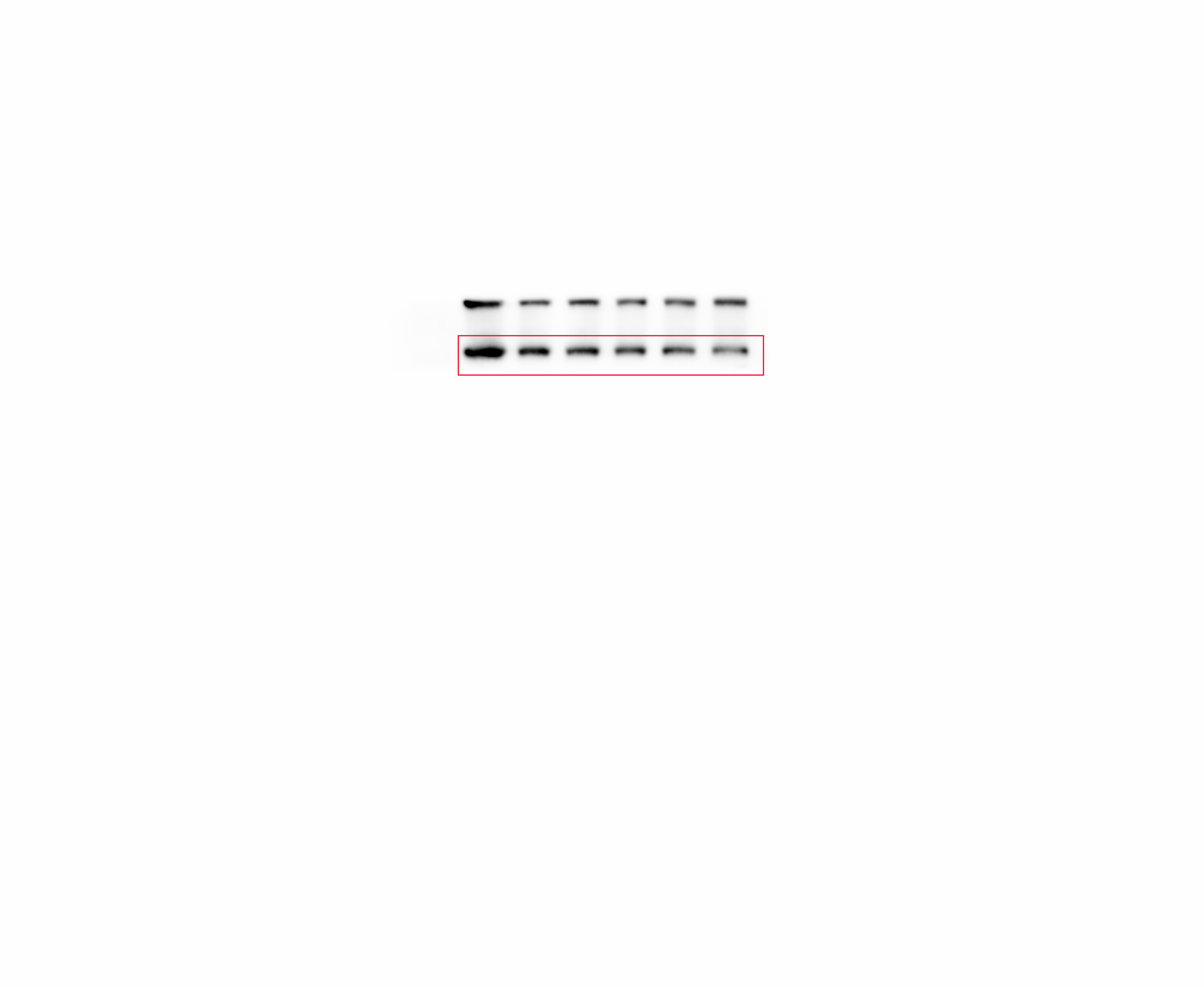

Supplement: Supplementary file 6 — Source data Fig. 5 [file 44318_2025_363_MOESM6_ESM.zip › Figure 5/5B/2 N-cad.tif]

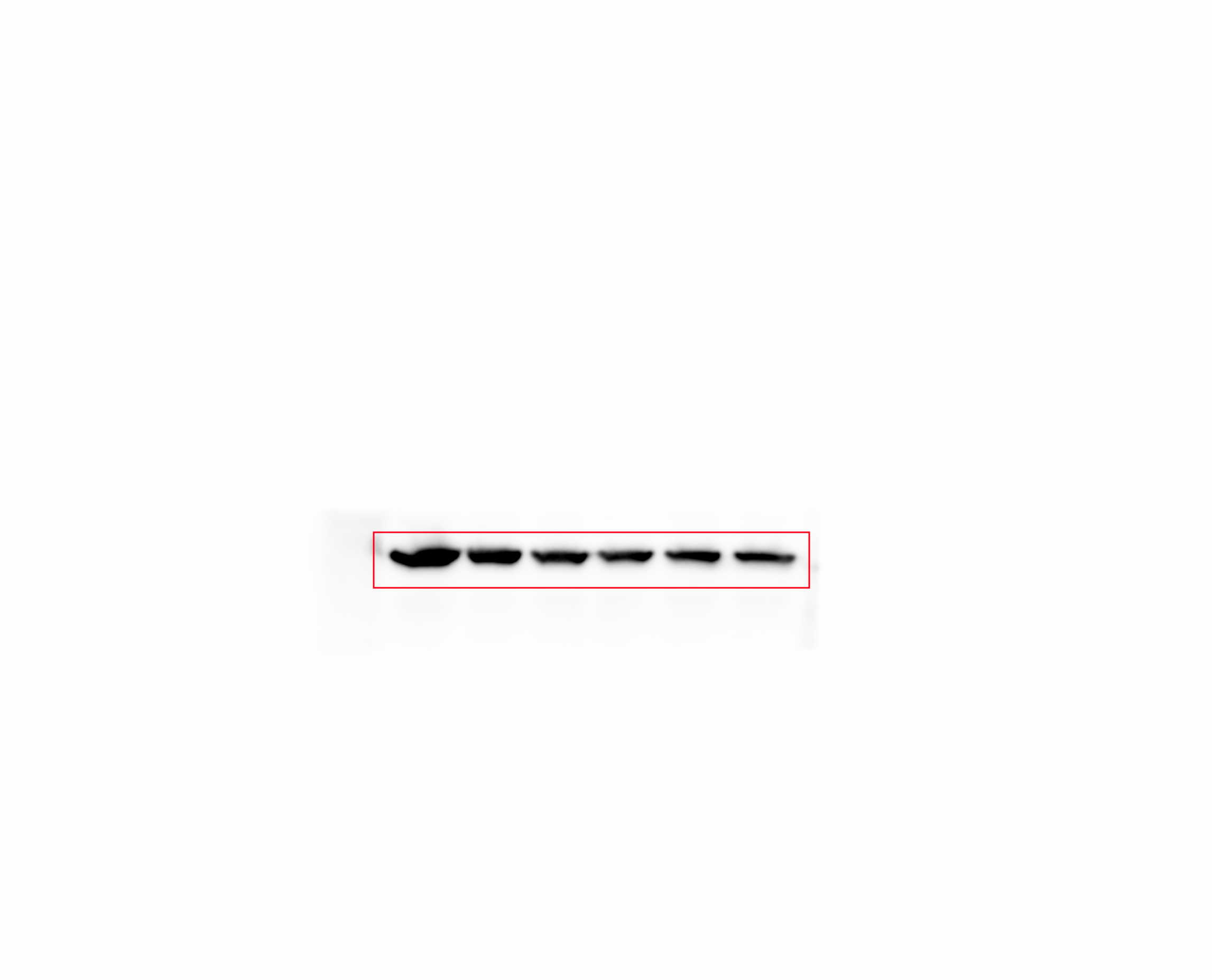

Supplement: Supplementary file 6 — Source data Fig. 5 [file 44318_2025_363_MOESM6_ESM.zip › Figure 5/5B/3 Vimentin.tif]

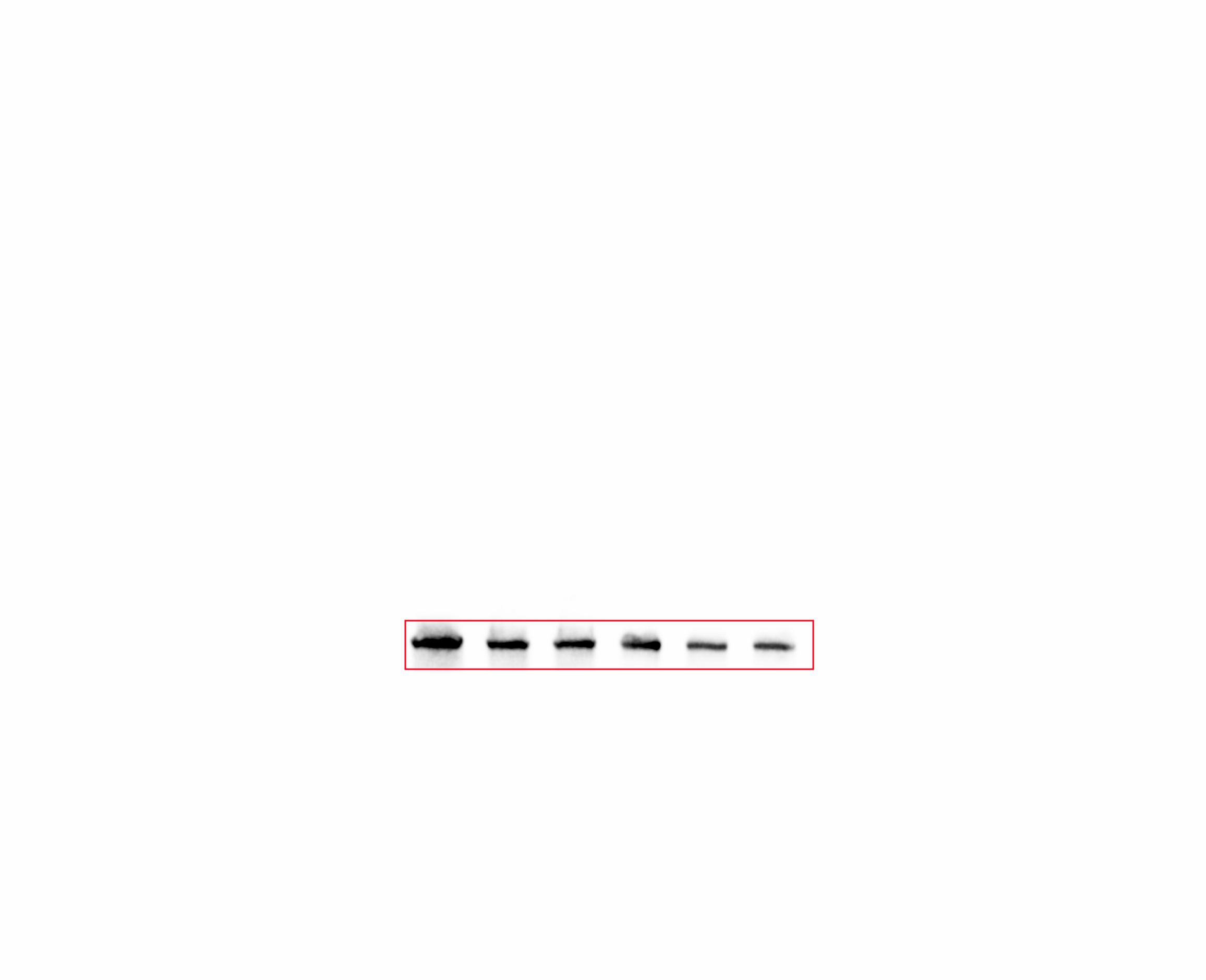

Supplement: Supplementary file 6 — Source data Fig. 5 [file 44318_2025_363_MOESM6_ESM.zip › Figure 5/5B/4 ZEB1.tif]

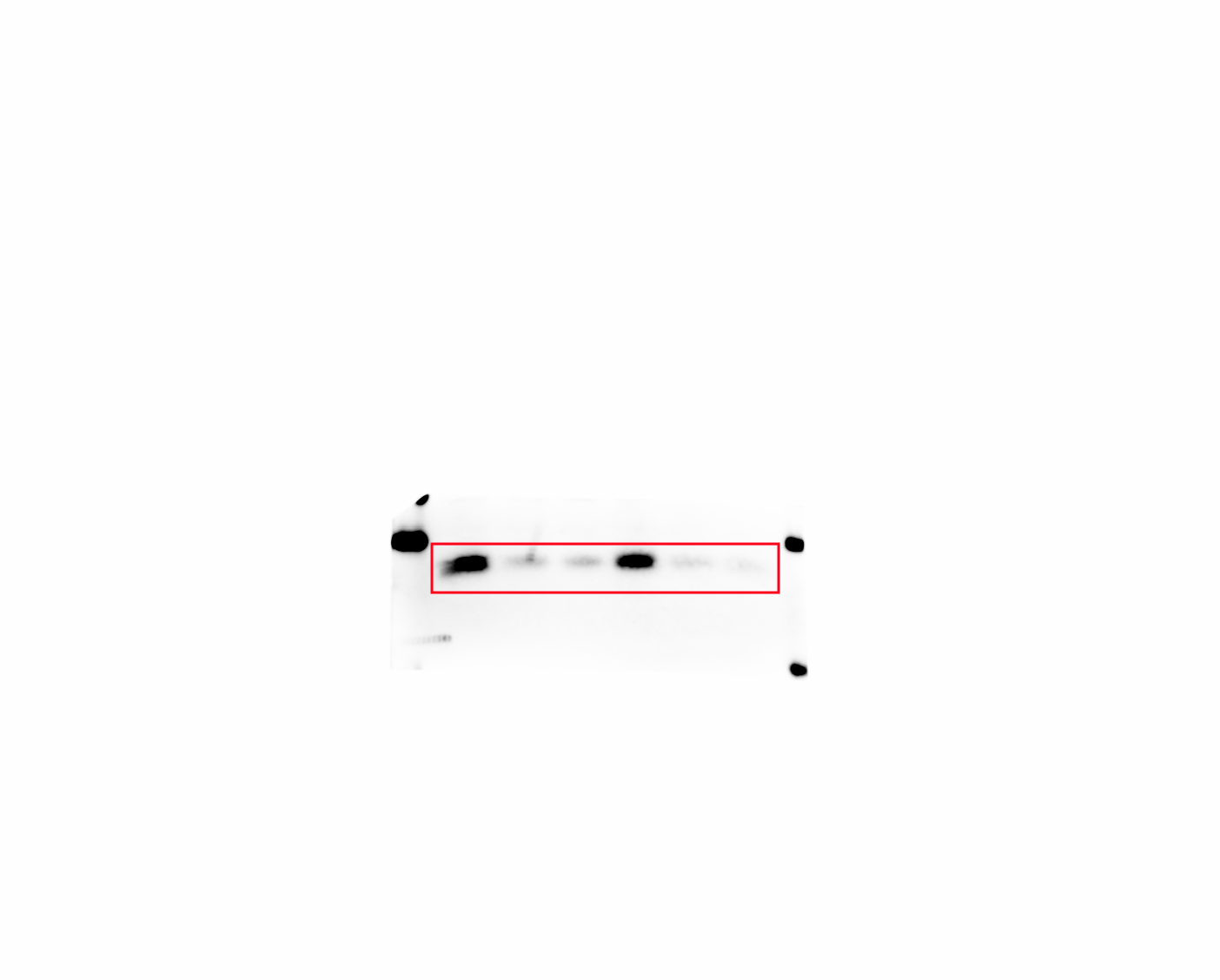

Supplement: Supplementary file 6 — Source data Fig. 5 [file 44318_2025_363_MOESM6_ESM.zip › Figure 5/5B/5 Ephrin A1.tif]

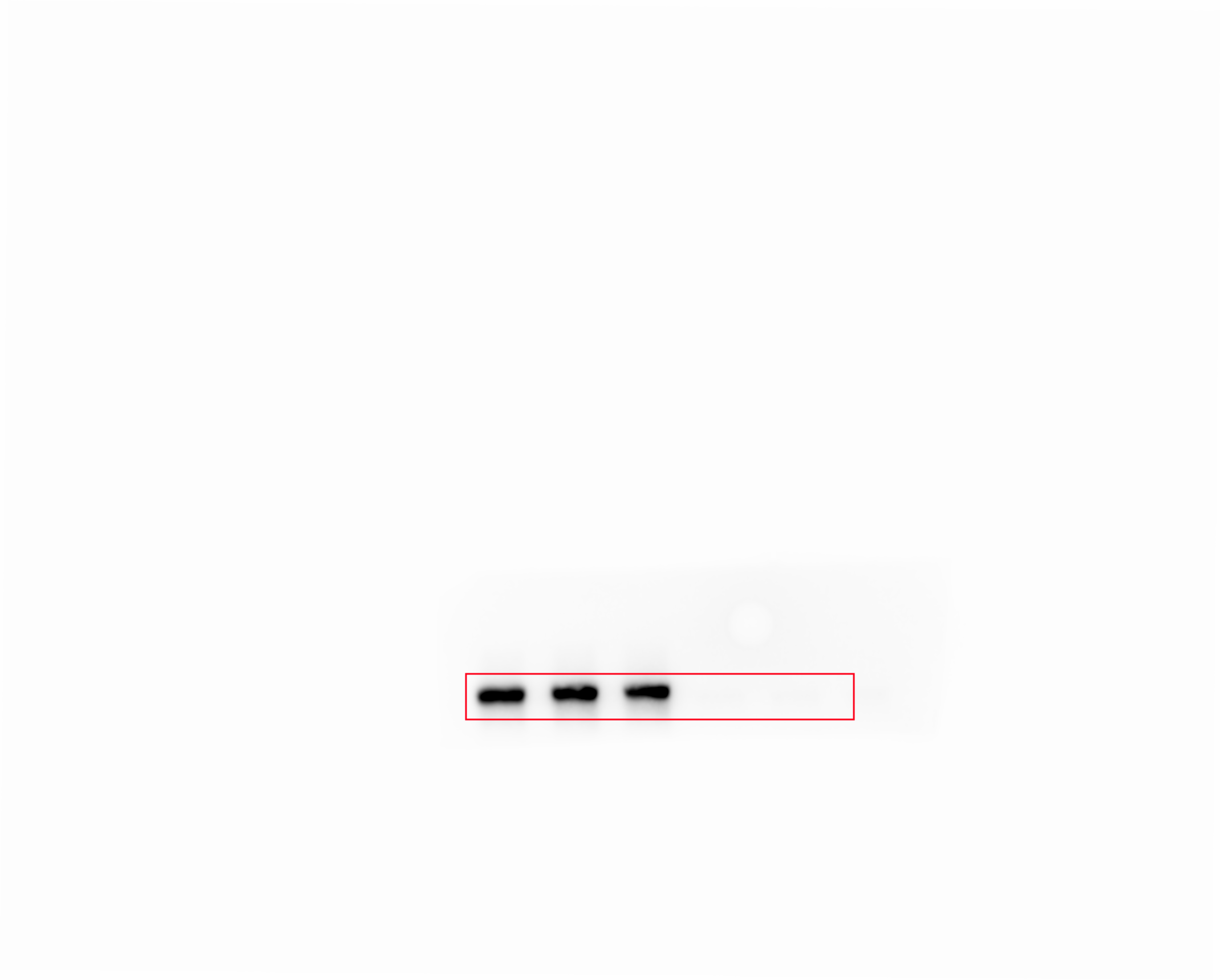

Supplement: Supplementary file 6 — Source data Fig. 5 [file 44318_2025_363_MOESM6_ESM.zip › Figure 5/5B/6 EGFR.tif]

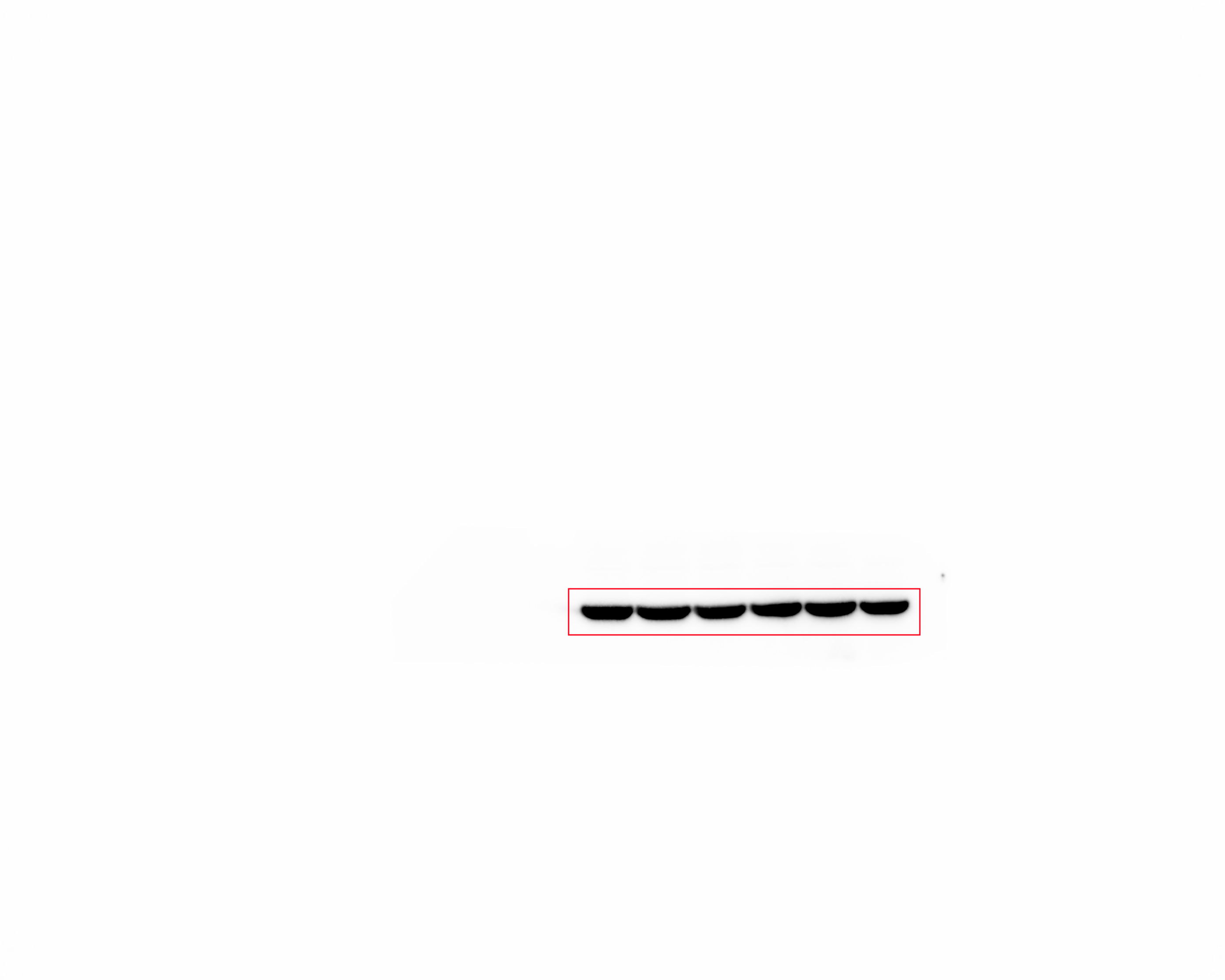

Supplement: Supplementary file 6 — Source data Fig. 5 [file 44318_2025_363_MOESM6_ESM.zip › Figure 5/5B/7 ACTIN.tif]

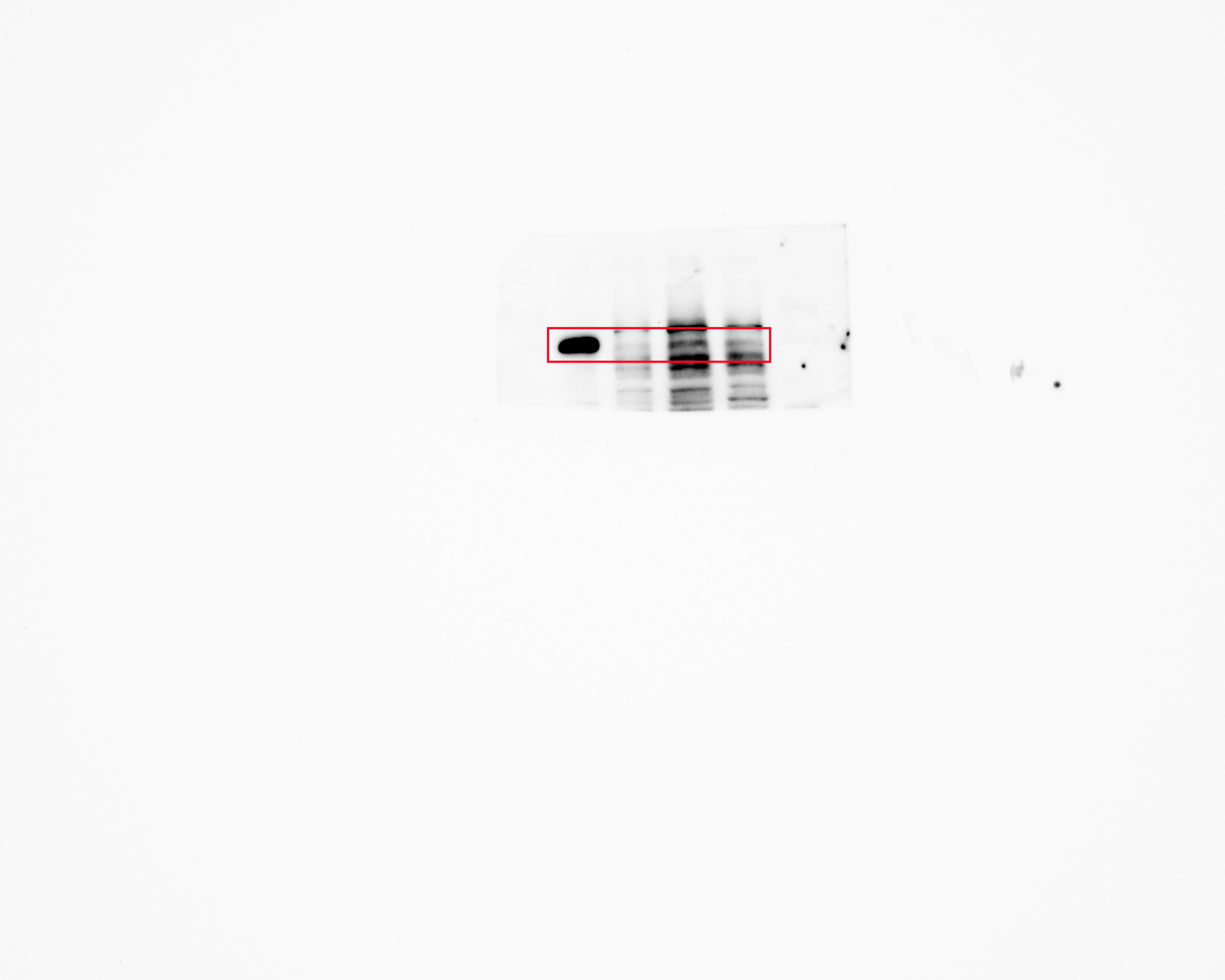

Supplement: Supplementary file 6 — Source data Fig. 5 [file 44318_2025_363_MOESM6_ESM.zip › Figure 5/5C/1 E-cad.tif]

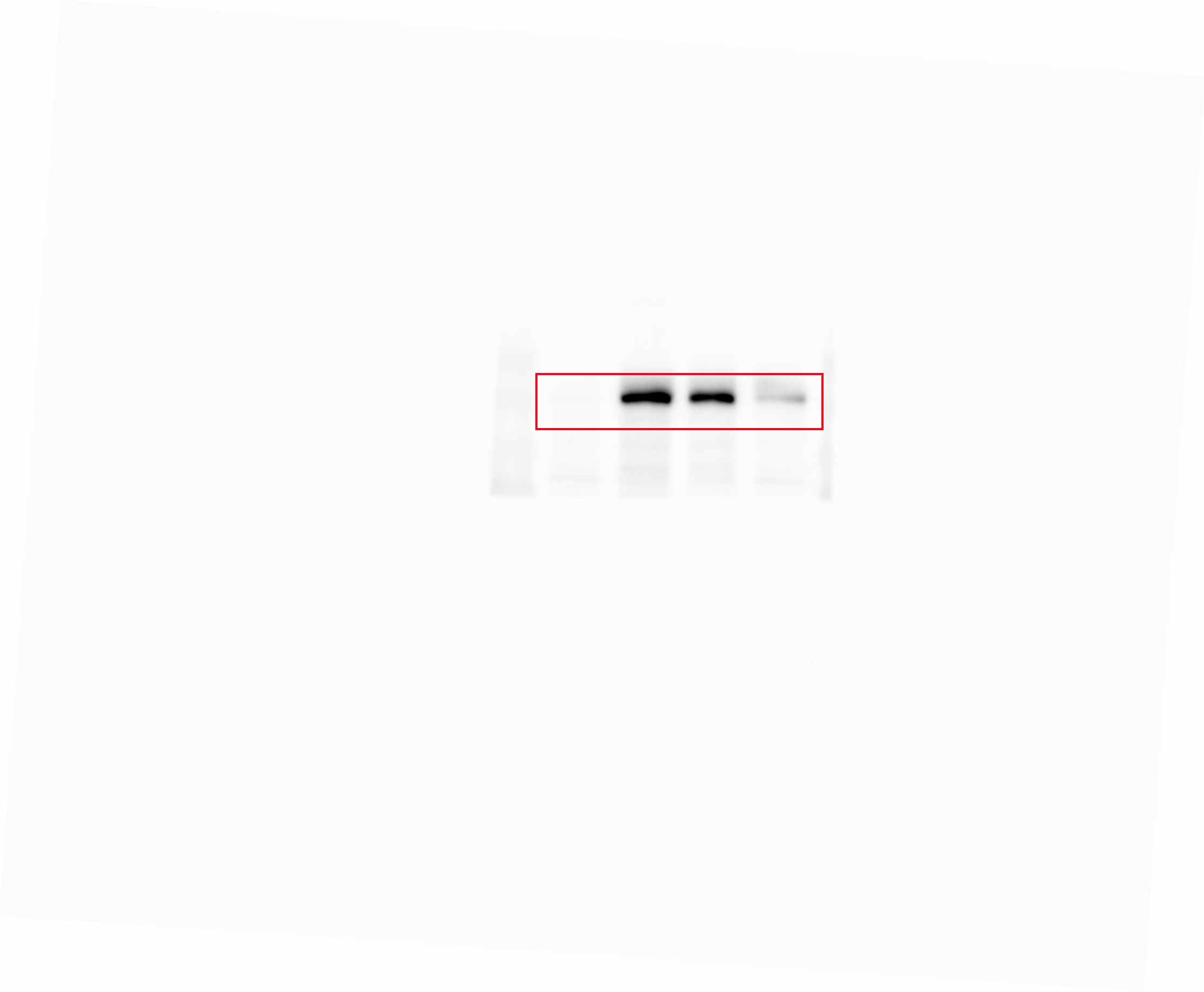

Supplement: Supplementary file 6 — Source data Fig. 5 [file 44318_2025_363_MOESM6_ESM.zip › Figure 5/5C/2 N-cad.tif]

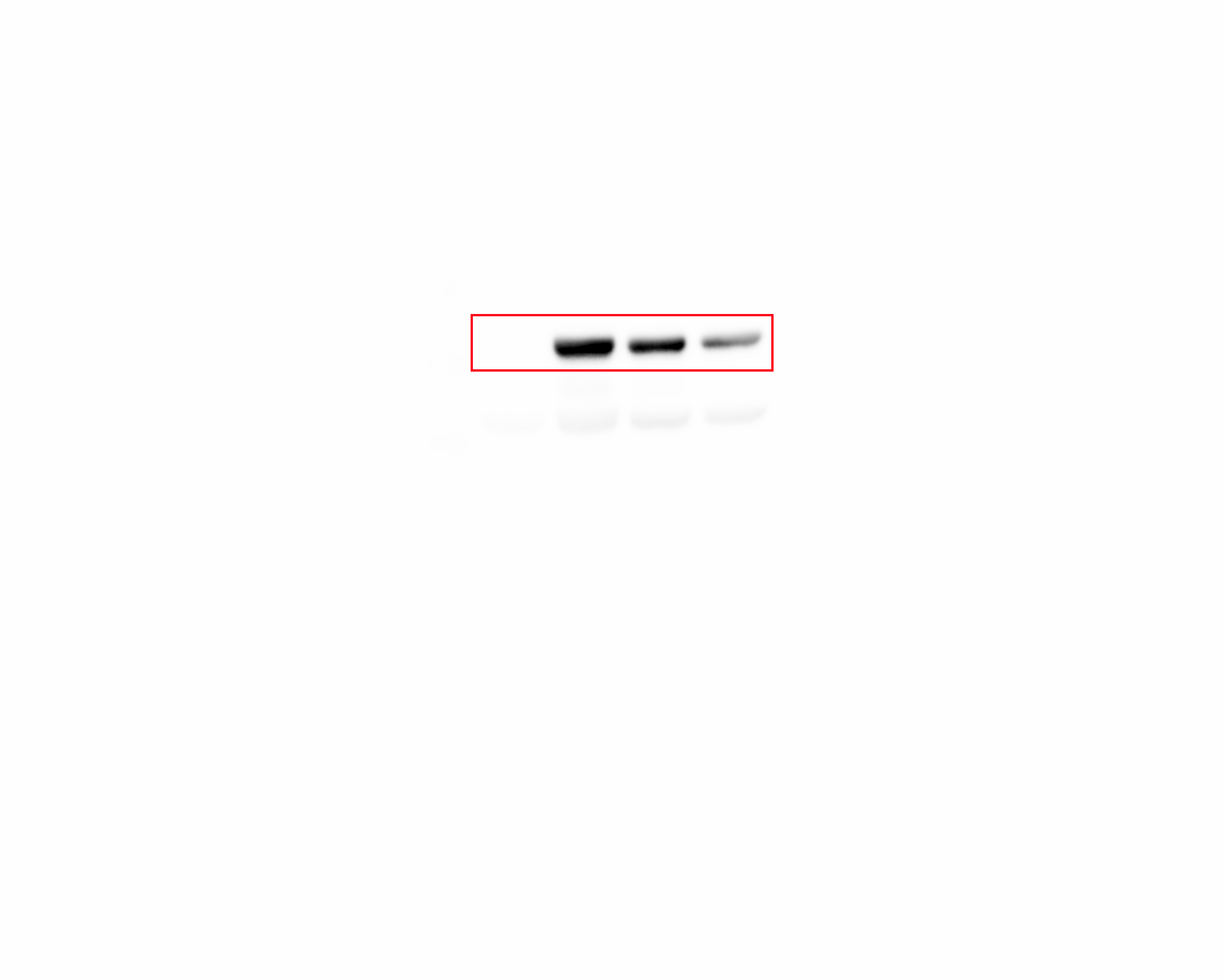

Supplement: Supplementary file 6 — Source data Fig. 5 [file 44318_2025_363_MOESM6_ESM.zip › Figure 5/5C/3 vimentin.tif]

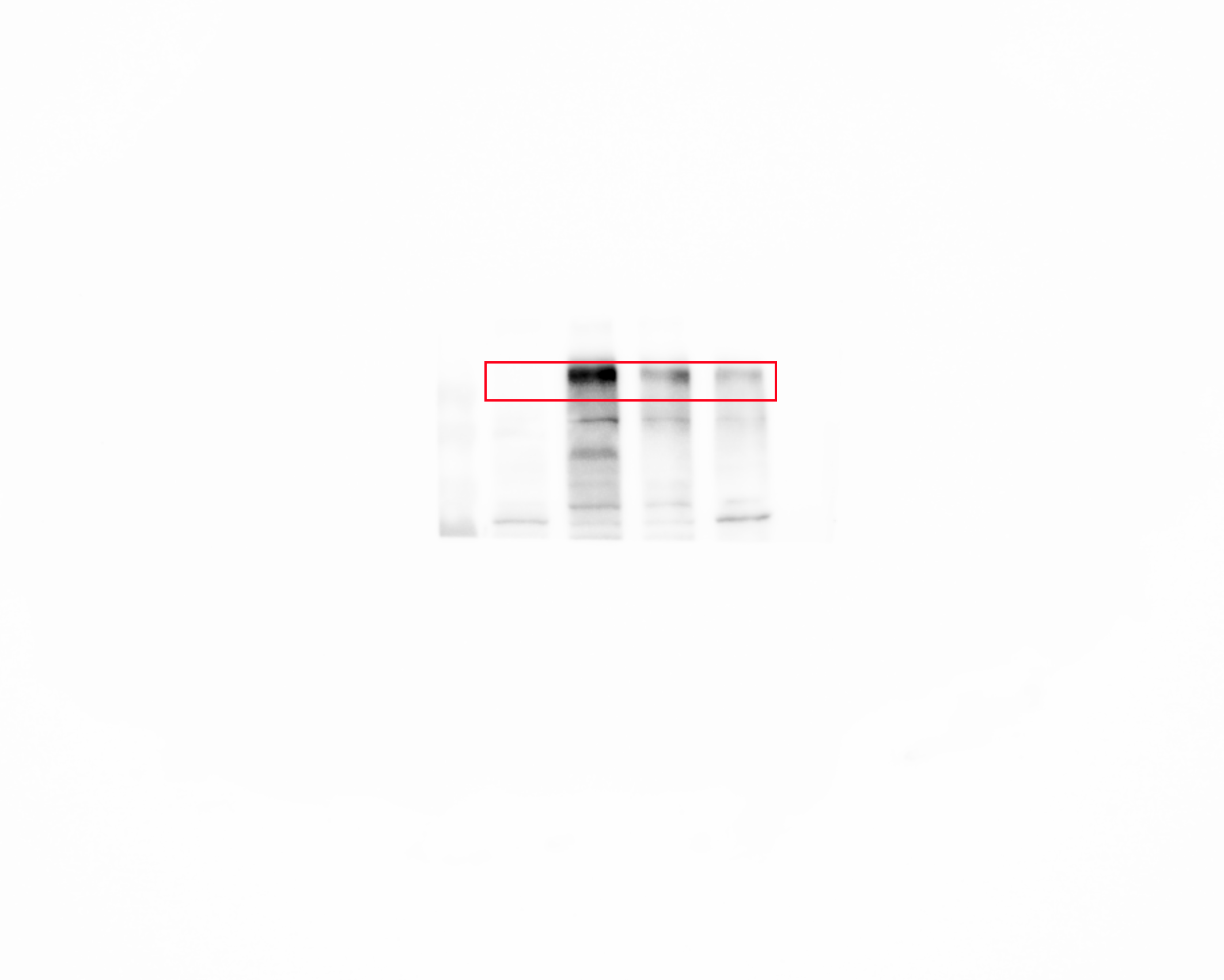

Supplement: Supplementary file 6 — Source data Fig. 5 [file 44318_2025_363_MOESM6_ESM.zip › Figure 5/5C/4 ZEB1.tif]

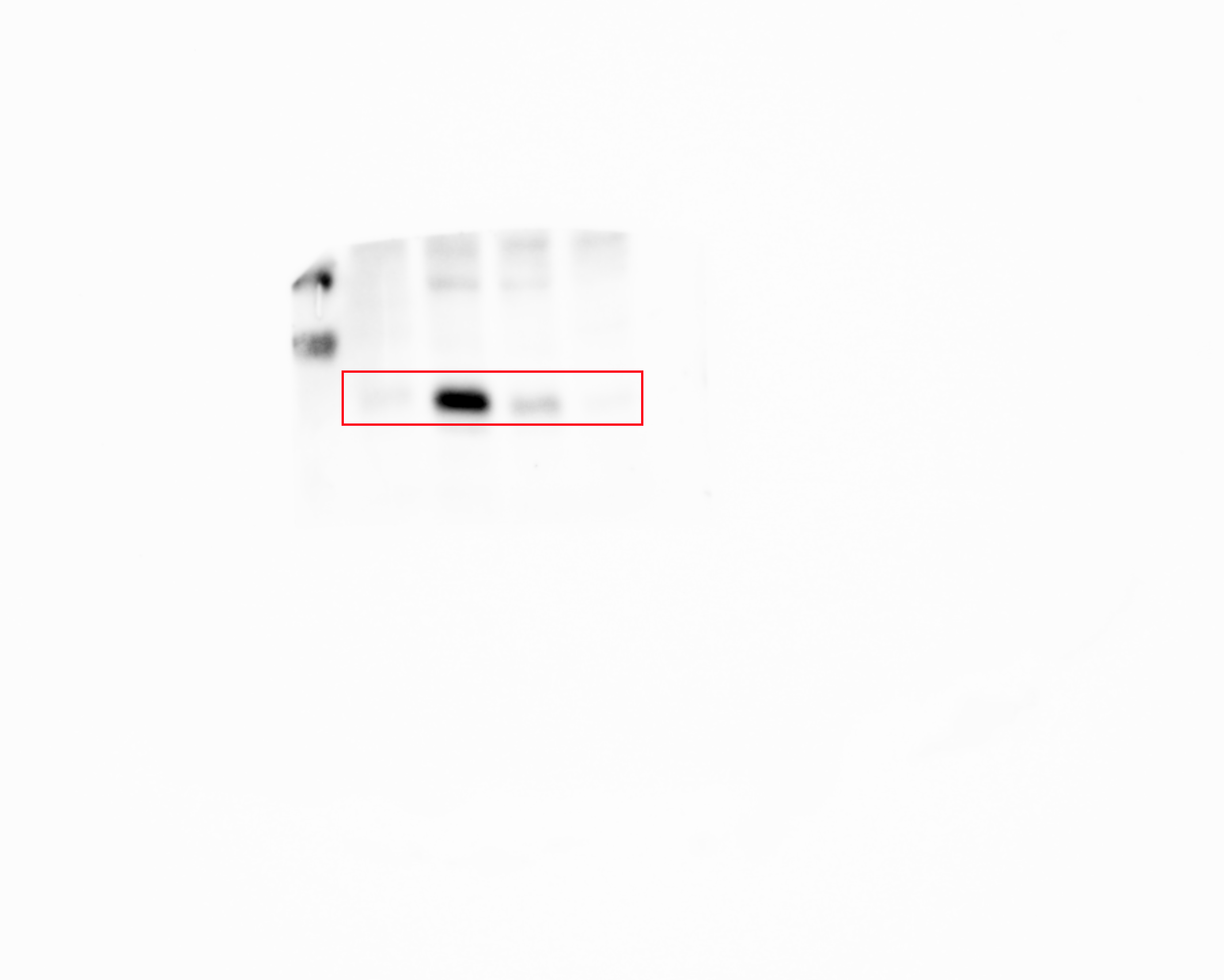

Supplement: Supplementary file 6 — Source data Fig. 5 [file 44318_2025_363_MOESM6_ESM.zip › Figure 5/5C/5 Ephrin A1.tif]

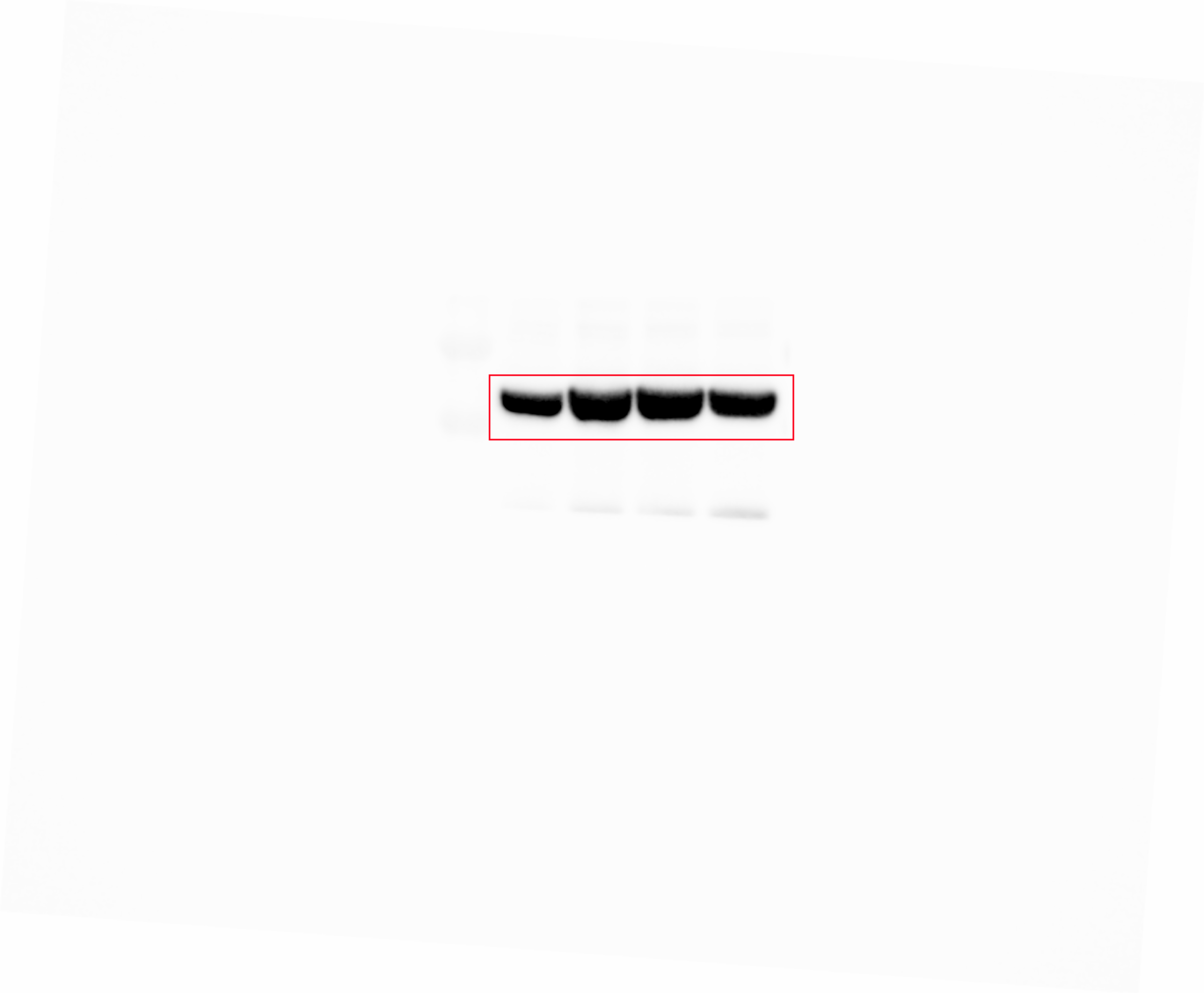

Supplement: Supplementary file 6 — Source data Fig. 5 [file 44318_2025_363_MOESM6_ESM.zip › Figure 5/5C/6 actin.tif]

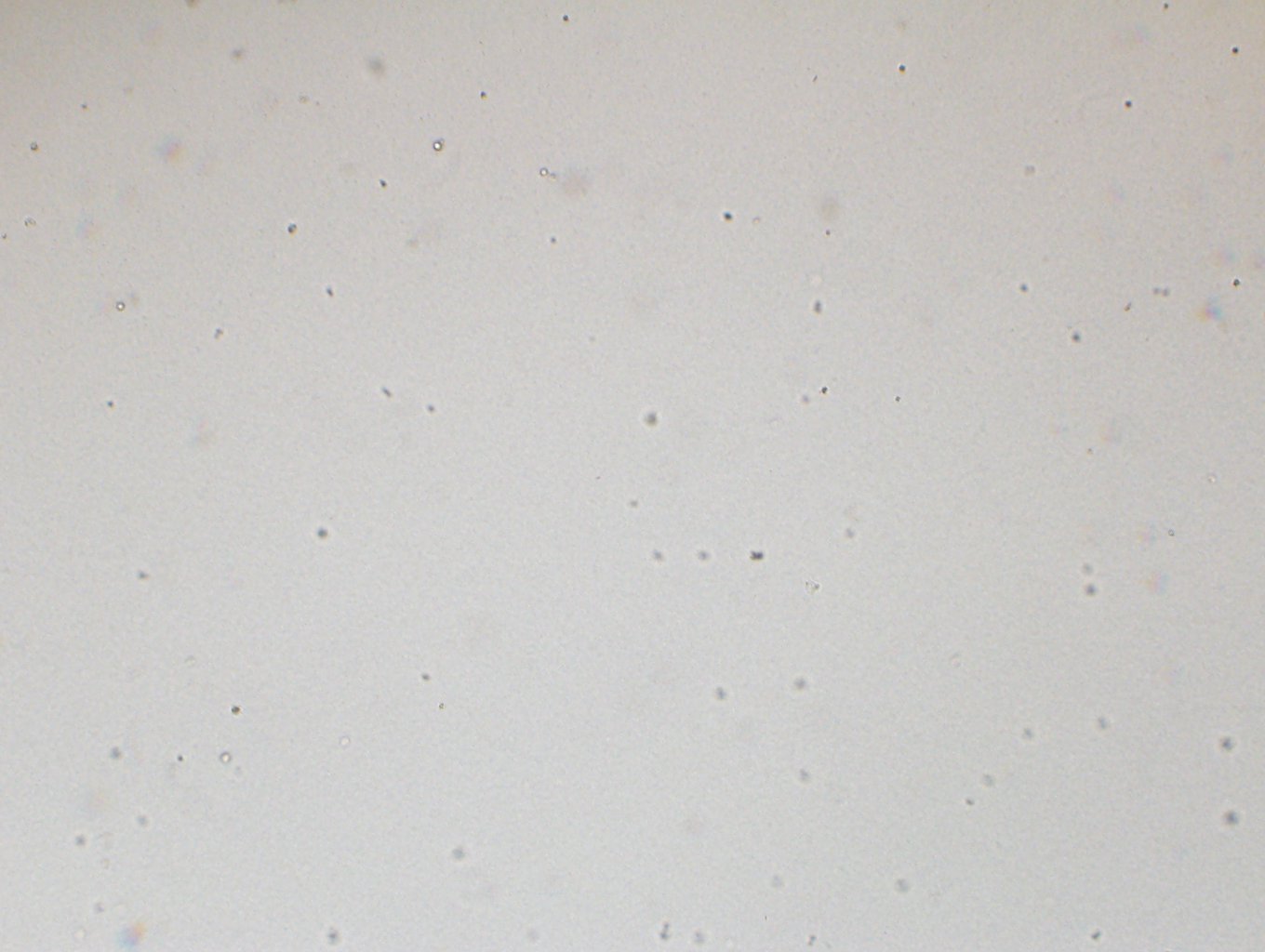

Supplement: Supplementary file 6 — Source data Fig. 5 [file 44318_2025_363_MOESM6_ESM.zip › Figure 5/5D/Control (1).jpg]

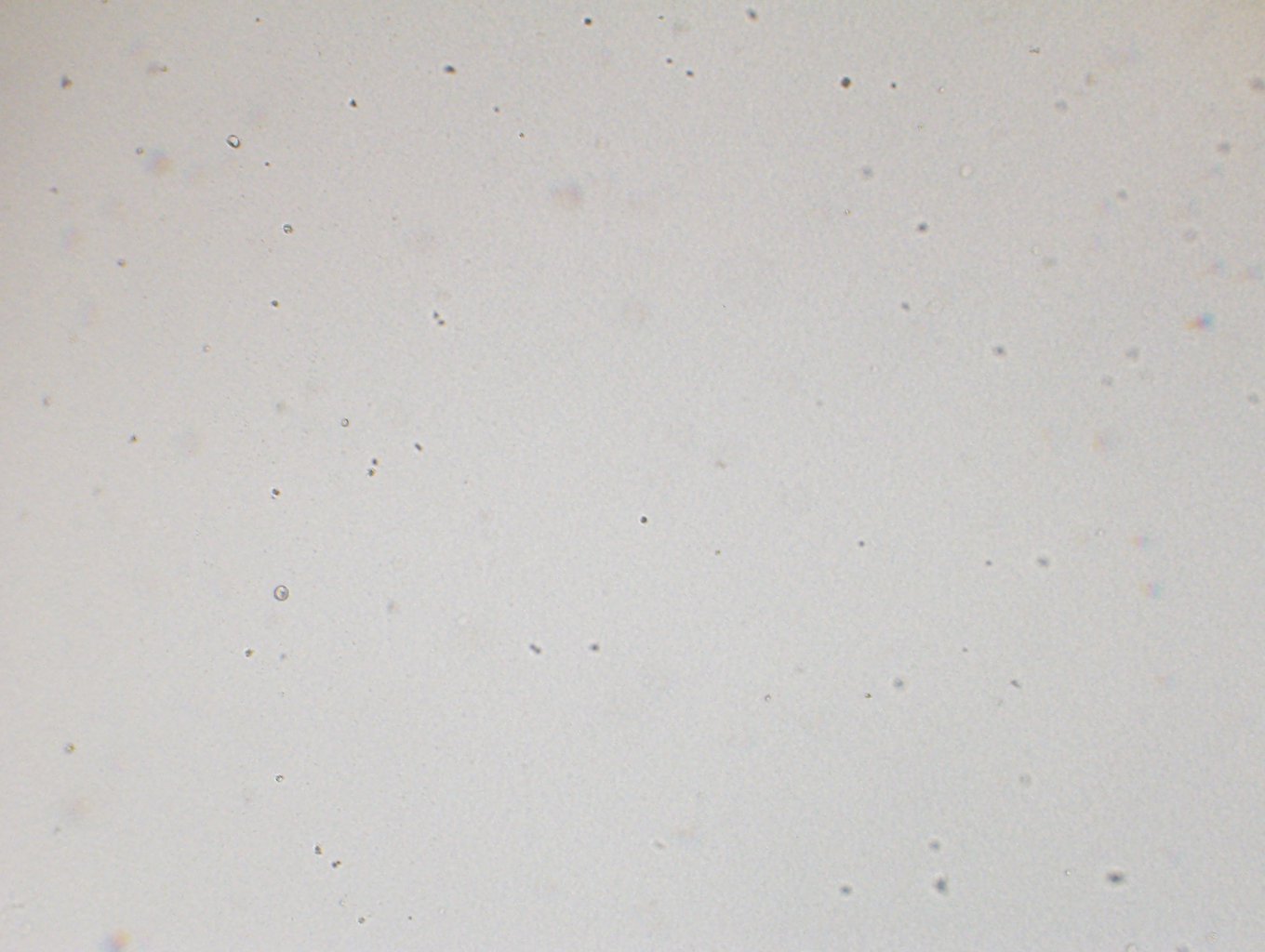

Supplement: Supplementary file 6 — Source data Fig. 5 [file 44318_2025_363_MOESM6_ESM.zip › Figure 5/5D/Control (2)-displayed in 5D.jpg]

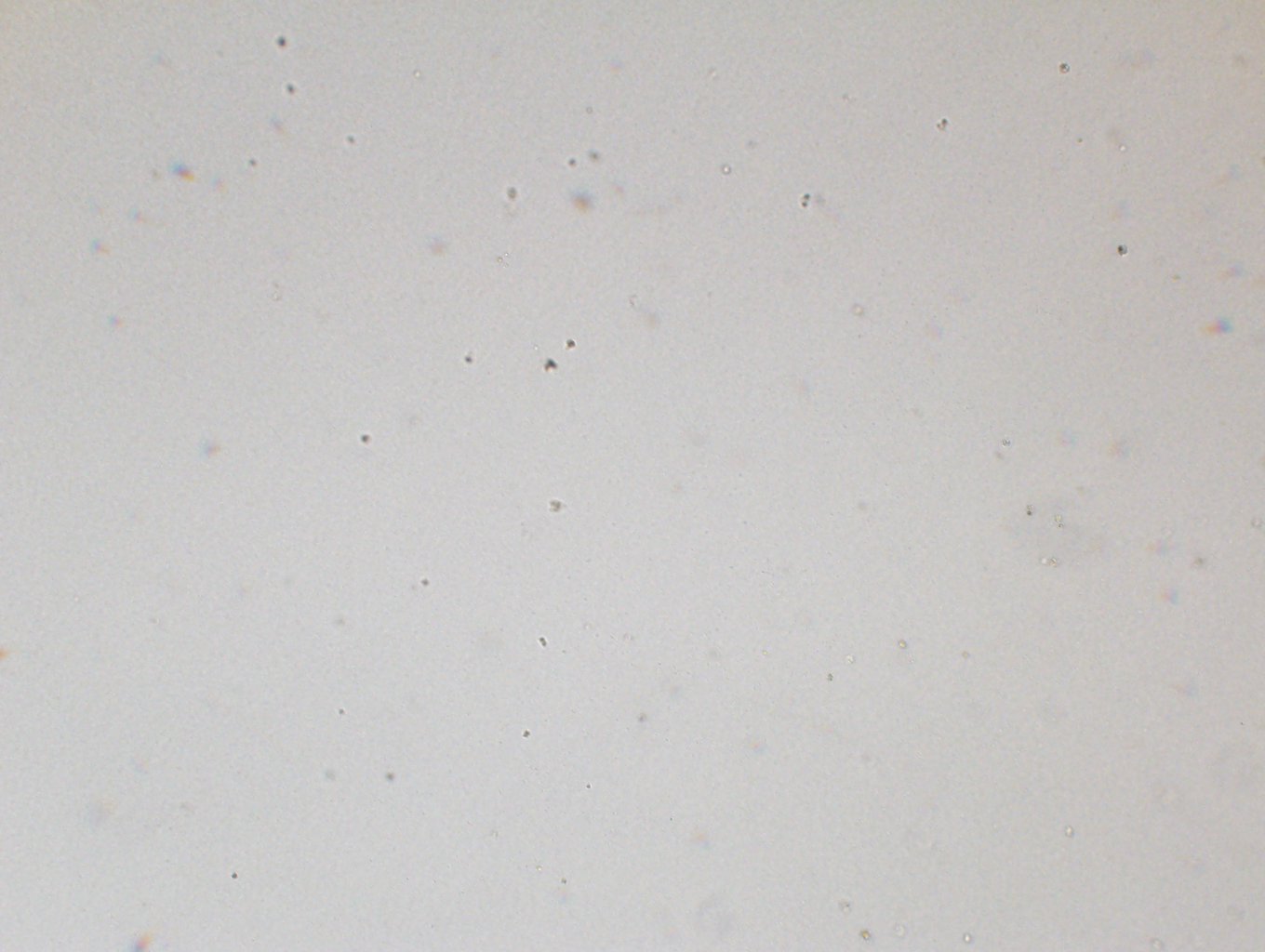

Supplement: Supplementary file 6 — Source data Fig. 5 [file 44318_2025_363_MOESM6_ESM.zip › Figure 5/5D/Control (3).jpg]

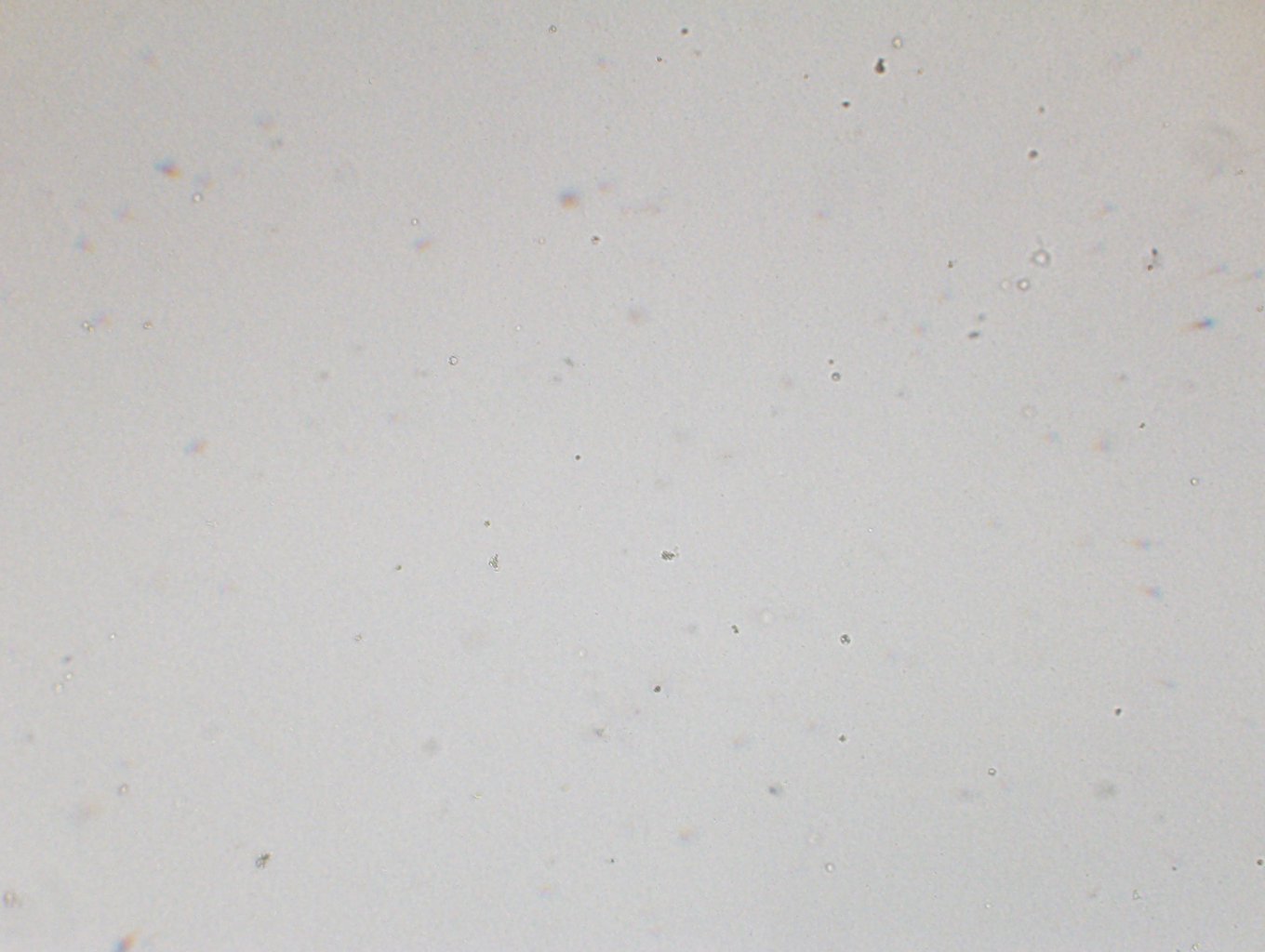

Supplement: Supplementary file 6 — Source data Fig. 5 [file 44318_2025_363_MOESM6_ESM.zip › Figure 5/5D/Control (4).jpg]

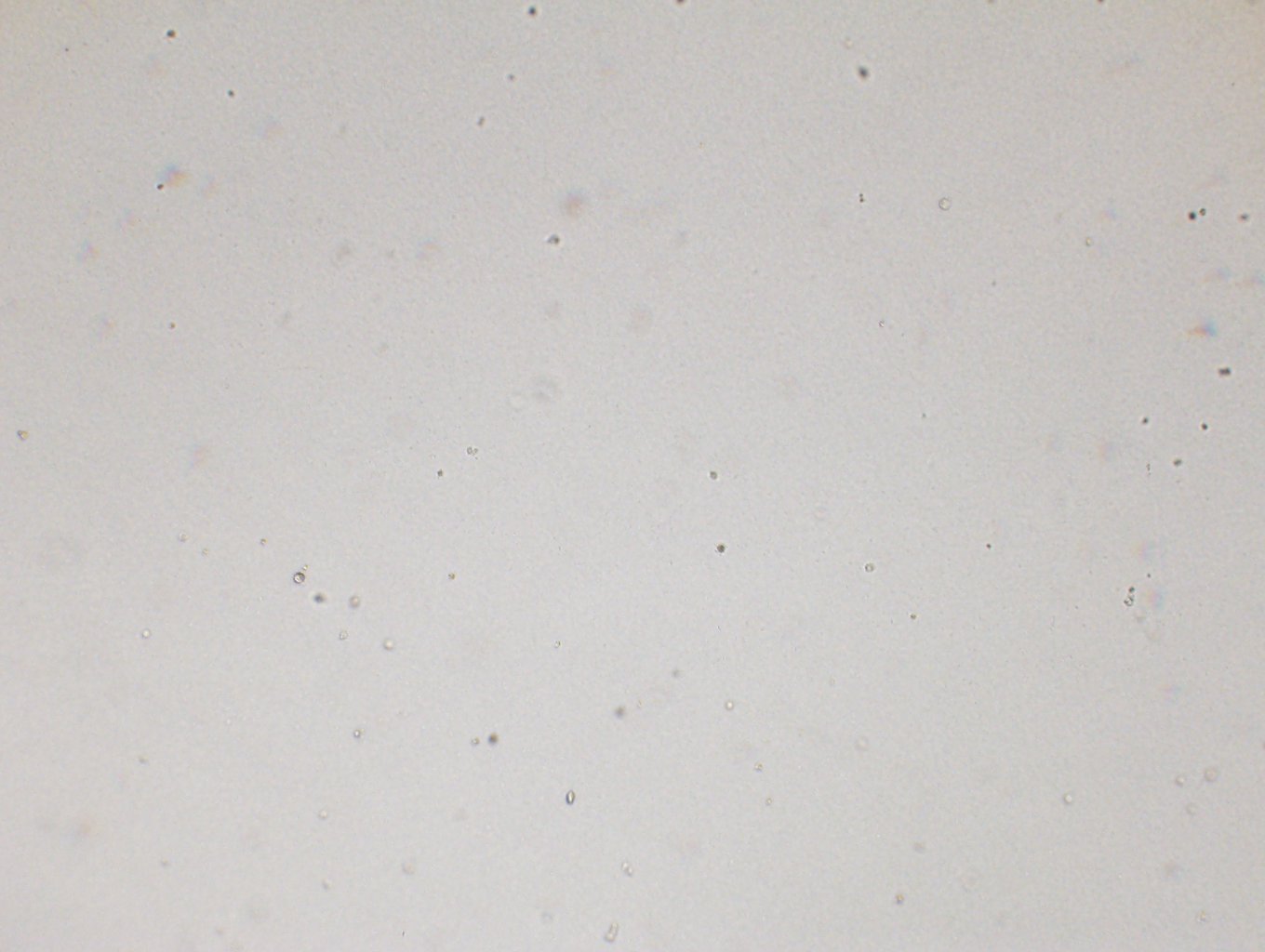

Supplement: Supplementary file 6 — Source data Fig. 5 [file 44318_2025_363_MOESM6_ESM.zip › Figure 5/5D/Control (5).jpg]

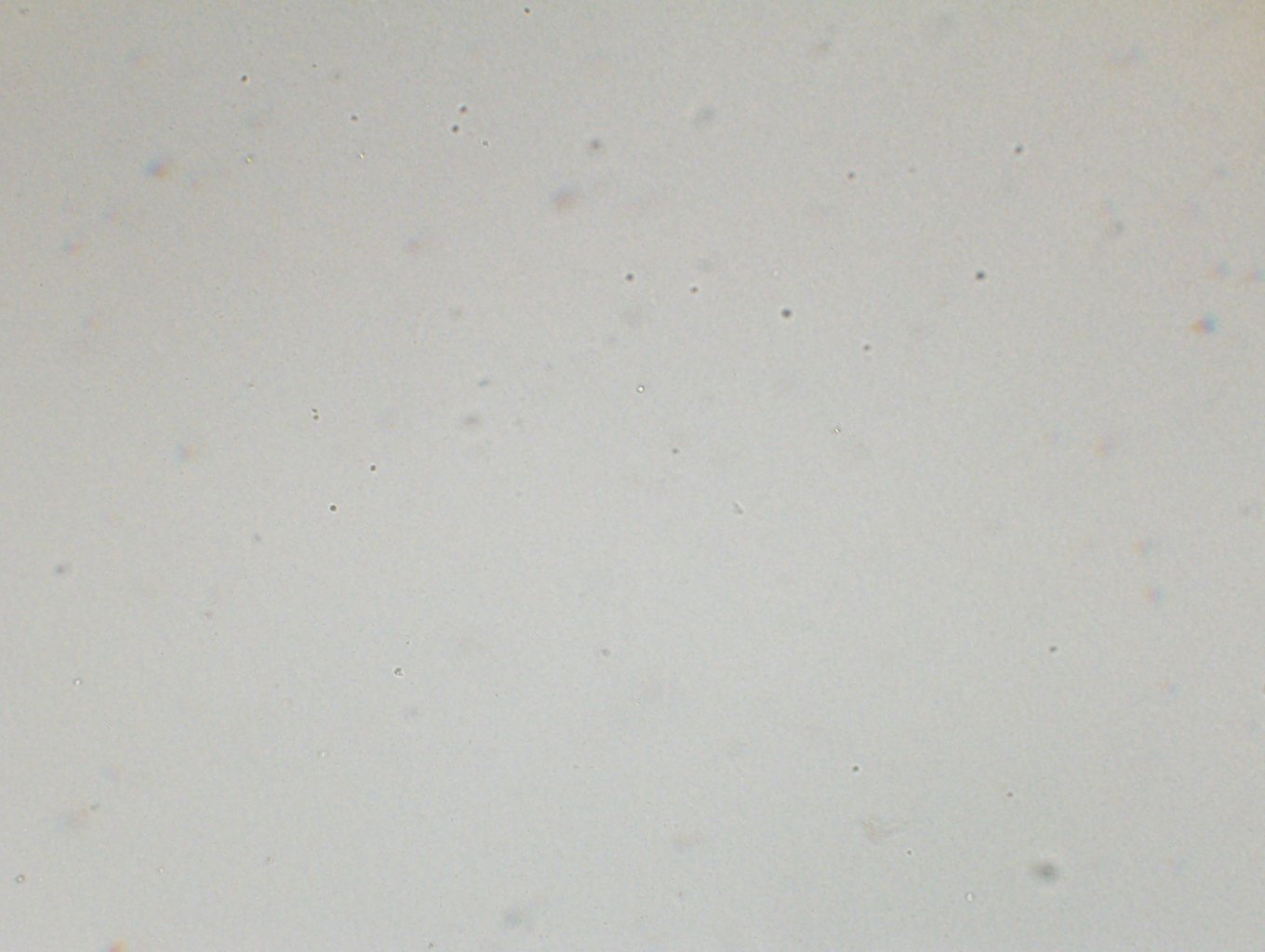

Supplement: Supplementary file 6 — Source data Fig. 5 [file 44318_2025_363_MOESM6_ESM.zip › Figure 5/5D/Control (6).jpg]

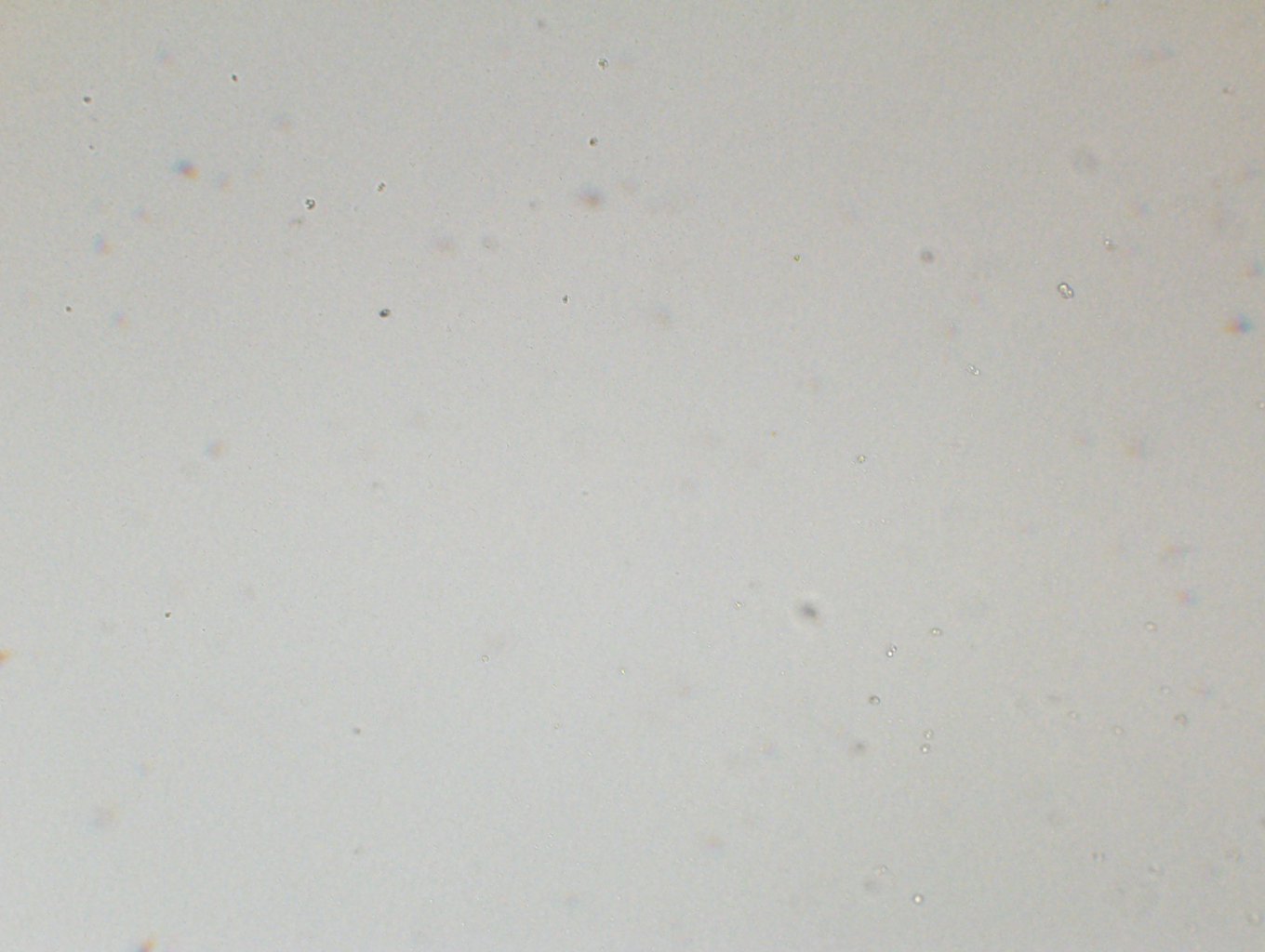

Supplement: Supplementary file 6 — Source data Fig. 5 [file 44318_2025_363_MOESM6_ESM.zip › Figure 5/5D/Control (7).jpg]

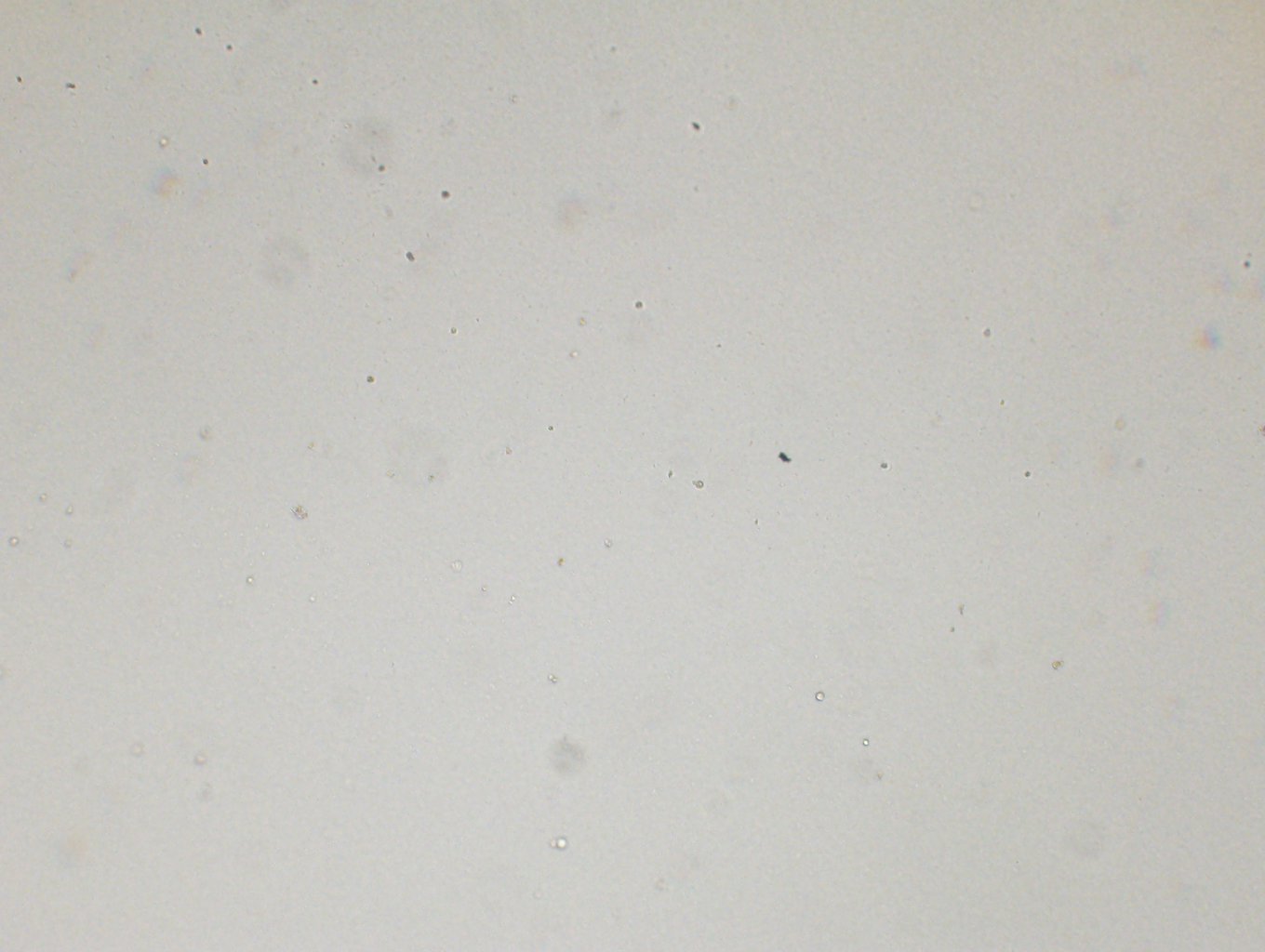

Supplement: Supplementary file 6 — Source data Fig. 5 [file 44318_2025_363_MOESM6_ESM.zip › Figure 5/5D/Control (8).jpg]

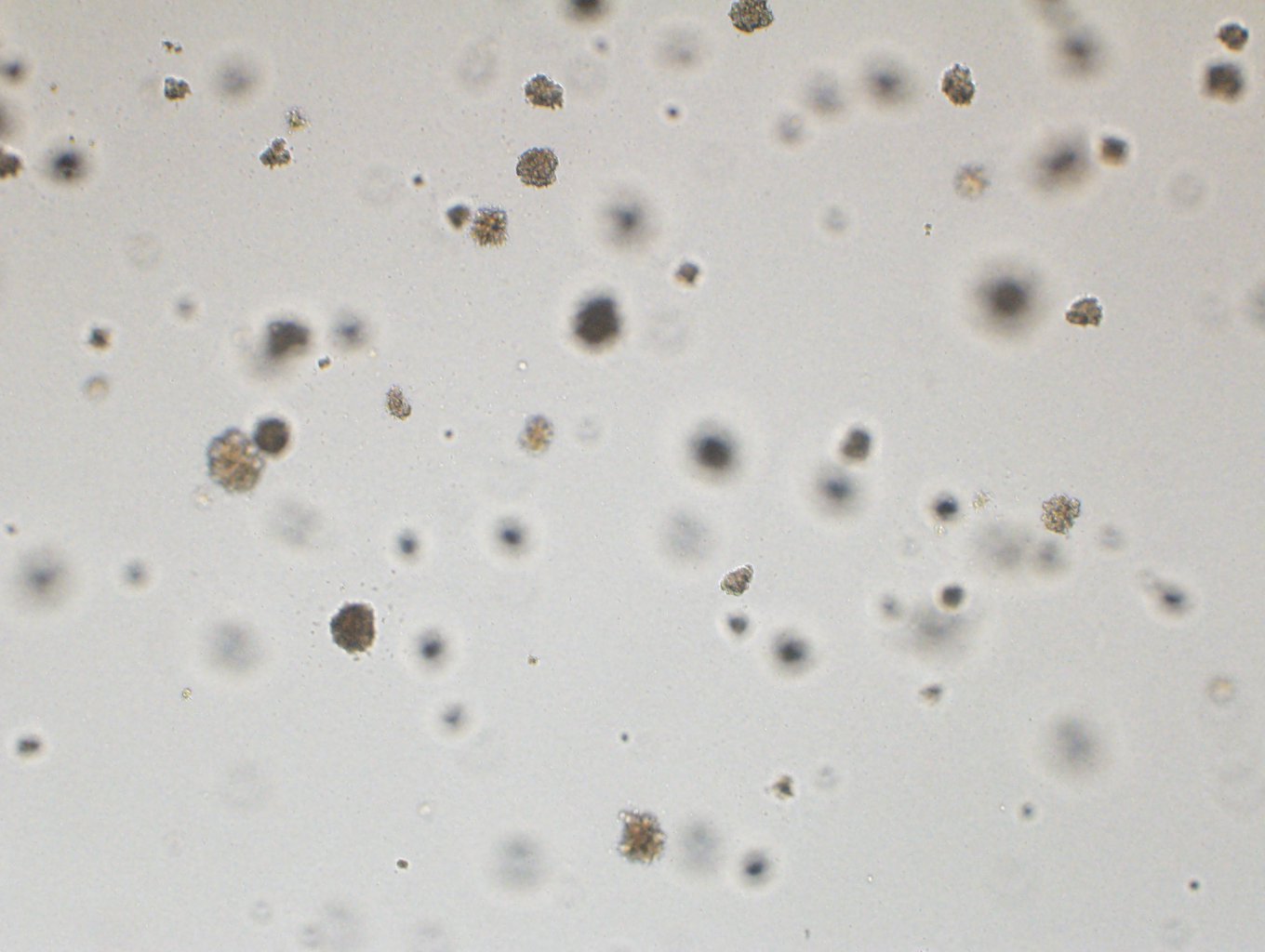

Supplement: Supplementary file 6 — Source data Fig. 5 [file 44318_2025_363_MOESM6_ESM.zip › Figure 5/5D/Ephrin A1 (1).jpg]

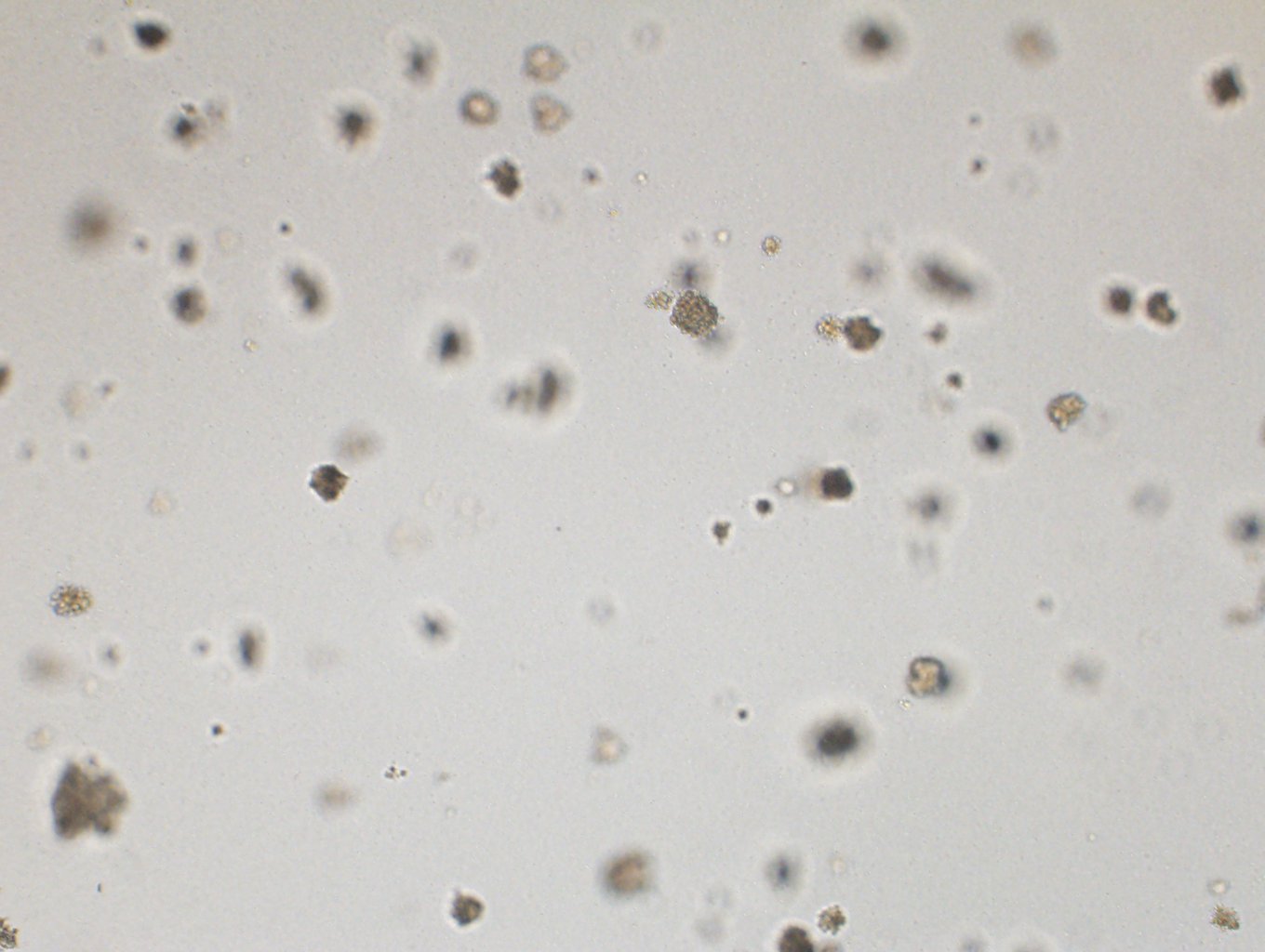

Supplement: Supplementary file 6 — Source data Fig. 5 [file 44318_2025_363_MOESM6_ESM.zip › Figure 5/5D/Ephrin A1 (2).jpg]

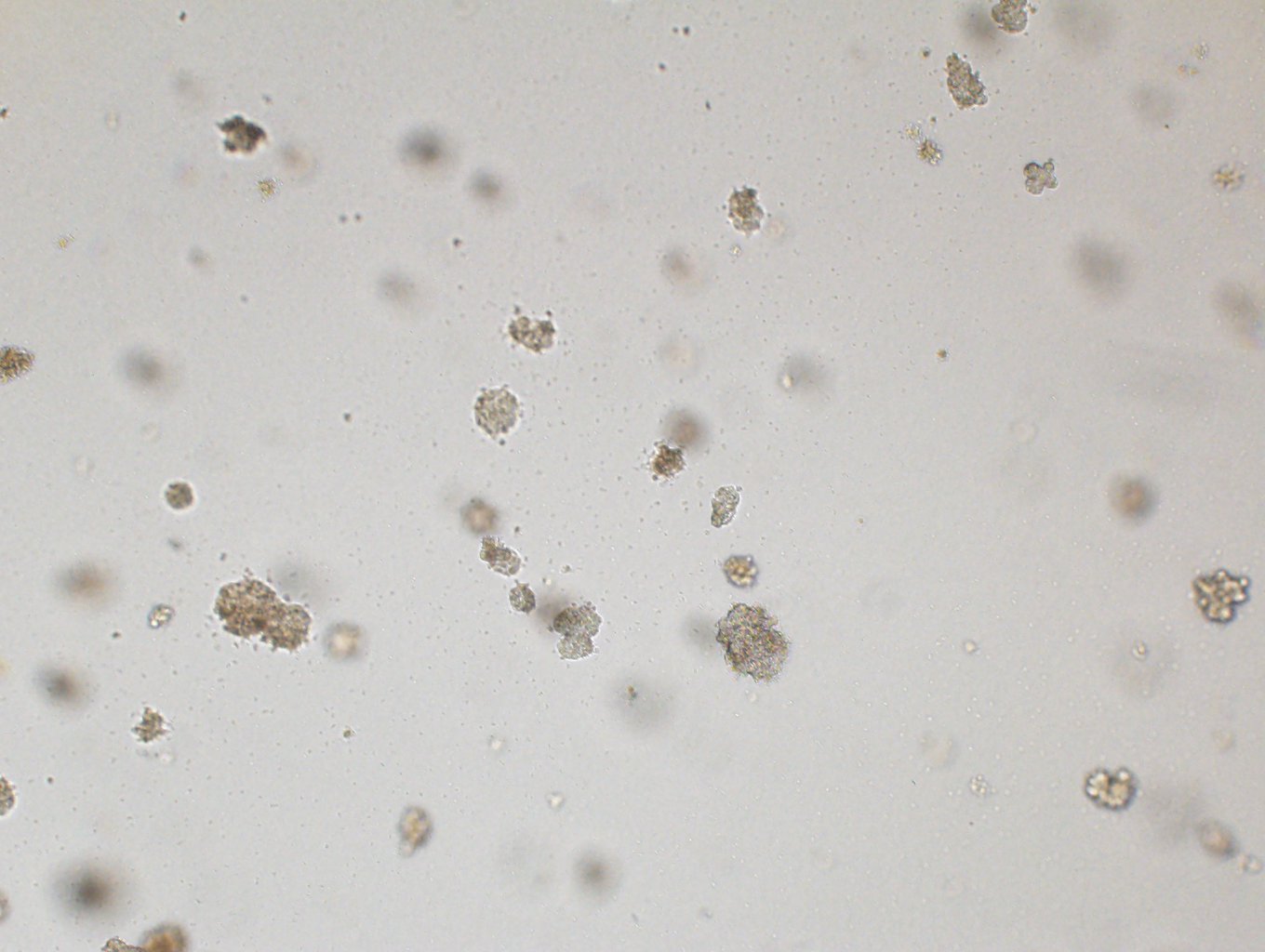

Supplement: Supplementary file 6 — Source data Fig. 5 [file 44318_2025_363_MOESM6_ESM.zip › Figure 5/5D/Ephrin A1 (3).jpg]

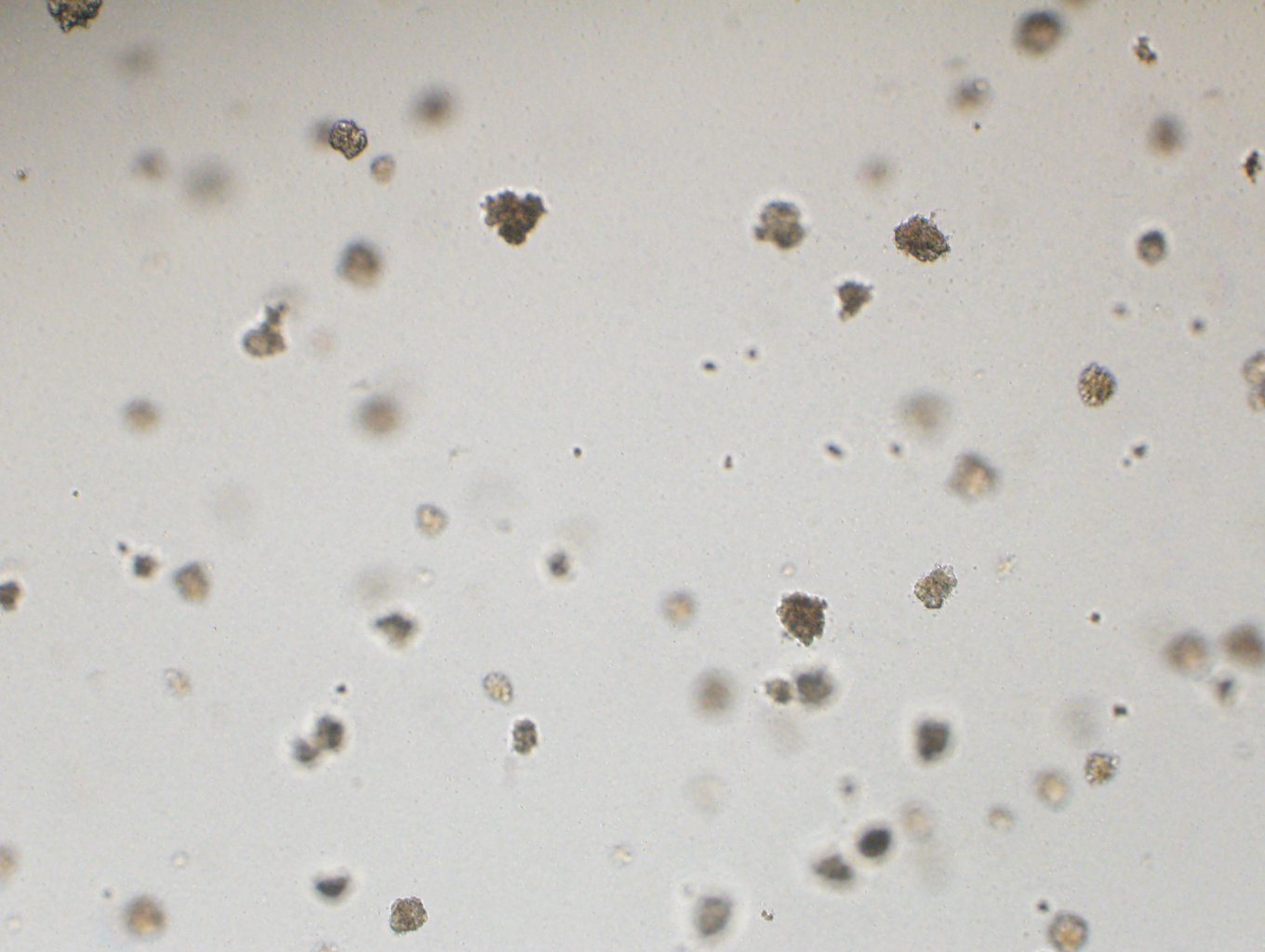

Supplement: Supplementary file 6 — Source data Fig. 5 [file 44318_2025_363_MOESM6_ESM.zip › Figure 5/5D/Ephrin A1 (4)-displayed in 5D.jpg]

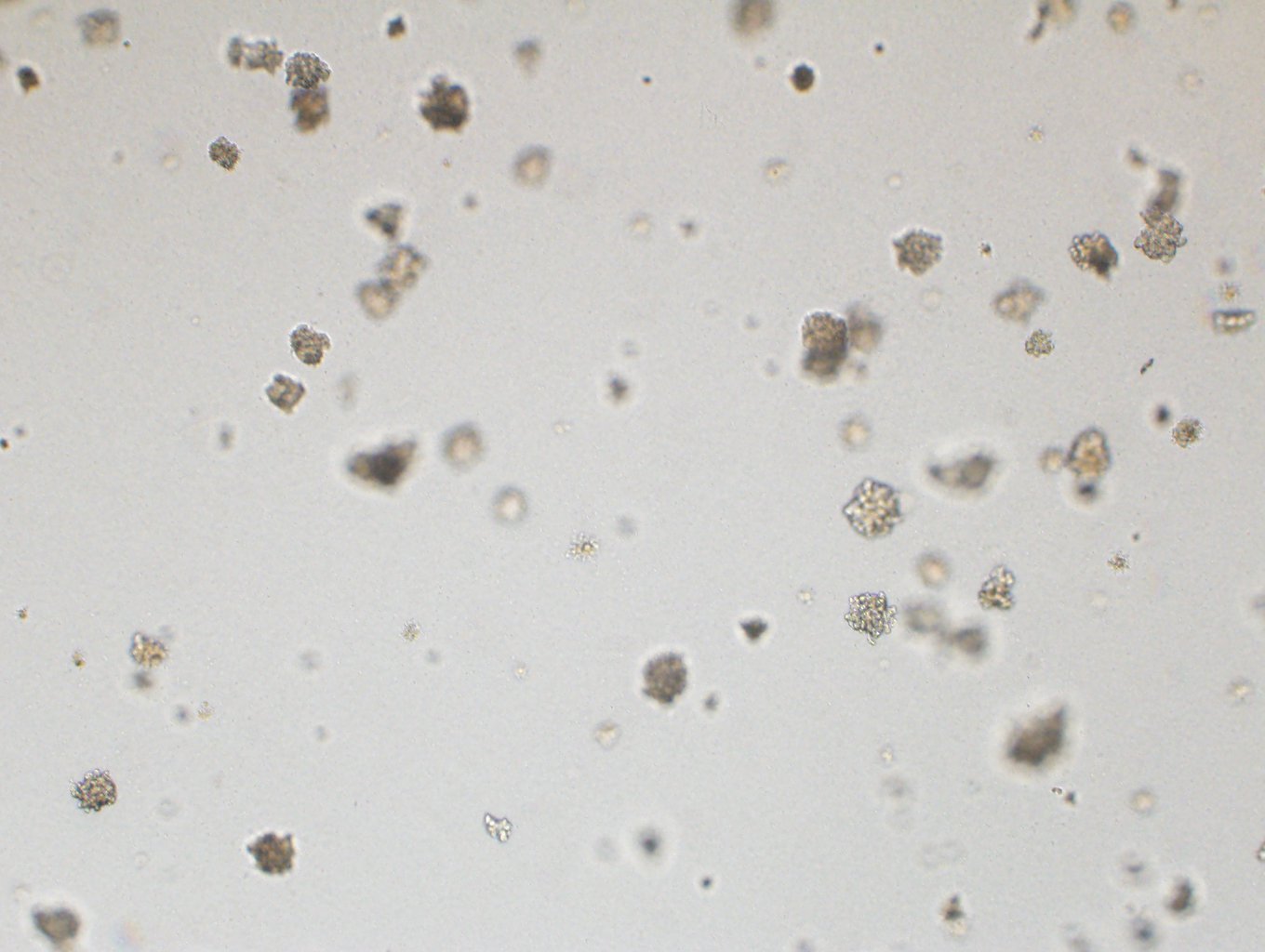

Supplement: Supplementary file 6 — Source data Fig. 5 [file 44318_2025_363_MOESM6_ESM.zip › Figure 5/5D/Ephrin A1 (5).jpg]

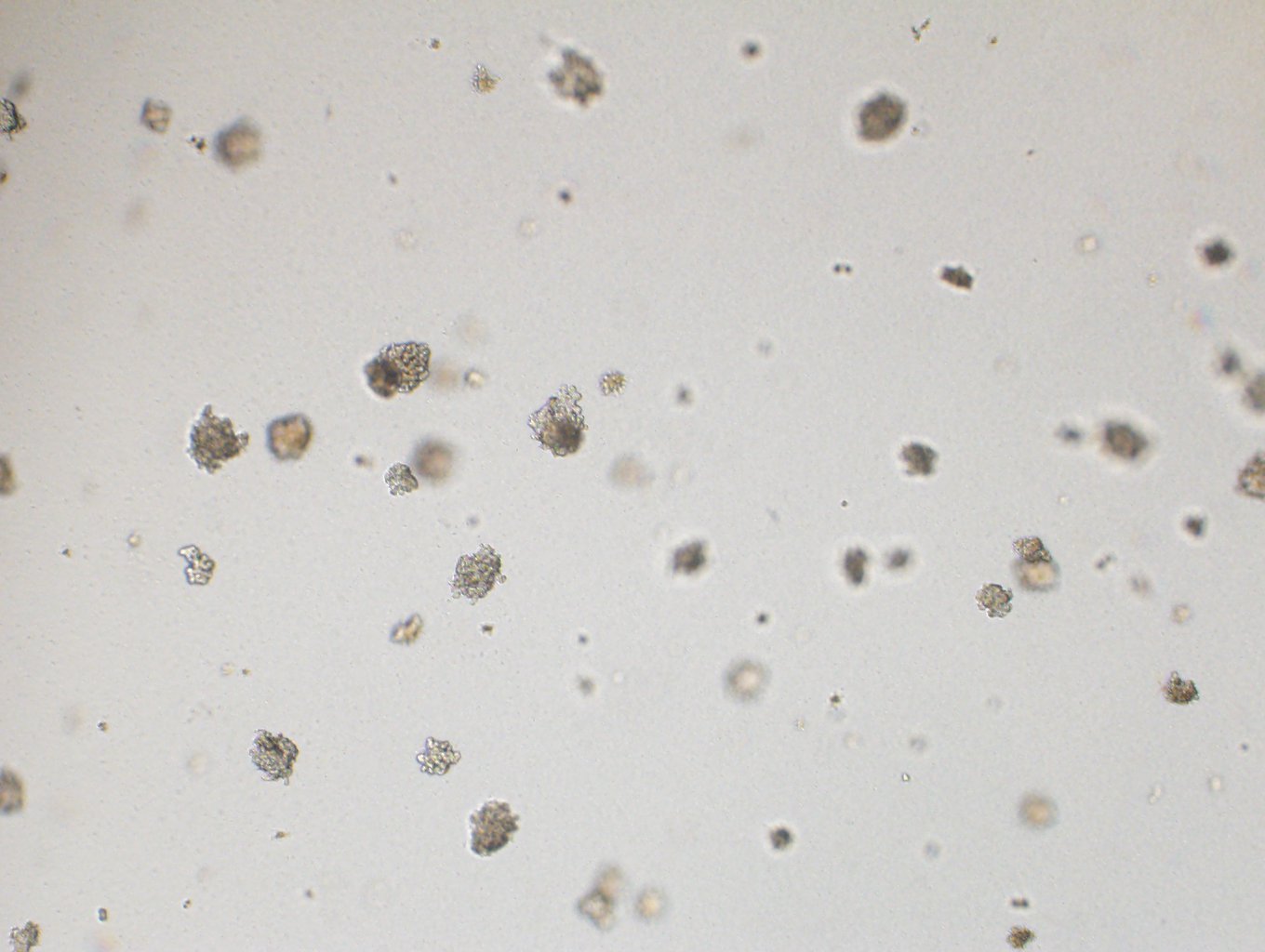

Supplement: Supplementary file 6 — Source data Fig. 5 [file 44318_2025_363_MOESM6_ESM.zip › Figure 5/5D/Ephrin A1 (6).jpg]

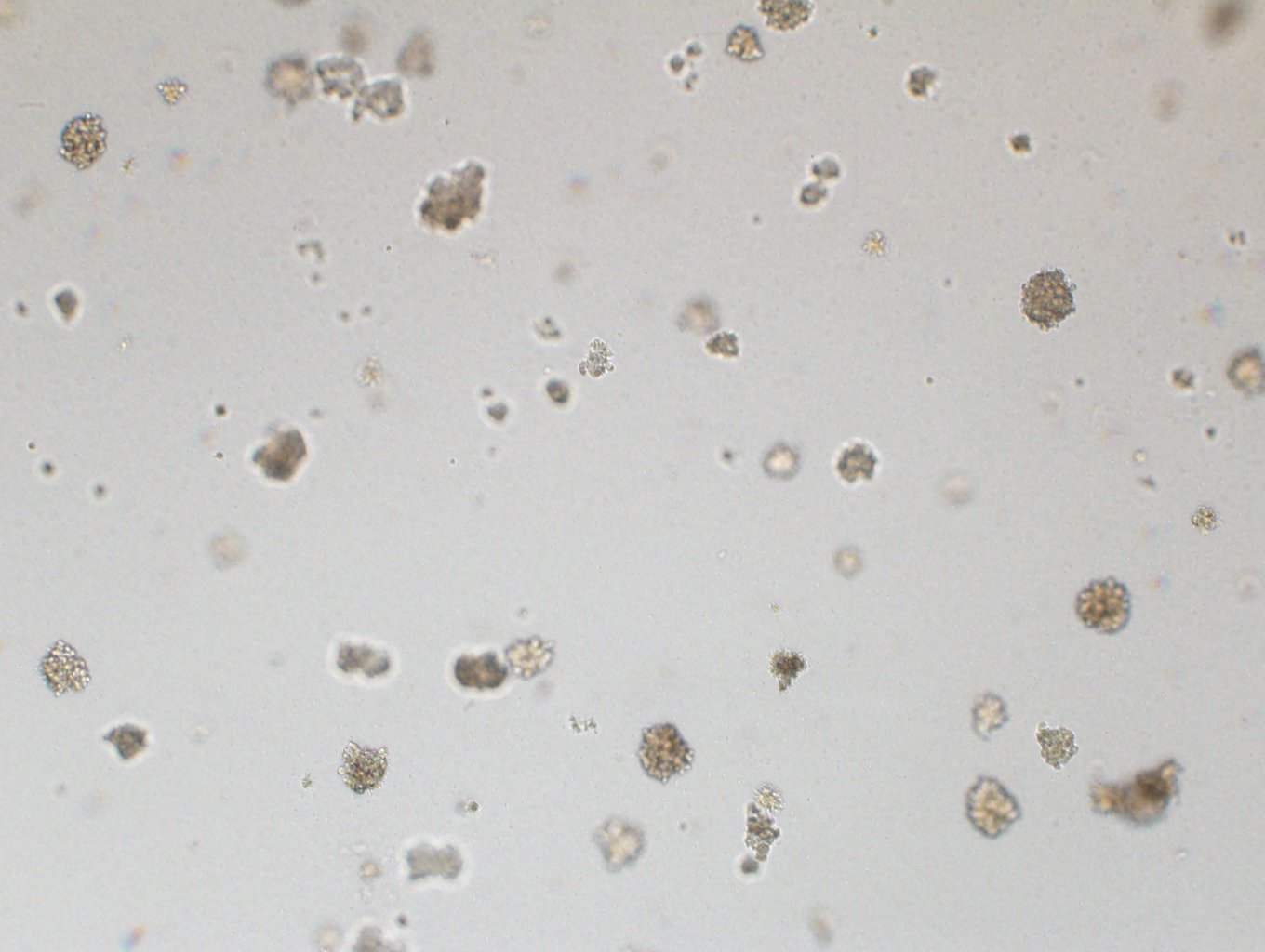

Supplement: Supplementary file 6 — Source data Fig. 5 [file 44318_2025_363_MOESM6_ESM.zip › Figure 5/5D/Ephrin A1 (7).jpg]

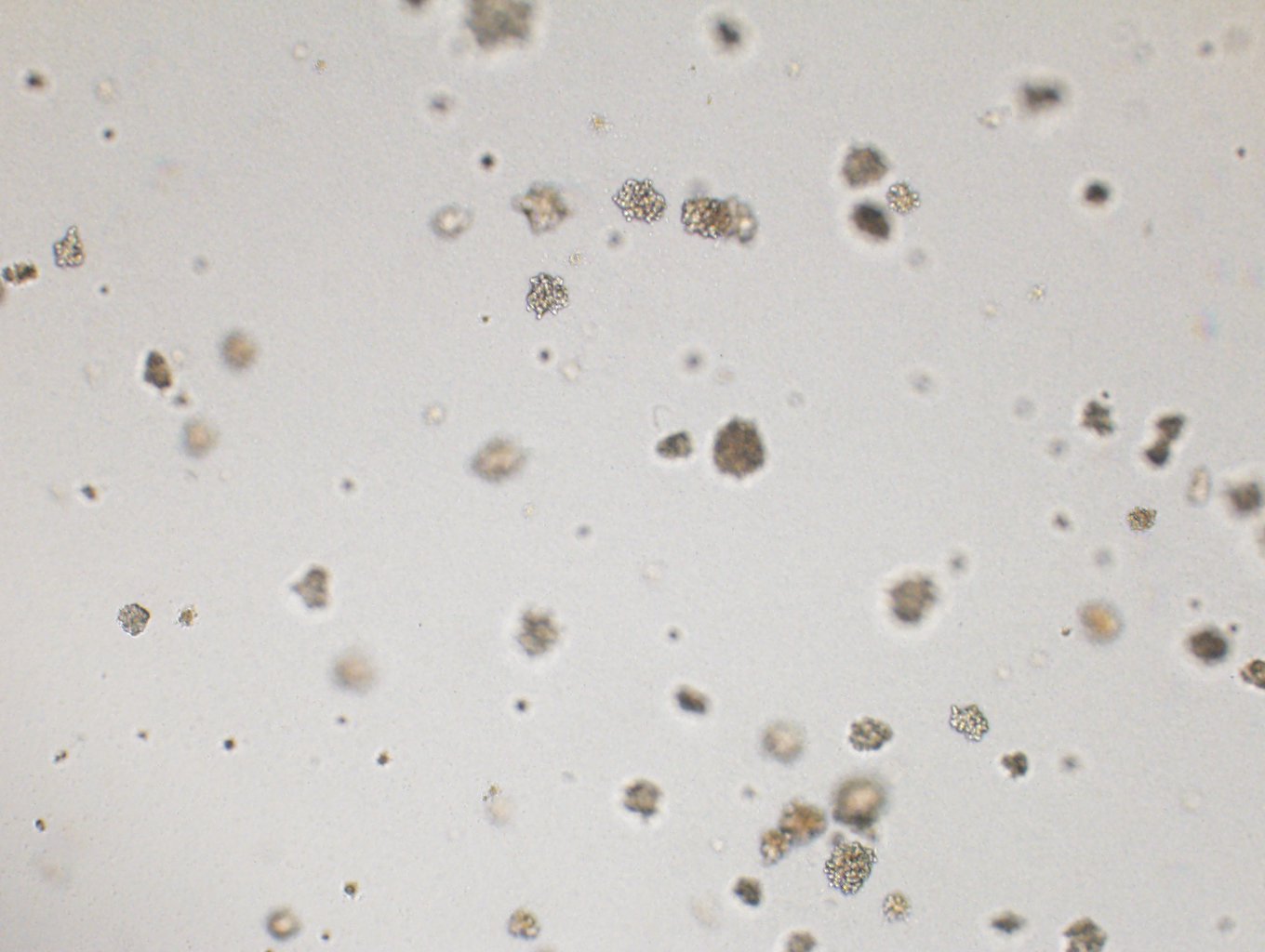

Supplement: Supplementary file 6 — Source data Fig. 5 [file 44318_2025_363_MOESM6_ESM.zip › Figure 5/5D/Ephrin A1 (8).jpg]

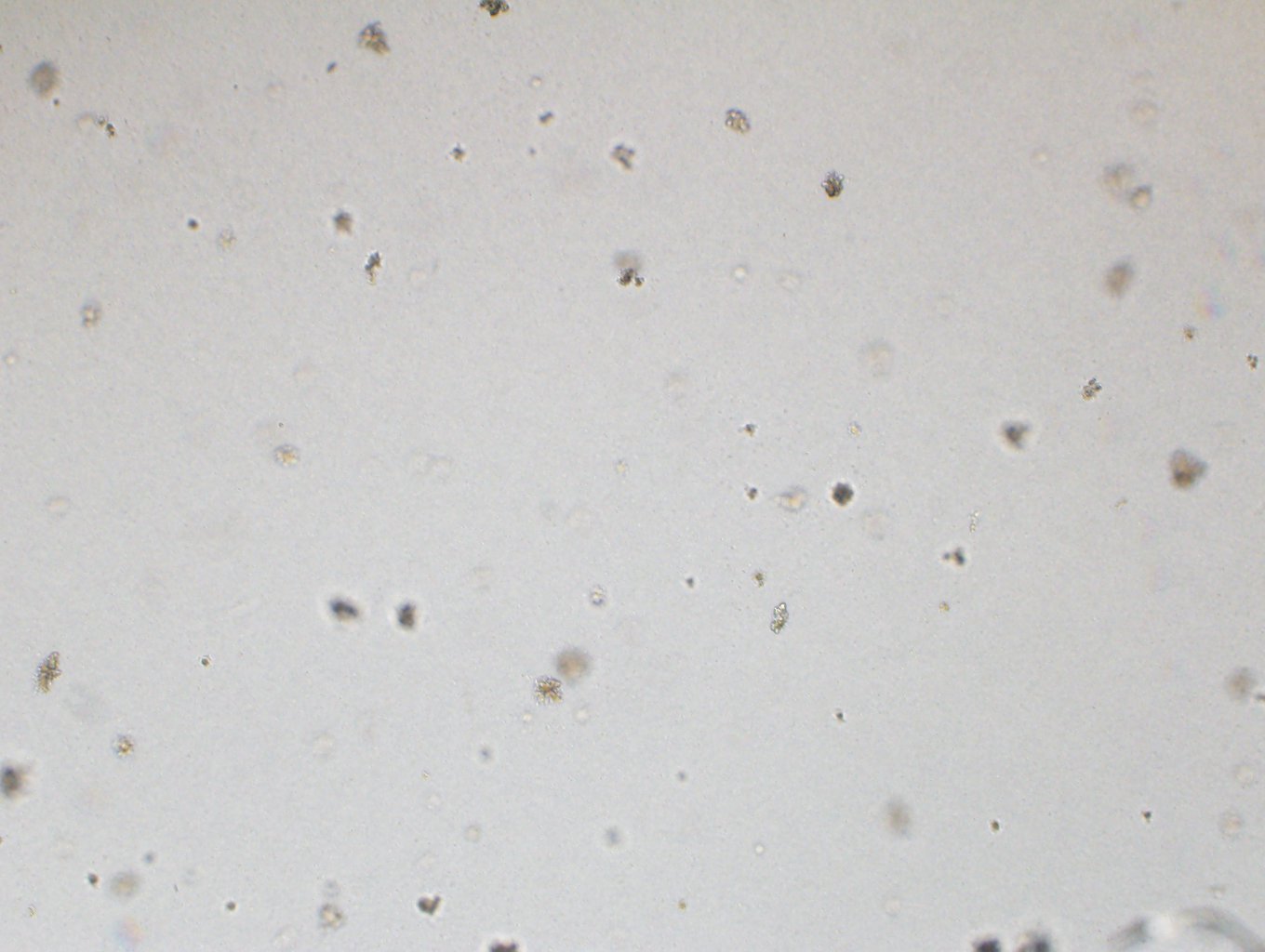

Supplement: Supplementary file 6 — Source data Fig. 5 [file 44318_2025_363_MOESM6_ESM.zip › Figure 5/5D/Ephrin A1+Defactinib (1).jpg]

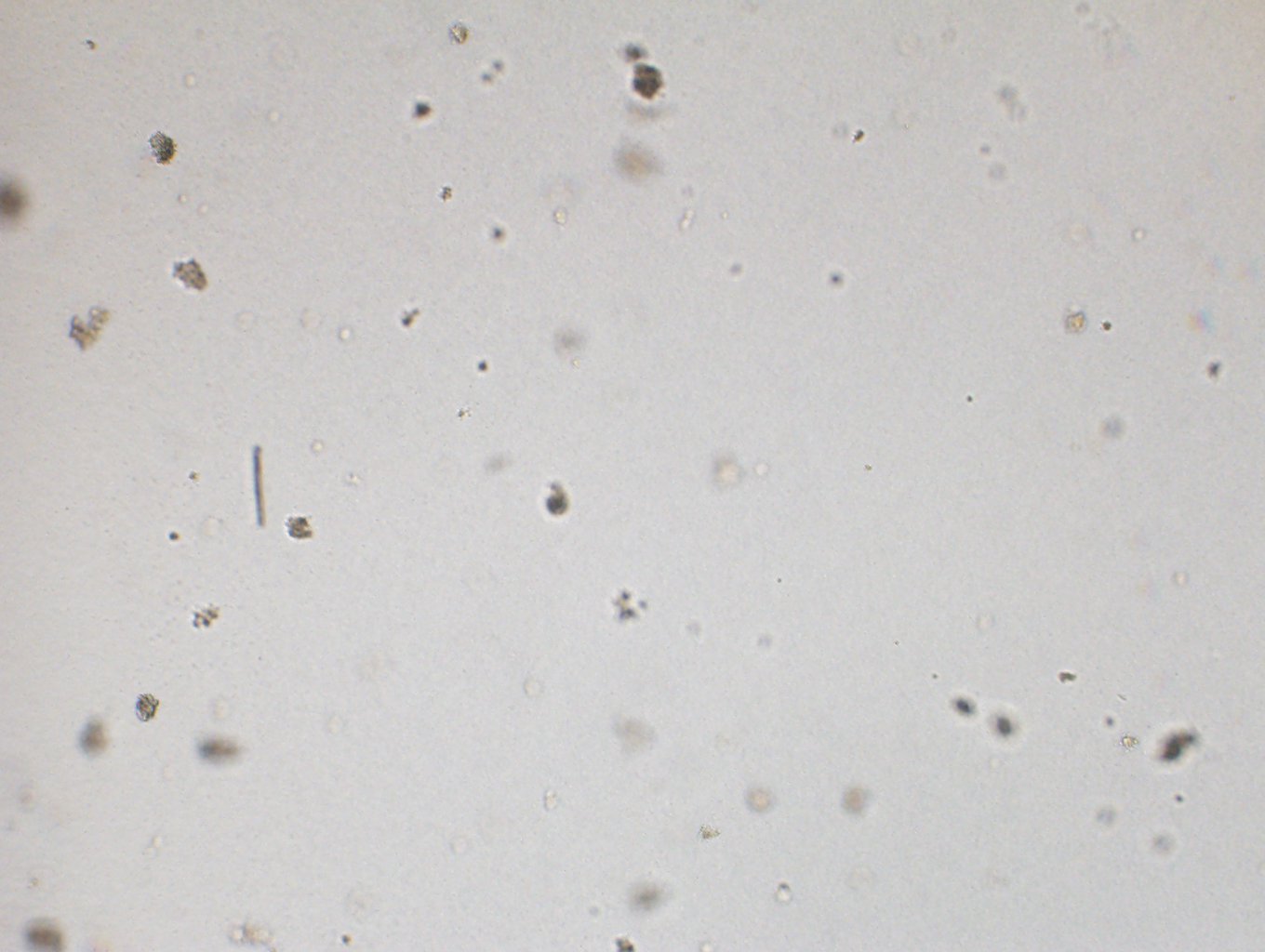

Supplement: Supplementary file 6 — Source data Fig. 5 [file 44318_2025_363_MOESM6_ESM.zip › Figure 5/5D/Ephrin A1+Defactinib (2)-displayed in 5D.jpg]

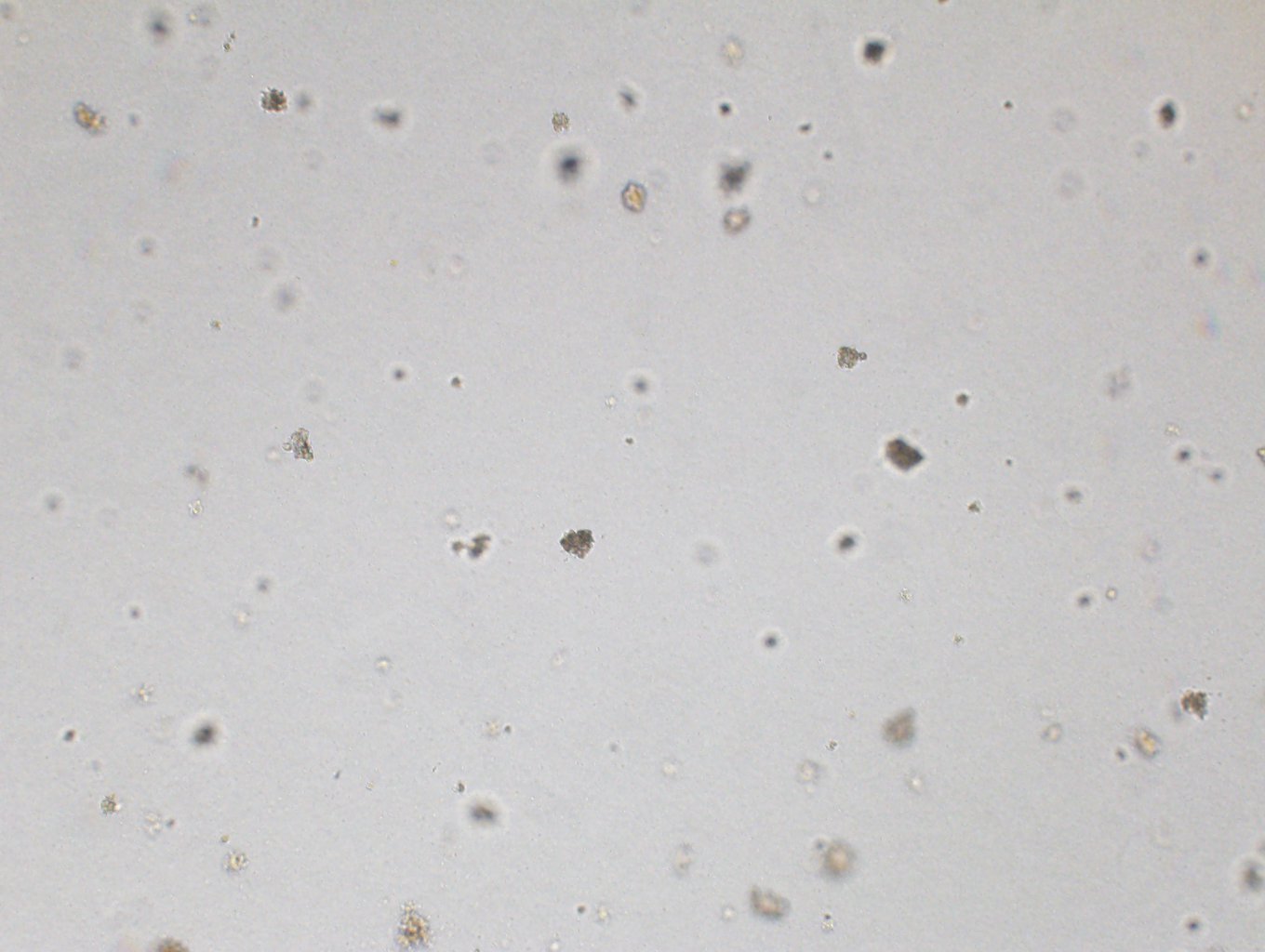

Supplement: Supplementary file 6 — Source data Fig. 5 [file 44318_2025_363_MOESM6_ESM.zip › Figure 5/5D/Ephrin A1+Defactinib (3).jpg]

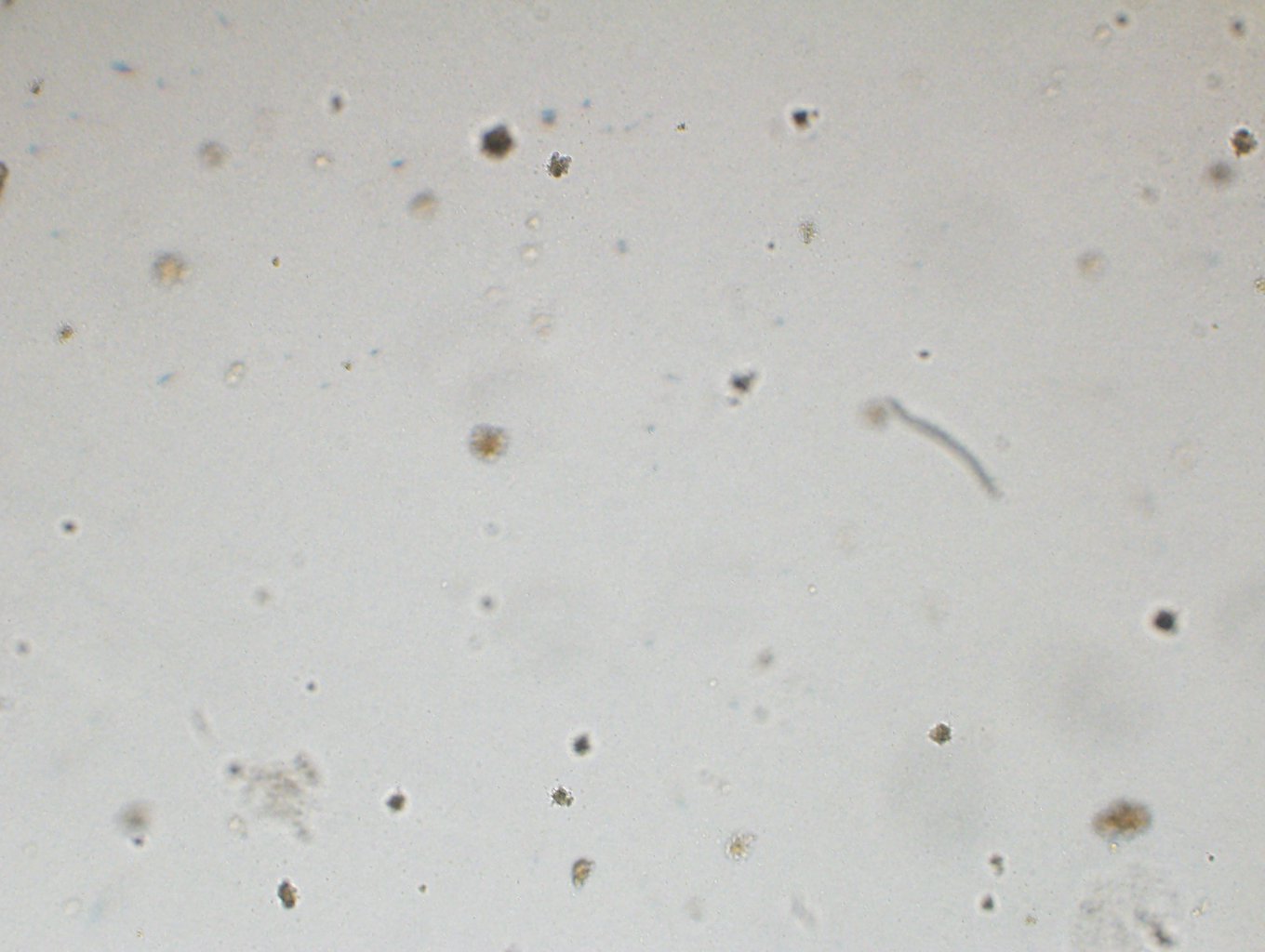

Supplement: Supplementary file 6 — Source data Fig. 5 [file 44318_2025_363_MOESM6_ESM.zip › Figure 5/5D/Ephrin A1+Defactinib (4).jpg]

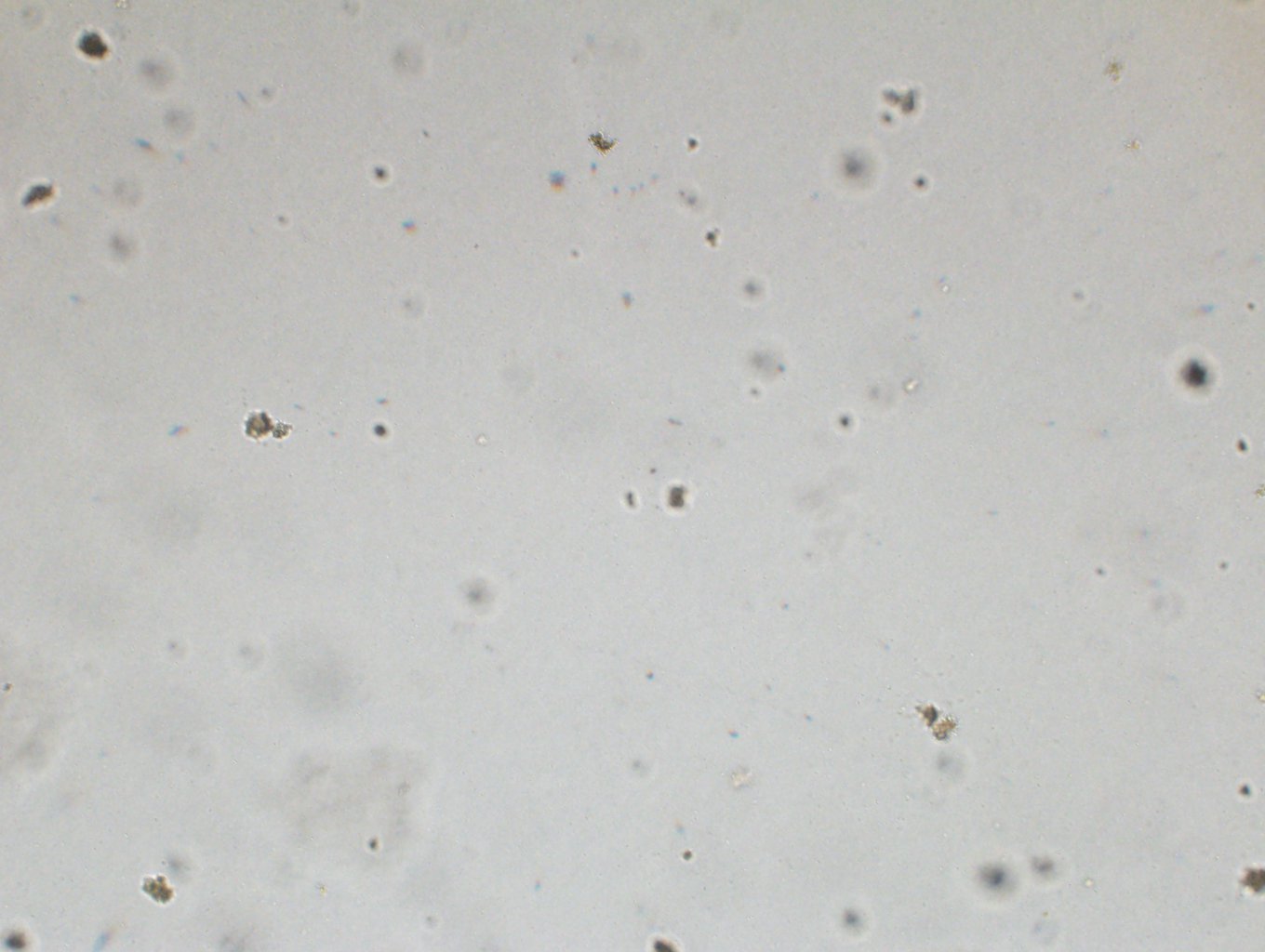

Supplement: Supplementary file 6 — Source data Fig. 5 [file 44318_2025_363_MOESM6_ESM.zip › Figure 5/5D/Ephrin A1+Defactinib (5).jpg]

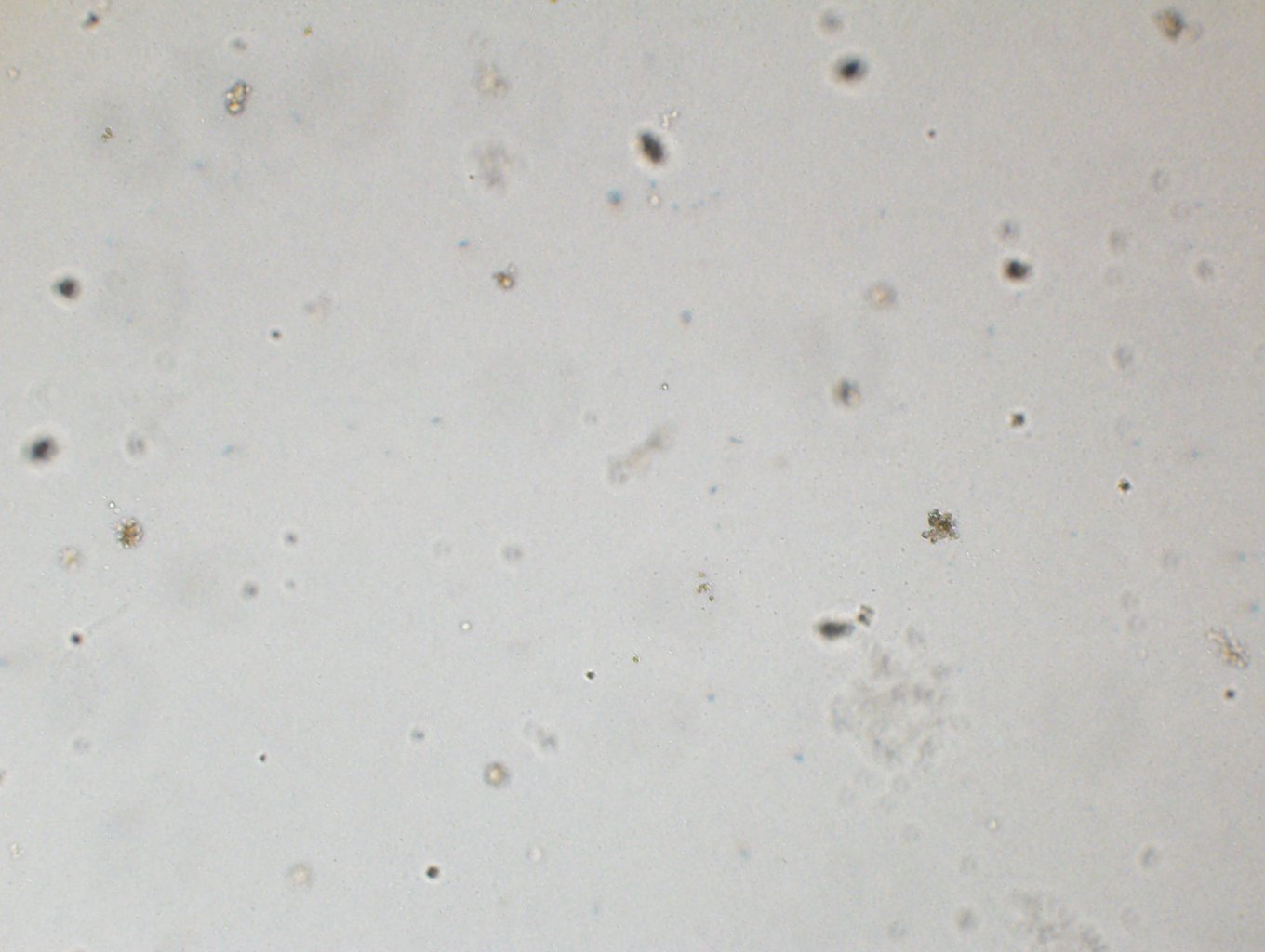

Supplement: Supplementary file 6 — Source data Fig. 5 [file 44318_2025_363_MOESM6_ESM.zip › Figure 5/5D/Ephrin A1+Defactinib (6).jpg]

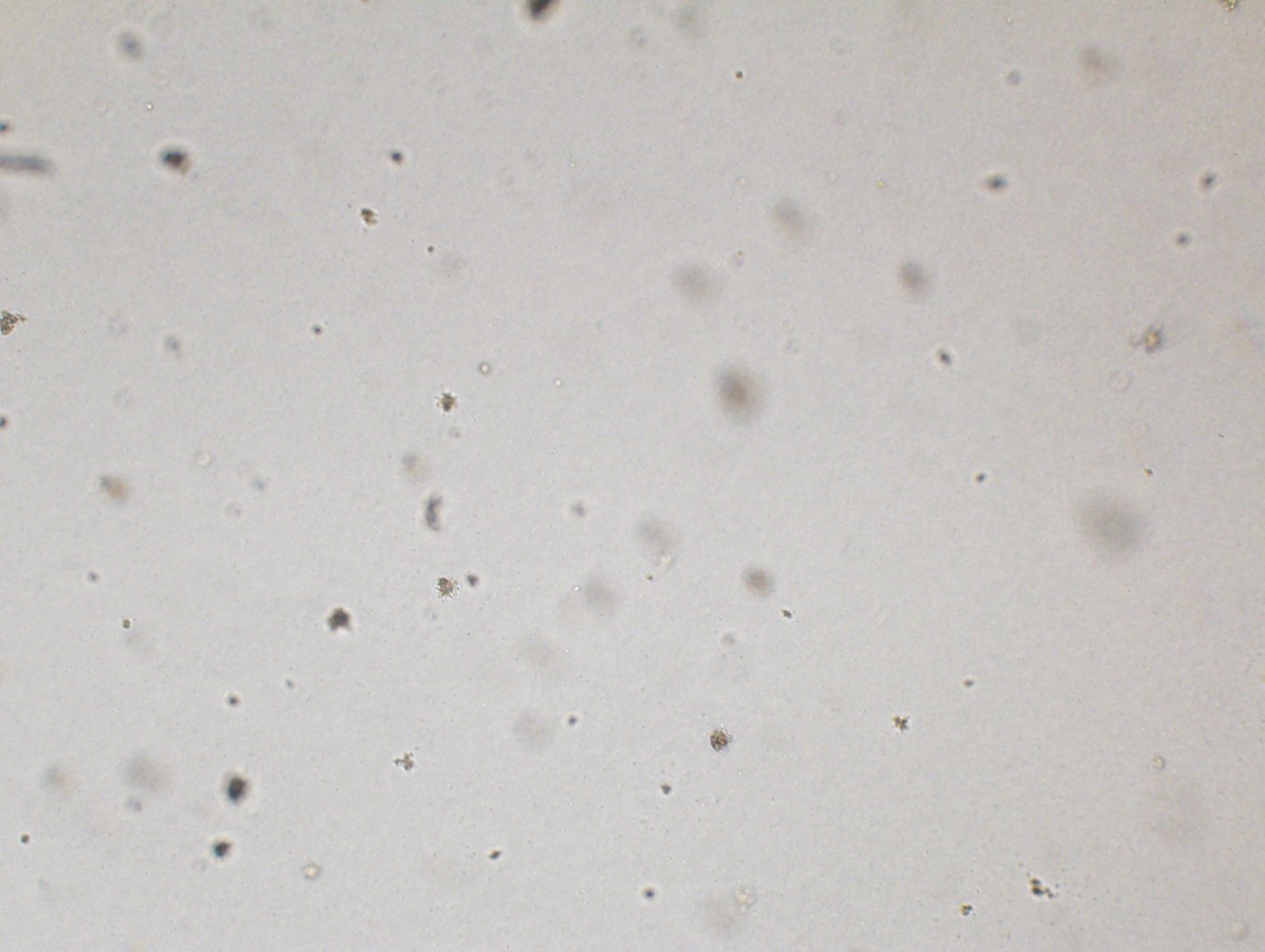

Supplement: Supplementary file 6 — Source data Fig. 5 [file 44318_2025_363_MOESM6_ESM.zip › Figure 5/5D/Ephrin A1+Defactinib (7).jpg]

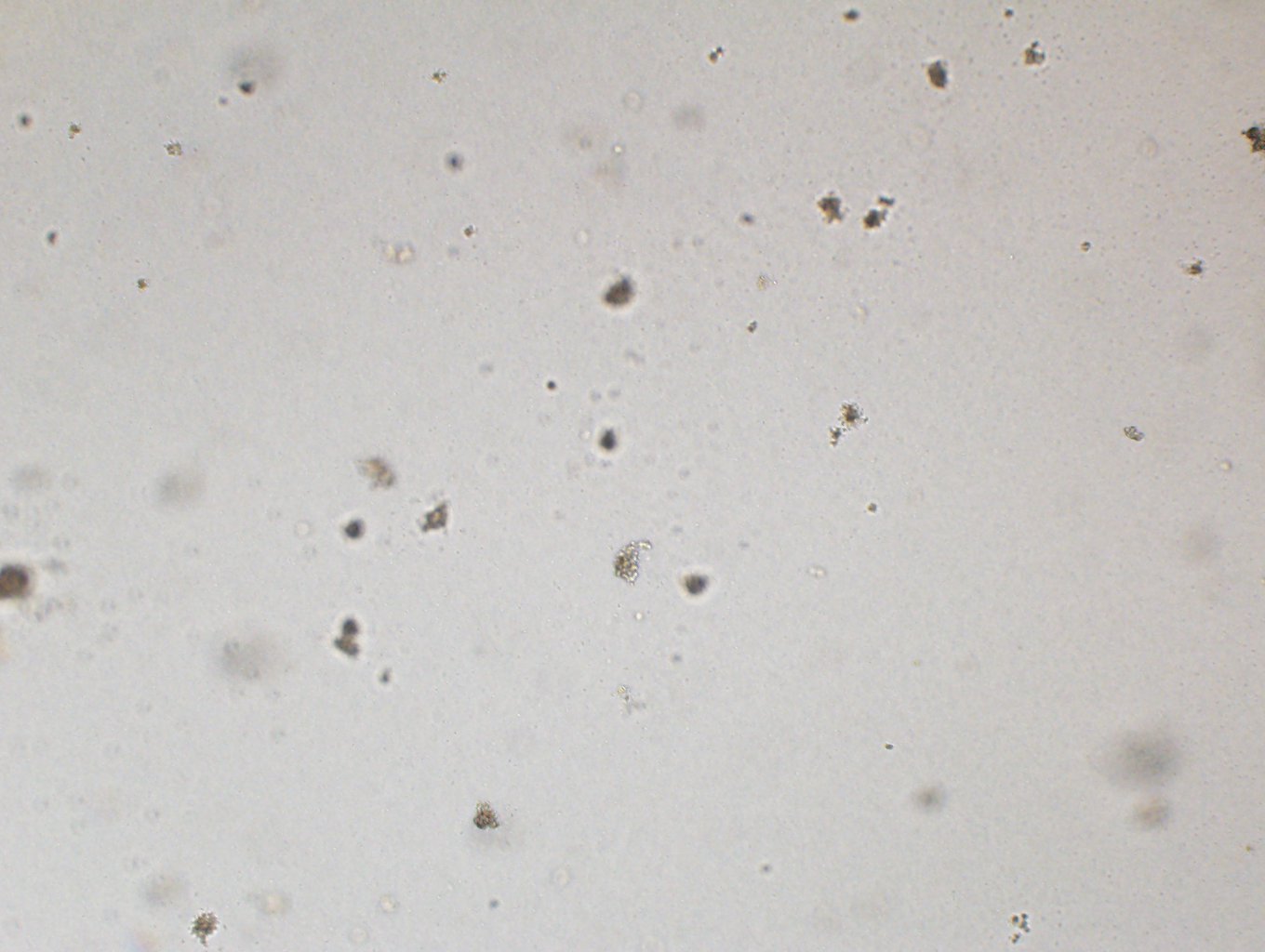

Supplement: Supplementary file 6 — Source data Fig. 5 [file 44318_2025_363_MOESM6_ESM.zip › Figure 5/5D/Ephrin A1+Defactinib (8).jpg]

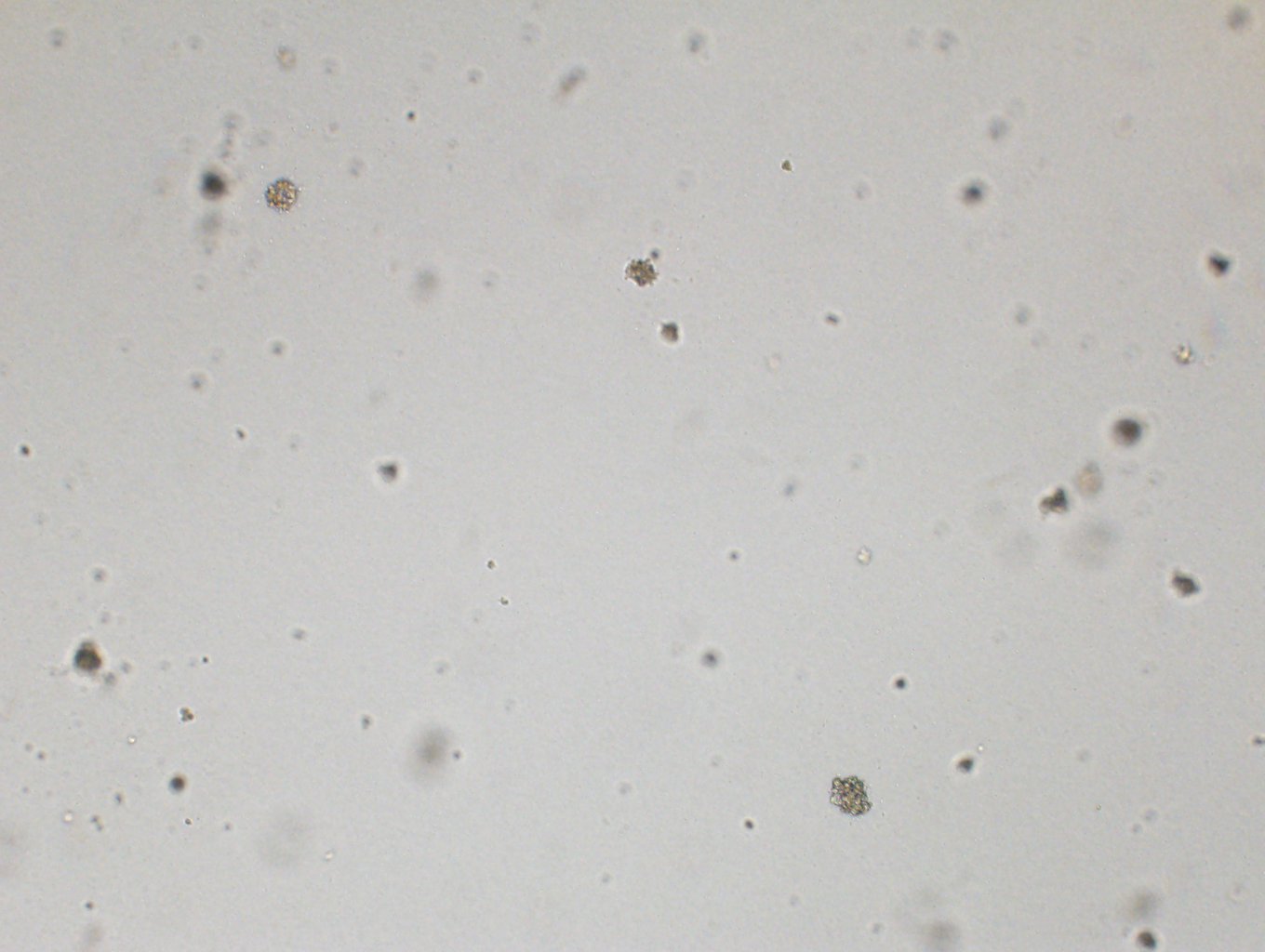

Supplement: Supplementary file 6 — Source data Fig. 5 [file 44318_2025_363_MOESM6_ESM.zip › Figure 5/5D/Ephrin A1+U0126 (1)-displayed in 5D.jpg]

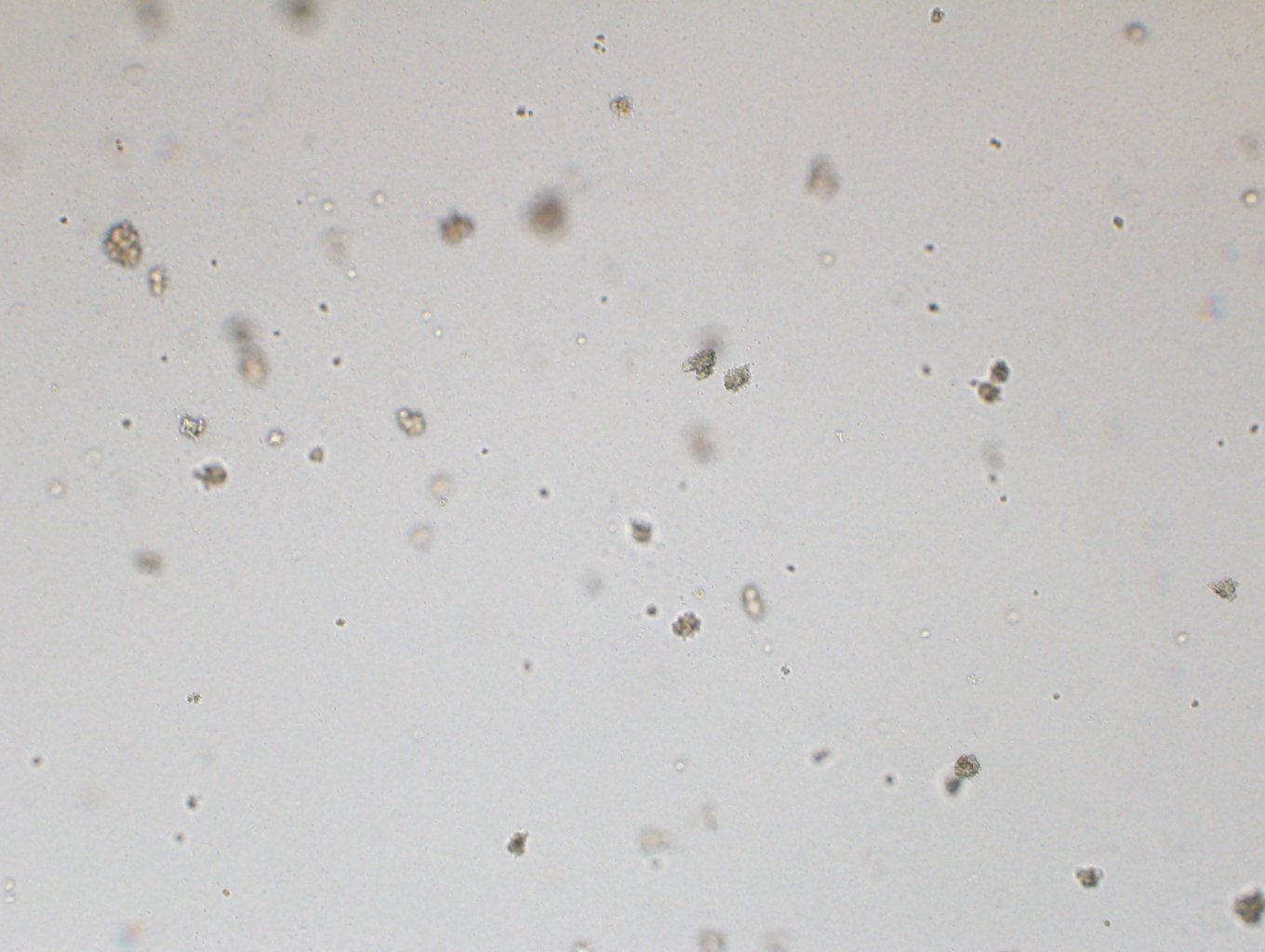

Supplement: Supplementary file 6 — Source data Fig. 5 [file 44318_2025_363_MOESM6_ESM.zip › Figure 5/5D/Ephrin A1+U0126 (2).jpg]

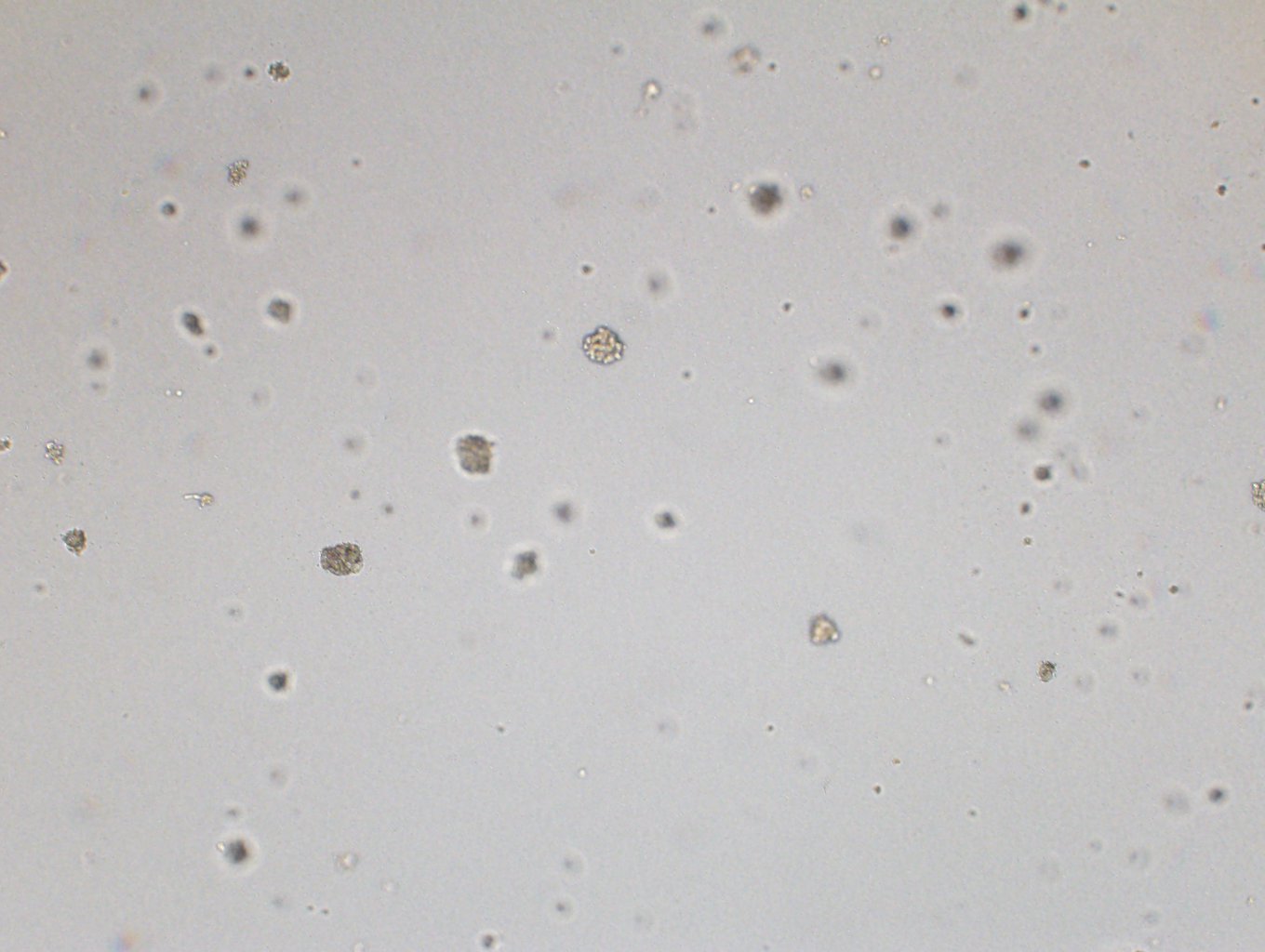

Supplement: Supplementary file 6 — Source data Fig. 5 [file 44318_2025_363_MOESM6_ESM.zip › Figure 5/5D/Ephrin A1+U0126 (3).jpg]

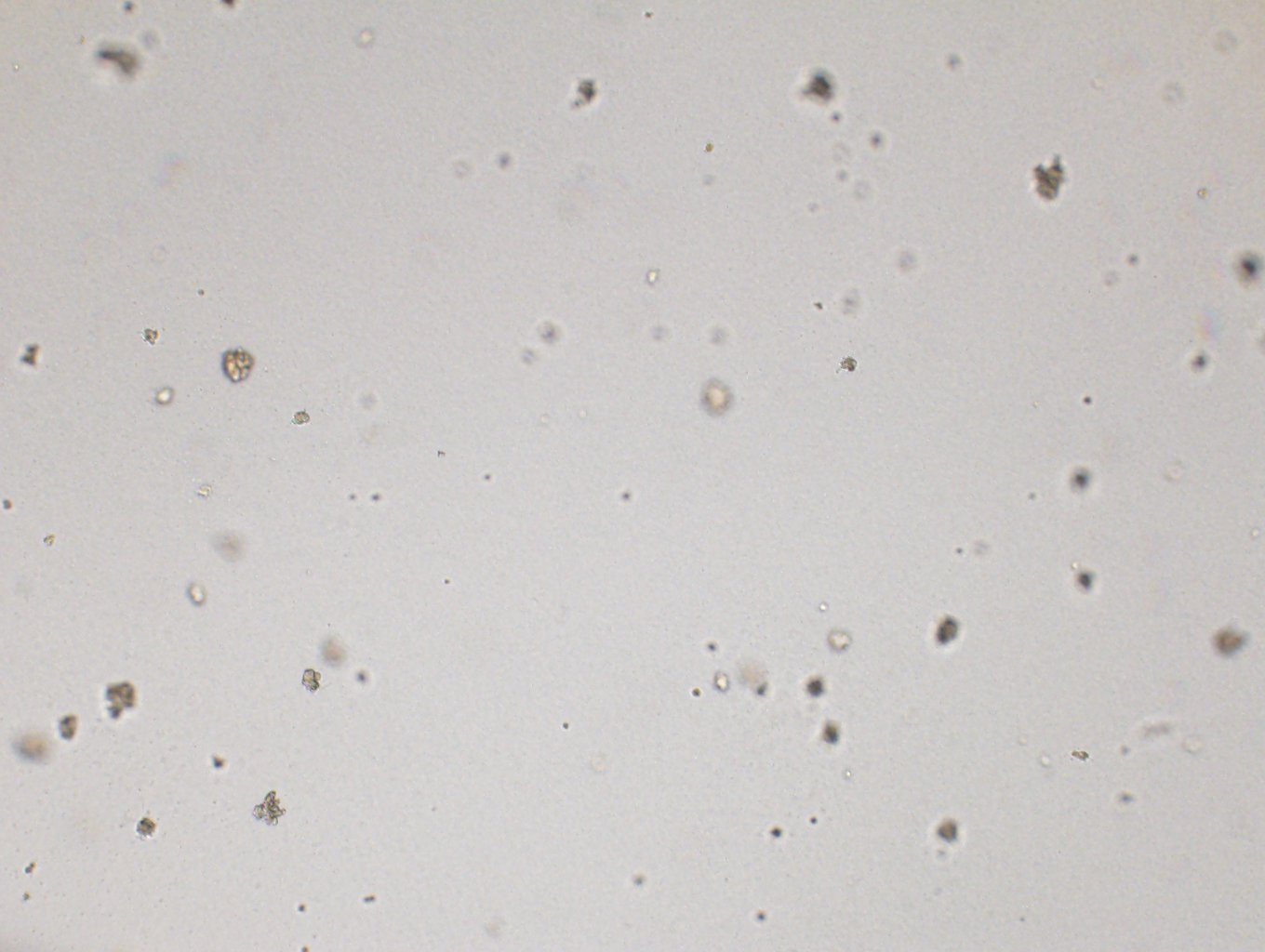

Supplement: Supplementary file 6 — Source data Fig. 5 [file 44318_2025_363_MOESM6_ESM.zip › Figure 5/5D/Ephrin A1+U0126 (4).jpg]

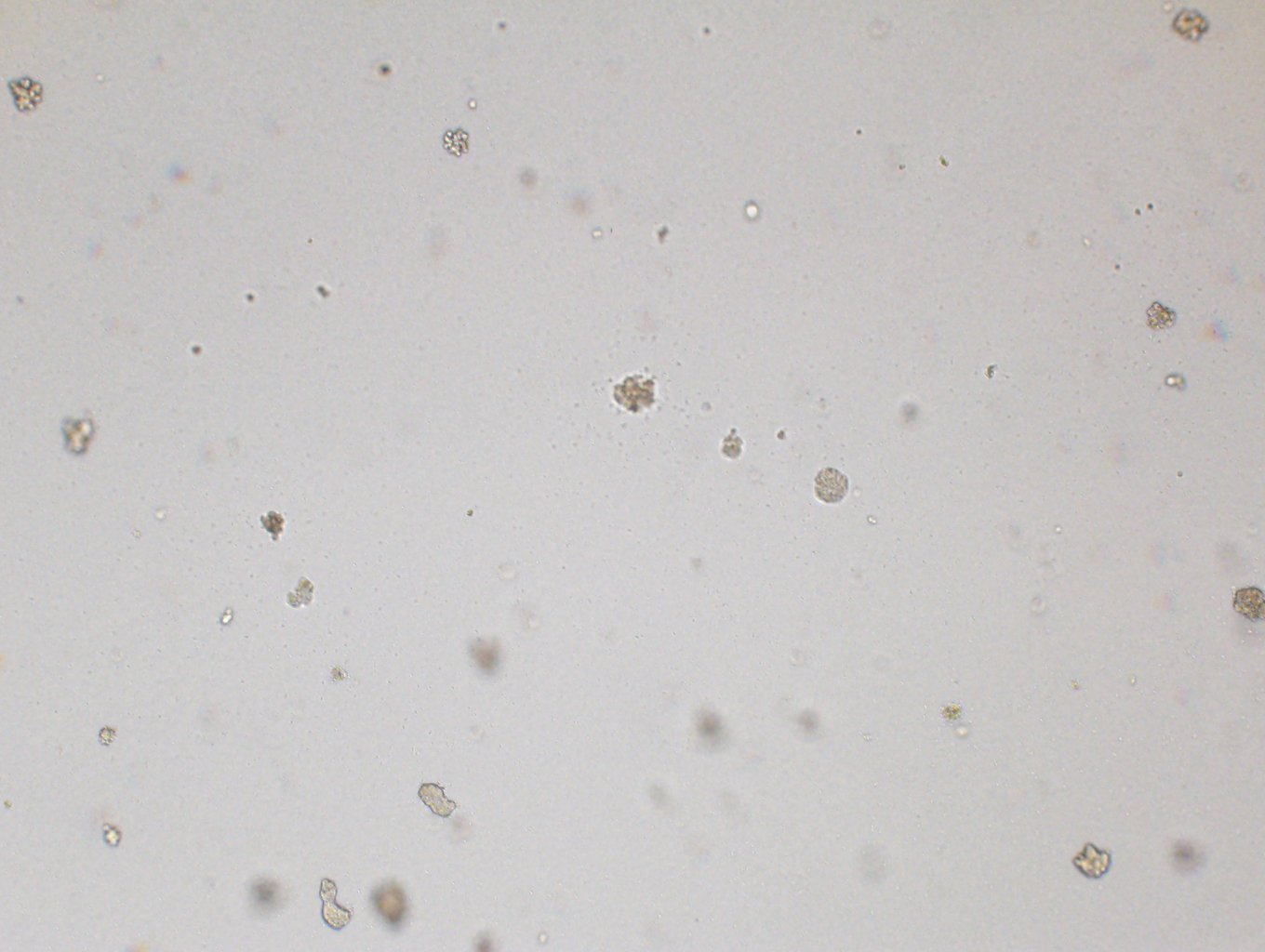

Supplement: Supplementary file 6 — Source data Fig. 5 [file 44318_2025_363_MOESM6_ESM.zip › Figure 5/5D/Ephrin A1+U0126 (5).jpg]

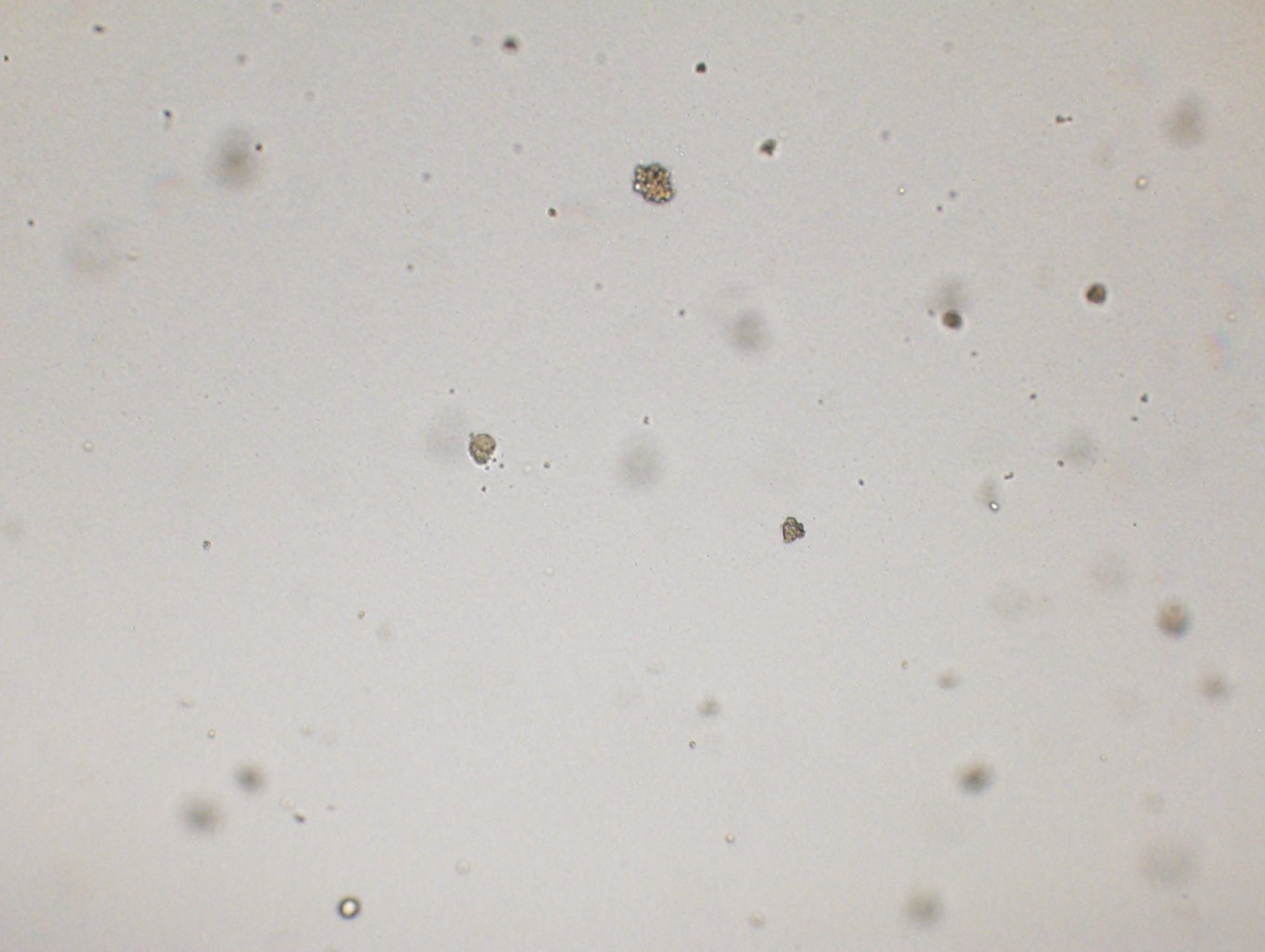

Supplement: Supplementary file 6 — Source data Fig. 5 [file 44318_2025_363_MOESM6_ESM.zip › Figure 5/5D/Ephrin A1+U0126 (6).jpg]

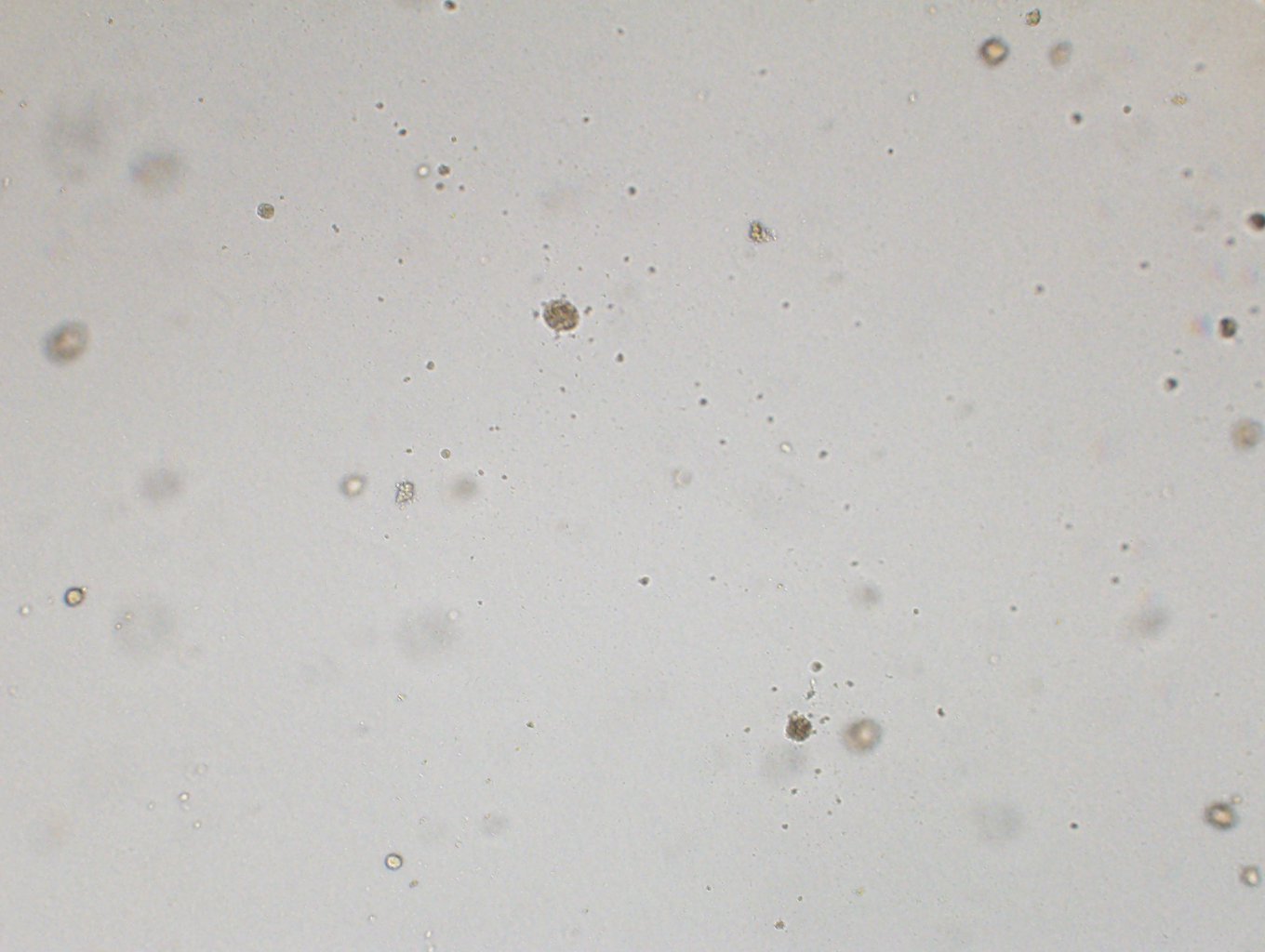

Supplement: Supplementary file 6 — Source data Fig. 5 [file 44318_2025_363_MOESM6_ESM.zip › Figure 5/5D/Ephrin A1+U0126 (7).jpg]

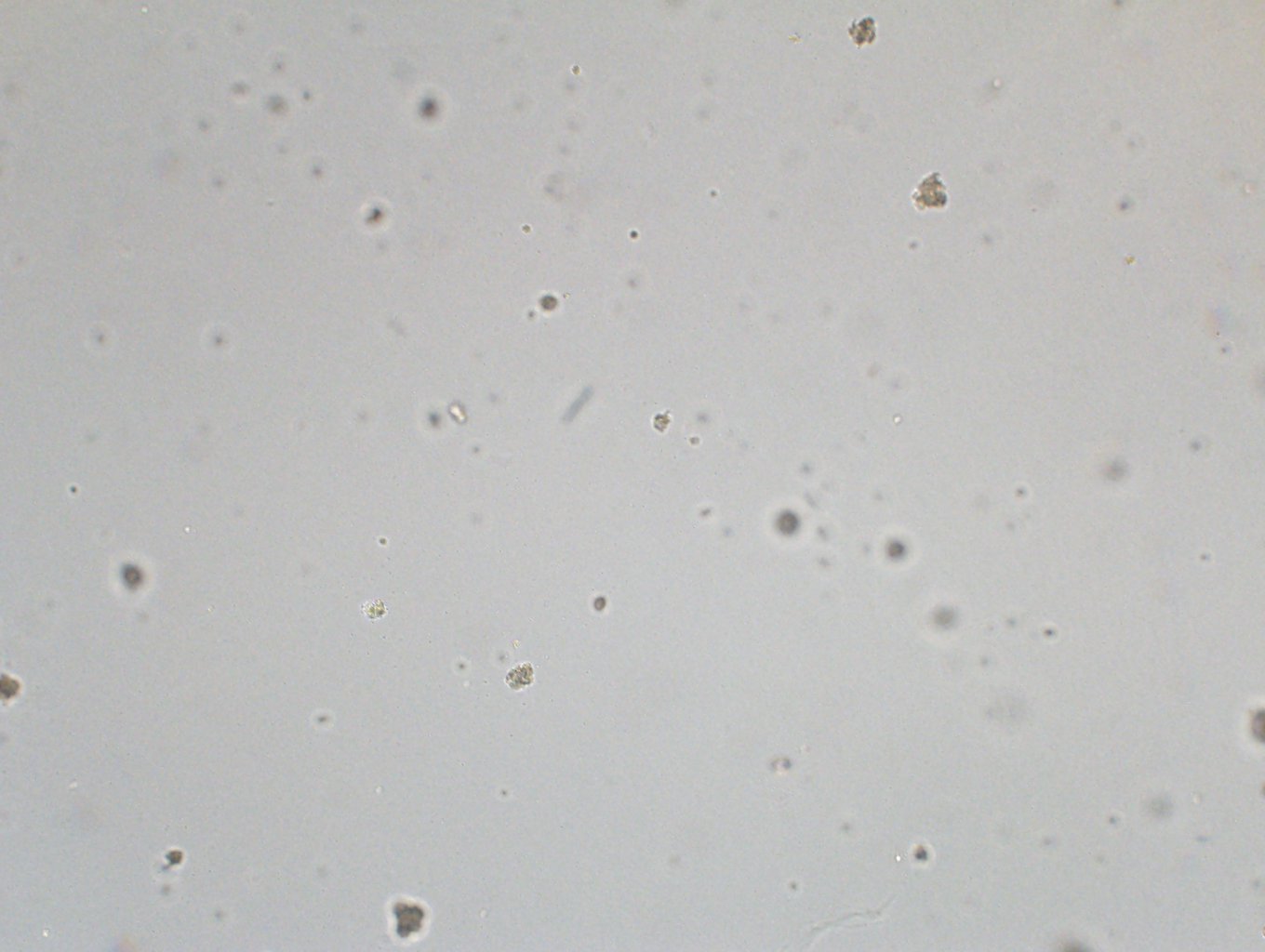

Supplement: Supplementary file 6 — Source data Fig. 5 [file 44318_2025_363_MOESM6_ESM.zip › Figure 5/5D/Ephrin A1+U0126 (8).jpg]

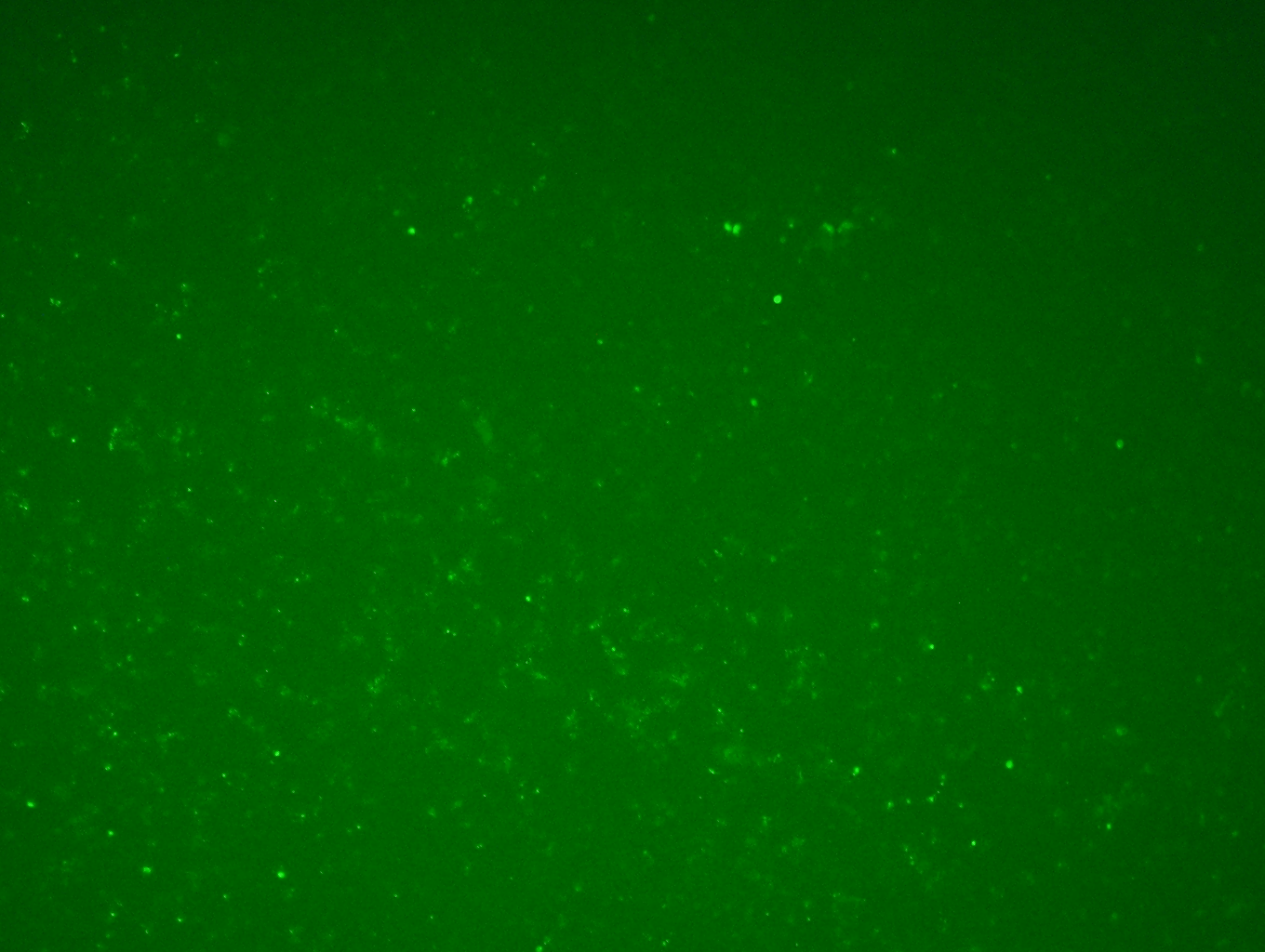

Supplement: Supplementary file 6 — Source data Fig. 5 [file 44318_2025_363_MOESM6_ESM.zip › Figure 5/5F/Control (1).tif]

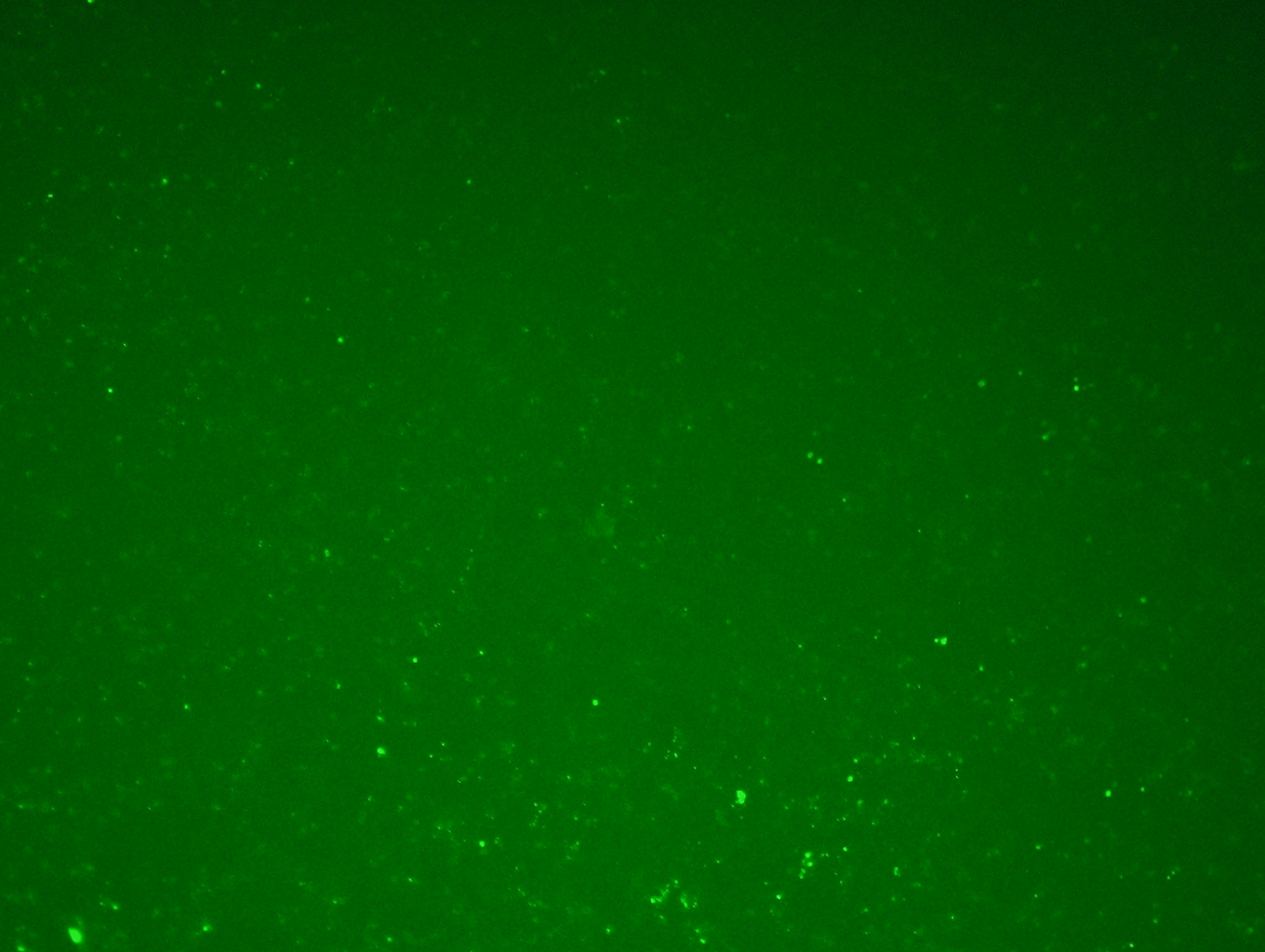

Supplement: Supplementary file 6 — Source data Fig. 5 [file 44318_2025_363_MOESM6_ESM.zip › Figure 5/5F/Control (2).tif]
