# Supplementary figures and images for: Ephrin A1 functions as a ligand of EGFR to promote EMT and metastasis in gastric cancer (part 4 of 5)
Source: EMBO J. 2025 Jan 21;44(5):1464–87. doi: 10.1038/s44318-025-00363-x (PMC11876641; doi:10.1038/s44318-025-00363-x)

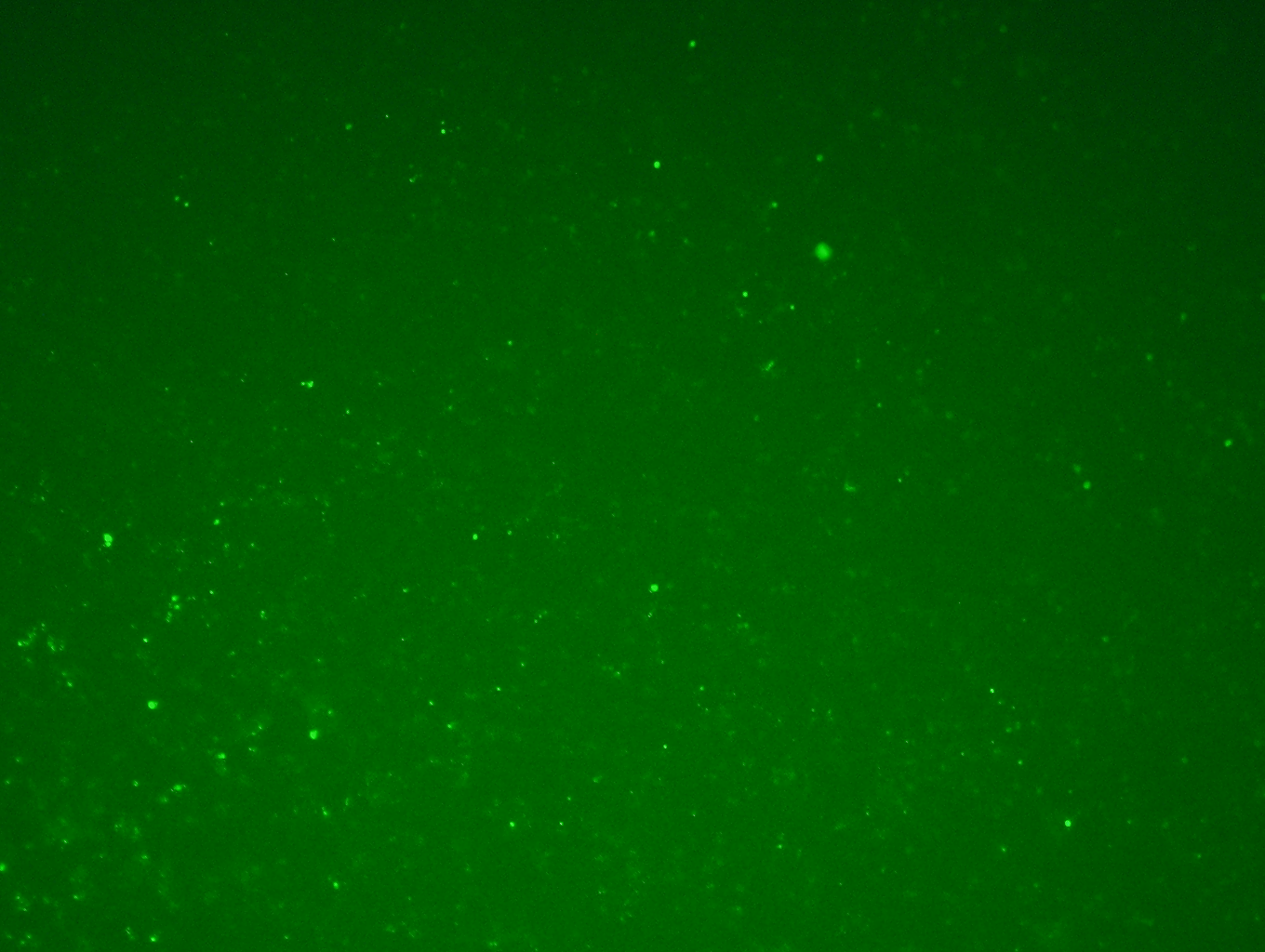

Supplement: Supplementary file 6 — Source data Fig. 5 [file 44318_2025_363_MOESM6_ESM.zip › Figure 5/5F/Control (3)-displayed in 3F.tif]

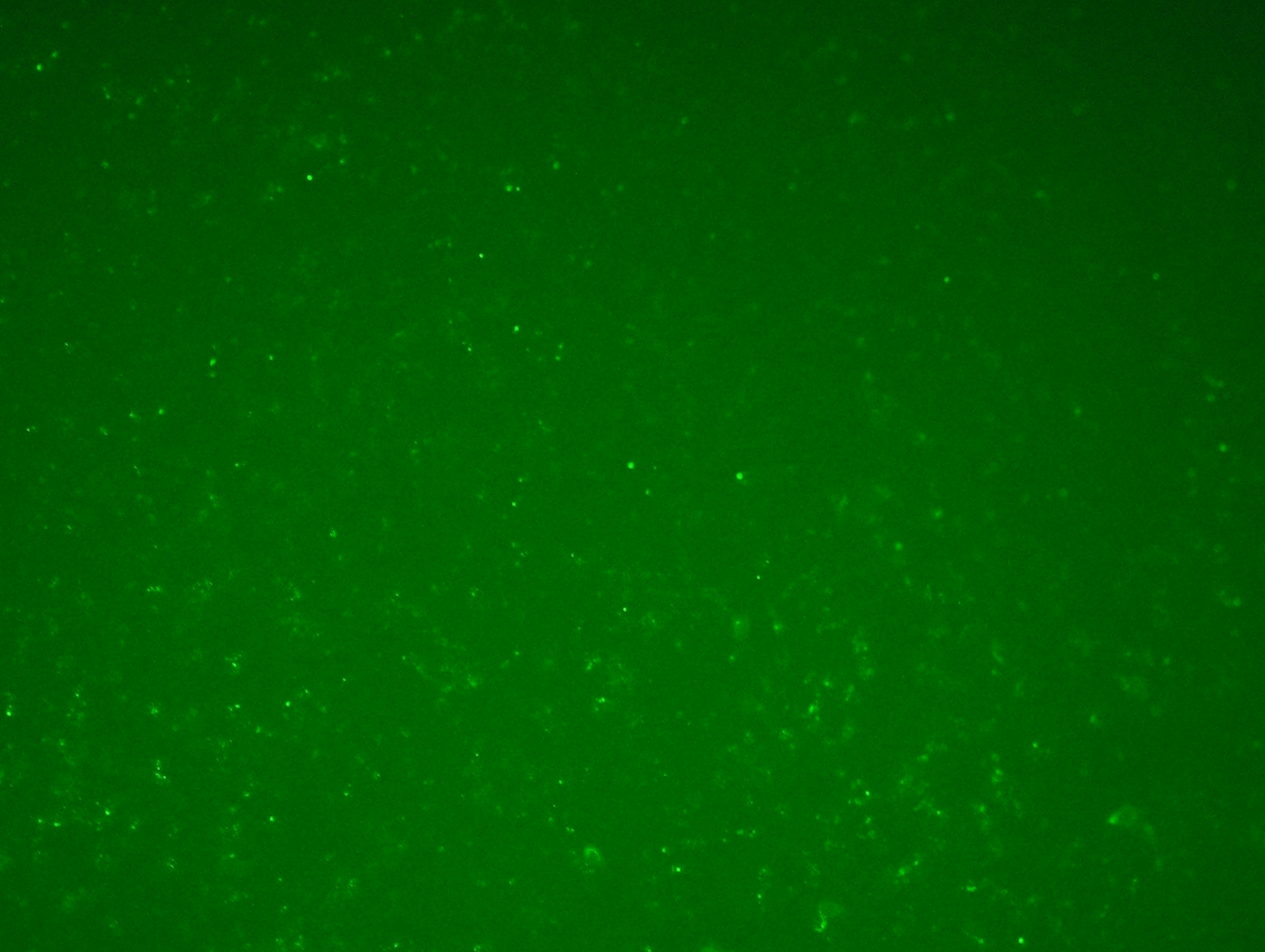

Supplement: Supplementary file 6 — Source data Fig. 5 [file 44318_2025_363_MOESM6_ESM.zip › Figure 5/5F/Control (4).tif]

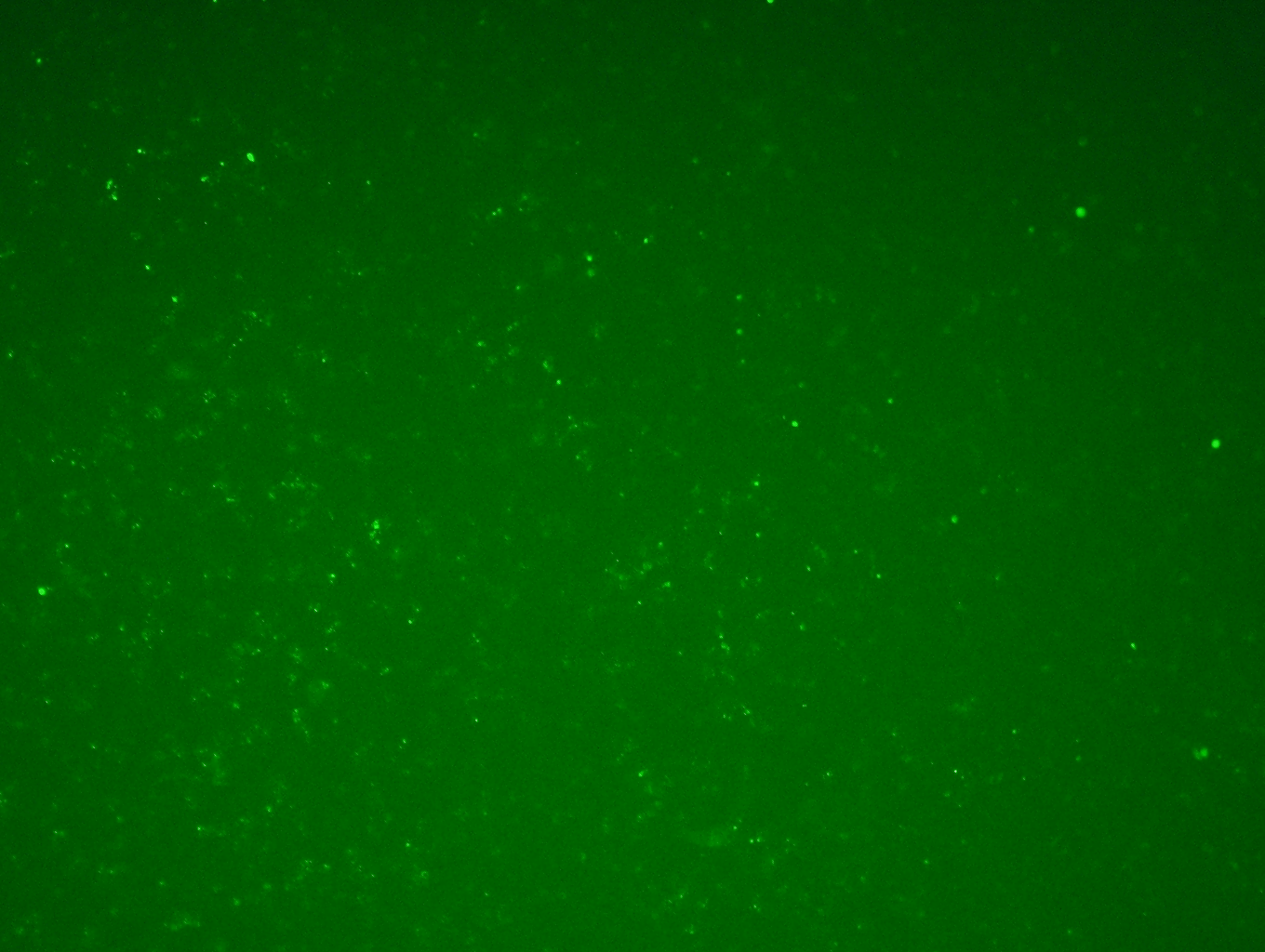

Supplement: Supplementary file 6 — Source data Fig. 5 [file 44318_2025_363_MOESM6_ESM.zip › Figure 5/5F/Control (5).tif]

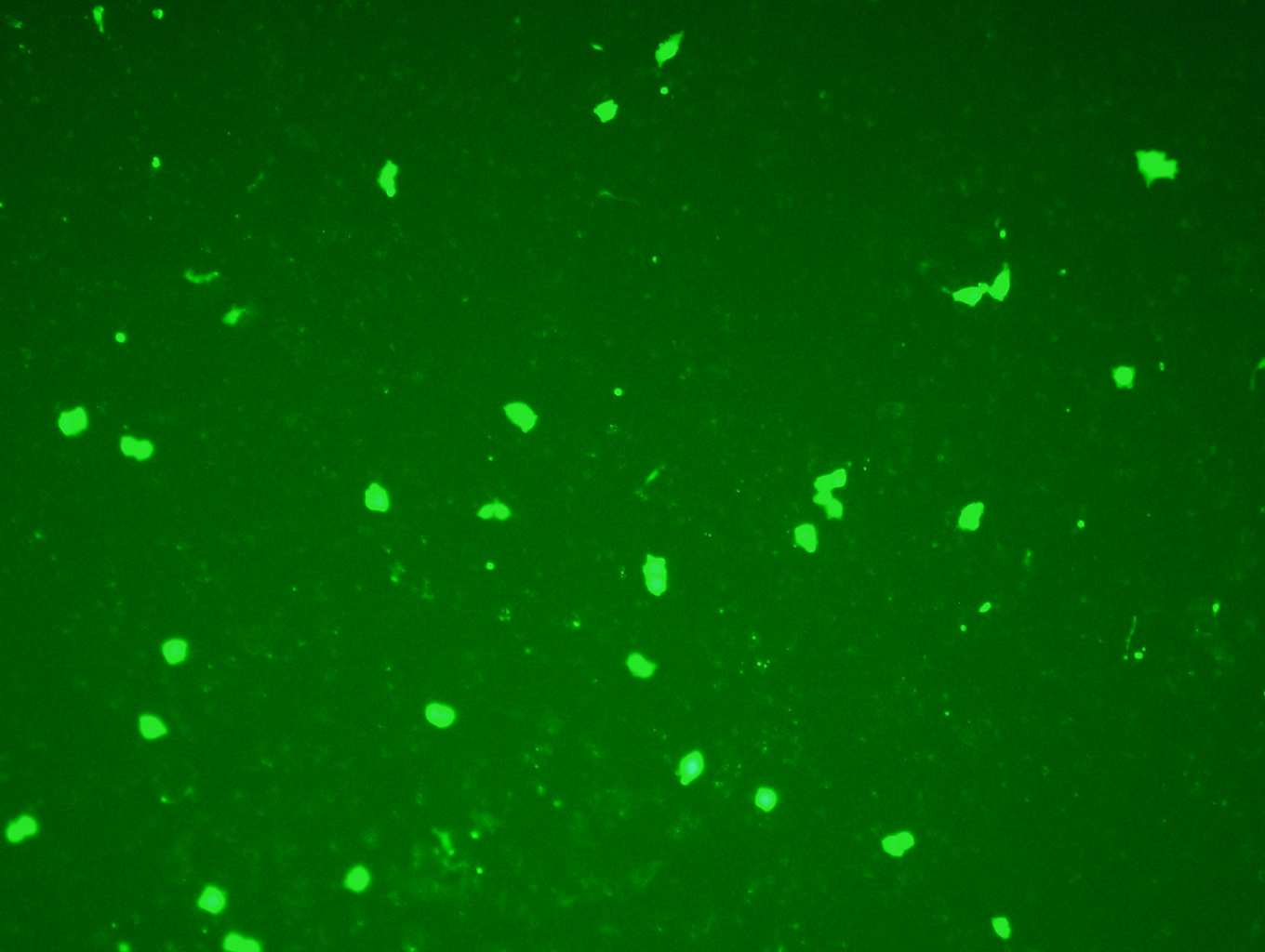

Supplement: Supplementary file 6 — Source data Fig. 5 [file 44318_2025_363_MOESM6_ESM.zip › Figure 5/5F/Ephrin A1 (1)-displayed in 3F.tif]

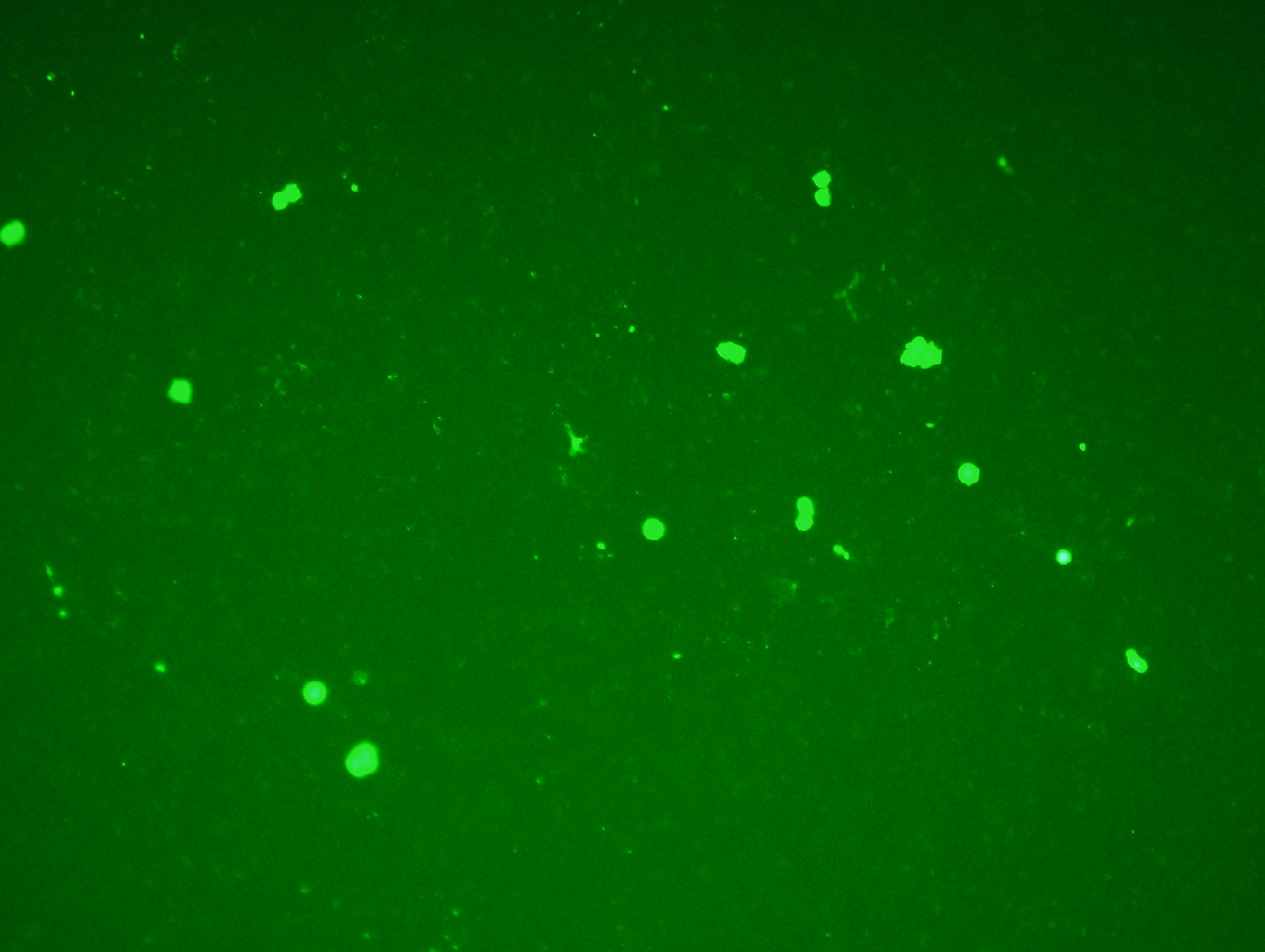

Supplement: Supplementary file 6 — Source data Fig. 5 [file 44318_2025_363_MOESM6_ESM.zip › Figure 5/5F/Ephrin A1 (2).tif]

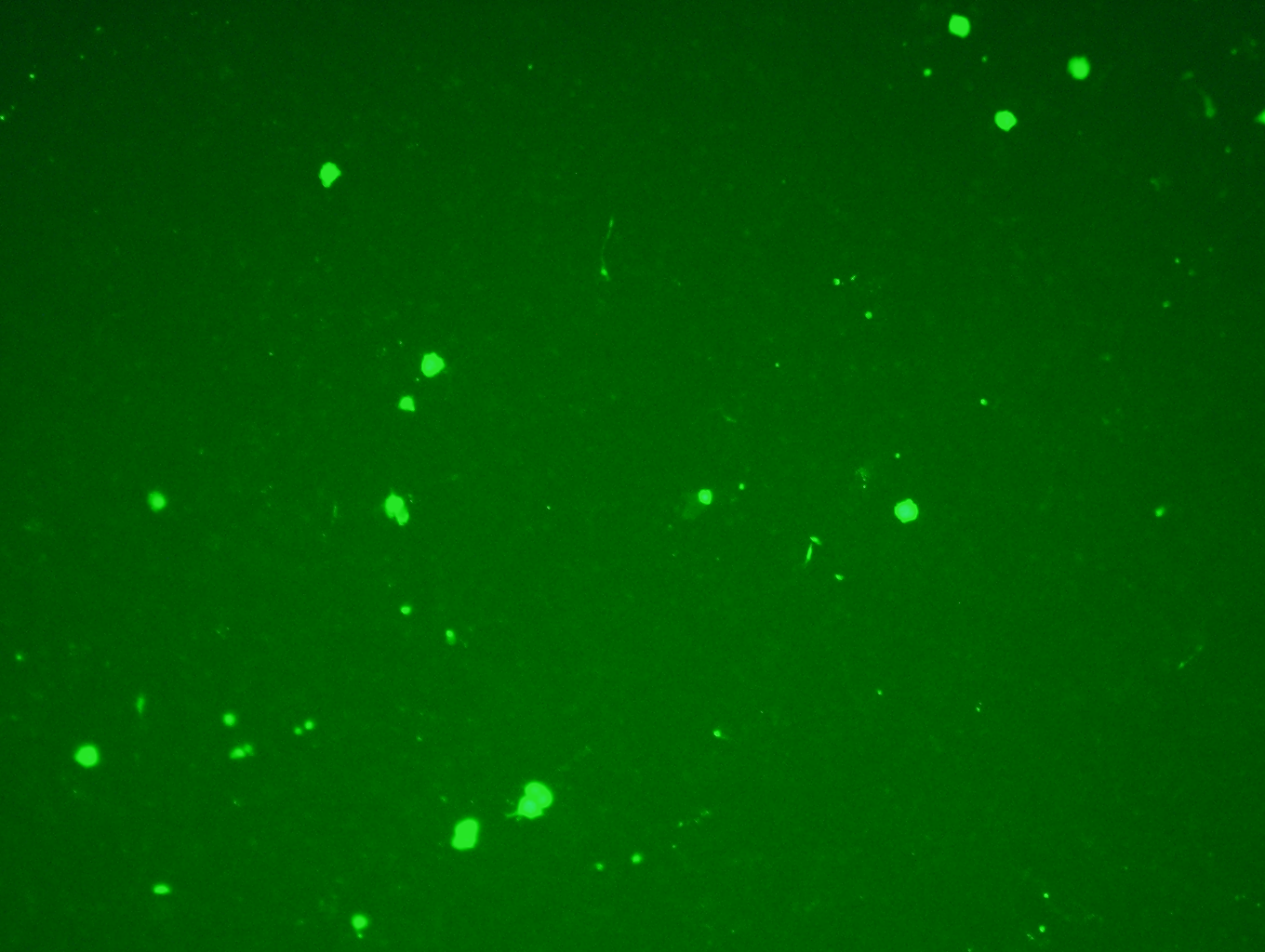

Supplement: Supplementary file 6 — Source data Fig. 5 [file 44318_2025_363_MOESM6_ESM.zip › Figure 5/5F/Ephrin A1 (3).tif]

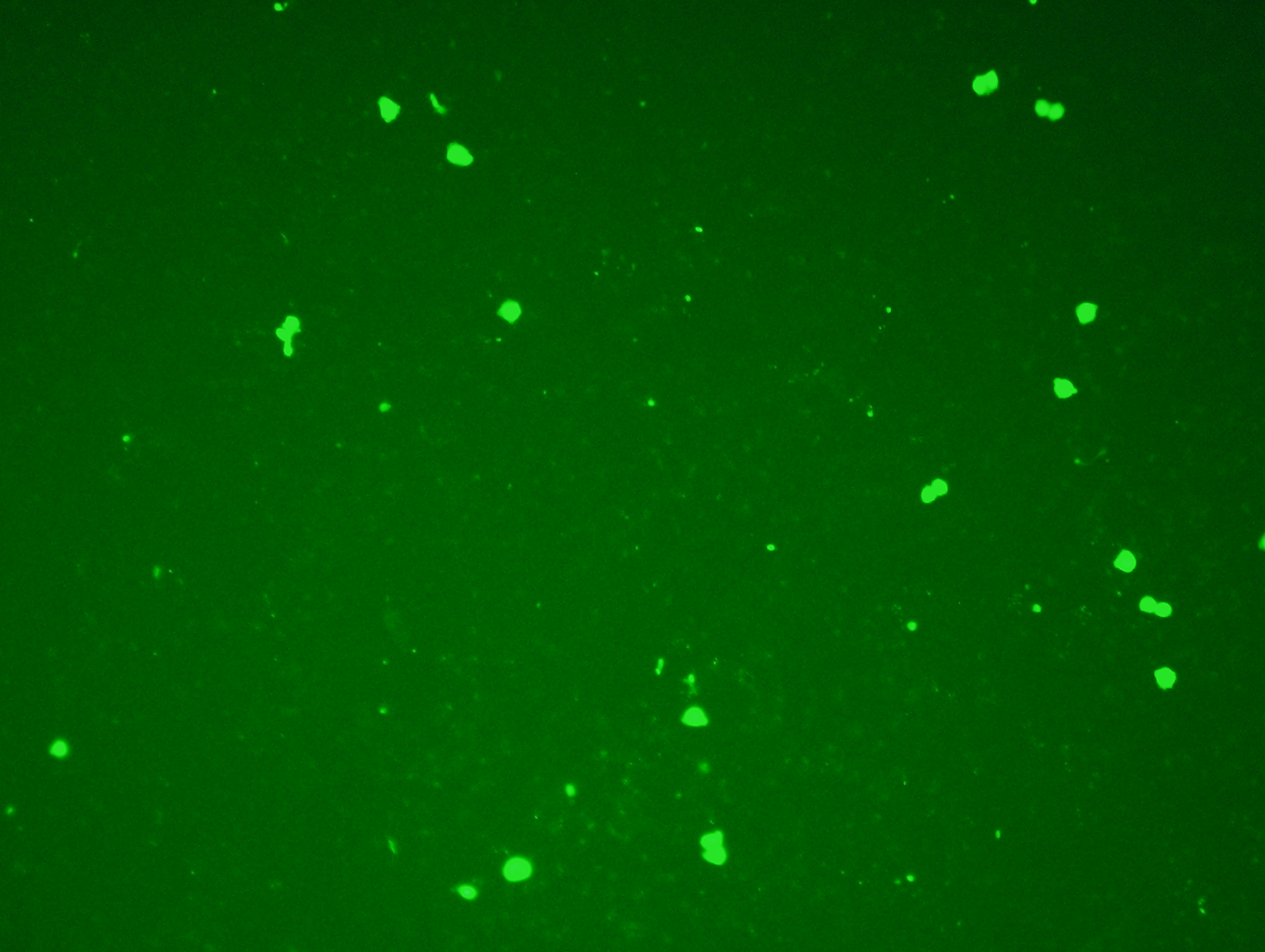

Supplement: Supplementary file 6 — Source data Fig. 5 [file 44318_2025_363_MOESM6_ESM.zip › Figure 5/5F/Ephrin A1 (4).tif]

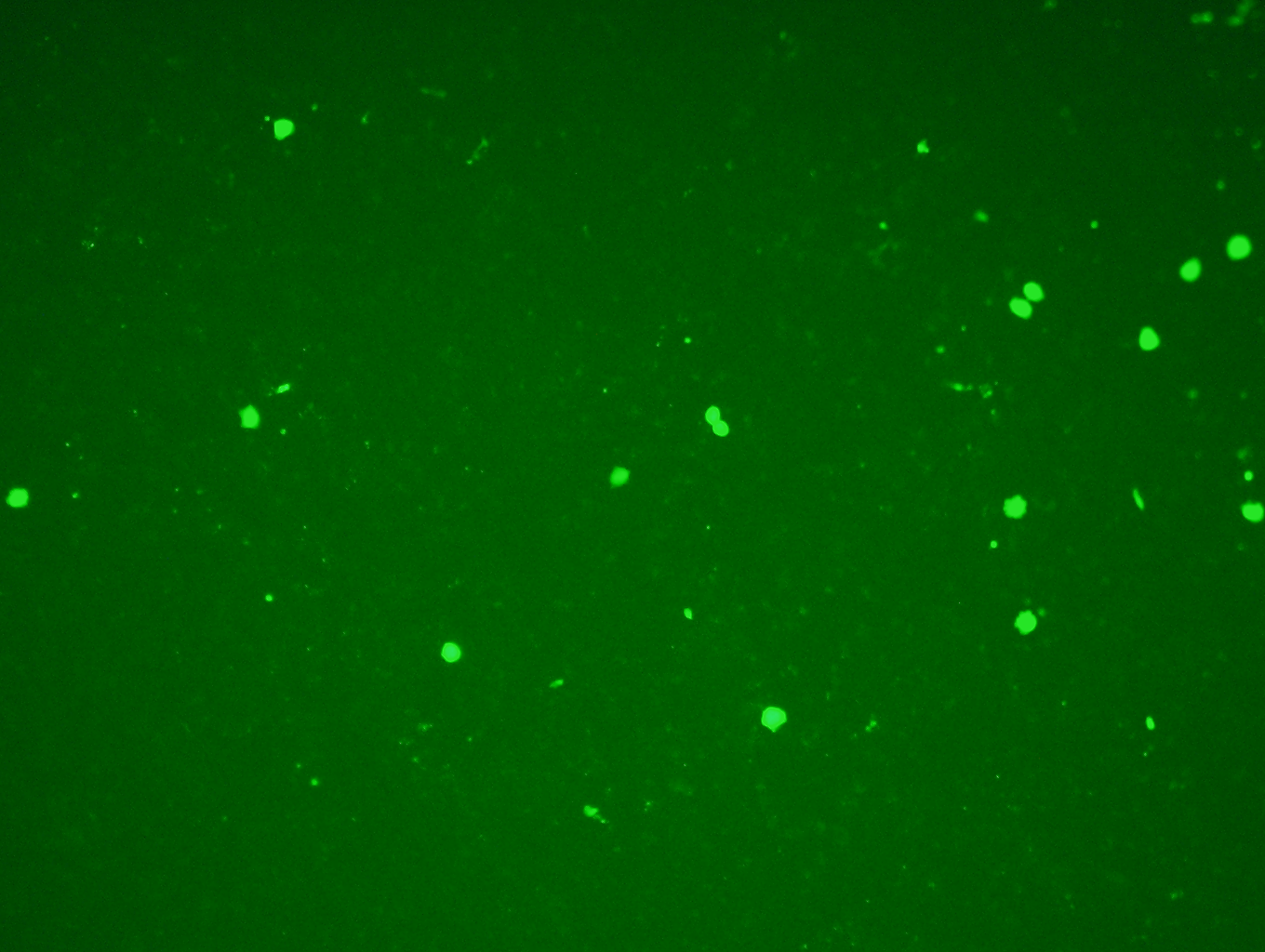

Supplement: Supplementary file 6 — Source data Fig. 5 [file 44318_2025_363_MOESM6_ESM.zip › Figure 5/5F/Ephrin A1 (5).tif]

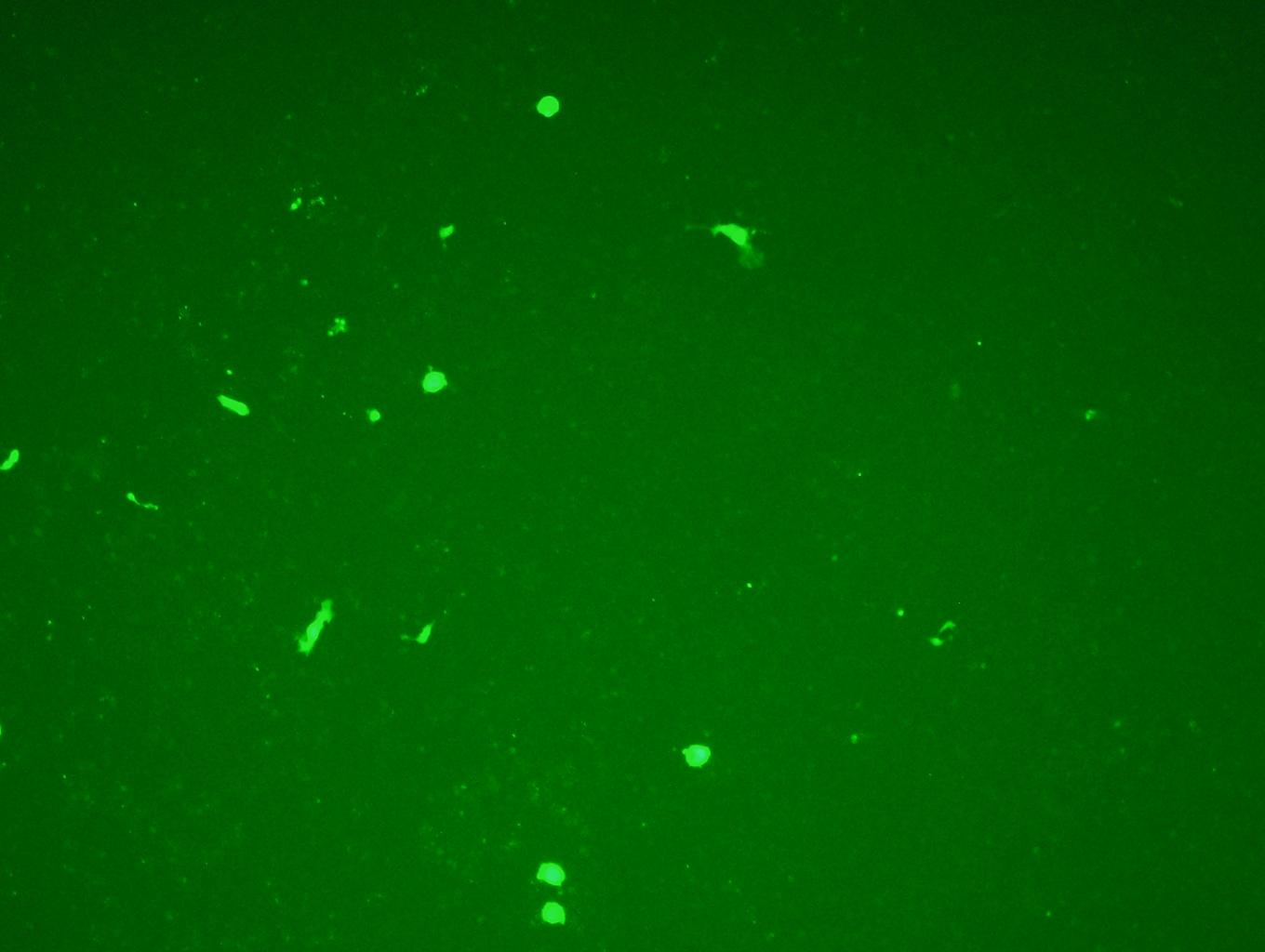

Supplement: Supplementary file 6 — Source data Fig. 5 [file 44318_2025_363_MOESM6_ESM.zip › Figure 5/5F/Ephrin A1+Defactinib(1).tif]

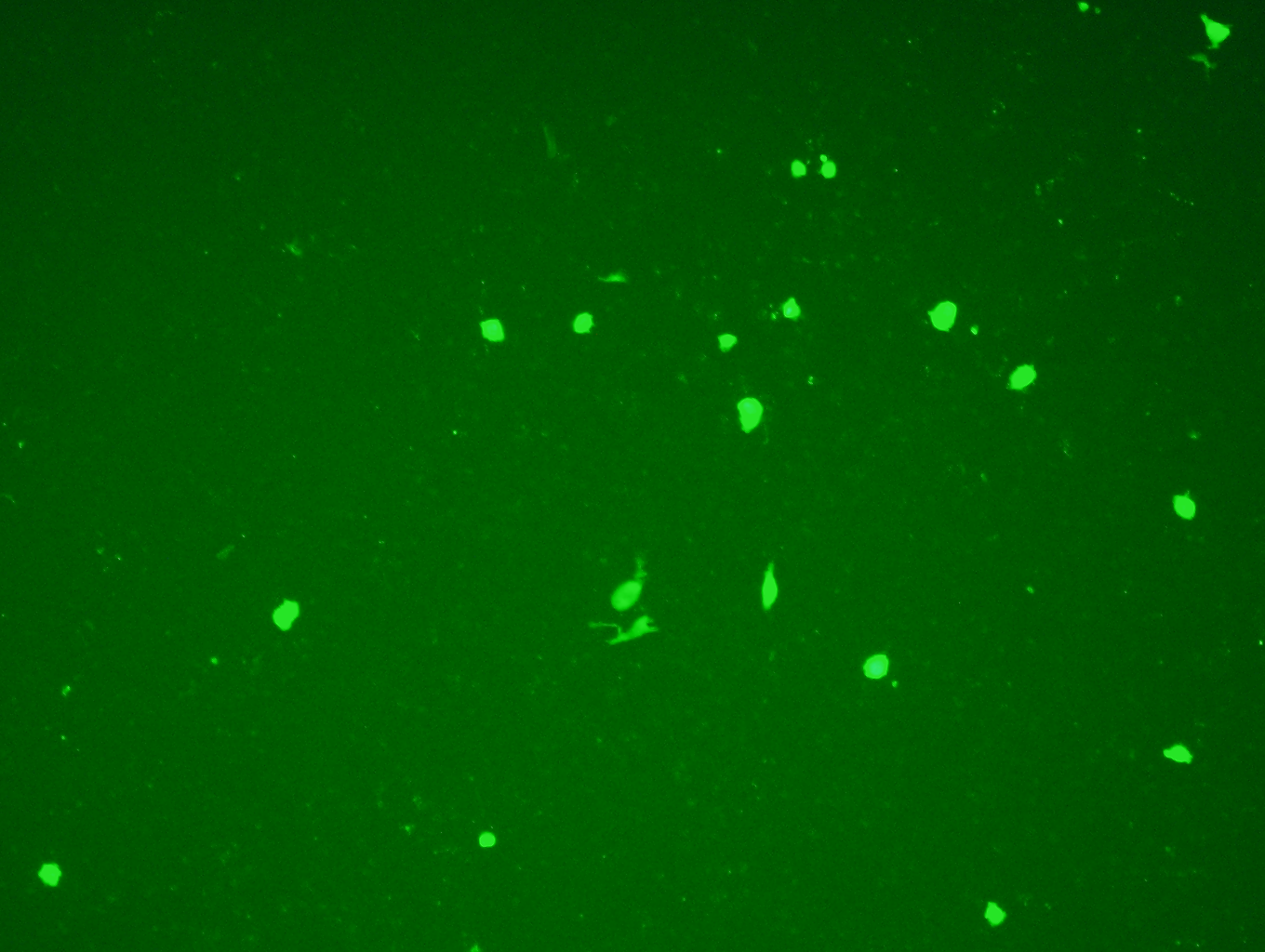

Supplement: Supplementary file 6 — Source data Fig. 5 [file 44318_2025_363_MOESM6_ESM.zip › Figure 5/5F/Ephrin A1+Defactinib(2).tif]

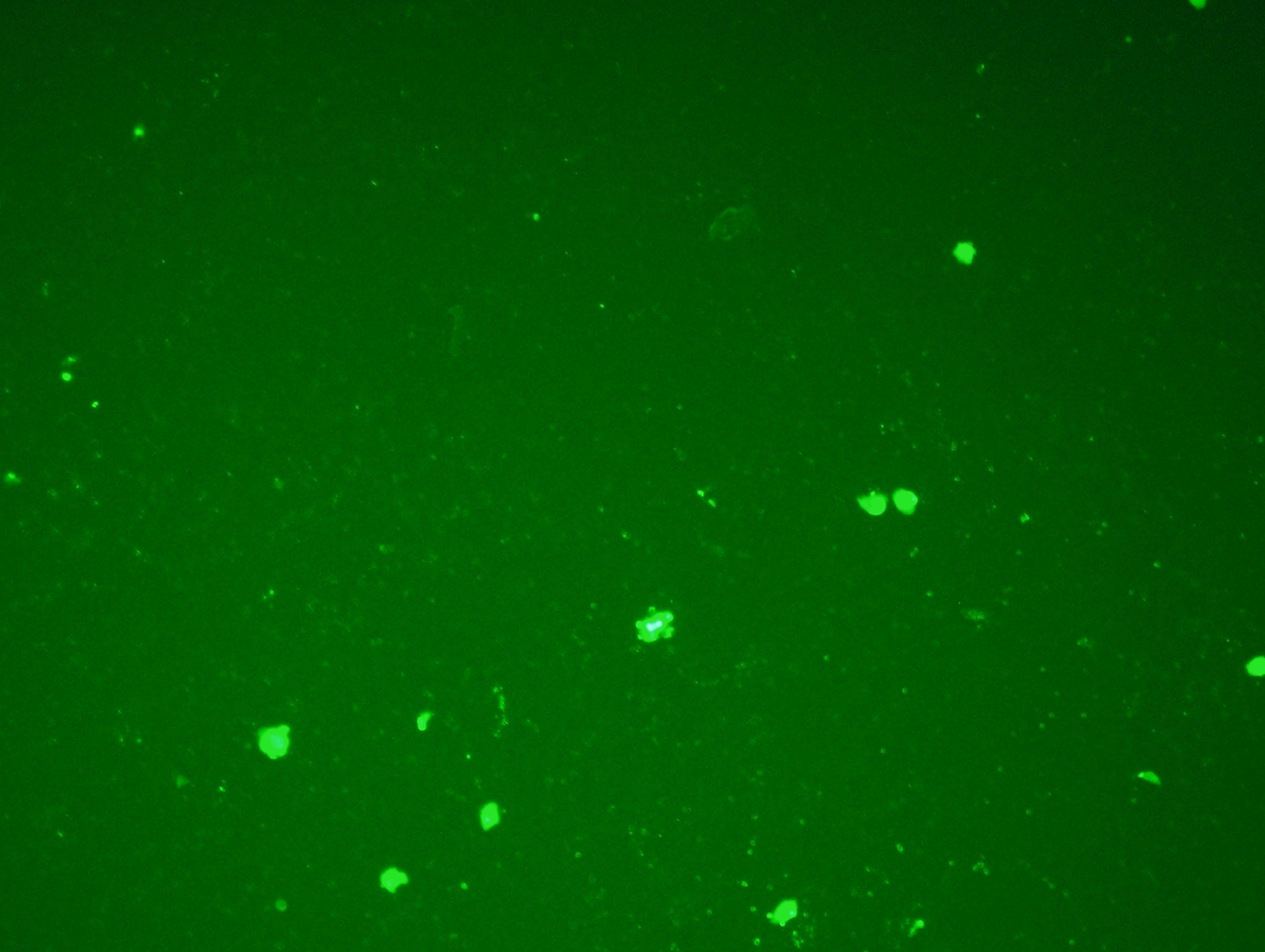

Supplement: Supplementary file 6 — Source data Fig. 5 [file 44318_2025_363_MOESM6_ESM.zip › Figure 5/5F/Ephrin A1+Defactinib(3).tif]

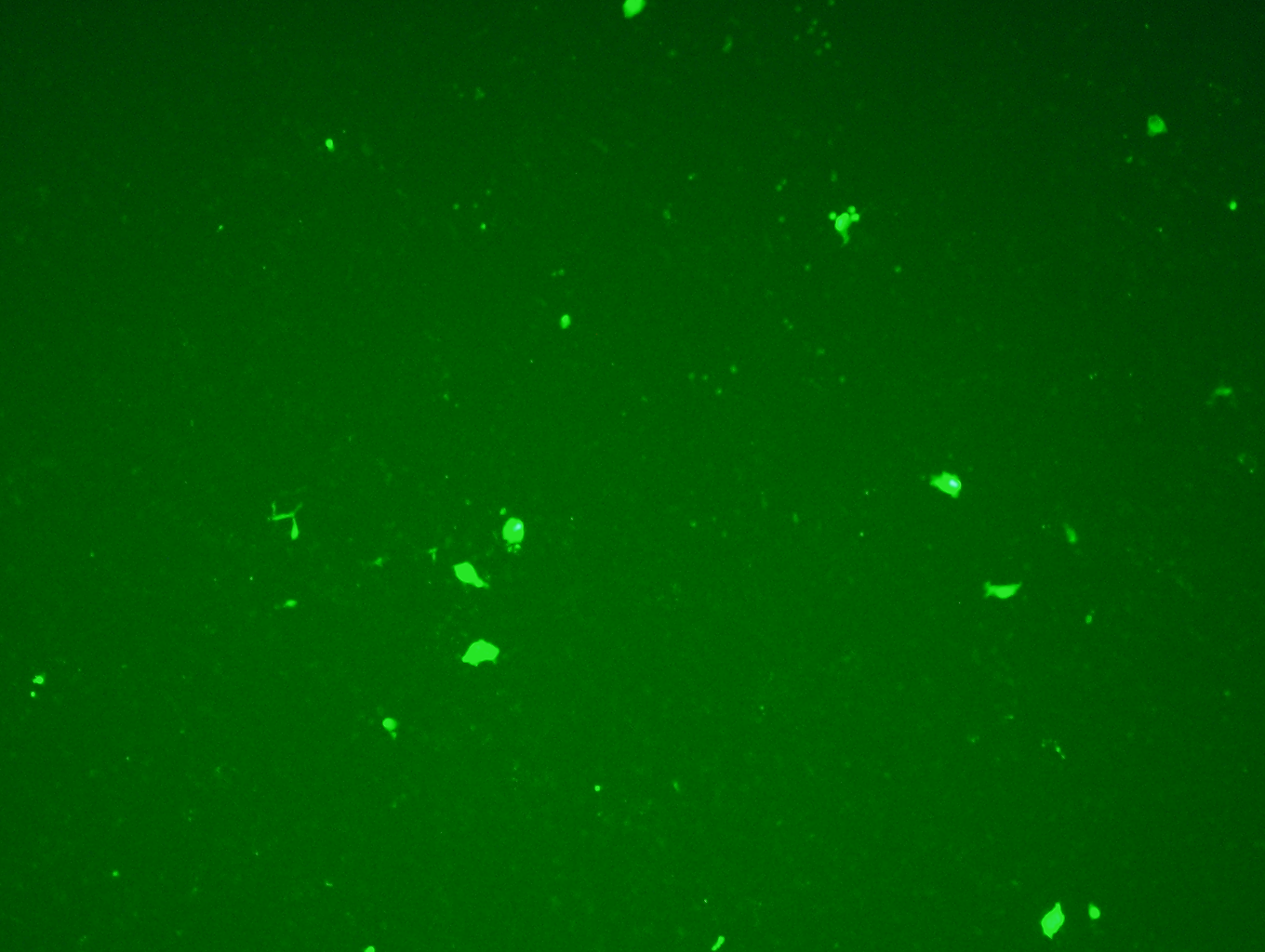

Supplement: Supplementary file 6 — Source data Fig. 5 [file 44318_2025_363_MOESM6_ESM.zip › Figure 5/5F/Ephrin A1+Defactinib(4)-displayed in 3F.tif]

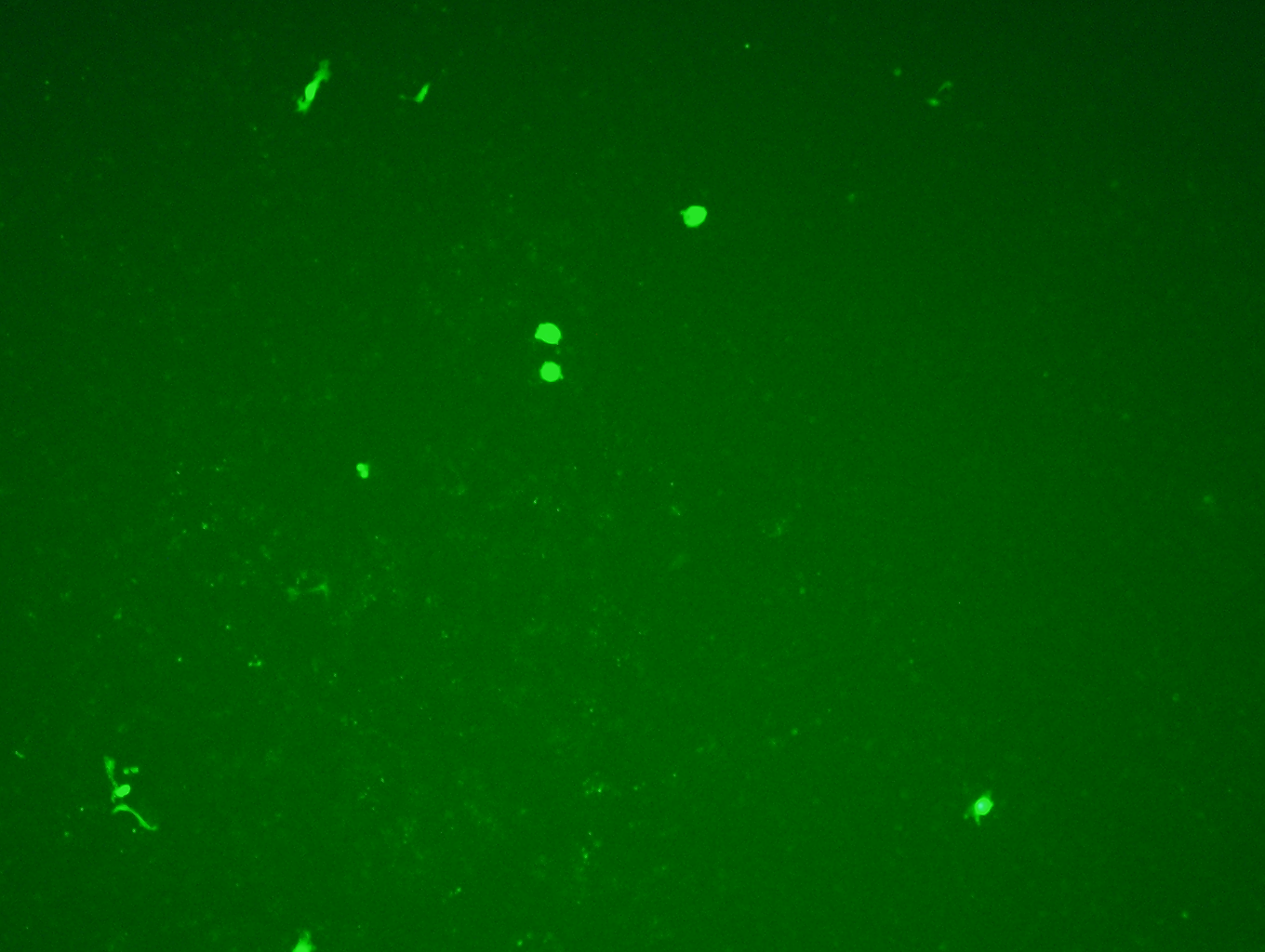

Supplement: Supplementary file 6 — Source data Fig. 5 [file 44318_2025_363_MOESM6_ESM.zip › Figure 5/5F/Ephrin A1+Defactinib(5).tif]

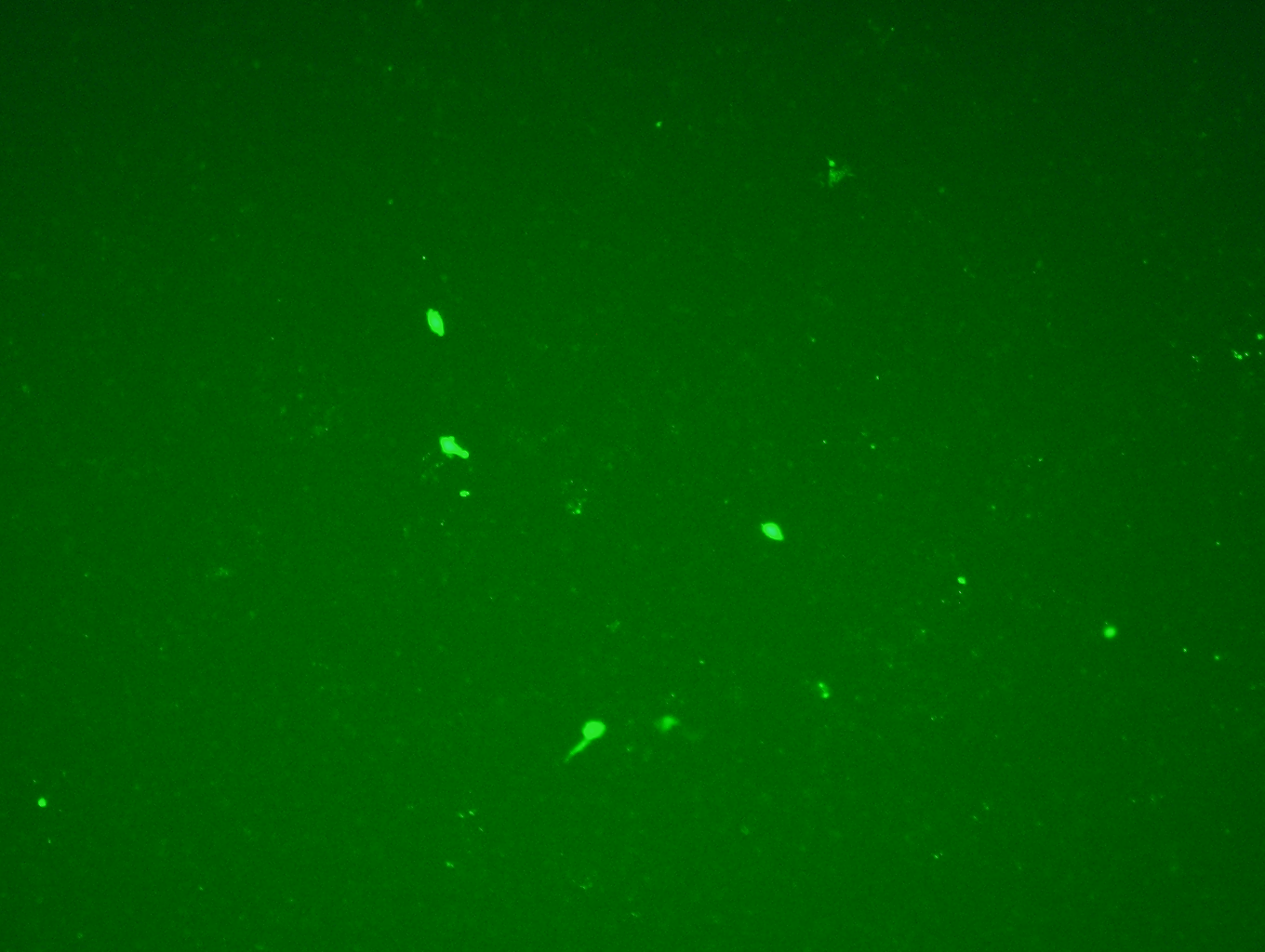

Supplement: Supplementary file 6 — Source data Fig. 5 [file 44318_2025_363_MOESM6_ESM.zip › Figure 5/5F/Ephrin A1+U0126 (1)-displayed in 3F.tif]

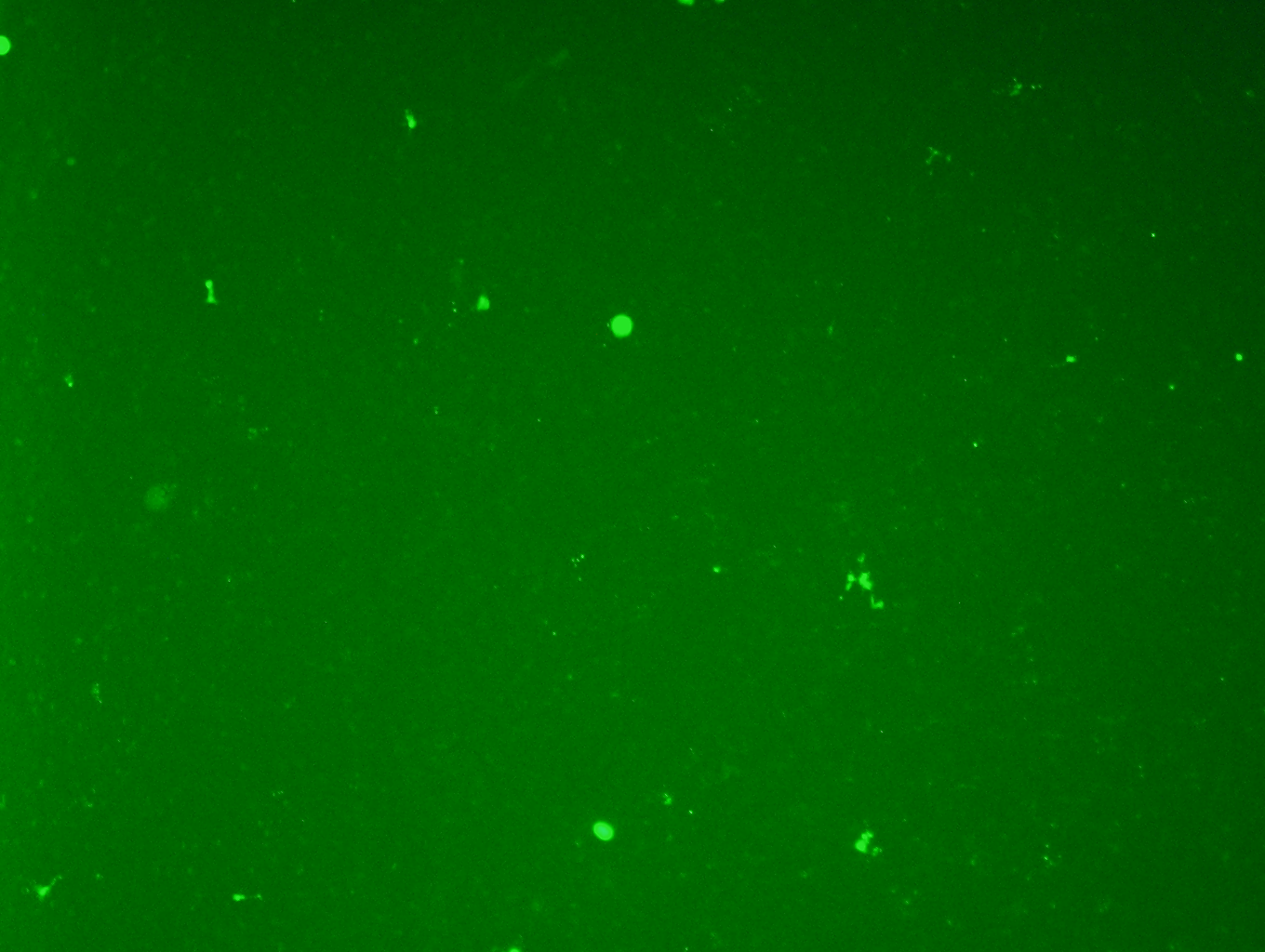

Supplement: Supplementary file 6 — Source data Fig. 5 [file 44318_2025_363_MOESM6_ESM.zip › Figure 5/5F/Ephrin A1+U0126 (2).tif]

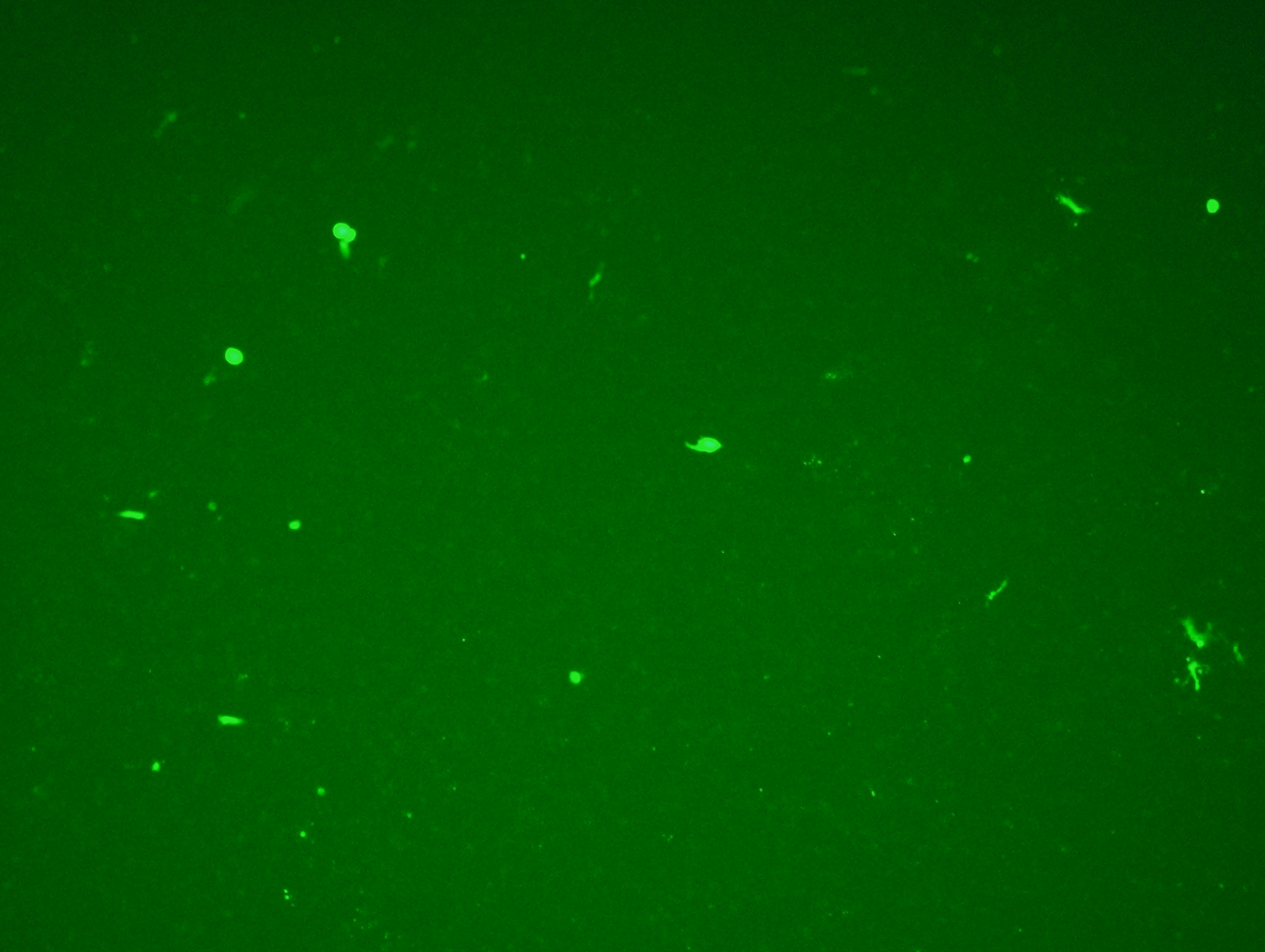

Supplement: Supplementary file 6 — Source data Fig. 5 [file 44318_2025_363_MOESM6_ESM.zip › Figure 5/5F/Ephrin A1+U0126 (3).tif]

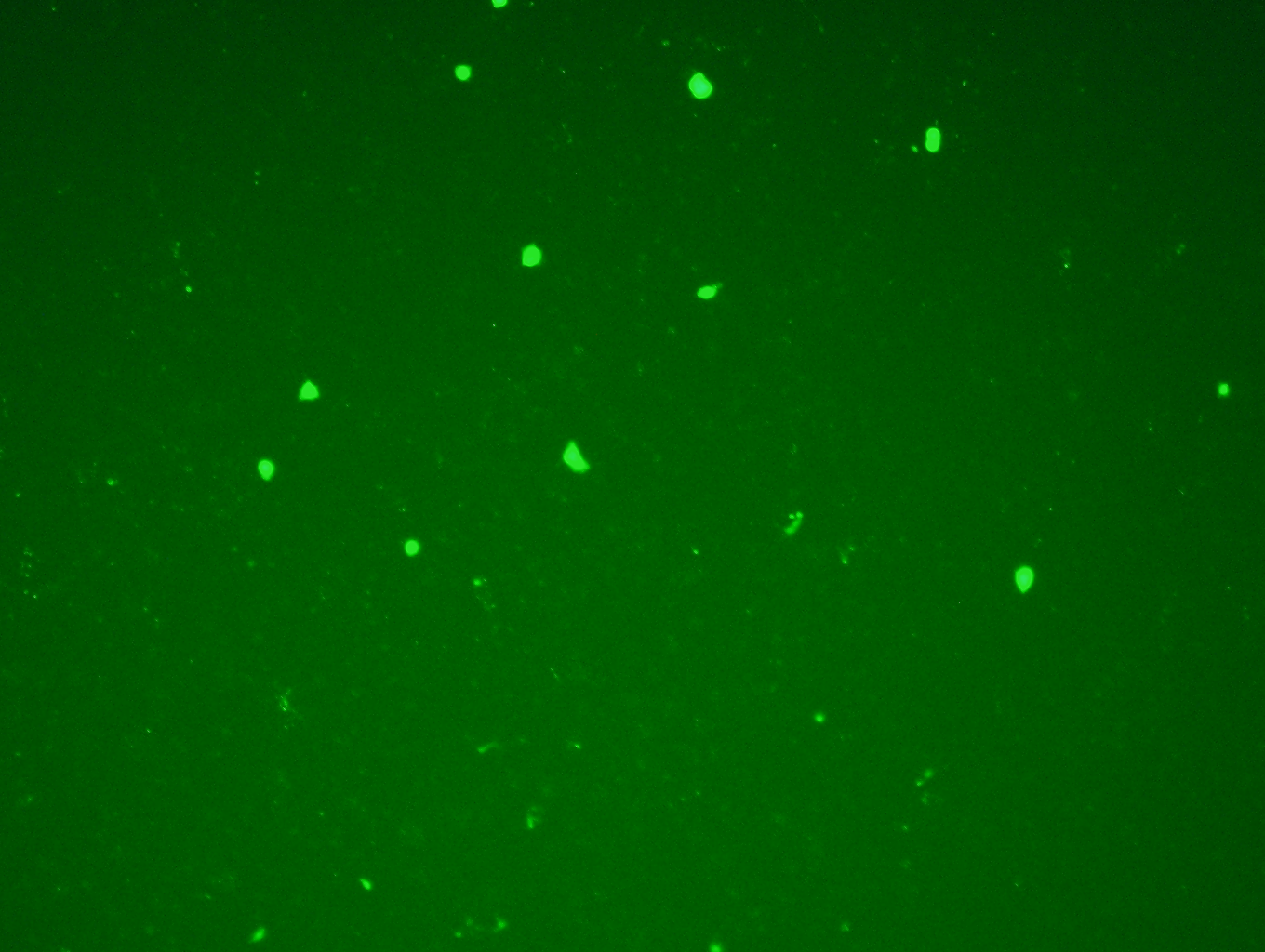

Supplement: Supplementary file 6 — Source data Fig. 5 [file 44318_2025_363_MOESM6_ESM.zip › Figure 5/5F/Ephrin A1+U0126 (4).tif]

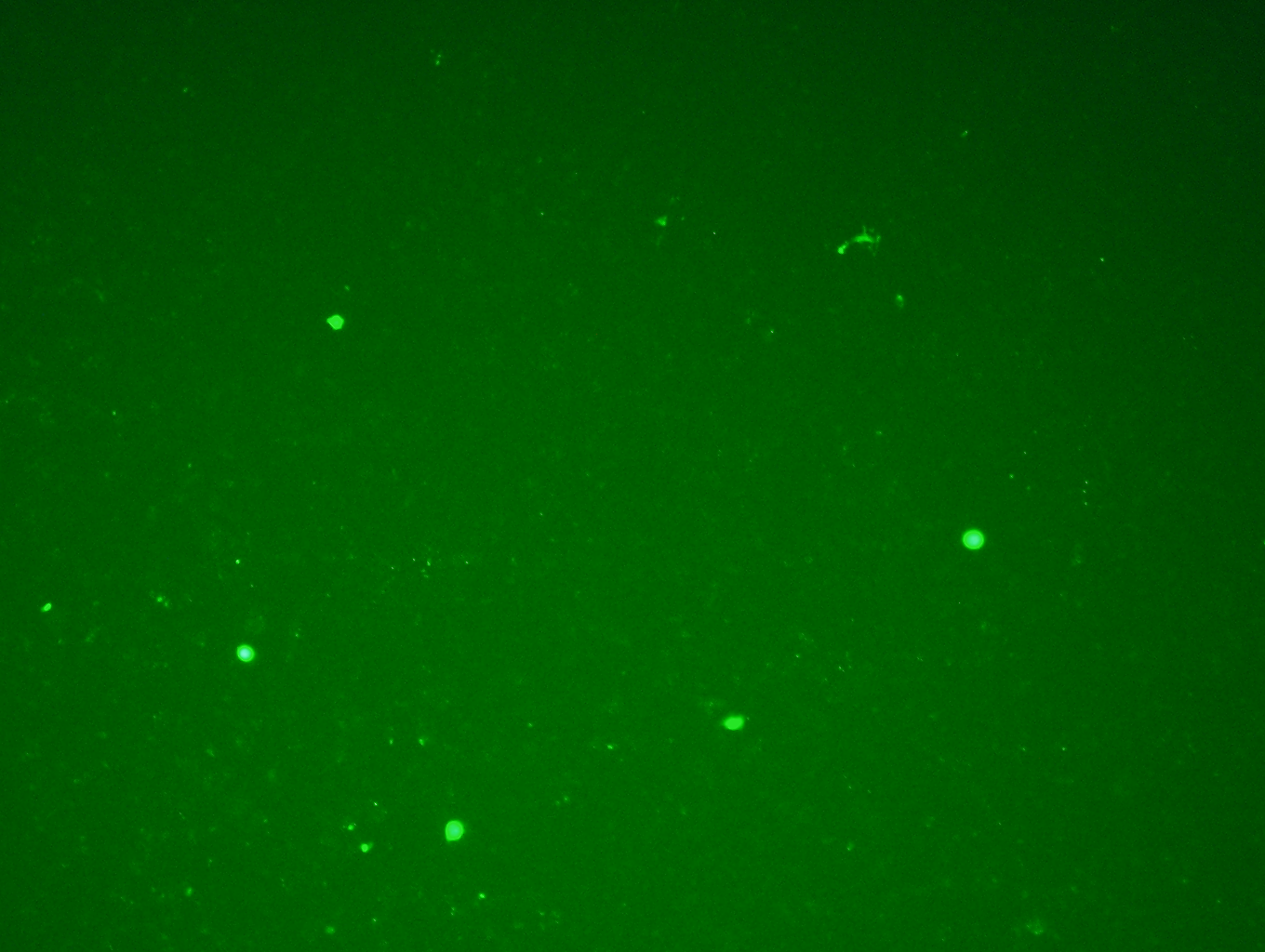

Supplement: Supplementary file 6 — Source data Fig. 5 [file 44318_2025_363_MOESM6_ESM.zip › Figure 5/5F/Ephrin A1+U0126 (5).tif]

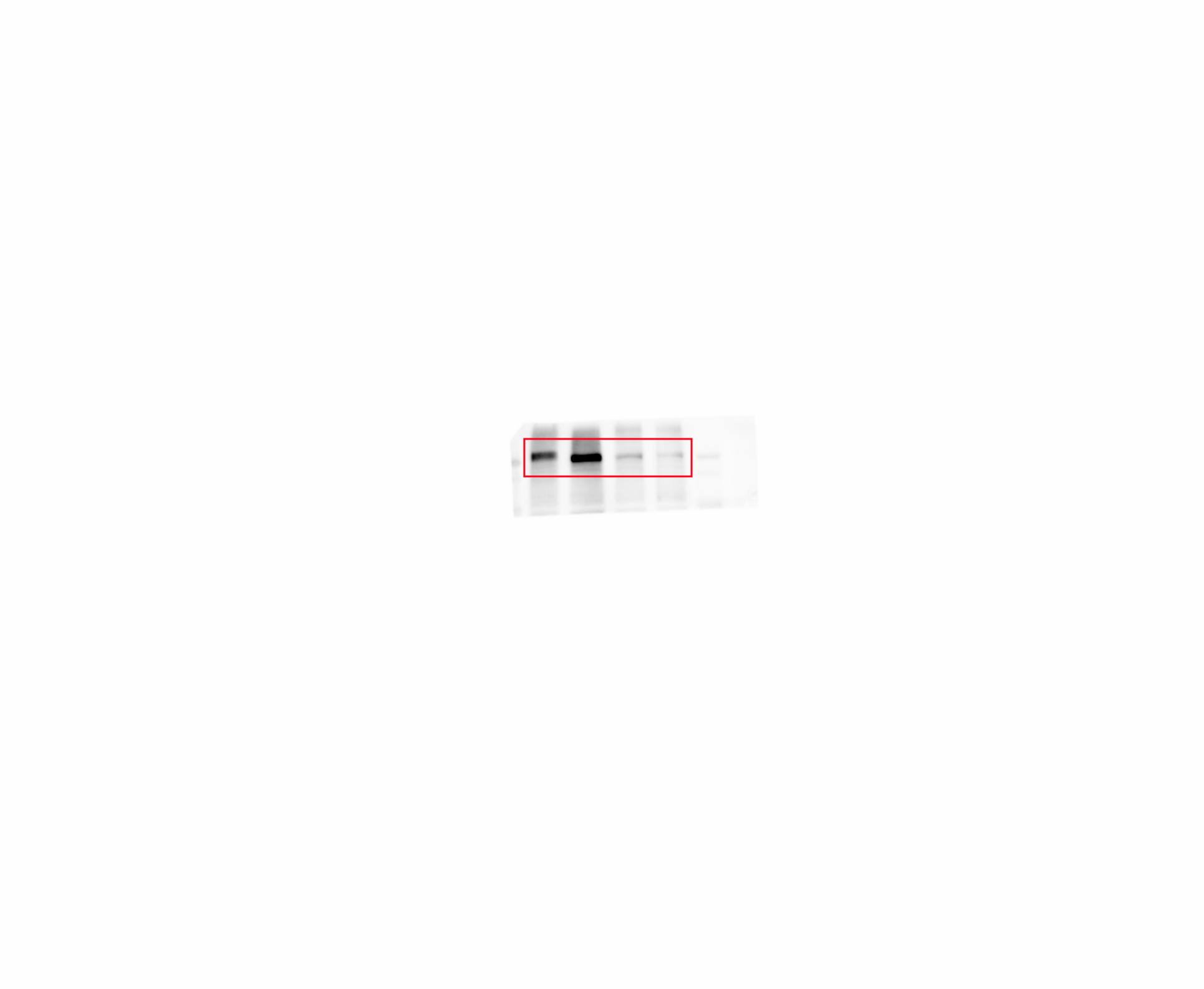

Supplement: Supplementary file 6 — Source data Fig. 5 [file 44318_2025_363_MOESM6_ESM.zip › Figure 5/5H/1 p-EGFR.tif]

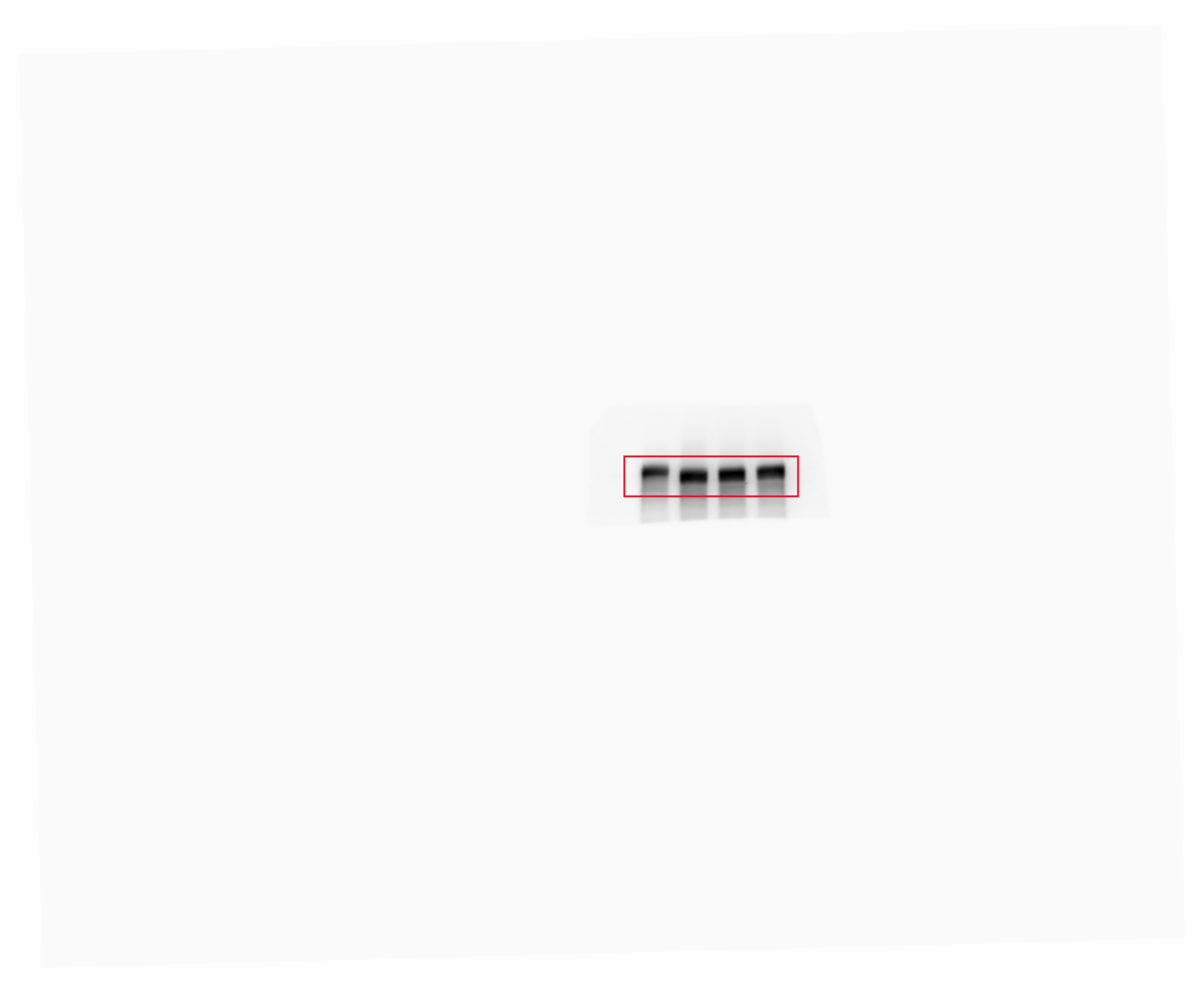

Supplement: Supplementary file 6 — Source data Fig. 5 [file 44318_2025_363_MOESM6_ESM.zip › Figure 5/5H/2 EGFR.tif]

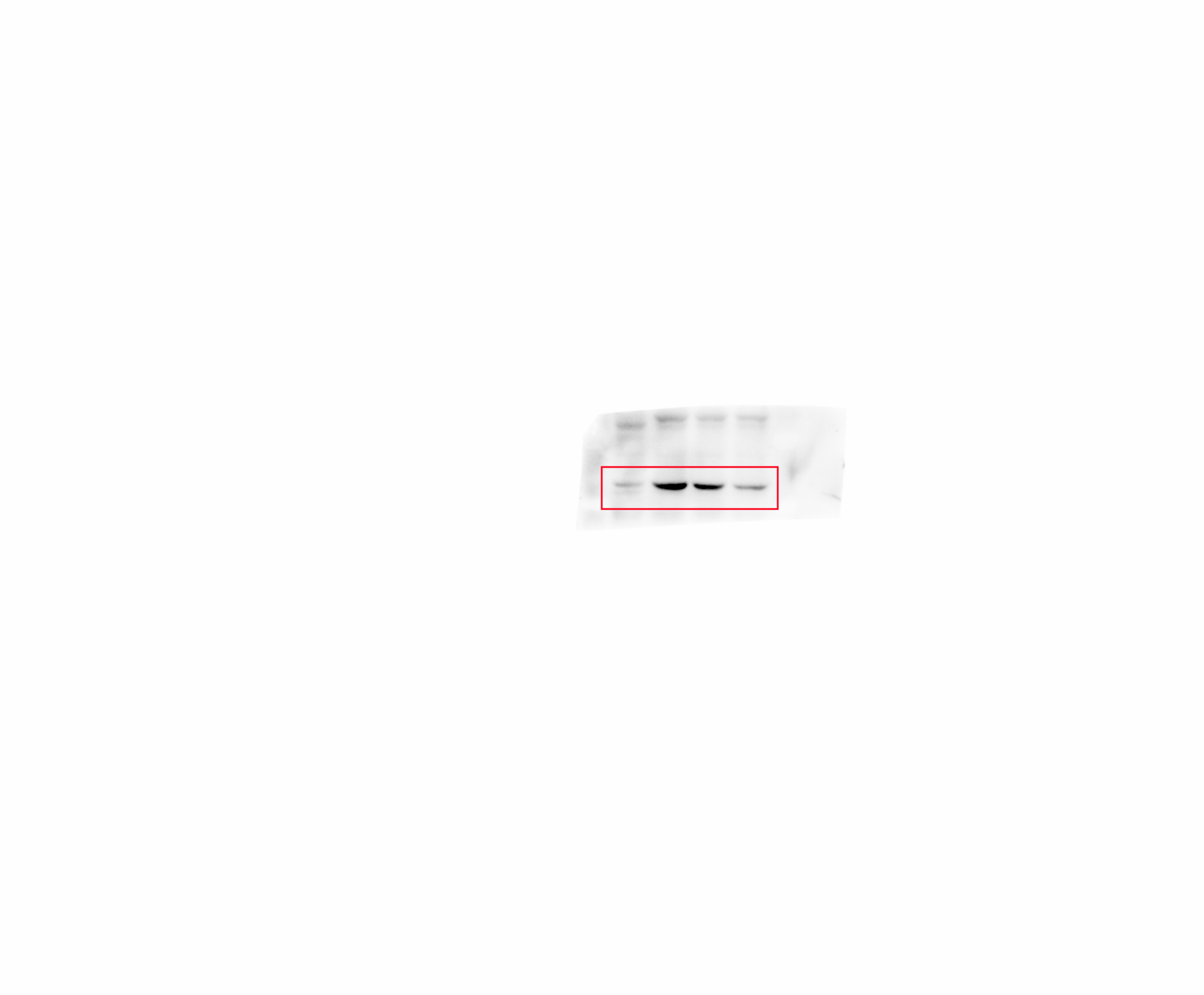

Supplement: Supplementary file 6 — Source data Fig. 5 [file 44318_2025_363_MOESM6_ESM.zip › Figure 5/5H/3 P-ERK.tif]

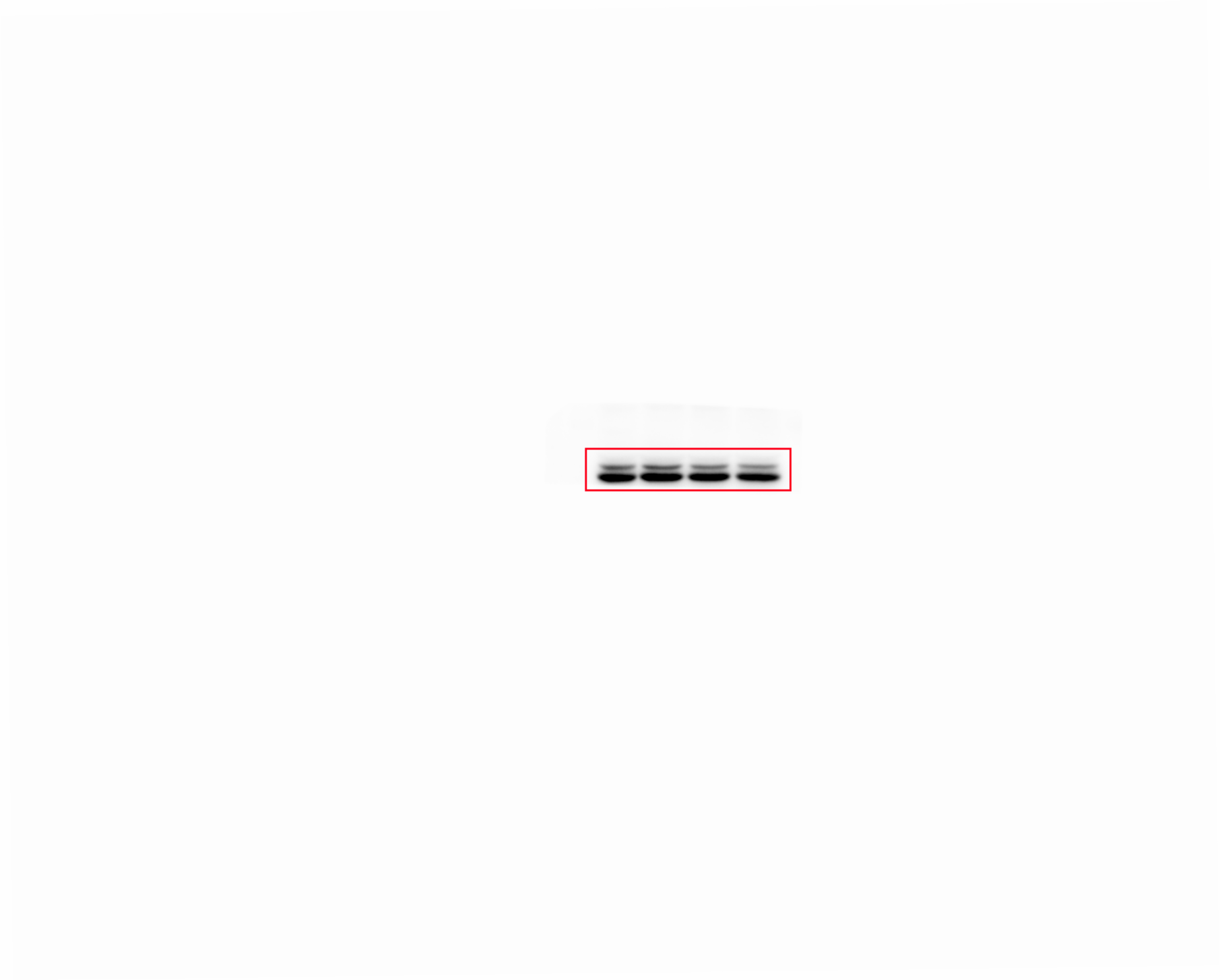

Supplement: Supplementary file 6 — Source data Fig. 5 [file 44318_2025_363_MOESM6_ESM.zip › Figure 5/5H/4 ERK.tif]

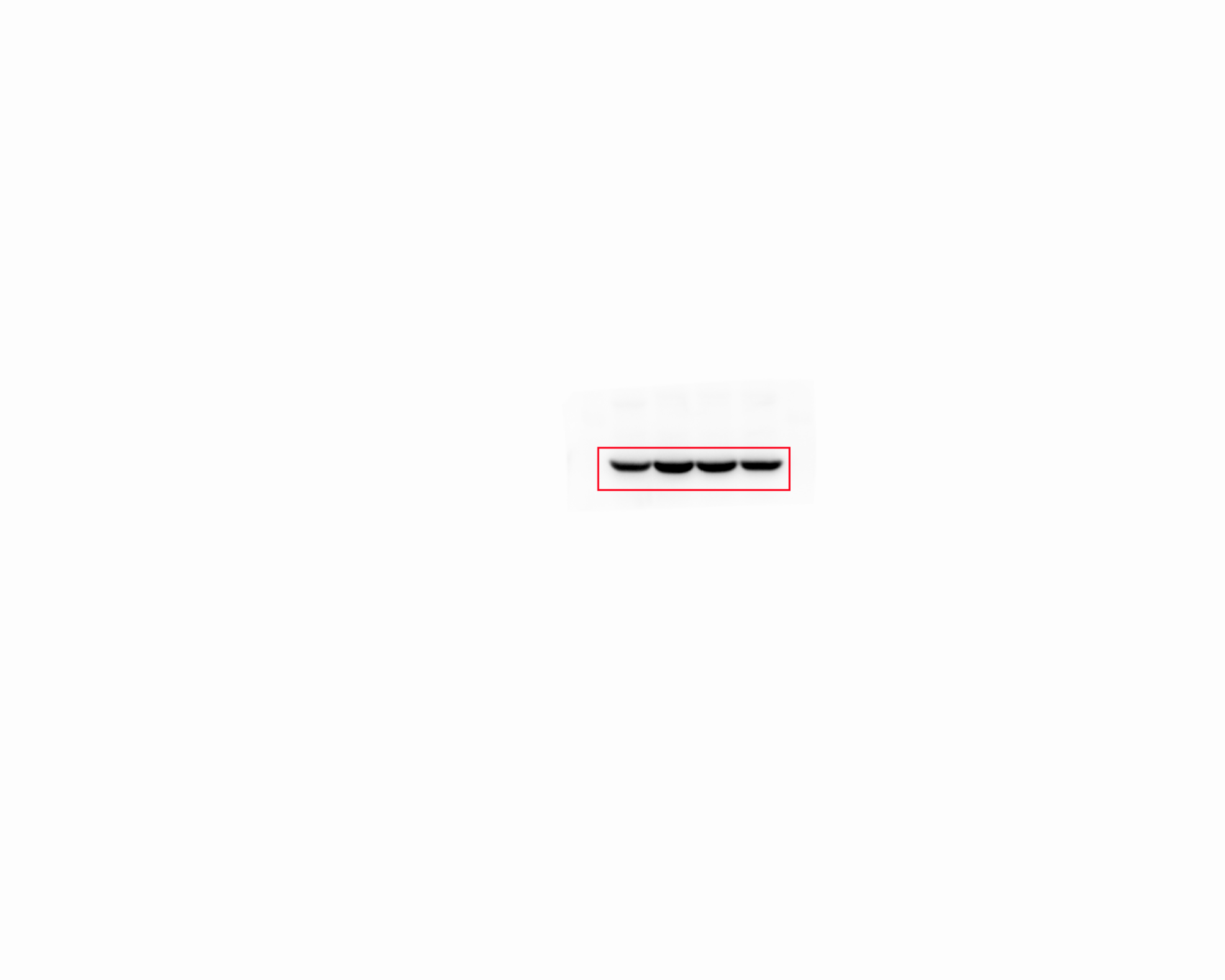

Supplement: Supplementary file 6 — Source data Fig. 5 [file 44318_2025_363_MOESM6_ESM.zip › Figure 5/5H/5 actin.tif]

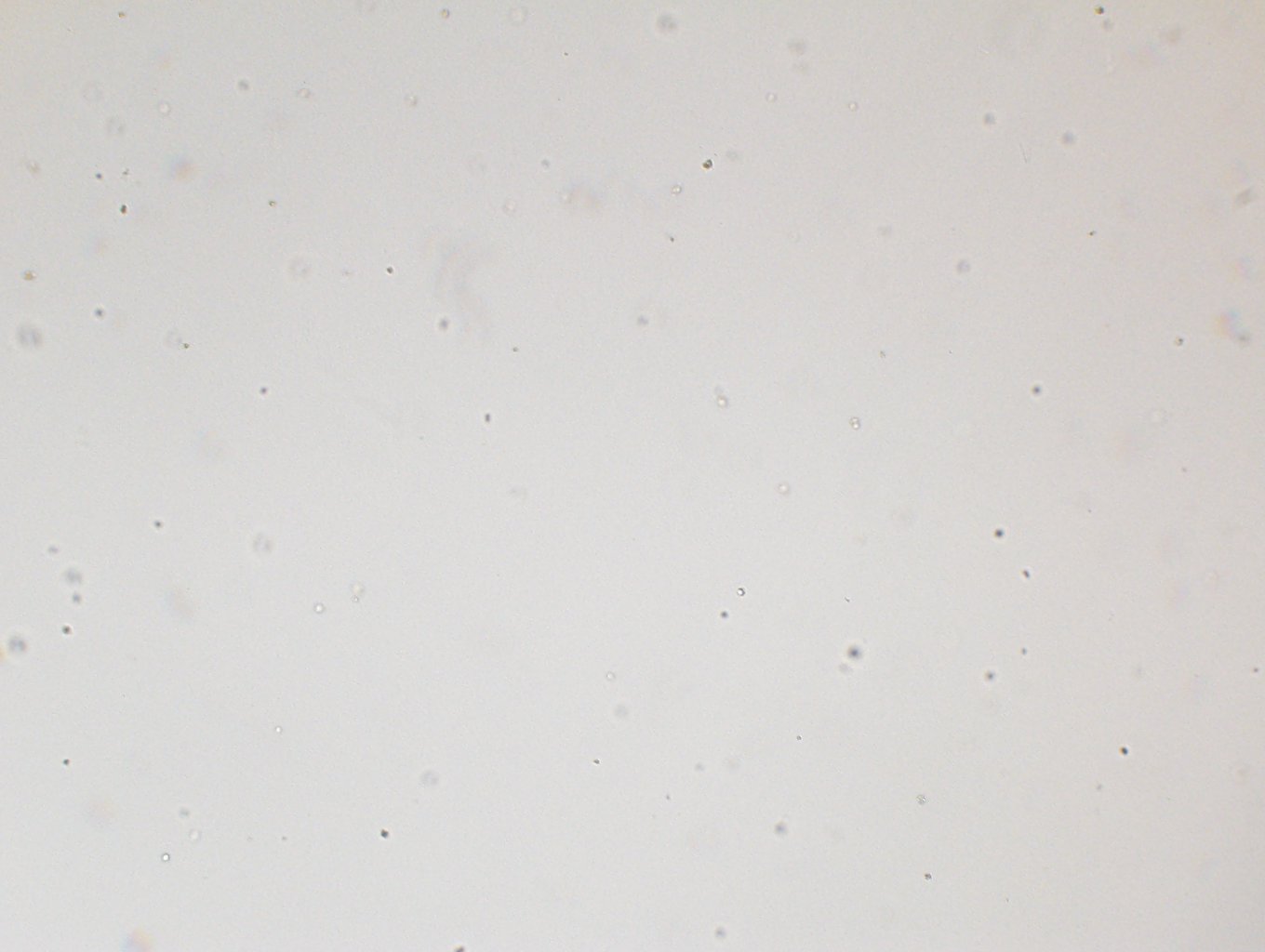

Supplement: Supplementary file 6 — Source data Fig. 5 [file 44318_2025_363_MOESM6_ESM.zip › Figure 5/5I/Control (1).jpg]

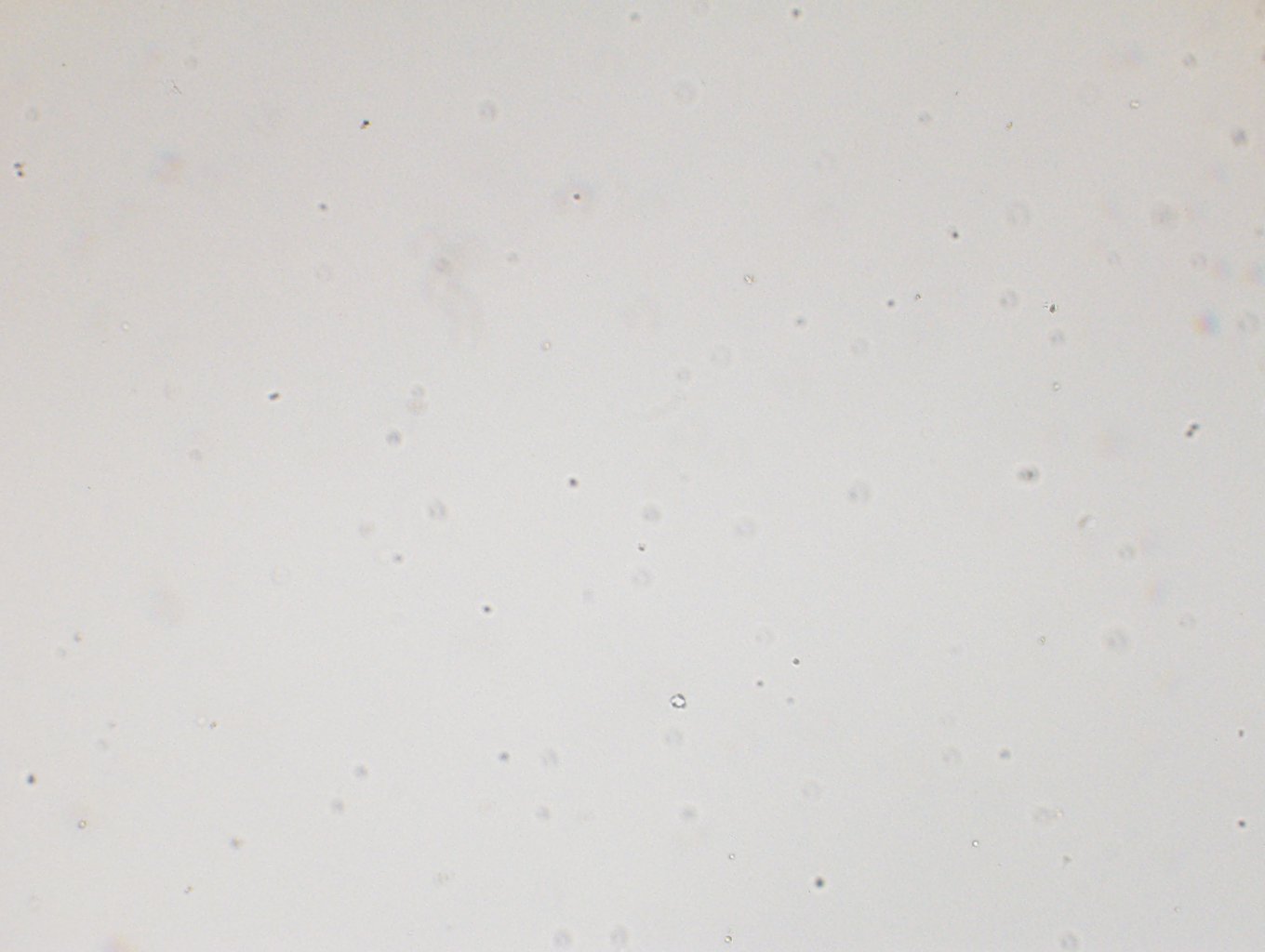

Supplement: Supplementary file 6 — Source data Fig. 5 [file 44318_2025_363_MOESM6_ESM.zip › Figure 5/5I/Control (2)-displayed in 5I.jpg]

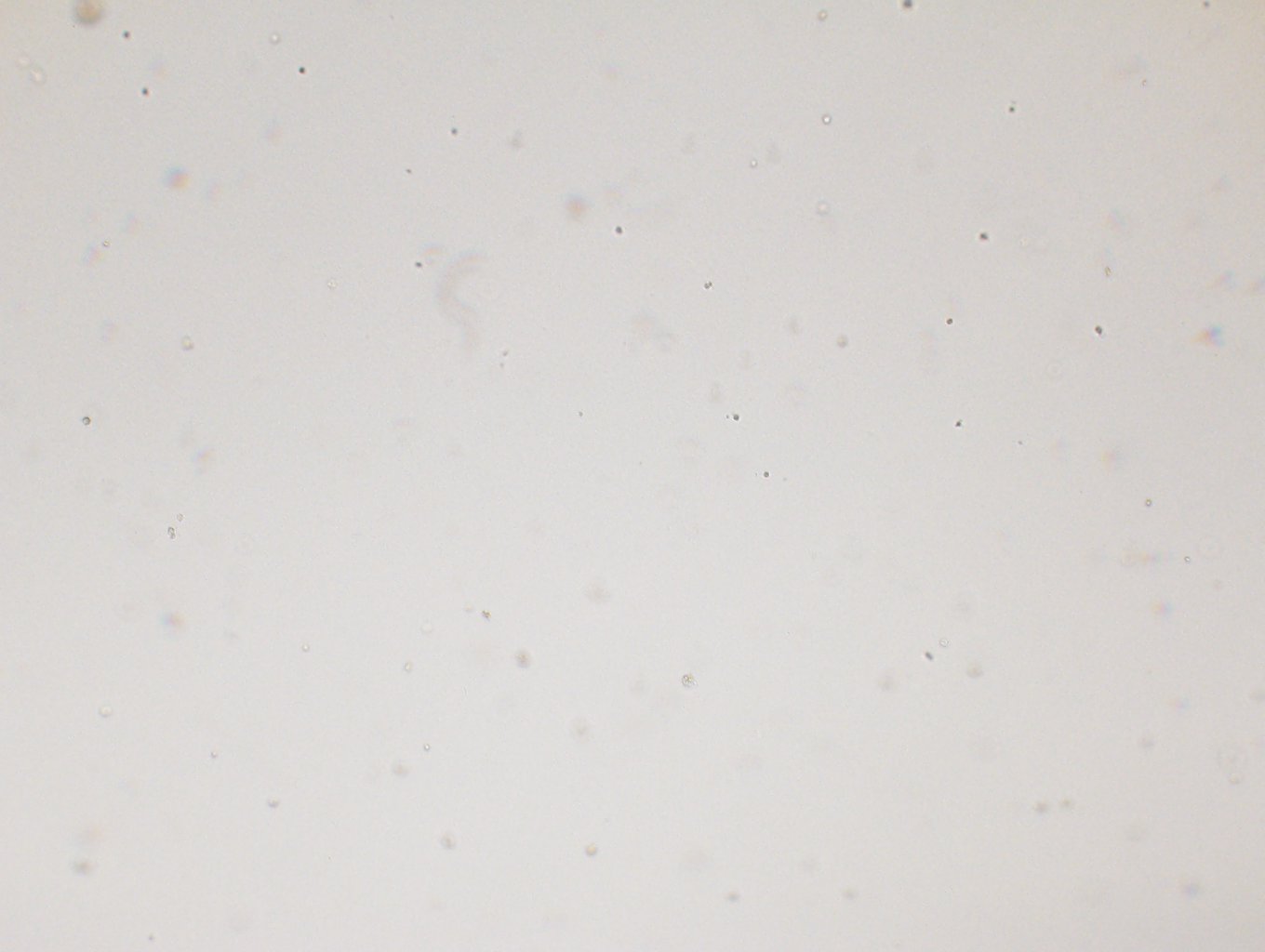

Supplement: Supplementary file 6 — Source data Fig. 5 [file 44318_2025_363_MOESM6_ESM.zip › Figure 5/5I/Control (3).jpg]

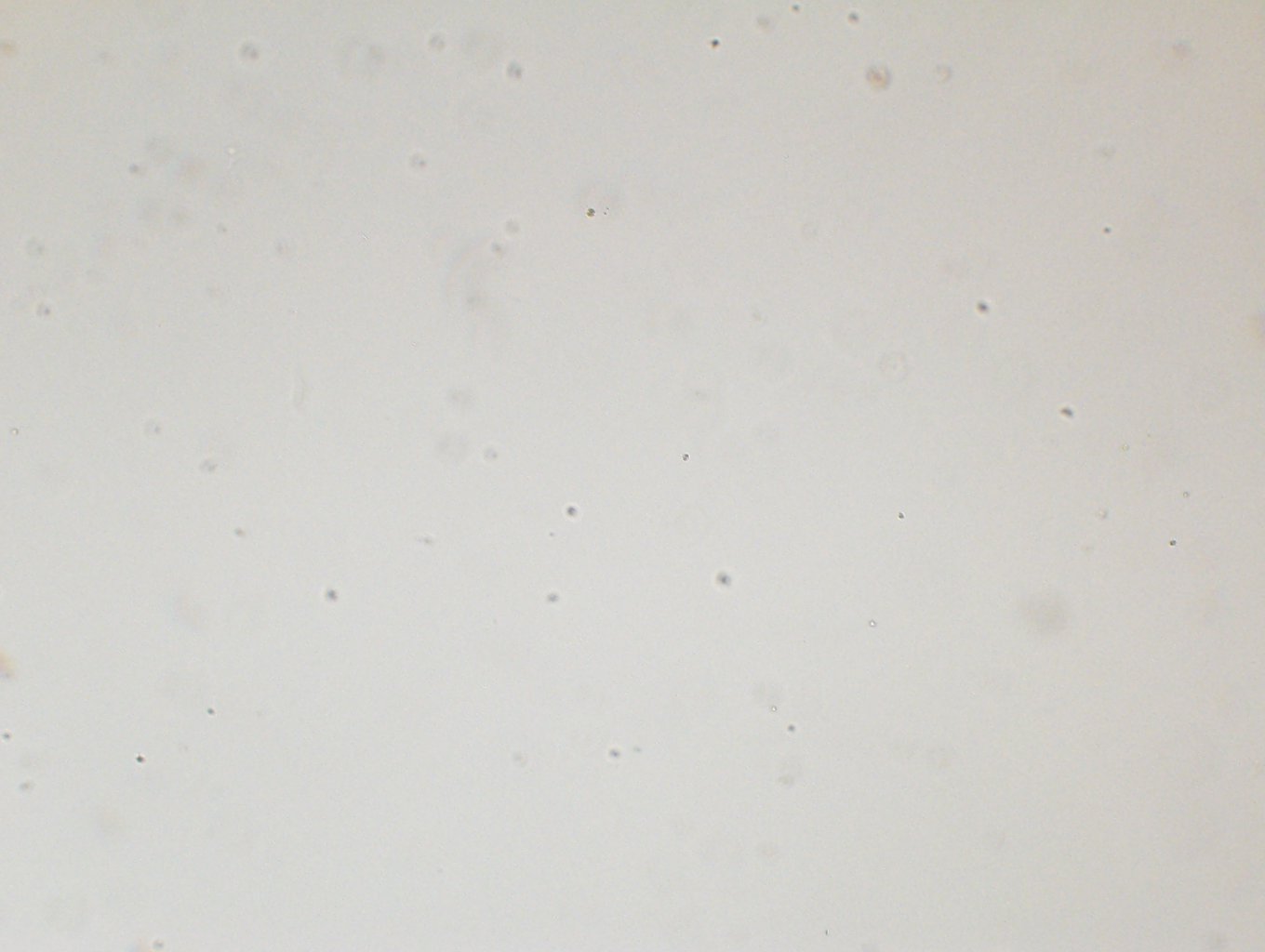

Supplement: Supplementary file 6 — Source data Fig. 5 [file 44318_2025_363_MOESM6_ESM.zip › Figure 5/5I/Control (4).jpg]

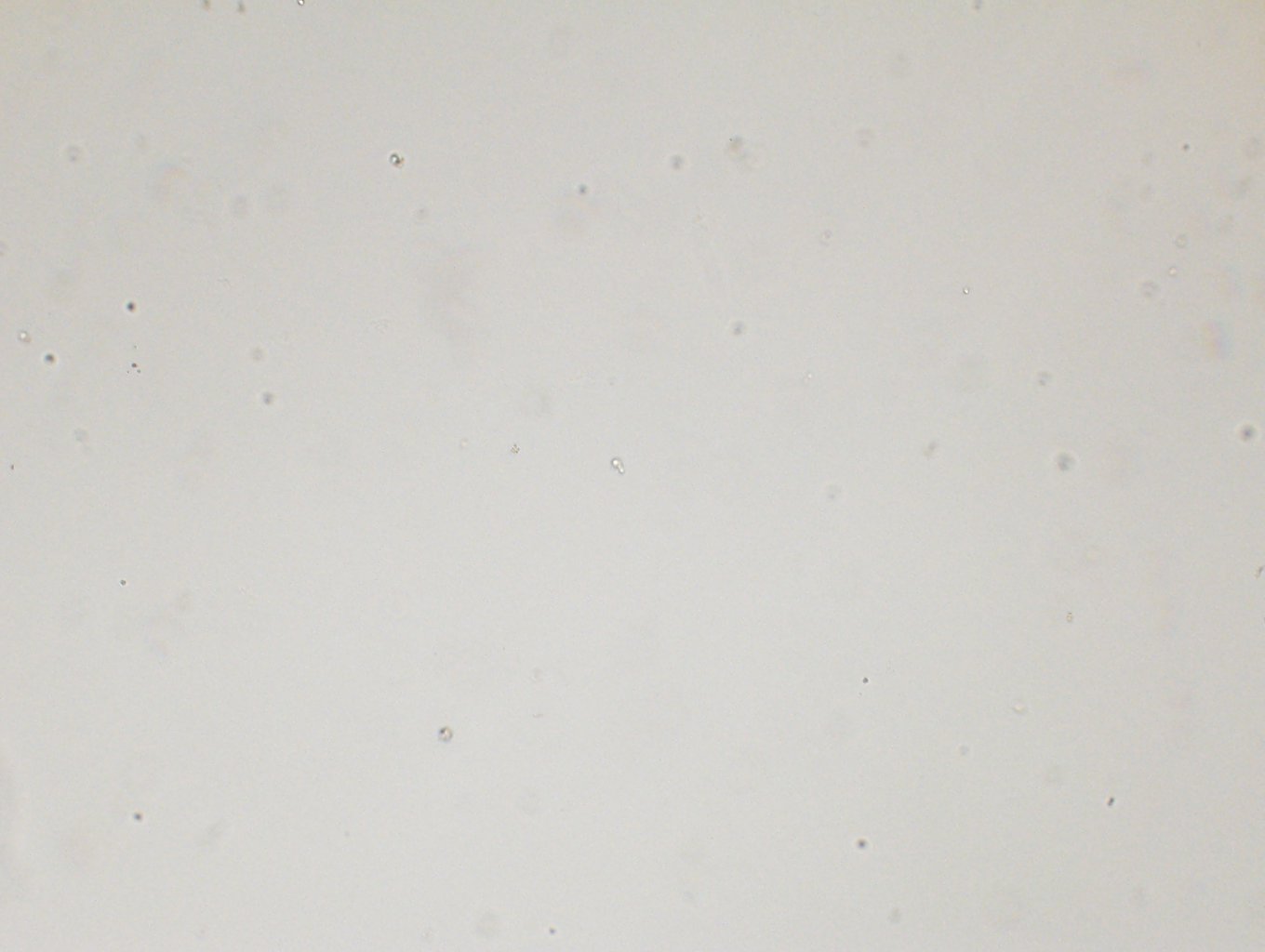

Supplement: Supplementary file 6 — Source data Fig. 5 [file 44318_2025_363_MOESM6_ESM.zip › Figure 5/5I/Control (5).jpg]

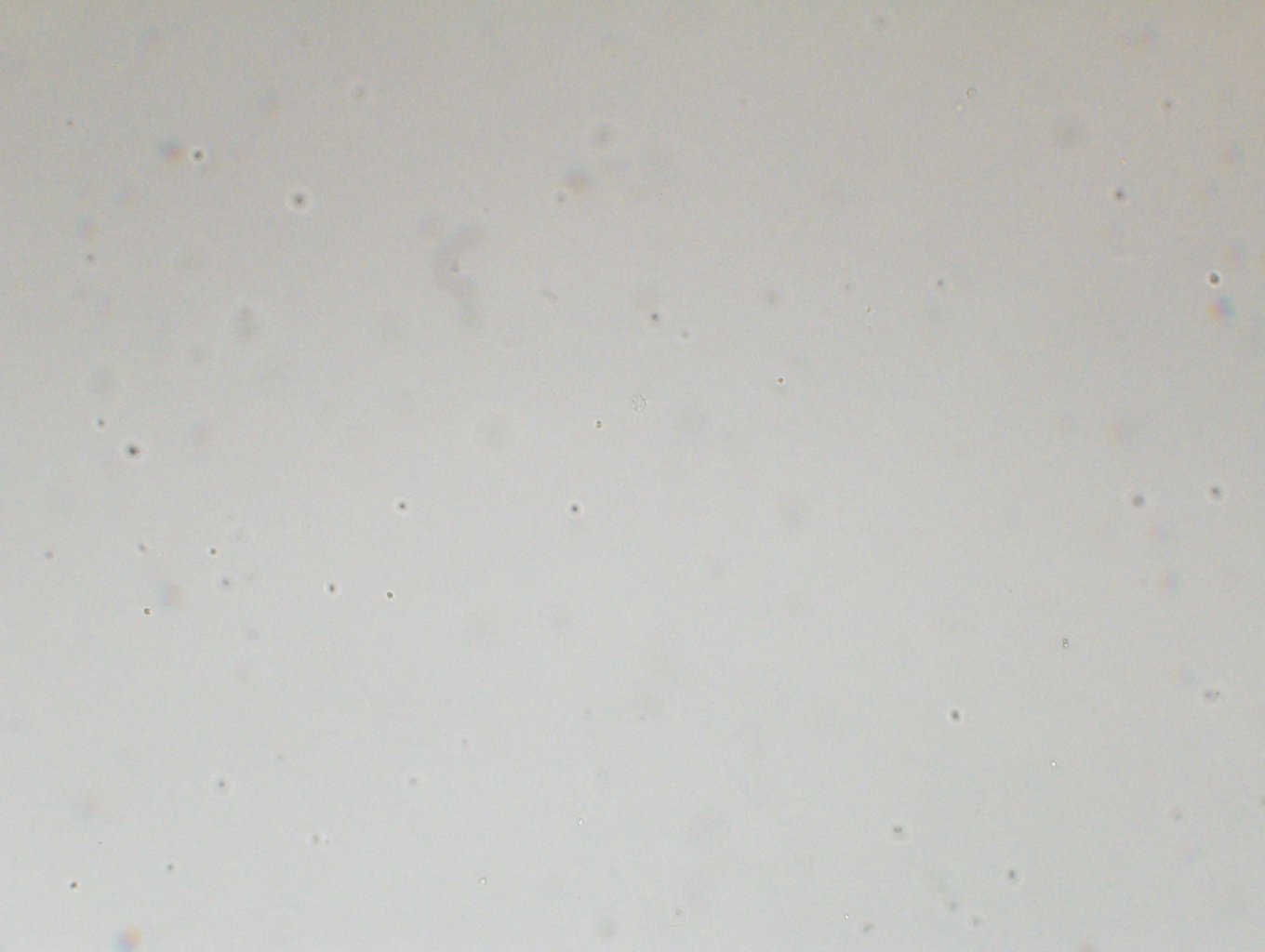

Supplement: Supplementary file 6 — Source data Fig. 5 [file 44318_2025_363_MOESM6_ESM.zip › Figure 5/5I/Control (6).jpg]

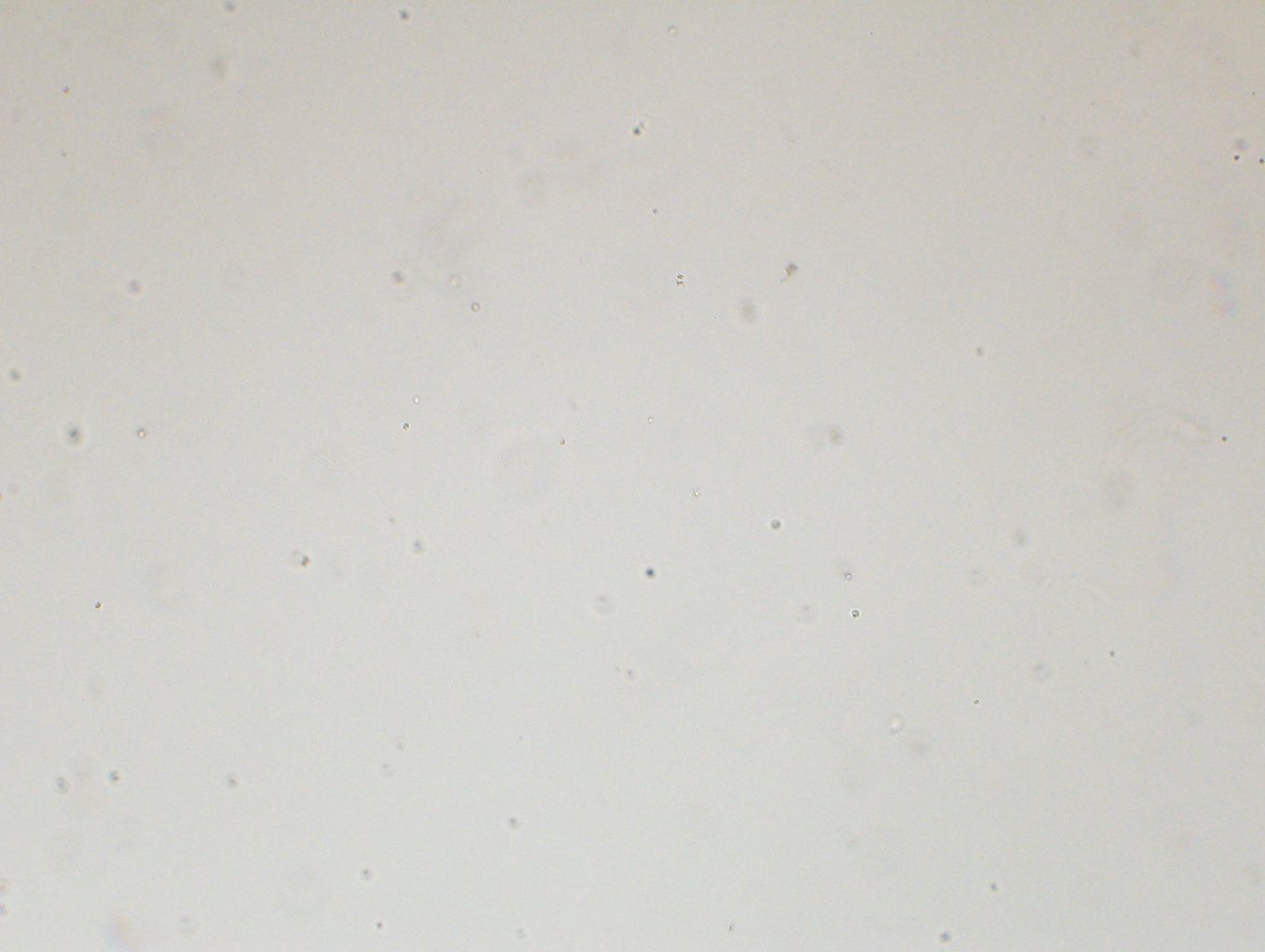

Supplement: Supplementary file 6 — Source data Fig. 5 [file 44318_2025_363_MOESM6_ESM.zip › Figure 5/5I/Control (7).jpg]

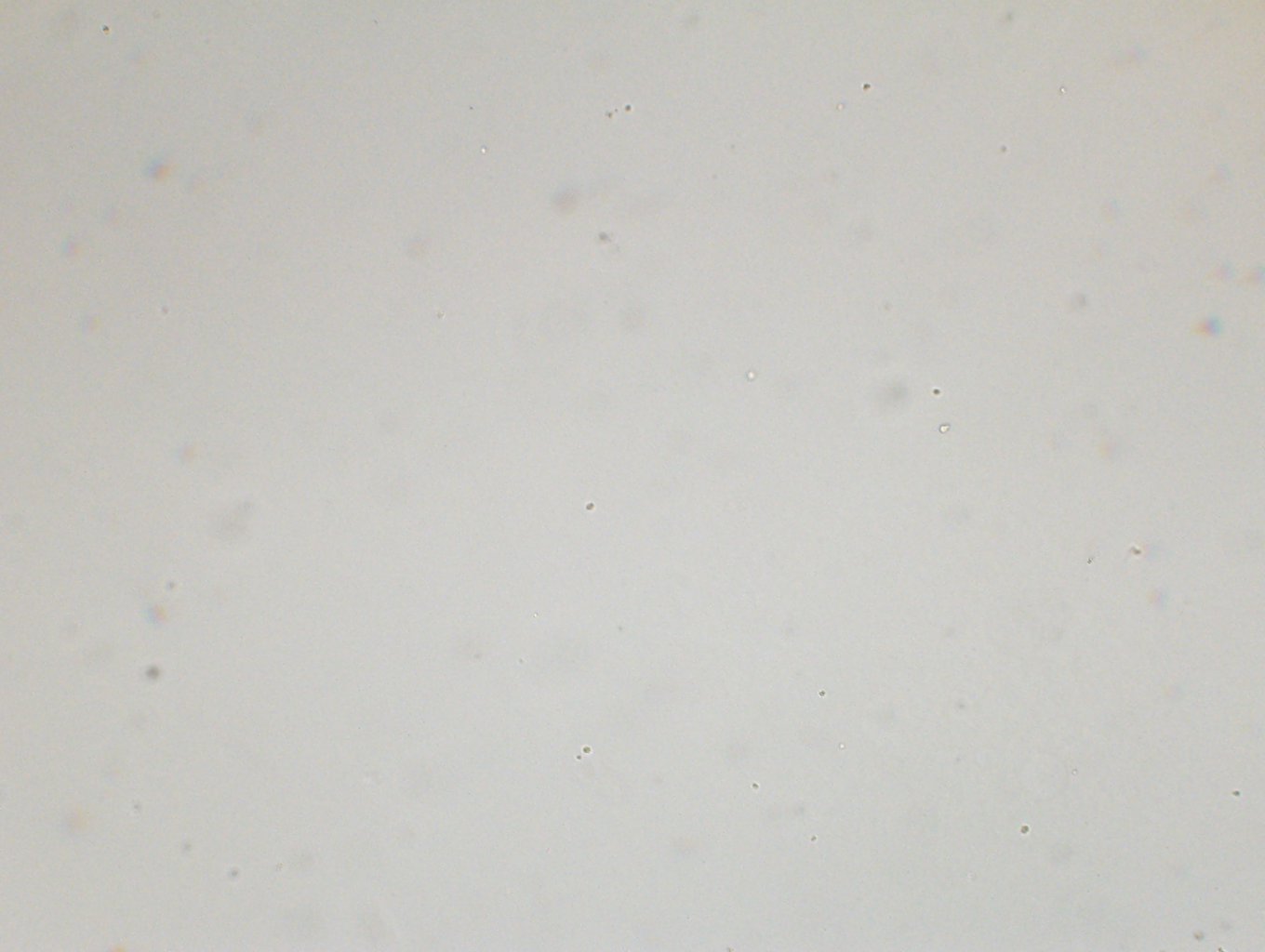

Supplement: Supplementary file 6 — Source data Fig. 5 [file 44318_2025_363_MOESM6_ESM.zip › Figure 5/5I/Control (8).jpg]

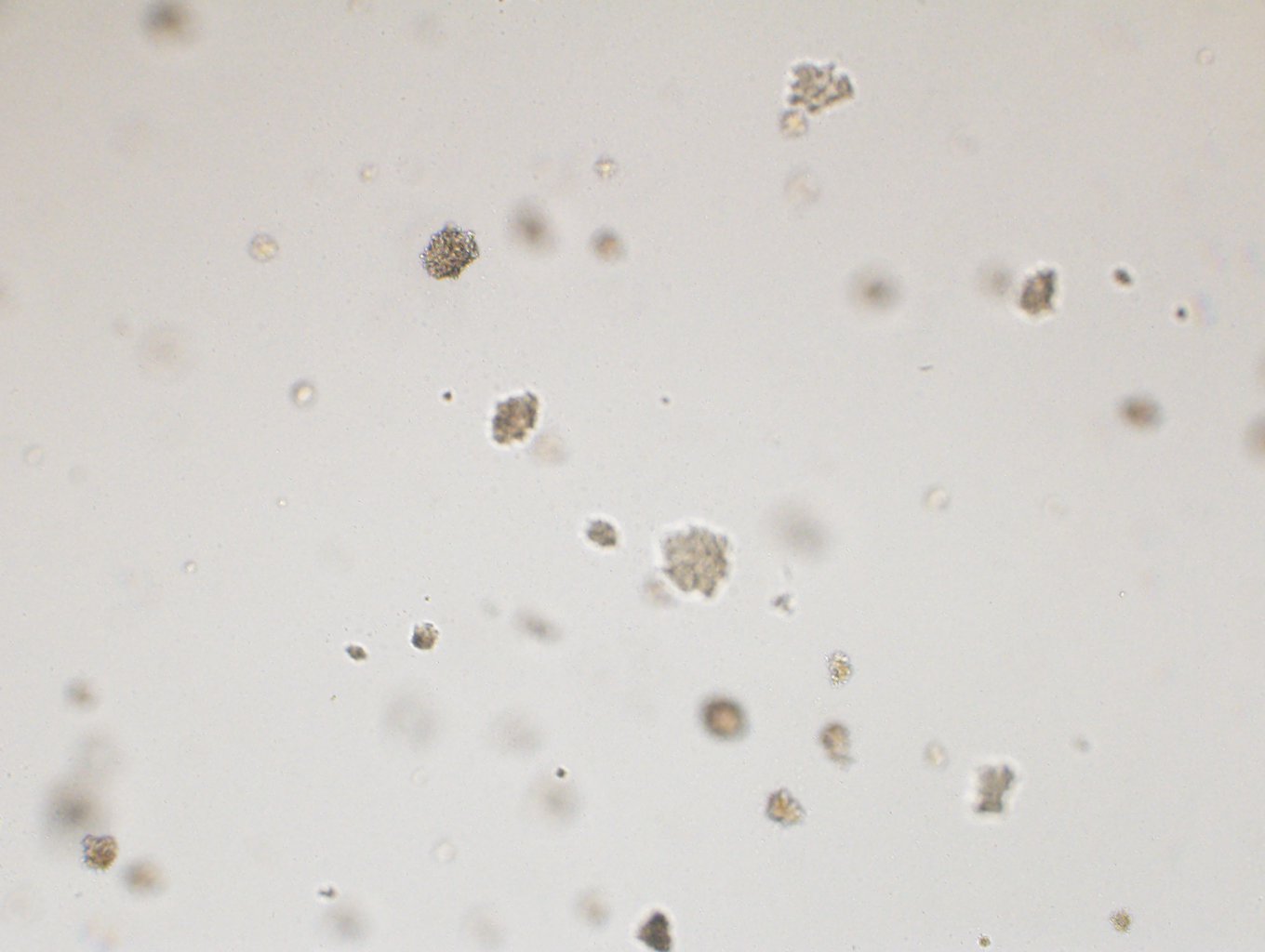

Supplement: Supplementary file 6 — Source data Fig. 5 [file 44318_2025_363_MOESM6_ESM.zip › Figure 5/5I/Ephrin A1 (1).jpg]

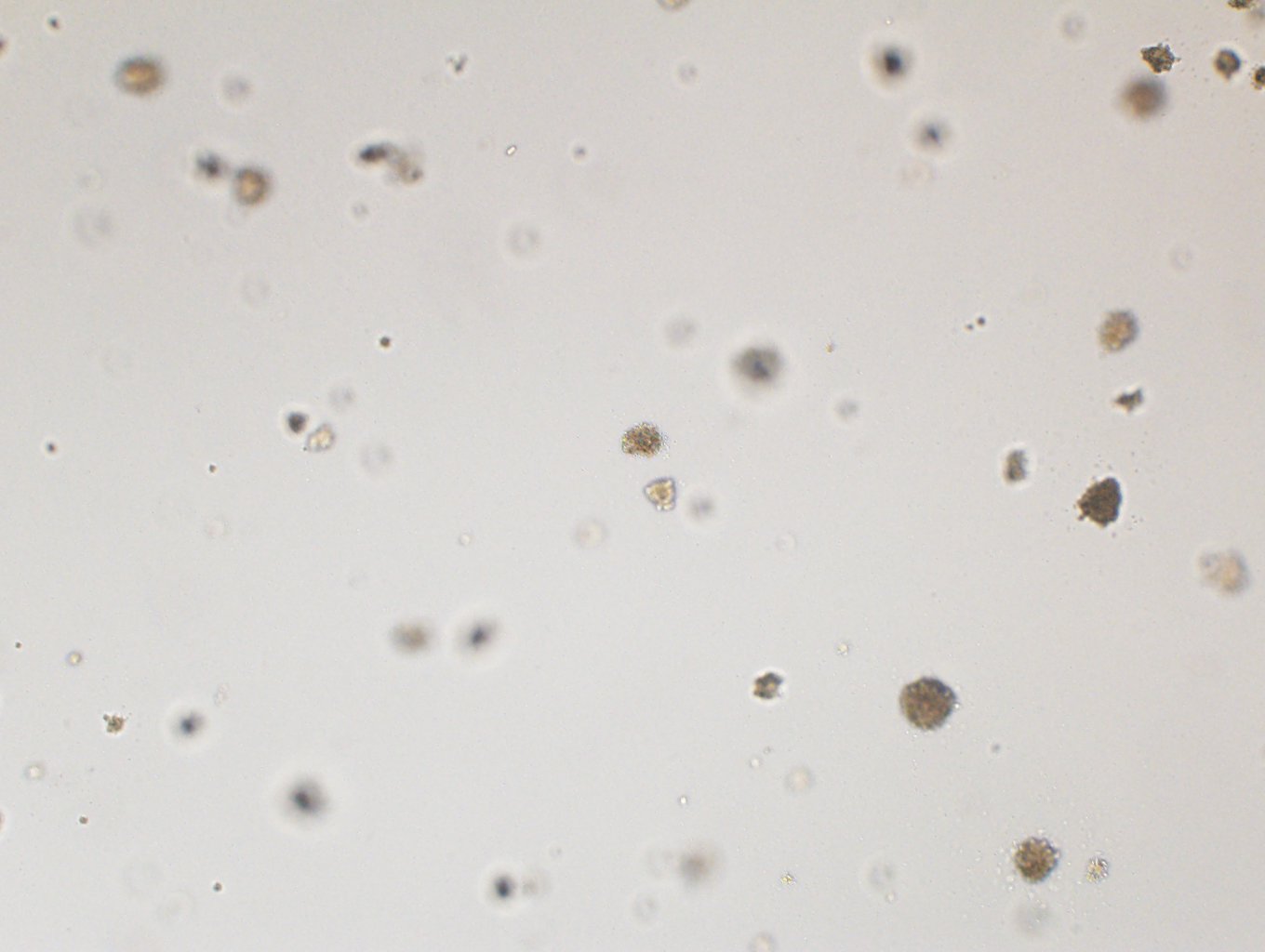

Supplement: Supplementary file 6 — Source data Fig. 5 [file 44318_2025_363_MOESM6_ESM.zip › Figure 5/5I/Ephrin A1 (2).jpg]

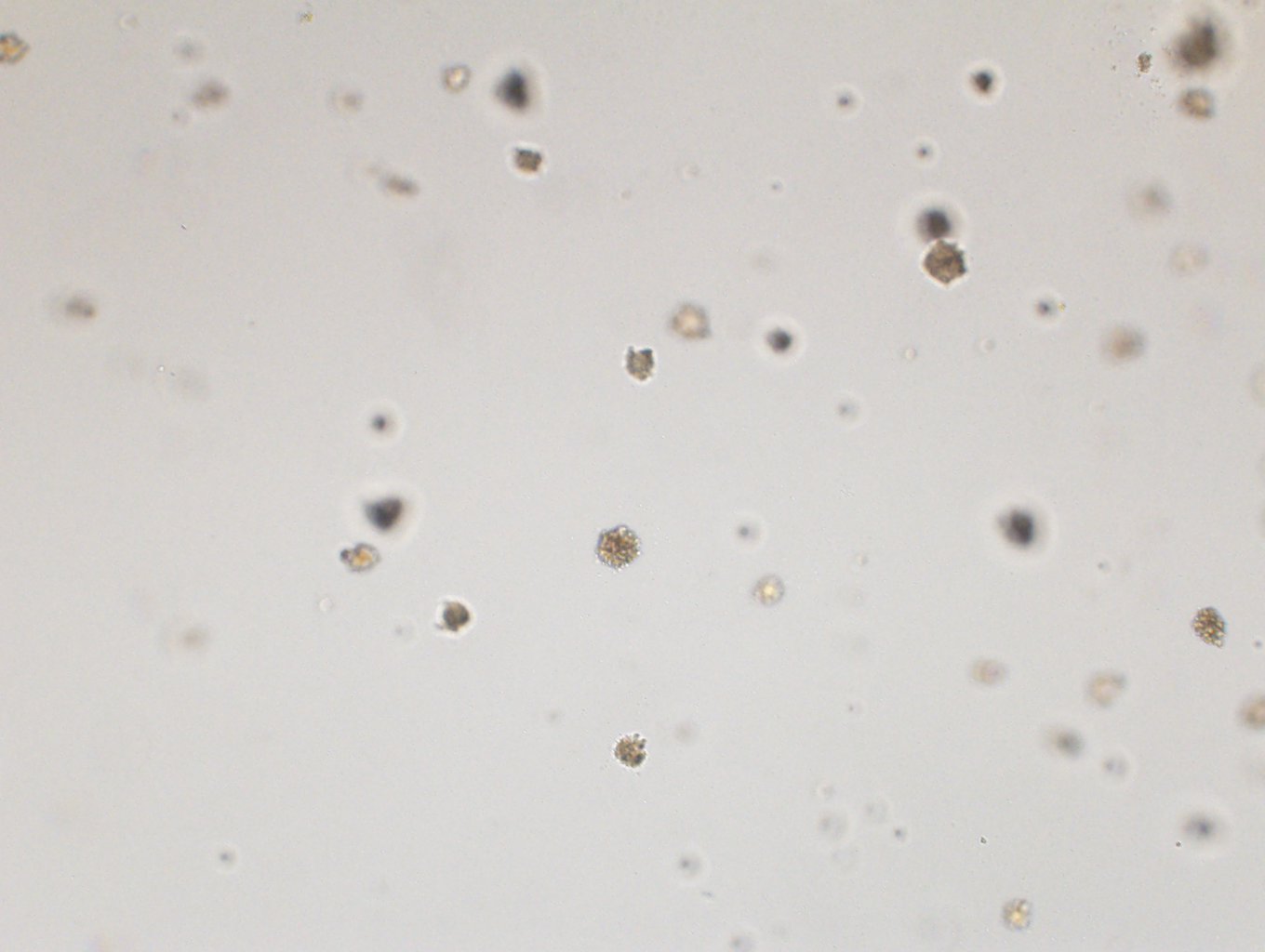

Supplement: Supplementary file 6 — Source data Fig. 5 [file 44318_2025_363_MOESM6_ESM.zip › Figure 5/5I/Ephrin A1 (3).jpg]

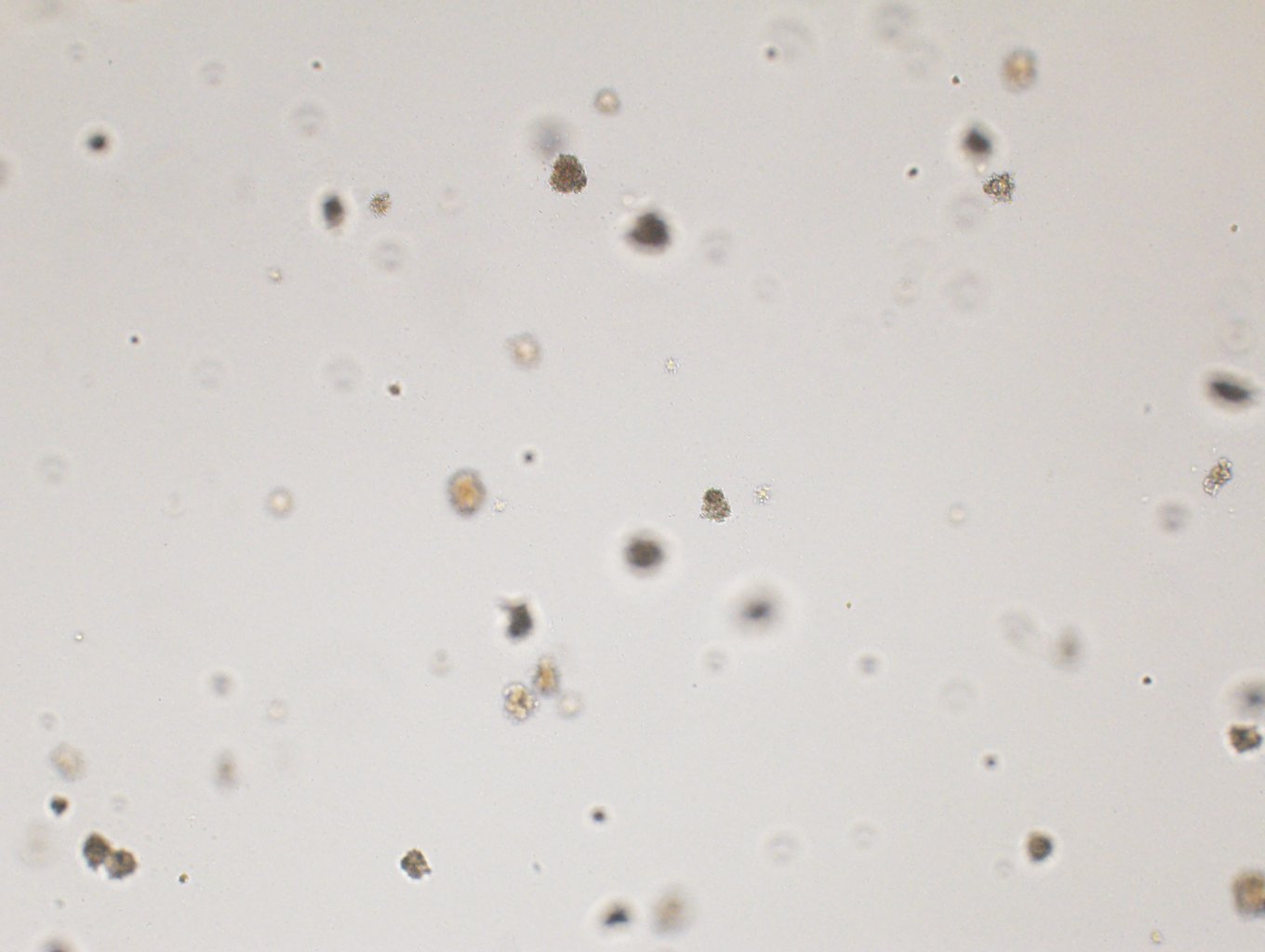

Supplement: Supplementary file 6 — Source data Fig. 5 [file 44318_2025_363_MOESM6_ESM.zip › Figure 5/5I/Ephrin A1 (4).jpg]

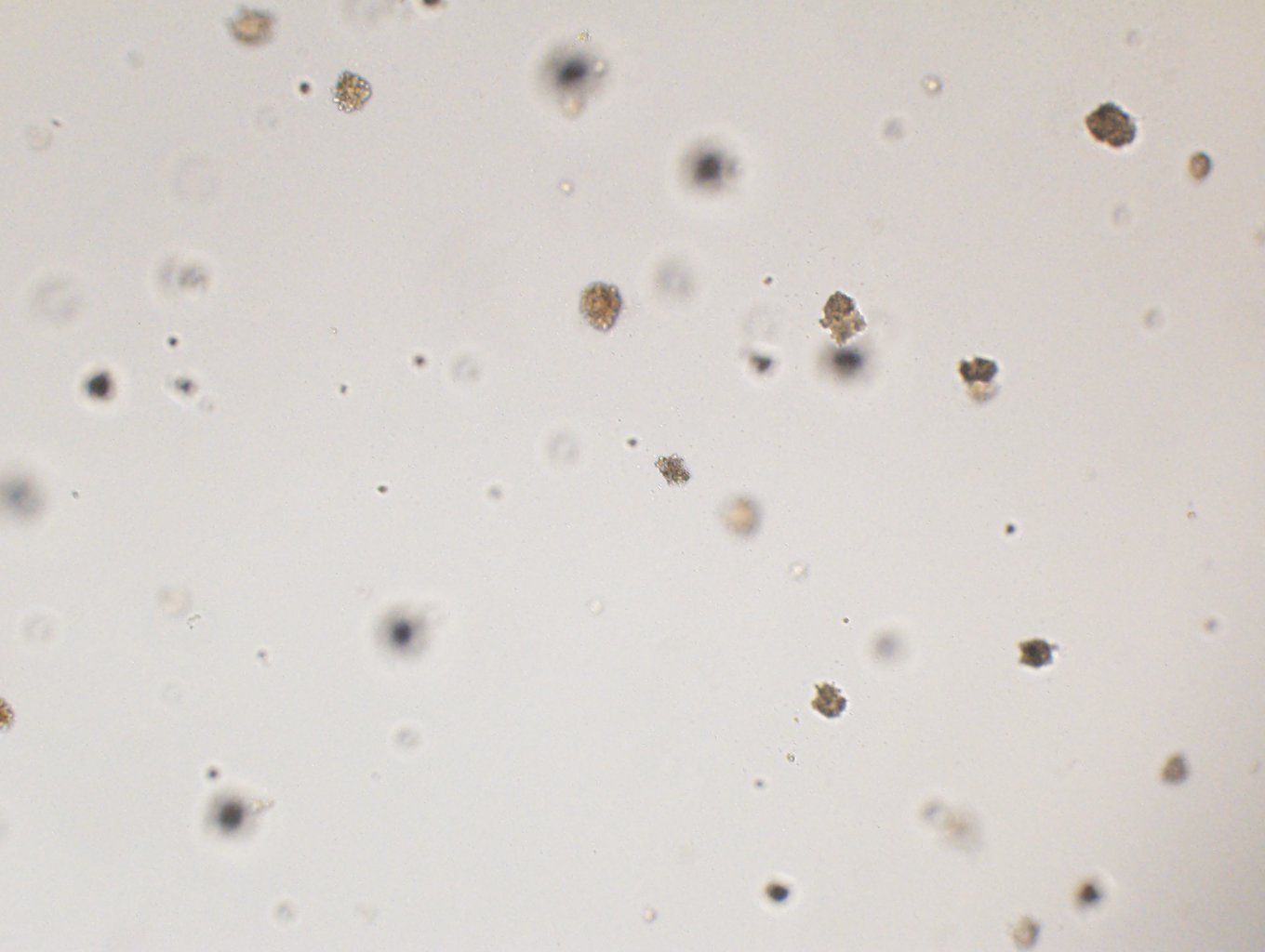

Supplement: Supplementary file 6 — Source data Fig. 5 [file 44318_2025_363_MOESM6_ESM.zip › Figure 5/5I/Ephrin A1 (5).jpg]

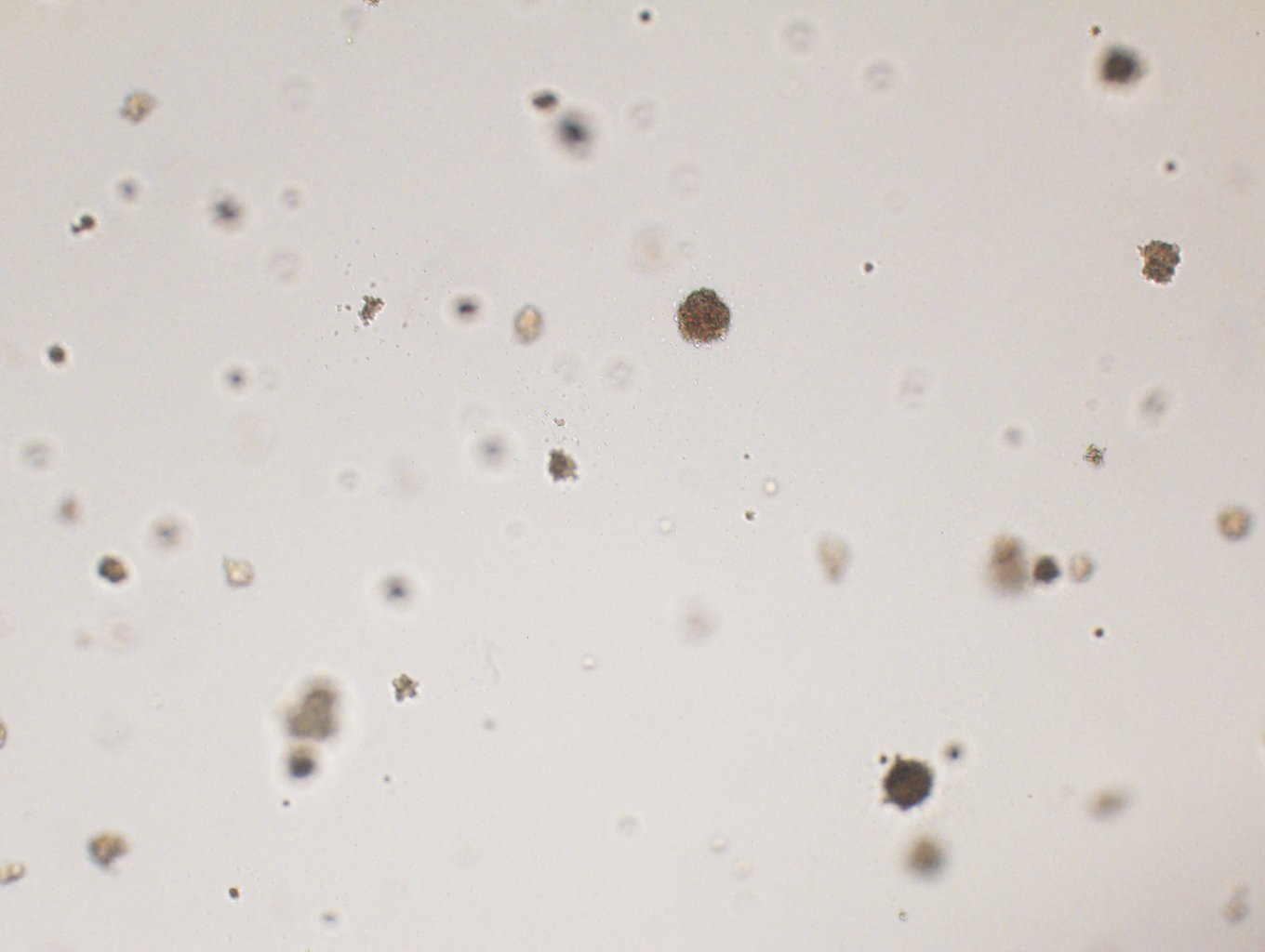

Supplement: Supplementary file 6 — Source data Fig. 5 [file 44318_2025_363_MOESM6_ESM.zip › Figure 5/5I/Ephrin A1 (6).jpg]

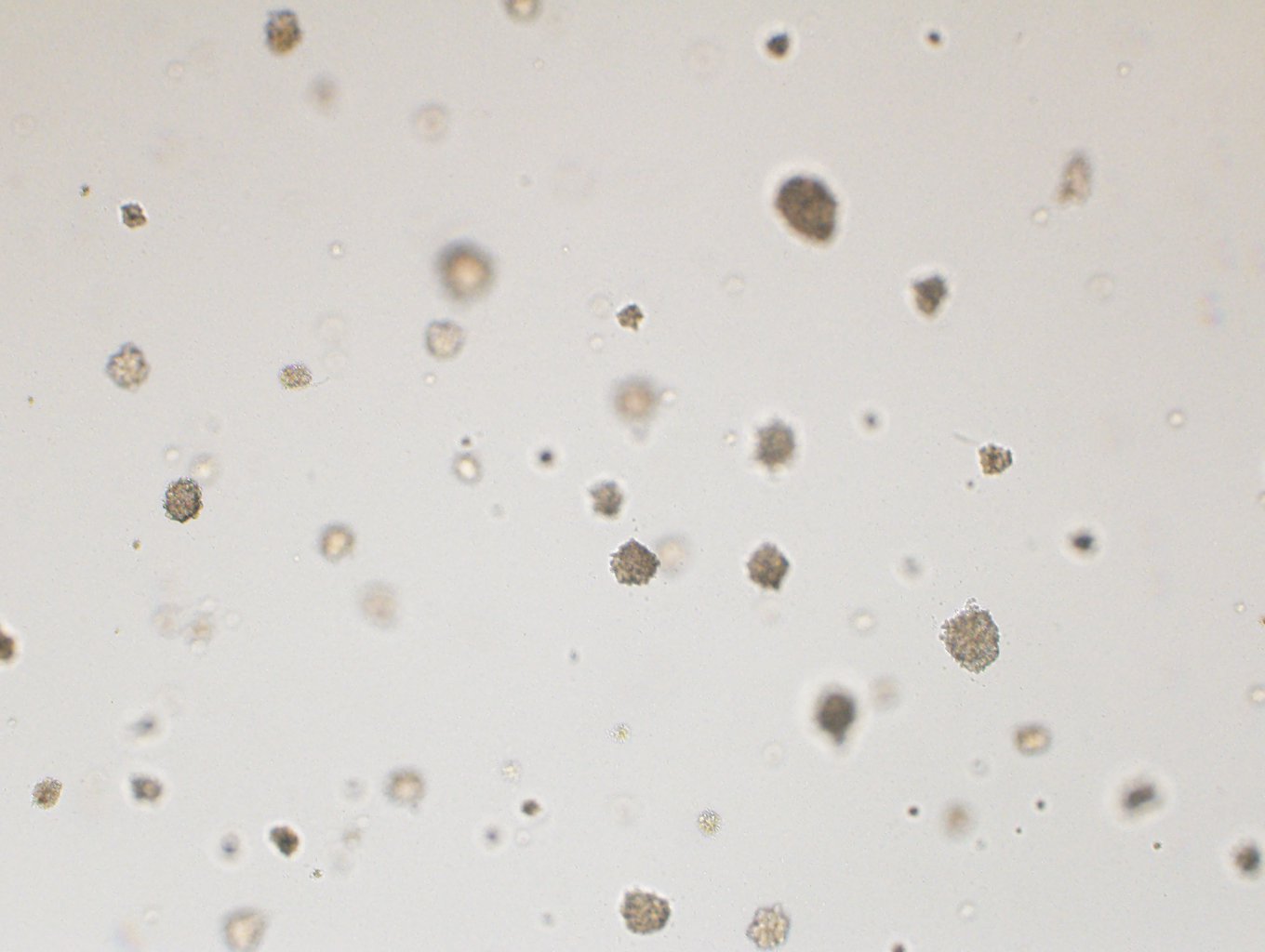

Supplement: Supplementary file 6 — Source data Fig. 5 [file 44318_2025_363_MOESM6_ESM.zip › Figure 5/5I/Ephrin A1 (7).jpg]

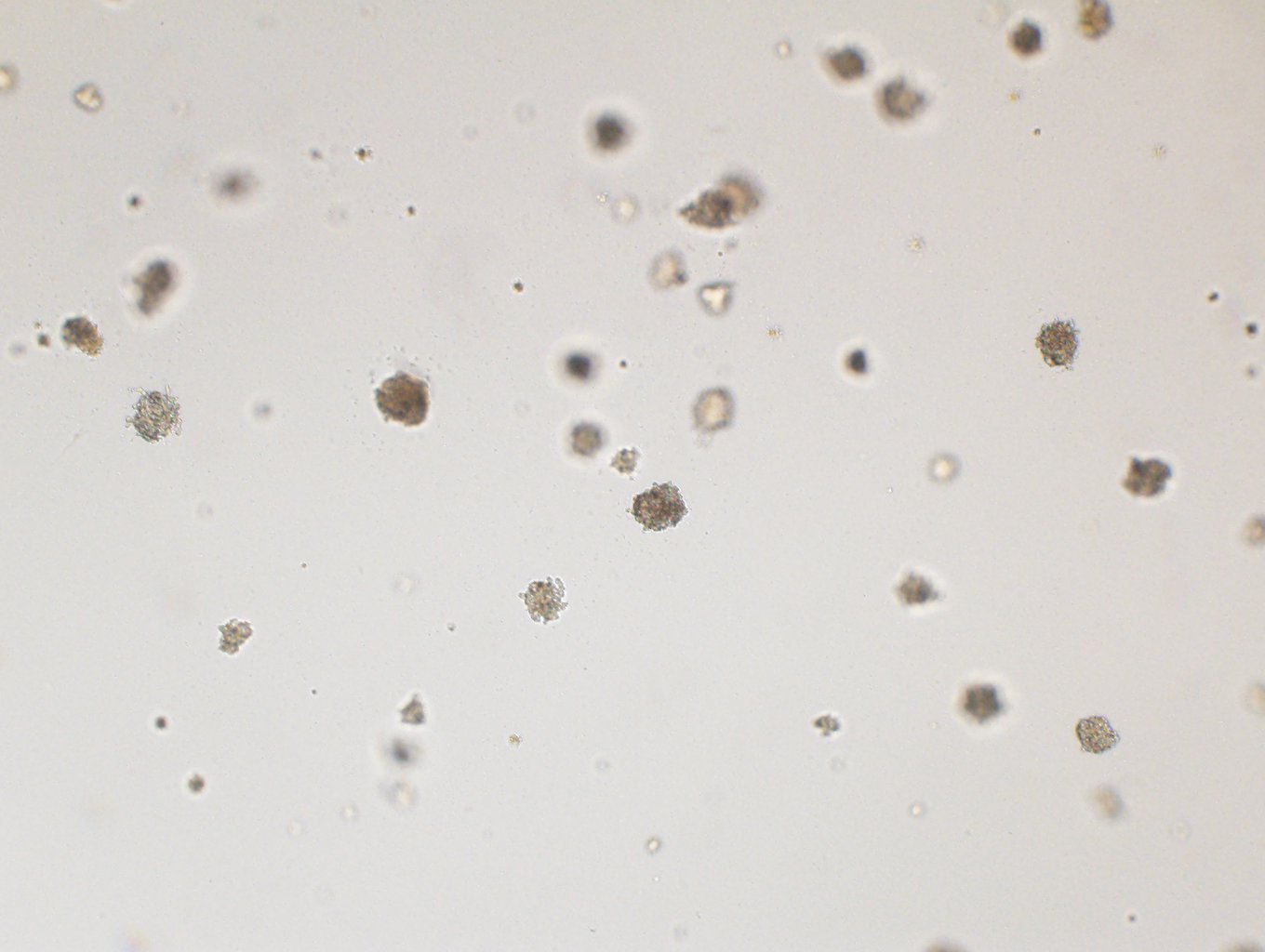

Supplement: Supplementary file 6 — Source data Fig. 5 [file 44318_2025_363_MOESM6_ESM.zip › Figure 5/5I/Ephrin A1 (8)-displayed in 5I.jpg]

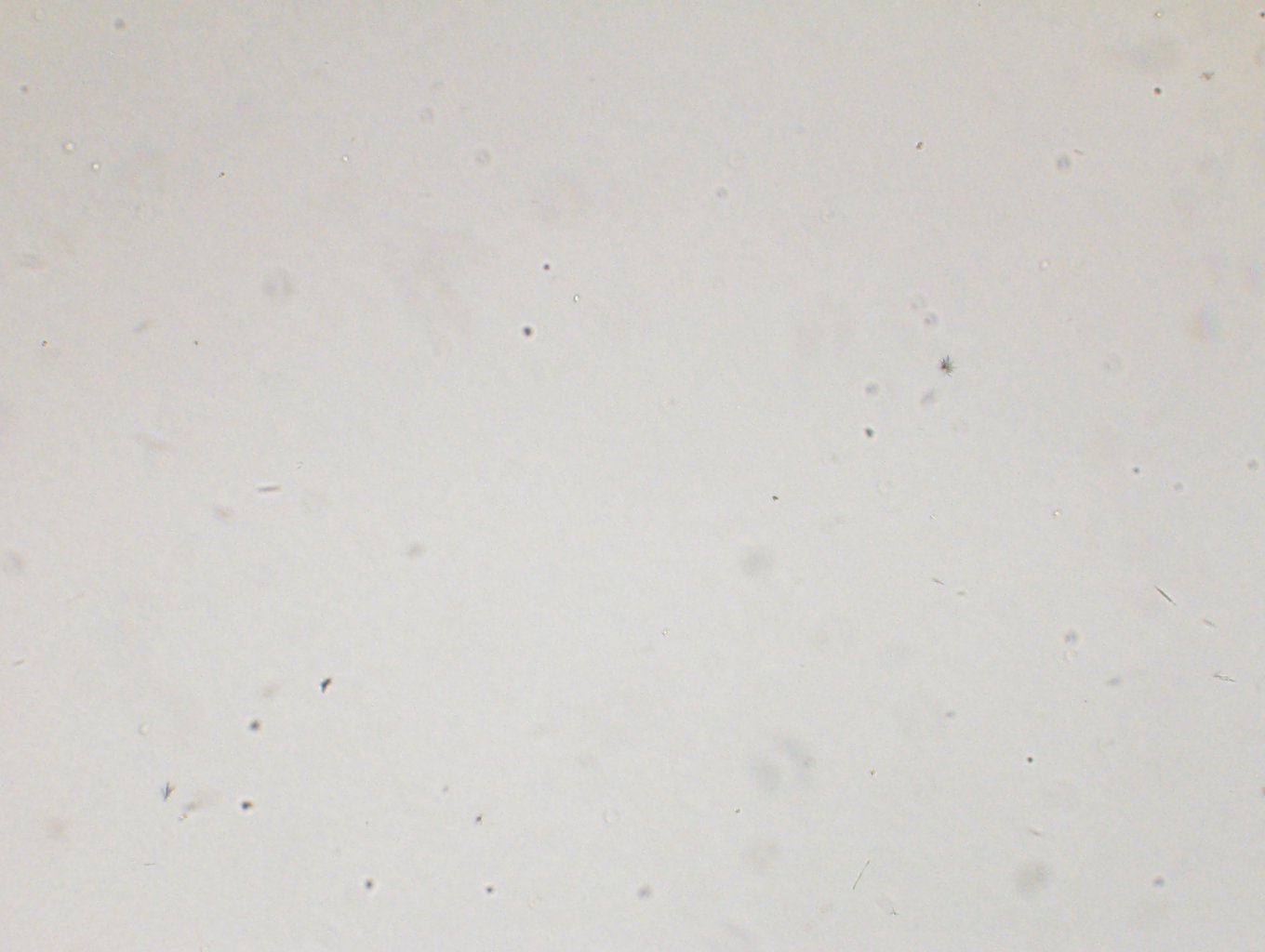

Supplement: Supplementary file 6 — Source data Fig. 5 [file 44318_2025_363_MOESM6_ESM.zip › Figure 5/5I/Ephrin A1+10um (1).jpg]

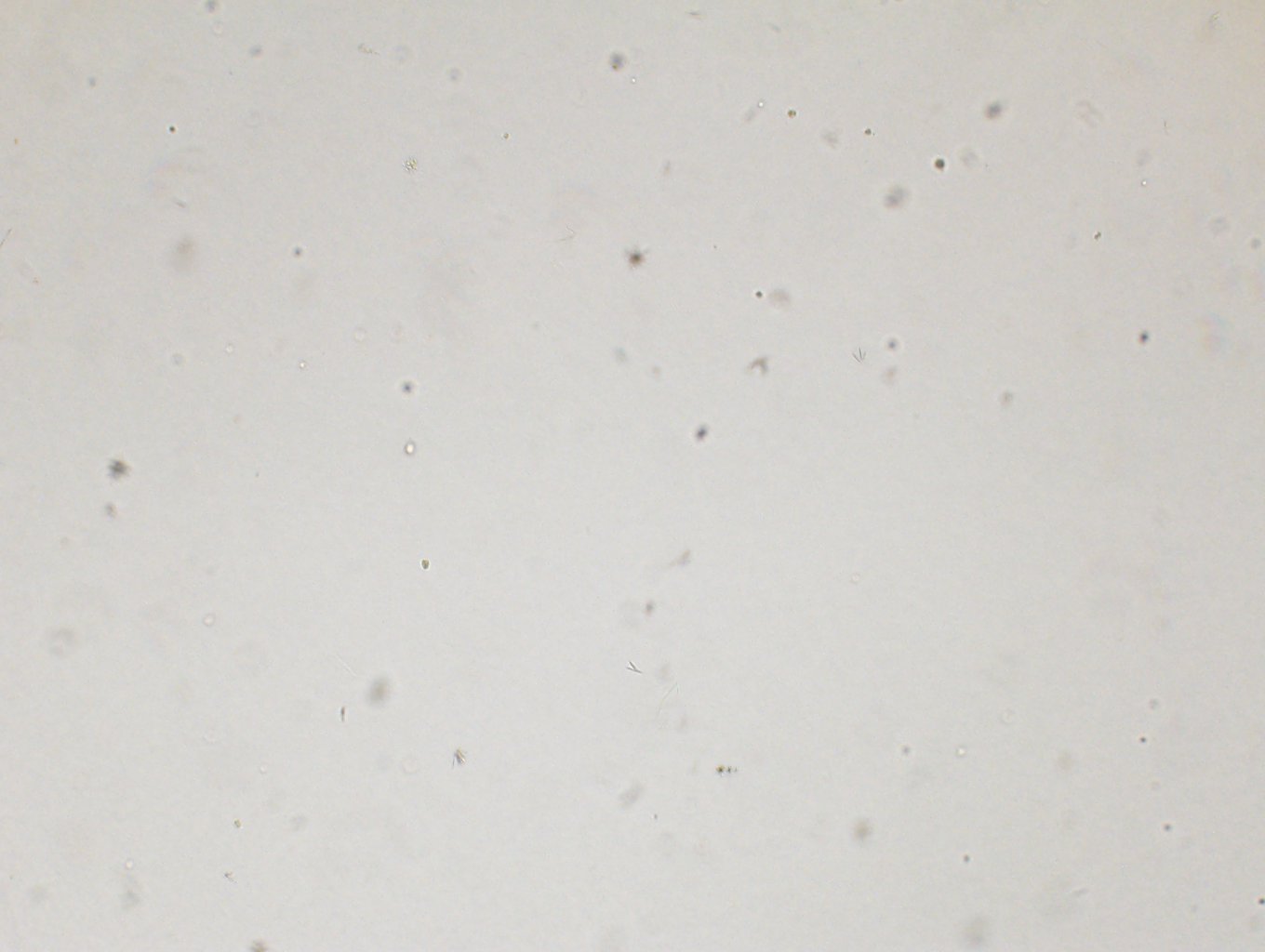

Supplement: Supplementary file 6 — Source data Fig. 5 [file 44318_2025_363_MOESM6_ESM.zip › Figure 5/5I/Ephrin A1+10um (2).jpg]

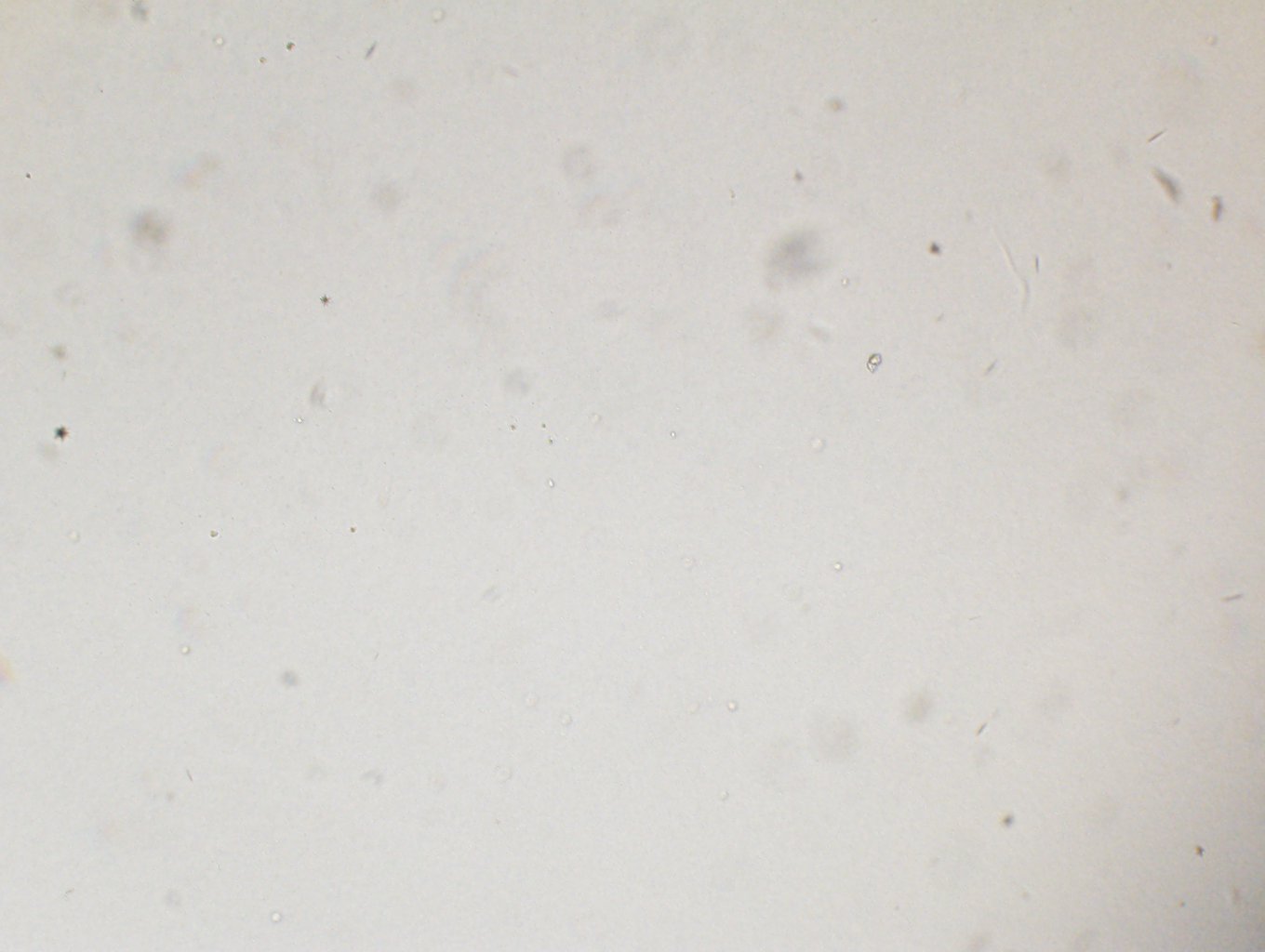

Supplement: Supplementary file 6 — Source data Fig. 5 [file 44318_2025_363_MOESM6_ESM.zip › Figure 5/5I/Ephrin A1+10um (3).jpg]

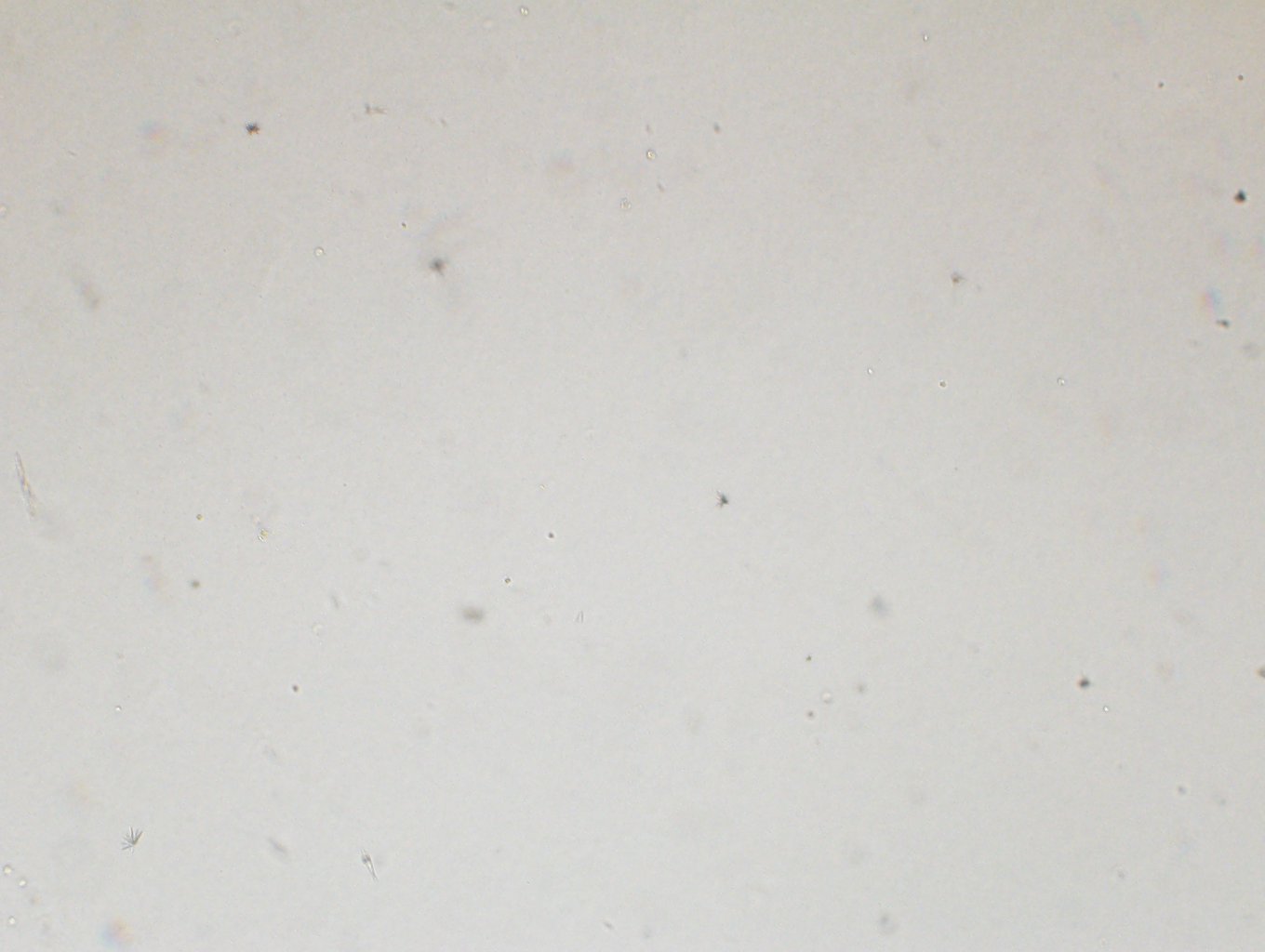

Supplement: Supplementary file 6 — Source data Fig. 5 [file 44318_2025_363_MOESM6_ESM.zip › Figure 5/5I/Ephrin A1+10um (4).jpg]

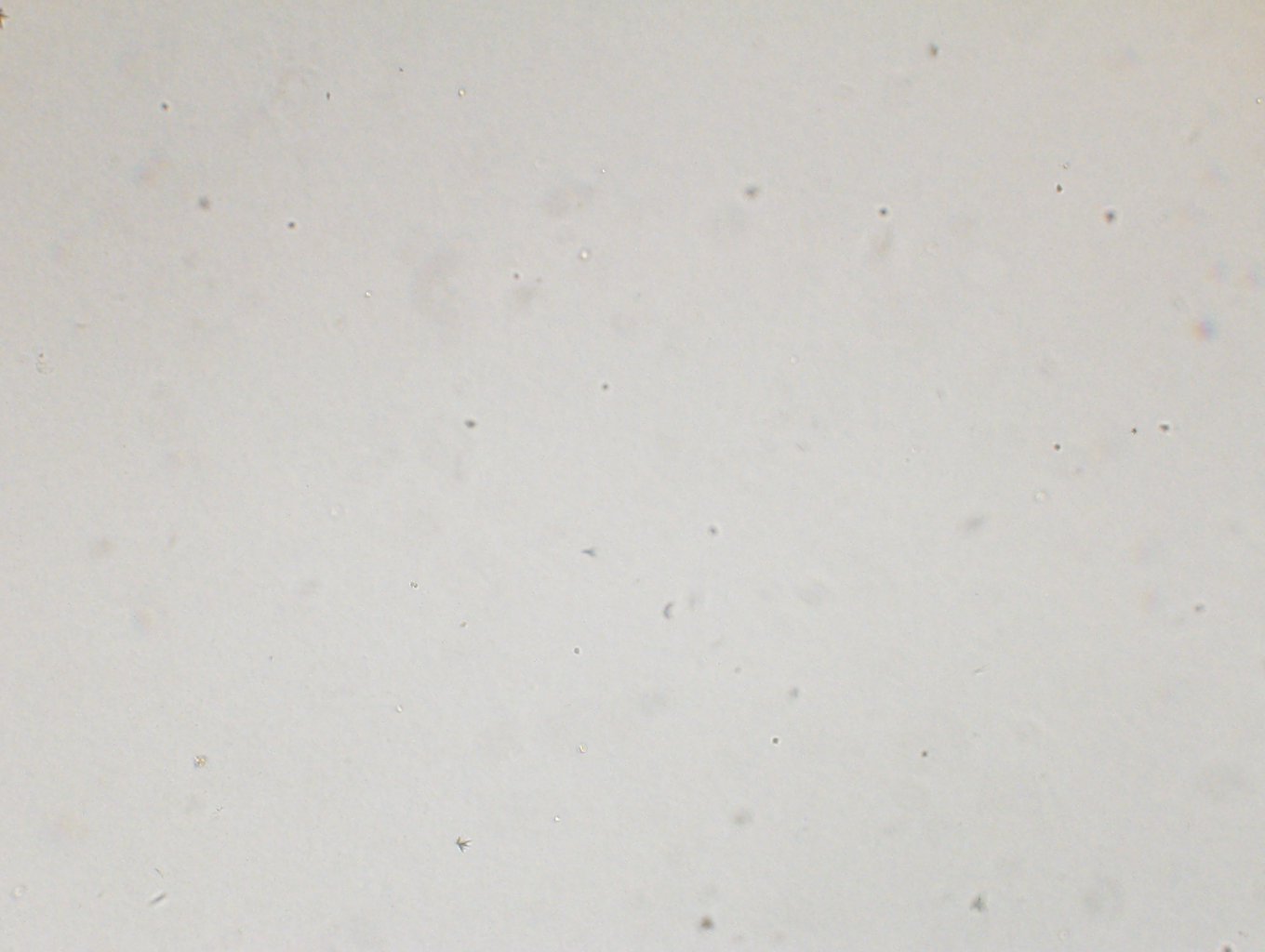

Supplement: Supplementary file 6 — Source data Fig. 5 [file 44318_2025_363_MOESM6_ESM.zip › Figure 5/5I/Ephrin A1+10um (5).jpg]

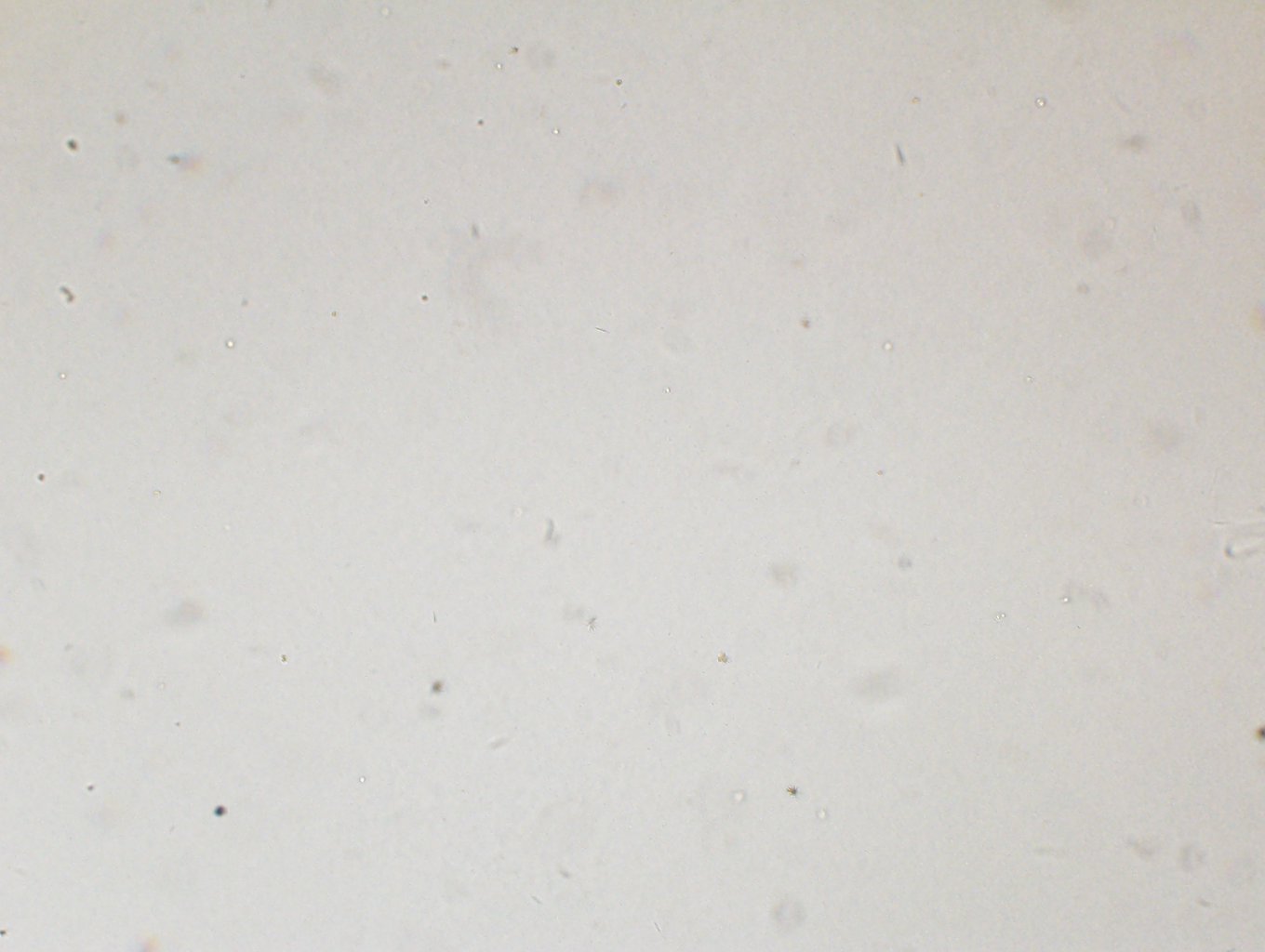

Supplement: Supplementary file 6 — Source data Fig. 5 [file 44318_2025_363_MOESM6_ESM.zip › Figure 5/5I/Ephrin A1+10um (6).jpg]

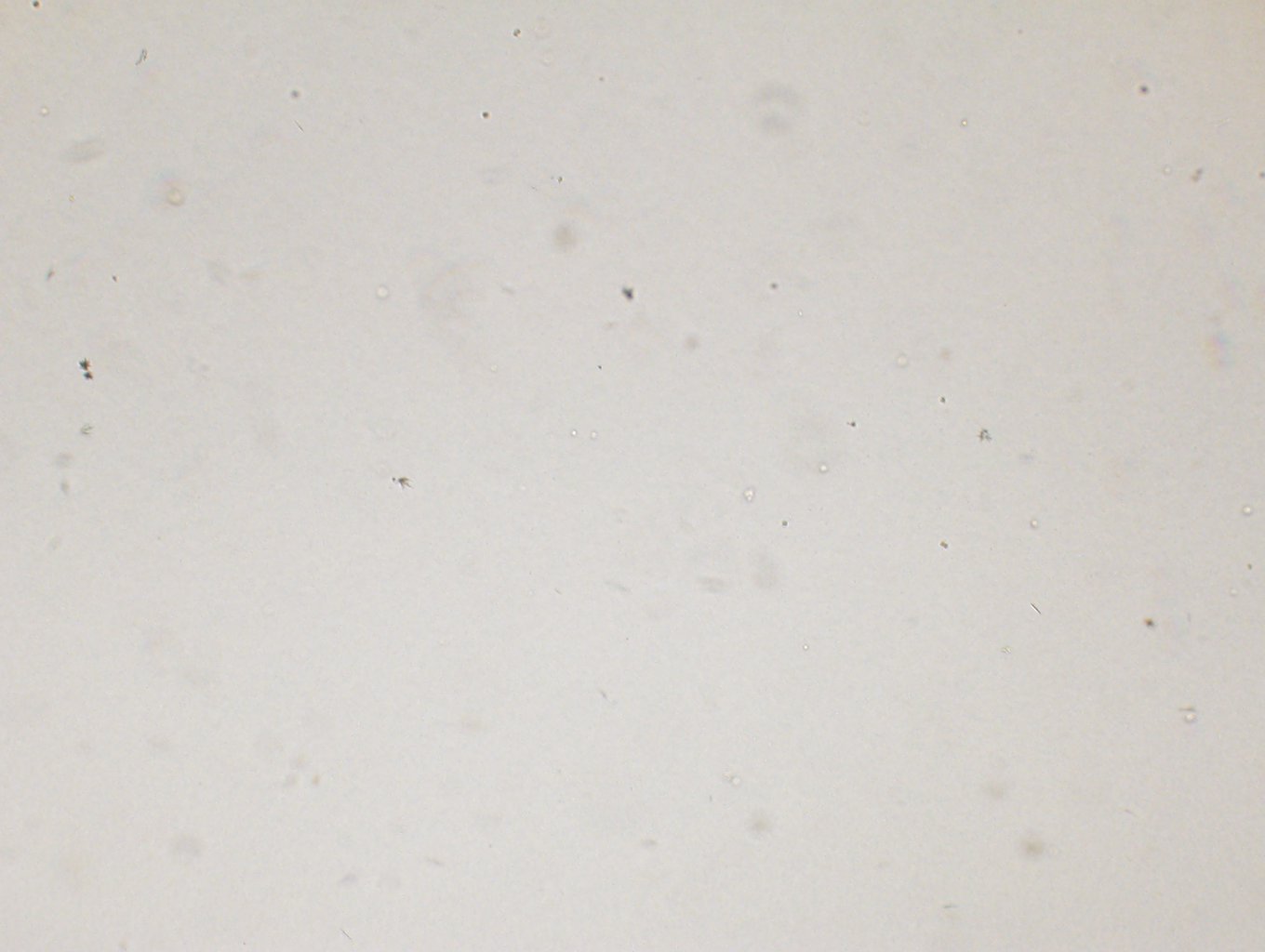

Supplement: Supplementary file 6 — Source data Fig. 5 [file 44318_2025_363_MOESM6_ESM.zip › Figure 5/5I/Ephrin A1+10um (7).jpg]

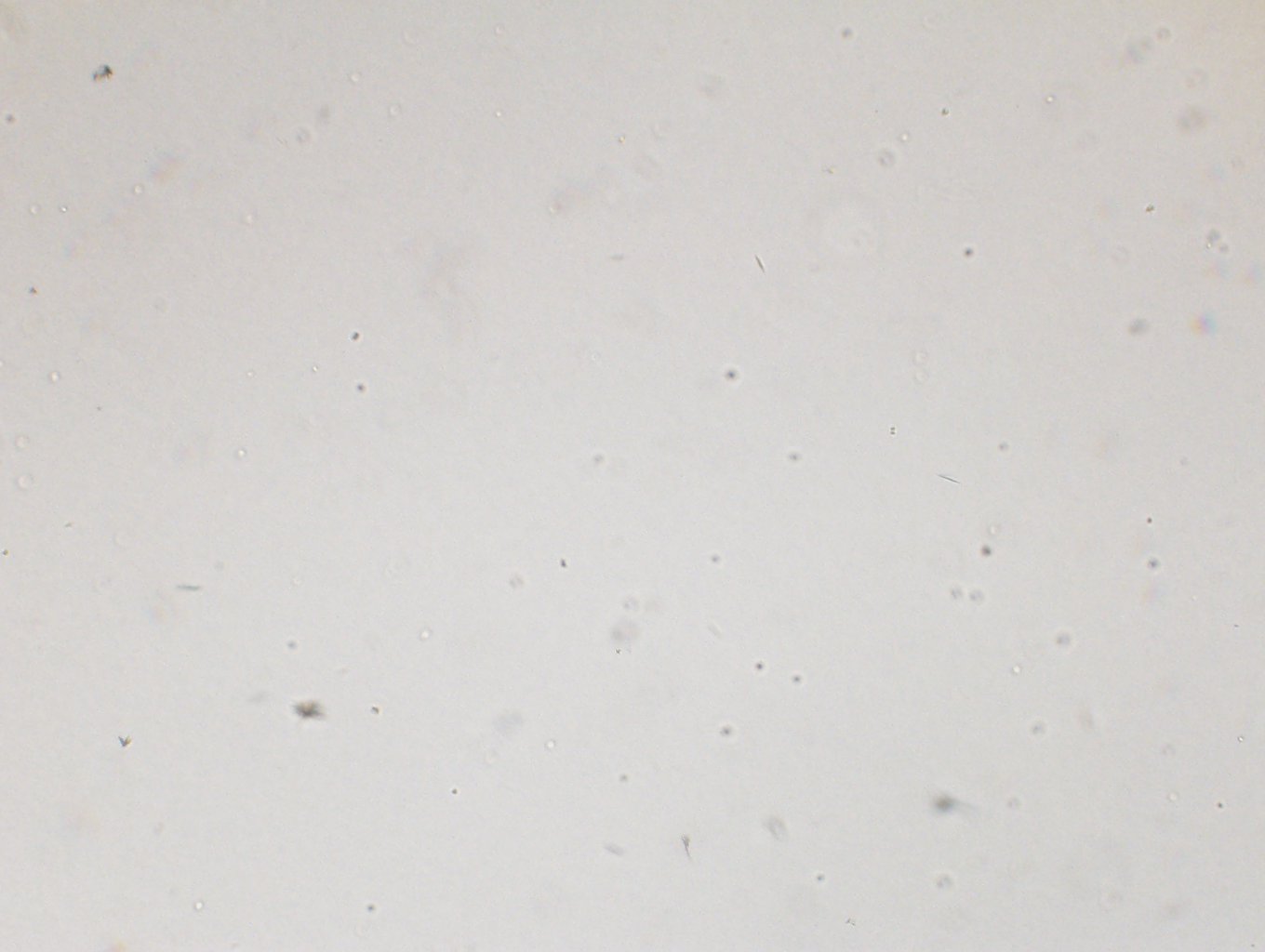

Supplement: Supplementary file 6 — Source data Fig. 5 [file 44318_2025_363_MOESM6_ESM.zip › Figure 5/5I/Ephrin A1+10um (8)-displayed in 5I.jpg]

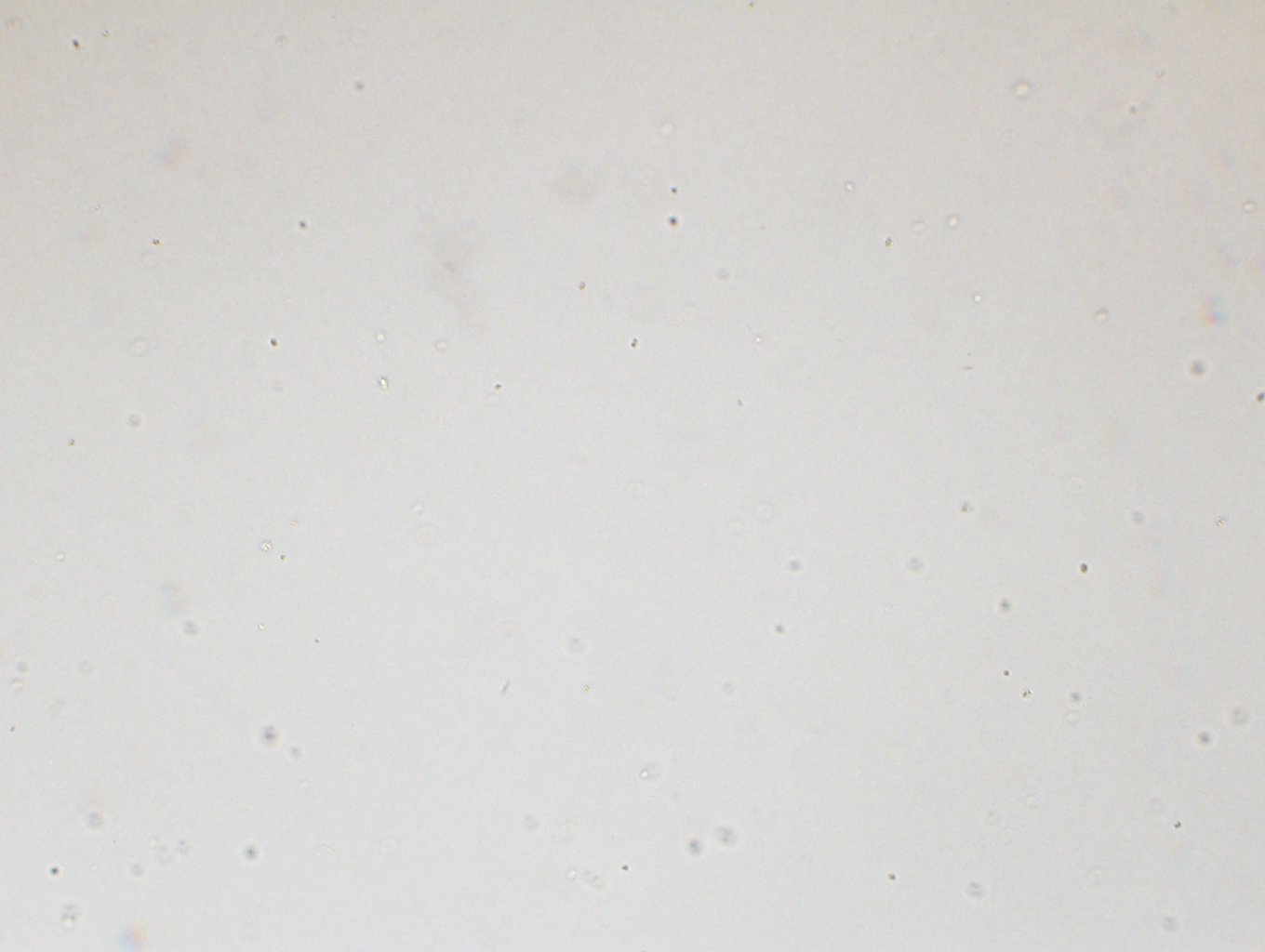

Supplement: Supplementary file 6 — Source data Fig. 5 [file 44318_2025_363_MOESM6_ESM.zip › Figure 5/5I/Ephrin A1+5um (1).jpg]

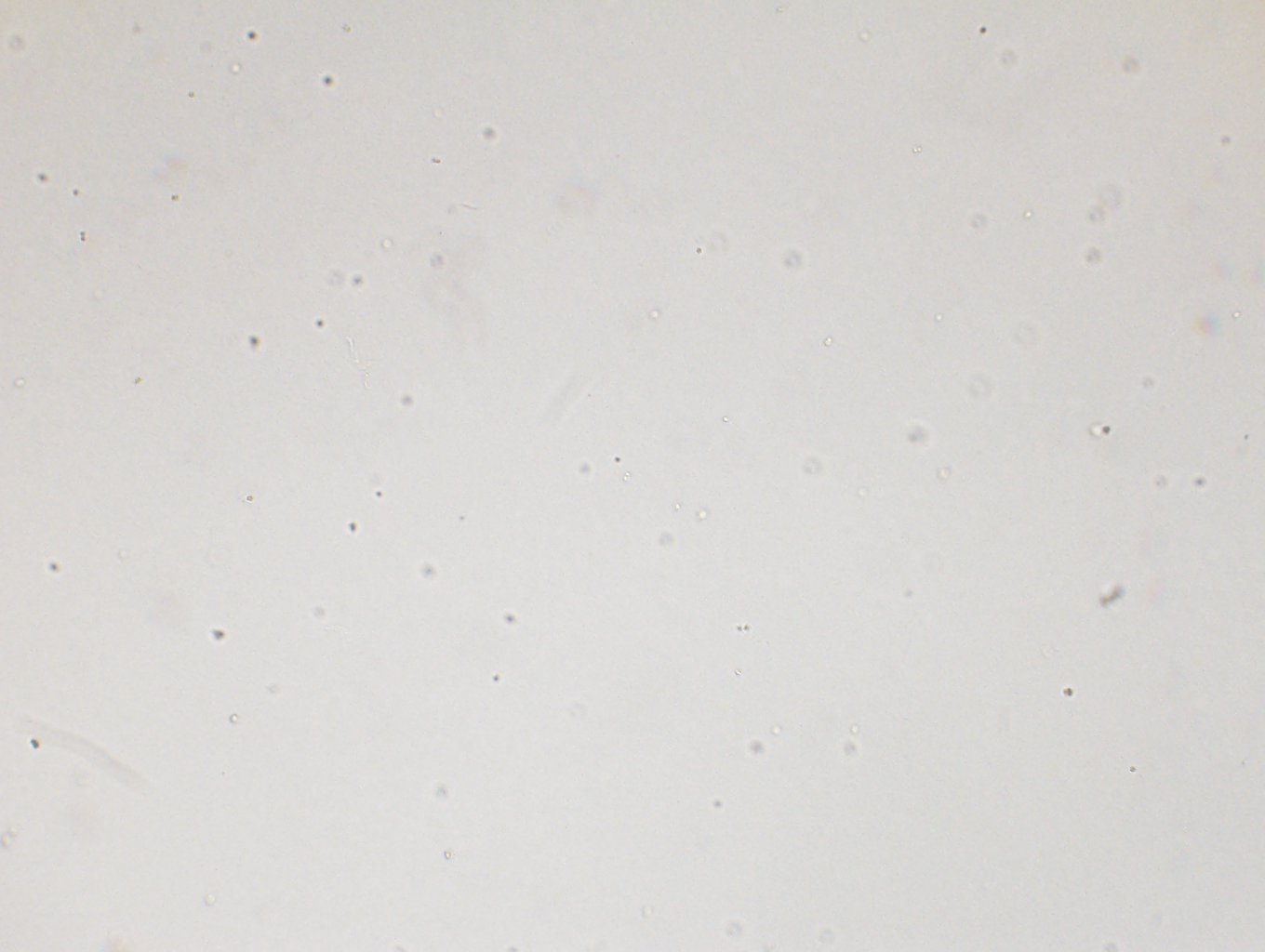

Supplement: Supplementary file 6 — Source data Fig. 5 [file 44318_2025_363_MOESM6_ESM.zip › Figure 5/5I/Ephrin A1+5um (2)-displayed in 5I.jpg]

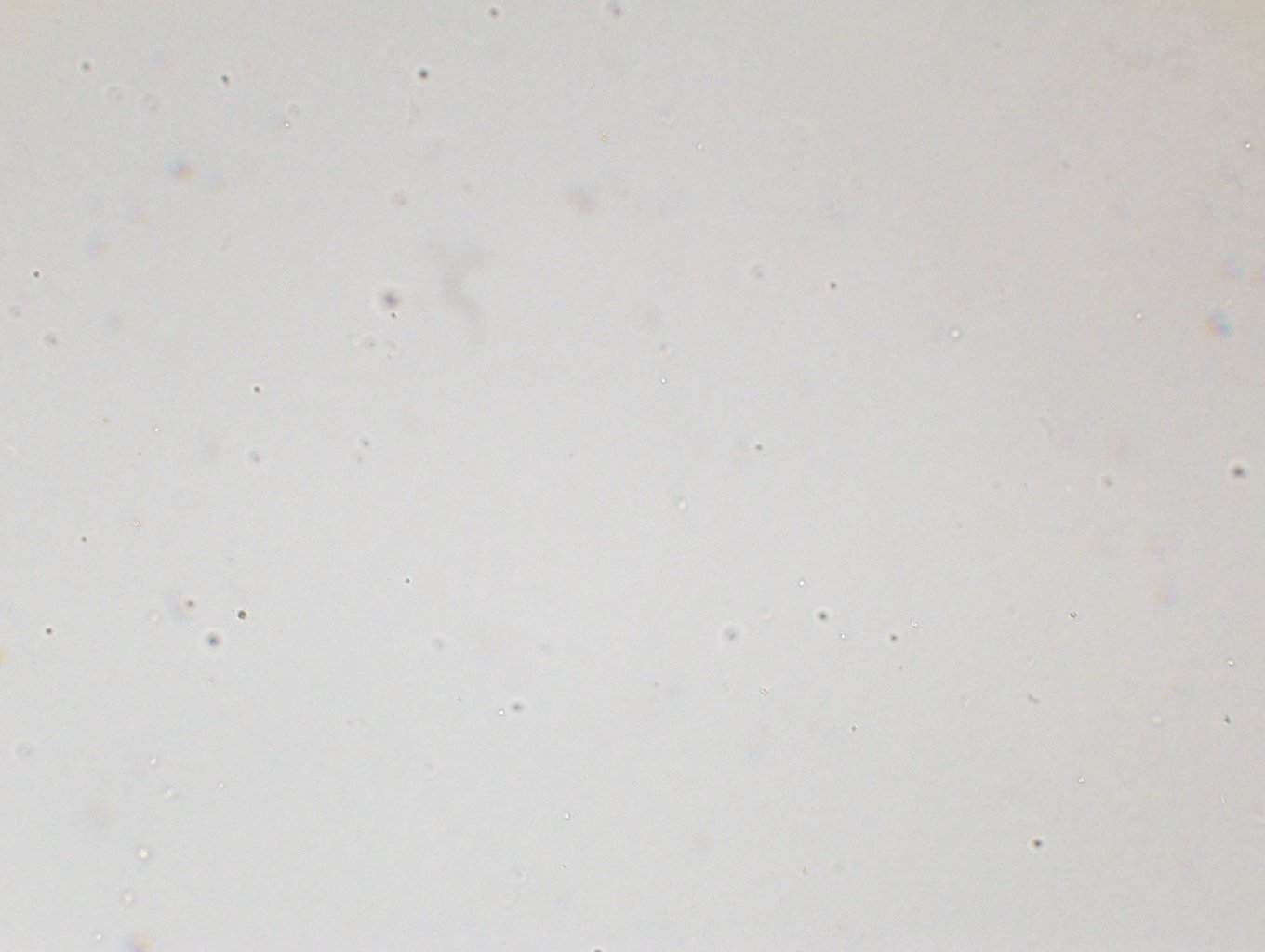

Supplement: Supplementary file 6 — Source data Fig. 5 [file 44318_2025_363_MOESM6_ESM.zip › Figure 5/5I/Ephrin A1+5um (3).jpg]

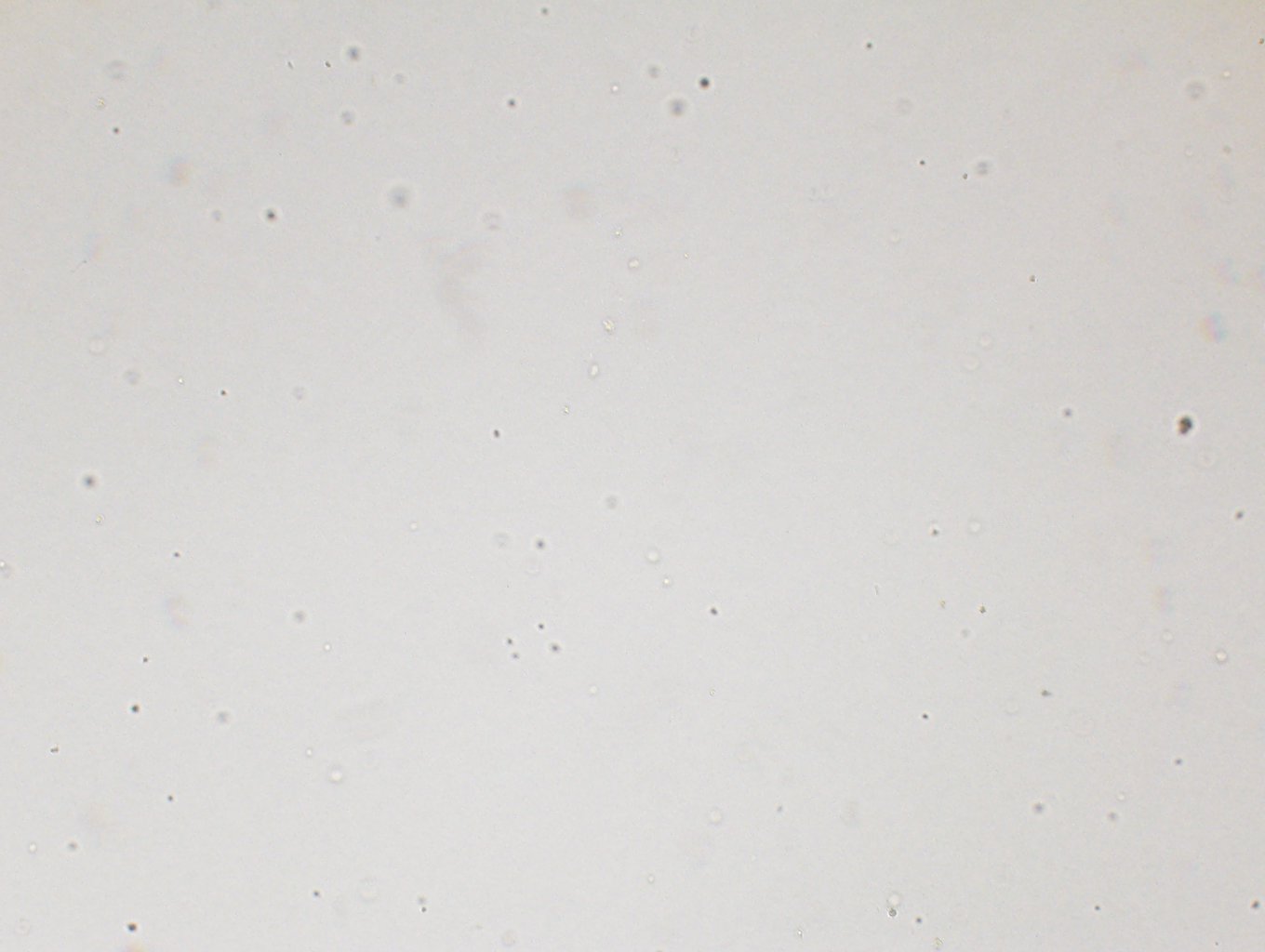

Supplement: Supplementary file 6 — Source data Fig. 5 [file 44318_2025_363_MOESM6_ESM.zip › Figure 5/5I/Ephrin A1+5um (4).jpg]

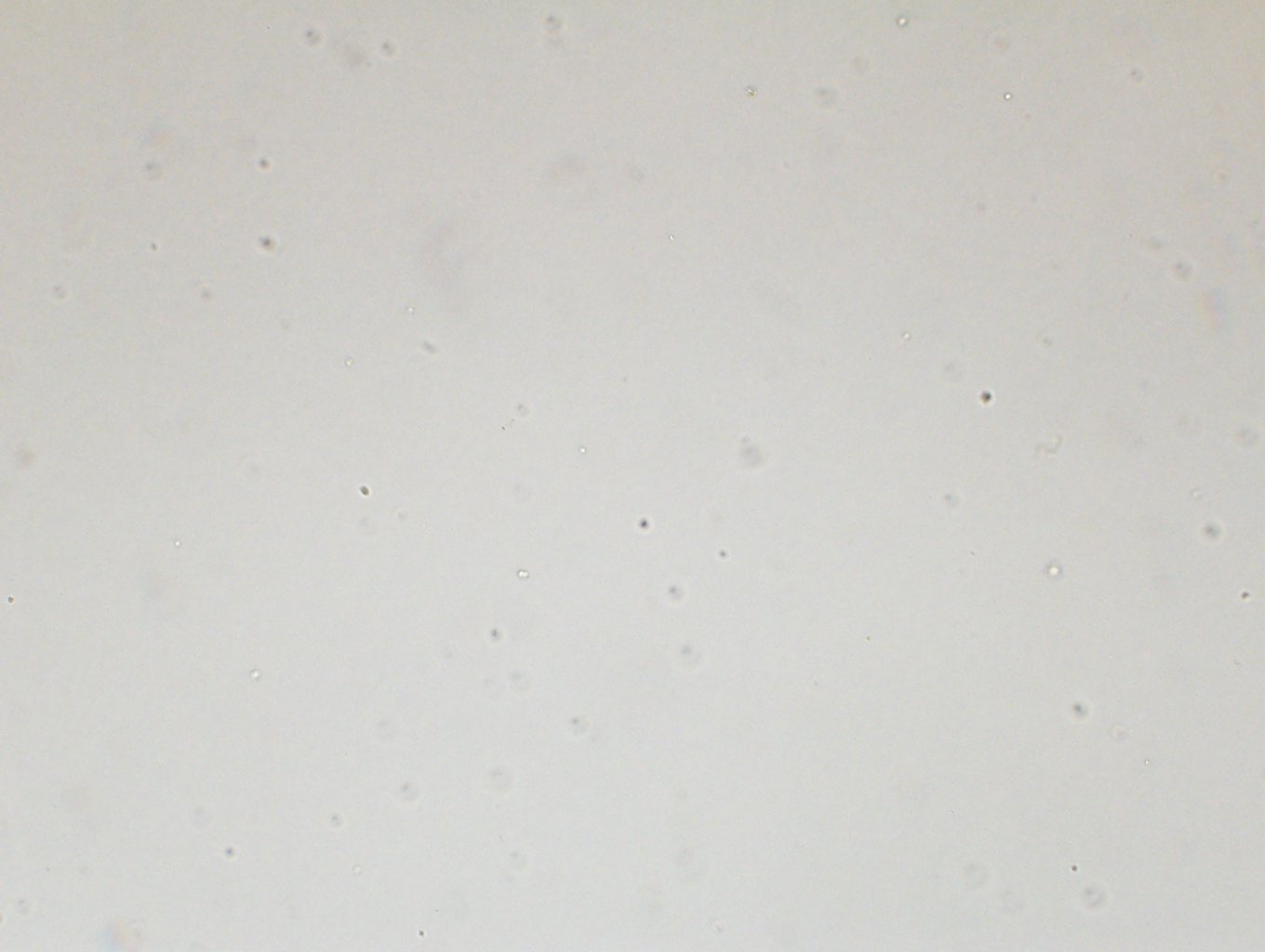

Supplement: Supplementary file 6 — Source data Fig. 5 [file 44318_2025_363_MOESM6_ESM.zip › Figure 5/5I/Ephrin A1+5um (5).jpg]

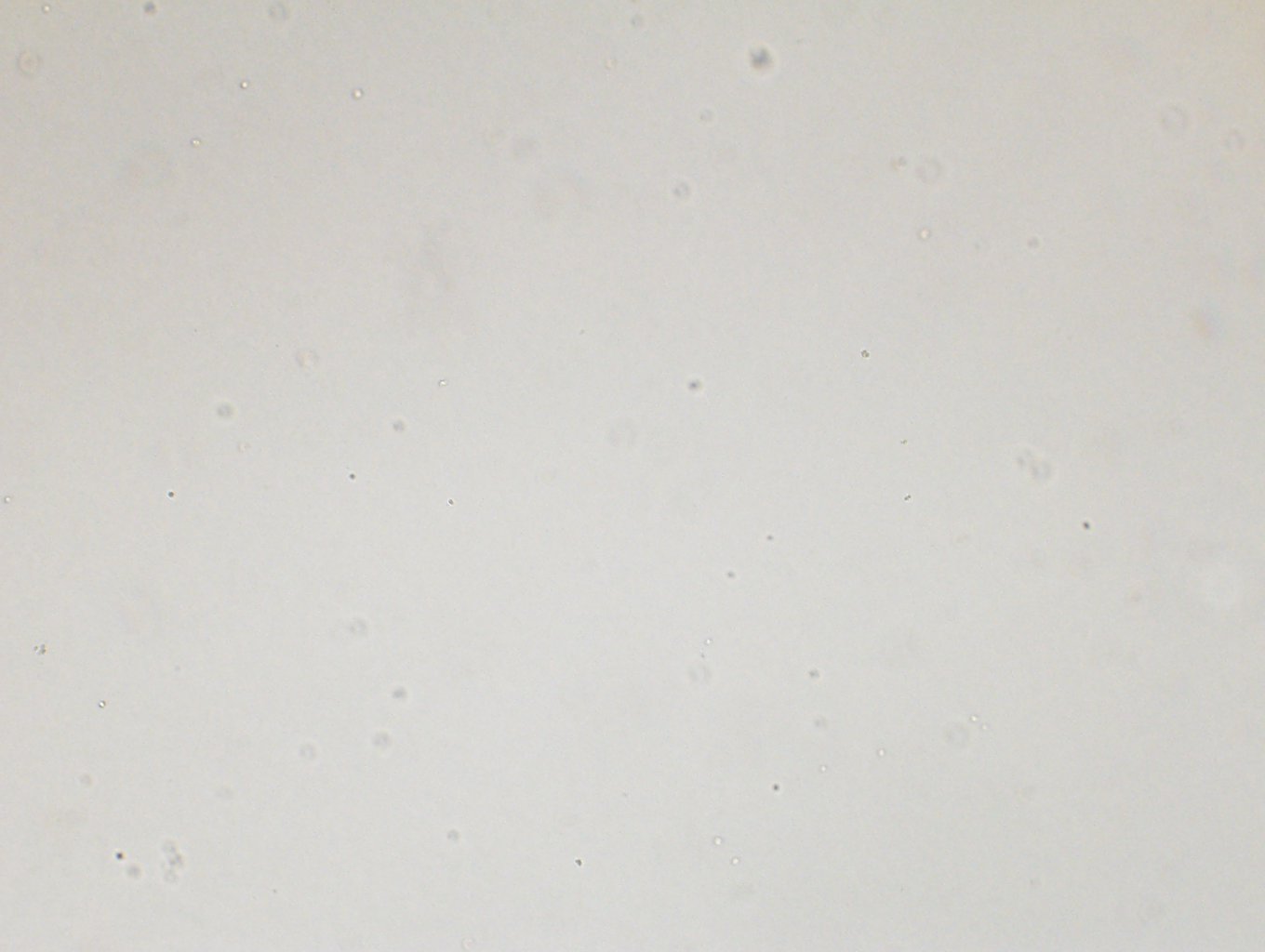

Supplement: Supplementary file 6 — Source data Fig. 5 [file 44318_2025_363_MOESM6_ESM.zip › Figure 5/5I/Ephrin A1+5um (6).jpg]

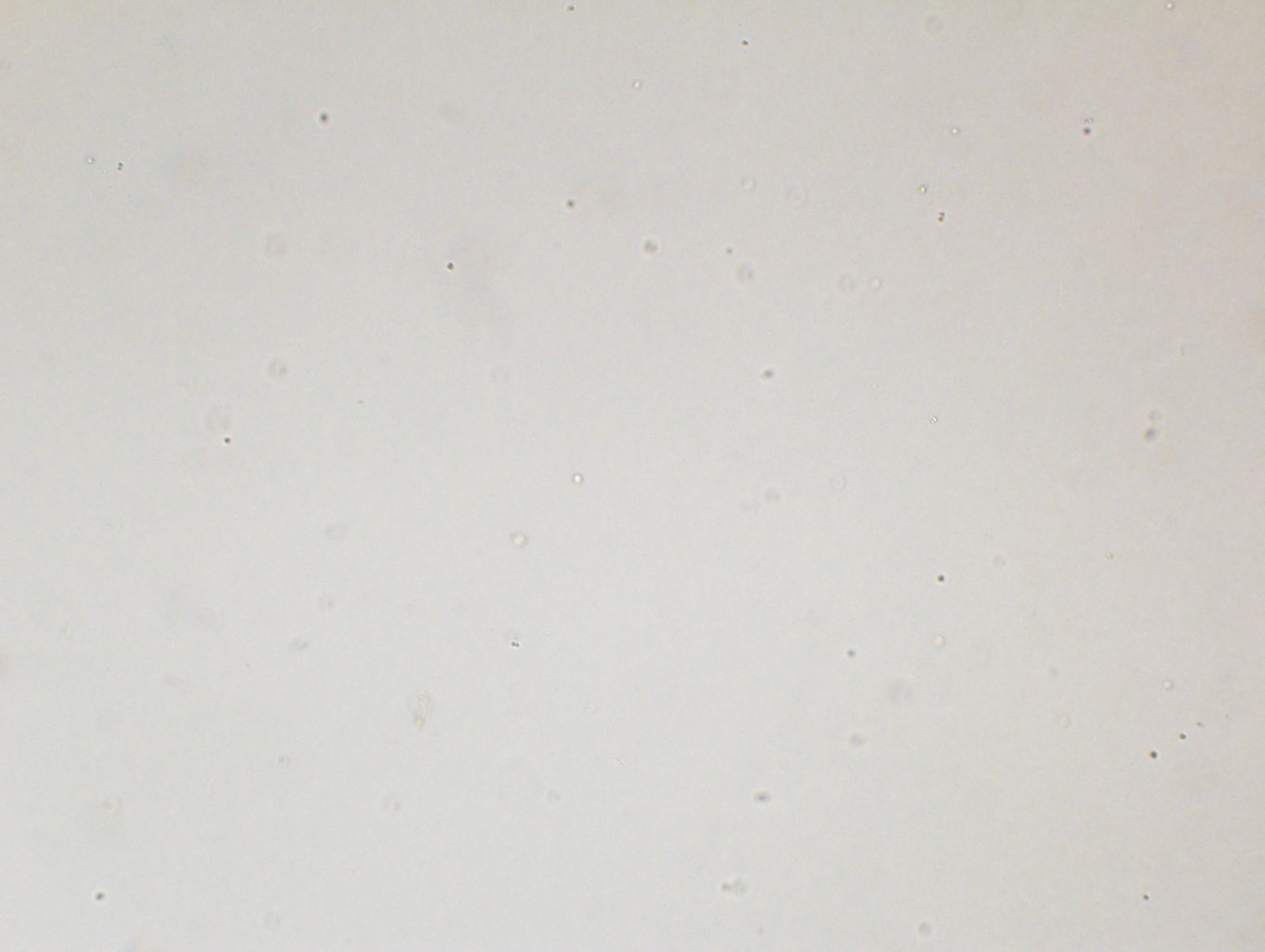

Supplement: Supplementary file 6 — Source data Fig. 5 [file 44318_2025_363_MOESM6_ESM.zip › Figure 5/5I/Ephrin A1+5um (7).jpg]

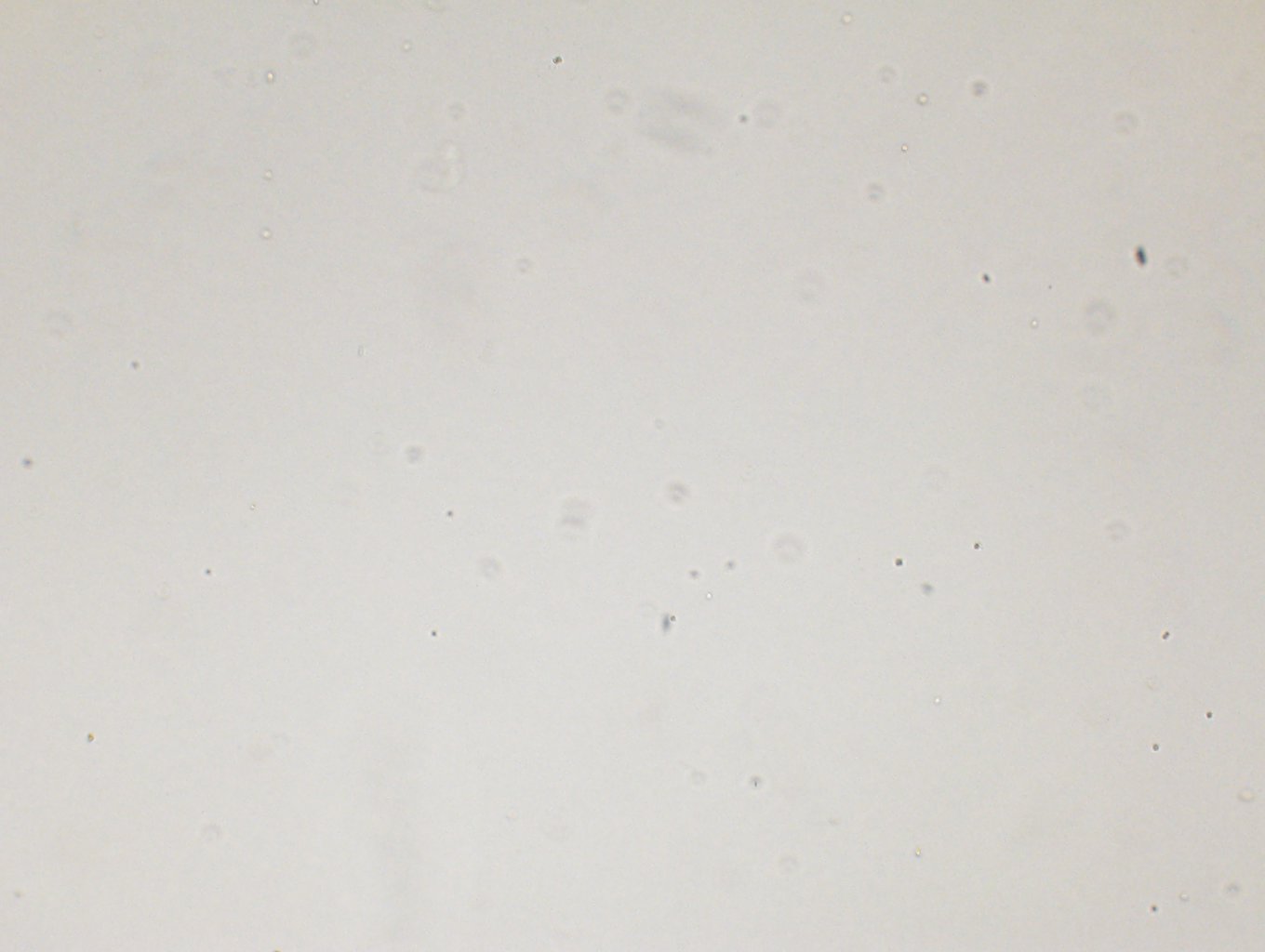

Supplement: Supplementary file 6 — Source data Fig. 5 [file 44318_2025_363_MOESM6_ESM.zip › Figure 5/5I/Ephrin A1+5um (8).jpg]

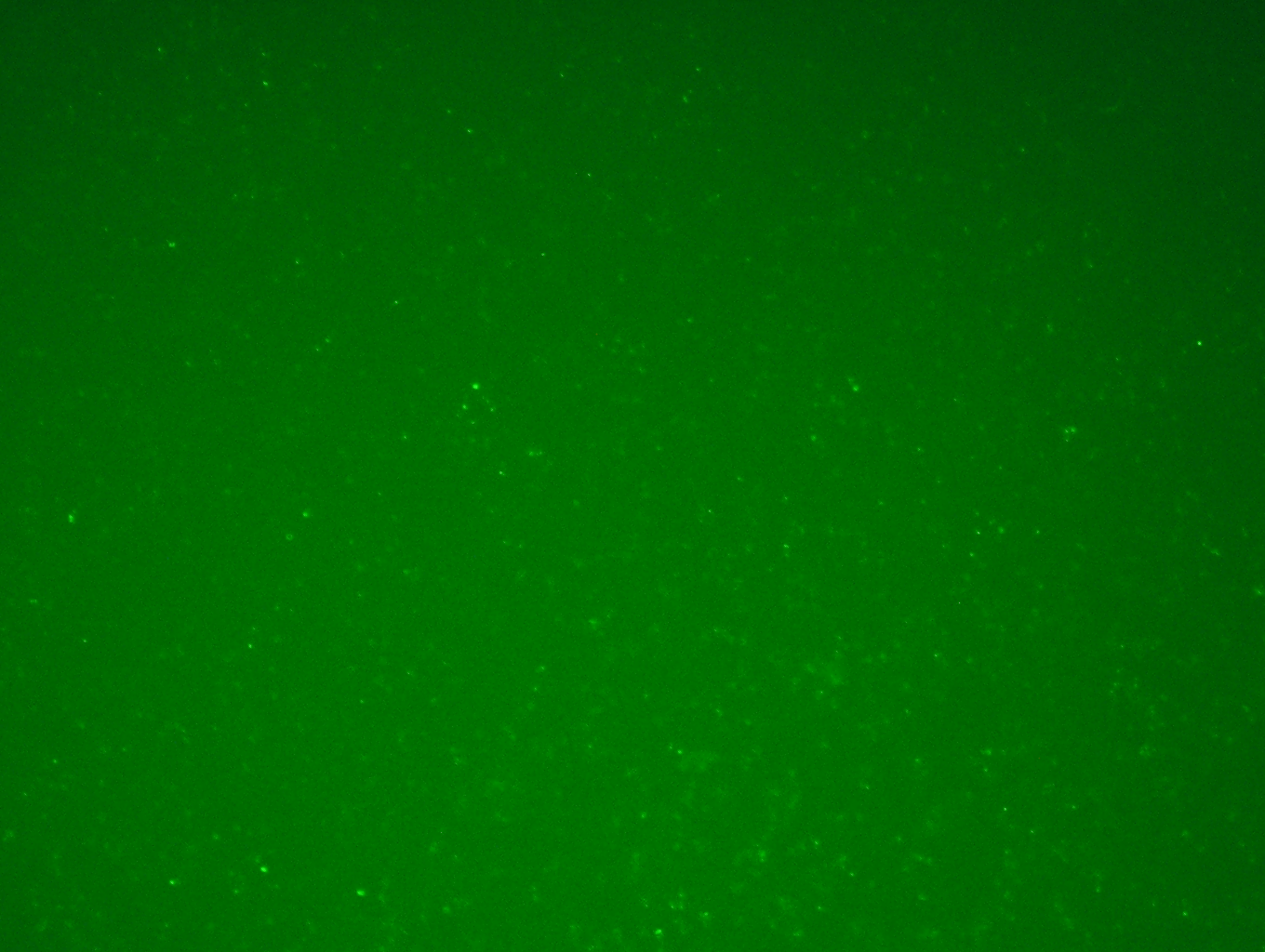

Supplement: Supplementary file 6 — Source data Fig. 5 [file 44318_2025_363_MOESM6_ESM.zip › Figure 5/5K/Control (1)-displayed in 5K.tif]

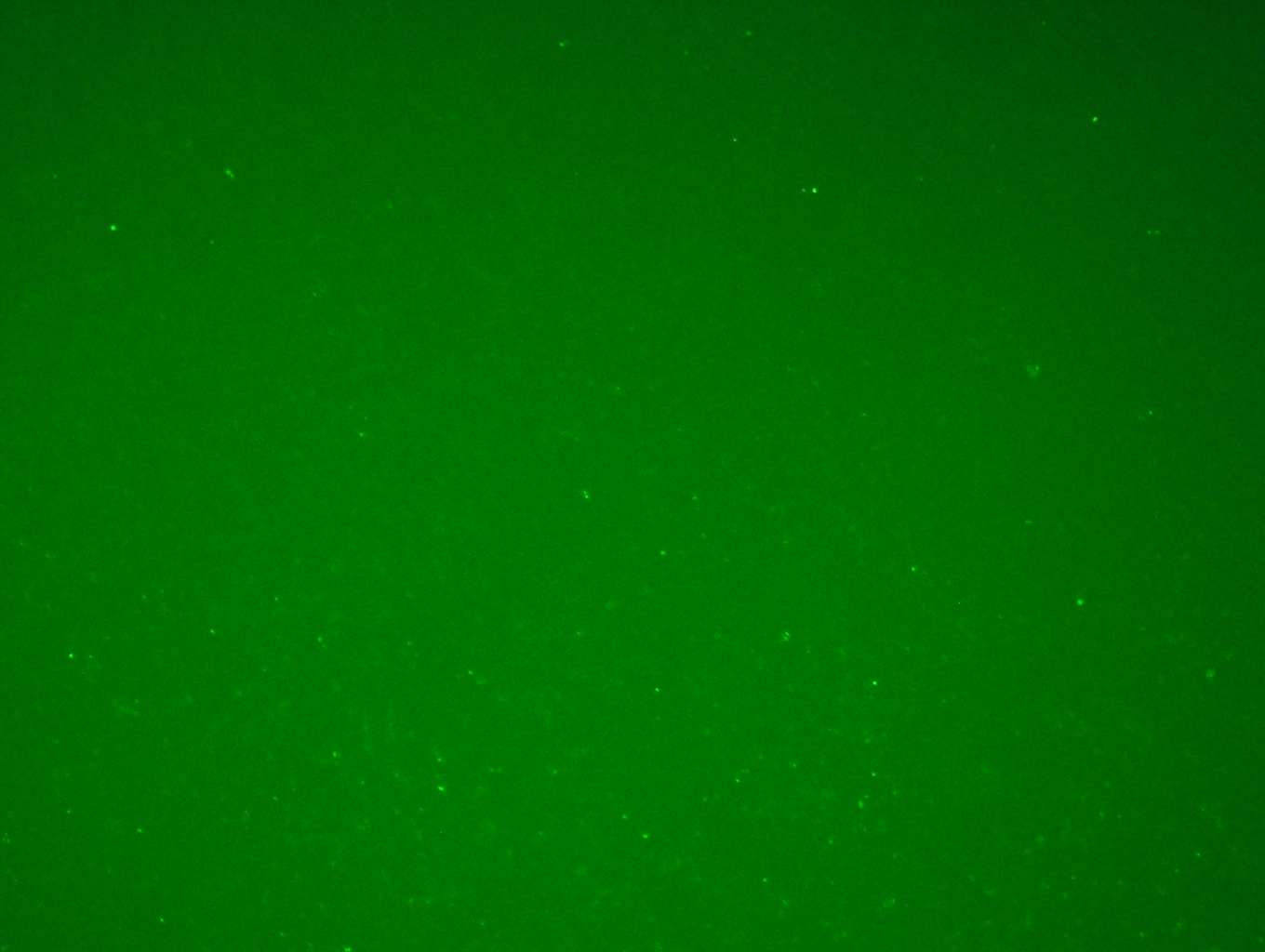

Supplement: Supplementary file 6 — Source data Fig. 5 [file 44318_2025_363_MOESM6_ESM.zip › Figure 5/5K/Control (2).tif]

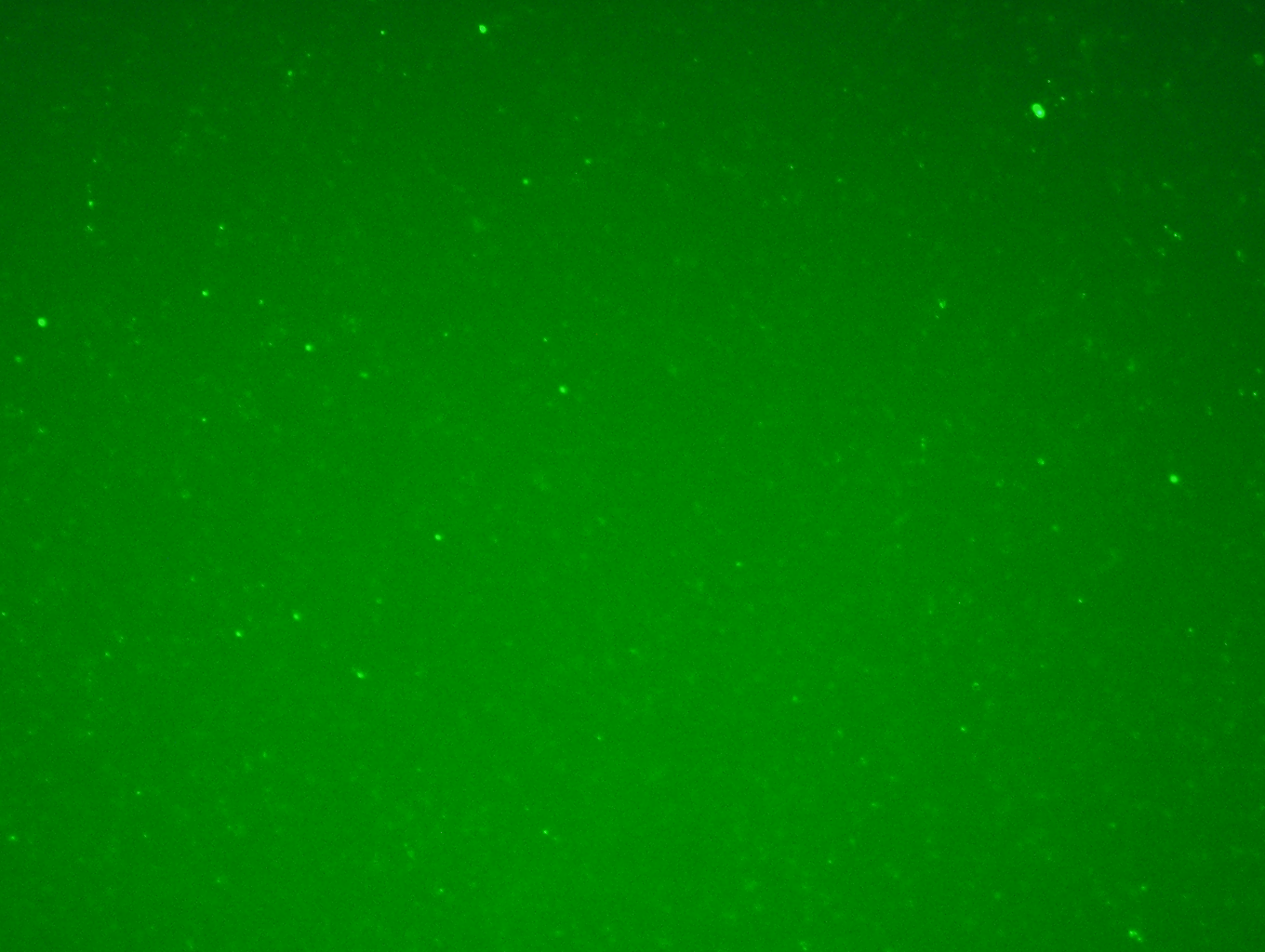

Supplement: Supplementary file 6 — Source data Fig. 5 [file 44318_2025_363_MOESM6_ESM.zip › Figure 5/5K/Control (3).tif]

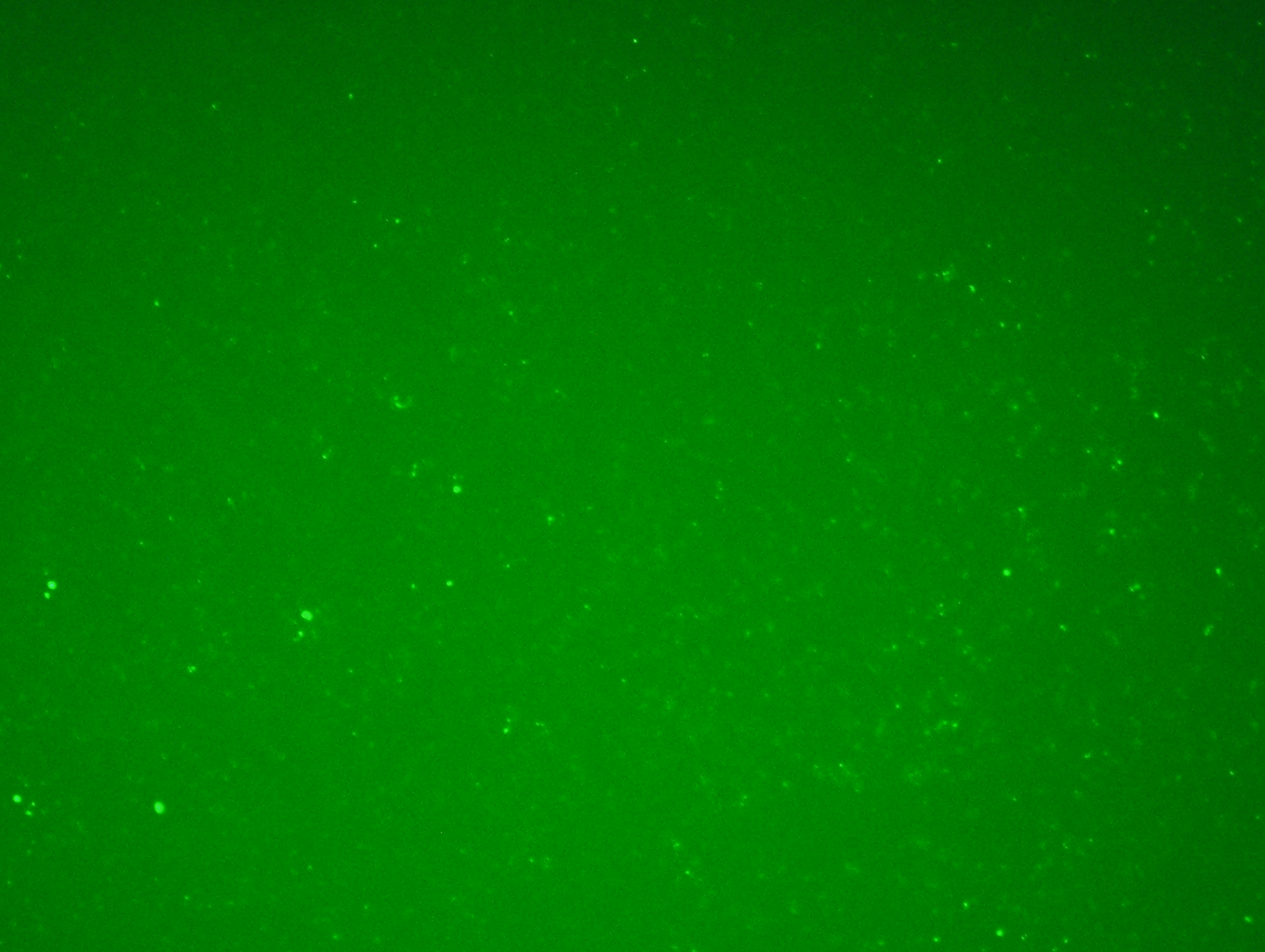

Supplement: Supplementary file 6 — Source data Fig. 5 [file 44318_2025_363_MOESM6_ESM.zip › Figure 5/5K/Control (4).tif]

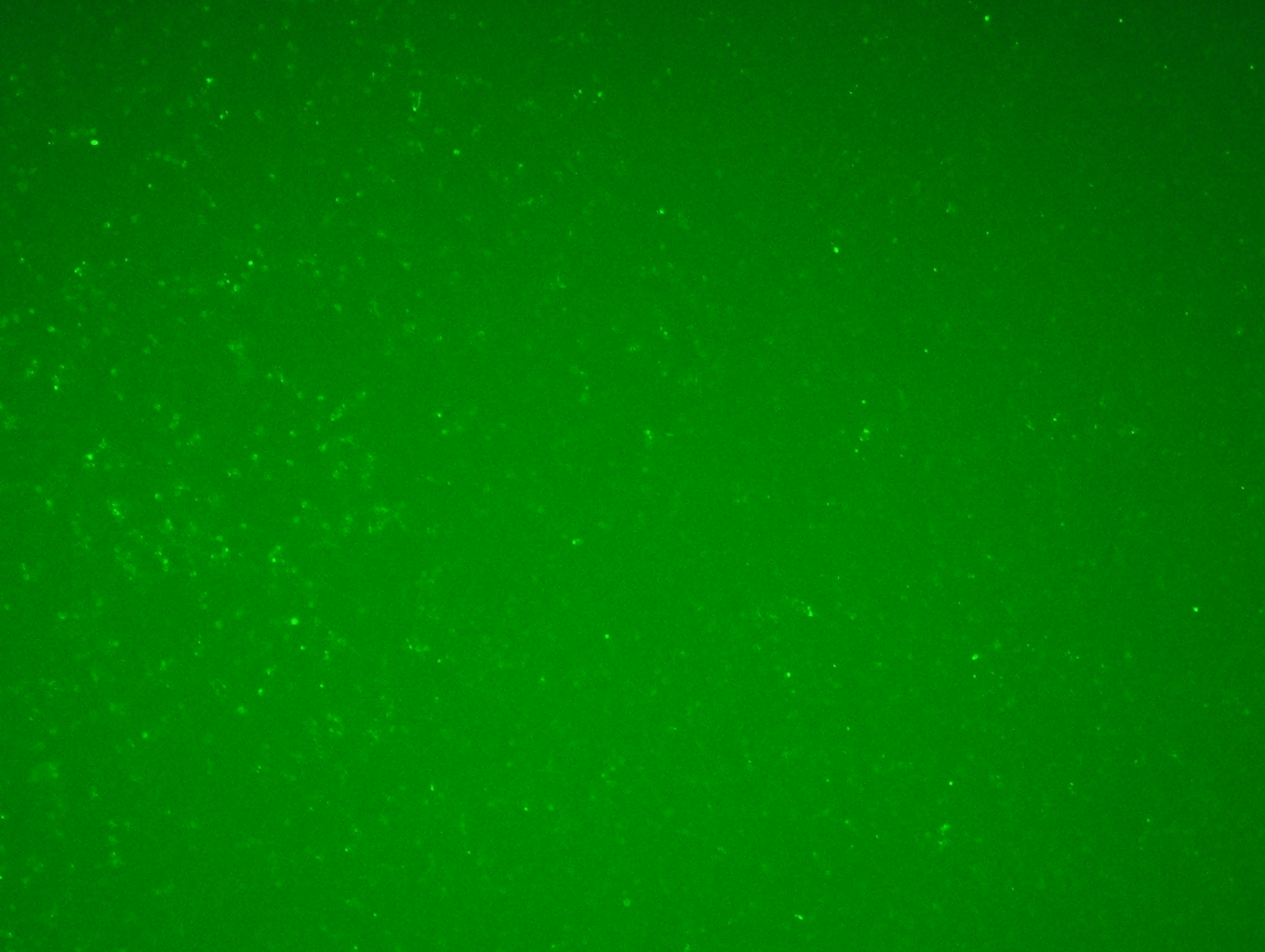

Supplement: Supplementary file 6 — Source data Fig. 5 [file 44318_2025_363_MOESM6_ESM.zip › Figure 5/5K/Control (5).tif]

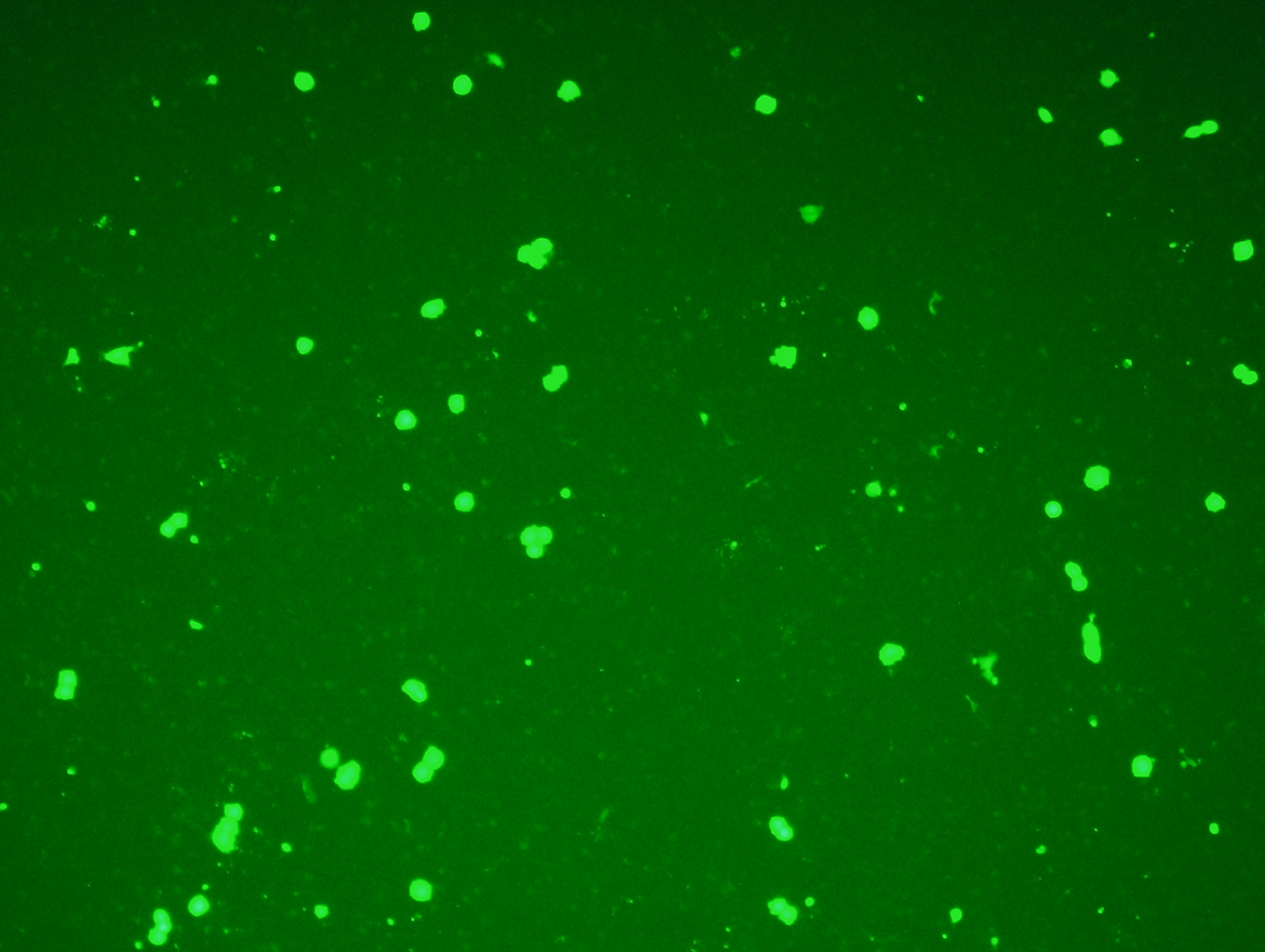

Supplement: Supplementary file 6 — Source data Fig. 5 [file 44318_2025_363_MOESM6_ESM.zip › Figure 5/5K/Ephrin A1 (1).tif]

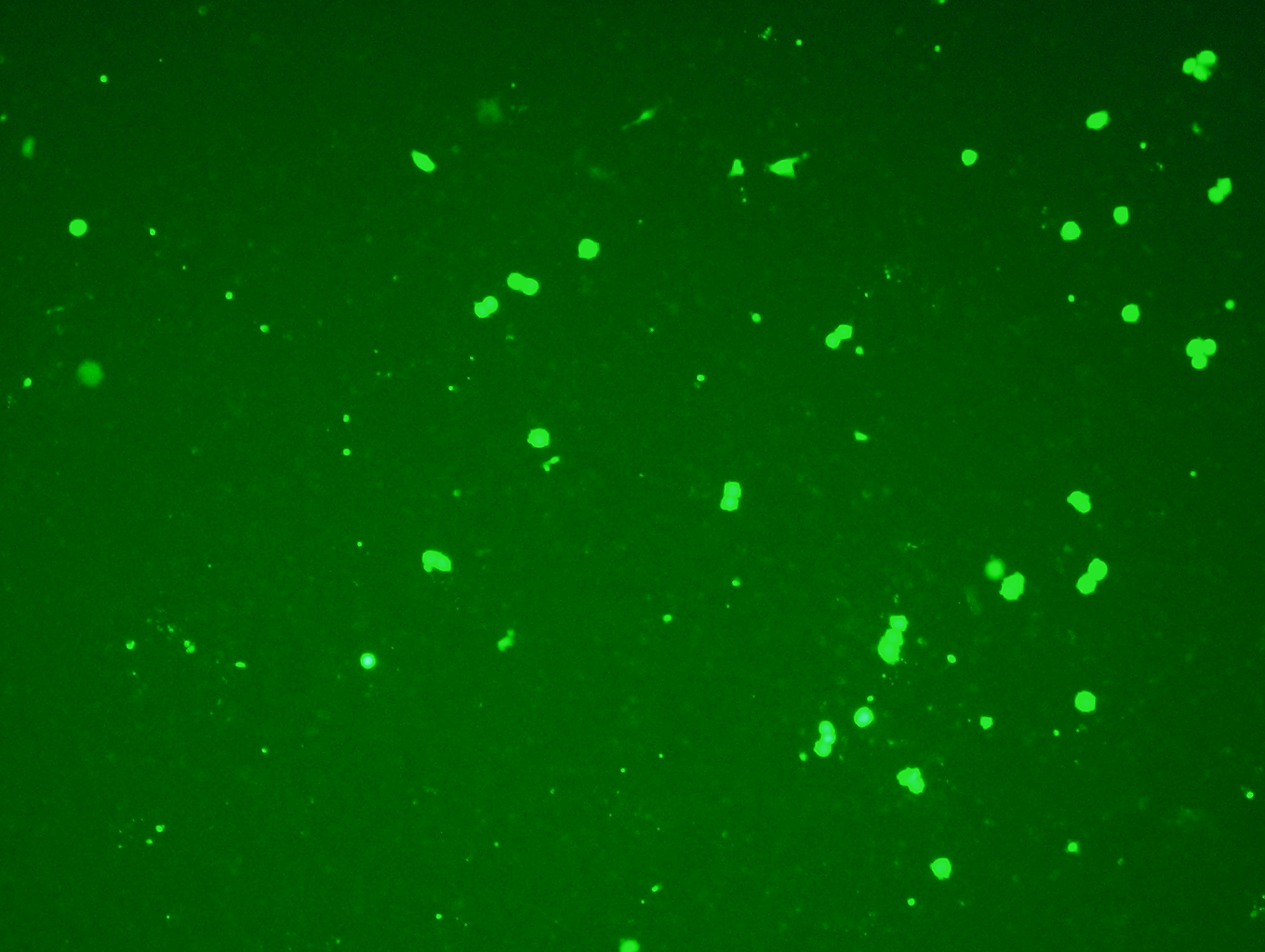

Supplement: Supplementary file 6 — Source data Fig. 5 [file 44318_2025_363_MOESM6_ESM.zip › Figure 5/5K/Ephrin A1 (2).tif]

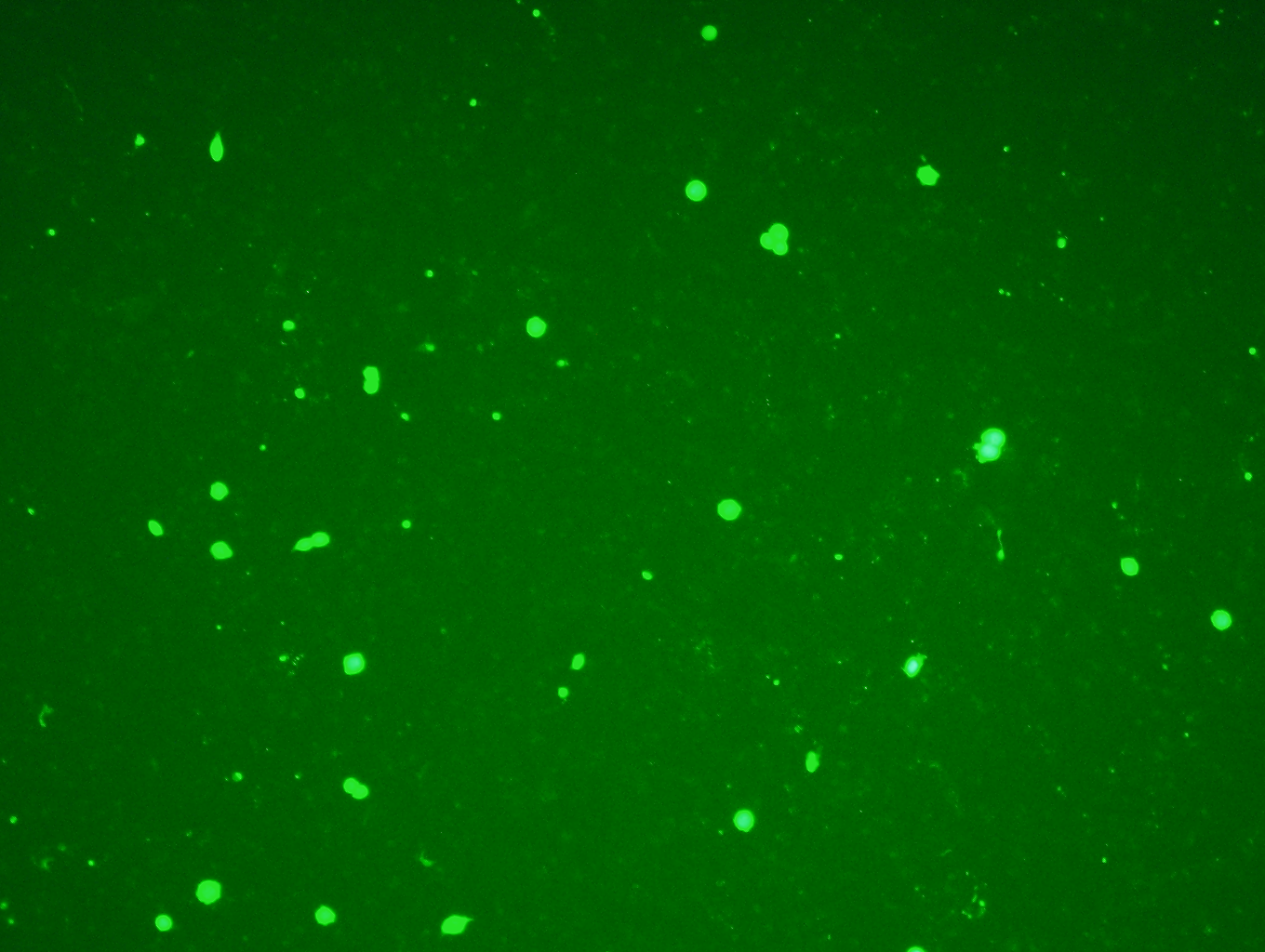

Supplement: Supplementary file 6 — Source data Fig. 5 [file 44318_2025_363_MOESM6_ESM.zip › Figure 5/5K/Ephrin A1 (3).tif]

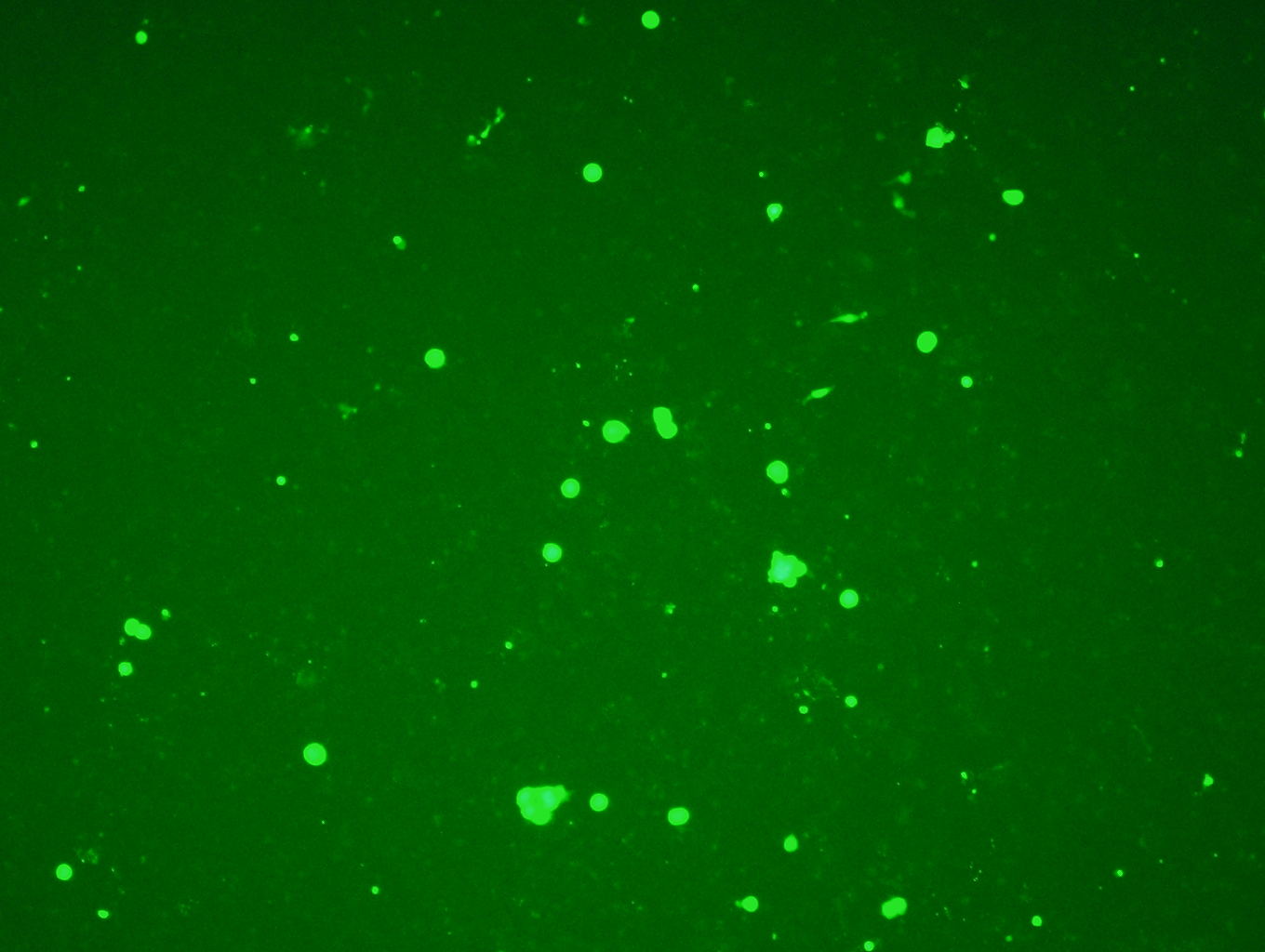

Supplement: Supplementary file 6 — Source data Fig. 5 [file 44318_2025_363_MOESM6_ESM.zip › Figure 5/5K/Ephrin A1 (4).tif]

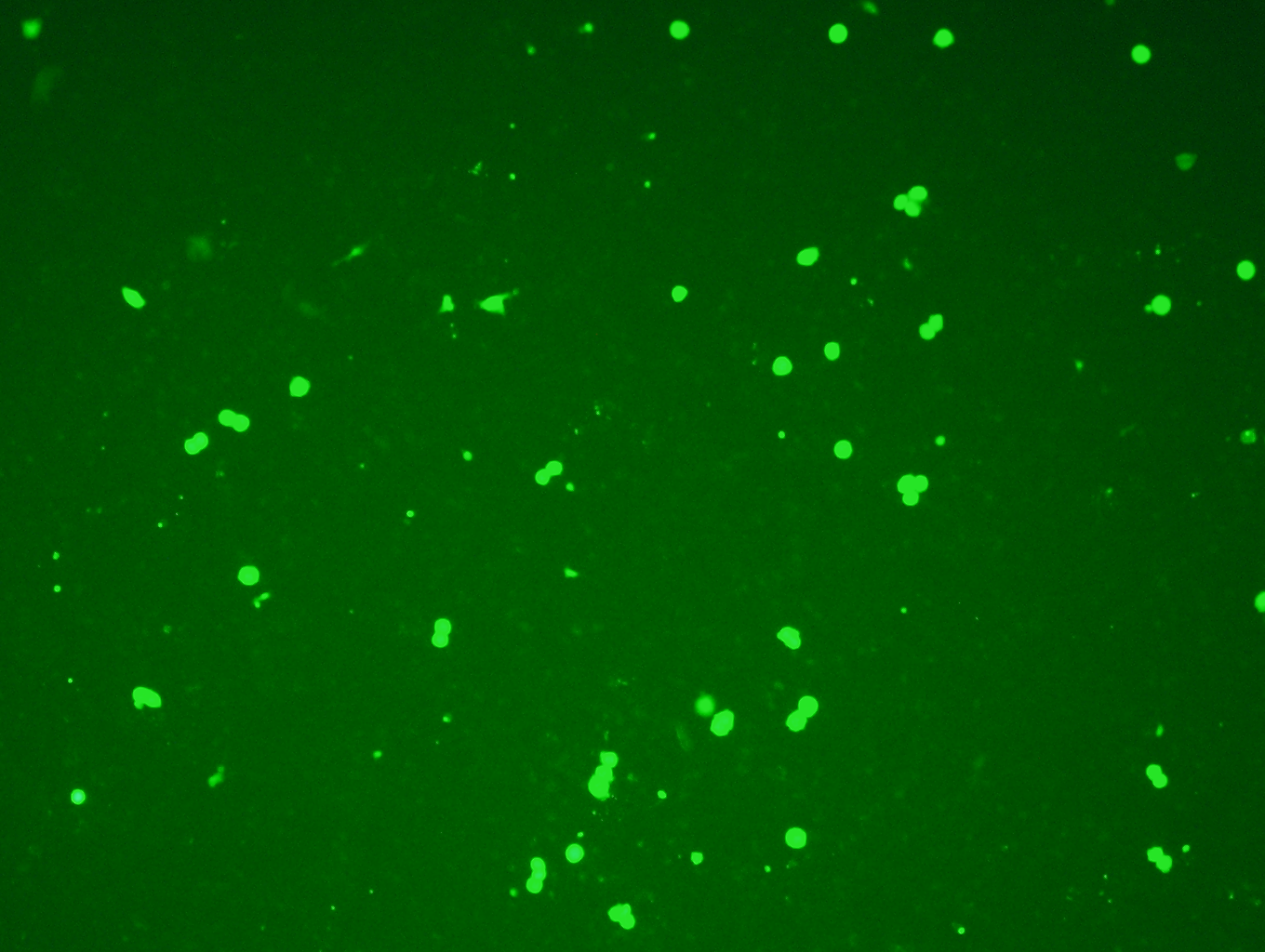

Supplement: Supplementary file 6 — Source data Fig. 5 [file 44318_2025_363_MOESM6_ESM.zip › Figure 5/5K/Ephrin A1 (5)-displayed in 5K.tif]

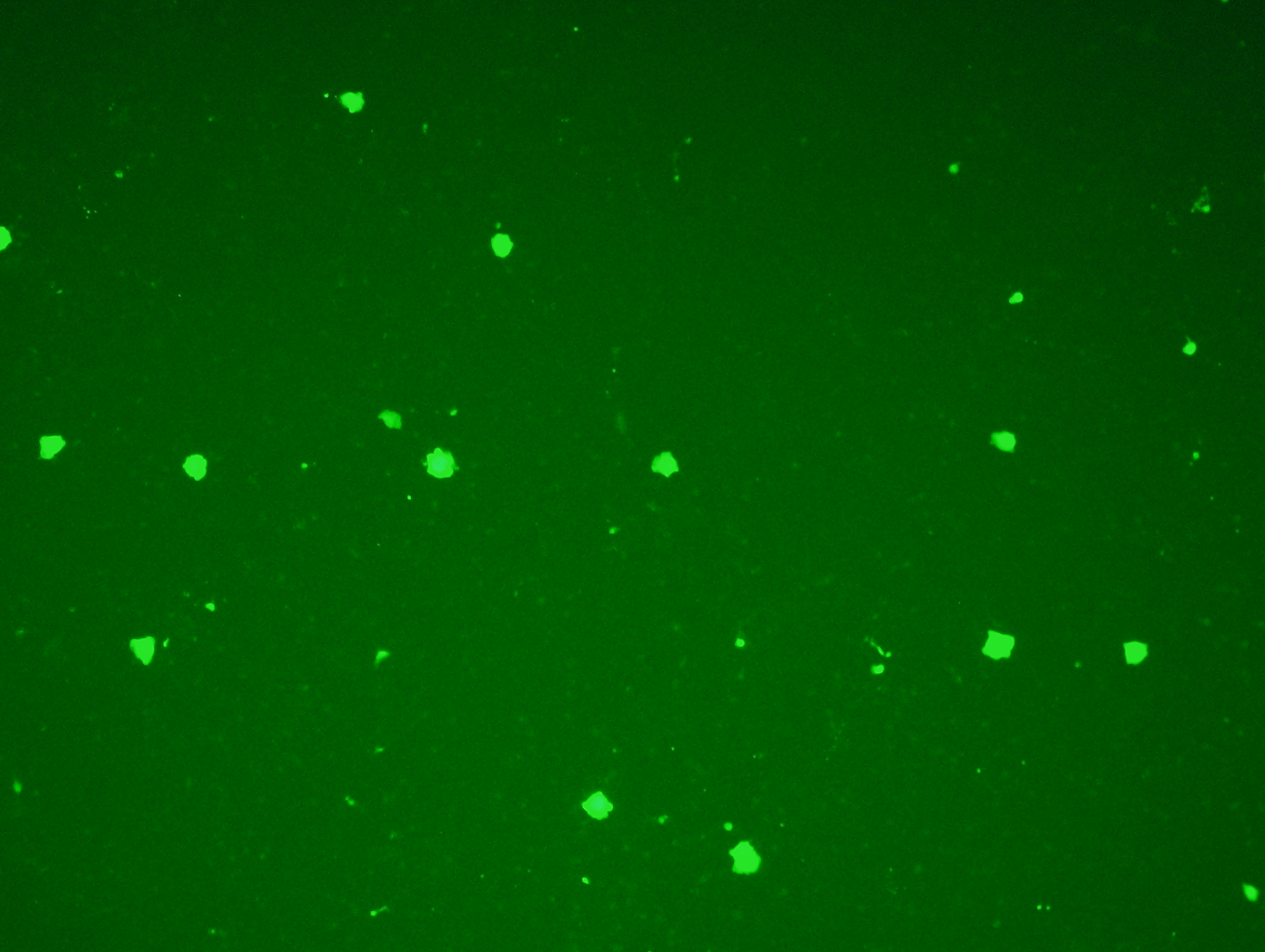

Supplement: Supplementary file 6 — Source data Fig. 5 [file 44318_2025_363_MOESM6_ESM.zip › Figure 5/5K/Ephrin A1+10um (1)-displayed in 5K.tif]

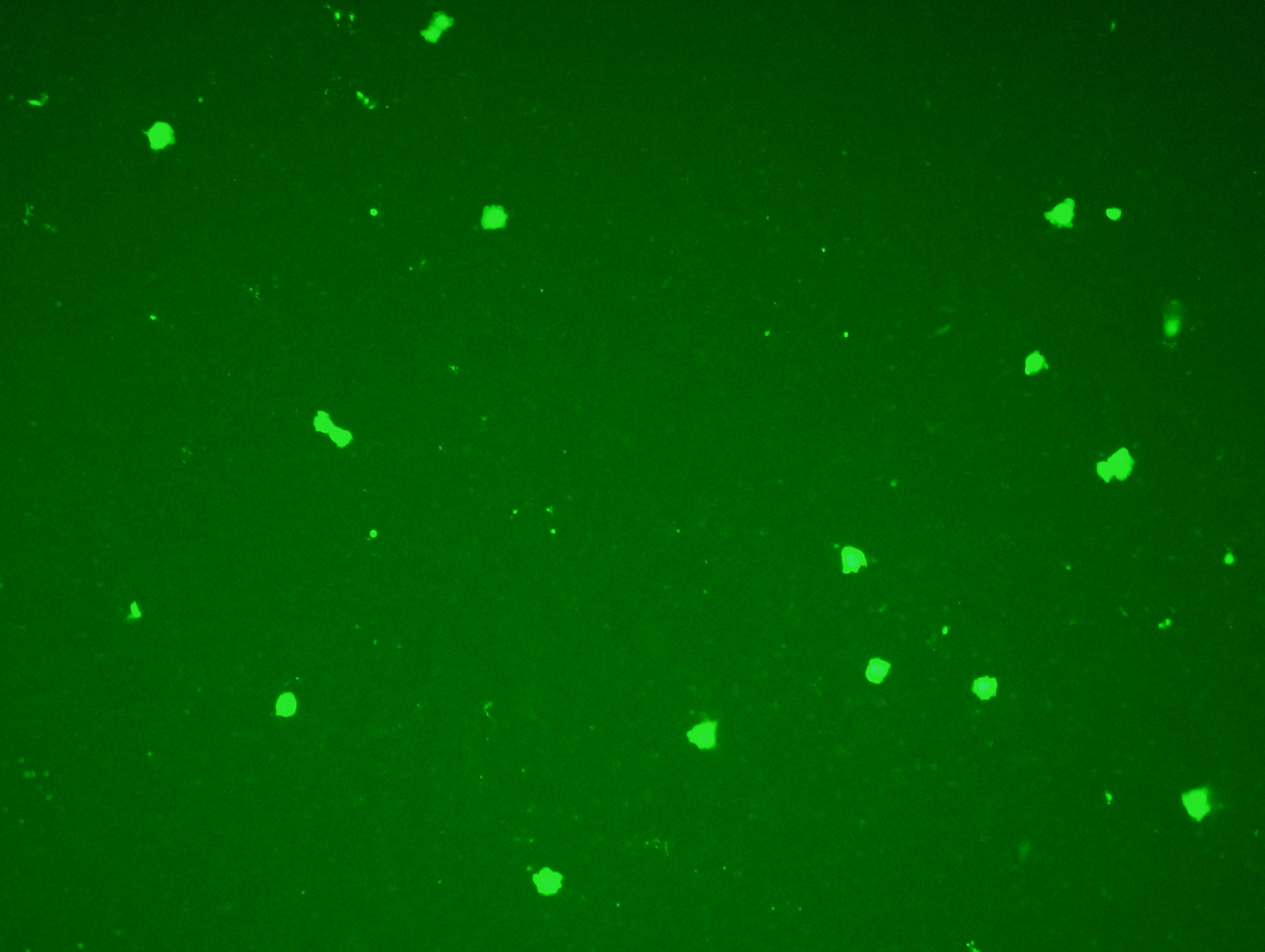

Supplement: Supplementary file 6 — Source data Fig. 5 [file 44318_2025_363_MOESM6_ESM.zip › Figure 5/5K/Ephrin A1+10um (2).tif]

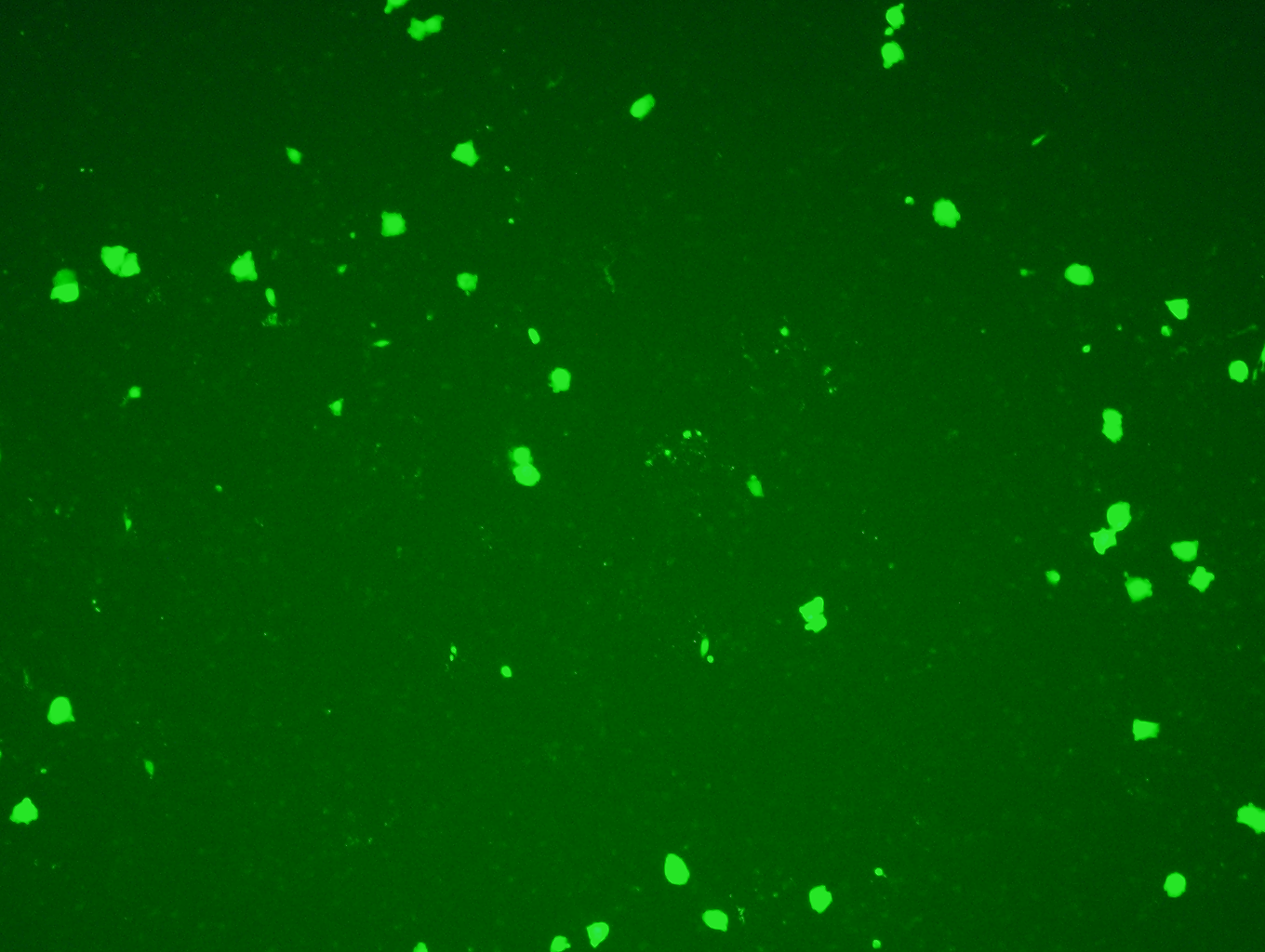

Supplement: Supplementary file 6 — Source data Fig. 5 [file 44318_2025_363_MOESM6_ESM.zip › Figure 5/5K/Ephrin A1+10um (3).tif]

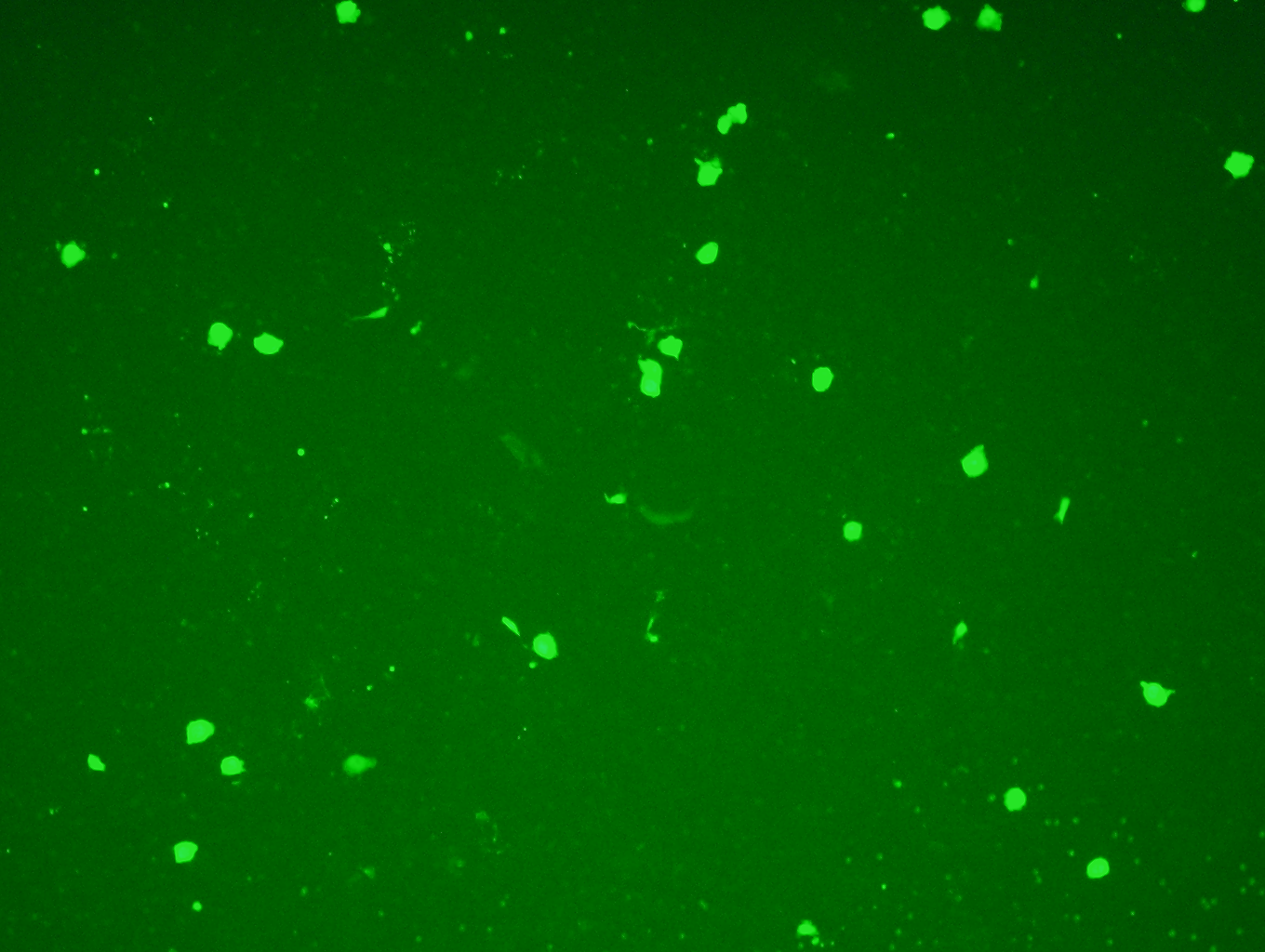

Supplement: Supplementary file 6 — Source data Fig. 5 [file 44318_2025_363_MOESM6_ESM.zip › Figure 5/5K/Ephrin A1+10um (4).tif]

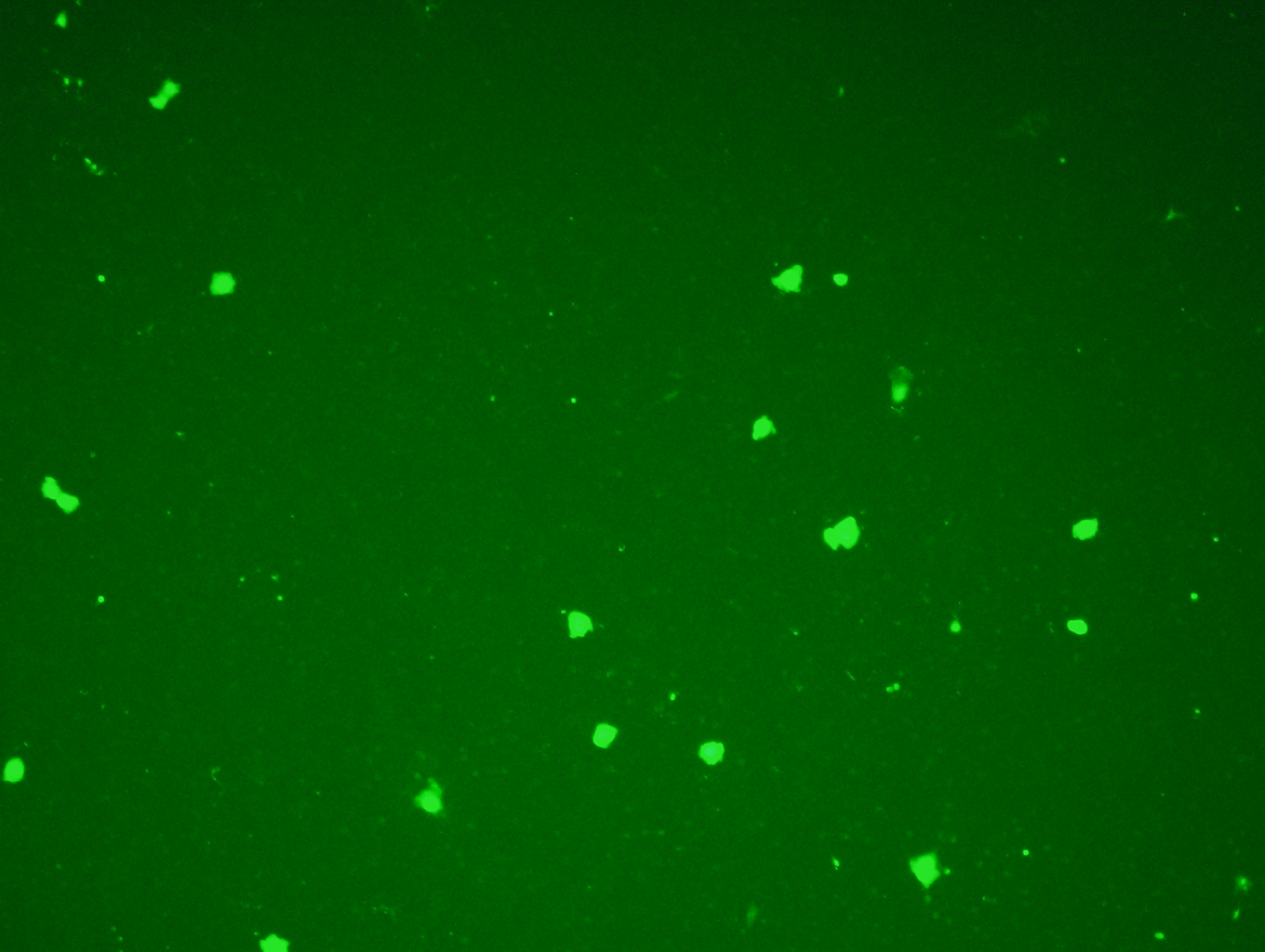

Supplement: Supplementary file 6 — Source data Fig. 5 [file 44318_2025_363_MOESM6_ESM.zip › Figure 5/5K/Ephrin A1+10um (5).tif]

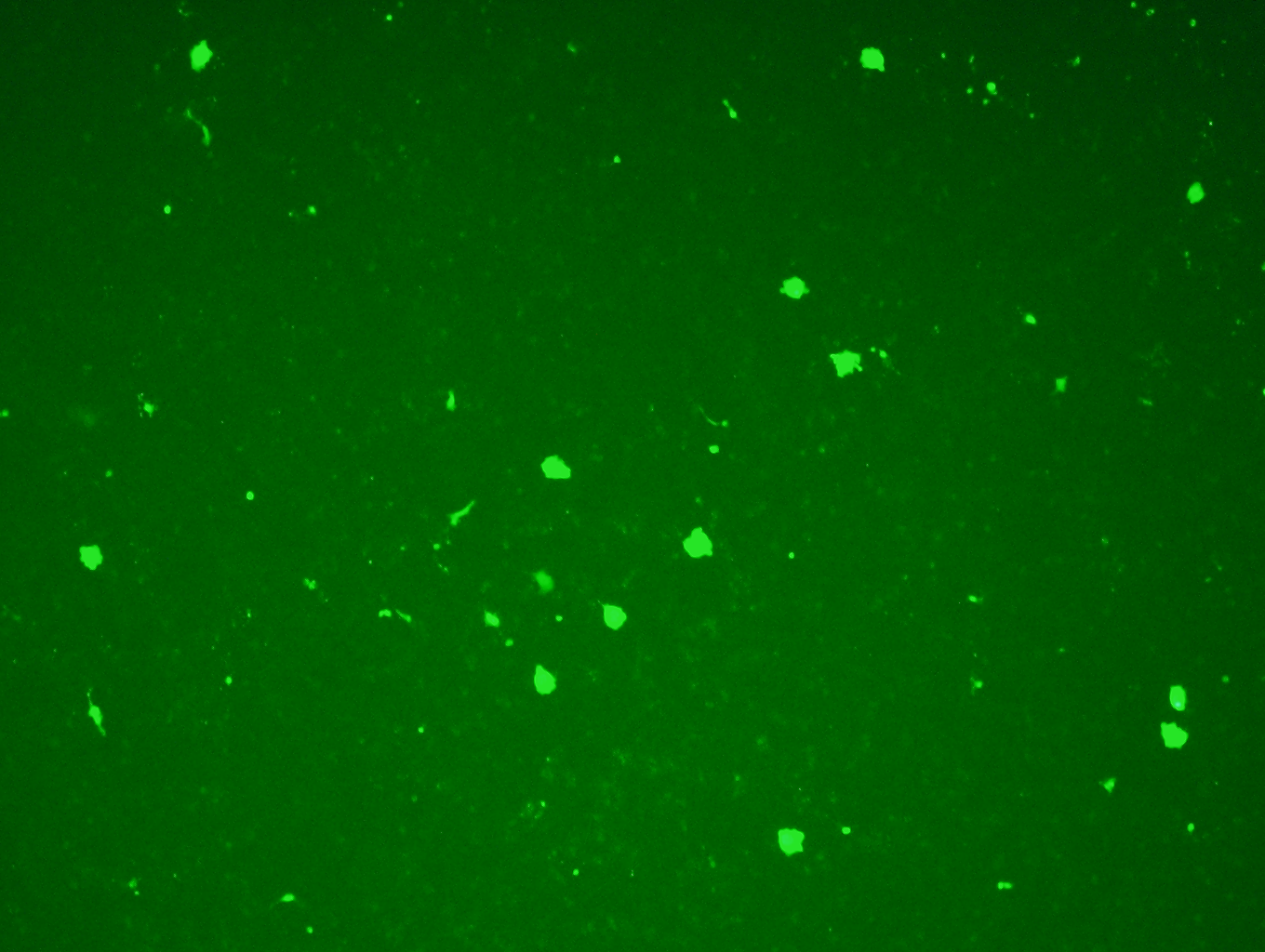

Supplement: Supplementary file 6 — Source data Fig. 5 [file 44318_2025_363_MOESM6_ESM.zip › Figure 5/5K/Ephrin A1+5um (1).tif]

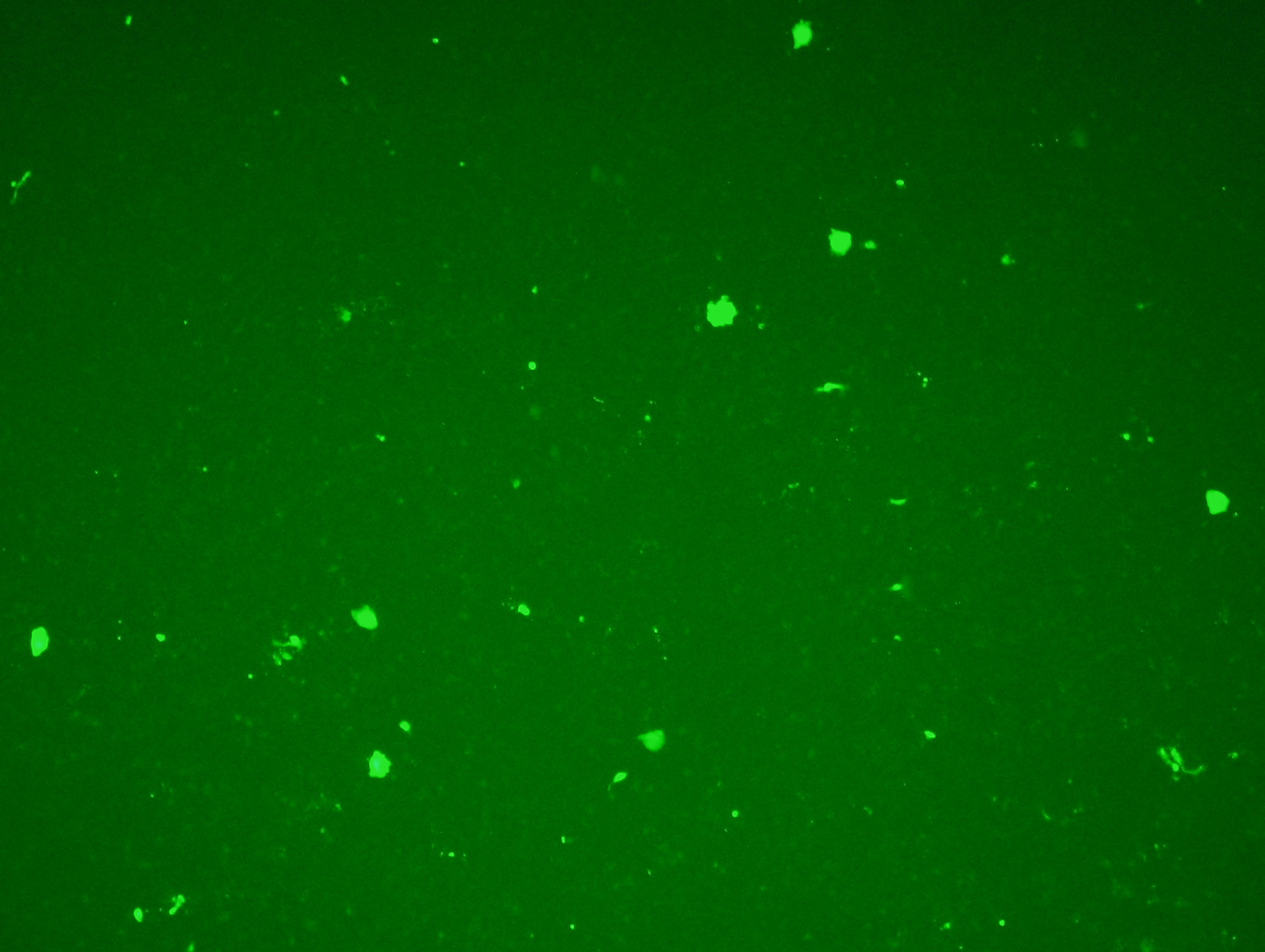

Supplement: Supplementary file 6 — Source data Fig. 5 [file 44318_2025_363_MOESM6_ESM.zip › Figure 5/5K/Ephrin A1+5um (2).tif]

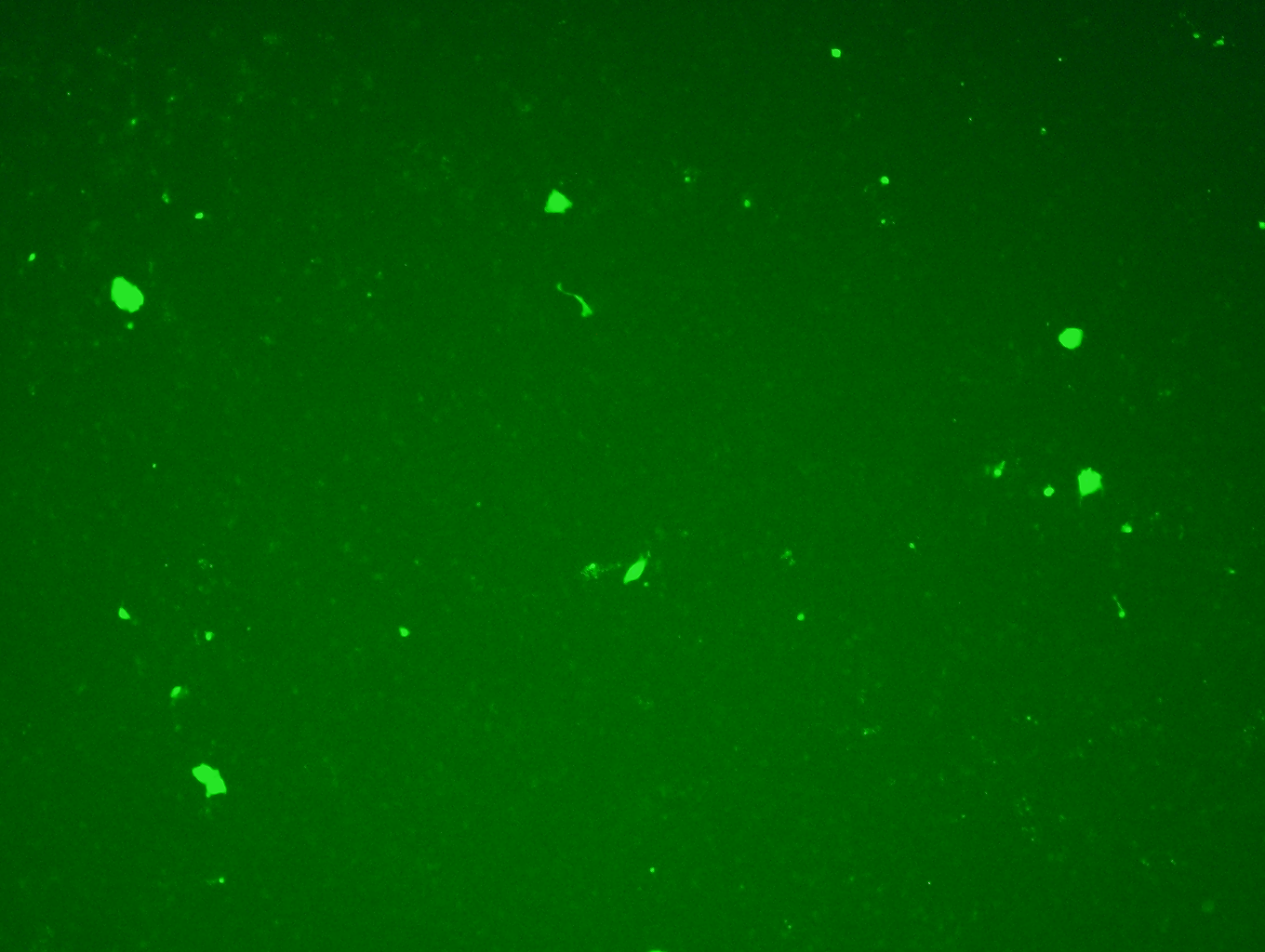

Supplement: Supplementary file 6 — Source data Fig. 5 [file 44318_2025_363_MOESM6_ESM.zip › Figure 5/5K/Ephrin A1+5um (3).tif]

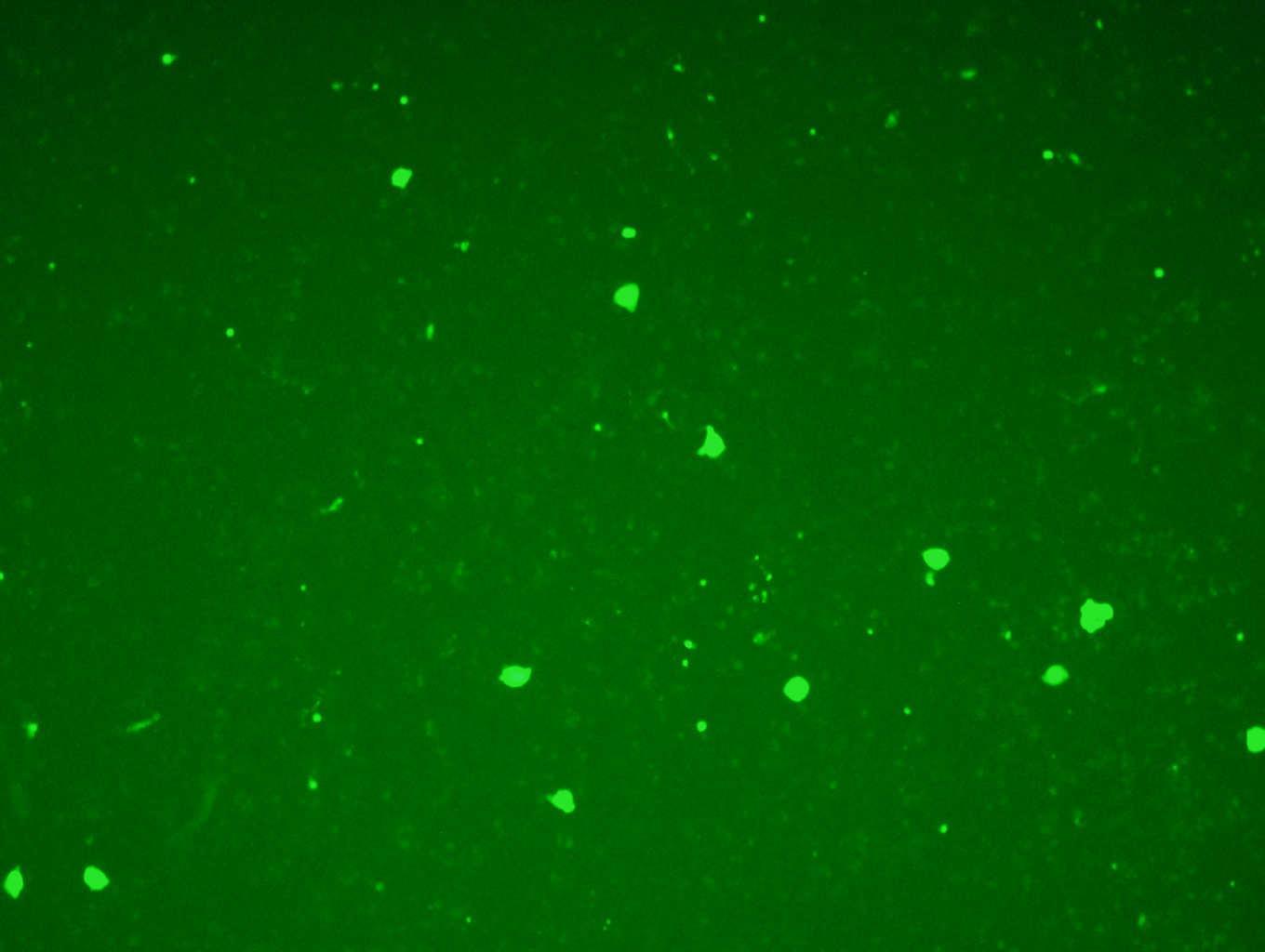

Supplement: Supplementary file 6 — Source data Fig. 5 [file 44318_2025_363_MOESM6_ESM.zip › Figure 5/5K/Ephrin A1+5um (4).tif]

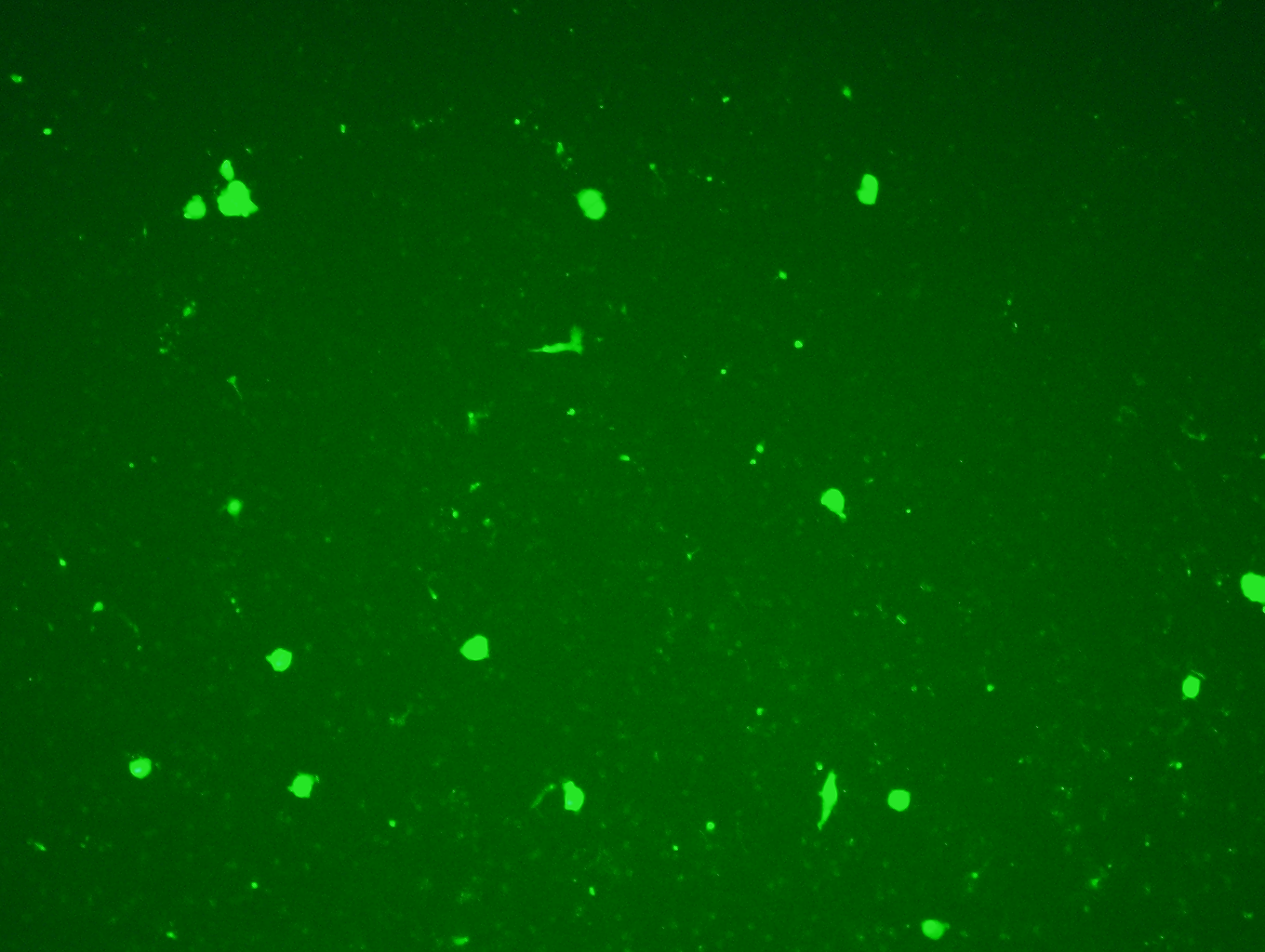

Supplement: Supplementary file 6 — Source data Fig. 5 [file 44318_2025_363_MOESM6_ESM.zip › Figure 5/5K/Ephrin A1+5um (5)-displayed in 5K.tif]

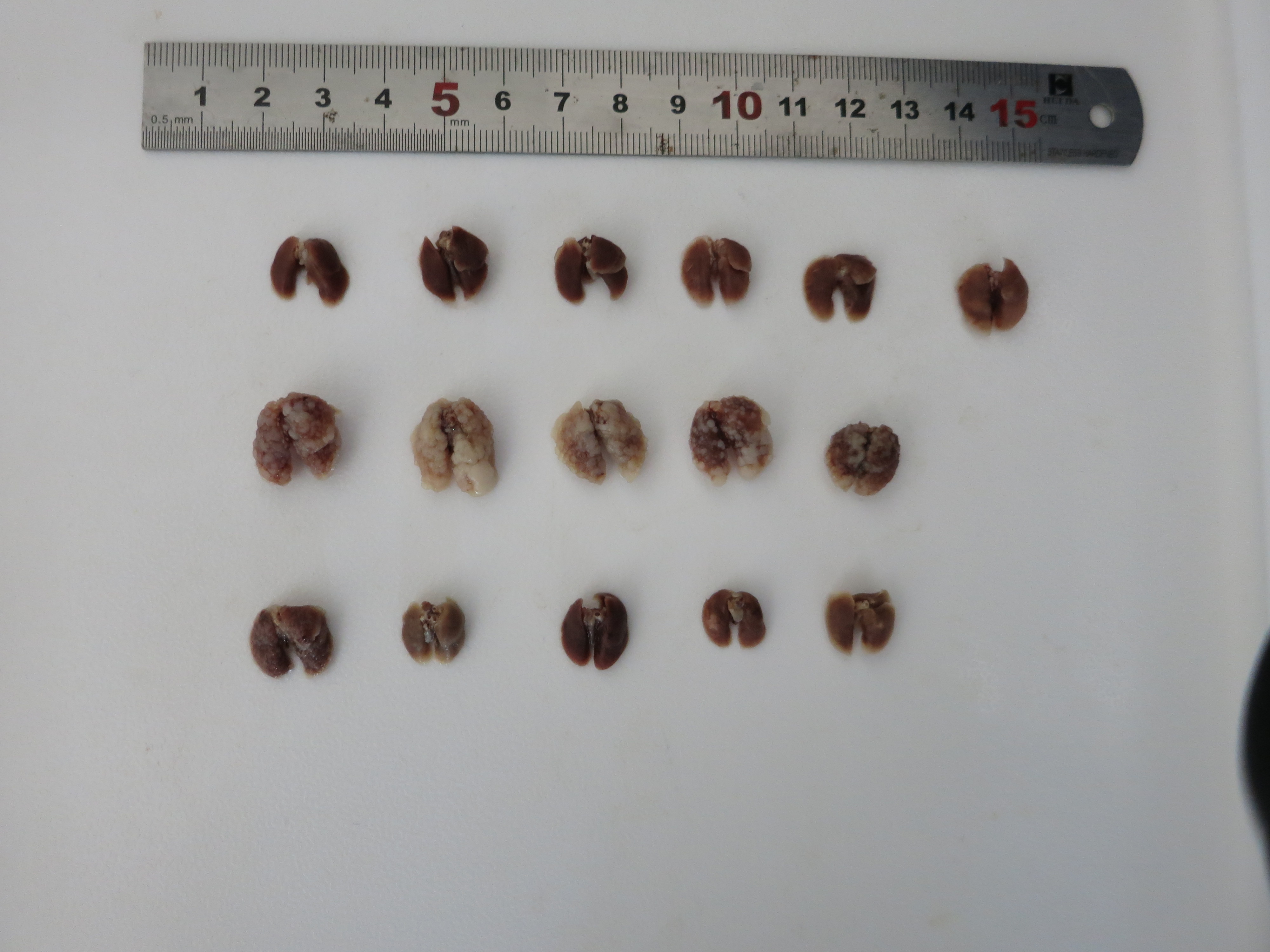

Supplement: Supplementary file 7 — Source data Fig. 6 [file 44318_2025_363_MOESM7_ESM.zip › Figure 6/6B/6B.JPG]

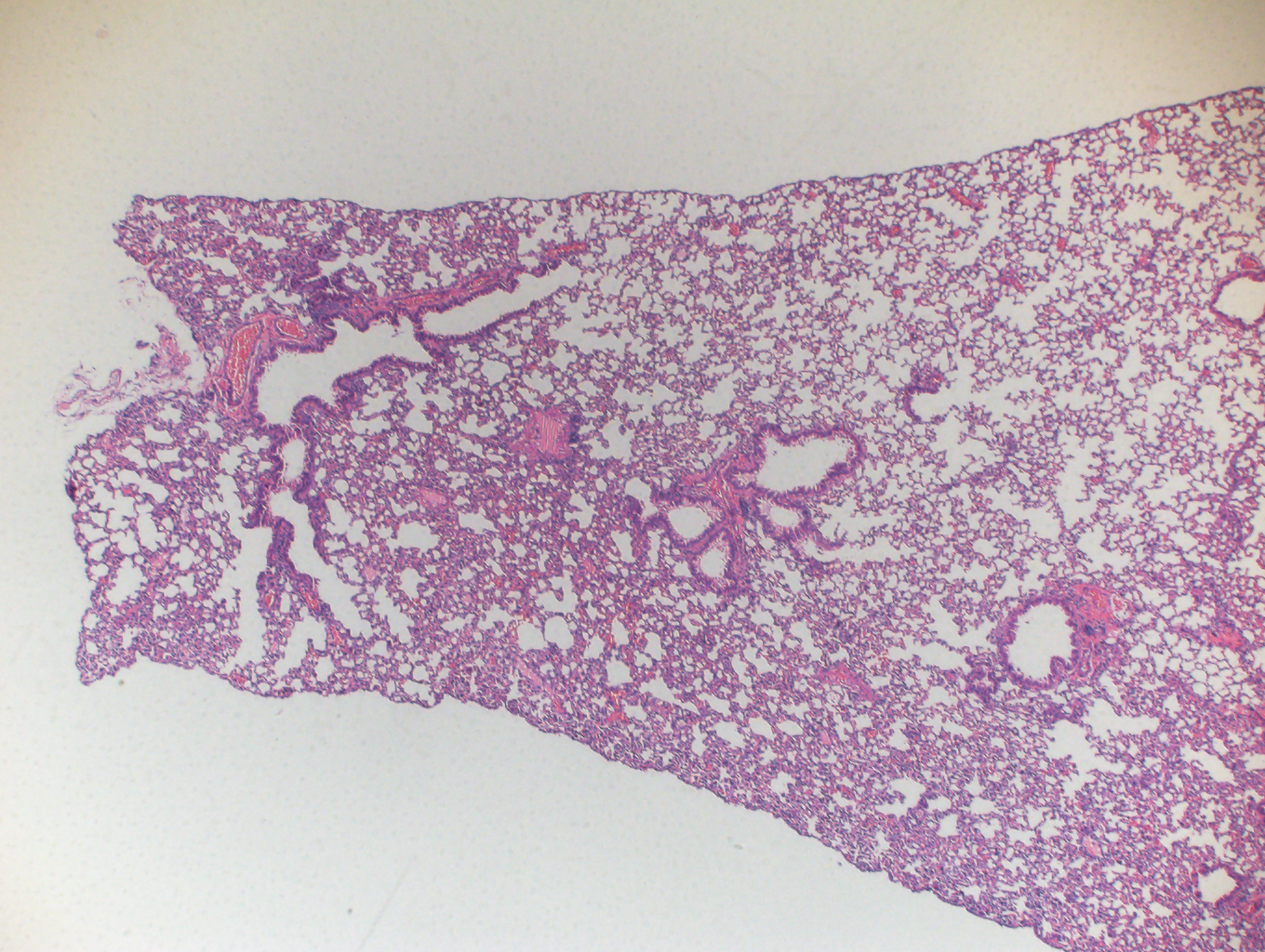

Supplement: Supplementary file 7 — Source data Fig. 6 [file 44318_2025_363_MOESM7_ESM.zip › Figure 6/6C/Control (1).tif]

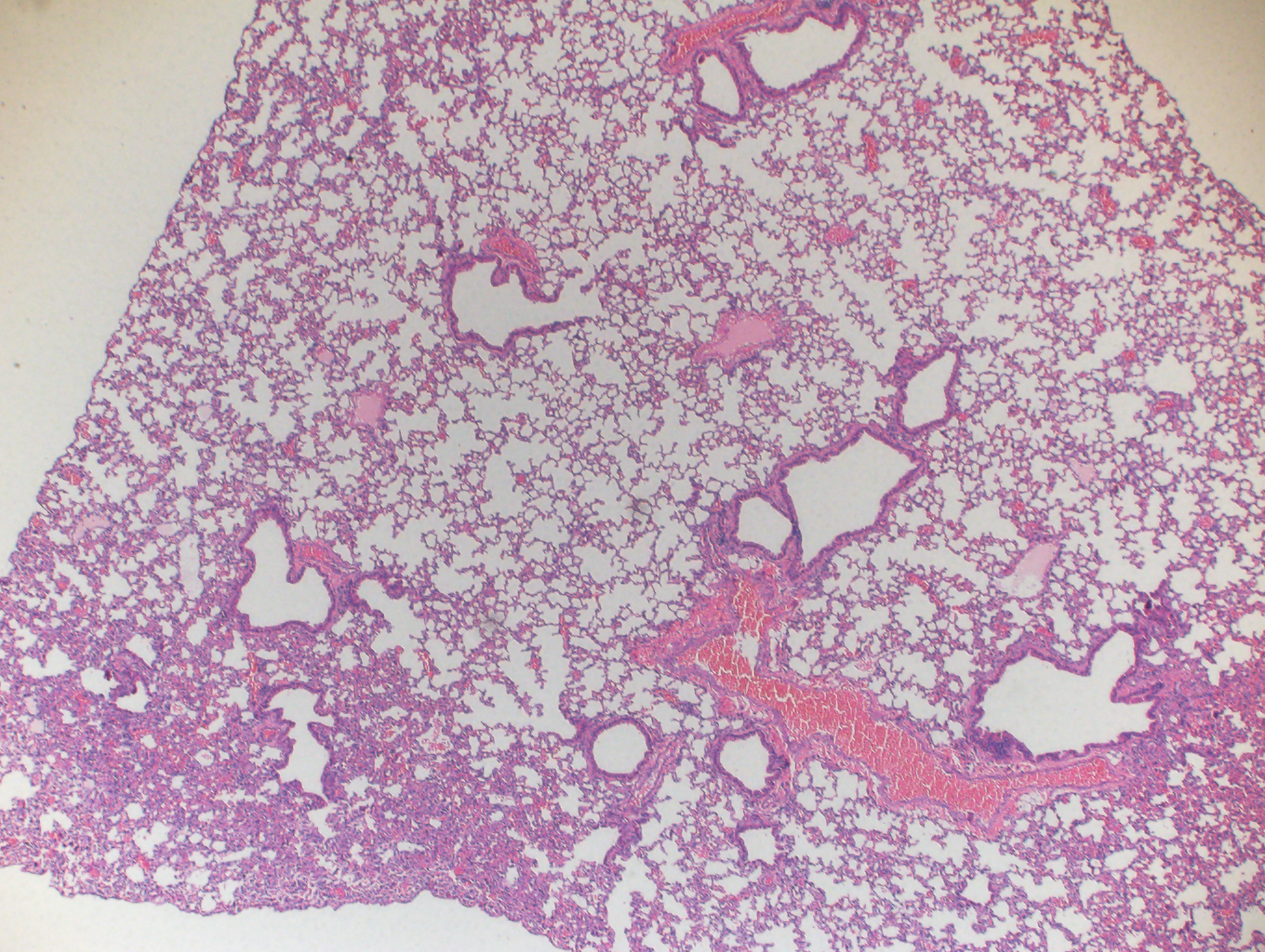

Supplement: Supplementary file 7 — Source data Fig. 6 [file 44318_2025_363_MOESM7_ESM.zip › Figure 6/6C/Control (2).tif]

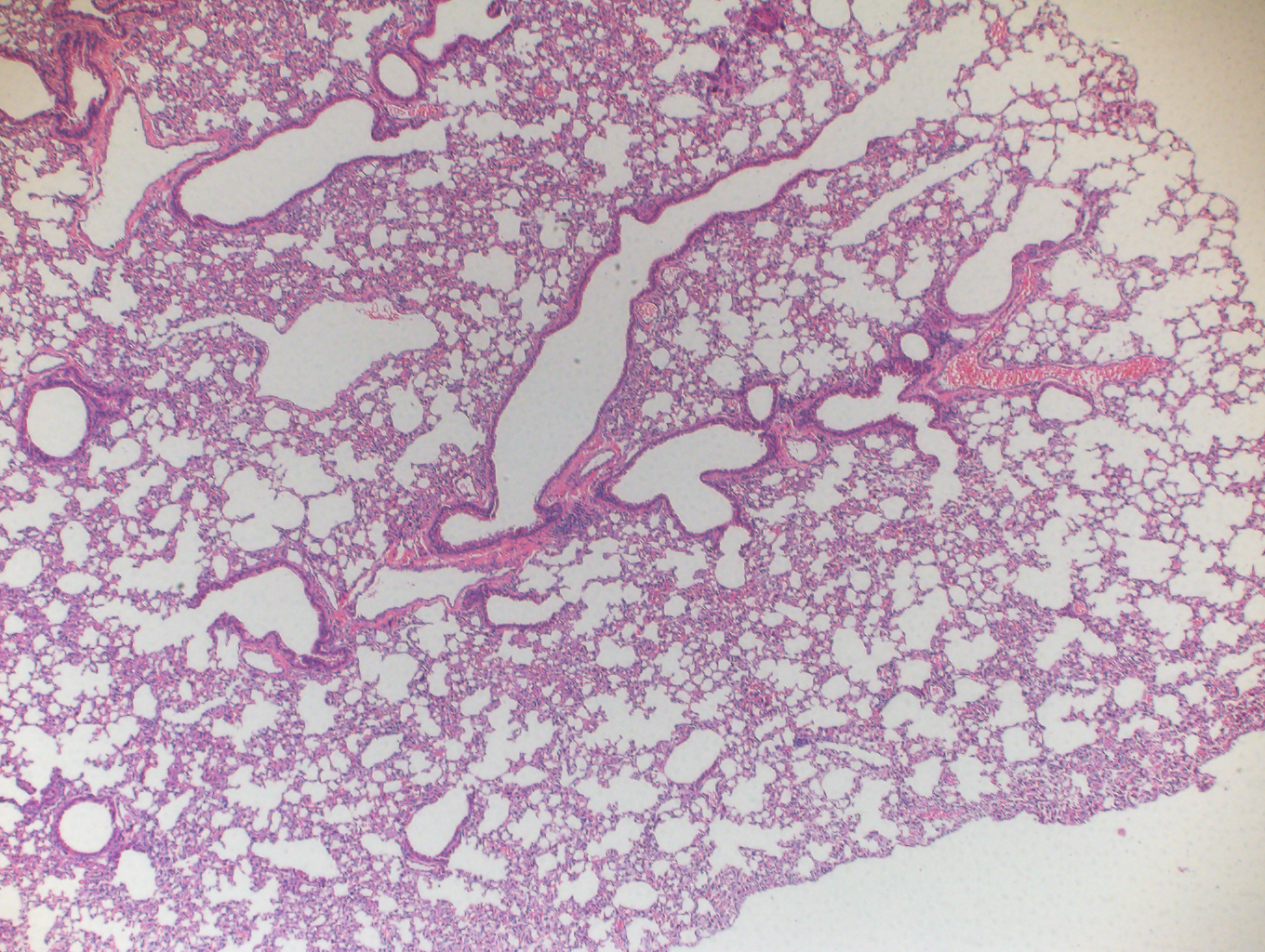

Supplement: Supplementary file 7 — Source data Fig. 6 [file 44318_2025_363_MOESM7_ESM.zip › Figure 6/6C/Control (3)-displayed in 6C.tif]

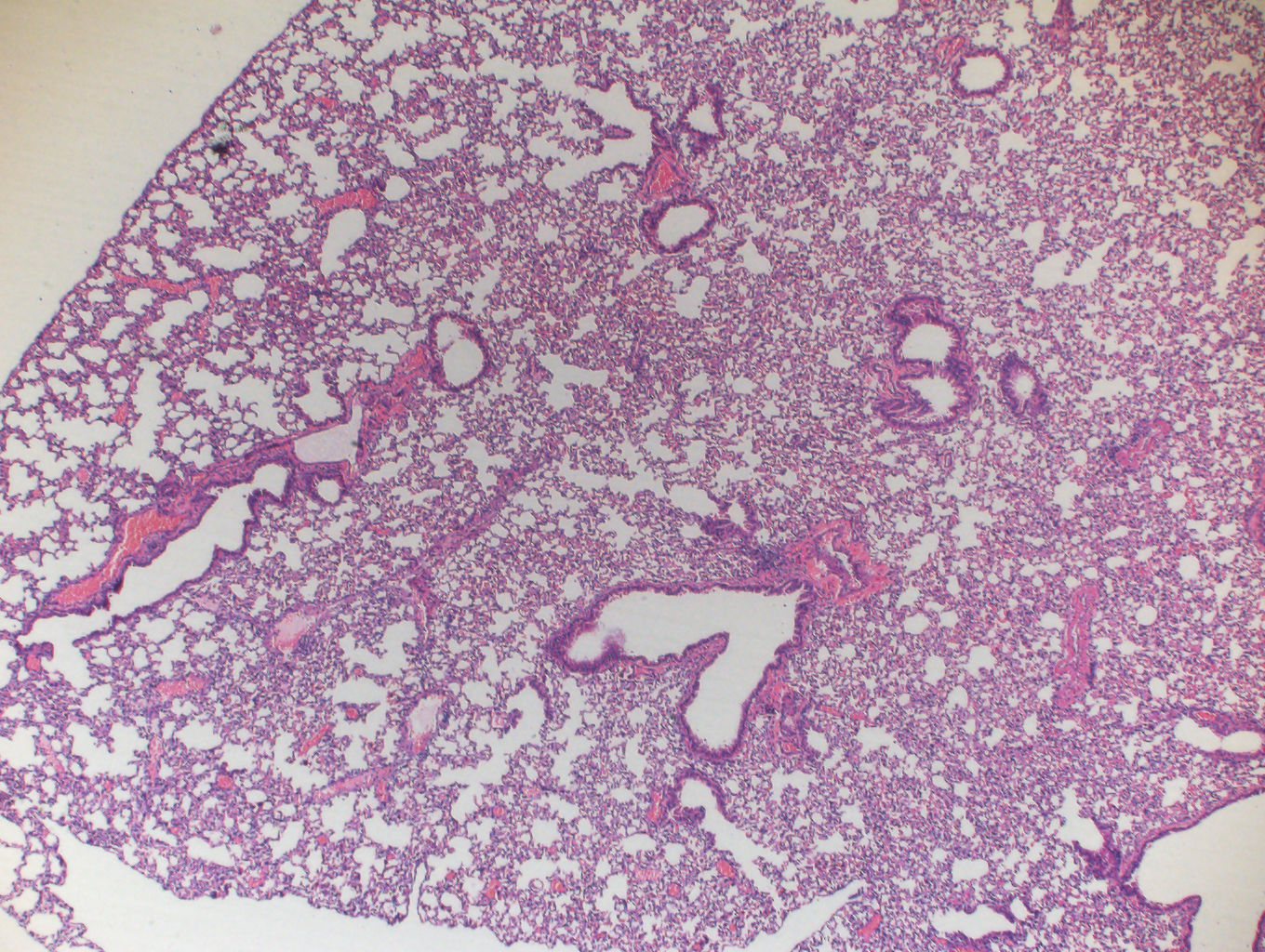

Supplement: Supplementary file 7 — Source data Fig. 6 [file 44318_2025_363_MOESM7_ESM.zip › Figure 6/6C/Control (4).tif]

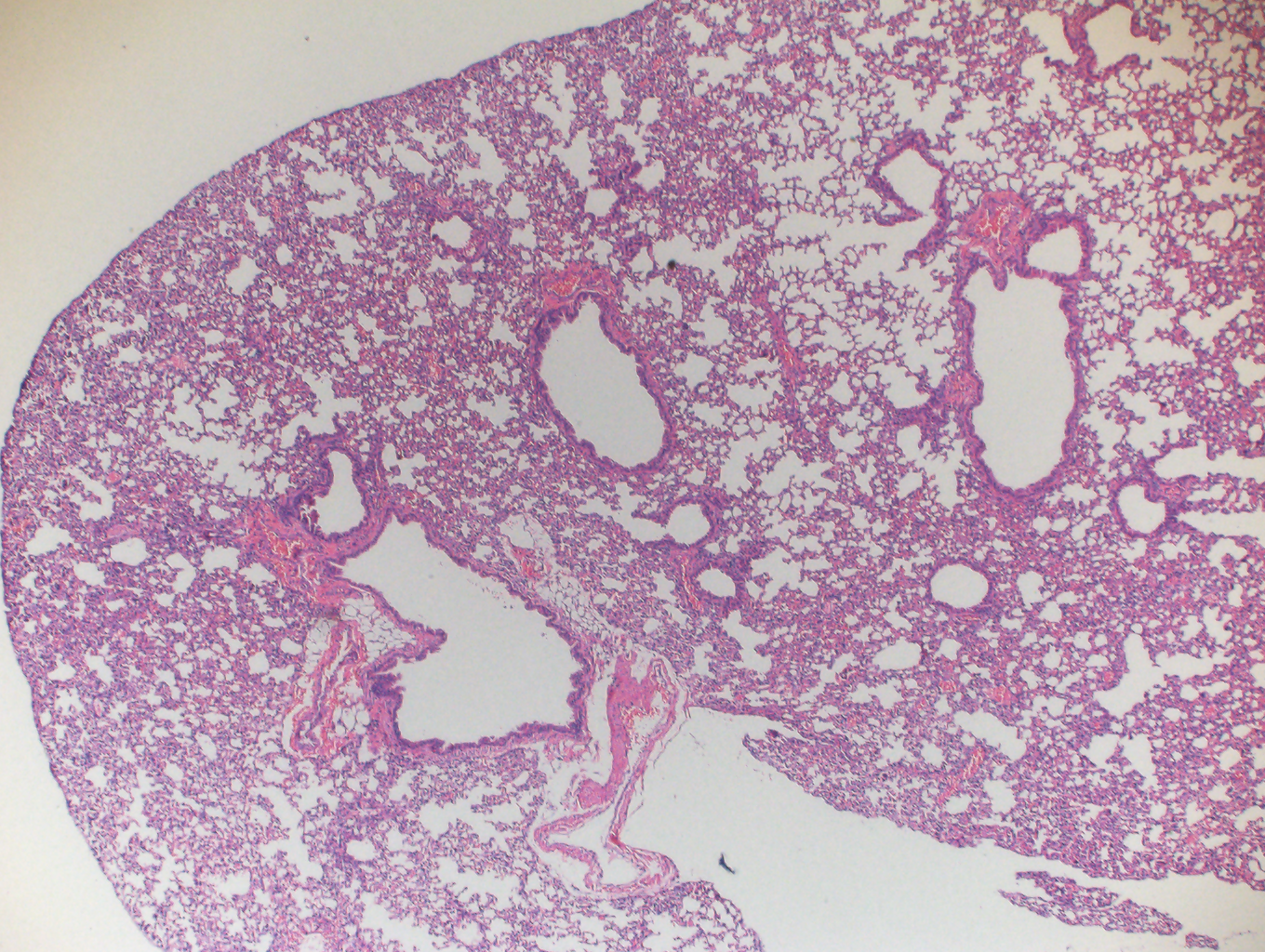

Supplement: Supplementary file 7 — Source data Fig. 6 [file 44318_2025_363_MOESM7_ESM.zip › Figure 6/6C/Control (5).tif]

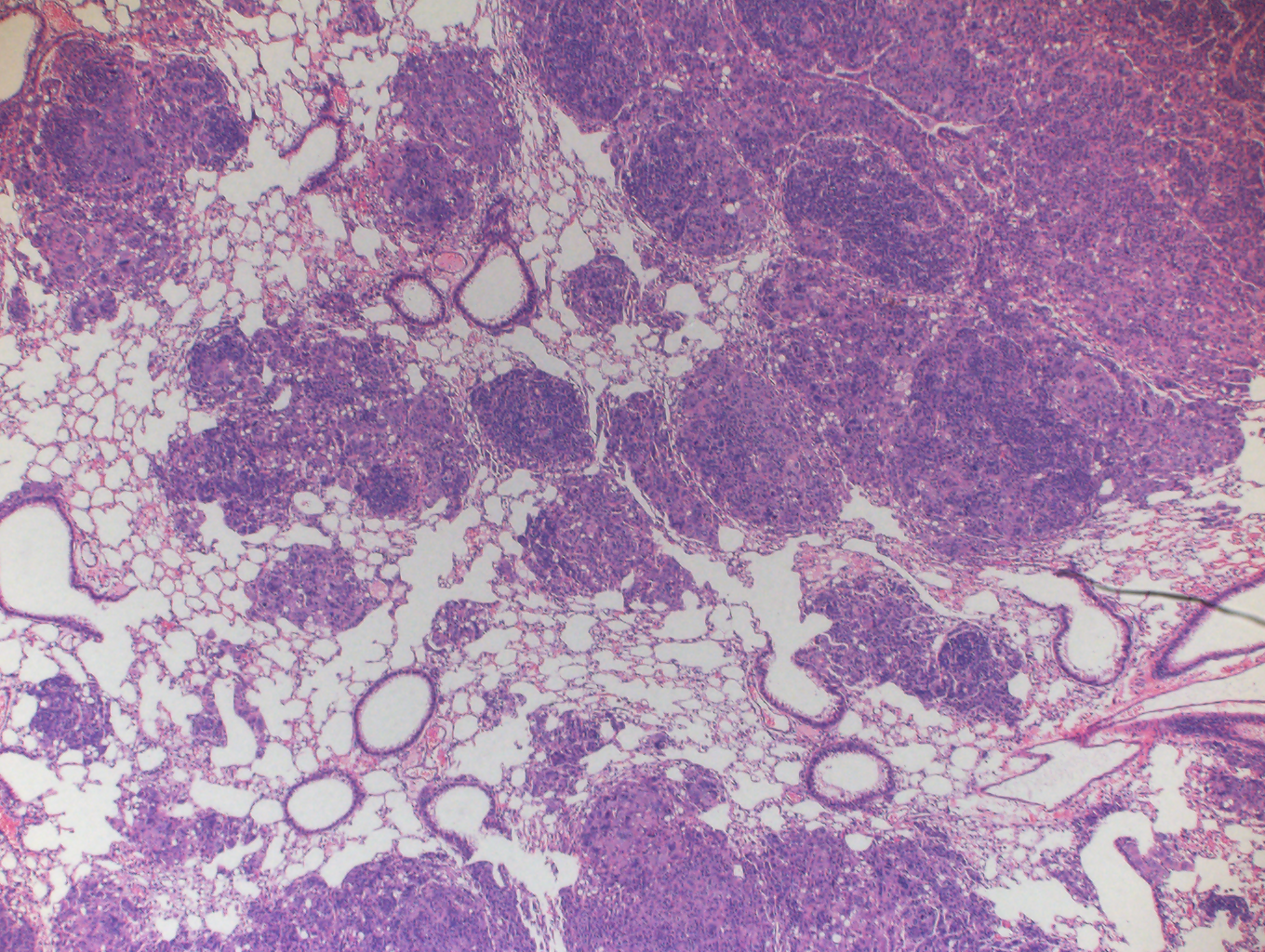

Supplement: Supplementary file 7 — Source data Fig. 6 [file 44318_2025_363_MOESM7_ESM.zip › Figure 6/6C/Ephrin A1 (1).tif]

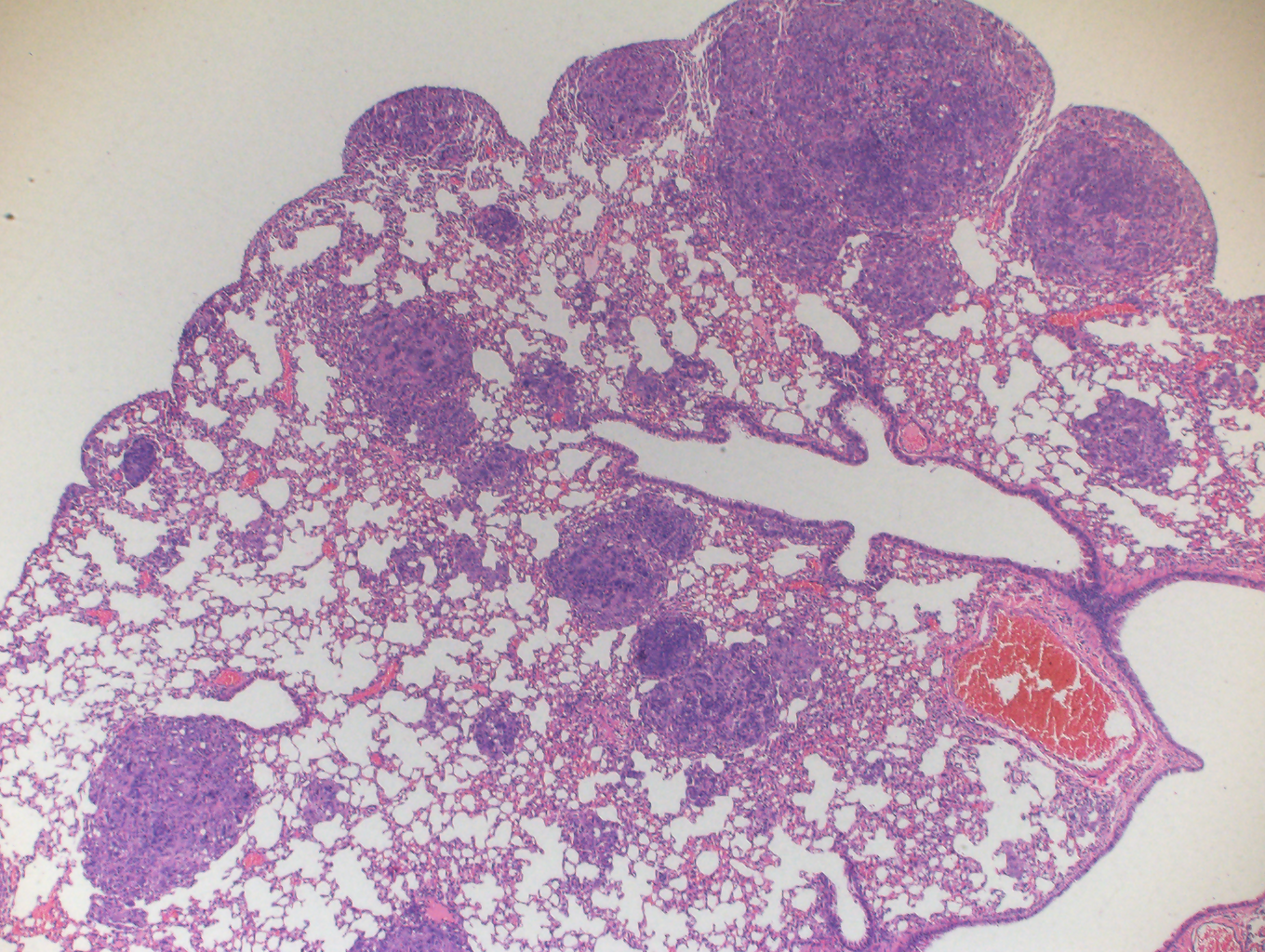

Supplement: Supplementary file 7 — Source data Fig. 6 [file 44318_2025_363_MOESM7_ESM.zip › Figure 6/6C/Ephrin A1 (2).tif]

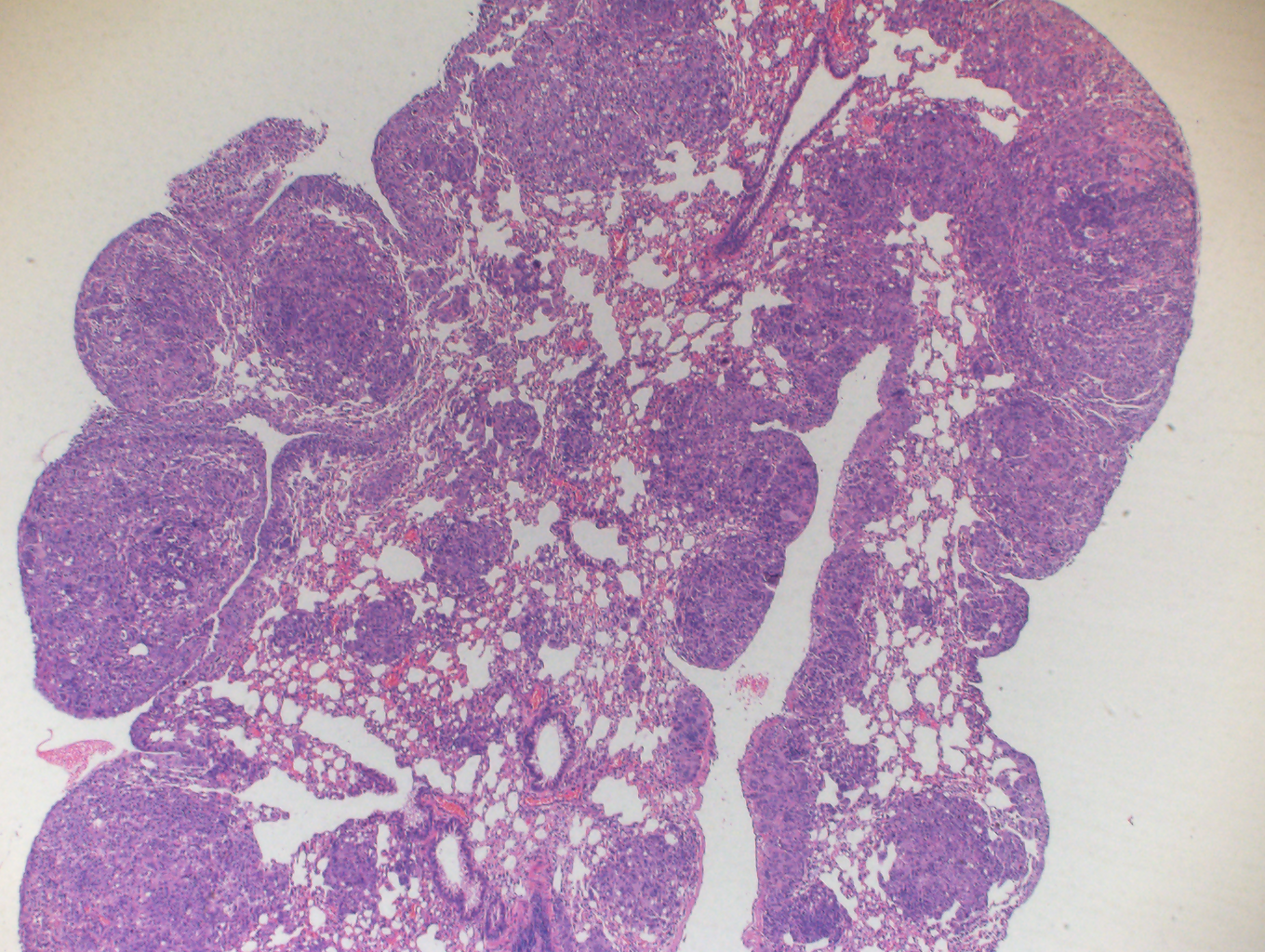

Supplement: Supplementary file 7 — Source data Fig. 6 [file 44318_2025_363_MOESM7_ESM.zip › Figure 6/6C/Ephrin A1 (3).tif]

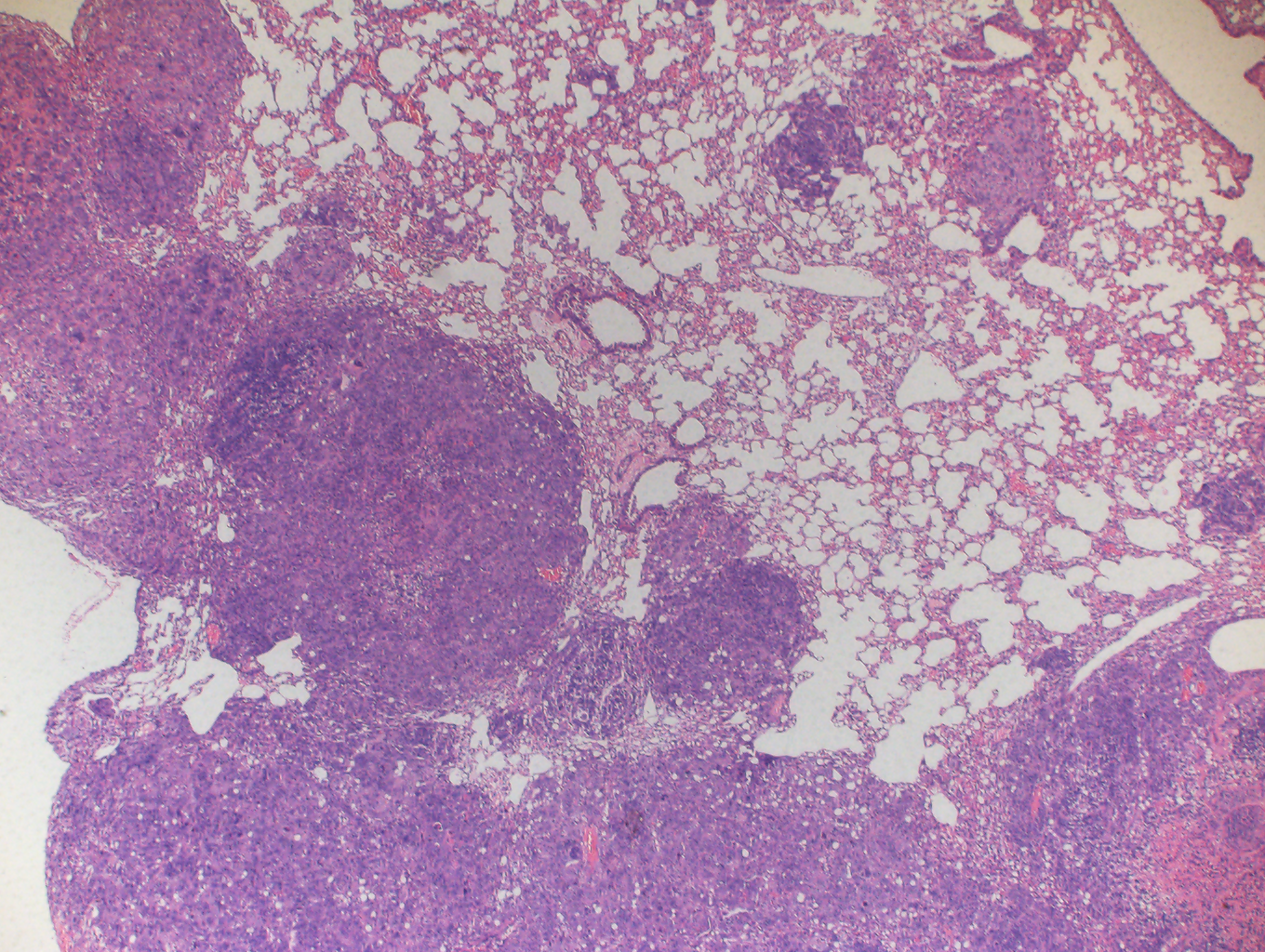

Supplement: Supplementary file 7 — Source data Fig. 6 [file 44318_2025_363_MOESM7_ESM.zip › Figure 6/6C/Ephrin A1 (4).tif]

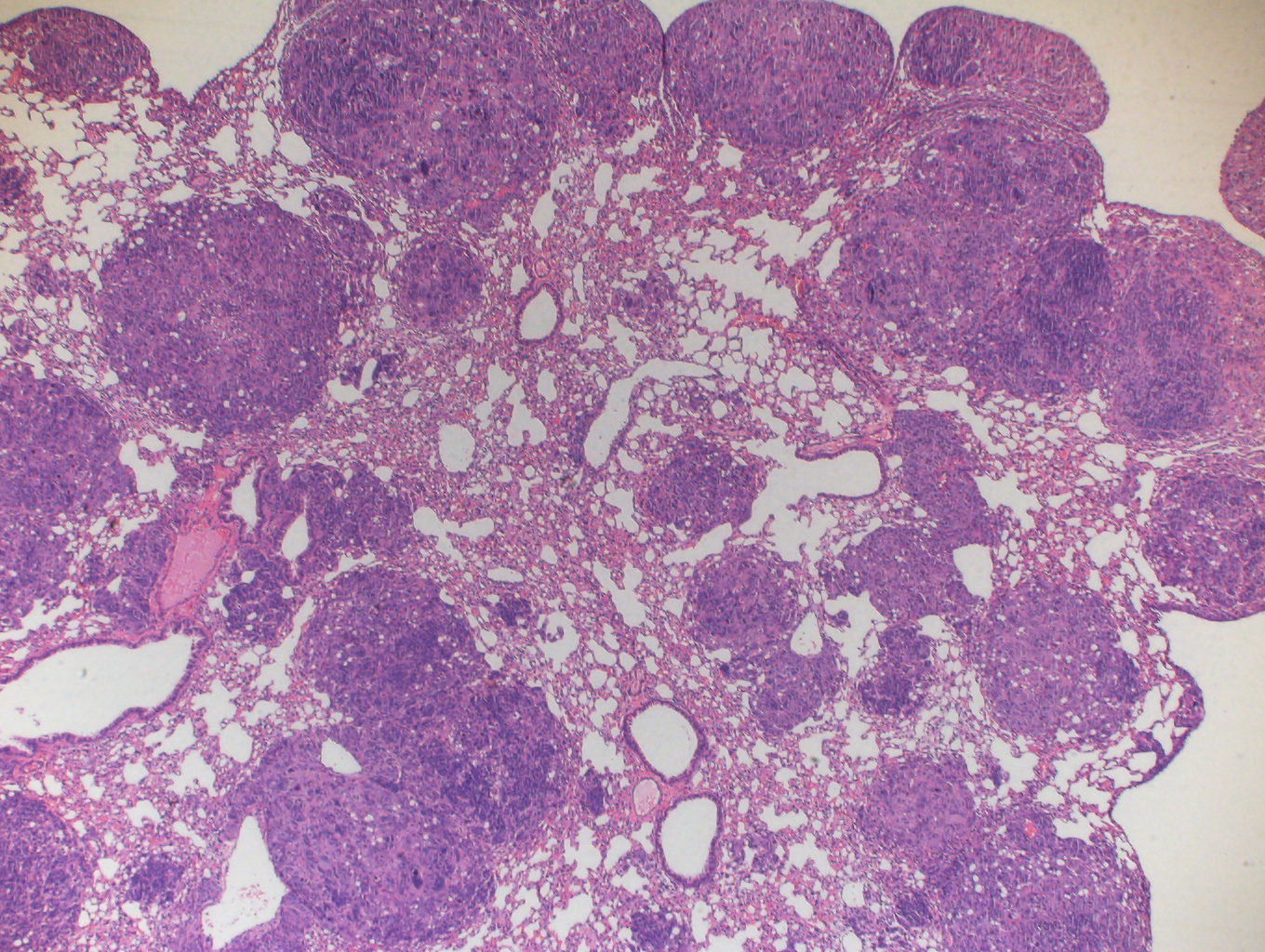

Supplement: Supplementary file 7 — Source data Fig. 6 [file 44318_2025_363_MOESM7_ESM.zip › Figure 6/6C/Ephrin A1 (5)-displayed in 6C.tif]

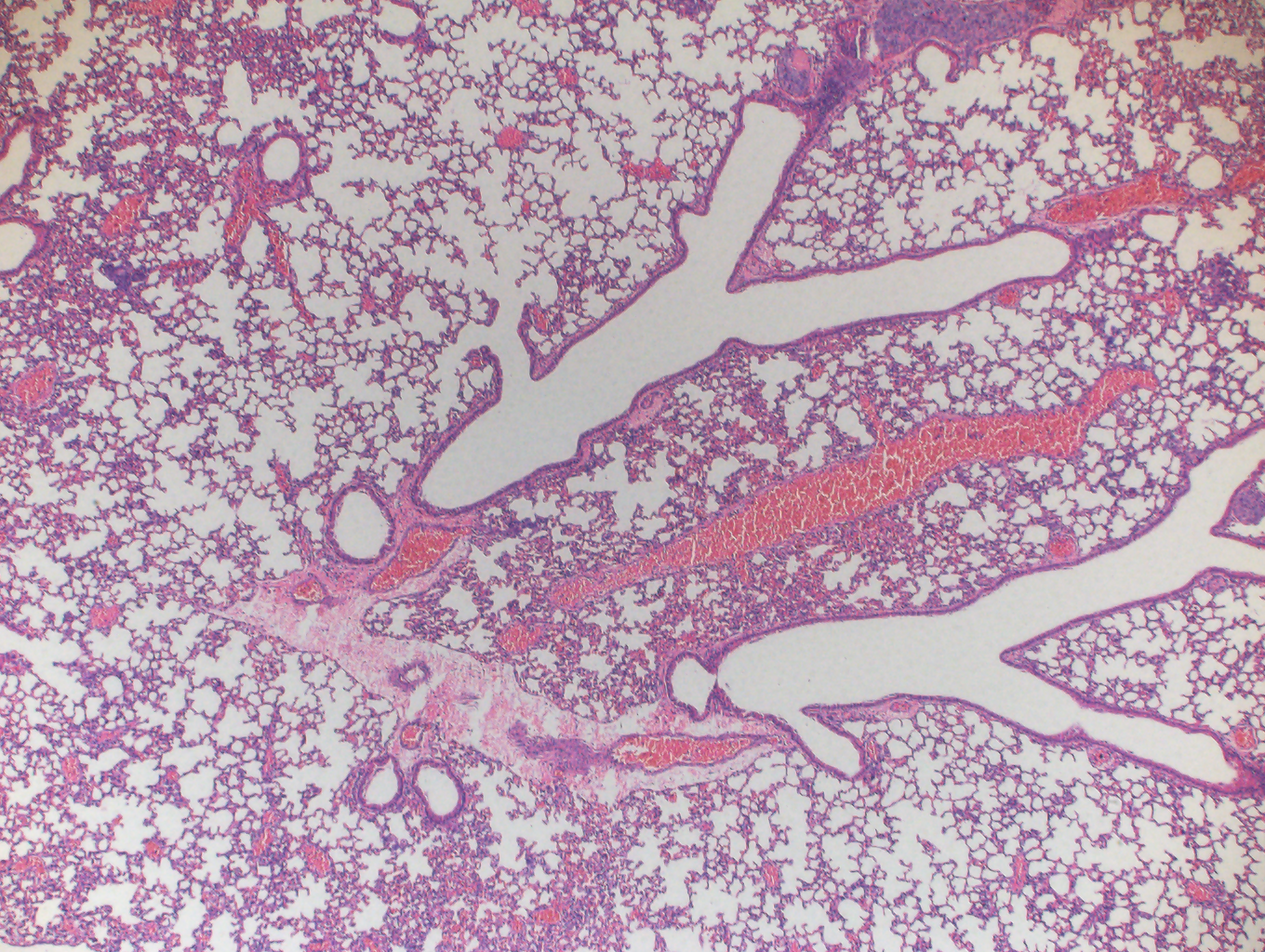

Supplement: Supplementary file 7 — Source data Fig. 6 [file 44318_2025_363_MOESM7_ESM.zip › Figure 6/6C/Ephrin A1+Erlo (1).tif]

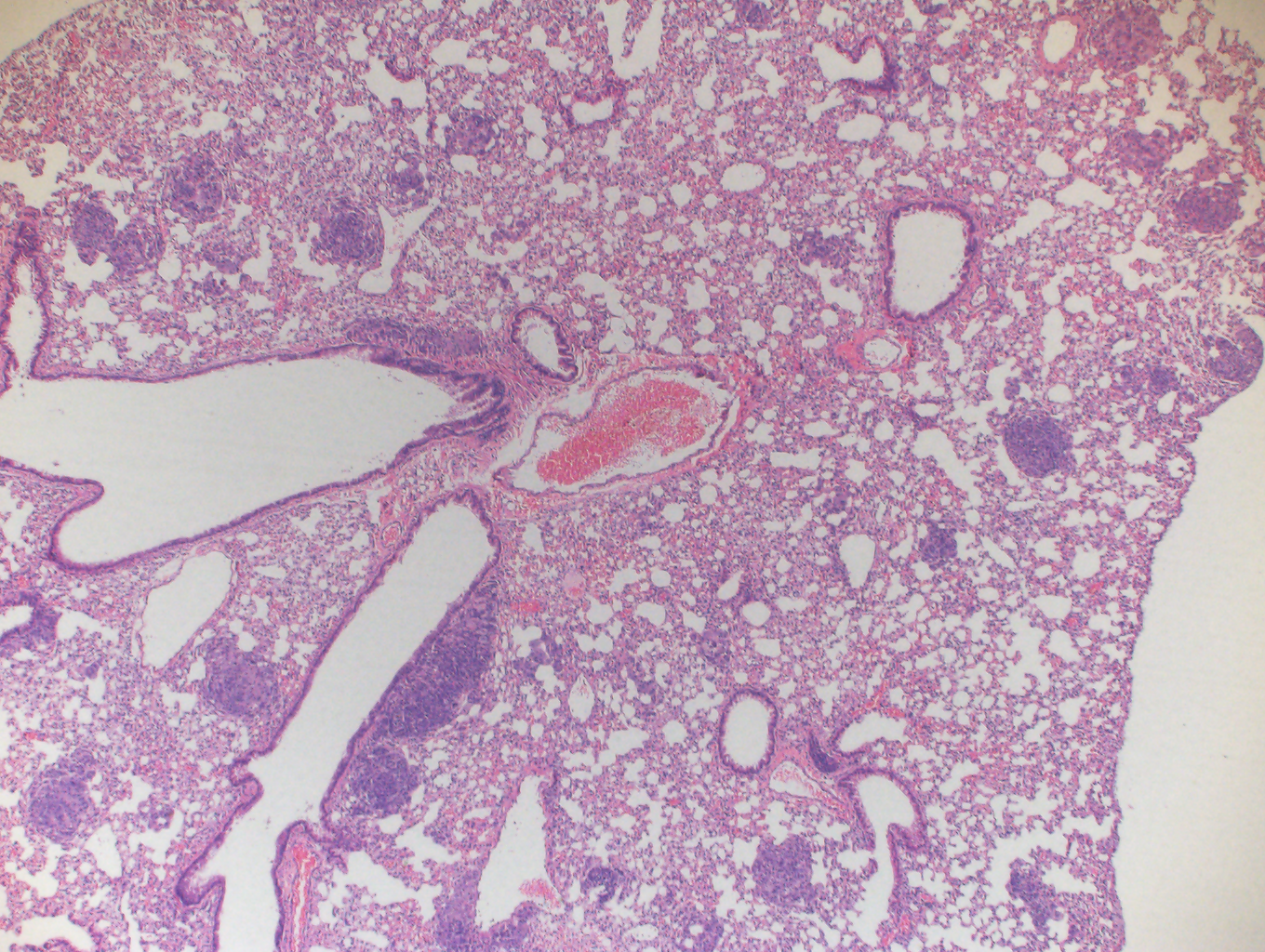

Supplement: Supplementary file 7 — Source data Fig. 6 [file 44318_2025_363_MOESM7_ESM.zip › Figure 6/6C/Ephrin A1+Erlo (2).tif]

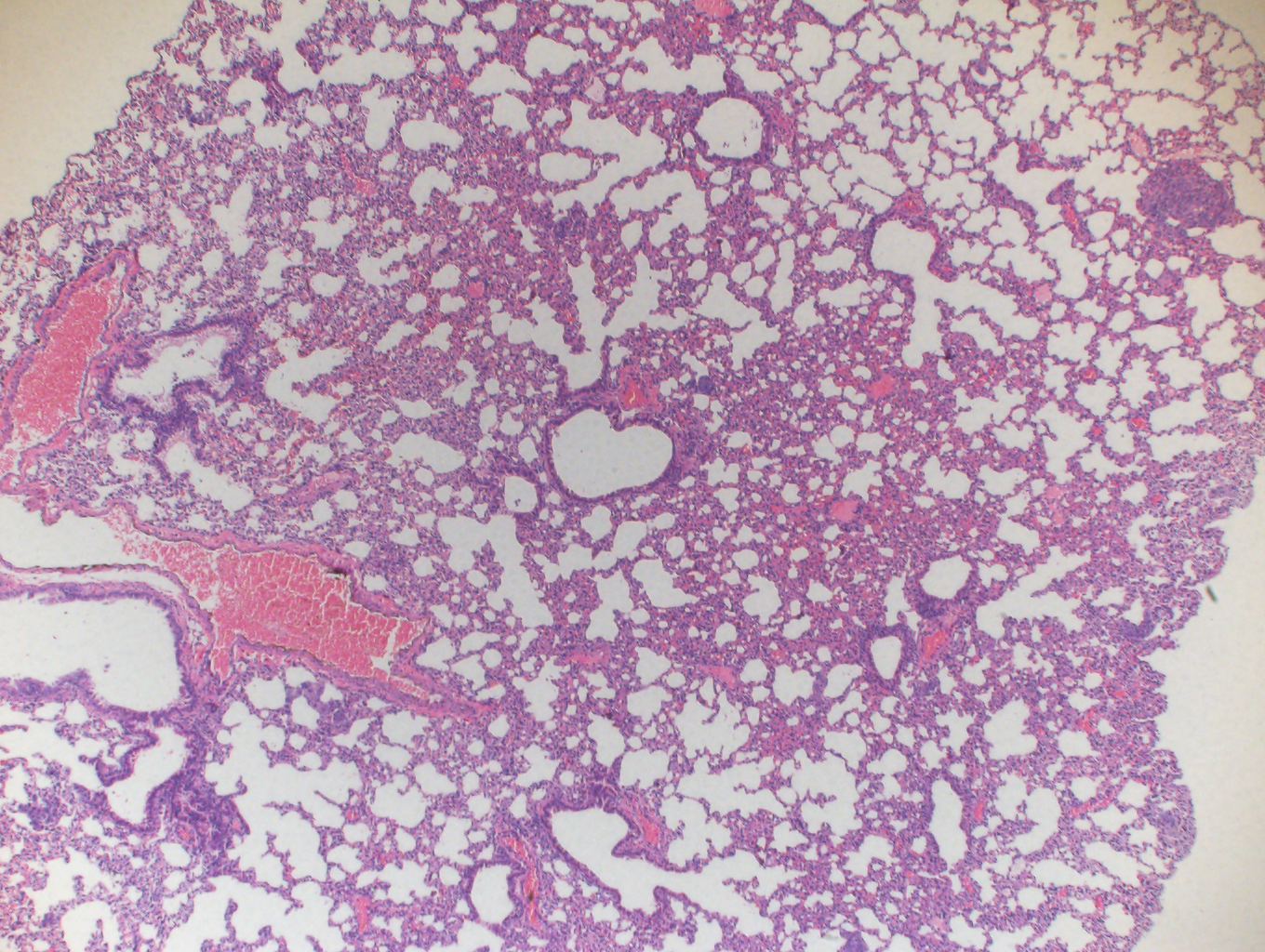

Supplement: Supplementary file 7 — Source data Fig. 6 [file 44318_2025_363_MOESM7_ESM.zip › Figure 6/6C/Ephrin A1+Erlo (3).tif]

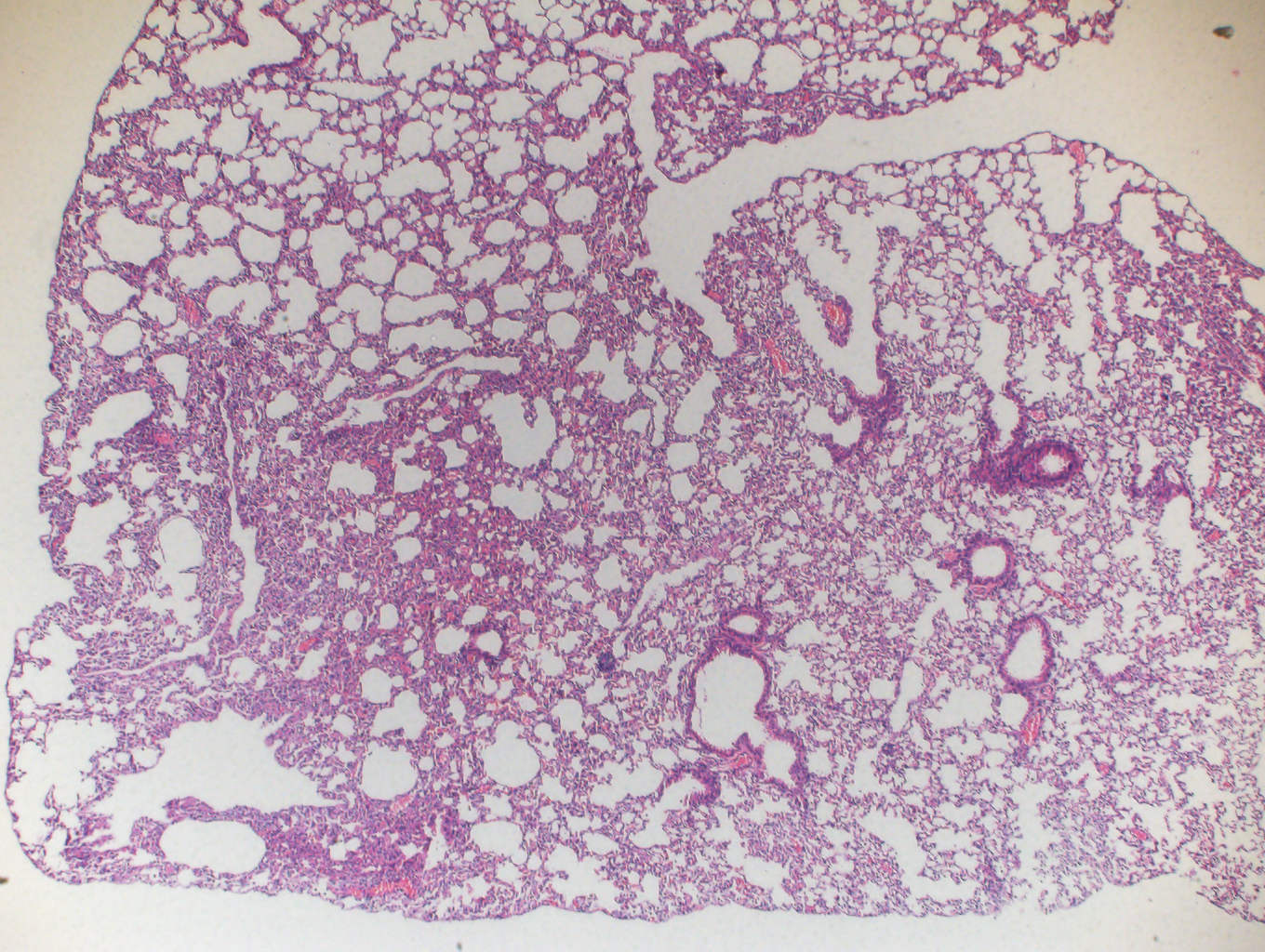

Supplement: Supplementary file 7 — Source data Fig. 6 [file 44318_2025_363_MOESM7_ESM.zip › Figure 6/6C/Ephrin A1+Erlo (4).tif]

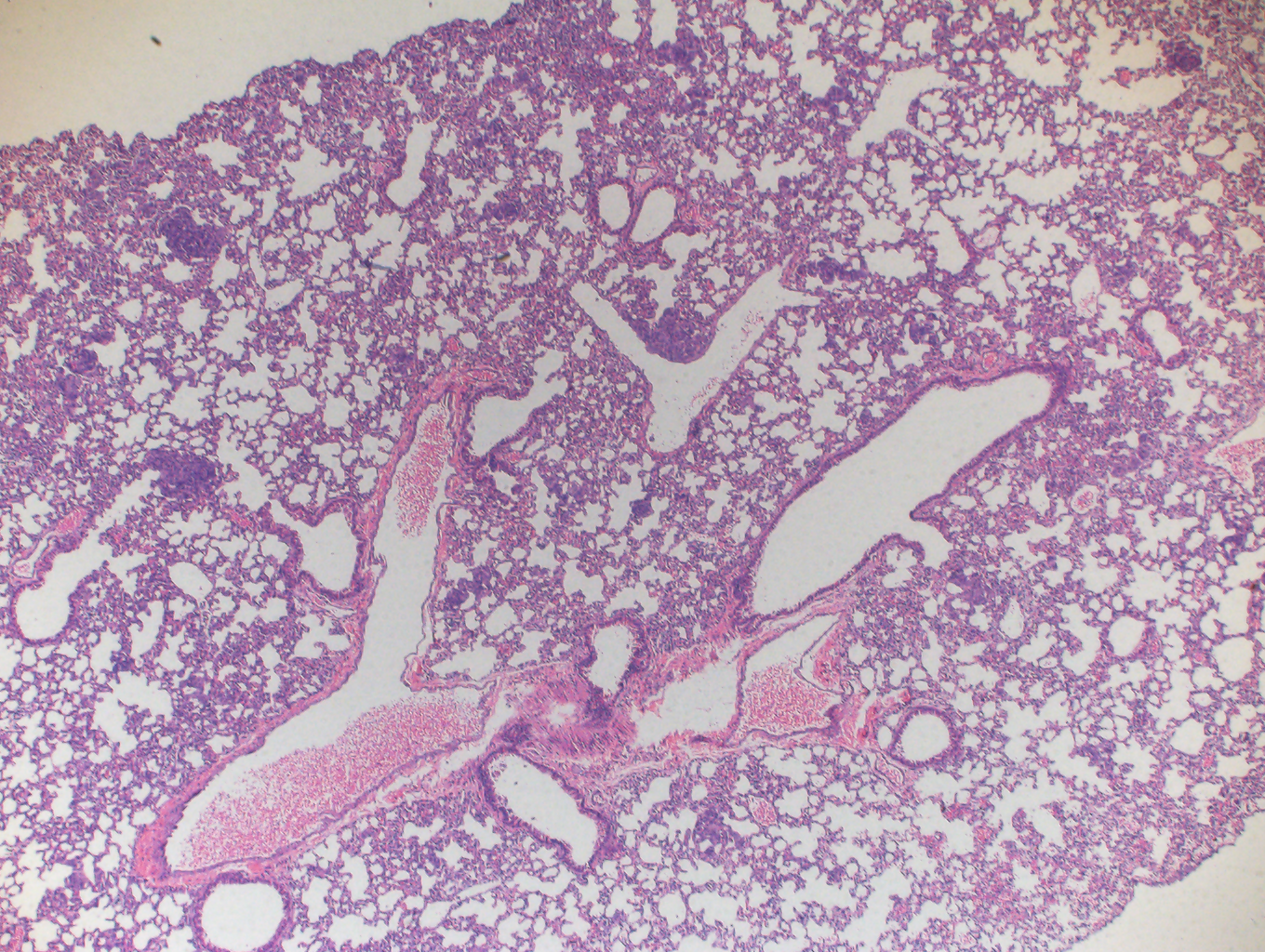

Supplement: Supplementary file 7 — Source data Fig. 6 [file 44318_2025_363_MOESM7_ESM.zip › Figure 6/6C/Ephrin A1+Erlo (5)-displayed in 6C.tif]

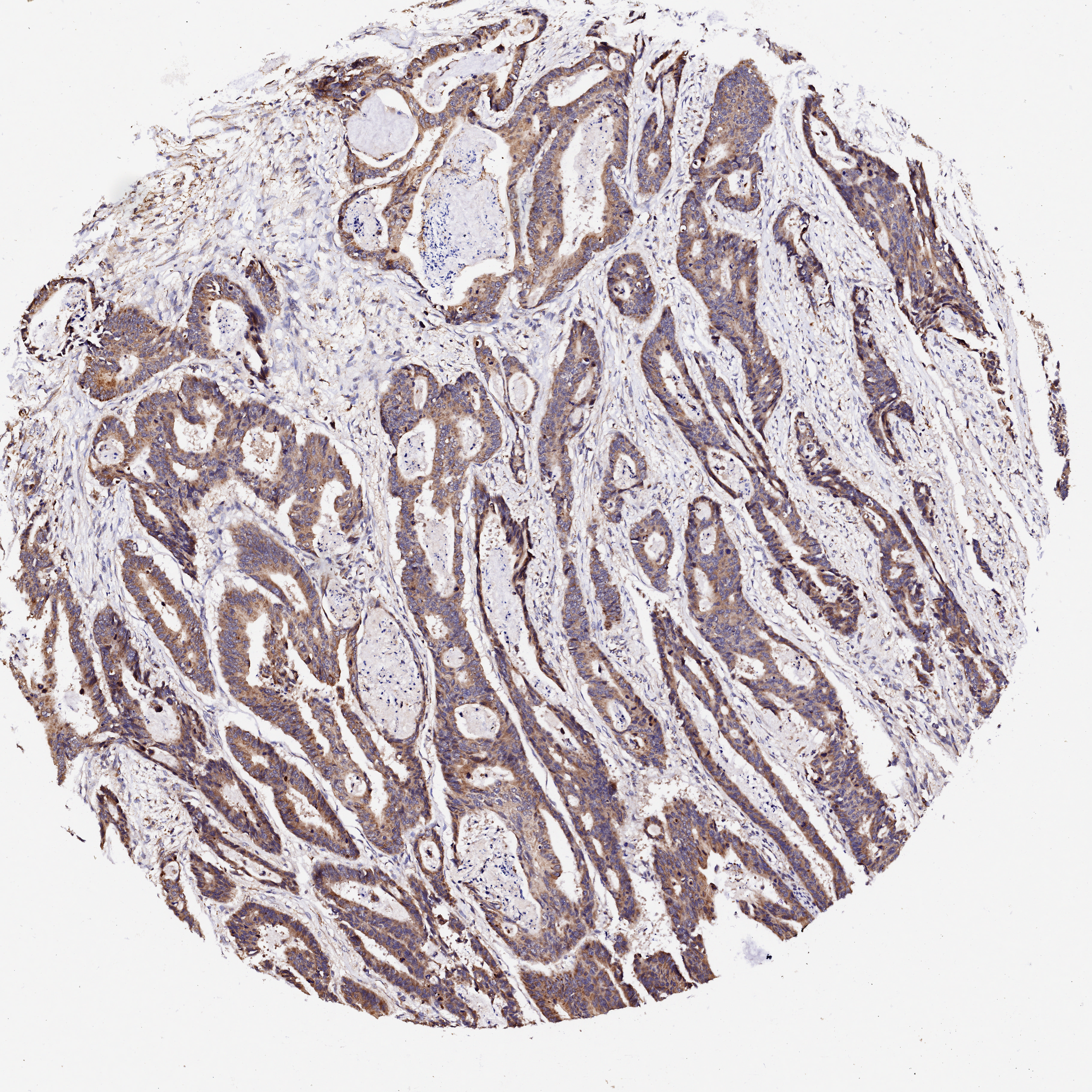

Supplement: Supplementary file 8 — Source data Fig. 7 [file 44318_2025_363_MOESM8_ESM.zip › Figure 7/7A/case 1-Ephrin A1.tif]

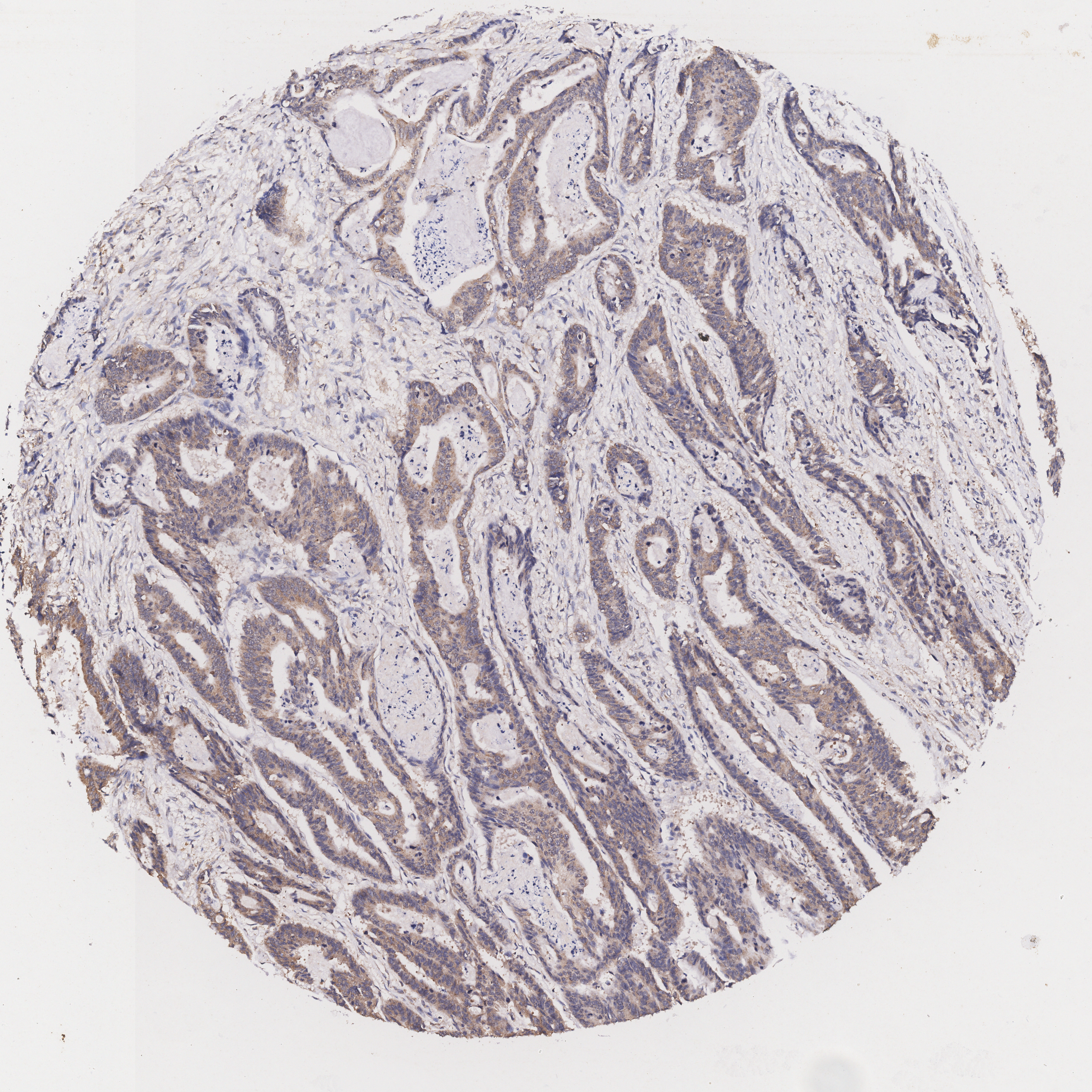

Supplement: Supplementary file 8 — Source data Fig. 7 [file 44318_2025_363_MOESM8_ESM.zip › Figure 7/7A/case 1-p-EGFR.tif]

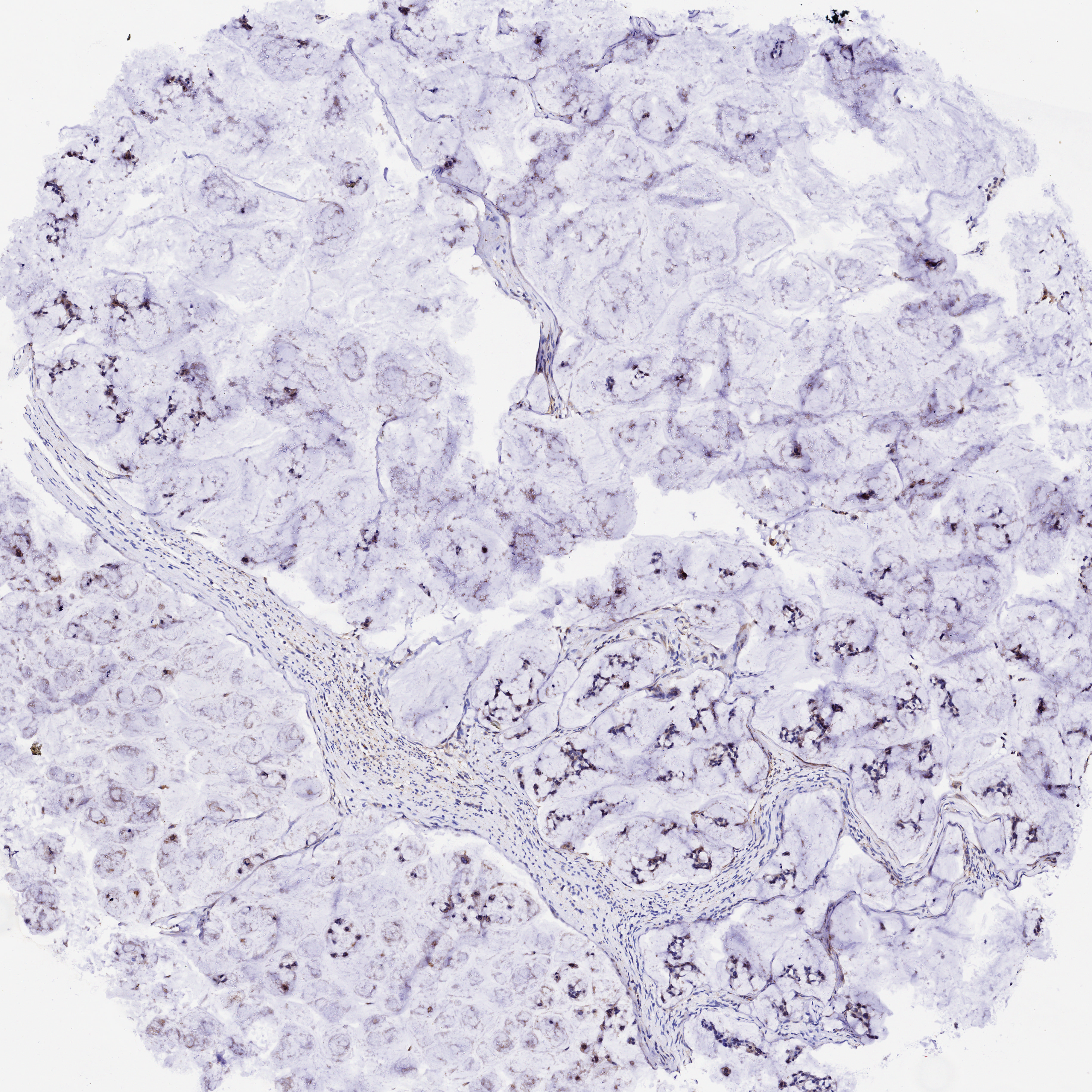

Supplement: Supplementary file 8 — Source data Fig. 7 [file 44318_2025_363_MOESM8_ESM.zip › Figure 7/7A/case 2-Ephrin A1.tif]

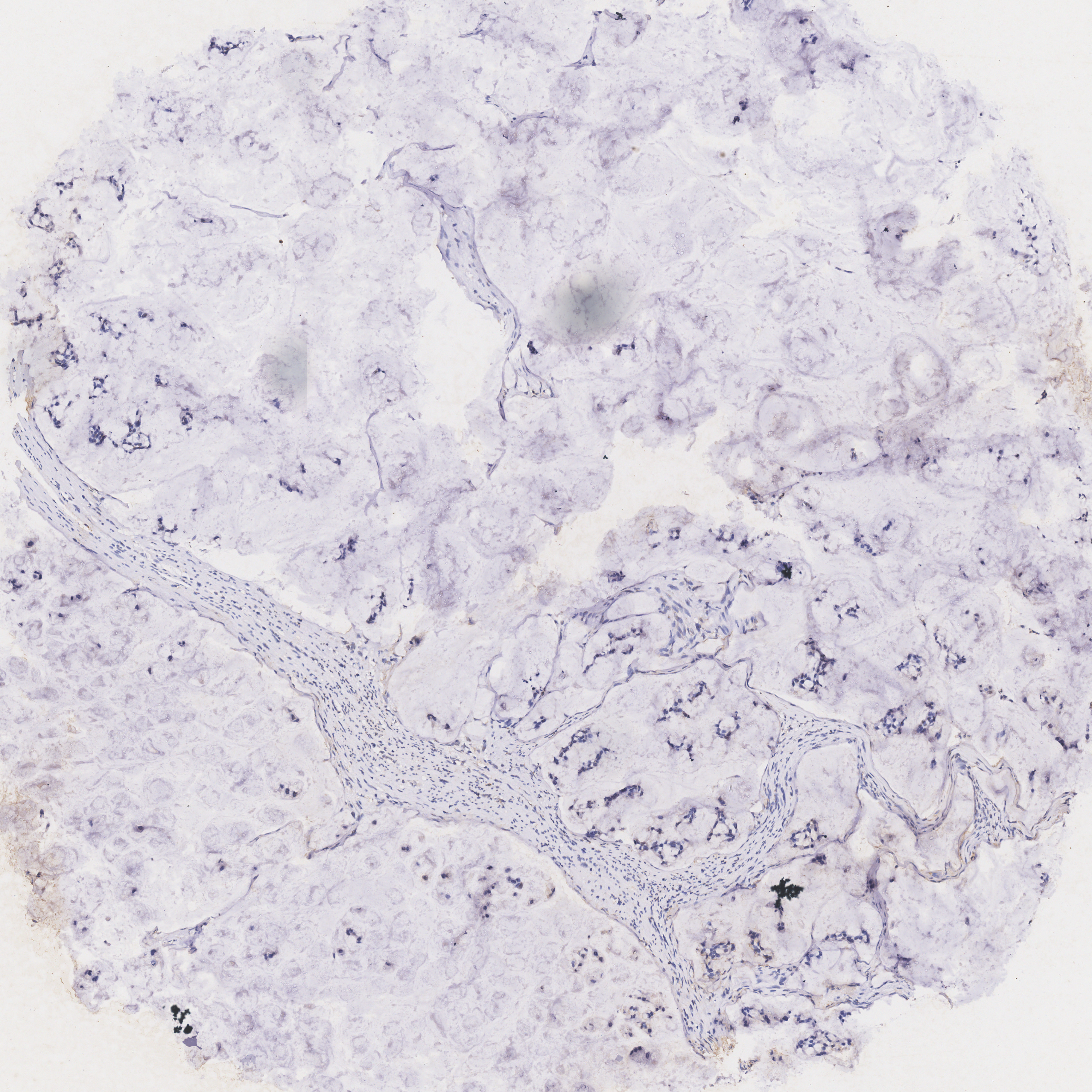

Supplement: Supplementary file 8 — Source data Fig. 7 [file 44318_2025_363_MOESM8_ESM.zip › Figure 7/7A/case 2-p-EGFR.tif]

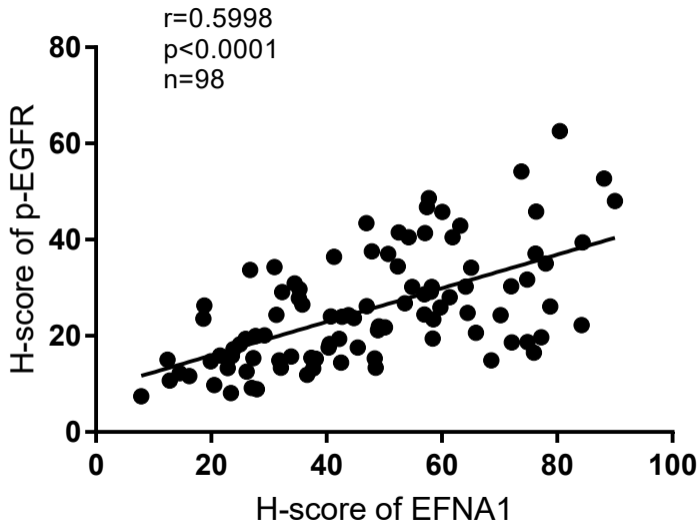

Supplement: Supplementary file 8 — Source data Fig. 7 [file 44318_2025_363_MOESM8_ESM.zip › Figure 7/7B/correlations-Fig 7B.pdf]

EFNA1 + low (n=15) + high (n=79)

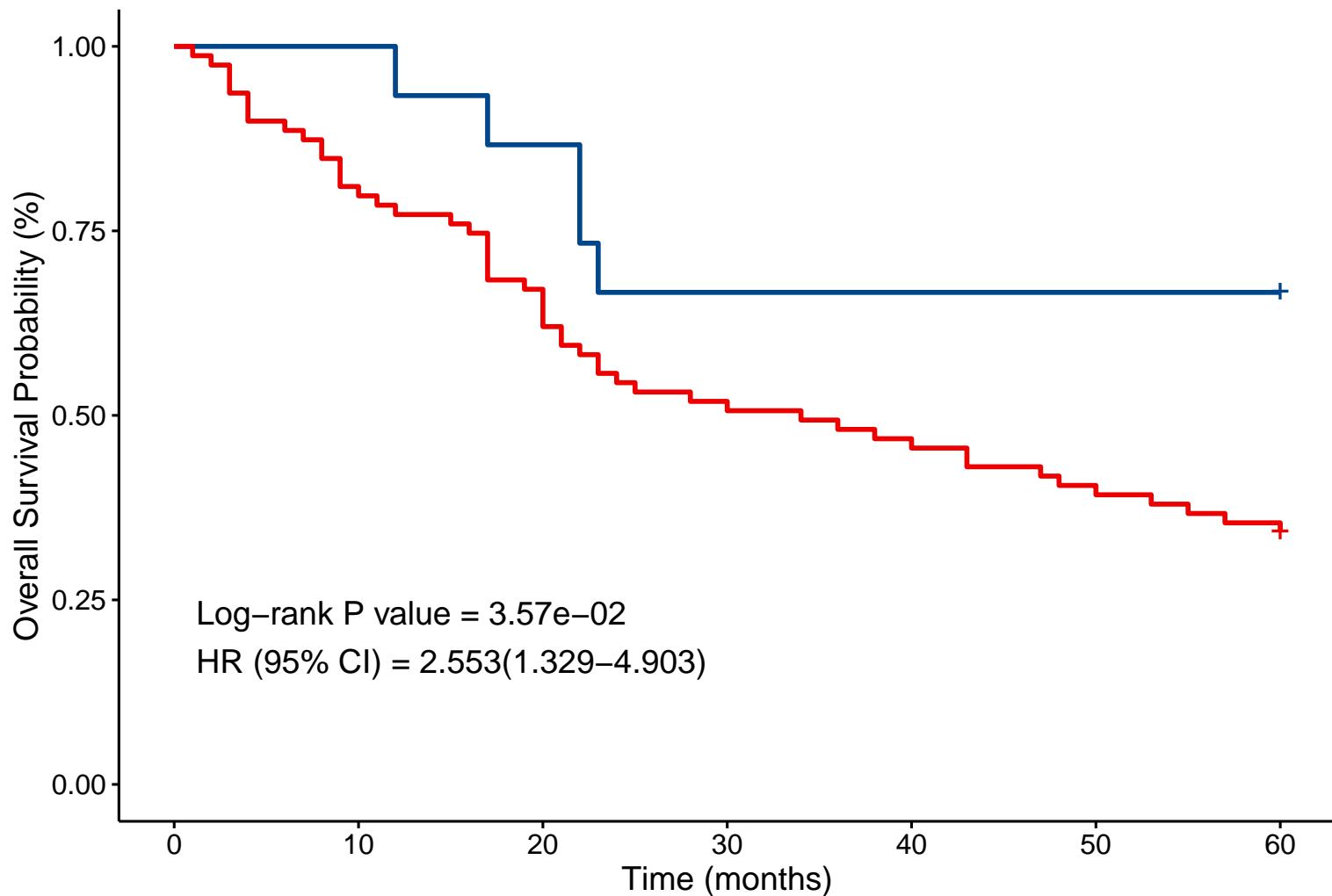

Supplement: Supplementary file 8 — Source data Fig. 7 [file 44318_2025_363_MOESM8_ESM.zip › Figure 7/7C/EFNA1_fiveyearssurvival-Fig 7C.pdf]

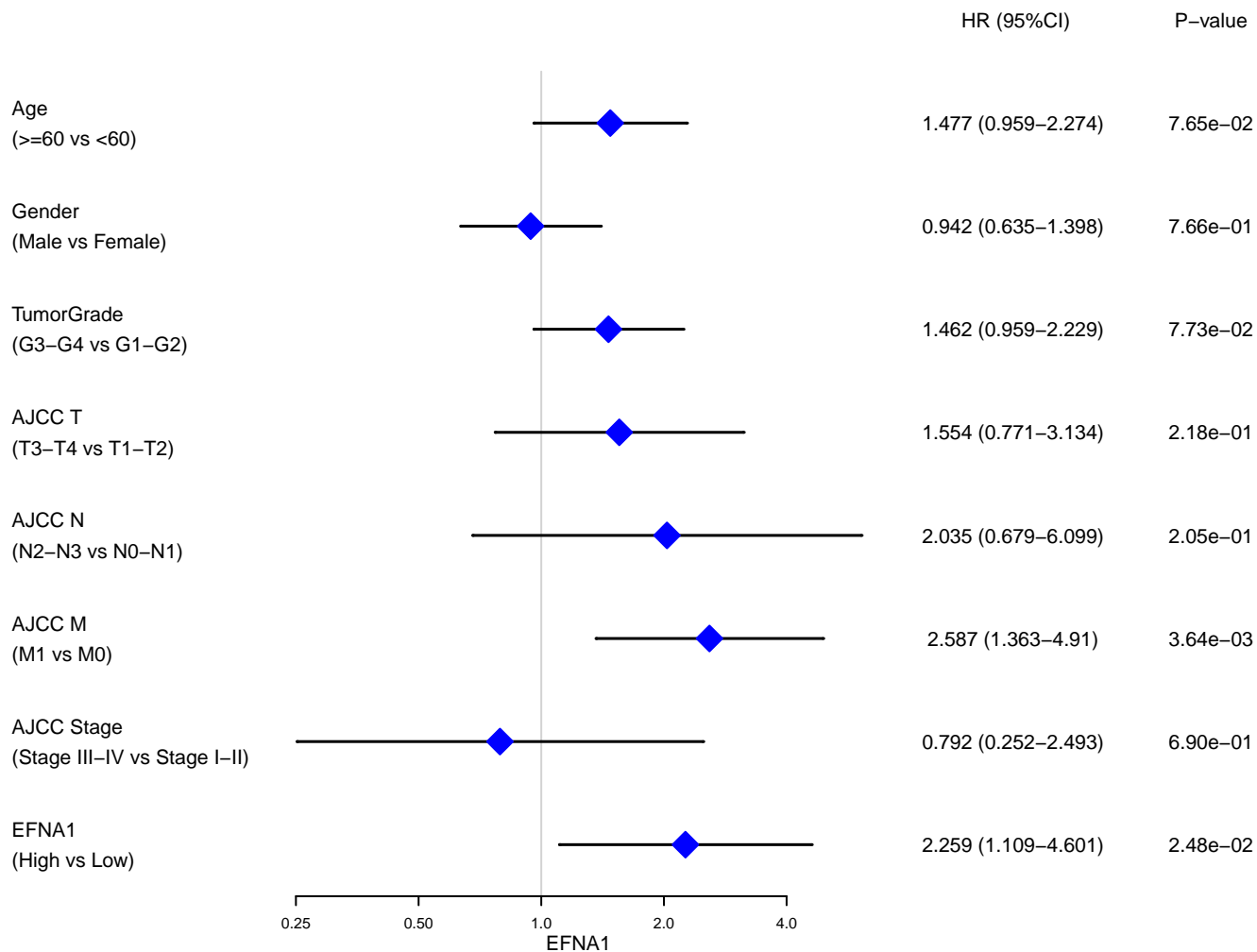

Supplement: Supplementary file 8 — Source data Fig. 7 [file 44318_2025_363_MOESM8_ESM.zip › Figure 7/7D/forestplot_EFNA1-Fig 7D.pdf]

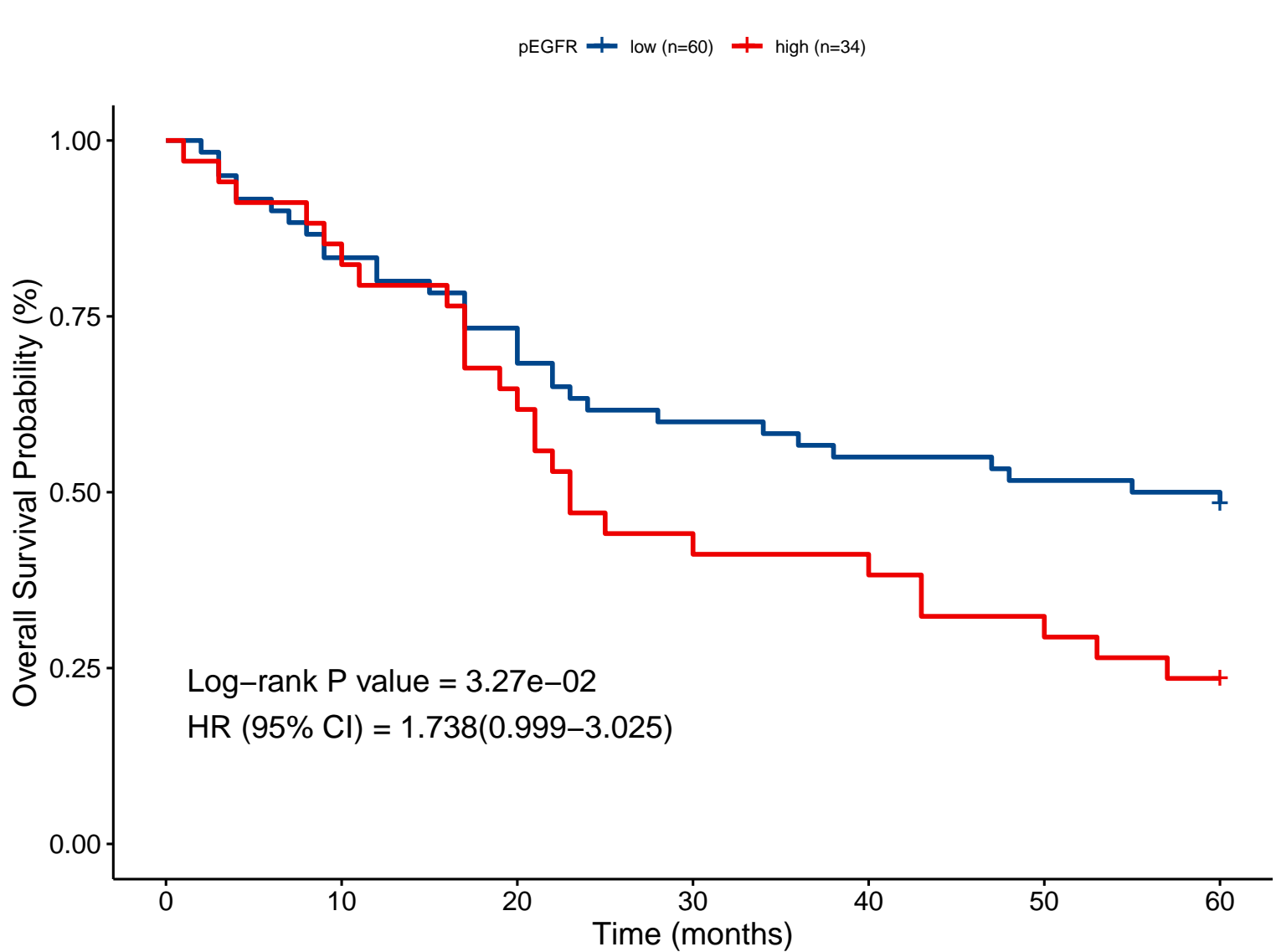

Supplement: Supplementary file 8 — Source data Fig. 7 [file 44318_2025_363_MOESM8_ESM.zip › Figure 7/7E/pEGFR_fiveyearssurvival-Fig 7E.pdf]

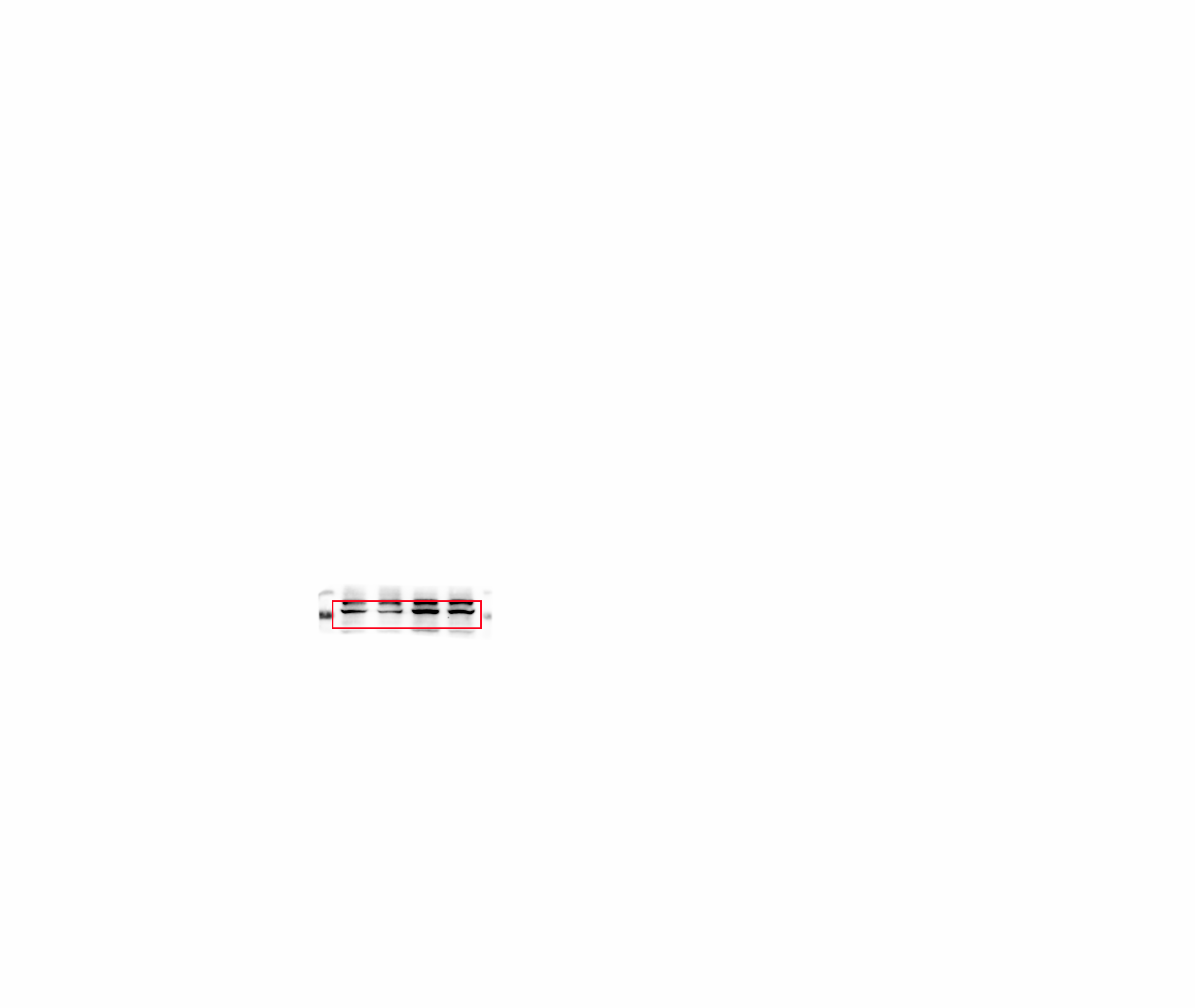

Supplement: Supplementary file 11 — EV Figure Source Data part 3 [file 44318_2025_363_MOESM11_ESM.zip › Figure EV6/EV6A/1 E-cad.tif]
