# Supplementary figures and images for: Ephrin A1 functions as a ligand of EGFR to promote EMT and metastasis in gastric cancer (part 5 of 5)
Source: EMBO J. 2025 Jan 21;44(5):1464–87. doi: 10.1038/s44318-025-00363-x (PMC11876641; doi:10.1038/s44318-025-00363-x)

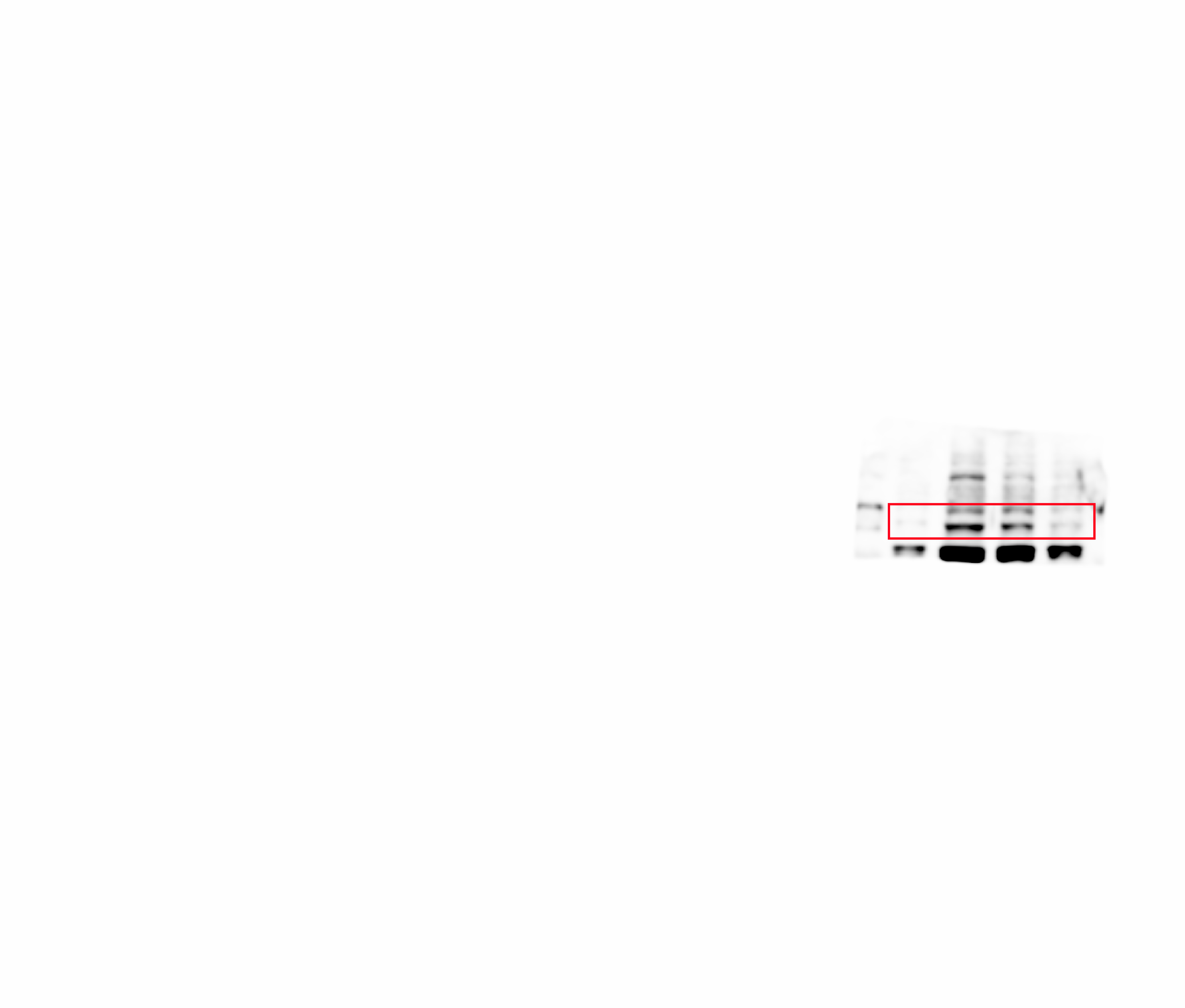

Supplement: Supplementary file 11 — EV Figure Source Data part 3 [file 44318_2025_363_MOESM11_ESM.zip › Figure EV6/EV6A/2 N-cad.tif]

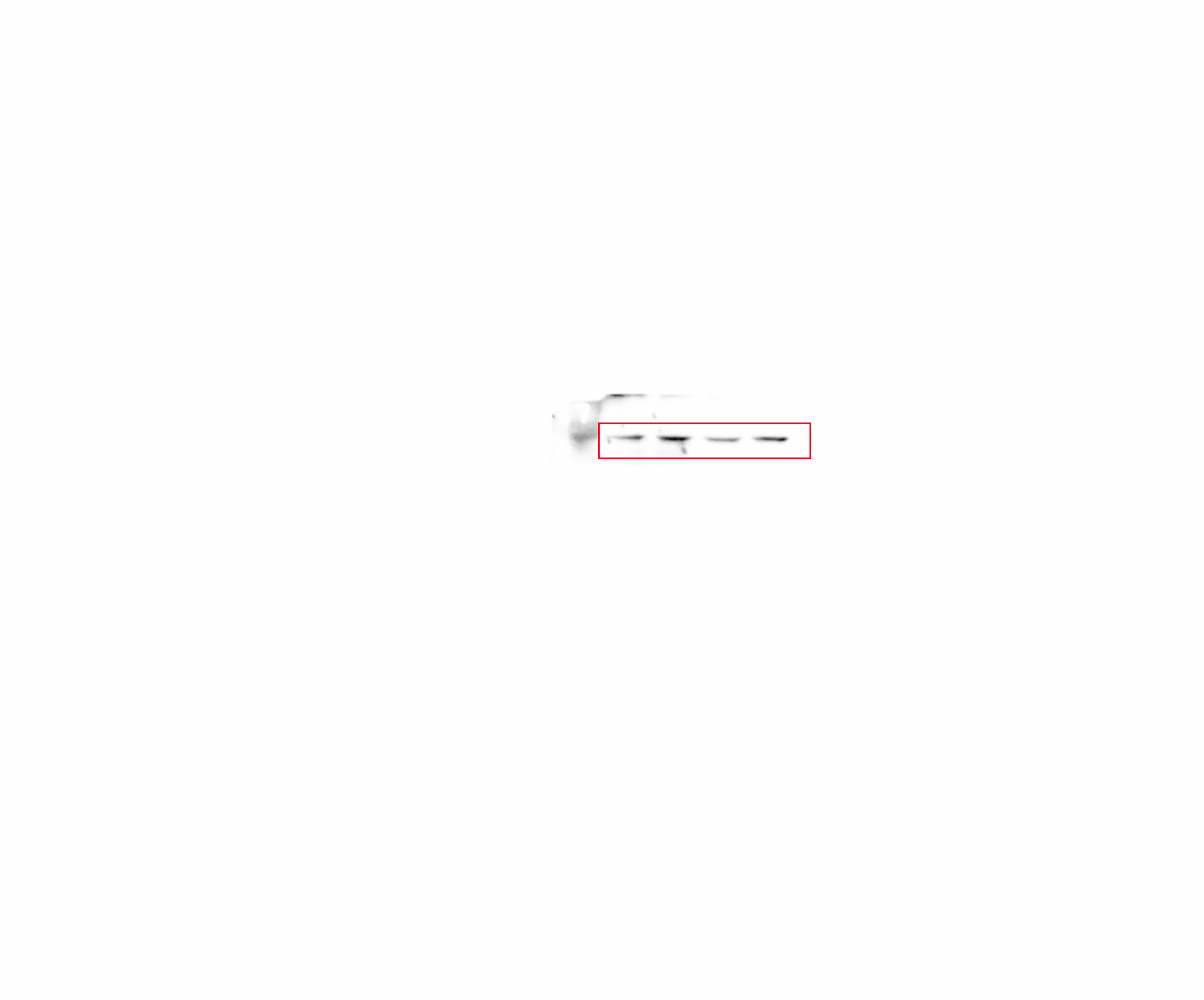

Supplement: Supplementary file 11 — EV Figure Source Data part 3 [file 44318_2025_363_MOESM11_ESM.zip › Figure EV6/EV6A/3 vimentin.tif]

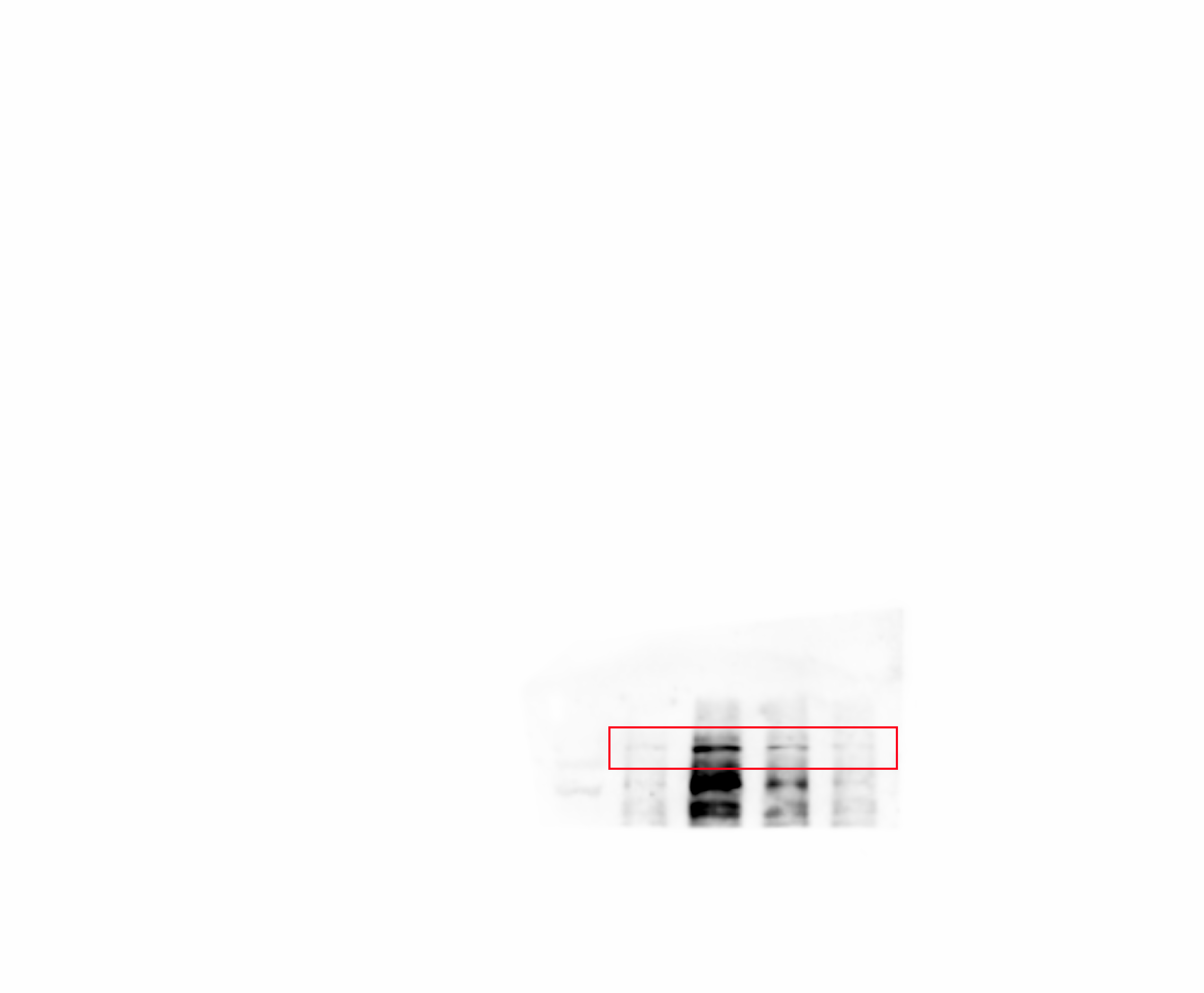

Supplement: Supplementary file 11 — EV Figure Source Data part 3 [file 44318_2025_363_MOESM11_ESM.zip › Figure EV6/EV6A/4 ZEB1.tif]

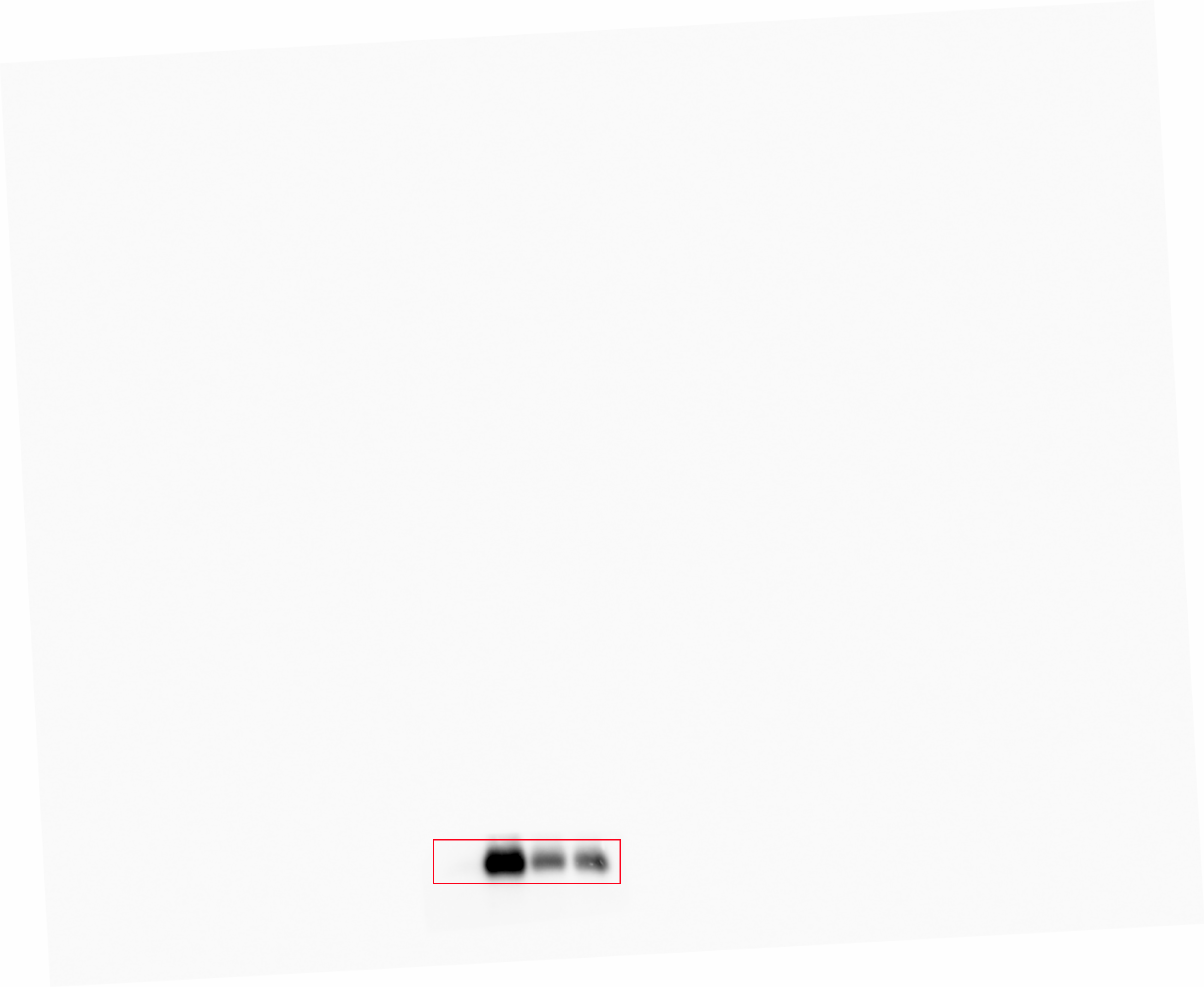

Supplement: Supplementary file 11 — EV Figure Source Data part 3 [file 44318_2025_363_MOESM11_ESM.zip › Figure EV6/EV6A/5 Ephrin A1.tif]

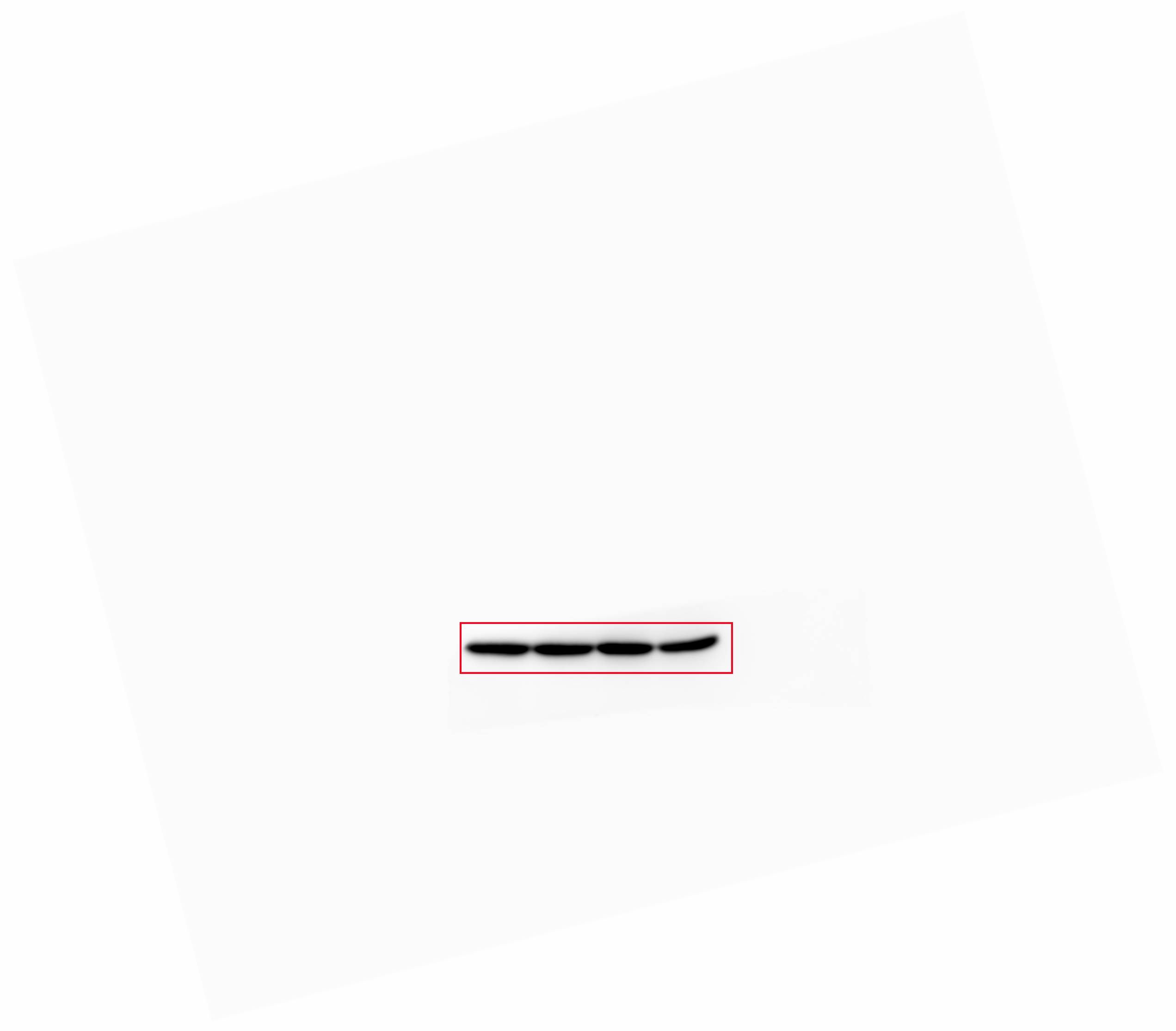

Supplement: Supplementary file 11 — EV Figure Source Data part 3 [file 44318_2025_363_MOESM11_ESM.zip › Figure EV6/EV6A/6 actin.tif]

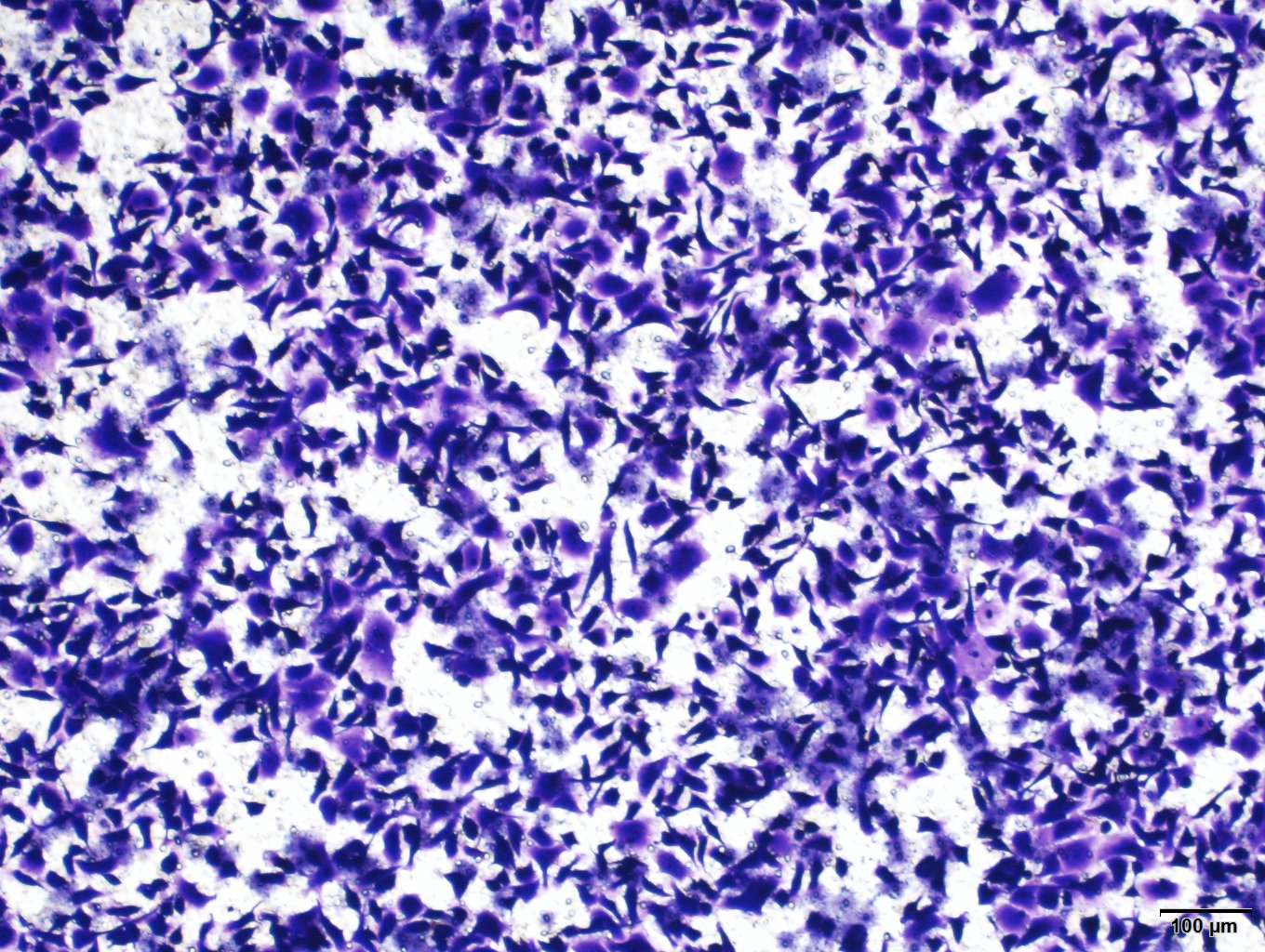

Supplement: Supplementary file 11 — EV Figure Source Data part 3 [file 44318_2025_363_MOESM11_ESM.zip › Figure EV6/EV6B/Control (1).jpg]

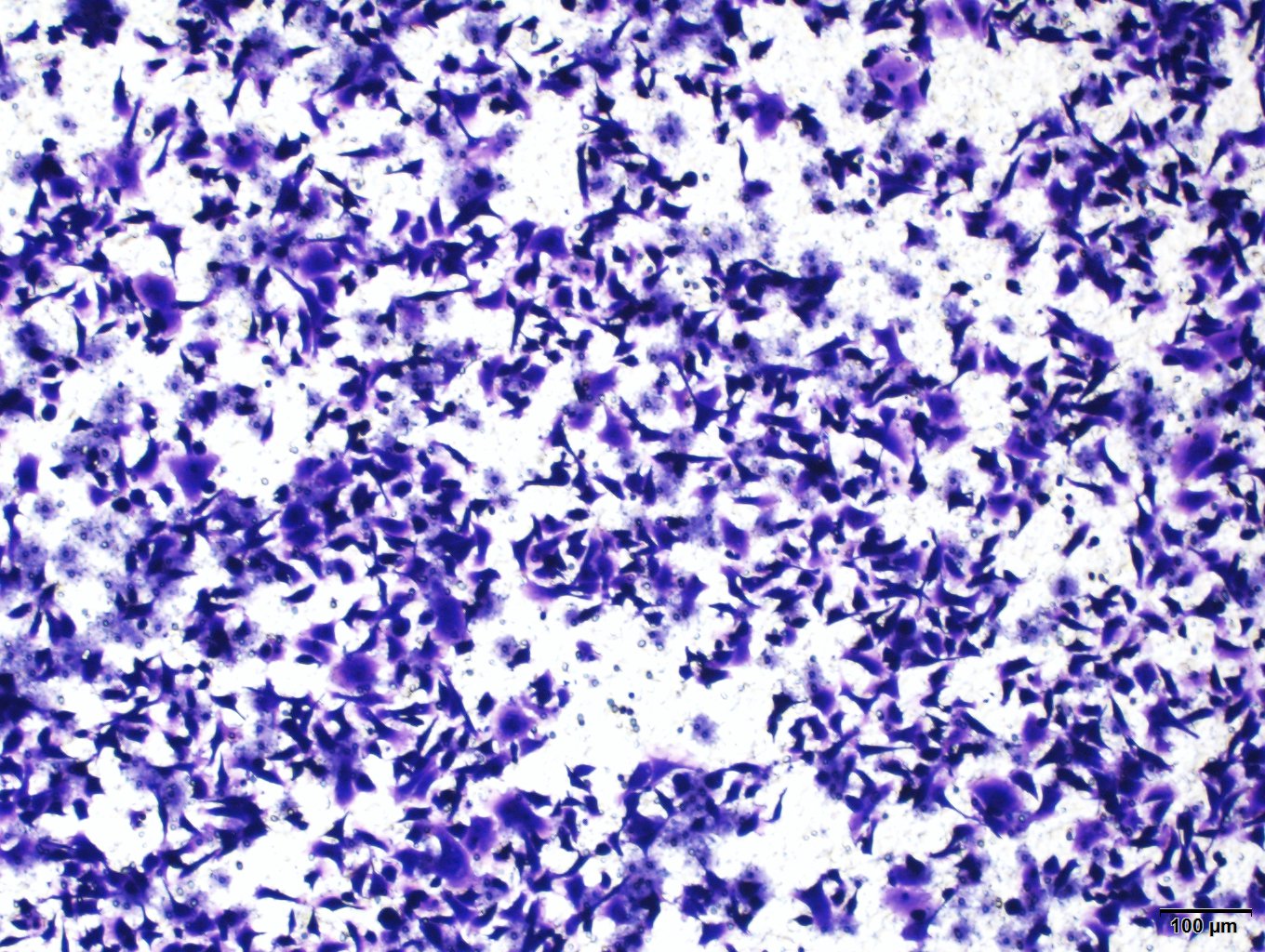

Supplement: Supplementary file 11 — EV Figure Source Data part 3 [file 44318_2025_363_MOESM11_ESM.zip › Figure EV6/EV6B/Control (2)-displayed in EV6B.jpg]

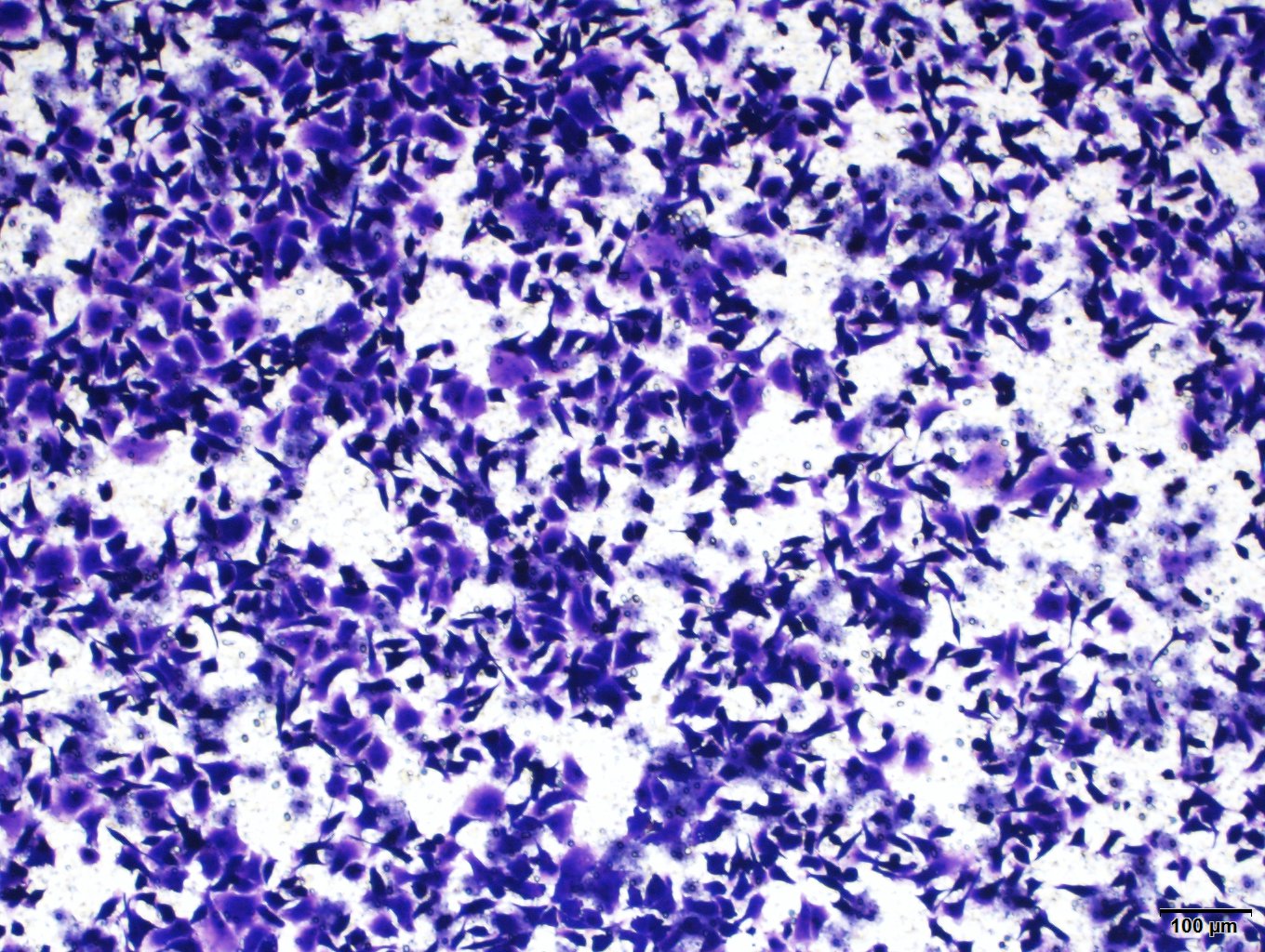

Supplement: Supplementary file 11 — EV Figure Source Data part 3 [file 44318_2025_363_MOESM11_ESM.zip › Figure EV6/EV6B/Control (3).jpg]

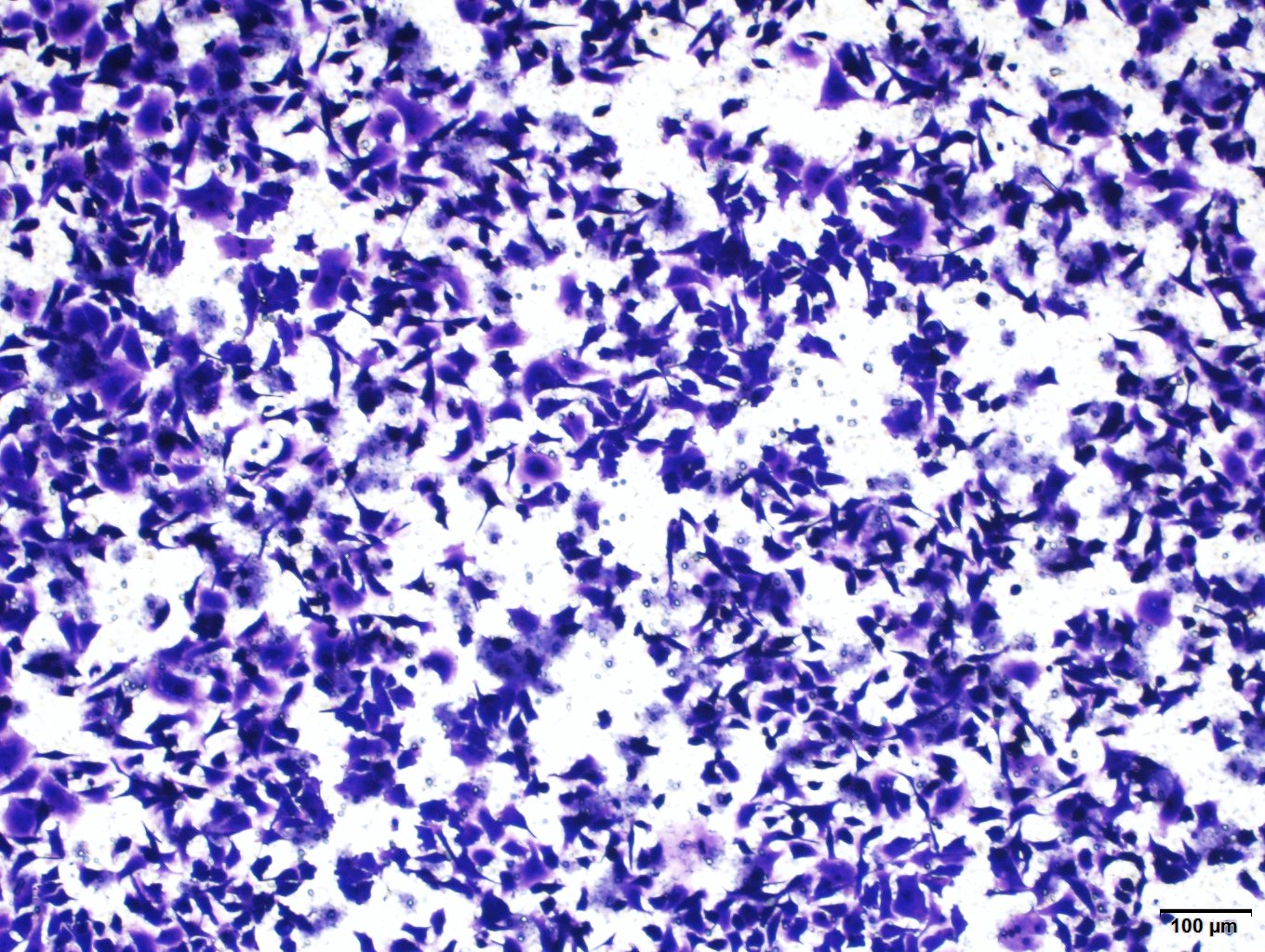

Supplement: Supplementary file 11 — EV Figure Source Data part 3 [file 44318_2025_363_MOESM11_ESM.zip › Figure EV6/EV6B/Control (4).jpg]

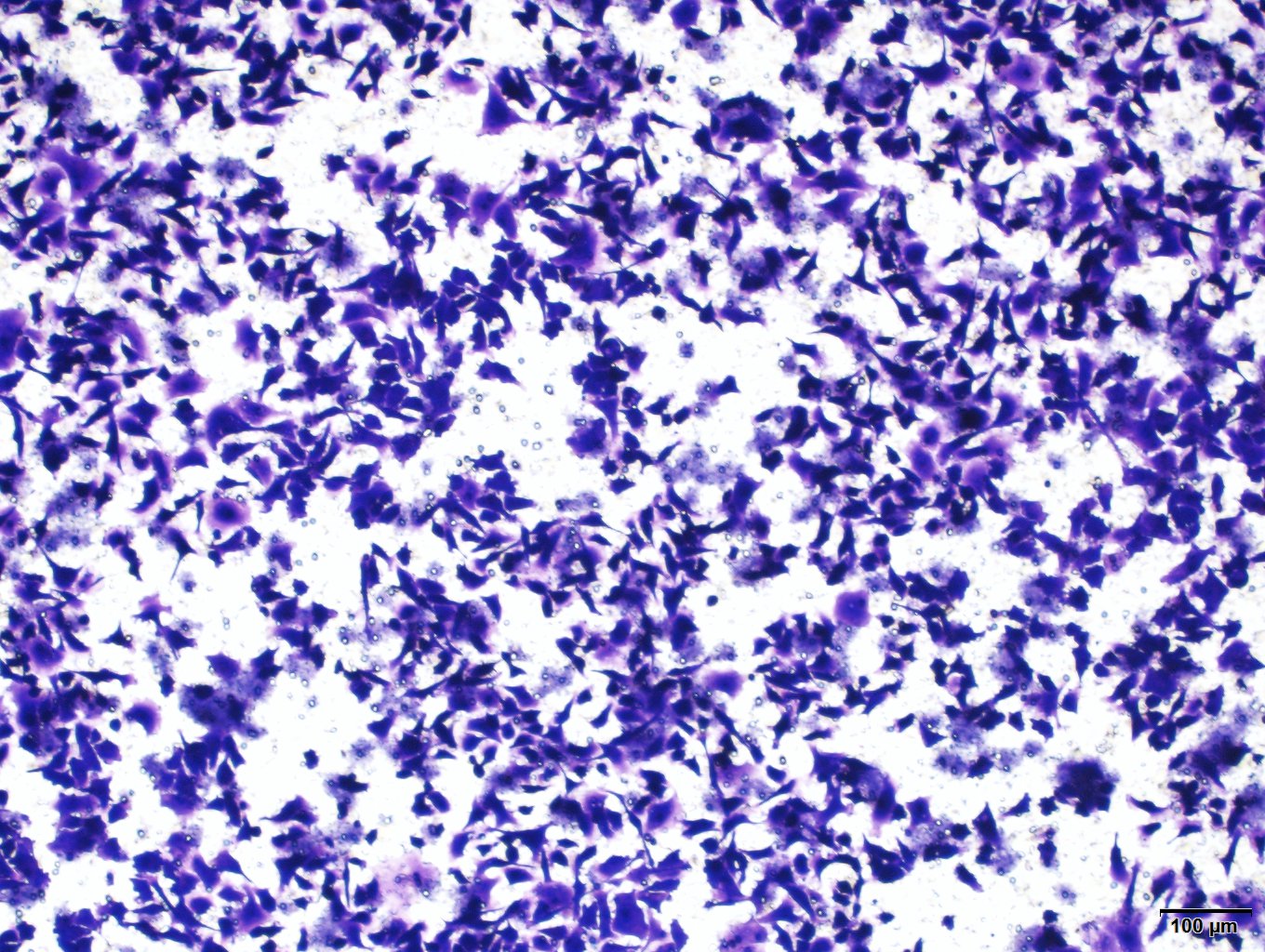

Supplement: Supplementary file 11 — EV Figure Source Data part 3 [file 44318_2025_363_MOESM11_ESM.zip › Figure EV6/EV6B/Control (5).jpg]

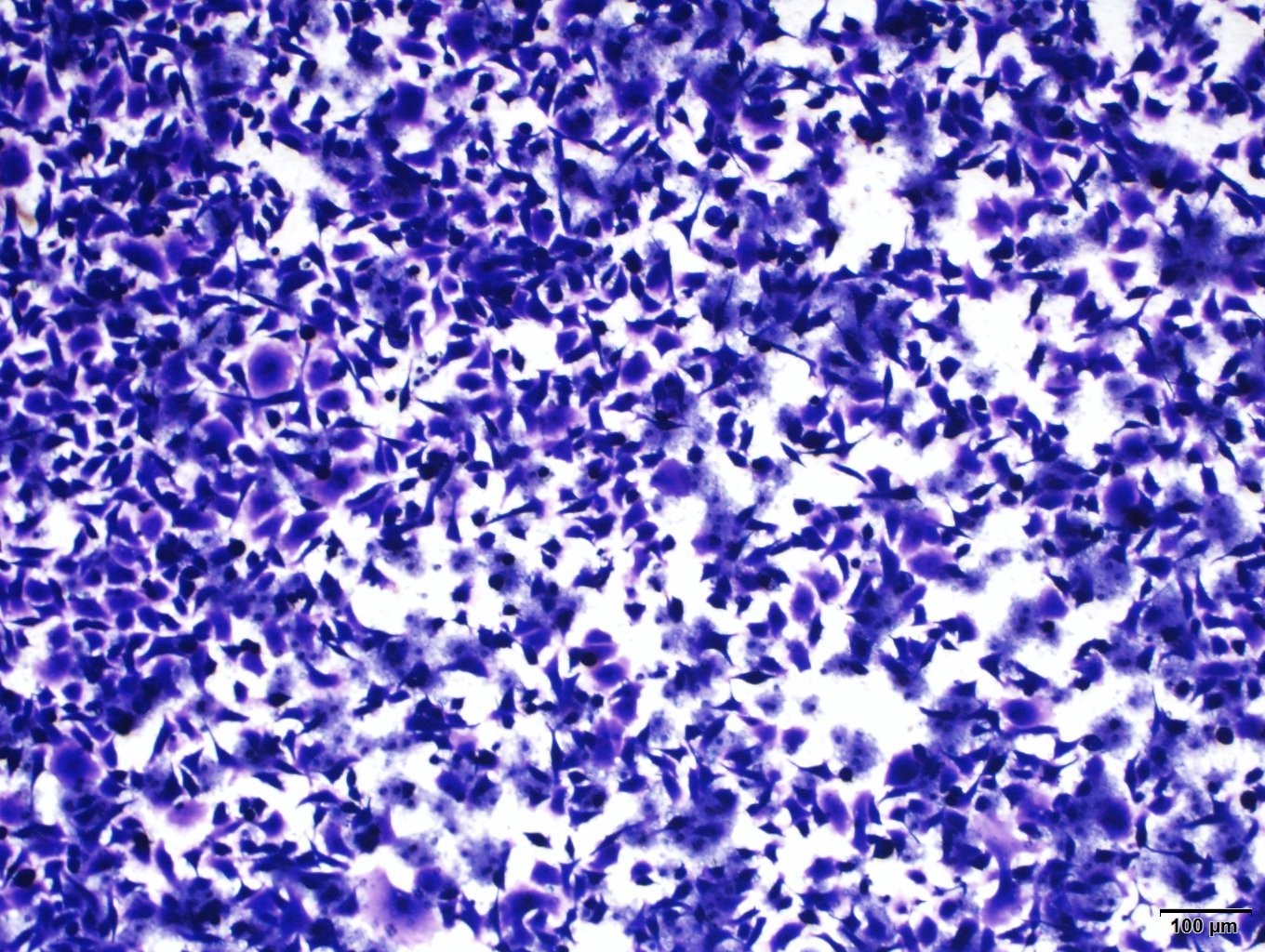

Supplement: Supplementary file 11 — EV Figure Source Data part 3 [file 44318_2025_363_MOESM11_ESM.zip › Figure EV6/EV6B/Ephrin A1 (1).jpg]

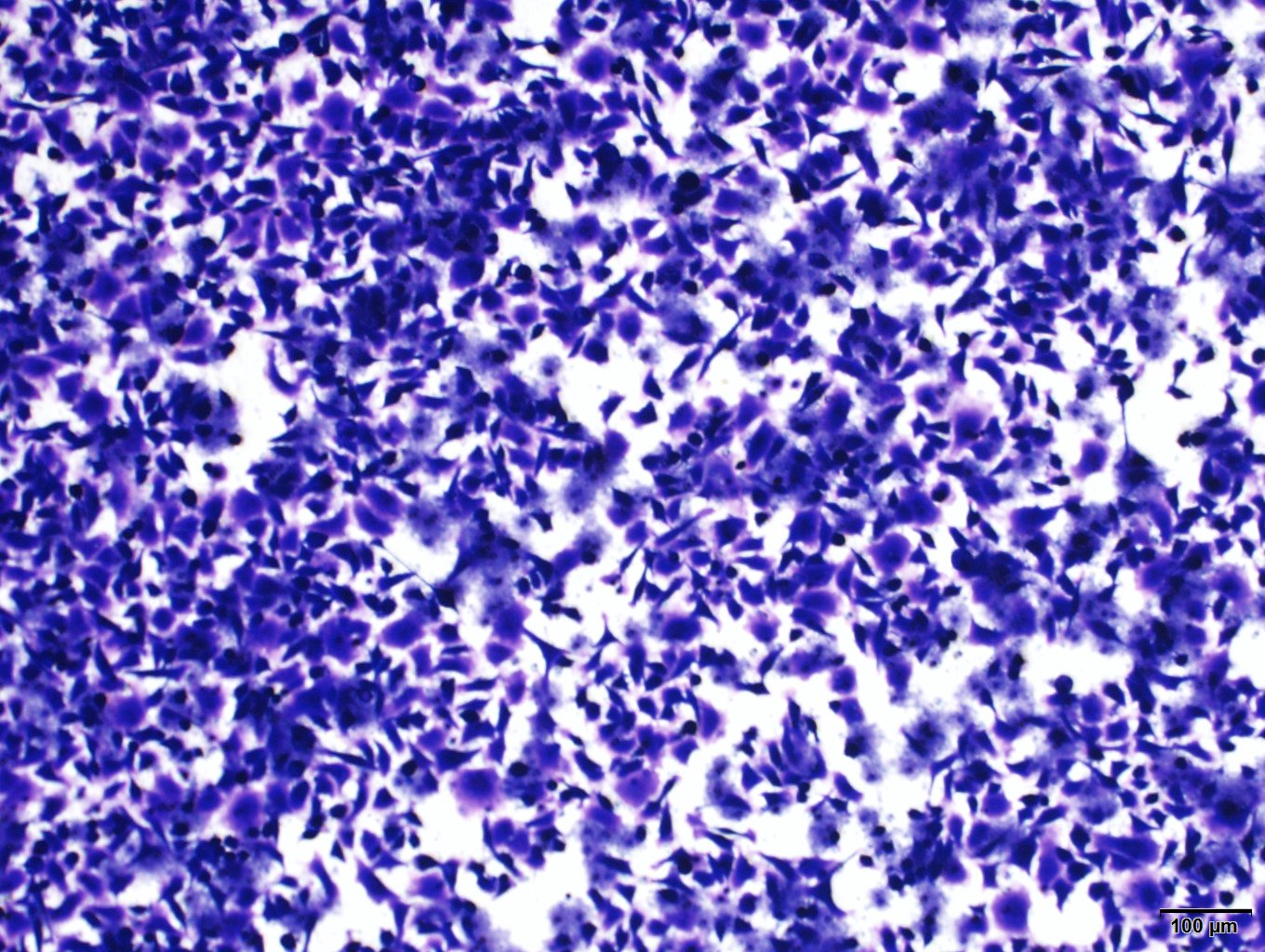

Supplement: Supplementary file 11 — EV Figure Source Data part 3 [file 44318_2025_363_MOESM11_ESM.zip › Figure EV6/EV6B/Ephrin A1 (2).jpg]

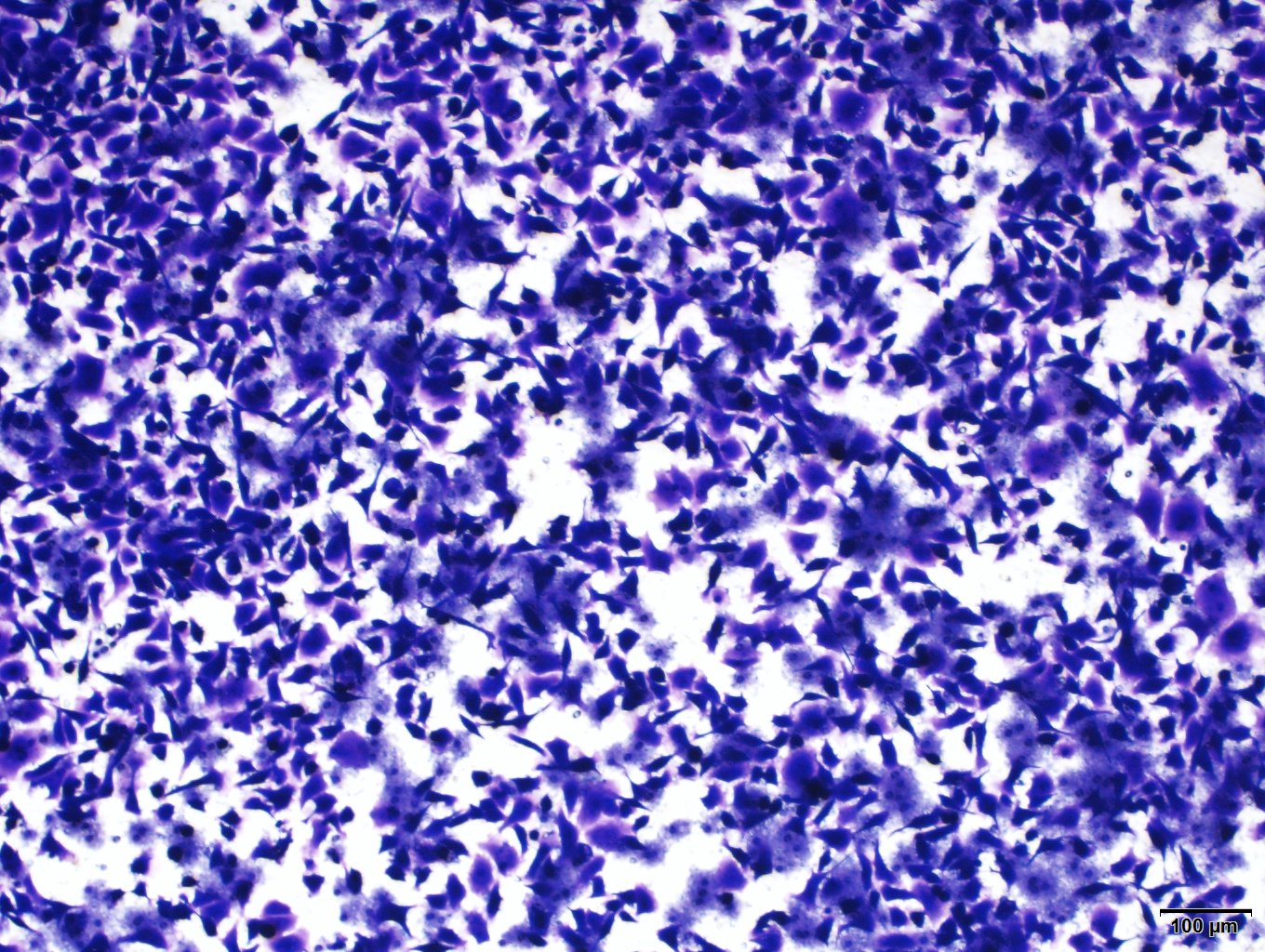

Supplement: Supplementary file 11 — EV Figure Source Data part 3 [file 44318_2025_363_MOESM11_ESM.zip › Figure EV6/EV6B/Ephrin A1 (3)-displayed in EV6B.jpg]

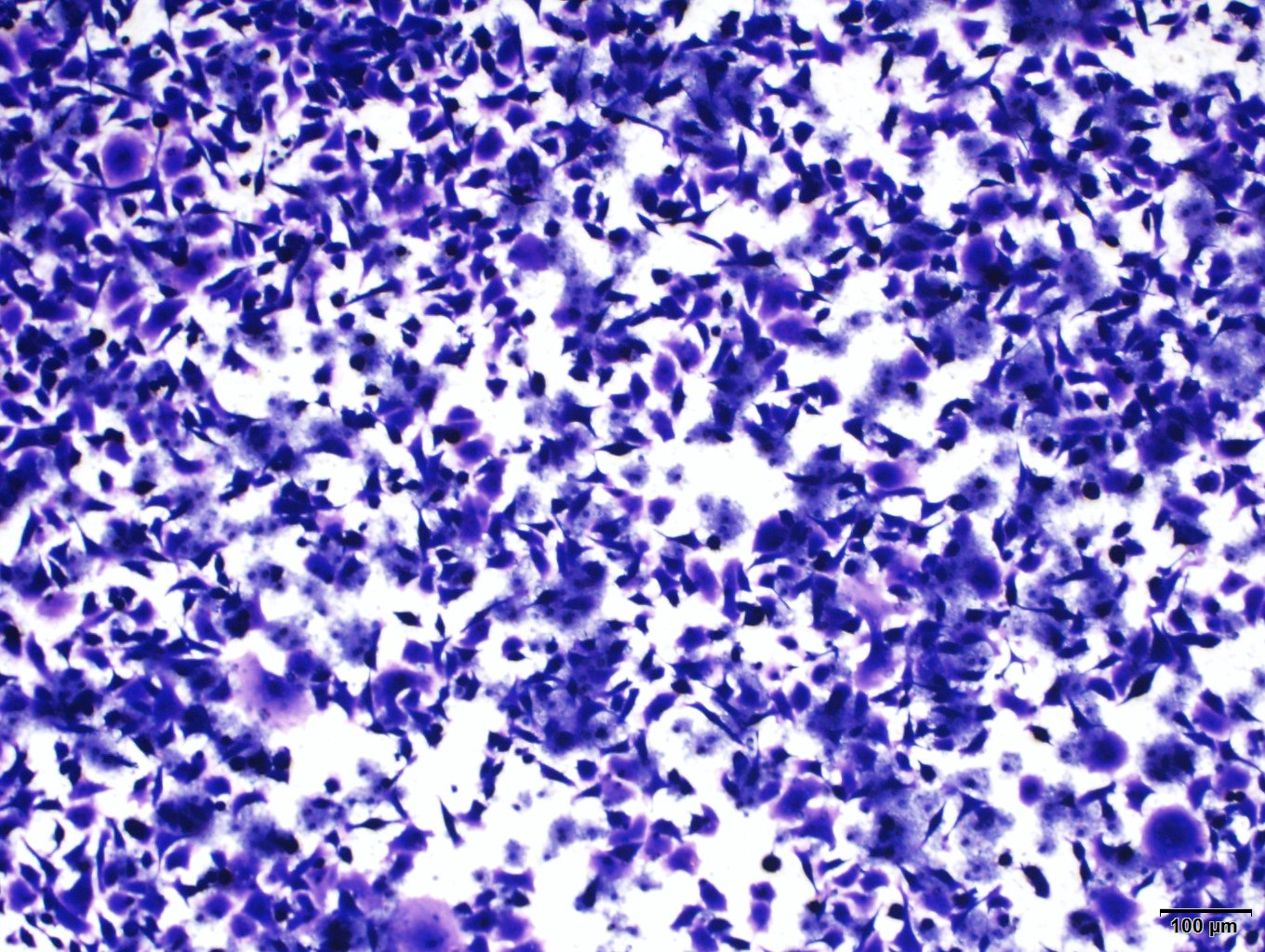

Supplement: Supplementary file 11 — EV Figure Source Data part 3 [file 44318_2025_363_MOESM11_ESM.zip › Figure EV6/EV6B/Ephrin A1 (4).jpg]

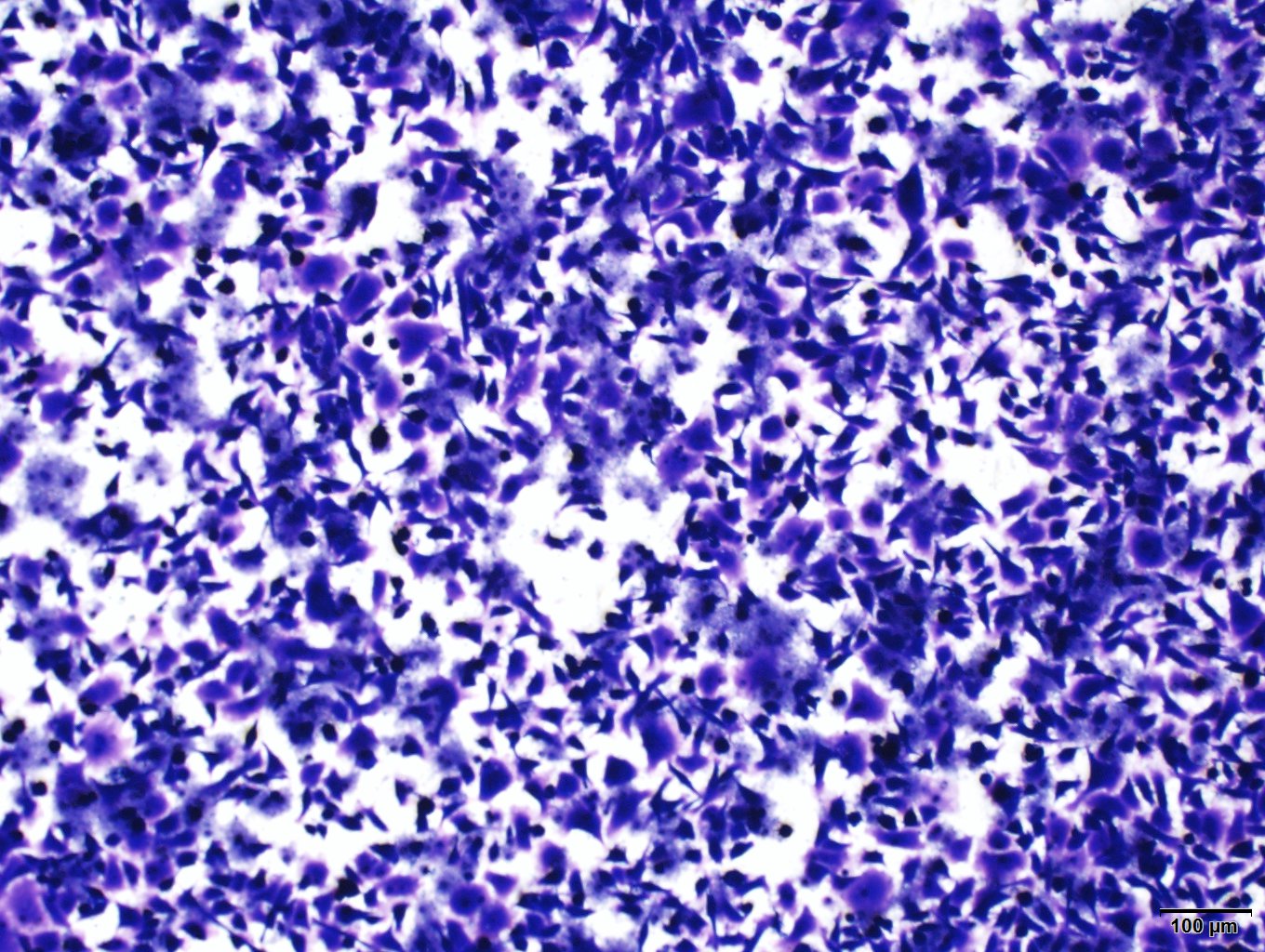

Supplement: Supplementary file 11 — EV Figure Source Data part 3 [file 44318_2025_363_MOESM11_ESM.zip › Figure EV6/EV6B/Ephrin A1 (5).jpg]

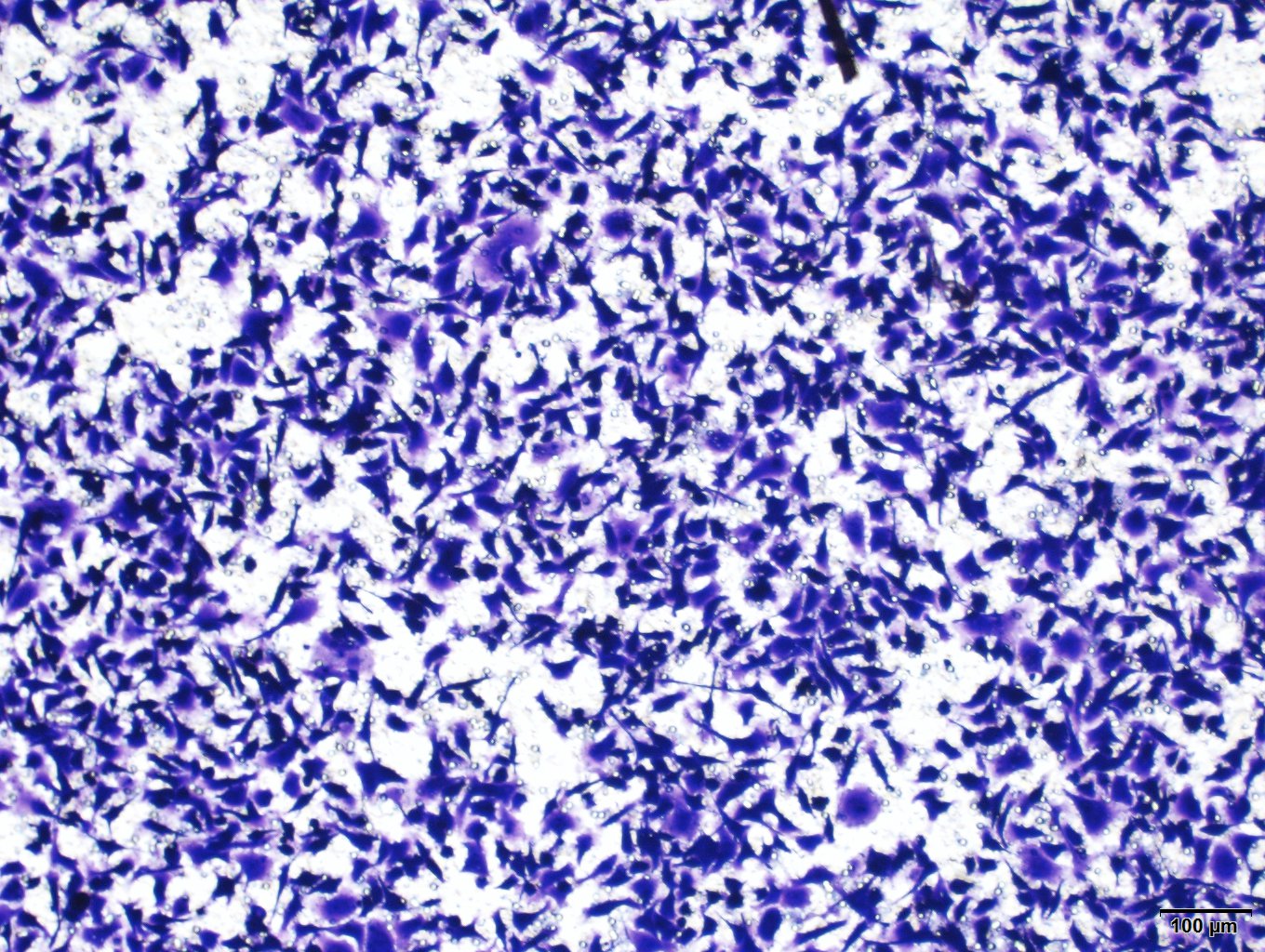

Supplement: Supplementary file 11 — EV Figure Source Data part 3 [file 44318_2025_363_MOESM11_ESM.zip › Figure EV6/EV6B/Ephrin A1+Defactinib (1).jpg]

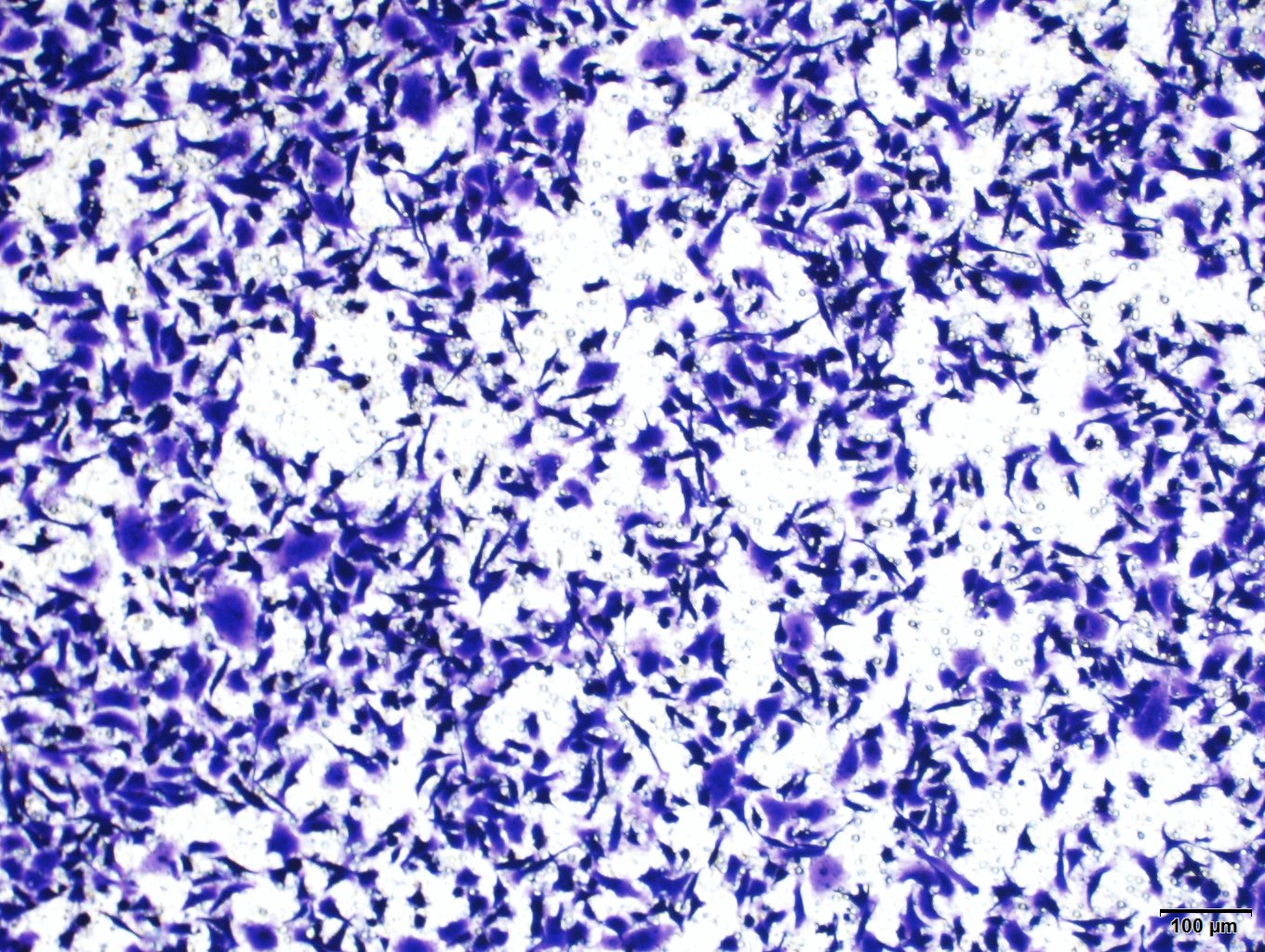

Supplement: Supplementary file 11 — EV Figure Source Data part 3 [file 44318_2025_363_MOESM11_ESM.zip › Figure EV6/EV6B/Ephrin A1+Defactinib (2).jpg]

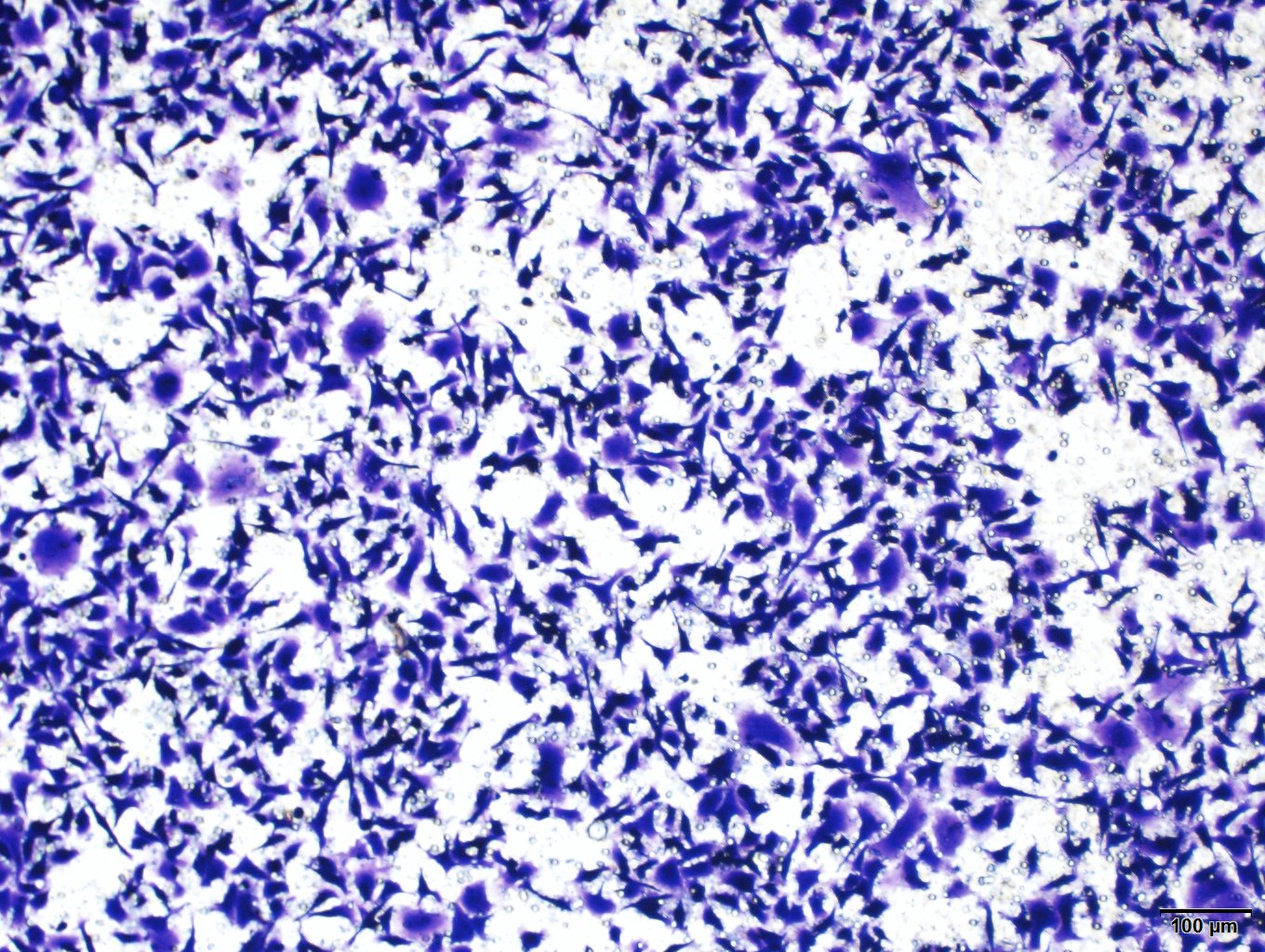

Supplement: Supplementary file 11 — EV Figure Source Data part 3 [file 44318_2025_363_MOESM11_ESM.zip › Figure EV6/EV6B/Ephrin A1+Defactinib (3)-displayed in EV6B.jpg]

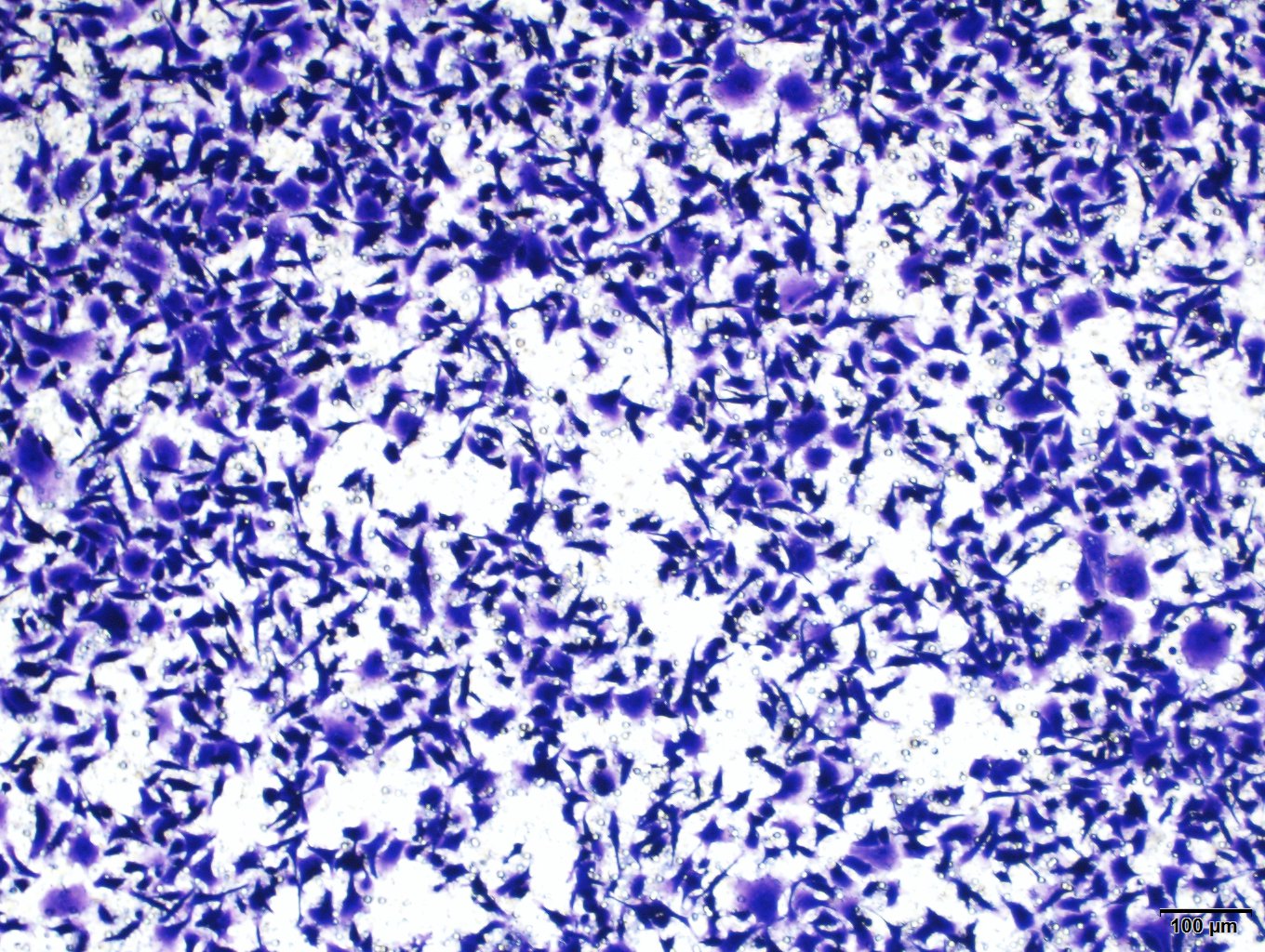

Supplement: Supplementary file 11 — EV Figure Source Data part 3 [file 44318_2025_363_MOESM11_ESM.zip › Figure EV6/EV6B/Ephrin A1+Defactinib (4).jpg]

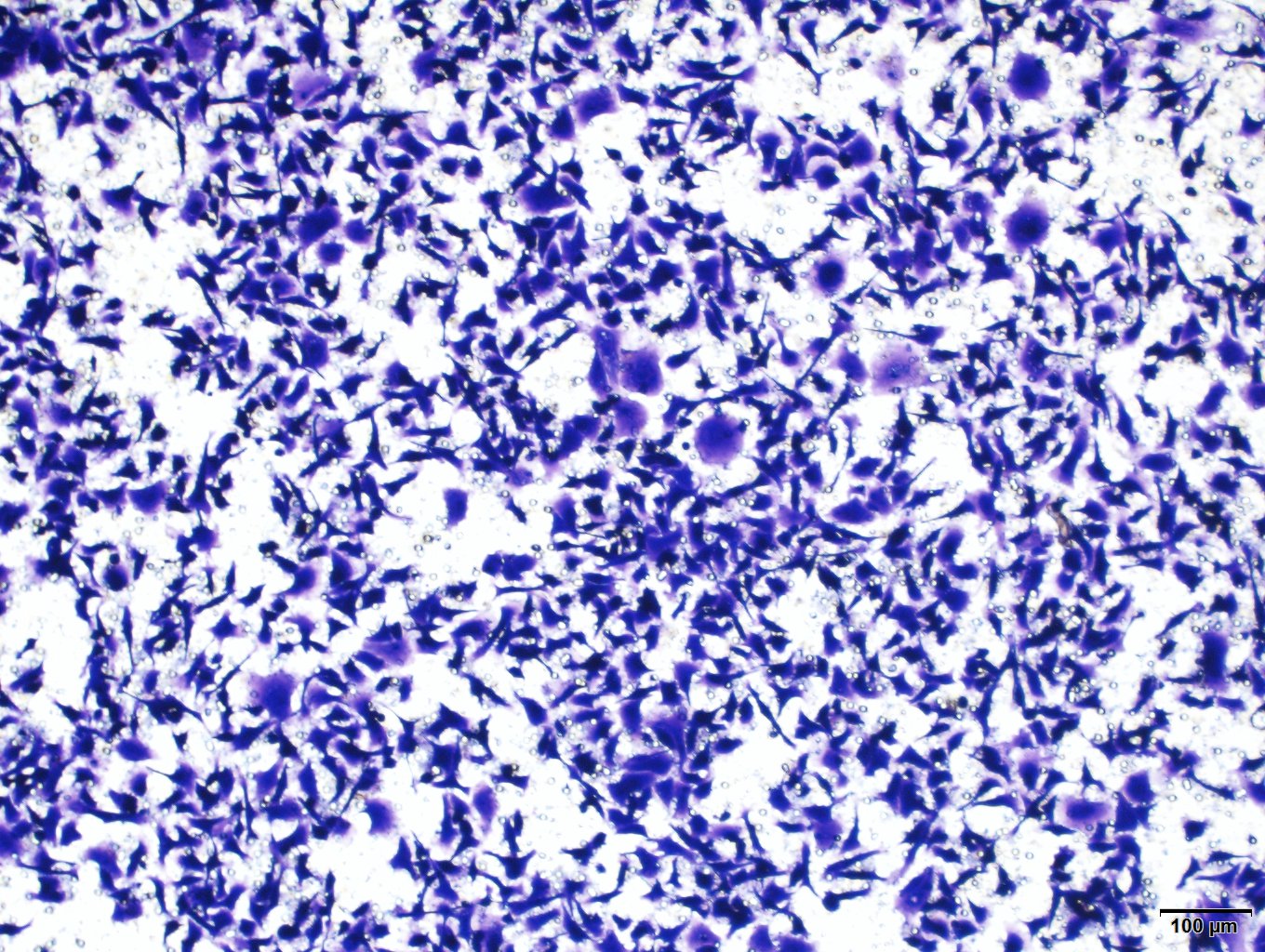

Supplement: Supplementary file 11 — EV Figure Source Data part 3 [file 44318_2025_363_MOESM11_ESM.zip › Figure EV6/EV6B/Ephrin A1+Defactinib (5).jpg]

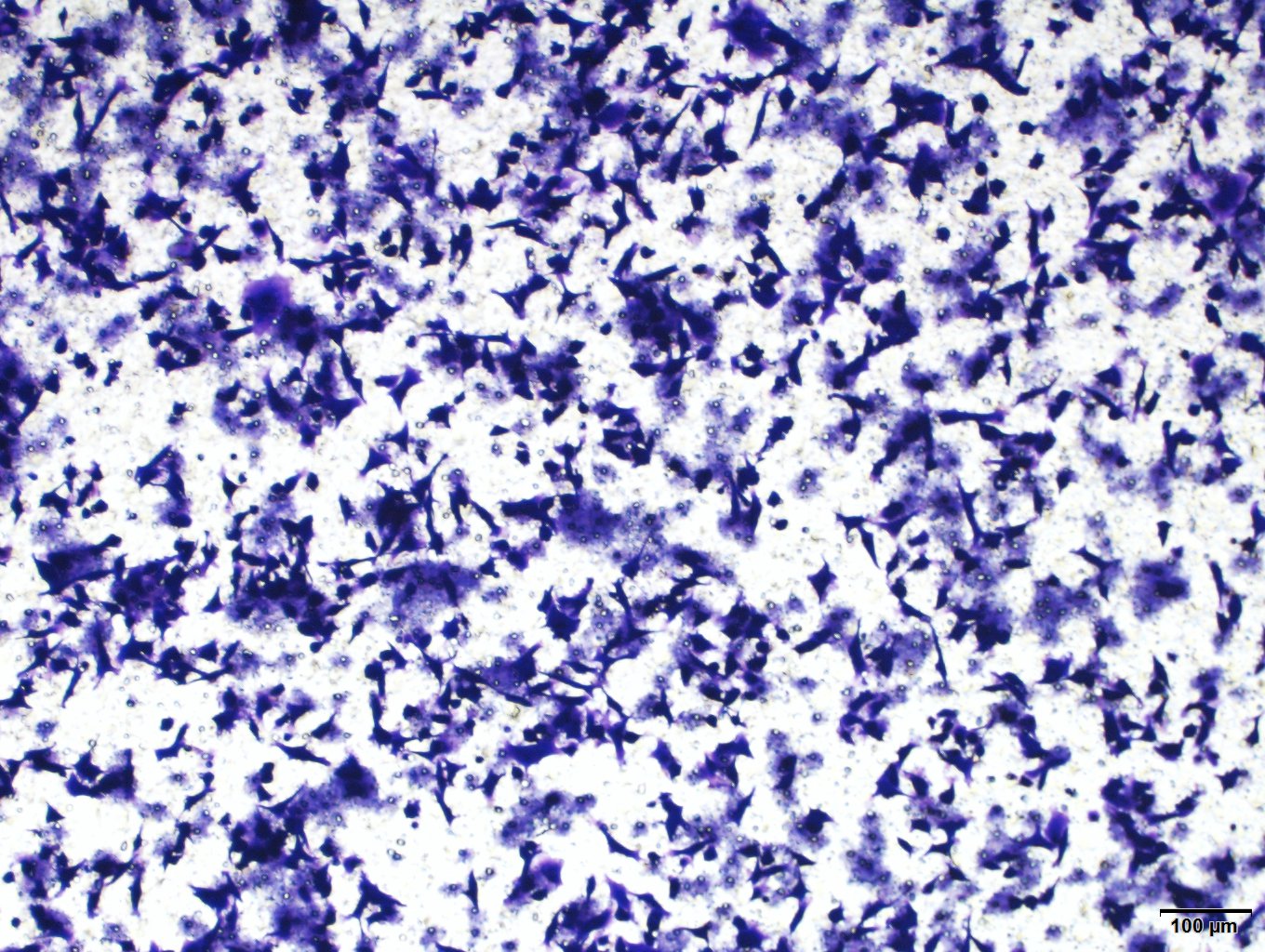

Supplement: Supplementary file 11 — EV Figure Source Data part 3 [file 44318_2025_363_MOESM11_ESM.zip › Figure EV6/EV6B/Ephrin A1+U0126 (1).jpg]

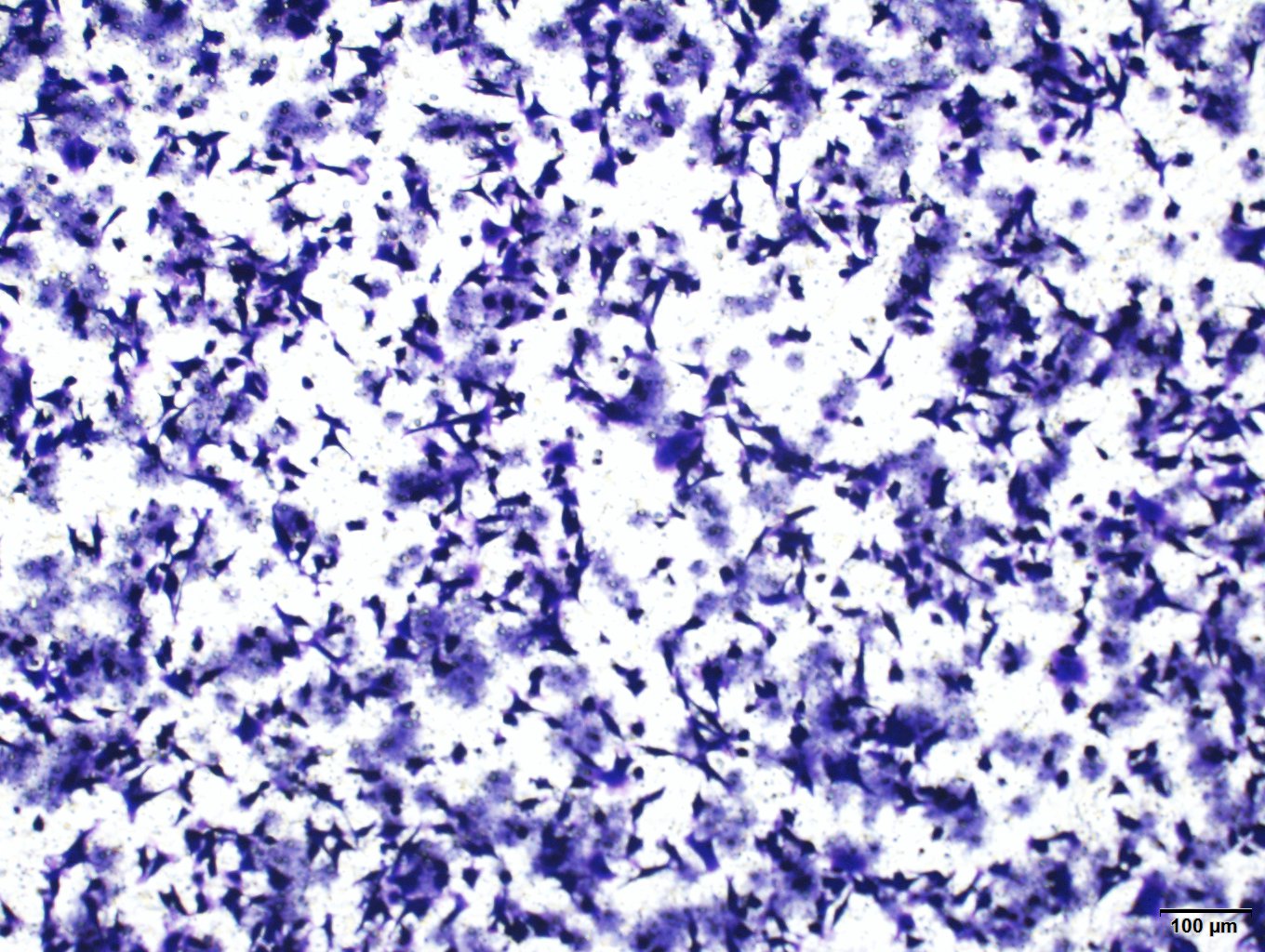

Supplement: Supplementary file 11 — EV Figure Source Data part 3 [file 44318_2025_363_MOESM11_ESM.zip › Figure EV6/EV6B/Ephrin A1+U0126 (2).jpg]

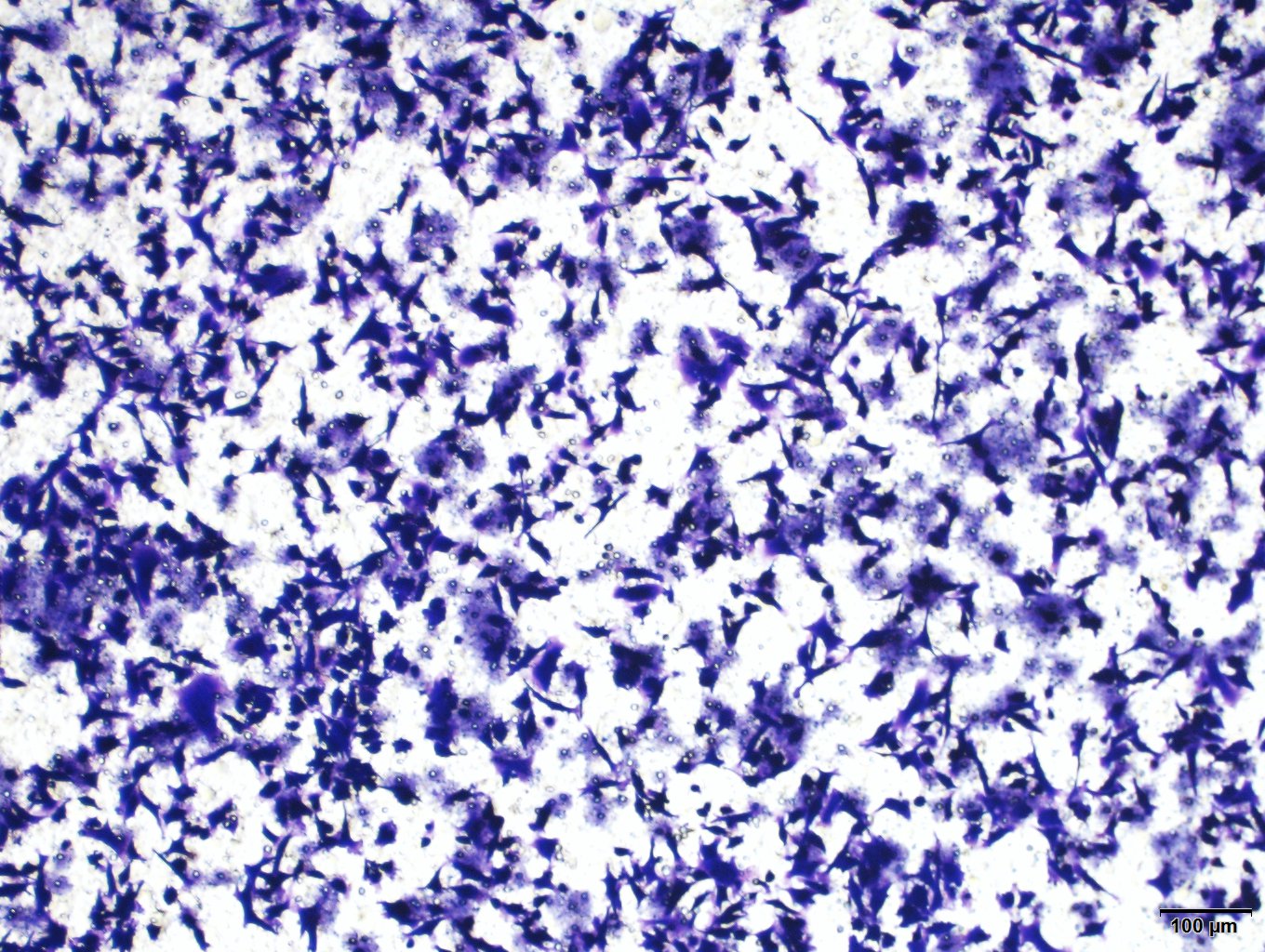

Supplement: Supplementary file 11 — EV Figure Source Data part 3 [file 44318_2025_363_MOESM11_ESM.zip › Figure EV6/EV6B/Ephrin A1+U0126 (3)-displayed in EV6B.jpg]

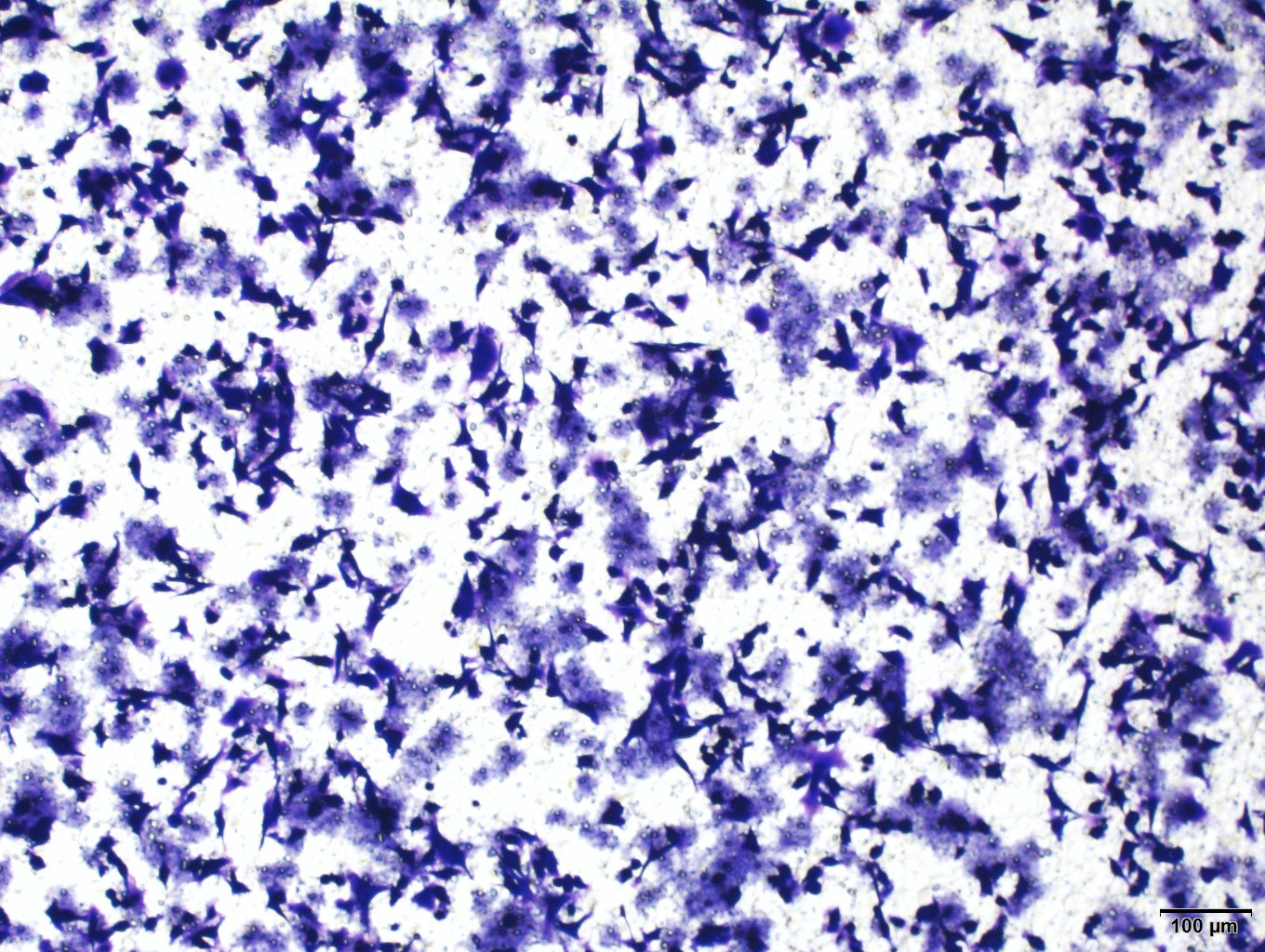

Supplement: Supplementary file 11 — EV Figure Source Data part 3 [file 44318_2025_363_MOESM11_ESM.zip › Figure EV6/EV6B/Ephrin A1+U0126 (4).jpg]

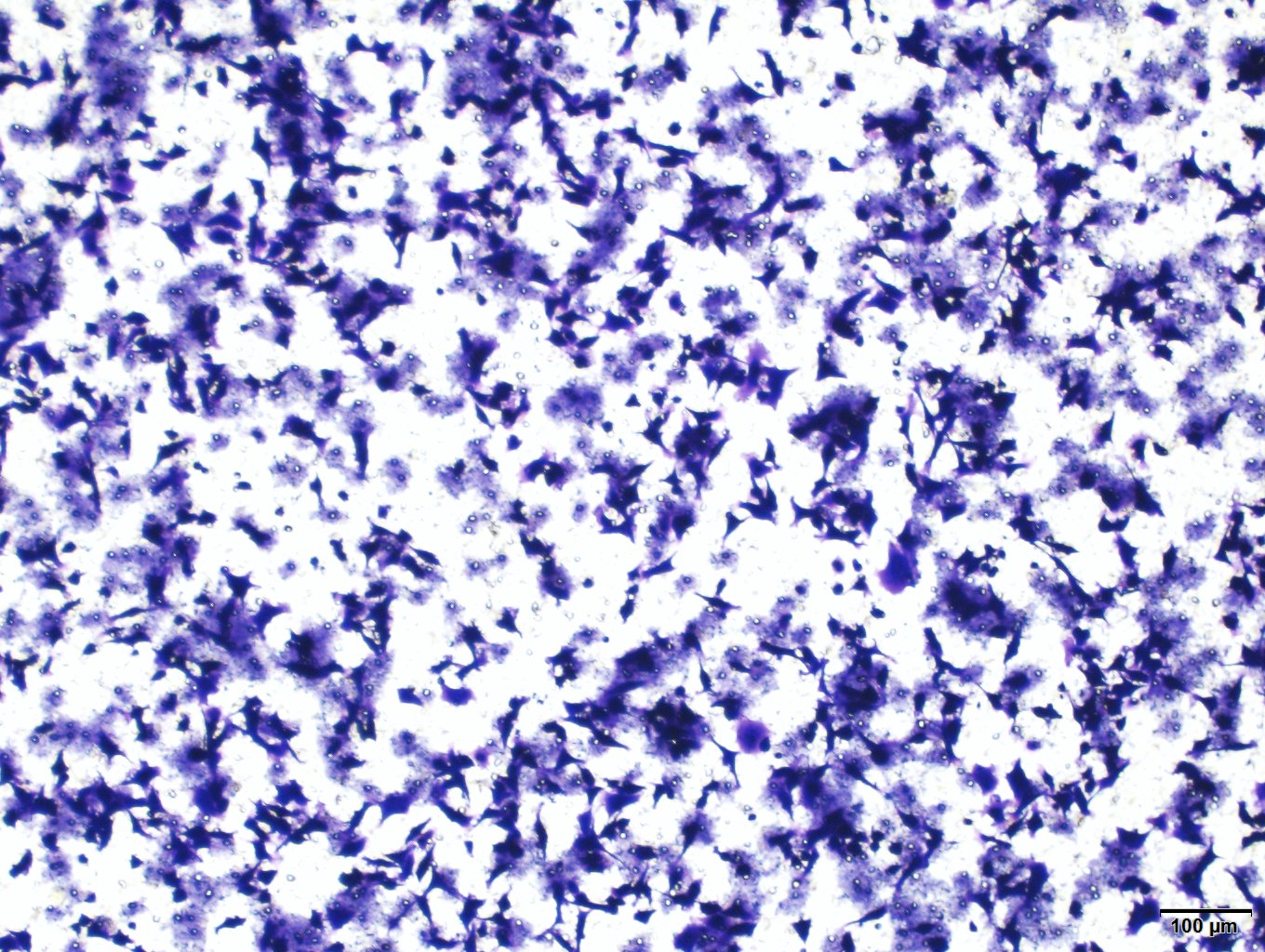

Supplement: Supplementary file 11 — EV Figure Source Data part 3 [file 44318_2025_363_MOESM11_ESM.zip › Figure EV6/EV6B/Ephrin A1+U0126 (5).jpg]

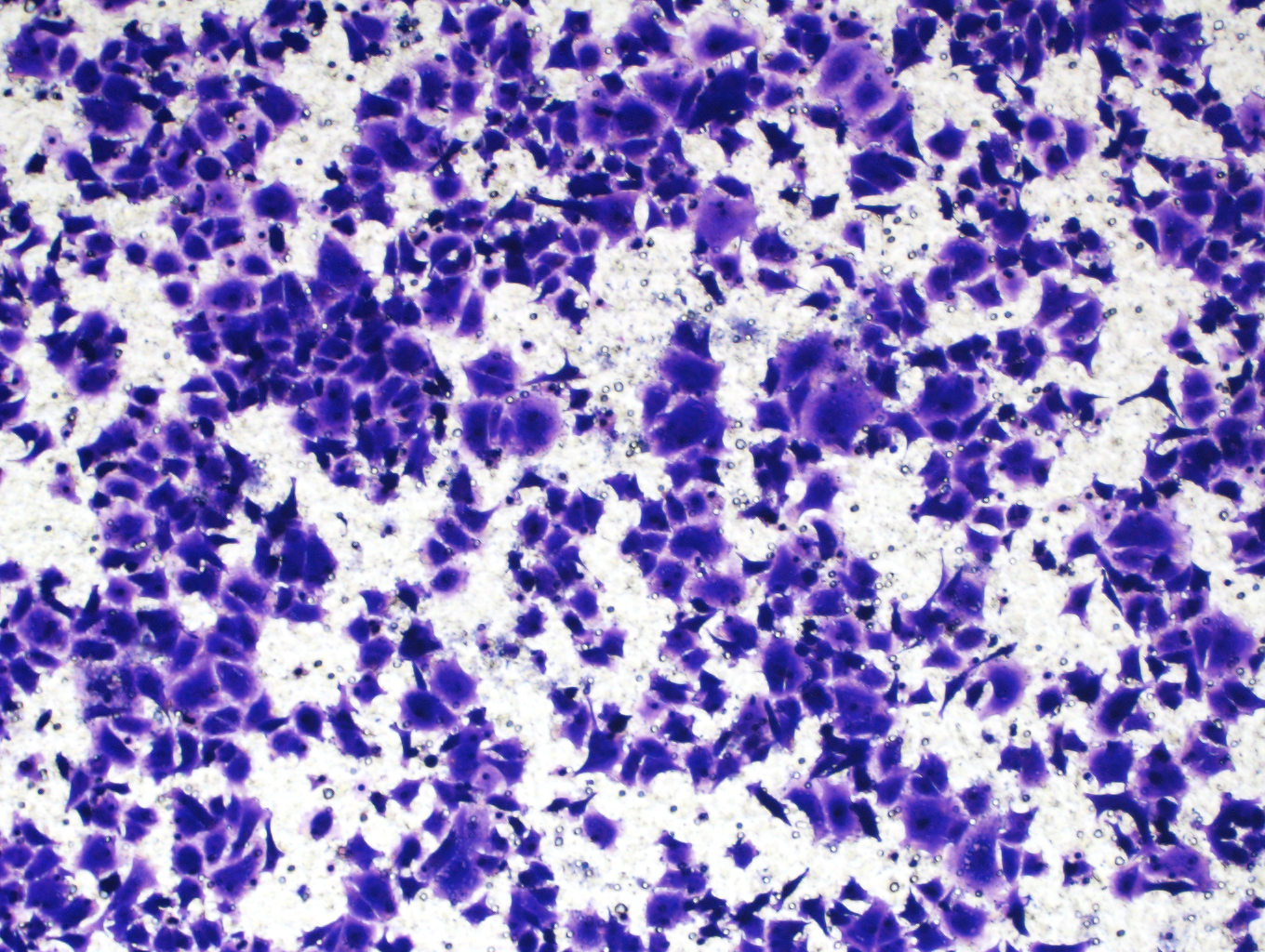

Supplement: Supplementary file 11 — EV Figure Source Data part 3 [file 44318_2025_363_MOESM11_ESM.zip › Figure EV6/EV6D/Control (1).tif]

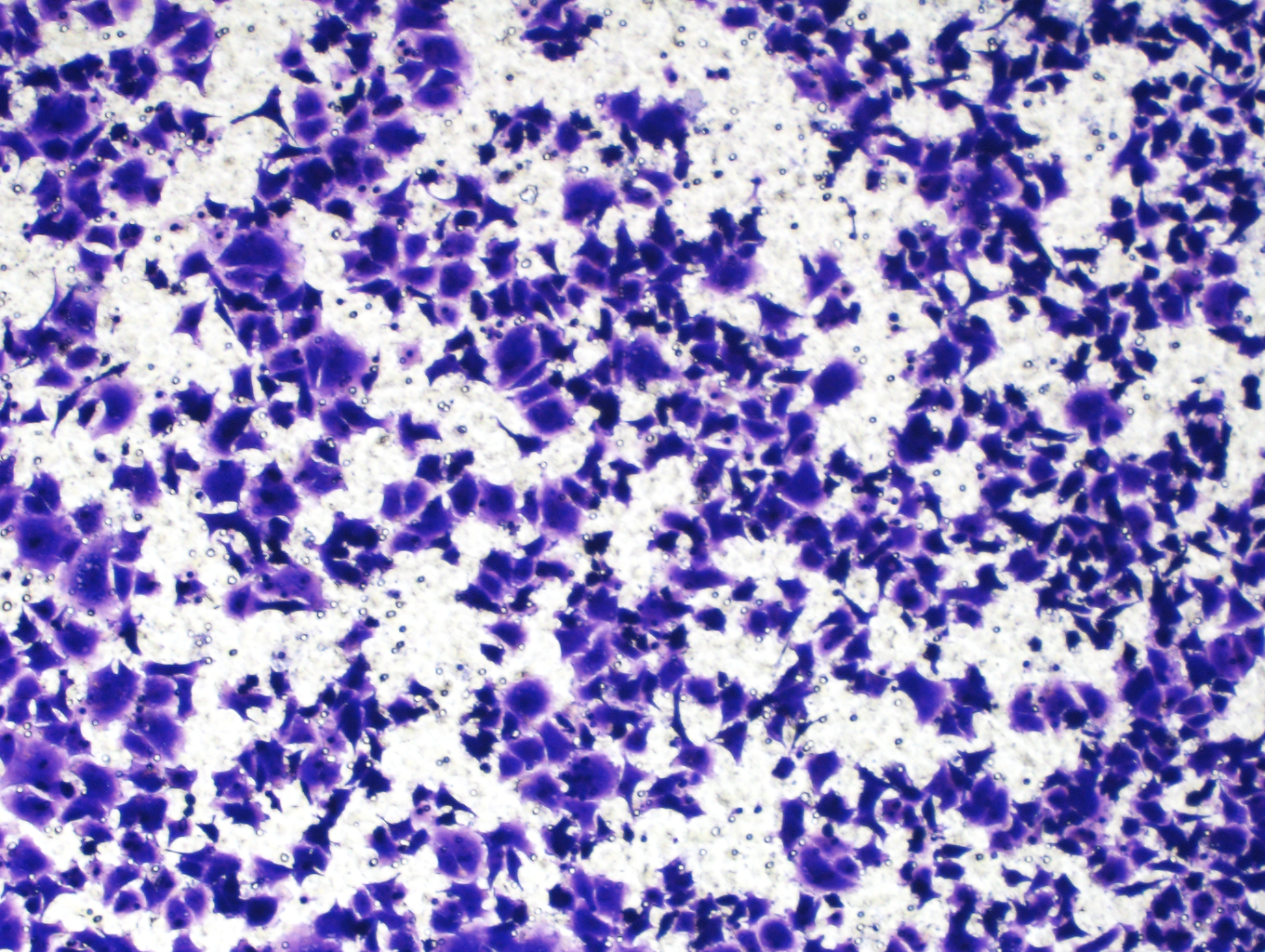

Supplement: Supplementary file 11 — EV Figure Source Data part 3 [file 44318_2025_363_MOESM11_ESM.zip › Figure EV6/EV6D/Control (2).tif]

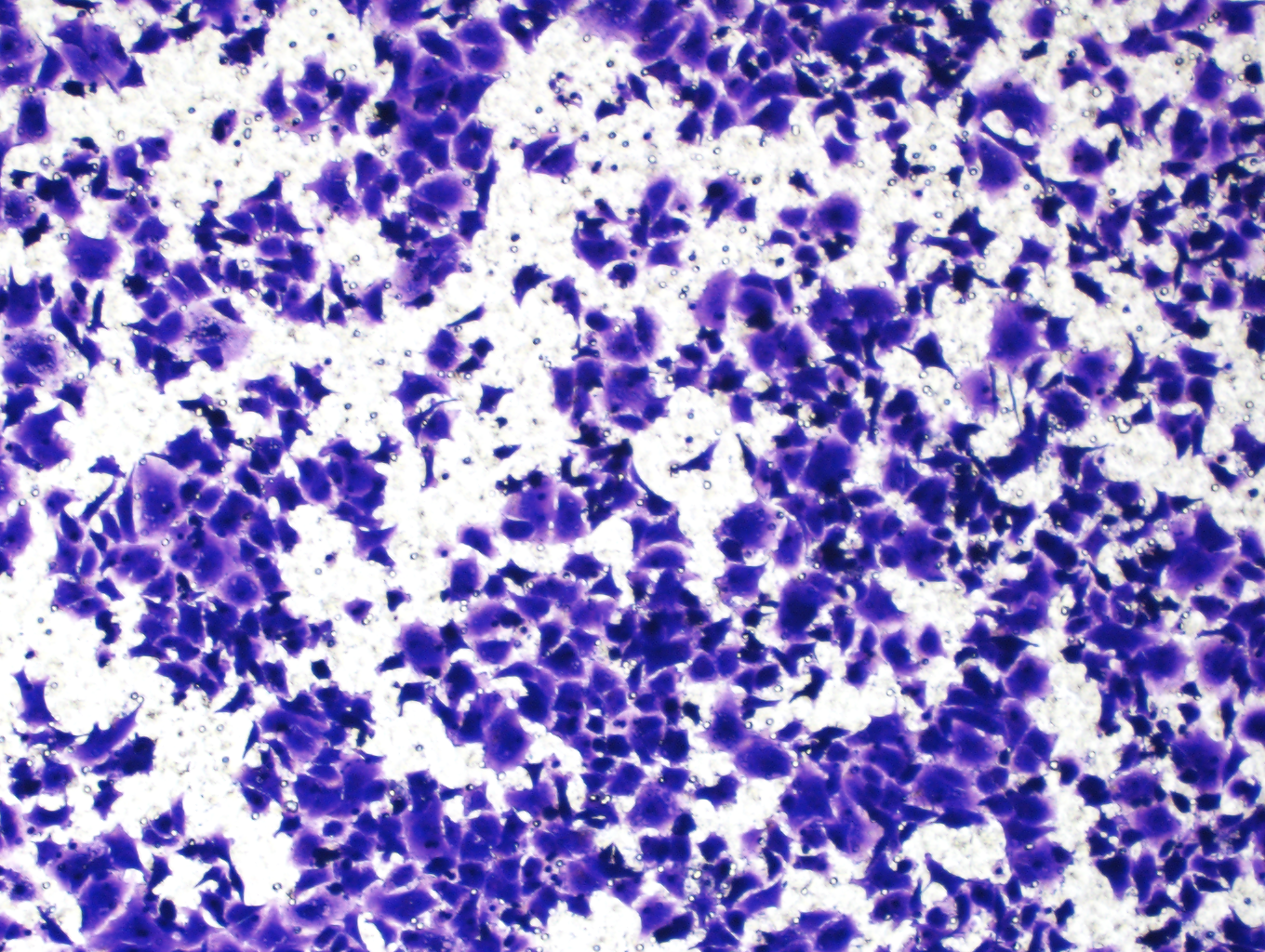

Supplement: Supplementary file 11 — EV Figure Source Data part 3 [file 44318_2025_363_MOESM11_ESM.zip › Figure EV6/EV6D/Control (3).tif]

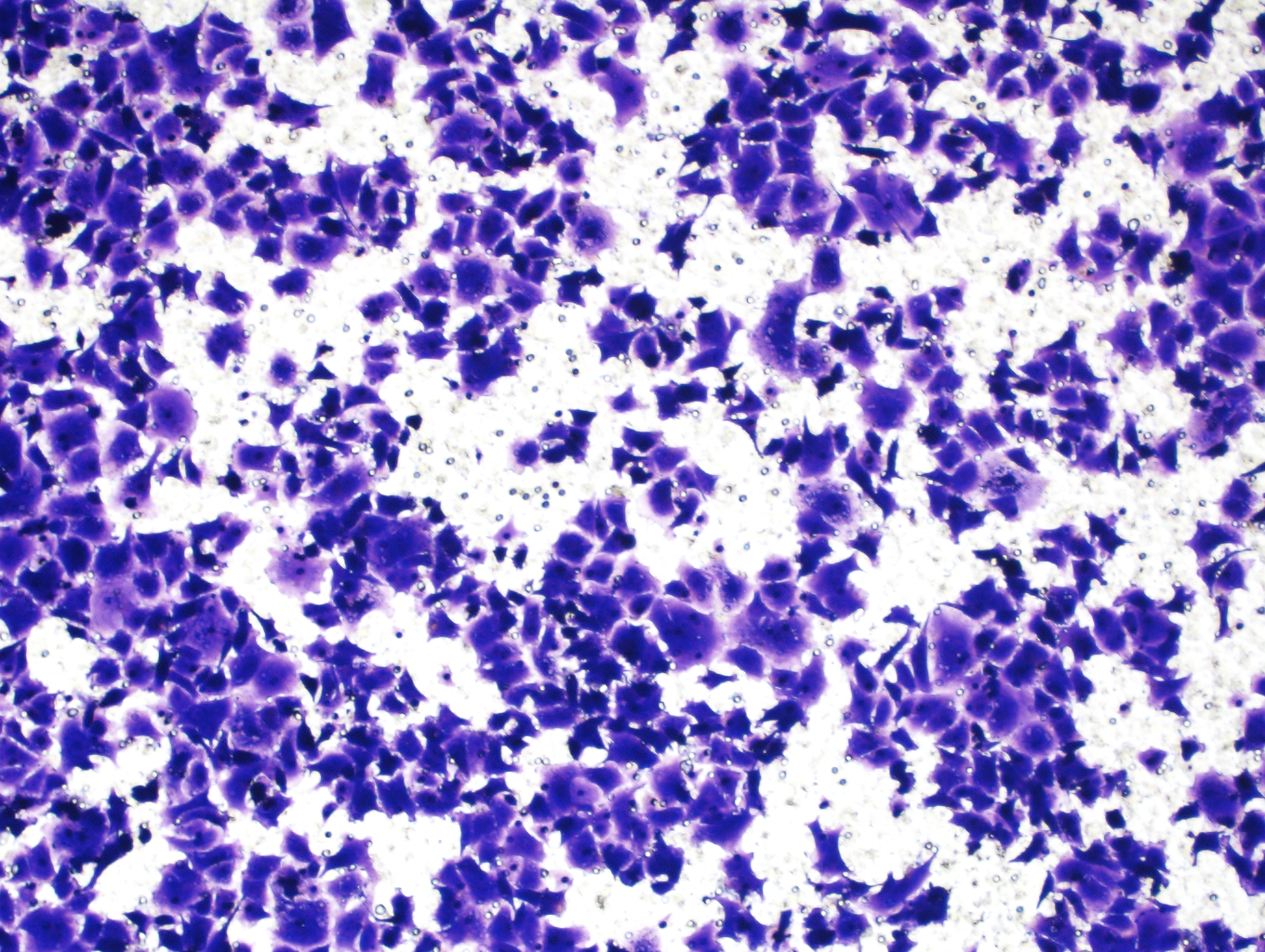

Supplement: Supplementary file 11 — EV Figure Source Data part 3 [file 44318_2025_363_MOESM11_ESM.zip › Figure EV6/EV6D/Control (4)-displayed in EV6D.tif]

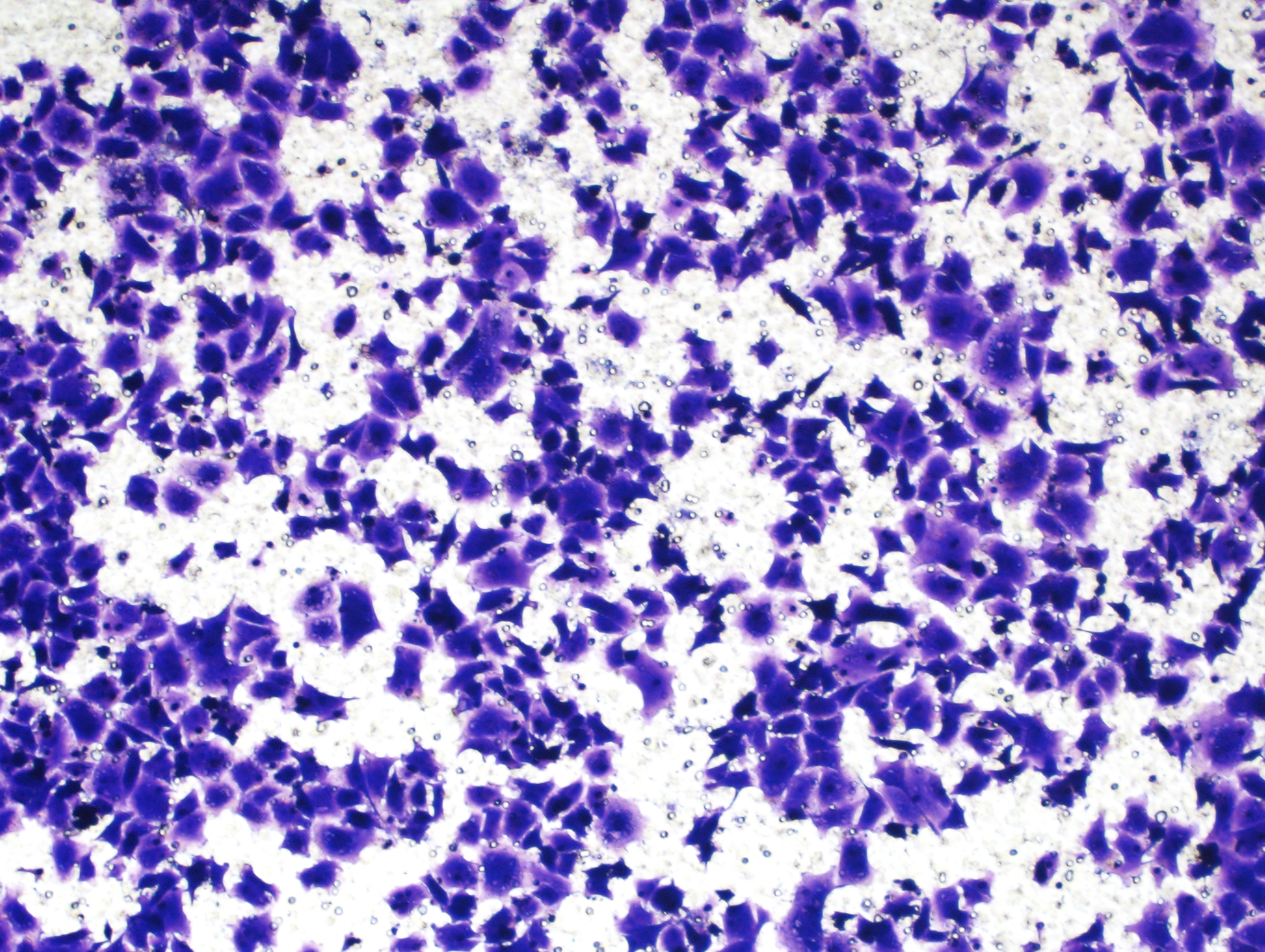

Supplement: Supplementary file 11 — EV Figure Source Data part 3 [file 44318_2025_363_MOESM11_ESM.zip › Figure EV6/EV6D/Control (5).tif]

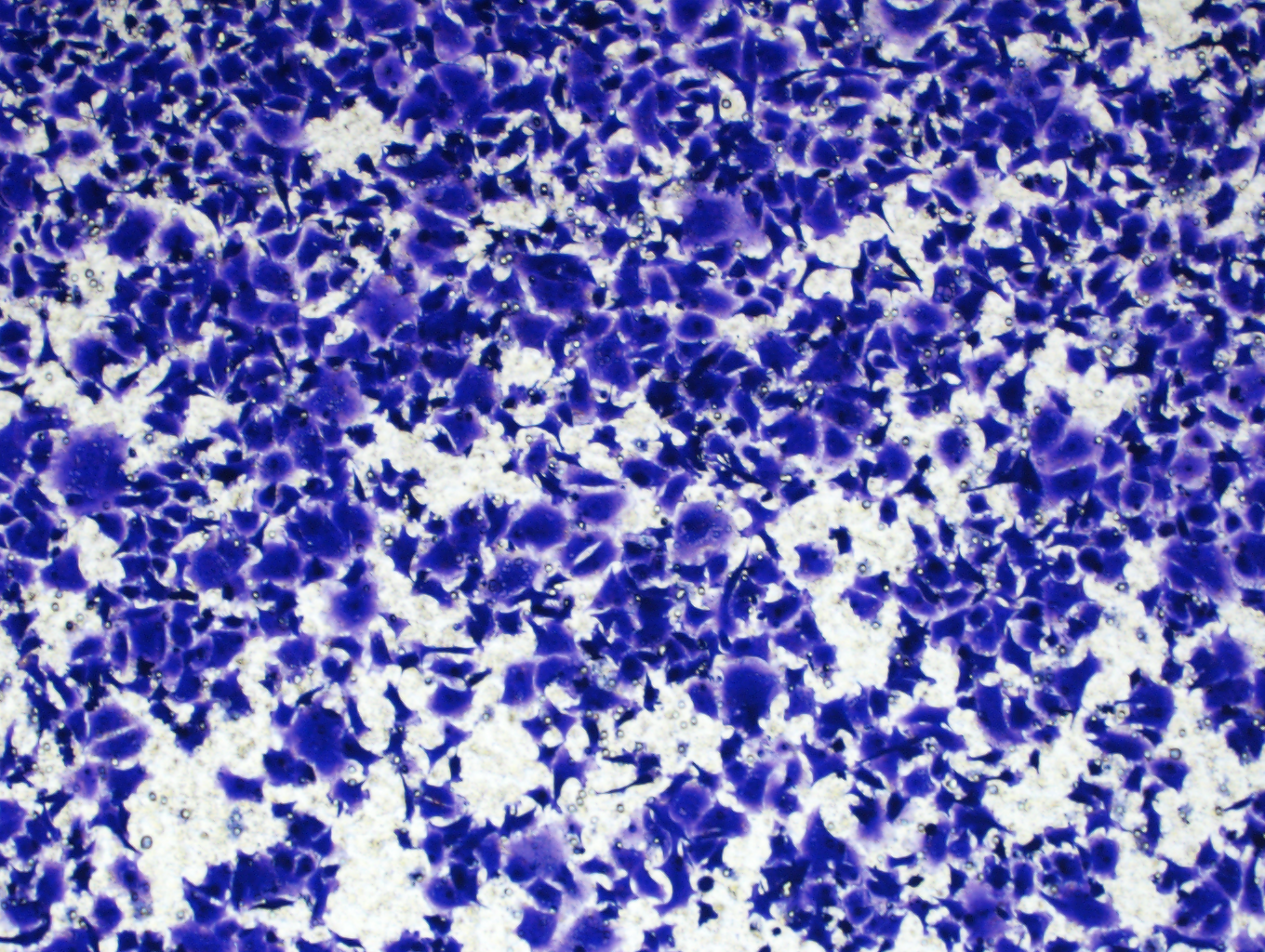

Supplement: Supplementary file 11 — EV Figure Source Data part 3 [file 44318_2025_363_MOESM11_ESM.zip › Figure EV6/EV6D/Ephrin A1 (1)-displayed in EV6D.tif]

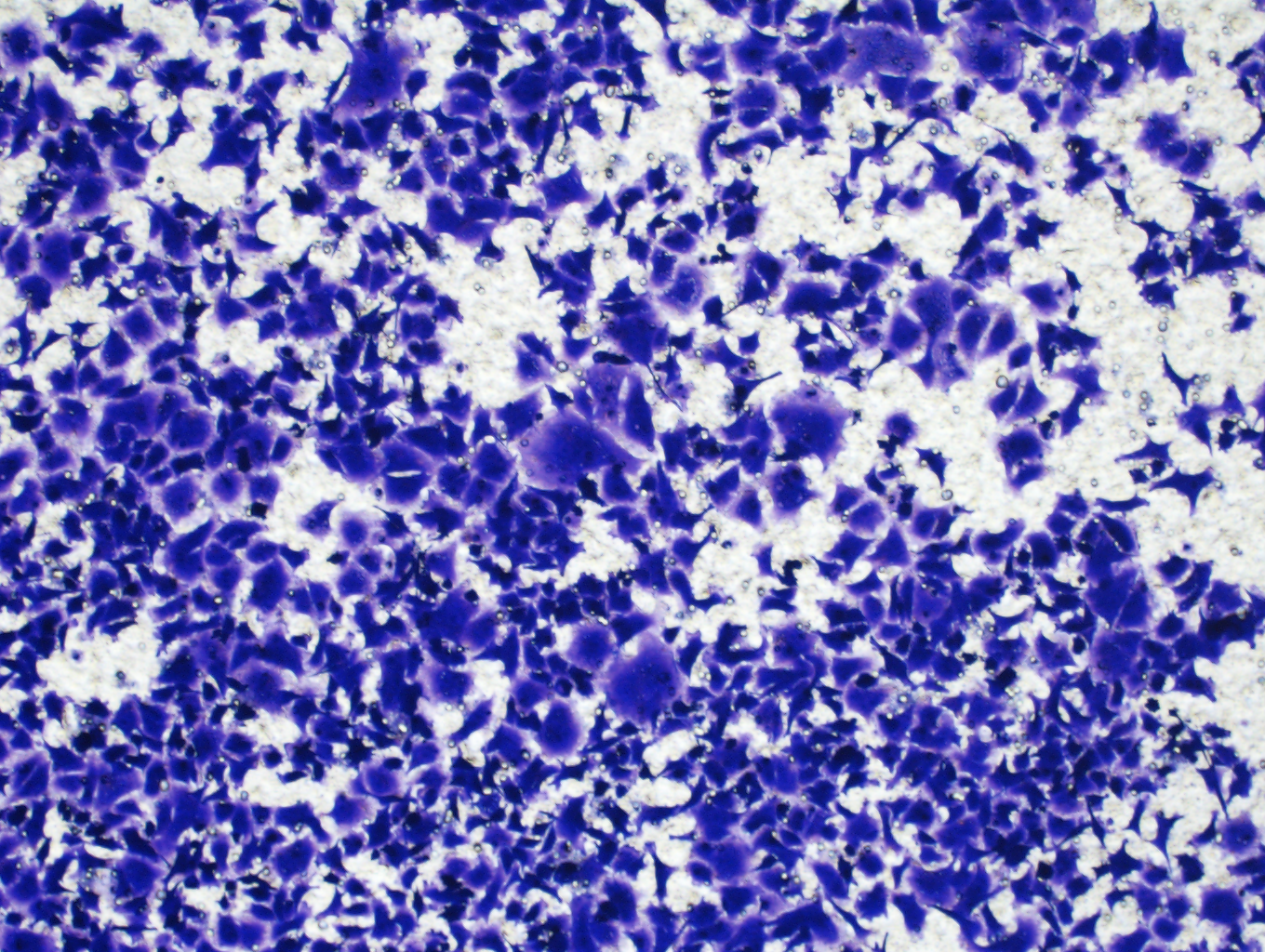

Supplement: Supplementary file 11 — EV Figure Source Data part 3 [file 44318_2025_363_MOESM11_ESM.zip › Figure EV6/EV6D/Ephrin A1 (2).tif]

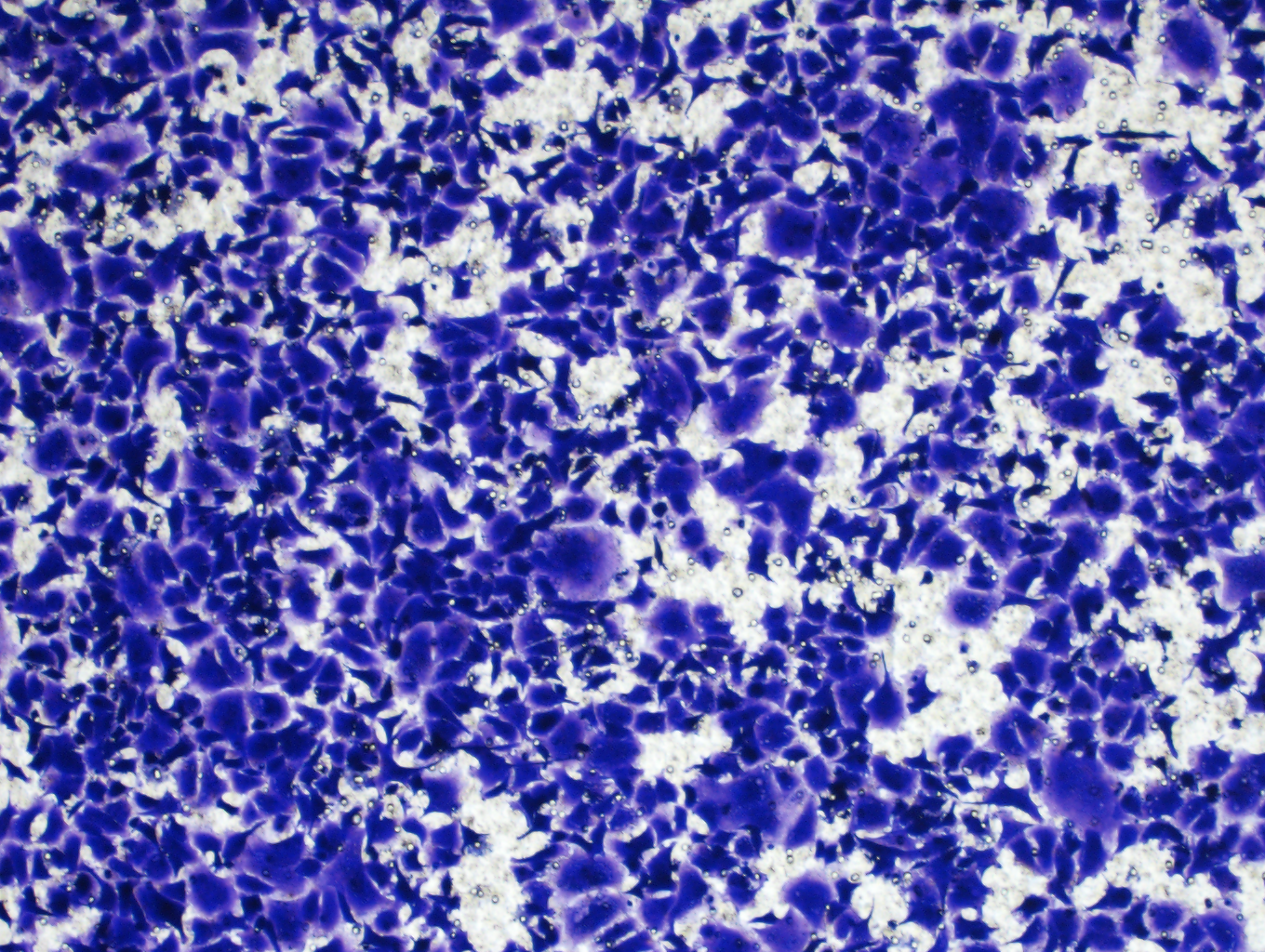

Supplement: Supplementary file 11 — EV Figure Source Data part 3 [file 44318_2025_363_MOESM11_ESM.zip › Figure EV6/EV6D/Ephrin A1 (3).tif]

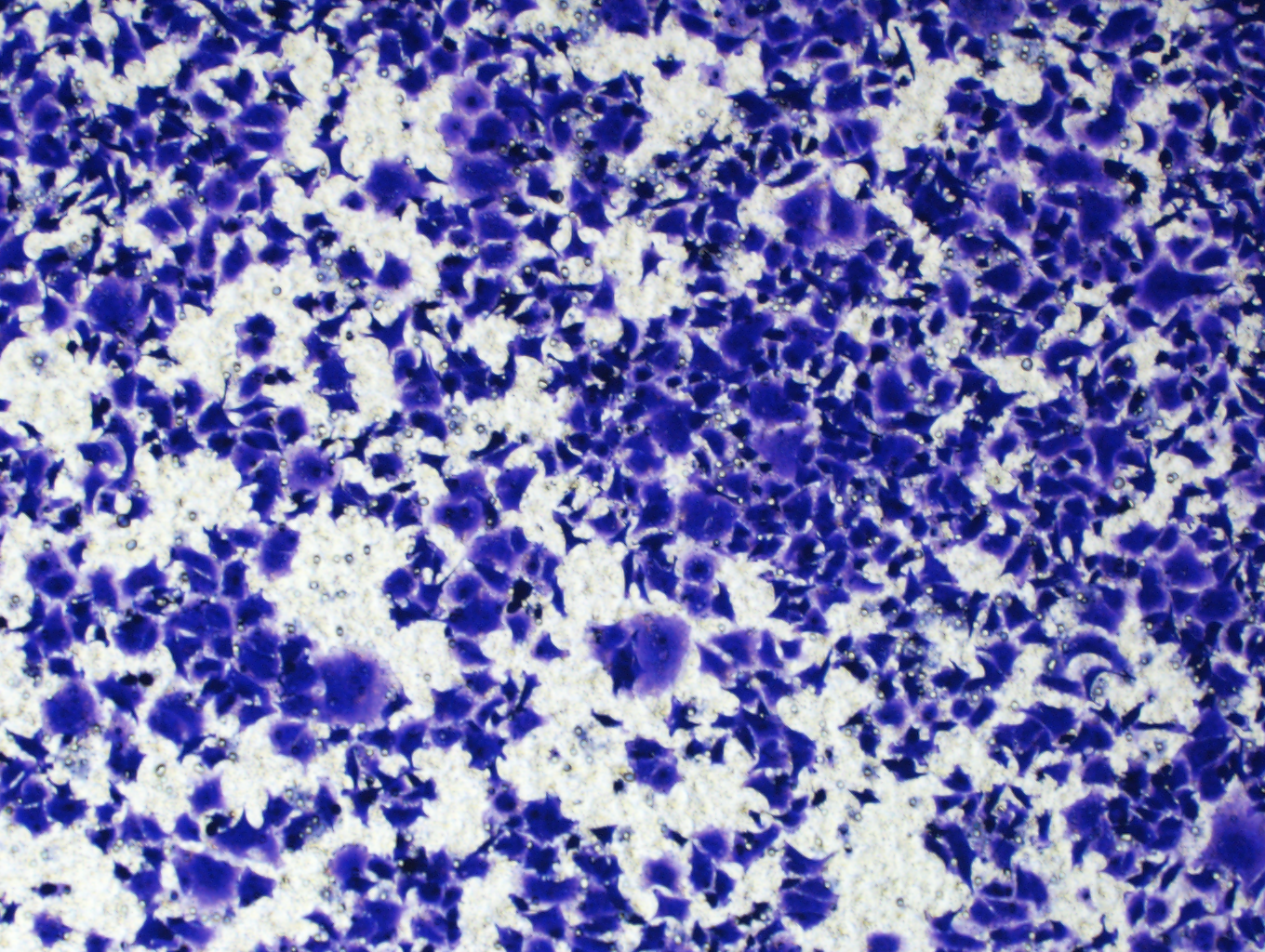

Supplement: Supplementary file 11 — EV Figure Source Data part 3 [file 44318_2025_363_MOESM11_ESM.zip › Figure EV6/EV6D/Ephrin A1 (4).tif]

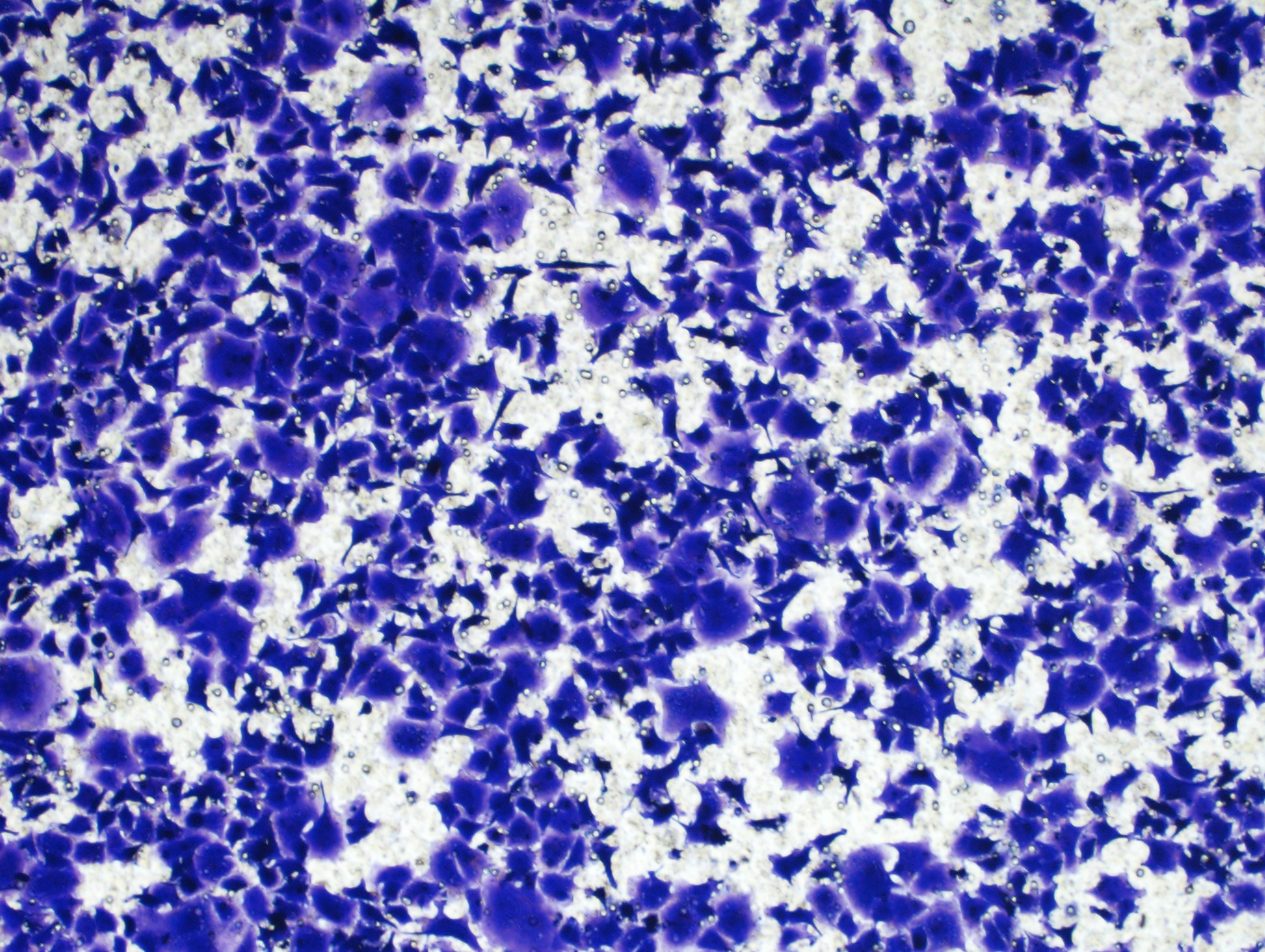

Supplement: Supplementary file 11 — EV Figure Source Data part 3 [file 44318_2025_363_MOESM11_ESM.zip › Figure EV6/EV6D/Ephrin A1 (5).tif]

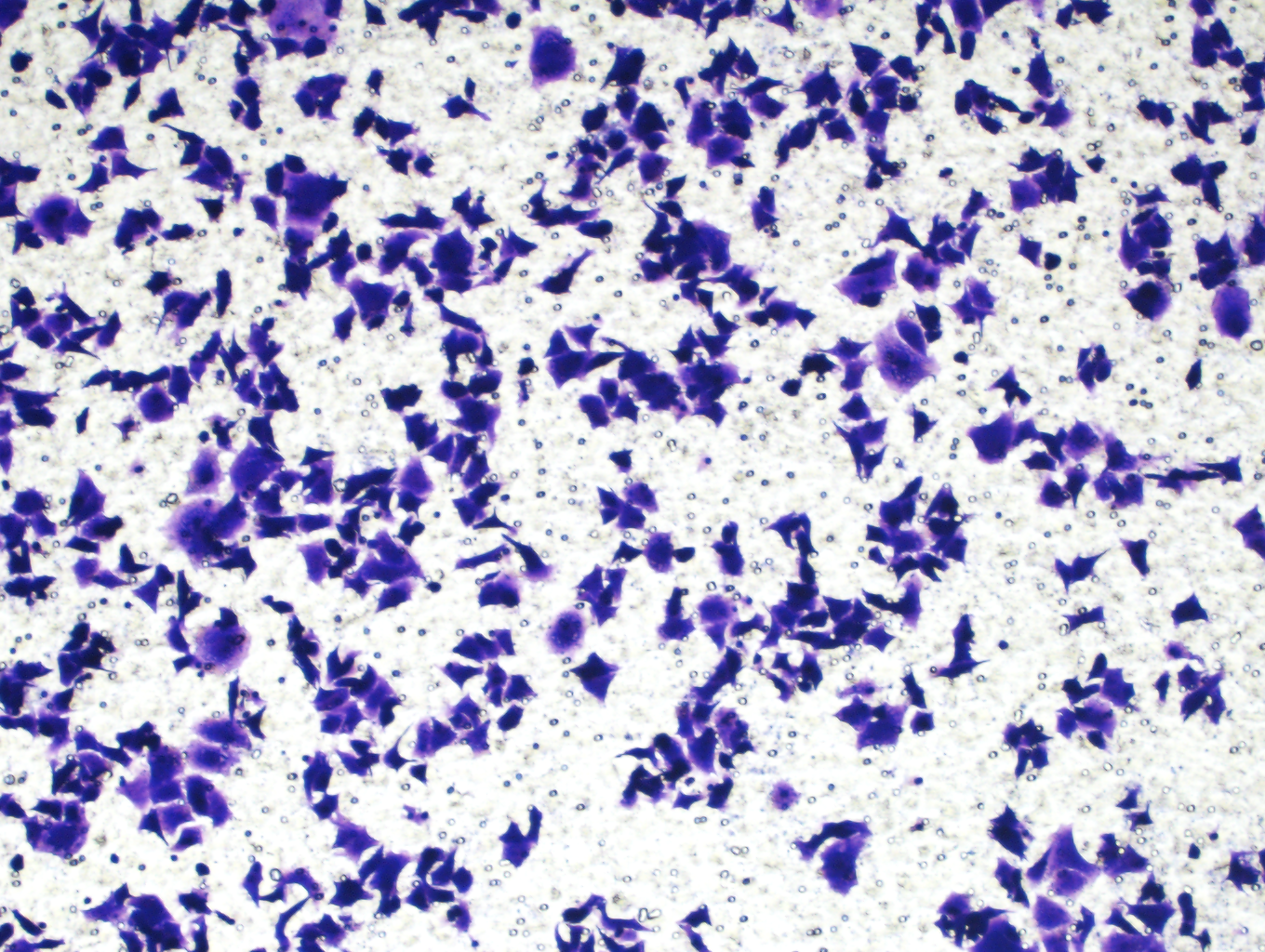

Supplement: Supplementary file 11 — EV Figure Source Data part 3 [file 44318_2025_363_MOESM11_ESM.zip › Figure EV6/EV6D/Ephrin A1+Defactinib (1).tif]

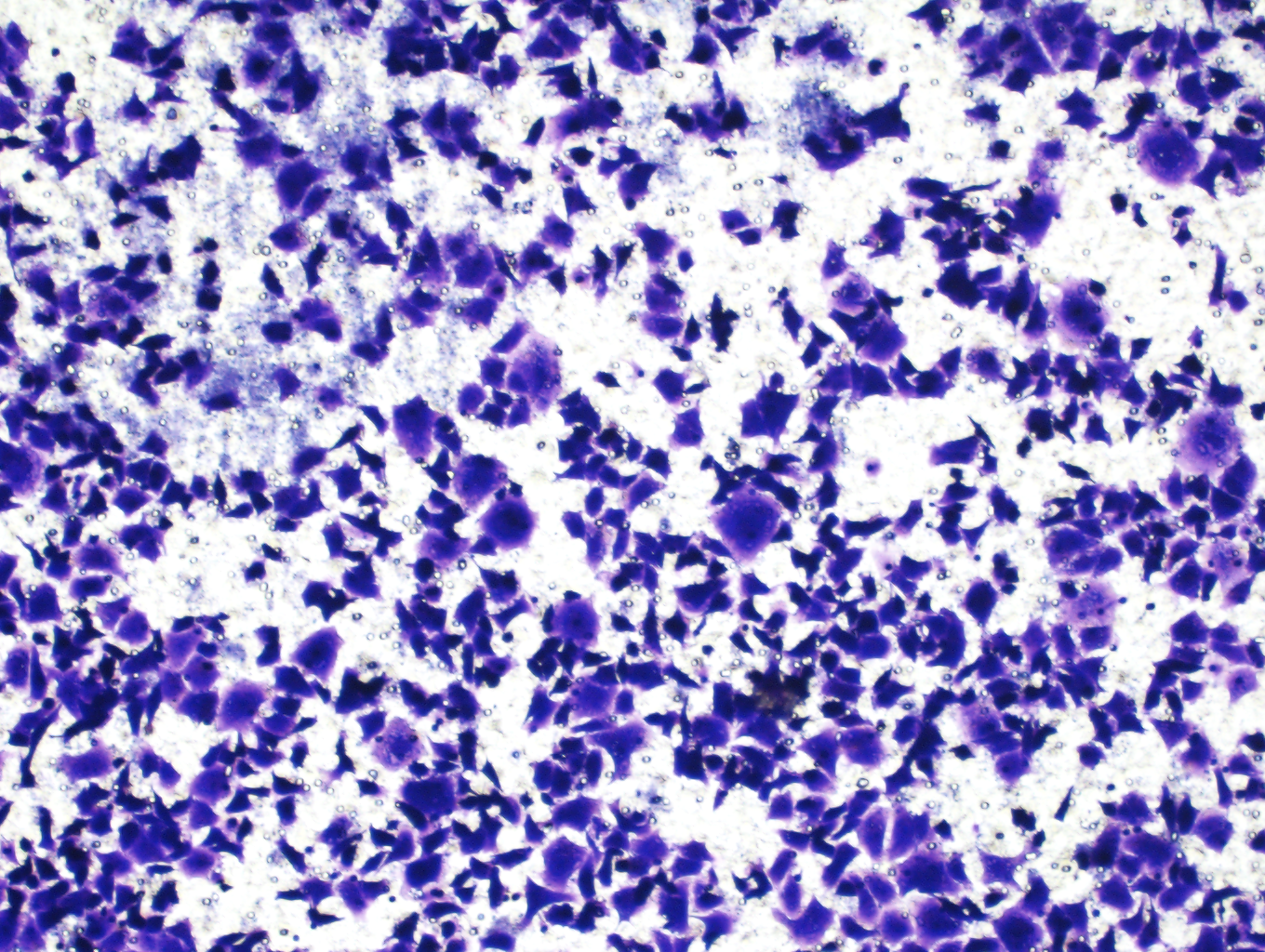

Supplement: Supplementary file 11 — EV Figure Source Data part 3 [file 44318_2025_363_MOESM11_ESM.zip › Figure EV6/EV6D/Ephrin A1+Defactinib (2).tif]

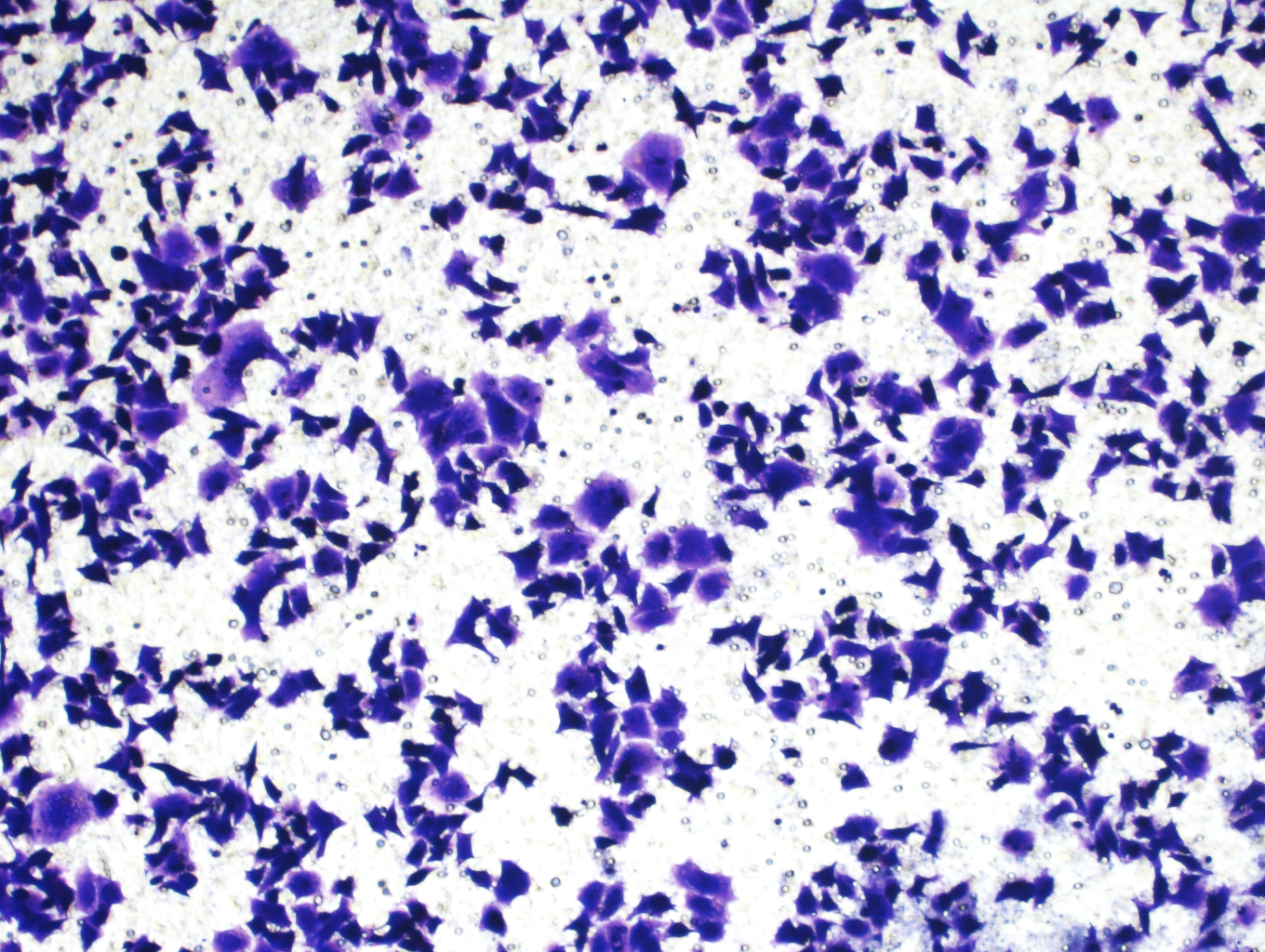

Supplement: Supplementary file 11 — EV Figure Source Data part 3 [file 44318_2025_363_MOESM11_ESM.zip › Figure EV6/EV6D/Ephrin A1+Defactinib (3).tif]

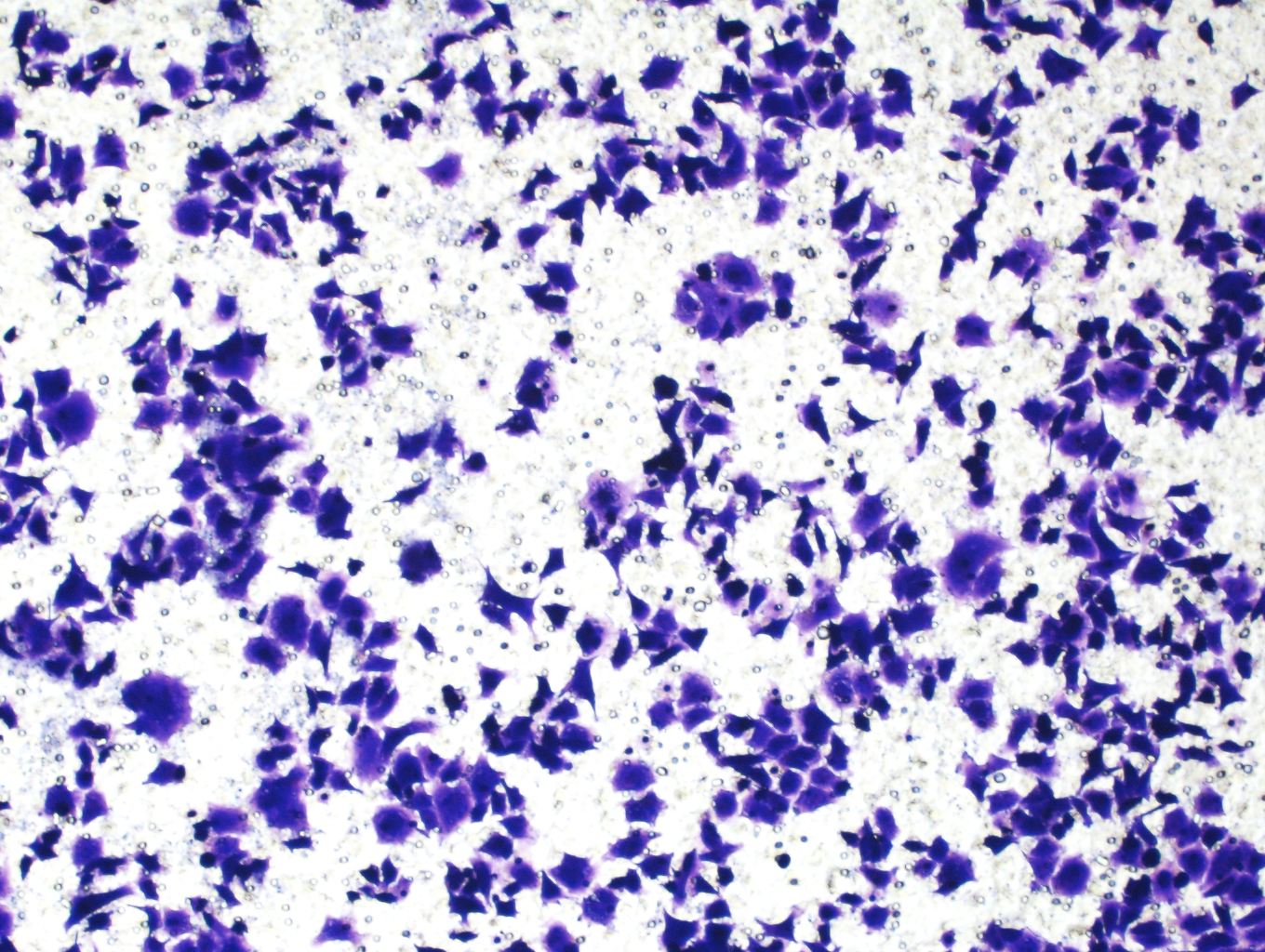

Supplement: Supplementary file 11 — EV Figure Source Data part 3 [file 44318_2025_363_MOESM11_ESM.zip › Figure EV6/EV6D/Ephrin A1+Defactinib (4)-displayed in EV6D.tif]

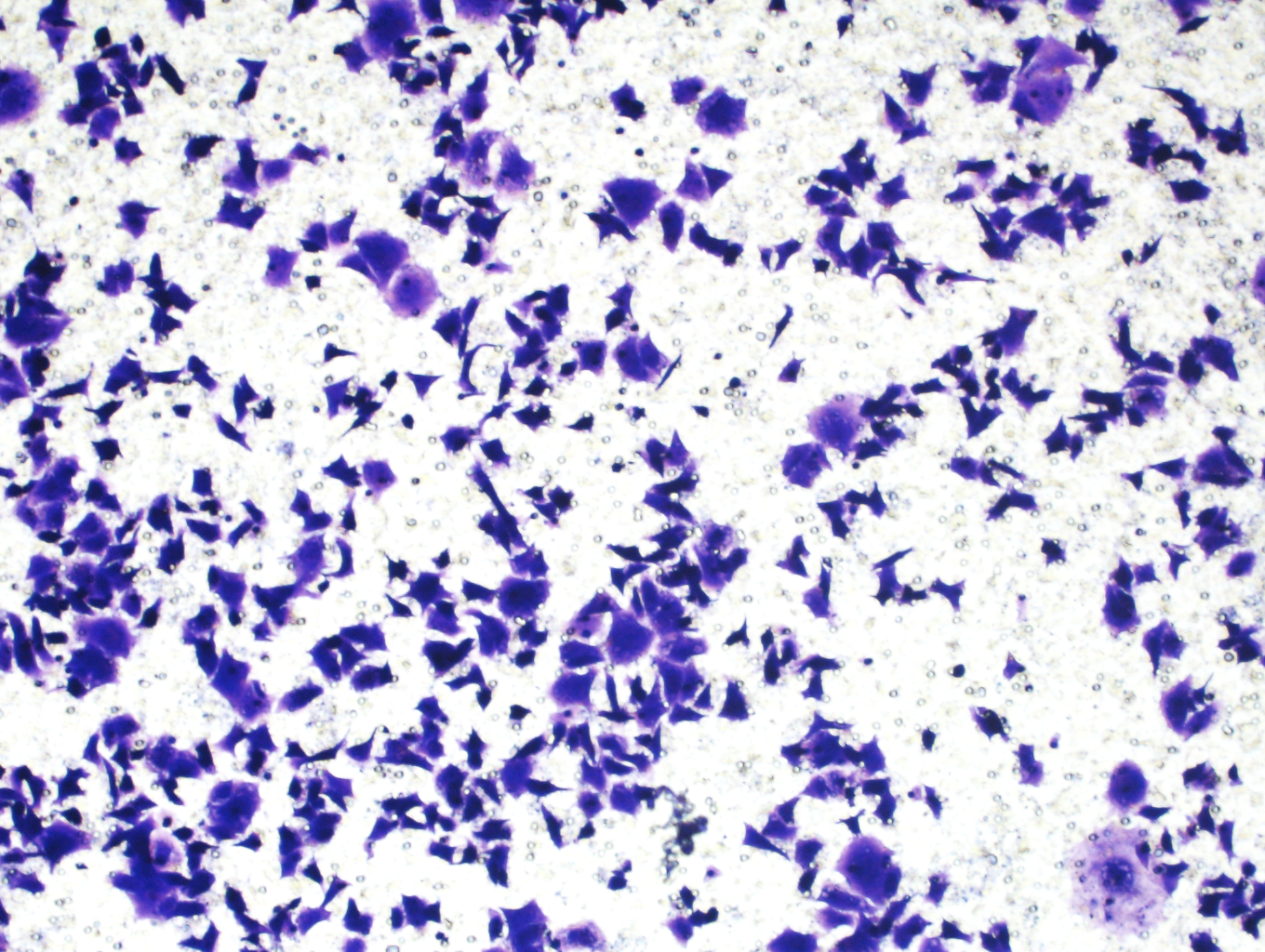

Supplement: Supplementary file 11 — EV Figure Source Data part 3 [file 44318_2025_363_MOESM11_ESM.zip › Figure EV6/EV6D/Ephrin A1+Defactinib (5).tif]

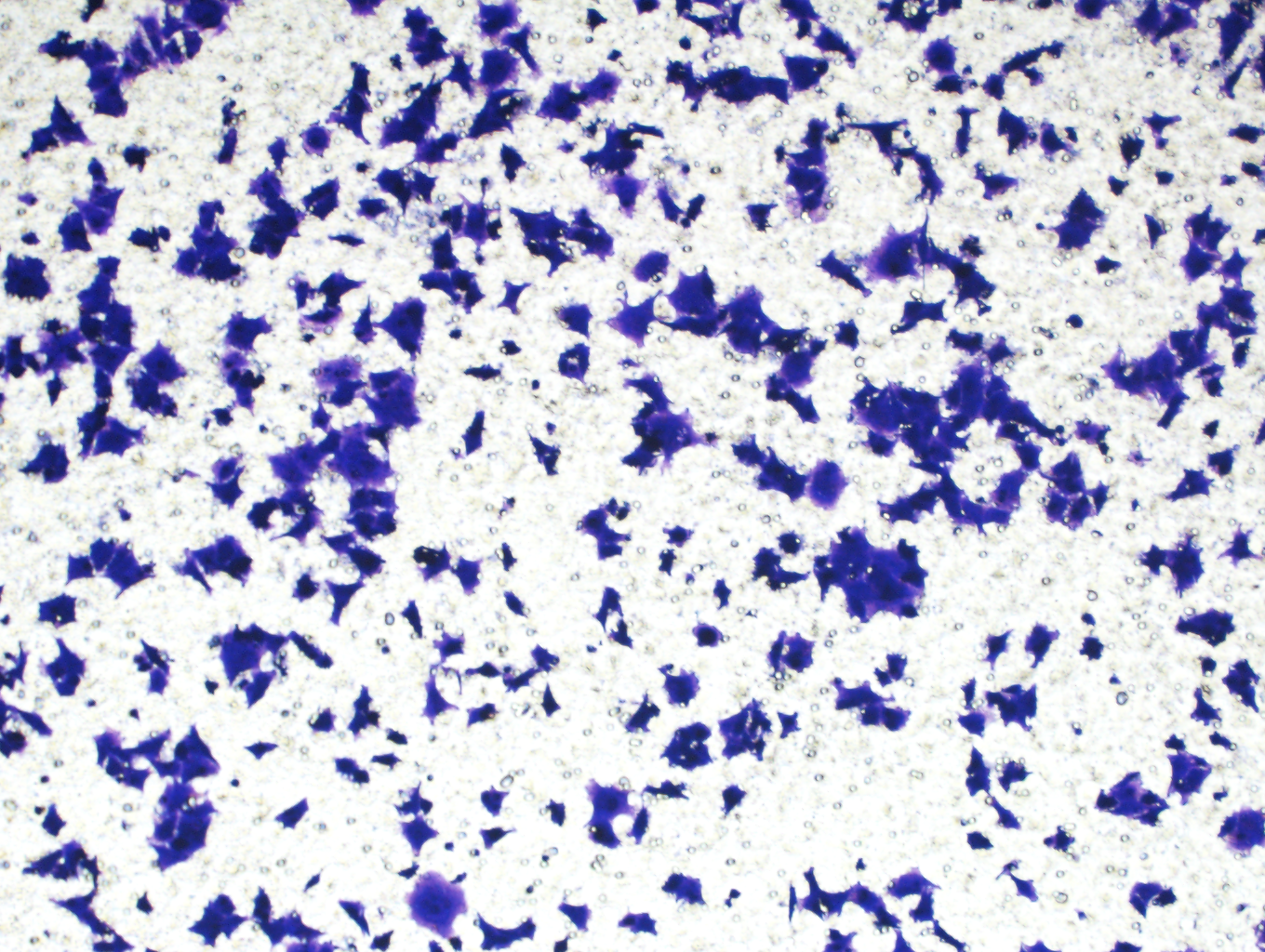

Supplement: Supplementary file 11 — EV Figure Source Data part 3 [file 44318_2025_363_MOESM11_ESM.zip › Figure EV6/EV6D/Ephrin A1+U0126 (1).tif]

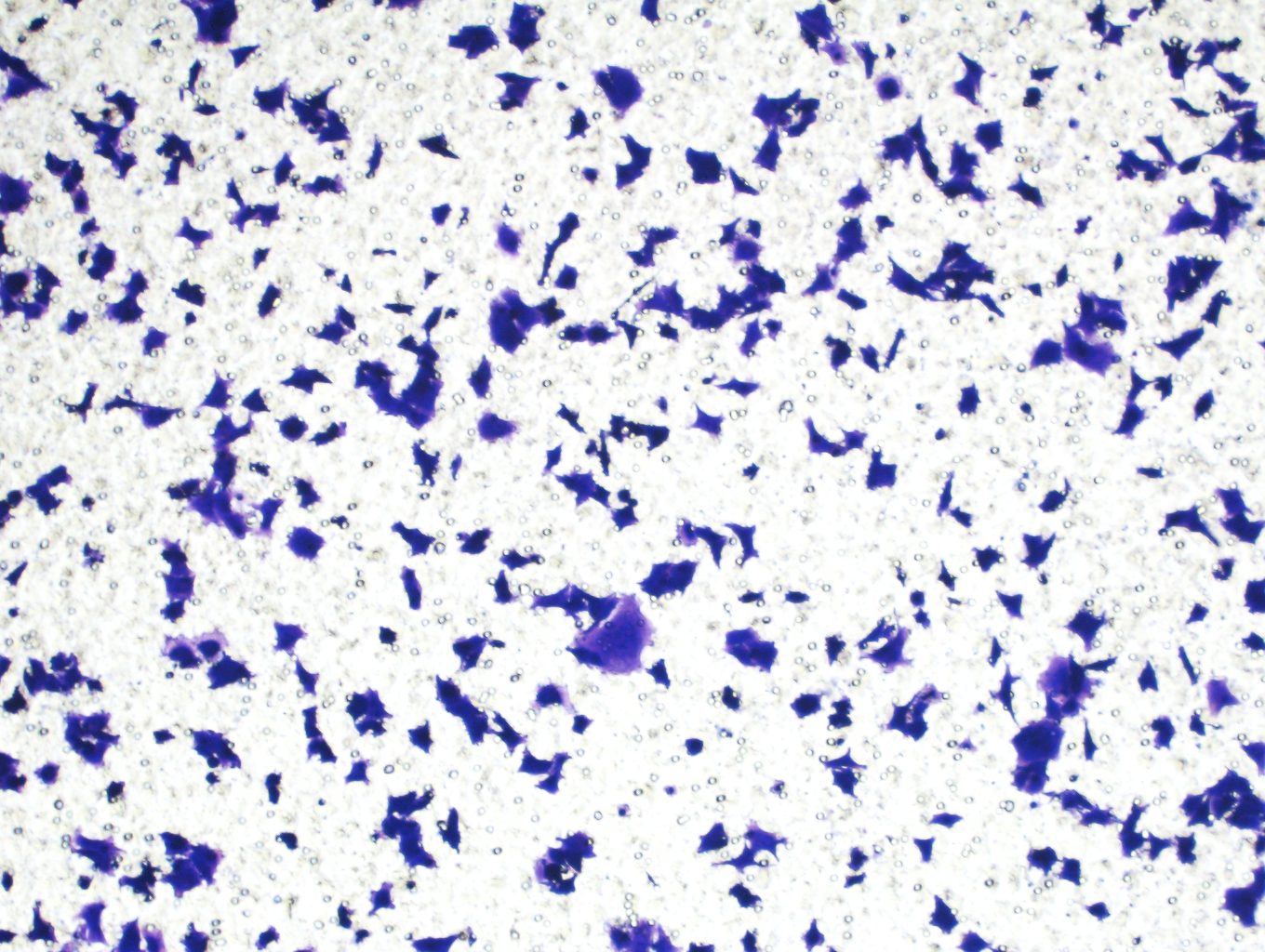

Supplement: Supplementary file 11 — EV Figure Source Data part 3 [file 44318_2025_363_MOESM11_ESM.zip › Figure EV6/EV6D/Ephrin A1+U0126 (2)-displayed in EV6D.tif]

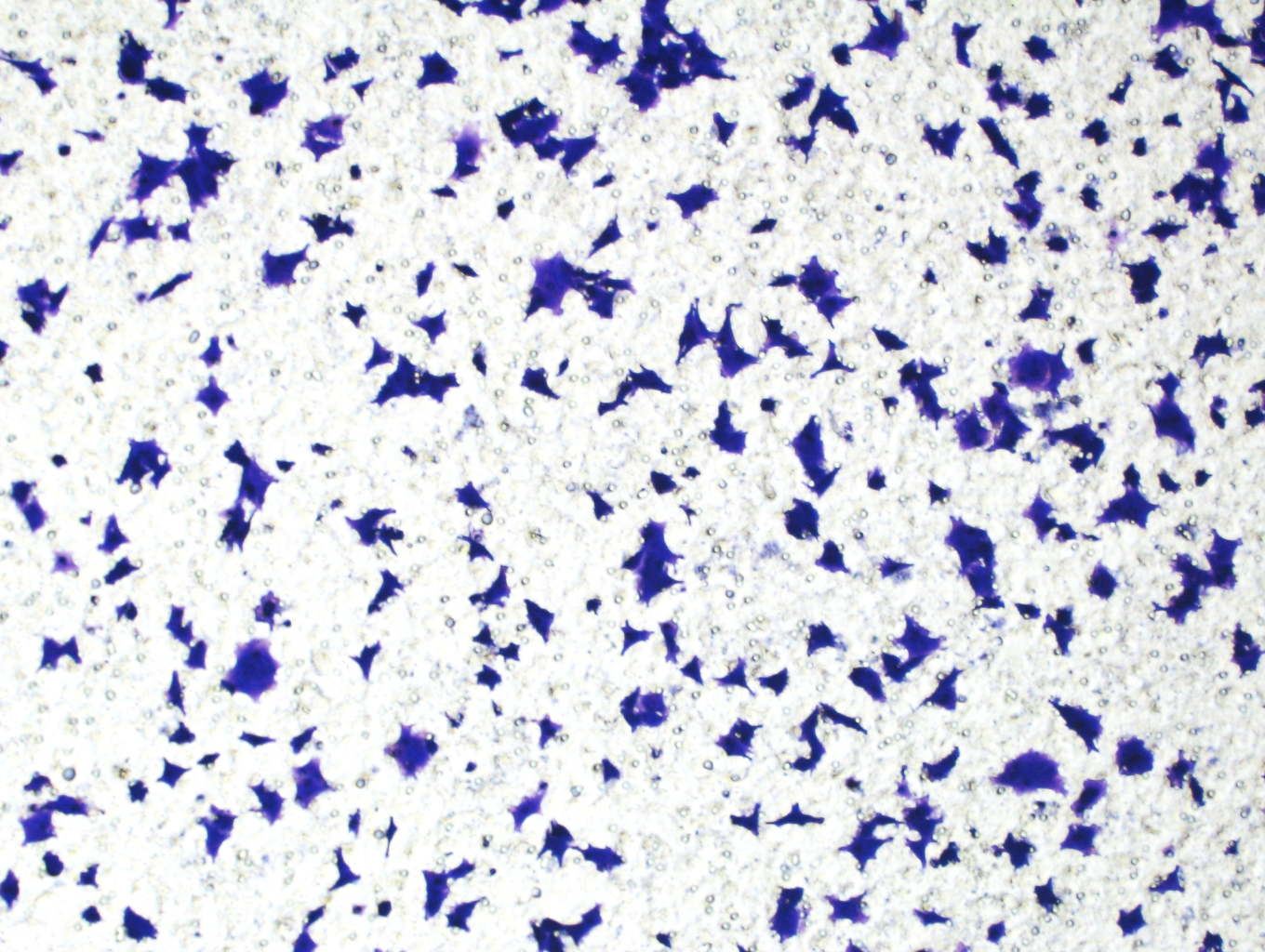

Supplement: Supplementary file 11 — EV Figure Source Data part 3 [file 44318_2025_363_MOESM11_ESM.zip › Figure EV6/EV6D/Ephrin A1+U0126 (3).tif]

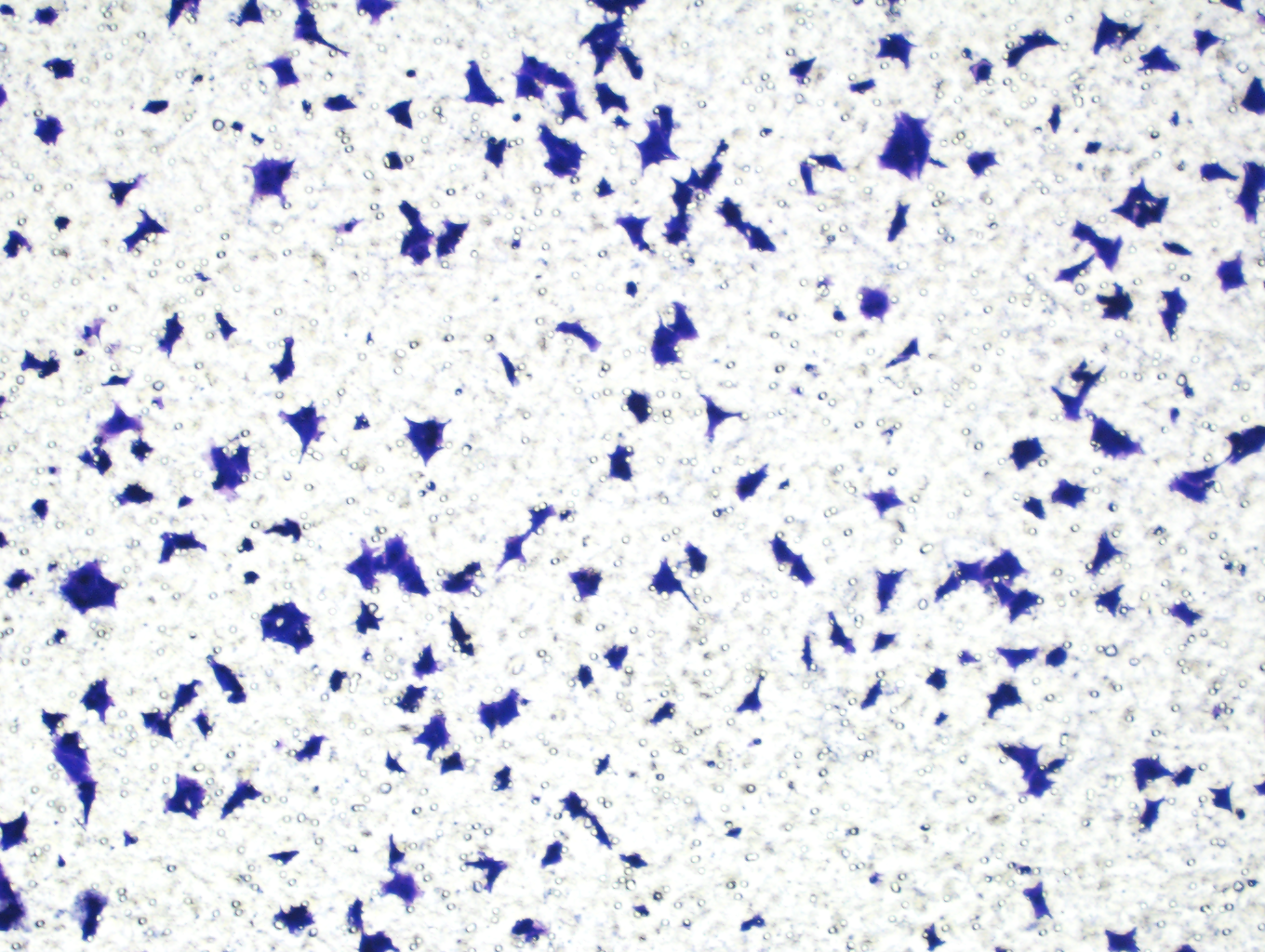

Supplement: Supplementary file 11 — EV Figure Source Data part 3 [file 44318_2025_363_MOESM11_ESM.zip › Figure EV6/EV6D/Ephrin A1+U0126 (4).tif]

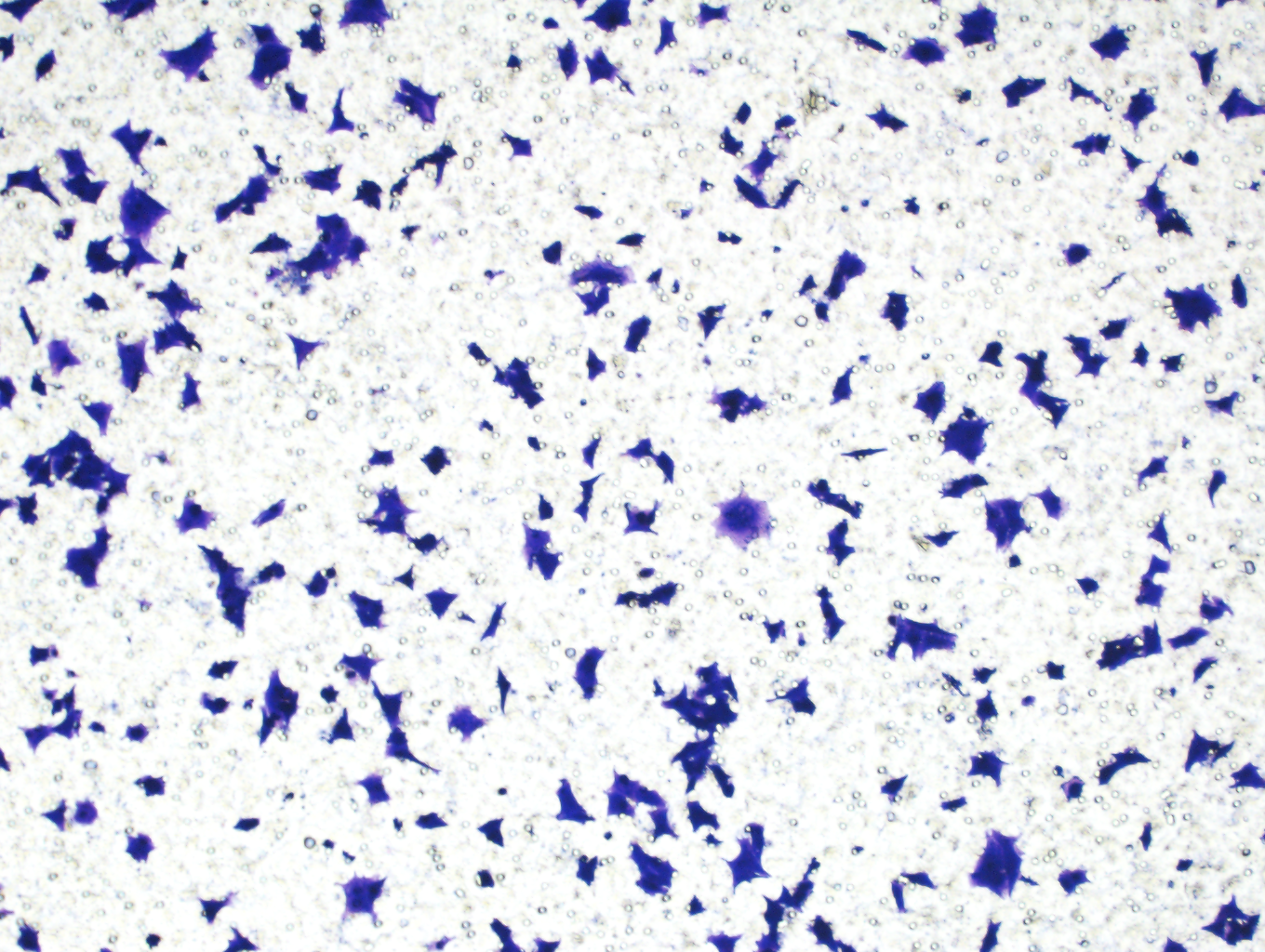

Supplement: Supplementary file 11 — EV Figure Source Data part 3 [file 44318_2025_363_MOESM11_ESM.zip › Figure EV6/EV6D/Ephrin A1+U0126 (5).tif]

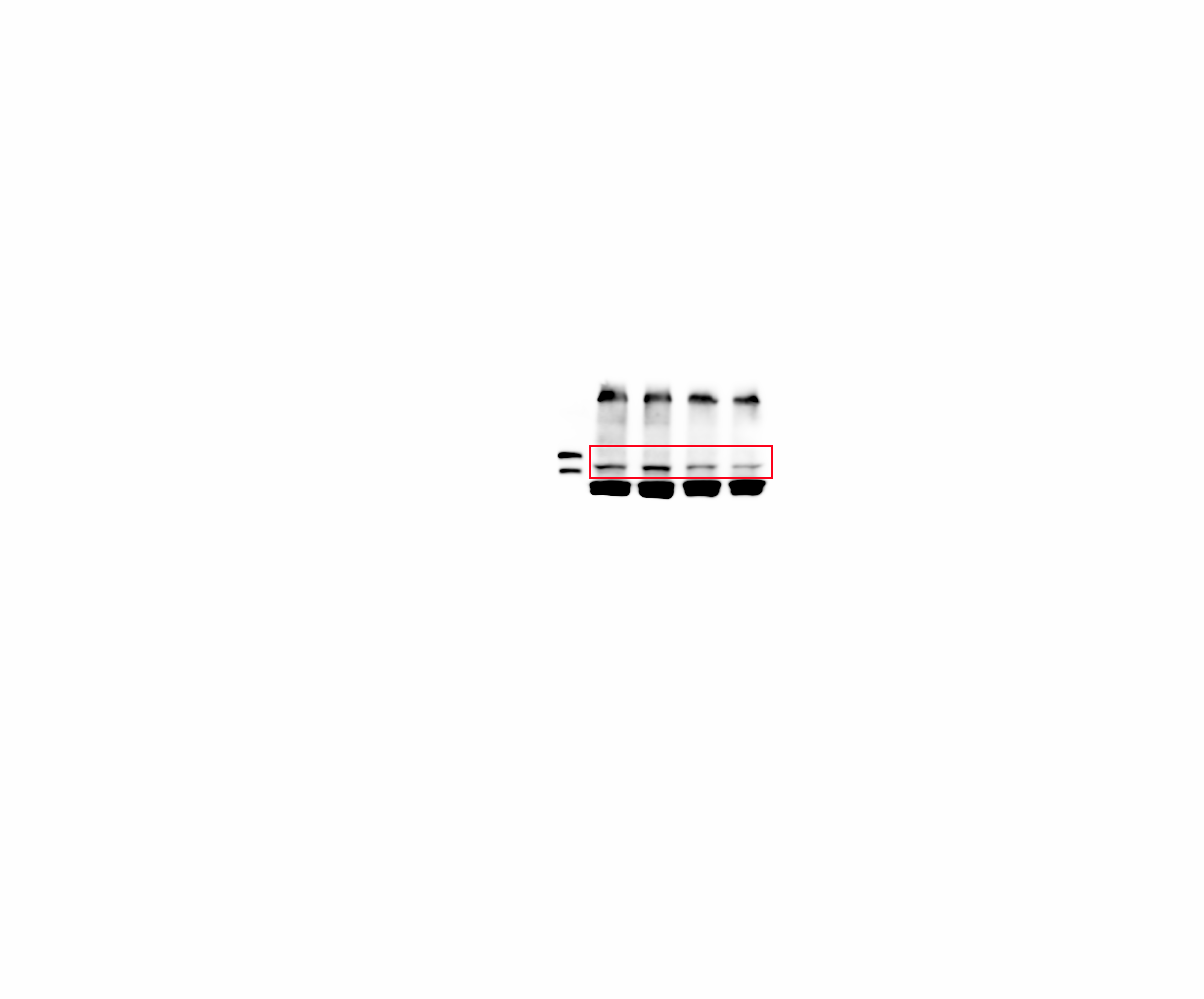

Supplement: Supplementary file 11 — EV Figure Source Data part 3 [file 44318_2025_363_MOESM11_ESM.zip › Figure EV6/EV6F/2 N-cad.tif]

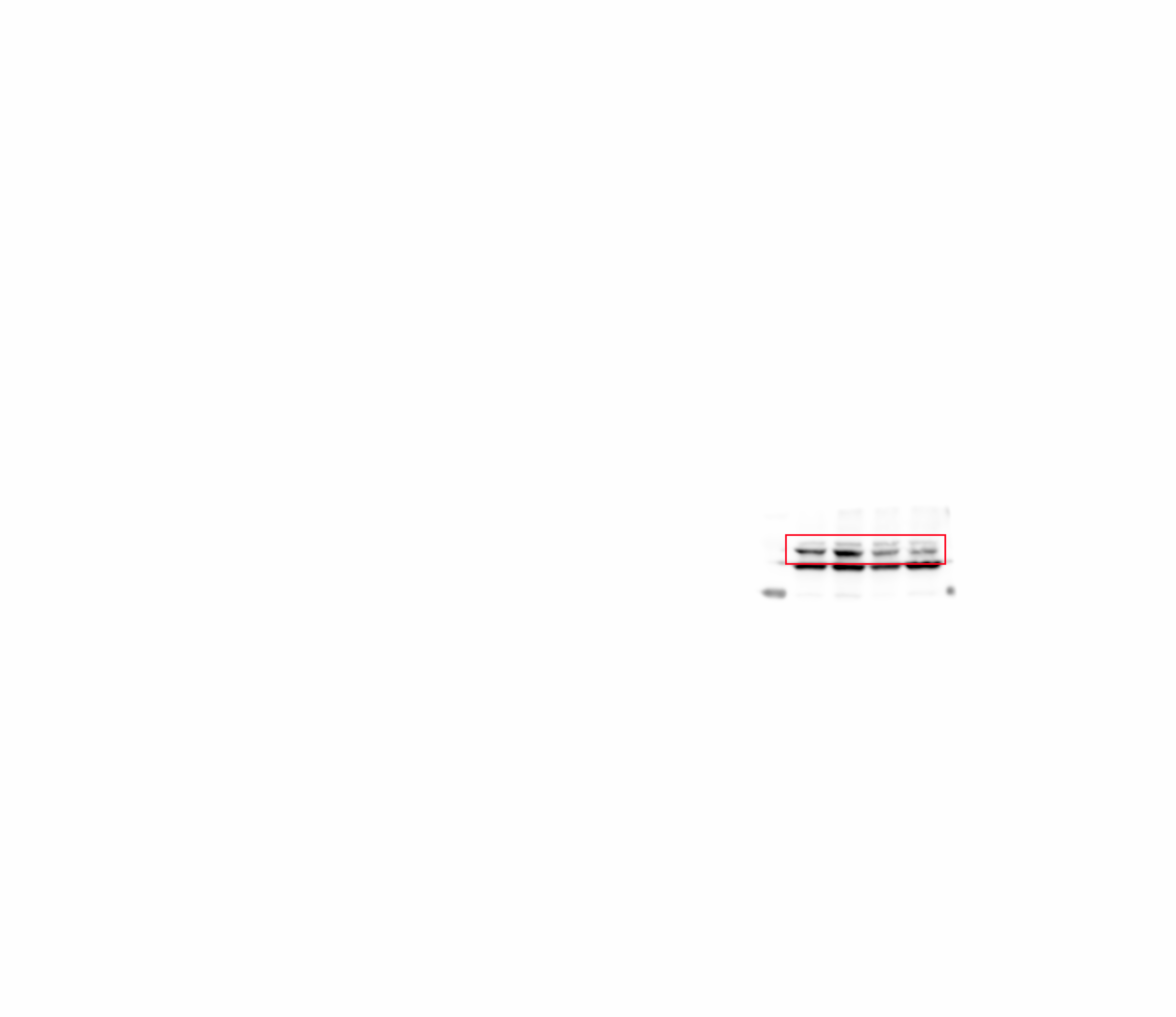

Supplement: Supplementary file 11 — EV Figure Source Data part 3 [file 44318_2025_363_MOESM11_ESM.zip › Figure EV6/EV6F/3 vimentin.tif]

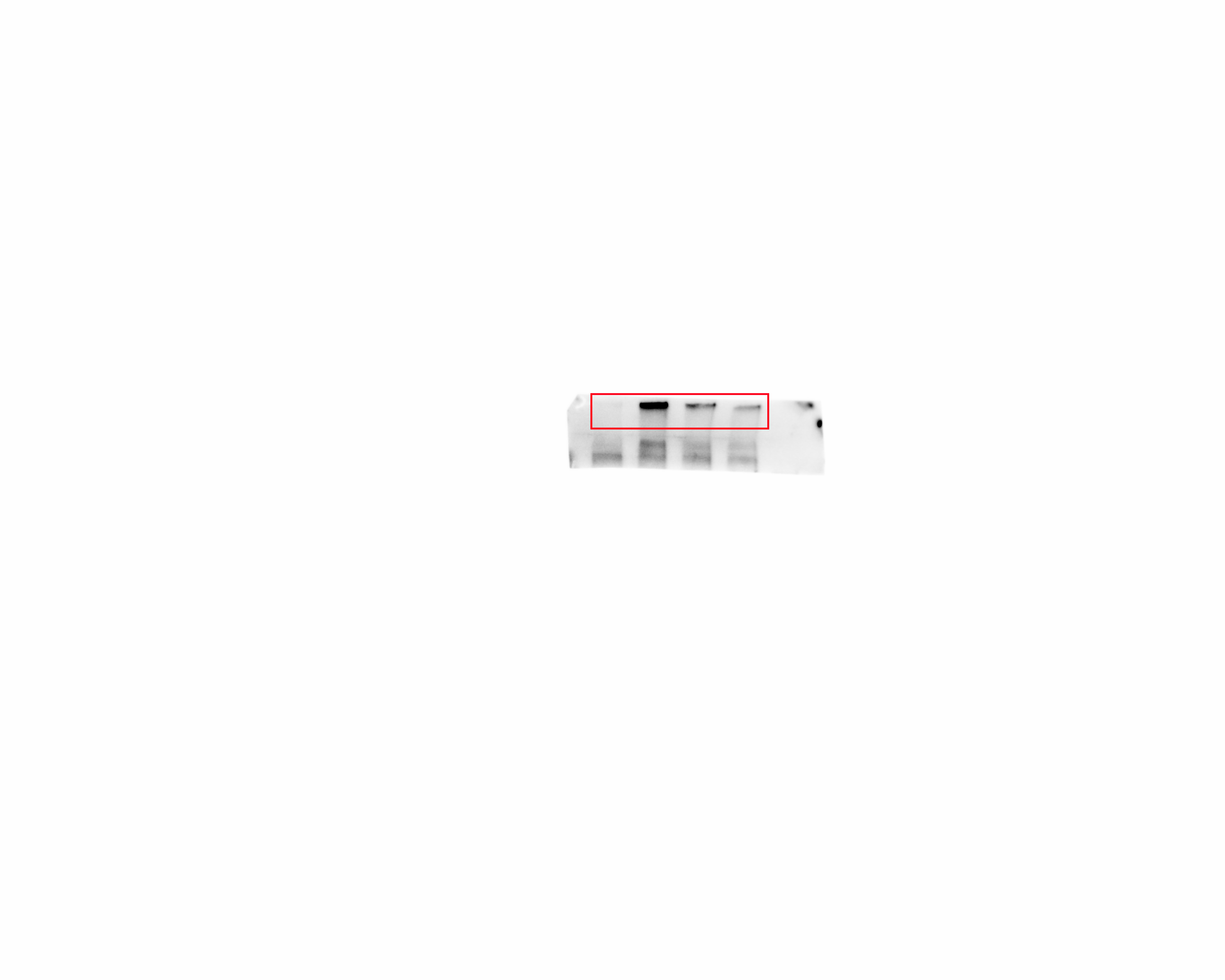

Supplement: Supplementary file 11 — EV Figure Source Data part 3 [file 44318_2025_363_MOESM11_ESM.zip › Figure EV6/EV6F/4 ZEB1.tif]

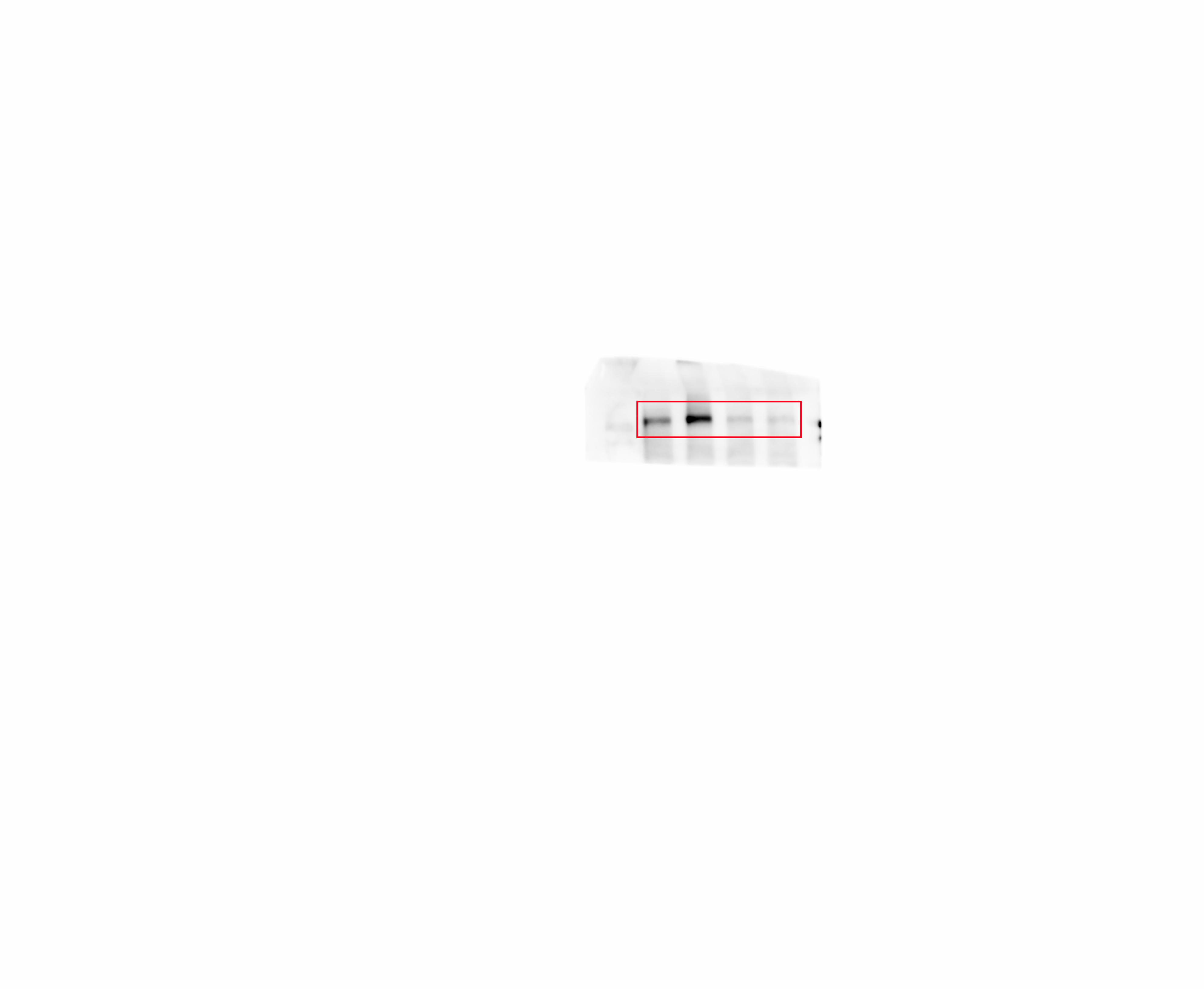

Supplement: Supplementary file 11 — EV Figure Source Data part 3 [file 44318_2025_363_MOESM11_ESM.zip › Figure EV6/EV6F/5 p-EGFR.tif]

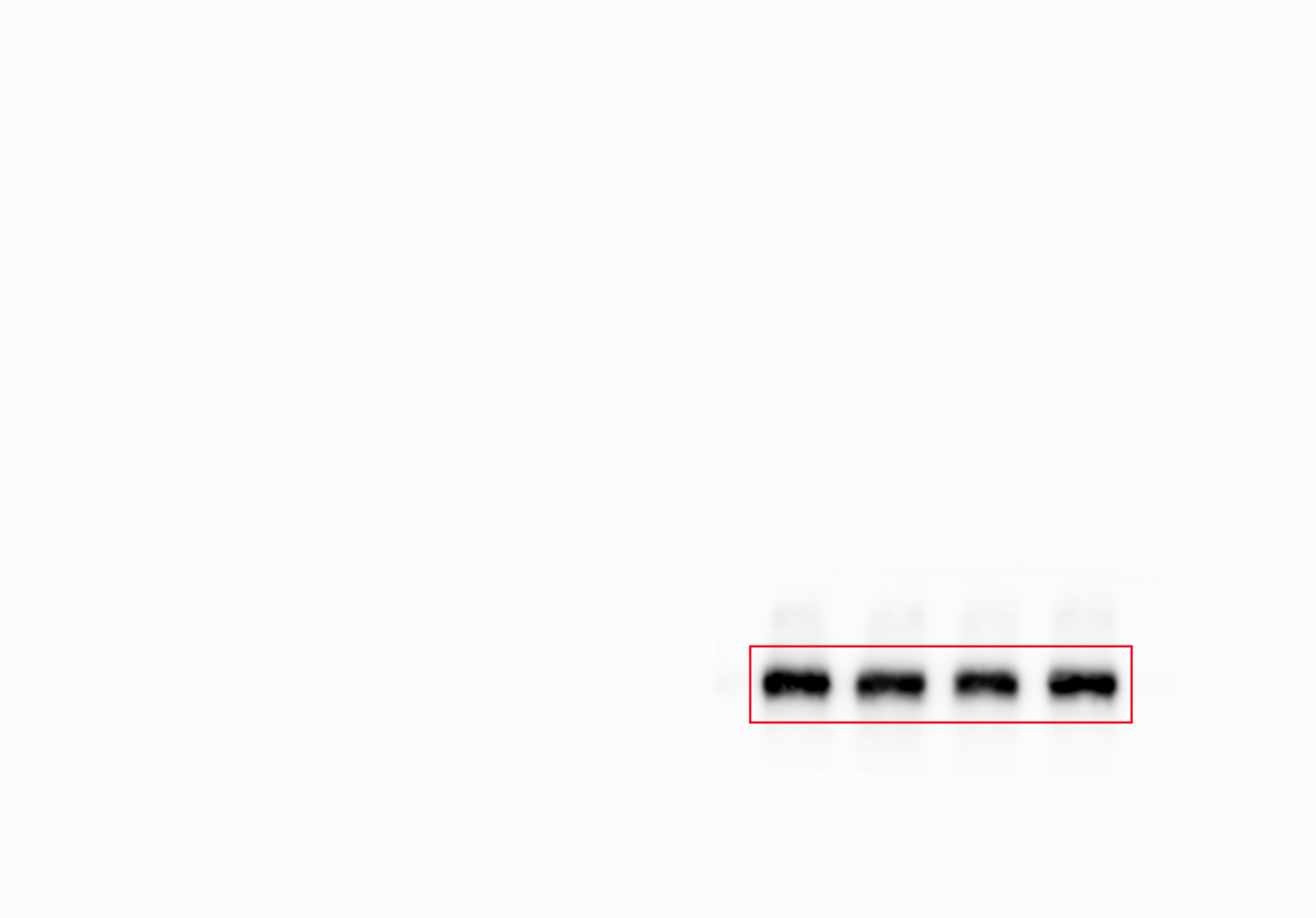

Supplement: Supplementary file 11 — EV Figure Source Data part 3 [file 44318_2025_363_MOESM11_ESM.zip › Figure EV6/EV6F/6 EGFR.tif]

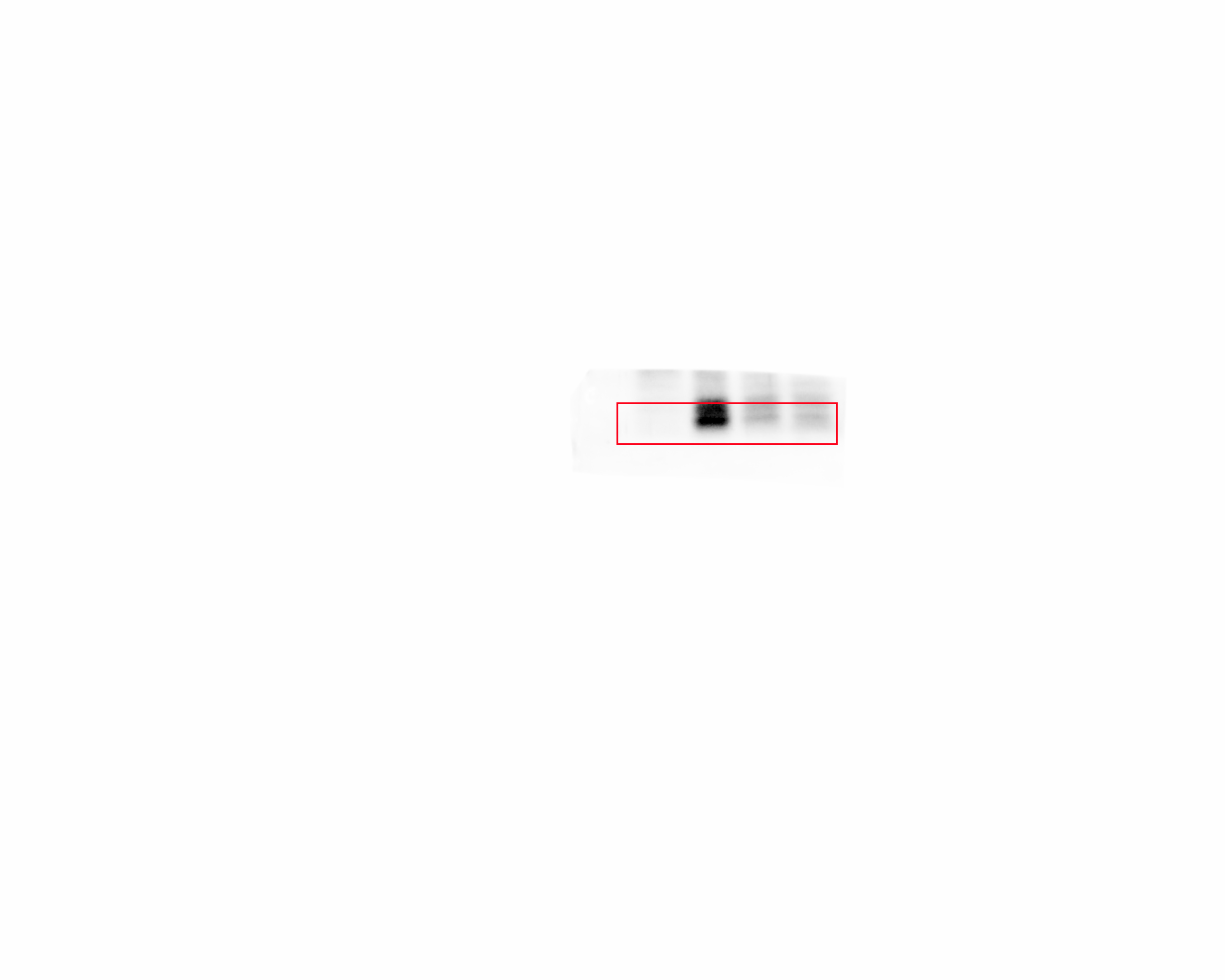

Supplement: Supplementary file 11 — EV Figure Source Data part 3 [file 44318_2025_363_MOESM11_ESM.zip › Figure EV6/EV6F/7 Ephrin A1.tif]

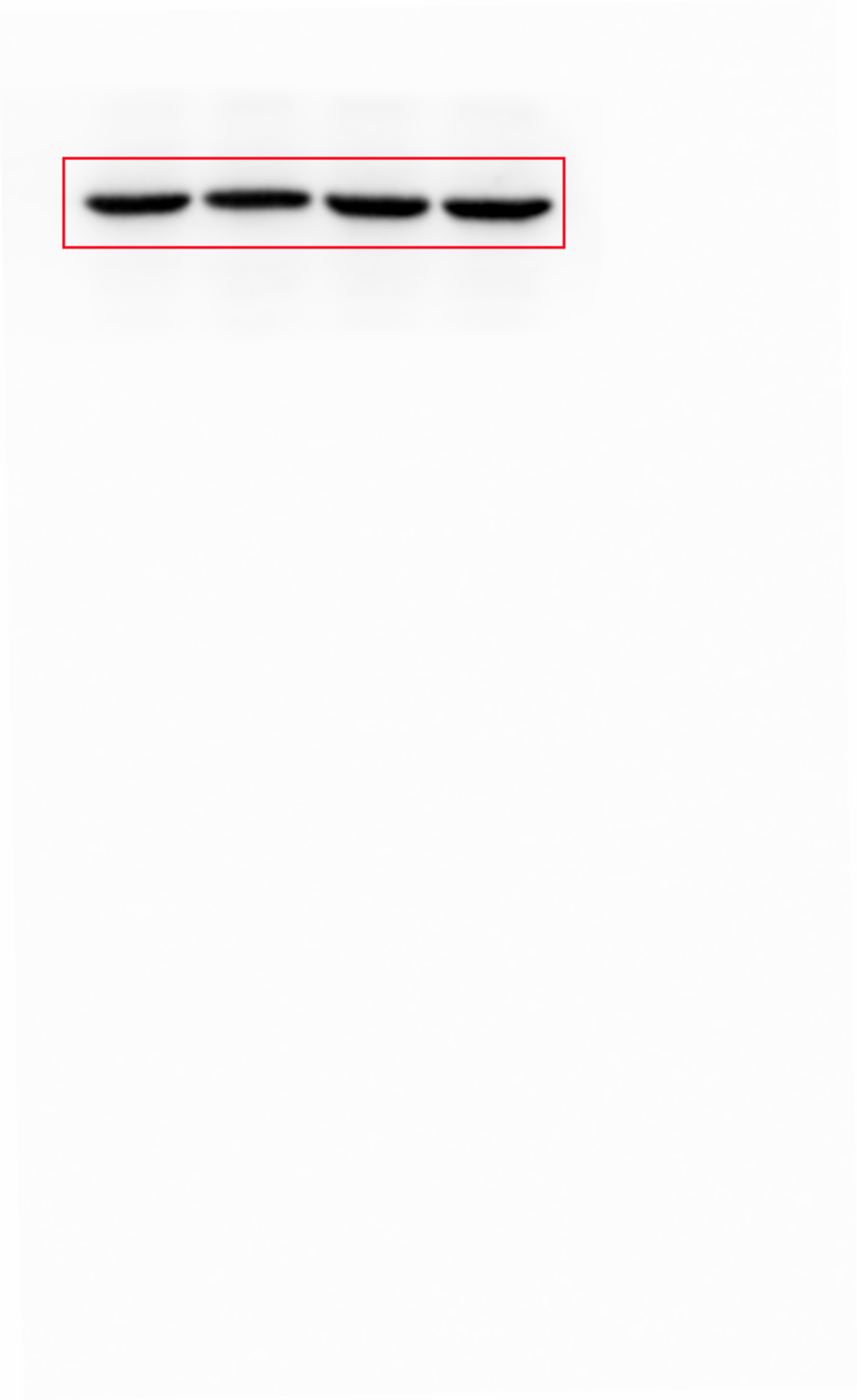

Supplement: Supplementary file 11 — EV Figure Source Data part 3 [file 44318_2025_363_MOESM11_ESM.zip › Figure EV6/EV6F/8 actin.tif]

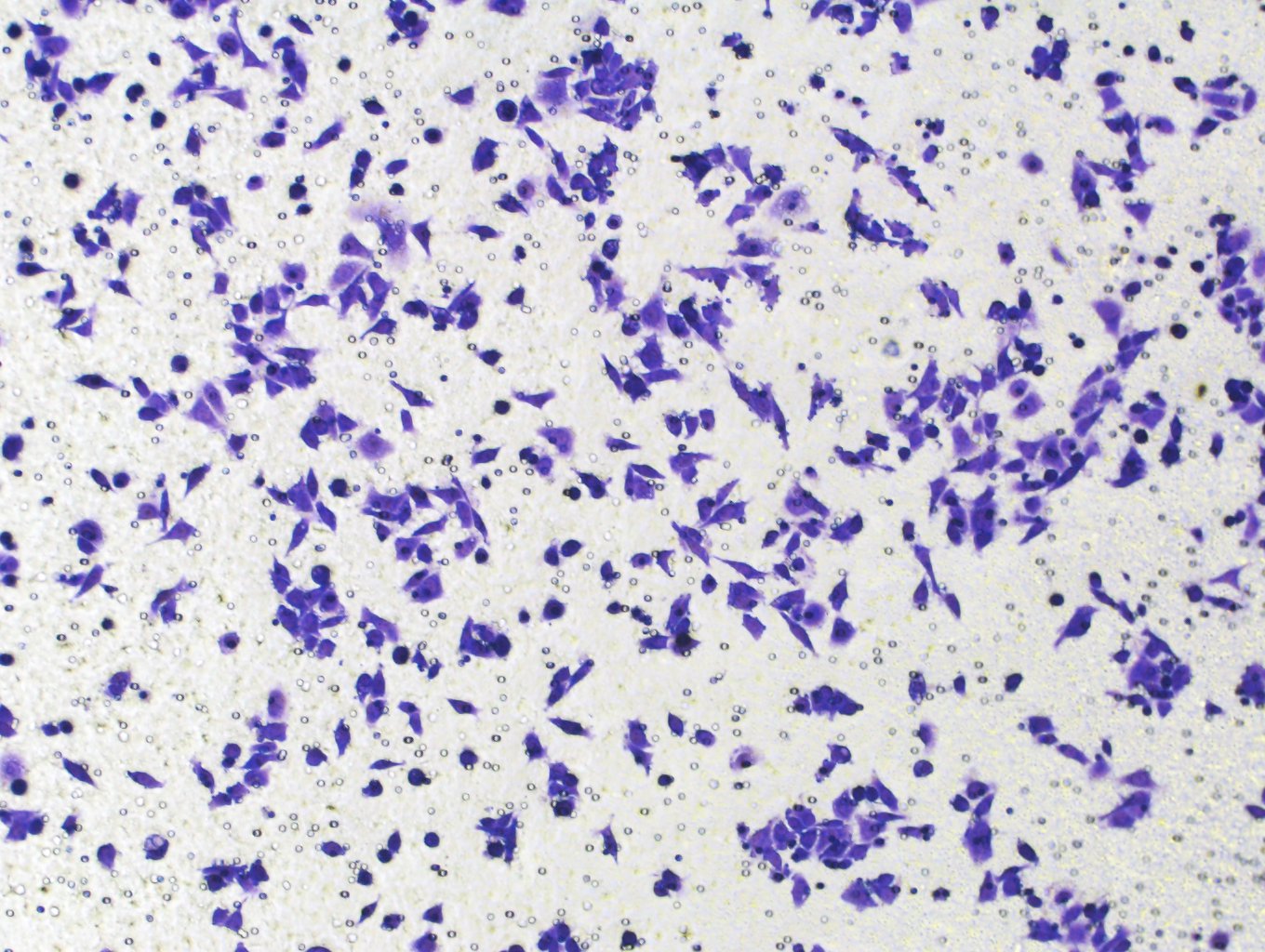

Supplement: Supplementary file 11 — EV Figure Source Data part 3 [file 44318_2025_363_MOESM11_ESM.zip › Figure EV6/EV6G/Control (1)-displayed in EV6G.jpg]

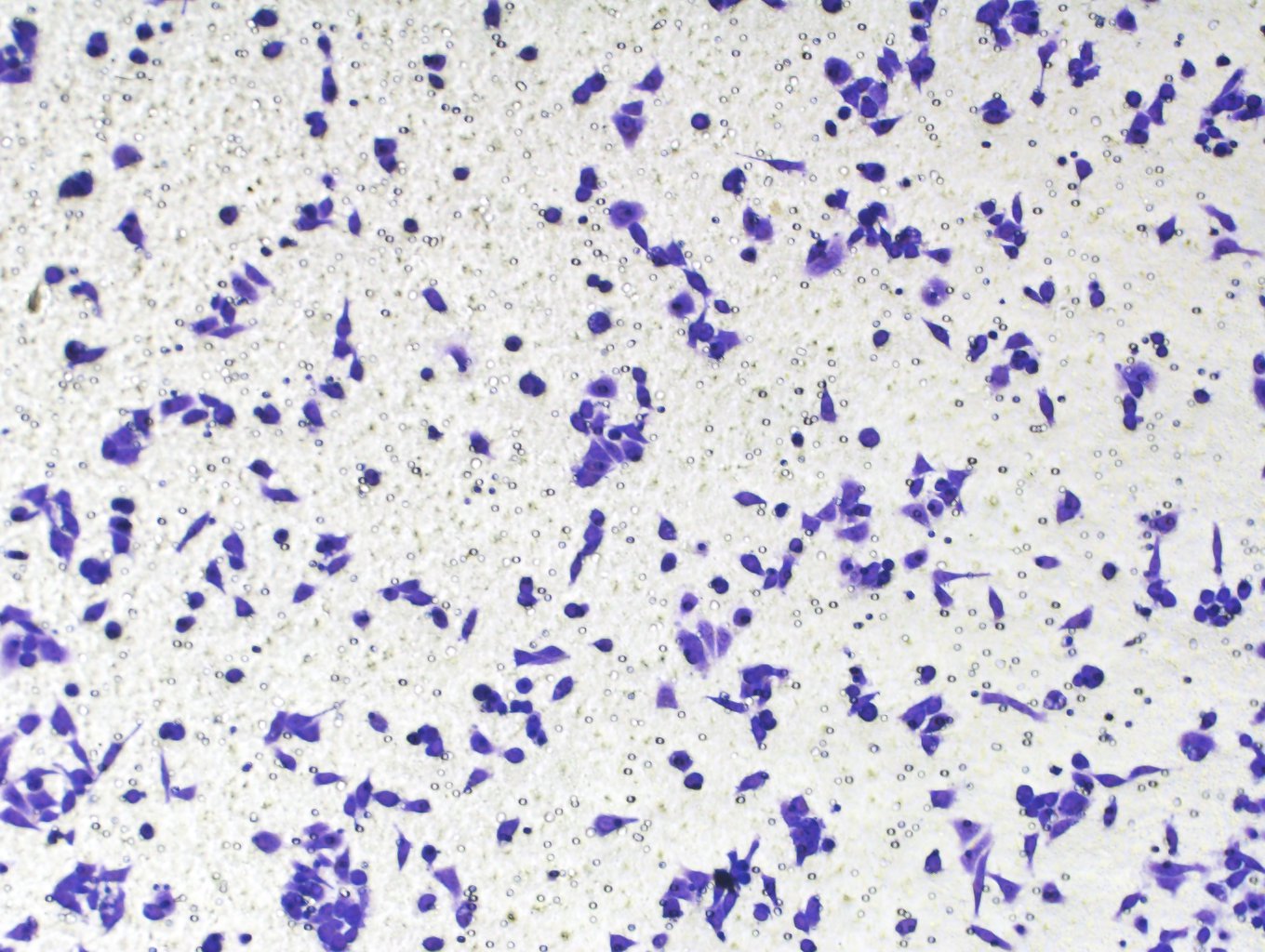

Supplement: Supplementary file 11 — EV Figure Source Data part 3 [file 44318_2025_363_MOESM11_ESM.zip › Figure EV6/EV6G/Control (2).jpg]

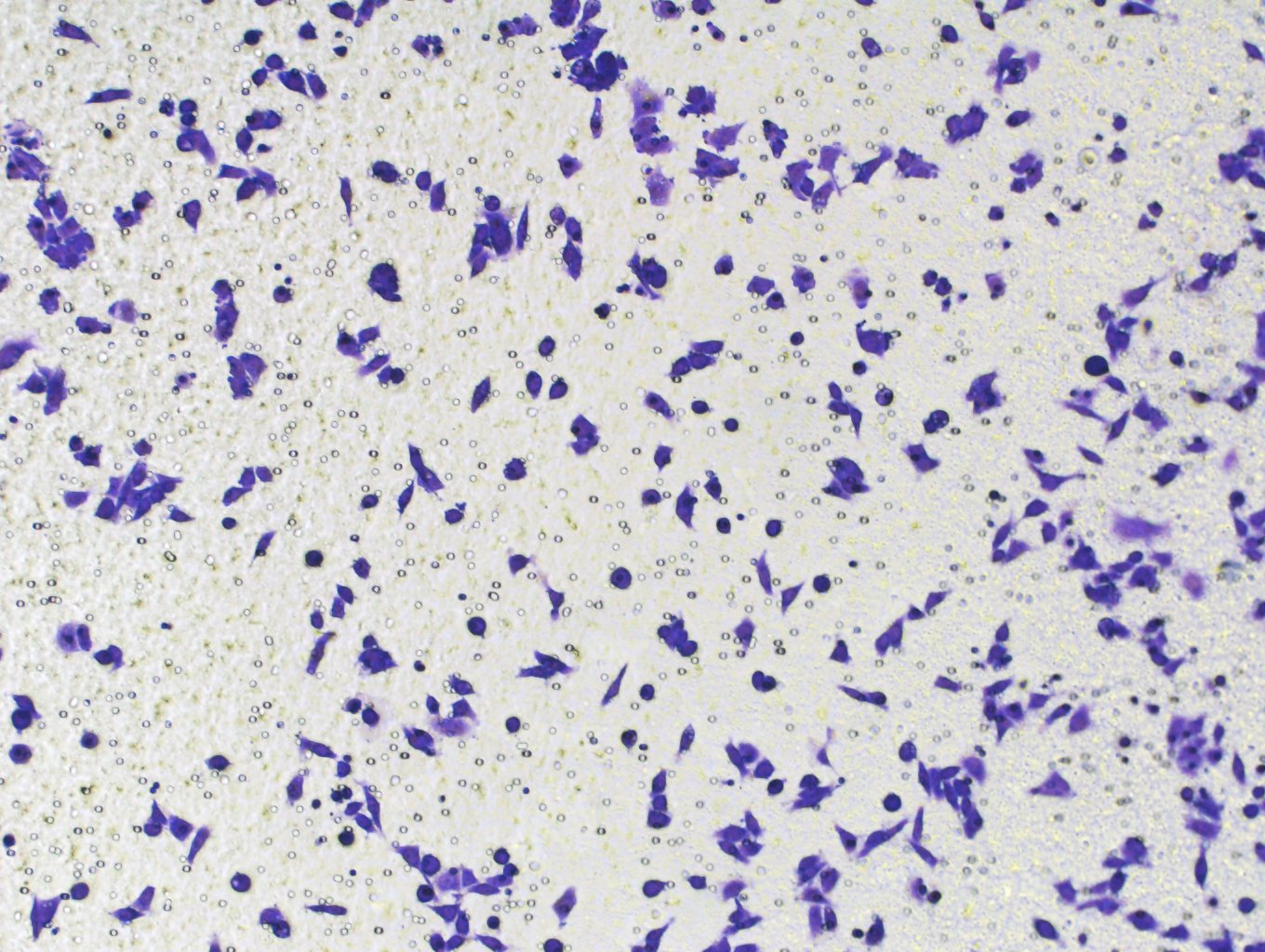

Supplement: Supplementary file 11 — EV Figure Source Data part 3 [file 44318_2025_363_MOESM11_ESM.zip › Figure EV6/EV6G/Control (3).jpg]

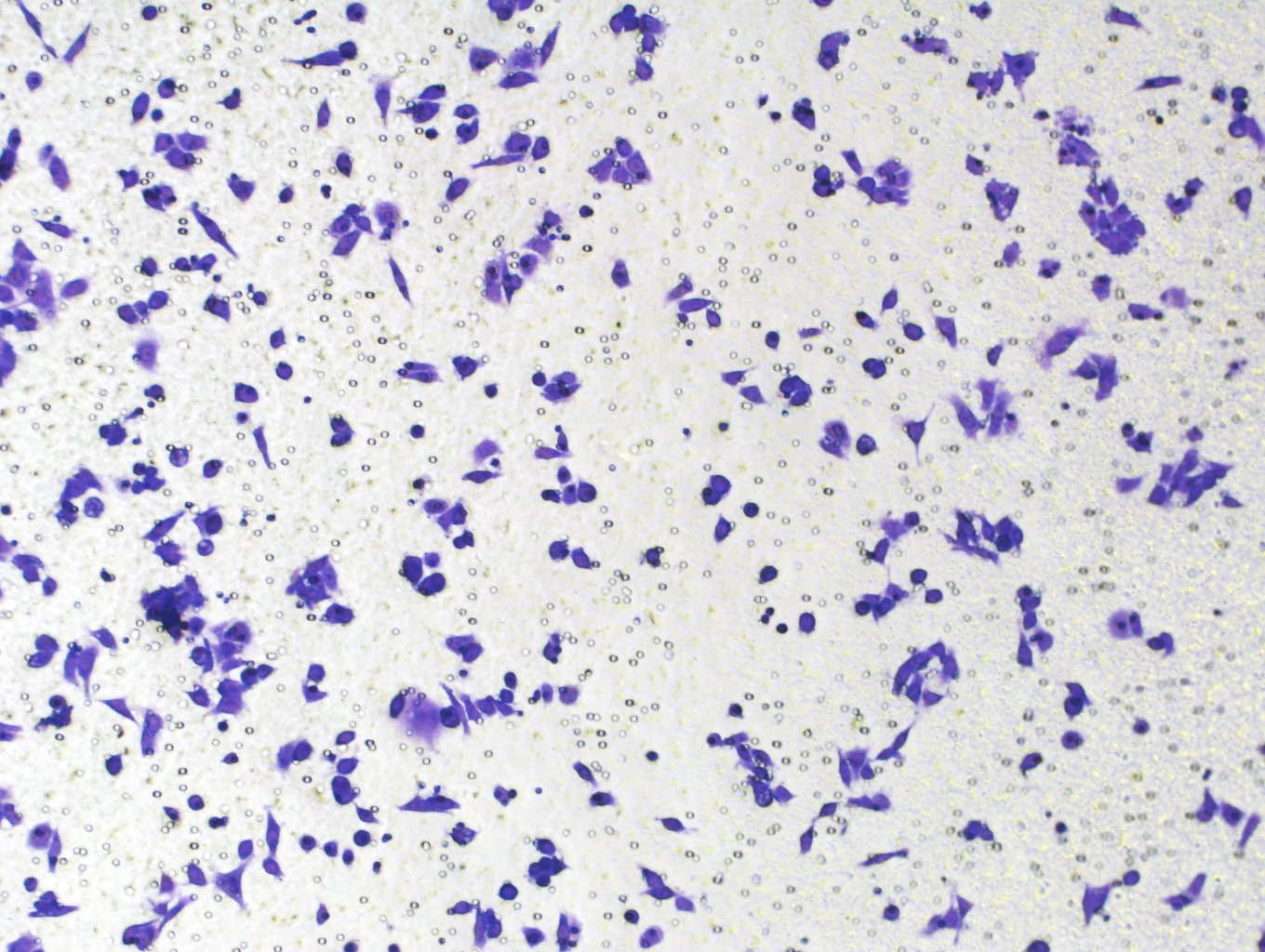

Supplement: Supplementary file 11 — EV Figure Source Data part 3 [file 44318_2025_363_MOESM11_ESM.zip › Figure EV6/EV6G/Control (4).jpg]

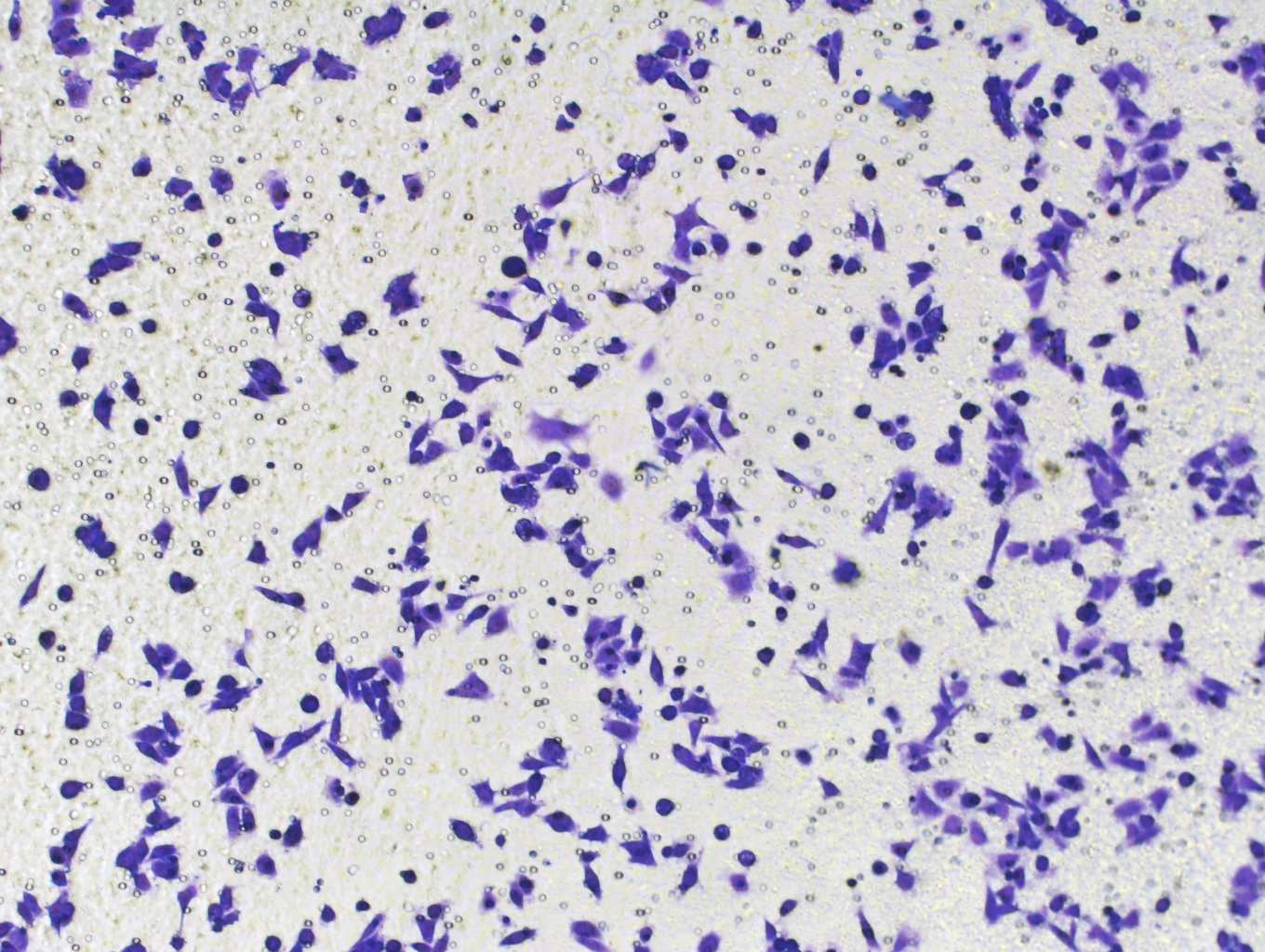

Supplement: Supplementary file 11 — EV Figure Source Data part 3 [file 44318_2025_363_MOESM11_ESM.zip › Figure EV6/EV6G/Control (5).jpg]

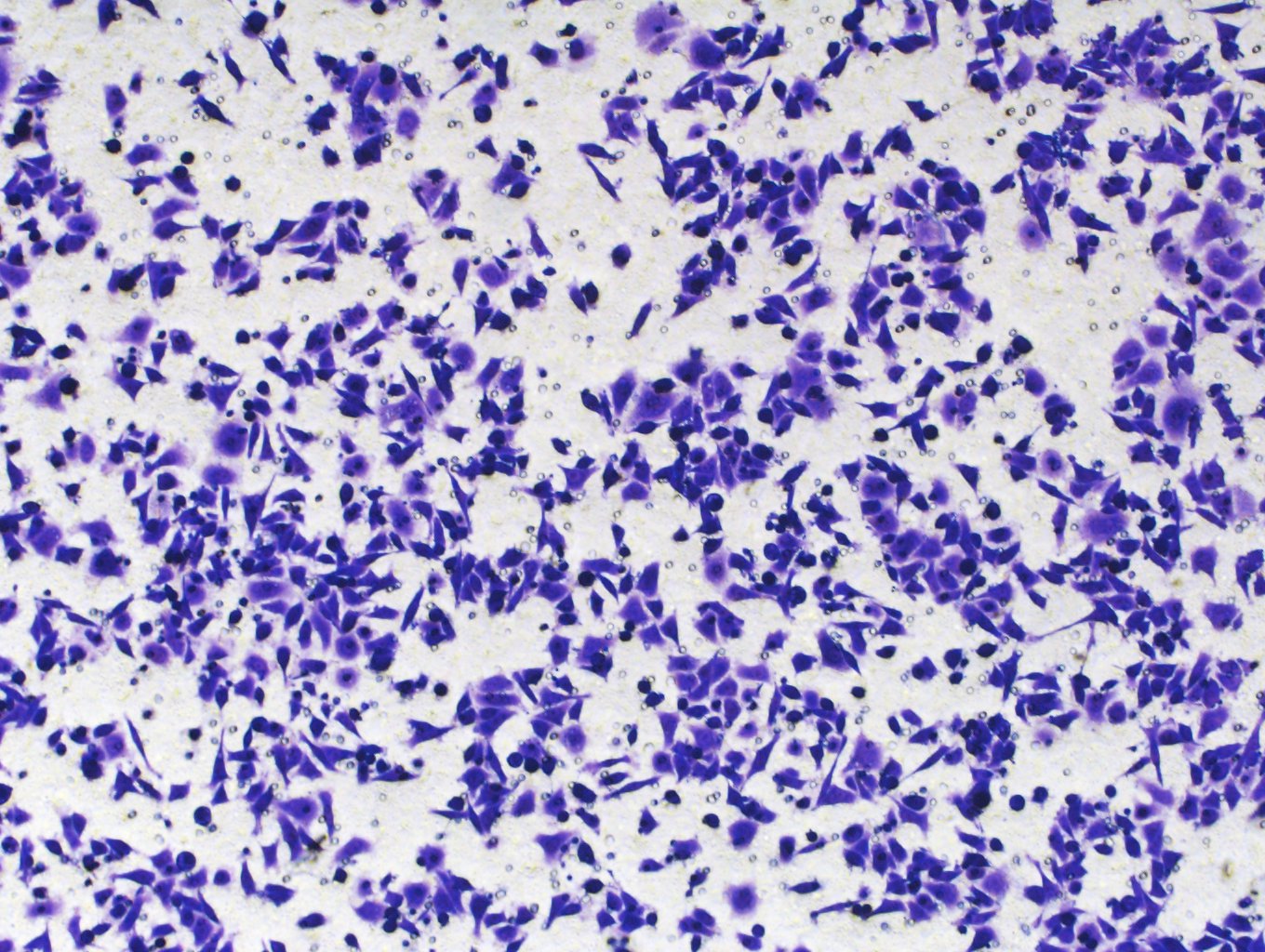

Supplement: Supplementary file 11 — EV Figure Source Data part 3 [file 44318_2025_363_MOESM11_ESM.zip › Figure EV6/EV6G/Ephrin A1 (1).jpg]

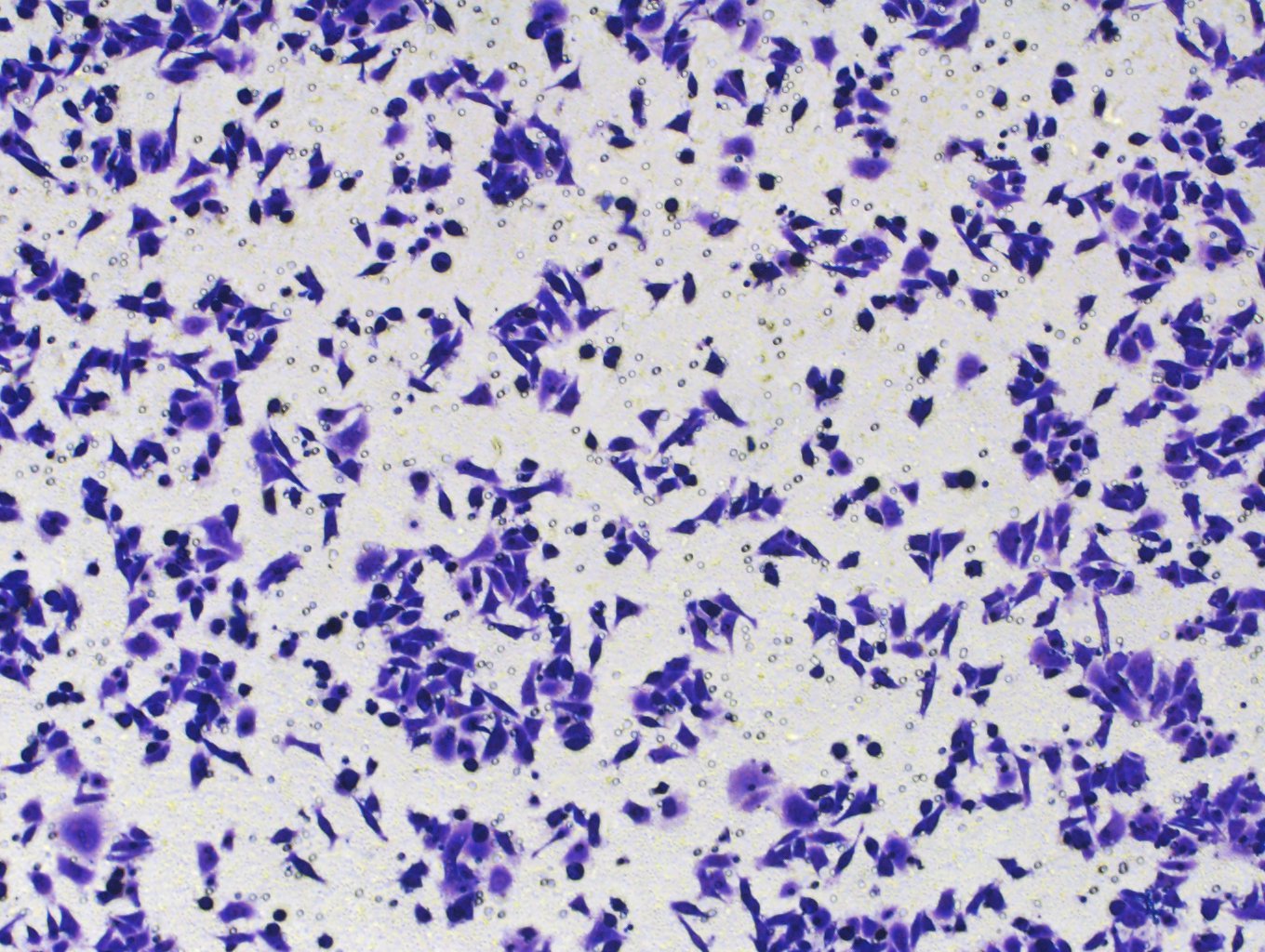

Supplement: Supplementary file 11 — EV Figure Source Data part 3 [file 44318_2025_363_MOESM11_ESM.zip › Figure EV6/EV6G/Ephrin A1 (2)-displayed in EV6G.jpg]

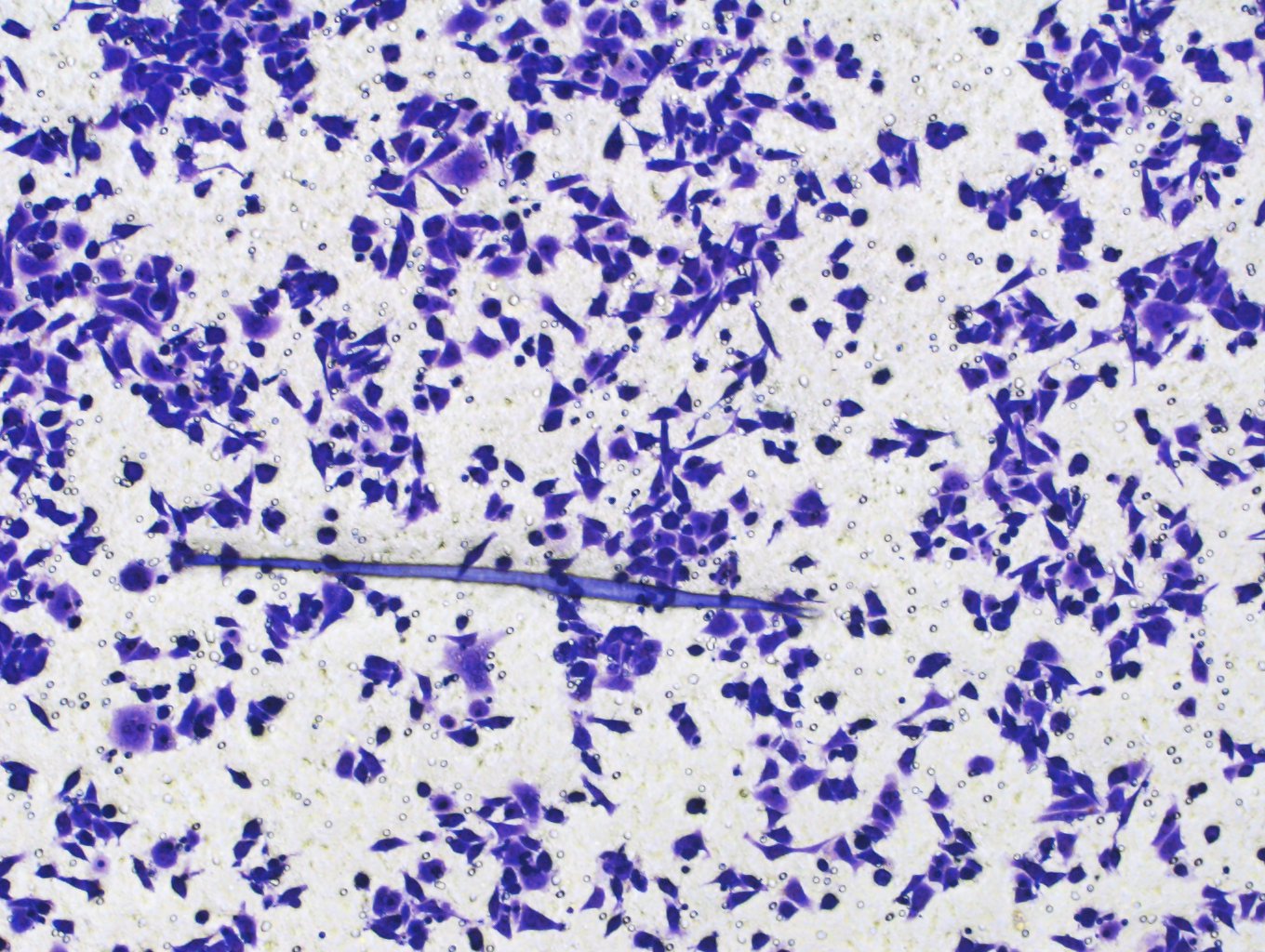

Supplement: Supplementary file 11 — EV Figure Source Data part 3 [file 44318_2025_363_MOESM11_ESM.zip › Figure EV6/EV6G/Ephrin A1 (3).jpg]

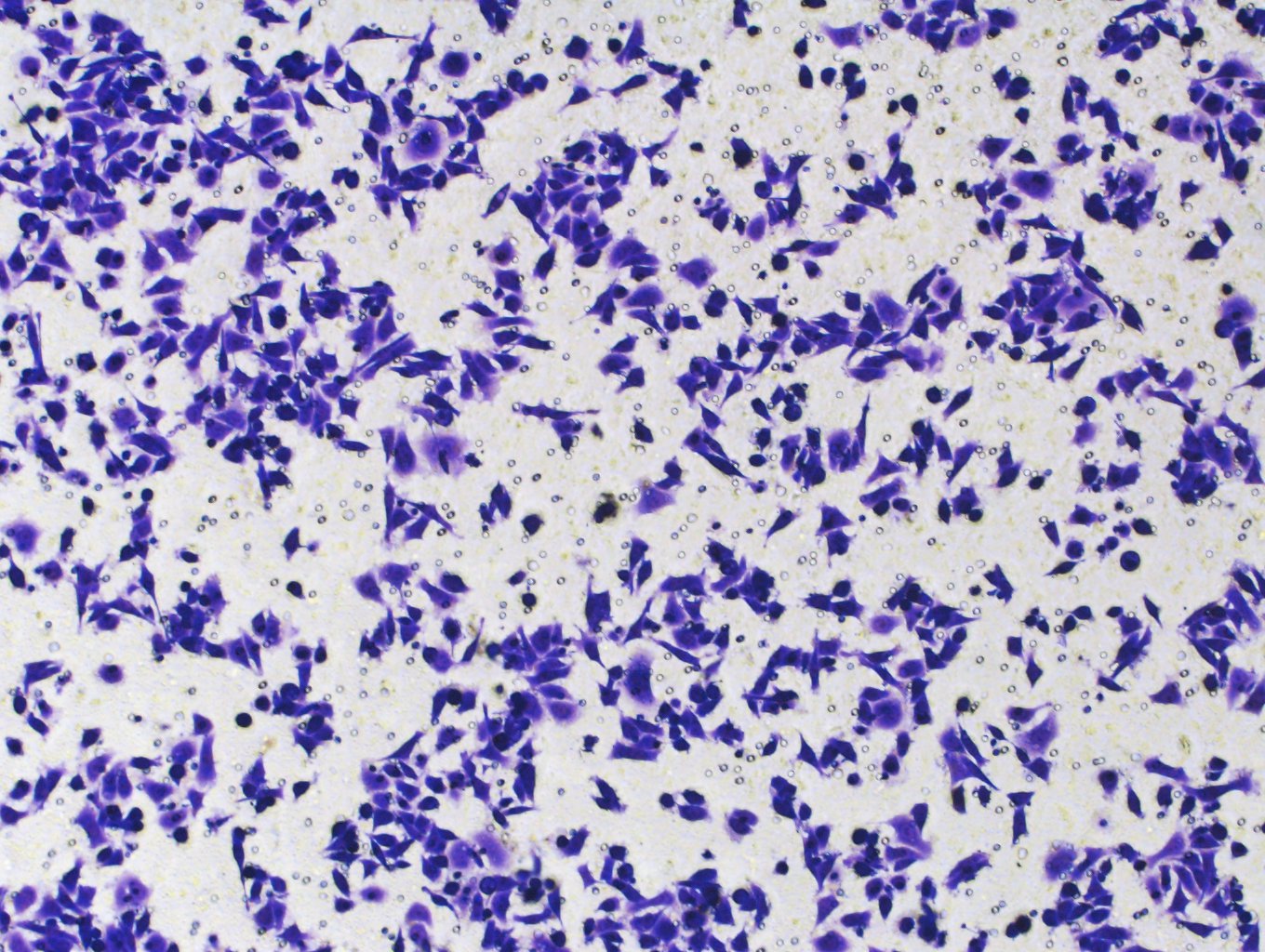

Supplement: Supplementary file 11 — EV Figure Source Data part 3 [file 44318_2025_363_MOESM11_ESM.zip › Figure EV6/EV6G/Ephrin A1 (4).jpg]

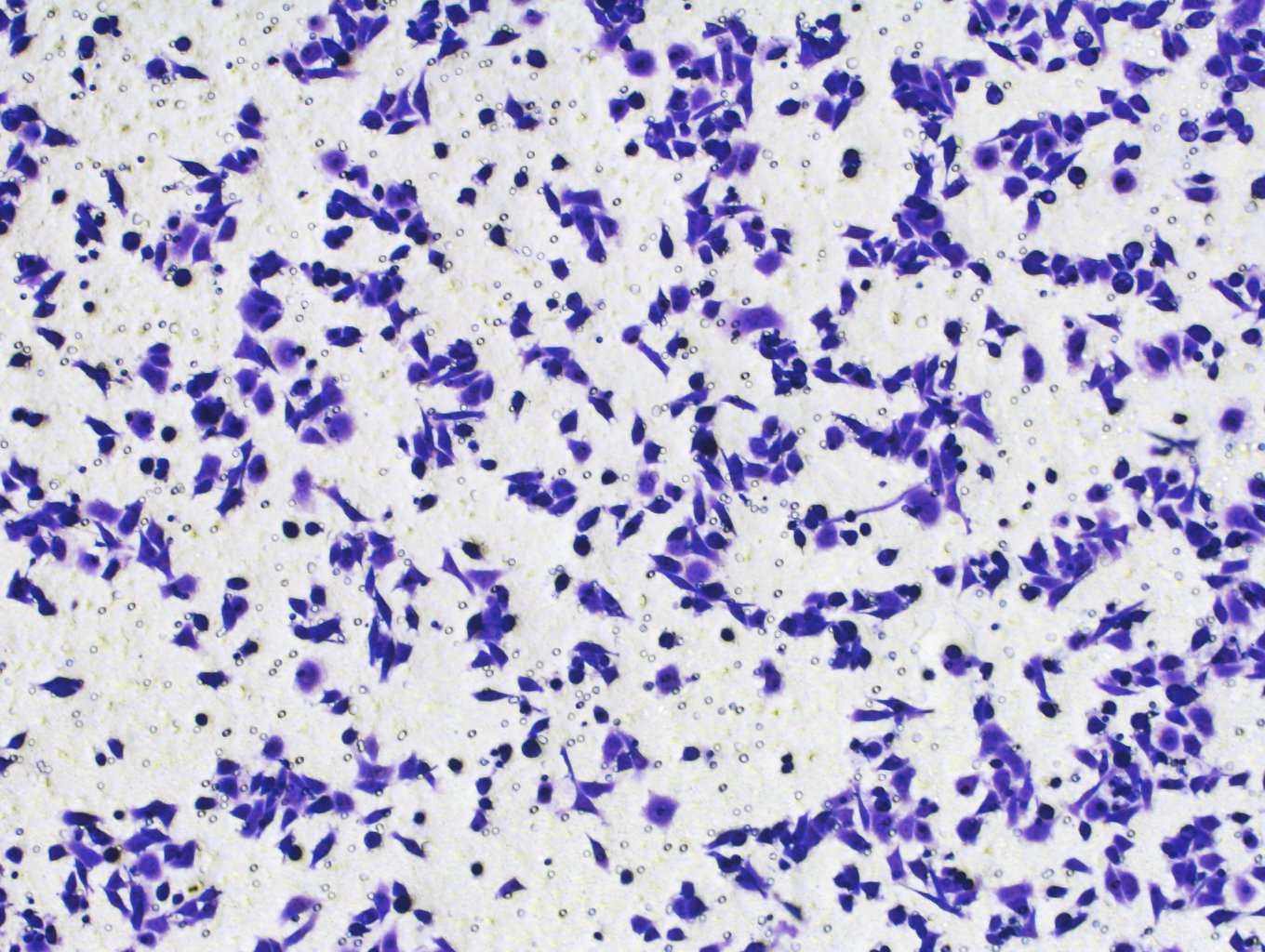

Supplement: Supplementary file 11 — EV Figure Source Data part 3 [file 44318_2025_363_MOESM11_ESM.zip › Figure EV6/EV6G/Ephrin A1 (5).jpg]

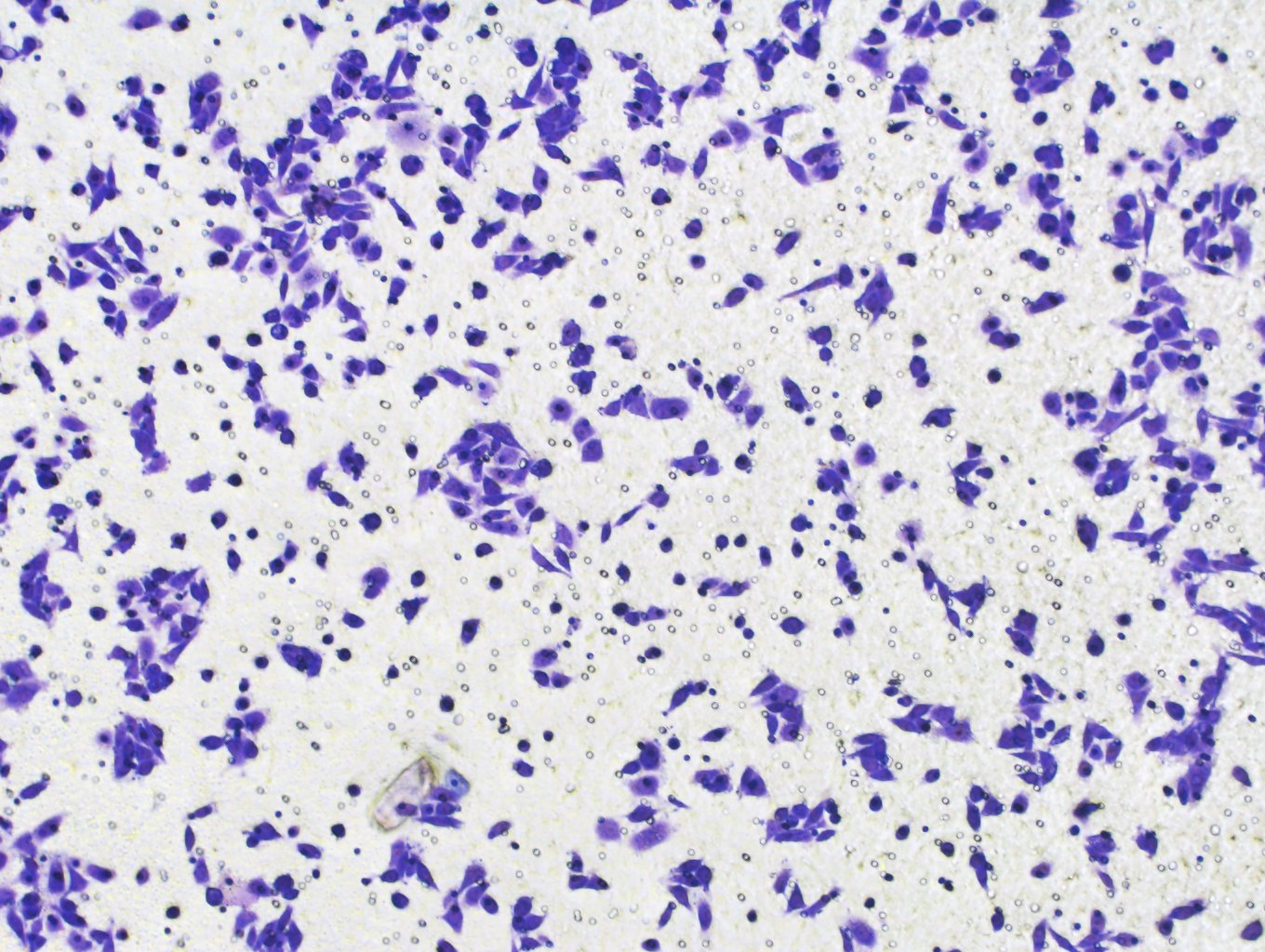

Supplement: Supplementary file 11 — EV Figure Source Data part 3 [file 44318_2025_363_MOESM11_ESM.zip › Figure EV6/EV6G/Ephrin A1+10um (1).jpg]

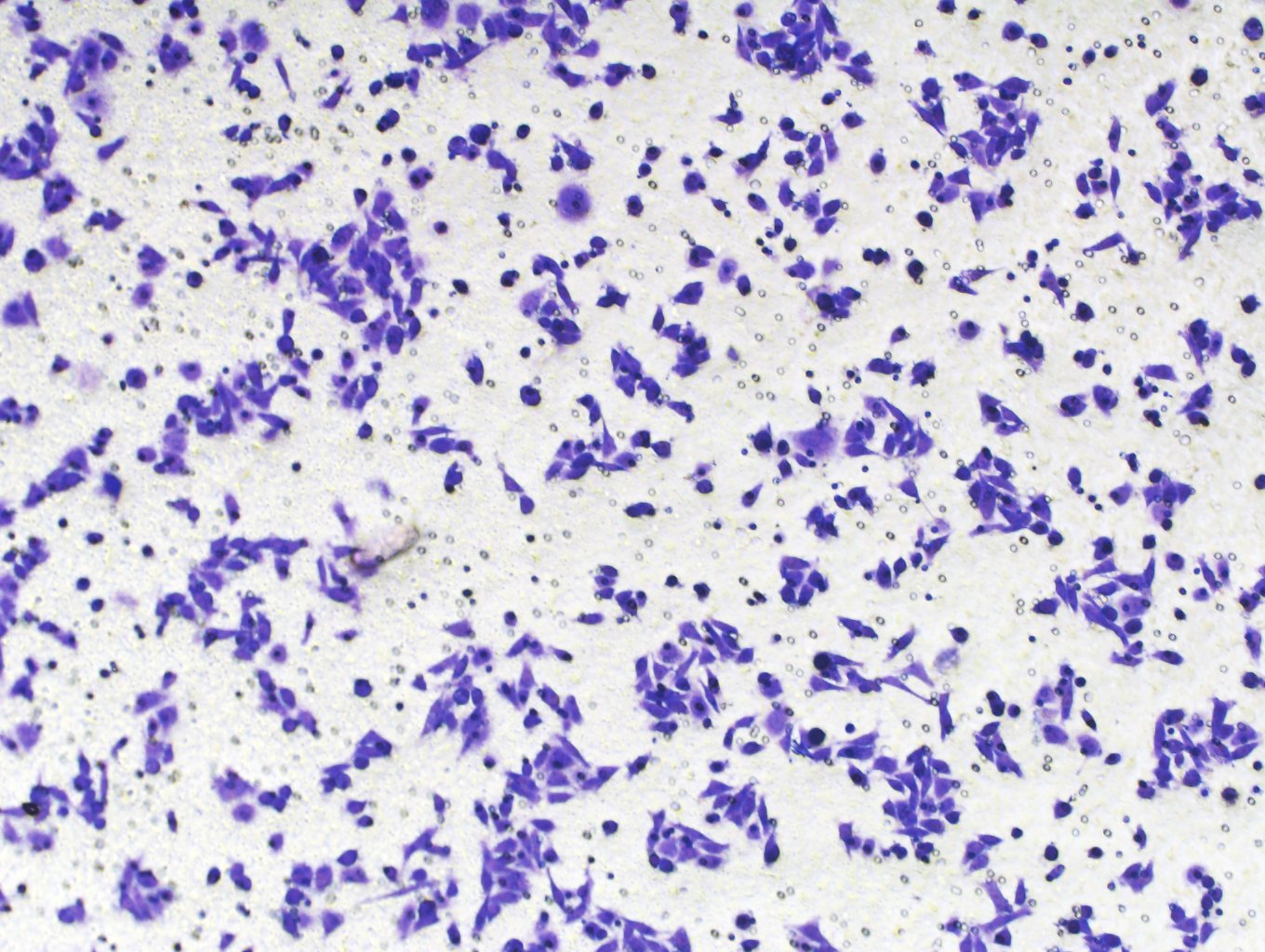

Supplement: Supplementary file 11 — EV Figure Source Data part 3 [file 44318_2025_363_MOESM11_ESM.zip › Figure EV6/EV6G/Ephrin A1+10um (2).jpg]

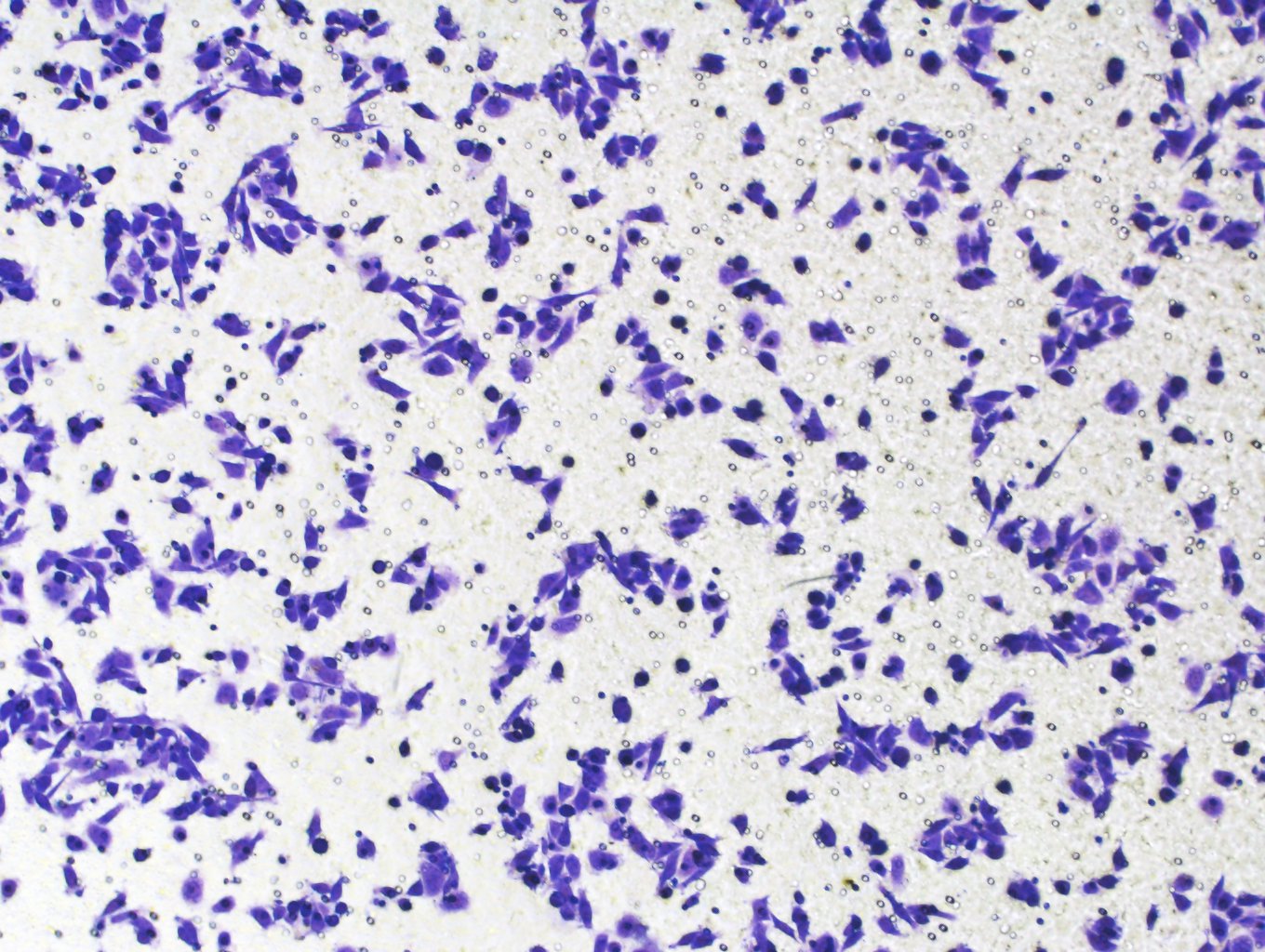

Supplement: Supplementary file 11 — EV Figure Source Data part 3 [file 44318_2025_363_MOESM11_ESM.zip › Figure EV6/EV6G/Ephrin A1+10um (3).jpg]

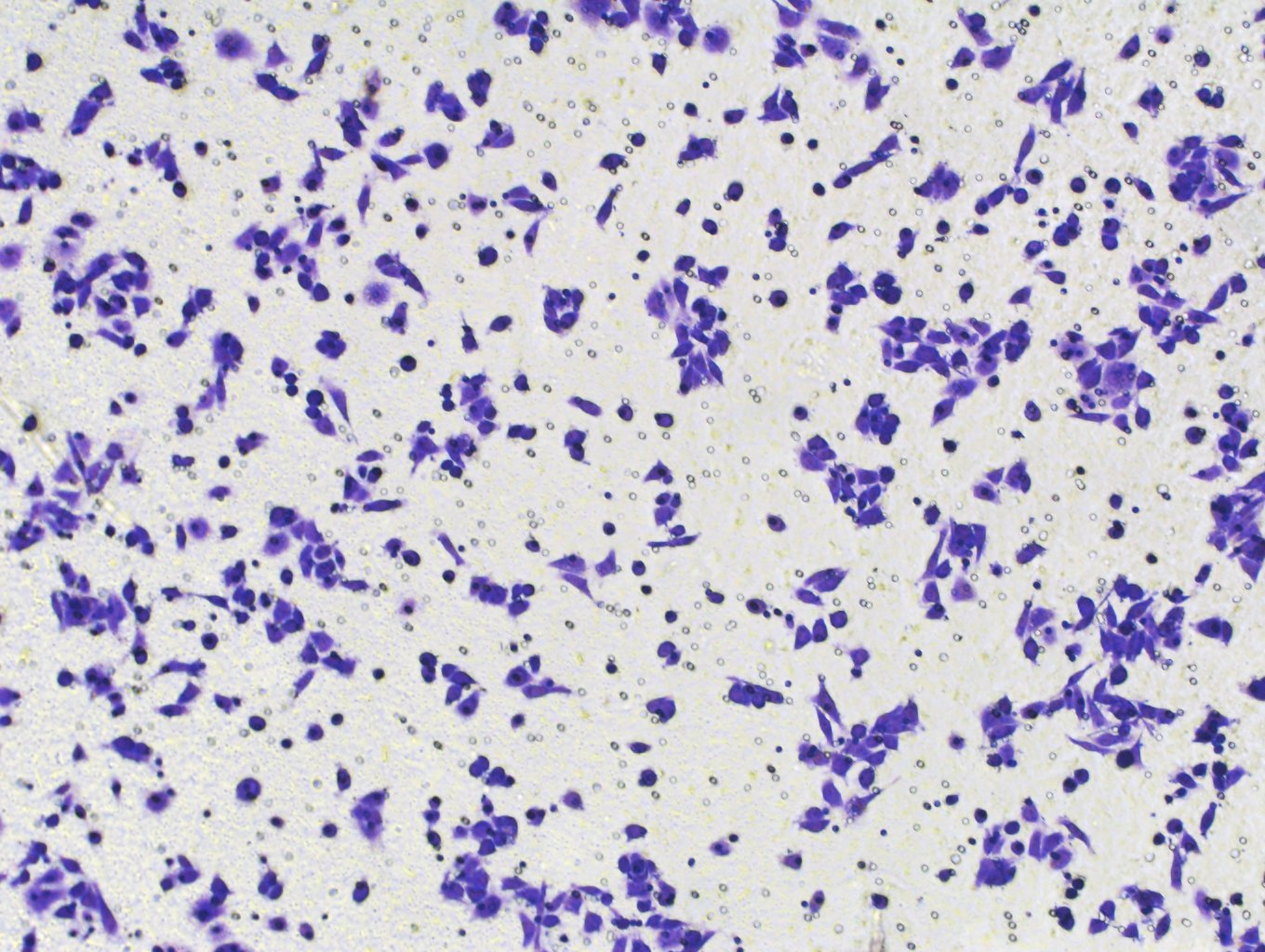

Supplement: Supplementary file 11 — EV Figure Source Data part 3 [file 44318_2025_363_MOESM11_ESM.zip › Figure EV6/EV6G/Ephrin A1+10um (4)-displayed in EV6G.jpg]

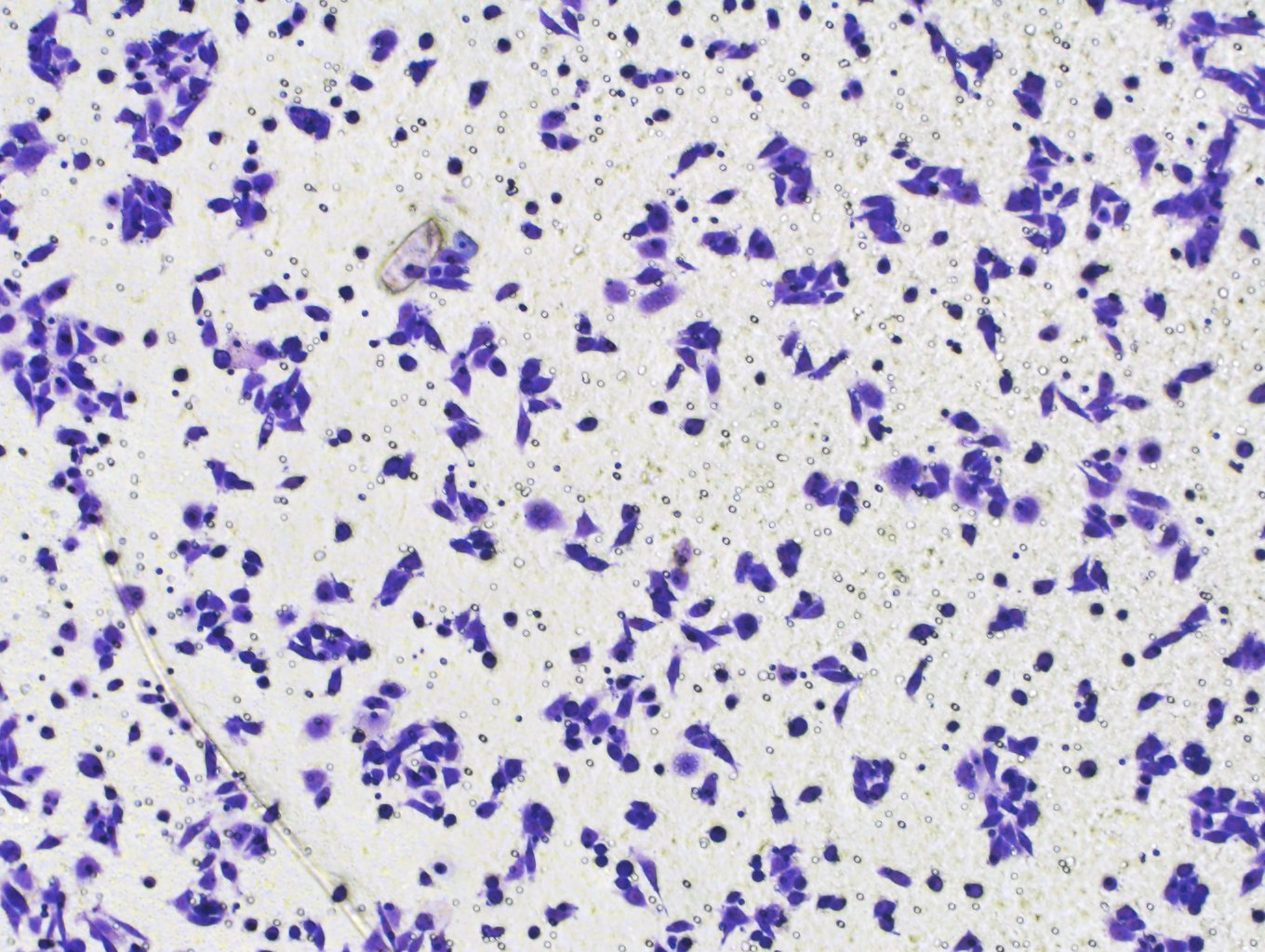

Supplement: Supplementary file 11 — EV Figure Source Data part 3 [file 44318_2025_363_MOESM11_ESM.zip › Figure EV6/EV6G/Ephrin A1+10um (5).jpg]

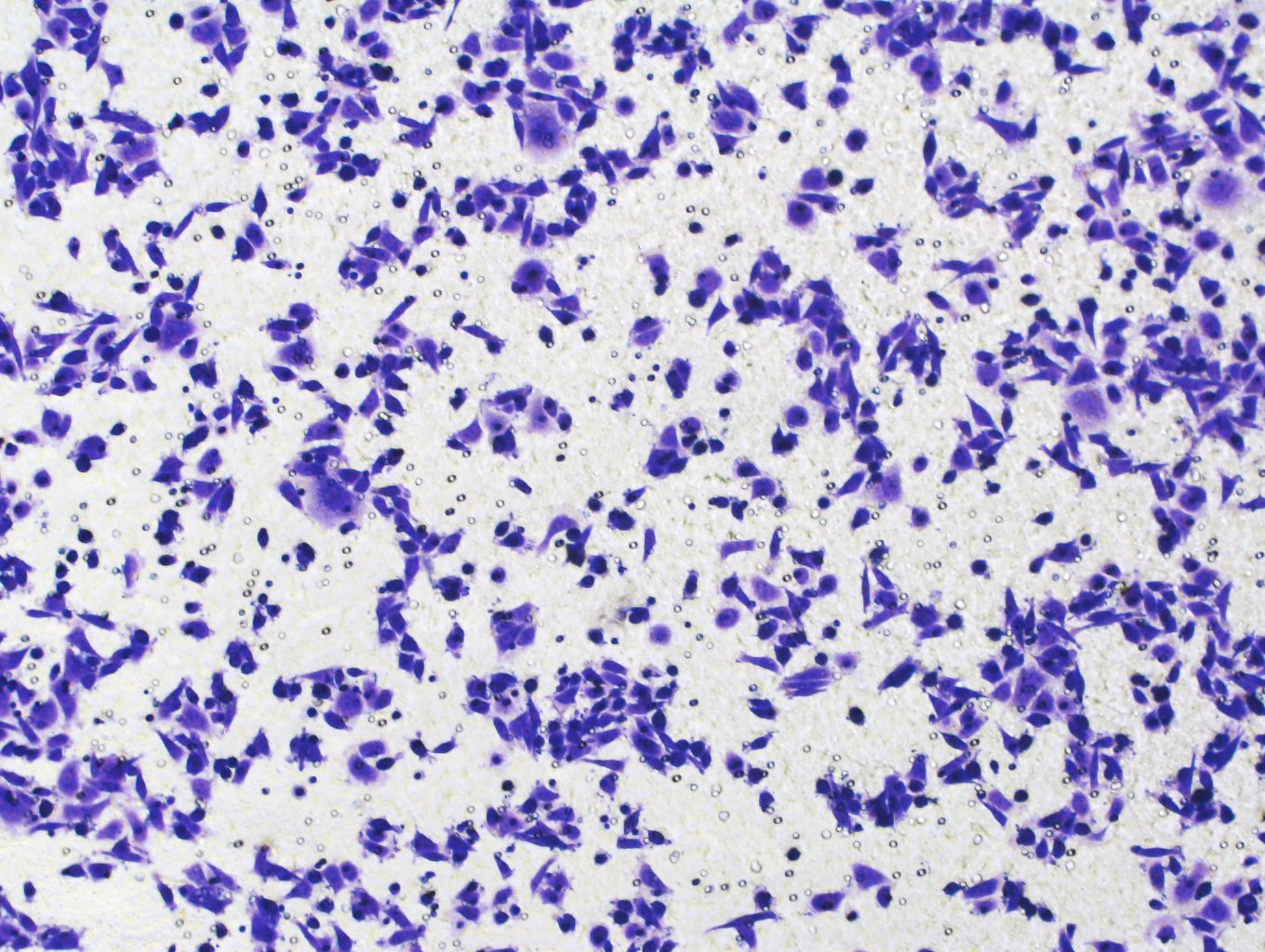

Supplement: Supplementary file 11 — EV Figure Source Data part 3 [file 44318_2025_363_MOESM11_ESM.zip › Figure EV6/EV6G/Ephrin A1+5um (1).jpg]

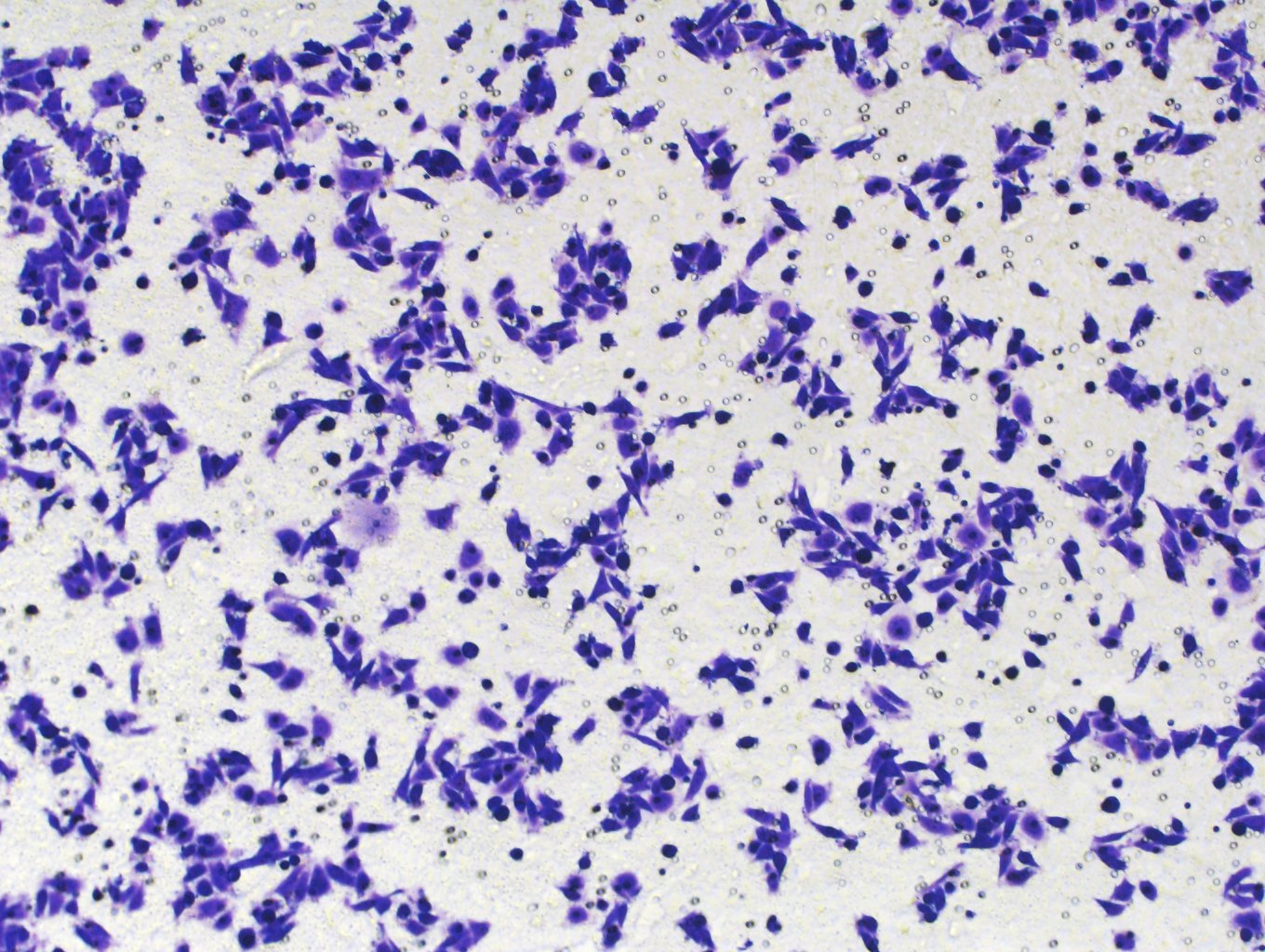

Supplement: Supplementary file 11 — EV Figure Source Data part 3 [file 44318_2025_363_MOESM11_ESM.zip › Figure EV6/EV6G/Ephrin A1+5um (2).jpg]

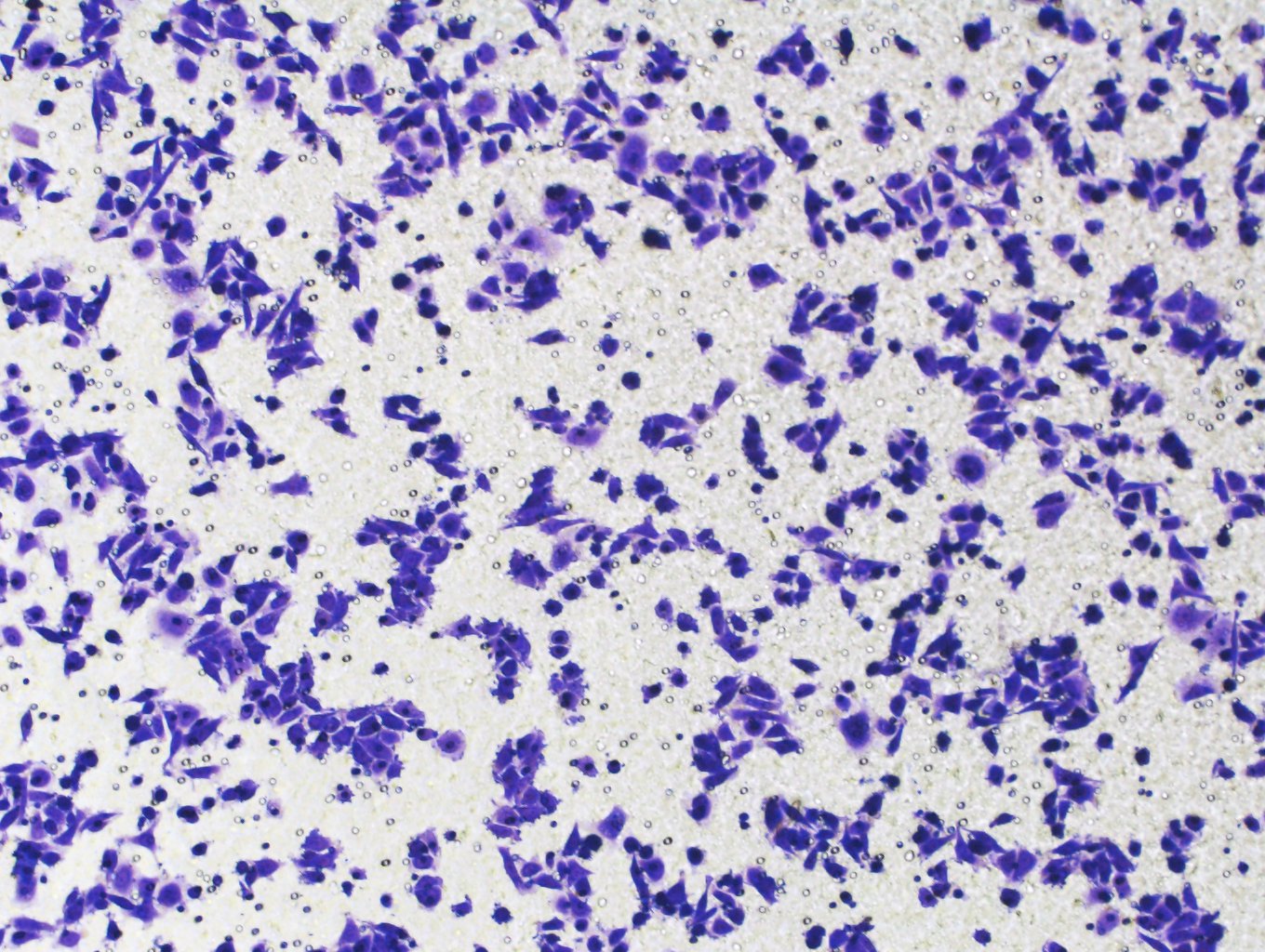

Supplement: Supplementary file 11 — EV Figure Source Data part 3 [file 44318_2025_363_MOESM11_ESM.zip › Figure EV6/EV6G/Ephrin A1+5um (3).jpg]

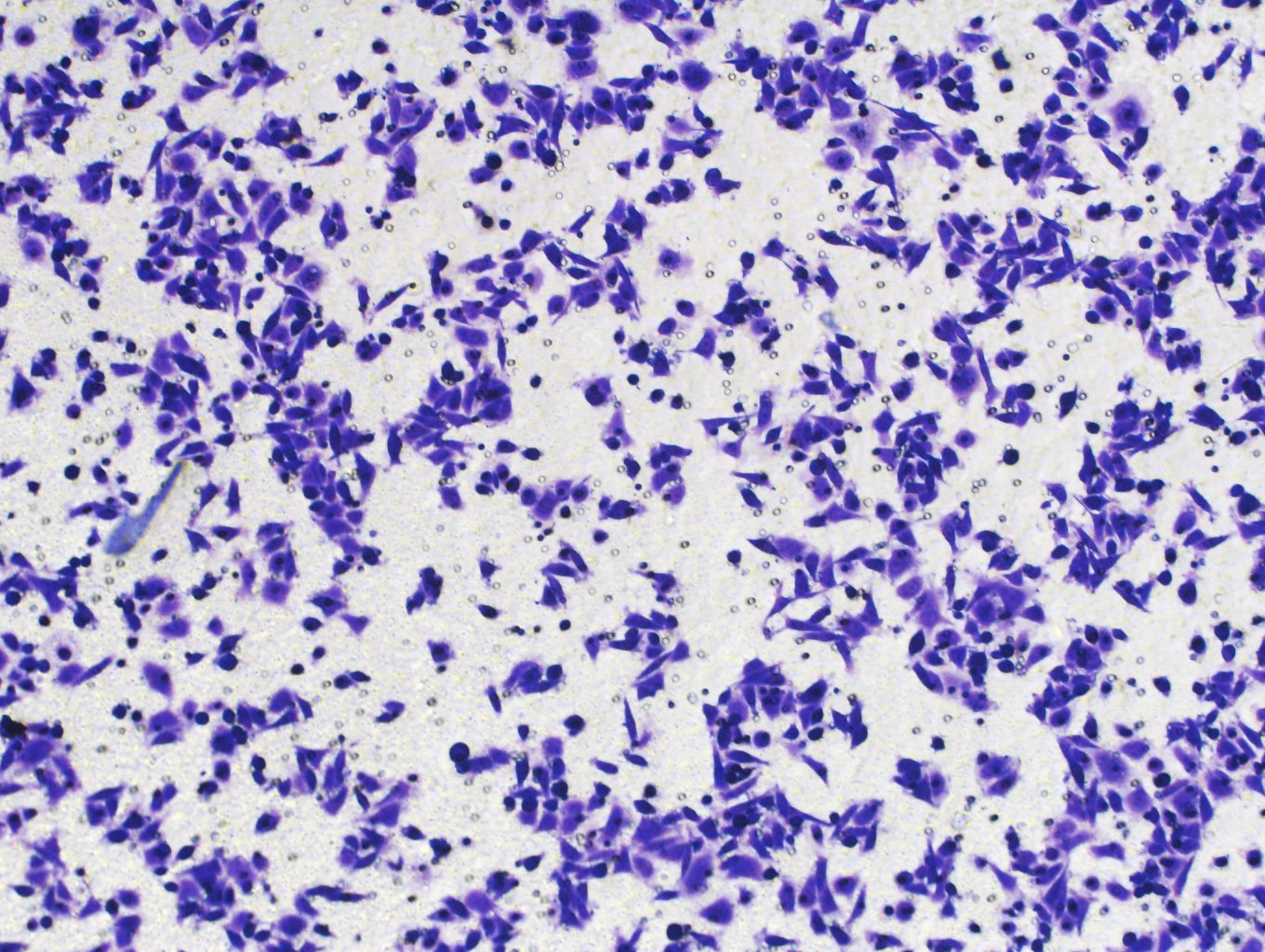

Supplement: Supplementary file 11 — EV Figure Source Data part 3 [file 44318_2025_363_MOESM11_ESM.zip › Figure EV6/EV6G/Ephrin A1+5um (4).jpg]

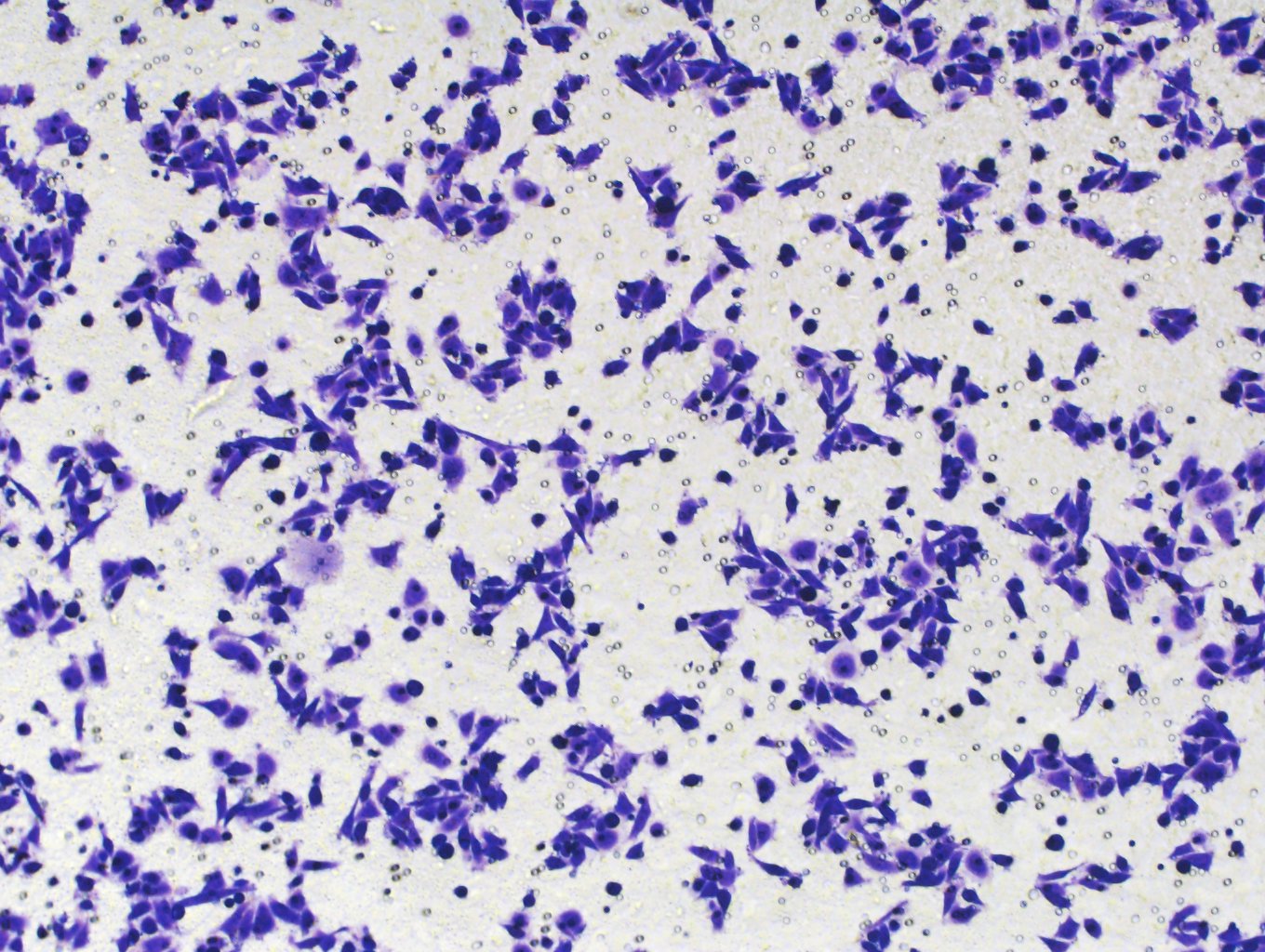

Supplement: Supplementary file 11 — EV Figure Source Data part 3 [file 44318_2025_363_MOESM11_ESM.zip › Figure EV6/EV6G/Ephrin A1+5um (5)-displayed in EV6G.jpg]

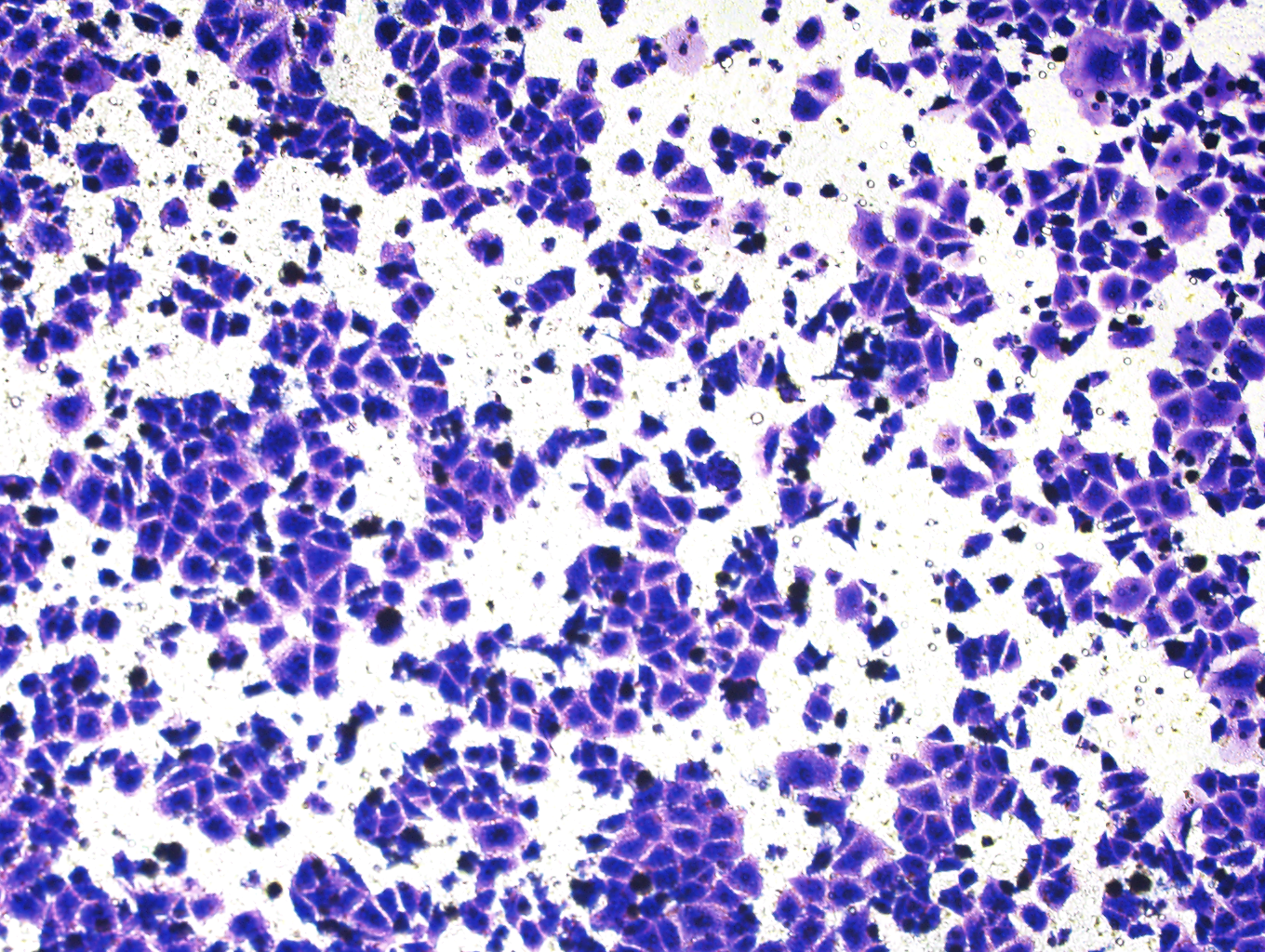

Supplement: Supplementary file 11 — EV Figure Source Data part 3 [file 44318_2025_363_MOESM11_ESM.zip › Figure EV6/EV6I/Control (1).tif]

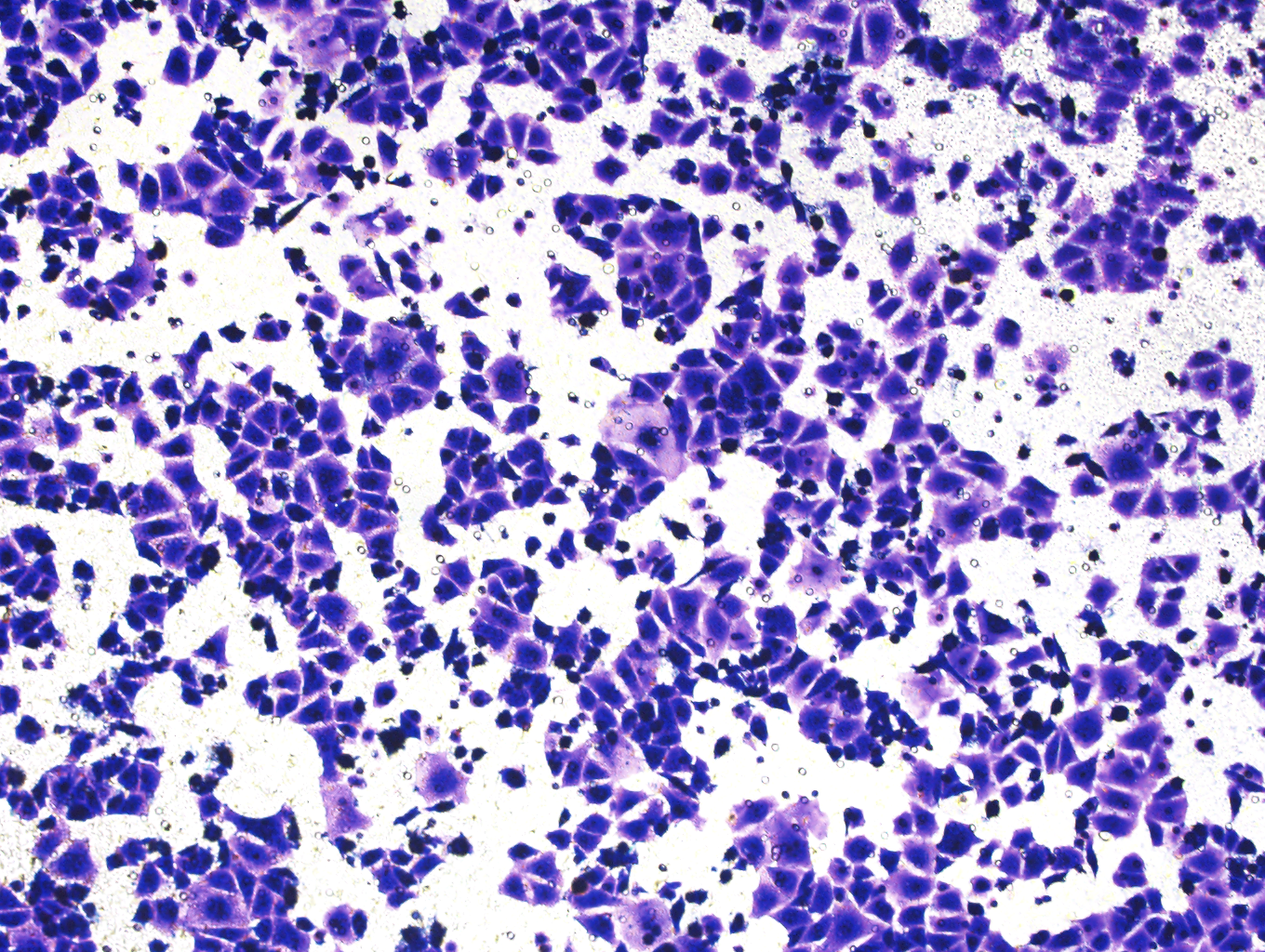

Supplement: Supplementary file 11 — EV Figure Source Data part 3 [file 44318_2025_363_MOESM11_ESM.zip › Figure EV6/EV6I/Control (2)-displayed in EV6I.tif]

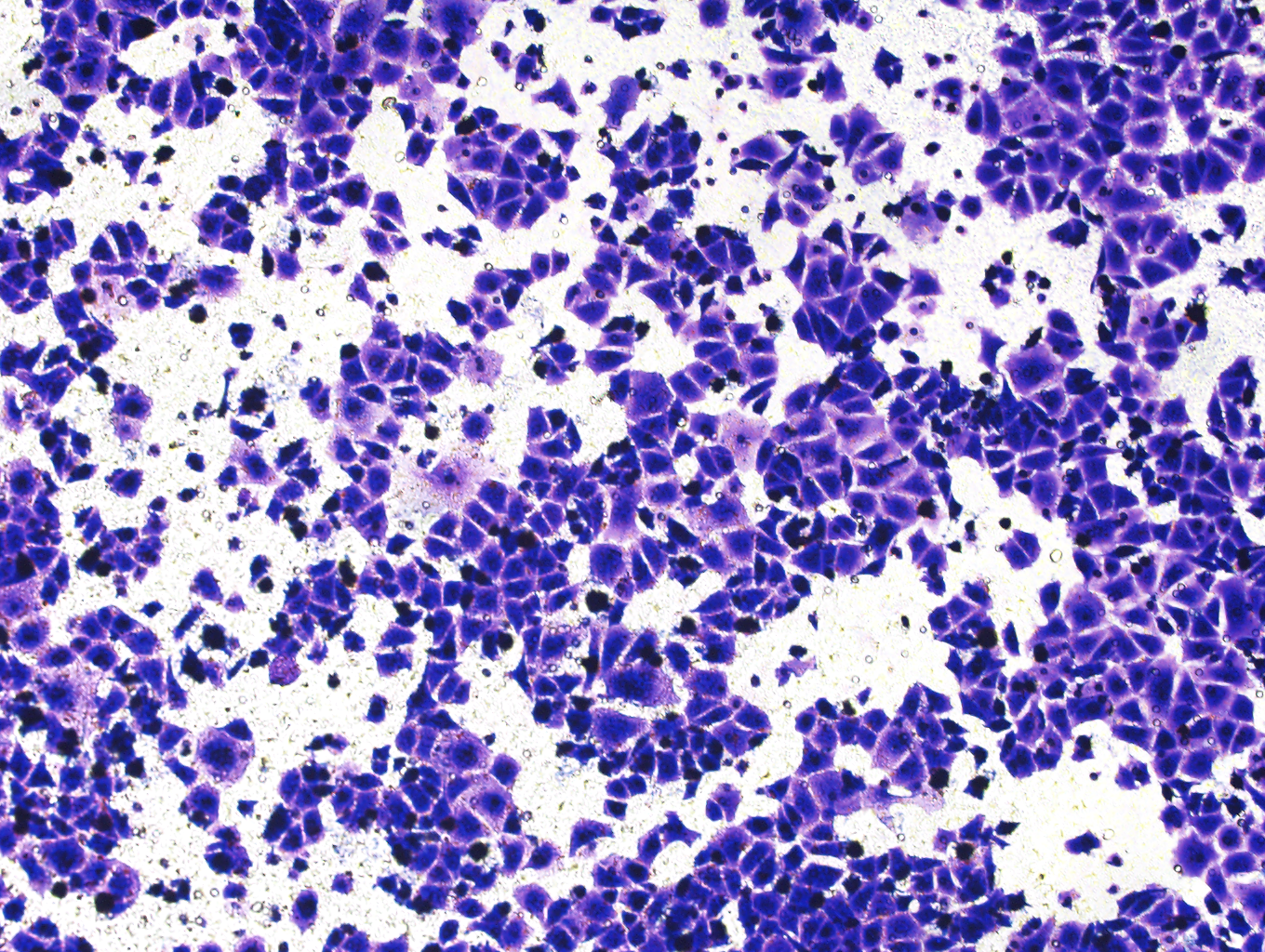

Supplement: Supplementary file 11 — EV Figure Source Data part 3 [file 44318_2025_363_MOESM11_ESM.zip › Figure EV6/EV6I/Control (3).tif]

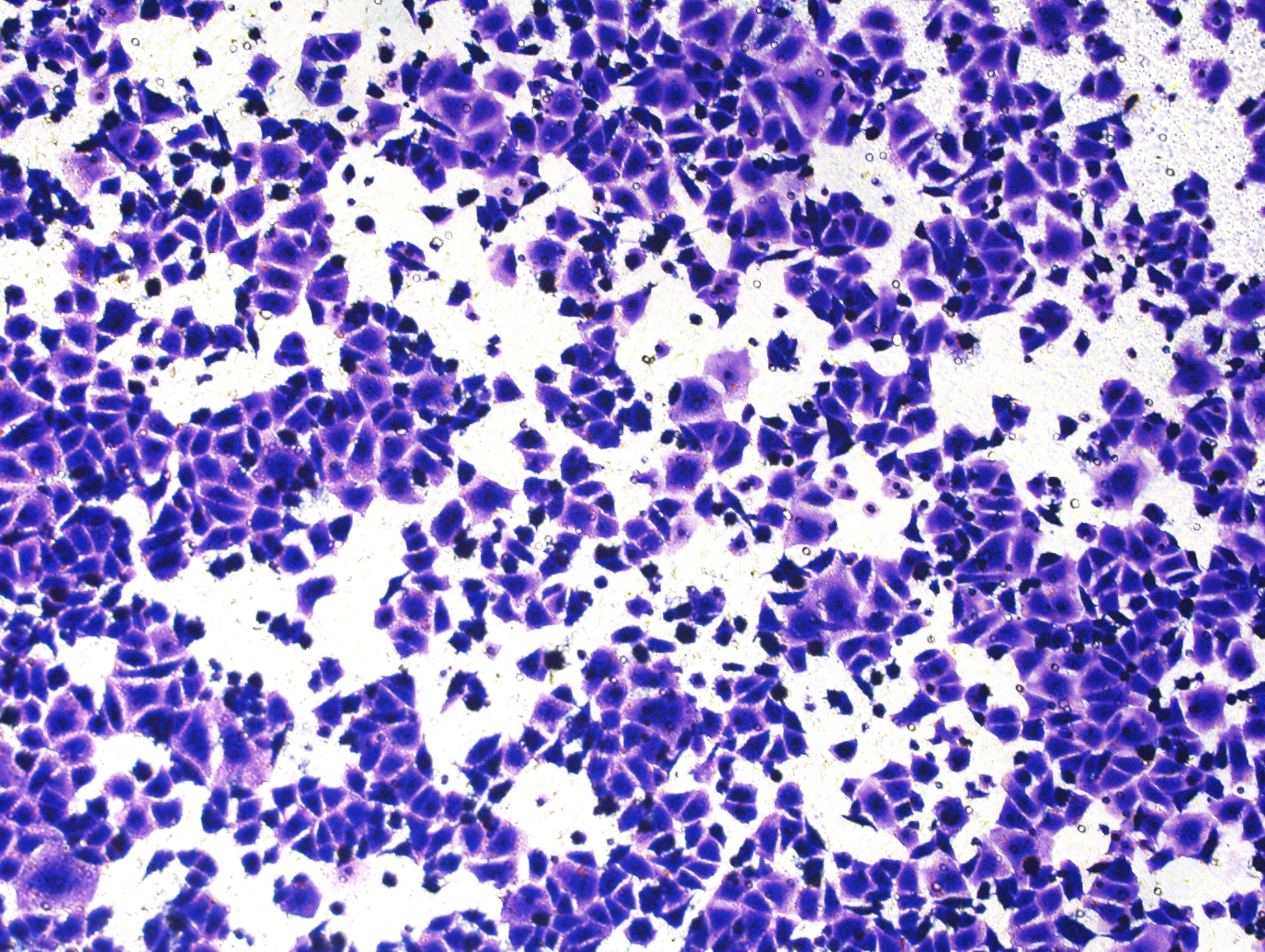

Supplement: Supplementary file 11 — EV Figure Source Data part 3 [file 44318_2025_363_MOESM11_ESM.zip › Figure EV6/EV6I/Control (4).tif]

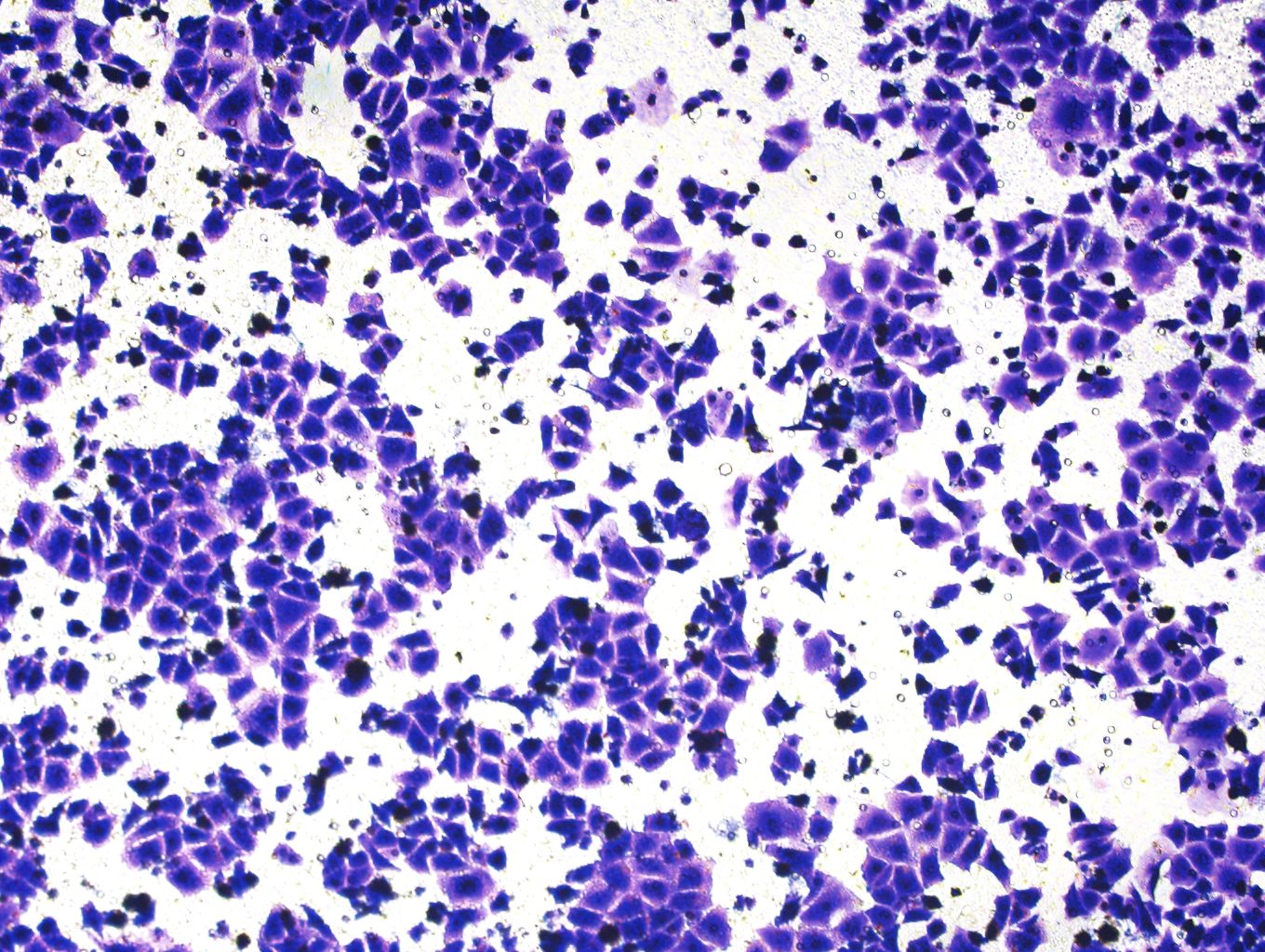

Supplement: Supplementary file 11 — EV Figure Source Data part 3 [file 44318_2025_363_MOESM11_ESM.zip › Figure EV6/EV6I/Control (5).tif]

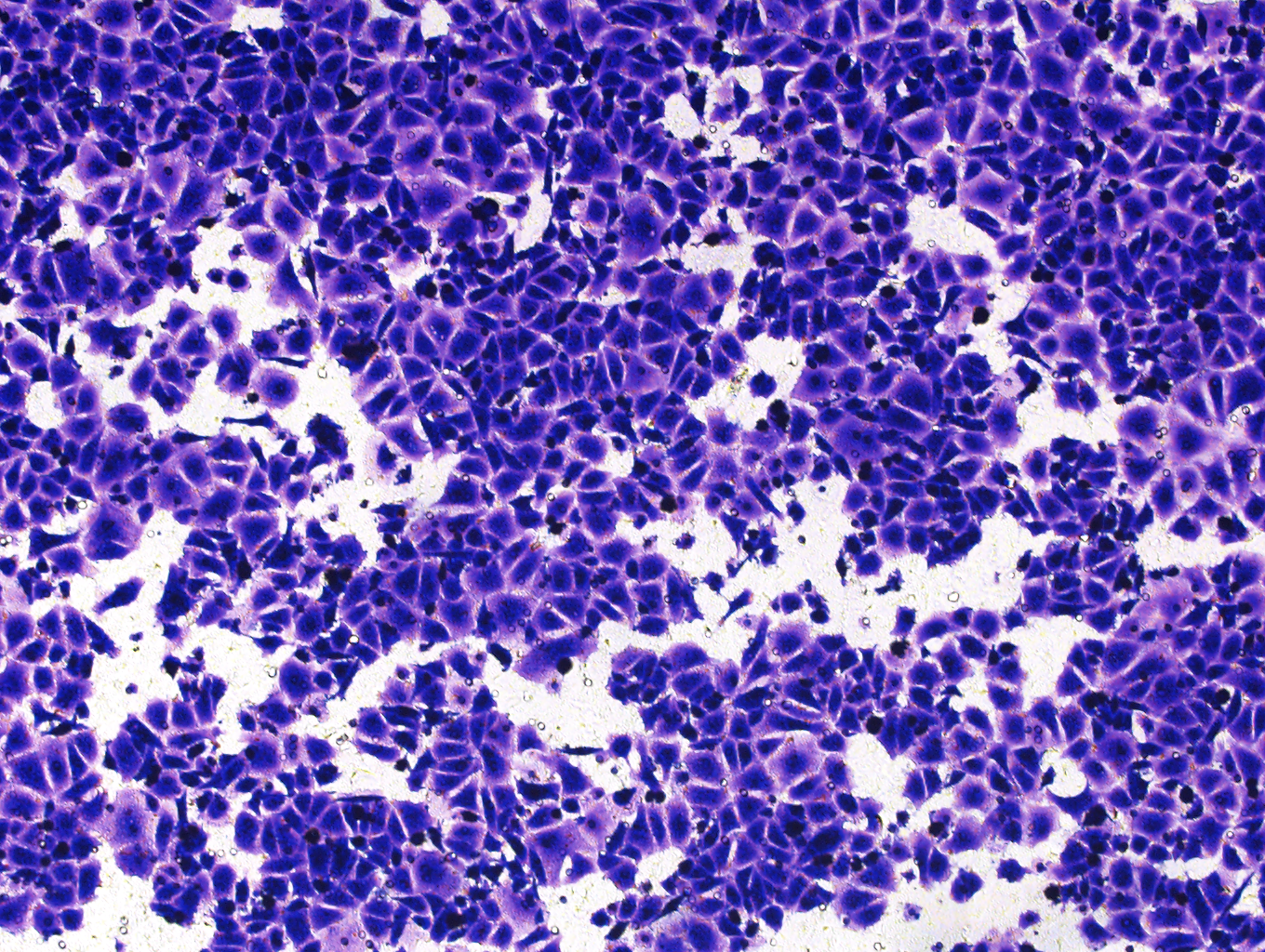

Supplement: Supplementary file 11 — EV Figure Source Data part 3 [file 44318_2025_363_MOESM11_ESM.zip › Figure EV6/EV6I/Ephrin A1 (1).tif]

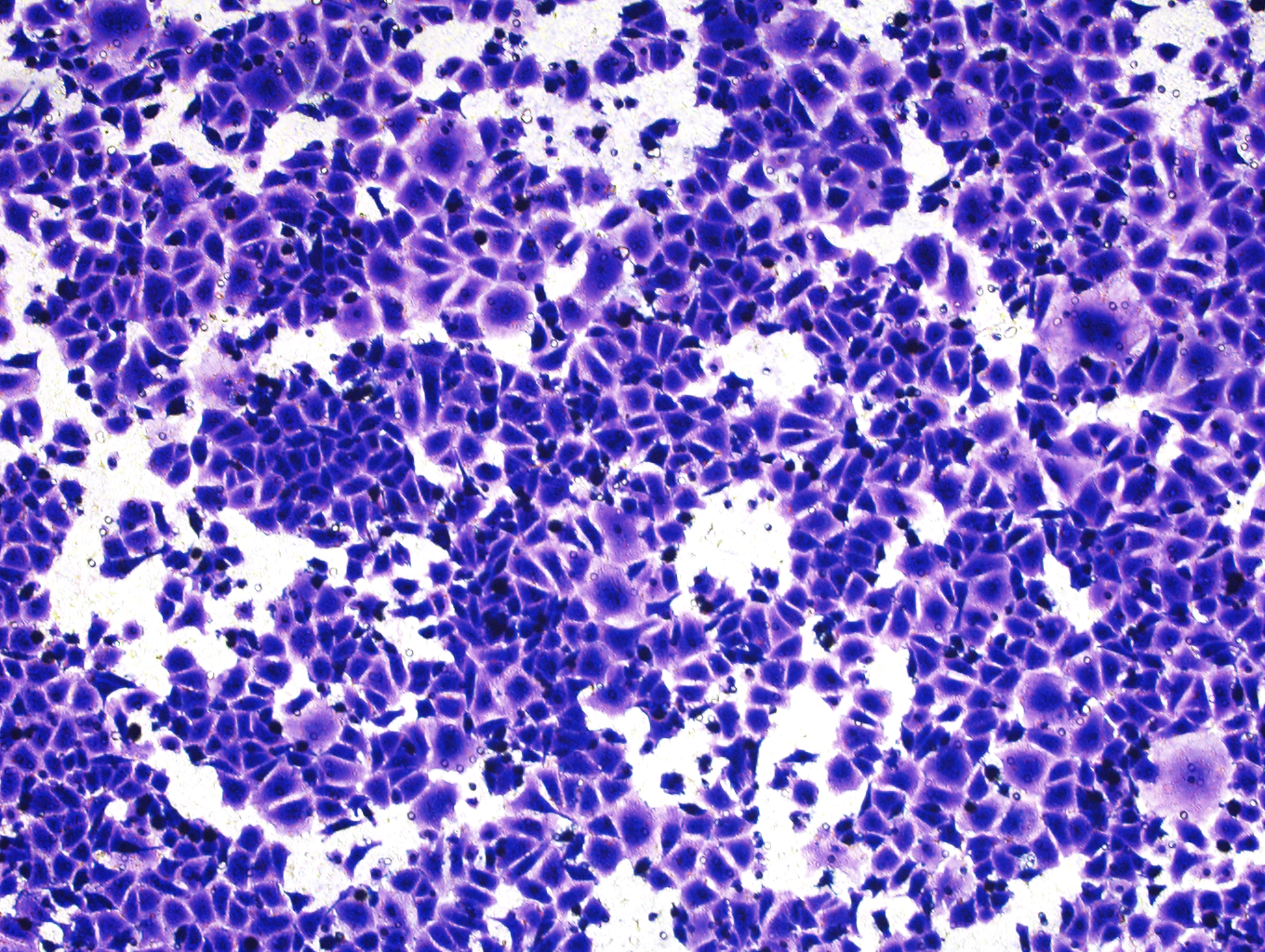

Supplement: Supplementary file 11 — EV Figure Source Data part 3 [file 44318_2025_363_MOESM11_ESM.zip › Figure EV6/EV6I/Ephrin A1 (2)-displayed in EV6I.tif]

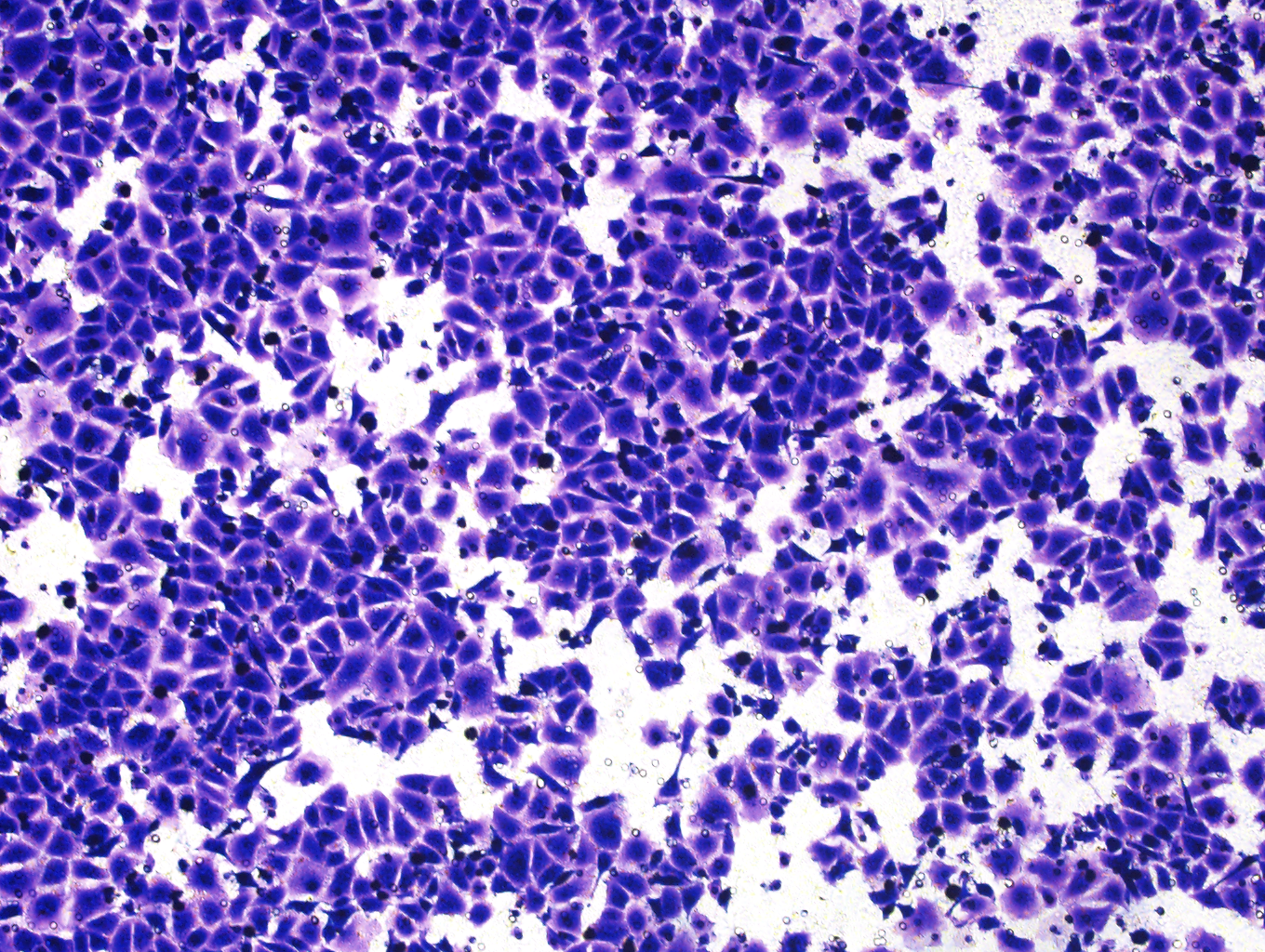

Supplement: Supplementary file 11 — EV Figure Source Data part 3 [file 44318_2025_363_MOESM11_ESM.zip › Figure EV6/EV6I/Ephrin A1 (3).tif]

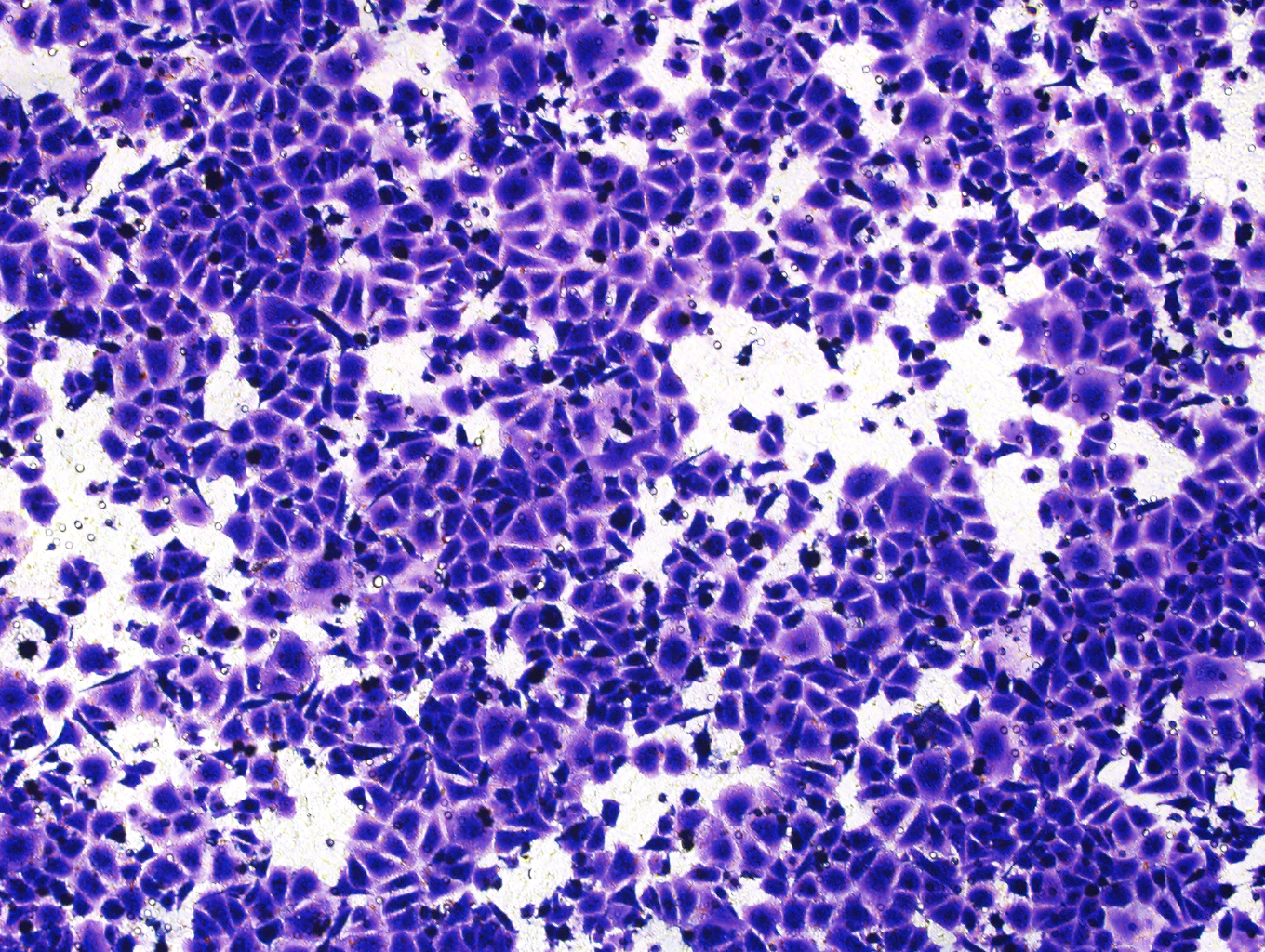

Supplement: Supplementary file 11 — EV Figure Source Data part 3 [file 44318_2025_363_MOESM11_ESM.zip › Figure EV6/EV6I/Ephrin A1 (4).tif]

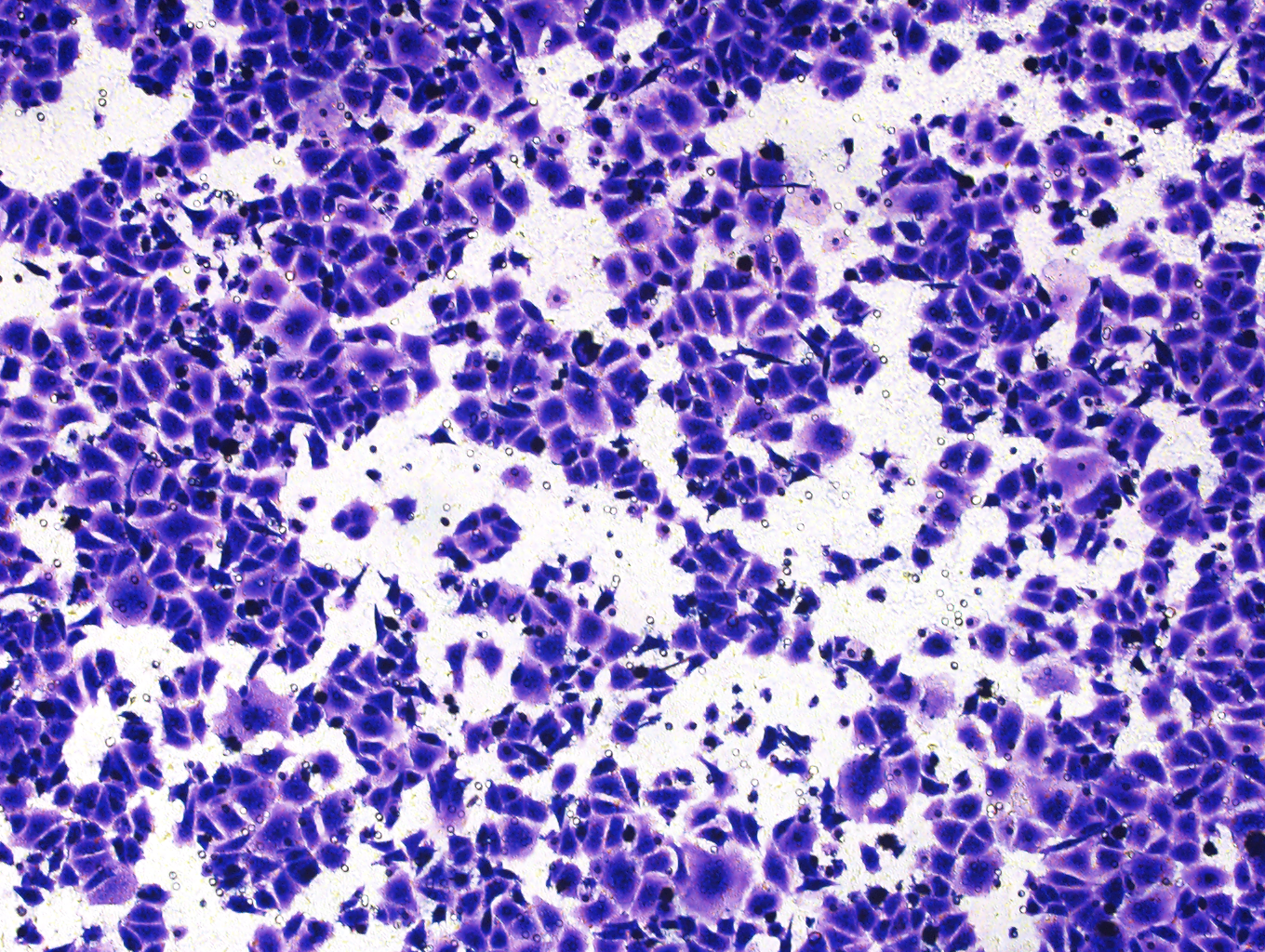

Supplement: Supplementary file 11 — EV Figure Source Data part 3 [file 44318_2025_363_MOESM11_ESM.zip › Figure EV6/EV6I/Ephrin A1 (5).tif]

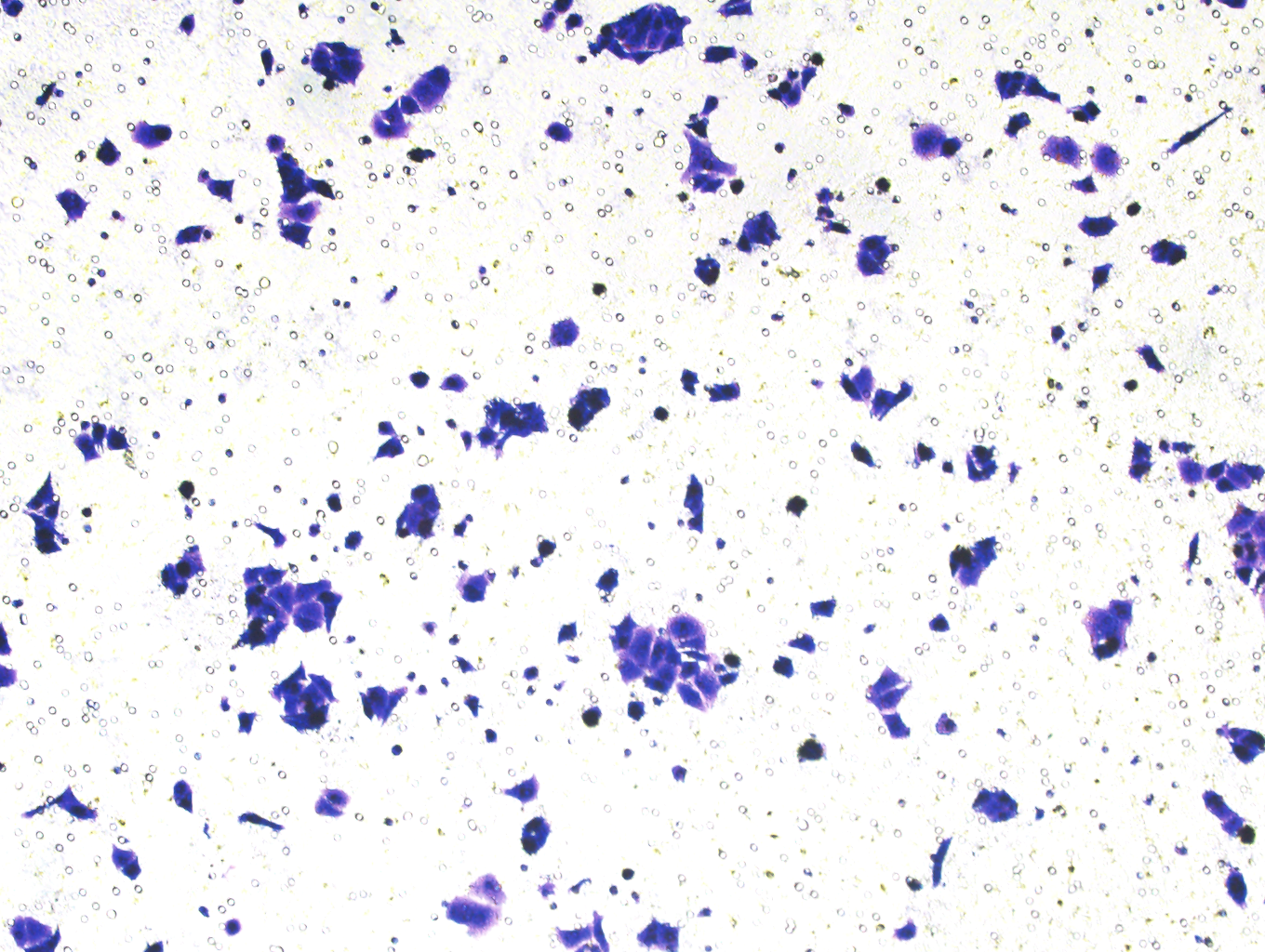

Supplement: Supplementary file 11 — EV Figure Source Data part 3 [file 44318_2025_363_MOESM11_ESM.zip › Figure EV6/EV6I/Ephrin A1+10 (1)-displayed in EV6I.tif]

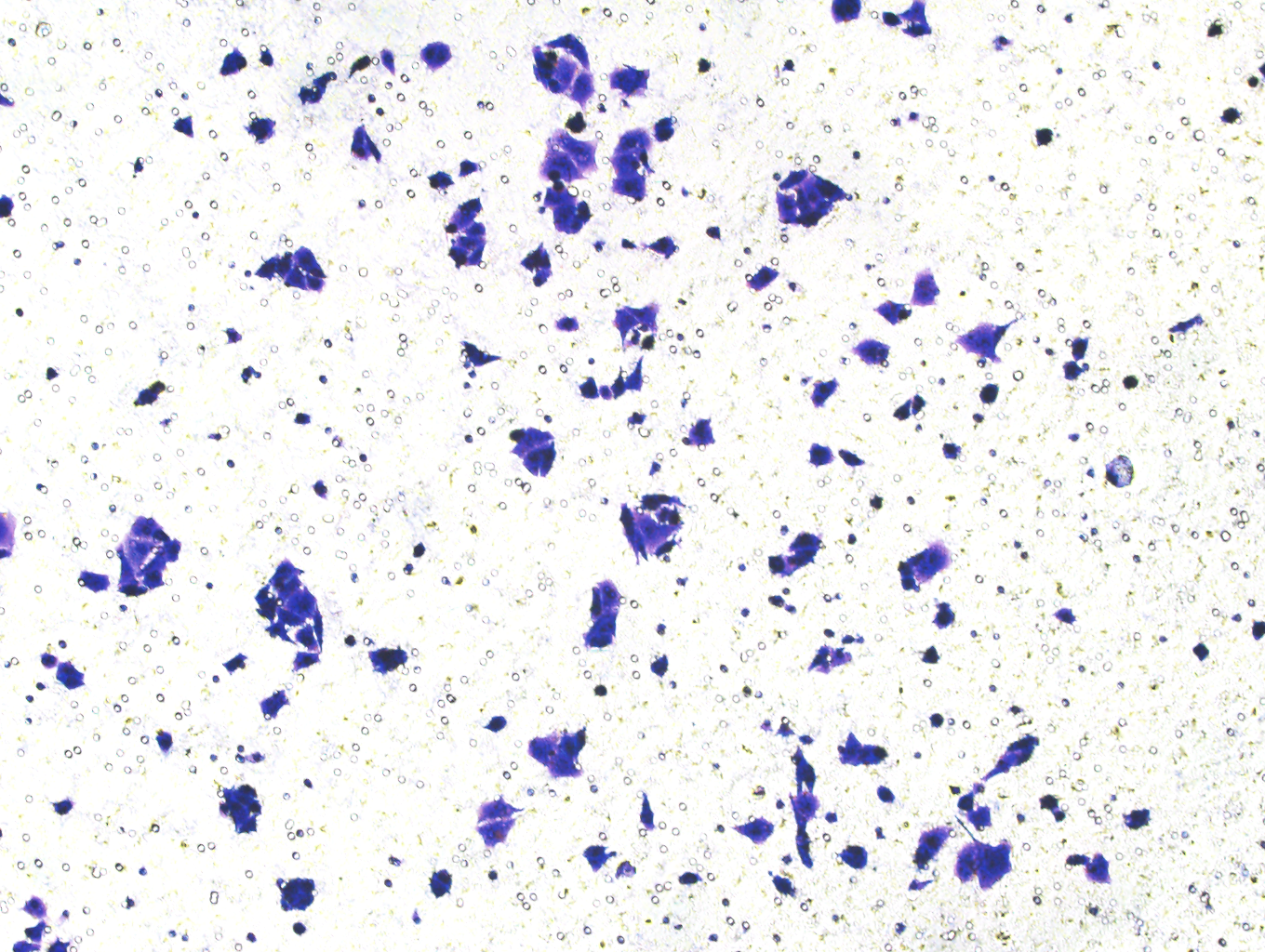

Supplement: Supplementary file 11 — EV Figure Source Data part 3 [file 44318_2025_363_MOESM11_ESM.zip › Figure EV6/EV6I/Ephrin A1+10 (2).tif]

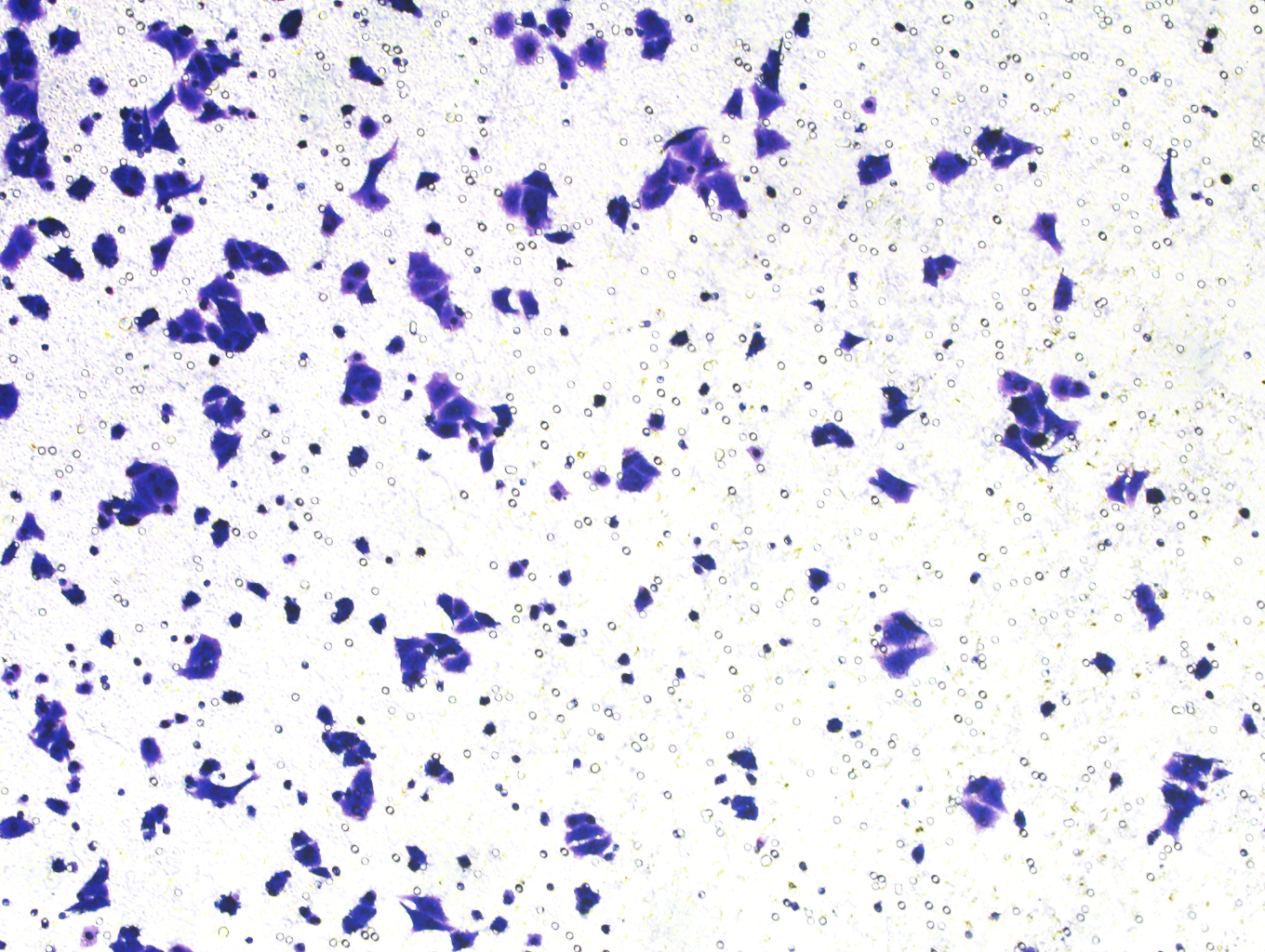

Supplement: Supplementary file 11 — EV Figure Source Data part 3 [file 44318_2025_363_MOESM11_ESM.zip › Figure EV6/EV6I/Ephrin A1+10 (3).tif]

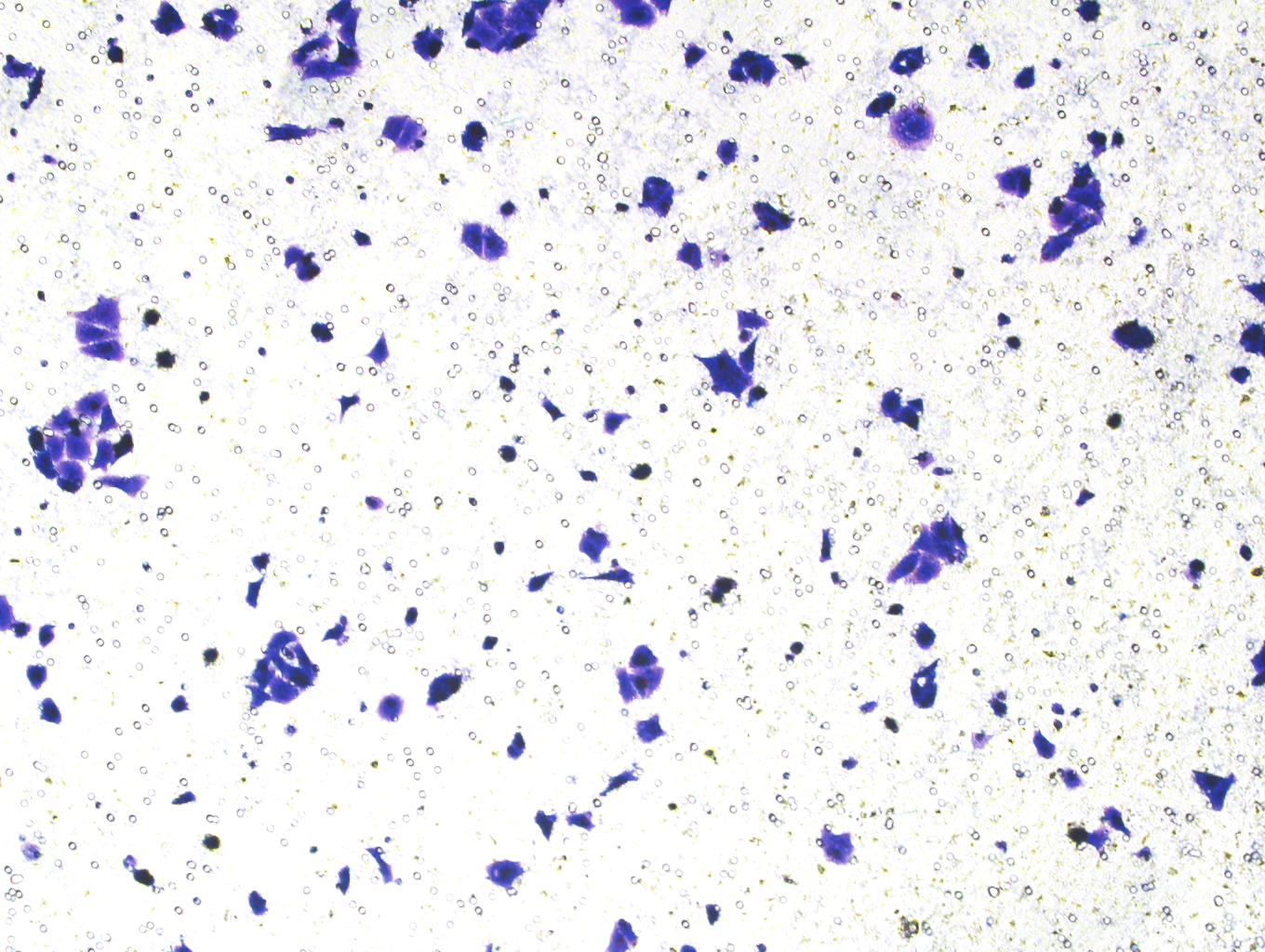

Supplement: Supplementary file 11 — EV Figure Source Data part 3 [file 44318_2025_363_MOESM11_ESM.zip › Figure EV6/EV6I/Ephrin A1+10 (4).tif]

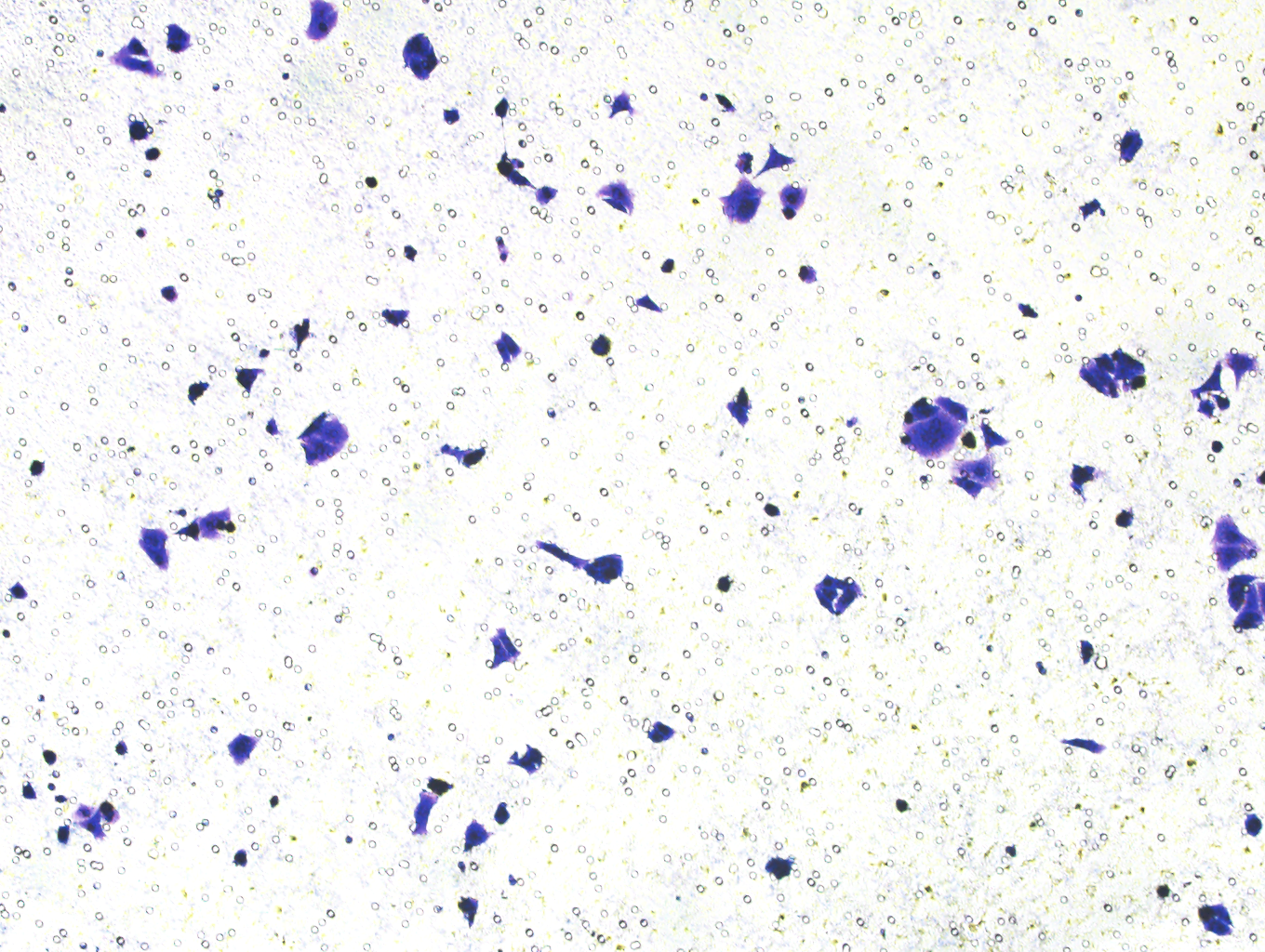

Supplement: Supplementary file 11 — EV Figure Source Data part 3 [file 44318_2025_363_MOESM11_ESM.zip › Figure EV6/EV6I/Ephrin A1+10 (5).tif]

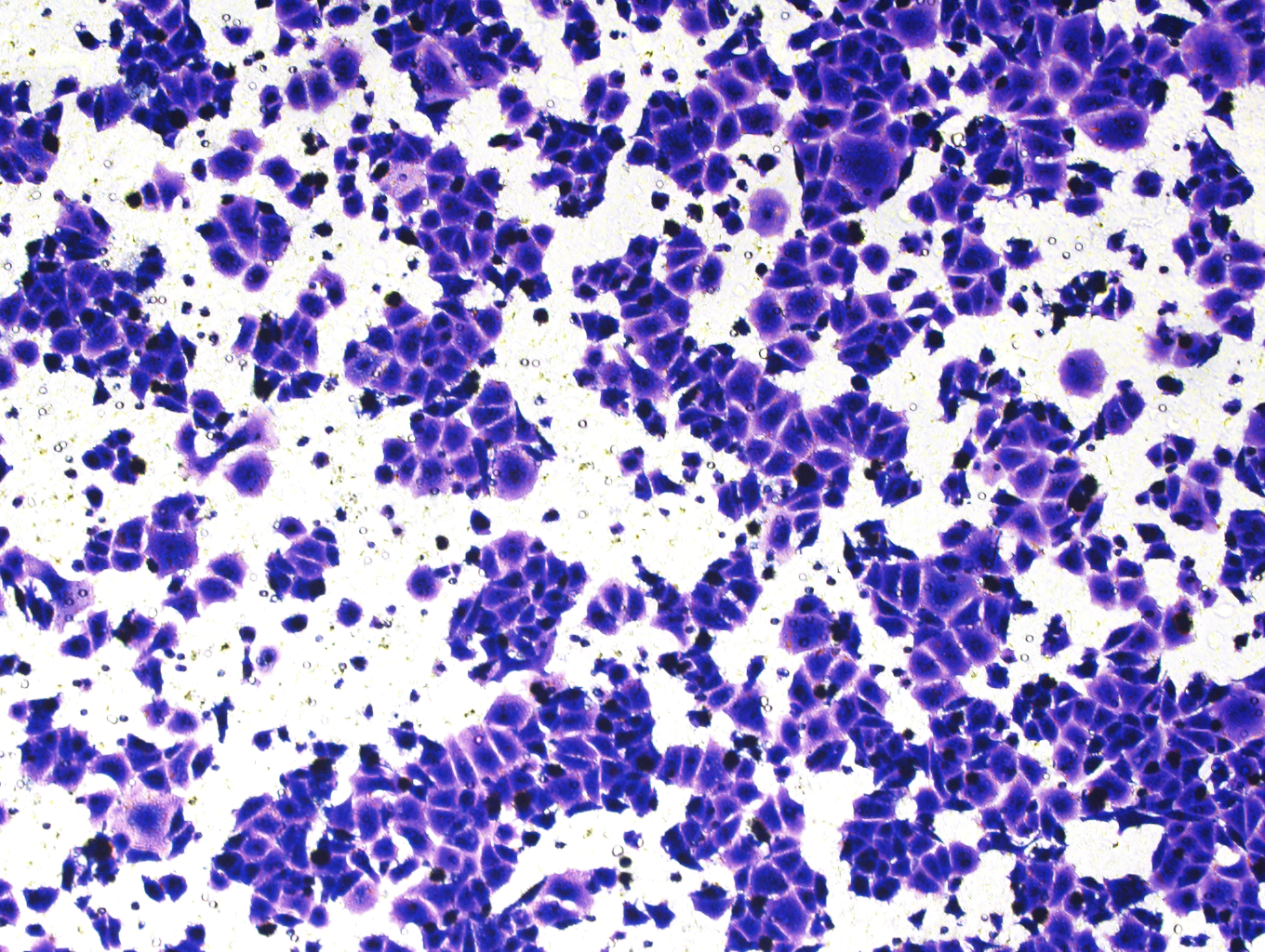

Supplement: Supplementary file 11 — EV Figure Source Data part 3 [file 44318_2025_363_MOESM11_ESM.zip › Figure EV6/EV6I/Ephrin A1+5 (1).tif]

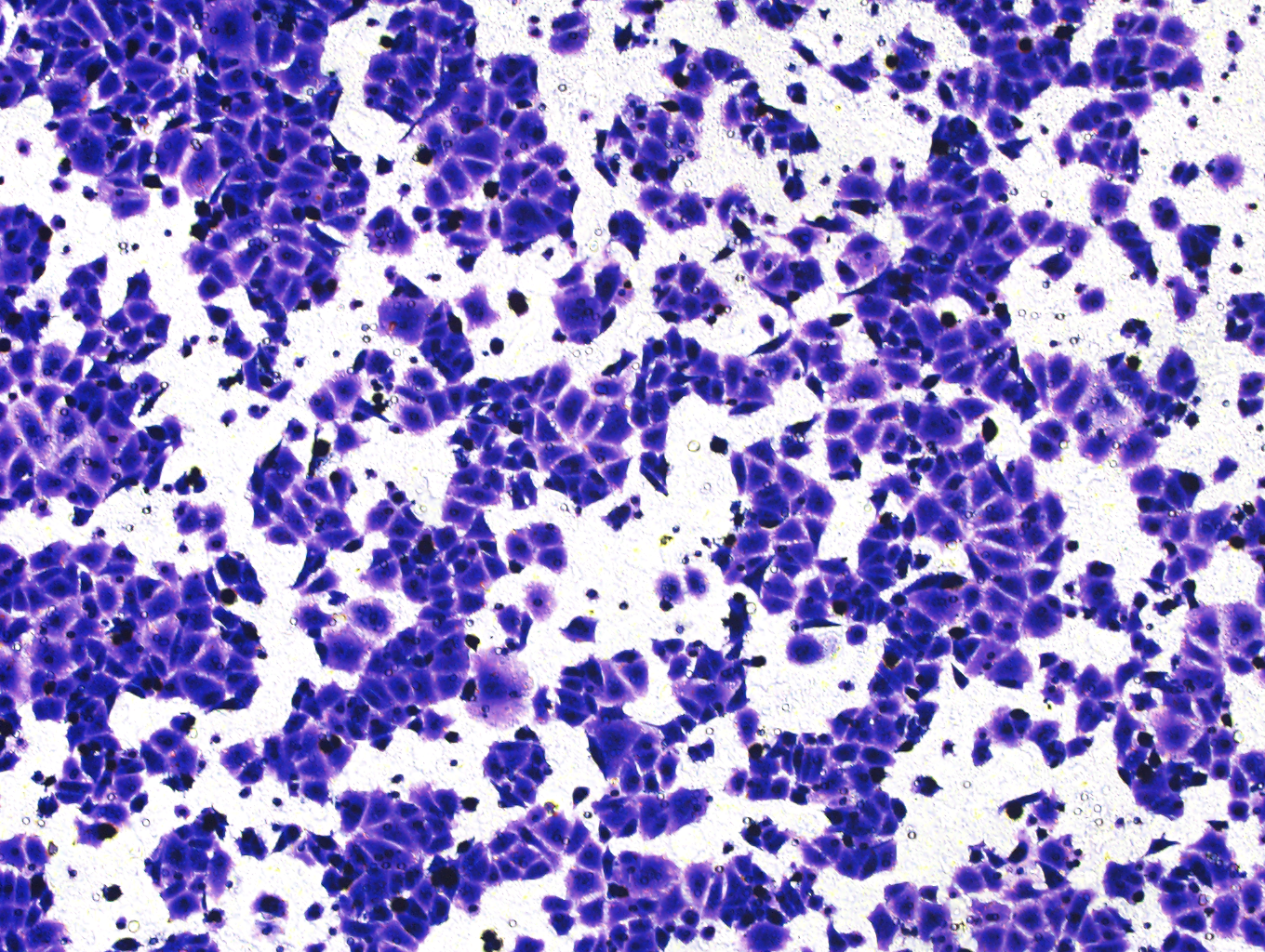

Supplement: Supplementary file 11 — EV Figure Source Data part 3 [file 44318_2025_363_MOESM11_ESM.zip › Figure EV6/EV6I/Ephrin A1+5 (2)-displayed in EV6I.tif]

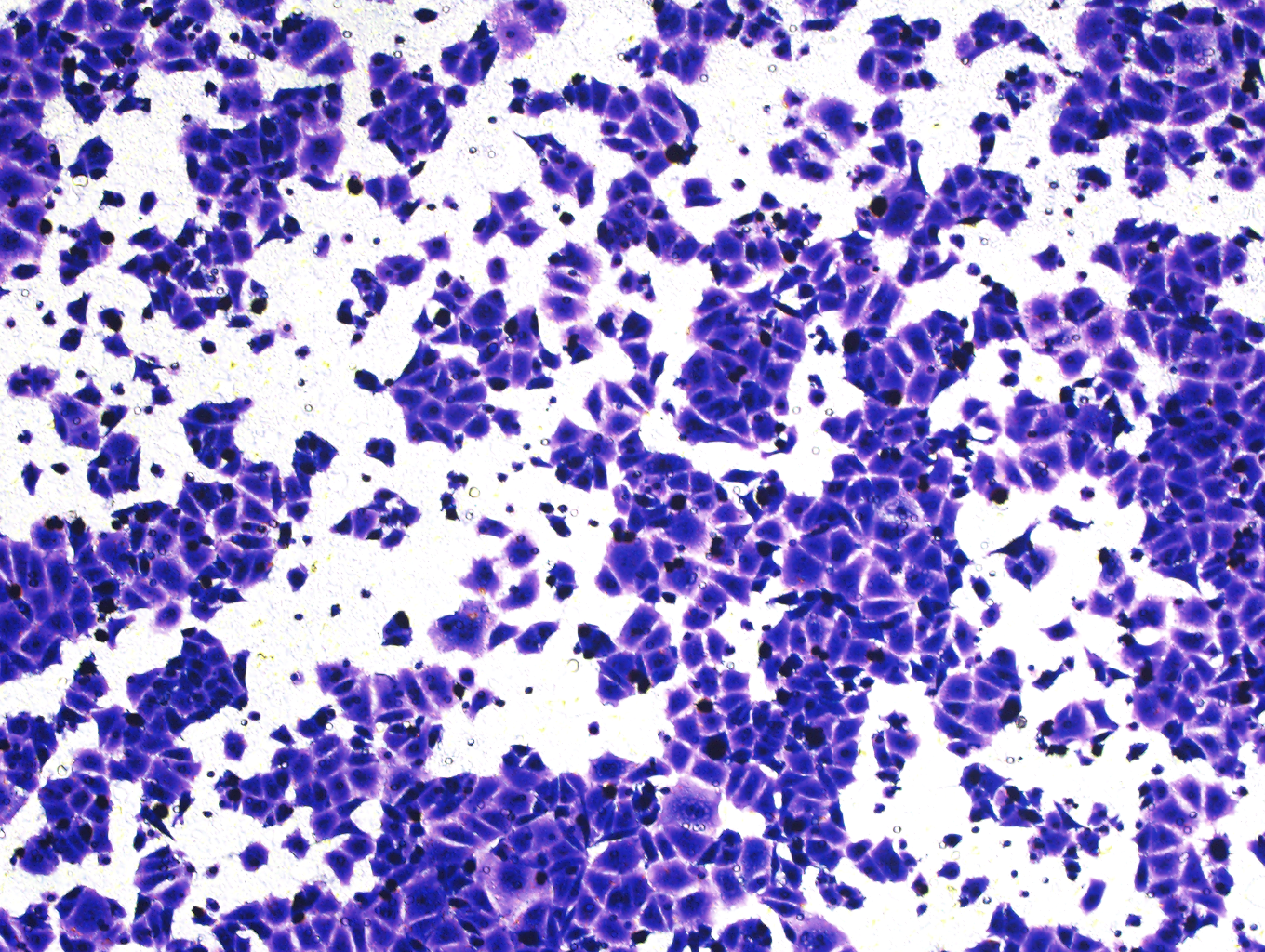

Supplement: Supplementary file 11 — EV Figure Source Data part 3 [file 44318_2025_363_MOESM11_ESM.zip › Figure EV6/EV6I/Ephrin A1+5 (3).tif]

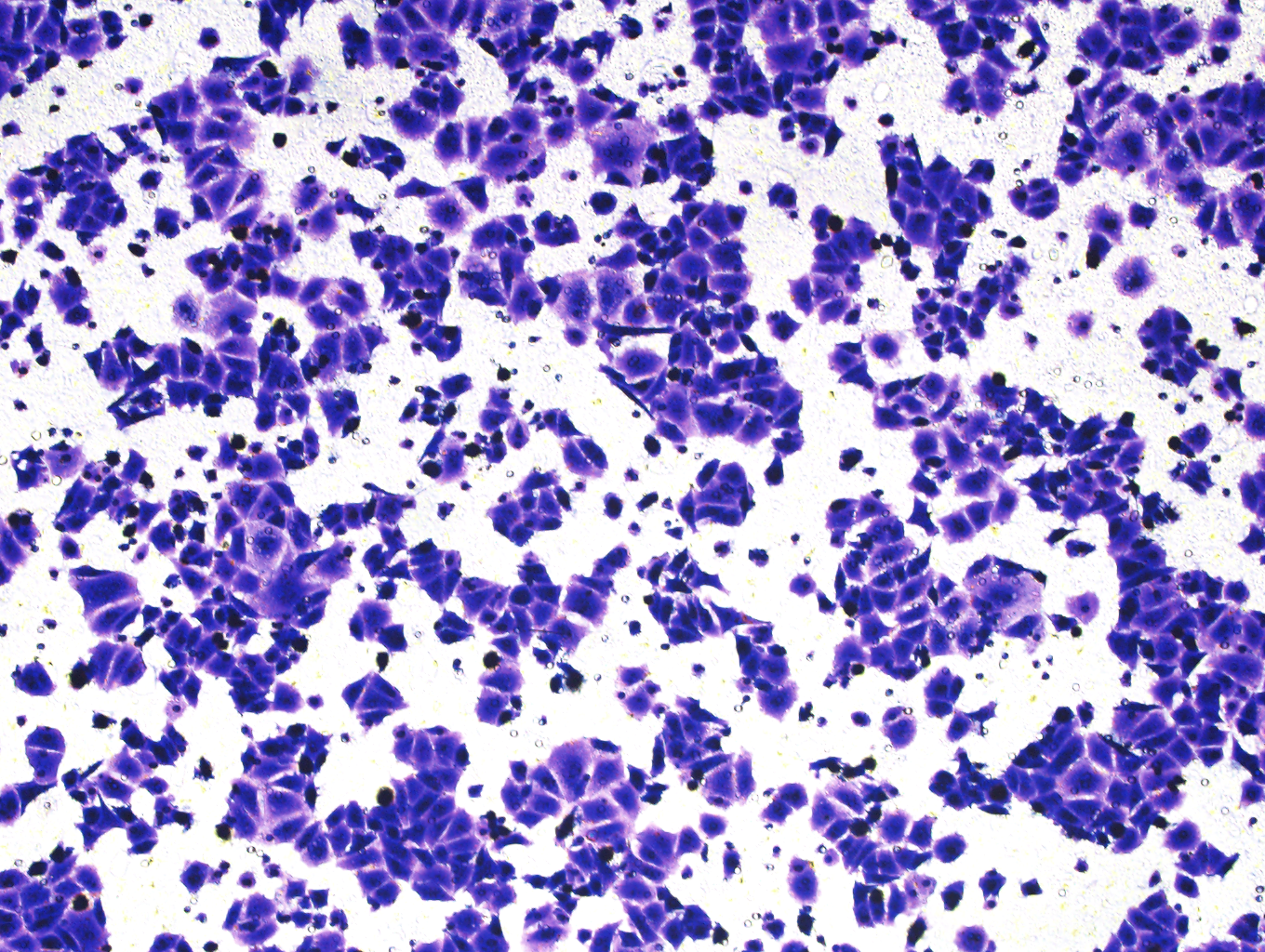

Supplement: Supplementary file 11 — EV Figure Source Data part 3 [file 44318_2025_363_MOESM11_ESM.zip › Figure EV6/EV6I/Ephrin A1+5 (4).tif]

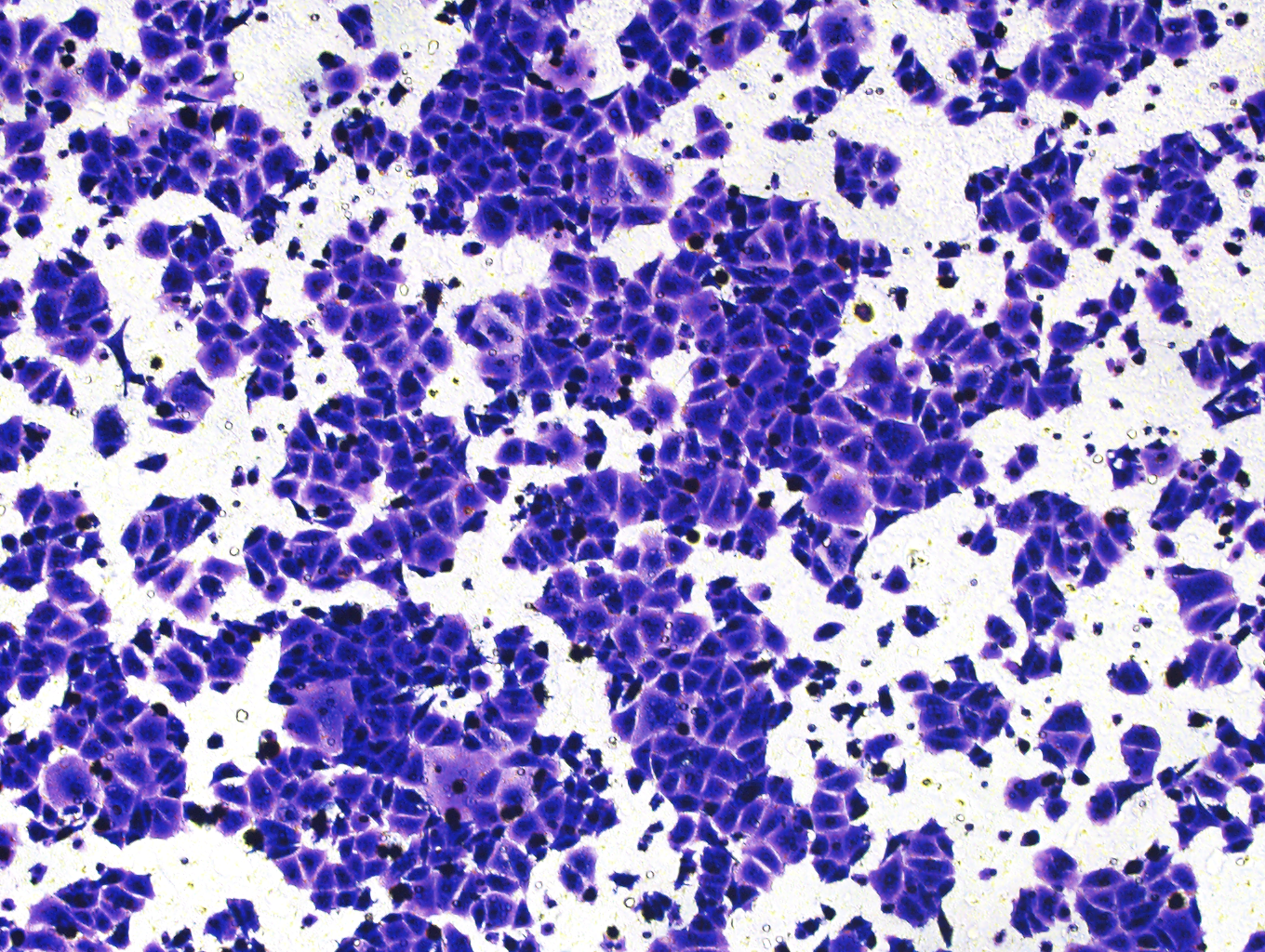

Supplement: Supplementary file 11 — EV Figure Source Data part 3 [file 44318_2025_363_MOESM11_ESM.zip › Figure EV6/EV6I/Ephrin A1+5 (5).tif]
