# Supplementary material for: Ephrin A1 functions as a ligand of EGFR to promote EMT and metastasis in gastric cancer
Source: EMBO J. 2025 Jan 21;44(5):1464–87. doi: 10.1038/s44318-025-00363-x (PMC11876641; doi:10.1038/s44318-025-00363-x)
Supplement: Supplementary file 12 — Expanded View Figures [file 44318_2025_363_MOESM12_ESM.pdf]

Expanded View Figures

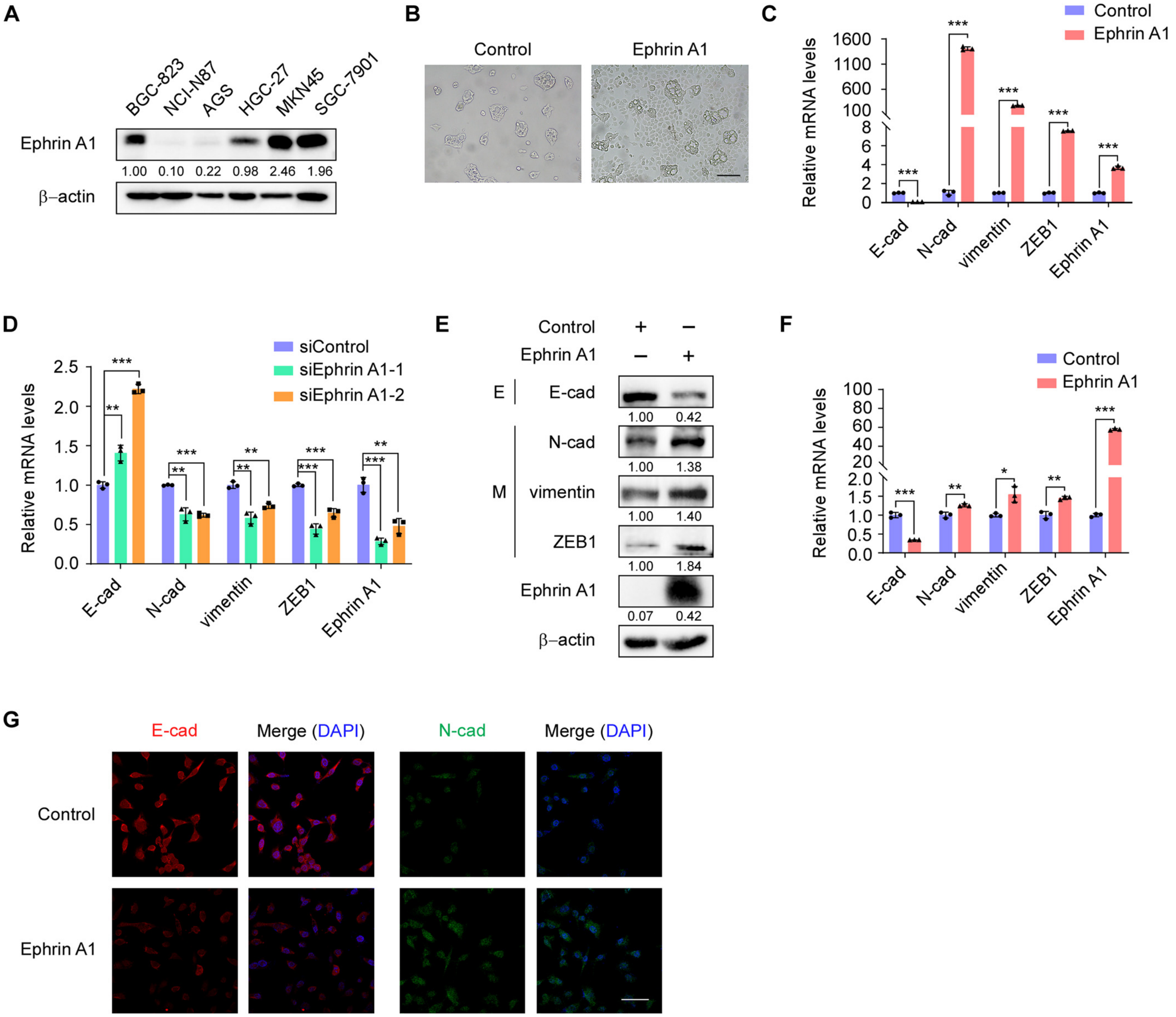

Figure EV1. Ephrin A1 promotes EMT of gastric cancer cells.

(**A**) Western blot analysis of the expression levels of Ephrin A1 in different gastric cancer cells. (**B**) Brightfield images showing the morphological changes in NCI-N87 cells stably expressing Ephrin A1. Scale bar, 200 µm. (**C**) Quantitative RT-PCR analysis of the expression levels of EMT markers in NCI-N87 cells transfected with pLVX-Ephrin A1 lentivirus or not. *P* values from left to right, *P* = 1.33e-6, *P* = 2.65e-7, *P* = 1.63e-7, *P* = 7.89e-9, *P* = 1.38e-5. (**D**) Quantitative RT-PCR analysis of EMT markers in MKN45 cells transfected with control or Ephrin A1 siRNAs. *P* values from left to right, *P* = 0.0029, *P* = 8.13e-6, *P* = 0.0015, *P* = 1.33e-5, *P* = 0.0011, *P* = 0.0010, *P* = 0.0001, *P* = 0.0004, *P* = 0.0003, *P* = 0.0026. (**E**, **F**) Western blot and quantitative RT-PCR analyses of the expression of EMT markers in control and Ephrin A1-overexpressing AGS cells. *P* values from left to right, *P* = 7.92e-5, *P* = 0.0068, *P* = 0.0111, *P* = 0.0017, *P* = 6.69e-8. (**G**) Immunofluorescence staining of EMT markers in control and Ephrin A1-overexpressing AGS cells. DNA was visualized by DAPI. Scale bar, 50 µm. Experiments were performed three times of biological replicates. Data are shown as mean ± SD. *$P < 0.05$, **$P < 0.01$, ***$P < 0.001$ (Student's *t* test). Source data are available online for this figure.

 © The Author(s)

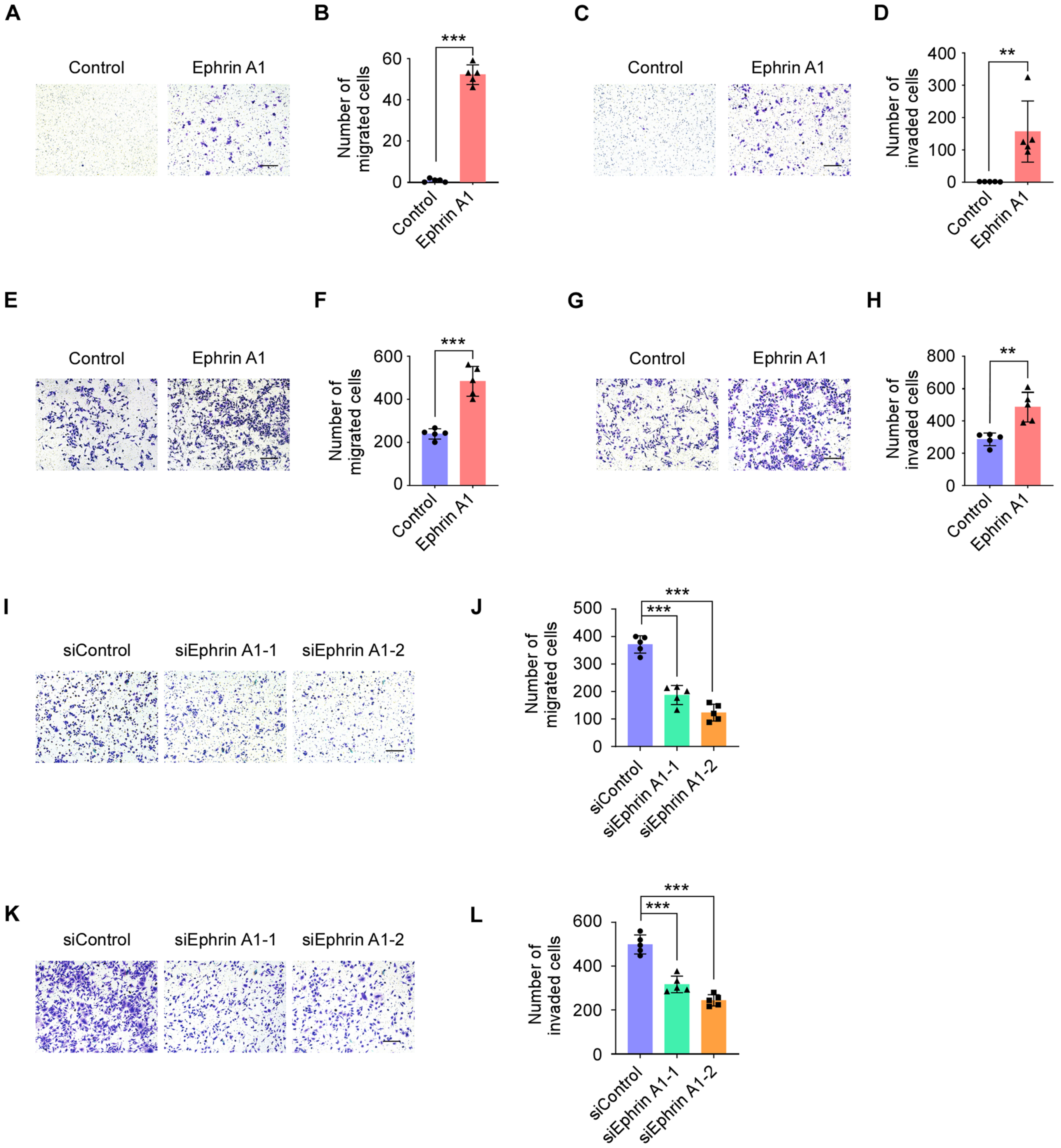

Figure EV2. Ephrin A1 promotes migration and invasion abilities of gastric cancer cells.

(**A–D**) Transwell migration and invasion assays were performed in NCI-N87 cells stably expressing Ephrin A1 or not. The migrated and invaded cells were counted (**B**, $P = 1.05e\text{-}8$; **D**, $P = 0.0064$). Scale bars, 200 μm. (**E–H**) Transwell migration and invasion analyses of control and Ephrin A1-overexpressing AGS cells. The migrated and invaded cells were counted (**F**, $P = 7.27e\text{-}5$; H, $P = 0.0021$). Scale bars, 200 μm. (**I–L**) Transwell migration and invasion analyses of MKN45 cells transfected with control or Ephrin A1 siRNAs. The migrated and invaded cells were counted (**J**, **L**). P values from left to right (**J**), $P = 3.05e\text{-}6$, $P = 1.19e\text{-}7$. P values from left to right (**L**), $P = 1.13e\text{-}5$, $P = 3.19e\text{-}7$. Scale bars, 200 μm. Experiments were performed three times of biological replicates. Data are shown as mean ± SD. Statistical significance was determined by Student's t test (**B**, **D**, **F**, **H**) and one-way ANOVA (**J**, **L**). $^{**}P < 0.01$, $^{***}P < 0.001$. Source data are available online for this figure.

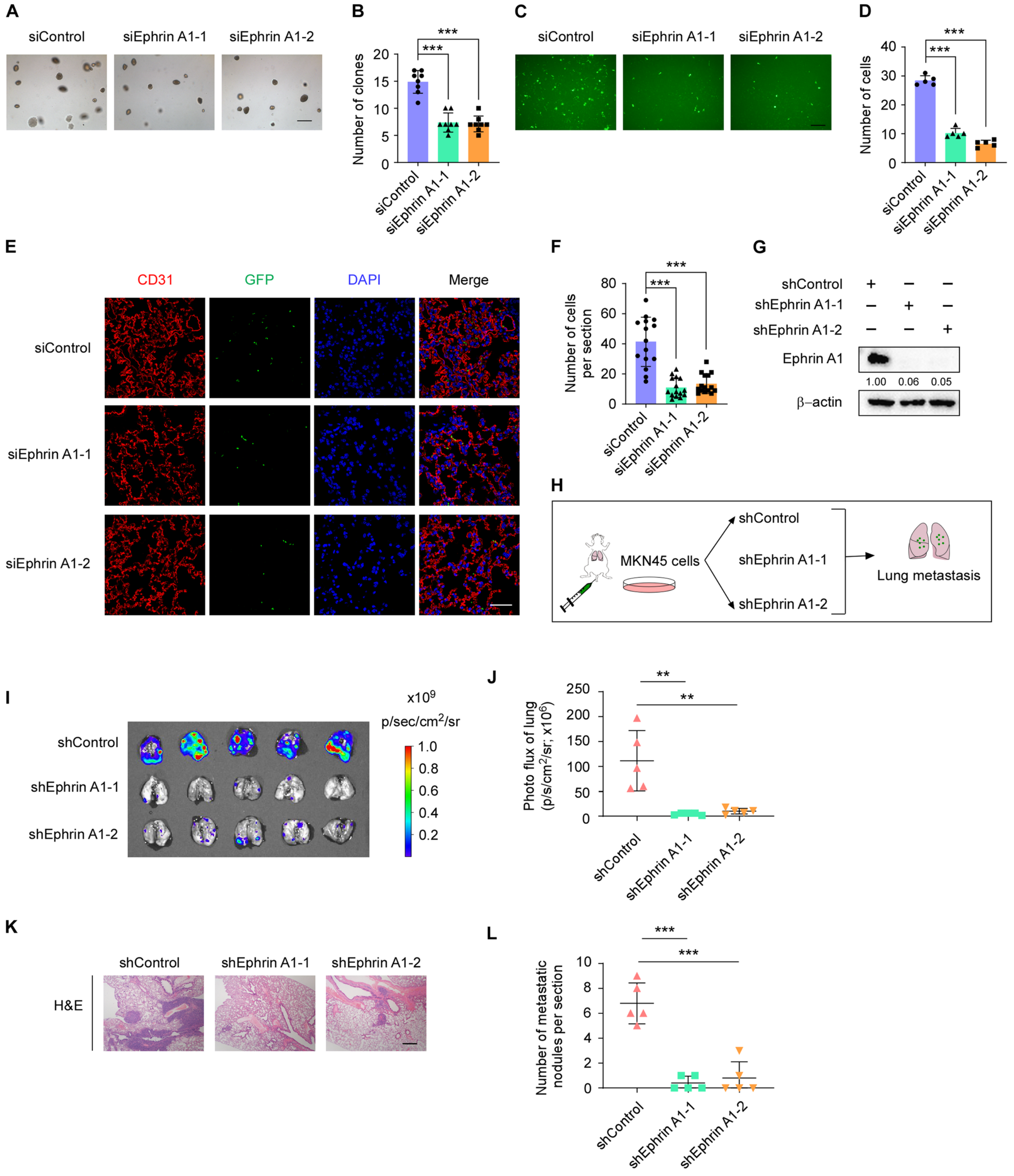
A
siControl
siEphrin A1-1
siEphrin A1-2
B
Number of clones
siControl
siEphrin A1-1
siEphrin A1-2
C
siControl
siEphrin A1-1
siEphrin A1-2
D
Number of cells
siControl
siEphrin A1-1
siEphrin A1-2
E
CD31
GFP
DAPI
Merge
siControl
siEphrin A1-1
siEphrin A1-2
F
Number of cells per section
siControl
siEphrin A1-1
siEphrin A1-2
G
shControl
shEphrin A1-1
shEphrin A1-2
Ephrin A1
1.00
0.06
0.05
β–actin
H
MKN45 cells
shControl
shEphrin A1-1
shEphrin A1-2
Lung metastasis
I
shControl
shEphrin A1-1
shEphrin A1-2
x10^9 p/sec/cm^2/sr
J
Photo flux of lung (p/s/cm^2/sr; x10^6)
shControl
shEphrin A1-1
shEphrin A1-2
K
shControl
shEphrin A1-1
shEphrin A1-2
H&E
L
Number of metastatic nodules per section
shControl
shEphrin A1-1
shEphrin A1-2

 © The Author(s)

Figure EV3. Downregulation of Ephrin A1 inhibits colonization and metastasis abilities of gastric cancer cells.

(**A**, **B**) 3D soft agar colony formation analysis of control or Ephrin A1 knockdown MKN45 cells. The number of clones was counted (**B**). *P* values from left to right, $P = 1.18e\text{-}7$, $P = 6.90e\text{-}8$. Scale bar, 400 µm. Experiments were performed three times of biological replicates. (**C**, **D**) TEM analysis of MKN45 cells transfected with control or Ephrin A1 siRNAs. The number of migrated cells was counted (**D**). *P* values from left to right, $P = 7.44e\text{-}10$, $P = 7.29e\text{-}11$. Scale bar, 200 µm. Experiments were performed three times of biological replicates. (**E**, **F**) Immunofluorescence analysis of the control or Ephrin A1 knockdown MKN45 cells that extravasated to lungs. The number of extravasated cells was counted (**F**). *P* values from left to right, $P = 4.17e\text{-}9$, $P = 3.39e\text{-}8$. Scale bar, 50 µm. (**G**) The expression of Ephrin A1 was detected by western blot assay in MKN45 and MKN45-Ephrin A1 KD cells. (**H**) Schematic representation of the in vivo lung metastasis model. Luciferase-labeled MKN45 and MKN45-Ephrin A1 KD cells were tail vein injection to NSG mice (5×10^5 cells, $n = 5$). (**I**) In vivo bioluminescence imaging (BLI) of lungs are shown. (**J**) Quantification of BLI signal intensity of lungs ($n = 5$ mice per group). *P* values from left to right, $P = 0.0012$, $P = 0.0018$. (**K**, **L**) H&E staining of cross sections of mice lungs (**K**) and quantification of lung metastases nodules (**L**). $N = 5$ in each group. *P* values from left to right, $P = 9.37e\text{-}6$, $P = 1.80e\text{-}5$. Scale bar, 400 µm. Data are shown as mean ± SD. Statistical significance was determined one-way ANOVA. $^{**}P < 0.01$, $^{***}P < 0.001$. Source data are available online for this figure.

© The Author(s)

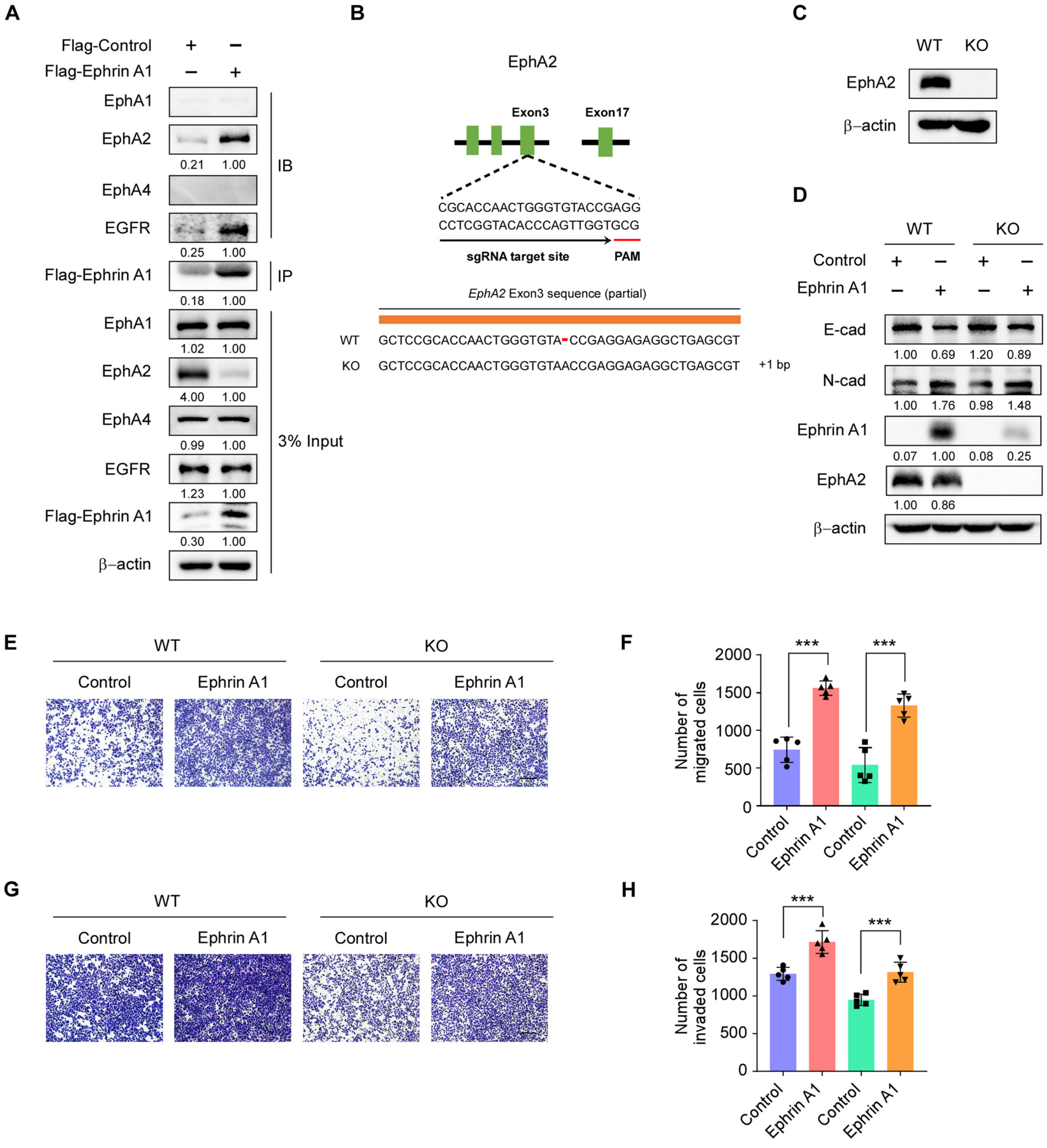

Figure EV4. Ephrin A1 inducing EMT in AGS cells is independent of EphA2.

(**A**) AGS cells were transfected with control-Flag or Ephrin A1-Flag vectors and applied to immunoprecipitation analysis with anti-Flag beads. The IP samples were subjected to western blot analysis with the indicated antibodies. (**B**) The construction of *EphA2* knockout (KO) cells by CRISPR-Cas9 system in AGS cells. (**C**) Western blot analysis of EphA2 expression in AGS wild type (WT) and AGS-*EphA2*-KO cells. (**D**) Western blot analysis of the expression of EMT markers in control and Ephrin A1-overexpressing AGS cells or *EphA2*-KO AGS cells. (**E**–**H**) Transwell migration (**E**) and invasion (**G**) analyses of AGS and AGS-*EphA2*-KO cells transfected with control or Ephrin A1 plasmid. The migrated and invaded cells were counted (**F**, **H**). *P* values from left to right (**F**), $P = 5.68e\text{-}6$, $P = 8.97e\text{-}6$. *P* values from left to right (**H**), $P = 0.0001$, $P = 0.0006$. Scale bars, 200 μm. Experiments were performed three times of biological replicates. Data are shown as mean ± SD. Statistical significance was determined one-way ANOVA. ***$P < 0.001$. Source data are available online for this figure.

 © The Author(s)

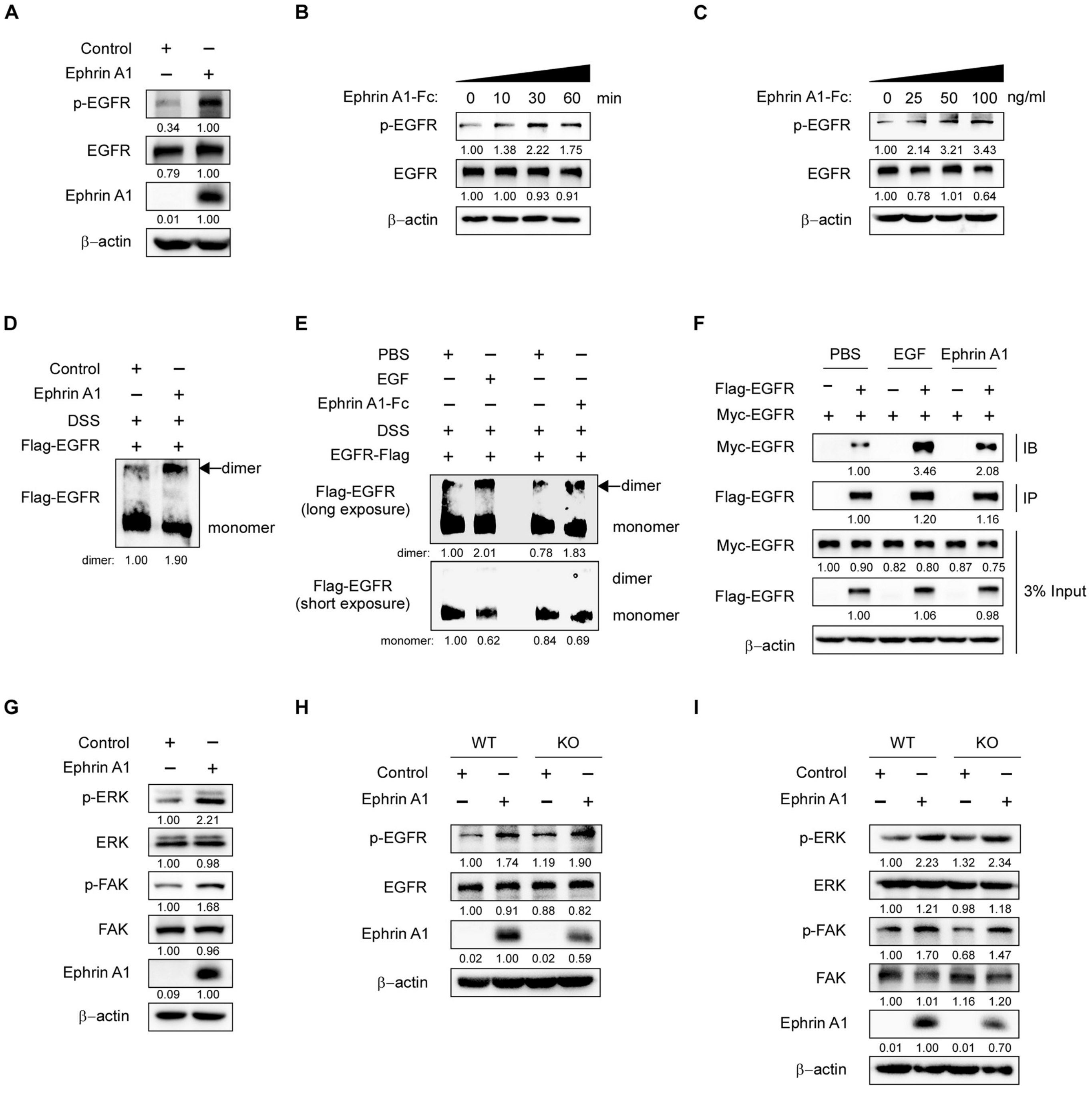

Figure EV5. Ephrin A1 activates EGFR signaling in AGS cells.

(**A**) Western blot analysis of phosphorylation level of EGFR in control or Ephrin A1-overexpressiong AGS cells. (**B**, **C**) AGS cells were treated with 100 ng/ml Ephrin A1-Fc for indicated times or treated with different concentrations of Ephrin A1-Fc for 30 min, and then subjected to western blot analysis. (**D**) Control and Ephrin A1-overexpressing AGS cells were collected and crosslinked with disuccinimidyl suberate (DSS) treatment, followed by western blot analysis. (**E**) AGS cells were treated with PBS, 100 ng/ml EGF or 1 μg/ml Ephrin A1-Fc proteins and crosslinked with DSS to detect the dimerization level of EGFR. (**F**) AGS cells were transfected with EGFR-Flag and EGFR-Myc plasmids. After 24 h, the cells were treated with or without PBS, 100 μg/ml EGF or 1 μg/ml Ephrin A1-Fc. The cells were then immunoprecipitated with anti-Flag beads and subjected to western blot analysis. (**G**) Western blot analysis of EGFR downstream signaling in control and Ephrin A1-overexpressiong AGS cells with indicated antibodies. (**H**, **I**) Western blot analysis of the phosphorylation levels of EGFR (**H**), ERK and FAK (**I**) in control or Ephrin A1-overexpressiong AGS and AGS-*EphA2*-KO cells. Experiments were performed three times of biological replicates. Source data are available online for this figure.

© The Author(s)

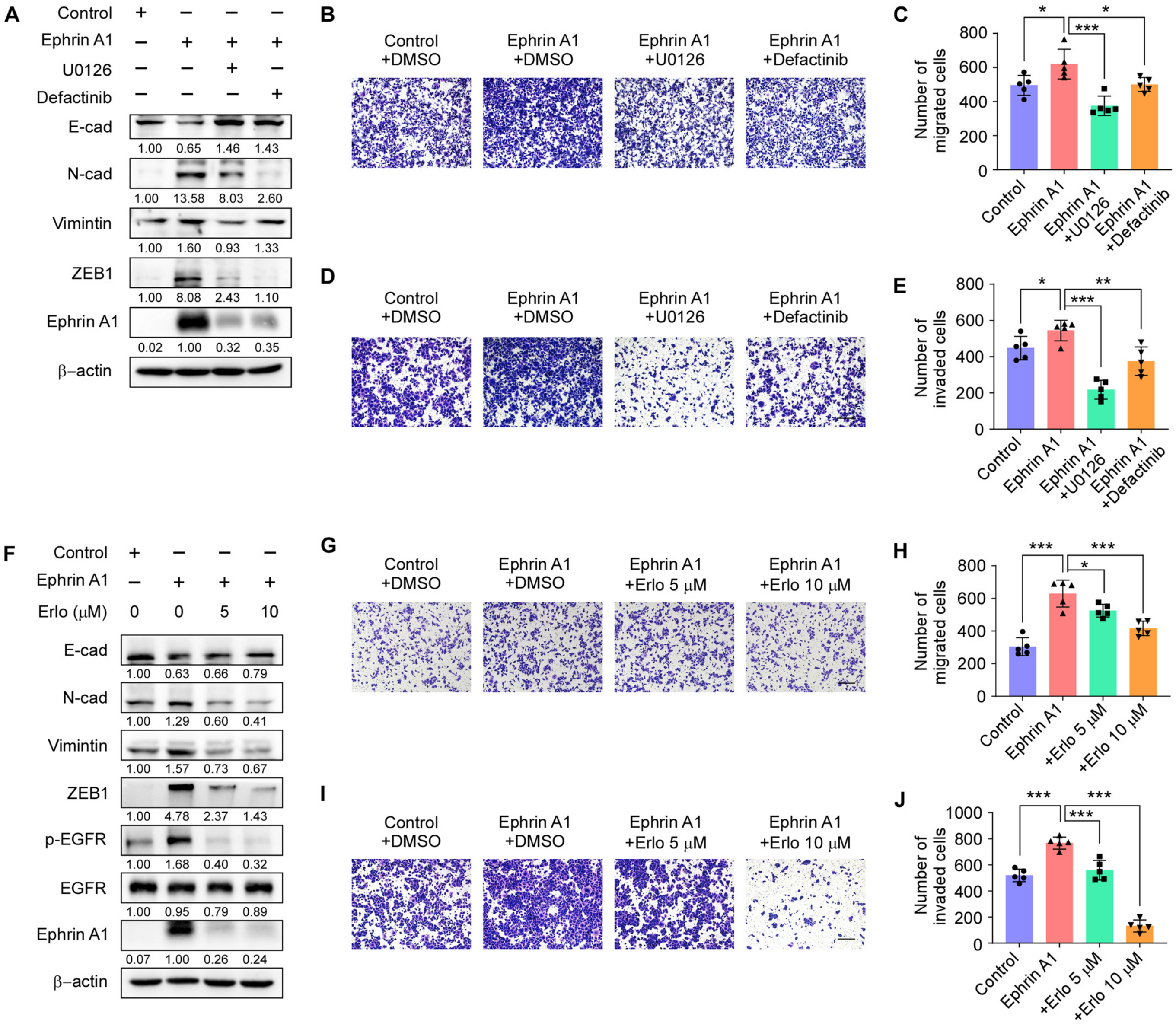

Figure EV6. Ephrin A1 promotes EMT of gastric cancer cells through EGFR activation.

(**A**) Control or Ephrin A1-overexpressing AGS cells were pre-treated with DMSO, 10 μM U0126 or 1 μM defactinib separately and applied to western blot analysis with indicated antibodies. (**B–E**) The transwell migration (**B**) and invasion (**C**) assays of control or Ephrin A1-overexpressing AGS cells that treated with 10 μM U0126 and 1 μM defactinib or not. The migrated and invaded cells were counted (**C**, **E**). *P* values from left to right (**C**), $P = 0.0306$, $P = 8.10e\text{-}5$, $P = 0.0387$. *P* values from left to right (**E**), $P = 0.0490$, $P = 6.86e\text{-}7$, $P = 0.0012$. Scale bars, 200 μm. (**F**) Control or Ephrin A1-overexpressing AGS cells were treated with DMSO or erlotinib and applied to western blot analysis with indicated antibodies. (**G–J**) The transwell migration (**G**) and invasion (**I**) analyses in control or Ephrin A1-overexpressing AGS cells that treated with DMSO or erlotinib at indicated concentration. The migrated and invaded cells were counted (**H**, **J**). *P* values from left to right (**H**), $P = 7.10e\text{-}7$, $P = 0.0486$, $P = 0.0001$. *P* values from left to right (**J**), $P = 1.39e\text{-}5$, $P = 0.0001$, $P = 2.21e\text{-}11$. Scale bars, 200 μm. Experiments were performed three times of biological replicates. Data are shown as mean ± SD. Statistical significance was determined one-way ANOVA. $^{*}P < 0.05$, $^{**}P < 0.01$, $^{***}P < 0.001$. Source data are available online for this figure.

 © The Author(s)
